# Gingival Fibroblasts Are Sensitive to Oral Cell Lysates Indicated by Their IL11 Expression

**DOI:** 10.3390/bioengineering10101193

**Published:** 2023-10-13

**Authors:** Layla Panahipour, Azarakhsh Oladzad Abbasabadi, Reinhard Gruber

**Affiliations:** 1 Department of Oral Biology, University Clinic of Dentistry, Medical University of Vienna, 1090 Vienna, Austria; layla.panahipour@meduniwien.ac.at (L.P.); az.azar66@gmail.com (A.O.A.); 2 Department of Periodontology, School of Dental Medicine, University of Bern, 3010 Bern, Switzerland; 3 Austrian Cluster for Tissue Regeneration, 1200 Vienna, Austria

**Keywords:** necrosis, cell lysates, dentistry, IL11, gingival fibroblast, TGF-β

## Abstract

Damaged cells that appear as a consequence of invasive dental procedures or in response to dental materials are supposed to release damage-associated signals. These damage-associated signals not only support tissue regeneration but might also contribute to unwanted fibrosis. The aim of this study was to identify a molecular target that reflects how fibroblasts respond to necrotic oral tissue cells. To simulate the cell damage, we prepared necrotic cell lysates by sonication of the osteocytic cell line IDG-SW3 and exposed them to gingival fibroblasts. RNAseq revealed a moderate increase in IL11 expression in the gingival fibroblasts, a pleiotropic cytokine involved in fibrosis and inflammation, and also in regeneration following trauma. Necrotic lysates of the human squamous carcinoma cell lines HSC2 and TR146, as well as of gingival fibroblasts, however, caused a robust increase in IL11 expression in the gingival fibroblasts. Consistently, immunoassay revealed significantly increased IL11 levels in the gingival fibroblasts when exposed to the respective lysates. Considering that IL11 is a TGF-β target gene, IL11 expression was partially blocked by SB431542, a TGF-β receptor type I kinase inhibitor. Moreover, lysates from the HSC2, TR146, and gingival fibroblasts caused a moderate smad2/3 nuclear translocation in the gingival fibroblasts. Taken together and based on IL11 expression, our findings show that fibroblasts are sensitive to damaged oral tissue cells.

## 1. Introduction

Dentistry today involves a spectrum of procedures, some of which are rather invasive, to maintain the integrity of the periodontal tissues with their complex interaction with the tooth-supporting periodontal ligament, the alveolar bone, with its connective tissue being covered by the oral epithelium [1,2]. Implant dentistry requires drilling and the insertion of dental implants, which do not cause only bone cells such as osteocytes to be damaged [3]. For instance, necrotic osteocytes release damage-associated molecular patterns affecting bone resorption [4] and, in general, osteocyte death promotes bone loss [5]. In vitro, the murine cementocyte cell line IDG-SW3 is an appropriate model for studying the biology of osteocyte necrosis [4]. Further invasive procedures include, but are not limited to, scaling and root planing [6], electrosurgery [7], and cryosurgery [8]. Thus, the epithelial cells and gingival fibroblasts of the connective tissue might be injured by physical (e.g., high pressures, temperatures, or osmotic forces) or mechanical (e.g., shear forces) stress. The same is true for clinical endodontics dealing with the treatment conditions of injured pulp tissue by physical or mechanical damage [9]. Not to be underestimated is the chemical damage to gingival fibroblasts and cell types caused by dental adhesives [10]. Reconstructive dentistry also takes advantage of connective tissue grafting where partial cell damage is likely [2]. Even though the damage to oral cells has not been systematically investigated, there is reason to assume that cell damage causes a response of local bystander cells such as gingival fibroblasts.

Cell damage caused by physical, mechanical, or chemical means culminates in the loss of membrane integrity; thus, the content of the cytoplasm and the disrupted membrane remnants are released [11]. This process of cell death is termed “necrosis” and stands in contrast to the caspase-related death of apoptosis [12], necroptosis [13], and pyroptosis [14]. Thus, necrotic cells can signal the need for repair, providing danger signals that are understood by the local bystander cells, i.e., healthy cells becoming responsible for initiating the defense and repair of the injured site. Necrotic cells, however, might generate a pathological microenvironment that is in favor of a fibrotic, more scar-like tissue. Even though the oral mucosa heals with minimal fibrosis, the formation of a scar cannot be ruled out [15]. Further evidence exists for denture-induced fibrous hyperplasia [16] and dental materials can also cause fibrosis [17]. Clinical scenarios like these have prompted research seeking to uncover the molecular link between cell damage, tissue regeneration, and scar-like repair.

IL11 is a pleiotropic cytokine that has recently received great attention based on the work of Cook and his group, who uncovered its central role not only in regeneration following trauma but also in fibrosis and inflammation [18]. From an evolutionary perspective, in fish, tadpoles, and axolotl, IL11 is uniquely upregulated in the regenerative organ, the blastema, following the loss of a fin, tail, or limb. IL11 in mammals is supposed to be rooted in its deep evolutionary role for epimorphic appendage regeneration, but this is a double-edged sword, since the same mechanisms can cause organ failure in mammals [18]; for instance, IL11 became a therapeutic target in kidney dysfunction [19], cardiovascular fibrosis [20], idiopathic pulmonary fibrosis [21], systemic sclerosis [22], and non-alcoholic steatohepatitis [23]. However, when produced by osteoblasts and osteocytes, IL11 links mechanical loading, bone formation, and WNT signaling [24]. Thus, accumulating evidence suggests that IL11 is emerging as a master regulator of fibrosis, inflammation, and bone remodeling.

Support for an implication for dentistry comes from observations that IL11 levels in the crevicular fluid are higher in periodontitis compared to gingivitis and healthy sites [25]. IL11 is further linked to the progression of radiation-induced oral mucositis simulating cancer treatment [26]. Moreover, IL11 was found in gingival mucosa during rehabilitation with implant-retained overdentures, at the first surgical stage [27]. Even though not explicitly shown by the immunostaining of gingival tissue, accumulating evidence suggests that gingival fibroblasts are among the potential sources of IL11 [28,29,30,31,32,33,34,35], similar to what is known from other cells [24], such as stromal cells–fibroblasts and smooth muscle cells [36,37]. Among the major drivers of IL11 expression in gingival fibroblasts, consistent with other types of fibroblasts, is TGF-β, a pleiotropic growth factor present in bone [28,29,30], dentine [31], platelet-rich plasma [32], enamel matrix derivative [33], and milk [34,35]. TGF-β and the consequent activation of an autocrine IL11 signaling loop in fibroblasts are critically linked to fibrosis but potentially also support tissue regeneration.

TGF-β can be expressed in a secreted form or be present on the cell surface in a membrane-bound form. For instance, the membrane-bound form can be expressed by lymphocytes [38] but also in head and neck squamous cell carcinoma cell lines [39], and singe cell analysis of oral squamous cell carcinoma revealed epithelia and cancer-associated fibroblasts to be major sources of TGF-β [40]. Moreover, osteocytes are recognized as being sensitive to TGF-β signaling, but whether this involves autocrine mechanisms remains unclear [41]. We can thus assume that lysates from our IDG-SW3 osteocytes and oral squamous cell carcinoma cell lines HSC2 and TR146, as well as from gingival fibroblasts, possess a TGF-β activity that might serve as a damage-associated molecular pattern driving the expression of IL11 by the gingival fibroblasts—a hypothesis driven by the increasing recognition of IL11 as a potential master regulator of oral health and disease. The aim of the present research was to introduce IL11 as a molecular marker that reflects how oral fibroblasts respond to necrotic cell lysates.

## 2. Methods

### 2.1. Cell Lines and Primary Cells

Human gingival fibroblasts were isolated from gingival explants prepared from extracted wisdom teeth from patients after obtaining informed consent. The protocol was approved by the local Ethical Committee (EK Nr. 631/2007). The HSC2 oral squamous cell carcinoma cell line was from the Japanese Collection of Research Bioresources Cell Bank (#CRB0622). The oral squamous cell carcinoma cell line TR146 originates from the European Collection of Authenticated Cell Cultures (#10032305) and the IDG-SW3 osteocytic cell line was from Kerafast Inc. (#CVCL_0P23; Boston, MA, USA). Cell expansion was performed in a CO_2_ incubator with DMEM, 10% fetal calf serum (FCS), and 1% antibiotics (Invitrogen Corporation, Carlsbad, CA, USA).

### 2.2. Cell Lysates and Basic Experimental Setting

Cells suspended at 4 × 10^6^ cells/mL DMEM were subjected to (i) sonication three times at 15 s intervals (Sonoplus; Bandelin electronic GmbH & Co. KG; Berlin, Germany) or (ii) three times freeze–thawing for 8 min at −80 °C and room temperature. Cell lysates underwent centrifugation at 2600 RCF for 5 min (Eppendorf SE, Hamburg, Germany). Necrotic cell lysates were freshly prepared for each independent test. In brief, gingival fibroblasts were seeded at 30.000 cells/cm^2^ and on the following day exposed to the undiluted cell lysates for another 24 h before analysis.

### 2.3. RNA Sequencing

Total RNA from gingival fibroblasts exposed to lysates from IDG-SW3 cells were subjected to analysis. Sequencing libraries from total RNA of the samples were prepared at the Core Facility Genomics, Medical University of Vienna, using the QuantSeq FWD with UDI V2 protocol (Lexogen GmbH, Vienna, Austria). In total, 16 PCR cycles were used for library preparation, as determined by qPCR according to the library preparation manual. Libraries were QC-checked on a Bioanalyzer 2100 (Agilent Technologies, Santa Clara, CA, USA) using a High Sensitivity DNA Kit for correct insert size and quantified using Qubit dsDNA HS Assay (Invitrogen). Pooled libraries were sequenced on a NextSeq500 instrument (Illumina, San Diego, CA, USA) in 1 × 75 bp single-end sequencing mode. Approximately 4.4 million reads per sample were generated. Reads in fastq format were generated using the Illumina bcl2fastq command line tool (v2.19.1.403). Reads were trimmed and filtered using cutadapt (1) version 2.8 to trim polyA tails, remove reads with Ns and trim bases with a quality of less than 30 from the 3′ ends of the reads. On average, 3 million reads were left after this procedure. Reads in fastq format were aligned to the mouse reference genome version GRCm38 with Gencode mV23 annotations using STAR aligner version 2.6.1a in 2 pass mode. Reads per gene were counted by STAR, and differential gene expression was calculated using DESeq2 version 1.22.2.

### 2.4. Reverse Transcription Quantitative Real-Time PCR (RT-qPCR)

Total RNA was isolated with the ExtractMe total RNA kit (Blirt S.A., Gda’nsk, Poland) and transcribed using the LabQ FirstStrand cDNA Synthesis Kit (LabQ, Labconsulting, Vienna, Austria). Reverse transcription–polymerase chain reaction (RT-PCR) was carried out (LabQ, Labconsulting, Vienna, Austria) on a CFX Connect™ Real-Time PCR Detection System (Bio-Rad Laboratories, Hercules, CA, USA). Primer sequences were IL11 Forward AAATAAGGCACAGATGCC, Reverse CCTTCCAAAGCCAGATC and GAPDH Forward AAGCCACATCGCTCAGACAC, and Reverse GCCCAATACGACCAAATCC. Expression levels were calculated by normalizing to GAPDH using the ∆∆Ct method.

### 2.5. Immunoassay

The immunoassay was carried out with the Quantikine ELISA kit R&D Systems. Gingival fibroblasts were treated with necrotic cell lysate overnight. Supernatants were collected and centrifuged prior to storing at −20 °C for not more than three weeks. The concentration of IL11 and TGF-β1 in the supernatant and cell lysates was measured by immunoassay according to the manufacturer’s instruction (DY218 and DY240, R&D Systems, Minneapolis, MN, USA).

### 2.6. Immunofluorescent Analysis

Gingival fibroblasts seeded onto Millicell EZ slides (Merck KGaA, Darmstadt, Germany) were serum-starved overnight before being exposed to the indicated cell lysates with and without SB431542 for 30 min. Then, paraformaldehyde fixation blocking was conducted with 5% bovine serum albumin and 0.3% Triton X-100 in PBS. After permeabilization with 0.1% Triton X-100, cells were incubated with Smad2/3 antibody (D7G7 XP rabbit mAb #8685, Cell Signaling Technology, Danvers, MA, USA) overnight at 4 °C. For detection, the Alexa Fluor 488-conjugated antibody (Cell Signaling Technology) was used. Images were captured with a fluorescent microscope (Axio Imager M2, Carl Zeiss AG, Oberkochen, Germany).

### 2.7. Statistical Analysis

The experiments were repeated at least three times. Statistical analysis was based on a ratio paired t-test and RM one-way ANOVA test using Prism v9 (GraphPad Software, La Jolla, CA, USA).

## 3. Results

### 3.1. Gene Expression Screening Assay

To identify the changes in the genetic signature of gingival fibroblasts exposed to lysates from IDG-SW3 osteocytes, we used an RNAseq approach. Among the most strongly upregulated genes (>20×) were not only our main target gene, IL11, but also a series of other genes including glycerol kinase, proto-oncogene c-Fos, cyclic AMP-dependent transcription factor ATF-3, leukemia inhibitory factor, amphiregulin, C11orf96, prostaglandin E synthase, cell migration-inducing and hyaluronan-binding protein, large neutral amino acids transporter small subunit 1, argininosuccinate lyase, DNA-binding protein inhibitor ID-4, and stanniocalcin-1, which we are partially focusing on in an ongoing study. Also, other genes showing no signal in the untreated cells became highly expressed by the lysates from the IDG-SW3, particularly IL24 and humanin-like protein 10 (Supplementary Table S1). Here, we concentrate on how cell lysates regulate the expression of IL11 in gingival fibroblasts.

### 3.2. IL11 Expression Analysis

**Figure 1:**
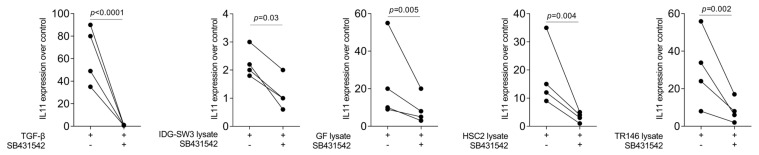
Gene expression of IL11 in gingival fibroblasts. TGF-β at 10 ng/mL and necrotic cell lysate of IDG-SW3, gingival fibroblasts, HSC2, and TR146 significantly increased IL11 expression, and 10 μM SB431542 reversed the effect. Data are expressed as x-fold over the respective untreated controls. Data points represent independent experiments. Statistical analysis was based on ratio-paired *t*-tests and *p*-values are indicated. Significance was set at *p* < 0.05.

To confirm the IL11 gene expression changes observed with RNAseq, we performed RT-PCR. As expected, recombinant TGF-β1 caused a robust increase in IL11 expression in the gingival fibroblasts, while the impact of the murine IDG-SW3 lysates was significant but weak (Figure 1). We then extended the research towards human cells. The lysates prepared from the gingival fibroblasts, the HSC2 cells, and particularly the TR146 cells caused a strong increase in IL11 expression by the gingival fibroblasts (Figure 1). Blocking the TGF-β receptor type I kinase with the inhibitor SB431542 attenuated the forced expression of IL11 induced by cell lysates (Figure 1).

### 3.3. IL11 Immunoassay

**Figure 2:**
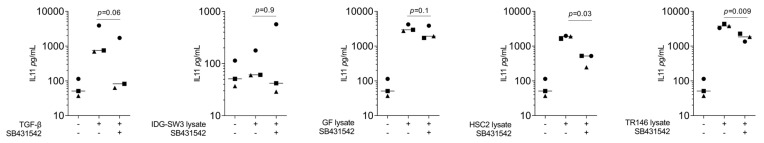
IL11 protein secretion in gingival fibroblasts. The supernatants of exposed cells by TGF-β at 10 ng/mL and necrotic cell lysate of IDG-SW3, gingival fibroblasts, HSC2, and TR146 increased the IL11 protein secretion, and 10 μM SB431542 reduced protein secretion. Data points in different symbol shapes represent three independent experiments. Statistical analysis was based on RM one-way ANOVA, with the Geisser–Greenhouse correction tests and *p*-values indicated. Significance was set at *p* < 0.05.

Consistently, the immunoassay confirmed that the lysates from the human gingival fibroblasts, HSC2 cells, and TR146 cells caused a sharp increase in IL11 in the respective supernatant, again being less pronounced with the IDG-SW3 lysates (Figure 2). In support of the expression changes, SB431542 reduced the IL11 levels in the supernatant of the gingival fibroblasts exposed to lysates from the gingival fibroblasts, HSC2 cells, and TR146 cells (Figure 2). These findings suggest that, in particular, the lysates prepared from human cells are potent inducers of IL11 expression in the gingival fibroblasts, a response that partially involves TGF-β receptor type I kinase signaling.

### 3.4. Smad2/3 Nuclear Translocation

**Figure 3:**
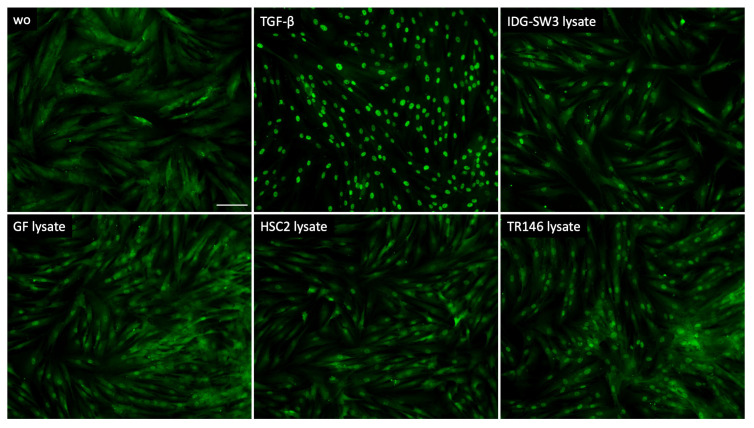
Smad2/3 nuclear translocation in gingival fibroblasts. A total of 10 ng/mL TGF-β and sonicated necrotic cell lysate of IDG-SW3, gingival fibroblasts, HSC2, and TR146 induced the nuclear translocation of Smad2/3. The positive cells show a focused signal indicating the nuclear translocation of the Smad2/3 immunofluorescence. The scale bar represents 100 µm.

**Figure 4:**
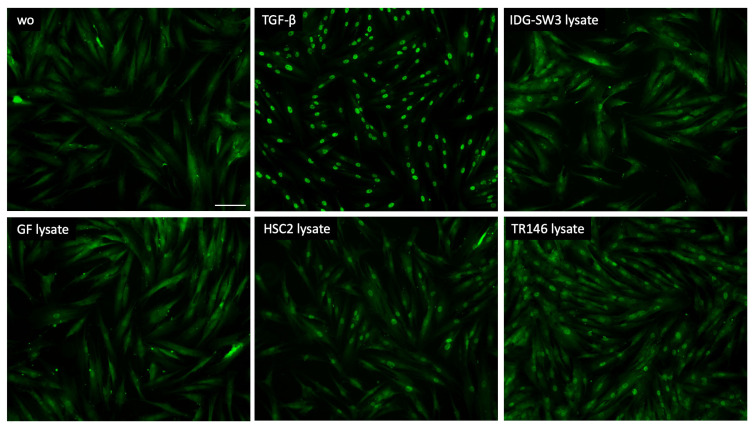
Smad2/3 nuclear translocation in gingival fibroblasts. A total of 10 ng/mL TGF-β and necrotic cell lysate of IDG-SW3, gingival fibroblasts, HSC2, and TR146 made by freeze–thawing induced the nuclear translocation of Smad2/3. The positive cells show a focused signal indicating the nuclear translocation of the Smad2/3 immunofluorescence. The scale bar represents 100 µm.

**Figure 5:**
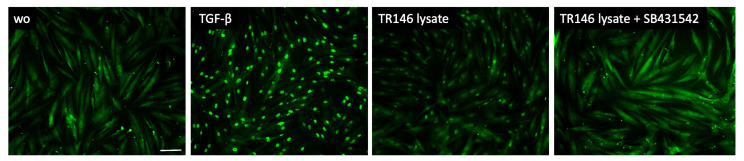
Smad2/3 nuclear translocation in gingival fibroblasts. A total of 10 ng/mL TGF-β and necrotic TR146 cell lysate induced the nuclear translocation of Smad2/3. The positive cells show a focused signal indicating the nuclear translocation of the Smad2/3 immunofluorescence. This effect was reversed with the application of SB431542. The scale bar represents 100 µm.

To further support the involvement of TGF-β signaling as a response of gingival fibroblasts to cell lysates, we analyzed the nuclear translocation of smad2/3. We could show that lysate prepared by sonication (Figure 3) as well as by freeze–thawing (Figure 4) of the IDG-SW3 cells, gingival fibroblasts, HSC2, and TR146 cells, all caused moderate but convincing nuclear staining, indicating a nuclear translocation of smad2/3 in the gingival fibroblasts. The recombinant TGF-β1, though, caused the ultimate nuclear translocation of the smad2/3 (Figure 3 and Figure 4). Moreover, and in support of the IL11 expression changes, blocking the TGF-β receptor type I kinase with the inhibitor SB431542 decreased the nuclear translocation of the smad2/3, supporting the assumption that cell lysates can activate canonical TGF-β signaling in gingival fibroblasts (Figure 5).

## 4. Discussion

This research was inspired by the clinical scenario where invasive dental procedures such as the insertion of dental implants, scaling, and root planning, as well as electrosurgery and cryosurgery, may cause local cell damage, i.e., cell injury involving the disruption of cell membranes [3,6,7,8]. Thus, the damaged cell itself, with its membrane fragments and the content of the cytoplasm, holds a large spectrum of damage-associated molecular patterns that signal the need for local tissue repair. The necrotic death of osteocytes occurs in the course of implant drilling [3] and we have learned recently that necrotic osteocytes release damage-associated molecular patterns, affecting bone resorption [4]. For screening purposes, we performed a RNAseq of gingival fibroblasts exposed to lysates from osteocyte-like IDG-SW3 cells and noticed a robust increase in IL11 expression, a TGF-β sensitive target gene we have worked on recently [28,29,30,31,32,33,34,35]. Apart from the osteocyte-like IDG-SW3 cells, lysates from HSC2 and TR146 cells with their epithelial characteristic, as well as lysates prepared from the gingival fibroblasts themselves, were capable of increasing IL11 in gingival fibroblasts—overall supporting our assumption that lysates from oral cells can cause a local TGF-β-related cell response.

Support for this claim comes from our observation that IL11 expression can, at least partially, be blocked by the TGF-β receptor type I kinase inhibitor SB431542, suggesting that canonical TGF-β signaling and potentially also other pathways are activated by the necrotic cell lysates that drive IL11 expression. In support of TGF-β signaling are our observations that all the lysates moderately activated the translocation of the smad2/3 into the nucleus. However, this is not causally linked to IL11 expression, but, considering that the SB431542 also blocked the TR146 lysate-induced smad2/3 translocation, there is reason to suggest that TGF-β signaling is at least partially involved in IL11 expression. These findings are in support of previous observations that lysates prepared from other cells hold TGF-β activity. For instance, fibroblasts seeded onto a biomimetic extracellular matrix showed TGF-β1 in the cell lysates [42], and the secretome of bone marrow stromal cells [43] and Tenon’s fibroblast [44] contains TGF-β1 and TGF-β2. Also, cardiac fibroblasts produce TGF-β [45]. Likewise, the bronchial epithelial cells BEAS-2B release TGF-β1 into the supernatant [46] and umbilical cord blood mesenchymal stromal cells show TGF-β in the supernatant and cell lysates [47]. Moreover, supernatants from three malignant glioma cell lines have TGF-β activity [48]. It is also important to note here that, while the HSC2 and TR146 are oral squamous cell carcinoma cells, the IDG-SW3 and particularly the gingival fibroblasts are regular non-transformed cells; thus, the capacity of cell lysates to drive IL11 expression in gingival fibroblasts is not restricted to tumor cells. There is, however, accumulating evidence that TGF-β is a paracrine signal released from fibroblastic and epithelial cell lines. Nevertheless, our IL11 bioassay is perhaps only an indicator of a more complex cellular response based on genes in the signature we identified by RNAseq analysis.

Care should be taken when interpreting these in vitro findings, as the activity of TGF-β is complex. For instance, fibroblast-specific IL11 transgene expression causes heart and kidney fibrosis and organ failure, whereas the genetic deletion of the interleukin 11 receptor alpha chain 1 protects against disease [49]. Moreover, IL11 is a critical driver of cardiovascular fibrosis [20], lung abnormalities [21,50], kidney injury and renal repair [19], and liver disease [23]. Considering the skull, mice lacking IL11 do not have craniosynostosis, and have normal long bone mass, suggesting that IL11 is not relevant in bone development [51]—but IL11 may play a key role in bone regeneration. It is, however, hard to interpret the clinical relevance of our observation that IL11 expression by gingival fibroblasts is induced by oral cell lysates. However, if we consider TGF-β and IL11 to be major drivers of collagen synthesis, that fibroblasts lacking smad3 exhibit impaired collagen lattice contraction, and that TGF-β target genes are annulated [52], we can speculate that the TGF-β–IL11 axis activated by necrotic cells upon tissue damage might support the early stages of oral regeneration.

Future research is necessary to understand the impact of this necrotic cell-driven IL11 expression; for instance, it can be hypothesized that mice with a fibroblast-specific deletion of IL11 have an impaired capacity to regenerate upon tissue damage; this is not limited to defects where necrotic cells are a potential source of TGF-β activity. Necrotic cells are only one aspect of how TGF-β drives IL11 expression by fibroblasts and may initiate a cascade of fibrotic events, as discovered by Stuart Cook [18]. Considering the many local therapies used in dentistry that support oral tissue regeneration, such as platelet-rich fibrin [53,54] and enamel matrix derivative [33], all of which are major drivers of the TGF-β–IL11 axis, our data add useful information to the complex mosaic of how a clinical treatment—be it destructive or supportive—affects cellular and molecular mechanisms in the defect site. Hence, it may be that IL11 levels in the crevicular fluid should not be interpreted as inflammatory markers in periodontal diseases, but reflect the attempt of the tissue to repair [25,55]. Thus, IL11 is among the sensitive molecules that are increasingly expressed when fibroblasts come into contact with damaged oral cells. IL11 expression might help to establish a bioassay to screen for damage-associated molecular patterns that have not been identified in our in vitro setting. Clinically, IL11 might be one of the biomarkers that indicate a response of the connective tissue to severe necrotic cell damage, but this is more of a supposition than a conclusion. However, our finding that lysates drive IL11 expression remains descriptive and future research should investigate how this increase modulates fibrosis, cell migration, osteogenesis, or other physiological processes that can be reproduced by in vitro models.

Considering that our mechanical and thermal approach to preparing cell lysates is harsh and does not reflect the caspase-driven forms of cell death, such as apoptosis [56,57], pyroptosis [14], and necroptosis [13], major questions remain unanswered, namely, “What is the impact of the supernatant of IDG-SW3, TR146, HSC2, and other cells undergoing a caspase-related form of cell death on gingival fibroblasts” and “Is IL11 also a biomarker for damage-associated molecular papers for apoptotic, pyroptotic and necroptotic cells?” Thus, a study limitation is that our setting is restricted to necrotic lysates and does not cover the classical caspase-mediated forms of cell death that are also relevant in maintaining oral tissue homeostasis. Another limitation and an inspiration for future research is to understand the molecular mechanisms of how our cell lysates caused the sharp increase of IL11 in gingival fibroblasts; according to our findings, this cannot be solely explained by TGF-β.

In conclusion, we show here that damaged cells, as they are presumably present at sites of invasive dental procedures, are not passive—they provoke an IL11 expression in gingival fibroblasts and other cells that we identified by RNAseq but which are not the focus of this investigation. Future research should therefore aim to better understand the local cellular response trigged by damaged cells, a response to some extent simulating the clinical environment of invasive dental procedures, which, in turn, could highlight a need to avoid tissue damage during minimally invasive treatment concepts. Perhaps this suggestion is an over-interpretation of our findings, but at least we know now that gingival fibroblasts are sensitive to damaged oral tissue cells.

## Data Availability

All data are available on demand.

## Supplementary Materials

The following supporting information can be downloaded at: https://www.mdpi.com/article/10.3390/bioengineering10101193/s1, Table S1: RNAseq data with >20-fold upregulated genes when gingival fibroblasts are exposed to lysates from IDG-SW3 cells.
