# Supplementary material for: Gingival Fibroblasts Are Sensitive to Oral Cell Lysates Indicated by Their IL11 Expression
Source: Bioengineering (Basel). 2023 Oct 13;10(10):1193. doi: 10.3390/bioengineering10101193 (PMC10604186; doi:10.3390/bioengineering10101193)
Supplement: Supplementary file 1 [file bioengineering-10-01193-s001.zip › bioengineering-2603237-supplementary.pdf]

Table S1: Gingival fibroblast being exposed to IDG-SW3 lysates for 24 hours

GeneType	GeneSymbol	ENSG	Control	IDG-SW3	Ratio of IDG-SW3_Co
protein_coding	GK	ENSG00000198814.12	1	56	56
protein_coding	FOS	ENSG00000170345.9	2	102	51
protein_coding	ATF3	ENSG00000162772.16	1	41	41
protein_coding	LIF	ENSG00000128342.4	1	37	37
protein_coding	AREG	ENSG00000109321.10	5	182	36.4
protein_coding	C11orf96	ENSG00000187479.6	49	1436	29.3
protein_coding	PTGES	ENSG00000148344.10	16	452	28.3
antisense	AL139385.1	ENSG00000275880.1	1	27	27
protein_coding	IL11	ENSG00000095752.6	2	50	25
protein_coding	CEMIP	ENSG00000103888.16	641	14840	23.2
protein_coding	SLC7A5	ENSG00000103257.8	5	112	22.4
protein_coding	ASL	ENSG00000126522.16	5	108	21.6
protein_coding	ID4	ENSG00000172201.11	1	21	21
protein_coding	STC1	ENSG00000159167.11	151	3168	21
protein_coding	TM4SF1	ENSG00000169908.11	26	502	19.3
antisense	AC083862.2	ENSG00000272941.1	1	18	18
protein_coding	NR4A3	ENSG00000119508.17	10	171	17.1
protein_coding	SLC16A6	ENSG00000108932.11	2	34	17
protein_coding	TBC1D32	ENSG00000146350.13	1	17	17
protein_coding	MEDAG	ENSG00000102802.9	11	164	14.9
protein_coding	WNT2	ENSG00000105989.9	5	74	14.8
protein_coding	ATP8B4	ENSG00000104043.14	4	56	14
protein_coding	RHOU	ENSG00000116574.5	1	14	14
protein_coding	SCARF2	ENSG00000244486.8	1	14	14
protein_coding	LXN	ENSG00000079257.7	8	106	13.3
protein_coding	C5AR2	ENSG00000134830.5	4	53	13.3
protein_coding	TCIM	ENSG00000176907.4	2	26	13
protein_coding	KLF2	ENSG00000127528.5	2	26	13
antisense	PAR	ENSG00000226179.6	1	13	13
protein_coding	RAET1G	ENSG00000203722.7	1	13	13
protein_coding	FGD4	ENSG00000139132.14	1	12	12
lincRNA	AC005736.1	ENSG00000262185.2	1	12	12
antisense	AC097376.2	ENSG00000273247.5	1	12	12
protein_coding	ABCA8	ENSG00000141338.13	2	23	11.5
protein_coding	CHST2	ENSG00000175040.5	50	560	11.2
protein_coding	SPON1	ENSG00000262655.3	1	11	11
sense_intronic	AC024075.1	ENSG00000267904.1	1	11	11
protein_coding	WHRN	ENSG00000095397.13	1	11	11
transcribed_unprocessed_pseudogene	C2orf27A	ENSG00000197927.12	1	11	11

protein_coding	MVK	ENSG00000110921.13	1	11	11
protein_coding	TEX9	ENSG00000151575.14	1	11	11
protein_coding	PTGS2	ENSG00000073756.11	92	937	10.2
protein_coding	NR4A2	ENSG00000153234.13	94	951	10.1
protein_coding	SCG2	ENSG00000171951.4	3	30	10
protein_coding	RELL1	ENSG00000181826.9	2	20	10
protein_coding	PRH1	ENSG00000231887.7	1	10	10
lincRNA	LINC00926	ENSG00000247982.6	1	10	10
protein_coding	ADORA2B	ENSG00000170425.3	1	10	10
protein_coding	DKK¬†1	ENSG00000107984.9	10	95	9.5
protein_coding	ARID5A	ENSG00000196843.15	4	38	9.5
protein_coding	ABHD17C	ENSG00000136379.11	50	468	9.4
protein_coding	RGS4	ENSG00000117152.13	24	220	9.2
protein_coding	SMOC1	ENSG00000198732.10	20	180	9
protein_coding	CFAP44	ENSG00000206530.10	1	9	9
protein_coding	KCNJ8	ENSG00000121361.3	1	9	9
protein_coding	PCBD2	ENSG00000132570.14	1	9	9
protein_coding	DEPDC1	ENSG00000024526.16	1	9	9
protein_coding	BAK1	ENSG00000030110.12	1	9	9
protein_coding	EMILIN2	ENSG00000132205.10	1	9	9
lincRNA	AC008035.1	ENSG00000272369.1	1	9	9
protein_coding	EDNRB	ENSG00000136160.15	12	106	8.8
processed_transcript	SOCS2-AS1	ENSG00000246985.7	4	35	8.8
transcribed_processed_pseudogene	RPLP0P2	ENSG00000243742.5	3	26	8.7
lincRNA	AC026979.4	ENSG00000285669.1	2	17	8.5
protein_coding	DNER	ENSG00000187957.7	2	17	8.5
antisense	AL161421.1	ENSG00000275202.1	9	74	8.2
protein_coding	HK2	ENSG00000159399.9	21	169	8
protein_coding	PDK4	ENSG00000004799.7	110	883	8
protein_coding	IL6R	ENSG00000160712.12	6	48	8
protein_coding	GALNT18	ENSG00000110328.5	2	16	8
protein_coding	ACKR3	ENSG00000144476.5	1	8	8
TEC	AC020910.5	ENSG00000279329.1	1	8	8
processed_pseudogene	AC122718.1	ENSG00000250461.1	1	8	8
processed_pseudogene	AC108463.1	ENSG00000227992.1	1	8	8
protein_coding	BRIP1	ENSG00000136492.8	1	8	8
lincRNA	AC006480.2	ENSG00000273448.1	1	8	8
protein_coding	ATP2A3	ENSG00000074370.17	1	8	8
antisense	AC003986.3	ENSG00000236536.1	1	8	8
protein_coding	STYK1	ENSG00000060140.8	1	8	8
protein_coding	NKX3-1	ENSG00000167034.9	31	233	7.5
protein_coding	GLIS3	ENSG00000107249.22	8	60	7.5

protein_coding	RGS18	ENSG00000150681.9	6	45	7.5
protein_coding	PCDHGA7	ENSG00000253537.2	2	15	7.5
protein_coding	C17orf58	ENSG00000186665.9	15	110	7.3
protein_coding	SYT12	ENSG00000173227.13	3	22	7.3
protein_coding	ETS2	ENSG00000157557.11	32	226	7.1
protein_coding	SORBS2	ENSG00000154556.18	20	141	7.1
protein_coding	PITPNC1	ENSG00000154217.14	62	435	7
protein_coding	THBD	ENSG00000178726.6	2	14	7
processed_transcript	LINC00511	ENSG00000227036.7	2	14	7
protein_coding	GJC2	ENSG00000198835.3	2	14	7
protein_coding	ENTPD7	ENSG00000198018.6	1	7	7
antisense	AC005229.4	ENSG00000273314.1	1	7	7
sense_overlapping	AP001486.2	ENSG00000260966.1	1	7	7
TEC	AL662907.1	ENSG00000278997.1	1	7	7
protein_coding	EXOSC2	ENSG00000130713.15	1	7	7
protein_coding	AP1S3	ENSG00000152056.16	1	7	7
protein_coding	AGT	ENSG00000135744.7	1	7	7
lincRNA	AC048341.1	ENSG00000257354.2	1	7	7
protein_coding	PCDHGA1	ENSG00000204956.5	1	7	7
protein_coding	SIX3	ENSG00000138083.4	1	7	7
protein_coding	ZNF669	ENSG00000188295.14	1	7	7
protein_coding	SPTY2D1OS	ENSG00000247595.4	1	7	7
sense_intronic	AC104564.3	ENSG00000264290.1	1	7	7
protein_coding	CSRP2	ENSG00000175183.9	10	68	6.8
protein_coding	PC	ENSG00000173599.13	5	34	6.8
protein_coding	ITPRIP	ENSG00000148841.16	29	194	6.7
protein_coding	NFATC2	ENSG00000101096.19	3	20	6.7
protein_coding	CENPJ	ENSG00000151849.14	4	26	6.5
protein_coding	MMP10	ENSG00000166670.9	2	13	6.5
protein_coding	SPATA5	ENSG00000145375.7	2	13	6.5
protein_coding	C1QL1	ENSG00000131094.3	2	13	6.5
protein_coding	CYP19A1	ENSG00000137869.14	2	13	6.5
protein_coding	GPR183	ENSG00000169508.6	2	13	6.5
protein_coding	AKR7A3	ENSG00000162482.4	2	13	6.5
protein_coding	FAM102B	ENSG00000162636.15	3	19	6.3
antisense	AL139393.2	ENSG00000272841.1	4	25	6.3
protein_coding	KYNU	ENSG00000115919.14	128	799	6.2
protein_coding	NR4A1	ENSG00000123358.19	31	186	6
protein_coding	DGKH	ENSG00000102780.16	6	36	6
protein_coding	PCDHGA12	ENSG00000253159.2	4	24	6
protein_coding	BRSK1	ENSG00000160469.16	2	12	6
protein_coding	DTNB	ENSG00000138101.18	2	12	6
protein_coding	MYPN	ENSG00000138347.15	2	12	6
protein_coding	ASPHD2	ENSG00000128203.6	1	6	6

protein_coding	TBC1D8	ENSG00000204634.12	1	6	6
protein_coding	MCHR1	ENSG00000128285.4	1	6	6
lincRNA	SND1-IT1	ENSG00000279078.1	1	6	6
TEC	AC009078.3	ENSG00000280152.1	1	6	6
protein_coding	RAB3A	ENSG00000105649.9	1	6	6
lincRNA	AC004825.2	ENSG00000274818.1	1	6	6
antisense	SBF2-AS1	ENSG00000246273.7	1	6	6
protein_coding	C8orf37	ENSG00000156172.5	1	6	6
antisense	AC090425.1	ENSG00000272660.1	1	6	6
processed_pseudogene	RPL7P8	ENSG00000235045.2	1	6	6
protein_coding	WASHC1	ENSG00000181404.16	1	6	6
lincRNA	AL021807.1	ENSG00000272468.1	1	6	6
protein_coding	LRP11	ENSG00000120256.10	1	6	6
lincRNA	LINC02298	ENSG00000257556.1	1	6	6
processed_pseudogene	RBM8B	ENSG00000258427.3	1	6	6
protein_coding	ALX4	ENSG00000052850.6	1	6	6
antisense	UBL7-AS1	ENSG00000247240.7	1	6	6
sense_intronic	AC008982.2	ENSG00000269688.1	1	6	6
protein_coding	GLIPR1L2	ENSG00000180481.10	1	6	6
antisense	AC104958.2	ENSG00000272502.1	1	6	6
protein_coding	SYCE1	ENSG00000171772.16	1	6	6
antisense	AL357033.4	ENSG00000277496.1	1	6	6
protein_coding	INTS6L	ENSG00000165359.15	1	6	6
protein_coding	GDPGP1	ENSG00000183208.12	1	6	6
protein_coding	FAHD2A	ENSG00000115042.9	1	6	6
antisense	AC067930.4	ENSG00000254859.1	1	6	6
protein_coding	ARHGEF33	ENSG00000214694.11	1	6	6
antisense	AP006623.1	ENSG00000250397.2	1	6	6
protein_coding	FAM151B	ENSG00000152380.9	1	6	6
protein_coding	ENOSF1	ENSG00000132199.19	15	89	5.9
protein_coding	KCNG1	ENSG00000026559.13	5	29	5.8
protein_coding	KCNK1	ENSG00000135750.14	4	23	5.8
protein_coding	SPHK1	ENSG00000176170.13	16	91	5.7
protein_coding	SLC30A1	ENSG00000170385.9	190	1080	5.7
protein_coding	AASS	ENSG00000008311.14	3	17	5.7
protein_coding	SLC2A13	ENSG00000151229.12	20	112	5.6
protein_coding	CAMK4	ENSG00000152495.10	5	28	5.6
antisense	DLGAP1-AS1	ENSG00000177337.7	5	28	5.6
protein_coding	GTF2I	ENSG00000263001.5	4	22	5.5
protein_coding	CXCL16	ENSG00000161921.14	4	22	5.5
protein_coding	FOXO1	ENSG00000150907.7	2	11	5.5
protein_coding	ZSCAN5A	ENSG00000131848.9	2	11	5.5
protein_coding	KBTBD11	ENSG00000176595.3	2	11	5.5
protein_coding	TWIST1	ENSG00000122691.12	102	556	5.5

protein_coding	IRX5	ENSG00000176842.14	3	16	5.3
protein_coding	SCARA5	ENSG00000168079.16	3	16	5.3
protein_coding	ATP1B1	ENSG00000143153.12	47	250	5.3
protein_coding	SLC27A4	ENSG00000167114.12	5	26	5.2
protein_coding	TGFB2	ENSG00000092969.11	48	247	5.1
protein_coding	ID2	ENSG00000115738.9	38	191	5
protein_coding	DENND3	ENSG00000105339.10	7	35	5
protein_coding	SFT2D3	ENSG00000173349.5	6	30	5
protein_coding	FSTL3	ENSG00000070404.9	5	25	5
protein_coding	NFKBIZ	ENSG00000144802.11	3	15	5
protein_coding	ARNTL	ENSG00000133794.17	3	15	5
processed_pseudogene	AC136628.3	ENSG00000253520.1	3	15	5
protein_coding	DOK5	ENSG00000101134.11	3	15	5
protein_coding	TNFAIP8L3	ENSG00000183578.7	3	15	5
protein_coding	SLCO4A1	ENSG00000101187.15	2	10	5
transcribed_unprocessed_pseudogene	CA5BP1	ENSG00000186312.10	2	10	5
protein_coding	SLC43A3	ENSG00000134802.17	2	10	5
protein_coding	HCST	ENSG00000126264.9	2	10	5
protein_coding	ABAT	ENSG00000183044.11	2	10	5
protein_coding	ZNF891	ENSG00000214029.4	2	10	5
protein_coding	CITED4	ENSG00000179862.6	1	5	5
snRNA	RNU4ATAC	ENSG00000264229.1	1	5	5
protein_coding	IL17D	ENSG00000172458.4	1	5	5
processed_transcript	HLA-F-AS1	ENSG00000214922.9	1	5	5
protein_coding	RADIL	ENSG00000157927.16	1	5	5
protein_coding	INTS3	ENSG00000143624.13	1	5	5
protein_coding	ARID3B	ENSG00000179361.17	1	5	5
protein_coding	RNF227	ENSG00000179859.9	1	5	5
protein_coding	PIWIL4	ENSG00000134627.11	1	5	5
processed_pseudogene	AC023043.2	ENSG00000267404.1	1	5	5
sense_intronic	AL606834.1	ENSG00000269906.1	1	5	5
protein_coding	AGMAT	ENSG00000116771.5	1	5	5
protein_coding	NXNL2	ENSG00000130045.16	1	5	5
protein_coding	SLC37A1	ENSG00000160190.13	1	5	5
protein_coding	RAB3IP	ENSG00000127328.21	1	5	5
sense_overlapping	AL359752.1	ENSG00000261662.1	1	5	5
protein_coding	PPP1R13B	ENSG00000088808.17	1	5	5
protein_coding	CFAP58	ENSG00000120051.14	1	5	5
protein_coding	MRPL23	ENSG00000214026.10	1	5	5
protein_coding	SKA3	ENSG00000165480.15	1	5	5
protein_coding	ULK4	ENSG00000168038.10	1	5	5
protein_coding	ZNF595	ENSG00000272602.5	1	5	5

transcribed_unprocessed_pseudogene	ANO7L1	ENSG00000237276.8	1	5	5
antisense	AL109811.2	ENSG00000230337.1	1	5	5
protein_coding	PDGFRL	ENSG00000104213.12	1	5	5
processed_pseudogene	RPL17P34	ENSG00000213432.2	1	5	5
antisense	AL359921.2	ENSG00000273058.2	1	5	5
protein_coding	TPGS1	ENSG00000141933.9	1	5	5
protein_coding	FRAS1	ENSG00000138759.18	1	5	5
antisense	AL353194.1	ENSG00000229539.1	1	5	5
antisense	AC011468.5	ENSG00000275055.1	1	5	5
protein_coding	ADAMTS10	ENSG00000142303.13	1	5	5
processed_pseudogene	RPL35P5	ENSG00000225573.4	1	5	5
protein_coding	SLC2A4	ENSG00000181856.14	1	5	5
antisense	AC004130.1	ENSG00000271133.5	1	5	5
protein_coding	ID3	ENSG00000117318.8	64	317	5
protein_coding	IFT81	ENSG00000122970.15	13	64	4.9
protein_coding	ADAMTS4	ENSG00000158859.9	11	54	4.9
protein_coding	VEGFA	ENSG00000112715.21	173	841	4.9
protein_coding	SMOX	ENSG00000088826.17	37	178	4.8
protein_coding	FRMPD4	ENSG00000169933.13	5	24	4.8
protein_coding	LYSMD1	ENSG00000163155.11	4	19	4.8
lincRNA	CARMN	ENSG00000249669.9	7	33	4.7
lincRNA	AC015726.1	ENSG00000270091.1	3	14	4.7
protein_coding	SHPK	ENSG00000197417.7	3	14	4.7
protein_coding	PLAT	ENSG00000104368.17	82	381	4.6
protein_coding	INHBB	ENSG00000163083.5	5	23	4.6
protein_coding	PRXL2A	ENSG00000122378.13	14	64	4.6
protein_coding	DGKD	ENSG00000077044.10	7	32	4.6
antisense	LUCAT1	ENSG00000248323.6	9	41	4.6
protein_coding	CASP10	ENSG00000003400.14	9	41	4.6
protein_coding	MAPKAPK2	ENSG00000162889.10	280	1272	4.5
protein_coding	ACSL4	ENSG00000068366.19	227	1025	4.5
protein_coding	GPR146	ENSG00000164849.9	2	9	4.5
sense_intronic	AC016949.1	ENSG00000230732.4	2	9	4.5
lincRNA	AF117829.1	ENSG00000251136.8	2	9	4.5
protein_coding	MINK1	ENSG00000141503.15	2	9	4.5
processed_pseudogene	HMGB1P21	ENSG00000248909.1	2	9	4.5
protein_coding	ZNF778	ENSG00000170100.13	2	9	4.5
protein_coding	SLC25A10	ENSG00000183048.11	2	9	4.5
protein_coding	OTULINL	ENSG00000145569.5	2	9	4.5
protein_coding	PCDHB15	ENSG00000113248.5	2	9	4.5
protein_coding	NFIL3	ENSG00000165030.3	59	264	4.5
protein_coding	ADARB1	ENSG00000197381.15	42	187	4.5

protein_coding	NEXN	ENSG00000162614.18	252	1118	4.4
protein_coding	DGKI	ENSG00000157680.15	30	132	4.4
lincRNA	MUC20-OT1	ENSG00000242086.8	5	22	4.4
protein_coding	TCF21	ENSG00000118526.6	15	65	4.3
protein_coding	ATOH8	ENSG00000168874.12	6	26	4.3
protein_coding	ZNF470	ENSG00000197016.11	6	26	4.3
protein_coding	PTPRE	ENSG00000132334.16	3	13	4.3
protein_coding	ENKD1	ENSG00000124074.11	3	13	4.3
processed_pseudogene	AP000902.1	ENSG00000266891.1	3	13	4.3
protein_coding	DHX37	ENSG00000150990.7	3	13	4.3
protein_coding	PDPN	ENSG00000162493.16	3	13	4.3
protein_coding	FPGT	ENSG00000254685.6	3	13	4.3
protein_coding	ISPD	ENSG00000214960.9	10	43	4.3
protein_coding	FAM20C	ENSG00000177706.8	189	812	4.3
protein_coding	RASD1	ENSG00000108551.4	35	150	4.3
protein_coding	CTH	ENSG00000116761.11	7	30	4.3
protein_coding	RDH10	ENSG00000121039.9	175	744	4.3
protein_coding	DNAJC24	ENSG00000170946.14	4	17	4.3
protein_coding	CPM	ENSG00000135678.11	10	42	4.2
protein_coding	PANX2	ENSG00000073150.13	10	42	4.2
protein_coding	ADAMTS6	ENSG00000049192.14	5	21	4.2
antisense	AC067852.2	ENSG00000266962.2	5	21	4.2
lincRNA	AC093627.6	ENSG00000242611.2	5	21	4.2
protein_coding	CHMP1B	ENSG00000255112.2	246	1025	4.2
protein_coding	LRFN4	ENSG00000173621.8	20	83	4.2
protein_coding	DAB2IP	ENSG00000136848.17	83	344	4.1
protein_coding	KLF9	ENSG00000119138.4	64	264	4.1
antisense	PRR34-AS1	ENSG00000241990.5	8	33	4.1
protein_coding	CRISPLD2	ENSG00000103196.11	119	486	4.1
protein_coding	KIF26B	ENSG00000162849.15	25	102	4.1
protein_coding	GALNT15	ENSG00000131386.18	76	308	4.1
protein_coding	GPCPD1	ENSG00000125772.12	53	214	4
protein_coding	FNDC3A	ENSG00000102531.16	137	549	4
protein_coding	GYG2	ENSG00000056998.19	16	64	4
protein_coding	PLEKHA8	ENSG00000106086.19	6	24	4
protein_coding	SMYD4	ENSG00000186532.11	5	20	4
protein_coding	TRAF6	ENSG00000175104.14	4	16	4
lincRNA	AL031055.1	ENSG00000271784.1	4	16	4
protein_coding	TBC1D10A	ENSG00000099992.15	3	12	4
protein_coding	PIGW	ENSG00000277161.1	3	12	4
antisense	CARD8-AS1	ENSG00000268001.1	3	12	4
protein_coding	VARS2	ENSG00000137411.17	3	12	4
protein_coding	WWC1	ENSG00000113645.14	2	8	4
protein_coding	PET117	ENSG00000232838.3	2	8	4

protein_coding	AL135905.2	ENSG00000285976.1	2	8	4
protein_coding	CENPK	ENSG00000123219.12	2	8	4
protein_coding	OBSCN	ENSG00000154358.20	2	8	4
lincRNA	SCAMP1-	ENSG00000245556.2	2	8	4
protein_coding	MB21D2	ENSG00000180611.6	2	8	4
protein_coding	CYB5R2	ENSG00000166394.14	2	8	4
protein_coding	ACTR3B	ENSG00000133627.17	2	8	4
protein_coding	HAUS8	ENSG00000131351.14	2	8	4
protein_coding	TMEM221	ENSG00000188051.6	2	8	4
protein_coding	PSG2	ENSG00000242221.8	2	8	4
antisense	AL122035.2	ENSG00000272909.1	2	8	4
protein_coding	COQ8B	ENSG00000123815.11	2	8	4
lincRNA	AC011978.2	ENSG00000261542.1	1	4	4
antisense	DLGAP1-AS2	ENSG00000262001.1	1	4	4
sense_intronic	PEG13	ENSG00000282164.3	1	4	4
protein_coding	SYT14	ENSG00000143469.18	1	4	4
protein_coding	ACTR5	ENSG00000101442.9	1	4	4
antisense	AC003102.1	ENSG00000260793.2	1	4	4
protein_coding	DUS2	ENSG00000167264.17	1	4	4
lincRNA	AC006213.2	ENSG00000267058.1	1	4	4
protein_coding	SCN11A	ENSG00000168356.11	1	4	4
protein_coding	HUNK	ENSG00000142149.8	1	4	4
protein_coding	KCNE5	ENSG00000176076.7	1	4	4
protein_coding	SYTL5	ENSG00000147041.11	1	4	4
processed_transcript	ERVK13-1	ENSG00000260565.6	1	4	4
protein_coding	GPR85	ENSG00000164604.12	1	4	4
antisense	AC004846.1	ENSG00000258376.2	1	4	4
protein_coding	FANCA	ENSG00000187741.14	1	4	4
protein_coding	CEP72	ENSG00000112877.7	1	4	4
protein_coding	KIAA1324L	ENSG00000164659.14	1	4	4
antisense	MZF1-AS1	ENSG00000267858.5	1	4	4
processed_pseudogene	NACA3P	ENSG00000121089.4	1	4	4
processed_pseudogene	NSRP1P1	ENSG00000235613.2	1	4	4
protein_coding	TMC1	ENSG00000165091.16	1	4	4
antisense	PARD6G-	ENSG00000267270.5	1	4	4
protein_coding	CENPH	ENSG00000153044.9	1	4	4
lincRNA	AC008280.3	ENSG00000272156.1	1	4	4
protein_coding	DGAT2	ENSG00000062282.14	1	4	4
lincRNA	AC010245.2	ENSG00000272040.1	1	4	4
lincRNA	AP005329.3	ENSG00000272688.1	1	4	4
protein_coding	NBPF12	ENSG00000268043.7	1	4	4
processed_pseudogene	RPL21P10	ENSG00000239272.1	1	4	4
protein_coding	ARHGAP33	ENSG00000004777.18	1	4	4
lincRNA	AL391121.1	ENSG00000272933.1	1	4	4

protein_coding	CDNF	ENSG00000185267.9	1	4	4
antisense	AC003986.2	ENSG00000232821.1	1	4	4
lincRNA	SERPINB9P1	ENSG00000230438.6	1	4	4
lincRNA	AC098479.1	ENSG00000273493.1	1	4	4
protein_coding	ZNF223	ENSG00000178386.12	1	4	4
transcribed_processed_pseudogene	EEF1DP7	ENSG00000263883.1	1	4	4
transcribed_processed_pseudogene	IPO5P1	ENSG00000269837.1	1	4	4
processed_pseudogene	PABPC1P4	ENSG00000255642.1	1	4	4
lincRNA	AL135910.1	ENSG00000272848.2	1	4	4
TEC	AC131212.2	ENSG00000279700.1	1	4	4
antisense	AL049780.1	ENSG00000258646.1	1	4	4
protein_coding	IFITM10	ENSG00000244242.1	1	4	4
protein_coding	RGN	ENSG00000130988.12	1	4	4
lincRNA	AC090409.1	ENSG00000267279.1	1	4	4
processed_pseudogene	TCEA1P2	ENSG00000230409.3	1	4	4
antisense	AC010531.6	ENSG00000270006.2	1	4	4
protein_coding	VILL	ENSG00000136059.14	1	4	4
protein_coding	ZNF709	ENSG00000242852.6	1	4	4
transcribed_unprocessed_pseudogene	TMEM191A	ENSG00000226287.8	1	4	4
protein_coding	CCDC153	ENSG00000248712.7	1	4	4
antisense	TAF1A-AS1	ENSG00000225265.1	1	4	4
protein_coding	KCNJ14	ENSG00000182324.6	1	4	4
antisense	ELOA-AS1	ENSG00000236810.5	1	4	4
lincRNA	AC145343.1	ENSG00000265055.1	1	4	4
lincRNA	AC069234.4	ENSG00000276188.1	1	4	4
protein_coding	ZFPM2	ENSG00000169946.13	1	4	4
protein_coding	FERMT3	ENSG00000149781.12	1	4	4
unprocessed_pseudogene	AL354751.1	ENSG00000234537.1	1	4	4
protein_coding	HIRA	ENSG00000100084.14	1	4	4
processed_pseudogene	RPL26P19	ENSG00000226221.1	1	4	4
protein_coding	GABBR2	ENSG00000136928.6	81	322	4
protein_coding	PLD1	ENSG00000075651.15	15	59	3.9
protein_coding	PENK	ENSG00000181195.10	19	74	3.9
protein_coding	PRR5L	ENSG00000135362.13	8	31	3.9
protein_coding	PTPRJ	ENSG00000149177.12	14	54	3.9
protein_coding	EHHADH	ENSG00000113790.10	7	27	3.9
protein_coding	TNFRSF11B	ENSG00000164761.8	1953	7526	3.9
protein_coding	TFPI2	ENSG00000105825.12	337	1294	3.8
protein_coding	CMIP	ENSG00000153815.16	38	145	3.8
protein_coding	CTSC	ENSG00000109861.15	117	445	3.8

protein_coding	KLHL41	ENSG00000239474.6	10	38	3.8
protein_coding	NFAT5	ENSG00000102908.20	51	193	3.8
protein_coding	PTHLH	ENSG00000087494.15	32	121	3.8
protein_coding	CCDC77	ENSG00000120647.9	9	34	3.8
protein_coding	KCNK6	ENSG00000099337.4	22	83	3.8
protein_coding	DOCK4	ENSG00000128512.20	25	94	3.8
protein_coding	CCNE1	ENSG00000105173.13	4	15	3.8
protein_coding	COX16	ENSG00000133983.14	4	15	3.8
processed_pseudogene	AC073610.1	ENSG00000239617.1	4	15	3.8
protein_coding	SLC4A2	ENSG00000164889.13	27	101	3.7
protein_coding	HOMER1	ENSG00000152413.14	11	41	3.7
protein_coding	FCMR	ENSG00000162894.11	11	41	3.7
protein_coding	RGS2	ENSG00000116741.7	285	1060	3.7
protein_coding	AURKA	ENSG00000087586.17	7	26	3.7
protein_coding	MAP7	ENSG00000135525.18	7	26	3.7
protein_coding	PLA2G4A	ENSG00000116711.9	45	166	3.7
protein_coding	PCDHGA10	ENSG00000253846.2	9	33	3.7
snoRNA	RF00410	ENSG00000202343.1	3	11	3.7
protein_coding	ATP13A2	ENSG00000159363.17	3	11	3.7
protein_coding	RPGR	ENSG00000156313.13	3	11	3.7
processed_transcript	AC004160.1	ENSG00000230333.6	3	11	3.7
transcribed_unprocessed_pseudogene	DSTNP2	ENSG00000248593.3	3	11	3.7
protein_coding	TOE1	ENSG00000132773.11	3	11	3.7
protein_coding	GSTCD	ENSG00000138780.14	3	11	3.7
protein_coding	PHKA1	ENSG00000067177.14	3	11	3.7
protein_coding	GSC	ENSG00000133937.4	3	11	3.7
protein_coding	ATP2A2	ENSG00000174437.16	226	827	3.7
protein_coding	SMAP2	ENSG00000084070.11	14	51	3.6
protein_coding	ZSCAN21	ENSG00000166529.14	11	40	3.6
protein_coding	RBBP8	ENSG00000101773.18	16	58	3.6
protein_coding	TRIM62	ENSG00000116525.13	13	47	3.6
lincRNA	FENDRR	ENSG00000268388.5	69	249	3.6
protein_coding	CPEB4	ENSG00000113742.12	104	375	3.6
protein_coding	ALG9	ENSG00000086848.14	15	54	3.6
protein_coding	IMPA2	ENSG00000141401.11	10	36	3.6
protein_coding	CCDC191	ENSG00000163617.10	5	18	3.6
protein_coding	AP5B1	ENSG00000254470.2	7	25	3.6
protein_coding	BICRA	ENSG00000063169.10	7	25	3.6
protein_coding	IL1R1	ENSG00000115594.11	252	899	3.6
protein_coding	STK39	ENSG00000198648.10	55	195	3.5
protein_coding	BZW1	ENSG00000082153.17	110	387	3.5
protein_coding	DBF4	ENSG00000006634.7	8	28	3.5

protein_coding	SDCBP2	ENSG00000125775.14	6	21	3.5
protein_coding	ZNF808	ENSG00000198482.11	4	14	3.5
protein_coding	ASPM	ENSG00000066279.17	4	14	3.5
protein_coding	MNS1	ENSG00000138587.5	4	14	3.5
protein_coding	GPR173	ENSG00000184194.5	4	14	3.5
protein_coding	SH3TC1	ENSG00000125089.16	2	7	3.5
protein_coding	CDCA2	ENSG00000184661.13	2	7	3.5
protein_coding	EDARADD	ENSG00000186197.14	2	7	3.5
processed_transcript	SNHG26	ENSG00000228649.8	2	7	3.5
antisense	AL109615.3	ENSG00000237686.6	2	7	3.5
protein_coding	UCN	ENSG00000163794.6	2	7	3.5
protein_coding	ATXN7L2	ENSG00000162650.16	2	7	3.5
protein_coding	RNF212	ENSG00000178222.12	2	7	3.5
protein_coding	CMSS1	ENSG00000184220.11	22	76	3.5
protein_coding	ZMYM1	ENSG00000197056.10	9	31	3.4
protein_coding	SAMD5	ENSG00000203727.3	37	127	3.4
protein_coding	GLMN	ENSG00000174842.16	5	17	3.4
protein_coding	FGL2	ENSG00000127951.6	5	17	3.4
protein_coding	KIF18A	ENSG00000121621.6	8	27	3.4
protein_coding	B4GALT1	ENSG00000086062.12	662	2234	3.4
protein_coding	FAM20A	ENSG00000108950.11	14	47	3.4
protein_coding	ARRDC2	ENSG00000105643.9	24	80	3.3
protein_coding	ABCA6	ENSG00000154262.12	6	20	3.3
protein_coding	KDM1B	ENSG00000165097.14	6	20	3.3
protein_coding	SYNM	ENSG00000182253.14	6	20	3.3
protein_coding	MORN4	ENSG00000171160.17	6	20	3.3
protein_coding	CCL8	ENSG00000108700.4	3	10	3.3
protein_coding	C15orf48	ENSG00000166920.12	3	10	3.3
protein_coding	ARSG	ENSG00000141337.12	3	10	3.3
protein_coding	MYORG	ENSG00000164976.8	3	10	3.3
protein_coding	HAUS3	ENSG00000214367.7	3	10	3.3
protein_coding	C17orf67	ENSG00000214226.8	3	10	3.3
protein_coding	PCDHB2	ENSG00000112852.6	3	10	3.3
protein_coding	ZNF343	ENSG00000088876.11	3	10	3.3
protein_coding	CDKL3	ENSG00000006837.11	3	10	3.3
antisense	VAC14-AS1	ENSG00000214353.7	3	10	3.3
lincRNA	LINC00484	ENSG00000229694.6	3	10	3.3
antisense	BNC2-AS1	ENSG00000234779.1	3	10	3.3
protein_coding	HGF	ENSG00000019991.16	126	419	3.3
protein_coding	SMAD7	ENSG00000101665.9	16	53	3.3
protein_coding	FNDC1	ENSG00000164694.16	67	221	3.3
protein_coding	HTRA3	ENSG00000170801.9	108	356	3.3
protein_coding	BDNF	ENSG00000176697.18	224	738	3.3
protein_coding	SAMD4A	ENSG00000020577.13	34	112	3.3

protein_coding	AGPAT4	ENSG00000026652.13	24	79	3.3
sense_intronic	AC006504.8	ENSG00000281468.1	7	23	3.3
protein_coding	CAVIN2	ENSG00000168497.4	16	52	3.3
protein_coding	PAK4	ENSG00000130669.17	4	13	3.3
protein_coding	PPP1R3E	ENSG00000235194.8	4	13	3.3
processed_pseudogene	RPS20P14	ENSG00000223803.1	4	13	3.3
protein_coding	MDFIC	ENSG00000135272.10	130	420	3.2
protein_coding	BICD1	ENSG00000151746.13	13	42	3.2
protein_coding	CUL4B	ENSG00000158290.16	176	568	3.2
protein_coding	PEX7	ENSG00000112357.12	9	29	3.2
protein_coding	PLK2	ENSG00000145632.14	146	470	3.2
protein_coding	SNAI1	ENSG00000124216.3	32	103	3.2
protein_coding	FJX1	ENSG00000179431.6	5	16	3.2
protein_coding	ALKBH1	ENSG00000100601.9	5	16	3.2
protein_coding	SFRP1	ENSG00000104332.11	177	564	3.2
protein_coding	JUP	ENSG00000173801.16	27	86	3.2
protein_coding	MAP3K4	ENSG00000085511.19	23	73	3.2
protein_coding	CHST7	ENSG00000147119.3	48	152	3.2
processed_pseudogene	AC022868.1	ENSG00000253833.1	6	19	3.2
TEC	AL136164.3	ENSG00000279289.1	6	19	3.2
protein_coding	EEPD1	ENSG00000122547.10	19	60	3.2
protein_coding	RNF144A	ENSG00000151692.14	7	22	3.1
protein_coding	ANGPTL4	ENSG00000167772.11	70	219	3.1
protein_coding	SGK1	ENSG00000118515.11	63	197	3.1
protein_coding	GABARAPL1	ENSG00000139112.10	222	694	3.1
protein_coding	INPP4B	ENSG00000109452.12	8	25	3.1
protein_coding	SEPSECS	ENSG00000109618.11	8	25	3.1
protein_coding	SYTL4	ENSG00000102362.15	8	25	3.1
protein_coding	THAP12	ENSG00000137492.7	11	34	3.1
protein_coding	SLC39A6	ENSG00000141424.12	570	1758	3.1
protein_coding	ATG4C	ENSG00000125703.14	12	37	3.1
protein_coding	ZNF143	ENSG00000166478.9	14	43	3.1
protein_coding	SF3B3	ENSG00000189091.12	87	267	3.1
protein_coding	CCDC91	ENSG00000123106.10	86	263	3.1
protein_coding	PWWP2A	ENSG00000170234.12	21	64	3
protein_coding	DIRAS3	ENSG00000162595.6	21	64	3
protein_coding	IRX3	ENSG00000177508.11	64	195	3
protein_coding	PMEPA1	ENSG00000124225.15	75	227	3
protein_coding	INHBA	ENSG00000122641.10	640	1933	3
protein_coding	MMP11	ENSG00000099953.9	30	90	3
protein_coding	FAM13C	ENSG00000148541.12	23	69	3
protein_coding	C9orf64	ENSG00000165118.14	10	30	3
protein_coding	DNAL1	ENSG00000119661.14	10	30	3
protein_coding	TBC1D9	ENSG00000109436.7	10	30	3

protein_coding	MOCS3	ENSG00000124217.4	8	24	3
protein_coding	ALG11	ENSG00000253710.3	5	15	3
protein_coding	PDCL3	ENSG00000115539.13	5	15	3
protein_coding	DGKE	ENSG00000153933.9	5	15	3
lincRNA	AC145207.5	ENSG00000263731.1	5	15	3
antisense	AL441992.1	ENSG00000223478.1	5	15	3
protein_coding	SMCO4	ENSG00000166002.6	5	15	3
protein_coding	ZNRF3	ENSG00000183579.15	4	12	3
protein_coding	RALGAPA2	ENSG00000188559.14	4	12	3
protein_coding	LMTK2	ENSG00000164715.5	4	12	3
lincRNA	LINC01081	ENSG00000268754.3	4	12	3
protein_coding	SPECC1	ENSG00000128487.16	4	12	3
protein_coding	DAPK2	ENSG00000035664.11	4	12	3
protein_coding	C12orf66	ENSG00000174206.12	4	12	3
protein_coding	SLC19A2	ENSG00000117479.13	3	9	3
lincRNA	BX284668.5	ENSG00000238142.1	3	9	3
antisense	NEXN-AS1	ENSG00000235927.4	3	9	3
protein_coding	ZNF613	ENSG00000176024.17	3	9	3
antisense	AC006333.2	ENSG00000272686.1	3	9	3
Mt_tRNA	MT-TI	ENSG00000210100.1	3	9	3
protein_coding	BNIP1	ENSG00000113734.17	3	9	3
lincRNA	RAB30-AS1	ENSG00000246067.7	3	9	3
protein_coding	KIF20A	ENSG00000112984.11	3	9	3
protein_coding	C1QTNF2	ENSG00000145861.7	3	9	3
antisense	AC002456.1	ENSG00000223969.5	2	6	3
protein_coding	CFAP300	ENSG00000137691.12	2	6	3
protein_coding	GFPT2	ENSG00000131459.12	2	6	3
protein_coding	B3GNTL1	ENSG00000175711.8	2	6	3
antisense	AC083964.1	ENSG00000272293.1	2	6	3
protein_coding	ICAM1	ENSG00000090339.8	2	6	3
protein_coding	ARHGAP39	ENSG00000147799.11	2	6	3
antisense	PXN-AS1	ENSG00000255857.5	2	6	3
protein_coding	ODF3B	ENSG00000177989.13	2	6	3
processed_pseudogene	AC079140.2	ENSG00000250321.1	2	6	3
lincRNA	AC107068.1	ENSG00000259959.1	2	6	3
protein_coding	ASB13	ENSG00000196372.12	2	6	3
protein_coding	S100A2	ENSG00000196754.10	2	6	3
processed_pseudogene	RPL7AP30	ENSG00000241741.1	2	6	3
protein_coding	C6orf132	ENSG00000188112.8	2	6	3
processed_pseudogene	RPL22P1	ENSG00000213178.3	2	6	3
protein_coding	ZNF90	ENSG00000213988.10	2	6	3
protein_coding	SLX4	ENSG00000188827.10	2	6	3
protein_coding	C16orf86	ENSG00000159761.14	2	6	3
protein_coding	GGN	ENSG00000179168.14	2	6	3

bidirectional_promoter_lncRNA	AL132857.2	ENSG00000283098.1	2	6	3
protein_coding	ELN	ENSG00000049540.16	2	6	3
processed_pseudogene	RPL24P2	ENSG00000235065.1	2	6	3
protein_coding	SOX6	ENSG00000110693.17	2	6	3
lincRNA	AC003092.1	ENSG00000236453.5	1	3	3
TEC	AC093908.1	ENSG00000280219.1	1	3	3
lincRNA	AL391056.1	ENSG00000227619.1	1	3	3
transcribed_unprocessed_pseudogene	CCDC144B	ENSG00000154874.15	1	3	3
processed_pseudogene	RPL13AP25	ENSG00000136149.6	1	3	3
sense_overlapping	AC004656.1	ENSG00000260822.1	1	3	3
protein_coding	TMEM255B	ENSG00000184497.12	1	3	3
processed_pseudogene	HSP90AB3P	ENSG00000183199.6	1	3	3
lincRNA	MIR503HG	ENSG00000223749.9	1	3	3
protein_coding	GVQW3	ENSG00000179240.9	1	3	3
lincRNA	AC092687.3	ENSG00000272275.1	1	3	3
protein_coding	HMGN4	ENSG00000182952.4	1	3	3
protein_coding	MAP3K10	ENSG00000130758.7	1	3	3
processed_transcript	TOB1-AS1	ENSG00000229980.4	1	3	3
antisense	POC1B-AS1	ENSG00000270344.2	1	3	3
transcribed_processed_pseudogene	RPS12P26	ENSG00000225193.5	1	3	3
transcribed_unprocessed_pseudogene	GTF2H2B	ENSG00000226259.10	1	3	3
protein_coding	PROSER2	ENSG00000148426.12	1	3	3
protein_coding	DENND6B	ENSG00000205593.11	1	3	3
protein_coding	CCDC57	ENSG00000176155.18	1	3	3
protein_coding	PBX2	ENSG00000204304.11	1	3	3
lincRNA	AC026304.1	ENSG00000268129.1	1	3	3
lincRNA	AL139220.2	ENSG00000230615.6	1	3	3
protein_coding	PTGER1	ENSG00000160951.3	1	3	3
protein_coding	SHANK3	ENSG00000251322.7	1	3	3
protein_coding	DLL3	ENSG00000090932.10	1	3	3
TEC	AL136164.4	ENSG00000279312.1	1	3	3
processed_pseudogene	RPL7P1	ENSG00000214485.6	1	3	3
processed_pseudogene	EEF1DP1	ENSG00000228887.3	1	3	3
antisense	AC022364.1	ENSG00000247934.4	1	3	3
lincRNA	AC108062.1	ENSG00000251432.6	1	3	3
lincRNA	AC009237.14	ENSG00000272913.1	1	3	3
protein_coding	HSD11B1	ENSG00000117594.9	1	3	3
protein_coding	PSG4	ENSG00000243137.7	1	3	3
TEC	AC079331.1	ENSG00000279361.1	1	3	3

protein_coding	OXT	ENSG00000101405.3	1	3	3
protein_coding	PLEK2	ENSG00000100558.8	1	3	3
3prime_overlapping_ncRNA	AC011472.2	ENSG00000267174.5	1	3	3
processed_pseudogene	RPS26P8	ENSG00000204652.6	1	3	3
protein_coding	AC005726.1	ENSG00000258472.8	1	3	3
protein_coding	SPAG4	ENSG00000061656.9	1	3	3
lincRNA	YTHDF3-AS1	ENSG00000270673.1	1	3	3
sense_intronic	AL844908.2	ENSG00000273027.1	1	3	3
sense_intronic	NPTN-IT1	ENSG00000281183.1	1	3	3
lincRNA	AC019131.2	ENSG00000272777.1	1	3	3
antisense	AC007773.1	ENSG00000267213.4	1	3	3
protein_coding	LRRC37A2	ENSG00000238083.7	1	3	3
protein_coding	APOBEC3H	ENSG00000100298.15	1	3	3
protein_coding	TIAF1	ENSG00000221995.5	1	3	3
lincRNA	AL512329.2	ENSG00000272279.1	1	3	3
protein_coding	GDF7	ENSG00000143869.6	1	3	3
protein_coding	ACTL10	ENSG00000182584.4	1	3	3
sense_intronic	AC022467.1	ENSG00000274654.1	1	3	3
lincRNA	AL606491.1	ENSG00000225643.1	1	3	3
antisense	AC016876.3	ENSG00000276384.1	1	3	3
protein_coding	PRSS36	ENSG00000178226.10	1	3	3
transcribed_unprocessed_pseudogene	AC106795.1	ENSG00000170089.15	1	3	3
protein_coding	FZD5	ENSG00000163251.3	1	3	3
antisense	ZNF337-AS1	ENSG00000213742.6	1	3	3
antisense	ENTPD1-AS1	ENSG00000226688.6	1	3	3
protein_coding	KRT15	ENSG00000171346.15	1	3	3
lincRNA	AC006058.1	ENSG00000261786.1	1	3	3
protein_coding	NGEF	ENSG00000066248.14	1	3	3
processed_pseudogene	AC008038.1	ENSG00000283041.1	1	3	3
sense_intronic	AC068792.1	ENSG00000275769.1	1	3	3
protein_coding	ADHFE1	ENSG00000147576.16	1	3	3
protein_coding	PMCH	ENSG00000183395.4	1	3	3
processed_pseudogene	AC106872.2	ENSG00000240674.1	1	3	3
protein_coding	UBAP1L	ENSG00000246922.8	1	3	3
transcribed_unprocessed_pseudogene	LINC00933	ENSG00000259728.5	1	3	3
antisense	AL354732.1	ENSG00000231407.5	1	3	3
lincRNA	LINC01693	ENSG00000227764.1	1	3	3
protein_coding	IL10RB	ENSG00000243646.9	1	3	3
protein_coding	LRRC69	ENSG00000214954.8	1	3	3
lincRNA	LINC02454	ENSG00000256268.1	1	3	3

processed_pseudogene	AL049873.1	ENSG00000213598.4	1	3	3
protein_coding	CHKB	ENSG00000100288.19	1	3	3
antisense	AC138150.1	ENSG00000224505.2	1	3	3
lincRNA	LINC01091	ENSG00000249464.5	1	3	3
transcribed_unprocessed_pseudogene	FAM86B3P	ENSG00000173295.7	1	3	3
protein_coding	ZNF837	ENSG00000152475.6	1	3	3
lincRNA	UBAC2-AS1	ENSG00000228889.6	1	3	3
protein_coding	AVIL	ENSG00000135407.10	1	3	3
protein_coding	UBQLN4	ENSG00000160803.7	1	3	3
protein_coding	HSF2BP	ENSG00000160207.8	1	3	3
antisense	AC015982.1	ENSG00000272606.1	1	3	3
protein_coding	HIST1H2AE	ENSG00000277075.2	1	3	3
antisense	AC139530.1	ENSG00000262049.1	1	3	3
protein_coding	VAMP8	ENSG00000118640.10	1	3	3
lincRNA	LINC00115	ENSG00000225880.5	1	3	3
processed_pseudogene	SNRPGP2	ENSG00000264350.1	1	3	3
protein_coding	ACOT11	ENSG00000162390.17	1	3	3
transcribed_unprocessed_pseudogene	PMS2CL	ENSG00000187953.10	1	3	3
sense_intronic	AC087501.4	ENSG00000271851.1	1	3	3
lincRNA	LINC01521	ENSG00000213888.3	1	3	3
lincRNA	AC055713.1	ENSG00000247373.3	1	3	3
protein_coding	PLB1	ENSG00000163803.12	1	3	3
antisense	AL121672.3	ENSG00000273289.1	1	3	3
antisense	AC092376.2	ENSG00000277954.1	1	3	3
protein_coding	FAM71D	ENSG00000172717.16	1	3	3
lincRNA	AC091965.4	ENSG00000272049.1	1	3	3
lincRNA	LINC02202	ENSG00000245812.2	1	3	3
lincRNA	AL589684.1	ENSG00000223811.1	1	3	3
lincRNA	LINC00900	ENSG00000246100.3	1	3	3
lincRNA	AC016705.2	ENSG00000259495.2	1	3	3
processed_transcript	SPAG5-AS1	ENSG00000227543.4	1	3	3
protein_coding	IL12A	ENSG00000168811.6	1	3	3
protein_coding	PGA5	ENSG00000256713.7	1	3	3
protein_coding	CARNS1	ENSG00000172508.10	1	3	3
sense_overlapping	AC004917.1	ENSG00000243797.6	1	3	3
misc_RNA	RNY1	ENSG00000201098.1	1	3	3
processed_pseudogene	RPL4P4	ENSG00000229638.1	1	3	3
lincRNA	AL117379.1	ENSG00000273759.1	1	3	3
protein_coding	PPP2R1B	ENSG00000137713.15	67	200	3
protein_coding	ZNF217	ENSG00000171940.13	42	125	3
protein_coding	HIF1A	ENSG00000100644.16	342	1017	3

protein_coding	SNRK	ENSG00000163788.13	27	80	3
protein_coding	MOCOS	ENSG00000075643.5	88	260	3
protein_coding	PTMA	ENSG00000187514.16	795	2341	2.9
protein_coding	P3H2	ENSG00000090530.9	16	47	2.9
protein_coding	CEBPD	ENSG00000221869.4	263	772	2.9
protein_coding	ITGA4	ENSG00000115232.13	14	41	2.9
protein_coding	CSGALNAC	ENSG00000169826.7	55	161	2.9
protein_coding	TRPA1	ENSG00000104321.10	26	76	2.9
antisense	AC020915.3	ENSG00000268516.2	13	38	2.9
protein_coding	CSGALNAC	ENSG00000147408.14	261	762	2.9
TEC	AP000892.3	ENSG00000280143.1	10	29	2.9
protein_coding	SOCS2	ENSG00000120833.13	129	374	2.9
protein_coding	KCNJ2	ENSG00000123700.4	9	26	2.9
protein_coding	LYPD1	ENSG00000150551.10	80	231	2.9
protein_coding	CHD1L	ENSG00000131778.18	16	46	2.9
protein_coding	LDLR	ENSG00000130164.13	16	46	2.9
protein_coding	NRF1	ENSG00000106459.14	8	23	2.9
protein_coding	NCEH1	ENSG00000144959.9	31	89	2.9
protein_coding	MED11	ENSG00000161920.9	22	63	2.9
protein_coding	KCNIP3	ENSG00000115041.12	22	63	2.9
protein_coding	ACTR6	ENSG00000075089.9	14	40	2.9
antisense	STXBP5-AS1	ENSG00000233452.6	14	40	2.9
protein_coding	CCDC136	ENSG00000128596.16	7	20	2.9
protein_coding	AZIN2	ENSG00000142920.16	6	17	2.8
protein_coding	BMF	ENSG00000104081.13	6	17	2.8
protein_coding	APBA2	ENSG00000034053.14	11	31	2.8
protein_coding	CCDC152	ENSG00000198865.9	11	31	2.8
protein_coding	ABCC4	ENSG00000125257.14	216	608	2.8
protein_coding	GLCE	ENSG00000138604.9	16	45	2.8
protein_coding	TMTC4	ENSG00000125247.15	30	84	2.8
protein_coding	PCDHGA6	ENSG00000253731.2	10	28	2.8
protein_coding	ARL¬†6	ENSG00000113966.9	5	14	2.8
protein_coding	GTF2IRD2	ENSG00000196275.13	5	14	2.8
protein_coding	MARC1	ENSG00000186205.12	5	14	2.8
lincRNA	MIR99AHG	ENSG00000215386.12	5	14	2.8
protein_coding	ADGRG1	ENSG00000205336.11	5	14	2.8
protein_coding	PKNOX1	ENSG00000160199.14	19	53	2.8
protein_coding	GCLC	ENSG00000001084.12	37	103	2.8
protein_coding	SMPDL3A	ENSG00000172594.12	50	139	2.8
protein_coding	DNAAF2	ENSG00000165506.14	9	25	2.8
protein_coding	LTO1	ENSG00000149716.12	13	36	2.8
protein_coding	NHSL1	ENSG00000135540.11	13	36	2.8
protein_coding	GON4L	ENSG00000116580.18	21	58	2.8
protein_coding	ST3GAL1	ENSG00000008513.15	88	242	2.8

protein_coding	AOX1	ENSG00000138356.13	40	110	2.8
protein_coding	KIAA0753	ENSG00000198920.9	8	22	2.8
protein_coding	BAMBI	ENSG00000095739.10	8	22	2.8
protein_coding	ISM1	ENSG00000101230.5	4	11	2.8
protein_coding	CLDN15	ENSG00000106404.13	4	11	2.8
protein_coding	PLSCR1	ENSG00000188313.12	30	82	2.7
protein_coding	ADO	ENSG00000181915.4	26	71	2.7
protein_coding	PHACTR2	ENSG00000112419.14	193	524	2.7
protein_coding	TM9SF1	ENSG00000100926.14	7	19	2.7
protein_coding	SS18L1	ENSG00000184402.14	7	19	2.7
protein_coding	ZNF610	ENSG00000167554.14	7	19	2.7
lincRNA	AC008124.1	ENSG00000273015.2	7	19	2.7
protein_coding	SIRPA	ENSG00000198053.11	44	119	2.7
protein_coding	DYRK3	ENSG00000143479.16	10	27	2.7
protein_coding	PDE4D	ENSG00000113448.18	29	78	2.7
protein_coding	ITGB8	ENSG00000105855.9	16	43	2.7
protein_coding	PRKAR2B	ENSG00000005249.12	44	118	2.7
protein_coding	MCTP2	ENSG00000140563.14	33	88	2.7
protein_coding	RUNX3	ENSG00000020633.18	27	72	2.7
protein_coding	NPAS2	ENSG00000170485.16	24	64	2.7
protein_coding	CABLES1	ENSG00000134508.12	24	64	2.7
protein_coding	TCTEX1D2	ENSG00000213123.10	15	40	2.7
protein_coding	SYNE2	ENSG00000054654.16	9	24	2.7
protein_coding	ZNF829	ENSG00000185869.14	9	24	2.7
protein_coding	PUS7	ENSG00000091127.13	6	16	2.7
protein_coding	NFKB2	ENSG00000077150.18	6	16	2.7
protein_coding	LIMD2	ENSG00000136490.8	6	16	2.7
protein_coding	ZNF138	ENSG00000197008.9	6	16	2.7
protein_coding	CORO7	ENSG00000262246.5	3	8	2.7
antisense	TTN-AS1	ENSG00000237298.9	3	8	2.7
protein_coding	CCNJL	ENSG00000135083.15	3	8	2.7
protein_coding	ZFP41	ENSG00000181638.17	3	8	2.7
sense_intronic	AC006141.1	ENSG00000264895.1	3	8	2.7
protein_coding	NSUN6	ENSG00000241058.3	3	8	2.7
transcribed_unprocessed_pseudogene	LINC00674	ENSG00000237854.3	3	8	2.7
protein_coding	FBLN7	ENSG00000144152.12	3	8	2.7
protein_coding	FBXO6	ENSG00000116663.10	3	8	2.7
protein_coding	GMIP	ENSG00000089639.10	3	8	2.7
protein_coding	C5AR1	ENSG00000197405.7	3	8	2.7
protein_coding	GRK3	ENSG00000100077.14	3	8	2.7
protein_coding	ZNF433	ENSG00000197647.11	3	8	2.7
protein_coding	RFC5	ENSG00000111445.13	3	8	2.7

protein_coding	LRRC66	ENSG00000188993.3	3	8	2.7
protein_coding	MT-ND5	ENSG00000198786.2	1345	3576	2.7
protein_coding	STARD13	ENSG00000133121.20	90	239	2.7
protein_coding	LCA5	ENSG00000135338.13	29	77	2.7
protein_coding	HSPB3	ENSG00000169271.2	23	61	2.7
protein_coding	GOLIM4	ENSG00000173905.8	379	1005	2.7
protein_coding	THBS2	ENSG00000186340.15	1796	4761	2.7
protein_coding	LRRC8C	ENSG00000171488.14	14	37	2.6
protein_coding	FAM167A	ENSG00000154319.15	14	37	2.6
protein_coding	NET1	ENSG00000173848.18	44	116	2.6
protein_coding	EFNB2	ENSG00000125266.7	11	29	2.6
protein_coding	CASTOR3	ENSG00000239521.8	11	29	2.6
protein_coding	SYNJ2	ENSG00000078269.14	194	510	2.6
protein_coding	NAMPT	ENSG00000105835.11	51	134	2.6
protein_coding	ITPRID2	ENSG00000138434.16	323	848	2.6
protein_coding	RAPH1	ENSG00000173166.17	40	105	2.6
protein_coding	NXT2	ENSG00000101888.11	16	42	2.6
protein_coding	CLCF1	ENSG00000175505.10	8	21	2.6
protein_coding	ULK3	ENSG00000140474.13	8	21	2.6
protein_coding	IL4R	ENSG00000077238.13	37	97	2.6
protein_coding	OSBPL10	ENSG00000144645.13	29	76	2.6
protein_coding	AGL	ENSG00000162688.16	39	102	2.6
protein_coding	PLCB1	ENSG00000182621.17	36	94	2.6
protein_coding	RBM28	ENSG00000106344.8	18	47	2.6
protein_coding	PCNX1	ENSG00000100731.15	89	232	2.6
protein_coding	ZFYVE9	ENSG00000157077.14	15	39	2.6
protein_coding	CCND3	ENSG00000112576.12	15	39	2.6
lincRNA	AL356515.1	ENSG00000276527.1	5	13	2.6
protein_coding	VASH1	ENSG00000071246.10	5	13	2.6
protein_coding	LYSMD4	ENSG00000183060.15	5	13	2.6
protein_coding	VPS13D	ENSG00000048707.15	27	70	2.6
protein_coding	SEMA4B	ENSG00000185033.14	17	44	2.6
protein_coding	CDKL5	ENSG00000008086.11	12	31	2.6
protein_coding	KIAA0355	ENSG00000166398.12	43	111	2.6
protein_coding	FGF2	ENSG00000138685.15	152	392	2.6
protein_coding	BTN2A1	ENSG00000112763.16	21	54	2.6
protein_coding	IQCG	ENSG00000114473.13	7	18	2.6
protein_coding	EMC9	ENSG00000100908.13	7	18	2.6
protein_coding	OSGEPL1	ENSG00000128694.11	7	18	2.6
protein_coding	ZNF142	ENSG00000115568.15	7	18	2.6
protein_coding	PRPF40B	ENSG00000110844.13	7	18	2.6
protein_coding	ALG10B	ENSG00000175548.8	7	18	2.6
protein_coding	USP28	ENSG00000048028.11	16	41	2.6
protein_coding	ZC3H12C	ENSG00000149289.10	25	64	2.6

protein_coding	CDR2	ENSG00000140743.7	43	110	2.6
protein_coding	TMEM56	ENSG00000152078.9	9	23	2.6
protein_coding	LRRC6	ENSG00000129295.8	9	23	2.6
protein_coding	MBD4	ENSG00000129071.9	128	327	2.6
protein_coding	ARHGAP6	ENSG00000047648.21	22	56	2.5
protein_coding	SYCP2	ENSG00000196074.12	11	28	2.5
protein_coding	FOXC1	ENSG00000054598.7	54	137	2.5
protein_coding	TGM2	ENSG00000198959.11	224	567	2.5
protein_coding	KLHL2	ENSG00000109466.13	17	43	2.5
protein_coding	TMEM178B	ENSG00000261115.5	55	139	2.5
protein_coding	ZCCHC2	ENSG00000141664.9	19	48	2.5
protein_coding	SDF2L1	ENSG00000128228.4	14	35	2.5
protein_coding	MTHFS	ENSG00000136371.10	12	30	2.5
protein_coding	NSUN4	ENSG00000117481.10	10	25	2.5
protein_coding	B3GALT4	ENSG00000235863.3	10	25	2.5
protein_coding	TAZ	ENSG00000102125.15	10	25	2.5
protein_coding	SLC5A6	ENSG00000138074.14	6	15	2.5
protein_coding	TRPC4	ENSG00000133107.14	6	15	2.5
protein_coding	VRK1	ENSG00000100749.7	6	15	2.5
protein_coding	SEMA6C	ENSG00000143434.15	6	15	2.5
protein_coding	ZNF502	ENSG00000196653.11	6	15	2.5
sense_intronic	AL606834.2	ENSG00000270062.1	4	10	2.5
protein_coding	PDE1C	ENSG00000154678.16	4	10	2.5
protein_coding	ZNF597	ENSG00000167981.6	4	10	2.5
protein_coding	BCDIN3D	ENSG00000186666.5	4	10	2.5
protein_coding	TRPC6	ENSG00000137672.12	4	10	2.5
protein_coding	MSTO1	ENSG00000125459.15	4	10	2.5
protein_coding	ZNF133	ENSG00000125846.15	4	10	2.5
antisense	EXOC3-AS1	ENSG00000221990.4	4	10	2.5
protein_coding	DEPDC5	ENSG00000100150.17	4	10	2.5
protein_coding	FAM24B	ENSG00000213185.6	4	10	2.5
protein_coding	BCL7A	ENSG00000110987.8	4	10	2.5
protein_coding	KIF23	ENSG00000137807.15	2	5	2.5
protein_coding	PPAN	ENSG00000130810.19	2	5	2.5
protein_coding	MFSD4B	ENSG00000173214.5	2	5	2.5
protein_coding	RAB20	ENSG00000139832.4	2	5	2.5
protein_coding	ADPRHL1	ENSG00000153531.13	2	5	2.5
processed_pseudogene	RPS27AP12	ENSG00000225224.1	2	5	2.5
protein_coding	SPATA2L	ENSG00000158792.15	2	5	2.5
lincRNA	AC027575.2	ENSG00000278107.1	2	5	2.5
protein_coding	PAQR4	ENSG00000162073.13	2	5	2.5
protein_coding	HIST2H2AC	ENSG00000184260.5	2	5	2.5
protein_coding	ZNF707	ENSG00000181135.15	2	5	2.5
protein_coding	C11orf87	ENSG00000185742.6	2	5	2.5

TEC	AP002847.1	ENSG00000279138.1	2	5	2.5
protein_coding	ZNF554	ENSG00000172006.11	2	5	2.5
sense_intronic	AL133520.1	ENSG00000273893.1	2	5	2.5
protein_coding	CCDC40	ENSG00000141519.14	2	5	2.5
antisense	THAP7-AS1	ENSG00000230513.1	2	5	2.5
processed_transcript	AC060766.7	ENSG00000267745.1	2	5	2.5
antisense	AL162458.1	ENSG00000285796.1	2	5	2.5
sense_overlapping	AL390195.2	ENSG00000260948.1	2	5	2.5
lincRNA	AL133406.3	ENSG00000272102.1	2	5	2.5
lincRNA	AL391244.3	ENSG00000272455.1	2	5	2.5
antisense	AL022328.4	ENSG00000273253.2	2	5	2.5
lincRNA	LINC01094	ENSG00000251442.5	2	5	2.5
protein_coding	NBPF26	ENSG00000273136.6	2	5	2.5
antisense	ENTPD3-AS1	ENSG00000223797.5	2	5	2.5
protein_coding	CCDC61	ENSG00000104983.8	2	5	2.5
protein_coding	PRKCH	ENSG00000027075.14	2	5	2.5
processed_pseudogene	ZNF204P	ENSG00000204789.4	2	5	2.5
protein_coding	GARNL3	ENSG00000136895.18	2	5	2.5
protein_coding	ABCA3	ENSG00000167972.13	2	5	2.5
protein_coding	LEKR1	ENSG00000197980.12	2	5	2.5
processed_transcript	ARMCX5-GPRASP2	ENSG00000271147.7	2	5	2.5
protein_coding	ZNF816	ENSG00000180257.13	2	5	2.5
protein_coding	SLC18B1	ENSG00000146409.11	2	5	2.5
processed_pseudogene	RPL30P4	ENSG00000237676.1	2	5	2.5
protein_coding	CA13	ENSG00000185015.7	2	5	2.5
protein_coding	AMIGO1	ENSG00000181754.6	2	5	2.5
protein_coding	ZNF239	ENSG00000196793.13	2	5	2.5
processed_pseudogene	RPL7AP66	ENSG00000175886.10	2	5	2.5
protein_coding	CCDC150	ENSG00000144395.17	2	5	2.5
TEC	AC008555.8	ENSG00000280106.1	2	5	2.5
protein_coding	GPATCH8	ENSG00000186566.12	62	154	2.5
protein_coding	DHRS3	ENSG00000162496.8	19	47	2.5
protein_coding	FOXF1	ENSG00000103241.6	174	430	2.5
protein_coding	CMC2	ENSG00000103121.8	17	42	2.5
protein_coding	ARRDC3	ENSG00000113369.8	100	247	2.5
protein_coding	ECT2	ENSG00000114346.13	32	79	2.5
protein_coding	C1GALT1C1	ENSG00000171155.7	28	69	2.5
protein_coding	WBP1L	ENSG00000166272.17	104	256	2.5
protein_coding	CLIP2	ENSG00000106665.15	13	32	2.5
protein_coding	EXPH5	ENSG00000110723.11	13	32	2.5
protein_coding	STAMBPL1	ENSG00000138134.11	24	59	2.5
protein_coding	TNFRSF21	ENSG00000146072.6	33	81	2.5

protein_coding	CCDC58	ENSG00000160124.9	11	27	2.5
protein_coding	CD36	ENSG00000135218.18	11	27	2.5
protein_coding	BBS12	ENSG00000181004.9	11	27	2.5
protein_coding	ZNF506	ENSG00000081665.13	11	27	2.5
protein_coding	BBS7	ENSG00000138686.9	31	76	2.5
protein_coding	BRCA2	ENSG00000139618.14	9	22	2.4
protein_coding	RPS6KA5	ENSG00000100784.11	9	22	2.4
protein_coding	SLC26A6	ENSG00000225697.12	9	22	2.4
protein_coding	ERI3	ENSG00000117419.15	9	22	2.4
protein_coding	KCNAB2	ENSG00000069424.14	9	22	2.4
protein_coding	BDP1	ENSG00000145734.18	86	210	2.4
protein_coding	GPR161	ENSG00000143147.14	39	95	2.4
protein_coding	SERPINB8	ENSG00000166401.14	7	17	2.4
protein_coding	FAM200A	ENSG00000221909.2	7	17	2.4
protein_coding	ADM	ENSG00000148926.9	265	643	2.4
protein_coding	TFAP2C	ENSG00000087510.6	269	651	2.4
protein_coding	DIRC2	ENSG00000138463.8	31	75	2.4
protein_coding	ITSN1	ENSG00000205726.14	60	145	2.4
protein_coding	B3GAT3	ENSG00000149541.9	12	29	2.4
protein_coding	NDRG1	ENSG00000104419.14	193	466	2.4
protein_coding	ZFYVE27	ENSG00000155256.17	17	41	2.4
protein_coding	C16orf45	ENSG00000166780.10	211	508	2.4
protein_coding	TGIF1	ENSG00000177426.20	32	77	2.4
protein_coding	MT-ATP8	ENSG00000228253.1	1271	3056	2.4
protein_coding	B3GLCT	ENSG00000187676.7	25	60	2.4
protein_coding	GAS8	ENSG00000141013.16	10	24	2.4
protein_coding	ATPAF2	ENSG00000171953.15	10	24	2.4
protein_coding	GBP4	ENSG00000162654.8	5	12	2.4
protein_coding	PHLDB3	ENSG00000176531.10	5	12	2.4
protein_coding	SLC22A5	ENSG00000197375.12	5	12	2.4
protein_coding	TMEM234	ENSG00000160055.19	5	12	2.4
protein_coding	MIF	ENSG00000240972.1	5	12	2.4
protein_coding	GTF2H2	ENSG00000145736.14	5	12	2.4
protein_coding	MACROD1	ENSG00000133315.10	5	12	2.4
protein_coding	HIST3H2A	ENSG00000181218.5	5	12	2.4
protein_coding	ZNF879	ENSG00000234284.6	5	12	2.4
protein_coding	AL031777.3	ENSG00000282988.2	5	12	2.4
protein_coding	HOOK2	ENSG00000095066.11	13	31	2.4
protein_coding	RNF219	ENSG00000152193.7	13	31	2.4
protein_coding	FKRP	ENSG00000181027.10	21	50	2.4
protein_coding	ZNF720	ENSG00000197302.10	21	50	2.4
protein_coding	VCAN	ENSG00000038427.15	424	1009	2.4
protein_coding	PLCL2	ENSG00000154822.17	24	57	2.4
processed_transcript	LINC01128	ENSG00000228794.8	16	38	2.4

protein_coding	KATNBL1	ENSG00000134152.10	16	38	2.4
protein_coding	SETD9	ENSG00000155542.11	8	19	2.4
protein_coding	FAM107B	ENSG00000065809.13	113	268	2.4
protein_coding	TJAP1	ENSG00000137221.14	19	45	2.4
protein_coding	ALDOC	ENSG00000109107.13	11	26	2.4
protein_coding	ADCY9	ENSG00000162104.9	69	163	2.4
protein_coding	TGFBI	ENSG00000120708.16	1607	3793	2.4
protein_coding	SNAP25	ENSG00000132639.12	74	174	2.4
protein_coding	TLNRD1	ENSG00000140406.3	23	54	2.3
protein_coding	CHST11	ENSG00000171310.10	23	54	2.3
protein_coding	LIMCH1	ENSG00000064042.17	75	176	2.3
protein_coding	TYW3	ENSG00000162623.15	93	218	2.3
protein_coding	TLCD2	ENSG00000185561.9	38	89	2.3
protein_coding	SSH1	ENSG00000084112.14	41	96	2.3
protein_coding	RHOB	ENSG00000143878.9	128	299	2.3
protein_coding	INSR	ENSG00000171105.13	24	56	2.3
protein_coding	APAF1	ENSG00000120868.13	18	42	2.3
protein_coding	RGS5	ENSG00000143248.12	18	42	2.3
protein_coding	ANKRD13D	ENSG00000172932.14	15	35	2.3
protein_coding	FAIM	ENSG00000158234.12	15	35	2.3
protein_coding	AMPD3	ENSG00000133805.15	15	35	2.3
protein_coding	HRH1	ENSG00000196639.6	9	21	2.3
protein_coding	HEATR5B	ENSG00000008869.11	9	21	2.3
protein_coding	TTC33	ENSG00000113638.13	9	21	2.3
protein_coding	ATG4D	ENSG00000130734.9	9	21	2.3
lincRNA	LINC01588	ENSG00000214900.9	6	14	2.3
protein_coding	SMTN	ENSG00000183963.18	6	14	2.3
protein_coding	C8orf88	ENSG00000253250.2	6	14	2.3
unprocessed_pseudogene	MTND2P28	ENSG00000225630.1	6	14	2.3
protein_coding	TFAP2A	ENSG00000137203.11	6	14	2.3
protein_coding	ZBTB39	ENSG00000166860.2	3	7	2.3
transcribed_unitary_pseudogene	AFG3L1P	ENSG00000223959.8	3	7	2.3
protein_coding	GCNA	ENSG00000147174.11	3	7	2.3
protein_coding	ZNF587B	ENSG00000269343.6	3	7	2.3
protein_coding	HIST1H2AG	ENSG00000196787.3	3	7	2.3
protein_coding	CHMP4A	ENSG00000254505.9	3	7	2.3
protein_coding	TMEM130	ENSG00000166448.14	3	7	2.3
protein_coding	PGR	ENSG00000082175.14	3	7	2.3
protein_coding	TEAD4	ENSG00000197905.8	3	7	2.3
protein_coding	ZNF589	ENSG00000164048.13	3	7	2.3
protein_coding	AC114490.3	ENSG00000284773.1	3	7	2.3
protein_coding	LCAT	ENSG00000213398.7	3	7	2.3

sense_intronic	AC139768.1	ENSG00000278126.1	3	7	2.3
protein_coding	ARHGEF5	ENSG00000050327.14	3	7	2.3
protein_coding	SF3A2	ENSG00000104897.9	3	7	2.3
protein_coding	ULBP1	ENSG00000111981.4	3	7	2.3
protein_coding	TBCCD1	ENSG00000113838.12	3	7	2.3
protein_coding	KNL1	ENSG00000137812.19	3	7	2.3
protein_coding	EPB41L4B	ENSG00000095203.14	3	7	2.3
lincRNA	AC011374.2	ENSG00000272112.1	3	7	2.3
protein_coding	ADAMTS14	ENSG00000138316.10	3	7	2.3
protein_coding	HSPA1L	ENSG00000204390.9	3	7	2.3
protein_coding	CLTCL1	ENSG00000070371.15	3	7	2.3
protein_coding	RGMA	ENSG00000182175.14	3	7	2.3
protein_coding	FGD6	ENSG00000180263.13	3	7	2.3
protein_coding	HTR2A	ENSG00000102468.10	3	7	2.3
protein_coding	MESD	ENSG00000117899.10	241	562	2.3
protein_coding	BTAF1	ENSG00000095564.13	34	79	2.3
protein_coding	DCP2	ENSG00000172795.15	19	44	2.3
protein_coding	KCTD7	ENSG00000243335.9	19	44	2.3
protein_coding	CCDC18	ENSG00000122483.17	16	37	2.3
protein_coding	ZNF75A	ENSG00000162086.14	13	30	2.3
protein_coding	GFER	ENSG00000127554.12	13	30	2.3
protein_coding	CDK17	ENSG00000059758.7	93	214	2.3
protein_coding	MIS18BP1	ENSG00000129534.13	30	69	2.3
protein_coding	CDK5	ENSG00000164885.12	20	46	2.3
protein_coding	BBOF1	ENSG00000119636.15	10	23	2.3
protein_coding	GPKOW	ENSG00000068394.10	10	23	2.3
protein_coding	PTPN11	ENSG00000179295.17	97	223	2.3
protein_coding	N4BP2	ENSG00000078177.13	27	62	2.3
protein_coding	LRRN3	ENSG00000173114.12	27	62	2.3
protein_coding	PHKG2	ENSG00000156873.15	17	39	2.3
protein_coding	SLC16A4	ENSG00000168679.17	267	612	2.3
protein_coding	PVR	ENSG00000073008.14	24	55	2.3
protein_coding	SAT1	ENSG00000130066.16	296	677	2.3
protein_coding	ARMC5	ENSG00000140691.16	14	32	2.3
transcribed_unprocessed_pseudogene	UBE2Q2P1	ENSG00000189136.9	7	16	2.3
protein_coding	PDE3A	ENSG00000172572.6	7	16	2.3
transcribed_unprocessed_pseudogene	FRG1HP	ENSG00000276291.5	7	16	2.3
transcribed_unprocessed_pseudogene	CIDECP	ENSG00000186162.10	7	16	2.3
miRNA	MIR1244-2	ENSG00000283498.1	7	16	2.3
protein_coding	NAV2	ENSG00000166833.20	7	16	2.3

protein_coding	AP4S1	ENSG00000100478.14	7	16	2.3
TEC	AC126474.2	ENSG00000280088.1	7	16	2.3
protein_coding	BEX1	ENSG00000133169.5	50	114	2.3
protein_coding	PRUNE1	ENSG00000143363.16	18	41	2.3
protein_coding	SNCAIP	ENSG00000064692.18	138	314	2.3
protein_coding	BCL2L2	ENSG00000129473.9	48	109	2.3
protein_coding	ZBTB1	ENSG00000126804.13	37	84	2.3
lincRNA	MEG8	ENSG00000225746.11	26	59	2.3
protein_coding	SV2A	ENSG00000159164.9	60	136	2.3
protein_coding	FASTKD5	ENSG00000215251.3	30	68	2.3
protein_coding	GLDN	ENSG00000186417.13	15	34	2.3
protein_coding	ATL1	ENSG00000198513.11	19	43	2.3
protein_coding	MFSD6	ENSG00000151690.14	54	122	2.3
protein_coding	ADGRA3	ENSG00000152990.13	59	133	2.3
protein_coding	SPTSSA	ENSG00000165389.6	179	403	2.3
lincRNA	LINC02035	ENSG00000273033.2	28	63	2.3
protein_coding	CC2D1B	ENSG00000154222.14	20	45	2.3
protein_coding	SLF1	ENSG00000133302.12	8	18	2.3
protein_coding	ZNF626	ENSG00000188171.15	8	18	2.3
protein_coding	ZBTB24	ENSG00000112365.4	8	18	2.3
protein_coding	Sep-01	ENSG00000180096.11	8	18	2.3
protein_coding	KIF14	ENSG00000118193.11	4	9	2.3
protein_coding	ASPHD1	ENSG00000174939.10	4	9	2.3
protein_coding	SLC1A2	ENSG00000110436.12	4	9	2.3
protein_coding	RASIP1	ENSG00000105538.9	4	9	2.3
protein_coding	GDAP1	ENSG00000104381.12	4	9	2.3
protein_coding	Sep-03	ENSG00000100167.20	4	9	2.3
protein_coding	IBA57	ENSG00000181873.12	4	9	2.3
protein_coding	NKAPL	ENSG00000189134.3	4	9	2.3
lincRNA	AC108673.3	ENSG00000273437.1	4	9	2.3
transcribed_processed_pseudogene	PGAM1P8	ENSG00000255200.1	4	9	2.3
antisense	AC091729.3	ENSG00000229043.2	4	9	2.3
protein_coding	GPR89B	ENSG00000188092.14	4	9	2.3
protein_coding	C1orf50	ENSG00000164008.15	4	9	2.3
protein_coding	PPFIBP1	ENSG00000110841.13	142	319	2.2
protein_coding	PSMC1	ENSG00000100764.13	25	56	2.2
protein_coding	SHISAL1	ENSG00000138944.7	92	206	2.2
protein_coding	PBX1	ENSG00000185630.18	76	170	2.2
protein_coding	ASB7	ENSG00000183475.12	17	38	2.2
protein_coding	GLIS1	ENSG00000174332.5	17	38	2.2
lincRNA	LINC00667	ENSG00000263753.7	17	38	2.2
protein_coding	PRKAR1A	ENSG00000108946.14	1727	3857	2.2

protein_coding	DSP	ENSG00000096696.13	30	67	2.2
protein_coding	SLC12A4	ENSG00000124067.16	39	87	2.2
protein_coding	DUSP16	ENSG00000111266.8	13	29	2.2
protein_coding	GPC4	ENSG00000076716.8	13	29	2.2
protein_coding	ANKRD39	ENSG00000213337.8	13	29	2.2
protein_coding	LUZP1	ENSG00000169641.13	85	189	2.2
protein_coding	ZNF638	ENSG00000075292.18	81	180	2.2
TEC	AL662795.2	ENSG00000280128.1	18	40	2.2
protein_coding	TAF8	ENSG00000137413.15	18	40	2.2
protein_coding	TMEM268	ENSG00000157693.14	9	20	2.2
protein_coding	SDHAF1	ENSG00000205138.3	9	20	2.2
protein_coding	FOXO3	ENSG00000118689.14	113	251	2.2
protein_coding	SATB1	ENSG00000182568.16	41	91	2.2
protein_coding	AMD¬†1	ENSG00000123505.15	73	162	2.2
protein_coding	FKBP8	ENSG00000105701.15	28	62	2.2
protein_coding	TMEM206	ENSG00000065600.12	14	31	2.2
protein_coding	RORA	ENSG00000069667.15	19	42	2.2
protein_coding	TMEM107	ENSG00000179029.14	19	42	2.2
protein_coding	HMGXB3	ENSG00000113716.12	24	53	2.2
protein_coding	PRPF4	ENSG00000136875.12	24	53	2.2
protein_coding	PCGF5	ENSG00000180628.14	85	187	2.2
protein_coding	ZBTB8A	ENSG00000160062.14	30	66	2.2
protein_coding	DLL1	ENSG00000198719.8	15	33	2.2
protein_coding	RAMAC	ENSG00000169612.3	10	22	2.2
protein_coding	HSPBP1	ENSG00000133265.10	10	22	2.2
protein_coding	PRRT2	ENSG00000167371.19	10	22	2.2
protein_coding	CCDC126	ENSG00000169193.11	10	22	2.2
protein_coding	MCAM	ENSG00000076706.16	5	11	2.2
protein_coding	JRKL	ENSG00000183340.6	5	11	2.2
protein_coding	PNP	ENSG00000198805.11	5	11	2.2
protein_coding	ZNF678	ENSG00000181450.17	5	11	2.2
transcribed_unprocessed_pseudogene	ADAMTS7P4	ENSG00000218052.5	5	11	2.2
protein_coding	HENMT1	ENSG00000162639.15	5	11	2.2
protein_coding	KPTN	ENSG00000118162.13	5	11	2.2
lincRNA	AL137026.1	ENSG00000229116.2	5	11	2.2
antisense	RAP2C-AS1	ENSG00000232160.6	5	11	2.2
protein_coding	UBA5	ENSG00000081307.12	66	145	2.2
protein_coding	WIPI1	ENSG00000070540.12	299	656	2.2
protein_coding	RNASEL	ENSG00000135828.11	21	46	2.2
protein_coding	ITGA2	ENSG00000164171.10	235	514	2.2
protein_coding	RASSF8	ENSG00000123094.15	188	411	2.2
protein_coding	DHRS7B	ENSG00000109016.17	27	59	2.2

protein_coding	RAI14	ENSG00000039560.13	272	594	2.2
protein_coding	NUP160	ENSG00000030066.13	55	120	2.2
protein_coding	SLC9B2	ENSG00000164038.14	22	48	2.2
protein_coding	POM121C	ENSG00000272391.5	11	24	2.2
protein_coding	MCEE	ENSG00000124370.10	11	24	2.2
protein_coding	SQLE	ENSG00000104549.11	28	61	2.2
protein_coding	DUSP4	ENSG00000120875.8	17	37	2.2
protein_coding	BANP	ENSG00000172530.20	17	37	2.2
protein_coding	ENDOG	ENSG00000167136.6	17	37	2.2
protein_coding	ITGB1	ENSG00000150093.18	838	1822	2.2
protein_coding	HSPA2	ENSG00000126803.9	23	50	2.2
protein_coding	ZNF33B	ENSG00000196693.14	23	50	2.2
protein_coding	TWIST2	ENSG00000233608.3	242	526	2.2
protein_coding	EGLN1	ENSG00000135766.8	52	113	2.2
protein_coding	RALGAPA1	ENSG00000174373.16	12	26	2.2
protein_coding	MCOLN1	ENSG00000090674.15	12	26	2.2
protein_coding	C22orf46	ENSG00000184208.10	12	26	2.2
protein_coding	GCFC2	ENSG00000005436.13	6	13	2.2
protein_coding	ADAM23	ENSG00000114948.12	6	13	2.2
protein_coding	CENPQ	ENSG00000031691.6	6	13	2.2
protein_coding	GRAMD1C	ENSG00000178075.19	6	13	2.2
protein_coding	ZNF26	ENSG00000198393.7	6	13	2.2
antisense	GAS6-AS1	ENSG00000233695.2	6	13	2.2
protein_coding	TOP¬†1	ENSG00000198900.5	170	368	2.2
protein_coding	TTC39B	ENSG00000155158.20	43	93	2.2
protein_coding	HDAC5	ENSG00000108840.15	68	147	2.2
protein_coding	SLC4A4	ENSG00000080493.16	62	134	2.2
lincRNA	MALAT1	ENSG00000251562.8	14990	32366	2.2
protein_coding	MAGI1	ENSG00000151276.23	103	222	2.2
protein_coding	SS18L2	ENSG00000008324.11	13	28	2.2
protein_coding	INSIG2	ENSG00000125629.14	60	129	2.2
protein_coding	L3MBTL3	ENSG00000198945.7	20	43	2.2
protein_coding	DNAJA1	ENSG00000086061.15	390	836	2.1
protein_coding	ADSS	ENSG00000035687.9	91	195	2.1
protein_coding	NQO2	ENSG00000124588.19	28	60	2.1
protein_coding	LRFN3	ENSG00000126243.8	14	30	2.1
protein_coding	LPCAT1	ENSG00000153395.9	7	15	2.1
protein_coding	ATP6V0A2	ENSG00000185344.13	7	15	2.1
protein_coding	ZNF614	ENSG00000142556.18	7	15	2.1
protein_coding	TBL3	ENSG00000183751.14	7	15	2.1
processed_transcript	AC018521.5	ENSG00000264920.1	7	15	2.1
protein_coding	PLA2G4C	ENSG00000105499.13	7	15	2.1
protein_coding	TMEM87A	ENSG00000103978.15	310	664	2.1
protein_coding	PLIN2	ENSG00000147872.9	395	845	2.1

protein_coding	WISP1	ENSG00000104415.14	72	154	2.1
protein_coding	SECISBP2L	ENSG00000138593.8	218	466	2.1
protein_coding	CTSZ	ENSG00000101160.13	15	32	2.1
protein_coding	BZW2	ENSG00000136261.14	100	213	2.1
protein_coding	CHSY1	ENSG00000131873.6	77	164	2.1
protein_coding	AATF	ENSG00000275700.4	31	66	2.1
protein_coding	GPAT4	ENSG00000158669.11	95	202	2.1
protein_coding	MT-ND6	ENSG00000198695.2	24	51	2.1
protein_coding	SELENOI	ENSG00000138018.17	16	34	2.1
protein_coding	TRMT11	ENSG00000066651.19	8	17	2.1
protein_coding	METTL4	ENSG00000101574.14	8	17	2.1
protein_coding	ZNF780B	ENSG00000128000.15	8	17	2.1
processed_transcript	WDFY3-AS2	ENSG00000180769.8	8	17	2.1
protein_coding	RNF113A	ENSG00000125352.5	25	53	2.1
protein_coding	CEBPB	ENSG00000172216.5	439	929	2.1
protein_coding	GAB2	ENSG00000033327.12	27	57	2.1
protein_coding	NAP1L5	ENSG00000177432.7	9	19	2.1
protein_coding	ZNF582	ENSG00000018869.16	9	19	2.1
protein_coding	UBALD2	ENSG00000185262.8	66	139	2.1
protein_coding	VLDLR	ENSG00000147852.15	19	40	2.1
protein_coding	PNPLA8	ENSG00000135241.16	105	221	2.1
protein_coding	ERBIN	ENSG00000112851.14	144	303	2.1
protein_coding	OSGIN2	ENSG00000164823.10	48	101	2.1
protein_coding	RBM41	ENSG00000089682.16	29	61	2.1
protein_coding	IGFBP4	ENSG00000141753.6	3814	8016	2.1
protein_coding	GPRC5B	ENSG00000167191.11	40	84	2.1
protein_coding	GNL3	ENSG00000163938.16	20	42	2.1
protein_coding	PHYKPL	ENSG00000175309.14	10	21	2.1
protein_coding	PLAUR	ENSG00000011422.11	133	279	2.1
protein_coding	ARL4C	ENSG00000188042.7	246	516	2.1
protein_coding	TTF1	ENSG00000125482.12	32	67	2.1
protein_coding	IER3	ENSG00000137331.11	338	707	2.1
protein_coding	BCL2L11	ENSG00000153094.22	77	161	2.1
protein_coding	DGAT1	ENSG00000185000.11	11	23	2.1
protein_coding	PLAG1	ENSG00000181690.7	11	23	2.1
protein_coding	GON7	ENSG00000170270.4	11	23	2.1
protein_coding	EBF3	ENSG00000108001.13	11	23	2.1
protein_coding	PLEKHN1	ENSG00000187583.10	11	23	2.1
protein_coding	RASA3	ENSG00000185989.10	144	301	2.1
protein_coding	PLAGL1	ENSG00000118495.19	36	75	2.1
protein_coding	PDCD11	ENSG00000148843.14	12	25	2.1
protein_coding	PCDHGA2	ENSG00000081853.14	12	25	2.1
protein_coding	ZNF644	ENSG00000122482.20	49	102	2.1
protein_coding	KLF13	ENSG00000169926.10	114	237	2.1

protein_coding	MEF2D	ENSG00000116604.17	130	270	2.1
protein_coding	SPDL1	ENSG00000040275.16	13	27	2.1
protein_coding	NCOA5	ENSG00000124160.11	13	27	2.1
protein_coding	FANCF	ENSG00000183161.5	13	27	2.1
protein_coding	ELP2	ENSG00000134759.13	172	357	2.1
protein_coding	DIS3	ENSG00000083520.14	53	110	2.1
protein_coding	SIK2	ENSG00000170145.4	226	469	2.1
protein_coding	AGAP1	ENSG00000157985.18	54	112	2.1
protein_coding	CYB5B	ENSG00000103018.16	68	141	2.1
protein_coding	LIN54	ENSG00000189308.10	28	58	2.1
protein_coding	TMEM104	ENSG00000109066.13	14	29	2.1
protein_coding	SYNJ1	ENSG00000159082.17	14	29	2.1
protein_coding	FBXW8	ENSG00000174989.12	14	29	2.1
protein_coding	GID4	ENSG00000141034.9	14	29	2.1
protein_coding	PDCD7	ENSG00000090470.14	29	60	2.1
protein_coding	RIMKLB	ENSG00000166532.15	15	31	2.1
protein_coding	SQOR	ENSG00000137767.13	101	208	2.1
protein_coding	KLHL26	ENSG00000167487.11	17	35	2.1
protein_coding	ATP1B3	ENSG00000069849.10	496	1021	2.1
protein_coding	IRF2	ENSG00000168310.10	19	39	2.1
protein_coding	ELL2	ENSG00000118985.15	229	470	2.1
protein_coding	MARK4	ENSG00000007047.14	77	158	2.1
protein_coding	HMOX1	ENSG00000100292.16	77	158	2.1
protein_coding	MLLT3	ENSG00000171843.15	39	80	2.1
protein_coding	PAPPA	ENSG00000182752.9	378	775	2.1
protein_coding	NRBF2	ENSG00000148572.15	40	82	2.1
protein_coding	C16orf87	ENSG00000155330.9	20	41	2.1
protein_coding	FAM122B	ENSG00000156504.16	20	41	2.1
protein_coding	FGF7	ENSG00000140285.9	390	799	2
protein_coding	GYPC	ENSG00000136732.15	194	397	2
protein_coding	DDX58	ENSG00000107201.9	22	45	2
protein_coding	HSF2	ENSG00000025156.12	22	45	2
protein_coding	HS3ST3A1	ENSG00000153976.2	24	49	2
protein_coding	UBXN2A	ENSG00000173960.13	73	149	2
protein_coding	DEPP1	ENSG00000165507.8	53	108	2
protein_coding	EPC2	ENSG00000135999.11	29	59	2
protein_coding	CLMP	ENSG00000166250.11	618	1256	2
protein_coding	CLASP1	ENSG00000074054.18	33	67	2
protein_coding	PTGS1	ENSG00000095303.15	173	351	2
protein_coding	CHD1	ENSG00000153922.10	38	77	2
protein_coding	ITGA1	ENSG00000213949.8	43	87	2
protein_coding	ITPRIPL2	ENSG00000205730.6	186	376	2
protein_coding	ANKMY2	ENSG00000106524.8	49	99	2
protein_coding	DYNC2H1	ENSG00000187240.14	51	103	2

protein_coding	HSPH1	ENSG00000120694.19	208	420	2
protein_coding	Sep-06	ENSG00000125354.22	55	111	2
protein_coding	TFPI	ENSG00000003436.15	429	865	2
protein_coding	ATP13A3	ENSG00000133657.15	126	254	2
protein_coding	ARMC9	ENSG00000135931.17	101	203	2
protein_coding	ABHD2	ENSG00000140526.17	132	264	2
protein_coding	NARF	ENSG00000141562.17	40	80	2
protein_coding	C11orf54	ENSG00000182919.14	33	66	2
protein_coding	LEMD3	ENSG00000174106.2	32	64	2
protein_coding	PNKP	ENSG00000039650.11	32	64	2
protein_coding	TNFSF13B	ENSG00000102524.11	30	60	2
protein_coding	WDR12	ENSG00000138442.9	29	58	2
protein_coding	SYDE1	ENSG00000105137.12	26	52	2
protein_coding	SLC25A25	ENSG00000148339.12	26	52	2
protein_coding	ABHD17B	ENSG00000107362.13	25	50	2
protein_coding	ABCF3	ENSG00000161204.11	23	46	2
protein_coding	VPS37B	ENSG00000139722.6	19	38	2
protein_coding	CD14	ENSG00000170458.13	19	38	2
protein_coding	PKD1	ENSG00000008710.19	19	38	2
protein_coding	CLPB	ENSG00000162129.13	17	34	2
protein_coding	NUFIP1	ENSG00000083635.7	17	34	2
protein_coding	NFATC1	ENSG00000131196.17	16	32	2
protein_coding	C7orf26	ENSG00000146576.12	16	32	2
protein_coding	PPT2	ENSG00000221988.12	16	32	2
protein_coding	CCDC137	ENSG00000185298.12	15	30	2
antisense	AC097534.2	ENSG00000272870.1	15	30	2
protein_coding	MLLT10	ENSG00000078403.16	15	30	2
protein_coding	C1QTNF6	ENSG00000133466.13	15	30	2
protein_coding	CHRNA9	ENSG00000174343.5	15	30	2
protein_coding	RMND5B	ENSG00000145916.18	15	30	2
protein_coding	ZBTB17	ENSG00000116809.11	13	26	2
protein_coding	RMI2	ENSG00000175643.9	13	26	2
protein_coding	N6AMT1	ENSG00000156239.11	13	26	2
protein_coding	CENPS	ENSG00000175279.21	12	24	2
protein_coding	LRP5	ENSG00000162337.11	12	24	2
protein_coding	MSX2	ENSG00000120149.8	12	24	2
protein_coding	TMEM203	ENSG00000187713.6	11	22	2
protein_coding	HDAC9	ENSG00000048052.21	11	22	2
protein_coding	POT1	ENSG00000128513.14	11	22	2
protein_coding	RAD50	ENSG00000113522.13	11	22	2
protein_coding	MOB3C	ENSG00000142961.14	11	22	2
protein_coding	HDAC6	ENSG00000094631.19	10	20	2

transcribed_unprocessed_pseudogene	EP400P1	ENSG00000185684.14	10	20	2
protein_coding	RAPGEF6	ENSG00000158987.20	10	20	2
protein_coding	EFCAB11	ENSG00000140025.15	10	20	2
protein_coding	SPRY2	ENSG00000136158.11	9	18	2
protein_coding	POLR1E	ENSG00000137054.15	9	18	2
protein_coding	LRRC27	ENSG00000148814.17	9	18	2
protein_coding	LNP1	ENSG00000206535.7	9	18	2
protein_coding	ACVR2B	ENSG00000114739.13	9	18	2
Mt_tRNA	MT-TT	ENSG00000210195.2	9	18	2
protein_coding	ZNF510	ENSG00000081386.12	9	18	2
protein_coding	SEMA6D	ENSG00000137872.16	8	16	2
protein_coding	TMEM217	ENSG00000172738.11	8	16	2
protein_coding	CFAP69	ENSG00000105792.19	8	16	2
protein_coding	C10orf88	ENSG00000119965.12	8	16	2
protein_coding	MRTFA	ENSG00000196588.15	8	16	2
protein_coding	PTGER3	ENSG00000050628.20	8	16	2
protein_coding	SLC25A13	ENSG00000004864.13	7	14	2
protein_coding	CRLF3	ENSG00000176390.11	7	14	2
protein_coding	PIM2	ENSG00000102096.9	7	14	2
protein_coding	ZBTB3	ENSG00000185670.7	7	14	2
protein_coding	PITPNM1	ENSG00000110697.12	7	14	2
protein_coding	GAD1	ENSG00000128683.13	7	14	2
protein_coding	MELK	ENSG00000165304.7	7	14	2
protein_coding	G0S2	ENSG00000123689.5	7	14	2
protein_coding	ZNF468	ENSG00000204604.10	6	12	2
protein_coding	DIS3L2	ENSG00000144535.19	6	12	2
protein_coding	PLK3	ENSG00000173846.12	6	12	2
protein_coding	WDR74	ENSG00000133316.15	6	12	2
protein_coding	TMEM121	ENSG00000184986.10	6	12	2
protein_coding	NRIP3	ENSG00000175352.10	6	12	2
transcribed_unprocessed_pseudogene	AC006453.2	ENSG00000283196.2	6	12	2
lincRNA	AC104825.1	ENSG00000251615.3	6	12	2
protein_coding	ZC3H12A	ENSG00000163874.10	5	10	2
protein_coding	POC5	ENSG00000152359.14	5	10	2
protein_coding	POLG2	ENSG00000256525.6	5	10	2
protein_coding	FAM227A	ENSG00000184949.15	5	10	2
protein_coding	TGFBR3L	ENSG00000260001.6	5	10	2
lincRNA	LINC00476	ENSG00000175611.11	5	10	2
protein_coding	ANKRD13B	ENSG00000198720.12	5	10	2
protein_coding	HIST1H2BJ	ENSG00000124635.8	4	8	2
protein_coding	TRIB1	ENSG00000173334.3	4	8	2

protein_coding	SPTLC3	ENSG00000172296.12	4	8	2
protein_coding	SFRP4	ENSG00000106483.11	4	8	2
protein_coding	ZNF154	ENSG00000179909.15	4	8	2
protein_coding	RAB6B	ENSG00000154917.10	4	8	2
antisense	AC021097.1	ENSG00000272661.1	4	8	2
protein_coding	PSRC1	ENSG00000134222.16	4	8	2
protein_coding	THNSL1	ENSG00000185875.12	4	8	2
protein_coding	C8orf48	ENSG00000164743.4	4	8	2
protein_coding	ZNF526	ENSG00000167625.10	4	8	2
protein_coding	TET1	ENSG00000138336.8	4	8	2
protein_coding	PHF7	ENSG00000010318.20	4	8	2
protein_coding	PCDHB13	ENSG00000187372.11	4	8	2
lincRNA	LINC00562	ENSG00000260388.2	4	8	2
protein_coding	ZNF736	ENSG00000234444.9	4	8	2
protein_coding	CISH	ENSG00000114737.15	4	8	2
protein_coding	TAF5	ENSG00000148835.10	3	6	2
protein_coding	PCDHGB7	ENSG00000254122.2	3	6	2
protein_coding	BACH2	ENSG00000112182.14	3	6	2
protein_coding	SPINT2	ENSG00000167642.12	3	6	2
protein_coding	ADAMTS9	ENSG00000163638.13	3	6	2
protein_coding	MILR1	ENSG00000271605.5	3	6	2
antisense	H1FX-AS1	ENSG00000206417.8	3	6	2
lincRNA	SUCLG2-AS1	ENSG00000241316.7	3	6	2
protein_coding	MARS2	ENSG00000247626.4	3	6	2
lincRNA	AP004609.3	ENSG00000278376.1	3	6	2
processed_pseudogene	BX679664.3	ENSG00000244716.3	3	6	2
protein_coding	ITGB2	ENSG00000160255.17	3	6	2
antisense	AC008966.1	ENSG00000247796.2	3	6	2
processed_pseudogene	NOP56P1	ENSG00000235559.1	3	6	2
protein_coding	ZNF563	ENSG00000188868.13	3	6	2
protein_coding	AGRN	ENSG00000188157.14	3	6	2
protein_coding	RAD52	ENSG00000002016.17	3	6	2
protein_coding	AC233723.1	ENSG00000262165.2	3	6	2
protein_coding	C17orf107	ENSG00000205710.3	3	6	2
protein_coding	SERPINB7	ENSG00000166396.12	3	6	2
antisense	AC009403.1	ENSG00000216895.9	3	6	2
unprocessed_pseudogene	MTND1P23	ENSG00000225972.1	3	6	2
processed_pseudogene	EEF1A1P13	ENSG00000250182.3	3	6	2
protein_coding	CENPV	ENSG00000166582.9	3	6	2
processed_pseudogene	RPL23AP65	ENSG00000243964.1	3	6	2
protein_coding	SRGAP3	ENSG00000196220.15	3	6	2
protein_coding	HCAR1	ENSG00000196917.5	3	6	2
protein_coding	HBEGF	ENSG00000113070.7	2	4	2
antisense	BOLA3-AS1	ENSG00000225439.2	2	4	2

lincRNA	AL117190.1	ENSG00000258399.7	2	4	2
processed_transcript	AL157392.3	ENSG00000239665.8	2	4	2
protein_coding	TBXAS1	ENSG00000059377.16	2	4	2
antisense	A1BG-AS1	ENSG00000268895.5	2	4	2
protein_coding	FPR1	ENSG00000171051.8	2	4	2
bidirectional_promoter_lncRNA	AL391684.1	ENSG00000224934.4	2	4	2
protein_coding	RASSF7	ENSG00000099849.14	2	4	2
protein_coding	TLR4	ENSG00000136869.14	2	4	2
antisense	ARRDC1-	ENSG00000203993.4	2	4	2
processed_transcript	AL590094.1	ENSG00000233593.8	2	4	2
protein_coding	NDC80	ENSG00000080986.12	2	4	2
lincRNA	AC025165.5	ENSG00000270039.1	2	4	2
transcribed_unprocessed_pseudogene	WHAMMP2	ENSG00000248334.6	2	4	2
lincRNA	SNHG4	ENSG00000281398.4	2	4	2
antisense	AC113383.1	ENSG00000250320.5	2	4	2
protein_coding	AC119674.2	ENSG00000284686.1	2	4	2
protein_coding	GHR	ENSG00000112964.13	2	4	2
protein_coding	PLCXD1	ENSG00000182378.13	2	4	2
protein_coding	ARHGEF37	ENSG00000183111.11	2	4	2
protein_coding	TMEFF2	ENSG00000144339.11	2	4	2
protein_coding	CYTL1	ENSG00000170891.10	2	4	2
lincRNA	AC093227.1	ENSG00000267152.1	2	4	2
protein_coding	ZBTB48	ENSG00000204859.12	2	4	2
antisense	AC124066.1	ENSG00000265263.1	2	4	2
antisense	AC073508.3	ENSG00000278834.1	2	4	2
TEC	AC046185.3	ENSG00000279369.1	2	4	2
antisense	MELTF-AS1	ENSG00000228109.1	2	4	2
lincRNA	GASAL1	ENSG00000253669.3	2	4	2
lincRNA	AC134312.5	ENSG00000261327.4	2	4	2
protein_coding	DNAH10	ENSG00000197653.15	2	4	2
protein_coding	DTX4	ENSG00000110042.7	2	4	2
protein_coding	PINLYP	ENSG00000234465.10	2	4	2
protein_coding	SHROOM4	ENSG00000158352.15	2	4	2
lincRNA	AC027288.3	ENSG00000257894.2	2	4	2
protein_coding	HSD17B1	ENSG00000108786.10	2	4	2
protein_coding	ZIK1	ENSG00000171649.11	2	4	2
protein_coding	DHRS4	ENSG00000157326.18	2	4	2
protein_coding	ATP6V1FNB	ENSG00000272899.3	2	4	2
protein_coding	CACNB1	ENSG00000067191.15	2	4	2
protein_coding	SHLD1	ENSG00000171984.14	2	4	2
protein_coding	USP43	ENSG00000154914.16	2	4	2

protein_coding	ALS2CL	ENSG00000178038.16	2	4	2
protein_coding	TTC25	ENSG00000204815.9	2	4	2
sense_intronic	AC008870.2	ENSG00000260751.2	2	4	2
TEC	AC104581.4	ENSG00000280046.1	2	4	2
lincRNA	AC109479.1	ENSG00000253163.1	2	4	2
lincRNA	LINC00654	ENSG00000205181.5	2	4	2
snRNA	RNU7-57P	ENSG00000238365.3	2	4	2
protein_coding	PTP4A3	ENSG00000184489.11	2	4	2
protein_coding	FHDC1	ENSG00000137460.8	2	4	2
antisense	Z95115.1	ENSG00000261188.1	2	4	2
antisense	AC245452.1	ENSG00000224086.5	2	4	2
lincRNA	AC022007.1	ENSG00000206567.9	2	4	2
antisense	AC009126.1	ENSG00000247121.6	2	4	2
antisense	AC009228.1	ENSG00000242628.5	2	4	2
protein_coding	SERHL2	ENSG00000183569.17	2	4	2
processed_pseudogene	HSP90AA2P	ENSG00000224411.3	2	4	2
protein_coding	NRGN	ENSG00000154146.12	1	2	2
antisense	AC138696.2	ENSG00000272172.1	1	2	2
antisense	AC037459.3	ENSG00000253200.1	1	2	2
protein_coding	ZNF202	ENSG00000166261.10	1	2	2
protein_coding	PPM1N	ENSG00000213889.10	1	2	2
protein_coding	FNDC11	ENSG00000125531.6	1	2	2
transcribed_processed_pseudogene	SSR4P1	ENSG00000235374.2	1	2	2
protein_coding	C16orf46	ENSG00000166455.13	1	2	2
protein_coding	SPRED3	ENSG00000188766.12	1	2	2
protein_coding	CDK2AP1	ENSG00000111328.6	1	2	2
lincRNA	AL139424.2	ENSG00000284642.1	1	2	2
lincRNA	AL391832.2	ENSG00000238005.2	1	2	2
protein_coding	ZSWIM4	ENSG00000132003.9	1	2	2
snoRNA	RF00012	ENSG00000238297.2	1	2	2
protein_coding	SPATA1	ENSG00000122432.17	1	2	2
antisense	NDUFB2-	ENSG00000240889.1	1	2	2
protein_coding	PTGIR	ENSG00000160013.8	1	2	2
antisense	U47924.2	ENSG00000272173.1	1	2	2
protein_coding	MKI67	ENSG00000148773.13	1	2	2
protein_coding	TTK	ENSG00000112742.9	1	2	2
protein_coding	C19orf44	ENSG00000105072.8	1	2	2
transcribed_processed_pseudogene	DUXAP10	ENSG00000244306.11	1	2	2
protein_coding	AKNAD1	ENSG00000162641.18	1	2	2
protein_coding	ARHGAP27	ENSG00000159314.11	1	2	2
sense_intronic	AP005482.3	ENSG00000267249.1	1	2	2

protein_coding	CNFN	ENSG00000105427.9	1	2	2
protein_coding	MPV17L	ENSG00000156968.8	1	2	2
transcribed_processed_pseudogene	AC018755.3	ENSG00000269388.1	1	2	2
protein_coding	OVGP1	ENSG00000085465.12	1	2	2
processed_pseudogene	GAPDHP65	ENSG00000235587.2	1	2	2
lincRNA	SCGB1B2P	ENSG00000268751.1	1	2	2
protein_coding	SLC25A45	ENSG00000162241.12	1	2	2
protein_coding	RHPN2	ENSG00000131941.7	1	2	2
protein_coding	RPH3AL	ENSG00000181031.15	1	2	2
protein_coding	PROCA1	ENSG00000167525.13	1	2	2
protein_coding	MXD3	ENSG00000213347.10	1	2	2
lincRNA	AC092171.5	ENSG00000273084.1	1	2	2
protein_coding	FAM71F2	ENSG00000205085.11	1	2	2
lincRNA	AC104051.2	ENSG00000254139.1	1	2	2
protein_coding	LRRC3	ENSG00000160233.7	1	2	2
lincRNA	AC103706.1	ENSG00000261220.2	1	2	2
lincRNA	AC103736.1	ENSG00000254427.1	1	2	2
protein_coding	C1orf87	ENSG00000162598.13	1	2	2
protein_coding	KCNMB1	ENSG00000145936.8	1	2	2
protein_coding	SGK2	ENSG00000101049.15	1	2	2
snRNA	RNU6-1216P	ENSG00000199460.2	1	2	2
lincRNA	AC026471.4	ENSG00000261474.1	1	2	2
sense_overlapping	AC095057.3	ENSG00000260296.1	1	2	2
TEC	AC008764.10	ENSG00000279977.1	1	2	2
protein_coding	CEACAM19	ENSG00000186567.12	1	2	2
protein_coding	SLPI	ENSG00000124107.5	1	2	2
protein_coding	GDPD5	ENSG00000158555.14	1	2	2
sense_intronic	AL133243.3	ENSG00000276517.1	1	2	2
protein_coding	GRB14	ENSG00000115290.9	1	2	2
antisense	AL354836.1	ENSG00000226332.2	1	2	2
antisense	AC083798.2	ENSG00000272758.5	1	2	2
Mt_tRNA	MT-TD	ENSG00000210154.1	1	2	2
antisense	AL359091.4	ENSG00000272696.1	1	2	2
protein_coding	UQCRHL	ENSG00000233954.6	1	2	2
processed_pseudogene	RPS12P16	ENSG00000215184.2	1	2	2
lincRNA	LINC02057	ENSG00000249279.5	1	2	2
processed_pseudogene	PTMAP1	ENSG00000228415.3	1	2	2
processed_pseudogene	RPS2P32	ENSG00000232818.2	1	2	2
protein_coding	CSNK2A3	ENSG00000254598.2	1	2	2
antisense	GACAT2	ENSG00000265962.1	1	2	2
antisense	AC130324.1	ENSG00000263531.1	1	2	2
protein_coding	MEIG1	ENSG00000197889.9	1	2	2

antisense	AL353593.1	ENSG00000269890.1	1	2	2
lincRNA	LINC00922	ENSG00000261742.5	1	2	2
protein_coding	FAM198A	ENSG00000144649.8	1	2	2
protein_coding	C5orf49	ENSG00000215217.6	1	2	2
protein_coding	CD163L1	ENSG00000177675.8	1	2	2
protein_coding	C12orf60	ENSG00000182993.4	1	2	2
processed_pseudogene	FBXW4P1	ENSG00000230701.2	1	2	2
protein_coding	MRM1	ENSG00000278619.4	1	2	2
protein_coding	MATN3	ENSG00000132031.12	1	2	2
lincRNA	AC018553.1	ENSG00000277559.1	1	2	2
protein_coding	MRAP2	ENSG00000135324.5	1	2	2
lincRNA	LINC00526	ENSG00000264575.1	1	2	2
protein_coding	FRK	ENSG00000111816.7	1	2	2
protein_coding	FBXO15	ENSG00000141665.12	1	2	2
protein_coding	TMPPE	ENSG00000188167.8	1	2	2
TEC	AC000123.2	ENSG00000279265.1	1	2	2
protein_coding	GNG5	ENSG00000174021.10	1	2	2
protein_coding	MBLAC1	ENSG00000214309.4	1	2	2
antisense	KCNMB2-	ENSG00000237978.5	1	2	2
lincRNA	AC124045.1	ENSG00000272077.1	1	2	2
transcribed_unprocessed_pseudogene	KLRA1P	ENSG00000256667.6	1	2	2
protein_coding	PFN4	ENSG00000176732.6	1	2	2
snRNA	RNU1-16P	ENSG00000202347.1	1	2	2
antisense	AL590560.1	ENSG00000272668.2	1	2	2
misc_RNA	RN7SL471P	ENSG00000263426.2	1	2	2
processed_pseudogene	RPS11P5	ENSG00000232888.4	1	2	2
protein_coding	MT1A	ENSG00000205362.11	1	2	2
protein_coding	PDE6G	ENSG00000185527.11	1	2	2
transcribed_unprocessed_pseudogene	AC092070.2	ENSG00000269001.2	1	2	2
protein_coding	MAMDC4	ENSG00000177943.13	1	2	2
lincRNA	AC093788.1	ENSG00000273449.1	1	2	2
protein_coding	AC007906.2	ENSG00000277639.2	1	2	2
protein_coding	GSTO2	ENSG00000065621.14	1	2	2
antisense	AC006449.3	ENSG00000275665.1	1	2	2
transcribed_processed_pseudogene	ABCA11P	ENSG00000251595.7	1	2	2
protein_coding	CDCA8	ENSG00000134690.10	1	2	2
protein_coding	ZNF19	ENSG00000157429.15	1	2	2
protein_coding	SLC45A3	ENSG00000158715.5	1	2	2
transcribed_unprocessed_pseudogene	AC124944.3	ENSG00000260261.2	1	2	2

protein_coding	RAB9B	ENSG00000123570.3	1	2	2
protein_coding	OIP5	ENSG00000104147.8	1	2	2
protein_coding	ZNF41	ENSG00000147124.12	1	2	2
protein_coding	AGBL2	ENSG00000165923.15	1	2	2
protein_coding	MAMSTR	ENSG00000176909.11	1	2	2
lincRNA	AC069307.1	ENSG00000269949.1	1	2	2
protein_coding	LGALS9	ENSG00000168961.16	1	2	2
protein_coding	AIM2	ENSG00000163568.14	1	2	2
lincRNA	RFX3-AS1	ENSG00000232104.3	1	2	2
processed_pseudogene	RPSAP12	ENSG00000240087.3	1	2	2
antisense	AC079834.2	ENSG00000272944.1	1	2	2
antisense	CHKB-DT	ENSG00000205559.4	1	2	2
transcribed_unprocessed_pseudogene	HLA-J	ENSG00000204622.11	1	2	2
protein_coding	CASC10	ENSG00000204682.5	1	2	2
lincRNA	AL080317.2	ENSG00000271789.1	1	2	2
lincRNA	AC007336.1	ENSG00000261997.1	1	2	2
sense_intronic	AP002840.2	ENSG00000270179.1	1	2	2
antisense	RUSC1-AS1	ENSG00000225855.6	1	2	2
lincRNA	AL162426.1	ENSG00000279571.3	1	2	2
processed_pseudogene	EIF3FP3	ENSG00000233426.3	1	2	2
antisense	AL359711.2	ENSG00000260273.1	1	2	2
antisense	CEBPA-DT	ENSG00000267296.2	1	2	2
protein_coding	PILRA	ENSG00000085514.15	1	2	2
antisense	ID2-AS1	ENSG00000235092.5	1	2	2
lincRNA	AC004477.3	ENSG00000278765.1	1	2	2
processed_pseudogene	AL031133.1	ENSG00000219553.2	1	2	2
protein_coding	ATP5MGL	ENSG00000249222.1	1	2	2
lincRNA	AL031432.5	ENSG00000284657.1	1	2	2
protein_coding	SMIM10L2B	ENSG00000196972.8	1	2	2
lincRNA	AC107959.4	ENSG00000284948.1	1	2	2
protein_coding	MAGED4	ENSG00000154545.16	1	2	2
antisense	AC051619.5	ENSG00000259520.5	1	2	2
lincRNA	AC016877.3	ENSG00000271930.1	1	2	2
lincRNA	GAS1RR	ENSG00000226237.1	1	2	2
lincRNA	AL158071.3	ENSG00000237422.1	1	2	2
lincRNA	AC087392.5	ENSG00000277491.1	1	2	2
lincRNA	AC091806.1	ENSG00000236393.1	1	2	2
processed_pseudogene	GCSHP5	ENSG00000224837.1	1	2	2
processed_pseudogene	HMGN1P36	ENSG00000235734.4	1	2	2
processed_pseudogene	AC110994.2	ENSG00000282978.1	1	2	2
protein_coding	LY75	ENSG00000054219.10	1	2	2
protein_coding	SCN5A	ENSG00000183873.15	1	2	2

protein_coding	SLC28A3	ENSG00000197506.7	1	2	2
protein_coding	LCTL	ENSG00000188501.11	1	2	2
protein_coding	ASPA	ENSG00000108381.10	1	2	2
lincRNA	AC110792.3	ENSG00000272650.1	1	2	2
processed_pseudogene	FTLP3	ENSG00000226608.3	1	2	2
protein_coding	ASAH2	ENSG00000188611.14	1	2	2
protein_coding	SMCO2	ENSG00000165935.9	1	2	2
lincRNA	AL359504.2	ENSG00000271576.1	1	2	2
processed_pseudogene	AC114495.2	ENSG00000229447.2	1	2	2
antisense	AP000487.1	ENSG00000246889.2	1	2	2
protein_coding	CTU1	ENSG00000142544.6	1	2	2
protein_coding	ORC1	ENSG00000085840.12	1	2	2
protein_coding	KLHL13	ENSG00000003096.14	98	195	2
protein_coding	JUNB	ENSG00000171223.5	73	145	2
protein_coding	ARNT2	ENSG00000172379.20	68	135	2
protein_coding	RB1CC1	ENSG00000023287.12	395	783	2
protein_coding	UPF3B	ENSG00000125351.11	95	188	2
protein_coding	PDS5B	ENSG00000083642.18	92	182	2
protein_coding	BACH1	ENSG00000156273.15	41	81	2
protein_coding	BICD2	ENSG00000185963.13	72	142	2
protein_coding	ETV6	ENSG00000139083.10	36	71	2
protein_coding	FADS1	ENSG00000149485.18	227	447	2
protein_coding	PHF21A	ENSG00000135365.15	94	185	2
protein_coding	TARDBP	ENSG00000120948.17	29	57	2
protein_coding	FAM199X	ENSG00000123575.8	28	55	2
protein_coding	RTN2	ENSG00000125744.11	28	55	2
protein_coding	PHLDA1	ENSG00000139289.13	842	1653	2
antisense	GABPB1-AS1	ENSG00000244879.7	49	96	2
protein_coding	SLC2A10	ENSG00000197496.5	212	415	2
protein_coding	IWS1	ENSG00000163166.14	70	137	2
protein_coding	MN1	ENSG00000169184.5	44	86	2
protein_coding	GNB4	ENSG00000114450.9	83	162	2
protein_coding	EML4	ENSG00000143924.18	140	273	2
protein_coding	SLC23A2	ENSG00000089057.14	60	117	2
protein_coding	CHORDC1	ENSG00000110172.11	20	39	2
lincRNA	MEG3	ENSG00000214548.16	277	540	1.9
protein_coding	HMGN5	ENSG00000198157.10	19	37	1.9
protein_coding	CFLAR	ENSG00000003402.19	205	399	1.9
protein_coding	GNAI1	ENSG00000127955.16	74	144	1.9
protein_coding	UTRN	ENSG00000152818.18	147	286	1.9
protein_coding	SUDS3	ENSG00000111707.11	54	105	1.9
protein_coding	ENC1	ENSG00000171617.13	18	35	1.9
protein_coding	SLC2A1	ENSG00000117394.21	71	138	1.9
protein_coding	GPR155	ENSG00000163328.13	34	66	1.9

protein_coding	SOAT1	ENSG00000057252.12	84	163	1.9
protein_coding	ENDOD1	ENSG00000149218.4	50	97	1.9
protein_coding	TSR1	ENSG00000167721.10	33	64	1.9
protein_coding	CENPE	ENSG00000138778.11	16	31	1.9
protein_coding	CEMIP2	ENSG00000135048.13	16	31	1.9
protein_coding	L3MBTL2	ENSG00000100395.14	16	31	1.9
protein_coding	WASL	ENSG00000106299.7	107	207	1.9
protein_coding	PDE4B	ENSG00000184588.17	30	58	1.9
protein_coding	FURIN	ENSG00000140564.11	112	216	1.9
protein_coding	FANK1	ENSG00000203780.10	14	27	1.9
protein_coding	P4HA3	ENSG00000149380.11	69	133	1.9
protein_coding	ZC3H18	ENSG00000158545.15	27	52	1.9
protein_coding	XYLT2	ENSG00000015532.9	27	52	1.9
protein_coding	AK4	ENSG00000162433.14	40	77	1.9
protein_coding	SLC35D1	ENSG00000116704.7	52	100	1.9
protein_coding	IL6	ENSG00000136244.11	13	25	1.9
protein_coding	SPOPL	ENSG00000144228.8	13	25	1.9
protein_coding	ATG7	ENSG00000197548.12	13	25	1.9
protein_coding	HSP90B1	ENSG00000166598.14	1332	2560	1.9
protein_coding	WWTR1	ENSG00000018408.14	368	707	1.9
protein_coding	RBM12	ENSG00000244462.7	38	73	1.9
protein_coding	HLCS	ENSG00000159267.14	38	73	1.9
protein_coding	PUDP	ENSG00000130021.13	25	48	1.9
protein_coding	CTSL	ENSG00000135047.14	643	1234	1.9
protein_coding	SEL1L3	ENSG00000091490.10	37	71	1.9
protein_coding	CREBZF	ENSG00000137504.13	72	138	1.9
protein_coding	POLR3E	ENSG00000058600.15	12	23	1.9
protein_coding	LIN52	ENSG00000205659.10	12	23	1.9
protein_coding	LTN1	ENSG00000198862.13	35	67	1.9
protein_coding	PRICKLE2	ENSG00000163637.12	23	44	1.9
protein_coding	RAB23	ENSG00000112210.11	148	283	1.9
protein_coding	MOB1B	ENSG00000173542.8	91	174	1.9
protein_coding	TNFAIP6	ENSG00000123610.4	125	239	1.9
protein_coding	CLP¬†1	ENSG00000172409.5	11	21	1.9
protein_coding	SLC31A1	ENSG00000136868.10	120	229	1.9
protein_coding	SUSD6	ENSG00000100647.7	76	145	1.9
protein_coding	MFSD12	ENSG00000161091.12	268	511	1.9
protein_coding	TOB1	ENSG00000141232.4	168	320	1.9
protein_coding	CNTLN	ENSG00000044459.14	42	80	1.9
protein_coding	CASP9	ENSG00000132906.17	21	40	1.9
protein_coding	USP38	ENSG00000170185.9	21	40	1.9
protein_coding	BLOC1S3	ENSG00000189114.6	21	40	1.9
protein_coding	HYAL2	ENSG00000068001.13	60	114	1.9
protein_coding	ACSL1	ENSG00000151726.13	30	57	1.9

lincRNA	CASC15	ENSG00000272168.6	30	57	1.9
protein_coding	ZNF513	ENSG00000163795.13	10	19	1.9
protein_coding	SRRD	ENSG00000100104.13	10	19	1.9
protein_coding	LBH	ENSG00000213626.12	10	19	1.9
protein_coding	MRM3	ENSG00000171861.10	10	19	1.9
protein_coding	ARAP3	ENSG00000120318.15	10	19	1.9
protein_coding	ZNF420	ENSG00000197050.10	10	19	1.9
protein_coding	ZFYVE16	ENSG00000039319.16	39	74	1.9
protein_coding	BRCC3	ENSG00000185515.14	39	74	1.9
protein_coding	INPP5A	ENSG00000068383.18	39	74	1.9
protein_coding	CLDN12	ENSG00000157224.15	29	55	1.9
protein_coding	SLC25A37	ENSG00000147454.13	143	271	1.9
protein_coding	MARCKSL1	ENSG00000175130.6	57	108	1.9
protein_coding	ZDHHC14	ENSG00000175048.16	85	161	1.9
protein_coding	UBE2W	ENSG00000104343.19	37	70	1.9
protein_coding	GADD45B	ENSG00000099860.8	45	85	1.9
protein_coding	DMWD	ENSG00000185800.11	18	34	1.9
protein_coding	ZNF140	ENSG00000196387.9	18	34	1.9
protein_coding	TVP23B	ENSG00000171928.13	9	17	1.9
protein_coding	ZNF346	ENSG00000113761.11	9	17	1.9
protein_coding	BRF1	ENSG00000185024.16	9	17	1.9
protein_coding	C5orf22	ENSG00000082213.17	26	49	1.9
protein_coding	NPTX1	ENSG00000171246.5	43	81	1.9
protein_coding	NDRG4	ENSG00000103034.14	60	113	1.9
protein_coding	PRPF18	ENSG00000165630.13	17	32	1.9
protein_coding	ZNF618	ENSG00000157657.14	76	143	1.9
protein_coding	YTHDC1	ENSG00000083896.12	75	141	1.9
antisense	HCG18	ENSG00000231074.8	25	47	1.9
antisense	ZSCAN16-	ENSG00000269293.2	25	47	1.9
protein_coding	TRABD2A	ENSG00000186854.10	25	47	1.9
protein_coding	ACADM	ENSG00000117054.13	41	77	1.9
protein_coding	SH3PXD2A	ENSG00000107957.16	229	430	1.9
protein_coding	NPAT	ENSG00000149308.16	40	75	1.9
protein_coding	HMGCR	ENSG00000113161.15	32	60	1.9
protein_coding	CNNM2	ENSG00000148842.17	24	45	1.9
protein_coding	GORAB	ENSG00000120370.12	24	45	1.9
protein_coding	SMAD9	ENSG00000120693.13	24	45	1.9
protein_coding	PRR12	ENSG00000126464.13	16	30	1.9
protein_coding	NADK	ENSG00000008130.15	8	15	1.9
protein_coding	ZNF776	ENSG00000152443.12	8	15	1.9
protein_coding	ZCCHC8	ENSG00000033030.13	8	15	1.9
protein_coding	ZNF230	ENSG00000159882.12	8	15	1.9
sense_overlapping	AC025171.1	ENSG00000177738.3	8	15	1.9
protein_coding	CCDC7	ENSG00000216937.13	8	15	1.9

protein_coding	SPIN3	ENSG00000204271.12	8	15	1.9
protein_coding	AHI1	ENSG00000135541.20	77	144	1.9
protein_coding	SAV1	ENSG00000151748.14	115	215	1.9
protein_coding	SLC52A2	ENSG00000185803.9	46	86	1.9
protein_coding	PRTFDC1	ENSG00000099256.18	23	43	1.9
protein_coding	HSPA1A	ENSG00000204389.9	99	185	1.9
lincRNA	AC027031.2	ENSG00000254615.2	15	28	1.9
protein_coding	DCAF1	ENSG00000145041.15	15	28	1.9
protein_coding	SLC39A11	ENSG00000133195.11	37	69	1.9
protein_coding	AKT3	ENSG00000117020.16	81	151	1.9
protein_coding	ZNF562	ENSG00000171466.9	22	41	1.9
protein_coding	ZFAND5	ENSG00000107372.12	446	831	1.9
processed_pseudogene	AC116533.1	ENSG00000244398.1	29	54	1.9
protein_coding	BAG1	ENSG00000107262.21	121	225	1.9
protein_coding	RFLNB	ENSG00000183688.4	249	463	1.9
protein_coding	PPIF	ENSG00000108179.13	84	156	1.9
protein_coding	PRRC2A	ENSG00000204469.12	28	52	1.9
protein_coding	CHST3	ENSG00000122863.5	14	26	1.9
lincRNA	AC015922.3	ENSG00000276855.1	14	26	1.9
protein_coding	SERTAD3	ENSG00000167565.12	14	26	1.9
protein_coding	NAA80	ENSG00000243477.5	7	13	1.9
protein_coding	GALE	ENSG00000117308.14	7	13	1.9
protein_coding	HLA-F	ENSG00000204642.13	7	13	1.9
protein_coding	MX2	ENSG00000183486.12	7	13	1.9
protein_coding	BRCA1	ENSG00000012048.21	7	13	1.9
protein_coding	MAP6	ENSG00000171533.11	7	13	1.9
protein_coding	STAG2	ENSG00000101972.18	285	529	1.9
protein_coding	AP2A2	ENSG00000183020.13	55	102	1.9
protein_coding	CYB5R4	ENSG00000065615.13	34	63	1.9
protein_coding	NFKBIB	ENSG00000104825.16	27	50	1.9
protein_coding	TMEM38B	ENSG00000095209.11	27	50	1.9
protein_coding	CCDC112	ENSG00000164221.12	40	74	1.9
protein_coding	B4GALNT1	ENSG00000135454.13	20	37	1.9
protein_coding	AHCYL2	ENSG00000158467.16	20	37	1.9
protein_coding	RGS12	ENSG00000159788.19	20	37	1.9
protein_coding	RC3H2	ENSG00000056586.15	59	109	1.8
protein_coding	MAP9	ENSG00000164114.18	78	144	1.8
protein_coding	FAM174A	ENSG00000174132.8	39	72	1.8
protein_coding	FBXL20	ENSG00000108306.12	39	72	1.8
protein_coding	AMBRA1	ENSG00000110497.14	13	24	1.8
protein_coding	TRANK1	ENSG00000168016.14	13	24	1.8
protein_coding	DYRK1B	ENSG00000105204.13	13	24	1.8
protein_coding	EHD3	ENSG00000013016.15	77	142	1.8
protein_coding	PRXL2C	ENSG00000158122.11	147	271	1.8

protein_coding	HDAC8	ENSG00000147099.20	19	35	1.8
protein_coding	SH2B3	ENSG00000111252.10	50	92	1.8
protein_coding	STARD4	ENSG00000164211.12	25	46	1.8
protein_coding	STAT3	ENSG00000168610.14	171	314	1.8
protein_coding	ABCF1	ENSG00000204574.12	126	231	1.8
protein_coding	PNPLA2	ENSG00000177666.16	120	220	1.8
protein_coding	CDR2L	ENSG00000109089.7	84	154	1.8
protein_coding	PNO1	ENSG00000115946.7	18	33	1.8
protein_coding	CCDC122	ENSG00000151773.12	18	33	1.8
protein_coding	ZFPM1	ENSG00000179588.8	12	22	1.8
protein_coding	TTC31	ENSG00000115282.19	12	22	1.8
protein_coding	SPSB2	ENSG00000111671.9	12	22	1.8
protein_coding	GPRC5A	ENSG00000013588.8	6	11	1.8
protein_coding	MSANTD2	ENSG00000120458.11	6	11	1.8
protein_coding	AQP1	ENSG00000240583.11	6	11	1.8
lincRNA	AC016831.4	ENSG00000271204.1	6	11	1.8
protein_coding	HLX	ENSG00000136630.12	6	11	1.8
protein_coding	ZNF649	ENSG00000198093.10	6	11	1.8
protein_coding	CCDC125	ENSG00000183323.12	6	11	1.8
protein_coding	GCAT	ENSG00000100116.16	6	11	1.8
protein_coding	AADAT	ENSG00000109576.13	6	11	1.8
protein_coding	LRRC75B	ENSG00000178026.12	6	11	1.8
unprocessed_pseudogene	FRG1GP	ENSG00000283023.1	6	11	1.8
protein_coding	NBL1	ENSG00000158747.14	190	348	1.8
protein_coding	PAM	ENSG00000145730.20	398	728	1.8
protein_coding	CREBL2	ENSG00000111269.2	87	159	1.8
lincRNA	AC092747.4	ENSG00000275764.1	29	53	1.8
protein_coding	PPP1R3D	ENSG00000132825.6	23	42	1.8
protein_coding	KANSL3	ENSG00000114982.17	74	135	1.8
protein_coding	ATXN1	ENSG00000124788.18	34	62	1.8
protein_coding	CLK2	ENSG00000176444.18	17	31	1.8
protein_coding	TSSC4	ENSG00000184281.14	17	31	1.8
protein_coding	ANKRD12	ENSG00000101745.16	440	801	1.8
protein_coding	C12orf10	ENSG00000139637.13	22	40	1.8
protein_coding	DDX56	ENSG00000136271.10	22	40	1.8
protein_coding	TIMELESS	ENSG00000111602.11	11	20	1.8
protein_coding	DIAPH3	ENSG00000139734.18	11	20	1.8
protein_coding	ATRN	ENSG00000088812.17	60	109	1.8
protein_coding	CLUAP1	ENSG00000103351.12	49	89	1.8
protein_coding	SPTY2D1	ENSG00000179119.14	38	69	1.8
protein_coding	TAOK3	ENSG00000135090.13	381	691	1.8
protein_coding	TSR2	ENSG00000158526.7	32	58	1.8
protein_coding	ZNF518A	ENSG00000177853.14	16	29	1.8
protein_coding	LRRC2	ENSG00000163827.12	16	29	1.8

protein_coding	REXO4	ENSG00000148300.11	37	67	1.8
protein_coding	COQ2	ENSG00000173085.14	37	67	1.8
protein_coding	MED13	ENSG00000108510.9	95	172	1.8
protein_coding	CASP1	ENSG00000137752.23	21	38	1.8
protein_coding	CPED1	ENSG00000106034.17	68	123	1.8
protein_coding	GNG11	ENSG00000127920.5	733	1323	1.8
protein_coding	C1orf131	ENSG00000143633.12	20	36	1.8
protein_coding	ENDOV	ENSG00000173818.16	20	36	1.8
protein_coding	CYREN	ENSG00000122783.16	20	36	1.8
protein_coding	TTC32	ENSG00000183891.5	15	27	1.8
protein_coding	PALB2	ENSG00000083093.9	10	18	1.8
protein_coding	SLC46A1	ENSG00000076351.12	10	18	1.8
lincRNA	LINC00665	ENSG00000232677.6	10	18	1.8
protein_coding	SYT7	ENSG00000011347.9	10	18	1.8
protein_coding	ZNF408	ENSG00000175213.2	10	18	1.8
processed_transcript	SNHG11	ENSG00000174365.19	10	18	1.8
protein_coding	ZNF200	ENSG00000010539.11	10	18	1.8
protein_coding	GPD1L	ENSG00000152642.10	10	18	1.8
protein_coding	KLHL15	ENSG00000174010.9	5	9	1.8
protein_coding	CACHD1	ENSG00000158966.14	5	9	1.8
protein_coding	RANBP17	ENSG00000204764.13	5	9	1.8
lincRNA	MEG9	ENSG00000223403.4	5	9	1.8
protein_coding	ZNF354B	ENSG00000178338.10	5	9	1.8
protein_coding	ZNF596	ENSG00000172748.13	5	9	1.8
protein_coding	CDKN2AIPN	ENSG00000237190.3	5	9	1.8
protein_coding	PICK1	ENSG00000100151.15	5	9	1.8
protein_coding	ZNF487	ENSG00000243660.9	5	9	1.8
protein_coding	NUP35	ENSG00000163002.12	5	9	1.8
protein_coding	ZNF417	ENSG00000173480.10	5	9	1.8
protein_coding	PSMC3IP	ENSG00000131470.14	5	9	1.8
processed_pseudogene	TOB2P1	ENSG00000176933.5	5	9	1.8
protein_coding	TM6SF2	ENSG00000213996.12	5	9	1.8
protein_coding	AC073111.5	ENSG00000284691.1	5	9	1.8
protein_coding	DUSP19	ENSG00000162999.12	5	9	1.8
lincRNA	AC090877.2	ENSG00000259721.1	5	9	1.8
protein_coding	RASSF2	ENSG00000101265.15	118	212	1.8
protein_coding	TUBB2A	ENSG00000137267.5	54	97	1.8
protein_coding	CREM	ENSG00000095794.19	49	88	1.8
protein_coding	REST	ENSG00000084093.16	176	316	1.8
protein_coding	AP2A1	ENSG00000196961.12	44	79	1.8
protein_coding	TRPM7	ENSG00000092439.14	122	219	1.8
protein_coding	HTT	ENSG00000197386.12	34	61	1.8
protein_coding	ABCC5	ENSG00000114770.16	34	61	1.8
protein_coding	IGFBP5	ENSG00000115461.4	1249	2240	1.8

protein_coding	MIB1	ENSG00000101752.11	121	217	1.8
protein_coding	KPNA2	ENSG00000182481.8	29	52	1.8
protein_coding	IKBKB	ENSG00000104365.14	48	86	1.8
protein_coding	PTPRG	ENSG00000144724.19	196	351	1.8
protein_coding	ANKRD26	ENSG00000107890.16	62	111	1.8
protein_coding	CAMSAP2	ENSG00000118200.14	228	408	1.8
protein_coding	CHML	ENSG00000203668.2	19	34	1.8
protein_coding	PTCD2	ENSG00000049883.14	19	34	1.8
protein_coding	FHOD3	ENSG00000134775.15	52	93	1.8
protein_coding	PTPN3	ENSG00000070159.13	14	25	1.8
protein_coding	RBM47	ENSG00000163694.14	14	25	1.8
protein_coding	TFPT	ENSG00000105619.13	14	25	1.8
processed_transcript	AC021078.1	ENSG00000230551.4	14	25	1.8
protein_coding	DCUN1D2	ENSG00000150401.14	14	25	1.8
protein_coding	C12orf4	ENSG00000047621.11	14	25	1.8
protein_coding	GOLGA4	ENSG00000144674.16	534	953	1.8
protein_coding	BAZ2B	ENSG00000123636.17	130	232	1.8
protein_coding	PPARD	ENSG00000112033.13	51	91	1.8
protein_coding	STXBP5	ENSG00000164506.14	180	321	1.8
protein_coding	RABGGTB	ENSG00000137955.15	60	107	1.8
protein_coding	LRP8	ENSG00000157193.16	23	41	1.8
protein_coding	ALG1	ENSG00000033011.12	32	57	1.8
protein_coding	AMIGO2	ENSG00000139211.6	41	73	1.8
protein_coding	FKBP15	ENSG00000119321.8	54	96	1.8
protein_coding	ARHGAP29	ENSG00000137962.12	27	48	1.8
protein_coding	NINL	ENSG00000101004.14	18	32	1.8
protein_coding	MDC¬†1	ENSG00000137337.14	9	16	1.8
protein_coding	DPH2	ENSG00000132768.13	9	16	1.8
protein_coding	DDX51	ENSG00000185163.9	9	16	1.8
protein_coding	MYEF2	ENSG00000104177.17	9	16	1.8
protein_coding	FANCM	ENSG00000187790.10	9	16	1.8
protein_coding	HOMEZ	ENSG00000215271.7	9	16	1.8
protein_coding	SFPQ	ENSG00000116560.10	210	373	1.8
protein_coding	NBEAL1	ENSG00000144426.18	40	71	1.8
protein_coding	VGLL4	ENSG00000144560.14	71	126	1.8
protein_coding	ATP6V1G1	ENSG00000136888.6	327	580	1.8
protein_coding	TRAPPC6B	ENSG00000182400.14	22	39	1.8
protein_coding	RAB28	ENSG00000157869.14	22	39	1.8
protein_coding	IQCK	ENSG00000174628.16	22	39	1.8
protein_coding	MANBA	ENSG00000109323.9	101	179	1.8
protein_coding	TBCC	ENSG00000124659.6	35	62	1.8
protein_coding	DNAJB11	ENSG00000090520.11	74	131	1.8
protein_coding	C2CD2	ENSG00000157617.16	39	69	1.8
protein_coding	ADCY6	ENSG00000174233.11	26	46	1.8

protein_coding	AVEN	ENSG00000169857.7	26	46	1.8
protein_coding	GRK6	ENSG00000198055.10	13	23	1.8
protein_coding	MSI2	ENSG00000153944.10	13	23	1.8
protein_coding	GYS1	ENSG00000104812.14	30	53	1.8
protein_coding	SNX9	ENSG00000130340.15	260	459	1.8
Mt_rRNA	MT-RNR2	ENSG00000210082.2	42782	75525	1.8
protein_coding	ATP5F1D	ENSG00000099624.7	85	150	1.8
protein_coding	DUSP10	ENSG00000143507.17	51	90	1.8
protein_coding	HMGCL	ENSG00000117305.14	34	60	1.8
protein_coding	PROSER1	ENSG00000120685.19	34	60	1.8
protein_coding	TUBGCP4	ENSG00000137822.12	17	30	1.8
protein_coding	RSF1	ENSG00000048649.13	293	517	1.8
protein_coding	TRIM11	ENSG00000154370.15	38	67	1.8
protein_coding	LSG1	ENSG00000041802.10	38	67	1.8
protein_coding	SUCO	ENSG00000094975.13	59	104	1.8
protein_coding	PDE12	ENSG00000174840.8	21	37	1.8
protein_coding	DPH7	ENSG00000148399.12	21	37	1.8
transcribed_unprocessed_pseudogene	HIST2H2BD	ENSG00000220323.4	21	37	1.8
protein_coding	ABHD5	ENSG00000011198.9	142	250	1.8
protein_coding	CDH11	ENSG00000140937.13	526	926	1.8
protein_coding	DDIT4	ENSG00000168209.4	271	477	1.8
protein_coding	CDKN1C	ENSG00000129757.13	50	88	1.8
protein_coding	SOD2	ENSG00000112096.17	104	183	1.8
protein_coding	SSC5D	ENSG00000179954.15	33	58	1.8
protein_coding	PRPF38B	ENSG00000134186.11	136	239	1.8
protein_coding	IMPAD1	ENSG00000104331.8	239	420	1.8
protein_coding	HDAC4	ENSG00000068024.16	37	65	1.8
protein_coding	ID1	ENSG00000125968.8	41	72	1.8
protein_coding	CCDC186	ENSG00000165813.19	86	151	1.8
protein_coding	H1F0	ENSG00000189060.5	90	158	1.8
protein_coding	ANKRD17	ENSG00000132466.18	234	410	1.8
protein_coding	WASHC4	ENSG00000136051.14	108	189	1.8
protein_coding	DNAJC2	ENSG00000105821.14	64	112	1.8
protein_coding	CHD8	ENSG00000100888.13	48	84	1.8
protein_coding	MICA	ENSG00000204520.12	36	63	1.8
protein_coding	LRIF1	ENSG00000121931.15	24	42	1.8
protein_coding	TMEM164	ENSG00000157600.11	24	42	1.8
protein_coding	CMTM4	ENSG00000183723.12	20	35	1.8
protein_coding	FAM89A	ENSG00000182118.7	20	35	1.8
protein_coding	WDR37	ENSG00000047056.15	20	35	1.8
protein_coding	BRD1	ENSG00000100425.18	16	28	1.8
protein_coding	TBP	ENSG00000112592.13	16	28	1.8

protein_coding	C6orf226	ENSG00000221821.3	16	28	1.8
protein_coding	PATJ	ENSG00000132849.20	12	21	1.8
protein_coding	FBXO42	ENSG00000037637.10	12	21	1.8
protein_coding	RHOD	ENSG00000173156.6	12	21	1.8
protein_coding	FRY	ENSG00000073910.21	12	21	1.8
protein_coding	ZNF493	ENSG00000196268.11	12	21	1.8
protein_coding	CLCN4	ENSG00000073464.11	12	21	1.8
protein_coding	FICD	ENSG00000198855.6	8	14	1.8
protein_coding	CHEK2	ENSG00000183765.21	8	14	1.8
protein_coding	MTSS1	ENSG00000170873.18	8	14	1.8
protein_coding	KLHL29	ENSG00000119771.14	8	14	1.8
protein_coding	ABHD3	ENSG00000158201.9	8	14	1.8
protein_coding	MIEF2	ENSG00000177427.12	8	14	1.8
protein_coding	DSCC1	ENSG00000136982.5	4	7	1.8
protein_coding	ZNF14	ENSG00000105708.8	4	7	1.8
antisense	AC027097.1	ENSG00000267040.6	4	7	1.8
protein_coding	POLM	ENSG00000122678.16	4	7	1.8
antisense	AC027644.3	ENSG00000272831.1	4	7	1.8
protein_coding	PECAM1	ENSG00000261371.5	4	7	1.8
protein_coding	FLVCR1	ENSG00000162769.12	4	7	1.8
protein_coding	LRRC45	ENSG00000169683.7	4	7	1.8
antisense	AC138207.2	ENSG00000264107.1	4	7	1.8
antisense	LRP4-AS1	ENSG00000247675.6	4	7	1.8
protein_coding	CCNA2	ENSG00000145386.9	4	7	1.8
processed_pseudogene	COX6CP1	ENSG00000260318.1	4	7	1.8
protein_coding	ATAD3B	ENSG00000160072.19	4	7	1.8
protein_coding	POLA1	ENSG00000101868.10	4	7	1.8
Mt_tRNA	MT-TC	ENSG00000210140.1	4	7	1.8
sense_intronic	AC084824.4	ENSG00000275854.1	4	7	1.8
protein_coding	IDNK	ENSG00000148057.15	4	7	1.8
protein_coding	YJEFN3	ENSG00000250067.11	4	7	1.8
protein_coding	DAGLA	ENSG00000134780.9	4	7	1.8
protein_coding	ADAMTS3	ENSG00000156140.9	4	7	1.8
transcribed_processed_pseudogene	AC010326.4	ENSG00000270804.1	4	7	1.8
protein_coding	ZNF112	ENSG00000062370.16	4	7	1.8
protein_coding	SBDS	ENSG00000126524.9	206	360	1.7
protein_coding	TBX3	ENSG00000135111.15	463	808	1.7
protein_coding	HSPA1B	ENSG00000204388.6	51	89	1.7
protein_coding	SYNJ2BP	ENSG00000213463.4	47	82	1.7
protein_coding	DDX60L	ENSG00000181381.13	39	68	1.7
protein_coding	GCC2	ENSG00000135968.20	248	432	1.7
protein_coding	IL7R	ENSG00000168685.14	27	47	1.7

protein_coding	ARSA	ENSG00000100299.17	27	47	1.7
protein_coding	TMEM183A	ENSG00000163444.11	27	47	1.7
protein_coding	CEP126	ENSG00000110318.13	77	134	1.7
protein_coding	GSTO1	ENSG00000148834.12	223	388	1.7
protein_coding	GPR137B	ENSG00000077585.13	46	80	1.7
protein_coding	FGFRL1	ENSG00000127418.14	23	40	1.7
protein_coding	RBM24	ENSG00000112183.14	19	33	1.7
protein_coding	CEP295	ENSG00000166004.14	19	33	1.7
protein_coding	SLC25A28	ENSG00000155287.10	19	33	1.7
protein_coding	MAP1B	ENSG00000131711.14	1476	2562	1.7
protein_coding	RMDN3	ENSG00000137824.15	34	59	1.7
protein_coding	PPME1	ENSG00000214517.9	45	78	1.7
protein_coding	ZBTB11	ENSG00000066422.4	30	52	1.7
protein_coding	TCP11L2	ENSG00000166046.10	15	26	1.7
protein_coding	ACVR1B	ENSG00000135503.12	15	26	1.7
protein_coding	TTC21B	ENSG00000123607.14	15	26	1.7
protein_coding	TMEM140	ENSG00000146859.6	41	71	1.7
protein_coding	RRBP1	ENSG00000125844.15	257	445	1.7
protein_coding	HIPK3	ENSG00000110422.11	93	161	1.7
protein_coding	ATG14	ENSG00000126775.8	52	90	1.7
protein_coding	C20orf194	ENSG00000088854.12	26	45	1.7
protein_coding	WARS2	ENSG00000116874.11	26	45	1.7
protein_coding	TMEM94	ENSG00000177728.16	26	45	1.7
protein_coding	HDHD2	ENSG00000167220.11	26	45	1.7
protein_coding	PTGER2	ENSG00000125384.6	26	45	1.7
protein_coding	TMEM33	ENSG00000109133.12	89	154	1.7
protein_coding	DDI2	ENSG00000197312.11	63	109	1.7
protein_coding	UTP25	ENSG00000117597.17	37	64	1.7
protein_coding	UEVLD	ENSG00000151116.16	48	83	1.7
protein_coding	TUSC1	ENSG00000198680.4	22	38	1.7
protein_coding	IPPK	ENSG00000127080.9	11	19	1.7
protein_coding	RPGRIP1L	ENSG00000103494.13	11	19	1.7
protein_coding	SURF2	ENSG00000148291.9	11	19	1.7
protein_coding	ZNF708	ENSG00000182141.10	11	19	1.7
protein_coding	TMEM178A	ENSG00000152154.10	11	19	1.7
protein_coding	HERC3	ENSG00000138641.16	11	19	1.7
protein_coding	CHAF1A	ENSG00000167670.15	11	19	1.7
protein_coding	MARVELD2	ENSG00000152939.15	11	19	1.7
protein_coding	MRPL40	ENSG00000185608.8	51	88	1.7
protein_coding	CSNK1A1	ENSG00000113712.17	320	552	1.7
protein_coding	DNAJB9	ENSG00000128590.4	69	119	1.7
protein_coding	ZNF770	ENSG00000198146.4	87	150	1.7
protein_coding	CNIH4	ENSG00000143771.11	191	329	1.7
protein_coding	HELQ	ENSG00000163312.10	18	31	1.7

protein_coding	TRUB2	ENSG00000167112.9	18	31	1.7
protein_coding	BTN2A2	ENSG00000124508.16	18	31	1.7
protein_coding	GPATCH2L	ENSG00000089916.17	86	148	1.7
protein_coding	TLN2	ENSG00000171914.16	43	74	1.7
protein_coding	UTP3	ENSG00000132467.3	68	117	1.7
protein_coding	PTGFRN	ENSG00000134247.9	25	43	1.7
protein_coding	EOGT	ENSG00000163378.13	57	98	1.7
protein_coding	USP12	ENSG00000152484.13	39	67	1.7
protein_coding	ETS1	ENSG00000134954.14	46	79	1.7
protein_coding	SELENOS	ENSG00000131871.14	159	273	1.7
protein_coding	TM7SF3	ENSG00000064115.10	74	127	1.7
protein_coding	GOLGB1	ENSG00000173230.15	414	710	1.7
protein_coding	BTBD10	ENSG00000148925.10	42	72	1.7
protein_coding	RPL36A	ENSG00000241343.9	28	48	1.7
protein_coding	CXorf38	ENSG00000185753.12	28	48	1.7
protein_coding	B3GNT2	ENSG00000170340.10	21	36	1.7
protein_coding	UBL7	ENSG00000138629.15	21	36	1.7
protein_coding	GMNN	ENSG00000112312.9	21	36	1.7
protein_coding	MCM3	ENSG00000112118.18	21	36	1.7
protein_coding	CNPY3	ENSG00000137161.16	21	36	1.7
protein_coding	ZBTB18	ENSG00000179456.10	14	24	1.7
protein_coding	ARL13B	ENSG00000169379.15	14	24	1.7
protein_coding	ANKRD54	ENSG00000100124.14	14	24	1.7
protein_coding	AASDH	ENSG00000157426.13	14	24	1.7
protein_coding	TUBGCP6	ENSG00000128159.11	14	24	1.7
protein_coding	SPACA9	ENSG00000165698.15	14	24	1.7
protein_coding	RHBDF2	ENSG00000129667.12	7	12	1.7
protein_coding	C19orf47	ENSG00000160392.13	7	12	1.7
protein_coding	PRDM5	ENSG00000138738.10	7	12	1.7
protein_coding	MMACHC	ENSG00000132763.14	7	12	1.7
processed_transcript	LINC00641	ENSG00000258441.1	7	12	1.7
protein_coding	MAP3K6	ENSG00000142733.15	7	12	1.7
protein_coding	ZNF784	ENSG00000179922.5	7	12	1.7
transcribed_unprocessed_pseudogene	MTMR9LP	ENSG00000220785.7	7	12	1.7
protein_coding	ZNF287	ENSG00000141040.14	7	12	1.7
protein_coding	TMEM86A	ENSG00000151117.8	7	12	1.7
protein_coding	EYA2	ENSG00000064655.18	7	12	1.7
protein_coding	SESTD1	ENSG00000187231.13	94	161	1.7
protein_coding	FAM192A	ENSG00000172775.16	66	113	1.7
protein_coding	DYNC1LI1	ENSG00000144635.8	170	291	1.7
protein_coding	SGTA	ENSG00000104969.9	45	77	1.7
protein_coding	TUBGCP2	ENSG00000130640.13	83	142	1.7

protein_coding	NOP58	ENSG00000055044.10	93	159	1.7
protein_coding	MBD2	ENSG00000134046.11	148	253	1.7
protein_coding	ELMOD2	ENSG00000179387.9	48	82	1.7
protein_coding	SH2D4A	ENSG00000104611.11	24	41	1.7
protein_coding	PIK3CB	ENSG00000051382.8	24	41	1.7
protein_coding	CD40	ENSG00000101017.13	34	58	1.7
protein_coding	ZNF263	ENSG00000006194.9	17	29	1.7
protein_coding	LENG8	ENSG00000167615.16	17	29	1.7
protein_coding	POLR3B	ENSG00000013503.9	17	29	1.7
protein_coding	PIGO	ENSG00000165282.13	17	29	1.7
protein_coding	BNIP3L	ENSG00000104765.15	774	1320	1.7
protein_coding	RNF152	ENSG00000176641.10	61	104	1.7
protein_coding	AKAP12	ENSG00000131016.16	44	75	1.7
protein_coding	SPEN	ENSG00000065526.10	64	109	1.7
protein_coding	POMT2	ENSG00000009830.11	37	63	1.7
antisense	LINC00632	ENSG00000203930.11	47	80	1.7
protein_coding	ZMIZ1	ENSG00000108175.16	151	257	1.7
protein_coding	AGFG1	ENSG00000173744.17	110	187	1.7
protein_coding	TRAF3IP2	ENSG00000056972.18	80	136	1.7
protein_coding	CBWD1	ENSG00000172785.18	20	34	1.7
protein_coding	ANAPC15	ENSG00000110200.8	20	34	1.7
protein_coding	TNFAIP3	ENSG00000118503.14	10	17	1.7
protein_coding	ERN¬†1	ENSG00000178607.15	10	17	1.7
protein_coding	C2CD2L	ENSG00000172375.13	10	17	1.7
antisense	AC092069.1	ENSG00000267458.1	10	17	1.7
protein_coding	FXN	ENSG00000165060.12	10	17	1.7
protein_coding	ZNF577	ENSG00000161551.14	10	17	1.7
protein_coding	PPP1R26	ENSG00000196422.10	10	17	1.7
protein_coding	WDR5B	ENSG00000196981.3	10	17	1.7
protein_coding	C15orf41	ENSG00000186073.12	10	17	1.7
protein_coding	CCAR1	ENSG00000060339.13	266	452	1.7
protein_coding	SREK1	ENSG00000153914.15	159	270	1.7
protein_coding	ASS1	ENSG00000130707.17	43	73	1.7
protein_coding	PRKCA	ENSG00000154229.11	171	290	1.7
protein_coding	HAT1	ENSG00000128708.12	46	78	1.7
protein_coding	FOSL2	ENSG00000075426.11	406	688	1.7
protein_coding	ARPP19	ENSG00000128989.10	88	149	1.7
protein_coding	ARHGAP21	ENSG00000107863.17	267	452	1.7
protein_coding	TOR1B	ENSG00000136816.15	65	110	1.7
protein_coding	ADAM19	ENSG00000135074.15	39	66	1.7
protein_coding	NSMF	ENSG00000165802.22	13	22	1.7
protein_coding	ST7L	ENSG00000007341.18	13	22	1.7
protein_coding	SLC25A22	ENSG00000177542.10	13	22	1.7
protein_coding	CRCP	ENSG00000241258.6	71	120	1.7

lincRNA	CRNDE	ENSG00000245694.9	29	49	1.7
protein_coding	PRRC2C	ENSG00000117523.16	428	723	1.7
protein_coding	DCAF12	ENSG00000198876.12	45	76	1.7
protein_coding	EYA4	ENSG00000112319.18	106	179	1.7
protein_coding	ZFR	ENSG00000056097.15	228	385	1.7
protein_coding	ODF2L	ENSG00000122417.15	93	157	1.7
protein_coding	CYP2U1	ENSG00000155016.17	48	81	1.7
protein_coding	ZNF331	ENSG00000130844.17	32	54	1.7
protein_coding	CYB561D1	ENSG00000174151.14	16	27	1.7
protein_coding	GOLGA2	ENSG00000167110.17	115	194	1.7
protein_coding	SEPHS2	ENSG00000179918.18	99	167	1.7
protein_coding	PGRMC2	ENSG00000164040.16	830	1400	1.7
protein_coding	LPGAT1	ENSG00000123684.12	83	140	1.7
protein_coding	CEP104	ENSG00000116198.12	54	91	1.7
protein_coding	TNRC18	ENSG00000182095.14	57	96	1.7
protein_coding	C6orf47	ENSG00000204439.3	19	32	1.7
protein_coding	TBC1D12	ENSG00000108239.8	19	32	1.7
protein_coding	WDYHV1	ENSG00000156795.7	19	32	1.7
protein_coding	ARID4B	ENSG00000054267.21	183	308	1.7
protein_coding	UBN2	ENSG00000157741.14	41	69	1.7
protein_coding	CEP63	ENSG00000182923.17	47	79	1.7
protein_coding	PGGT1B	ENSG00000164219.9	47	79	1.7
protein_coding	IGF1R	ENSG00000140443.14	150	252	1.7
protein_coding	WDR44	ENSG00000131725.13	25	42	1.7
protein_coding	HMGB2	ENSG00000164104.11	25	42	1.7
protein_coding	RNF19A	ENSG00000034677.12	28	47	1.7
protein_coding	RFWD3	ENSG00000168411.13	28	47	1.7
protein_coding	PAFAH2	ENSG00000158006.13	28	47	1.7
processed_transcript	LINC01578	ENSG00000272888.6	124	208	1.7
protein_coding	MMD	ENSG00000108960.8	31	52	1.7
protein_coding	RABL2B	ENSG00000079974.17	31	52	1.7
protein_coding	WDR60	ENSG00000126870.15	37	62	1.7
protein_coding	PRRX2	ENSG00000167157.10	163	273	1.7
protein_coding	SOCS6	ENSG00000170677.5	43	72	1.7
protein_coding	PANX1	ENSG00000110218.8	49	82	1.7
protein_coding	IPMK	ENSG00000151151.5	49	82	1.7
protein_coding	PRPF4B	ENSG00000112739.16	208	348	1.7
protein_coding	PKN2	ENSG00000065243.19	58	97	1.7
protein_coding	SYAP1	ENSG00000169895.5	342	570	1.7
protein_coding	RDH11	ENSG00000072042.12	216	360	1.7
protein_coding	SCRN1	ENSG00000136193.16	138	230	1.7
protein_coding	TMEM189	ENSG00000240849.10	63	105	1.7
protein_coding	MSL2	ENSG00000174579.3	39	65	1.7
protein_coding	MEPCE	ENSG00000146834.13	36	60	1.7

protein_coding	CPTP	ENSG00000224051.6	33	55	1.7
protein_coding	DUSP5	ENSG00000138166.5	30	50	1.7
protein_coding	TCAF1	ENSG00000198420.9	30	50	1.7
protein_coding	NTMT1	ENSG00000148335.14	24	40	1.7
protein_coding	SNRNP35	ENSG00000184209.14	18	30	1.7
protein_coding	PAQR3	ENSG00000163291.14	18	30	1.7
antisense	AC005034.3	ENSG00000270696.1	18	30	1.7
protein_coding	DUS1L	ENSG00000169718.17	18	30	1.7
protein_coding	TRAPPC6A	ENSG00000007255.10	15	25	1.7
protein_coding	NMNAT1	ENSG00000173614.13	12	20	1.7
protein_coding	RABGGTA	ENSG00000100949.14	12	20	1.7
protein_coding	GMPPB	ENSG00000173540.12	12	20	1.7
protein_coding	FAM162B	ENSG00000183807.7	12	20	1.7
protein_coding	PTPDC1	ENSG00000158079.15	9	15	1.7
protein_coding	NCAPH2	ENSG00000025770.18	9	15	1.7
protein_coding	BORCS5	ENSG00000165714.10	9	15	1.7
protein_coding	DARS2	ENSG00000117593.10	9	15	1.7
protein_coding	BCL2A1	ENSG00000140379.7	6	10	1.7
protein_coding	POP1	ENSG00000104356.10	6	10	1.7
protein_coding	FANCI	ENSG00000140525.17	6	10	1.7
protein_coding	NOC4L	ENSG00000184967.6	6	10	1.7
protein_coding	NEU3	ENSG00000162139.9	6	10	1.7
protein_coding	BBS5	ENSG00000163093.11	6	10	1.7
processed_pseudogene	AC016739.1	ENSG00000218175.2	6	10	1.7
sense_overlapping	AL157394.1	ENSG00000261438.1	6	10	1.7
protein_coding	WDPCP	ENSG00000143951.15	6	10	1.7
protein_coding	BMPER	ENSG00000164619.9	6	10	1.7
protein_coding	AMER1	ENSG00000184675.10	6	10	1.7
protein_coding	ME3	ENSG00000151376.16	6	10	1.7
protein_coding	SH2D2A	ENSG00000027869.11	3	5	1.7
protein_coding	RFX8	ENSG00000196460.13	3	5	1.7
protein_coding	PARP10	ENSG00000178685.13	3	5	1.7
protein_coding	CTXN1	ENSG00000178531.5	3	5	1.7
protein_coding	GALNT12	ENSG00000119514.6	3	5	1.7
protein_coding	SPAG5	ENSG00000076382.16	3	5	1.7
protein_coding	WWOX	ENSG00000186153.16	3	5	1.7
bidirectional_promoter_lncRNA	AC244033.2	ENSG00000285184.2	3	5	1.7
protein_coding	RGS14	ENSG00000169220.17	3	5	1.7
protein_coding	TMEM170B	ENSG00000205269.5	3	5	1.7
protein_coding	GABRE	ENSG00000102287.18	3	5	1.7
protein_coding	TACC3	ENSG00000013810.18	3	5	1.7
processed_pseudogene	RPL21P44	ENSG00000229585.4	3	5	1.7

protein_coding	SEMA3D	ENSG00000153993.13	3	5	1.7
protein_coding	CFAP52	ENSG00000166596.14	3	5	1.7
lincRNA	AL135925.1	ENSG00000272447.1	3	5	1.7
protein_coding	GRTP1	ENSG00000139835.13	3	5	1.7
protein_coding	CFD	ENSG00000197766.7	3	5	1.7
processed_pseudogene	AC090543.2	ENSG00000243403.1	3	5	1.7
lincRNA	GS1-124K5.4	ENSG00000237310.1	3	5	1.7
antisense	TARID	ENSG00000227954.6	3	5	1.7
protein_coding	RFC3	ENSG00000133119.12	3	5	1.7
processed_pseudogene	AC004453.1	ENSG00000146677.7	3	5	1.7
antisense	DLEU2	ENSG00000231607.10	3	5	1.7
protein_coding	CFAP53	ENSG00000172361.5	3	5	1.7
protein_coding	SWSAP1	ENSG00000173928.2	3	5	1.7
protein_coding	SEMA4G	ENSG00000095539.15	3	5	1.7
protein_coding	STAT4	ENSG00000138378.18	3	5	1.7
protein_coding	RNF215	ENSG00000099999.14	3	5	1.7
protein_coding	PERM1	ENSG00000187642.9	3	5	1.7
protein_coding	CCDC134	ENSG00000100147.13	3	5	1.7
protein_coding	SORD	ENSG00000140263.13	3	5	1.7
antisense	AL391834.2	ENSG00000273226.1	3	5	1.7
protein_coding	INSC	ENSG00000188487.11	3	5	1.7
transcribed_unprocessed_pseudogene	EMC3-AS1	ENSG00000180385.8	3	5	1.7
processed_pseudogene	NPM1P27	ENSG00000249353.2	3	5	1.7
protein_coding	ZNF416	ENSG00000083817.8	3	5	1.7
antisense	LPP-AS2	ENSG00000270959.1	3	5	1.7
lincRNA	AL450326.1	ENSG00000230555.2	3	5	1.7
protein_coding	ZNF724	ENSG00000196081.9	3	5	1.7
lincRNA	AC026367.2	ENSG00000275409.1	3	5	1.7
protein_coding	AMACR	ENSG00000242110.7	3	5	1.7
transcribed_unprocessed_pseudogene	AL669831.4	ENSG00000230092.7	3	5	1.7
protein_coding	ARL¬†9	ENSG00000196503.4	3	5	1.7
lincRNA	LINC00607	ENSG00000235770.5	3	5	1.7
protein_coding	DKK¬†2	ENSG00000155011.8	3	5	1.7
protein_coding	SCN2B	ENSG00000149575.5	3	5	1.7
protein_coding	CCL2	ENSG00000108691.9	137	228	1.7
protein_coding	GIGYF2	ENSG00000204120.14	116	193	1.7
protein_coding	COL8A1	ENSG00000144810.15	1368	2276	1.7
protein_coding	MAN1A2	ENSG00000198162.12	282	469	1.7
protein_coding	TWISTNB	ENSG00000105849.5	65	108	1.7
protein_coding	EBNA1BP2	ENSG00000117395.12	62	103	1.7
protein_coding	C11orf95	ENSG00000188070.9	118	196	1.7

protein_coding	MAP4K3	ENSG00000011566.14	50	83	1.7
protein_coding	HCFC2	ENSG00000111727.11	79	131	1.7
protein_coding	ANKRD29	ENSG00000154065.16	111	184	1.7
protein_coding	WNT5A	ENSG00000114251.14	2785	4616	1.7
protein_coding	SLC16A3	ENSG00000141526.16	178	295	1.7
protein_coding	KRR1	ENSG00000111615.13	140	232	1.7
protein_coding	MAP3K5	ENSG00000197442.9	35	58	1.7
processed_transcript	SNHG14	ENSG00000224078.13	35	58	1.7
protein_coding	KAT6B	ENSG00000156650.13	137	227	1.7
protein_coding	ISG20L2	ENSG00000143319.16	29	48	1.7
protein_coding	ATP11A	ENSG00000068650.18	26	43	1.7
protein_coding	NSDHL	ENSG00000147383.10	26	43	1.7
protein_coding	ALDH2	ENSG00000111275.12	49	81	1.7
protein_coding	CHD9	ENSG00000177200.17	144	238	1.7
protein_coding	MEF2A	ENSG00000068305.17	69	114	1.7
protein_coding	CSNK1G3	ENSG00000151292.17	69	114	1.7
protein_coding	SFXN1	ENSG00000164466.12	66	109	1.7
protein_coding	HNRNPA3	ENSG00000170144.20	66	109	1.7
protein_coding	GRPEL1	ENSG00000109519.12	43	71	1.7
protein_coding	ACSS2	ENSG00000131069.19	20	33	1.7
protein_coding	RFTN1	ENSG00000131378.13	324	534	1.6
protein_coding	FNBP4	ENSG00000109920.12	85	140	1.6
protein_coding	CWC25	ENSG00000273559.4	34	56	1.6
protein_coding	PIGQ	ENSG00000007541.16	17	28	1.6
protein_coding	CDK9	ENSG00000136807.13	17	28	1.6
protein_coding	KIF7	ENSG00000166813.14	17	28	1.6
protein_coding	HLA-DPB1	ENSG00000223865.10	17	28	1.6
protein_coding	SLC22A4	ENSG00000197208.5	17	28	1.6
protein_coding	SRD5A1	ENSG00000145545.11	62	102	1.6
protein_coding	LIN7A	ENSG00000111052.7	31	51	1.6
protein_coding	USP1	ENSG00000162607.12	45	74	1.6
protein_coding	VKORC1L1	ENSG00000196715.6	59	97	1.6
protein_coding	FUBP1	ENSG00000162613.16	73	120	1.6
protein_coding	SMARCA5	ENSG00000153147.5	188	309	1.6
protein_coding	CHD6	ENSG00000124177.14	56	92	1.6
protein_coding	EXOC8	ENSG00000116903.7	28	46	1.6
protein_coding	ZNF721	ENSG00000182903.15	14	23	1.6
protein_coding	KSR1	ENSG00000141068.14	14	23	1.6
protein_coding	UTP15	ENSG00000164338.9	14	23	1.6
protein_coding	FRAT1	ENSG00000165879.8	14	23	1.6
protein_coding	GGA3	ENSG00000125447.17	14	23	1.6
protein_coding	XPO4	ENSG00000132953.16	14	23	1.6
protein_coding	TMEM187	ENSG00000177854.7	14	23	1.6
protein_coding	MED9	ENSG00000141026.5	14	23	1.6

protein_coding	C12orf49	ENSG00000111412.5	39	64	1.6
protein_coding	IRS2	ENSG00000185950.8	114	187	1.6
protein_coding	CD276	ENSG00000103855.17	100	164	1.6
protein_coding	TMEM192	ENSG00000170088.13	61	100	1.6
protein_coding	TPST1	ENSG00000169902.14	133	218	1.6
protein_coding	ROCK1	ENSG00000067900.7	349	572	1.6
protein_coding	MIA2	ENSG00000150527.17	36	59	1.6
protein_coding	NARS	ENSG00000134440.11	171	280	1.6
protein_coding	ODR4	ENSG00000157181.15	44	72	1.6
protein_coding	CD2AP	ENSG00000198087.7	33	54	1.6
protein_coding	COPS7A	ENSG00000111652.9	33	54	1.6
protein_coding	DCP1B	ENSG00000151065.13	33	54	1.6
protein_coding	SNX30	ENSG00000148158.16	22	36	1.6
protein_coding	ENOX2	ENSG00000165675.16	22	36	1.6
protein_coding	ZNF117	ENSG00000152926.14	11	18	1.6
protein_coding	C18orf54	ENSG00000166845.14	11	18	1.6
protein_coding	LNPK	ENSG00000144320.13	228	373	1.6
protein_coding	ADIPOR1	ENSG00000159346.12	96	157	1.6
protein_coding	NFIC	ENSG00000141905.18	1083	1771	1.6
protein_coding	DPY19L3	ENSG00000178904.18	41	67	1.6
protein_coding	LURAP1L	ENSG00000153714.5	41	67	1.6
antisense	AL391422.4	ENSG00000270504.1	30	49	1.6
protein_coding	LMAN1	ENSG00000074695.5	1167	1905	1.6
protein_coding	MIGA1	ENSG00000180488.15	57	93	1.6
protein_coding	SLC35B4	ENSG00000205060.10	38	62	1.6
protein_coding	RGS16	ENSG00000143333.6	19	31	1.6
protein_coding	ITGB3BP	ENSG00000142856.16	19	31	1.6
protein_coding	TACO1	ENSG00000136463.7	19	31	1.6
protein_coding	SERPINB1	ENSG00000021355.12	84	137	1.6
protein_coding	CFAP36	ENSG00000163001.11	127	207	1.6
protein_coding	KIAA0556	ENSG00000047578.12	27	44	1.6
protein_coding	RNF123	ENSG00000164068.15	27	44	1.6
protein_coding	KIAA1586	ENSG00000168116.13	27	44	1.6
protein_coding	OSBPL11	ENSG00000144909.7	27	44	1.6
protein_coding	TMEM8B	ENSG00000137103.19	27	44	1.6
protein_coding	NID2	ENSG00000087303.17	251	409	1.6
protein_coding	DNAJC1	ENSG00000136770.10	151	246	1.6
protein_coding	ITSN2	ENSG00000198399.14	121	197	1.6
protein_coding	SLC35G2	ENSG00000168917.8	43	70	1.6
protein_coding	NCLN	ENSG00000125912.10	51	83	1.6
protein_coding	ZNF462	ENSG00000148143.12	59	96	1.6
protein_coding	RAD23A	ENSG00000179262.9	134	218	1.6
protein_coding	PGM2L1	ENSG00000165434.7	281	457	1.6
protein_coding	TANC2	ENSG00000170921.15	120	195	1.6

protein_coding	GRAMD1A	ENSG00000089351.14	64	104	1.6
protein_coding	KIF3A	ENSG00000131437.15	40	65	1.6
protein_coding	TEP1	ENSG00000129566.12	40	65	1.6
protein_coding	PAXBP1	ENSG00000159086.14	32	52	1.6
protein_coding	KRI1	ENSG00000129347.19	24	39	1.6
protein_coding	ZNF576	ENSG00000124444.15	16	26	1.6
protein_coding	CSAD	ENSG00000139631.18	16	26	1.6
protein_coding	GNE	ENSG00000159921.15	16	26	1.6
protein_coding	PLEKHM3	ENSG00000178385.14	16	26	1.6
protein_coding	MED7	ENSG00000155868.7	16	26	1.6
protein_coding	PRPS1	ENSG00000147224.11	16	26	1.6
protein_coding	NOVA1	ENSG00000139910.19	16	26	1.6
protein_coding	ANKRD37	ENSG00000186352.8	8	13	1.6
protein_coding	SPRY4	ENSG00000187678.9	8	13	1.6
protein_coding	FMNL1	ENSG00000184922.13	8	13	1.6
TEC	AL359922.3	ENSG00000279670.1	8	13	1.6
lincRNA	PWAR6	ENSG00000257151.1	8	13	1.6
protein_coding	SPATA5L1	ENSG00000171763.18	8	13	1.6
protein_coding	UBXN2B	ENSG00000215114.8	8	13	1.6
protein_coding	JKAMP	ENSG00000050130.17	117	190	1.6
protein_coding	GREM1	ENSG00000166923.10	5051	8201	1.6
protein_coding	MAP7D3	ENSG00000129680.15	130	211	1.6
protein_coding	TTC28	ENSG00000100154.14	90	146	1.6
protein_coding	LUC7L	ENSG00000007392.16	45	73	1.6
protein_coding	RCOR1	ENSG00000089902.9	37	60	1.6
protein_coding	RPRD1A	ENSG00000141425.17	37	60	1.6
protein_coding	BCOR	ENSG00000183337.16	29	47	1.6
protein_coding	ANO8	ENSG00000074855.10	29	47	1.6
protein_coding	CBLB	ENSG00000114423.20	42	68	1.6
protein_coding	ENTR1	ENSG00000165689.16	21	34	1.6
protein_coding	IFFO2	ENSG00000169991.10	21	34	1.6
protein_coding	CDC27	ENSG00000004897.11	212	343	1.6
protein_coding	LAMTOR4	ENSG00000188186.10	102	165	1.6
protein_coding	CMKLR1	ENSG00000174600.13	68	110	1.6
protein_coding	RALGPS2	ENSG00000116191.17	81	131	1.6
protein_coding	REV3L	ENSG00000009413.15	316	511	1.6
protein_coding	CWC27	ENSG00000153015.15	94	152	1.6
protein_coding	CCND1	ENSG00000110092.3	1076	1739	1.6
protein_coding	PPP3CA	ENSG00000138814.16	429	693	1.6
protein_coding	FLNB	ENSG00000136068.14	104	168	1.6
protein_coding	RBL2	ENSG00000103479.15	65	105	1.6
protein_coding	SATB2	ENSG00000119042.16	52	84	1.6
protein_coding	TGS1	ENSG00000137574.10	26	42	1.6
protein_coding	MED18	ENSG00000130772.13	26	42	1.6

protein_coding	NUP58	ENSG00000139496.15	13	21	1.6
protein_coding	ITFG2	ENSG00000111203.12	13	21	1.6
protein_coding	HEXIM2	ENSG00000168517.10	13	21	1.6
protein_coding	MED20	ENSG00000124641.15	13	21	1.6
protein_coding	KIAA1614	ENSG00000135835.11	13	21	1.6
protein_coding	DHRS4L2	ENSG00000187630.16	13	21	1.6
protein_coding	COQ7	ENSG00000167186.10	13	21	1.6
protein_coding	NOTCH1	ENSG00000148400.10	13	21	1.6
protein_coding	RBMS1	ENSG00000153250.19	457	738	1.6
protein_coding	ESF1	ENSG00000089048.14	88	142	1.6
protein_coding	PEG10	ENSG00000242265.5	62	100	1.6
protein_coding	ZDHHC12	ENSG00000160446.18	31	50	1.6
protein_coding	SLK	ENSG00000065613.13	328	529	1.6
protein_coding	ARL6IP5	ENSG00000144746.6	958	1545	1.6
protein_coding	AGPAT1	ENSG00000204310.12	98	158	1.6
protein_coding	PCNT	ENSG00000160299.16	49	79	1.6
protein_coding	VASN	ENSG00000168140.4	188	303	1.6
protein_coding	STAM2	ENSG00000115145.9	54	87	1.6
protein_coding	NFIB	ENSG00000147862.16	18	29	1.6
protein_coding	OXCT1	ENSG00000083720.12	18	29	1.6
protein_coding	PKN1	ENSG00000123143.12	18	29	1.6
protein_coding	ZFP14	ENSG00000142065.13	18	29	1.6
protein_coding	BCL3	ENSG00000069399.14	59	95	1.6
protein_coding	MOSPD2	ENSG00000130150.11	41	66	1.6
protein_coding	ATP6V0B	ENSG00000117410.13	87	140	1.6
protein_coding	FKBP2	ENSG00000173486.12	110	177	1.6
protein_coding	OSR2	ENSG00000164920.9	110	177	1.6
protein_coding	RAB31	ENSG00000168461.12	665	1070	1.6
protein_coding	HLTF	ENSG00000071794.15	97	156	1.6
protein_coding	ANKRD10	ENSG00000088448.14	135	217	1.6
protein_coding	OTUD4	ENSG00000164164.16	28	45	1.6
protein_coding	SLC39A7	ENSG00000112473.17	208	334	1.6
protein_coding	WTIP	ENSG00000142279.12	38	61	1.6
protein_coding	CPSF3	ENSG00000119203.13	43	69	1.6
protein_coding	METTL8	ENSG00000123600.19	43	69	1.6
protein_coding	SOS¬†2	ENSG00000100485.11	48	77	1.6
protein_coding	CSRNP2	ENSG00000110925.6	48	77	1.6
protein_coding	BIRC6	ENSG00000115760.14	106	170	1.6
protein_coding	TPR	ENSG00000047410.13	270	433	1.6
protein_coding	SLTM	ENSG00000137776.16	179	287	1.6
protein_coding	CMAS	ENSG00000111726.12	68	109	1.6
protein_coding	APBB1IP	ENSG00000077420.15	156	250	1.6
protein_coding	MLEC	ENSG00000110917.7	196	314	1.6
protein_coding	PDE7B	ENSG00000171408.13	175	280	1.6

protein_coding	TFDP2	ENSG00000114126.17	95	152	1.6
protein_coding	SKIL	ENSG00000136603.13	70	112	1.6
protein_coding	TRAF3IP1	ENSG00000204104.11	60	96	1.6
protein_coding	CNOT4	ENSG00000080802.18	55	88	1.6
protein_coding	DBT	ENSG00000137992.14	55	88	1.6
protein_coding	SDC3	ENSG00000162512.15	50	80	1.6
protein_coding	GGH	ENSG00000137563.11	50	80	1.6
protein_coding	MICALL2	ENSG00000164877.18	45	72	1.6
protein_coding	ZBTB43	ENSG00000169155.9	35	56	1.6
protein_coding	SH2B1	ENSG00000178188.14	35	56	1.6
protein_coding	SHC3	ENSG00000148082.9	30	48	1.6
protein_coding	EDNRA	ENSG00000151617.16	25	40	1.6
protein_coding	ATXN1L	ENSG00000224470.7	25	40	1.6
protein_coding	LCLAT1	ENSG00000172954.13	20	32	1.6
protein_coding	UPP1	ENSG00000183696.13	15	24	1.6
protein_coding	ZFP30	ENSG00000120784.16	15	24	1.6
protein_coding	SEMA4F	ENSG00000135622.12	15	24	1.6
protein_coding	ZSWIM9	ENSG00000185453.12	10	16	1.6
protein_coding	MTRNR2L8	ENSG00000255823.4	10	16	1.6
protein_coding	PRMT9	ENSG00000164169.12	10	16	1.6
protein_coding	TMEM231	ENSG00000205084.10	10	16	1.6
protein_coding	TSPAN13	ENSG00000106537.7	5	8	1.6
protein_coding	FRMD4B	ENSG00000114541.14	5	8	1.6
protein_coding	CENPT	ENSG00000102901.12	5	8	1.6
protein_coding	FUT10	ENSG00000172728.15	5	8	1.6
protein_coding	IRF7	ENSG00000185507.20	5	8	1.6
protein_coding	ADGRB2	ENSG00000121753.12	5	8	1.6
protein_coding	TTLL11	ENSG00000175764.14	5	8	1.6
protein_coding	CD3EAP	ENSG00000117877.10	5	8	1.6
protein_coding	EXOC6	ENSG00000138190.16	5	8	1.6
antisense	AP000254.1	ENSG00000273271.1	5	8	1.6
protein_coding	HTR7	ENSG00000148680.15	5	8	1.6
protein_coding	IL15	ENSG00000164136.16	5	8	1.6
processed_pseudogene	HNRNPA3P6	ENSG00000213300.5	5	8	1.6
protein_coding	ST8SIA2	ENSG00000140557.11	5	8	1.6
protein_coding	TSPAN12	ENSG00000106025.8	5	8	1.6
protein_coding	MID2	ENSG00000080561.13	5	8	1.6
protein_coding	RFC4	ENSG00000163918.10	5	8	1.6
protein_coding	SOX15	ENSG00000129194.7	5	8	1.6
antisense	AL160153.1	ENSG00000276012.1	5	8	1.6
protein_coding	ZNF273	ENSG00000198039.11	5	8	1.6
protein_coding	CREB3L4	ENSG00000143578.15	5	8	1.6
protein_coding	TNNC2	ENSG00000101470.9	5	8	1.6
protein_coding	ANKRD11	ENSG00000167522.15	440	703	1.6

protein_coding	BSG	ENSG00000172270.19	348	556	1.6
protein_coding	LTV1	ENSG00000135521.8	82	131	1.6
protein_coding	MPDZ	ENSG00000107186.16	72	115	1.6
protein_coding	RBM25	ENSG00000119707.13	233	372	1.6
protein_coding	PPID	ENSG00000171497.4	57	91	1.6
protein_coding	CIAO2A	ENSG00000166797.10	57	91	1.6
protein_coding	TP53RK	ENSG00000172315.5	94	150	1.6
protein_coding	GXYLT1	ENSG00000151233.10	79	126	1.6
protein_coding	TOPBP1	ENSG00000163781.13	32	51	1.6
protein_coding	NEIL2	ENSG00000154328.15	32	51	1.6
protein_coding	NMRAL1	ENSG00000153406.13	32	51	1.6
protein_coding	ACO1	ENSG00000122729.18	123	196	1.6
protein_coding	GALNT2	ENSG00000143641.9	312	497	1.6
protein_coding	DCUN1D4	ENSG00000109184.14	54	86	1.6
protein_coding	CDADC1	ENSG00000102543.14	27	43	1.6
protein_coding	TDP2	ENSG00000111802.13	103	164	1.6
protein_coding	PIK3CA	ENSG00000121879.4	125	199	1.6
protein_coding	MZT1	ENSG00000204899.5	22	35	1.6
protein_coding	DYNLT3	ENSG00000165169.10	166	264	1.6
protein_coding	SAMD4B	ENSG00000179134.15	166	264	1.6
protein_coding	TANC1	ENSG00000115183.14	83	132	1.6
protein_coding	KIF1BP	ENSG00000198954.7	61	97	1.6
protein_coding	KCTD3	ENSG00000136636.12	117	186	1.6
protein_coding	JMJD1C	ENSG00000171988.18	78	124	1.6
protein_coding	SIRT1	ENSG00000096717.11	39	62	1.6
protein_coding	SMIM12	ENSG00000163866.8	39	62	1.6
protein_coding	SREK1IP1	ENSG00000153006.15	158	251	1.6
protein_coding	KLHL28	ENSG00000179454.13	34	54	1.6
protein_coding	RANBP6	ENSG00000137040.9	34	54	1.6
protein_coding	SLC16A5	ENSG00000170190.15	17	27	1.6
protein_coding	CCDC97	ENSG00000142039.3	17	27	1.6
protein_coding	PCTP	ENSG00000141179.13	17	27	1.6
protein_coding	PAN3	ENSG00000152520.13	29	46	1.6
protein_coding	PHC2	ENSG00000134686.18	70	111	1.6
protein_coding	ADCY3	ENSG00000138031.14	41	65	1.6
protein_coding	ASXL2	ENSG00000143970.16	77	122	1.6
protein_coding	AP5Z1	ENSG00000242802.7	77	122	1.6
protein_coding	PNRC2	ENSG00000189266.12	72	114	1.6
transcribed_unprocessed_pseudogene	LINC00888	ENSG00000240024.5	36	57	1.6
transcribed_unprocessed_pseudogene	HIST2H2BC	ENSG00000261716.2	12	19	1.6
protein_coding	TTC26	ENSG00000105948.13	12	19	1.6

protein_coding	PXMP2	ENSG00000176894.9	12	19	1.6
protein_coding	DENND4B	ENSG00000198837.9	12	19	1.6
protein_coding	FAM117B	ENSG00000138439.11	12	19	1.6
protein_coding	TESK2	ENSG00000070759.16	12	19	1.6
protein_coding	ZNF469	ENSG00000225614.2	12	19	1.6
protein_coding	RBBP6	ENSG00000122257.19	215	340	1.6
protein_coding	E2F3	ENSG00000112242.14	74	117	1.6
protein_coding	PRRG1	ENSG00000130962.17	62	98	1.6
protein_coding	ARID5B	ENSG00000150347.15	607	959	1.6
protein_coding	AFF4	ENSG00000072364.12	247	390	1.6
protein_coding	NELFCD	ENSG00000101158.13	38	60	1.6
protein_coding	CBX7	ENSG00000100307.12	38	60	1.6
protein_coding	MTFR1	ENSG00000066855.15	19	30	1.6
protein_coding	SPATA2	ENSG00000158480.10	19	30	1.6
protein_coding	SMC2	ENSG00000136824.18	85	134	1.6
protein_coding	MRPL32	ENSG00000106591.3	85	134	1.6
protein_coding	SLC35A2	ENSG00000102100.15	33	52	1.6
protein_coding	YME1L1	ENSG00000136758.18	205	323	1.6
protein_coding	ZC3H13	ENSG00000123200.16	219	345	1.6
protein_coding	ZBTB41	ENSG00000177888.7	40	63	1.6
protein_coding	LCORL	ENSG00000178177.15	40	63	1.6
protein_coding	YES1	ENSG00000176105.13	134	211	1.6
protein_coding	LRRC8A	ENSG00000136802.11	122	192	1.6
lincRNA	FTX	ENSG00000230590.9	89	140	1.6
protein_coding	SAR1A	ENSG00000079332.14	213	335	1.6
protein_coding	TMEM63B	ENSG00000137216.18	110	173	1.6
protein_coding	PPP4R2	ENSG00000163605.14	182	286	1.6
protein_coding	FAM208A	ENSG00000163946.13	105	165	1.6
protein_coding	RDH14	ENSG00000240857.1	63	99	1.6
protein_coding	SOCS4	ENSG00000180008.8	56	88	1.6
protein_coding	CCDC174	ENSG00000154781.15	49	77	1.6
protein_coding	KPNA5	ENSG00000196911.10	28	44	1.6
protein_coding	LBR	ENSG00000143815.14	28	44	1.6
protein_coding	CXXC1	ENSG00000154832.14	21	33	1.6
protein_coding	ANG	ENSG00000214274.9	21	33	1.6
transcribed_unprocessed_pseudogene	SVIL-AS1	ENSG00000224597.10	14	22	1.6
protein_coding	ADCK2	ENSG00000133597.10	14	22	1.6
protein_coding	FANCC	ENSG00000158169.13	14	22	1.6
protein_coding	CCNB1	ENSG00000134057.14	14	22	1.6
protein_coding	CACTIN	ENSG00000105298.13	14	22	1.6
protein_coding	ZFAND2A	ENSG00000178381.11	14	22	1.6
protein_coding	ITGA6	ENSG00000091409.14	7	11	1.6

protein_coding	DZIP1L	ENSG00000158163.14	7	11	1.6
protein_coding	VSIG10L	ENSG00000186806.5	7	11	1.6
protein_coding	ENTPD1	ENSG00000138185.19	7	11	1.6
protein_coding	YOD1	ENSG00000180667.10	7	11	1.6
protein_coding	TMEM100	ENSG00000166292.11	7	11	1.6
protein_coding	ZNF813	ENSG00000198346.10	7	11	1.6
protein_coding	PROSER3	ENSG00000167595.15	7	11	1.6
protein_coding	PNPLA3	ENSG00000100344.10	7	11	1.6
antisense	ZNF503-AS2	ENSG00000237149.5	7	11	1.6
protein_coding	SP9	ENSG00000217236.1	7	11	1.6
protein_coding	AFG1L	ENSG00000135537.16	7	11	1.6
protein_coding	PHKG1	ENSG00000164776.9	7	11	1.6
protein_coding	ATP6AP1L	ENSG00000205464.12	7	11	1.6
protein_coding	FLRT2	ENSG00000185070.10	328	515	1.6
protein_coding	IL17RA	ENSG00000177663.13	86	135	1.6
protein_coding	MANF	ENSG00000145050.16	79	124	1.6
protein_coding	PDIA3	ENSG00000167004.12	460	722	1.6
protein_coding	FAAP20	ENSG00000162585.16	51	80	1.6
protein_coding	KCTD9	ENSG00000104756.15	44	69	1.6
lincRNA	LINC00294	ENSG00000280798.1	44	69	1.6
protein_coding	MOSMO	ENSG00000185716.11	37	58	1.6
protein_coding	ECD	ENSG00000122882.10	37	58	1.6
protein_coding	TERF2IP	ENSG00000166848.5	201	315	1.6
protein_coding	C15orf40	ENSG00000169609.13	30	47	1.6
protein_coding	DYNC2LI1	ENSG00000138036.18	30	47	1.6
protein_coding	CEP68	ENSG00000011523.13	83	130	1.6
protein_coding	ATRX	ENSG00000085224.22	843	1320	1.6
protein_coding	NECTIN2	ENSG00000130202.9	76	119	1.6
protein_coding	TUT7	ENSG00000083223.17	69	108	1.6
protein_coding	CENPX	ENSG00000169689.14	23	36	1.6
protein_coding	YTHDC2	ENSG00000047188.15	23	36	1.6
protein_coding	CPNE2	ENSG00000140848.16	23	36	1.6
protein_coding	TUBGCP3	ENSG00000126216.14	23	36	1.6
protein_coding	ABCE1	ENSG00000164163.10	39	61	1.6
protein_coding	MFSD11	ENSG00000092931.11	39	61	1.6
protein_coding	NUCB2	ENSG00000070081.16	415	649	1.6
protein_coding	DDR1	ENSG00000204580.13	94	147	1.6
protein_coding	PDE8A	ENSG00000073417.14	71	111	1.6
protein_coding	SMARCC1	ENSG00000173473.10	135	211	1.6
protein_coding	TNFRSF10D	ENSG00000173530.5	279	436	1.6
protein_coding	SMC5	ENSG00000198887.8	64	100	1.6
protein_coding	RNF34	ENSG00000170633.16	32	50	1.6
protein_coding	NAT10	ENSG00000135372.8	32	50	1.6
protein_coding	TSKU	ENSG00000182704.7	32	50	1.6

protein_coding	SLC24A3	ENSG00000185052.11	32	50	1.6
protein_coding	SLC43A1	ENSG00000149150.8	16	25	1.6
protein_coding	C1orf198	ENSG00000119280.16	89	139	1.6
protein_coding	GLA	ENSG00000102393.10	57	89	1.6
protein_coding	SLC4A1AP	ENSG00000163798.13	66	103	1.6
protein_coding	LIMS1	ENSG00000169756.16	116	181	1.6
protein_coding	DLG5	ENSG00000151208.16	166	259	1.6
protein_coding	DACT3	ENSG00000197380.10	25	39	1.6
protein_coding	PRKDC	ENSG00000253729.7	84	131	1.6
protein_coding	CDV3	ENSG00000091527.15	374	583	1.6
protein_coding	LARP1B	ENSG00000138709.18	34	53	1.6
protein_coding	CSTF2T	ENSG00000177613.8	34	53	1.6
protein_coding	PEX5	ENSG00000139197.10	34	53	1.6
protein_coding	NFU1	ENSG00000169599.12	120	187	1.6
protein_coding	TP53INP2	ENSG00000078804.12	129	201	1.6
protein_coding	ZNF621	ENSG00000172888.11	43	67	1.6
protein_coding	GBP2	ENSG00000162645.12	113	176	1.6
protein_coding	MIDN	ENSG00000167470.12	113	176	1.6
protein_coding	FADD	ENSG00000168040.4	61	95	1.6
protein_coding	NCBP2-AS2	ENSG00000270170.1	61	95	1.6
protein_coding	MFSD14B	ENSG00000148110.15	88	137	1.6
protein_coding	ARFGAP3	ENSG00000242247.10	207	322	1.6
protein_coding	ATP11B	ENSG00000058063.15	54	84	1.6
transcribed_processed_pseudogene	ZNF37BP	ENSG00000234420.7	36	56	1.6
protein_coding	ANKRD9	ENSG00000156381.8	27	42	1.6
protein_coding	VRK2	ENSG00000028116.17	27	42	1.6
protein_coding	GIGYF1	ENSG00000146830.10	27	42	1.6
protein_coding	ZNF687	ENSG00000143373.17	18	28	1.6
protein_coding	ACVRL1	ENSG00000139567.12	18	28	1.6
protein_coding	MTRF1	ENSG00000120662.15	9	14	1.6
antisense	AC020916.1	ENSG00000267519.6	9	14	1.6
protein_coding	DISC1	ENSG00000162946.22	9	14	1.6
protein_coding	ZNF608	ENSG00000168916.15	9	14	1.6
protein_coding	DNALI1	ENSG00000163879.10	9	14	1.6
protein_coding	LARS2	ENSG00000011376.10	9	14	1.6
protein_coding	NAT1	ENSG00000171428.13	9	14	1.6
protein_coding	MTERF2	ENSG00000120832.9	9	14	1.6
protein_coding	SGIP1	ENSG00000118473.21	103	160	1.6
protein_coding	GTF2H5	ENSG00000272047.2	94	146	1.6
protein_coding	IFT74	ENSG00000096872.16	47	73	1.6
protein_coding	ZFP36L1	ENSG00000185650.9	2212	3433	1.6
protein_coding	FAM234A	ENSG00000167930.15	58	90	1.6

protein_coding	TTLL12	ENSG00000100304.12	29	45	1.6
protein_coding	MTERF4	ENSG00000122085.16	29	45	1.6
protein_coding	SIPA1L1	ENSG00000197555.9	167	259	1.6
protein_coding	HERC4	ENSG00000148634.15	178	276	1.6
protein_coding	DNAJC30	ENSG00000176410.7	20	31	1.6
protein_coding	FUOM	ENSG00000148803.11	20	31	1.6
protein_coding	ILKAP	ENSG00000132323.8	20	31	1.6
protein_coding	WNT2B	ENSG00000134245.17	151	234	1.5
protein_coding	AMOTL1	ENSG00000166025.17	82	127	1.5
protein_coding	GDI1	ENSG00000203879.11	73	113	1.5
protein_coding	KIAA0319L	ENSG00000142687.17	106	164	1.5
protein_coding	VAV2	ENSG00000160293.16	53	82	1.5
transcribed_unprocessed_pseudogene	AP000769.1	ENSG00000173727.12	75	116	1.5
protein_coding	ANKH	ENSG00000154122.13	141	218	1.5
protein_coding	TMF1	ENSG00000144747.16	163	252	1.5
protein_coding	GLIS2	ENSG00000126603.8	44	68	1.5
protein_coding	IFIT5	ENSG00000152778.8	33	51	1.5
protein_coding	SPACA6	ENSG00000182310.14	22	34	1.5
protein_coding	CARD16	ENSG00000204397.7	22	34	1.5
protein_coding	PPP1R3B	ENSG00000173281.4	11	17	1.5
protein_coding	KCTD21	ENSG00000188997.7	11	17	1.5
protein_coding	PRUNE2	ENSG00000106772.17	11	17	1.5
protein_coding	CNTROB	ENSG00000170037.13	11	17	1.5
protein_coding	IL27RA	ENSG00000104998.3	11	17	1.5
protein_coding	ALDH1B1	ENSG00000137124.7	11	17	1.5
protein_coding	ZNF441	ENSG00000197044.10	11	17	1.5
protein_coding	WDR78	ENSG00000152763.16	11	17	1.5
protein_coding	COL8A2	ENSG00000171812.12	11	17	1.5
protein_coding	FST	ENSG00000134363.11	1487	2298	1.5
protein_coding	PNISR	ENSG00000132424.15	323	499	1.5
protein_coding	IDH1	ENSG00000138413.13	213	329	1.5
protein_coding	MIER1	ENSG00000198160.14	101	156	1.5
protein_coding	CEBPZ	ENSG00000115816.14	79	122	1.5
protein_coding	CDC42BPA	ENSG00000143776.18	125	193	1.5
protein_coding	LYPLA1	ENSG00000120992.17	57	88	1.5
protein_coding	NAXD	ENSG00000213995.11	46	71	1.5
protein_coding	KRAS	ENSG00000133703.11	116	179	1.5
protein_coding	TAOK1	ENSG00000160551.11	361	557	1.5
protein_coding	GRB2	ENSG00000177885.14	70	108	1.5
protein_coding	DNM1L	ENSG00000087470.17	70	108	1.5
protein_coding	GRIPAP1	ENSG00000068400.13	35	54	1.5
protein_coding	IMPA1	ENSG00000133731.9	35	54	1.5

protein_coding	CRIPT	ENSG00000119878.5	59	91	1.5
protein_coding	TTC14	ENSG00000163728.10	107	165	1.5
lincRNA	MIR4458HG	ENSG00000247516.6	48	74	1.5
protein_coding	CPPED1	ENSG00000103381.11	48	74	1.5
protein_coding	UBXN8	ENSG00000104691.14	24	37	1.5
protein_coding	TOM1L1	ENSG00000141198.15	24	37	1.5
protein_coding	ETF1	ENSG00000120705.12	170	262	1.5
protein_coding	FTO	ENSG00000140718.20	87	134	1.5
protein_coding	PPP2R2A	ENSG00000221914.9	76	117	1.5
protein_coding	PIK3CD	ENSG00000171608.15	26	40	1.5
protein_coding	SLC31A2	ENSG00000136867.10	26	40	1.5
protein_coding	FASTKD1	ENSG00000138399.17	13	20	1.5
protein_coding	EFNB3	ENSG00000108947.4	13	20	1.5
protein_coding	HIST1H4C	ENSG00000197061.4	13	20	1.5
protein_coding	MAST3	ENSG00000099308.10	13	20	1.5
lincRNA	AC102953.2	ENSG00000273230.1	13	20	1.5
protein_coding	HNRNPA1P4	ENSG00000224578.5	13	20	1.5
antisense	AC023043.1	ENSG00000260552.1	13	20	1.5
transcribed_unprocessed_pseudogene	STAG3L4	ENSG00000106610.15	13	20	1.5
protein_coding	SMC3	ENSG00000108055.9	147	226	1.5
protein_coding	TRIM38	ENSG00000112343.10	155	238	1.5
protein_coding	SIX2	ENSG00000170577.7	155	238	1.5
protein_coding	SGCB	ENSG00000163069.12	490	752	1.5
protein_coding	RPS19BP1	ENSG00000187051.8	58	89	1.5
protein_coding	DNAJB14	ENSG00000164031.16	103	158	1.5
protein_coding	NKTR	ENSG00000114857.17	120	184	1.5
protein_coding	RARS	ENSG00000113643.8	120	184	1.5
protein_coding	FAM131A	ENSG00000175182.14	30	46	1.5
protein_coding	CEP152	ENSG00000103995.13	15	23	1.5
protein_coding	UHMK1	ENSG00000152332.15	92	141	1.5
protein_coding	RPAP2	ENSG00000122484.8	109	167	1.5
protein_coding	MOSPD1	ENSG00000101928.12	47	72	1.5
protein_coding	NHS	ENSG00000188158.15	47	72	1.5
protein_coding	GTF2A1	ENSG00000165417.11	143	219	1.5
protein_coding	DNAJB2	ENSG00000135924.15	64	98	1.5
protein_coding	UCK2	ENSG00000143179.15	32	49	1.5
protein_coding	CYBC1	ENSG00000178927.17	32	49	1.5
protein_coding	ING3	ENSG00000071243.15	32	49	1.5
protein_coding	ICE2	ENSG00000128915.11	32	49	1.5
protein_coding	KIF13A	ENSG00000137177.19	177	271	1.5
protein_coding	CTNNB1	ENSG00000168036.17	415	635	1.5
protein_coding	MLXIP	ENSG00000175727.13	85	130	1.5

protein_coding	HGS	ENSG00000185359.12	85	130	1.5
protein_coding	CEP19	ENSG00000174007.7	17	26	1.5
protein_coding	ZBTB6	ENSG00000186130.4	17	26	1.5
protein_coding	TCEANC2	ENSG00000116205.13	17	26	1.5
protein_coding	SESN3	ENSG00000149212.11	89	136	1.5
protein_coding	ALG13	ENSG00000101901.11	36	55	1.5
protein_coding	SNRNP48	ENSG00000168566.12	36	55	1.5
protein_coding	EPB41L1	ENSG00000088367.21	112	171	1.5
protein_coding	OSBPL8	ENSG00000091039.16	376	574	1.5
protein_coding	ISCA1	ENSG00000135070.14	38	58	1.5
protein_coding	DNAJC9	ENSG00000213551.4	19	29	1.5
protein_coding	SPA17	ENSG00000064199.6	19	29	1.5
protein_coding	ARMCX3	ENSG00000102401.19	515	786	1.5
protein_coding	HMGB1	ENSG00000189403.14	177	270	1.5
protein_coding	CCDC88A	ENSG00000115355.16	147	224	1.5
protein_coding	RNF8	ENSG00000112130.16	42	64	1.5
antisense	PAXIP1-AS2	ENSG00000214106.8	21	32	1.5
protein_coding	SAP130	ENSG00000136715.18	21	32	1.5
protein_coding	ORC2	ENSG00000115942.8	21	32	1.5
protein_coding	ZNF629	ENSG00000102870.5	21	32	1.5
protein_coding	TMED8	ENSG00000100580.7	21	32	1.5
protein_coding	PSMD14	ENSG00000115233.11	65	99	1.5
protein_coding	CCDC127	ENSG00000164366.3	44	67	1.5
protein_coding	JUND	ENSG00000130522.5	67	102	1.5
protein_coding	CFAP97	ENSG00000164323.13	159	242	1.5
protein_coding	CTNNBL1	ENSG00000132792.18	46	70	1.5
protein_coding	CTIF	ENSG00000134030.13	46	70	1.5
protein_coding	C1D	ENSG00000197223.11	23	35	1.5
protein_coding	AQP3	ENSG00000165272.15	23	35	1.5
protein_coding	RIF1	ENSG00000080345.17	142	216	1.5
protein_coding	SSNA1	ENSG00000176101.11	48	73	1.5
protein_coding	NT5C2	ENSG00000076685.18	48	73	1.5
protein_coding	PLOD2	ENSG00000152952.11	642	976	1.5
protein_coding	GRK5	ENSG00000198873.11	25	38	1.5
protein_coding	NOTCH3	ENSG00000074181.8	129	196	1.5
protein_coding	SWAP70	ENSG00000133789.14	54	82	1.5
protein_coding	RTL5	ENSG00000242732.4	27	41	1.5
protein_coding	NDC1	ENSG00000058804.11	27	41	1.5
protein_coding	ZNF397	ENSG00000186812.12	27	41	1.5
protein_coding	MTDH	ENSG00000147649.9	841	1277	1.5
protein_coding	CCNL1	ENSG00000163660.11	56	85	1.5
protein_coding	NUMB	ENSG00000133961.20	114	173	1.5
protein_coding	RBM23	ENSG00000100461.17	58	88	1.5
protein_coding	CDKN2B	ENSG00000147883.10	29	44	1.5

antisense	AP001453.2	ENSG00000256940.1	29	44	1.5
protein_coding	IRGQ	ENSG00000167378.8	29	44	1.5
protein_coding	PRKCSH	ENSG00000130175.9	89	135	1.5
protein_coding	SIN3B	ENSG00000127511.9	60	91	1.5
protein_coding	UBE2Q1	ENSG00000160714.9	122	185	1.5
protein_coding	NDUFV1	ENSG00000167792.12	66	100	1.5
protein_coding	WBP4	ENSG00000120688.8	68	103	1.5
protein_coding	SMC6	ENSG00000163029.15	68	103	1.5
protein_coding	RAB5IF	ENSG00000101084.17	72	109	1.5
protein_coding	VAMP2	ENSG00000220205.8	37	56	1.5
protein_coding	AKAP11	ENSG00000023516.8	115	174	1.5
protein_coding	SLC35F6	ENSG00000213699.8	117	177	1.5
protein_coding	CCNH	ENSG00000134480.14	39	59	1.5
protein_coding	SCAF11	ENSG00000139218.17	279	422	1.5
protein_coding	RIN2	ENSG00000132669.13	121	183	1.5
protein_coding	RPAP3	ENSG00000005175.9	43	65	1.5
protein_coding	MRPS36	ENSG00000134056.11	90	136	1.5
protein_coding	DNAJB4	ENSG00000162616.8	45	68	1.5
protein_coding	ISG15	ENSG00000187608.9	45	68	1.5
protein_coding	YBX3	ENSG00000060138.12	722	1091	1.5
protein_coding	GTPBP4	ENSG00000107937.18	49	74	1.5
protein_coding	MFN1	ENSG00000171109.18	51	77	1.5
protein_coding	C2orf49	ENSG00000135974.9	53	80	1.5
protein_coding	TMEM165	ENSG00000134851.12	222	335	1.5
protein_coding	HERC2	ENSG00000128731.16	57	86	1.5
protein_coding	SUCLA2	ENSG00000136143.15	57	86	1.5
protein_coding	EHMT1	ENSG00000181090.20	61	92	1.5
protein_coding	SLC36A4	ENSG00000180773.14	69	104	1.5
protein_coding	NFATC2IP	ENSG00000176953.12	69	104	1.5
protein_coding	PPP2R5D	ENSG00000112640.14	73	110	1.5
protein_coding	EFS	ENSG00000100842.12	75	113	1.5
protein_coding	NUFIP2	ENSG00000108256.8	162	244	1.5
protein_coding	RSRP1	ENSG00000117616.17	129	194	1.5
protein_coding	BAZ1A	ENSG00000198604.10	145	218	1.5
antisense	FGD5-AS1	ENSG00000225733.5	124	186	1.5
protein_coding	CASK	ENSG00000147044.21	68	102	1.5
protein_coding	TSPAN14	ENSG00000108219.14	66	99	1.5
protein_coding	PREP	ENSG00000085377.13	50	75	1.5
protein_coding	LRIG1	ENSG00000144749.13	50	75	1.5
protein_coding	KBTBD2	ENSG00000170852.10	42	63	1.5
protein_coding	AIFM2	ENSG00000042286.14	40	60	1.5
protein_coding	KDM3A	ENSG00000115548.16	36	54	1.5
sense_intronic	BREA2	ENSG00000181097.5	34	51	1.5
protein_coding	TMED1	ENSG00000099203.6	34	51	1.5

protein_coding	PRPF3	ENSG00000117360.12	32	48	1.5
protein_coding	FAT4	ENSG00000196159.11	32	48	1.5
protein_coding	LGR4	ENSG00000205213.13	30	45	1.5
protein_coding	DNAJC11	ENSG00000007923.15	28	42	1.5
protein_coding	KMT5A	ENSG00000183955.12	26	39	1.5
protein_coding	PAR	ENSG00000169093.15	26	39	1.5
protein_coding	SLC17A9	ENSG00000101194.17	26	39	1.5
protein_coding	LRRC40	ENSG00000066557.5	26	39	1.5
protein_coding	HSPA12A	ENSG00000165868.14	22	33	1.5
protein_coding	NTNG2	ENSG00000196358.10	22	33	1.5
protein_coding	RNF4	ENSG00000063978.15	18	27	1.5
protein_coding	PTPRU	ENSG00000060656.19	18	27	1.5
protein_coding	ARRB2	ENSG00000141480.17	18	27	1.5
protein_coding	PHETA1	ENSG00000198324.13	18	27	1.5
protein_coding	PGM2	ENSG00000169299.13	18	27	1.5
protein_coding	PTER	ENSG00000165983.14	16	24	1.5
protein_coding	GSPT2	ENSG00000189369.8	16	24	1.5
protein_coding	NOC3L	ENSG00000173145.11	14	21	1.5
protein_coding	ARNTL2	ENSG00000029153.14	14	21	1.5
protein_coding	TCIRG1	ENSG00000110719.9	14	21	1.5
protein_coding	ZNF646	ENSG00000167395.10	14	21	1.5
protein_coding	METRN	ENSG00000103260.8	14	21	1.5
protein_coding	E2F6	ENSG00000169016.16	14	21	1.5
protein_coding	FAM135A	ENSG00000082269.16	12	18	1.5
protein_coding	RBM48	ENSG00000127993.15	12	18	1.5
protein_coding	CWF19L1	ENSG00000095485.17	12	18	1.5
protein_coding	GPR89A	ENSG00000117262.18	12	18	1.5
protein_coding	KDM7A	ENSG00000006459.10	10	15	1.5
protein_coding	B9D2	ENSG00000123810.7	10	15	1.5
protein_coding	ZNF615	ENSG00000197619.13	10	15	1.5
protein_coding	CENPW	ENSG00000203760.8	10	15	1.5
transcribed_unprocessed_pseudogene	ZNF826P	ENSG00000231205.11	10	15	1.5
protein_coding	LIAS	ENSG00000121897.14	10	15	1.5
protein_coding	MPP7	ENSG00000150054.18	8	12	1.5
sense_overlapping	AL158212.3	ENSG00000260917.1	8	12	1.5
protein_coding	SWT1	ENSG00000116668.12	8	12	1.5
protein_coding	RAVER2	ENSG00000162437.14	8	12	1.5
protein_coding	ZNF700	ENSG00000196757.7	8	12	1.5
protein_coding	PIGL	ENSG00000108474.16	8	12	1.5
protein_coding	PDE7A	ENSG00000205268.10	8	12	1.5
protein_coding	ABTB1	ENSG00000114626.17	6	9	1.5
protein_coding	ZXDB	ENSG00000198455.4	6	9	1.5

transcribed_unprocessed_pseudogene	AC026412.1	ENSG00000188002.10	6	9	1.5
antisense	AP002807.1	ENSG00000255031.5	6	9	1.5
protein_coding	ZNF253	ENSG00000256771.3	6	9	1.5
protein_coding	ZNF773	ENSG00000152439.12	6	9	1.5
protein_coding	GSDMB	ENSG00000073605.18	6	9	1.5
protein_coding	SDHAF3	ENSG00000196636.7	6	9	1.5
protein_coding	ARL4A	ENSG00000122644.12	6	9	1.5
protein_coding	ZNF527	ENSG00000189164.14	6	9	1.5
protein_coding	FOXL1	ENSG00000176678.5	6	9	1.5
protein_coding	RRP9	ENSG00000114767.6	6	9	1.5
lincRNA	LINC00865	ENSG00000232229.5	6	9	1.5
protein_coding	TUBD1	ENSG00000108423.14	4	6	1.5
protein_coding	CSMD1	ENSG00000183117.18	4	6	1.5
protein_coding	ROM1	ENSG00000149489.8	4	6	1.5
lincRNA	PSMB8-AS1	ENSG00000204261.8	4	6	1.5
protein_coding	ATP6V1E2	ENSG00000250565.6	4	6	1.5
protein_coding	ZBTB47	ENSG00000114853.13	4	6	1.5
lincRNA	HCG17	ENSG00000270604.5	4	6	1.5
lincRNA	AP001505.1	ENSG00000276529.1	4	6	1.5
protein_coding	SOX13	ENSG00000143842.14	4	6	1.5
protein_coding	KIF27	ENSG00000165115.14	4	6	1.5
processed_pseudogene	AL031727.1	ENSG00000226396.1	4	6	1.5
antisense	AC093799.1	ENSG00000272950.1	4	6	1.5
protein_coding	ADAMTS13	ENSG00000160323.18	4	6	1.5
antisense	AC024933.1	ENSG00000272656.1	4	6	1.5
snRNA	RNU6-817P	ENSG00000212385.1	4	6	1.5
protein_coding	SLC29A4	ENSG00000164638.10	4	6	1.5
protein_coding	PJVK	ENSG00000204311.13	4	6	1.5
protein_coding	KLHDC9	ENSG00000162755.13	4	6	1.5
lincRNA	AL450270.1	ENSG00000272428.1	4	6	1.5
protein_coding	SLC46A2	ENSG00000119457.7	4	6	1.5
protein_coding	GPM6B	ENSG00000046653.14	2	3	1.5
protein_coding	ZNF738	ENSG00000172687.13	2	3	1.5
protein_coding	C3AR1	ENSG00000171860.4	2	3	1.5
protein_coding	NCR3LG1	ENSG00000188211.8	2	3	1.5
protein_coding	HID1	ENSG00000167861.15	2	3	1.5
sense_intronic	AL162424.1	ENSG00000269970.1	2	3	1.5
antisense	AC010327.3	ENSG00000267577.1	2	3	1.5
antisense	AC004854.2	ENSG00000272768.1	2	3	1.5
protein_coding	SUV39H2	ENSG00000152455.15	2	3	1.5
lincRNA	AL365356.5	ENSG00000242147.1	2	3	1.5
misc_RNA	RF00019	ENSG00000252759.1	2	3	1.5

protein_coding	MGAT5B	ENSG00000167889.12	2	3	1.5
protein_coding	MAP3K8	ENSG00000107968.9	2	3	1.5
processed_pseudogene	Z74021.1	ENSG00000232346.1	2	3	1.5
protein_coding	PCDHGA8	ENSG00000253767.2	2	3	1.5
protein_coding	TEDC1	ENSG00000185347.17	2	3	1.5
protein_coding	ZNF473	ENSG00000142528.15	2	3	1.5
protein_coding	LSR	ENSG00000105699.16	2	3	1.5
antisense	RNASEH1-	ENSG00000234171.2	2	3	1.5
protein_coding	FRS3	ENSG00000137218.10	2	3	1.5
protein_coding	MICAL3	ENSG00000243156.7	2	3	1.5
protein_coding	CCDC183	ENSG00000213213.13	2	3	1.5
lincRNA	LINC02104	ENSG00000271334.5	2	3	1.5
antisense	AC004241.1	ENSG00000257433.5	2	3	1.5
protein_coding	DCAF15	ENSG00000132017.10	2	3	1.5
antisense	NCK1-DT	ENSG00000239213.5	2	3	1.5
antisense	SCOC-AS1	ENSG00000196951.10	2	3	1.5
lincRNA	AC078846.1	ENSG00000273329.1	2	3	1.5
protein_coding	FOXP4	ENSG00000137166.14	2	3	1.5
processed_transcript	AL354740.1	ENSG00000225339.3	2	3	1.5
processed_transcript	RAD51-AS1	ENSG00000245849.7	2	3	1.5
protein_coding	C10orf143	ENSG00000237489.4	2	3	1.5
lincRNA	AC125807.2	ENSG00000250899.3	2	3	1.5
antisense	AC022148.1	ENSG00000180458.2	2	3	1.5
protein_coding	EFHD1	ENSG00000115468.11	2	3	1.5
antisense	AL031963.3	ENSG00000272277.1	2	3	1.5
protein_coding	NRG2	ENSG00000158458.19	2	3	1.5
antisense	AC004846.2	ENSG00000258944.1	2	3	1.5
transcribed_processed_pseudogene	RPS10P7	ENSG00000223396.4	2	3	1.5
protein_coding	PMS2	ENSG00000122512.15	2	3	1.5
protein_coding	INO80B	ENSG00000115274.14	2	3	1.5
protein_coding	FERMT1	ENSG00000101311.15	2	3	1.5
processed_pseudogene	RPL24P4	ENSG00000181524.6	2	3	1.5
processed_pseudogene	FNBP1P1	ENSG00000257800.1	2	3	1.5
protein_coding	ATXN7	ENSG00000285258.1	2	3	1.5
TEC	AC118344.2	ENSG00000279759.1	2	3	1.5
transcribed_processed_pseudogene	RPL37P6	ENSG00000241431.1	2	3	1.5
protein_coding	FMO4	ENSG00000076258.9	2	3	1.5
protein_coding	ZNF670	ENSG00000277462.1	2	3	1.5
antisense	AL357060.1	ENSG00000237499.6	2	3	1.5
transcribed_unprocessed_pseudogene	PKD1P6	ENSG00000250251.6	2	3	1.5

antisense	AC068768.1	ENSG00000235423.8	2	3	1.5
lincRNA	LINC00327	ENSG00000232977.6	2	3	1.5
protein_coding	FZD9	ENSG00000188763.4	2	3	1.5
protein_coding	RGS11	ENSG00000076344.15	2	3	1.5
TEC	AC099494.2	ENSG00000279415.1	2	3	1.5
unitary_pseudogene	AC112198.2	ENSG00000232517.2	2	3	1.5
protein_coding	SYNGR3	ENSG00000127561.14	2	3	1.5
lincRNA	AC046143.2	ENSG00000272707.1	2	3	1.5
processed_pseudogene	EIF2S2P4	ENSG00000128692.8	2	3	1.5
protein_coding	ZNF571	ENSG00000180479.13	2	3	1.5
protein_coding	CCNB2	ENSG00000157456.7	2	3	1.5
lincRNA	AC010894.2	ENSG00000226853.2	2	3	1.5
processed_pseudogene	RPL23AP74	ENSG00000227694.1	2	3	1.5
antisense	AC008622.2	ENSG00000275719.1	2	3	1.5
sense_intronic	AC005632.2	ENSG00000261596.2	2	3	1.5
protein_coding	GNAZ	ENSG00000128266.8	2	3	1.5
protein_coding	BCAM	ENSG00000187244.11	2	3	1.5
processed_transcript	LINC00174	ENSG00000179406.7	2	3	1.5
protein_coding	ZNF763	ENSG00000197054.11	2	3	1.5
antisense	AC243772.2	ENSG00000233030.2	2	3	1.5
protein_coding	AKAP3	ENSG00000111254.7	2	3	1.5
protein_coding	CHDH	ENSG00000016391.10	2	3	1.5
sense_overlapping	AC096921.2	ENSG00000261468.1	2	3	1.5
TEC	AL137784.3	ENSG00000279170.1	2	3	1.5
antisense	RARA-AS1	ENSG00000265666.1	2	3	1.5
lincRNA	LINC01655	ENSG00000227925.1	2	3	1.5
lincRNA	AL023806.2	ENSG00000270828.1	2	3	1.5
protein_coding	GSTM5	ENSG00000134201.11	2	3	1.5
protein_coding	CAVIN4	ENSG00000170681.6	2	3	1.5
antisense	AC027097.2	ENSG00000267787.6	2	3	1.5
antisense	AC023908.3	ENSG00000261136.1	2	3	1.5
protein_coding	IRF2BP1	ENSG00000170604.4	2	3	1.5
protein_coding	PHOSPHO2	ENSG00000144362.11	2	3	1.5
processed_pseudogene	MTND4P12	ENSG00000247627.2	2	3	1.5
lincRNA	AL118558.4	ENSG00000272444.1	2	3	1.5
antisense	AC007541.1	ENSG00000260329.1	2	3	1.5
protein_coding	IRX2	ENSG00000170561.12	2	3	1.5
processed_pseudogene	AC104763.1	ENSG00000241157.1	2	3	1.5
processed_pseudogene	AC007683.1	ENSG00000224415.1	2	3	1.5
lincRNA	AC064807.4	ENSG00000272024.1	2	3	1.5
protein_coding	STRADA	ENSG00000266173.6	2	3	1.5
protein_coding	PLBD1	ENSG00000121316.10	2	3	1.5
sense_intronic	AC113139.1	ENSG00000271555.1	2	3	1.5
lincRNA	LINC00944	ENSG00000256128.5	2	3	1.5

protein_coding	PDZD7	ENSG00000186862.18	2	3	1.5
lincRNA	AL357055.3	ENSG00000238198.1	2	3	1.5
lincRNA	LINC01703	ENSG00000225518.2	2	3	1.5
lincRNA	MIR3142HG	ENSG00000253522.6	2	3	1.5
protein_coding	COL9A2	ENSG00000049089.14	2	3	1.5
protein_coding	NRXN2	ENSG00000110076.18	2	3	1.5
protein_coding	GNB3	ENSG00000111664.10	2	3	1.5
protein_coding	RHBDL3	ENSG00000141314.12	2	3	1.5
processed_pseudogene	AC007688.1	ENSG00000241352.3	2	3	1.5
protein_coding	LENG9	ENSG00000275183.1	2	3	1.5
protein_coding	KLF6	ENSG00000067082.14	323	484	1.5
protein_coding	ARSB	ENSG00000113273.16	229	343	1.5
protein_coding	KDM2A	ENSG00000173120.14	145	217	1.5
protein_coding	ATP2B1	ENSG00000070961.15	268	401	1.5
protein_coding	TRIM8	ENSG00000171206.14	105	157	1.5
protein_coding	MAFK	ENSG00000198517.9	93	139	1.5
protein_coding	KLHL42	ENSG00000087448.10	91	136	1.5
protein_coding	PSMD12	ENSG00000197170.9	85	127	1.5
protein_coding	ERGIC2	ENSG00000087502.17	160	239	1.5
protein_coding	CERK	ENSG00000100422.13	79	118	1.5
protein_coding	LRRC15	ENSG00000172061.8	79	118	1.5
protein_coding	MEGF9	ENSG00000106780.8	77	115	1.5
protein_coding	SGPP1	ENSG00000126821.7	75	112	1.5
protein_coding	IPO8	ENSG00000133704.9	75	112	1.5
protein_coding	MGAT1	ENSG00000131446.16	215	321	1.5
protein_coding	ZEB1	ENSG00000148516.21	140	209	1.5
protein_coding	SLC26A2	ENSG00000155850.7	55	82	1.5
protein_coding	SRRM1	ENSG00000133226.16	161	240	1.5
protein_coding	TRPT1	ENSG00000149743.13	51	76	1.5
protein_coding	MPST	ENSG00000128309.16	100	149	1.5
protein_coding	MYO1B	ENSG00000128641.18	317	472	1.5
protein_coding	MTF1	ENSG00000188786.9	45	67	1.5
protein_coding	PIK3IP1	ENSG00000100100.12	219	326	1.5
protein_coding	PALLD	ENSG00000129116.18	477	710	1.5
protein_coding	GPN3	ENSG00000111231.8	41	61	1.5
protein_coding	BAG2	ENSG00000112208.11	121	180	1.5
protein_coding	CASP7	ENSG00000165806.19	39	58	1.5
protein_coding	RUNX1	ENSG00000159216.18	37	55	1.5
protein_coding	TMEM246	ENSG00000165152.8	37	55	1.5
protein_coding	SHOC2	ENSG00000108061.11	107	159	1.5
protein_coding	PRKRIP1	ENSG00000128563.13	35	52	1.5
protein_coding	HNRNPR	ENSG00000125944.19	138	205	1.5
protein_coding	GLIPR1	ENSG00000139278.9	377	560	1.5
protein_coding	SP3	ENSG00000172845.14	99	147	1.5

protein_coding	ZNF281	ENSG00000162702.7	99	147	1.5
protein_coding	SMARCAL1	ENSG00000138375.12	33	49	1.5
protein_coding	RNMT	ENSG00000101654.17	64	95	1.5
protein_coding	AHRR	ENSG00000063438.16	60	89	1.5
protein_coding	ARGLU1	ENSG00000134884.14	118	175	1.5
protein_coding	GABPA	ENSG00000154727.10	58	86	1.5
protein_coding	LRRK2	ENSG00000188906.15	29	43	1.5
protein_coding	ANGEL2	ENSG00000174606.13	29	43	1.5
protein_coding	NAV1	ENSG00000134369.15	166	246	1.5
protein_coding	SLC2A3	ENSG00000059804.15	81	120	1.5
protein_coding	PABPN1	ENSG00000100836.10	27	40	1.5
protein_coding	BORCS6	ENSG00000196544.7	27	40	1.5
protein_coding	AAMDC	ENSG00000087884.14	27	40	1.5
protein_coding	BOD1L1	ENSG00000038219.12	347	514	1.5
protein_coding	NUDT15	ENSG00000136159.3	25	37	1.5
protein_coding	NBEA	ENSG00000172915.18	25	37	1.5
protein_coding	CEP162	ENSG00000135315.11	25	37	1.5
protein_coding	TMEM185B	ENSG00000226479.3	25	37	1.5
protein_coding	KMT2C	ENSG00000055609.17	146	216	1.5
protein_coding	TPM3	ENSG00000143549.19	169	250	1.5
protein_coding	NPC1	ENSG00000141458.12	48	71	1.5
protein_coding	CPLANE1	ENSG00000197603.13	48	71	1.5
protein_coding	C12orf57	ENSG00000111678.10	142	210	1.5
protein_coding	MT-ND4L	ENSG00000212907.2	378	559	1.5
protein_coding	POR	ENSG00000127948.15	111	164	1.5
protein_coding	ASB1	ENSG00000065802.11	44	65	1.5
protein_coding	TPRKB	ENSG00000144034.14	44	65	1.5
protein_coding	U2SURP	ENSG00000163714.17	107	158	1.5
protein_coding	NUAK1	ENSG00000074590.13	21	31	1.5
protein_coding	TMEM222	ENSG00000186501.14	21	31	1.5
protein_coding	PARG	ENSG00000227345.8	21	31	1.5
protein_coding	XYLT1	ENSG00000103489.11	78	115	1.5
protein_coding	SOS¬†1	ENSG00000115904.12	173	255	1.5
protein_coding	TBC1D1	ENSG00000065882.15	57	84	1.5
protein_coding	ZNF226	ENSG00000167380.16	57	84	1.5
protein_coding	CASP8AP2	ENSG00000118412.12	38	56	1.5
protein_coding	CEP164	ENSG00000110274.15	38	56	1.5
protein_coding	SLC30A4	ENSG00000104154.6	19	28	1.5
protein_coding	BPTF	ENSG00000171634.17	338	498	1.5
protein_coding	TNPO1	ENSG00000083312.17	487	717	1.5
protein_coding	KIF1B	ENSG00000054523.17	108	159	1.5
protein_coding	MSL3	ENSG00000005302.18	36	53	1.5
protein_coding	ZBTB33	ENSG00000177485.6	36	53	1.5
protein_coding	ZCCHC17	ENSG00000121766.15	89	131	1.5

protein_coding	DPY19L1	ENSG00000173852.14	155	228	1.5
protein_coding	GADD45GIP	ENSG00000179271.2	102	150	1.5
protein_coding	USF2	ENSG00000105698.15	51	75	1.5
protein_coding	COA5	ENSG00000183513.8	34	50	1.5
protein_coding	TCP11L1	ENSG00000176148.15	17	25	1.5
protein_coding	SH3BGRL2	ENSG00000198478.7	17	25	1.5
protein_coding	COLEC12	ENSG00000158270.11	83	122	1.5
protein_coding	FOXK1	ENSG00000164916.10	98	144	1.5
protein_coding	USP47	ENSG00000170242.17	139	204	1.5
protein_coding	USP16	ENSG00000156256.14	152	223	1.5
protein_coding	SAC3D1	ENSG00000168061.14	30	44	1.5
protein_coding	EHD4	ENSG00000103966.10	15	22	1.5
protein_coding	ABHD6	ENSG00000163686.14	15	22	1.5
protein_coding	POLD3	ENSG00000077514.8	15	22	1.5
protein_coding	APEX2	ENSG00000169188.4	15	22	1.5
protein_coding	EIF3A	ENSG00000107581.12	663	972	1.5
protein_coding	TIMP3	ENSG00000100234.11	481	705	1.5
processed_pseudogene	AC024293.1	ENSG00000244313.3	28	41	1.5
protein_coding	TBC1D13	ENSG00000107021.15	28	41	1.5
protein_coding	M6PR	ENSG00000003056.7	95	139	1.5
protein_coding	GNL2	ENSG00000134697.12	149	218	1.5
protein_coding	BLOC1S6	ENSG00000104164.10	162	237	1.5
protein_coding	ZNF292	ENSG00000188994.12	108	158	1.5
protein_coding	FUCA2	ENSG00000001036.13	121	177	1.5
protein_coding	ZMAT2	ENSG00000146007.10	188	275	1.5
protein_coding	FEM1B	ENSG00000169018.5	104	152	1.5
protein_coding	PCF11	ENSG00000165494.11	78	114	1.5
protein_coding	TSPAN17	ENSG00000048140.17	52	76	1.5
protein_coding	ZBTB40	ENSG00000184677.17	39	57	1.5
protein_coding	HIVEP1	ENSG00000095951.16	26	38	1.5
protein_coding	SIRT6	ENSG00000077463.14	13	19	1.5
protein_coding	PDGFA	ENSG00000197461.13	13	19	1.5
protein_coding	DNAJC22	ENSG00000178401.15	13	19	1.5
antisense	AC007388.1	ENSG00000231312.6	13	19	1.5
lincRNA	AC006504.5	ENSG00000267575.6	13	19	1.5
protein_coding	NACC1	ENSG00000160877.5	37	54	1.5
protein_coding	CREB3L2	ENSG00000182158.14	207	302	1.5
protein_coding	EIF4G1	ENSG00000114867.20	288	420	1.5
protein_coding	SENP2	ENSG00000163904.12	48	70	1.5
protein_coding	BAHCC1	ENSG00000266074.8	24	35	1.5
protein_coding	RAPGEF2	ENSG00000109756.9	59	86	1.5
protein_coding	SUPT6H	ENSG00000109111.14	59	86	1.5
protein_coding	RPF2	ENSG00000197498.12	59	86	1.5
protein_coding	POLR2M	ENSG00000255529.8	105	153	1.5

protein_coding	MPHOSPH6	ENSG00000135698.9	70	102	1.5
protein_coding	CACNA1A	ENSG00000141837.19	35	51	1.5
protein_coding	ACOX3	ENSG00000087008.15	35	51	1.5
protein_coding	SGPL1	ENSG00000166224.16	35	51	1.5
protein_coding	NKAP	ENSG00000101882.9	92	134	1.5
protein_coding	FAM214A	ENSG00000047346.12	92	134	1.5
protein_coding	ECHDC1	ENSG00000093144.18	125	182	1.5
protein_coding	TCF4	ENSG00000196628.16	430	626	1.5
protein_coding	C6orf106	ENSG00000196821.9	158	230	1.5
protein_coding	INAFM1	ENSG00000257704.3	33	48	1.5
protein_coding	ABTB2	ENSG00000166016.5	22	32	1.5
protein_coding	IFT27	ENSG00000100360.14	22	32	1.5
protein_coding	SLC22A23	ENSG00000137266.14	22	32	1.5
protein_coding	ZNF544	ENSG00000198131.13	11	16	1.5
protein_coding	NUDT14	ENSG00000183828.14	11	16	1.5
protein_coding	WFDC1	ENSG00000103175.10	11	16	1.5
sense_overlapping	AC007406.5	ENSG00000261799.1	11	16	1.5
protein_coding	GSAP	ENSG00000186088.15	11	16	1.5
protein_coding	SDF2	ENSG00000132581.9	139	202	1.5
unprocessed_pseudogene	FRG1CP	ENSG00000282826.1	64	93	1.5
protein_coding	TRIP11	ENSG00000100815.12	245	356	1.5
protein_coding	LDHA	ENSG00000134333.13	1342	1950	1.5
protein_coding	NKAPD1	ENSG00000150776.17	106	154	1.5
protein_coding	UPF3A	ENSG00000169062.14	53	77	1.5
protein_coding	TST	ENSG00000128311.13	53	77	1.5
protein_coding	USP33	ENSG00000077254.14	126	183	1.5
protein_coding	NKIRAS2	ENSG00000168256.17	42	61	1.5
protein_coding	MYO1E	ENSG00000157483.8	31	45	1.5
protein_coding	STX10	ENSG00000104915.14	31	45	1.5
protein_coding	IRF2BPL	ENSG00000119669.4	237	344	1.5
protein_coding	TRA2A	ENSG00000164548.10	144	209	1.5
protein_coding	ZNF639	ENSG00000121864.9	51	74	1.5
protein_coding	PURA	ENSG00000185129.5	342	496	1.5
protein_coding	MTR	ENSG00000116984.12	100	145	1.5
protein_coding	SBNO2	ENSG00000064932.15	40	58	1.5
protein_coding	ANKRD49	ENSG00000168876.8	20	29	1.5
protein_coding	GDAP2	ENSG00000196505.10	20	29	1.5
protein_coding	TARBP2	ENSG00000139546.10	20	29	1.5
protein_coding	ZBED3	ENSG00000132846.5	20	29	1.5
protein_coding	SRRT	ENSG00000087087.19	49	71	1.4
protein_coding	TBL1X	ENSG00000101849.16	87	126	1.4
protein_coding	MCRIP1	ENSG00000225663.7	87	126	1.4
protein_coding	CSTF1	ENSG00000101138.11	29	42	1.4
protein_coding	XPNPEP3	ENSG00000196236.12	29	42	1.4

protein_coding	PRKAB1	ENSG00000111725.10	29	42	1.4
protein_coding	IFNAR2	ENSG00000159110.19	29	42	1.4
protein_coding	LRRC59	ENSG00000108829.9	183	265	1.4
protein_coding	PSMD7	ENSG00000103035.10	172	249	1.4
protein_coding	RABGAP1	ENSG00000011454.16	105	152	1.4
protein_coding	RAD21	ENSG00000164754.14	219	317	1.4
protein_coding	SRPRB	ENSG00000144867.11	114	165	1.4
protein_coding	SNF8	ENSG00000159210.9	76	110	1.4
protein_coding	TSPYL2	ENSG00000184205.14	38	55	1.4
protein_coding	MACF1	ENSG00000127603.25	389	563	1.4
protein_coding	LEMD2	ENSG00000161904.11	47	68	1.4
protein_coding	PEX13	ENSG00000162928.8	47	68	1.4
antisense	MSC-AS1	ENSG00000235531.9	47	68	1.4
protein_coding	TBC1D15	ENSG00000121749.15	65	94	1.4
protein_coding	SLC33A1	ENSG00000169359.14	65	94	1.4
protein_coding	FAM204A	ENSG00000165669.13	92	133	1.4
protein_coding	USP15	ENSG00000135655.15	72	104	1.4
protein_coding	MEIS3	ENSG00000105419.17	72	104	1.4
protein_coding	WDR13	ENSG00000101940.17	54	78	1.4
protein_coding	PPP4C	ENSG00000149923.13	54	78	1.4
protein_coding	SOCS7	ENSG00000274211.4	36	52	1.4
protein_coding	MAST2	ENSG00000086015.20	36	52	1.4
protein_coding	METTL13	ENSG00000010165.19	27	39	1.4
protein_coding	SGO2	ENSG00000163535.17	18	26	1.4
protein_coding	SLC35F2	ENSG00000110660.14	18	26	1.4
protein_coding	TNFSF10	ENSG00000121858.10	18	26	1.4
protein_coding	FAM207A	ENSG00000160256.12	9	13	1.4
protein_coding	HECTD2	ENSG00000165338.16	9	13	1.4
protein_coding	IQCB1	ENSG00000173226.16	9	13	1.4
protein_coding	INO80C	ENSG00000153391.15	9	13	1.4
protein_coding	PELI1	ENSG00000197329.11	9	13	1.4
protein_coding	PHKA2	ENSG00000044446.11	9	13	1.4
protein_coding	RRP8	ENSG00000132275.10	9	13	1.4
protein_coding	BTBD8	ENSG00000189195.12	9	13	1.4
antisense	AC012467.2	ENSG00000271976.1	9	13	1.4
protein_coding	CADPS2	ENSG00000081803.15	9	13	1.4
protein_coding	APOBEC3F	ENSG00000128394.16	9	13	1.4
protein_coding	ZNF76	ENSG00000065029.14	9	13	1.4
protein_coding	CSNK1E	ENSG00000213923.11	169	244	1.4
protein_coding	COX17	ENSG00000138495.6	133	192	1.4
protein_coding	CA5B	ENSG00000169239.12	79	114	1.4
Mt_tRNA	MT-TF	ENSG00000210049.1	79	114	1.4
protein_coding	JMY	ENSG00000152409.8	70	101	1.4
protein_coding	METRNL	ENSG00000176845.12	542	782	1.4

protein_coding	EDEM2	ENSG00000088298.12	61	88	1.4
protein_coding	MAN2B2	ENSG00000013288.8	156	225	1.4
protein_coding	EID2	ENSG00000176396.10	52	75	1.4
protein_coding	CCDC82	ENSG00000149231.13	43	62	1.4
protein_coding	NAGPA	ENSG00000103174.12	43	62	1.4
protein_coding	SMURF1	ENSG00000198742.9	34	49	1.4
protein_coding	HUWE1	ENSG00000086758.15	161	232	1.4
protein_coding	CACUL1	ENSG00000151893.14	152	219	1.4
processed_pseudogene	AC090498.1	ENSG00000279483.2	25	36	1.4
protein_coding	GPATCH2	ENSG00000092978.10	25	36	1.4
protein_coding	SVIL	ENSG00000197321.14	323	465	1.4
protein_coding	SRSF11	ENSG00000116754.13	469	675	1.4
protein_coding	MPHOSPH10	ENSG00000124383.8	82	118	1.4
protein_coding	FBXL12	ENSG00000127452.8	41	59	1.4
protein_coding	PPP2R5A	ENSG00000066027.11	98	141	1.4
protein_coding	DNAJC3	ENSG00000102580.14	219	315	1.4
protein_coding	UBE2B	ENSG00000119048.7	368	529	1.4
protein_coding	DCBLD1	ENSG00000164465.18	32	46	1.4
protein_coding	SCLT1	ENSG00000151466.11	16	23	1.4
protein_coding	MPHOSPH8	ENSG00000196199.13	215	309	1.4
protein_coding	SH3KBP1	ENSG00000147010.17	426	612	1.4
protein_coding	NASP	ENSG00000132780.16	71	102	1.4
protein_coding	TAB2	ENSG00000055208.18	149	214	1.4
protein_coding	TEAD1	ENSG00000187079.16	188	270	1.4
protein_coding	ALG12	ENSG00000182858.13	39	56	1.4
protein_coding	RBSN	ENSG00000131381.12	39	56	1.4
protein_coding	LAMB1	ENSG00000091136.13	753	1081	1.4
protein_coding	NSRP1	ENSG00000126653.17	186	267	1.4
protein_coding	TMX4	ENSG00000125827.8	138	198	1.4
protein_coding	CCSAP	ENSG00000154429.10	23	33	1.4
protein_coding	DPM2	ENSG00000136908.17	23	33	1.4
protein_coding	SETDB2	ENSG00000136169.16	23	33	1.4
protein_coding	ADAMTS5	ENSG00000154736.5	74	106	1.4
protein_coding	BNIP3	ENSG00000176171.11	169	242	1.4
protein_coding	IFT43	ENSG00000119650.12	44	63	1.4
protein_coding	SMIM13	ENSG00000224531.5	44	63	1.4
protein_coding	RBM33	ENSG00000184863.10	65	93	1.4
protein_coding	GATC	ENSG00000257218.5	65	93	1.4
protein_coding	P4HA1	ENSG00000122884.12	281	402	1.4
protein_coding	LTBP1	ENSG00000049323.15	316	452	1.4
protein_coding	PRDX2	ENSG00000167815.11	79	113	1.4
protein_coding	BET1	ENSG00000105829.12	86	123	1.4
protein_coding	MAP3K20	ENSG00000091436.16	472	675	1.4
protein_coding	KDM5B	ENSG00000117139.17	100	143	1.4

protein_coding	GPR107	ENSG00000148358.19	114	163	1.4
protein_coding	ASH1L	ENSG00000116539.12	140	200	1.4
protein_coding	PLD3	ENSG00000105223.19	77	110	1.4
protein_coding	INTS6	ENSG00000102786.14	49	70	1.4
protein_coding	RAB27A	ENSG00000069974.15	49	70	1.4
protein_coding	SNX33	ENSG00000173548.8	42	60	1.4
protein_coding	SPAG16	ENSG00000144451.18	42	60	1.4
protein_coding	FAM173A	ENSG00000103254.9	35	50	1.4
protein_coding	CEP120	ENSG00000168944.15	35	50	1.4
protein_coding	FAM114A2	ENSG00000055147.18	35	50	1.4
protein_coding	ME2	ENSG00000082212.12	28	40	1.4
protein_coding	INVS	ENSG00000119509.12	14	20	1.4
protein_coding	MED22	ENSG00000148297.15	14	20	1.4
protein_coding	HSPB2	ENSG00000170276.5	14	20	1.4
protein_coding	ZNF772	ENSG00000197128.11	14	20	1.4
protein_coding	ELFN1	ENSG00000225968.7	14	20	1.4
lincRNA	AC025181.2	ENSG00000272086.1	14	20	1.4
TEC	AC243964.3	ENSG00000279095.1	7	10	1.4
protein_coding	ZFP28	ENSG00000196867.7	7	10	1.4
lincRNA	AC009812.4	ENSG00000260317.1	7	10	1.4
protein_coding	ZNF322	ENSG00000181315.10	7	10	1.4
protein_coding	MMS22L	ENSG00000146263.11	7	10	1.4
protein_coding	INTS2	ENSG00000108506.12	7	10	1.4
antisense	PITPNA-AS1	ENSG00000236618.2	7	10	1.4
protein_coding	ZNF600	ENSG00000189190.10	7	10	1.4
protein_coding	APOL3	ENSG00000128284.19	7	10	1.4
protein_coding	ANKEF1	ENSG00000132623.15	7	10	1.4
protein_coding	ANKHD1	ENSG00000131503.20	7	10	1.4
protein_coding	CEP76	ENSG00000101624.10	7	10	1.4
protein_coding	LRP12	ENSG00000147650.11	159	227	1.4
protein_coding	AFMID	ENSG00000183077.15	61	87	1.4
protein_coding	TCEAL9	ENSG00000185222.9	1029	1467	1.4
protein_coding	TAF13	ENSG00000197780.9	134	191	1.4
protein_coding	PHF3	ENSG00000118482.11	207	295	1.4
protein_coding	ADAMTS7	ENSG00000136378.14	73	104	1.4
protein_coding	GMCL1	ENSG00000087338.4	33	47	1.4
protein_coding	TXLNG	ENSG00000086712.12	33	47	1.4
protein_coding	CDK11B	ENSG00000248333.8	33	47	1.4
protein_coding	FAM120B	ENSG00000112584.13	52	74	1.4
protein_coding	ZNF512B	ENSG00000196700.8	26	37	1.4
protein_coding	ANKRD28	ENSG00000206560.11	201	286	1.4
protein_coding	KLHL24	ENSG00000114796.15	149	212	1.4
antisense	WAC-AS1	ENSG00000254635.5	45	64	1.4
protein_coding	UBE2Q2	ENSG00000140367.11	166	236	1.4

protein_coding	TMED5	ENSG00000117500.12	171	243	1.4
protein_coding	EIF2S1	ENSG00000134001.12	95	135	1.4
protein_coding	SPRY1	ENSG00000164056.10	38	54	1.4
protein_coding	ARMCX4	ENSG00000196440.11	19	27	1.4
protein_coding	CHCHD4	ENSG00000163528.12	19	27	1.4
protein_coding	CLIC1	ENSG00000213719.8	19	27	1.4
protein_coding	KIAA0586	ENSG00000100578.14	19	27	1.4
protein_coding	ME1	ENSG00000065833.8	221	314	1.4
protein_coding	SH3PXD2B	ENSG00000174705.12	202	287	1.4
protein_coding	PICALM	ENSG00000073921.17	264	375	1.4
protein_coding	MED4	ENSG00000136146.14	88	125	1.4
protein_coding	SLC39A14	ENSG00000104635.13	157	223	1.4
protein_coding	KTN1	ENSG00000126777.17	1053	1495	1.4
protein_coding	ARMT1	ENSG00000146476.10	31	44	1.4
protein_coding	CEP41	ENSG00000106477.18	31	44	1.4
protein_coding	E2F4	ENSG00000205250.8	31	44	1.4
protein_coding	FCF1	ENSG00000119616.11	136	193	1.4
protein_coding	RNF126	ENSG00000070423.17	74	105	1.4
protein_coding	CEP290	ENSG00000198707.14	43	61	1.4
protein_coding	RNF14	ENSG00000013561.17	98	139	1.4
protein_coding	PDXDC1	ENSG00000179889.18	134	190	1.4
protein_coding	SLC3A2	ENSG00000168003.16	194	275	1.4
protein_coding	AKIRIN1	ENSG00000174574.15	103	146	1.4
protein_coding	PHF20L1	ENSG00000129292.20	218	309	1.4
protein_coding	MAN2A1	ENSG00000112893.9	278	394	1.4
protein_coding	LMO4	ENSG00000143013.12	96	136	1.4
protein_coding	MCM4	ENSG00000104738.17	48	68	1.4
protein_coding	STK38L	ENSG00000211455.7	48	68	1.4
protein_coding	GANC	ENSG00000214013.9	24	34	1.4
protein_coding	ASAP3	ENSG00000088280.18	24	34	1.4
protein_coding	EBP	ENSG00000147155.10	12	17	1.4
protein_coding	ALDH4A1	ENSG00000159423.16	12	17	1.4
protein_coding	ZNF175	ENSG00000105497.7	12	17	1.4
protein_coding	NFE2	ENSG00000123405.13	12	17	1.4
protein_coding	PDCD10	ENSG00000114209.14	77	109	1.4
protein_coding	CYGB	ENSG00000161544.9	53	75	1.4
protein_coding	PSEN1	ENSG00000080815.18	70	99	1.4
protein_coding	LEPROT	ENSG00000213625.8	505	714	1.4
protein_coding	JAK1	ENSG00000162434.11	319	451	1.4
protein_coding	C18orf25	ENSG00000152242.10	29	41	1.4
protein_coding	SPSB1	ENSG00000171621.13	29	41	1.4
protein_coding	ARID4A	ENSG00000032219.18	104	147	1.4
protein_coding	MAFG	ENSG00000197063.10	131	185	1.4
protein_coding	DTWD1	ENSG00000104047.14	131	185	1.4

protein_coding	LARP4	ENSG00000161813.21	51	72	1.4
protein_coding	SLC35A5	ENSG00000138459.8	51	72	1.4
protein_coding	FTSJ3	ENSG00000108592.16	34	48	1.4
protein_coding	MRPS31	ENSG00000102738.7	34	48	1.4
protein_coding	TRAF5	ENSG00000082512.14	17	24	1.4
protein_coding	ZSWIM8	ENSG00000214655.10	17	24	1.4
protein_coding	RFT1	ENSG00000163933.9	17	24	1.4
protein_coding	NCKIPSD	ENSG00000213672.7	17	24	1.4
protein_coding	ZNF407	ENSG00000215421.9	17	24	1.4
protein_coding	FAM118A	ENSG00000100376.11	17	24	1.4
protein_coding	SEMA3B	ENSG00000012171.19	17	24	1.4
protein_coding	SLC39A10	ENSG00000196950.13	197	278	1.4
protein_coding	DYRK1A	ENSG00000157540.20	73	103	1.4
protein_coding	G3BP1	ENSG00000145907.14	112	158	1.4
protein_coding	NUP153	ENSG00000124789.11	39	55	1.4
protein_coding	RNF103	ENSG00000239305.6	100	141	1.4
protein_coding	PSKH1	ENSG00000159792.9	61	86	1.4
protein_coding	KPNA3	ENSG00000102753.9	127	179	1.4
lincRNA	MIR22HG	ENSG00000186594.14	44	62	1.4
protein_coding	PHF10	ENSG00000130024.14	44	62	1.4
protein_coding	HACL1	ENSG00000131373.14	22	31	1.4
protein_coding	TTC7B	ENSG00000165914.14	22	31	1.4
protein_coding	ZNF512	ENSG00000243943.9	22	31	1.4
protein_coding	NR1D1	ENSG00000126368.5	22	31	1.4
protein_coding	SNX1	ENSG00000028528.14	269	379	1.4
protein_coding	OSBPL1A	ENSG00000141447.17	203	286	1.4
protein_coding	ZNF827	ENSG00000151612.15	71	100	1.4
protein_coding	SEMA5A	ENSG00000112902.11	622	876	1.4
protein_coding	EVI5	ENSG00000067208.14	120	169	1.4
protein_coding	TCEAL1	ENSG00000172465.13	49	69	1.4
protein_coding	RNF145	ENSG00000145860.11	184	259	1.4
protein_coding	CSRP1	ENSG00000159176.13	135	190	1.4
protein_coding	HAUS6	ENSG00000147874.10	54	76	1.4
protein_coding	BBIP1	ENSG00000214413.7	54	76	1.4
protein_coding	LDLRAD3	ENSG00000179241.12	27	38	1.4
protein_coding	ANKS6	ENSG00000165138.17	27	38	1.4
protein_coding	BORCS7	ENSG00000166275.15	113	159	1.4
lincRNA	NEAT1	ENSG00000245532.8	1683	2368	1.4
protein_coding	SNRNP70	ENSG00000104852.14	86	121	1.4
protein_coding	UNC119	ENSG00000109103.11	59	83	1.4
protein_coding	ZNF503	ENSG00000165655.16	246	346	1.4
protein_coding	TRPS1	ENSG00000104447.12	128	180	1.4
protein_coding	EFCAB14	ENSG00000159658.10	165	232	1.4
protein_coding	KIAA1109	ENSG00000138688.15	212	298	1.4

protein_coding	CCNK	ENSG00000090061.17	79	111	1.4
protein_coding	INA	ENSG00000148798.10	42	59	1.4
protein_coding	ITPR2	ENSG00000123104.11	42	59	1.4
protein_coding	ARL14EP	ENSG00000152219.4	42	59	1.4
protein_coding	RNASEH2C	ENSG00000172922.9	47	66	1.4
protein_coding	CCDC34	ENSG00000109881.16	47	66	1.4
protein_coding	ZNF268	ENSG00000090612.20	47	66	1.4
protein_coding	MAPK1IP1L	ENSG00000168175.14	203	285	1.4
protein_coding	EMP3	ENSG00000142227.10	208	292	1.4
protein_coding	SMARCA2	ENSG00000080503.23	161	226	1.4
protein_coding	SLC7A1	ENSG00000139514.12	124	174	1.4
protein_coding	RBM39	ENSG00000131051.22	283	397	1.4
protein_coding	MSN	ENSG00000147065.16	442	620	1.4
protein_coding	PGM3	ENSG00000013375.15	97	136	1.4
protein_coding	MAVS	ENSG00000088888.17	234	328	1.4
protein_coding	ABL1	ENSG00000097007.17	244	342	1.4
protein_coding	TMCO3	ENSG00000150403.17	175	245	1.4
protein_coding	GALNT1	ENSG00000141429.13	145	203	1.4
protein_coding	TIMM13	ENSG00000099800.7	105	147	1.4
protein_coding	ALG2	ENSG00000119523.9	100	140	1.4
protein_coding	VPS8	ENSG00000156931.15	60	84	1.4
protein_coding	MRPL36	ENSG00000171421.12	60	84	1.4
protein_coding	MRPL19	ENSG00000115364.13	50	70	1.4
protein_coding	POFUT1	ENSG00000101346.13	50	70	1.4
protein_coding	HACE1	ENSG00000085382.11	30	42	1.4
protein_coding	RIC8B	ENSG00000111785.19	20	28	1.4
protein_coding	LY6K	ENSG00000160886.13	20	28	1.4
protein_coding	MAGI3	ENSG00000081026.18	20	28	1.4
protein_coding	ZNF836	ENSG00000196267.12	20	28	1.4
protein_coding	ACAD8	ENSG00000151498.11	15	21	1.4
protein_coding	TTLL7	ENSG00000137941.16	15	21	1.4
protein_coding	LPCAT4	ENSG00000176454.13	15	21	1.4
protein_coding	TNFRSF10A	ENSG00000104689.9	15	21	1.4
protein_coding	NUP205	ENSG00000155561.14	15	21	1.4
protein_coding	RPAP1	ENSG00000103932.11	15	21	1.4
processed_transcript	SNHG1	ENSG00000255717.6	15	21	1.4
protein_coding	C12orf73	ENSG00000204954.9	15	21	1.4
protein_coding	CCDC167	ENSG00000198937.8	15	21	1.4
protein_coding	ZXDC	ENSG00000070476.14	15	21	1.4
antisense	AC009133.1	ENSG00000238045.9	15	21	1.4
protein_coding	ATF5	ENSG00000169136.10	10	14	1.4
protein_coding	FBXO46	ENSG00000177051.5	10	14	1.4
protein_coding	SLC4A8	ENSG00000050438.16	10	14	1.4
protein_coding	EFNA4	ENSG00000243364.7	10	14	1.4

protein_coding	LINS1	ENSG00000140471.16	10	14	1.4
protein_coding	ABHD8	ENSG00000127220.5	10	14	1.4
transcribed_processed_pseudogene	HTR7P1	ENSG00000183935.5	10	14	1.4
protein_coding	FASTKD3	ENSG00000124279.11	10	14	1.4
protein_coding	CUL9	ENSG00000112659.13	10	14	1.4
protein_coding	RTN4IP1	ENSG00000130347.12	10	14	1.4
processed_transcript	SNHG17	ENSG00000196756.11	10	14	1.4
protein_coding	ZKSCAN4	ENSG00000187626.8	10	14	1.4
protein_coding	HIST1H3H	ENSG00000278828.1	10	14	1.4
protein_coding	GUF1	ENSG00000151806.13	10	14	1.4
protein_coding	GAS2L3	ENSG00000139354.10	5	7	1.4
protein_coding	DOT1L	ENSG00000104885.17	5	7	1.4
protein_coding	TMEM177	ENSG00000144120.12	5	7	1.4
protein_coding	CYP46A1	ENSG00000036530.8	5	7	1.4
protein_coding	FSIP1	ENSG00000150667.7	5	7	1.4
antisense	AL359915.2	ENSG00000231365.5	5	7	1.4
antisense	LINC01481	ENSG00000257815.5	5	7	1.4
antisense	ZBTB11-AS1	ENSG00000256628.3	5	7	1.4
protein_coding	ICA1L	ENSG00000163596.16	5	7	1.4
sense_intronic	AL353150.1	ENSG00000224945.1	5	7	1.4
protein_coding	ZNF486	ENSG00000256229.7	5	7	1.4
protein_coding	HAPLN3	ENSG00000140511.11	5	7	1.4
protein_coding	PFDN6	ENSG00000204220.11	5	7	1.4
protein_coding	ZNF300	ENSG00000145908.12	5	7	1.4
protein_coding	E2F5	ENSG00000133740.10	5	7	1.4
protein_coding	HSD17B6	ENSG00000025423.11	5	7	1.4
protein_coding	ANKRD16	ENSG00000134461.15	5	7	1.4
protein_coding	CBX8	ENSG00000141570.10	5	7	1.4
protein_coding	ECHDC3	ENSG00000134463.14	5	7	1.4
protein_coding	CNNM3	ENSG00000168763.15	5	7	1.4
processed_pseudogene	AC007969.1	ENSG00000233762.3	5	7	1.4
TEC	AC000123.3	ENSG00000280347.1	5	7	1.4
protein_coding	MORN3	ENSG00000139714.12	5	7	1.4
protein_coding	MYH11	ENSG00000133392.17	5	7	1.4
protein_coding	LRRFIP1	ENSG00000124831.18	293	410	1.4
protein_coding	ZFC3H1	ENSG00000133858.15	163	228	1.4
unprocessed_pseudogene	MTATP6P1	ENSG00000248527.1	148	207	1.4
protein_coding	CDC5L	ENSG00000096401.7	138	193	1.4
protein_coding	CUX1	ENSG00000257923.10	241	337	1.4
protein_coding	NNMT	ENSG00000166741.7	118	165	1.4
protein_coding	PTN	ENSG00000105894.11	231	323	1.4
protein_coding	PCMTD1	ENSG00000168300.13	216	302	1.4

protein_coding	GLRX	ENSG00000173221.13	201	281	1.4
protein_coding	FKBP7	ENSG00000079150.17	78	109	1.4
protein_coding	RRN3	ENSG00000085721.12	63	88	1.4
protein_coding	SLC9A6	ENSG00000198689.11	63	88	1.4
protein_coding	RCAN1	ENSG00000159200.17	58	81	1.4
protein_coding	CUL5	ENSG00000166266.13	164	229	1.4
protein_coding	EML2	ENSG00000125746.16	53	74	1.4
protein_coding	NISCH	ENSG00000010322.15	48	67	1.4
protein_coding	SETD2	ENSG00000181555.20	91	127	1.4
protein_coding	NIN	ENSG00000100503.23	205	286	1.4
protein_coding	ZEB2	ENSG00000169554.19	152	212	1.4
protein_coding	ATP6AP1	ENSG00000071553.16	180	251	1.4
protein_coding	DAPK3	ENSG00000167657.13	71	99	1.4
protein_coding	DERL1	ENSG00000136986.9	170	237	1.4
protein_coding	NSD1	ENSG00000165671.20	66	92	1.4
protein_coding	ATP13A1	ENSG00000105726.16	33	46	1.4
protein_coding	KATNAL1	ENSG00000102781.13	28	39	1.4
protein_coding	ARL5A	ENSG00000162980.16	28	39	1.4
protein_coding	CCDC124	ENSG00000007080.10	28	39	1.4
protein_coding	RGS3	ENSG00000138835.22	102	142	1.4
protein_coding	CTR9	ENSG00000198730.8	102	142	1.4
protein_coding	TBC1D16	ENSG00000167291.15	51	71	1.4
protein_coding	HS6ST1	ENSG00000136720.6	97	135	1.4
protein_coding	CLN5	ENSG00000102805.14	92	128	1.4
protein_coding	ZNF511	ENSG00000198546.14	23	32	1.4
protein_coding	KDM6B	ENSG00000132510.10	23	32	1.4
protein_coding	PCYOX1	ENSG00000116005.11	410	570	1.4
protein_coding	ELF1	ENSG00000120690.15	82	114	1.4
protein_coding	ZBED1	ENSG00000214717.11	77	107	1.4
protein_coding	AHSA1	ENSG00000100591.7	95	132	1.4
protein_coding	COL18A1	ENSG00000182871.14	185	257	1.4
protein_coding	NIPBL	ENSG00000164190.17	239	332	1.4
protein_coding	UTP14C	ENSG00000253797.2	36	50	1.4
protein_coding	TXNDC9	ENSG00000115514.11	36	50	1.4
protein_coding	TBCK	ENSG00000145348.16	36	50	1.4
protein_coding	STAT5A	ENSG00000126561.16	18	25	1.4
protein_coding	AKT1S1	ENSG00000204673.10	18	25	1.4
protein_coding	FAM76B	ENSG00000077458.12	18	25	1.4
protein_coding	XRCC4	ENSG00000152422.15	18	25	1.4
protein_coding	LONRF1	ENSG00000154359.12	18	25	1.4
protein_coding	FAAP100	ENSG00000185504.16	18	25	1.4
protein_coding	CHFR	ENSG00000072609.17	18	25	1.4
antisense	ZBED5-AS1	ENSG00000247271.6	18	25	1.4
protein_coding	DDA1	ENSG00000130311.10	85	118	1.4

protein_coding	CCNDBP1	ENSG00000166946.13	98	136	1.4
protein_coding	PTS	ENSG00000150787.7	80	111	1.4
protein_coding	EEF1E1	ENSG00000124802.11	31	43	1.4
protein_coding	ZMYM5	ENSG00000132950.18	31	43	1.4
protein_coding	OTUB1	ENSG00000167770.11	44	61	1.4
protein_coding	VPS26A	ENSG00000122958.14	127	176	1.4
protein_coding	C1RL	ENSG00000139178.10	70	97	1.4
protein_coding	URI1	ENSG00000105176.17	223	309	1.4
protein_coding	PHIP	ENSG00000146247.13	187	259	1.4
protein_coding	DDX21	ENSG00000165732.12	182	252	1.4
protein_coding	NELFE	ENSG00000204356.13	65	90	1.4
protein_coding	MAPKAPK5	ENSG00000089022.13	52	72	1.4
protein_coding	RNF214	ENSG00000167257.10	39	54	1.4
protein_coding	MDN1	ENSG00000112159.11	39	54	1.4
protein_coding	TGFBRAP1	ENSG00000135966.12	39	54	1.4
protein_coding	CDKN2D	ENSG00000129355.6	26	36	1.4
protein_coding	NLK	ENSG00000087095.12	26	36	1.4
protein_coding	KDM6A	ENSG00000147050.14	13	18	1.4
protein_coding	ERI1	ENSG00000104626.14	13	18	1.4
protein_coding	LCMT2	ENSG00000168806.7	13	18	1.4
protein_coding	METTL22	ENSG00000067365.14	13	18	1.4
protein_coding	HKR1	ENSG00000181666.17	13	18	1.4
protein_coding	OPTN	ENSG00000123240.16	495	685	1.4
protein_coding	RABAC1	ENSG00000105404.10	232	321	1.4
protein_coding	ZC3H4	ENSG00000130749.9	60	83	1.4
protein_coding	MPPE1	ENSG00000154889.16	47	65	1.4
protein_coding	INPPL1	ENSG00000165458.13	162	224	1.4
protein_coding	RAPGEF1	ENSG00000107263.18	81	112	1.4
protein_coding	KIAA0232	ENSG00000170871.11	81	112	1.4
protein_coding	SMARCB1	ENSG00000099956.19	55	76	1.4
protein_coding	COL4A1	ENSG00000187498.15	524	724	1.4
protein_coding	FGFR1	ENSG00000077782.20	1028	1420	1.4
protein_coding	UNKL	ENSG00000059145.18	63	87	1.4
protein_coding	CYFIP2	ENSG00000055163.19	21	29	1.4
protein_coding	SCARB1	ENSG00000073060.15	21	29	1.4
protein_coding	CEP112	ENSG00000154240.16	21	29	1.4
protein_coding	JAK2	ENSG00000096968.13	21	29	1.4
protein_coding	CRY2	ENSG00000121671.11	21	29	1.4
protein_coding	PARP16	ENSG00000138617.15	21	29	1.4
protein_coding	DNAJC7	ENSG00000168259.14	50	69	1.4
protein_coding	UBA3	ENSG00000144744.16	79	109	1.4
protein_coding	EMC7	ENSG00000134153.9	290	400	1.4
protein_coding	TUT4	ENSG00000134744.13	58	80	1.4
protein_coding	PARVB	ENSG00000188677.14	29	40	1.4

protein_coding	GART	ENSG00000159131.16	29	40	1.4
protein_coding	TACC2	ENSG00000138162.18	29	40	1.4
protein_coding	STK32C	ENSG00000165752.16	37	51	1.4
protein_coding	VANGL1	ENSG00000173218.14	45	62	1.4
protein_coding	RAB11FIP2	ENSG00000107560.11	98	135	1.4
protein_coding	PTBP3	ENSG00000119314.15	106	146	1.4
protein_coding	CHCHD3	ENSG00000106554.12	53	73	1.4
protein_coding	AP3B1	ENSG00000132842.13	114	157	1.4
protein_coding	CTTNBP2NL	ENSG00000143079.14	122	168	1.4
protein_coding	VPS13A	ENSG00000197969.12	61	84	1.4
protein_coding	AZIN1	ENSG00000155096.13	313	431	1.4
protein_coding	PPP1R10	ENSG00000204569.9	77	106	1.4
protein_coding	SEC62	ENSG00000008952.16	964	1327	1.4
protein_coding	HS2ST1	ENSG00000153936.16	85	117	1.4
protein_coding	AUP1	ENSG00000115307.16	178	245	1.4
protein_coding	TIMM17A	ENSG00000134375.10	93	128	1.4
protein_coding	MT-ND2	ENSG00000198763.3	2674	3679	1.4
protein_coding	STK38	ENSG00000112079.8	165	227	1.4
protein_coding	MSMO1	ENSG00000052802.12	48	66	1.4
protein_coding	NUS1	ENSG00000153989.7	32	44	1.4
protein_coding	RACGAP1	ENSG00000161800.12	16	22	1.4
protein_coding	DAGLB	ENSG00000164535.14	16	22	1.4
protein_coding	MYL5	ENSG00000215375.6	16	22	1.4
antisense	NUTM2B-	ENSG00000225484.6	8	11	1.4
protein_coding	APBA1	ENSG00000107282.7	8	11	1.4
protein_coding	RGL3	ENSG00000205517.12	8	11	1.4
protein_coding	XRCC1	ENSG00000073050.11	8	11	1.4
protein_coding	C2orf42	ENSG00000115998.7	8	11	1.4
protein_coding	REL	ENSG00000162924.14	8	11	1.4
antisense	AC068888.1	ENSG00000257337.6	8	11	1.4
protein_coding	ZBTB37	ENSG00000185278.15	8	11	1.4
protein_coding	CCDC171	ENSG00000164989.16	8	11	1.4
protein_coding	RECQL5	ENSG00000108469.14	8	11	1.4
antisense	AL356599.1	ENSG00000235652.7	8	11	1.4
protein_coding	MMAB	ENSG00000139428.11	8	11	1.4
protein_coding	TTYH3	ENSG00000136295.14	265	364	1.4
protein_coding	PSMA4	ENSG00000041357.15	233	320	1.4
protein_coding	HADH	ENSG00000138796.16	75	103	1.4
protein_coding	ABRACL	ENSG00000146386.7	75	103	1.4
protein_coding	CLDND1	ENSG00000080822.16	59	81	1.4
protein_coding	OGFOD3	ENSG00000181396.12	59	81	1.4
protein_coding	SLC7A8	ENSG00000092068.19	110	151	1.4
protein_coding	ING2	ENSG00000168556.6	51	70	1.4
lincRNA	AC144831.1	ENSG00000261888.1	51	70	1.4

protein_coding	EMC8	ENSG00000131148.8	51	70	1.4
protein_coding	ZRANB2	ENSG00000132485.13	145	199	1.4
protein_coding	SURF4	ENSG00000148248.13	608	834	1.4
protein_coding	ARHGAP24	ENSG00000138639.17	35	48	1.4
protein_coding	TBC1D7	ENSG00000145979.17	35	48	1.4
protein_coding	HSPA5	ENSG00000044574.7	781	1071	1.4
protein_coding	HNRNPL	ENSG00000104824.17	159	218	1.4
protein_coding	SLC41A3	ENSG00000114544.16	62	85	1.4
protein_coding	MAF	ENSG00000178573.6	143	196	1.4
protein_coding	NUP50	ENSG00000093000.18	81	111	1.4
protein_coding	AHCTF1	ENSG00000153207.15	54	74	1.4
protein_coding	DIAPH1	ENSG00000131504.16	54	74	1.4
protein_coding	UTP6	ENSG00000108651.9	54	74	1.4
protein_coding	CD58	ENSG00000116815.15	27	37	1.4
protein_coding	HPS6	ENSG00000166189.7	27	37	1.4
protein_coding	SPAST	ENSG00000021574.12	27	37	1.4
protein_coding	TDRD7	ENSG00000196116.7	27	37	1.4
protein_coding	UBAP2L	ENSG00000143569.18	73	100	1.4
protein_coding	NCS1	ENSG00000107130.9	73	100	1.4
protein_coding	PNN	ENSG00000100941.8	46	63	1.4
protein_coding	AZI2	ENSG00000163512.13	249	341	1.4
protein_coding	LY6E	ENSG00000160932.10	176	241	1.4
protein_coding	COPB2	ENSG00000184432.9	306	419	1.4
protein_coding	SYVN1	ENSG00000162298.18	65	89	1.4
protein_coding	EHD1	ENSG00000110047.17	84	115	1.4
protein_coding	COMMD5	ENSG00000170619.9	38	52	1.4
protein_coding	SPHK2	ENSG00000063176.15	19	26	1.4
protein_coding	TBRG4	ENSG00000136270.13	19	26	1.4
protein_coding	CAPRIN2	ENSG00000110888.17	19	26	1.4
protein_coding	FAM53B	ENSG00000189319.13	19	26	1.4
protein_coding	MAK16	ENSG00000198042.10	19	26	1.4
protein_coding	AMMECR1L	ENSG00000144233.9	49	67	1.4
protein_coding	APBB1	ENSG00000166313.18	49	67	1.4
protein_coding	NFKBIA	ENSG00000100906.10	177	242	1.4
protein_coding	CMTR2	ENSG00000180917.17	30	41	1.4
protein_coding	SLC27A1	ENSG00000130304.16	30	41	1.4
protein_coding	TMED7	ENSG00000134970.13	227	310	1.4
protein_coding	PTP4A2	ENSG00000184007.20	717	979	1.4
protein_coding	COA4	ENSG00000181924.6	52	71	1.4
protein_coding	NDFIP2	ENSG00000102471.14	63	86	1.4
protein_coding	HSD17B11	ENSG00000198189.10	85	116	1.4
protein_coding	FYN	ENSG00000010810.17	96	131	1.4
protein_coding	C11orf58	ENSG00000110696.9	269	367	1.4
protein_coding	PCM1	ENSG00000078674.17	140	191	1.4

protein_coding	PSME4	ENSG00000068878.14	151	206	1.4
protein_coding	RAB21	ENSG00000080371.5	195	266	1.4
protein_coding	MYH10	ENSG00000133026.12	88	120	1.4
protein_coding	VBP1	ENSG00000155959.10	88	120	1.4
protein_coding	ERCC6L2	ENSG00000182150.15	55	75	1.4
protein_coding	OGFR	ENSG00000060491.16	33	45	1.4
protein_coding	SNIP1	ENSG00000163877.10	33	45	1.4
protein_coding	PNPO	ENSG00000108439.10	33	45	1.4
protein_coding	PLEKHH3	ENSG00000068137.14	22	30	1.4
protein_coding	FOXN2	ENSG00000170802.15	22	30	1.4
protein_coding	FOXRED2	ENSG00000100350.14	22	30	1.4
protein_coding	PTGES2	ENSG00000148334.14	22	30	1.4
sense_intronic	AC092718.4	ENSG00000261061.1	11	15	1.4
protein_coding	METTL21A	ENSG00000144401.14	11	15	1.4
protein_coding	GTPBP10	ENSG00000105793.15	11	15	1.4
protein_coding	ZNF222	ENSG00000159885.13	11	15	1.4
protein_coding	PIF1	ENSG00000140451.12	11	15	1.4
protein_coding	PAK2	ENSG00000180370.10	135	184	1.4
protein_coding	SRP68	ENSG00000167881.14	80	109	1.4
protein_coding	GOLGA5	ENSG00000066455.12	69	94	1.4
protein_coding	TRIQK	ENSG00000205133.11	69	94	1.4
protein_coding	MTSS1L	ENSG00000132613.14	263	358	1.4
protein_coding	GEM	ENSG00000164949.7	36	49	1.4
protein_coding	UCKL1	ENSG00000198276.15	36	49	1.4
protein_coding	TATDN1	ENSG00000147687.18	36	49	1.4
protein_coding	KREMEN1	ENSG00000183762.12	61	83	1.4
protein_coding	CERS2	ENSG00000143418.19	283	385	1.4
protein_coding	ZBTB4	ENSG00000174282.11	272	370	1.4
protein_coding	HBS1L	ENSG00000112339.14	75	102	1.4
protein_coding	UNC119B	ENSG00000175970.10	50	68	1.4
protein_coding	ZNF740	ENSG00000139651.10	25	34	1.4
protein_coding	SNX25	ENSG00000109762.15	25	34	1.4
protein_coding	MADD	ENSG00000110514.19	25	34	1.4
protein_coding	TRPC4AP	ENSG00000100991.11	139	189	1.4
protein_coding	POLE3	ENSG00000148229.12	78	106	1.4
protein_coding	FAM13B	ENSG00000031003.10	39	53	1.4
protein_coding	AMPH	ENSG00000078053.16	39	53	1.4
protein_coding	CACNA2D3	ENSG00000157445.14	39	53	1.4
protein_coding	DDX55	ENSG00000111364.15	39	53	1.4
lincRNA	HCG11	ENSG00000228223.3	53	72	1.4
protein_coding	SETX	ENSG00000107290.13	226	307	1.4
protein_coding	LGMN	ENSG00000100600.14	67	91	1.4
protein_coding	CHD3	ENSG00000170004.16	215	292	1.4
protein_coding	UBE2A	ENSG00000077721.15	229	311	1.4

protein_coding	NUB1	ENSG00000013374.16	109	148	1.4
protein_coding	RETREG3	ENSG00000141699.10	56	76	1.4
protein_coding	TCEA3	ENSG00000204219.10	42	57	1.4
protein_coding	GRAMD1B	ENSG00000023171.17	28	38	1.4
protein_coding	PIGN	ENSG00000197563.10	28	38	1.4
protein_coding	SUPV3L1	ENSG00000156502.13	28	38	1.4
protein_coding	ZSCAN32	ENSG00000140987.19	14	19	1.4
protein_coding	NIPA1	ENSG00000170113.15	14	19	1.4
protein_coding	PCNX3	ENSG00000197136.4	14	19	1.4
protein_coding	SHTN1	ENSG00000187164.19	14	19	1.4
protein_coding	PAXIP1	ENSG00000157212.18	14	19	1.4
protein_coding	PIGBOS1	ENSG00000225973.3	14	19	1.4
protein_coding	LRIG2	ENSG00000198799.11	14	19	1.4
protein_coding	TRIM66	ENSG00000166436.16	14	19	1.4
protein_coding	ERAL1	ENSG00000132591.11	14	19	1.4
protein_coding	WSB1	ENSG00000109046.14	269	365	1.4
protein_coding	DNAJB6	ENSG00000105993.14	171	232	1.4
protein_coding	SNAPC1	ENSG00000023608.4	59	80	1.4
protein_coding	PRPS2	ENSG00000101911.12	59	80	1.4
protein_coding	GOLT1B	ENSG00000111711.9	104	141	1.4
protein_coding	PTPN1	ENSG00000196396.9	284	385	1.4
protein_coding	NAA30	ENSG00000139977.13	90	122	1.4
protein_coding	RELCH	ENSG00000134444.14	31	42	1.4
protein_coding	OXLD1	ENSG00000204237.4	31	42	1.4
protein_coding	TOR1AIP2	ENSG00000169905.12	223	302	1.4
protein_coding	SLC17A5	ENSG00000119899.12	322	436	1.4
protein_coding	TANK	ENSG00000136560.13	82	111	1.4
protein_coding	TUBA1C	ENSG00000167553.15	280	379	1.4
protein_coding	TNIP2	ENSG00000168884.14	51	69	1.4
protein_coding	SKIV2L	ENSG00000204351.11	34	46	1.4
protein_coding	THADA	ENSG00000115970.18	34	46	1.4
protein_coding	TRMT5	ENSG00000126814.6	17	23	1.4
protein_coding	CDKAL1	ENSG00000145996.11	17	23	1.4
protein_coding	SSB	ENSG00000138385.15	349	472	1.4
protein_coding	EIF3I	ENSG00000084623.11	162	219	1.4
protein_coding	DDRGK1	ENSG00000198171.12	74	100	1.4
protein_coding	VPS33A	ENSG00000139719.10	37	50	1.4
protein_coding	CHURC1	ENSG00000258289.8	77	104	1.4
protein_coding	PLEKHA3	ENSG00000116095.10	80	108	1.4
protein_coding	GCC1	ENSG00000179562.2	60	81	1.4
protein_coding	DNMT3A	ENSG00000119772.16	40	54	1.4
protein_coding	IGSF8	ENSG00000162729.13	20	27	1.4
protein_coding	ZMYND19	ENSG00000165724.5	20	27	1.4
protein_coding	RIOX1	ENSG00000170468.7	20	27	1.4

protein_coding	TRPC1	ENSG00000144935.14	20	27	1.4
protein_coding	ROGDI	ENSG00000067836.12	20	27	1.4
protein_coding	FARS2	ENSG00000145982.12	20	27	1.4
protein_coding	TNS3	ENSG00000136205.16	166	224	1.3
protein_coding	MAPRE1	ENSG00000101367.8	83	112	1.3
protein_coding	TPP2	ENSG00000134900.11	86	116	1.3
protein_coding	AFDN	ENSG00000130396.20	43	58	1.3
protein_coding	ZBTB10	ENSG00000205189.11	43	58	1.3
protein_coding	MFSD14C	ENSG00000196312.13	43	58	1.3
protein_coding	SIGIRR	ENSG00000185187.12	43	58	1.3
protein_coding	CHAMP1	ENSG00000198824.6	43	58	1.3
protein_coding	DYNC1H1	ENSG00000197102.11	1051	1417	1.3
protein_coding	DHX36	ENSG00000174953.13	184	248	1.3
protein_coding	TCTN3	ENSG00000119977.20	92	124	1.3
protein_coding	EXOC3	ENSG00000180104.15	46	62	1.3
protein_coding	FPGS	ENSG00000136877.14	46	62	1.3
protein_coding	ABCB8	ENSG00000197150.12	23	31	1.3
protein_coding	ENTPD5	ENSG00000187097.12	23	31	1.3
protein_coding	RIPK1	ENSG00000137275.13	49	66	1.3
protein_coding	NEMF	ENSG00000165525.17	225	303	1.3
protein_coding	TMEM87B	ENSG00000153214.10	153	206	1.3
protein_coding	YIPF2	ENSG00000130733.10	78	105	1.3
protein_coding	SERAC1	ENSG00000122335.15	26	35	1.3
protein_coding	ACADS	ENSG00000122971.8	26	35	1.3
protein_coding	RAB13	ENSG00000143545.8	246	331	1.3
protein_coding	PIP4K2C	ENSG00000166908.17	58	78	1.3
protein_coding	ALKBH7	ENSG00000125652.7	58	78	1.3
protein_coding	ZNF326	ENSG00000162664.16	29	39	1.3
protein_coding	PRPF38A	ENSG00000134748.12	29	39	1.3
protein_coding	CEP44	ENSG00000164118.12	29	39	1.3
protein_coding	DENND2A	ENSG00000146966.12	29	39	1.3
protein_coding	MAPKAP1	ENSG00000119487.16	151	203	1.3
protein_coding	PHRF1	ENSG00000070047.11	61	82	1.3
protein_coding	YWHAH	ENSG00000128245.14	93	125	1.3
protein_coding	ARFGEF2	ENSG00000124198.8	64	86	1.3
protein_coding	DIMT1	ENSG00000086189.9	64	86	1.3
protein_coding	ZNF451	ENSG00000112200.16	99	133	1.3
protein_coding	C11orf24	ENSG00000171067.10	67	90	1.3
protein_coding	SEC61G	ENSG00000132432.13	452	607	1.3
protein_coding	MUL1	ENSG00000090432.6	35	47	1.3
protein_coding	C8orf33	ENSG00000182307.13	35	47	1.3
protein_coding	SLC25A29	ENSG00000197119.12	35	47	1.3
protein_coding	CYP1B1	ENSG00000138061.11	555	745	1.3
protein_coding	C9orf3	ENSG00000148120.16	111	149	1.3

protein_coding	FNBP1	ENSG00000187239.16	190	255	1.3
protein_coding	STAG1	ENSG00000118007.12	123	165	1.3
protein_coding	RCN3	ENSG00000142552.7	126	169	1.3
protein_coding	UNC50	ENSG00000115446.11	88	118	1.3
protein_coding	ESCO1	ENSG00000141446.10	44	59	1.3
protein_coding	IGFBP2	ENSG00000115457.9	267	358	1.3
protein_coding	FOXJ3	ENSG00000198815.8	47	63	1.3
protein_coding	MED21	ENSG00000152944.8	50	67	1.3
protein_coding	AP2S1	ENSG00000042753.11	153	205	1.3
protein_coding	AKAP13	ENSG00000170776.21	156	209	1.3
protein_coding	PGK¬†1	ENSG00000102144.14	471	631	1.3
protein_coding	HECA	ENSG00000112406.4	159	213	1.3
protein_coding	RPF1	ENSG00000117133.10	53	71	1.3
protein_coding	AP2B1	ENSG00000006125.17	277	371	1.3
protein_coding	WRB	ENSG00000182093.15	59	79	1.3
protein_coding	TUBB4B	ENSG00000188229.5	62	83	1.3
protein_coding	SUN2	ENSG00000100242.15	127	170	1.3
protein_coding	TOPORS	ENSG00000197579.7	127	170	1.3
protein_coding	IRS1	ENSG00000169047.5	257	344	1.3
protein_coding	HIF1AN	ENSG00000166135.13	71	95	1.3
protein_coding	PCGF3	ENSG00000185619.18	71	95	1.3
protein_coding	ATP8B2	ENSG00000143515.16	216	289	1.3
protein_coding	SETD7	ENSG00000145391.13	465	622	1.3
protein_coding	HM13	ENSG00000101294.17	332	444	1.3
protein_coding	FZD1	ENSG00000157240.3	86	115	1.3
protein_coding	WAPL	ENSG00000062650.18	89	119	1.3
protein_coding	CUL1	ENSG00000055130.15	95	127	1.3
protein_coding	SMIM26	ENSG00000232388.4	95	127	1.3
protein_coding	CYB561	ENSG00000008283.15	110	147	1.3
protein_coding	BAZ1B	ENSG00000009954.10	226	302	1.3
protein_coding	CDK14	ENSG00000058091.16	113	151	1.3
protein_coding	VDAC2	ENSG00000165637.13	179	239	1.3
protein_coding	RPS6KA2	ENSG00000071242.11	171	228	1.3
protein_coding	SCAP	ENSG00000114650.19	105	140	1.3
protein_coding	SUN1	ENSG00000164828.17	96	128	1.3
protein_coding	MAP2K3	ENSG00000034152.18	93	124	1.3
protein_coding	DPM1	ENSG00000000419.12	87	116	1.3
protein_coding	ZDHHC8	ENSG00000099904.15	81	108	1.3
protein_coding	SAMM50	ENSG00000100347.14	72	96	1.3
protein_coding	TIA1	ENSG00000116001.15	69	92	1.3
protein_coding	SIKE1	ENSG00000052723.11	60	80	1.3
protein_coding	TMEM11	ENSG00000178307.9	54	72	1.3
protein_coding	CDK12	ENSG00000167258.13	51	68	1.3
protein_coding	MED24	ENSG00000008838.19	51	68	1.3

protein_coding	ELF2	ENSG00000109381.19	51	68	1.3
protein_coding	MINPP1	ENSG00000107789.15	48	64	1.3
protein_coding	STK35	ENSG00000125834.12	48	64	1.3
protein_coding	NOL11	ENSG00000130935.9	48	64	1.3
protein_coding	SRPX2	ENSG00000102359.6	45	60	1.3
protein_coding	CRTC2	ENSG00000160741.16	45	60	1.3
protein_coding	PNPLA6	ENSG00000032444.16	42	56	1.3
protein_coding	CCNQ	ENSG00000262919.7	42	56	1.3
protein_coding	LMAN2L	ENSG00000114988.11	36	48	1.3
antisense	KCNQ1OT1	ENSG00000269821.1	33	44	1.3
protein_coding	TTLL5	ENSG00000119685.19	33	44	1.3
protein_coding	PIK3R4	ENSG00000196455.7	30	40	1.3
transcribed_processed_pseudogene	AC073869.1	ENSG00000152117.17	27	36	1.3
protein_coding	TLE3	ENSG00000140332.15	27	36	1.3
protein_coding	SART1	ENSG00000175467.14	27	36	1.3
protein_coding	FBXO8	ENSG00000164117.13	27	36	1.3
protein_coding	NFYA	ENSG00000001167.14	27	36	1.3
protein_coding	SAYSD1	ENSG00000112167.9	27	36	1.3
protein_coding	MAP1LC3A	ENSG00000101460.12	27	36	1.3
protein_coding	SDE2	ENSG00000143751.9	24	32	1.3
protein_coding	RMDN2	ENSG00000115841.19	24	32	1.3
protein_coding	ACSS3	ENSG00000111058.7	24	32	1.3
protein_coding	TRAF4	ENSG00000076604.14	21	28	1.3
protein_coding	WDR18	ENSG00000065268.10	21	28	1.3
protein_coding	GABPB2	ENSG00000143458.11	21	28	1.3
protein_coding	LAS1L	ENSG00000001497.16	21	28	1.3
protein_coding	ZNF232	ENSG00000167840.13	21	28	1.3
protein_coding	SULF2	ENSG00000196562.14	21	28	1.3
protein_coding	TRNT1	ENSG00000072756.16	18	24	1.3
protein_coding	MED16	ENSG00000175221.14	18	24	1.3
protein_coding	ABCA5	ENSG00000154265.15	15	20	1.3
protein_coding	TPCN2	ENSG00000162341.16	15	20	1.3
protein_coding	BARD1	ENSG00000138376.10	15	20	1.3
protein_coding	RNF167	ENSG00000108523.15	15	20	1.3
protein_coding	MIS12	ENSG00000167842.15	12	16	1.3
protein_coding	FANCL	ENSG00000115392.11	12	16	1.3
protein_coding	MASTL	ENSG00000120539.14	12	16	1.3
protein_coding	ATP10A	ENSG00000206190.11	12	16	1.3
sense_intronic	AC135050.6	ENSG00000278133.1	12	16	1.3
protein_coding	ANAPC1	ENSG00000153107.12	12	16	1.3
protein_coding	PTPMT1	ENSG00000110536.13	12	16	1.3
protein_coding	DGCR8	ENSG00000128191.15	12	16	1.3

protein_coding	POLE	ENSG00000177084.16	12	16	1.3
protein_coding	KRBOX1	ENSG00000240747.7	12	16	1.3
protein_coding	OTUD1	ENSG00000165312.6	9	12	1.3
protein_coding	LYPD3	ENSG00000124466.8	9	12	1.3
protein_coding	TRMT12	ENSG00000183665.5	9	12	1.3
protein_coding	USP35	ENSG00000118369.12	9	12	1.3
protein_coding	ZFP1	ENSG00000184517.11	9	12	1.3
protein_coding	ZNF675	ENSG00000197372.9	9	12	1.3
protein_coding	WDR35	ENSG00000118965.14	9	12	1.3
protein_coding	TSPAN11	ENSG00000110900.14	9	12	1.3
processed_transcript	ZNF561-AS1	ENSG00000267106.5	9	12	1.3
protein_coding	ZNF44	ENSG00000197857.13	6	8	1.3
protein_coding	ABHD11	ENSG00000106077.18	6	8	1.3
protein_coding	URB2	ENSG00000135763.9	6	8	1.3
protein_coding	ZNF10	ENSG00000256223.5	6	8	1.3
antisense	FLJ46906	ENSG00000225177.5	6	8	1.3
protein_coding	OXNAD1	ENSG00000154814.13	6	8	1.3
protein_coding	MFAP5	ENSG00000197614.10	6	8	1.3
protein_coding	KCNC4	ENSG00000116396.14	6	8	1.3
protein_coding	STX1A	ENSG00000106089.11	6	8	1.3
protein_coding	TMEM185A	ENSG00000269556.7	6	8	1.3
antisense	AC004918.1	ENSG00000244701.1	6	8	1.3
lincRNA	SRP14-AS1	ENSG00000248508.6	6	8	1.3
lincRNA	BX537318.1	ENSG00000273145.1	6	8	1.3
protein_coding	FAM83G	ENSG00000188522.14	3	4	1.3
lincRNA	Z68871.1	ENSG00000239407.5	3	4	1.3
protein_coding	RTTN	ENSG00000176225.13	3	4	1.3
lincRNA	AC067838.1	ENSG00000272338.2	3	4	1.3
lincRNA	PAR	ENSG00000236871.7	3	4	1.3
antisense	AL031058.1	ENSG00000261189.1	3	4	1.3
protein_coding	GPS2	ENSG00000132522.15	3	4	1.3
protein_coding	DNAH14	ENSG00000185842.15	3	4	1.3
protein_coding	ACVR1C	ENSG00000123612.15	3	4	1.3
transcribed_unprocessed_pseudogene	AL390728.4	ENSG00000227671.4	3	4	1.3
protein_coding	ZSCAN22	ENSG00000182318.5	3	4	1.3
sense_intronic	AC005726.3	ENSG00000264608.1	3	4	1.3
protein_coding	TMCC2	ENSG00000133069.16	3	4	1.3
sense_intronic	AP000240.1	ENSG00000273017.1	3	4	1.3
antisense	AL135999.1	ENSG00000258727.1	3	4	1.3
transcribed_unprocessed_pseudogene	AC027559.1	ENSG00000259658.5	3	4	1.3
protein_coding	PCDHGB5	ENSG00000276547.1	3	4	1.3

antisense	MHENCR	ENSG00000232442.1	3	4	1.3
antisense	LIPE-AS1	ENSG00000213904.8	3	4	1.3
processed_pseudogene	RPS19P7	ENSG00000228462.1	3	4	1.3
protein_coding	PIP5KL1	ENSG00000167103.11	3	4	1.3
bidirectional_promoter_lnc RNA	AC096537.1	ENSG00000233384.2	3	4	1.3
protein_coding	KLF15	ENSG00000163884.3	3	4	1.3
lincRNA	WWC2-AS2	ENSG00000251359.4	3	4	1.3
sense_intronic	AL008721.2	ENSG00000272977.1	3	4	1.3
protein_coding	MPP3	ENSG00000161647.18	3	4	1.3
protein_coding	ZNF572	ENSG00000180938.5	3	4	1.3
antisense	RUNDC3A-	ENSG00000267750.5	3	4	1.3
protein_coding	COL11A2	ENSG00000204248.10	3	4	1.3
protein_coding	FRRS1L	ENSG00000260230.3	3	4	1.3
protein_coding	GTSE1	ENSG00000075218.18	3	4	1.3
antisense	ZNF571-AS1	ENSG00000267470.5	3	4	1.3
sense_overlapping	AC026803.1	ENSG00000260366.1	3	4	1.3
antisense	AL355472.1	ENSG00000231663.1	3	4	1.3
antisense	NRAV	ENSG00000248008.2	3	4	1.3
antisense	AC021054.1	ENSG00000177406.4	3	4	1.3
antisense	AC068338.3	ENSG00000275645.1	3	4	1.3
protein_coding	CDRT4	ENSG00000239704.10	3	4	1.3
antisense	MRGPRF-	ENSG00000256508.2	3	4	1.3
protein_coding	VSTM2L	ENSG00000132821.11	3	4	1.3
protein_coding	TDRD12	ENSG00000173809.18	3	4	1.3
lincRNA	LINC01730	ENSG00000275491.1	3	4	1.3
protein_coding	HYAL3	ENSG00000186792.16	3	4	1.3
antisense	AC116913.1	ENSG00000261351.2	3	4	1.3
TEC	AC131009.4	ENSG00000279283.1	3	4	1.3
antisense	KCNJ2-AS1	ENSG00000267365.1	3	4	1.3
protein_coding	TRAF1	ENSG00000056558.10	3	4	1.3
TEC	AC005225.4	ENSG00000279026.1	3	4	1.3
protein_coding	REXO5	ENSG00000005189.19	3	4	1.3
protein_coding	TMTC1	ENSG00000133687.15	175	233	1.3
protein_coding	SCYL1	ENSG00000142186.16	175	233	1.3
protein_coding	LMBRD1	ENSG00000168216.11	172	229	1.3
protein_coding	THOC7	ENSG00000163634.11	133	177	1.3
protein_coding	SMC1A	ENSG00000072501.17	121	161	1.3
protein_coding	Sep-07	ENSG00000122545.19	451	600	1.3
protein_coding	EDIL3	ENSG00000164176.12	1164	1548	1.3
protein_coding	TRAK2	ENSG00000115993.12	170	226	1.3
protein_coding	PBRM1	ENSG00000163939.18	161	214	1.3
protein_coding	FEM1C	ENSG00000145780.7	76	101	1.3

protein_coding	PPP1R12C	ENSG00000125503.12	64	85	1.3
protein_coding	SLC20A2	ENSG00000168575.9	61	81	1.3
protein_coding	MORF4L2	ENSG00000123562.16	629	835	1.3
protein_coding	CCNT1	ENSG00000129315.10	55	73	1.3
protein_coding	ZRANB1	ENSG00000019995.6	104	138	1.3
protein_coding	RAE1	ENSG00000101146.12	52	69	1.3
protein_coding	CASP6	ENSG00000138794.9	52	69	1.3
protein_coding	UBA2	ENSG00000126261.12	147	195	1.3
protein_coding	DZIP1	ENSG00000134874.17	98	130	1.3
protein_coding	DEK	ENSG00000124795.15	484	642	1.3
protein_coding	CALCOCO2	ENSG00000136436.14	236	313	1.3
protein_coding	TMEM259	ENSG00000182087.13	236	313	1.3
protein_coding	EIF2AK3	ENSG00000172071.11	46	61	1.3
protein_coding	SRSF8	ENSG00000263465.4	46	61	1.3
protein_coding	MRPL44	ENSG00000135900.3	46	61	1.3
protein_coding	DNAJA2	ENSG00000069345.11	86	114	1.3
protein_coding	STX16	ENSG00000124222.22	86	114	1.3
protein_coding	RNF10	ENSG00000022840.15	209	277	1.3
protein_coding	ELMO2	ENSG00000062598.17	80	106	1.3
protein_coding	ZDHHC6	ENSG00000023041.11	40	53	1.3
protein_coding	EBAG9	ENSG00000147654.14	40	53	1.3
protein_coding	GNG12	ENSG00000172380.5	345	457	1.3
protein_coding	SYNE1	ENSG00000131018.22	74	98	1.3
protein_coding	ZBED5	ENSG00000236287.7	74	98	1.3
protein_coding	SNRNP27	ENSG00000124380.10	71	94	1.3
protein_coding	LUC7L2	ENSG00000146963.17	105	139	1.3
protein_coding	PHF12	ENSG00000109118.13	34	45	1.3
protein_coding	ITPK1	ENSG00000100605.16	34	45	1.3
protein_coding	SPATA7	ENSG00000042317.16	34	45	1.3
protein_coding	CAPZB	ENSG00000077549.17	719	951	1.3
protein_coding	FLT1	ENSG00000102755.11	62	82	1.3
protein_coding	SMARCAD1	ENSG00000163104.17	62	82	1.3
protein_coding	SF3B4	ENSG00000143368.9	62	82	1.3
protein_coding	SYTL2	ENSG00000137501.17	62	82	1.3
protein_coding	CEP57	ENSG00000166037.10	121	160	1.3
protein_coding	TRIM4	ENSG00000146833.15	90	119	1.3
lincRNA	JPX	ENSG00000225470.7	118	156	1.3
protein_coding	HABP4	ENSG00000130956.13	87	115	1.3
protein_coding	MTLN	ENSG00000175701.10	56	74	1.3
protein_coding	TMEM120B	ENSG00000188735.12	28	37	1.3
protein_coding	FAM149B1	ENSG00000138286.14	28	37	1.3
protein_coding	CYP27A1	ENSG00000135929.8	28	37	1.3
protein_coding	ALYREF	ENSG00000183684.7	28	37	1.3
protein_coding	CCDC149	ENSG00000181982.18	28	37	1.3

protein_coding	UBE2D1	ENSG00000072401.14	81	107	1.3
protein_coding	MLLT11	ENSG00000213190.3	53	70	1.3
protein_coding	TFCP2	ENSG00000135457.9	50	66	1.3
protein_coding	SKAP2	ENSG00000005020.12	50	66	1.3
protein_coding	MOAP1	ENSG00000165943.4	25	33	1.3
protein_coding	WRN	ENSG00000165392.9	25	33	1.3
protein_coding	SEC23B	ENSG00000101310.16	25	33	1.3
protein_coding	GMPPA	ENSG00000144591.18	25	33	1.3
protein_coding	ATG16L1	ENSG00000085978.21	25	33	1.3
protein_coding	JHY	ENSG00000109944.10	25	33	1.3
protein_coding	RUFY2	ENSG00000204130.13	47	62	1.3
protein_coding	UBR5	ENSG00000104517.12	345	455	1.3
protein_coding	HNRNPU	ENSG00000153187.19	606	799	1.3
protein_coding	CFDP1	ENSG00000153774.8	110	145	1.3
protein_coding	MAGEF1	ENSG00000177383.4	66	87	1.3
protein_coding	INPP4A	ENSG00000040933.15	44	58	1.3
protein_coding	ACCS	ENSG00000110455.13	22	29	1.3
protein_coding	PBLD	ENSG00000108187.15	22	29	1.3
protein_coding	STT3B	ENSG00000163527.9	217	286	1.3
protein_coding	STX2	ENSG00000111450.13	85	112	1.3
protein_coding	CAMK2N1	ENSG00000162545.5	167	220	1.3
protein_coding	EMP2	ENSG00000213853.9	186	245	1.3
protein_coding	WDFY1	ENSG00000085449.14	82	108	1.3
protein_coding	KIFC3	ENSG00000140859.15	41	54	1.3
protein_coding	TPM4	ENSG00000167460.15	2020	2660	1.3
protein_coding	H6PD	ENSG00000049239.12	101	133	1.3
protein_coding	DCAF7	ENSG00000136485.14	155	204	1.3
protein_coding	AIMP1	ENSG00000164022.16	95	125	1.3
protein_coding	RPAIN	ENSG00000129197.14	95	125	1.3
protein_coding	SAFB2	ENSG00000130254.11	76	100	1.3
protein_coding	PIGS	ENSG00000087111.20	76	100	1.3
protein_coding	CDK2AP2	ENSG00000167797.7	38	50	1.3
protein_coding	COASY	ENSG00000068120.14	38	50	1.3
protein_coding	DNAJA3	ENSG00000103423.13	38	50	1.3
protein_coding	ZBTB45	ENSG00000119574.12	19	25	1.3
protein_coding	SENP7	ENSG00000138468.15	19	25	1.3
protein_coding	STOML1	ENSG00000067221.13	19	25	1.3
protein_coding	SACS	ENSG00000151835.15	203	267	1.3
protein_coding	ORMDL1	ENSG00000128699.13	92	121	1.3
protein_coding	PFKL	ENSG00000141959.16	92	121	1.3
protein_coding	PI4KA	ENSG00000241973.10	54	71	1.3
protein_coding	C1orf21	ENSG00000116667.13	280	368	1.3
protein_coding	UGGT2	ENSG00000102595.19	105	138	1.3
protein_coding	GPBP1L1	ENSG00000159592.10	105	138	1.3

protein_coding	CERCAM	ENSG00000167123.18	105	138	1.3
protein_coding	PPCS	ENSG00000127125.8	70	92	1.3
protein_coding	ARHGEF12	ENSG00000196914.8	172	226	1.3
protein_coding	ST3GAL2	ENSG00000157350.12	102	134	1.3
protein_coding	BCCIP	ENSG00000107949.16	51	67	1.3
protein_coding	PSMB7	ENSG00000136930.12	303	398	1.3
protein_coding	MARCH5	ENSG00000198060.9	83	109	1.3
protein_coding	RAB10	ENSG00000084733.10	364	478	1.3
protein_coding	HIST2H2BE	ENSG00000184678.10	48	63	1.3
protein_coding	DGCR6L	ENSG00000128185.9	48	63	1.3
protein_coding	DPCD	ENSG00000166171.12	16	21	1.3
protein_coding	C1orf109	ENSG00000116922.14	16	21	1.3
protein_coding	RXRA	ENSG00000186350.10	109	143	1.3
protein_coding	VPS13C	ENSG00000129003.17	170	223	1.3
protein_coding	B4GAT1	ENSG00000174684.6	77	101	1.3
protein_coding	DNAJB12	ENSG00000148719.14	90	118	1.3
protein_coding	PAR	ENSG00000197976.11	45	59	1.3
protein_coding	ZBTB38	ENSG00000177311.11	493	646	1.3
protein_coding	XRN1	ENSG00000114127.10	116	152	1.3
protein_coding	LIN7C	ENSG00000148943.11	87	114	1.3
protein_coding	PLTP	ENSG00000100979.14	29	38	1.3
protein_coding	ZNF22	ENSG00000165512.4	29	38	1.3
protein_coding	ADAL	ENSG00000168803.15	29	38	1.3
protein_coding	DPYSL3	ENSG00000113657.12	242	317	1.3
protein_coding	NUDT5	ENSG00000165609.12	126	165	1.3
protein_coding	NUP214	ENSG00000126883.16	42	55	1.3
protein_coding	KCTD2	ENSG00000180901.10	42	55	1.3
protein_coding	ZMYM2	ENSG00000121741.16	81	106	1.3
protein_coding	PTPRM	ENSG00000173482.16	185	242	1.3
protein_coding	NCOR1	ENSG00000141027.20	341	446	1.3
protein_coding	ATG12	ENSG00000145782.12	169	221	1.3
protein_coding	MON1B	ENSG00000103111.14	104	136	1.3
protein_coding	PSMA5	ENSG00000143106.12	104	136	1.3
protein_coding	DNTTIP2	ENSG00000067334.13	78	102	1.3
protein_coding	SNN	ENSG00000184602.5	52	68	1.3
protein_coding	RMND5A	ENSG00000153561.12	39	51	1.3
protein_coding	PDE6D	ENSG00000156973.13	39	51	1.3
protein_coding	TOP2A	ENSG00000131747.14	26	34	1.3
protein_coding	YARS2	ENSG00000139131.12	26	34	1.3
protein_coding	NKRF	ENSG00000186416.14	13	17	1.3
protein_coding	IKZF4	ENSG00000123411.14	13	17	1.3
protein_coding	METTL18	ENSG00000171806.11	13	17	1.3
protein_coding	DONSON	ENSG00000159147.17	13	17	1.3
protein_coding	SARNP	ENSG00000205323.8	13	17	1.3

protein_coding	HMGN2	ENSG00000198830.10	13	17	1.3
protein_coding	NACAD	ENSG00000136274.8	13	17	1.3
protein_coding	SBF1	ENSG00000100241.20	101	132	1.3
protein_coding	SPCS3	ENSG00000129128.12	326	426	1.3
protein_coding	SPTLC2	ENSG00000100596.6	124	162	1.3
protein_coding	SGTB	ENSG00000197860.9	49	64	1.3
protein_coding	MLH3	ENSG00000119684.15	49	64	1.3
snoRNA	SNORD13	ENSG00000239039.1	49	64	1.3
protein_coding	CARNMT1	ENSG00000156017.12	49	64	1.3
protein_coding	RHOQ	ENSG00000119729.11	624	815	1.3
protein_coding	COL4A2	ENSG00000134871.18	288	376	1.3
protein_coding	MAST4	ENSG00000069020.18	108	141	1.3
protein_coding	LYSMD3	ENSG00000176018.12	36	47	1.3
protein_coding	CALCOCO1	ENSG00000012822.15	36	47	1.3
protein_coding	BRD3	ENSG00000169925.16	220	287	1.3
protein_coding	BCAP29	ENSG00000075790.10	184	240	1.3
protein_coding	TFRC	ENSG00000072274.12	184	240	1.3
protein_coding	DERL2	ENSG00000072849.10	69	90	1.3
protein_coding	PPP1R13L	ENSG00000104881.15	23	30	1.3
protein_coding	UTP18	ENSG00000011260.13	23	30	1.3
protein_coding	UFD1	ENSG00000070010.18	148	193	1.3
protein_coding	MT-CO1	ENSG00000198804.2	1488	1940	1.3
protein_coding	ITGB5	ENSG00000082781.11	854	1113	1.3
protein_coding	ATM	ENSG00000149311.18	122	159	1.3
protein_coding	ARHGEF25	ENSG00000240771.6	66	86	1.3
protein_coding	MLYCD	ENSG00000103150.5	33	43	1.3
protein_coding	RAB22A	ENSG00000124209.3	142	185	1.3
protein_coding	CAST	ENSG00000153113.23	677	882	1.3
protein_coding	MRFAP1L1	ENSG00000178988.10	152	198	1.3
protein_coding	UBE2M	ENSG00000130725.7	76	99	1.3
protein_coding	RNF220	ENSG00000187147.17	76	99	1.3
protein_coding	RSPH3	ENSG00000130363.11	76	99	1.3
protein_coding	CCDC25	ENSG00000147419.17	195	254	1.3
protein_coding	YIPF1	ENSG00000058799.14	43	56	1.3
protein_coding	CLIP1	ENSG00000130779.20	311	405	1.3
protein_coding	SLC6A6	ENSG00000131389.17	182	237	1.3
protein_coding	SUMO1	ENSG00000116030.16	149	194	1.3
protein_coding	NDUFS2	ENSG00000158864.12	149	194	1.3
protein_coding	TMEM35A	ENSG00000126950.7	106	138	1.3
protein_coding	TGFBR1	ENSG00000106799.12	53	69	1.3
protein_coding	RTL6	ENSG00000188636.3	53	69	1.3
protein_coding	SRA1	ENSG00000213523.10	53	69	1.3
protein_coding	SPAG9	ENSG00000008294.20	156	203	1.3
protein_coding	CWC15	ENSG00000150316.11	226	294	1.3

protein_coding	DLD	ENSG00000091140.13	113	147	1.3
protein_coding	TMEM45A	ENSG00000181458.10	140	182	1.3
protein_coding	MIA3	ENSG00000154305.16	140	182	1.3
protein_coding	COL4A3BP	ENSG00000113163.16	110	143	1.3
protein_coding	SNRPG	ENSG00000143977.13	70	91	1.3
protein_coding	AP3M2	ENSG00000070718.11	60	78	1.3
protein_coding	C11orf68	ENSG00000175573.6	50	65	1.3
protein_coding	RPE	ENSG00000197713.14	50	65	1.3
protein_coding	BRAF	ENSG00000157764.13	40	52	1.3
protein_coding	ZNF516	ENSG00000101493.10	30	39	1.3
protein_coding	TMEM199	ENSG00000244045.12	30	39	1.3
transcribed_unprocessed_pseudogene	SBDSP1	ENSG00000225648.5	10	13	1.3
protein_coding	USP45	ENSG00000123552.17	10	13	1.3
processed_pseudogene	AC246787.1	ENSG00000225200.2	10	13	1.3
protein_coding	SHB	ENSG00000107338.9	10	13	1.3
protein_coding	HDX	ENSG00000165259.13	10	13	1.3
protein_coding	CSTA	ENSG00000121552.3	10	13	1.3
protein_coding	LIG1	ENSG00000105486.13	10	13	1.3
protein_coding	PPP5C	ENSG00000011485.14	10	13	1.3
protein_coding	PCGF1	ENSG00000115289.13	10	13	1.3
protein_coding	MIPOL1	ENSG00000151338.18	10	13	1.3
protein_coding	GEN1	ENSG00000178295.14	10	13	1.3
protein_coding	MEMO1	ENSG00000162959.13	10	13	1.3
protein_coding	WDR89	ENSG00000140006.11	10	13	1.3
protein_coding	ZNF765	ENSG00000196417.12	10	13	1.3
protein_coding	THAP5	ENSG00000177683.13	57	74	1.3
protein_coding	NDUFA5	ENSG00000128609.14	141	183	1.3
protein_coding	TMEM204	ENSG00000131634.13	94	122	1.3
protein_coding	SRPK1	ENSG00000096063.15	84	109	1.3
protein_coding	NLN	ENSG00000123213.22	37	48	1.3
protein_coding	RAB2A	ENSG00000104388.14	637	826	1.3
protein_coding	FMC1	ENSG00000164898.12	54	70	1.3
protein_coding	CCDC32	ENSG00000128891.15	27	35	1.3
protein_coding	CCNB1IP1	ENSG00000100814.17	27	35	1.3
protein_coding	FLAD1	ENSG00000160688.18	27	35	1.3
protein_coding	ATXN3	ENSG00000066427.22	71	92	1.3
protein_coding	DST	ENSG00000151914.19	836	1083	1.3
protein_coding	AKT1	ENSG00000142208.16	193	250	1.3
protein_coding	AKAP9	ENSG00000127914.16	346	448	1.3
protein_coding	DLGAP4	ENSG00000080845.17	180	233	1.3
protein_coding	RRM1	ENSG00000167325.14	231	299	1.3
protein_coding	PAQR5	ENSG00000137819.13	68	88	1.3

protein_coding	LYN	ENSG00000254087.7	51	66	1.3
protein_coding	ATG2A	ENSG00000110046.12	34	44	1.3
protein_coding	THAP6	ENSG00000174796.12	17	22	1.3
processed_transcript	TPT1-AS1	ENSG00000170919.15	17	22	1.3
protein_coding	TBC1D10B	ENSG00000169221.13	17	22	1.3
protein_coding	WASF3	ENSG00000132970.12	17	22	1.3
antisense	ASB16-AS1	ENSG00000267080.5	17	22	1.3
protein_coding	ACAD10	ENSG00000111271.14	17	22	1.3
protein_coding	SSR3	ENSG00000114850.6	361	467	1.3
protein_coding	DDX27	ENSG00000124228.14	109	141	1.3
protein_coding	PFDN2	ENSG00000143256.4	75	97	1.3
protein_coding	KLHL7	ENSG00000122550.17	58	75	1.3
protein_coding	HSP90AA1	ENSG00000080824.18	2150	2780	1.3
Mt_rRNA	MT-RNR1	ENSG00000211459.2	4846	6265	1.3
protein_coding	FER	ENSG00000151422.12	41	53	1.3
protein_coding	MAN1A1	ENSG00000111885.6	144	186	1.3
protein_coding	PREB	ENSG00000138073.13	48	62	1.3
protein_coding	HMBOX1	ENSG00000147421.17	24	31	1.3
antisense	AL121603.2	ENSG00000258738.1	24	31	1.3
protein_coding	LRRC58	ENSG00000163428.3	151	195	1.3
protein_coding	SCO1	ENSG00000133028.11	79	102	1.3
protein_coding	PPP3R1	ENSG00000221823.10	189	244	1.3
protein_coding	HBP1	ENSG00000105856.13	244	315	1.3
protein_coding	ATP5MG	ENSG00000167283.7	705	910	1.3
protein_coding	UBE2Z	ENSG00000159202.17	234	302	1.3
protein_coding	FXR1	ENSG00000114416.17	272	351	1.3
protein_coding	ARHGAP35	ENSG00000160007.18	186	240	1.3
protein_coding	SELENOK	ENSG00000113811.10	62	80	1.3
protein_coding	ZNF593	ENSG00000142684.8	31	40	1.3
protein_coding	ATR	ENSG00000175054.14	31	40	1.3
protein_coding	NAB1	ENSG00000138386.16	31	40	1.3
protein_coding	RNASEH2B	ENSG00000136104.20	31	40	1.3
protein_coding	CYB561D2	ENSG00000114395.10	31	40	1.3
protein_coding	SLC2A11	ENSG00000133460.19	31	40	1.3
protein_coding	ATXN2	ENSG00000204842.15	193	249	1.3
protein_coding	ASCC3	ENSG00000112249.13	131	169	1.3
protein_coding	MAGEH1	ENSG00000187601.4	114	147	1.3
protein_coding	PACSIN2	ENSG00000100266.18	76	98	1.3
protein_coding	TBC1D22A	ENSG00000054611.13	38	49	1.3
protein_coding	RPP38	ENSG00000152464.14	38	49	1.3
protein_coding	CHPF	ENSG00000123989.13	204	263	1.3
protein_coding	SLC25A24	ENSG00000085491.16	135	174	1.3
protein_coding	BCAR1	ENSG00000050820.16	104	134	1.3
protein_coding	ADPGK	ENSG00000159322.17	104	134	1.3

protein_coding	SLU7	ENSG00000164609.9	215	277	1.3
protein_coding	TMX3	ENSG00000166479.9	309	398	1.3
protein_coding	CLCC1	ENSG00000121940.15	73	94	1.3
protein_coding	FDPS	ENSG00000160752.14	73	94	1.3
protein_coding	CCDC47	ENSG00000108588.13	153	197	1.3
protein_coding	PTPN12	ENSG00000127947.15	181	233	1.3
protein_coding	RBMS3	ENSG00000144642.21	202	260	1.3
protein_coding	VAPB	ENSG00000124164.15	108	139	1.3
protein_coding	MICAL2	ENSG00000133816.15	331	426	1.3
protein_coding	IFNAR1	ENSG00000142166.12	115	148	1.3
protein_coding	VSTM4	ENSG00000165633.12	342	440	1.3
protein_coding	LUC7L3	ENSG00000108848.15	385	495	1.3
protein_coding	DSE	ENSG00000111817.17	189	243	1.3
protein_coding	ANKLE2	ENSG00000176915.14	84	108	1.3
protein_coding	RFK	ENSG00000135002.11	63	81	1.3
protein_coding	NUP133	ENSG00000069248.11	63	81	1.3
protein_coding	SNX4	ENSG00000114520.10	49	63	1.3
protein_coding	TMEM41B	ENSG00000166471.10	42	54	1.3
protein_coding	MPDU1	ENSG00000129255.15	42	54	1.3
protein_coding	CRADD	ENSG00000169372.12	35	45	1.3
protein_coding	LZTFL1	ENSG00000163818.16	35	45	1.3
protein_coding	PARN	ENSG00000140694.16	35	45	1.3
protein_coding	RASGRF2	ENSG00000113319.12	28	36	1.3
lincRNA	AC245060.5	ENSG00000274422.1	28	36	1.3
protein_coding	RUSC2	ENSG00000198853.11	21	27	1.3
protein_coding	TTC23	ENSG00000103852.12	21	27	1.3
protein_coding	PPP1R3F	ENSG00000049769.12	14	18	1.3
protein_coding	TECR	ENSG00000099797.14	14	18	1.3
protein_coding	FRAT2	ENSG00000181274.6	14	18	1.3
protein_coding	ZNF627	ENSG00000198551.9	14	18	1.3
protein_coding	TMEM106A	ENSG00000184988.8	14	18	1.3
protein_coding	SFXN5	ENSG00000144040.12	14	18	1.3
protein_coding	YDJC	ENSG00000161179.13	14	18	1.3
protein_coding	NCAPD3	ENSG00000151503.12	7	9	1.3
protein_coding	NETO2	ENSG00000171208.9	7	9	1.3
protein_coding	GPR157	ENSG00000180758.11	7	9	1.3
protein_coding	TIPIN	ENSG00000075131.9	7	9	1.3
protein_coding	PAQR7	ENSG00000182749.5	7	9	1.3
protein_coding	RABL2A	ENSG00000144134.18	7	9	1.3
protein_coding	ZNF569	ENSG00000196437.10	7	9	1.3
processed_transcript	AC008969.1	ENSG00000176593.7	7	9	1.3
protein_coding	ZNF668	ENSG00000167394.12	7	9	1.3
protein_coding	ITPKB	ENSG00000143772.9	7	9	1.3
protein_coding	FAM155A	ENSG00000204442.3	7	9	1.3

processed_transcript	COX10-AS1	ENSG00000236088.9	7	9	1.3
protein_coding	IRAK1BP1	ENSG00000146243.13	7	9	1.3
protein_coding	ACTRT3	ENSG00000184378.2	7	9	1.3
protein_coding	N4BP3	ENSG00000145911.5	7	9	1.3
protein_coding	CLHC1	ENSG00000162994.15	7	9	1.3
protein_coding	SMAD2	ENSG00000175387.15	151	194	1.3
protein_coding	ZMYND11	ENSG00000015171.19	137	176	1.3
protein_coding	PPM1A	ENSG00000100614.17	123	158	1.3
protein_coding	TM9SF3	ENSG00000077147.15	457	587	1.3
protein_coding	GABARAPL2	ENSG00000034713.7	275	353	1.3
protein_coding	TBC1D5	ENSG00000131374.14	134	172	1.3
protein_coding	HSD17B12	ENSG00000149084.12	254	326	1.3
protein_coding	PRRC2B	ENSG00000130723.19	173	222	1.3
protein_coding	ALG8	ENSG00000159063.12	53	68	1.3
protein_coding	FAXDC2	ENSG00000170271.10	53	68	1.3
protein_coding	NDUFA1	ENSG00000125356.6	368	472	1.3
protein_coding	SERINC5	ENSG00000164300.16	92	118	1.3
protein_coding	HIPK1	ENSG00000163349.21	92	118	1.3
protein_coding	GNA12	ENSG00000146535.13	85	109	1.3
protein_coding	USP8	ENSG00000138592.13	202	259	1.3
protein_coding	PSMG3	ENSG00000157778.8	39	50	1.3
protein_coding	KIAA1191	ENSG00000122203.14	167	214	1.3
protein_coding	CEP70	ENSG00000114107.8	32	41	1.3
protein_coding	GOLGA8B	ENSG00000215252.11	32	41	1.3
protein_coding	CBR4	ENSG00000145439.11	32	41	1.3
protein_coding	HIC2	ENSG00000169635.9	32	41	1.3
protein_coding	RTF1	ENSG00000137815.14	217	278	1.3
protein_coding	HEBP2	ENSG00000051620.10	338	433	1.3
protein_coding	PYGB	ENSG00000100994.11	82	105	1.3
protein_coding	PRELID3B	ENSG00000101166.15	50	64	1.3
protein_coding	ZMYM6	ENSG00000163867.16	50	64	1.3
protein_coding	SHLD2	ENSG00000122376.11	25	32	1.3
protein_coding	CDK5RAP2	ENSG00000136861.17	118	151	1.3
protein_coding	TERF1	ENSG00000147601.13	93	119	1.3
protein_coding	TTC3	ENSG00000182670.13	1102	1410	1.3
protein_coding	SOCS3	ENSG00000184557.4	136	174	1.3
protein_coding	MRPL47	ENSG00000136522.13	86	110	1.3
protein_coding	OBSL1	ENSG00000124006.14	86	110	1.3
protein_coding	COIL	ENSG00000121058.4	43	55	1.3
protein_coding	SLC39A9	ENSG00000029364.11	97	124	1.3
protein_coding	PUM3	ENSG00000080608.9	54	69	1.3
protein_coding	KCTD13	ENSG00000174943.10	18	23	1.3
protein_coding	FAM160A2	ENSG00000051009.10	18	23	1.3
protein_coding	SCYL3	ENSG00000000457.13	18	23	1.3

protein_coding	TRO	ENSG00000067445.20	18	23	1.3
protein_coding	MOXD1	ENSG00000079931.14	256	327	1.3
protein_coding	FBXO32	ENSG00000156804.7	375	479	1.3
protein_coding	SRP54	ENSG00000100883.11	83	106	1.3
protein_coding	CWC22	ENSG00000163510.13	65	83	1.3
protein_coding	MDM4	ENSG00000198625.12	47	60	1.3
protein_coding	METTL15	ENSG00000169519.20	47	60	1.3
protein_coding	BUD31	ENSG00000106245.10	76	97	1.3
protein_coding	ARFGEF1	ENSG00000066777.8	116	148	1.3
protein_coding	ACTL6A	ENSG00000136518.16	29	37	1.3
protein_coding	DIP2B	ENSG00000066084.12	29	37	1.3
protein_coding	NOP9	ENSG00000196943.13	29	37	1.3
protein_coding	PSMD3	ENSG00000108344.14	185	236	1.3
protein_coding	BCLAF1	ENSG00000029363.16	178	227	1.3
protein_coding	TAGLN2	ENSG00000158710.14	476	607	1.3
protein_coding	FBXO21	ENSG00000135108.14	160	204	1.3
bidirectional_promoter_lncRNA	LINC00205	ENSG00000223768.2	40	51	1.3
protein_coding	TMUB2	ENSG00000168591.15	40	51	1.3
protein_coding	KIF2A	ENSG00000068796.16	102	130	1.3
protein_coding	AGO3	ENSG00000126070.19	51	65	1.3
protein_coding	PNRC1	ENSG00000146278.10	616	785	1.3
protein_coding	ATP5PO	ENSG00000241837.6	365	465	1.3
protein_coding	ZNF146	ENSG00000167635.11	117	149	1.3
protein_coding	NEK9	ENSG00000119638.12	117	149	1.3
protein_coding	RANBP2	ENSG00000153201.15	139	177	1.3
protein_coding	POLR2A	ENSG00000181222.15	352	448	1.3
protein_coding	FXYD5	ENSG00000089327.14	143	182	1.3
protein_coding	ELOVL1	ENSG00000066322.14	66	84	1.3
protein_coding	NSUN2	ENSG00000037474.14	55	70	1.3
protein_coding	ZC3H6	ENSG00000188177.13	55	70	1.3
protein_coding	CTDP1	ENSG00000060069.16	44	56	1.3
protein_coding	ZDHHC21	ENSG00000175893.11	33	42	1.3
protein_coding	WDFY2	ENSG00000139668.8	33	42	1.3
protein_coding	RNF169	ENSG00000166439.5	33	42	1.3
protein_coding	GALC	ENSG00000054983.16	33	42	1.3
protein_coding	SLC2A8	ENSG00000136856.17	22	28	1.3
protein_coding	BCORL1	ENSG00000085185.15	22	28	1.3
protein_coding	GEMIN6	ENSG00000152147.10	22	28	1.3
protein_coding	PANK1	ENSG00000152782.16	11	14	1.3
protein_coding	HSD11B1L	ENSG00000167733.13	11	14	1.3
protein_coding	MCM8	ENSG00000125885.13	11	14	1.3
protein_coding	SLC24A1	ENSG00000074621.13	11	14	1.3

protein_coding	ANKRD34A	ENSG00000272031.2	11	14	1.3
protein_coding	KRIT1	ENSG00000001631.15	11	14	1.3
protein_coding	NANP	ENSG00000170191.4	11	14	1.3
protein_coding	WDR25	ENSG00000176473.13	11	14	1.3
protein_coding	NECAB3	ENSG00000125967.16	11	14	1.3
protein_coding	C17orf97	ENSG00000187624.8	11	14	1.3
protein_coding	XRCC5	ENSG00000079246.15	415	528	1.3
protein_coding	TPBG	ENSG00000146242.8	261	332	1.3
protein_coding	PHLDA2	ENSG00000181649.6	92	117	1.3
protein_coding	PAR	ENSG00000169084.13	92	117	1.3
protein_coding	PON2	ENSG00000105854.12	361	459	1.3
protein_coding	ZMYND8	ENSG00000101040.19	70	89	1.3
protein_coding	SYNGR2	ENSG00000108639.7	70	89	1.3
protein_coding	UBXN4	ENSG00000144224.16	505	642	1.3
protein_coding	ZMPSTE24	ENSG00000084073.8	118	150	1.3
protein_coding	PLEKHA5	ENSG00000052126.14	321	408	1.3
protein_coding	SAMD8	ENSG00000156671.13	144	183	1.3
protein_coding	SHROOM1	ENSG00000164403.14	37	47	1.3
protein_coding	CCP110	ENSG00000103540.16	37	47	1.3
protein_coding	RABEPK	ENSG00000136933.16	37	47	1.3
protein_coding	FAM32A	ENSG00000105058.11	248	315	1.3
protein_coding	HDAC7	ENSG00000061273.17	241	306	1.3
protein_coding	TCOF1	ENSG00000070814.19	52	66	1.3
protein_coding	RFX1	ENSG00000132005.8	26	33	1.3
protein_coding	HNRNPA2B1	ENSG00000122566.21	535	679	1.3
protein_coding	LOX	ENSG00000113083.13	2056	2608	1.3
protein_coding	RNF168	ENSG00000163961.4	41	52	1.3
protein_coding	TRIM27	ENSG00000204713.10	41	52	1.3
protein_coding	NOL9	ENSG00000162408.10	41	52	1.3
protein_coding	FAM208B	ENSG00000108021.20	56	71	1.3
protein_coding	AIFM1	ENSG00000156709.13	56	71	1.3
protein_coding	CRK	ENSG00000167193.7	187	237	1.3
protein_coding	SCYL2	ENSG00000136021.18	176	223	1.3
protein_coding	SEC63	ENSG00000025796.13	367	465	1.3
protein_coding	HEXIM1	ENSG00000186834.3	180	228	1.3
protein_coding	YIPF6	ENSG00000181704.11	105	133	1.3
protein_coding	CHM	ENSG00000188419.13	75	95	1.3
protein_coding	PSMB8	ENSG00000204264.9	75	95	1.3
protein_coding	TMEM50B	ENSG00000142188.16	60	76	1.3
protein_coding	PLEKHA1	ENSG00000107679.14	45	57	1.3
protein_coding	RIPOR3	ENSG00000042062.11	45	57	1.3
protein_coding	TESK1	ENSG00000107140.15	30	38	1.3
protein_coding	ODC1	ENSG00000115758.12	30	38	1.3
protein_coding	PRRT3	ENSG00000163704.11	30	38	1.3

protein_coding	NCDN	ENSG00000020129.15	30	38	1.3
protein_coding	VPS16	ENSG00000215305.9	30	38	1.3
protein_coding	CRY1	ENSG00000008405.11	15	19	1.3
protein_coding	ZUP1	ENSG00000153975.9	15	19	1.3
protein_coding	TTC13	ENSG00000143643.12	15	19	1.3
protein_coding	ERVK3-1	ENSG00000142396.10	15	19	1.3
protein_coding	BRI3BP	ENSG00000184992.10	15	19	1.3
protein_coding	BCKDHB	ENSG00000083123.14	15	19	1.3
protein_coding	NPHP1	ENSG00000144061.12	15	19	1.3
protein_coding	WASHC2C	ENSG00000172661.18	64	81	1.3
protein_coding	HTATSF1	ENSG00000102241.11	113	143	1.3
protein_coding	FRMD8	ENSG00000126391.13	113	143	1.3
protein_coding	STX6	ENSG00000135823.13	49	62	1.3
protein_coding	ARMCX2	ENSG00000184867.13	204	258	1.3
protein_coding	NAALADL2	ENSG00000177694.15	34	43	1.3
protein_coding	TLK2	ENSG00000146872.17	53	67	1.3
protein_coding	TMED2	ENSG00000086598.10	281	355	1.3
protein_coding	CCDC28A	ENSG00000024862.17	57	72	1.3
protein_coding	UAP1L1	ENSG00000197355.10	19	24	1.3
protein_coding	REEP4	ENSG00000168476.11	19	24	1.3
protein_coding	NPRL3	ENSG00000103148.15	19	24	1.3
protein_coding	KMT2E	ENSG00000005483.20	289	365	1.3
protein_coding	LAPTM4A	ENSG00000068697.6	4473	5649	1.3
protein_coding	MT2A	ENSG00000125148.6	894	1129	1.3
protein_coding	EIF4G3	ENSG00000075151.20	331	418	1.3
protein_coding	SZRD1	ENSG00000055070.16	156	197	1.3
protein_coding	ECPAS	ENSG00000136813.14	198	250	1.3
protein_coding	POLK	ENSG00000122008.15	80	101	1.3
protein_coding	DDX24	ENSG00000089737.16	417	526	1.3
protein_coding	RSL24D1	ENSG00000137876.9	207	261	1.3
protein_coding	PDS5A	ENSG00000121892.14	161	203	1.3
protein_coding	ZNF689	ENSG00000156853.12	23	29	1.3
protein_coding	BLOC1S5	ENSG00000188428.19	23	29	1.3
protein_coding	LMBR1L	ENSG00000139636.15	23	29	1.3
protein_coding	ZNF227	ENSG00000131115.15	23	29	1.3
protein_coding	LITAF	ENSG00000189067.12	1175	1481	1.3
protein_coding	NBN	ENSG00000104320.13	96	121	1.3
protein_coding	ALDH6A1	ENSG00000119711.12	96	121	1.3
protein_coding	PIK3C3	ENSG00000078142.12	73	92	1.3
protein_coding	FAM210B	ENSG00000124098.9	538	678	1.3
protein_coding	MRPS35	ENSG00000061794.12	127	160	1.3
protein_coding	NCL	ENSG00000115053.15	693	873	1.3
protein_coding	NREP	ENSG00000134986.13	77	97	1.3
protein_coding	FAM160B1	ENSG00000151553.14	54	68	1.3

protein_coding	CALHM5	ENSG00000178033.5	54	68	1.3
protein_coding	DENND1A	ENSG00000119522.16	27	34	1.3
protein_coding	TMOD2	ENSG00000128872.9	27	34	1.3
protein_coding	CD46	ENSG00000117335.19	386	486	1.3
protein_coding	RBBP4	ENSG00000162521.18	112	141	1.3
protein_coding	ZNRF1	ENSG00000186187.11	62	78	1.3
protein_coding	IFRD1	ENSG00000006652.13	62	78	1.3
protein_coding	TMEM30A	ENSG00000112697.15	752	946	1.3
protein_coding	CPSF7	ENSG00000149532.15	66	83	1.3
protein_coding	DLG4	ENSG00000132535.19	101	127	1.3
protein_coding	NLGN2	ENSG00000169992.9	101	127	1.3
protein_coding	SCAMP1	ENSG00000085365.17	206	259	1.3
protein_coding	ARIH1	ENSG00000166233.14	175	220	1.3
protein_coding	FAM214B	ENSG00000005238.19	105	132	1.3
protein_coding	COG8	ENSG00000213380.15	35	44	1.3
processed_pseudogene	RPL3P4	ENSG00000232573.1	35	44	1.3
protein_coding	DDX3X	ENSG00000215301.10	619	778	1.3
protein_coding	TRIP12	ENSG00000153827.13	335	421	1.3
protein_coding	PPIL4	ENSG00000131013.3	113	142	1.3
protein_coding	HACD1	ENSG00000165996.13	39	49	1.3
protein_coding	HELZ2	ENSG00000130589.16	39	49	1.3
protein_coding	MVB12A	ENSG00000141971.12	39	49	1.3
protein_coding	HOOK3	ENSG00000168172.8	394	495	1.3
protein_coding	MCMBP	ENSG00000197771.12	43	54	1.3
protein_coding	FAM49B	ENSG00000153310.19	43	54	1.3
protein_coding	LYRM1	ENSG00000102897.9	43	54	1.3
protein_coding	CTSF	ENSG00000174080.10	43	54	1.3
protein_coding	RNF114	ENSG00000124226.10	227	285	1.3
protein_coding	USP14	ENSG00000101557.14	184	231	1.3
protein_coding	SCNM1	ENSG00000163156.11	47	59	1.3
protein_coding	MSC	ENSG00000178860.8	47	59	1.3
protein_coding	SNAP29	ENSG00000099940.11	98	123	1.3
protein_coding	C3orf58	ENSG00000181744.8	102	128	1.3
protein_coding	PXDN	ENSG00000130508.10	1227	1539	1.3
protein_coding	TMCO1	ENSG00000143183.16	354	444	1.3
protein_coding	UXS1	ENSG00000115652.14	118	148	1.3
protein_coding	PHLDB2	ENSG00000144824.20	67	84	1.3
protein_coding	UGGT1	ENSG00000136731.12	142	178	1.3
protein_coding	C19orf70	ENSG00000174917.8	71	89	1.3
protein_coding	CTBP2	ENSG00000175029.16	249	312	1.3
protein_coding	TCEAL3	ENSG00000196507.10	198	248	1.3
protein_coding	ZNHIT1	ENSG00000106400.11	99	124	1.3
protein_coding	MXI1	ENSG00000119950.20	123	154	1.3
protein_coding	DAZAP2	ENSG00000183283.15	362	453	1.3

protein_coding	KIAA0930	ENSG00000100364.18	195	244	1.3
protein_coding	EIF5B	ENSG00000158417.10	849	1062	1.3
protein_coding	HECTD1	ENSG00000092148.12	220	275	1.3
protein_coding	DCBLD2	ENSG00000057019.15	144	180	1.3
protein_coding	SEZ6L2	ENSG00000174938.14	132	165	1.3
protein_coding	SCCPDH	ENSG00000143653.9	120	150	1.3
protein_coding	NDEL1	ENSG00000166579.15	88	110	1.3
protein_coding	MRPL16	ENSG00000166902.4	88	110	1.3
protein_coding	CISD2	ENSG00000145354.11	72	90	1.3
protein_coding	DDX52	ENSG00000278053.4	64	80	1.3
protein_coding	MTMR9	ENSG00000104643.9	60	75	1.3
protein_coding	CCDC117	ENSG00000159873.9	56	70	1.3
protein_coding	ACBD5	ENSG00000107897.18	48	60	1.3
protein_coding	SLC35C1	ENSG00000181830.8	48	60	1.3
protein_coding	CEP89	ENSG00000121289.17	48	60	1.3
protein_coding	RNF111	ENSG00000157450.15	44	55	1.3
protein_coding	ARHGEF11	ENSG00000132694.18	44	55	1.3
protein_coding	DUSP11	ENSG00000144048.10	40	50	1.3
protein_coding	GPR180	ENSG00000152749.7	36	45	1.3
protein_coding	B4GALT4	ENSG00000121578.12	36	45	1.3
protein_coding	CEP97	ENSG00000182504.10	32	40	1.3
protein_coding	HEATR1	ENSG00000119285.10	32	40	1.3
protein_coding	CAPN7	ENSG00000131375.9	32	40	1.3
protein_coding	ERG28	ENSG00000133935.6	32	40	1.3
protein_coding	CHUK	ENSG00000213341.10	28	35	1.3
protein_coding	CPT2	ENSG00000157184.6	28	35	1.3
protein_coding	PLPP7	ENSG00000160539.5	28	35	1.3
protein_coding	AP4E1	ENSG00000081014.10	24	30	1.3
protein_coding	PRDM1	ENSG00000057657.16	20	25	1.3
protein_coding	KNSTRN	ENSG00000128944.13	20	25	1.3
protein_coding	MAP4K2	ENSG00000168067.11	20	25	1.3
protein_coding	FMN1	ENSG00000248905.8	16	20	1.3
protein_coding	NAF1	ENSG00000145414.8	16	20	1.3
protein_coding	IRAK3	ENSG00000090376.10	16	20	1.3
protein_coding	FAM104B	ENSG00000182518.13	16	20	1.3
lincRNA	AC104794.2	ENSG00000260077.1	16	20	1.3
protein_coding	NEDD9	ENSG00000111859.16	12	15	1.3
protein_coding	ZBTB7B	ENSG00000160685.13	12	15	1.3
protein_coding	STARD10	ENSG00000214530.9	12	15	1.3
protein_coding	PLA2R1	ENSG00000153246.12	12	15	1.3
protein_coding	RFX3	ENSG00000080298.15	12	15	1.3
protein_coding	MTMR14	ENSG00000163719.19	12	15	1.3
protein_coding	MCAT	ENSG00000100294.12	12	15	1.3
protein_coding	NHLRC1	ENSG00000187566.5	12	15	1.3

protein_coding	MTHFD2L	ENSG00000163738.18	8	10	1.3
protein_coding	DMPK	ENSG00000104936.17	8	10	1.3
protein_coding	ZNHIT2	ENSG00000174276.6	8	10	1.3
protein_coding	RCL1	ENSG00000120158.11	8	10	1.3
protein_coding	TRNP1	ENSG00000253368.3	8	10	1.3
protein_coding	ZNF790	ENSG00000197863.8	8	10	1.3
protein_coding	TMEM186	ENSG00000184857.7	8	10	1.3
protein_coding	ZNF599	ENSG00000153896.17	8	10	1.3
protein_coding	ZSWIM3	ENSG00000132801.6	8	10	1.3
protein_coding	FKBP11	ENSG00000134285.10	8	10	1.3
protein_coding	ENO3	ENSG00000108515.17	8	10	1.3
protein_coding	DDX31	ENSG00000125485.17	8	10	1.3
protein_coding	LRRC32	ENSG00000137507.11	8	10	1.3
processed_transcript	ZFHX2-AS1	ENSG00000157306.14	4	5	1.3
protein_coding	CDC14A	ENSG00000079335.19	4	5	1.3
transcribed_unprocessed_pseudogene	HERC2P9	ENSG00000206149.10	4	5	1.3
protein_coding	KIAA0825	ENSG00000185261.14	4	5	1.3
protein_coding	LMNB1	ENSG00000113368.11	4	5	1.3
processed_pseudogene	RPS15AP12	ENSG00000232134.1	4	5	1.3
protein_coding	KLHL17	ENSG00000187961.13	4	5	1.3
TEC	AC120114.3	ENSG00000279789.1	4	5	1.3
lincRNA	NBAT1	ENSG00000260455.1	4	5	1.3
protein_coding	FAR2	ENSG00000064763.10	4	5	1.3
antisense	AC004148.2	ENSG00000263272.1	4	5	1.3
antisense	AC021092.1	ENSG00000186019.10	4	5	1.3
protein_coding	ZFAND4	ENSG00000172671.19	4	5	1.3
antisense	AC109454.2	ENSG00000248559.1	4	5	1.3
protein_coding	CCDC96	ENSG00000173013.5	4	5	1.3
protein_coding	ERI2	ENSG00000196678.13	4	5	1.3
protein_coding	MYOM2	ENSG00000036448.9	4	5	1.3
lincRNA	DIRC3	ENSG00000231672.6	4	5	1.3
bidirectional_promoter_lncRNA	AC112220.2	ENSG00000271643.2	4	5	1.3
protein_coding	GLYCTK	ENSG00000168237.17	4	5	1.3
protein_coding	ELOVL2	ENSG00000197977.3	4	5	1.3
antisense	INTS6-AS1	ENSG00000236778.7	4	5	1.3
protein_coding	SLC45A1	ENSG00000162426.14	4	5	1.3
protein_coding	QRICH2	ENSG00000129646.14	4	5	1.3
protein_coding	SMN1	ENSG00000172062.16	4	5	1.3
sense_intronic	AC019080.5	ENSG00000280374.1	4	5	1.3
protein_coding	STARD5	ENSG00000172345.13	4	5	1.3
protein_coding	RAB3GAP1	ENSG00000115839.17	205	256	1.2

protein_coding	MMADHC	ENSG00000168288.12	386	482	1.2
protein_coding	DUSP3	ENSG00000108861.8	125	156	1.2
protein_coding	MOB4	ENSG00000115540.14	109	136	1.2
protein_coding	STRN3	ENSG00000196792.11	97	121	1.2
protein_coding	TMEM179B	ENSG00000185475.10	97	121	1.2
protein_coding	STXBP1	ENSG00000136854.20	93	116	1.2
protein_coding	FAM3C	ENSG00000196937.10	69	86	1.2
protein_coding	ATMIN	ENSG00000166454.9	134	167	1.2
protein_coding	TNFAIP1	ENSG00000109079.9	130	162	1.2
protein_coding	RBM18	ENSG00000119446.13	65	81	1.2
protein_coding	KIAA1549	ENSG00000122778.9	61	76	1.2
protein_coding	TMEM115	ENSG00000126062.3	57	71	1.2
protein_coding	EIF2AK1	ENSG00000086232.12	155	193	1.2
protein_coding	HNRNPH1	ENSG00000169045.17	306	381	1.2
protein_coding	MYO9A	ENSG00000066933.15	98	122	1.2
protein_coding	ASNA1	ENSG00000198356.11	49	61	1.2
protein_coding	ATG5	ENSG00000057663.15	49	61	1.2
protein_coding	SPATA20	ENSG00000006282.20	49	61	1.2
protein_coding	NT5E	ENSG00000135318.11	846	1053	1.2
protein_coding	ERMN	ENSG00000136541.14	45	56	1.2
protein_coding	TCEAL4	ENSG00000133142.17	262	326	1.2
protein_coding	PABPC1	ENSG00000070756.15	2693	3350	1.2
protein_coding	POC1B	ENSG00000139323.13	41	51	1.2
protein_coding	UTP23	ENSG00000147679.11	41	51	1.2
protein_coding	HECTD4	ENSG00000173064.12	41	51	1.2
protein_coding	EDEM3	ENSG00000116406.18	115	143	1.2
protein_coding	MARF1	ENSG00000166783.21	74	92	1.2
protein_coding	PHTF1	ENSG00000116793.15	37	46	1.2
protein_coding	CLK3	ENSG00000179335.18	37	46	1.2
protein_coding	MYO10	ENSG00000145555.14	177	220	1.2
protein_coding	CIR1	ENSG00000138433.15	272	338	1.2
protein_coding	EMC3	ENSG00000125037.12	202	251	1.2
protein_coding	BLOC1S4	ENSG00000186222.4	66	82	1.2
protein_coding	PLPPR2	ENSG00000105520.10	33	41	1.2
protein_coding	DNAL4	ENSG00000100246.12	33	41	1.2
protein_coding	MMP1	ENSG00000196611.4	355	441	1.2
protein_coding	SIN3A	ENSG00000169375.15	62	77	1.2
protein_coding	CASP3	ENSG00000164305.18	62	77	1.2
protein_coding	AGPS	ENSG00000018510.14	62	77	1.2
protein_coding	PAF1	ENSG00000006712.14	62	77	1.2
protein_coding	P3H3	ENSG00000110811.19	91	113	1.2
protein_coding	ERO1A	ENSG00000197930.12	178	221	1.2
protein_coding	CAT	ENSG00000121691.5	439	545	1.2
protein_coding	USP7	ENSG00000187555.14	145	180	1.2

protein_coding	FBXW7	ENSG00000109670.14	87	108	1.2
protein_coding	GAS2L1	ENSG00000185340.15	58	72	1.2
protein_coding	TMEM63A	ENSG00000196187.11	29	36	1.2
protein_coding	MED6	ENSG00000133997.11	29	36	1.2
protein_coding	PISD	ENSG00000241878.11	29	36	1.2
protein_coding	ZNF75D	ENSG00000186376.14	29	36	1.2
protein_coding	MKNK1	ENSG00000079277.20	29	36	1.2
protein_coding	DDX19B	ENSG00000157349.16	29	36	1.2
protein_coding	CTNS	ENSG00000040531.14	29	36	1.2
protein_coding	TUBB6	ENSG00000176014.12	266	330	1.2
protein_coding	CASC3	ENSG00000108349.16	133	165	1.2
protein_coding	TNIP1	ENSG00000145901.15	79	98	1.2
protein_coding	BICC1	ENSG00000122870.11	183	227	1.2
protein_coding	SH3BP5L	ENSG00000175137.10	50	62	1.2
protein_coding	ATF1	ENSG00000123268.8	50	62	1.2
protein_coding	AXIN1	ENSG00000103126.14	25	31	1.2
transcribed_unitary_pseudogene	TP73-AS1	ENSG00000227372.12	25	31	1.2
transcribed_unprocessed_pseudogene	LINC00475	ENSG00000225511.7	25	31	1.2
protein_coding	ERGIC1	ENSG00000113719.15	421	522	1.2
protein_coding	SLC35B1	ENSG00000121073.14	96	119	1.2
protein_coding	RSL1D1	ENSG00000171490.12	142	176	1.2
protein_coding	TRIAP1	ENSG00000170855.3	117	145	1.2
protein_coding	SEMA4C	ENSG00000168758.10	46	57	1.2
protein_coding	MAZ	ENSG00000103495.13	46	57	1.2
protein_coding	MRE11	ENSG00000020922.12	46	57	1.2
protein_coding	SF3B5	ENSG00000169976.6	243	301	1.2
protein_coding	HYOU1	ENSG00000149428.18	88	109	1.2
protein_coding	TMEM123	ENSG00000152558.14	348	431	1.2
protein_coding	STK4	ENSG00000101109.11	214	265	1.2
protein_coding	SSR1	ENSG00000124783.13	462	572	1.2
protein_coding	XG	ENSG00000124343.13	168	208	1.2
protein_coding	STXBP3	ENSG00000116266.10	63	78	1.2
protein_coding	C20orf27	ENSG00000101220.17	21	26	1.2
protein_coding	NGRN	ENSG00000182768.8	21	26	1.2
protein_coding	OAZ2	ENSG00000180304.14	122	151	1.2
protein_coding	YWHAZ	ENSG00000164924.17	944	1168	1.2
protein_coding	TOR1A	ENSG00000136827.11	114	141	1.2
protein_coding	RALGDS	ENSG00000160271.15	38	47	1.2
protein_coding	NELFA	ENSG00000185049.14	38	47	1.2
protein_coding	HTRA2	ENSG00000115317.11	38	47	1.2
protein_coding	SMU1	ENSG00000122692.8	55	68	1.2

protein_coding	ATN1	ENSG00000111676.14	127	157	1.2
protein_coding	CREB1	ENSG00000118260.14	72	89	1.2
protein_coding	SETD1B	ENSG00000139718.10	89	110	1.2
protein_coding	SELENOT	ENSG00000198843.12	225	278	1.2
protein_coding	C6orf62	ENSG00000112308.12	289	357	1.2
protein_coding	RNF7	ENSG00000114125.13	221	273	1.2
protein_coding	NDUFS1	ENSG00000023228.13	187	231	1.2
protein_coding	CYLD	ENSG00000083799.17	136	168	1.2
protein_coding	BCAP31	ENSG00000185825.16	136	168	1.2
protein_coding	TSNAX	ENSG00000116918.13	85	105	1.2
protein_coding	DOCK7	ENSG00000116641.17	68	84	1.2
protein_coding	NUDT2	ENSG00000164978.17	51	63	1.2
protein_coding	ZNF274	ENSG00000171606.17	34	42	1.2
protein_coding	SLC43A2	ENSG00000167703.14	34	42	1.2
protein_coding	GZF1	ENSG00000125812.15	17	21	1.2
processed_pseudogene	TTC3P1	ENSG00000215105.4	17	21	1.2
protein_coding	NPEPL1	ENSG00000215440.11	17	21	1.2
protein_coding	TMEM53	ENSG00000126106.13	17	21	1.2
protein_coding	UBE2G2	ENSG00000184787.18	98	121	1.2
protein_coding	NFX1	ENSG00000086102.18	81	100	1.2
protein_coding	PYCARD	ENSG00000103490.13	64	79	1.2
protein_coding	SERINC3	ENSG00000132824.13	973	1201	1.2
bidirectional_promoter_lncRNA	TUG1	ENSG00000253352.9	235	290	1.2
protein_coding	SDAD1	ENSG00000198301.11	94	116	1.2
protein_coding	TAP2	ENSG00000204267.13	124	153	1.2
protein_coding	JARID2	ENSG00000008083.13	60	74	1.2
protein_coding	NUP54	ENSG00000138750.14	30	37	1.2
protein_coding	C11orf74	ENSG00000166352.15	30	37	1.2
protein_coding	BCR	ENSG00000186716.20	30	37	1.2
protein_coding	EID1	ENSG00000255302.4	1078	1329	1.2
protein_coding	LAYN	ENSG00000204381.11	189	233	1.2
protein_coding	MRPL9	ENSG00000143436.10	86	106	1.2
protein_coding	IGF2BP2	ENSG00000073792.15	112	138	1.2
protein_coding	ARFGAP1	ENSG00000101199.12	56	69	1.2
protein_coding	IKBIP	ENSG00000166130.14	345	425	1.2
protein_coding	TNKS2	ENSG00000107854.5	82	101	1.2
protein_coding	TWF1	ENSG00000151239.13	82	101	1.2
protein_coding	R3HDM1	ENSG00000048991.16	95	117	1.2
protein_coding	TMEM184C	ENSG00000164168.7	95	117	1.2
protein_coding	ATL3	ENSG00000184743.12	372	458	1.2
protein_coding	PJA2	ENSG00000198961.9	225	277	1.2
protein_coding	STK16	ENSG00000115661.13	78	96	1.2

protein_coding	BMP2K	ENSG00000138756.17	52	64	1.2
protein_coding	RRP1B	ENSG00000160208.12	39	48	1.2
protein_coding	CCDC130	ENSG00000104957.13	26	32	1.2
protein_coding	CDCP1	ENSG00000163814.7	13	16	1.2
protein_coding	ZBTB21	ENSG00000173276.13	13	16	1.2
protein_coding	ENGASE	ENSG00000167280.16	13	16	1.2
protein_coding	IPO4	ENSG00000196497.16	13	16	1.2
protein_coding	METTL2B	ENSG00000165055.15	13	16	1.2
protein_coding	MBTD1	ENSG00000011258.15	13	16	1.2
protein_coding	SLC2A6	ENSG00000160326.13	13	16	1.2
protein_coding	PCBP3	ENSG00000183570.16	13	16	1.2
protein_coding	CSDE1	ENSG00000009307.15	1753	2157	1.2
protein_coding	PPP2R5E	ENSG00000154001.13	304	374	1.2
protein_coding	MTMR2	ENSG00000087053.18	74	91	1.2
protein_coding	TMEM9	ENSG00000116857.16	135	166	1.2
protein_coding	PRRC1	ENSG00000164244.20	196	241	1.2
protein_coding	NIFK	ENSG00000155438.11	61	75	1.2
protein_coding	FAM120A	ENSG00000048828.16	959	1179	1.2
protein_coding	ATP6V1D	ENSG00000100554.11	109	134	1.2
protein_coding	SH3BP4	ENSG00000130147.15	109	134	1.2
protein_coding	SNAPC3	ENSG00000164975.15	157	193	1.2
protein_coding	SMARCA1	ENSG00000102038.15	271	333	1.2
protein_coding	CES2	ENSG00000172831.11	140	172	1.2
protein_coding	ASNSD1	ENSG00000138381.9	105	129	1.2
protein_coding	ERBB2	ENSG00000141736.13	35	43	1.2
protein_coding	PER1	ENSG00000179094.15	35	43	1.2
protein_coding	DDX20	ENSG00000064703.11	35	43	1.2
protein_coding	HNRNPAB	ENSG00000197451.11	171	210	1.2
protein_coding	ETV1	ENSG00000006468.13	79	97	1.2
protein_coding	TSPYL4	ENSG00000187189.10	66	81	1.2
protein_coding	TMEM161B	ENSG00000164180.13	22	27	1.2
protein_coding	NUP107	ENSG00000111581.9	22	27	1.2
protein_coding	IFT88	ENSG00000032742.17	22	27	1.2
protein_coding	CEP57L1	ENSG00000183137.14	22	27	1.2
protein_coding	SHPRH	ENSG00000146414.15	22	27	1.2
lincRNA	AC093297.2	ENSG00000272335.1	22	27	1.2
protein_coding	ZNF431	ENSG00000196705.8	22	27	1.2
protein_coding	USP34	ENSG00000115464.14	339	416	1.2
protein_coding	PHKB	ENSG00000102893.15	75	92	1.2
protein_coding	PIAS2	ENSG00000078043.15	53	65	1.2
protein_coding	PTPN2	ENSG00000175354.19	53	65	1.2
protein_coding	ANO10	ENSG00000160746.12	186	228	1.2
protein_coding	GNPTG	ENSG00000090581.9	155	190	1.2
protein_coding	SAFB	ENSG00000160633.12	62	76	1.2

protein_coding	NEDD1	ENSG00000139350.11	31	38	1.2
protein_coding	TRAF3	ENSG00000131323.14	31	38	1.2
protein_coding	ORAI2	ENSG00000160991.15	102	125	1.2
protein_coding	CTGF	ENSG00000118523.5	275	337	1.2
protein_coding	GLRX2	ENSG00000023572.9	40	49	1.2
protein_coding	RAP2A	ENSG00000125249.6	89	109	1.2
protein_coding	ENG	ENSG00000106991.13	187	229	1.2
protein_coding	MAP1A	ENSG00000166963.12	1079	1321	1.2
protein_coding	USP48	ENSG00000090686.15	201	246	1.2
protein_coding	ATXN2L	ENSG00000168488.18	134	164	1.2
protein_coding	NUDT22	ENSG00000149761.8	67	82	1.2
protein_coding	IER2	ENSG00000160888.6	67	82	1.2
protein_coding	UNC93B1	ENSG00000110057.7	67	82	1.2
protein_coding	INF2	ENSG00000203485.12	85	104	1.2
protein_coding	KIF5B	ENSG00000170759.10	604	739	1.2
protein_coding	NDFIP1	ENSG00000131507.10	336	411	1.2
protein_coding	ACYP1	ENSG00000119640.8	112	137	1.2
protein_coding	SYNCRIP	ENSG00000135316.17	354	433	1.2
protein_coding	IST1	ENSG00000182149.20	139	170	1.2
protein_coding	KIDINS220	ENSG00000134313.15	238	291	1.2
protein_coding	RAB3B	ENSG00000169213.6	495	605	1.2
protein_coding	LRCH3	ENSG00000186001.13	72	88	1.2
protein_coding	KMT5B	ENSG00000110066.14	72	88	1.2
protein_coding	POLR3GL	ENSG00000121851.12	72	88	1.2
protein_coding	FBXO38	ENSG00000145868.16	54	66	1.2
protein_coding	SPOUT1	ENSG00000198917.12	45	55	1.2
protein_coding	NXF1	ENSG00000162231.13	45	55	1.2
protein_coding	C8orf76	ENSG00000189376.11	36	44	1.2
protein_coding	NFRKB	ENSG00000170322.14	27	33	1.2
protein_coding	TMEM106C	ENSG00000134291.11	27	33	1.2
protein_coding	ISCA2	ENSG00000165898.13	27	33	1.2
protein_coding	ZNF787	ENSG00000142409.5	18	22	1.2
transcribed_processed_pseudogene	RPS18P9	ENSG00000220848.5	18	22	1.2
protein_coding	ZDHHC13	ENSG00000177054.13	18	22	1.2
protein_coding	ZNF335	ENSG00000198026.7	18	22	1.2
protein_coding	MMP3	ENSG00000149968.11	18	22	1.2
lincRNA	MIR210HG	ENSG00000247095.2	9	11	1.2
protein_coding	CCDC170	ENSG00000120262.9	9	11	1.2
protein_coding	CAMKMT	ENSG00000143919.14	9	11	1.2
protein_coding	KCTD11	ENSG00000213859.5	9	11	1.2
antisense	GSEC	ENSG00000280832.1	9	11	1.2
protein_coding	ZNF280C	ENSG00000056277.15	9	11	1.2

protein_coding	TUBB2B	ENSG00000137285.9	9	11	1.2
protein_coding	NUDT6	ENSG00000170917.13	9	11	1.2
lincRNA	DUBR	ENSG00000243701.5	9	11	1.2
protein_coding	ZNF135	ENSG00000176293.19	9	11	1.2
protein_coding	PDGFD	ENSG00000170962.12	9	11	1.2
protein_coding	ERLEC1	ENSG00000068912.13	204	249	1.2
protein_coding	YAF2	ENSG00000015153.14	59	72	1.2
protein_coding	TSC22D3	ENSG00000157514.16	59	72	1.2
protein_coding	MAP7D1	ENSG00000116871.15	209	255	1.2
protein_coding	KLHDC2	ENSG00000165516.10	100	122	1.2
protein_coding	CRYZL1	ENSG00000205758.11	50	61	1.2
protein_coding	RMC1	ENSG00000141452.9	41	50	1.2
protein_coding	TOM1	ENSG00000100284.20	41	50	1.2
protein_coding	CPT1A	ENSG00000110090.12	319	389	1.2
protein_coding	SLC16A1	ENSG00000155380.11	228	278	1.2
protein_coding	RCN1	ENSG00000049449.9	288	351	1.2
protein_coding	ANKRA2	ENSG00000164331.9	64	78	1.2
protein_coding	GTF3C5	ENSG00000148308.17	32	39	1.2
protein_coding	PTOV1	ENSG00000104960.15	151	184	1.2
protein_coding	PGM1	ENSG00000079739.16	87	106	1.2
protein_coding	DHRS7	ENSG00000100612.13	275	335	1.2
protein_coding	BACE1	ENSG00000186318.16	275	335	1.2
protein_coding	ZBTB8OS	ENSG00000176261.15	55	67	1.2
protein_coding	BUD23	ENSG00000071462.11	55	67	1.2
protein_coding	PPM1B	ENSG00000138032.20	55	67	1.2
protein_coding	MT-ND4	ENSG00000198886.2	10103	12301	1.2
protein_coding	LRPAP1	ENSG00000163956.11	161	196	1.2
protein_coding	SPOCD1	ENSG00000134668.12	92	112	1.2
protein_coding	CREB3	ENSG00000107175.11	69	84	1.2
protein_coding	SLC50A1	ENSG00000169241.18	46	56	1.2
protein_coding	RXYLT1	ENSG00000118600.11	23	28	1.2
processed_transcript	SNHG16	ENSG00000163597.14	175	213	1.2
protein_coding	EFTUD2	ENSG00000108883.12	60	73	1.2
protein_coding	UBE2V2	ENSG00000169139.11	157	191	1.2
protein_coding	EIF5A	ENSG00000132507.17	171	208	1.2
protein_coding	HAUS1	ENSG00000152240.12	37	45	1.2
protein_coding	RAB8A	ENSG00000167461.11	88	107	1.2
protein_coding	EMC4	ENSG00000128463.12	408	496	1.2
protein_coding	DCUN1D5	ENSG00000137692.11	51	62	1.2
protein_coding	TFAM	ENSG00000108064.10	51	62	1.2
protein_coding	ZC3H15	ENSG00000065548.17	269	327	1.2
protein_coding	CEBPZOS	ENSG00000218739.9	116	141	1.2
protein_coding	FAM104A	ENSG00000133193.12	79	96	1.2
protein_coding	TCF25	ENSG00000141002.19	516	627	1.2

protein_coding	N4BP2L2	ENSG00000244754.8	163	198	1.2
protein_coding	YWHAQ	ENSG00000134308.13	503	611	1.2
protein_coding	ACLY	ENSG00000131473.16	340	413	1.2
protein_coding	MNAT1	ENSG00000020426.10	56	68	1.2
protein_coding	LYPLA2	ENSG00000011009.10	56	68	1.2
protein_coding	UHRF1BP1L	ENSG00000111647.12	42	51	1.2
protein_coding	WDR92	ENSG00000243667.6	28	34	1.2
protein_coding	MTMR12	ENSG00000150712.10	28	34	1.2
protein_coding	ZNF267	ENSG00000185947.14	28	34	1.2
processed_transcript	DANCR	ENSG00000226950.6	28	34	1.2
protein_coding	CAD	ENSG00000084774.13	14	17	1.2
protein_coding	ACTG2	ENSG00000163017.13	14	17	1.2
protein_coding	TIMM8A	ENSG00000126953.6	14	17	1.2
protein_coding	LIN7B	ENSG00000104863.11	14	17	1.2
protein_coding	ULBP2	ENSG00000131015.4	14	17	1.2
protein_coding	DOK1	ENSG00000115325.13	89	108	1.2
protein_coding	TRIOBP	ENSG00000100106.20	600	728	1.2
protein_coding	PPP6R2	ENSG00000100239.15	75	91	1.2
protein_coding	HERC1	ENSG00000103657.13	75	91	1.2
protein_coding	EFHD2	ENSG00000142634.12	61	74	1.2
protein_coding	APC	ENSG00000134982.16	94	114	1.2
protein_coding	TMEM200A	ENSG00000164484.11	47	57	1.2
protein_coding	ZDHHC5	ENSG00000156599.10	80	97	1.2
protein_coding	KPNA6	ENSG00000025800.13	273	331	1.2
protein_coding	THOC2	ENSG00000125676.19	339	411	1.2
protein_coding	RSRC2	ENSG00000111011.17	132	160	1.2
protein_coding	FAM122A	ENSG00000187866.8	33	40	1.2
protein_coding	SLAIN2	ENSG00000109171.14	85	103	1.2
protein_coding	CHST14	ENSG00000169105.7	85	103	1.2
protein_coding	XKR8	ENSG00000158156.7	52	63	1.2
protein_coding	IGIP	ENSG00000182700.4	52	63	1.2
protein_coding	COG1	ENSG00000166685.11	52	63	1.2
protein_coding	MSH6	ENSG00000116062.14	76	92	1.2
protein_coding	GNPAT	ENSG00000116906.12	76	92	1.2
protein_coding	XXYLT1	ENSG00000173950.15	57	69	1.2
protein_coding	GATD1	ENSG00000177225.16	38	46	1.2
protein_coding	CYP20A1	ENSG00000119004.15	38	46	1.2
protein_coding	PARP4	ENSG00000102699.5	38	46	1.2
lincRNA	FAM225B	ENSG00000225684.3	19	23	1.2
protein_coding	RRAGB	ENSG00000083750.12	19	23	1.2
protein_coding	ATAT1	ENSG00000137343.17	19	23	1.2
protein_coding	RALB	ENSG00000144118.13	100	121	1.2
protein_coding	KDM1A	ENSG00000004487.16	81	98	1.2
protein_coding	WWC3	ENSG00000047644.18	105	127	1.2

protein_coding	GCLM	ENSG00000023909.9	105	127	1.2
protein_coding	BAZ2A	ENSG00000076108.11	86	104	1.2
protein_coding	C2orf69	ENSG00000178074.5	86	104	1.2
protein_coding	MOB3A	ENSG00000172081.13	43	52	1.2
protein_coding	ANO6	ENSG00000177119.15	153	185	1.2
protein_coding	PRPSAP1	ENSG00000161542.16	67	81	1.2
protein_coding	THUMPD1	ENSG00000066654.13	115	139	1.2
protein_coding	FSTL1	ENSG00000163430.11	3020	3650	1.2
protein_coding	SCML1	ENSG00000047634.14	48	58	1.2
protein_coding	DMTF1	ENSG00000135164.18	24	29	1.2
protein_coding	GRINA	ENSG00000178719.16	510	616	1.2
protein_coding	DDX46	ENSG00000145833.15	154	186	1.2
protein_coding	RNASEH1	ENSG00000171865.9	77	93	1.2
protein_coding	BRD2	ENSG00000204256.12	284	343	1.2
protein_coding	PPP1CA	ENSG00000172531.14	130	157	1.2
protein_coding	OCIAD1	ENSG00000109180.14	574	693	1.2
protein_coding	SRSF4	ENSG00000116350.16	145	175	1.2
protein_coding	ECH1	ENSG00000104823.8	145	175	1.2
protein_coding	AGA	ENSG00000038002.8	87	105	1.2
protein_coding	ZNF260	ENSG00000254004.6	87	105	1.2
protein_coding	FAM228B	ENSG00000219626.8	29	35	1.2
protein_coding	MRPL49	ENSG00000149792.8	150	181	1.2
protein_coding	DNMT1	ENSG00000130816.15	92	111	1.2
protein_coding	SAR1B	ENSG00000152700.13	126	152	1.2
protein_coding	GPC1	ENSG00000063660.8	325	392	1.2
protein_coding	ZFP36L2	ENSG00000152518.7	699	843	1.2
protein_coding	CHMP5	ENSG00000086065.13	306	369	1.2
protein_coding	PGS1	ENSG00000087157.18	34	41	1.2
protein_coding	ZSWIM7	ENSG00000214941.7	34	41	1.2
protein_coding	ARHGEF6	ENSG00000129675.15	34	41	1.2
protein_coding	CHMP4B	ENSG00000101421.3	209	252	1.2
protein_coding	SMIM3	ENSG00000256235.2	107	129	1.2
protein_coding	GALM	ENSG00000143891.16	73	88	1.2
processed_transcript	MIR100HG	ENSG00000255248.8	307	370	1.2
protein_coding	ARL¬†10	ENSG00000175414.6	39	47	1.2
protein_coding	WASF1	ENSG00000112290.12	39	47	1.2
protein_coding	TOMM7	ENSG00000196683.10	1102	1328	1.2
protein_coding	RELA	ENSG00000173039.18	122	147	1.2
protein_coding	GPC6	ENSG00000183098.10	44	53	1.2
protein_coding	PIGX	ENSG00000163964.14	54	65	1.2
protein_coding	FLOT2	ENSG00000132589.15	54	65	1.2
protein_coding	FECH	ENSG00000066926.10	54	65	1.2
protein_coding	ARL¬†3	ENSG00000138175.8	172	207	1.2
protein_coding	TMEM208	ENSG00000168701.18	59	71	1.2

protein_coding	TMEM14B	ENSG00000137210.13	59	71	1.2
protein_coding	SCOC	ENSG00000153130.17	197	237	1.2
lincRNA	XIST	ENSG00000229807.11	404	486	1.2
protein_coding	FKBP14	ENSG00000106080.10	143	172	1.2
protein_coding	CSF1	ENSG00000184371.13	79	95	1.2
protein_coding	USP11	ENSG00000102226.9	79	95	1.2
protein_coding	DEGS1	ENSG00000143753.12	317	381	1.2
protein_coding	MARCH6	ENSG00000145495.15	322	387	1.2
protein_coding	PPDPF	ENSG00000125534.9	1180	1418	1.2
protein_coding	EXOC5	ENSG00000070367.15	129	155	1.2
protein_coding	GREM2	ENSG00000180875.4	503	604	1.2
protein_coding	YBX1	ENSG00000065978.18	728	874	1.2
protein_coding	ENSA	ENSG00000143420.18	120	144	1.2
protein_coding	MRPS10	ENSG00000048544.5	90	108	1.2
protein_coding	GTF2E2	ENSG00000197265.8	75	90	1.2
protein_coding	TLE4	ENSG00000106829.18	60	72	1.2
protein_coding	FMR1	ENSG00000102081.14	60	72	1.2
protein_coding	TXNDC11	ENSG00000153066.12	50	60	1.2
protein_coding	C9orf16	ENSG00000171159.4	50	60	1.2
protein_coding	TRMT1L	ENSG00000121486.11	45	54	1.2
protein_coding	MAFF	ENSG00000185022.11	40	48	1.2
protein_coding	LACC1	ENSG00000179630.10	35	42	1.2
protein_coding	GTPBP1	ENSG00000100226.15	30	36	1.2
protein_coding	CCDC66	ENSG00000180376.16	30	36	1.2
TEC	AC093535.2	ENSG00000279118.1	20	24	1.2
protein_coding	SELPLG	ENSG00000110876.9	20	24	1.2
protein_coding	RCHY1	ENSG00000163743.13	15	18	1.2
protein_coding	TRMT10A	ENSG00000145331.13	15	18	1.2
protein_coding	GAREM2	ENSG00000157833.12	15	18	1.2
protein_coding	EDC3	ENSG00000179151.12	15	18	1.2
protein_coding	SNRNP25	ENSG00000161981.10	15	18	1.2
protein_coding	DECR2	ENSG00000242612.6	15	18	1.2
protein_coding	SERP2	ENSG00000151778.10	15	18	1.2
protein_coding	BHMT2	ENSG00000132840.9	15	18	1.2
protein_coding	SETMAR	ENSG00000170364.12	15	18	1.2
protein_coding	KAT2A	ENSG00000108773.10	10	12	1.2
protein_coding	HIST1H1E	ENSG00000168298.6	10	12	1.2
protein_coding	CAPN10	ENSG00000142330.19	10	12	1.2
protein_coding	ANGEL1	ENSG00000013523.9	10	12	1.2
protein_coding	TKFC	ENSG00000149476.15	10	12	1.2
protein_coding	ZNF583	ENSG00000198440.9	10	12	1.2
processed_pseudogene	RPS2P35	ENSG00000238172.2	10	12	1.2
protein_coding	STK11IP	ENSG00000144589.21	10	12	1.2
protein_coding	MEF2C	ENSG00000081189.15	10	12	1.2

protein_coding	MTM1	ENSG00000171100.14	10	12	1.2
protein_coding	MAOA	ENSG00000189221.9	10	12	1.2
protein_coding	CHST10	ENSG00000115526.10	10	12	1.2
protein_coding	ZNF567	ENSG00000189042.13	10	12	1.2
protein_coding	KCNN4	ENSG00000104783.12	5	6	1.2
lincRNA	LINC00960	ENSG00000242516.1	5	6	1.2
protein_coding	ADCK5	ENSG00000173137.11	5	6	1.2
protein_coding	CABYR	ENSG00000154040.20	5	6	1.2
protein_coding	MMP16	ENSG00000156103.15	5	6	1.2
lincRNA	LINC-PINT	ENSG00000226380.9	5	6	1.2
protein_coding	DHRS12	ENSG00000102796.10	5	6	1.2
bidirectional_promoter_lncRNA	NBR2	ENSG00000198496.11	5	6	1.2
protein_coding	NAPB	ENSG00000125814.17	5	6	1.2
protein_coding	TMEM198	ENSG00000188760.10	5	6	1.2
protein_coding	C2CD6	ENSG00000155754.14	5	6	1.2
protein_coding	L3MBTL1	ENSG00000185513.16	5	6	1.2
TEC	AC126474.1	ENSG00000279148.1	5	6	1.2
protein_coding	RTP4	ENSG00000136514.2	5	6	1.2
lincRNA	LINC00957	ENSG00000235314.1	5	6	1.2
protein_coding	FRZB	ENSG00000162998.4	5	6	1.2
protein_coding	PAQR8	ENSG00000170915.8	5	6	1.2
protein_coding	C11orf71	ENSG00000180425.11	5	6	1.2
antisense	AL138762.1	ENSG00000272572.1	5	6	1.2
protein_coding	ITGAV	ENSG00000138448.11	467	560	1.2
protein_coding	KDSR	ENSG00000119537.17	181	217	1.2
protein_coding	ANTXR2	ENSG00000163297.16	242	290	1.2
protein_coding	MCTS1	ENSG00000232119.7	91	109	1.2
protein_coding	HSDL2	ENSG00000119471.14	91	109	1.2
protein_coding	BAG5	ENSG00000166170.9	76	91	1.2
protein_coding	DNAJC10	ENSG00000077232.17	218	261	1.2
protein_coding	ARHGAP5	ENSG00000100852.12	137	164	1.2
protein_coding	RAB1A	ENSG00000138069.17	560	670	1.2
protein_coding	ZER1	ENSG00000160445.10	56	67	1.2
antisense	AC099786.1	ENSG00000224127.1	56	67	1.2
protein_coding	DYNC1I2	ENSG00000077380.15	321	384	1.2
protein_coding	TMEM181	ENSG00000146433.8	153	183	1.2
protein_coding	NAPG	ENSG00000134265.12	51	61	1.2
protein_coding	COP¬†1	ENSG00000143207.20	51	61	1.2
protein_coding	DAP	ENSG00000112977.15	327	391	1.2
protein_coding	TAF1	ENSG00000147133.15	92	110	1.2
protein_coding	EYA3	ENSG00000158161.15	46	55	1.2
protein_coding	DDX10	ENSG00000178105.10	46	55	1.2

protein_coding	TMED9	ENSG00000184840.11	778	930	1.2
protein_coding	SLC35E2B	ENSG00000189339.11	128	153	1.2
protein_coding	ARNT	ENSG00000143437.20	82	98	1.2
protein_coding	ELK1	ENSG00000126767.17	41	49	1.2
protein_coding	UBE2D2	ENSG00000131508.15	236	282	1.2
protein_coding	MRPL42	ENSG00000198015.12	118	141	1.2
protein_coding	RALA	ENSG00000006451.7	272	325	1.2
protein_coding	CARD19	ENSG00000165233.17	113	135	1.2
protein_coding	STAU1	ENSG00000124214.19	370	442	1.2
protein_coding	TAF3	ENSG00000165632.7	72	86	1.2
protein_coding	USP46	ENSG00000109189.12	72	86	1.2
protein_coding	BVES	ENSG00000112276.13	36	43	1.2
protein_coding	MUS81	ENSG00000172732.11	36	43	1.2
protein_coding	RFFL	ENSG00000092871.16	36	43	1.2
protein_coding	HIPK2	ENSG00000064393.15	407	486	1.2
protein_coding	MBD5	ENSG00000204406.13	67	80	1.2
protein_coding	SOCS1	ENSG00000185338.5	31	37	1.2
protein_coding	NIP7	ENSG00000132603.14	31	37	1.2
protein_coding	SZT2	ENSG00000198198.16	31	37	1.2
protein_coding	GIT1	ENSG00000108262.15	31	37	1.2
protein_coding	DOLK	ENSG00000175283.7	31	37	1.2
protein_coding	KIAA1143	ENSG00000163807.5	150	179	1.2
protein_coding	REEP3	ENSG00000165476.13	264	315	1.2
protein_coding	Sep-10	ENSG00000186522.14	176	210	1.2
protein_coding	PLEKHM2	ENSG00000116786.12	88	105	1.2
protein_coding	PBXIP1	ENSG00000163346.16	145	173	1.2
protein_coding	NUCKS1	ENSG00000069275.12	927	1106	1.2
protein_coding	GNPTAB	ENSG00000111670.15	83	99	1.2
protein_coding	DDOST	ENSG00000244038.9	52	62	1.2
protein_coding	CDC37L1	ENSG00000106993.11	52	62	1.2
protein_coding	PTRH2	ENSG00000141378.14	26	31	1.2
protein_coding	GPT2	ENSG00000166123.13	26	31	1.2
lincRNA	FAM225A	ENSG00000231528.2	26	31	1.2
protein_coding	ELP6	ENSG00000163832.15	26	31	1.2
protein_coding	TRAPPC2B	ENSG00000256060.2	26	31	1.2
protein_coding	EEF1AKMT2	ENSG00000203791.14	26	31	1.2
protein_coding	PAFAH1B1	ENSG00000007168.12	255	304	1.2
protein_coding	MRPS5	ENSG00000144029.11	99	118	1.2
protein_coding	SLC8A1	ENSG00000183023.18	47	56	1.2
protein_coding	MYSM1	ENSG00000162601.9	47	56	1.2
protein_coding	COMMD2	ENSG00000114744.8	47	56	1.2
protein_coding	KBTBD6	ENSG00000165572.7	47	56	1.2
protein_coding	CNPY2	ENSG00000257727.5	162	193	1.2
protein_coding	VTI1B	ENSG00000100568.10	277	330	1.2

protein_coding	HMGN3	ENSG00000118418.14	68	81	1.2
protein_coding	CAMLG	ENSG00000164615.4	262	312	1.2
protein_coding	RPS24	ENSG00000138326.19	3710	4417	1.2
protein_coding	UBE2J2	ENSG00000160087.20	84	100	1.2
protein_coding	NCBP3	ENSG00000074356.16	84	100	1.2
protein_coding	ANP32E	ENSG00000143401.14	63	75	1.2
protein_coding	STYX	ENSG00000198252.11	42	50	1.2
protein_coding	CACNB3	ENSG00000167535.7	42	50	1.2
protein_coding	YEATS4	ENSG00000127337.6	21	25	1.2
protein_coding	MAPK13	ENSG00000156711.16	21	25	1.2
protein_coding	EIPR1	ENSG00000032389.12	21	25	1.2
protein_coding	PLXNB1	ENSG00000164050.12	21	25	1.2
protein_coding	BDKRB2	ENSG00000168398.6	184	219	1.2
protein_coding	IRF2BP2	ENSG00000168264.10	558	664	1.2
protein_coding	PDIA6	ENSG00000143870.12	869	1034	1.2
protein_coding	FEZ2	ENSG00000171055.14	269	320	1.2
protein_coding	SORT1	ENSG00000134243.11	148	176	1.2
protein_coding	TIMM8B	ENSG00000150779.11	74	88	1.2
protein_coding	PRSS35	ENSG00000146250.6	37	44	1.2
protein_coding	CCDC107	ENSG00000159884.11	37	44	1.2
protein_coding	SNX5	ENSG00000089006.16	164	195	1.2
protein_coding	LTA4H	ENSG00000111144.9	196	233	1.2
protein_coding	DBN1	ENSG00000113758.13	159	189	1.2
protein_coding	CEP350	ENSG00000135837.15	313	372	1.2
protein_coding	RALY	ENSG00000125970.11	69	82	1.2
protein_coding	UFL1	ENSG00000014123.9	85	101	1.2
protein_coding	CLINT1	ENSG00000113282.13	186	221	1.2
protein_coding	YIF1A	ENSG00000174851.15	144	171	1.2
protein_coding	GTF2H1	ENSG00000110768.11	112	133	1.2
protein_coding	FBXO44	ENSG00000132879.13	32	38	1.2
protein_coding	TRMT1	ENSG00000104907.12	16	19	1.2
protein_coding	RAB15	ENSG00000139998.15	16	19	1.2
protein_coding	PCGF6	ENSG00000156374.15	16	19	1.2
protein_coding	DAAM2	ENSG00000146122.16	16	19	1.2
protein_coding	ZNF846	ENSG00000196605.7	16	19	1.2
protein_coding	ATP5PD	ENSG00000167863.11	459	545	1.2
protein_coding	SRP14	ENSG00000140319.10	1282	1522	1.2
protein_coding	PGF	ENSG00000119630.13	150	178	1.2
protein_coding	ROCK2	ENSG00000134318.13	268	318	1.2
protein_coding	MRPS33	ENSG00000090263.15	102	121	1.2
protein_coding	ARMC1	ENSG00000104442.9	129	153	1.2
protein_coding	UNC45A	ENSG00000140553.17	86	102	1.2
protein_coding	GDNF	ENSG00000168621.14	43	51	1.2
protein_coding	CHMP2A	ENSG00000130724.8	113	134	1.2

protein_coding	RETSAT	ENSG00000042445.13	517	613	1.2
protein_coding	BMS1	ENSG00000165733.7	81	96	1.2
protein_coding	ZFP90	ENSG00000184939.15	54	64	1.2
protein_coding	SRD5A3	ENSG00000128039.10	27	32	1.2
protein_coding	NOC2L	ENSG00000188976.10	27	32	1.2
protein_coding	ALG14	ENSG00000172339.9	27	32	1.2
protein_coding	DENND5B	ENSG00000170456.15	27	32	1.2
protein_coding	RNF13	ENSG00000082996.19	92	109	1.2
protein_coding	MAD2L2	ENSG00000116670.14	38	45	1.2
protein_coding	GTF3C1	ENSG00000077235.17	38	45	1.2
protein_coding	NRBP2	ENSG00000185189.17	38	45	1.2
protein_coding	ELOC	ENSG00000154582.16	163	193	1.2
protein_coding	PDCD6	ENSG00000249915.7	212	251	1.2
protein_coding	FOXP1	ENSG00000114861.20	261	309	1.2
protein_coding	TBC1D8B	ENSG00000133138.19	49	58	1.2
protein_coding	PRKX	ENSG00000183943.5	49	58	1.2
protein_coding	TRAPPC4	ENSG00000196655.11	109	129	1.2
protein_coding	CPQ	ENSG00000104324.15	109	129	1.2
protein_coding	FAM217B	ENSG00000196227.10	60	71	1.2
protein_coding	ESRRA	ENSG00000173153.13	60	71	1.2
protein_coding	AP3D1	ENSG00000065000.17	333	394	1.2
protein_coding	MBNL1	ENSG00000152601.17	415	491	1.2
protein_coding	BTBD6	ENSG00000184887.13	71	84	1.2
protein_coding	SLMAP	ENSG00000163681.14	82	97	1.2
protein_coding	NPR¬†2	ENSG00000159899.14	93	110	1.2
protein_coding	STAT6	ENSG00000166888.11	307	363	1.2
protein_coding	LMAN2	ENSG00000169223.14	203	240	1.2
protein_coding	NFATC4	ENSG00000100968.13	341	403	1.2
protein_coding	NRDC	ENSG00000078618.21	176	208	1.2
protein_coding	C15orf61	ENSG00000189227.5	55	65	1.2
protein_coding	HFE	ENSG00000010704.18	44	52	1.2
protein_coding	TMEM134	ENSG00000172663.8	33	39	1.2
protein_coding	RPUSD1	ENSG00000007376.7	22	26	1.2
transcribed_unprocessed_pseudogene	AC006001.3	ENSG00000229180.7	22	26	1.2
protein_coding	BTBD1	ENSG00000064726.9	22	26	1.2
protein_coding	MLKL	ENSG00000168404.12	11	13	1.2
protein_coding	LRRC8E	ENSG00000171017.10	11	13	1.2
lincRNA	ATP2B1-AS1	ENSG00000271614.1	11	13	1.2
protein_coding	PEMT	ENSG00000133027.17	11	13	1.2
protein_coding	AFAP1L1	ENSG00000157510.13	11	13	1.2
protein_coding	SFXN4	ENSG00000183605.16	11	13	1.2
protein_coding	ZNF8	ENSG00000278129.1	11	13	1.2

protein_coding	PES¬†1	ENSG00000100029.17	11	13	1.2
protein_coding	TRMT10B	ENSG00000165275.9	11	13	1.2
protein_coding	TBC1D31	ENSG00000156787.16	11	13	1.2
protein_coding	HADHA	ENSG00000084754.11	287	339	1.2
protein_coding	PLOD3	ENSG00000106397.11	116	137	1.2
protein_coding	DHX15	ENSG00000109606.12	83	98	1.2
protein_coding	UBR1	ENSG00000159459.11	72	85	1.2
protein_coding	NEDD4	ENSG00000069869.16	61	72	1.2
protein_coding	TWF2	ENSG00000247596.8	61	72	1.2
protein_coding	SON	ENSG00000159140.19	771	910	1.2
protein_coding	TXNIP	ENSG00000265972.5	805	950	1.2
protein_coding	SLC25A43	ENSG00000077713.18	50	59	1.2
protein_coding	ARSK	ENSG00000164291.16	50	59	1.2
protein_coding	DDX19A	ENSG00000168872.16	50	59	1.2
protein_coding	DENR	ENSG00000139726.10	167	197	1.2
protein_coding	HEXA	ENSG00000213614.9	117	138	1.2
protein_coding	PIK3C2A	ENSG00000011405.13	78	92	1.2
protein_coding	RINT1	ENSG00000135249.7	39	46	1.2
protein_coding	DEDD	ENSG00000158796.16	39	46	1.2
protein_coding	SERINC2	ENSG00000168528.11	385	454	1.2
protein_coding	PUF60	ENSG00000179950.13	67	79	1.2
processed_transcript	CD27-AS1	ENSG00000215039.6	67	79	1.2
protein_coding	SLC11A2	ENSG00000110911.15	95	112	1.2
protein_coding	USP22	ENSG00000124422.11	565	666	1.2
protein_coding	ARPC5	ENSG00000162704.15	207	244	1.2
protein_coding	WDR36	ENSG00000134987.11	28	33	1.2
protein_coding	QSOX2	ENSG00000165661.16	28	33	1.2
protein_coding	LMNB2	ENSG00000176619.12	28	33	1.2
protein_coding	UBXN7	ENSG00000163960.11	174	205	1.2
protein_coding	SNRPC	ENSG00000124562.9	146	172	1.2
protein_coding	CPNE3	ENSG00000085719.12	270	318	1.2
protein_coding	PARL	ENSG00000175193.13	45	53	1.2
protein_coding	USP25	ENSG00000155313.15	45	53	1.2
protein_coding	SEC16A	ENSG00000148396.18	107	126	1.2
protein_coding	ZNF277	ENSG00000198839.9	62	73	1.2
protein_coding	PPP2CA	ENSG00000113575.9	147	173	1.2
protein_coding	XPO1	ENSG00000082898.16	198	233	1.2
protein_coding	MCFD2	ENSG00000180398.12	515	606	1.2
protein_coding	EIF2B4	ENSG00000115211.15	51	60	1.2
protein_coding	RUNDC1	ENSG00000198863.7	34	40	1.2
protein_coding	PEX11A	ENSG00000166821.8	34	40	1.2
protein_coding	SCG5	ENSG00000166922.8	17	20	1.2
protein_coding	S100A13	ENSG00000189171.14	403	474	1.2
protein_coding	BTBD7	ENSG00000011114.14	125	147	1.2

protein_coding	RCN2	ENSG00000117906.13	228	268	1.2
protein_coding	JOSD1	ENSG00000100221.10	57	67	1.2
protein_coding	MYO6	ENSG00000196586.13	382	449	1.2
protein_coding	NR2F1	ENSG00000175745.12	177	208	1.2
protein_coding	CAPZA1	ENSG00000116489.12	497	584	1.2
protein_coding	STC2	ENSG00000113739.10	160	188	1.2
protein_coding	FAS	ENSG00000026103.21	80	94	1.2
protein_coding	CIB1	ENSG00000185043.11	103	121	1.2
protein_coding	PSMA7	ENSG00000101182.14	826	970	1.2
protein_coding	TMEM167A	ENSG00000174695.9	247	290	1.2
protein_coding	FAM177A1	ENSG00000151327.12	184	216	1.2
protein_coding	TBRG1	ENSG00000154144.12	115	135	1.2
protein_coding	RAB11B	ENSG00000185236.11	115	135	1.2
protein_coding	PEX26	ENSG00000215193.12	92	108	1.2
protein_coding	SEC11C	ENSG00000166562.8	69	81	1.2
protein_coding	EFL1	ENSG00000140598.14	46	54	1.2
protein_coding	NCOA7	ENSG00000111912.19	98	115	1.2
protein_coding	CUL2	ENSG00000108094.14	52	61	1.2
protein_coding	ZCCHC9	ENSG00000131732.11	52	61	1.2
protein_coding	RPN2	ENSG00000118705.16	614	720	1.2
protein_coding	BTRC	ENSG00000166167.17	58	68	1.2
protein_coding	ZNF33A	ENSG00000189180.15	29	34	1.2
protein_coding	DFFA	ENSG00000160049.11	128	150	1.2
protein_coding	MBNL2	ENSG00000139793.18	64	75	1.2
protein_coding	TRIM2	ENSG00000109654.14	99	116	1.2
protein_coding	PRPF6	ENSG00000101161.7	140	164	1.2
protein_coding	MPP5	ENSG00000072415.8	105	123	1.2
protein_coding	C9orf85	ENSG00000155621.14	35	41	1.2
protein_coding	RCAN3	ENSG00000117602.11	35	41	1.2
protein_coding	TMEM50A	ENSG00000183726.10	496	581	1.2
protein_coding	C14orf119	ENSG00000179933.5	187	219	1.2
protein_coding	NUP88	ENSG00000108559.11	41	48	1.2
protein_coding	GPX7	ENSG00000116157.5	41	48	1.2
protein_coding	STRAP	ENSG00000023734.10	334	391	1.2
protein_coding	SERINC1	ENSG00000111897.6	829	970	1.2
protein_coding	MRPL14	ENSG00000180992.6	100	117	1.2
protein_coding	SLCO3A1	ENSG00000176463.13	106	124	1.2
protein_coding	PRDM4	ENSG00000110851.11	53	62	1.2
protein_coding	MIEN1	ENSG00000141741.11	53	62	1.2
protein_coding	ZC3H14	ENSG00000100722.19	112	131	1.2
protein_coding	YLPM1	ENSG00000119596.17	177	207	1.2
protein_coding	PHC3	ENSG00000173889.15	118	138	1.2
protein_coding	LANCL1	ENSG00000115365.11	124	145	1.2
protein_coding	CD59	ENSG00000085063.15	2097	2452	1.2

protein_coding	TMEM131	ENSG00000075568.16	195	228	1.2
protein_coding	VMA21	ENSG00000160131.13	130	152	1.2
protein_coding	SEC24A	ENSG00000113615.12	65	76	1.2
protein_coding	MRPS22	ENSG00000175110.11	65	76	1.2
protein_coding	MBTPS1	ENSG00000140943.16	343	401	1.2
protein_coding	DYNC1LI2	ENSG00000135720.12	355	415	1.2
protein_coding	FXR2	ENSG00000129245.11	77	90	1.2
protein_coding	SRSF5	ENSG00000100650.15	279	326	1.2
protein_coding	WBP11	ENSG00000084463.7	95	111	1.2
protein_coding	BIRC2	ENSG00000110330.8	196	229	1.2
protein_coding	LARP7	ENSG00000174720.15	220	257	1.2
protein_coding	MRPL21	ENSG00000197345.12	119	139	1.2
protein_coding	USP9X	ENSG00000124486.12	256	299	1.2
protein_coding	PLEKHO1	ENSG00000023902.13	179	209	1.2
protein_coding	CHD2	ENSG00000173575.20	198	231	1.2
protein_coding	HSPA13	ENSG00000155304.5	90	105	1.2
protein_coding	STEAP2	ENSG00000157214.13	84	98	1.2
protein_coding	FAM20B	ENSG00000116199.11	72	84	1.2
protein_coding	SMYD3	ENSG00000185420.18	60	70	1.2
protein_coding	TMEM237	ENSG00000155755.18	60	70	1.2
protein_coding	RAD54L2	ENSG00000164080.13	60	70	1.2
protein_coding	CEP85L	ENSG00000111860.13	48	56	1.2
protein_coding	PPP1R15B	ENSG00000158615.8	48	56	1.2
protein_coding	IDH3A	ENSG00000166411.13	42	49	1.2
protein_coding	SLC30A6	ENSG00000152683.14	42	49	1.2
protein_coding	CHCHD7	ENSG00000170791.17	36	42	1.2
protein_coding	AP1AR	ENSG00000138660.11	30	35	1.2
protein_coding	KANSL2	ENSG00000139620.12	30	35	1.2
protein_coding	AGBL5	ENSG00000084693.15	30	35	1.2
protein_coding	CYTH1	ENSG00000108669.16	24	28	1.2
protein_coding	ELOVL4	ENSG00000118402.5	24	28	1.2
protein_coding	EFHC1	ENSG00000096093.15	24	28	1.2
protein_coding	BRMS1L	ENSG00000100916.13	24	28	1.2
protein_coding	ELP4	ENSG00000109911.18	24	28	1.2
protein_coding	APLF	ENSG00000169621.9	24	28	1.2
protein_coding	KTI12	ENSG00000198841.3	18	21	1.2
protein_coding	ZNF768	ENSG00000169957.9	18	21	1.2
protein_coding	SERGEF	ENSG00000129158.10	18	21	1.2
protein_coding	ALKBH6	ENSG00000239382.10	18	21	1.2
protein_coding	FBXO10	ENSG00000147912.12	18	21	1.2
protein_coding	SLC25A33	ENSG00000171612.6	12	14	1.2
protein_coding	RNPC3	ENSG00000185946.15	12	14	1.2
protein_coding	ZNF611	ENSG00000213020.9	12	14	1.2
processed_pseudogene	RPL34P27	ENSG00000232858.1	12	14	1.2

protein_coding	CDCA7L	ENSG00000164649.19	12	14	1.2
protein_coding	TRMT2B	ENSG00000188917.14	12	14	1.2
protein_coding	TXNL4B	ENSG00000140830.8	12	14	1.2
protein_coding	TMEM26	ENSG00000196932.11	12	14	1.2
protein_coding	SDSL	ENSG00000139410.14	12	14	1.2
protein_coding	GFOD1	ENSG00000145990.10	12	14	1.2
protein_coding	AL592183.1	ENSG00000273748.1	6	7	1.2
sense_overlapping	AL133355.1	ENSG00000260461.1	6	7	1.2
TEC	AL353763.2	ENSG00000280077.1	6	7	1.2
protein_coding	VAC14	ENSG00000103043.14	6	7	1.2
protein_coding	FAAP24	ENSG00000131944.9	6	7	1.2
protein_coding	PPP1R3G	ENSG00000219607.3	6	7	1.2
protein_coding	GKAP1	ENSG00000165113.12	6	7	1.2
protein_coding	SLC13A3	ENSG00000158296.13	6	7	1.2
protein_coding	CELSR2	ENSG00000143126.7	6	7	1.2
antisense	AC009506.1	ENSG00000224152.1	6	7	1.2
protein_coding	LRRC29	ENSG00000125122.15	6	7	1.2
protein_coding	TTC39A	ENSG00000085831.15	6	7	1.2
transcribed_processed_pseudogene	RPL23AP49	ENSG00000243422.2	6	7	1.2
protein_coding	CXCL12	ENSG00000107562.16	1641	1914	1.2
protein_coding	ZBTB7A	ENSG00000178951.8	445	519	1.2
protein_coding	ATP5F1B	ENSG00000110955.8	747	871	1.2
protein_coding	RPL41	ENSG00000229117.8	169	197	1.2
protein_coding	DDX5	ENSG00000108654.14	930	1084	1.2
protein_coding	EI24	ENSG00000149547.14	272	317	1.2
protein_coding	MED28	ENSG00000118579.12	103	120	1.2
protein_coding	MRPL41	ENSG00000182154.7	91	106	1.2
protein_coding	EIF4A3	ENSG00000141543.10	146	170	1.2
protein_coding	KIAA0100	ENSG00000007202.14	67	78	1.2
protein_coding	RPS7	ENSG00000171863.14	1073	1249	1.2
protein_coding	NEU1	ENSG00000204386.10	122	142	1.2
protein_coding	IVD	ENSG00000128928.8	61	71	1.2
protein_coding	DAD1	ENSG00000129562.10	226	263	1.2
protein_coding	ATE1	ENSG00000107669.17	55	64	1.2
protein_coding	ERCC3	ENSG00000163161.13	55	64	1.2
protein_coding	COX6C	ENSG00000164919.10	813	946	1.2
protein_coding	RAB5B	ENSG00000111540.15	104	121	1.2
protein_coding	FADS2	ENSG00000134824.13	147	171	1.2
protein_coding	TRAPPC8	ENSG00000153339.13	49	57	1.2
protein_coding	PLA2G16	ENSG00000176485.11	92	107	1.2
protein_coding	XPR1	ENSG00000143324.13	86	100	1.2
protein_coding	DENND4A	ENSG00000174485.15	86	100	1.2

protein_coding	MGA	ENSG00000174197.16	37	43	1.2
protein_coding	CKAP2	ENSG00000136108.14	37	43	1.2
protein_coding	SPR	ENSG00000116096.5	37	43	1.2
protein_coding	LPP	ENSG00000145012.13	315	366	1.2
protein_coding	IBTK	ENSG00000005700.14	105	122	1.2
protein_coding	ANKRD40	ENSG00000154945.6	62	72	1.2
lincRNA	MIR29B2CH	ENSG00000203709.11	31	36	1.2
transcribed_unprocessed_pseudogene	ZNF542P	ENSG00000240225.10	31	36	1.2
protein_coding	ORMDL2	ENSG00000123353.9	31	36	1.2
protein_coding	C19orf24	ENSG00000228300.13	56	65	1.2
protein_coding	PRKCI	ENSG00000163558.12	56	65	1.2
protein_coding	ELOVL5	ENSG00000012660.13	212	246	1.2
protein_coding	GRSF1	ENSG00000132463.13	181	210	1.2
protein_coding	HEXB	ENSG00000049860.13	1181	1370	1.2
protein_coding	EIF3K	ENSG00000178982.9	200	232	1.2
protein_coding	SFSWAP	ENSG00000061936.9	75	87	1.2
protein_coding	C1orf216	ENSG00000142686.7	50	58	1.2
protein_coding	ZW10	ENSG00000086827.8	25	29	1.2
protein_coding	C14orf28	ENSG00000179476.7	25	29	1.2
protein_coding	EIF2S2	ENSG00000125977.6	613	711	1.2
protein_coding	MFF	ENSG00000168958.19	169	196	1.2
protein_coding	KDM5A	ENSG00000073614.12	238	276	1.2
protein_coding	PDIA4	ENSG00000155660.10	188	218	1.2
protein_coding	PQLC1	ENSG00000122490.18	69	80	1.2
protein_coding	DCTN6	ENSG00000104671.7	113	131	1.2
protein_coding	CALR	ENSG00000179218.13	1174	1361	1.2
protein_coding	CCT7	ENSG00000135624.15	176	204	1.2
protein_coding	POLE4	ENSG00000115350.11	44	51	1.2
protein_coding	HPS3	ENSG00000163755.8	44	51	1.2
protein_coding	TMEM200B	ENSG00000253304.1	44	51	1.2
protein_coding	PFDN1	ENSG00000113068.9	151	175	1.2
protein_coding	VPS13B	ENSG00000132549.18	63	73	1.2
protein_coding	RAB7A	ENSG00000075785.12	523	606	1.2
protein_coding	C5orf24	ENSG00000181904.8	227	263	1.2
protein_coding	CSNK2A1	ENSG00000101266.18	164	190	1.2
protein_coding	RALBP1	ENSG00000017797.12	221	256	1.2
protein_coding	UFC1	ENSG00000143222.11	240	278	1.2
protein_coding	PDLIM5	ENSG00000163110.14	120	139	1.2
protein_coding	PPIG	ENSG00000138398.15	380	440	1.2
protein_coding	PSMD4	ENSG00000159352.15	304	352	1.2
protein_coding	FAM120AOS	ENSG00000188938.16	76	88	1.2
protein_coding	ABCD3	ENSG00000117528.12	76	88	1.2

protein_coding	USP13	ENSG00000058056.8	57	66	1.2
protein_coding	ST7	ENSG00000004866.20	38	44	1.2
protein_coding	SLC25A20	ENSG00000178537.9	38	44	1.2
protein_coding	PRKAA2	ENSG00000162409.10	19	22	1.2
protein_coding	GAN	ENSG00000261609.6	19	22	1.2
protein_coding	DNASE1	ENSG00000213918.10	19	22	1.2
protein_coding	LIG3	ENSG00000005156.11	19	22	1.2
protein_coding	PTK2B	ENSG00000120899.17	19	22	1.2
protein_coding	OSCP1	ENSG00000116885.18	19	22	1.2
protein_coding	ERFE	ENSG00000178752.15	19	22	1.2
protein_coding	FAM53C	ENSG00000120709.10	89	103	1.2
protein_coding	EIF1AX	ENSG00000173674.10	248	287	1.2
protein_coding	AES	ENSG00000104964.14	299	346	1.2
protein_coding	LSM¬†5	ENSG00000106355.9	70	81	1.2
protein_coding	P3H1	ENSG00000117385.15	153	177	1.2
protein_coding	PIKFYVE	ENSG00000115020.16	51	59	1.2
protein_coding	GALNS	ENSG00000141012.12	51	59	1.2
protein_coding	VMP1	ENSG00000062716.12	198	229	1.2
protein_coding	KRCC1	ENSG00000172086.7	115	133	1.2
protein_coding	GANAB	ENSG00000089597.17	768	888	1.2
protein_coding	FAM50B	ENSG00000145945.6	32	37	1.2
protein_coding	AGAP3	ENSG00000133612.18	77	89	1.2
protein_coding	ADRM1	ENSG00000130706.12	122	141	1.2
protein_coding	WNT5B	ENSG00000111186.12	315	364	1.2
protein_coding	SLC38A10	ENSG00000157637.12	135	156	1.2
protein_coding	ARFRP1	ENSG00000101246.19	45	52	1.2
protein_coding	LRP6	ENSG00000070018.8	45	52	1.2
protein_coding	AASDHPPT	ENSG00000149313.10	103	119	1.2
protein_coding	MAP3K7	ENSG00000135341.17	161	186	1.2
protein_coding	ZBTB44	ENSG00000196323.13	161	186	1.2
protein_coding	AACS	ENSG00000081760.16	58	67	1.2
protein_coding	ASXL1	ENSG00000171456.18	58	67	1.2
protein_coding	PAIP2	ENSG00000120727.12	271	313	1.2
protein_coding	STARD3	ENSG00000131748.15	71	82	1.2
protein_coding	VPS51	ENSG00000149823.8	71	82	1.2
protein_coding	DNASE2	ENSG00000105612.8	123	142	1.2
protein_coding	APH1A	ENSG00000117362.12	201	232	1.2
protein_coding	CBX3	ENSG00000122565.18	694	801	1.2
protein_coding	GAPVD1	ENSG00000165219.21	91	105	1.2
protein_coding	UBTD2	ENSG00000168246.5	78	90	1.2
protein_coding	GSKIP	ENSG00000100744.14	52	60	1.2
protein_coding	OGDH	ENSG00000105953.14	39	45	1.2
protein_coding	GSK3A	ENSG00000105723.11	26	30	1.2
protein_coding	CENPBD1	ENSG00000177946.6	26	30	1.2

protein_coding	SMARCD3	ENSG00000082014.16	26	30	1.2
protein_coding	FBXL2	ENSG00000153558.15	26	30	1.2
lincRNA	AC017002.3	ENSG00000240350.2	13	15	1.2
protein_coding	CACFD1	ENSG00000160325.14	13	15	1.2
protein_coding	CIAO3	ENSG00000103245.13	13	15	1.2
protein_coding	MYCBP	ENSG00000214114.8	13	15	1.2
protein_coding	GPATCH1	ENSG00000076650.6	13	15	1.2
protein_coding	KIAA1147	ENSG00000257093.6	13	15	1.2
protein_coding	PRR5	ENSG00000186654.20	13	15	1.2
protein_coding	POLR3F	ENSG00000132664.11	13	15	1.2
protein_coding	ATAD1	ENSG00000138138.13	176	203	1.2
protein_coding	PDZD8	ENSG00000165650.11	85	98	1.2
protein_coding	TSR3	ENSG00000007520.3	118	136	1.2
protein_coding	NDUFB9	ENSG00000147684.8	223	257	1.2
protein_coding	SF3A1	ENSG00000099995.18	164	189	1.2
protein_coding	CLPTM1	ENSG00000104853.15	46	53	1.2
protein_coding	ECE1	ENSG00000117298.15	434	500	1.2
protein_coding	KIF1C	ENSG00000129250.11	375	432	1.2
protein_coding	RHBDD2	ENSG00000005486.16	79	91	1.2
protein_coding	SLC39A13	ENSG00000165915.13	231	266	1.2
protein_coding	TSTA3	ENSG00000104522.15	33	38	1.2
protein_coding	MYADM	ENSG00000179820.15	417	480	1.2
protein_coding	ARF6	ENSG00000165527.6	192	221	1.2
protein_coding	PSMC4	ENSG00000013275.7	106	122	1.2
protein_coding	TUBG1	ENSG00000131462.7	53	61	1.2
protein_coding	ZNF605	ENSG00000196458.10	53	61	1.2
protein_coding	GLE1	ENSG00000119392.14	53	61	1.2
protein_coding	MICU3	ENSG00000155970.11	53	61	1.2
protein_coding	TGOLN2	ENSG00000152291.13	889	1023	1.2
protein_coding	XIAP	ENSG00000101966.12	146	168	1.2
protein_coding	ENPP1	ENSG00000197594.12	73	84	1.2
protein_coding	MMGT1	ENSG00000169446.5	113	130	1.2
protein_coding	LTBP3	ENSG00000168056.15	100	115	1.2
protein_coding	MRPS7	ENSG00000125445.10	60	69	1.2
protein_coding	G6PC3	ENSG00000141349.8	60	69	1.2
protein_coding	TAPT1	ENSG00000169762.16	40	46	1.2
protein_coding	KMT2B	ENSG00000272333.5	40	46	1.2
protein_coding	POLL	ENSG00000166169.16	20	23	1.2
protein_coding	SLC39A4	ENSG00000147804.9	20	23	1.2
protein_coding	ATG10	ENSG00000152348.15	20	23	1.2
protein_coding	NPTN	ENSG00000156642.16	707	813	1.1
protein_coding	GAPDH	ENSG00000111640.14	4460	5127	1.1
protein_coding	BMPR2	ENSG00000204217.13	301	346	1.1
protein_coding	PDIA5	ENSG00000065485.19	87	100	1.1

protein_coding	SRSF10	ENSG00000188529.14	134	154	1.1
protein_coding	BBS2	ENSG00000125124.11	67	77	1.1
protein_coding	TGFBR2	ENSG00000163513.17	295	339	1.1
protein_coding	CALD1	ENSG00000122786.19	3748	4307	1.1
protein_coding	HNRNPC	ENSG00000092199.17	228	262	1.1
protein_coding	YWHAE	ENSG00000108953.16	758	871	1.1
protein_coding	DSEL	ENSG00000171451.13	1805	2074	1.1
protein_coding	FAU	ENSG00000149806.10	1134	1303	1.1
protein_coding	ATP5IF1	ENSG00000130770.17	376	432	1.1
protein_coding	NDUFAB1	ENSG00000004779.9	215	247	1.1
protein_coding	NF1	ENSG00000196712.17	148	170	1.1
protein_coding	ZSCAN18	ENSG00000121413.12	74	85	1.1
protein_coding	MTMR6	ENSG00000139505.11	108	124	1.1
protein_coding	MYO9B	ENSG00000099331.13	54	62	1.1
protein_coding	TOGARAM1	ENSG00000198718.12	54	62	1.1
protein_coding	MECP2	ENSG00000169057.21	88	101	1.1
protein_coding	TNRC6A	ENSG00000090905.18	183	210	1.1
protein_coding	FRA10AC1	ENSG00000148690.11	122	140	1.1
protein_coding	TMEM70	ENSG00000175606.10	61	70	1.1
protein_coding	FNDC3B	ENSG00000075420.12	312	358	1.1
protein_coding	GALNT7	ENSG00000109586.11	68	78	1.1
protein_coding	IFT57	ENSG00000114446.4	68	78	1.1
protein_coding	CKS2	ENSG00000123975.4	34	39	1.1
protein_coding	PIBF1	ENSG00000083535.15	34	39	1.1
protein_coding	VPS9D1	ENSG00000075399.13	34	39	1.1
protein_coding	ZNF318	ENSG00000171467.15	41	47	1.1
protein_coding	USF3	ENSG00000176542.9	144	165	1.1
protein_coding	SMIM10	ENSG00000184785.5	48	55	1.1
protein_coding	SLC44A1	ENSG00000070214.15	1537	1761	1.1
protein_coding	C9orf78	ENSG00000136819.15	158	181	1.1
protein_coding	OSTM1	ENSG00000081087.14	165	189	1.1
protein_coding	STX18	ENSG00000168818.9	55	63	1.1
protein_coding	PTK2	ENSG00000169398.19	62	71	1.1
protein_coding	CCT8	ENSG00000156261.12	304	348	1.1
protein_coding	SRSF3	ENSG00000112081.16	76	87	1.1
protein_coding	ANKIB1	ENSG00000001629.9	166	190	1.1
protein_coding	CWF19L2	ENSG00000152404.15	83	95	1.1
protein_coding	MAP4K4	ENSG00000071054.16	519	594	1.1
protein_coding	KPNA4	ENSG00000186432.8	180	206	1.1
protein_coding	FBXW5	ENSG00000159069.13	97	111	1.1
protein_coding	SRSF1	ENSG00000136450.12	97	111	1.1
protein_coding	AMZ2	ENSG00000196704.11	104	119	1.1
protein_coding	DAP3	ENSG00000132676.15	118	135	1.1
protein_coding	DECR1	ENSG00000104325.6	237	271	1.1

protein_coding	TMOD3	ENSG00000138594.13	280	320	1.1
protein_coding	PPTC7	ENSG00000196850.5	91	104	1.1
protein_coding	S1PR3	ENSG00000213694.4	84	96	1.1
protein_coding	GATAD2B	ENSG00000143614.9	77	88	1.1
protein_coding	LCOR	ENSG00000196233.13	63	72	1.1
protein_coding	STAU2	ENSG00000040341.17	49	56	1.1
protein_coding	NCBP1	ENSG00000136937.12	49	56	1.1
protein_coding	TCF20	ENSG00000100207.18	42	48	1.1
protein_coding	KIAA1551	ENSG00000174718.11	42	48	1.1
protein_coding	MRPL54	ENSG00000183617.4	42	48	1.1
protein_coding	SNTA1	ENSG00000101400.5	42	48	1.1
protein_coding	EPN2	ENSG00000072134.15	35	40	1.1
protein_coding	MCM6	ENSG00000076003.4	35	40	1.1
protein_coding	KAT14	ENSG00000149474.13	28	32	1.1
protein_coding	PIP4P1	ENSG00000165782.10	28	32	1.1
protein_coding	ZCCHC10	ENSG00000155329.11	21	24	1.1
protein_coding	HPRT1	ENSG00000165704.14	21	24	1.1
protein_coding	CSRNP1	ENSG00000144655.14	21	24	1.1
protein_coding	SCAMP4	ENSG00000227500.9	21	24	1.1
processed_transcript	C5orf56	ENSG00000197536.11	21	24	1.1
protein_coding	MELTF	ENSG00000163975.11	21	24	1.1
antisense	RAB11B-AS1	ENSG00000269386.5	21	24	1.1
protein_coding	BCL9	ENSG00000116128.10	21	24	1.1
protein_coding	ARHGEF1	ENSG00000076928.17	14	16	1.1
protein_coding	FAM161A	ENSG00000170264.12	14	16	1.1
protein_coding	PRMT7	ENSG00000132600.16	14	16	1.1
protein_coding	IFT172	ENSG00000138002.14	14	16	1.1
protein_coding	NRSN2	ENSG00000125841.12	14	16	1.1
protein_coding	RANBP10	ENSG00000141084.10	14	16	1.1
protein_coding	TOP3A	ENSG00000177302.14	7	8	1.1
protein_coding	SLX4IP	ENSG00000149346.14	7	8	1.1
protein_coding	EXO5	ENSG00000164002.11	7	8	1.1
protein_coding	ELOVL3	ENSG00000119915.4	7	8	1.1
protein_coding	TANGO6	ENSG00000103047.7	7	8	1.1
protein_coding	ATP7B	ENSG00000123191.14	7	8	1.1
protein_coding	MCM5	ENSG00000100297.15	7	8	1.1
unprocessed_pseudogene	FRG1BP	ENSG00000149531.15	7	8	1.1
protein_coding	PTGR2	ENSG00000140043.11	7	8	1.1
protein_coding	CCDC113	ENSG00000103021.9	7	8	1.1
lincRNA	BX890604.2	ENSG00000285756.1	7	8	1.1
antisense	PIK3CD-AS2	ENSG00000231789.2	7	8	1.1
protein_coding	STAP2	ENSG00000178078.11	7	8	1.1
sense_overlapping	AL158206.1	ENSG00000260912.1	7	8	1.1
sense_overlapping	AL353796.1	ENSG00000259994.1	7	8	1.1

protein_coding	NUDT4	ENSG00000173598.13	190	217	1.1
protein_coding	PPIP5K2	ENSG00000145725.19	106	121	1.1
protein_coding	PRKAG1	ENSG00000181929.12	106	121	1.1
protein_coding	KIF3B	ENSG00000101350.7	205	234	1.1
protein_coding	FOXK2	ENSG00000141568.20	92	105	1.1
protein_coding	SRGN	ENSG00000122862.4	1112	1269	1.1
protein_coding	EIF4E2	ENSG00000135930.13	149	170	1.1
protein_coding	ATG4B	ENSG00000168397.16	71	81	1.1
protein_coding	ZBTB20	ENSG00000181722.16	490	559	1.1
protein_coding	CDK7	ENSG00000134058.11	64	73	1.1
protein_coding	ATP6V0A1	ENSG00000033627.16	64	73	1.1
protein_coding	MLLT6	ENSG00000275023.4	57	65	1.1
protein_coding	ZNF609	ENSG00000180357.9	50	57	1.1
protein_coding	GIT2	ENSG00000139436.20	93	106	1.1
protein_coding	MT-ATP6	ENSG00000198899.2	7577	8636	1.1
processed_pseudogene	RPL23AP42	ENSG00000234851.4	86	98	1.1
protein_coding	UIMC1	ENSG00000087206.16	43	49	1.1
protein_coding	CCDC84	ENSG00000186166.8	43	49	1.1
protein_coding	TSPYL5	ENSG00000180543.4	43	49	1.1
protein_coding	ACTR3	ENSG00000115091.11	302	344	1.1
protein_coding	C16orf72	ENSG00000182831.11	108	123	1.1
protein_coding	RIOK3	ENSG00000101782.14	108	123	1.1
protein_coding	QDPR	ENSG00000151552.11	72	82	1.1
protein_coding	BRAP	ENSG00000089234.15	36	41	1.1
protein_coding	NANS	ENSG00000095380.10	65	74	1.1
protein_coding	TMTC3	ENSG00000139324.11	376	428	1.1
protein_coding	AURKAIP1	ENSG00000175756.13	181	206	1.1
protein_coding	COL14A1	ENSG00000187955.11	87	99	1.1
protein_coding	PPP1R21	ENSG00000162869.15	29	33	1.1
protein_coding	EMSY	ENSG00000158636.16	29	33	1.1
protein_coding	MMP14	ENSG00000157227.12	1569	1785	1.1
protein_coding	CKAP4	ENSG00000136026.13	1660	1888	1.1
protein_coding	ZNF148	ENSG00000163848.19	153	174	1.1
protein_coding	LYRM4	ENSG00000214113.10	51	58	1.1
protein_coding	EXTL3	ENSG00000012232.8	73	83	1.1
protein_coding	WFS1	ENSG00000109501.13	73	83	1.1
protein_coding	UBAC2	ENSG00000134882.15	205	233	1.1
protein_coding	RPL36AL	ENSG00000165502.6	484	550	1.1
protein_coding	STEAP1	ENSG00000164647.8	88	100	1.1
protein_coding	NAA15	ENSG00000164134.12	66	75	1.1
protein_coding	SHARPIN	ENSG00000179526.16	44	50	1.1
protein_coding	GPATCH11	ENSG00000152133.14	22	25	1.1
protein_coding	FLYWCH1	ENSG00000059122.16	22	25	1.1
protein_coding	TRMO	ENSG00000136932.13	22	25	1.1

protein_coding	CCAR2	ENSG00000158941.16	22	25	1.1
protein_coding	GHITM	ENSG00000165678.20	367	417	1.1
protein_coding	DARS	ENSG00000115866.10	279	317	1.1
protein_coding	COPS2	ENSG00000166200.14	294	334	1.1
protein_coding	MAT2B	ENSG00000038274.16	147	167	1.1
protein_coding	RUNX2	ENSG00000124813.21	103	117	1.1
protein_coding	CCM2	ENSG00000136280.16	81	92	1.1
protein_coding	ACP2	ENSG00000134575.9	81	92	1.1
protein_coding	UBFD1	ENSG00000103353.15	155	176	1.1
protein_coding	MRPL17	ENSG00000158042.8	96	109	1.1
protein_coding	CCDC59	ENSG00000133773.11	96	109	1.1
protein_coding	UBQLN1	ENSG00000135018.13	266	302	1.1
protein_coding	GTF3A	ENSG00000122034.14	133	151	1.1
protein_coding	ABT1	ENSG00000146109.4	37	42	1.1
protein_coding	ACAT2	ENSG00000120437.8	37	42	1.1
protein_coding	UQCRC2	ENSG00000140740.10	274	311	1.1
protein_coding	NUCB1	ENSG00000104805.15	215	244	1.1
protein_coding	MTHFD1L	ENSG00000120254.15	52	59	1.1
protein_coding	ZRSR2	ENSG00000169249.12	52	59	1.1
protein_coding	TYW1	ENSG00000198874.12	52	59	1.1
protein_coding	ADAM15	ENSG00000143537.13	119	135	1.1
protein_coding	RFC1	ENSG00000035928.15	186	211	1.1
protein_coding	ATP6V1E1	ENSG00000131100.12	261	296	1.1
protein_coding	RAI1	ENSG00000108557.18	97	110	1.1
protein_coding	MT-CYB	ENSG00000198727.2	8685	9843	1.1
protein_coding	H2AFZ	ENSG00000164032.11	405	459	1.1
protein_coding	RYBP	ENSG00000163602.10	210	238	1.1
protein_coding	SIX4	ENSG00000100625.8	105	119	1.1
protein_coding	MINDY3	ENSG00000148481.13	75	85	1.1
protein_coding	ZNF654	ENSG00000175105.6	45	51	1.1
protein_coding	PYROXD1	ENSG00000121350.15	45	51	1.1
antisense	AP001062.1	ENSG00000184441.4	30	34	1.1
protein_coding	PRPF39	ENSG00000185246.17	30	34	1.1
protein_coding	ZNF785	ENSG00000197162.9	30	34	1.1
protein_coding	DDX49	ENSG00000105671.11	30	34	1.1
protein_coding	ENO2	ENSG00000111674.8	15	17	1.1
protein_coding	MYBBP1A	ENSG00000132382.14	15	17	1.1
protein_coding	WDR73	ENSG00000177082.12	15	17	1.1
protein_coding	ZNF383	ENSG00000188283.11	15	17	1.1
protein_coding	PQLC2	ENSG00000040487.12	15	17	1.1
lincRNA	AC060780.1	ENSG00000267002.3	15	17	1.1
protein_coding	TCF7	ENSG00000081059.19	15	17	1.1
protein_coding	NOL6	ENSG00000165271.16	15	17	1.1
protein_coding	RHOBTB3	ENSG00000164292.12	429	486	1.1

protein_coding	RPL6	ENSG00000089009.15	828	938	1.1
protein_coding	FBXL3	ENSG00000005812.10	98	111	1.1
protein_coding	UBE2K	ENSG00000078140.13	189	214	1.1
protein_coding	UBLCP1	ENSG00000164332.7	121	137	1.1
protein_coding	CTCF	ENSG00000102974.15	53	60	1.1
protein_coding	IDH2	ENSG00000182054.9	53	60	1.1
protein_coding	C5orf15	ENSG00000113583.7	409	463	1.1
protein_coding	SERBP1	ENSG00000142864.14	341	386	1.1
protein_coding	WDR6	ENSG00000178252.17	91	103	1.1
protein_coding	MSRB3	ENSG00000174099.11	243	275	1.1
protein_coding	MGAT5	ENSG00000152127.8	281	318	1.1
protein_coding	RER1	ENSG00000157916.19	342	387	1.1
protein_coding	SEH1L	ENSG00000085415.15	38	43	1.1
protein_coding	AKAP8	ENSG00000105127.8	38	43	1.1
protein_coding	KLHL18	ENSG00000114648.11	38	43	1.1
protein_coding	TXNDC15	ENSG00000113621.14	206	233	1.1
protein_coding	RPS14	ENSG00000164587.12	1832	2072	1.1
protein_coding	MAGT1	ENSG00000102158.19	168	190	1.1
protein_coding	AP1B1	ENSG00000100280.16	107	121	1.1
protein_coding	IARS2	ENSG00000067704.9	138	156	1.1
protein_coding	KATNA1	ENSG00000186625.13	46	52	1.1
protein_coding	LIG4	ENSG00000174405.13	23	26	1.1
protein_coding	FUNDC2	ENSG00000165775.17	23	26	1.1
protein_coding	VRK3	ENSG00000105053.10	23	26	1.1
protein_coding	ZNF91	ENSG00000167232.13	23	26	1.1
protein_coding	ULK1	ENSG00000177169.9	192	217	1.1
protein_coding	OSBP	ENSG00000110048.11	100	113	1.1
protein_coding	RAB18	ENSG00000099246.16	309	349	1.1
protein_coding	GRAMD2B	ENSG00000155324.9	85	96	1.1
protein_coding	POPDC3	ENSG00000132429.9	116	131	1.1
protein_coding	HNRNPDL	ENSG00000152795.17	294	332	1.1
protein_coding	SLC35C2	ENSG00000080189.14	62	70	1.1
protein_coding	RRAD	ENSG00000166592.11	31	35	1.1
protein_coding	RNF2	ENSG00000121481.10	31	35	1.1
protein_coding	STXBP4	ENSG00000166263.13	31	35	1.1
protein_coding	MICAL1	ENSG00000135596.17	31	35	1.1
protein_coding	FAM83H	ENSG00000180921.6	31	35	1.1
protein_coding	COMMD3	ENSG00000148444.15	101	114	1.1
protein_coding	CCDC93	ENSG00000125633.10	70	79	1.1
protein_coding	POLDIP3	ENSG00000100227.17	70	79	1.1
protein_coding	SNX13	ENSG00000071189.21	148	167	1.1
protein_coding	ACTR2	ENSG00000138071.13	522	589	1.1
protein_coding	SLC25A26	ENSG00000144741.17	39	44	1.1
antisense	AC100810.1	ENSG00000253982.1	39	44	1.1

protein_coding	CRIM1	ENSG00000150938.9	562	634	1.1
protein_coding	TEAD2	ENSG00000074219.13	164	185	1.1
protein_coding	EXTL2	ENSG00000162694.13	125	141	1.1
protein_coding	SEC22B	ENSG00000265808.3	133	150	1.1
protein_coding	MPC2	ENSG00000143158.10	141	159	1.1
protein_coding	RPL14	ENSG00000188846.13	948	1069	1.1
protein_coding	SCARB2	ENSG00000138760.9	627	707	1.1
protein_coding	KCTD12	ENSG00000178695.5	518	584	1.1
protein_coding	INTS10	ENSG00000104613.11	110	124	1.1
protein_coding	CDC40	ENSG00000168438.14	63	71	1.1
protein_coding	RPS6KB1	ENSG00000108443.13	63	71	1.1
protein_coding	HNRNPM	ENSG00000099783.11	221	249	1.1
protein_coding	PITPNB	ENSG00000180957.17	174	196	1.1
protein_coding	WDR43	ENSG00000163811.11	87	98	1.1
protein_coding	QSER1	ENSG00000060749.15	95	107	1.1
protein_coding	TCF7L2	ENSG00000148737.16	95	107	1.1
protein_coding	JAM3	ENSG00000166086.12	198	223	1.1
protein_coding	AGPAT2	ENSG00000169692.12	103	116	1.1
protein_coding	ANP32B	ENSG00000136938.8	420	473	1.1
protein_coding	DR1	ENSG00000117505.12	111	125	1.1
protein_coding	XRN2	ENSG00000088930.7	460	518	1.1
protein_coding	TTC1	ENSG00000113312.10	143	161	1.1
protein_coding	GSTP1	ENSG00000084207.16	949	1068	1.1
protein_coding	CHCHD2	ENSG00000106153.12	1318	1483	1.1
protein_coding	MXRA7	ENSG00000182534.13	272	306	1.1
protein_coding	MRPL33	ENSG00000243147.7	240	270	1.1
protein_coding	ZNF24	ENSG00000172466.15	160	180	1.1
protein_coding	MACO1	ENSG00000204178.10	120	135	1.1
protein_coding	PITRM1	ENSG00000107959.15	112	126	1.1
protein_coding	FBXW11	ENSG00000072803.17	96	108	1.1
protein_coding	PGP	ENSG00000184207.8	40	45	1.1
protein_coding	AREL1	ENSG00000119682.16	40	45	1.1
protein_coding	MTFMT	ENSG00000103707.9	40	45	1.1
protein_coding	TIMM9	ENSG00000100575.13	40	45	1.1
protein_coding	USP53	ENSG00000145390.11	32	36	1.1
protein_coding	SLC8B1	ENSG00000089060.11	32	36	1.1
protein_coding	SPRYD7	ENSG00000123178.14	24	27	1.1
protein_coding	DNAJC14	ENSG00000135392.16	24	27	1.1
protein_coding	ARHGEF9	ENSG00000131089.15	24	27	1.1
protein_coding	MTRNR2L12	ENSG00000269028.3	16	18	1.1
protein_coding	ZNF350	ENSG00000256683.6	16	18	1.1
protein_coding	MROH1	ENSG00000179832.17	16	18	1.1
protein_coding	SPATA6	ENSG00000132122.11	16	18	1.1
protein_coding	SLC25A23	ENSG00000125648.14	16	18	1.1

protein_coding	HVCN1	ENSG00000122986.13	16	18	1.1
protein_coding	SLFN12	ENSG00000172123.12	16	18	1.1
protein_coding	ARMC6	ENSG00000105676.13	16	18	1.1
protein_coding	MAPKBP1	ENSG00000137802.13	16	18	1.1
protein_coding	IRAK2	ENSG00000134070.4	8	9	1.1
protein_coding	B3GALNT1	ENSG00000169255.14	8	9	1.1
protein_coding	ITPR1	ENSG00000150995.19	8	9	1.1
bidirectional_promoter_lncRNA	NIPBL-DT	ENSG00000285967.1	8	9	1.1
protein_coding	KLHDC1	ENSG00000197776.7	8	9	1.1
protein_coding	EVC	ENSG00000072840.12	8	9	1.1
antisense	AC105446.1	ENSG00000227053.1	8	9	1.1
protein_coding	CLEC2D	ENSG00000069493.14	8	9	1.1
protein_coding	EPM2A	ENSG00000112425.14	8	9	1.1
protein_coding	LIPC	ENSG00000166035.10	8	9	1.1
protein_coding	QPRT	ENSG00000103485.17	8	9	1.1
protein_coding	EZH2	ENSG00000106462.10	8	9	1.1
protein_coding	TMEM143	ENSG00000161558.10	8	9	1.1
protein_coding	KLC1	ENSG00000126214.21	153	172	1.1
protein_coding	GGNBP2	ENSG00000278311.4	290	326	1.1
protein_coding	ITM2B	ENSG00000136156.13	1276	1434	1.1
protein_coding	BUB3	ENSG00000154473.17	178	200	1.1
protein_coding	TMEM248	ENSG00000106609.16	259	291	1.1
protein_coding	YTHDF3	ENSG00000185728.16	170	191	1.1
protein_coding	PRDX3	ENSG00000165672.6	324	364	1.1
protein_coding	DHX9	ENSG00000135829.16	162	182	1.1
protein_coding	PAFAH1B2	ENSG00000168092.13	446	501	1.1
protein_coding	LSAMP	ENSG00000185565.11	73	82	1.1
protein_coding	HIST1H2AC	ENSG00000180573.9	211	237	1.1
protein_coding	SUMO3	ENSG00000184900.15	560	629	1.1
protein_coding	CCT6A	ENSG00000146731.10	317	356	1.1
protein_coding	ENTPD4	ENSG00000197217.12	57	64	1.1
protein_coding	ZNF264	ENSG00000083844.10	57	64	1.1
protein_coding	SEC13	ENSG00000157020.17	253	284	1.1
protein_coding	DIDO1	ENSG00000101191.16	49	55	1.1
protein_coding	TMEM159	ENSG00000011638.10	49	55	1.1
protein_coding	COPE	ENSG00000105669.13	90	101	1.1
protein_coding	RICTOR	ENSG00000164327.12	41	46	1.1
protein_coding	TRIM23	ENSG00000113595.14	41	46	1.1
protein_coding	SYF2	ENSG00000117614.9	230	258	1.1
protein_coding	TMEM8A	ENSG00000129925.10	148	166	1.1
protein_coding	GCN1	ENSG00000089154.10	74	83	1.1
protein_coding	USP10	ENSG00000103194.15	74	83	1.1

protein_coding	DPYD	ENSG00000188641.13	74	83	1.1
protein_coding	SAP18	ENSG00000150459.12	362	406	1.1
protein_coding	CNOT6L	ENSG00000138767.13	66	74	1.1
protein_coding	MBTPS2	ENSG00000012174.11	66	74	1.1
protein_coding	SC5D	ENSG00000109929.9	33	37	1.1
protein_coding	VPS54	ENSG00000143952.19	33	37	1.1
protein_coding	EPAS1	ENSG00000116016.13	1314	1473	1.1
protein_coding	SSPN	ENSG00000123096.11	58	65	1.1
protein_coding	NPDC1	ENSG00000107281.9	83	93	1.1
protein_coding	ARL8A	ENSG00000143862.7	108	121	1.1
protein_coding	MT-ND3	ENSG00000198840.2	12924	14478	1.1
protein_coding	SLIRP	ENSG00000119705.9	341	382	1.1
protein_coding	SUGT1	ENSG00000165416.14	283	317	1.1
protein_coding	ZHX3	ENSG00000174306.21	75	84	1.1
protein_coding	MEN1	ENSG00000133895.14	50	56	1.1
protein_coding	THOP1	ENSG00000172009.14	25	28	1.1
protein_coding	LYSMD2	ENSG00000140280.13	25	28	1.1
protein_coding	ST5	ENSG00000166444.18	25	28	1.1
protein_coding	TMEM106B	ENSG00000106460.18	192	215	1.1
protein_coding	HSPA4	ENSG00000170606.14	284	318	1.1
protein_coding	ACTR1A	ENSG00000138107.12	159	178	1.1
protein_coding	NAA50	ENSG00000121579.12	226	253	1.1
protein_coding	IMMT	ENSG00000132305.20	134	150	1.1
protein_coding	MRPS30	ENSG00000112996.10	67	75	1.1
protein_coding	ZFAND6	ENSG00000086666.18	143	160	1.1
protein_coding	GPI	ENSG00000105220.15	404	452	1.1
protein_coding	PYCR2	ENSG00000143811.18	118	132	1.1
protein_coding	MRPL15	ENSG00000137547.8	59	66	1.1
protein_coding	MARVELD1	ENSG00000155254.12	135	151	1.1
protein_coding	MYL12A	ENSG00000101608.12	743	831	1.1
protein_coding	CLPP	ENSG00000125656.9	76	85	1.1
protein_coding	RHEB	ENSG00000106615.9	76	85	1.1
protein_coding	GPSM1	ENSG00000160360.12	110	123	1.1
protein_coding	MED1	ENSG00000125686.11	144	161	1.1
protein_coding	TROVE2	ENSG00000116747.12	161	180	1.1
protein_coding	EEF1A1	ENSG00000156508.17	178	199	1.1
protein_coding	GNB2	ENSG00000172354.9	246	275	1.1
protein_coding	GLUL	ENSG00000135821.18	399	446	1.1
protein_coding	PHF6	ENSG00000156531.16	51	57	1.1
protein_coding	MYO18A	ENSG00000196535.16	34	38	1.1
protein_coding	EED	ENSG00000074266.19	34	38	1.1
protein_coding	TMEM168	ENSG00000146802.12	34	38	1.1
protein_coding	CRIP2	ENSG00000182809.10	17	19	1.1
protein_coding	ZNF134	ENSG00000213762.11	17	19	1.1

protein_coding	SFMBT1	ENSG00000163935.13	17	19	1.1
protein_coding	SNX16	ENSG00000104497.13	17	19	1.1
protein_coding	ERF	ENSG00000105722.9	17	19	1.1
protein_coding	GATB	ENSG00000059691.11	17	19	1.1
protein_coding	CCSER2	ENSG00000107771.16	154	172	1.1
protein_coding	UGP2	ENSG00000169764.15	137	153	1.1
protein_coding	FHOD1	ENSG00000135723.13	120	134	1.1
protein_coding	MCCC2	ENSG00000131844.15	60	67	1.1
protein_coding	SLC1A5	ENSG00000105281.12	163	182	1.1
protein_coding	FAM92A	ENSG00000188343.12	86	96	1.1
protein_coding	HARS2	ENSG00000112855.15	43	48	1.1
protein_coding	RPL7L1	ENSG00000146223.14	241	269	1.1
protein_coding	TTL	ENSG00000114999.7	190	212	1.1
protein_coding	GNAS	ENSG00000087460.24	2220	2477	1.1
protein_coding	CHPF2	ENSG00000033100.16	225	251	1.1
protein_coding	ASPH	ENSG00000198363.17	1031	1150	1.1
protein_coding	RC3H1	ENSG00000135870.11	52	58	1.1
protein_coding	SHQ1	ENSG00000144736.13	26	29	1.1
protein_coding	TTC17	ENSG00000052841.14	148	165	1.1
protein_coding	WDR11	ENSG00000120008.15	61	68	1.1
protein_coding	BECN1	ENSG00000126581.12	245	273	1.1
protein_coding	CPD	ENSG00000108582.11	210	234	1.1
protein_coding	FUNDC1	ENSG00000069509.5	35	39	1.1
protein_coding	RBM15	ENSG00000162775.14	35	39	1.1
protein_coding	TFIP11	ENSG00000100109.16	35	39	1.1
protein_coding	MKRN1	ENSG00000133606.10	219	244	1.1
protein_coding	SBNO1	ENSG00000139697.13	184	205	1.1
protein_coding	PRR13	ENSG00000205352.10	184	205	1.1
protein_coding	RAB14	ENSG00000119396.10	272	303	1.1
protein_coding	PRDX4	ENSG00000123131.12	351	391	1.1
protein_coding	LONP2	ENSG00000102910.13	158	176	1.1
protein_coding	GSDME	ENSG00000105928.14	79	88	1.1
protein_coding	NEO1	ENSG00000067141.16	88	98	1.1
protein_coding	INIP	ENSG00000148153.13	44	49	1.1
lincRNA	CYTOR	ENSG00000222041.11	97	108	1.1
protein_coding	NAP1L1	ENSG00000187109.13	1227	1366	1.1
protein_coding	CCNT2	ENSG00000082258.12	53	59	1.1
protein_coding	TAX1BP1	ENSG00000106052.13	751	836	1.1
protein_coding	ZC2HC1A	ENSG00000104427.11	62	69	1.1
protein_coding	SMARCA4	ENSG00000127616.18	133	148	1.1
protein_coding	NDUFA2	ENSG00000131495.8	142	158	1.1
protein_coding	STT3A	ENSG00000134910.13	311	346	1.1
protein_coding	RNF170	ENSG00000120925.15	80	89	1.1
protein_coding	MT-CO2	ENSG00000198712.1	7355	8182	1.1

protein_coding	RPL34	ENSG00000109475.16	3269	3636	1.1
protein_coding	PA2G4	ENSG00000170515.13	205	228	1.1
protein_coding	HTATIP2	ENSG00000109854.13	125	139	1.1
protein_coding	DENND5A	ENSG00000184014.7	134	149	1.1
protein_coding	UBXN6	ENSG00000167671.11	286	318	1.1
protein_coding	MOCS2	ENSG00000164172.18	170	189	1.1
protein_coding	CSNK2A2	ENSG00000070770.8	188	209	1.1
protein_coding	EPDR1	ENSG00000086289.11	197	219	1.1
protein_coding	DCAF6	ENSG00000143164.15	215	239	1.1
protein_coding	LRP1	ENSG00000123384.13	1673	1859	1.1
protein_coding	SMS	ENSG00000102172.15	135	150	1.1
protein_coding	CTTNBP2	ENSG00000077063.10	54	60	1.1
protein_coding	TSC2	ENSG00000103197.17	45	50	1.1
protein_coding	LRCH1	ENSG00000136141.14	45	50	1.1
protein_coding	AVL9	ENSG00000105778.18	36	40	1.1
protein_coding	POLR2D	ENSG00000144231.10	36	40	1.1
protein_coding	ST3GAL4	ENSG00000110080.18	27	30	1.1
protein_coding	TRPM4	ENSG00000130529.15	27	30	1.1
protein_coding	PEX14	ENSG00000142655.12	27	30	1.1
protein_coding	QRSL1	ENSG00000130348.11	27	30	1.1
protein_coding	ZNF354A	ENSG00000169131.12	18	20	1.1
protein_coding	ZBTB34	ENSG00000177125.5	18	20	1.1
protein_coding	SIMC1	ENSG00000170085.17	18	20	1.1
protein_coding	AGPAT5	ENSG00000155189.11	9	10	1.1
protein_coding	NFXL1	ENSG00000170448.11	9	10	1.1
protein_coding	OPHN1	ENSG00000079482.12	9	10	1.1
protein_coding	AP1S1	ENSG00000106367.14	9	10	1.1
protein_coding	FAM161B	ENSG00000156050.8	9	10	1.1
protein_coding	ICAM3	ENSG00000076662.9	9	10	1.1
protein_coding	TEFM	ENSG00000172171.10	9	10	1.1
protein_coding	TICAM1	ENSG00000127666.9	9	10	1.1
protein_coding	CCDC74A	ENSG00000163040.14	9	10	1.1
protein_coding	SLC35G1	ENSG00000176273.14	9	10	1.1
protein_coding	CASTOR1	ENSG00000239282.7	9	10	1.1
protein_coding	MRFAP1	ENSG00000179010.14	1018	1131	1.1
protein_coding	RPS26	ENSG00000197728.11	289	321	1.1
protein_coding	SSBP1	ENSG00000106028.10	226	251	1.1
protein_coding	RAN	ENSG00000132341.11	561	623	1.1
protein_coding	NBR1	ENSG00000188554.13	471	523	1.1
protein_coding	HNRNPH2	ENSG00000126945.8	308	342	1.1
protein_coding	MGAT4B	ENSG00000161013.16	464	515	1.1
protein_coding	PANK2	ENSG00000125779.22	73	81	1.1
protein_coding	PTPN9	ENSG00000169410.9	73	81	1.1
protein_coding	MFSD1	ENSG00000118855.18	192	213	1.1

protein_coding	CPSF2	ENSG00000165934.12	128	142	1.1
protein_coding	LGALS8	ENSG00000116977.18	165	183	1.1
protein_coding	FAM111A	ENSG00000166801.15	55	61	1.1
protein_coding	YAP1	ENSG00000137693.13	211	234	1.1
protein_coding	MORF4L1	ENSG00000185787.14	819	908	1.1
protein_coding	ARID3A	ENSG00000116017.10	83	92	1.1
protein_coding	PIK3R1	ENSG00000145675.14	240	266	1.1
protein_coding	IK	ENSG00000113141.17	222	246	1.1
protein_coding	GFM2	ENSG00000164347.17	37	41	1.1
antisense	OTUD6B-	ENSG00000253738.1	65	72	1.1
protein_coding	KHDC4	ENSG00000132680.10	65	72	1.1
protein_coding	WDR5	ENSG00000196363.9	65	72	1.1
protein_coding	UPF1	ENSG00000005007.12	140	155	1.1
protein_coding	PPP1R8	ENSG00000117751.17	56	62	1.1
antisense	KANSL1-AS1	ENSG00000214401.4	28	31	1.1
protein_coding	SMAD4	ENSG00000141646.13	103	114	1.1
protein_coding	RECQL	ENSG00000004700.15	75	83	1.1
protein_coding	SPIN1	ENSG00000106723.16	272	301	1.1
protein_coding	VCP	ENSG00000165280.15	441	488	1.1
protein_coding	CDK19	ENSG00000155111.14	94	104	1.1
protein_coding	GBP3	ENSG00000117226.11	94	104	1.1
protein_coding	CRYZ	ENSG00000116791.13	254	281	1.1
protein_coding	RAD17	ENSG00000152942.18	66	73	1.1
protein_coding	FAM172A	ENSG00000113391.18	85	94	1.1
protein_coding	CUL3	ENSG00000036257.12	189	209	1.1
protein_coding	PRDX5	ENSG00000126432.13	189	209	1.1
protein_coding	RAB11A	ENSG00000103769.9	303	335	1.1
protein_coding	WBP2	ENSG00000132471.11	199	220	1.1
protein_coding	GLB1	ENSG00000170266.15	171	189	1.1
protein_coding	ERGIC3	ENSG00000125991.19	152	168	1.1
protein_coding	LOXL3	ENSG00000115318.11	114	126	1.1
protein_coding	BAG4	ENSG00000156735.10	76	84	1.1
protein_coding	ACTR1B	ENSG00000115073.7	76	84	1.1
protein_coding	NHP2	ENSG00000145912.8	57	63	1.1
protein_coding	ACER3	ENSG00000078124.11	38	42	1.1
protein_coding	SHROOM2	ENSG00000146950.12	19	21	1.1
antisense	AC009948.1	ENSG00000223960.6	19	21	1.1
lincRNA	MAPKAPK5-AS1	ENSG00000234608.7	19	21	1.1
protein_coding	INPP5E	ENSG00000148384.12	19	21	1.1
protein_coding	GBA2	ENSG00000070610.14	19	21	1.1
protein_coding	MINDY2	ENSG00000128923.10	105	116	1.1
protein_coding	VAPA	ENSG00000101558.13	344	380	1.1

protein_coding	PAPOLA	ENSG00000090060.17	336	371	1.1
protein_coding	NCOA6	ENSG00000198646.13	96	106	1.1
protein_coding	TBCD	ENSG00000141556.20	48	53	1.1
protein_coding	EVA1B	ENSG00000142694.6	202	223	1.1
protein_coding	CCNI	ENSG00000118816.9	1146	1265	1.1
protein_coding	SDF4	ENSG00000078808.16	907	1001	1.1
protein_coding	DDX6	ENSG00000110367.12	550	607	1.1
protein_coding	WASHC5	ENSG00000164961.15	87	96	1.1
protein_coding	RNF6	ENSG00000127870.16	58	64	1.1
protein_coding	ELL	ENSG00000105656.12	29	32	1.1
protein_coding	MANEA	ENSG00000172469.15	29	32	1.1
protein_coding	DENND1B	ENSG00000213047.12	29	32	1.1
protein_coding	EIF4EBP2	ENSG00000148730.6	155	171	1.1
protein_coding	CYCS	ENSG00000172115.8	97	107	1.1
protein_coding	SLC30A5	ENSG00000145740.18	97	107	1.1
protein_coding	ALAS1	ENSG00000023330.14	68	75	1.1
protein_coding	CDS2	ENSG00000101290.13	68	75	1.1
protein_coding	MBD1	ENSG00000141644.17	78	86	1.1
protein_coding	DDHD1	ENSG00000100523.14	39	43	1.1
protein_coding	KLHL22	ENSG00000099910.16	39	43	1.1
protein_coding	AK2	ENSG00000004455.16	88	97	1.1
protein_coding	NNT	ENSG00000112992.16	88	97	1.1
protein_coding	GUK1	ENSG00000143774.16	186	205	1.1
protein_coding	TNRC6B	ENSG00000100354.20	284	313	1.1
protein_coding	AP1G1	ENSG00000166747.12	147	162	1.1
protein_coding	PMM1	ENSG00000100417.11	98	108	1.1
protein_coding	CADM1	ENSG00000182985.17	49	54	1.1
protein_coding	CASD1	ENSG00000127995.16	49	54	1.1
protein_coding	TPGS2	ENSG00000134779.14	246	271	1.1
protein_coding	GPBP1	ENSG00000062194.15	128	141	1.1
protein_coding	UBAP1	ENSG00000165006.13	79	87	1.1
protein_coding	PLXNA3	ENSG00000130827.6	79	87	1.1
protein_coding	IFI16	ENSG00000163565.18	456	502	1.1
protein_coding	ANKFY1	ENSG00000185722.17	129	142	1.1
protein_coding	MYCBP2	ENSG00000005810.17	170	187	1.1
protein_coding	MRPL34	ENSG00000130312.6	150	165	1.1
protein_coding	RNF146	ENSG00000118518.15	120	132	1.1
protein_coding	ADIPOR2	ENSG00000006831.9	80	88	1.1
protein_coding	FCHO2	ENSG00000157107.13	40	44	1.1
protein_coding	NUBP2	ENSG00000095906.16	40	44	1.1
protein_coding	SYMPK	ENSG00000125755.18	30	33	1.1
lincRNA	LINC01615	ENSG00000223485.2	30	33	1.1
protein_coding	TOMM40	ENSG00000130204.12	20	22	1.1
protein_coding	ZNF304	ENSG00000131845.14	20	22	1.1

protein_coding	KANSL1L	ENSG00000144445.16	20	22	1.1
lincRNA	AC114284.1	ENSG00000248927.1	10	11	1.1
protein_coding	ABCD1	ENSG00000101986.11	10	11	1.1
protein_coding	ZNF845	ENSG00000213799.12	10	11	1.1
transcribed_processed_pseudogene	RPS27AP16	ENSG00000224631.4	10	11	1.1
protein_coding	COMTD1	ENSG00000165644.10	10	11	1.1
lincRNA	AC008915.2	ENSG00000260136.5	10	11	1.1
protein_coding	PCDHB16	ENSG00000272674.3	10	11	1.1
protein_coding	CA11	ENSG00000063180.8	10	11	1.1
protein_coding	FAM117A	ENSG00000121104.7	10	11	1.1
protein_coding	CRELD1	ENSG00000163703.17	10	11	1.1
protein_coding	TMEM160	ENSG00000130748.6	10	11	1.1
protein_coding	HRCT1	ENSG00000196196.2	10	11	1.1
protein_coding	GINM1	ENSG00000055211.13	331	364	1.1
protein_coding	ATP5F1E	ENSG00000124172.9	825	907	1.1
protein_coding	FARP1	ENSG00000152767.16	161	177	1.1
protein_coding	ERC1	ENSG00000082805.19	141	155	1.1
protein_coding	TOB2	ENSG00000183864.4	141	155	1.1
protein_coding	CALM1	ENSG00000198668.10	1502	1651	1.1
protein_coding	DCAF8	ENSG00000132716.18	81	89	1.1
protein_coding	ZCRB1	ENSG00000139168.7	315	346	1.1
protein_coding	POLR2H	ENSG00000163882.9	51	56	1.1
protein_coding	OTUD7B	ENSG00000264522.5	51	56	1.1
protein_coding	PDHX	ENSG00000110435.11	51	56	1.1
protein_coding	IDE	ENSG00000119912.16	51	56	1.1
protein_coding	COX7B	ENSG00000131174.5	286	314	1.1
protein_coding	RPS3A	ENSG00000145425.9	419	460	1.1
protein_coding	BEX3	ENSG00000166681.13	1444	1585	1.1
protein_coding	TAF2	ENSG00000064313.11	41	45	1.1
protein_coding	NOLC1	ENSG00000166197.16	41	45	1.1
protein_coding	ERICH1	ENSG00000104714.13	41	45	1.1
protein_coding	NADSYN1	ENSG00000172890.12	41	45	1.1
protein_coding	UQCRB	ENSG00000156467.9	1222	1341	1.1
protein_coding	ZKSCAN1	ENSG00000106261.16	411	451	1.1
protein_coding	RRAGA	ENSG00000155876.5	350	384	1.1
protein_coding	MFAP1	ENSG00000140259.6	93	102	1.1
protein_coding	TFB1M	ENSG00000029639.10	31	34	1.1
protein_coding	ZCCHC3	ENSG00000247315.3	31	34	1.1
protein_coding	UNK	ENSG00000132478.9	31	34	1.1
protein_coding	CNOT6	ENSG00000113300.11	31	34	1.1
protein_coding	TUBA4A	ENSG00000127824.13	31	34	1.1
protein_coding	ARPC1A	ENSG00000241685.9	166	182	1.1

protein_coding	HSPD1	ENSG00000144381.16	218	239	1.1
protein_coding	MED10	ENSG00000133398.3	135	148	1.1
protein_coding	SECISBP2	ENSG00000187742.14	52	57	1.1
protein_coding	PIN1	ENSG00000127445.13	52	57	1.1
protein_coding	EIF4E	ENSG00000151247.12	73	80	1.1
protein_coding	CCNG2	ENSG00000138764.14	168	184	1.1
protein_coding	NDUFB10	ENSG00000140990.14	168	184	1.1
protein_coding	PIN4	ENSG00000102309.12	105	115	1.1
protein_coding	C3orf38	ENSG00000179021.9	42	46	1.1
protein_coding	FN3KRP	ENSG00000141560.14	21	23	1.1
protein_coding	BRMS1	ENSG00000174744.13	21	23	1.1
protein_coding	TRIM35	ENSG00000104228.12	21	23	1.1
protein_coding	COX10	ENSG00000006695.10	21	23	1.1
protein_coding	PET100	ENSG00000229833.9	21	23	1.1
protein_coding	GK5	ENSG00000175066.15	21	23	1.1
protein_coding	ZNF236	ENSG00000130856.16	21	23	1.1
lincRNA	AC084033.3	ENSG00000257698.1	21	23	1.1
protein_coding	NLRC5	ENSG00000140853.15	21	23	1.1
protein_coding	CCT2	ENSG00000166226.12	200	219	1.1
protein_coding	CHID1	ENSG00000177830.17	116	127	1.1
protein_coding	NDUFB1	ENSG00000183648.9	370	405	1.1
protein_coding	MRPL27	ENSG00000108826.15	74	81	1.1
protein_coding	YKT6	ENSG00000106636.7	212	232	1.1
protein_coding	PIM1	ENSG00000137193.13	53	58	1.1
protein_coding	GLT8D2	ENSG00000120820.12	138	151	1.1
protein_coding	KLF3	ENSG00000109787.12	64	70	1.1
protein_coding	TMEM128	ENSG00000132406.11	32	35	1.1
antisense	RASSF8-AS1	ENSG00000246695.7	32	35	1.1
protein_coding	IFIT3	ENSG00000119917.13	32	35	1.1
protein_coding	IPO11	ENSG00000086200.16	32	35	1.1
protein_coding	PRCP	ENSG00000137509.10	331	362	1.1
protein_coding	ATP11C	ENSG00000101974.14	107	117	1.1
protein_coding	TMBIM4	ENSG00000155957.17	150	164	1.1
protein_coding	C11orf49	ENSG00000149179.13	43	47	1.1
protein_coding	ZDHHC20	ENSG00000180776.15	226	247	1.1
protein_coding	EHD2	ENSG00000024422.11	388	424	1.1
protein_coding	BRAT1	ENSG00000106009.15	54	59	1.1
protein_coding	NT5DC1	ENSG00000178425.13	76	83	1.1
protein_coding	PQLC3	ENSG00000162976.12	76	83	1.1
protein_coding	IL13RA1	ENSG00000131724.10	241	263	1.1
protein_coding	RPS6	ENSG00000137154.12	3660	3994	1.1
protein_coding	RCC2	ENSG00000179051.13	340	371	1.1
protein_coding	UBR2	ENSG00000024048.10	110	120	1.1
protein_coding	BRWD1	ENSG00000185658.13	88	96	1.1

protein_coding	FBXO25	ENSG00000147364.16	66	72	1.1
protein_coding	UBE2G1	ENSG00000132388.12	55	60	1.1
protein_coding	SIAH2	ENSG00000181788.3	33	36	1.1
protein_coding	GORASP1	ENSG00000114745.13	33	36	1.1
protein_coding	SPCS2	ENSG00000118363.11	33	36	1.1
protein_coding	BRPF3	ENSG00000096070.19	33	36	1.1
protein_coding	FASTKD2	ENSG00000118246.13	33	36	1.1
protein_coding	CTPS1	ENSG00000171793.14	22	24	1.1
protein_coding	AGO4	ENSG00000134698.10	22	24	1.1
lincRNA	LINC00909	ENSG00000264247.1	22	24	1.1
protein_coding	PUSL1	ENSG00000169972.11	22	24	1.1
protein_coding	ZNF559	ENSG00000188321.13	11	12	1.1
protein_coding	CSTF2	ENSG00000101811.13	11	12	1.1
protein_coding	ADPRM	ENSG00000170222.11	11	12	1.1
protein_coding	CROCC	ENSG00000058453.16	11	12	1.1
protein_coding	ZNF449	ENSG00000173275.12	11	12	1.1
protein_coding	MED17	ENSG00000042429.11	11	12	1.1
protein_coding	TAF9B	ENSG00000187325.4	11	12	1.1
protein_coding	ZKSCAN3	ENSG00000189298.13	11	12	1.1
sense_overlapping	AL049838.1	ENSG00000259969.1	11	12	1.1
protein_coding	PSTK	ENSG00000179988.14	11	12	1.1
protein_coding	HOMER2	ENSG00000103942.12	11	12	1.1
protein_coding	ENAH	ENSG00000154380.17	696	759	1.1
protein_coding	ARL6IP1	ENSG00000170540.14	144	157	1.1
protein_coding	CD9	ENSG00000010278.13	634	691	1.1
protein_coding	CAB39	ENSG00000135932.10	89	97	1.1
protein_coding	TUBA1A	ENSG00000167552.13	523	570	1.1
protein_coding	PCNX4	ENSG00000126773.12	156	170	1.1
protein_coding	CDK13	ENSG00000065883.15	78	85	1.1
protein_coding	MYD88	ENSG00000172936.13	78	85	1.1
protein_coding	PIEZO2	ENSG00000154864.12	78	85	1.1
protein_coding	TMEM9B	ENSG00000175348.10	212	231	1.1
protein_coding	OGA	ENSG00000198408.13	134	146	1.1
protein_coding	SMG1	ENSG00000157106.16	67	73	1.1
protein_coding	SNRPB	ENSG00000125835.18	112	122	1.1
protein_coding	TUBA1B	ENSG00000123416.15	527	574	1.1
protein_coding	FASN	ENSG00000169710.8	101	110	1.1
protein_coding	LZTS2	ENSG00000107816.17	146	159	1.1
protein_coding	PHLDA3	ENSG00000174307.6	135	147	1.1
protein_coding	MT-ND1	ENSG00000198888.2	1981	2157	1.1
protein_coding	ZMYM4	ENSG00000146463.11	124	135	1.1
protein_coding	MYDGF	ENSG00000074842.7	553	602	1.1
protein_coding	TBC1D2B	ENSG00000167202.11	79	86	1.1
protein_coding	TAF1B	ENSG00000115750.16	34	37	1.1

protein_coding	SH3RF1	ENSG00000154447.14	34	37	1.1
protein_coding	C1orf43	ENSG00000143612.20	455	495	1.1
protein_coding	TPST2	ENSG00000128294.15	114	124	1.1
protein_coding	COL5A3	ENSG00000080573.6	57	62	1.1
protein_coding	PAICS	ENSG00000128050.8	103	112	1.1
protein_coding	SRSF7	ENSG00000115875.18	69	75	1.1
protein_coding	RAD1	ENSG00000113456.18	46	50	1.1
protein_coding	GTF3C2	ENSG00000115207.13	46	50	1.1
processed_transcript	AL391244.1	ENSG00000224870.6	46	50	1.1
protein_coding	ZNF697	ENSG00000143067.4	23	25	1.1
protein_coding	SPIDR	ENSG00000164808.16	23	25	1.1
protein_coding	PLEKHB2	ENSG00000115762.16	196	213	1.1
protein_coding	ABHD12	ENSG00000100997.18	127	138	1.1
protein_coding	PPP4R3B	ENSG00000275052.4	104	113	1.1
protein_coding	WASHC3	ENSG00000120860.10	104	113	1.1
protein_coding	COX7A1	ENSG00000161281.10	81	88	1.1
protein_coding	NAA38	ENSG00000183011.13	81	88	1.1
protein_coding	SGSH	ENSG00000181523.12	174	189	1.1
protein_coding	DPP9	ENSG00000142002.17	58	63	1.1
protein_coding	EXOC1	ENSG00000090989.17	105	114	1.1
protein_coding	NAAA	ENSG00000138744.14	35	38	1.1
protein_coding	INTS5	ENSG00000185085.2	35	38	1.1
protein_coding	THRAP3	ENSG00000054118.14	339	368	1.1
protein_coding	RBM6	ENSG00000004534.14	82	89	1.1
protein_coding	SS18	ENSG00000141380.13	94	102	1.1
protein_coding	GRIA3	ENSG00000125675.17	47	51	1.1
protein_coding	UVRAG	ENSG00000198382.8	47	51	1.1
protein_coding	FBXL5	ENSG00000118564.14	153	166	1.1
protein_coding	CDK6	ENSG00000105810.9	271	294	1.1
protein_coding	GLO1	ENSG00000124767.6	165	179	1.1
protein_coding	PDXK	ENSG00000160209.18	118	128	1.1
protein_coding	POGLUT1	ENSG00000163389.11	59	64	1.1
protein_coding	DCAF13	ENSG00000164934.13	59	64	1.1
protein_coding	TBCA	ENSG00000171530.13	426	462	1.1
protein_coding	RTCA	ENSG00000137996.12	83	90	1.1
protein_coding	LATS2	ENSG00000150457.8	131	142	1.1
protein_coding	NDUFA6	ENSG00000184983.10	310	336	1.1
protein_coding	SUPT16H	ENSG00000092201.9	167	181	1.1
protein_coding	TM9SF2	ENSG00000125304.9	502	544	1.1
protein_coding	HIGD1A	ENSG00000181061.13	144	156	1.1
protein_coding	TTC19	ENSG00000011295.15	72	78	1.1
protein_coding	HACD2	ENSG00000206527.9	60	65	1.1
protein_coding	TMEM242	ENSG00000215712.10	60	65	1.1
protein_coding	IDI1	ENSG00000067064.11	60	65	1.1

protein_coding	PMPCA	ENSG00000165688.11	36	39	1.1
protein_coding	TIMM10B	ENSG00000132286.11	36	39	1.1
antisense	ST20-AS1	ENSG00000259642.2	24	26	1.1
protein_coding	MTRF1L	ENSG00000112031.15	24	26	1.1
protein_coding	ZNF25	ENSG00000175395.15	24	26	1.1
protein_coding	LRRC14	ENSG00000160959.7	24	26	1.1
protein_coding	TFB2M	ENSG00000162851.7	24	26	1.1
protein_coding	GRAMD4	ENSG00000075240.16	24	26	1.1
protein_coding	CLDN23	ENSG00000253958.1	24	26	1.1
protein_coding	SLC25A30	ENSG00000174032.16	12	13	1.1
protein_coding	KLC2	ENSG00000174996.11	12	13	1.1
protein_coding	C16orf91	ENSG00000174109.4	12	13	1.1
protein_coding	SHMT1	ENSG00000176974.19	12	13	1.1
protein_coding	MDM1	ENSG00000111554.14	12	13	1.1
protein_coding	GBP1	ENSG00000117228.9	12	13	1.1
lincRNA	LINC01089	ENSG00000212694.8	12	13	1.1
protein_coding	ARHGAP26	ENSG00000145819.16	12	13	1.1
protein_coding	IMMP1L	ENSG00000148950.10	12	13	1.1
protein_coding	BAIAP2L1	ENSG00000006453.13	12	13	1.1
protein_coding	MAGEE1	ENSG00000198934.4	12	13	1.1
protein_coding	DSTN	ENSG00000125868.15	1936	2097	1.1
protein_coding	AAK1	ENSG00000115977.18	217	235	1.1
protein_coding	CPXM2	ENSG00000121898.12	425	460	1.1
protein_coding	AGO2	ENSG00000123908.11	85	92	1.1
protein_coding	COPB1	ENSG00000129083.12	304	329	1.1
protein_coding	RDX	ENSG00000137710.15	464	502	1.1
protein_coding	FDFT1	ENSG00000079459.12	171	185	1.1
protein_coding	TWSG1	ENSG00000128791.11	600	649	1.1
protein_coding	NMT1	ENSG00000136448.11	184	199	1.1
protein_coding	LEPROTL1	ENSG00000104660.18	135	146	1.1
protein_coding	NIPSNAP2	ENSG00000146729.9	86	93	1.1
protein_coding	RRM2B	ENSG00000048392.11	185	200	1.1
protein_coding	H2AFJ	ENSG00000246705.4	74	80	1.1
protein_coding	RHOT1	ENSG00000126858.17	37	40	1.1
protein_coding	DNAJC18	ENSG00000170464.9	37	40	1.1
protein_coding	MAIP1	ENSG00000162972.10	37	40	1.1
protein_coding	LAMTOR5	ENSG00000134248.13	173	187	1.1
lincRNA	EPB41L4A-	ENSG00000224032.6	136	147	1.1
protein_coding	ITGAE	ENSG00000083457.11	62	67	1.1
protein_coding	SASH1	ENSG00000111961.17	460	497	1.1
protein_coding	ZFP91	ENSG00000186660.14	175	189	1.1
protein_coding	TXLNA	ENSG00000084652.15	75	81	1.1
protein_coding	NVL	ENSG00000143748.17	25	27	1.1
protein_coding	HSD17B4	ENSG00000133835.15	113	122	1.1

protein_coding	RAB3D	ENSG00000105514.7	38	41	1.1
protein_coding	ARHGAP10	ENSG00000071205.11	38	41	1.1
protein_coding	TNFRSF10B	ENSG00000120889.12	267	288	1.1
protein_coding	CETN3	ENSG00000153140.8	89	96	1.1
protein_coding	RUFY3	ENSG00000018189.12	102	110	1.1
protein_coding	UBA6	ENSG00000033178.12	166	179	1.1
protein_coding	NSF	ENSG00000073969.18	115	124	1.1
protein_coding	ANAPC5	ENSG00000089053.12	115	124	1.1
protein_coding	CHTF8	ENSG00000168802.12	115	124	1.1
protein_coding	RPS12	ENSG00000112306.7	7013	7559	1.1
protein_coding	AFF1	ENSG00000172493.20	90	97	1.1
protein_coding	TMEM14A	ENSG00000096092.5	117	126	1.1
protein_coding	SDHD	ENSG00000204370.10	91	98	1.1
protein_coding	WHAMM	ENSG00000156232.7	39	42	1.1
protein_coding	MYPOP	ENSG00000176182.5	39	42	1.1
protein_coding	COX19	ENSG00000240230.5	26	28	1.1
protein_coding	C3orf14	ENSG00000114405.10	26	28	1.1
protein_coding	MIER3	ENSG00000155545.19	26	28	1.1
protein_coding	HSPA14	ENSG00000284024.2	26	28	1.1
protein_coding	HIST1H2BC	ENSG00000180596.7	13	14	1.1
protein_coding	ZNF747	ENSG00000169955.7	13	14	1.1
protein_coding	NUDCD1	ENSG00000120526.10	13	14	1.1
protein_coding	NEIL1	ENSG00000140398.13	13	14	1.1
protein_coding	CHCHD5	ENSG00000125611.15	13	14	1.1
protein_coding	PSMD11	ENSG00000108671.10	248	267	1.1
protein_coding	SLC35E1	ENSG00000127526.14	118	127	1.1
protein_coding	TSPAN9	ENSG00000011105.13	197	212	1.1
protein_coding	PDPR	ENSG00000090857.13	92	99	1.1
protein_coding	AEN	ENSG00000181026.14	79	85	1.1
protein_coding	SLC38A2	ENSG00000134294.13	1330	1431	1.1
protein_coding	H1FX	ENSG00000184897.5	198	213	1.1
protein_coding	KDM5C	ENSG00000126012.11	66	71	1.1
protein_coding	TRAFD1	ENSG00000135148.11	66	71	1.1
protein_coding	CD47	ENSG00000196776.15	357	384	1.1
protein_coding	CMTM6	ENSG00000091317.7	544	585	1.1
protein_coding	RPL4	ENSG00000174444.14	1557	1674	1.1
protein_coding	ILF2	ENSG00000143621.16	173	186	1.1
protein_coding	B3GNT9	ENSG00000237172.3	80	86	1.1
protein_coding	MKRN2	ENSG00000075975.15	80	86	1.1
protein_coding	ABCC1	ENSG00000103222.18	214	230	1.1
protein_coding	CEP170	ENSG00000143702.15	107	115	1.1
protein_coding	API5	ENSG00000166181.12	67	72	1.1
protein_coding	NID1	ENSG00000116962.14	202	217	1.1
protein_coding	SMCHD1	ENSG00000101596.15	81	87	1.1

protein_coding	BCAS2	ENSG00000116752.5	54	58	1.1
protein_coding	RHOT2	ENSG00000140983.13	54	58	1.1
protein_coding	MAP3K11	ENSG00000173327.7	27	29	1.1
protein_coding	ARRDC4	ENSG00000140450.8	27	29	1.1
protein_coding	ADAT1	ENSG00000065457.10	27	29	1.1
protein_coding	CBLN3	ENSG00000139899.10	27	29	1.1
protein_coding	HIGD2A	ENSG00000146066.2	136	146	1.1
protein_coding	LTBR	ENSG00000111321.10	150	161	1.1
protein_coding	SLC16A7	ENSG00000118596.11	41	44	1.1
protein_coding	UBR4	ENSG00000127481.14	41	44	1.1
protein_coding	ATIC	ENSG00000138363.14	137	147	1.1
protein_coding	FBXO22	ENSG00000167196.13	96	103	1.1
protein_coding	DDX1	ENSG00000079785.14	247	265	1.1
protein_coding	CDIPT	ENSG00000103502.13	247	265	1.1
protein_coding	PPFIA1	ENSG00000131626.18	110	118	1.1
protein_coding	CHRAC1	ENSG00000104472.9	55	59	1.1
protein_coding	ACTN4	ENSG00000130402.11	551	591	1.1
protein_coding	ATP6V1B2	ENSG00000147416.10	138	148	1.1
protein_coding	CACNA1C	ENSG00000151067.21	69	74	1.1
protein_coding	SMIM10L1	ENSG00000256537.4	152	163	1.1
protein_coding	NDUFA8	ENSG00000119421.6	83	89	1.1
protein_coding	CBX6	ENSG00000183741.11	514	551	1.1
protein_coding	GSTM3	ENSG00000134202.10	139	149	1.1
protein_coding	SPTLC1	ENSG00000090054.14	237	254	1.1
protein_coding	VPS4B	ENSG00000119541.9	112	120	1.1
protein_coding	POLR2G	ENSG00000168002.11	112	120	1.1
protein_coding	DHX38	ENSG00000140829.11	98	105	1.1
protein_coding	TCAIM	ENSG00000179152.19	56	60	1.1
protein_coding	PLA2G12A	ENSG00000123739.10	42	45	1.1
protein_coding	SMIM19	ENSG00000176209.11	42	45	1.1
protein_coding	MED23	ENSG00000112282.17	42	45	1.1
protein_coding	VTI1A	ENSG00000151532.13	28	30	1.1
protein_coding	ZFPL1	ENSG00000162300.12	28	30	1.1
protein_coding	ZNF880	ENSG00000221923.8	28	30	1.1
protein_coding	THEMIS2	ENSG00000130775.15	28	30	1.1
protein_coding	RASSF1	ENSG00000068028.17	14	15	1.1
protein_coding	ZNF180	ENSG00000167384.10	14	15	1.1
protein_coding	SARS2	ENSG00000104835.14	14	15	1.1
lincRNA	AC005332.3	ENSG00000274561.1	14	15	1.1
protein_coding	RNF213	ENSG00000173821.19	155	166	1.1
protein_coding	JAGN1	ENSG00000171135.14	99	106	1.1
protein_coding	BBX	ENSG00000114439.18	425	455	1.1
protein_coding	KARS	ENSG00000065427.14	156	167	1.1
protein_coding	TSHZ3	ENSG00000121297.6	71	76	1.1

protein_coding	RBM42	ENSG00000126254.11	57	61	1.1
protein_coding	SUMF1	ENSG00000144455.13	100	107	1.1
protein_coding	PPIB	ENSG00000166794.4	258	276	1.1
protein_coding	IPO5	ENSG00000065150.18	172	184	1.1
protein_coding	UNC5B	ENSG00000107731.12	86	92	1.1
protein_coding	CDC73	ENSG00000134371.12	144	154	1.1
protein_coding	SDCCAG8	ENSG00000054282.15	72	77	1.1
protein_coding	FAM171A1	ENSG00000148468.16	72	77	1.1
protein_coding	PFDN4	ENSG00000101132.9	202	216	1.1
protein_coding	FBRS	ENSG00000156860.15	101	108	1.1
protein_coding	ZNF428	ENSG00000131116.11	101	108	1.1
protein_coding	FADS3	ENSG00000221968.8	101	108	1.1
protein_coding	CCNY	ENSG00000108100.17	159	170	1.1
protein_coding	COQ10B	ENSG00000115520.8	87	93	1.1
protein_coding	TFE3	ENSG00000068323.16	87	93	1.1
protein_coding	TRAPPC12	ENSG00000171853.15	58	62	1.1
protein_coding	ARL4D	ENSG00000175906.4	58	62	1.1
protein_coding	TEX264	ENSG00000164081.12	29	31	1.1
protein_coding	PORCN	ENSG00000102312.21	29	31	1.1
protein_coding	DHFR2	ENSG00000178700.7	29	31	1.1
protein_coding	POP5	ENSG00000167272.10	29	31	1.1
protein_coding	TAPBP	ENSG00000231925.11	320	342	1.1
protein_coding	RERE	ENSG00000142599.17	102	109	1.1
protein_coding	FUT11	ENSG00000196968.10	146	156	1.1
protein_coding	NPLOC4	ENSG00000182446.13	73	78	1.1
protein_coding	EFR3A	ENSG00000132294.14	161	172	1.1
protein_coding	CPSF1	ENSG00000071894.16	44	47	1.1
protein_coding	PEBP1	ENSG00000089220.4	1498	1600	1.1
protein_coding	SPAG7	ENSG00000091640.7	191	204	1.1
protein_coding	ITPA	ENSG00000125877.12	103	110	1.1
protein_coding	UBA1	ENSG00000130985.16	236	252	1.1
protein_coding	BRD4	ENSG00000141867.17	326	348	1.1
protein_coding	SP110	ENSG00000135899.17	104	111	1.1
protein_coding	FLNA	ENSG00000196924.15	477	509	1.1
protein_coding	ALKBH5	ENSG00000091542.8	105	112	1.1
protein_coding	ERLIN2	ENSG00000147475.15	90	96	1.1
protein_coding	DIXDC1	ENSG00000150764.13	75	80	1.1
protein_coding	LZIC	ENSG00000162441.11	45	48	1.1
protein_coding	WWP2	ENSG00000198373.12	45	48	1.1
protein_coding	AKIRIN2	ENSG00000135334.8	45	48	1.1
protein_coding	ZNF302	ENSG00000089335.20	30	32	1.1
protein_coding	ADCY4	ENSG00000129467.13	30	32	1.1
protein_coding	ADSL	ENSG00000239900.12	15	16	1.1
protein_coding	KIF13B	ENSG00000197892.12	15	16	1.1

protein_coding	SASS6	ENSG00000156876.9	15	16	1.1
protein_coding	TEPSIN	ENSG00000167302.10	15	16	1.1
protein_coding	TEF	ENSG00000167074.14	15	16	1.1
protein_coding	DNAJC8	ENSG00000126698.10	196	209	1.1
protein_coding	GAK	ENSG00000178950.16	76	81	1.1
protein_coding	MUM1	ENSG00000160953.15	76	81	1.1
protein_coding	TXNL4A	ENSG00000141759.14	183	195	1.1
protein_coding	ZNF395	ENSG00000186918.13	107	114	1.1
protein_coding	AGTRAP	ENSG00000177674.15	46	49	1.1
protein_coding	C6orf203	ENSG00000130349.9	46	49	1.1
protein_coding	ARHGEF40	ENSG00000165801.9	123	131	1.1
protein_coding	TNPO3	ENSG00000064419.13	200	213	1.1
protein_coding	MRC2	ENSG00000011028.13	910	969	1.1
protein_coding	NDUFB3	ENSG00000119013.8	139	148	1.1
protein_coding	CLOCK	ENSG00000134852.14	124	132	1.1
protein_coding	PDHA1	ENSG00000131828.13	62	66	1.1
protein_coding	COG6	ENSG00000133103.16	62	66	1.1
protein_coding	INO80E	ENSG00000169592.14	31	33	1.1
protein_coding	SCFD1	ENSG00000092108.20	187	199	1.1
protein_coding	PIGB	ENSG00000069943.9	78	83	1.1
protein_coding	WDR26	ENSG00000162923.15	329	350	1.1
protein_coding	SLC9A3R2	ENSG00000065054.13	47	50	1.1
protein_coding	SLC2A12	ENSG00000146411.5	47	50	1.1
protein_coding	MFGE8	ENSG00000140545.14	1445	1537	1.1
protein_coding	CLCN3	ENSG00000109572.13	189	201	1.1
protein_coding	GALNT6	ENSG00000139629.15	63	67	1.1
protein_coding	TRADD	ENSG00000102871.15	63	67	1.1
protein_coding	NUP98	ENSG00000110713.16	79	84	1.1
protein_coding	ARHGEF17	ENSG00000110237.4	190	202	1.1
protein_coding	CD2BP2	ENSG00000169217.8	111	118	1.1
protein_coding	DMAC2	ENSG00000105341.18	80	85	1.1
protein_coding	BCL6	ENSG00000113916.17	64	68	1.1
protein_coding	YIF1B	ENSG00000167645.16	64	68	1.1
protein_coding	RPA2	ENSG00000117748.9	48	51	1.1
protein_coding	CTDSPL2	ENSG00000137770.13	32	34	1.1
protein_coding	FAM3A	ENSG00000071889.16	32	34	1.1
protein_coding	PRDM8	ENSG00000152784.15	32	34	1.1
protein_coding	PLCG1	ENSG00000124181.14	32	34	1.1
transcribed_unprocessed_pseudogene	CRYBB2P1	ENSG00000100058.12	16	17	1.1
protein_coding	ANAPC10	ENSG00000164162.13	16	17	1.1
protein_coding	SMARCE1	ENSG00000073584.19	16	17	1.1
protein_coding	MIS18A	ENSG00000159055.3	16	17	1.1

protein_coding	UBIAD1	ENSG00000120942.13	16	17	1.1
protein_coding	PAFAH1B3	ENSG00000079462.7	16	17	1.1
protein_coding	RPN1	ENSG00000163902.11	449	477	1.1
protein_coding	ATP5ME	ENSG00000169020.9	353	375	1.1
protein_coding	HLA-E	ENSG00000204592.8	482	512	1.1
protein_coding	SMC4	ENSG00000113810.15	177	188	1.1
protein_coding	PIGT	ENSG00000124155.17	290	308	1.1
protein_coding	SAMD9	ENSG00000205413.7	113	120	1.1
protein_coding	TMEM184B	ENSG00000198792.12	291	309	1.1
protein_coding	MICU2	ENSG00000165487.13	65	69	1.1
protein_coding	COPA	ENSG00000122218.15	586	622	1.1
protein_coding	NCOA2	ENSG00000140396.12	49	52	1.1
protein_coding	FZD6	ENSG00000164930.11	49	52	1.1
protein_coding	SUPT7L	ENSG00000119760.15	49	52	1.1
protein_coding	RPL28	ENSG00000108107.14	1343	1425	1.1
protein_coding	GPD2	ENSG00000115159.15	115	122	1.1
protein_coding	HSF1	ENSG00000185122.10	99	105	1.1
protein_coding	ARF1	ENSG00000143761.15	1092	1158	1.1
protein_coding	ITFG1	ENSG00000129636.12	331	351	1.1
protein_coding	ACOX1	ENSG00000161533.11	149	158	1.1
protein_coding	NOL8	ENSG00000198000.11	100	106	1.1
protein_coding	WNK1	ENSG00000060237.16	469	497	1.1
protein_coding	TEX2	ENSG00000136478.7	67	71	1.1
protein_coding	CLPTM1L	ENSG00000049656.13	219	232	1.1
protein_coding	ACBD3	ENSG00000182827.8	287	304	1.1
protein_coding	VDAC1	ENSG00000213585.10	390	413	1.1
protein_coding	SF3B1	ENSG00000115524.15	272	288	1.1
antisense	LMO7-AS1	ENSG00000261105.5	255	270	1.1
protein_coding	MSL1	ENSG00000188895.11	238	252	1.1
protein_coding	TMEM14C	ENSG00000111843.13	187	198	1.1
protein_coding	HELZ	ENSG00000198265.11	102	108	1.1
protein_coding	RXRB	ENSG00000204231.10	68	72	1.1
protein_coding	CFH	ENSG00000000971.15	34	36	1.1
protein_coding	LYAR	ENSG00000145220.13	34	36	1.1
protein_coding	DUSP22	ENSG00000112679.14	34	36	1.1
protein_coding	MOB2	ENSG00000182208.13	34	36	1.1
protein_coding	NEMP1	ENSG00000166881.9	17	18	1.1
protein_coding	TARS2	ENSG00000143374.16	17	18	1.1
protein_coding	CDYL2	ENSG00000166446.14	17	18	1.1
protein_coding	GABBR1	ENSG00000204681.10	17	18	1.1
antisense	AL591895.1	ENSG00000242861.1	17	18	1.1
protein_coding	FOXO4	ENSG00000184481.16	17	18	1.1
protein_coding	ENTPD6	ENSG00000197586.12	17	18	1.1
protein_coding	CASKIN2	ENSG00000177303.9	17	18	1.1

protein_coding	ERH	ENSG00000100632.10	258	273	1.1
protein_coding	POLR2K	ENSG00000147669.10	86	91	1.1
protein_coding	STEAP1B	ENSG00000105889.15	86	91	1.1
protein_coding	NME1	ENSG00000239672.7	69	73	1.1
protein_coding	NDUFV3	ENSG00000160194.17	104	110	1.1
protein_coding	CDC23	ENSG00000094880.10	52	55	1.1
protein_coding	TRRAP	ENSG00000196367.13	52	55	1.1
protein_coding	FCHSD2	ENSG00000137478.14	52	55	1.1
protein_coding	CD99L2	ENSG00000102181.20	244	258	1.1
protein_coding	TMEM263	ENSG00000151135.9	490	518	1.1
lincRNA	AC010969.2	ENSG00000269973.1	35	37	1.1
protein_coding	DCTPP1	ENSG00000179958.8	35	37	1.1
protein_coding	MARK3	ENSG00000075413.17	158	167	1.1
protein_coding	EEF1D	ENSG00000104529.17	600	634	1.1
protein_coding	BASP1	ENSG00000176788.8	1042	1101	1.1
protein_coding	GLG1	ENSG00000090863.11	601	635	1.1
protein_coding	PFDN5	ENSG00000123349.13	549	580	1.1
protein_coding	MRPL51	ENSG00000111639.7	284	300	1.1
protein_coding	HNRNPUL2	ENSG00000214753.3	142	150	1.1
protein_coding	FOXN3	ENSG00000053254.15	517	546	1.1
protein_coding	CNPY4	ENSG00000166997.7	107	113	1.1
protein_coding	PITPNA	ENSG00000174238.14	125	132	1.1
protein_coding	SLC12A6	ENSG00000140199.11	72	76	1.1
protein_coding	VCPIP1	ENSG00000175073.7	72	76	1.1
protein_coding	SLC36A1	ENSG00000123643.12	54	57	1.1
protein_coding	SAP30BP	ENSG00000161526.14	36	38	1.1
protein_coding	SCRN2	ENSG00000141295.13	36	38	1.1
protein_coding	XPA	ENSG00000136936.10	36	38	1.1
protein_coding	ZNF561	ENSG00000171469.10	36	38	1.1
protein_coding	TMEM267	ENSG00000151881.14	36	38	1.1
protein_coding	LRCH2	ENSG00000130224.14	18	19	1.1
protein_coding	NEK4	ENSG00000114904.12	18	19	1.1
protein_coding	FAM98C	ENSG00000130244.12	18	19	1.1
protein_coding	GRWD1	ENSG00000105447.12	18	19	1.1
protein_coding	PGAP3	ENSG00000161395.13	18	19	1.1
protein_coding	EIF2AK4	ENSG00000128829.11	271	286	1.1
protein_coding	MPZL1	ENSG00000197965.11	416	439	1.1
protein_coding	ACAP2	ENSG00000114331.13	181	191	1.1
protein_coding	ATP1A1	ENSG00000163399.15	599	632	1.1
protein_coding	RPL27A	ENSG00000166441.12	3040	3207	1.1
protein_coding	SGCE	ENSG00000127990.17	164	173	1.1
protein_coding	COLGALT1	ENSG00000130309.10	201	212	1.1
protein_coding	ANPEP	ENSG00000166825.13	257	271	1.1
protein_coding	UBTD1	ENSG00000165886.4	74	78	1.1

protein_coding	ASB6	ENSG00000148331.11	37	39	1.1
protein_coding	SMCR8	ENSG00000176994.10	37	39	1.1
protein_coding	UMAD1	ENSG00000219545.11	37	39	1.1
protein_coding	MYH9	ENSG00000100345.21	2317	2442	1.1
protein_coding	TCEAL8	ENSG00000180964.16	260	274	1.1
protein_coding	HCCS	ENSG00000004961.14	93	98	1.1
protein_coding	DDX18	ENSG00000088205.12	149	157	1.1
protein_coding	PHAX	ENSG00000164902.13	168	177	1.1
protein_coding	BTG3	ENSG00000154640.14	75	79	1.1
protein_coding	MT-CO3	ENSG00000198938.2	14864	15656	1.1
protein_coding	OSTC	ENSG00000198856.12	665	700	1.1
protein_coding	DNAJC5	ENSG00000101152.10	133	140	1.1
protein_coding	EBPL	ENSG00000123179.13	95	100	1.1
protein_coding	DHX32	ENSG00000089876.11	57	60	1.1
protein_coding	POU2F1	ENSG00000143190.22	57	60	1.1
protein_coding	MED27	ENSG00000160563.13	57	60	1.1
protein_coding	PACSIN3	ENSG00000165912.15	38	40	1.1
protein_coding	TPMT	ENSG00000137364.4	38	40	1.1
protein_coding	MNT	ENSG00000070444.14	38	40	1.1
protein_coding	YPEL2	ENSG00000175155.8	19	20	1.1
protein_coding	ATAD3A	ENSG00000197785.13	19	20	1.1
protein_coding	FKBP4	ENSG00000004478.7	19	20	1.1
protein_coding	ZFAND1	ENSG00000104231.10	19	20	1.1
processed_pseudogene	RPL10AP2	ENSG00000188873.4	19	20	1.1
protein_coding	L3HYPDH	ENSG00000126790.11	19	20	1.1
protein_coding	ACVR2A	ENSG00000121989.14	19	20	1.1
protein_coding	ADAMTSL1	ENSG00000178031.16	19	20	1.1
protein_coding	MRPL20	ENSG00000242485.5	191	201	1.1
protein_coding	SH3GL1	ENSG00000141985.9	172	181	1.1
protein_coding	RPS9	ENSG00000170889.13	309	325	1.1
lincRNA	AC099786.3	ENSG00000261213.1	116	122	1.1
protein_coding	UBL4A	ENSG00000102178.12	58	61	1.1
protein_coding	BRD3OS	ENSG00000235106.9	58	61	1.1
protein_coding	SLC41A1	ENSG00000133065.10	97	102	1.1
protein_coding	ZNF207	ENSG00000010244.18	175	184	1.1
protein_coding	BLZF1	ENSG00000117475.13	39	41	1.1
protein_coding	TMEM80	ENSG00000177042.14	39	41	1.1
protein_coding	PAPSS2	ENSG00000198682.12	333	350	1.1
protein_coding	DDB1	ENSG00000167986.13	431	453	1.1
protein_coding	UTP4	ENSG00000141076.17	59	62	1.1
protein_coding	MAF¬†1	ENSG00000179632.9	138	145	1.1
protein_coding	PSMC3	ENSG00000165916.8	138	145	1.1
protein_coding	TBC1D20	ENSG00000125875.13	119	125	1.1
protein_coding	SDHA	ENSG00000073578.16	120	126	1.1

protein_coding	DVL1	ENSG00000107404.19	100	105	1.1
protein_coding	UBE2N	ENSG00000177889.9	100	105	1.1
protein_coding	ZNF37A	ENSG00000075407.18	100	105	1.1
protein_coding	ETNK1	ENSG00000139163.15	80	84	1.1
protein_coding	NEPRO	ENSG00000163608.14	80	84	1.1
protein_coding	NGDN	ENSG00000129460.15	40	42	1.1
protein_coding	RPP30	ENSG00000148688.13	40	42	1.1
protein_coding	CXorf56	ENSG00000018610.14	20	21	1.1
protein_coding	VPS18	ENSG00000104142.10	20	21	1.1
protein_coding	RPS6KA4	ENSG00000162302.12	20	21	1.1
protein_coding	COMT	ENSG00000093010.13	1009	1059	1
protein_coding	CNIH3	ENSG00000143786.7	81	85	1
protein_coding	PNKD	ENSG00000127838.13	81	85	1
protein_coding	GPAA1	ENSG00000197858.10	122	128	1
protein_coding	AP3S1	ENSG00000177879.15	143	150	1
protein_coding	SYNRG	ENSG00000275066.4	184	193	1
protein_coding	PTGR1	ENSG00000106853.19	369	387	1
protein_coding	CHEK1	ENSG00000149554.12	41	43	1
protein_coding	RPL18A	ENSG00000105640.12	308	323	1
protein_coding	PURB	ENSG00000146676.8	103	108	1
protein_coding	ERP44	ENSG00000023318.7	207	217	1
protein_coding	MFSD10	ENSG00000109736.14	145	152	1
protein_coding	NELFB	ENSG00000188986.6	83	87	1
protein_coding	KXD1	ENSG00000105700.10	125	131	1
protein_coding	CACNA2D1	ENSG00000153956.15	292	306	1
protein_coding	STK17B	ENSG00000081320.10	210	220	1
protein_coding	DCAF5	ENSG00000139990.17	147	154	1
protein_coding	KIAA2026	ENSG00000183354.11	126	132	1
protein_coding	APOOL	ENSG00000155008.13	84	88	1
protein_coding	YPEL3	ENSG00000090238.11	63	66	1
protein_coding	CYB5D2	ENSG00000167740.9	63	66	1
protein_coding	KLHL12	ENSG00000117153.15	42	44	1
protein_coding	MRPL58	ENSG00000167862.9	42	44	1
protein_coding	PHF19	ENSG00000119403.14	21	22	1
lincRNA	BAIAP2-DT	ENSG00000226137.5	21	22	1
protein_coding	TTC5	ENSG00000136319.11	21	22	1
protein_coding	LPIN3	ENSG00000132793.11	21	22	1
protein_coding	GLTP	ENSG00000139433.9	128	134	1
protein_coding	CLK1	ENSG00000013441.15	64	67	1
protein_coding	MRPS23	ENSG00000181610.12	64	67	1
protein_coding	SRRM2	ENSG00000167978.16	535	560	1
protein_coding	SET	ENSG00000119335.16	407	426	1
protein_coding	CNBP	ENSG00000169714.16	279	292	1
protein_coding	PHYH	ENSG00000107537.13	86	90	1

protein_coding	MAP2K7	ENSG00000076984.17	43	45	1
protein_coding	ANAPC16	ENSG00000166295.8	173	181	1
protein_coding	RFX7	ENSG00000181827.14	87	91	1
protein_coding	TIPRL	ENSG00000143155.12	175	183	1
protein_coding	EEF1B2	ENSG00000114942.13	768	803	1
protein_coding	SRP72	ENSG00000174780.16	484	506	1
protein_coding	RAB6A	ENSG00000175582.19	242	253	1
protein_coding	TCEA1	ENSG00000187735.13	132	138	1
protein_coding	MSH3	ENSG00000113318.9	88	92	1
protein_coding	THYN1	ENSG00000151500.14	66	69	1
protein_coding	DHX8	ENSG00000067596.11	44	46	1
protein_coding	MRPL39	ENSG00000154719.13	44	46	1
protein_coding	FBXL4	ENSG00000112234.8	44	46	1
protein_coding	C17orf80	ENSG00000141219.15	44	46	1
protein_coding	TYRO3	ENSG00000092445.11	22	23	1
protein_coding	MIPEP	ENSG00000027001.9	22	23	1
protein_coding	SLC35A1	ENSG00000164414.17	22	23	1
protein_coding	SIRT5	ENSG00000124523.15	22	23	1
protein_coding	ARPC3	ENSG00000111229.15	551	576	1
protein_coding	HDGFL3	ENSG00000166503.8	222	232	1
protein_coding	RAB12	ENSG00000206418.4	200	209	1
protein_coding	TRMT112	ENSG00000173113.6	402	420	1
protein_coding	PSIP1	ENSG00000164985.14	157	164	1
protein_coding	PSMD8	ENSG00000099341.11	225	235	1
protein_coding	IER3IP1	ENSG00000134049.5	90	94	1
protein_coding	ZFP64	ENSG00000020256.19	45	47	1
protein_coding	SLC38A7	ENSG00000103042.8	45	47	1
protein_coding	TARS	ENSG00000113407.13	451	471	1
protein_coding	LPCAT3	ENSG00000111684.10	68	71	1
protein_coding	TMEM129	ENSG00000168936.10	114	119	1
protein_coding	TLK1	ENSG00000198586.13	160	167	1
protein_coding	UBE2R2	ENSG00000107341.4	414	432	1
protein_coding	ATG3	ENSG00000144848.10	92	96	1
protein_coding	PMP22	ENSG00000109099.14	69	72	1
protein_coding	MRPL28	ENSG00000086504.16	69	72	1
protein_coding	RIDA	ENSG00000132541.10	69	72	1
protein_coding	DLG3	ENSG00000082458.11	23	24	1
protein_coding	EXOSC3	ENSG00000107371.12	23	24	1
antisense	AC099786.2	ENSG00000224149.1	23	24	1
protein_coding	HEATR3	ENSG00000155393.12	23	24	1
protein_coding	SUSD5	ENSG00000173705.8	23	24	1
protein_coding	CASC4	ENSG00000166734.19	369	385	1
protein_coding	RIPK2	ENSG00000104312.7	70	73	1
protein_coding	PPP4R3A	ENSG00000100796.17	70	73	1

protein_coding	RPS19	ENSG00000105372.7	4288	4469	1
protein_coding	CD63	ENSG00000135404.11	6099	6356	1
protein_coding	NACA	ENSG00000196531.10	1501	1564	1
protein_coding	SNX3	ENSG00000112335.14	430	448	1
protein_coding	RPL3	ENSG00000100316.15	719	749	1
protein_coding	PLAA	ENSG00000137055.14	48	50	1
protein_coding	LSM¬†6	ENSG00000164167.11	24	25	1
protein_coding	RPL13	ENSG00000167526.13	1396	1454	1
protein_coding	SDHC	ENSG00000143252.14	291	303	1
protein_coding	EPM2AIP1	ENSG00000178567.7	97	101	1
protein_coding	RPL26	ENSG00000161970.14	3917	4078	1
protein_coding	RSBN1	ENSG00000081019.13	73	76	1
protein_coding	PRPF40A	ENSG00000196504.16	560	583	1
protein_coding	KDELR1	ENSG00000105438.8	1730	1801	1
protein_coding	FYTTD1	ENSG00000122068.12	123	128	1
protein_coding	MAPK3	ENSG00000102882.11	148	154	1
protein_coding	PDCL	ENSG00000136940.13	74	77	1
protein_coding	CAVIN3	ENSG00000170955.9	224	233	1
protein_coding	SNX8	ENSG00000106266.10	75	78	1
protein_coding	WRNIP1	ENSG00000124535.15	50	52	1
protein_coding	ZNF598	ENSG00000167962.13	50	52	1
protein_coding	MCRIP2	ENSG00000172366.19	25	26	1
protein_coding	CARD6	ENSG00000132357.13	25	26	1
protein_coding	YAE1	ENSG00000241127.7	25	26	1
protein_coding	RGS9	ENSG00000108370.16	25	26	1
protein_coding	TTC38	ENSG00000075234.16	25	26	1
protein_coding	RPS27A	ENSG00000143947.13	5031	5232	1
protein_coding	TRAM1	ENSG00000067167.7	958	996	1
protein_coding	SLC30A9	ENSG00000014824.13	101	105	1
protein_coding	SGCD	ENSG00000170624.13	481	500	1
protein_coding	UBE4A	ENSG00000110344.9	152	158	1
protein_coding	AFG3L2	ENSG00000141385.9	76	79	1
protein_coding	DCP1A	ENSG00000272886.5	76	79	1
protein_coding	PLXND1	ENSG00000004399.12	127	132	1
protein_coding	NEK6	ENSG00000119408.16	127	132	1
protein_coding	CHPT1	ENSG00000111666.10	153	159	1
protein_coding	TOLLIP	ENSG00000078902.15	102	106	1
protein_coding	KIF3C	ENSG00000084731.14	51	53	1
protein_coding	RPS6KC1	ENSG00000136643.11	51	53	1
protein_coding	SEC23A	ENSG00000100934.14	308	320	1
protein_coding	FKBP9	ENSG00000122642.10	154	160	1
protein_coding	MAGED1	ENSG00000179222.17	873	907	1
protein_coding	CTNNA1	ENSG00000044115.20	518	538	1
protein_coding	HEATR5A	ENSG00000129493.14	78	81	1

protein_coding	NAXE	ENSG00000163382.11	52	54	1
protein_coding	PDK1	ENSG00000152256.13	26	27	1
protein_coding	RABIF	ENSG00000183155.4	26	27	1
protein_coding	EXOSC4	ENSG00000178896.8	26	27	1
protein_coding	NUP188	ENSG00000095319.14	26	27	1
protein_coding	C12orf45	ENSG00000151131.10	26	27	1
protein_coding	ARHGAP11	ENSG00000198826.10	26	27	1
protein_coding	TRERF1	ENSG00000124496.12	26	27	1
protein_coding	SUCLG2	ENSG00000172340.14	157	163	1
protein_coding	MRPS21	ENSG00000266472.5	262	272	1
protein_coding	ARFIP2	ENSG00000132254.12	53	55	1
protein_coding	RNF41	ENSG00000181852.17	53	55	1
protein_coding	ZNF746	ENSG00000181220.16	53	55	1
protein_coding	EIF2A	ENSG00000144895.11	133	138	1
protein_coding	HP1BP3	ENSG00000127483.17	400	415	1
protein_coding	SPPL2A	ENSG00000138600.9	294	305	1
protein_coding	FHL1	ENSG00000022267.16	108	112	1
protein_coding	USB1	ENSG00000103005.11	81	84	1
protein_coding	TBC1D4	ENSG00000136111.13	27	28	1
protein_coding	KIAA1841	ENSG00000162929.13	27	28	1
protein_coding	ZNF84	ENSG00000198040.10	27	28	1
protein_coding	ZNF558	ENSG00000167785.8	27	28	1
protein_coding	HDAC1	ENSG00000116478.11	136	141	1
protein_coding	FAM189B	ENSG00000160767.20	136	141	1
protein_coding	SPCS1	ENSG00000114902.13	245	254	1
protein_coding	TMED10	ENSG00000170348.8	899	932	1
protein_coding	PSMD1	ENSG00000173692.12	545	565	1
protein_coding	POLR2F	ENSG00000100142.14	82	85	1
protein_coding	PINK1	ENSG00000158828.7	301	312	1
protein_coding	CENPB	ENSG00000125817.7	137	142	1
protein_coding	C12orf65	ENSG00000130921.7	55	57	1
protein_coding	NTN4	ENSG00000074527.11	138	143	1
protein_coding	SCAMP2	ENSG00000140497.16	138	143	1
protein_coding	FAF2	ENSG00000113194.12	221	229	1
protein_coding	PGD	ENSG00000142657.20	195	202	1
protein_coding	NCKAP1	ENSG00000061676.14	391	405	1
protein_coding	EIF3J	ENSG00000104131.12	112	116	1
protein_coding	RUFY1	ENSG00000176783.14	84	87	1
protein_coding	ATF2	ENSG00000115966.16	56	58	1
protein_coding	BABAM2	ENSG00000158019.20	56	58	1
protein_coding	TMEM223	ENSG00000168569.7	28	29	1
protein_coding	CSPP1	ENSG00000104218.14	28	29	1
protein_coding	INO80D	ENSG00000114933.15	28	29	1
protein_coding	ISOC1	ENSG00000066583.11	28	29	1

protein_coding	SLC1A4	ENSG00000115902.10	28	29	1
protein_coding	SEM1	ENSG00000127922.9	477	494	1
protein_coding	IPO9	ENSG00000198700.9	141	146	1
protein_coding	TMX1	ENSG00000139921.12	171	177	1
protein_coding	WDR75	ENSG00000115368.9	57	59	1
protein_coding	AIDA	ENSG00000186063.12	57	59	1
protein_coding	NOP14	ENSG00000087269.15	86	89	1
protein_coding	SRPRA	ENSG00000182934.11	201	208	1
protein_coding	WLS	ENSG00000116729.13	433	448	1
protein_coding	SLC35F5	ENSG00000115084.13	87	90	1
protein_coding	SLIT3	ENSG00000184347.14	58	60	1
lincRNA	LINC01943	ENSG00000280721.1	29	30	1
protein_coding	BAHD1	ENSG00000140320.11	29	30	1
protein_coding	ARMH4	ENSG00000139971.15	29	30	1
protein_coding	TCP1	ENSG00000120438.11	353	365	1
protein_coding	ZNF426	ENSG00000130818.11	59	61	1
protein_coding	IFFO1	ENSG00000010295.19	89	92	1
protein_coding	FAP	ENSG00000078098.13	268	277	1
protein_coding	MYO1D	ENSG00000176658.16	180	186	1
protein_coding	TMEM132A	ENSG00000006118.14	30	31	1
protein_coding	FBXO33	ENSG00000165355.7	151	156	1
protein_coding	COPG1	ENSG00000181789.14	151	156	1
protein_coding	YY1	ENSG00000100811.12	335	346	1
protein_coding	HNRNPLL	ENSG00000143889.15	61	63	1
protein_coding	EEF2K	ENSG00000103319.11	61	63	1
protein_coding	LMLN	ENSG00000185621.11	31	32	1
protein_coding	LY96	ENSG00000154589.6	31	32	1
protein_coding	APLP2	ENSG00000084234.17	1923	1985	1
protein_coding	CUL4A	ENSG00000139842.14	187	193	1
protein_coding	C14orf132	ENSG00000227051.6	157	162	1
protein_coding	SOX4	ENSG00000124766.6	1980	2043	1
protein_coding	GFM1	ENSG00000168827.14	63	65	1
protein_coding	TMEM59	ENSG00000116209.11	928	957	1
protein_coding	GMPR2	ENSG00000100938.17	96	99	1
protein_coding	ABCB7	ENSG00000131269.16	64	66	1
protein_coding	COL11A1	ENSG00000060718.21	64	66	1
protein_coding	SCAF4	ENSG00000156304.14	32	33	1
protein_coding	NOL4L	ENSG00000197183.14	32	33	1
protein_coding	GPR153	ENSG00000158292.6	32	33	1
protein_coding	SCRN3	ENSG00000144306.14	32	33	1
transcribed_unitary_pseudogene	TMEM198B	ENSG00000182796.14	32	33	1
protein_coding	ADGRL1	ENSG00000072071.16	32	33	1

protein_coding	GOLGA7	ENSG00000147533.16	161	166	1
protein_coding	TAP1	ENSG00000168394.11	65	67	1
protein_coding	CCDC43	ENSG00000180329.13	65	67	1
protein_coding	HDGF	ENSG00000143321.18	457	471	1
protein_coding	SEC61A1	ENSG00000058262.9	426	439	1
protein_coding	AK3	ENSG00000147853.16	427	440	1
protein_coding	TMA7	ENSG00000232112.3	230	237	1
protein_coding	TMEM173	ENSG00000184584.12	99	102	1
protein_coding	TIMM22	ENSG00000177370.4	33	34	1
protein_coding	DHTKD1	ENSG00000181192.11	33	34	1
protein_coding	CDH13	ENSG00000140945.16	133	137	1
protein_coding	YIPF4	ENSG00000119820.10	233	240	1
protein_coding	GOSR1	ENSG00000108587.15	100	103	1
protein_coding	FNTA	ENSG00000168522.12	100	103	1
protein_coding	OAZ1	ENSG00000104904.12	2336	2406	1
protein_coding	GNA11	ENSG00000088256.8	235	242	1
protein_coding	EIF4G2	ENSG00000110321.16	2955	3043	1
protein_coding	GNB1	ENSG00000078369.17	672	692	1
protein_coding	CDC42BPB	ENSG00000198752.10	203	209	1
protein_coding	MIEF1	ENSG00000100335.14	102	105	1
sense_intronic	GABPB1-IT1	ENSG00000285410.1	34	35	1
protein_coding	TRIM32	ENSG00000119401.10	34	35	1
protein_coding	TRAPPC11	ENSG00000168538.15	34	35	1
protein_coding	MTPAP	ENSG00000107951.14	34	35	1
protein_coding	FGD1	ENSG00000102302.7	34	35	1
protein_coding	DNAJB1	ENSG00000132002.7	103	106	1
protein_coding	NDUFB4	ENSG00000065518.7	517	532	1
protein_coding	CDKN1B	ENSG00000111276.10	138	142	1
protein_coding	SERP1	ENSG00000120742.10	350	360	1
protein_coding	SIVA1	ENSG00000184990.12	105	108	1
protein_coding	POLR2C	ENSG00000102978.12	105	108	1
protein_coding	MFSD5	ENSG00000182544.8	105	108	1
protein_coding	TAF12	ENSG00000120656.11	70	72	1
protein_coding	HPS5	ENSG00000110756.17	35	36	1
protein_coding	PUS3	ENSG00000110060.8	35	36	1
protein_coding	PSMF1	ENSG00000125818.17	211	217	1
protein_coding	ORMDL3	ENSG00000172057.9	141	145	1
protein_coding	CYB561A3	ENSG00000162144.9	142	146	1
protein_coding	SOD1	ENSG00000142168.14	783	805	1
protein_coding	PSME3	ENSG00000131467.10	107	110	1
protein_coding	ZNF518B	ENSG00000178163.7	72	74	1
protein_coding	FBRSL1	ENSG00000112787.13	36	37	1
protein_coding	ZFP82	ENSG00000181007.8	36	37	1
protein_coding	FZD7	ENSG00000155760.2	181	186	1

protein_coding	STRIP1	ENSG00000143093.14	73	75	1
protein_coding	ZNF358	ENSG00000198816.6	73	75	1
protein_coding	DIAPH2	ENSG00000147202.17	110	113	1
protein_coding	CREBRF	ENSG00000164463.12	258	265	1
protein_coding	UBE3C	ENSG00000009335.17	185	190	1
protein_coding	CISD1	ENSG00000122873.11	148	152	1
protein_coding	SFT2D1	ENSG00000198818.9	111	114	1
protein_coding	FOCAD	ENSG00000188352.12	111	114	1
protein_coding	GALNT10	ENSG00000164574.15	74	76	1
protein_coding	KCTD17	ENSG00000100379.17	37	38	1
protein_coding	ATG2B	ENSG00000066739.11	75	77	1
protein_coding	SLC25A38	ENSG00000144659.12	75	77	1
protein_coding	SLC38A1	ENSG00000111371.15	114	117	1
protein_coding	IQCE	ENSG00000106012.17	38	39	1
protein_coding	CCDC69	ENSG00000198624.12	38	39	1
protein_coding	CREBBP	ENSG00000005339.14	192	197	1
protein_coding	NUDT9	ENSG00000170502.12	77	79	1
protein_coding	CYB5R3	ENSG00000100243.20	1120	1149	1
protein_coding	ATXN7L3	ENSG00000087152.15	78	80	1
protein_coding	MRTO4	ENSG00000053372.4	39	40	1
protein_coding	TMEM167B	ENSG00000215717.5	118	121	1
protein_coding	CANX	ENSG00000127022.14	1223	1254	1
protein_coding	EXT1	ENSG00000182197.11	474	486	1
protein_coding	USO1	ENSG00000138768.14	160	164	1
protein_coding	LARP4B	ENSG00000107929.14	80	82	1
protein_coding	DLAT	ENSG00000150768.15	40	41	1
protein_coding	BOD1	ENSG00000145919.10	121	124	1
protein_coding	ZNF280D	ENSG00000137871.20	41	42	1
protein_coding	ALAD	ENSG00000148218.15	165	169	1
protein_coding	DNAAF5	ENSG00000164818.15	83	85	1
protein_coding	PIM3	ENSG00000198355.4	125	128	1
protein_coding	KIAA2013	ENSG00000116685.15	125	128	1
protein_coding	WDR59	ENSG00000103091.14	42	43	1
protein_coding	OARD1	ENSG00000124596.16	42	43	1
protein_coding	COQ5	ENSG00000110871.14	42	43	1
protein_coding	BLOC1S2	ENSG00000196072.11	169	173	1
protein_coding	FBXO7	ENSG00000100225.17	170	174	1
protein_coding	RPS6KA3	ENSG00000177189.13	128	131	1
protein_coding	RECK	ENSG00000122707.11	427	437	1
protein_coding	B4GALT7	ENSG00000027847.13	43	44	1
protein_coding	NDUFS7	ENSG00000115286.19	43	44	1
protein_coding	GRHPR	ENSG00000137106.17	218	223	1
protein_coding	WSB2	ENSG00000176871.8	131	134	1
protein_coding	THY1	ENSG00000154096.13	2142	2191	1

protein_coding	SNW1	ENSG00000100603.13	132	135	1
protein_coding	TRMT10C	ENSG00000174173.6	44	45	1
protein_coding	TP53BP1	ENSG00000067369.13	177	181	1
protein_coding	GGA2	ENSG00000103365.15	45	46	1
protein_coding	GALT	ENSG00000213930.11	45	46	1
protein_coding	PPP1R9B	ENSG00000108819.10	45	46	1
protein_coding	C1orf174	ENSG00000198912.10	45	46	1
protein_coding	HNRNPD	ENSG00000138668.18	181	185	1
protein_coding	ALG5	ENSG00000120697.8	136	139	1
protein_coding	CMPK1	ENSG00000162368.13	363	371	1
protein_coding	ASAH1	ENSG00000104763.18	365	373	1
protein_coding	SKI	ENSG00000157933.9	229	234	1
protein_coding	SPRYD3	ENSG00000167778.8	46	47	1
protein_coding	EZR	ENSG00000092820.17	46	47	1
protein_coding	ERP29	ENSG00000089248.6	277	283	1
protein_coding	ZYX	ENSG00000159840.15	185	189	1
protein_coding	KAT2B	ENSG00000114166.7	47	48	1
protein_coding	CALM3	ENSG00000160014.16	621	634	1
protein_coding	RABGAP1L	ENSG00000152061.23	48	49	1
protein_coding	ZNF570	ENSG00000171827.10	48	49	1
protein_coding	ARCN1	ENSG00000095139.13	290	296	1
protein_coding	DRAM1	ENSG00000136048.13	145	148	1
protein_coding	SNRPE	ENSG00000182004.12	97	99	1
protein_coding	DESI2	ENSG00000121644.18	98	100	1
protein_coding	HUS1	ENSG00000136273.12	49	50	1
protein_coding	NME3	ENSG00000103024.7	49	50	1
protein_coding	TPRG1L	ENSG00000158109.14	198	202	1
protein_coding	UBR3	ENSG00000144357.16	101	103	1
protein_coding	UBE2E1	ENSG00000170142.11	354	361	1
protein_coding	POLB	ENSG00000070501.11	51	52	1
protein_coding	SLC25A11	ENSG00000108528.13	51	52	1
protein_coding	BNIP2	ENSG00000140299.11	256	261	1
protein_coding	SETD3	ENSG00000183576.12	103	105	1
protein_coding	SENP6	ENSG00000112701.17	155	158	1
protein_coding	EPC1	ENSG00000120616.15	155	158	1
protein_coding	ARHGDIA	ENSG00000141522.11	207	211	1
protein_coding	TM2D3	ENSG00000184277.12	156	159	1
protein_coding	BTD	ENSG00000169814.13	52	53	1
protein_coding	ABHD13	ENSG00000139826.5	52	53	1
protein_coding	NOB1	ENSG00000141101.12	157	160	1
protein_coding	SAP30L	ENSG00000164576.11	105	107	1
protein_coding	NXN	ENSG00000167693.16	212	216	1
protein_coding	MAP2K1	ENSG00000169032.9	106	108	1
protein_coding	FAM98A	ENSG00000119812.18	106	108	1

protein_coding	DEAF1	ENSG00000177030.16	53	54	1
protein_coding	STRADB	ENSG00000082146.12	53	54	1
protein_coding	TM2D2	ENSG00000169490.16	160	163	1
protein_coding	MRPS15	ENSG00000116898.11	107	109	1
protein_coding	TCERG1	ENSG00000113649.11	161	164	1
protein_coding	FAM193A	ENSG00000125386.15	54	55	1
protein_coding	NR1H2	ENSG00000131408.13	109	111	1
protein_coding	SETD5	ENSG00000168137.15	279	284	1
protein_coding	DUSP1	ENSG00000120129.5	56	57	1
protein_coding	COG4	ENSG00000103051.18	56	57	1
protein_coding	RBM26	ENSG00000139746.15	170	173	1
protein_coding	RASA2	ENSG00000155903.11	57	58	1
protein_coding	KEAP1	ENSG00000079999.13	172	175	1
protein_coding	SNX2	ENSG00000205302.6	173	176	1
protein_coding	NSA2	ENSG00000164346.9	760	773	1
protein_coding	NFYB	ENSG00000120837.7	59	60	1
protein_coding	DOCK5	ENSG00000147459.17	60	61	1
protein_coding	NIPSNAP3A	ENSG00000136783.9	60	61	1
protein_coding	PHF5A	ENSG00000100410.7	60	61	1
protein_coding	DNAJC13	ENSG00000138246.16	182	185	1
protein_coding	NSFL1C	ENSG00000088833.17	61	62	1
protein_coding	TOM1L2	ENSG00000175662.17	62	63	1
protein_coding	STOML2	ENSG00000165283.15	130	132	1
protein_coding	VPS37A	ENSG00000155975.9	65	66	1
protein_coding	SNAP23	ENSG00000092531.9	131	133	1
protein_coding	CAPRIN1	ENSG00000135387.20	267	271	1
protein_coding	CBL	ENSG00000110395.6	67	68	1
protein_coding	PBDC1	ENSG00000102390.10	67	68	1
protein_coding	PRKAB2	ENSG00000131791.7	67	68	1
protein_coding	DBI	ENSG00000155368.16	479	486	1
protein_coding	TMED3	ENSG00000166557.12	276	280	1
protein_coding	MAP1LC3B	ENSG00000140941.12	623	632	1
protein_coding	TSPAN6	ENSG00000000003.14	70	71	1
protein_coding	NIPAL2	ENSG00000104361.9	70	71	1
protein_coding	ARPC1B	ENSG00000130429.14	71	72	1
protein_coding	PYCR1	ENSG00000183010.16	71	72	1
protein_coding	PRKD3	ENSG00000115825.9	73	74	1
protein_coding	RPL18	ENSG00000063177.12	1546	1567	1
protein_coding	NOP53	ENSG00000105373.18	225	228	1
protein_coding	BRD9	ENSG00000028310.17	75	76	1
protein_coding	NMT2	ENSG00000152465.17	75	76	1
protein_coding	STX12	ENSG00000117758.13	152	154	1
protein_coding	TAF9	ENSG00000273841.4	157	159	1
protein_coding	RPL13A	ENSG00000142541.16	3404	3447	1

protein_coding	IMP3	ENSG00000177971.8	159	161	1
protein_coding	CRLS1	ENSG00000088766.11	80	81	1
protein_coding	ROMO1	ENSG00000125995.15	246	249	1
protein_coding	TIMM23	ENSG00000265354.3	82	83	1
protein_coding	BET1L	ENSG00000177951.17	84	85	1
protein_coding	PLXNB2	ENSG00000196576.14	170	172	1
protein_coding	ADNP2	ENSG00000101544.8	85	86	1
protein_coding	ARID2	ENSG00000189079.15	86	87	1
protein_coding	WASHC2A	ENSG00000099290.16	86	87	1
protein_coding	SRPK2	ENSG00000135250.16	348	352	1
protein_coding	ZC3HAV1	ENSG00000105939.12	174	176	1
protein_coding	ICE1	ENSG00000164151.11	88	89	1
protein_coding	PTAR1	ENSG00000188647.12	88	89	1
protein_coding	ACP1	ENSG00000143727.15	181	183	1
protein_coding	BIVM	ENSG00000134897.13	92	93	1
protein_coding	ZFP36	ENSG00000128016.5	94	95	1
protein_coding	HACD3	ENSG00000074696.12	378	382	1
protein_coding	ZFHX3	ENSG00000140836.16	190	192	1
protein_coding	DBNL	ENSG00000136279.20	96	97	1
protein_coding	SCD	ENSG00000099194.5	97	98	1
protein_coding	LRRC42	ENSG00000116212.14	98	99	1
protein_coding	CIAO2B	ENSG00000166595.11	200	202	1
protein_coding	GAS6	ENSG00000183087.14	1203	1215	1
protein_coding	TSG101	ENSG00000074319.12	101	102	1
protein_coding	CALU	ENSG00000128595.16	3498	3532	1
protein_coding	PRKRA	ENSG00000180228.12	106	107	1
protein_coding	GNAI2	ENSG00000114353.16	974	983	1
protein_coding	GOLGA3	ENSG00000090615.14	218	220	1
transcribed_unitary_pseudogene	ZNF271P	ENSG00000257267.3	110	111	1
protein_coding	SRP19	ENSG00000153037.14	110	111	1
protein_coding	MTCH2	ENSG00000109919.9	226	228	1
protein_coding	MARS	ENSG00000166986.14	114	115	1
protein_coding	KDELC2	ENSG00000178202.12	115	116	1
protein_coding	PRKACA	ENSG00000072062.13	117	118	1
protein_coding	DCTN5	ENSG00000166847.9	118	119	1
protein_coding	UBE2J1	ENSG00000198833.6	119	120	1
protein_coding	NUDT21	ENSG00000167005.13	119	120	1
protein_coding	PIEZO1	ENSG00000103335.22	124	125	1
protein_coding	PTMS	ENSG00000159335.15	259	261	1
protein_coding	ATP6V1C1	ENSG00000155097.11	136	137	1
protein_coding	SACM1L	ENSG00000211456.10	142	143	1
protein_coding	RPL23	ENSG00000125691.12	3318	3341	1

protein_coding	SELENOF	ENSG00000183291.16	435	438	1
protein_coding	SNAI2	ENSG00000019549.11	438	441	1
protein_coding	FAM89B	ENSG00000176973.7	155	156	1
protein_coding	ANGPT1	ENSG00000154188.9	317	319	1
protein_coding	YIPF3	ENSG00000137207.11	162	163	1
protein_coding	P4HA2	ENSG00000072682.18	331	333	1
protein_coding	ADAM9	ENSG00000168615.11	843	848	1
protein_coding	PCBP1	ENSG00000169564.6	687	691	1
protein_coding	TOX4	ENSG00000092203.13	172	173	1
protein_coding	KRT10	ENSG00000186395.7	181	182	1
protein_coding	SARAF	ENSG00000133872.13	761	765	1
protein_coding	HSPE1	ENSG00000115541.10	191	192	1
protein_coding	FKBP3	ENSG00000100442.10	191	192	1
protein_coding	HSP90AB1	ENSG00000096384.19	1969	1979	1
protein_coding	PSMB1	ENSG00000008018.8	412	414	1
protein_coding	ATP6V1F	ENSG00000128524.4	217	218	1
protein_coding	SPTBN1	ENSG00000115306.15	496	498	1
protein_coding	SYT11	ENSG00000132718.8	250	251	1
protein_coding	CHMP2B	ENSG00000083937.8	262	263	1
protein_coding	MAPK1	ENSG00000100030.14	264	265	1
protein_coding	ATP5MD	ENSG00000173915.15	537	539	1
protein_coding	POMP	ENSG00000132963.7	807	810	1
protein_coding	PDCD4	ENSG00000150593.17	329	330	1
protein_coding	HMGN1	ENSG00000205581.10	381	382	1
protein_coding	METTL9	ENSG00000197006.13	422	423	1
protein_coding	PGRMC1	ENSG00000101856.9	858	860	1
protein_coding	RPL38	ENSG00000172809.12	2186	2191	1
protein_coding	RPL35	ENSG00000136942.14	994	995	1
protein_coding	KCNK3	ENSG00000171303.6	1	1	1
protein_coding	BIRC3	ENSG00000023445.13	3	3	1
antisense	AL645568.1	ENSG00000203739.3	1	1	1
protein_coding	FOSB	ENSG00000125740.13	1	1	1
protein_coding	RECQL4	ENSG00000160957.12	1	1	1
protein_coding	BUB1	ENSG00000169679.14	1	1	1
processed_pseudogene	RPS3AP49	ENSG00000242060.1	1	1	1
processed_pseudogene	GAPDHP40	ENSG00000248626.1	1	1	1
lincRNA	SFTA1P	ENSG00000225383.7	1	1	1
protein_coding	AATK	ENSG00000181409.12	1	1	1
transcribed_processed_pseudogene	AC025048.6	ENSG00000280852.2	1	1	1
protein_coding	F12	ENSG00000131187.9	1	1	1
protein_coding	PLK1	ENSG00000166851.14	1	1	1
protein_coding	CLCN5	ENSG00000171365.16	2	2	1

protein_coding	GTF2IRD2B	ENSG00000174428.16	1	1	1
lincRNA	LINC00472	ENSG00000233237.6	1	1	1
lincRNA	VASH1-AS1	ENSG00000258301.3	2	2	1
protein_coding	CLCN2	ENSG00000114859.15	2	2	1
protein_coding	OTUD3	ENSG00000169914.5	3	3	1
protein_coding	DCLRE1B	ENSG00000118655.5	2	2	1
lincRNA	AC008608.2	ENSG00000271737.1	1	1	1
antisense	PPP1R26-AS1	ENSG00000225361.3	1	1	1
lincRNA	AC092910.3	ENSG00000242622.1	1	1	1
protein_coding	CYP27C1	ENSG00000186684.12	1	1	1
lincRNA	AC005014.2	ENSG00000272361.2	1	1	1
lincRNA	FLJ22447	ENSG00000232774.7	1	1	1
lincRNA	LINC01436	ENSG00000231106.2	1	1	1
protein_coding	BFSP1	ENSG00000125864.13	6	6	1
protein_coding	SP4	ENSG00000105866.14	9	9	1
antisense	MMP25-AS1	ENSG00000261971.7	2	2	1
protein_coding	LNX2	ENSG00000139517.8	4	4	1
protein_coding	EGR3	ENSG00000179388.8	2	2	1
lincRNA	SMIM25	ENSG00000224397.5	2	2	1
unprocessed_pseudogene	WASH8P	ENSG00000226210.3	1	1	1
protein_coding	OTUB2	ENSG00000089723.9	1	1	1
protein_coding	FANCD2	ENSG00000144554.10	2	2	1
protein_coding	HJURP	ENSG00000123485.11	2	2	1
processed_pseudogene	AP001024.1	ENSG00000240652.1	1	1	1
antisense	TRAF3IP2-	ENSG00000231889.7	1	1	1
protein_coding	COL24A1	ENSG00000171502.14	1	1	1
protein_coding	ZNF77	ENSG00000175691.8	1	1	1
processed_pseudogene	AL137784.1	ENSG00000219755.1	1	1	1
sense_intronic	AC002558.3	ENSG00000262265.2	2	2	1
protein_coding	KLF5	ENSG00000102554.13	2	2	1
processed_pseudogene	RPL21P1	ENSG00000214760.3	1	1	1
sense_intronic	AC025857.2	ENSG00000269899.1	1	1	1
processed_pseudogene	AC079250.1	ENSG00000230979.3	1	1	1
processed_pseudogene	AC009120.1	ENSG00000239763.2	1	1	1
antisense	AC027682.6	ENSG00000276075.1	1	1	1
protein_coding	CATSPERE	ENSG00000179397.17	1	1	1
protein_coding	CCDC36	ENSG00000173421.16	1	1	1
antisense	HOMER3-	ENSG00000269019.1	1	1	1
protein_coding	CDC26	ENSG00000176386.8	1	1	1
lincRNA	AC004585.1	ENSG00000266088.5	1	1	1
protein_coding	TWNK	ENSG00000107815.9	4	4	1
protein_coding	ZNF550	ENSG00000251369.8	4	4	1
protein_coding	FBXL19	ENSG00000099364.16	4	4	1
protein_coding	ZNF141	ENSG00000131127.13	3	3	1

protein_coding	SEMA3F	ENSG00000001617.11	6	6	1
protein_coding	HCN3	ENSG00000143630.9	2	2	1
protein_coding	CAPS	ENSG00000105519.15	6	6	1
sense_overlapping	AL138756.1	ENSG00000259953.1	2	2	1
protein_coding	OSCAR	ENSG00000170909.13	2	2	1
protein_coding	DLX5	ENSG00000105880.6	2	2	1
bidirectional_promoter_lncRNA	AC007240.2	ENSG00000285872.1	2	2	1
transcribed_unprocessed_pseudogene	ANKRD20A5P	ENSG00000186481.16	2	2	1
processed_pseudogene	AC025449.1	ENSG00000270558.1	3	3	1
protein_coding	NUDT8	ENSG00000167799.9	3	3	1
protein_coding	CH25H	ENSG00000138135.6	3	3	1
protein_coding	SUSD3	ENSG00000157303.10	3	3	1
protein_coding	PM20D2	ENSG00000146281.5	8	8	1
protein_coding	PACRGL	ENSG00000163138.18	4	4	1
protein_coding	DBNDD1	ENSG00000003249.13	4	4	1
sense_intronic	AC023043.4	ENSG00000274849.1	5	5	1
protein_coding	FGFR1OP2	ENSG00000111790.13	54	54	1
protein_coding	GNPNAT1	ENSG00000100522.9	55	55	1
antisense	ARHGAP5-	ENSG00000258655.2	1	1	1
processed_pseudogene	DDX50P1	ENSG00000229816.1	2	2	1
protein_coding	KIF2C	ENSG00000142945.12	1	1	1
processed_pseudogene	AL590867.2	ENSG00000218426.5	4	4	1
protein_coding	PHYHIP	ENSG00000168490.13	1	1	1
protein_coding	USP21	ENSG00000143258.15	3	3	1
protein_coding	ZNF556	ENSG00000172000.7	1	1	1
lincRNA	AC020910.4	ENSG00000274104.1	1	1	1
protein_coding	SHLD3	ENSG00000253251.2	1	1	1
processed_pseudogene	RPL14P1	ENSG00000139239.7	3	3	1
processed_transcript	PCBP1-AS1	ENSG00000179818.13	4	4	1
protein_coding	BMPR1B	ENSG00000138696.10	1	1	1
transcribed_unprocessed_pseudogene	RRP7BP	ENSG00000182841.12	1	1	1
protein_coding	OAS3	ENSG00000111331.12	2	2	1
protein_coding	TNNT1	ENSG00000105048.16	3	3	1
protein_coding	ZNF57	ENSG00000171970.12	1	1	1
protein_coding	RFX2	ENSG00000087903.12	2	2	1
lincRNA	CERNA1	ENSG00000259577.1	1	1	1
protein_coding	PAX8	ENSG00000125618.16	2	2	1
lincRNA	LINC01569	ENSG00000262468.6	2	2	1
sense_intronic	AC006213.4	ENSG00000277806.1	2	2	1
protein_coding	CST6	ENSG00000175315.2	5	5	1

transcribed_unprocessed_pseudogene	PPP4R1L	ENSG00000124224.16	10	10	1
protein_coding	PPP2R5B	ENSG00000068971.13	7	7	1
processed_pseudogene	RPL24P8	ENSG00000236801.1	1	1	1
protein_coding	SYT1	ENSG00000067715.13	1	1	1
processed_transcript	AP000873.2	ENSG00000247137.8	1	1	1
misc_RNA	RN7SKP30	ENSG00000223305.1	1	1	1
snoRNA	RF00139	ENSG00000201944.1	2	2	1
protein_coding	SORL1	ENSG00000137642.12	1	1	1
protein_coding	FRMD3	ENSG00000172159.15	1	1	1
lincRNA	RASAL2-AS1	ENSG00000224687.1	1	1	1
protein_coding	TSHZ2	ENSG00000182463.15	1	1	1
protein_coding	LRRC56	ENSG00000161328.10	1	1	1
protein_coding	GPR135	ENSG00000181619.11	2	2	1
lincRNA	AL451064.1	ENSG00000272137.1	1	1	1
antisense	CDKN2B-	ENSG00000240498.7	1	1	1
antisense	AC018797.2	ENSG00000246560.2	1	1	1
snRNA	RNU6-268P	ENSG00000201044.1	1	1	1
antisense	AC114956.1	ENSG00000248240.1	1	1	1
protein_coding	AC020915.1	ENSG00000267216.1	1	1	1
protein_coding	C9orf43	ENSG00000157653.11	1	1	1
lincRNA	AC108134.2	ENSG00000261889.1	2	2	1
protein_coding	HEYL	ENSG00000163909.7	2	2	1
TEC	AC132872.4	ENSG00000280407.2	2	2	1
antisense	SLC8A1-AS1	ENSG00000227028.6	1	1	1
antisense	AC139795.2	ENSG00000247679.2	1	1	1
protein_coding	OR11G2	ENSG00000196832.4	1	1	1
transcribed_unitary_pseudogene	SEC1P	ENSG00000232871.8	1	1	1
processed_pseudogene	AC016596.1	ENSG00000264281.3	1	1	1
protein_coding	KCND1	ENSG00000102057.9	1	1	1
protein_coding	MMP24	ENSG00000125966.9	1	1	1
antisense	AC040169.1	ENSG00000260018.1	2	2	1
protein_coding	NOTCH2NL	ENSG00000264343.5	2	2	1
transcribed_unprocessed_pseudogene	RRN3P1	ENSG00000248124.7	2	2	1
protein_coding	ZNF367	ENSG00000165244.6	2	2	1
protein_coding	ST20	ENSG00000180953.11	43	43	1
protein_coding	HDHD5	ENSG00000069998.12	19	19	1
protein_coding	NPHP4	ENSG00000131697.17	10	10	1
protein_coding	MAD2L1	ENSG00000164109.13	9	9	1
protein_coding	DDX59	ENSG00000118197.13	23	23	1
protein_coding	RDH13	ENSG00000160439.15	7	7	1

protein_coding	BRWD3	ENSG00000165288.10	21	21	1
protein_coding	PPARG	ENSG00000132170.20	13	13	1
transcribed_unprocessed_pseudogene	AL353743.1	ENSG00000165121.11	5	5	1
protein_coding	TMEM25	ENSG00000149582.15	5	5	1
protein_coding	BUD13	ENSG00000137656.11	14	14	1
protein_coding	DMXL2	ENSG00000104093.13	43	43	1
protein_coding	C5orf63	ENSG00000164241.13	4	4	1
protein_coding	RCE1	ENSG00000173653.7	8	8	1
protein_coding	PRRT1	ENSG00000204314.11	4	4	1
protein_coding	POLR3K	ENSG00000161980.5	29	29	1
protein_coding	NT5C	ENSG00000125458.6	25	25	1
protein_coding	ANKZF1	ENSG00000163516.13	32	32	1
protein_coding	GINS4	ENSG00000147536.11	3	3	1
protein_coding	PCED1B	ENSG00000179715.12	3	3	1
protein_coding	CLDND2	ENSG00000160318.6	3	3	1
protein_coding	HHLA3	ENSG00000197568.13	6	6	1
protein_coding	SCX	ENSG00000260428.2	6	6	1
sense_intronic	AL442125.1	ENSG00000276248.1	3	3	1
lincRNA	AC005838.2	ENSG00000266538.1	3	3	1
antisense	AL137186.2	ENSG00000232807.2	3	3	1
protein_coding	DAPK1	ENSG00000196730.12	3	3	1
transcribed_unprocessed_pseudogene	WHAMMP3	ENSG00000276141.4	14	14	1
protein_coding	CPOX	ENSG00000080819.7	57	57	1
protein_coding	PREX1	ENSG00000124126.13	8	8	1
protein_coding	SHC4	ENSG00000185634.11	18	18	1
protein_coding	FSD1L	ENSG00000106701.11	5	5	1
transcribed_unprocessed_pseudogene	AC132008.2	ENSG00000214135.8	5	5	1
protein_coding	ZNF786	ENSG00000197362.14	5	5	1
protein_coding	AP1G2	ENSG00000213983.11	5	5	1
protein_coding	PGAP1	ENSG00000197121.14	14	14	1
protein_coding	RHOF	ENSG00000139725.7	7	7	1
protein_coding	SPINDOC	ENSG00000168005.8	23	23	1
protein_coding	FLI1	ENSG00000151702.16	18	18	1
protein_coding	ATL2	ENSG00000119787.13	37	37	1
lincRNA	AC019069.1	ENSG00000272711.1	4	4	1
protein_coding	ZPR1	ENSG00000109917.10	44	44	1
processed_pseudogene	RPS27P21	ENSG00000240759.1	2	2	1
protein_coding	ZHX1-	ENSG00000259305.6	4	4	1
protein_coding	TADA2A	ENSG00000276234.4	18	18	1
protein_coding	DPH6	ENSG00000134146.11	8	8	1

processed_pseudogene	RPS6P25	ENSG00000240616.1	2	2	1
lincRNA	DNAJC3-DT	ENSG00000247400.3	2	2	1
TEC	AC004877.2	ENSG00000280149.1	2	2	1
antisense	AL139260.1	ENSG00000228436.2	4	4	1
protein_coding	EPS8L1	ENSG00000131037.14	6	6	1
protein_coding	NFKBIL1	ENSG00000204498.10	2	2	1
lincRNA	OLMALINC	ENSG00000235823.2	2	2	1
macro_lncRNA	HELLPAR	ENSG00000281344.1	4	4	1
processed_pseudogene	RPL13AP5	ENSG00000236552.2	4	4	1
protein_coding	INCA1	ENSG00000196388.8	2	2	1
protein_coding	PWP2	ENSG00000241945.7	2	2	1
protein_coding	RP2	ENSG00000102218.5	33	33	1
protein_coding	USP37	ENSG00000135913.10	19	19	1
antisense	THUMPD3-	ENSG00000206573.8	15	15	1
lincRNA	AP002852.1	ENSG00000253633.1	11	11	1
protein_coding	AP1S2	ENSG00000182287.14	148	148	1
protein_coding	KIAA0391	ENSG00000100890.15	7	7	1
protein_coding	EIF5A2	ENSG00000163577.7	26	26	1
protein_coding	TCHP	ENSG00000139437.17	20	20	1
protein_coding	NAGS	ENSG00000161653.10	5	5	1
antisense	LINC00517	ENSG00000259091.1	5	5	1
protein_coding	ZC2HC1C	ENSG00000119703.13	5	5	1
protein_coding	PARP6	ENSG00000137817.16	28	28	1
protein_coding	TDP1	ENSG00000042088.13	16	16	1
protein_coding	CNBD2	ENSG00000149646.12	8	8	1
protein_coding	RGP1	ENSG00000107185.9	122	122	1
protein_coding	WDR55	ENSG00000120314.18	41	41	1
processed_pseudogene	AC093591.1	ENSG00000244021.4	3	3	1
protein_coding	CENPN	ENSG00000166451.13	6	6	1
protein_coding	DNAJC28	ENSG00000177692.11	3	3	1
processed_pseudogene	AC012618.1	ENSG00000213293.4	3	3	1
lincRNA	MIATNB	ENSG00000244625.5	3	3	1
protein_coding	THAP9	ENSG00000168152.12	3	3	1
protein_coding	ROR2	ENSG00000169071.14	3	3	1
protein_coding	LIMS2	ENSG00000072163.19	6	6	1
processed_transcript	LINC00339	ENSG00000218510.8	10	10	1
protein_coding	NFYC	ENSG00000066136.20	37	37	1
protein_coding	WDR53	ENSG00000185798.7	7	7	1
protein_coding	UBL3	ENSG00000122042.9	130	130	1
protein_coding	DENND4C	ENSG00000137145.20	41	41	1
protein_coding	ARL¬†16	ENSG00000214087.8	23	23	1
protein_coding	NAT9	ENSG00000109065.11	27	27	1
protein_coding	CDK8	ENSG00000132964.11	35	35	1
sense_overlapping	AP003119.3	ENSG00000261578.1	4	4	1

protein_coding	EEFSEC	ENSG00000132394.10	4	4	1
protein_coding	ISOC2	ENSG00000063241.7	8	8	1
protein_coding	GMFB	ENSG00000197045.12	112	112	1
protein_coding	MICALL1	ENSG00000100139.13	20	20	1
lincRNA	AL109976.1	ENSG00000277287.1	4	4	1
lincRNA	BX322562.1	ENSG00000273796.1	4	4	1
protein_coding	HLA-DPA1	ENSG00000231389.7	4	4	1
protein_coding	GRASP	ENSG00000161835.10	8	8	1
protein_coding	RBM8A	ENSG00000265241.6	185	185	1
protein_coding	CDC42SE1	ENSG00000197622.12	104	104	1
protein_coding	NLGN1	ENSG00000169760.17	26	26	1
protein_coding	KDM4B	ENSG00000127663.14	87	87	1
protein_coding	TTC37	ENSG00000198677.11	200	200	1
protein_coding	PRMT3	ENSG00000185238.12	9	9	1
antisense	AL356488.3	ENSG00000273382.1	9	9	1
protein_coding	R3HDM2	ENSG00000179912.20	79	79	1
protein_coding	SCAF1	ENSG00000126461.14	5	5	1
protein_coding	REEP6	ENSG00000115255.10	5	5	1
processed_transcript	DLG5-AS1	ENSG00000233871.2	5	5	1
protein_coding	CEP95	ENSG00000258890.6	42	42	1
protein_coding	RBPJ	ENSG00000168214.20	346	346	1
protein_coding	ZNF444	ENSG00000167685.14	27	27	1
protein_coding	GMDS	ENSG00000112699.10	38	38	1
protein_coding	PRKAA1	ENSG00000132356.11	111	111	1
protein_coding	FBXL18	ENSG00000155034.18	6	6	1
antisense	AC006449.2	ENSG00000275532.1	6	6	1
protein_coding	KLHDC3	ENSG00000124702.17	62	62	1
protein_coding	LSM14B	ENSG00000149657.19	56	56	1
protein_coding	TAF5L	ENSG00000135801.9	20	20	1
protein_coding	PEX1	ENSG00000127980.15	20	20	1
transcribed_processed_pseudogene	RPL13P5	ENSG00000240370.6	7	7	1
protein_coding	MON2	ENSG00000061987.15	50	50	1
protein_coding	PIGC	ENSG00000135845.9	38	38	1
protein_coding	AP5S1	ENSG00000125843.10	39	39	1
protein_coding	PNPT1	ENSG00000138035.14	16	16	1
protein_coding	TNFRSF1B	ENSG00000028137.18	8	8	1
protein_coding	TRIP10	ENSG00000125733.17	41	41	1
protein_coding	CCDC12	ENSG00000160799.11	34	34	1
protein_coding	TRIM21	ENSG00000132109.9	18	18	1
protein_coding	NSUN5	ENSG00000130305.16	19	19	1
protein_coding	SNTB1	ENSG00000172164.14	105	105	1
protein_coding	ZNF439	ENSG00000171291.8	10	10	1

protein_coding	ZNF382	ENSG00000161298.17	10	10	1
protein_coding	CPT1C	ENSG00000169169.14	20	20	1
protein_coding	PRR3	ENSG00000204576.11	11	11	1
protein_coding	TDG	ENSG00000139372.14	34	34	1
protein_coding	ZNF514	ENSG00000144026.11	12	12	1
protein_coding	CLASRP	ENSG00000104859.14	12	12	1
protein_coding	UBE2D4	ENSG00000078967.12	13	13	1
protein_coding	CBARP	ENSG00000099625.13	14	14	1
protein_coding	FRS2	ENSG00000166225.8	45	45	1
protein_coding	TAF11	ENSG00000064995.17	80	80	1
protein_coding	SIRT2	ENSG00000068903.19	33	33	1
protein_coding	CCT5	ENSG00000150753.11	234	234	1
protein_coding	ACAP3	ENSG00000131584.18	18	18	1
protein_coding	SSRP1	ENSG00000149136.8	165	165	1
protein_coding	PPHLN1	ENSG00000134283.17	147	147	1
protein_coding	SAMD1	ENSG00000141858.11	39	39	1
protein_coding	GEMIN5	ENSG00000082516.8	21	21	1
protein_coding	CSNK1D	ENSG00000141551.14	185	185	1
protein_coding	SNX24	ENSG00000064652.10	27	27	1
protein_coding	ZNF480	ENSG00000198464.13	58	58	1
protein_coding	CGRRF1	ENSG00000100532.11	32	32	1
protein_coding	CRNKL1	ENSG00000101343.14	66	66	1
protein_coding	LMF2	ENSG00000100258.17	129	129	1
lincRNA	AL365203.2	ENSG00000273038.2	93	93	1
protein_coding	OTUD5	ENSG00000068308.13	102	102	1
protein_coding	CENPU	ENSG00000151725.11	4	4	1
protein_coding	RAB36	ENSG00000100228.12	4	4	1
protein_coding	ATRIP	ENSG00000164053.20	7	7	1
antisense	SNHG12	ENSG00000197989.14	10	10	1
processed_pseudogene	RPL7P6	ENSG00000227525.4	1	1	1
antisense	AC106795.2	ENSG00000249684.5	2	2	1
processed_pseudogene	RPL41P5	ENSG00000256393.1	1	1	1
protein_coding	PTPRD	ENSG00000153707.16	3	3	1
protein_coding	CAPN15	ENSG00000103326.11	32	32	1
processed_pseudogene	ATP5F1EP2	ENSG00000180389.7	1	1	1
transcribed_unprocessed_pseudogene	NSUN5P2	ENSG00000106133.17	4	4	1
protein_coding	CCDC138	ENSG00000163006.11	2	2	1
protein_coding	B4GALT3	ENSG00000158850.14	40	40	1
transcribed_unprocessed_pseudogene	CLUHP3	ENSG00000131797.12	1	1	1
protein_coding	NUSAP1	ENSG00000137804.12	2	2	1
protein_coding	KLHL35	ENSG00000149243.15	1	1	1

protein_coding	SF3A3	ENSG00000183431.11	71	71	1
protein_coding	AUTS2	ENSG00000158321.16	20	20	1
protein_coding	CPSF4	ENSG00000160917.14	25	25	1
protein_coding	MSR1	ENSG00000038945.14	1	1	1
lincRNA	AC005479.2	ENSG00000270000.1	1	1	1
antisense	AC011466.1	ENSG00000268583.1	1	1	1
antisense	AC106791.1	ENSG00000250159.6	1	1	1
processed_pseudogene	MTCO1P40	ENSG00000262902.1	4	4	1
antisense	AL445524.1	ENSG00000233461.5	7	7	1
protein_coding	SERPINB2	ENSG00000197632.8	1	1	1
protein_coding	ZNF107	ENSG00000196247.11	2	2	1
protein_coding	TRMT61B	ENSG00000171103.10	23	23	1
protein_coding	PIH1D2	ENSG00000150773.10	1	1	1
protein_coding	CSMD2	ENSG00000121904.17	1	1	1
protein_coding	LIPE	ENSG00000079435.9	4	4	1
transcribed_unitary_pseudogene	FER1L4	ENSG00000088340.15	2	2	1
protein_coding	ZC3HC1	ENSG00000091732.16	21	21	1
antisense	BX322234.1	ENSG00000226445.1	1	1	1
processed_pseudogene	FAM133CP	ENSG00000183055.5	6	6	1
processed_pseudogene	AC110749.1	ENSG00000244086.1	2	2	1
protein_coding	EFNA3	ENSG00000143590.13	2	2	1
protein_coding	MBD6	ENSG00000166987.14	34	34	1
protein_coding	CADM4	ENSG00000105767.2	1	1	1
antisense	AC011472.4	ENSG00000273733.1	1	1	1
protein_coding	ZNF429	ENSG00000197013.9	1	1	1
lincRNA	AC012603.1	ENSG00000271849.1	1	1	1
bidirectional_promoter_lncRNA	TIPARP-AS1	ENSG00000243926.1	1	1	1
processed_transcript	AC017116.2	ENSG00000285596.1	2	2	1
processed_transcript	BTG3-AS1	ENSG00000280594.1	2	2	1
antisense	AC022382.1	ENSG00000269886.1	2	2	1
lincRNA	AC018638.6	ENSG00000271344.1	2	2	1
protein_coding	CALCRL	ENSG00000064989.12	3	3	1
protein_coding	ABLIM1	ENSG00000099204.19	4	4	1
protein_coding	KIAA0895	ENSG00000164542.12	2	2	1
protein_coding	ZNF585A	ENSG00000196967.10	18	18	1
protein_coding	GPR160	ENSG00000173890.16	1	1	1
protein_coding	PRICKLE3	ENSG00000012211.12	1	1	1
protein_coding	CCDC121	ENSG00000176714.9	1	1	1
antisense	AC073332.1	ENSG00000237773.6	3	3	1
antisense	NOP14-AS1	ENSG00000249673.6	10	10	1
protein_coding	TMEM86B	ENSG00000180089.5	5	5	1

transcribed_unitary_pseudogene	ZNRD1ASP	ENSG00000204623.9	3	3	1
protein_coding	KLHL25	ENSG00000183655.12	5	5	1
protein_coding	SRCAP	ENSG00000080603.16	3	3	1
antisense	AL049597.2	ENSG00000261737.1	1	1	1
protein_coding	ATP8A1	ENSG00000124406.16	2	2	1
processed_pseudogene	MORF4L1P1	ENSG00000218283.2	4	4	1
antisense	AC005840.4	ENSG00000276718.1	1	1	1
antisense	AC062029.1	ENSG00000234028.3	1	1	1
transcribed_processed_pseudogene	RPSAP21	ENSG00000226498.2	1	1	1
antisense	AL360270.3	ENSG00000273010.1	1	1	1
protein_coding	PILRB	ENSG00000121716.20	2	2	1
protein_coding	RBM34	ENSG00000188739.14	2	2	1
sense_overlapping	AL513534.1	ENSG00000260400.1	1	1	1
lincRNA	AF165147.1	ENSG00000232855.6	2	2	1
processed_transcript	AC009120.2	ENSG00000259972.2	6	6	1
transcribed_unprocessed_pseudogene	ZNF788P	ENSG00000214189.9	1	1	1
lincRNA	AL359715.3	ENSG00000272129.1	1	1	1
sense_intronic	AC008764.7	ENSG00000269578.1	1	1	1
sense_intronic	AC015871.3	ENSG00000278600.1	1	1	1
sense_overlapping	AC008669.1	ENSG00000260686.1	2	2	1
processed_pseudogene	PDCD5P1	ENSG00000255909.1	1	1	1
protein_coding	MMP12	ENSG00000262406.2	1	1	1
protein_coding	SH2D3C	ENSG00000095370.19	1	1	1
antisense	HDAC2-AS2	ENSG00000228624.7	1	1	1
protein_coding	PPP1R36	ENSG00000165807.7	1	1	1
protein_coding	PIPOX	ENSG00000179761.11	1	1	1
lincRNA	AC009120.5	ENSG00000275236.1	2	2	1
lincRNA	AC000403.1	ENSG00000278727.1	2	2	1
protein_coding	SOAT2	ENSG00000167780.11	2	2	1
protein_coding	SPATA9	ENSG00000145757.15	3	3	1
lincRNA	AL627171.1	ENSG00000278002.1	1	1	1
protein_coding	VEGFD	ENSG00000165197.4	1	1	1
antisense	OBSCN-AS1	ENSG00000162913.9	1	1	1
antisense	AF111167.2	ENSG00000259319.1	1	1	1
antisense	ADPGK-AS1	ENSG00000260898.5	1	1	1
antisense	AC005790.1	ENSG00000274447.1	1	1	1
lincRNA	AL365184.2	ENSG00000230015.3	1	1	1
lincRNA	LINC01956	ENSG00000258910.3	1	1	1
lincRNA	AC063944.3	ENSG00000272597.1	1	1	1
lincRNA	LINC02015	ENSG00000231574.5	1	1	1

lincRNA	AC091932.1	ENSG00000260981.1	1	1	1
lincRNA	LINC00574	ENSG00000231690.2	1	1	1
lincRNA	LINC00908	ENSG00000263812.5	1	1	1
lincRNA	HAR1A	ENSG00000225978.3	1	1	1
protein_coding	SYPL2	ENSG00000143028.8	1	1	1
protein_coding	FCER1G	ENSG00000158869.10	1	1	1
protein_coding	ARMC12	ENSG00000157343.8	1	1	1
protein_coding	GCK	ENSG00000106633.15	1	1	1
protein_coding	BATF2	ENSG00000168062.9	1	1	1
protein_coding	SLC26A10	ENSG00000135502.17	1	1	1
sense_intronic	AC121493.1	ENSG00000270059.1	1	1	1
sense_intronic	AC011726.2	ENSG00000253430.1	1	1	1
sense_intronic	AC023449.2	ENSG00000274307.1	1	1	1
TEC	AC003973.3	ENSG00000279377.1	1	1	1
transcribed_unprocessed_pseudogene	AC009237.3	ENSG00000229689.3	1	1	1
sense_intronic	AC079174.1	ENSG00000258355.1	1	1	1
protein_coding	CFAP45	ENSG00000213085.9	1	1	1
lincRNA	AC067747.1	ENSG00000273153.1	1	1	1
lincRNA	AL355512.1	ENSG00000273143.1	1	1	1
processed_pseudogene	RAC1P2	ENSG00000249936.3	1	1	1
3prime_overlapping_ncRNA	AL137127.1	ENSG00000272084.1	1	1	1
TEC	AC068491.4	ENSG00000279191.1	1	1	1
antisense	DCST1-AS1	ENSG00000232093.1	1	1	1
processed_pseudogene	OSTCP4	ENSG00000220924.4	1	1	1
protein_coding	BATF	ENSG00000156127.6	2	2	1
protein_coding	WBP2NL	ENSG00000183066.14	2	2	1
sense_intronic	AC124017.1	ENSG00000249341.1	2	2	1
processed_pseudogene	AC026462.1	ENSG00000234337.4	2	2	1
antisense	AC096586.1	ENSG00000272936.1	2	2	1
antisense	ATP2A1-AS1	ENSG00000260442.5	3	3	1
transcribed_unprocessed_pseudogene	FAAHP1	ENSG00000232022.7	3	3	1
protein_coding	CDK15	ENSG00000138395.14	4	4	1
protein_coding	MYOCD	ENSG00000141052.17	4	4	1
antisense	AC080038.1	ENSG00000274565.1	5	5	1
protein_coding	DIRAS1	ENSG00000176490.4	10	10	1
protein_coding	BEX4	ENSG00000102409.9	18	18	1
protein_coding	TMEM230	ENSG00000089063.14	243	243	1
protein_coding	RBM10	ENSG00000182872.15	48	48	1
protein_coding	KMT2A	ENSG00000118058.21	134	134	1
protein_coding	NUTF2	ENSG00000102898.11	504	504	1

protein_coding	ATP6AP2	ENSG00000182220.14	767	767	1
protein_coding	ATXN7L3B	ENSG00000253719.3	84	84	1
protein_coding	MRPL22	ENSG00000082515.17	76	76	1
protein_coding	IP6K1	ENSG00000176095.11	49	49	1
protein_coding	RAD18	ENSG00000070950.9	23	23	1
protein_coding	ZBTB25	ENSG00000089775.11	22	22	1
protein_coding	PPIL3	ENSG00000240344.8	40	40	1
protein_coding	SGK3	ENSG00000104205.13	19	19	1
protein_coding	P4HTM	ENSG00000178467.17	18	18	1
protein_coding	PLPP2	ENSG00000141934.9	18	18	1
protein_coding	NDUFB2	ENSG00000090266.12	325	325	1
protein_coding	CCDC28B	ENSG00000160050.14	15	15	1
protein_coding	NBPF19	ENSG00000271383.6	14	14	1
protein_coding	CHST12	ENSG00000136213.9	89	89	1
protein_coding	APOPT1	ENSG00000256053.7	49	49	1
protein_coding	ZFP37	ENSG00000136866.13	11	11	1
protein_coding	DEPDC7	ENSG00000121690.10	11	11	1
protein_coding	EGFL7	ENSG00000172889.15	11	11	1
protein_coding	PGPEP1	ENSG00000130517.13	118	118	1
protein_coding	RPRD2	ENSG00000163125.15	84	84	1
protein_coding	EXOC2	ENSG00000112685.13	50	50	1
protein_coding	TMEM91	ENSG00000142046.14	10	10	1
protein_coding	IFNGR1	ENSG00000027697.14	148	148	1
protein_coding	ZNF623	ENSG00000183309.11	29	29	1
protein_coding	CTU2	ENSG00000174177.12	9	9	1
protein_coding	HSCB	ENSG00000100209.9	35	35	1
protein_coding	IFIT2	ENSG00000119922.9	17	17	1
protein_coding	ERO1B	ENSG00000086619.13	16	16	1
protein_coding	PLPP6	ENSG00000205808.5	16	16	1
protein_coding	STARD9	ENSG00000159433.11	15	15	1
protein_coding	PITX1	ENSG00000069011.15	15	15	1
protein_coding	NT5DC3	ENSG00000111696.11	14	14	1
protein_coding	C16orf58	ENSG00000140688.16	42	42	1
protein_coding	MIIP	ENSG00000116691.10	7	7	1
protein_coding	CORO6	ENSG00000167549.18	7	7	1
protein_coding	AKNA	ENSG00000106948.16	27	27	1
protein_coding	SLC25A16	ENSG00000122912.14	25	25	1
protein_coding	EP400	ENSG00000183495.13	90	90	1
protein_coding	ARMCX5	ENSG00000125962.14	12	12	1
protein_coding	MUC1	ENSG00000185499.16	6	6	1
protein_coding	PLPP5	ENSG00000147535.16	132	132	1
protein_coding	LSM¬†1	ENSG00000175324.9	149	149	1
protein_coding	SRBD1	ENSG00000068784.12	23	23	1
protein_coding	DSN1	ENSG00000149636.15	39	39	1

protein_coding	HELLS	ENSG00000119969.14	11	11	1
processed_pseudogene	AP001324.1	ENSG00000227615.1	11	11	1
protein_coding	BST1	ENSG00000109743.10	125	125	1
protein_coding	BBS4	ENSG00000140463.13	38	38	1
protein_coding	METTL6	ENSG00000206562.11	16	16	1
protein_coding	INPP5K	ENSG00000132376.19	36	36	1
protein_coding	CENPL	ENSG00000120334.15	10	10	1
protein_coding	TPRN	ENSG00000176058.12	15	15	1
antisense	AC008966.2	ENSG00000272123.1	5	5	1
lincRNA	AC142472.1	ENSG00000276728.1	5	5	1
lincRNA	GAS6-DT	ENSG00000272695.1	5	5	1
protein_coding	PEX10	ENSG00000157911.10	29	29	1
protein_coding	THOC1	ENSG00000079134.11	24	24	1
protein_coding	RBM14	ENSG00000239306.4	19	19	1
protein_coding	TMEM44	ENSG00000145014.17	14	14	1
protein_coding	EPG5	ENSG00000152223.13	51	51	1
protein_coding	ZNF641	ENSG00000167528.12	9	9	1
protein_coding	GTF2H3	ENSG00000111358.13	45	45	1
protein_coding	DUSP12	ENSG00000081721.11	40	40	1
protein_coding	TMEM147	ENSG00000105677.11	185	185	1
protein_coding	HMBS	ENSG00000256269.8	22	22	1
protein_coding	TIMM10	ENSG00000134809.8	21	21	1
protein_coding	STK36	ENSG00000163482.11	25	25	1
protein_coding	IFI6	ENSG00000126709.14	115	115	1
protein_coding	DDX11	ENSG00000013573.16	4	4	1
antisense	MAFG-DT	ENSG00000265688.1	4	4	1
protein_coding	EPHB1	ENSG00000154928.17	4	4	1
lincRNA	FLJ20021	ENSG00000254531.1	20	20	1
processed_pseudogene	AP000936.3	ENSG00000234268.1	8	8	1
protein_coding	KDM4C	ENSG00000107077.18	16	16	1
sense_intronic	AC104695.3	ENSG00000270640.1	4	4	1
antisense	AC002467.1	ENSG00000241764.3	4	4	1
protein_coding	SH3BP2	ENSG00000087266.15	16	16	1
protein_coding	NUDT13	ENSG00000166321.13	4	4	1
transcribed_unitary_pseudogene	ABHD11-AS1	ENSG00000225969.2	4	4	1
antisense	ZMIZ1-AS1	ENSG00000224596.7	4	4	1
protein_coding	COQ4	ENSG00000167113.10	38	38	1
protein_coding	LAMTOR2	ENSG00000116586.11	56	56	1
protein_coding	ANKRD52	ENSG00000139645.9	37	37	1
protein_coding	C9orf116	ENSG00000160345.12	11	11	1
protein_coding	MICU1	ENSG00000107745.18	80	80	1
protein_coding	TYMP	ENSG00000025708.13	14	14	1

transcribed_processed_pseudogene	RPL6P27	ENSG00000235552.4	7	7	1
protein_coding	CCS	ENSG00000173992.8	27	27	1
protein_coding	HEMK1	ENSG00000114735.9	10	10	1
protein_coding	MRPS27	ENSG00000113048.16	124	124	1
protein_coding	PTRH1	ENSG00000187024.14	13	13	1
protein_coding	MAT2A	ENSG00000168906.12	39	39	1
protein_coding	FGFR1OP	ENSG00000213066.12	26	26	1
protein_coding	MPND	ENSG00000008382.15	16	16	1
protein_coding	OXR1	ENSG00000164830.18	96	96	1
protein_coding	MRPL50	ENSG00000136897.7	31	31	1
protein_coding	ORC5	ENSG00000164815.10	34	34	1
antisense	AC008443.4	ENSG00000250222.1	6	6	1
protein_coding	ZNF286A	ENSG00000187607.15	3	3	1
protein_coding	TLCD1	ENSG00000160606.10	3	3	1
antisense	AP001029.2	ENSG00000267199.1	6	6	1
protein_coding	FAM173B	ENSG00000150756.13	24	24	1
lincRNA	AP002360.1	ENSG00000255135.3	3	3	1
sense_intronic	AL133243.2	ENSG00000276334.1	6	6	1
protein_coding	PAN2	ENSG00000135473.14	12	12	1
processed_pseudogene	RPL7AP11	ENSG00000242445.1	3	3	1
processed_pseudogene	AL138785.1	ENSG00000225616.2	3	3	1
protein_coding	DNAH7	ENSG00000118997.13	3	3	1
protein_coding	KLC4	ENSG00000137171.14	9	9	1
lincRNA	CDC37L1-DT	ENSG00000273061.1	3	3	1
antisense	AC093726.2	ENSG00000273183.1	3	3	1
protein_coding	TTC21A	ENSG00000168026.18	3	3	1
protein_coding	AFF3	ENSG00000144218.18	3	3	1
protein_coding	CSDC2	ENSG00000172346.14	6	6	1
protein_coding	SLC7A7	ENSG00000155465.18	21	21	1
protein_coding	CHMP1A	ENSG00000131165.14	95	95	1
protein_coding	ZNF761	ENSG00000160336.14	20	20	1
protein_coding	TRIM25	ENSG00000121060.17	114	114	1
antisense	AC018647.2	ENSG00000271122.1	17	17	1
protein_coding	WDR81	ENSG00000167716.18	17	17	1
protein_coding	CHSY3	ENSG00000198108.3	28	28	1
protein_coding	DFFB	ENSG00000169598.15	14	14	1
protein_coding	LLPH	ENSG00000139233.6	47	47	1
protein_coding	B3GALNT2	ENSG00000162885.12	11	11	1
protein_coding	NR2C2AP	ENSG00000184162.14	8	8	1
protein_coding	FAM120C	ENSG00000184083.11	24	24	1
protein_coding	WDR46	ENSG00000227057.9	29	29	1
protein_coding	ZNF184	ENSG00000096654.15	18	18	1

lincRNA	AC097468.3	ENSG00000272644.1	5	5	1
antisense	ZEB1-AS1	ENSG00000237036.4	5	5	1
protein_coding	TMEM17	ENSG00000186889.9	5	5	1
protein_coding	IFT140	ENSG00000187535.13	15	15	1
protein_coding	KCND3	ENSG00000171385.9	5	5	1
protein_coding	DIS3L	ENSG00000166938.12	26	26	1
protein_coding	BCS1L	ENSG00000074582.13	14	14	1
protein_coding	RILP	ENSG00000167705.11	16	16	1
protein_coding	MAP3K12	ENSG00000139625.12	41	41	1
protein_coding	NCAPD2	ENSG00000010292.12	54	54	1
protein_coding	SPRYD4	ENSG00000176422.13	13	13	1
protein_coding	SERPING1	ENSG00000149131.15	15	15	1
protein_coding	MTMR11	ENSG00000014914.20	37	37	1
antisense	AC083855.2	ENSG00000285906.1	2	2	1
protein_coding	KIF11	ENSG00000138160.5	12	12	1
antisense	AC114811.2	ENSG00000260641.1	8	8	1
antisense	AP000692.2	ENSG00000273199.1	2	2	1
lincRNA	PSMG3-AS1	ENSG00000230487.7	6	6	1
processed_pseudogene	RPLP0P6	ENSG00000213553.4	2	2	1
snoRNA	SNORD13E	ENSG00000238311.1	2	2	1
protein_coding	MTF2	ENSG00000143033.17	26	26	1
antisense	AP003119.2	ENSG00000255100.1	2	2	1
protein_coding	FKBP5	ENSG00000096060.14	2	2	1
protein_coding	NIPSNAP3B	ENSG00000165028.11	2	2	1
protein_coding	NUDT17	ENSG00000186364.11	4	4	1
protein_coding	NOD1	ENSG00000106100.10	6	6	1
protein_coding	PIGV	ENSG00000060642.10	28	28	1
protein_coding	FAM84A	ENSG00000162981.13	6	6	1
protein_coding	LRTOMT	ENSG00000184154.14	2	2	1
antisense	AC016065.1	ENSG00000246089.3	4	4	1
antisense	AL096865.1	ENSG00000271857.1	2	2	1
protein_coding	RAPGEFL1	ENSG00000108352.12	2	2	1
protein_coding	PCDHB11	ENSG00000197479.6	2	2	1
protein_coding	LSMEM1	ENSG00000181016.9	2	2	1
misc_RNA	RNY4P34	ENSG00000201649.1	4	4	1
protein_coding	PSORS1C1	ENSG00000204540.10	2	2	1
antisense	AL132639.2	ENSG00000258940.2	2	2	1
protein_coding	ADSSL1	ENSG00000185100.10	10	10	1
protein_coding	GRIK2	ENSG00000164418.20	2	2	1
lincRNA	AL031663.3	ENSG00000277022.1	2	2	1
protein_coding	RAB26	ENSG00000167964.12	2	2	1
lincRNA	AC090229.1	ENSG00000267397.1	2	2	1
protein_coding	TNFSF4	ENSG00000117586.10	4	4	1
antisense	AC139530.3	ENSG00000275902.1	2	2	1

protein_coding	ZNF853	ENSG00000236609.3	2	2	1
protein_coding	GPR162	ENSG00000250510.7	2	2	1
TEC	AC105749.1	ENSG00000281100.1	2	2	1
protein_coding	TMEM269	ENSG00000274386.5	2	2	1
antisense	LINC01311	ENSG00000260924.2	2	2	1
protein_coding	MSTN	ENSG00000138379.4	4	4	1
lincRNA	MIR4435-	ENSG00000172965.15	41	41	1
protein_coding	ZHX2	ENSG00000178764.7	25	25	1
protein_coding	CMTM7	ENSG00000153551.13	32	32	1
protein_coding	GAB1	ENSG00000109458.8	24	24	1
protein_coding	CENPO	ENSG00000138092.10	11	11	1
antisense	AL117332.1	ENSG00000275457.1	11	11	1
protein_coding	B4GALNT4	ENSG00000182272.11	9	9	1
lincRNA	AC100814.1	ENSG00000272010.1	9	9	1
protein_coding	MTURN	ENSG00000180354.15	36	36	1
protein_coding	NBPF20	ENSG00000162825.16	12	12	1
protein_coding	SLC44A3	ENSG00000143036.16	5	5	1
antisense	AC105285.1	ENSG00000245213.6	5	5	1
protein_coding	ZNF347	ENSG00000197937.12	15	15	1
protein_coding	USP54	ENSG00000166348.18	8	8	1
protein_coding	WNT16	ENSG00000002745.12	8	8	1
lincRNA	AC127024.5	ENSG00000266490.1	3	3	1
antisense	AL928654.1	ENSG00000251602.6	3	3	1
protein_coding	MTBP	ENSG00000172167.7	3	3	1
protein_coding	SH3D21	ENSG00000214193.10	3	3	1
protein_coding	TIGD6	ENSG00000164296.6	15	15	1
sense_intronic	AC103769.1	ENSG00000253475.1	3	3	1
protein_coding	NCKAP5	ENSG00000176771.16	6	6	1
protein_coding	ARTN	ENSG00000117407.16	3	3	1
protein_coding	C1orf74	ENSG00000162757.4	6	6	1
antisense	AC245140.2	ENSG00000280195.1	3	3	1
protein_coding	TMLHE	ENSG00000185973.10	3	3	1
protein_coding	KLHL3	ENSG00000146021.14	6	6	1
protein_coding	TSSK6	ENSG00000178093.13	3	3	1
misc_RNA	RF00019	ENSG00000200164.1	3	3	1
antisense	AC006059.1	ENSG00000230084.5	3	3	1
antisense	C10orf25	ENSG00000165511.6	3	3	1
protein_coding	CNNM1	ENSG00000119946.10	3	3	1
protein_coding	ANO2	ENSG00000047617.14	3	3	1
protein_coding	KIF5A	ENSG00000155980.11	3	3	1
protein_coding	TEX29	ENSG00000153495.10	3	3	1
protein_coding	CDH15	ENSG00000129910.7	3	3	1
protein_coding	PCDH19	ENSG00000165194.15	3	3	1
antisense	AF131216.4	ENSG00000280273.2	3	3	1

protein_coding	FAM131B	ENSG00000159784.17	3	3	1
antisense	AC018413.1	ENSG00000265778.2	3	3	1
antisense	FLG-AS1	ENSG00000237975.6	6	6	1
protein_coding	GPC3	ENSG00000147257.13	15	15	1
protein_coding	USP51	ENSG00000247746.4	10	10	1
protein_coding	KALRN	ENSG00000160145.15	7	7	1
protein_coding	TRIM68	ENSG00000167333.12	15	15	1
protein_coding	NFIA	ENSG00000162599.15	19	19	1
transcribed_unprocessed_pseudogene	FAM86C2P	ENSG00000160172.10	4	4	1
antisense	AC009118.3	ENSG00000276259.1	4	4	1
protein_coding	SH2B2	ENSG00000160999.10	4	4	1
transcribed_unprocessed_pseudogene	LINC00680	ENSG00000215190.9	4	4	1
transcribed_processed_pseudogene	AL353625.1	ENSG00000213073.4	4	4	1
protein_coding	JAK3	ENSG00000105639.18	4	4	1
protein_coding	CCDC85B	ENSG00000175602.3	4	4	1
protein_coding	KCTD16	ENSG00000183775.10	19	19	1
protein_coding	ZC3H3	ENSG00000014164.6	10	10	1
protein_coding	ETV2	ENSG00000105672.14	5	5	1
protein_coding	ZNF775	ENSG00000196456.11	10	10	1
protein_coding	SGF29	ENSG00000176476.8	10	10	1
protein_coding	SLC25A21	ENSG00000183032.11	5	5	1
protein_coding	ADPRH	ENSG00000144843.11	5	5	1
protein_coding	KCND2	ENSG00000184408.9	5	5	1
protein_coding	TGDS	ENSG00000088451.10	17	17	1
protein_coding	GOLGA6L9	ENSG00000197978.9	6	6	1
protein_coding	CEP85	ENSG00000130695.15	8	8	1
protein_coding	HIRIP3	ENSG00000149929.15	10	10	1
lincRNA	AC009283.1	ENSG00000273576.1	10	10	1
protein_coding	PCDHB9	ENSG00000177839.6	15	15	1
protein_coding	CDC45	ENSG00000093009.9	1	1	1
lincRNA	AL035461.2	ENSG00000275632.1	2	2	1
protein_coding	FANCB	ENSG00000181544.14	1	1	1
protein_coding	KIF24	ENSG00000186638.16	1	1	1
protein_coding	BLM	ENSG00000197299.11	1	1	1
unprocessed_pseudogene	PMS2P1	ENSG00000078319.9	1	1	1
protein_coding	TSEN2	ENSG00000154743.17	5	5	1
protein_coding	BCL2	ENSG00000171791.12	1	1	1
lincRNA	MANCR	ENSG00000231298.6	1	1	1
protein_coding	DNA2	ENSG00000138346.14	2	2	1
protein_coding	CEP55	ENSG00000138180.15	3	3	1

processed_pseudogene	TBCAP1	ENSG00000226781.1	1	1	1
processed_transcript	AC092944.1	ENSG00000243176.5	1	1	1
misc_RNA	RN7SL3	ENSG00000278771.1	1	1	1
lincRNA	USP46-AS1	ENSG00000248866.1	3	3	1
protein_coding	ZNF311	ENSG00000197935.6	1	1	1
protein_coding	TESMIN	ENSG00000132749.10	1	1	1
lincRNA	AC008946.1	ENSG00000267939.1	2	2	1
antisense	AL121944.1	ENSG00000272009.1	3	3	1
lincRNA	ZKSCAN2-	ENSG00000274925.1	1	1	1
sense_intronic	AC004584.3	ENSG00000263120.1	1	1	1
protein_coding	C16orf74	ENSG00000154102.10	1	1	1
protein_coding	ITGB4	ENSG00000132470.13	1	1	1
protein_coding	TROAP	ENSG00000135451.12	1	1	1
transcribed_processed_pseudogene	Z97634.1	ENSG00000236829.9	1	1	1
protein_coding	DLGAP5	ENSG00000126787.12	2	2	1
lincRNA	AP002360.3	ENSG00000272301.1	1	1	1
antisense	SPINT1-AS1	ENSG00000261183.5	1	1	1
lincRNA	AC090515.2	ENSG00000245975.2	1	1	1
protein_coding	DEPDC1B	ENSG00000035499.12	1	1	1
protein_coding	SLC47A1	ENSG00000142494.13	2	2	1
protein_coding	KDM4D	ENSG00000186280.6	1	1	1
protein_coding	DPF1	ENSG00000011332.19	1	1	1
antisense	AL355816.2	ENSG00000273221.1	1	1	1
protein_coding	GATA6	ENSG00000141448.8	1	1	1
sense_intronic	AL163051.2	ENSG00000276182.1	1	1	1
antisense	AC078795.2	ENSG00000270096.1	1	1	1
lincRNA	AC012291.2	ENSG00000285667.1	1	1	1
protein_coding	LRRC75A	ENSG00000181350.11	1	1	1
antisense	AL391988.1	ENSG00000277879.1	1	1	1
lincRNA	LINC00857	ENSG00000237523.1	1	1	1
processed_pseudogene	AC008725.1	ENSG00000270442.1	1	1	1
snRNA	RNU2-68P	ENSG00000222810.1	1	1	1
transcribed_unitary_pseudogene	AC008105.2	ENSG00000233483.3	1	1	1
protein_coding	DHRS11	ENSG00000278535.4	1	1	1
lincRNA	AC012360.3	ENSG00000272994.1	2	2	1
antisense	AL162586.1	ENSG00000225032.5	2	2	1
transcribed_processed_pseudogene	UPF3AP3	ENSG00000234709.2	4	4	1
protein_coding	PROM2	ENSG00000155066.15	1	1	1
transcribed_processed_pseudogene	DUXAP9	ENSG00000225210.10	2	2	1

protein_coding	CPA4	ENSG00000128510.11	1	1	1
protein_coding	CCDC88C	ENSG00000015133.18	2	2	1
misc_RNA	RF00019	ENSG00000200397.1	1	1	1
protein_coding	ZFHX2	ENSG00000136367.13	3	3	1
protein_coding	RTN4RL1	ENSG00000185924.6	1	1	1
processed_pseudogene	AC079922.1	ENSG00000231747.1	2	2	1
protein_coding	PCSK9	ENSG00000169174.10	1	1	1
protein_coding	TNK1	ENSG00000174292.12	1	1	1
antisense	AL163051.1	ENSG00000260806.1	1	1	1
lincRNA	AP001528.1	ENSG00000246523.7	1	1	1
antisense	AC009121.2	ENSG00000263080.1	1	1	1
processed_pseudogene	HSP90AB4P	ENSG00000282100.1	2	2	1
protein_coding	CLDN1	ENSG00000163347.5	1	1	1
transcribed_unitary_pseudogene	CCDC162P	ENSG00000203799.12	1	1	1
antisense	AC123768.3	ENSG00000262728.5	1	1	1
TEC	AL603750.1	ENSG00000280099.1	1	1	1
antisense	MIR181A2H	ENSG00000224020.1	1	1	1
antisense	UNC5B-AS1	ENSG00000237512.6	2	2	1
antisense	AL021707.6	ENSG00000272669.1	1	1	1
antisense	AF129075.2	ENSG00000273254.1	1	1	1
snRNA	RNU6-36P	ENSG00000206899.1	1	1	1
antisense	AC105020.1	ENSG00000203392.3	1	1	1
processed_transcript	LINC01619	ENSG00000257242.7	1	1	1
protein_coding	CATSPER2	ENSG00000166762.17	1	1	1
antisense	AC134043.1	ENSG00000253256.1	1	1	1
processed_pseudogene	AC004890.1	ENSG00000239719.1	1	1	1
lincRNA	AL034374.1	ENSG00000271367.1	1	1	1
lincRNA	AP000808.1	ENSG00000250508.1	1	1	1
antisense	AC005740.4	ENSG00000271871.1	1	1	1
antisense	AC008429.1	ENSG00000204758.7	1	1	1
antisense	AL078581.1	ENSG00000233330.1	1	1	1
antisense	AL139353.2	ENSG00000250365.6	1	1	1
misc_RNA	RN7SL547P	ENSG00000240584.3	1	1	1
processed_pseudogene	TMSB10P1	ENSG00000228499.1	1	1	1
processed_pseudogene	AL031777.1	ENSG00000217275.2	1	1	1
protein_coding	C16orf71	ENSG00000166246.13	1	1	1
protein_coding	DNAH2	ENSG00000183914.14	1	1	1
antisense	WEE2-AS1	ENSG00000228775.7	2	2	1
protein_coding	ZNF18	ENSG00000154957.13	2	2	1
protein_coding	VAX2	ENSG00000116035.3	2	2	1
antisense	BCDIN3D-	ENSG00000258057.5	2	2	1
processed_pseudogene	TXNP1	ENSG00000232823.2	2	2	1

misc_RNA	RNY3	ENSG00000202354.1	2	2	1
protein_coding	SERPINC1	ENSG00000117601.13	2	2	1
protein_coding	MOV10L1	ENSG00000073146.15	3	3	1
antisense	TMCC1-AS1	ENSG00000271270.6	3	3	1
antisense	AL139011.1	ENSG00000228606.2	3	3	1
lincRNA	AC084018.2	ENSG00000274292.1	1	1	1
lincRNA	LINC01394	ENSG00000281809.1	1	1	1
antisense	AL121655.1	ENSG00000271228.1	1	1	1
lincRNA	AC090510.1	ENSG00000246283.2	1	1	1
lincRNA	AC061992.1	ENSG00000266970.1	1	1	1
processed_pseudogene	AC112191.2	ENSG00000254373.1	1	1	1
processed_pseudogene	RPL10P3	ENSG00000230734.1	1	1	1
processed_pseudogene	AC023906.1	ENSG00000242327.1	1	1	1
protein_coding	GCKR	ENSG00000084734.8	1	1	1
protein_coding	CYSRT1	ENSG00000197191.4	1	1	1
antisense	AC019171.1	ENSG00000269210.2	1	1	1
antisense	AC007364.1	ENSG00000231969.1	1	1	1
antisense	PCOLCE-AS1	ENSG00000224729.5	1	1	1
antisense	AC037459.2	ENSG00000251034.1	1	1	1
antisense	AL162254.1	ENSG00000234156.1	1	1	1
antisense	MKX-AS1	ENSG00000230500.1	1	1	1
antisense	AL136301.1	ENSG00000276436.1	1	1	1
antisense	AC091133.3	ENSG00000251461.3	1	1	1
bidirectional_promoter_lncRNA	AC012470.1	ENSG00000285587.1	1	1	1
lincRNA	AL591848.4	ENSG00000260855.1	1	1	1
lincRNA	LINC00578	ENSG00000228221.5	1	1	1
lincRNA	AC007953.2	ENSG00000270321.1	1	1	1
lincRNA	AL161669.3	ENSG00000278071.1	1	1	1
lincRNA	AC005498.2	ENSG00000267421.6	1	1	1
miRNA	MIR4521	ENSG00000283160.1	1	1	1
misc_RNA	RF00100	ENSG00000274303.1	1	1	1
misc_RNA	RF00019	ENSG00000207105.1	1	1	1
processed_pseudogene	AC116917.1	ENSG00000224012.3	1	1	1
processed_pseudogene	CLNS1AP1	ENSG00000213335.4	1	1	1
processed_pseudogene	AC022690.1	ENSG00000254425.1	1	1	1
processed_pseudogene	AC008026.2	ENSG00000239809.1	1	1	1
protein_coding	PLA2G5	ENSG00000127472.10	1	1	1
protein_coding	HIST2H4A	ENSG00000270882.2	1	1	1
protein_coding	DIRC1	ENSG00000174325.5	1	1	1
protein_coding	C2orf88	ENSG00000187699.10	1	1	1
protein_coding	SLC16A14	ENSG00000163053.10	1	1	1
protein_coding	TMEM108	ENSG00000144868.13	1	1	1

protein_coding	DGKG	ENSG00000058866.14	1	1	1
protein_coding	DDIT4L	ENSG00000145358.6	1	1	1
protein_coding	NLRP10	ENSG00000182261.3	1	1	1
protein_coding	CHRDL2	ENSG00000054938.15	1	1	1
protein_coding	LRRC43	ENSG00000158113.12	1	1	1
protein_coding	KCTD4	ENSG00000180332.6	1	1	1
protein_coding	TBX4	ENSG00000121075.10	1	1	1
protein_coding	ELANE	ENSG00000197561.6	1	1	1
protein_coding	CNTD2	ENSG00000105219.9	1	1	1
protein_coding	PNMA8B	ENSG00000204851.6	1	1	1
protein_coding	MAOB	ENSG00000069535.13	1	1	1
protein_coding	ZC3H12B	ENSG00000102053.12	1	1	1
sense_intronic	AL137060.1	ENSG00000274270.1	1	1	1
sense_intronic	AC051619.7	ENSG00000259932.1	1	1	1
TEC	AC127496.7	ENSG00000280351.2	1	1	1
transcribed_processed_pseudogene	AP006222.1	ENSG00000228463.10	1	1	1
transcribed_processed_pseudogene	AC231533.2	ENSG00000233585.2	1	1	1
transcribed_unitary_pseudogene	GGTA1P	ENSG00000204136.10	1	1	1
transcribed_unprocessed_pseudogene	FAM86HP	ENSG00000253540.5	1	1	1
transcribed_unprocessed_pseudogene	ASNSP1	ENSG00000248498.4	1	1	1
unprocessed_pseudogene	AC019176.2	ENSG00000273876.1	1	1	1
lincRNA	AC097634.1	ENSG00000270562.1	1	1	1
processed_pseudogene	AL391416.1	ENSG00000219951.4	1	1	1
processed_pseudogene	AP000568.1	ENSG00000229336.1	1	1	1
antisense	GDNF-AS1	ENSG00000248587.7	1	1	1
lincRNA	AP002381.2	ENSG00000285693.1	1	1	1
protein_coding	ART5	ENSG00000167311.13	1	1	1
lincRNA	AC011481.1	ENSG00000267114.1	1	1	1
processed_pseudogene	AC092597.1	ENSG00000242262.1	1	1	1
processed_pseudogene	AL807752.1	ENSG00000213590.2	1	1	1
protein_coding	AOC3	ENSG00000131471.6	1	1	1
transcribed_unprocessed_pseudogene	FAHD2CP	ENSG00000231584.8	1	1	1
transcribed_unprocessed_pseudogene	DPY19L2P3	ENSG00000227855.3	1	1	1
antisense	AC130343.2	ENSG00000277597.1	1	1	1
sense_intronic	AC002519.1	ENSG00000259810.2	1	1	1
protein_coding	GLI1	ENSG00000111087.9	1	1	1

processed_pseudogene	AL136116.1	ENSG00000216642.1	1	1	1
protein_coding	PBX4	ENSG00000105717.13	1	1	1
bidirectional_promoter_lncRNA	LCMT1-AS1	ENSG00000260448.5	2	2	1
protein_coding	PTCH2	ENSG00000117425.13	2	2	1
protein_coding	ZCWPW2	ENSG00000206559.7	2	2	1
antisense	C21orf62-AS1	ENSG00000205930.8	2	2	1
protein_coding	KCNK12	ENSG00000184261.4	2	2	1
protein_coding	ERC2	ENSG00000187672.12	2	2	1
protein_coding	RARRES2	ENSG00000106538.9	2	2	1
protein_coding	CEND1	ENSG00000184524.5	2	2	1
protein_coding	TSPAN18	ENSG00000157570.11	2	2	1
protein_coding	SYP	ENSG00000102003.10	2	2	1
lincRNA	AL157700.1	ENSG00000260118.1	2	2	1
protein_coding	MROH8	ENSG00000101353.14	2	2	1
antisense	AL133346.1	ENSG00000227220.1	3	3	1
antisense	AC036176.1	ENSG00000267390.1	4	4	1
protein_coding	RPS4X	ENSG00000198034.10	2875	2874	1
protein_coding	DDR2	ENSG00000162733.17	823	822	1
protein_coding	NDUFS5	ENSG00000168653.10	369	368	1
protein_coding	ELOB	ENSG00000103363.14	364	363	1
protein_coding	ARF4	ENSG00000168374.10	685	683	1
protein_coding	NFE2L1	ENSG00000082641.15	327	326	1
protein_coding	METAP2	ENSG00000111142.13	544	542	1
protein_coding	C4orf3	ENSG00000164096.12	266	265	1
protein_coding	ATP5MPL	ENSG00000156411.9	435	433	1
protein_coding	IGF2R	ENSG00000197081.13	629	626	1
protein_coding	DNAJC21	ENSG00000168724.16	191	190	1
protein_coding	OSMR	ENSG00000145623.12	171	170	1
protein_coding	EEA1	ENSG00000102189.16	339	337	1
protein_coding	CRYAB	ENSG00000109846.7	335	333	1
protein_coding	TPP1	ENSG00000166340.16	311	309	1
protein_coding	CBX4	ENSG00000141582.14	148	147	1
protein_coding	LRRC47	ENSG00000130764.9	139	138	1
protein_coding	SLC25A1	ENSG00000100075.9	135	134	1
protein_coding	SMIM7	ENSG00000214046.8	134	133	1
protein_coding	EIF2AK2	ENSG00000055332.17	268	266	1
protein_coding	ATXN10	ENSG00000130638.16	266	264	1
protein_coding	UFM1	ENSG00000120686.11	265	263	1
protein_coding	ABI1	ENSG00000136754.17	132	131	1
protein_coding	SEC22C	ENSG00000093183.13	130	129	1
protein_coding	MRPL13	ENSG00000172172.7	124	123	1
protein_coding	NINJ1	ENSG00000131669.9	119	118	1

protein_coding	UBE2H	ENSG00000186591.12	475	471	1
protein_coding	UBE2D3	ENSG00000109332.19	941	933	1
protein_coding	PHB	ENSG00000167085.11	114	113	1
protein_coding	FBN1	ENSG00000166147.13	1558	1544	1
protein_coding	TMSB10	ENSG00000034510.5	6267	6210	1
protein_coding	DPH3	ENSG00000154813.9	109	108	1
protein_coding	ATP9A	ENSG00000054793.13	215	213	1
lincRNA	AC005261.1	ENSG00000268205.1	107	106	1
protein_coding	RB1	ENSG00000139687.15	105	104	1
protein_coding	ATF7IP	ENSG00000171681.12	105	104	1
protein_coding	LYST	ENSG00000143669.13	102	101	1
protein_coding	NDUFS3	ENSG00000213619.9	102	101	1
protein_coding	VPS35L	ENSG00000103544.14	102	101	1
protein_coding	MCC	ENSG00000171444.17	96	95	1
protein_coding	ECI2	ENSG00000198721.12	191	189	1
protein_coding	QRICH1	ENSG00000198218.10	94	93	1
protein_coding	DDX54	ENSG00000123064.12	92	91	1
protein_coding	FKBP1A	ENSG00000088832.16	1191	1178	1
protein_coding	LYRM2	ENSG00000083099.10	91	90	1
protein_coding	KLHL20	ENSG00000076321.10	91	90	1
protein_coding	PARP14	ENSG00000173193.14	182	180	1
protein_coding	Sep-02	ENSG00000168385.17	721	713	1
protein_coding	HEG1	ENSG00000173706.13	360	356	1
protein_coding	FBN2	ENSG00000138829.11	628	621	1
protein_coding	EMC6	ENSG00000127774.6	179	177	1
protein_coding	POFUT2	ENSG00000186866.16	179	177	1
protein_coding	STOM	ENSG00000148175.12	1056	1044	1
protein_coding	IGFBP7	ENSG00000163453.11	11352	11221	1
protein_coding	RBM17	ENSG00000134453.15	173	171	1
protein_coding	TMEM18	ENSG00000151353.14	84	83	1
protein_coding	OFD1	ENSG00000046651.14	84	83	1
lincRNA	NORAD	ENSG00000260032.1	1080	1067	1
protein_coding	MYL9	ENSG00000101335.9	1842	1819	1
protein_coding	EIF3D	ENSG00000100353.17	399	394	1
protein_coding	CAMKK2	ENSG00000110931.18	79	78	1
protein_coding	AVPI1	ENSG00000119986.6	154	152	1
protein_coding	ADD3	ENSG00000148700.14	910	898	1
protein_coding	RNF40	ENSG00000103549.21	75	74	1
protein_coding	EIF3F	ENSG00000175390.13	220	217	1
protein_coding	GTF2F1	ENSG00000125651.13	73	72	1
protein_coding	PDHB	ENSG00000168291.12	145	143	1
protein_coding	EXOSC10	ENSG00000171824.13	71	70	1
protein_coding	BLOC1S1	ENSG00000135441.7	70	69	1
protein_coding	BSDC1	ENSG00000160058.18	137	135	1

protein_coding	PRNP	ENSG00000171867.16	1226	1208	1
protein_coding	INTS12	ENSG00000138785.14	68	67	1
protein_coding	HSPA9	ENSG00000113013.13	271	267	1
protein_coding	TIMMDC1	ENSG00000113845.9	267	263	1
protein_coding	GUCD1	ENSG00000138867.16	199	196	1
protein_coding	PIH1D1	ENSG00000104872.10	66	65	1
protein_coding	TFG	ENSG00000114354.13	396	390	1
protein_coding	METTL7A	ENSG00000185432.11	131	129	1
protein_coding	ATP2C1	ENSG00000017260.19	258	254	1
protein_coding	MALT1	ENSG00000172175.13	64	63	1
protein_coding	SLC35D2	ENSG00000130958.12	64	63	1
protein_coding	PRKACB	ENSG00000142875.19	63	62	1
protein_coding	AK1	ENSG00000106992.18	63	62	1
protein_coding	CBX1	ENSG00000108468.14	187	184	1
protein_coding	ACOT7	ENSG00000097021.19	62	61	1
protein_coding	RBX1	ENSG00000100387.8	186	183	1
protein_coding	RETREG2	ENSG00000144567.10	181	178	1
protein_coding	RPS10	ENSG00000124614.15	362	356	1
protein_coding	CNOT9	ENSG00000144580.13	60	59	1
protein_coding	CITED2	ENSG00000164442.9	238	234	1
protein_coding	NSD3	ENSG00000147548.16	236	232	1
protein_coding	COX5A	ENSG00000178741.11	352	346	1
protein_coding	COPS9	ENSG00000172428.10	288	283	1
protein_coding	BMP1	ENSG00000168487.18	57	56	1
protein_coding	LARS	ENSG00000133706.17	113	111	1
protein_coding	MYO1C	ENSG00000197879.16	282	277	1
protein_coding	DPP7	ENSG00000176978.13	56	55	1
protein_coding	EP300	ENSG00000100393.12	168	165	1
protein_coding	STMN1	ENSG00000117632.22	442	434	1
protein_coding	PPP3CB	ENSG00000107758.15	109	107	1
protein_coding	ARMC8	ENSG00000114098.17	54	53	1
protein_coding	MTIF3	ENSG00000122033.14	108	106	1
protein_coding	WAC	ENSG00000095787.21	481	472	1
protein_coding	B4GALT2	ENSG00000117411.16	160	157	1
protein_coding	POP7	ENSG00000172336.4	53	52	1
protein_coding	CDC34	ENSG00000099804.8	53	52	1
protein_coding	PYGO1	ENSG00000171016.12	53	52	1
protein_coding	MKX	ENSG00000150051.13	318	312	1
protein_coding	TSPAN3	ENSG00000140391.14	628	616	1
protein_coding	SMAD5	ENSG00000113658.17	260	255	1
protein_coding	SPRED1	ENSG00000166068.12	103	101	1
protein_coding	MAN1B1	ENSG00000177239.14	103	101	1
protein_coding	UPF2	ENSG00000151461.19	103	101	1
protein_coding	RTF2	ENSG00000022277.12	153	150	1

protein_coding	FOPNL	ENSG00000133393.12	153	150	1
protein_coding	PAPSS1	ENSG00000138801.8	203	199	1
protein_coding	BCKDK	ENSG00000103507.13	50	49	1
protein_coding	FBH1	ENSG00000134452.19	50	49	1
protein_coding	SVIP	ENSG00000198168.8	50	49	1
protein_coding	KLHL8	ENSG00000145332.13	50	49	1
protein_coding	EXOC7	ENSG00000182473.21	150	147	1
protein_coding	SEL1L	ENSG00000071537.13	347	340	1
protein_coding	SLC25A36	ENSG00000114120.12	99	97	1
protein_coding	YPEL5	ENSG00000119801.12	247	242	1
protein_coding	PPP2R3C	ENSG00000092020.10	49	48	1
protein_coding	APTX	ENSG00000137074.18	49	48	1
protein_coding	SMG6	ENSG00000070366.13	48	47	1
protein_coding	RILPL1	ENSG00000188026.12	48	47	1
protein_coding	MRPL4	ENSG00000105364.13	48	47	1
protein_coding	NT5C3A	ENSG00000122643.19	48	47	1
protein_coding	SMYD2	ENSG00000143499.13	47	46	1
protein_coding	LAMA2	ENSG00000196569.12	94	92	1
protein_coding	BABAM1	ENSG00000105393.15	141	138	1
protein_coding	HAPLN1	ENSG00000145681.10	421	412	1
protein_coding	SLC37A3	ENSG00000157800.17	93	91	1
protein_coding	PPP6R3	ENSG00000110075.14	92	90	1
protein_coding	LEO1	ENSG00000166477.12	91	89	1
protein_coding	VDAC3	ENSG00000078668.13	182	178	1
protein_coding	SRSF9	ENSG00000111786.8	136	133	1
protein_coding	MRPL30	ENSG00000185414.19	45	44	1
protein_coding	RBM4B	ENSG00000173914.11	45	44	1
protein_coding	HARS	ENSG00000170445.13	45	44	1
protein_coding	KNOP1	ENSG00000103550.13	89	87	1
protein_coding	ARL8B	ENSG00000134108.12	178	174	1
protein_coding	HINT1	ENSG00000169567.11	841	822	1
protein_coding	NCOR2	ENSG00000196498.13	221	216	1
protein_coding	C5orf51	ENSG00000205765.8	44	43	1
protein_coding	IGF2BP3	ENSG00000136231.13	44	43	1
protein_coding	ZNF532	ENSG00000074657.13	88	86	1
protein_coding	CHTOP	ENSG00000160679.12	87	85	1
protein_coding	OAT	ENSG00000065154.11	339	331	1
protein_coding	RBM12B	ENSG00000183808.11	42	41	1
protein_coding	TM2D1	ENSG00000162604.12	252	246	1
protein_coding	TSPAN4	ENSG00000214063.10	418	408	1
protein_coding	TRIM33	ENSG00000197323.11	125	122	1
protein_coding	NDUFA4	ENSG00000189043.9	1160	1132	1
protein_coding	SLC25A44	ENSG00000160785.13	41	40	1
protein_coding	CC2D2A	ENSG00000048342.15	82	80	1

protein_coding	ADI1	ENSG00000182551.13	246	240	1
protein_coding	CSNK1G2	ENSG00000133275.15	122	119	1
protein_coding	BIN1	ENSG00000136717.14	81	79	1
protein_coding	MARCH7	ENSG00000136536.14	162	158	1
protein_coding	NONO	ENSG00000147140.15	445	434	1
sense_intronic	AC037198.1	ENSG00000276107.1	161	157	1
protein_coding	PDCD5	ENSG00000105185.11	201	196	1
protein_coding	RTL8A	ENSG00000203950.6	239	233	1
protein_coding	H2AFV	ENSG00000105968.18	237	231	1
protein_coding	HSPG2	ENSG00000142798.19	236	230	1
protein_coding	LOXL2	ENSG00000134013.15	1016	990	1
protein_coding	VEPH1	ENSG00000197415.11	39	38	1
protein_coding	NME7	ENSG00000143156.13	39	38	1
protein_coding	IPO7	ENSG00000205339.9	268	261	1
protein_coding	TMBIM6	ENSG00000139644.12	1867	1818	1
protein_coding	ZFYVE26	ENSG00000072121.15	38	37	1
protein_coding	ARMH3	ENSG00000120029.12	38	37	1
protein_coding	MRRF	ENSG00000148187.17	38	37	1
protein_coding	OPA1	ENSG00000198836.9	114	111	1
protein_coding	ZDHHC9	ENSG00000188706.12	152	148	1
protein_coding	FIBP	ENSG00000172500.12	113	110	1
protein_coding	VPS37C	ENSG00000167987.10	37	36	1
protein_coding	ANAPC4	ENSG00000053900.10	37	36	1
protein_coding	SSR2	ENSG00000163479.13	332	323	1
protein_coding	BMT2	ENSG00000164603.11	73	71	1
protein_coding	TAX1BP3	ENSG00000213977.7	109	106	1
protein_coding	NPM1	ENSG00000181163.13	1191	1158	1
protein_coding	GTF2B	ENSG00000137947.11	36	35	1
protein_coding	CASP2	ENSG00000106144.19	36	35	1
protein_coding	PYM1	ENSG00000170473.16	36	35	1
protein_coding	JOSD2	ENSG00000161677.11	36	35	1
protein_coding	PYGO2	ENSG00000163348.3	36	35	1
processed_transcript	ZNF528-AS1	ENSG00000269834.5	36	35	1
protein_coding	FDX1	ENSG00000137714.2	107	104	1
protein_coding	NDUFA12	ENSG00000184752.12	142	138	1
protein_coding	ACTN1	ENSG00000072110.13	1487	1445	1
protein_coding	CDC123	ENSG00000151465.13	141	137	1
protein_coding	PHF23	ENSG00000040633.12	70	68	1
protein_coding	RBMX2	ENSG00000134597.15	105	102	1
protein_coding	PCBP2	ENSG00000197111.15	908	882	1
protein_coding	NECTIN3	ENSG00000177707.10	453	440	1
protein_coding	SPATA18	ENSG00000163071.10	139	135	1
protein_coding	DKC1	ENSG00000130826.17	69	67	1
protein_coding	CAND1	ENSG00000111530.12	137	133	1

protein_coding	LDOC1	ENSG00000182195.7	171	166	1
protein_coding	SRGAP2C	ENSG00000171943.11	34	33	1
protein_coding	FNDC10	ENSG00000228594.3	34	33	1
protein_coding	CDKN2AIP	ENSG00000168564.5	34	33	1
protein_coding	TES	ENSG00000135269.17	67	65	1
protein_coding	RPL32	ENSG00000144713.12	3978	3859	1
protein_coding	RPL5	ENSG00000122406.13	2129	2065	1
protein_coding	FAM210A	ENSG00000177150.12	33	32	1
protein_coding	NUP85	ENSG00000125450.10	33	32	1
protein_coding	INTS11	ENSG00000127054.20	33	32	1
protein_coding	ZBTB22	ENSG00000236104.2	33	32	1
protein_coding	NUDCD2	ENSG00000170584.10	33	32	1
protein_coding	QPCT	ENSG00000115828.16	33	32	1
protein_coding	PDE4A	ENSG00000065989.15	66	64	1
protein_coding	CLTA	ENSG00000122705.16	296	287	1
protein_coding	RABL6	ENSG00000196642.18	131	127	1
protein_coding	ARL¬†2	ENSG00000213465.7	97	94	1
protein_coding	METTL5	ENSG00000138382.14	97	94	1
protein_coding	CTSA	ENSG00000064601.18	385	373	1
protein_coding	RAB35	ENSG00000111737.11	96	93	1
protein_coding	SDHB	ENSG00000117118.9	128	124	1
protein_coding	CA12	ENSG00000074410.13	190	184	1
protein_coding	ACOT13	ENSG00000112304.10	190	184	1
protein_coding	SRP9	ENSG00000143742.12	253	245	1
protein_coding	MYL12B	ENSG00000118680.13	1010	978	1
protein_coding	SIGMAR1	ENSG00000147955.16	126	122	1
protein_coding	PLXNA1	ENSG00000114554.11	125	121	1
protein_coding	ZNF316	ENSG00000205903.6	31	30	1
protein_coding	DTX2	ENSG00000091073.19	31	30	1
protein_coding	NRAS	ENSG00000213281.4	62	60	1
protein_coding	CAPNS1	ENSG00000126247.10	831	804	1
protein_coding	APPL1	ENSG00000157500.11	153	148	1
protein_coding	PDZD11	ENSG00000120509.10	91	88	1
protein_coding	ERCC6	ENSG00000225830.12	30	29	1
protein_coding	WDTC1	ENSG00000142784.15	30	29	1
protein_coding	SP2	ENSG00000167182.14	30	29	1
protein_coding	BTBD9	ENSG00000183826.17	30	29	1
protein_coding	PLEKHM1	ENSG00000225190.10	30	29	1
protein_coding	SLC35E3	ENSG00000175782.9	60	58	1
protein_coding	ORAI3	ENSG00000175938.6	60	58	1
protein_coding	PCED1A	ENSG00000132635.16	60	58	1
protein_coding	CHMP7	ENSG00000147457.13	90	87	1
protein_coding	RPL24	ENSG00000114391.12	1645	1590	1
protein_coding	RPL36	ENSG00000130255.12	1220	1179	1

protein_coding	DGUOK	ENSG00000114956.19	89	86	1
protein_coding	FAM126A	ENSG00000122591.11	148	143	1
protein_coding	ZFX	ENSG00000005889.15	59	57	1
protein_coding	APEX1	ENSG00000100823.11	236	228	1
protein_coding	PROCR	ENSG00000101000.5	117	113	1
protein_coding	APMAP	ENSG00000101474.11	292	282	1
protein_coding	DYNLT1	ENSG00000146425.10	175	169	1
protein_coding	ING5	ENSG00000168395.15	29	28	1
protein_coding	SEC22A	ENSG00000121542.11	29	28	1
protein_coding	NFASC	ENSG00000163531.15	87	84	1
protein_coding	PSMC2	ENSG00000161057.11	203	196	1
protein_coding	GLUD1	ENSG00000148672.8	346	334	1
protein_coding	B2M	ENSG00000166710.18	4812	4645	1
protein_coding	BTG1	ENSG00000133639.4	1208	1166	1
protein_coding	RNF216	ENSG00000011275.18	115	111	1
protein_coding	LPIN2	ENSG00000101577.9	86	83	1
protein_coding	SH3BP5	ENSG00000131370.15	313	302	1
protein_coding	PHF20	ENSG00000025293.16	170	164	1
protein_coding	SENP1	ENSG00000079387.13	28	27	1
protein_coding	FGF5	ENSG00000138675.16	56	54	1
protein_coding	FMNL2	ENSG00000157827.19	84	81	1
protein_coding	MFN2	ENSG00000116688.16	112	108	1
protein_coding	TENT4B	ENSG00000121274.12	140	135	1
protein_coding	CCDC6	ENSG00000108091.10	166	160	1
protein_coding	CELF1	ENSG00000149187.18	304	293	1
protein_coding	FAM13A	ENSG00000138640.14	55	53	1
protein_coding	PDLIM7	ENSG00000196923.13	55	53	1
protein_coding	POLR2B	ENSG00000047315.15	219	211	1
protein_coding	MTIF2	ENSG00000085760.14	82	79	1
protein_coding	ANTXR1	ENSG00000169604.19	273	263	1
protein_coding	WTAP	ENSG00000146457.15	109	105	1
protein_coding	PANK3	ENSG00000120137.6	109	105	1
protein_coding	CBX5	ENSG00000094916.15	136	131	1
protein_coding	SFT2D2	ENSG00000213064.9	136	131	1
protein_coding	ITCH	ENSG00000078747.14	27	26	1
protein_coding	ALDH16A1	ENSG00000161618.9	27	26	1
protein_coding	TMEM99	ENSG00000167920.8	27	26	1
protein_coding	STN¬†1	ENSG00000107960.10	54	52	1
protein_coding	KHNYN	ENSG00000100441.9	54	52	1
protein_coding	CAP2	ENSG00000112186.11	108	104	1
protein_coding	UBE2E3	ENSG00000170035.15	241	232	1
protein_coding	DHX29	ENSG00000067248.9	214	206	1
protein_coding	ATP6V1A	ENSG00000114573.9	321	309	1
protein_coding	VASP	ENSG00000125753.13	80	77	1

protein_coding	CLPX	ENSG00000166855.9	80	77	1
protein_coding	SPART	ENSG00000133104.13	213	205	1
protein_coding	ZNF652	ENSG00000198740.8	53	51	1
protein_coding	SUMO2	ENSG00000188612.11	105	101	1
protein_coding	HCFC1R1	ENSG00000103145.10	157	151	1
protein_coding	TAGLN	ENSG00000149591.16	1016	977	1
protein_coding	CIT	ENSG00000122966.15	26	25	1
lincRNA	AC110597.1	ENSG00000260578.1	52	50	1
protein_coding	TMEM98	ENSG00000006042.11	311	299	1
protein_coding	LIPA	ENSG00000107798.17	103	99	1
protein_coding	ATP5MF	ENSG00000241468.7	206	198	1
protein_coding	PPT1	ENSG00000131238.17	77	74	1
protein_coding	REV1	ENSG00000135945.9	51	49	1
protein_coding	TRIM5	ENSG00000132256.18	76	73	1
protein_coding	AKR7A2	ENSG00000053371.12	177	170	1
protein_coding	PSMB6	ENSG00000142507.9	302	290	1
protein_coding	ZDBF2	ENSG00000204186.8	25	24	1
protein_coding	ETV3	ENSG00000117036.11	25	24	1
protein_coding	SERTAD1	ENSG00000197019.4	25	24	1
protein_coding	DBR1	ENSG00000138231.12	25	24	1
protein_coding	MEX3B	ENSG00000183496.5	25	24	1
protein_coding	ZNF844	ENSG00000223547.9	25	24	1
protein_coding	AEBP2	ENSG00000139154.14	50	48	1
protein_coding	KAT7	ENSG00000136504.11	50	48	1
protein_coding	SLC12A2	ENSG00000064651.13	125	120	1
protein_coding	NAA20	ENSG00000173418.11	174	167	1
protein_coding	PNMA1	ENSG00000176903.4	174	167	1
protein_coding	ATP6V0E1	ENSG00000113732.8	1285	1233	1
protein_coding	TSC22D1	ENSG00000102804.14	270	259	1
protein_coding	PRKCE	ENSG00000171132.13	49	47	1
protein_coding	PQBP1	ENSG00000102103.15	49	47	1
protein_coding	LMO7	ENSG00000136153.19	196	188	1
protein_coding	PSMC5	ENSG00000087191.12	196	188	1
protein_coding	EXOC6B	ENSG00000144036.15	73	70	1
protein_coding	PTPRA	ENSG00000132670.20	73	70	1
protein_coding	RMDN1	ENSG00000176623.11	73	70	1
protein_coding	IFT20	ENSG00000109083.13	97	93	1
protein_coding	FBXW2	ENSG00000119402.16	169	162	1
protein_coding	SOWAHC	ENSG00000198142.4	24	23	1
protein_coding	BACE2	ENSG00000182240.15	24	23	1
protein_coding	FOXD2	ENSG00000186564.5	24	23	1
antisense	AC027307.2	ENSG00000267317.2	24	23	1
protein_coding	ANKRD36C	ENSG00000174501.14	24	23	1
protein_coding	PNMA8A	ENSG00000182013.17	24	23	1

protein_coding	MED26	ENSG00000105085.10	24	23	1
protein_coding	GMPS	ENSG00000163655.15	48	46	1
protein_coding	SHOX2	ENSG00000168779.19	144	138	1
protein_coding	SERPINE2	ENSG00000135919.12	2670	2558	1
protein_coding	GDI2	ENSG00000057608.16	405	388	1
protein_coding	SH3GLB1	ENSG00000097033.14	333	319	1
protein_coding	SEPHS1	ENSG00000086475.14	95	91	1
protein_coding	TSPAN31	ENSG00000135452.9	71	68	1
protein_coding	TBC1D23	ENSG00000036054.12	142	136	1
protein_coding	RLF	ENSG00000117000.8	47	45	1
protein_coding	SURF6	ENSG00000148296.6	47	45	1
protein_coding	TRIM13	ENSG00000204977.9	116	111	1
protein_coding	AR	ENSG00000169083.16	116	111	1
protein_coding	TMBIM1	ENSG00000135926.14	602	576	1
protein_coding	PEAK1	ENSG00000173517.10	162	155	1
protein_coding	GNAI3	ENSG00000065135.10	324	310	1
protein_coding	SELENOW	ENSG00000178980.14	254	243	1
protein_coding	ARL6IP6	ENSG00000177917.10	23	22	1
protein_coding	RABL3	ENSG00000144840.9	23	22	1
protein_coding	ZNF445	ENSG00000185219.16	23	22	1
protein_coding	RNF185	ENSG00000138942.15	23	22	1
protein_coding	OXSR1	ENSG00000172939.8	115	110	1
protein_coding	TOP2B	ENSG00000077097.15	207	198	1
protein_coding	LAMP2	ENSG00000005893.15	483	462	1
protein_coding	LDLRAP1	ENSG00000157978.11	68	65	1
protein_coding	NOL10	ENSG00000115761.15	68	65	1
protein_coding	JMJD8	ENSG00000161999.11	68	65	1
protein_coding	TRAF7	ENSG00000131653.12	136	130	1
protein_coding	PPA2	ENSG00000138777.19	113	108	1
protein_coding	MCL1	ENSG00000143384.12	429	410	1
protein_coding	NHLRC2	ENSG00000196865.4	45	43	1
protein_coding	BPNT1	ENSG00000162813.17	45	43	1
protein_coding	ARHGAP18	ENSG00000146376.10	90	86	1
protein_coding	LARP1	ENSG00000155506.16	450	430	1
protein_coding	HIST1H1C	ENSG00000187837.3	292	279	1
protein_coding	ATP5PF	ENSG00000154723.12	449	429	1
protein_coding	TPT1	ENSG00000133112.16	10931	10444	1
protein_coding	AAMP	ENSG00000127837.9	202	193	1
protein_coding	COPS8	ENSG00000198612.10	157	150	1
protein_coding	KLHL9	ENSG00000198642.6	112	107	1
protein_coding	NUDC	ENSG00000090273.13	67	64	1
protein_coding	HIBCH	ENSG00000198130.15	89	85	1
protein_coding	DIP2C	ENSG00000151240.16	133	127	1
protein_coding	LEPR	ENSG00000116678.19	465	444	1

protein_coding	ROBO1	ENSG00000169855.19	287	274	1
protein_coding	MRM2	ENSG00000122687.17	22	21	1
protein_coding	EHBP1L1	ENSG00000173442.12	22	21	1
protein_coding	SLC9A8	ENSG00000197818.11	22	21	1
protein_coding	ACTR8	ENSG00000113812.13	44	42	1
protein_coding	ZNF362	ENSG00000160094.14	44	42	1
protein_coding	ATP6V0D1	ENSG00000159720.11	88	84	1
protein_coding	ENY2	ENSG00000120533.12	330	315	1
protein_coding	FAM114A1	ENSG00000197712.11	746	712	1
protein_coding	DICER1	ENSG00000100697.14	87	83	1
protein_coding	STK11	ENSG00000118046.14	87	83	1
protein_coding	PPP2R2D	ENSG00000175470.19	65	62	1
protein_coding	TSPO	ENSG00000100300.17	975	930	1
protein_coding	IGFBP6	ENSG00000167779.8	454	433	1
protein_coding	C12orf29	ENSG00000133641.17	43	41	1
protein_coding	FBXO31	ENSG00000103264.17	43	41	1
protein_coding	APPBP2	ENSG00000062725.9	129	123	1
protein_coding	UCHL1	ENSG00000154277.12	493	470	1
protein_coding	SEMA3A	ENSG00000075213.10	278	265	1
protein_coding	CAMTA2	ENSG00000108509.20	64	61	1
protein_coding	CTTN	ENSG00000085733.15	489	466	1
protein_coding	G3BP2	ENSG00000138757.14	170	162	1
protein_coding	UBTF	ENSG00000108312.14	106	101	1
protein_coding	RPL11	ENSG00000142676.14	2377	2264	1
transcribed_unprocessed_pseudogene	NPIPP1	ENSG00000188599.17	21	20	1
protein_coding	SALL2	ENSG00000165821.11	21	20	1
protein_coding	MAP3K1	ENSG00000095015.5	21	20	1
protein_coding	GMEB2	ENSG00000101216.10	42	40	1
protein_coding	DCAF16	ENSG00000163257.10	42	40	1
protein_coding	BRD7	ENSG00000166164.15	63	60	1
protein_coding	RAB11FIP5	ENSG00000135631.16	231	220	1
protein_coding	COX20	ENSG00000203667.9	209	199	1
protein_coding	GULP1	ENSG00000144366.15	188	179	1
protein_coding	GALNT5	ENSG00000136542.8	416	396	1
protein_coding	HNRNPUL1	ENSG00000105323.16	291	277	1
protein_coding	CANT1	ENSG00000171302.16	83	79	1
protein_coding	COPS6	ENSG00000168090.9	145	138	1
protein_coding	CTDSP2	ENSG00000175215.10	414	394	1
protein_coding	LMBRD2	ENSG00000164187.6	62	59	1
protein_coding	ARSD	ENSG00000006756.15	144	137	1
protein_coding	TPD52L2	ENSG00000101150.17	288	274	1
protein_coding	EIF1B	ENSG00000114784.3	185	176	1

protein_coding	MGRN1	ENSG00000102858.12	41	39	1
protein_coding	APPL2	ENSG00000136044.11	41	39	1
protein_coding	TRAPPC2L	ENSG00000167515.10	102	97	1
protein_coding	RPL10A	ENSG00000198755.10	1406	1337	1
protein_coding	MRPS14	ENSG00000120333.4	61	58	1
protein_coding	SMIM14	ENSG00000163683.11	446	424	1
protein_coding	FAHD1	ENSG00000180185.11	81	77	1
protein_coding	RPL23A	ENSG00000198242.13	3134	2978	1
protein_coding	ABHD15	ENSG00000168792.4	20	19	1
protein_coding	IFT122	ENSG00000163913.11	20	19	1
protein_coding	SMPD4	ENSG00000136699.19	40	38	1
protein_coding	AUH	ENSG00000148090.11	40	38	1
protein_coding	RRP36	ENSG00000124541.6	60	57	1
protein_coding	MTX3	ENSG00000177034.15	60	57	1
protein_coding	NDN	ENSG00000182636.6	60	57	1
protein_coding	RIT1	ENSG00000143622.10	80	76	1
protein_coding	DCTN4	ENSG00000132912.12	120	114	1
protein_coding	DYNLL1	ENSG00000088986.10	620	589	1
protein_coding	DGCR2	ENSG00000070413.19	99	94	0.9
protein_coding	EMC2	ENSG00000104412.7	99	94	0.9
protein_coding	ZNF12	ENSG00000164631.18	59	56	0.9
protein_coding	NUDT3	ENSG00000272325.1	236	224	0.9
protein_coding	DYNLRB1	ENSG00000125971.16	216	205	0.9
protein_coding	MRPS18B	ENSG00000204568.11	157	149	0.9
protein_coding	ADAM17	ENSG00000151694.13	176	167	0.9
protein_coding	BCLAF3	ENSG00000173681.16	39	37	0.9
protein_coding	GUSB	ENSG00000169919.16	39	37	0.9
protein_coding	GGCT	ENSG00000006625.17	39	37	0.9
protein_coding	MRPS18C	ENSG00000163319.10	97	92	0.9
protein_coding	NDUFB5	ENSG00000136521.12	194	184	0.9
protein_coding	MCUR1	ENSG00000050393.11	58	55	0.9
protein_coding	LONP1	ENSG00000196365.11	58	55	0.9
protein_coding	FBXO30	ENSG00000118496.4	58	55	0.9
protein_coding	RSRC1	ENSG00000174891.12	328	311	0.9
protein_coding	TBX2	ENSG00000121068.13	153	145	0.9
protein_coding	ATF4	ENSG00000128272.14	745	706	0.9
protein_coding	ARHGEF3	ENSG00000163947.11	19	18	0.9
protein_coding	PRMT6	ENSG00000198890.8	19	18	0.9
protein_coding	PPOX	ENSG00000143224.17	19	18	0.9
protein_coding	MTERF3	ENSG00000156469.8	19	18	0.9
protein_coding	ERCC2	ENSG00000104884.15	19	18	0.9
protein_coding	ZSCAN29	ENSG00000140265.12	57	54	0.9
protein_coding	AFTPH	ENSG00000119844.15	95	90	0.9
protein_coding	ZC3H7A	ENSG00000122299.11	114	108	0.9

protein_coding	RAB3GAP2	ENSG00000118873.15	133	126	0.9
protein_coding	P4HB	ENSG00000185624.14	1383	1310	0.9
protein_coding	PPP6C	ENSG00000119414.11	187	177	0.9
protein_coding	PTEN	ENSG00000171862.10	374	354	0.9
protein_coding	TNFAIP2	ENSG00000185215.8	56	53	0.9
protein_coding	IAH1	ENSG00000134330.18	149	141	0.9
protein_coding	BRD8	ENSG00000112983.17	93	88	0.9
protein_coding	TMED4	ENSG00000158604.14	186	176	0.9
protein_coding	LHX8	ENSG00000162624.14	297	281	0.9
protein_coding	CNOT7	ENSG00000198791.11	167	158	0.9
protein_coding	NAT14	ENSG00000090971.4	37	35	0.9
protein_coding	POLG	ENSG00000140521.13	37	35	0.9
protein_coding	PSPC1	ENSG00000121390.18	37	35	0.9
protein_coding	FAM168A	ENSG00000054965.10	37	35	0.9
protein_coding	FZR1	ENSG00000105325.13	37	35	0.9
protein_coding	SPPL2B	ENSG00000005206.16	37	35	0.9
protein_coding	HPS4	ENSG00000100099.20	37	35	0.9
protein_coding	STS	ENSG00000101846.6	92	87	0.9
protein_coding	LYRM7	ENSG00000186687.15	110	104	0.9
protein_coding	C6orf89	ENSG00000198663.16	403	381	0.9
protein_coding	PLRG1	ENSG00000171566.11	73	69	0.9
protein_coding	TNRC6C	ENSG00000078687.17	73	69	0.9
protein_coding	PTBP1	ENSG00000011304.19	182	172	0.9
protein_coding	NR1D2	ENSG00000174738.12	200	189	0.9
protein_coding	CLSTN1	ENSG00000171603.17	345	326	0.9
protein_coding	MVD	ENSG00000167508.11	18	17	0.9
protein_coding	ZMAT5	ENSG00000100319.12	18	17	0.9
protein_coding	NHLRC3	ENSG00000188811.13	18	17	0.9
protein_coding	GIN1	ENSG00000145723.16	18	17	0.9
lincRNA	AC090114.2	ENSG00000273270.1	18	17	0.9
protein_coding	PHC1	ENSG00000111752.10	18	17	0.9
protein_coding	TNK2	ENSG00000061938.17	36	34	0.9
protein_coding	POMT1	ENSG00000130714.15	36	34	0.9
lincRNA	LINC00662	ENSG00000261824.6	36	34	0.9
protein_coding	C18orf21	ENSG00000141428.16	54	51	0.9
protein_coding	ERCC4	ENSG00000175595.14	72	68	0.9
protein_coding	USP3	ENSG00000140455.16	90	85	0.9
protein_coding	RNPS1	ENSG00000205937.11	90	85	0.9
protein_coding	MKLN1	ENSG00000128585.17	180	170	0.9
protein_coding	TOMM70	ENSG00000154174.7	216	204	0.9
protein_coding	ADAR	ENSG00000160710.16	341	322	0.9
protein_coding	RND3	ENSG00000115963.13	1755	1657	0.9
protein_coding	NCK2	ENSG00000071051.13	89	84	0.9
protein_coding	ADAM10	ENSG00000137845.14	445	420	0.9

protein_coding	ARHGAP12	ENSG00000165322.17	160	151	0.9
protein_coding	BPGM	ENSG00000172331.11	53	50	0.9
protein_coding	SCAND1	ENSG00000171222.10	53	50	0.9
protein_coding	PARD3	ENSG00000148498.15	88	83	0.9
protein_coding	NUMA1	ENSG00000137497.17	211	199	0.9
protein_coding	RIN1	ENSG00000174791.10	35	33	0.9
protein_coding	PACS1	ENSG00000175115.11	35	33	0.9
protein_coding	CRTC1	ENSG00000105662.15	35	33	0.9
protein_coding	GPRASP2	ENSG00000158301.18	35	33	0.9
protein_coding	UNC13B	ENSG00000198722.14	70	66	0.9
protein_coding	PCBP4	ENSG00000090097.21	122	115	0.9
protein_coding	IVNS1ABP	ENSG00000116679.15	87	82	0.9
protein_coding	TMEM127	ENSG00000135956.8	174	164	0.9
protein_coding	PTDSS2	ENSG00000174915.11	52	49	0.9
protein_coding	PPP1R2	ENSG00000184203.7	104	98	0.9
protein_coding	YWHAB	ENSG00000166913.12	1905	1795	0.9
protein_coding	ZMAT3	ENSG00000172667.10	812	765	0.9
protein_coding	HGSNAT	ENSG00000165102.14	189	178	0.9
protein_coding	LDHB	ENSG00000111716.12	308	290	0.9
protein_coding	HDAC2	ENSG00000196591.11	222	209	0.9
protein_coding	PSMB3	ENSG00000277791.4	222	209	0.9
protein_coding	CTNND1	ENSG00000198561.13	256	241	0.9
protein_coding	GOLGA8A	ENSG00000175265.17	17	16	0.9
protein_coding	DGKQ	ENSG00000145214.13	17	16	0.9
protein_coding	RRS1	ENSG00000179041.3	17	16	0.9
protein_coding	DDX39A	ENSG00000123136.14	17	16	0.9
antisense	SLC16A1-	ENSG00000226419.7	17	16	0.9
protein_coding	TYW5	ENSG00000162971.10	17	16	0.9
protein_coding	TP53I13	ENSG00000167543.15	68	64	0.9
protein_coding	RPS13	ENSG00000110700.6	1557	1465	0.9
protein_coding	GLS	ENSG00000115419.12	203	191	0.9
protein_coding	EIF2B2	ENSG00000119718.10	168	158	0.9
protein_coding	ARPC4	ENSG00000241553.12	235	221	0.9
protein_coding	EFEMP2	ENSG00000172638.12	453	426	0.9
protein_coding	UQCR11	ENSG00000127540.11	520	489	0.9
protein_coding	SLBP	ENSG00000163950.12	67	63	0.9
protein_coding	RANBP1	ENSG00000099901.16	67	63	0.9
protein_coding	GSPT1	ENSG00000103342.12	334	314	0.9
protein_coding	LAPTM4B	ENSG00000104341.16	133	125	0.9
protein_coding	HPF1	ENSG00000056050.6	83	78	0.9
protein_coding	NR2F2	ENSG00000185551.14	232	218	0.9
processed_transcript	LRRC75A-	ENSG00000175061.17	1408	1323	0.9
protein_coding	UBQLN2	ENSG00000188021.8	149	140	0.9
protein_coding	BCAS4	ENSG00000124243.17	33	31	0.9

protein_coding	NCK1	ENSG00000158092.6	66	62	0.9
protein_coding	MLF¬†2	ENSG00000089693.10	214	201	0.9
protein_coding	PMPCB	ENSG00000105819.13	148	139	0.9
protein_coding	PABPC4	ENSG00000090621.13	755	709	0.9
protein_coding	EEF2	ENSG00000167658.15	4603	4322	0.9
protein_coding	NDUFB8	ENSG00000166136.15	180	169	0.9
protein_coding	TENT5A	ENSG00000112773.15	507	476	0.9
protein_coding	EML3	ENSG00000149499.11	49	46	0.9
protein_coding	RBBP5	ENSG00000117222.13	49	46	0.9
protein_coding	RAB40B	ENSG00000141542.10	49	46	0.9
protein_coding	GDE1	ENSG00000006007.11	538	505	0.9
protein_coding	PRXL2B	ENSG00000157870.15	65	61	0.9
protein_coding	CLU	ENSG00000120885.21	162	152	0.9
protein_coding	MSX1	ENSG00000163132.6	1117	1048	0.9
protein_coding	ILF3	ENSG00000129351.17	194	182	0.9
protein_coding	TCF12	ENSG00000140262.17	436	409	0.9
protein_coding	RPL22	ENSG00000116251.10	371	348	0.9
protein_coding	PDCD6IP	ENSG00000170248.14	306	287	0.9
Mt_tRNA	MT-TY	ENSG00000210144.1	16	15	0.9
protein_coding	ZNF566	ENSG00000186017.14	16	15	0.9
protein_coding	RIN3	ENSG00000100599.15	16	15	0.9
protein_coding	GGA1	ENSG00000100083.18	32	30	0.9
protein_coding	HAGH	ENSG00000063854.12	48	45	0.9
protein_coding	ABRAXAS2	ENSG00000165660.7	48	45	0.9
protein_coding	POGZ	ENSG00000143442.21	64	60	0.9
protein_coding	IRAK4	ENSG00000198001.13	64	60	0.9
protein_coding	MRPL57	ENSG00000173141.4	112	105	0.9
protein_coding	RAB1B	ENSG00000174903.15	192	180	0.9
protein_coding	CD151	ENSG00000177697.18	811	760	0.9
protein_coding	ZFHX4	ENSG00000091656.16	143	134	0.9
protein_coding	SNRPD1	ENSG00000167088.10	127	119	0.9
protein_coding	RAB5A	ENSG00000144566.10	222	208	0.9
protein_coding	CLIC4	ENSG00000169504.14	949	889	0.9
protein_coding	MAN2A2	ENSG00000196547.14	79	74	0.9
protein_coding	DLX1	ENSG00000144355.14	63	59	0.9
protein_coding	KCMF1	ENSG00000176407.17	157	147	0.9
protein_coding	CLEC11A	ENSG00000105472.12	204	191	0.9
protein_coding	RPS20	ENSG00000008988.9	2526	2365	0.9
protein_coding	ZNF800	ENSG00000048405.9	47	44	0.9
protein_coding	TECPR1	ENSG00000205356.9	47	44	0.9
protein_coding	MSRB2	ENSG00000148450.12	172	161	0.9
protein_coding	THRA	ENSG00000126351.12	125	117	0.9
protein_coding	CTDSPL	ENSG00000144677.14	171	160	0.9
protein_coding	CLK4	ENSG00000113240.12	31	29	0.9

protein_coding	GMEB1	ENSG00000162419.12	31	29	0.9
protein_coding	AKAP1	ENSG00000121057.12	31	29	0.9
protein_coding	LSM¬†10	ENSG00000181817.5	62	58	0.9
protein_coding	NIPSNAP1	ENSG00000184117.11	62	58	0.9
protein_coding	PLP2	ENSG00000102007.10	201	188	0.9
protein_coding	EDEM1	ENSG00000134109.10	46	43	0.9
protein_coding	CCDC71L	ENSG00000253276.2	138	129	0.9
protein_coding	GLRX5	ENSG00000182512.4	107	100	0.9
protein_coding	DNAJC25	ENSG00000059769.19	61	57	0.9
protein_coding	NXPE3	ENSG00000144815.15	76	71	0.9
protein_coding	SMIM20	ENSG00000250317.8	76	71	0.9
protein_coding	MEX3D	ENSG00000181588.16	152	142	0.9
protein_coding	TRIM44	ENSG00000166326.6	197	184	0.9
protein_coding	NFIX	ENSG00000008441.16	257	240	0.9
protein_coding	CDK5RAP1	ENSG00000101391.20	15	14	0.9
processed_pseudogene	AC144530.1	ENSG00000234742.1	15	14	0.9
protein_coding	EXOG	ENSG00000157036.12	15	14	0.9
protein_coding	WDCP	ENSG00000163026.11	15	14	0.9
protein_coding	CYP4V2	ENSG00000145476.15	15	14	0.9
protein_coding	SFXN3	ENSG00000107819.13	30	28	0.9
protein_coding	KMT2D	ENSG00000167548.15	45	42	0.9
protein_coding	CABIN1	ENSG00000099991.17	45	42	0.9
protein_coding	TBK1	ENSG00000183735.9	60	56	0.9
protein_coding	DCUN1D1	ENSG00000043093.13	60	56	0.9
protein_coding	TCTN1	ENSG00000204852.15	60	56	0.9
protein_coding	COPS4	ENSG00000138663.8	75	70	0.9
protein_coding	RCOR3	ENSG00000117625.13	90	84	0.9
protein_coding	MOB1A	ENSG00000114978.17	193	180	0.9
protein_coding	VPS35	ENSG00000069329.17	356	332	0.9
protein_coding	NCSTN	ENSG00000162736.16	251	234	0.9
protein_coding	PLEC	ENSG00000178209.15	310	289	0.9
protein_coding	RPL37	ENSG00000145592.13	4215	3929	0.9
protein_coding	NLRP1	ENSG00000091592.15	88	82	0.9
protein_coding	SNRPN	ENSG00000128739.22	322	300	0.9
protein_coding	ST13	ENSG00000100380.13	436	406	0.9
protein_coding	GALK1	ENSG00000108479.11	29	27	0.9
protein_coding	WDSUB1	ENSG00000196151.10	29	27	0.9
protein_coding	UBAP2	ENSG00000137073.21	29	27	0.9
protein_coding	TMEM131L	ENSG00000121210.15	29	27	0.9
protein_coding	NCOA3	ENSG00000124151.18	58	54	0.9
protein_coding	EXOSC9	ENSG00000123737.12	58	54	0.9
protein_coding	SLC35E2A	ENSG00000215790.7	58	54	0.9
protein_coding	CACYBP	ENSG00000116161.17	116	108	0.9
protein_coding	CNIH1	ENSG00000100528.11	348	324	0.9

protein_coding	CSPG4	ENSG00000173546.7	101	94	0.9
protein_coding	CD248	ENSG00000174807.3	505	470	0.9
protein_coding	HPCAL1	ENSG00000115756.12	273	254	0.9
protein_coding	COL5A2	ENSG00000204262.12	1149	1069	0.9
protein_coding	STX17	ENSG00000136874.10	43	40	0.9
protein_coding	IMPACT	ENSG00000154059.10	129	120	0.9
protein_coding	ACSL3	ENSG00000123983.13	428	398	0.9
protein_coding	ST6GALNAC	ENSG00000136840.18	57	53	0.9
protein_coding	NPHP3	ENSG00000113971.19	57	53	0.9
protein_coding	ZDHHC16	ENSG00000171307.18	57	53	0.9
protein_coding	PODN	ENSG00000174348.13	57	53	0.9
processed_transcript	SNHG6	ENSG00000245910.8	413	384	0.9
protein_coding	FASTK	ENSG00000164896.19	128	119	0.9
protein_coding	MGST1	ENSG00000008394.12	866	805	0.9
protein_coding	NPEPPS	ENSG00000141279.15	468	435	0.9
protein_coding	SMNDC1	ENSG00000119953.12	99	92	0.9
protein_coding	NEK1	ENSG00000137601.16	127	118	0.9
protein_coding	ATRAID	ENSG00000138085.16	197	183	0.9
protein_coding	NDUFS4	ENSG00000164258.11	267	248	0.9
protein_coding	SLC35E4	ENSG00000100036.12	14	13	0.9
protein_coding	NAIF1	ENSG00000171169.8	14	13	0.9
protein_coding	THAP2	ENSG00000173451.6	14	13	0.9
protein_coding	MFAP3	ENSG00000037749.12	14	13	0.9
protein_coding	MRPL12	ENSG00000262814.7	14	13	0.9
protein_coding	B9D1	ENSG00000108641.15	14	13	0.9
protein_coding	ALKBH4	ENSG00000160993.3	14	13	0.9
protein_coding	TCTN2	ENSG00000168778.11	14	13	0.9
protein_coding	EFCAB7	ENSG00000203965.12	14	13	0.9
lincRNA	LINC01305	ENSG00000231453.1	14	13	0.9
protein_coding	BYSL	ENSG00000112578.9	14	13	0.9
protein_coding	ANKS1A	ENSG00000064999.14	28	26	0.9
protein_coding	EDRF1	ENSG00000107938.17	28	26	0.9
protein_coding	SLC25A17	ENSG00000100372.14	28	26	0.9
protein_coding	PLD2	ENSG00000129219.13	28	26	0.9
lincRNA	AC005332.6	ENSG00000278730.1	42	39	0.9
protein_coding	AMPD2	ENSG00000116337.15	98	91	0.9
protein_coding	MR1	ENSG00000153029.14	98	91	0.9
protein_coding	DTX3L	ENSG00000163840.9	112	104	0.9
protein_coding	ANAPC13	ENSG00000129055.12	140	130	0.9
protein_coding	SMARCC2	ENSG00000139613.11	196	182	0.9
protein_coding	ISCU	ENSG00000136003.15	793	736	0.9
protein_coding	GLT8D1	ENSG00000016864.18	153	142	0.9
protein_coding	SF1	ENSG00000168066.20	125	116	0.9
protein_coding	MVP	ENSG00000013364.18	346	321	0.9

protein_coding	RCC1	ENSG00000180198.16	83	77	0.9
protein_coding	SHISA5	ENSG00000164054.15	249	231	0.9
protein_coding	PSG5	ENSG00000204941.13	415	385	0.9
protein_coding	DHX40	ENSG00000108406.9	152	141	0.9
protein_coding	VCL	ENSG00000035403.17	525	487	0.9
protein_coding	FBXW4	ENSG00000107829.13	69	64	0.9
protein_coding	MTOR	ENSG00000198793.12	69	64	0.9
protein_coding	SENP5	ENSG00000119231.10	55	51	0.9
protein_coding	TMEM260	ENSG00000070269.13	55	51	0.9
protein_coding	DEF8	ENSG00000140995.16	55	51	0.9
protein_coding	BANF1	ENSG00000175334.7	110	102	0.9
protein_coding	UCHL5	ENSG00000116750.13	41	38	0.9
protein_coding	MOGS	ENSG00000115275.12	41	38	0.9
protein_coding	JPT2	ENSG00000206053.12	41	38	0.9
protein_coding	CETN2	ENSG00000147400.8	123	114	0.9
protein_coding	SNX18	ENSG00000178996.13	287	266	0.9
protein_coding	THBS3	ENSG00000169231.13	27	25	0.9
protein_coding	TMEM175	ENSG00000127419.16	27	25	0.9
protein_coding	PMF1	ENSG00000160783.19	27	25	0.9
protein_coding	HMG20A	ENSG00000140382.14	54	50	0.9
protein_coding	TNFRSF14	ENSG00000157873.17	54	50	0.9
protein_coding	AHCY	ENSG00000101444.12	108	100	0.9
protein_coding	SLC35B2	ENSG00000157593.18	108	100	0.9
protein_coding	TMEM47	ENSG00000147027.3	1024	948	0.9
protein_coding	CCNL2	ENSG00000221978.11	202	187	0.9
protein_coding	SH3BGRL	ENSG00000131171.12	389	360	0.9
protein_coding	SNED1	ENSG00000162804.13	67	62	0.9
protein_coding	POP4	ENSG00000105171.9	67	62	0.9
protein_coding	ADIRF	ENSG00000148671.13	268	248	0.9
protein_coding	TIMP2	ENSG00000035862.12	3760	3479	0.9
protein_coding	LAMB2	ENSG00000172037.13	294	272	0.9
protein_coding	EIF5	ENSG00000100664.10	481	445	0.9
protein_coding	TOR1AIP1	ENSG00000143337.18	187	173	0.9
protein_coding	UQCRQ	ENSG00000164405.10	721	667	0.9
protein_coding	GTPBP2	ENSG00000172432.18	80	74	0.9
protein_coding	ILK	ENSG00000166333.13	120	111	0.9
protein_coding	MRPL43	ENSG00000055950.16	186	172	0.9
protein_coding	SEC23IP	ENSG00000107651.12	53	49	0.9
protein_coding	PIGP	ENSG00000185808.13	53	49	0.9
protein_coding	SNX14	ENSG00000135317.12	159	147	0.9
protein_coding	CCZ1	ENSG00000122674.11	172	159	0.9
protein_coding	SDCBP	ENSG00000137575.11	867	801	0.9
protein_coding	TBC1D9B	ENSG00000197226.12	105	97	0.9
protein_coding	MEAF6	ENSG00000163875.15	157	145	0.9

protein_coding	RPS15A	ENSG00000134419.15	562	519	0.9
protein_coding	PSMD2	ENSG00000175166.16	261	241	0.9
protein_coding	SRPX	ENSG00000101955.14	261	241	0.9
protein_coding	STAT1	ENSG00000115415.18	535	494	0.9
antisense	AGAP2-AS1	ENSG00000255737.2	13	12	0.9
protein_coding	GNL3L	ENSG00000130119.15	13	12	0.9
protein_coding	KATNB1	ENSG00000140854.12	13	12	0.9
protein_coding	DNAJA4	ENSG00000140403.12	13	12	0.9
protein_coding	SSSCA1	ENSG00000173465.7	13	12	0.9
protein_coding	DOCK9	ENSG00000088387.18	13	12	0.9
protein_coding	RBMXL1	ENSG00000213516.9	26	24	0.9
protein_coding	GAMT	ENSG00000130005.12	26	24	0.9
protein_coding	ANKRD42	ENSG00000137494.13	39	36	0.9
protein_coding	LMOD1	ENSG00000163431.12	39	36	0.9
protein_coding	ADAM33	ENSG00000149451.17	65	60	0.9
protein_coding	SORBS3	ENSG00000120896.13	91	84	0.9
protein_coding	SLC16A2	ENSG00000147100.10	91	84	0.9
protein_coding	PFKM	ENSG00000152556.16	91	84	0.9
protein_coding	RARS2	ENSG00000146282.17	117	108	0.9
protein_coding	MTA1	ENSG00000182979.17	182	168	0.9
protein_coding	SPOP	ENSG00000121067.17	221	204	0.9
protein_coding	SKP1	ENSG00000113558.18	906	836	0.9
protein_coding	RABEP1	ENSG00000029725.16	142	131	0.9
protein_coding	MAP3K2	ENSG00000169967.16	219	202	0.9
protein_coding	TPI1	ENSG00000111669.14	476	439	0.9
protein_coding	PTGES3	ENSG00000110958.15	398	367	0.9
protein_coding	MTMR4	ENSG00000108389.9	77	71	0.9
protein_coding	ECHS1	ENSG00000127884.4	154	142	0.9
protein_coding	RNF44	ENSG00000146083.11	64	59	0.9
protein_coding	RAP2C	ENSG00000123728.9	51	47	0.9
protein_coding	TM9SF4	ENSG00000101337.15	140	129	0.9
protein_coding	CNOT1	ENSG00000125107.17	140	129	0.9
protein_coding	PSAP	ENSG00000197746.13	2189	2017	0.9
protein_coding	WWP1	ENSG00000123124.13	89	82	0.9
protein_coding	MXRA8	ENSG00000162576.16	2081	1917	0.9
protein_coding	RPS21	ENSG00000171858.17	1382	1273	0.9
protein_coding	OS9	ENSG00000135506.15	431	397	0.9
protein_coding	THUMPD3	ENSG00000134077.15	38	35	0.9
protein_coding	RABEP2	ENSG00000177548.12	38	35	0.9
protein_coding	MFSD3	ENSG00000167700.8	38	35	0.9
protein_coding	ELP3	ENSG00000134014.16	38	35	0.9
protein_coding	NOL7	ENSG00000225921.6	228	210	0.9
protein_coding	AMFR	ENSG00000159461.14	240	221	0.9
protein_coding	LSM¬†3	ENSG00000170860.3	176	162	0.9

protein_coding	MRAS	ENSG00000158186.12	138	127	0.9
lincRNA	ZNF667-AS1	ENSG00000166770.10	25	23	0.9
protein_coding	PEX6	ENSG00000124587.13	25	23	0.9
protein_coding	MRNIP	ENSG00000161010.14	25	23	0.9
protein_coding	C9orf40	ENSG00000135045.6	25	23	0.9
protein_coding	AK9	ENSG00000155085.15	25	23	0.9
protein_coding	ELMSAN1	ENSG00000156030.13	50	46	0.9
protein_coding	SPPL3	ENSG00000157837.15	150	138	0.9
protein_coding	H3F3B	ENSG00000132475.10	878	807	0.9
protein_coding	HDLBP	ENSG00000115677.16	1149	1056	0.9
protein_coding	SMG7	ENSG00000116698.21	37	34	0.9
protein_coding	IRF3	ENSG00000126456.15	37	34	0.9
protein_coding	COL6A3	ENSG00000163359.15	3633	3338	0.9
protein_coding	MEX3C	ENSG00000176624.10	160	147	0.9
protein_coding	KPNB1	ENSG00000108424.9	909	835	0.9
protein_coding	NAA25	ENSG00000111300.9	49	45	0.9
protein_coding	PTPN13	ENSG00000163629.12	98	90	0.9
processed_transcript	MAGI2-AS3	ENSG00000234456.7	110	101	0.9
protein_coding	DDX42	ENSG00000198231.12	134	123	0.9
protein_coding	WDR45B	ENSG00000141580.15	219	201	0.9
protein_coding	MGLL	ENSG00000074416.14	170	156	0.9
protein_coding	NDUFS8	ENSG00000110717.12	109	100	0.9
protein_coding	RPL9	ENSG00000163682.16	1863	1709	0.9
protein_coding	SRI	ENSG00000075142.13	205	188	0.9
protein_coding	SELENOM	ENSG00000198832.10	505	463	0.9
protein_coding	PDCD1LG2	ENSG00000197646.7	12	11	0.9
protein_coding	THOC3	ENSG00000051596.9	12	11	0.9
protein_coding	TRIM14	ENSG00000106785.14	12	11	0.9
protein_coding	ZNF394	ENSG00000160908.14	12	11	0.9
protein_coding	BEND7	ENSG00000165626.17	12	11	0.9
protein_coding	PLGRKT	ENSG00000107020.9	12	11	0.9
protein_coding	TRMU	ENSG00000100416.13	12	11	0.9
protein_coding	USP27X	ENSG00000273820.1	12	11	0.9
protein_coding	NDUFAF5	ENSG00000101247.17	12	11	0.9
protein_coding	RMND1	ENSG00000155906.17	12	11	0.9
protein_coding	NEK3	ENSG00000136098.16	12	11	0.9
protein_coding	IMMP2L	ENSG00000184903.9	12	11	0.9
protein_coding	BHLHB9	ENSG00000198908.11	12	11	0.9
protein_coding	MARC2	ENSG00000117791.15	12	11	0.9
protein_coding	DMD	ENSG00000198947.15	12	11	0.9
protein_coding	CDK20	ENSG00000156345.17	12	11	0.9
protein_coding	PEX12	ENSG00000108733.9	12	11	0.9
protein_coding	SMO	ENSG00000128602.9	12	11	0.9
protein_coding	THAP7	ENSG00000184436.11	24	22	0.9

protein_coding	MRI1	ENSG00000037757.13	24	22	0.9
protein_coding	TTI1	ENSG00000101407.12	24	22	0.9
protein_coding	TMEM42	ENSG00000169964.7	36	33	0.9
protein_coding	SLC9A9	ENSG00000181804.14	48	44	0.9
protein_coding	GPN2	ENSG00000142751.14	48	44	0.9
protein_coding	UFSP2	ENSG00000109775.10	60	55	0.9
protein_coding	MAML2	ENSG00000184384.13	72	66	0.9
protein_coding	SVBP	ENSG00000177868.11	84	77	0.9
protein_coding	PPP1R11	ENSG00000204619.7	96	88	0.9
protein_coding	SMIM15	ENSG00000188725.7	120	110	0.9
protein_coding	IREB2	ENSG00000136381.12	156	143	0.9
protein_coding	PHTF2	ENSG00000006576.16	180	165	0.9
protein_coding	FNIP1	ENSG00000217128.11	180	165	0.9
protein_coding	GFPT1	ENSG00000198380.12	215	197	0.9
protein_coding	TPM2	ENSG00000198467.14	1884	1726	0.9
protein_coding	LHFPL2	ENSG00000145685.13	155	142	0.9
protein_coding	CFL1	ENSG00000172757.12	1715	1571	0.9
protein_coding	SIAE	ENSG00000110013.12	83	76	0.9
protein_coding	NDRG3	ENSG00000101079.20	154	141	0.9
protein_coding	PLOD1	ENSG00000083444.16	450	412	0.9
protein_coding	MRPL45	ENSG00000278845.4	142	130	0.9
protein_coding	LNPEP	ENSG00000113441.15	130	119	0.9
protein_coding	EAPP	ENSG00000129518.8	165	151	0.9
protein_coding	YWHAG	ENSG00000170027.6	318	291	0.9
protein_coding	GPATCH4	ENSG00000160818.16	47	43	0.9
protein_coding	WWC2	ENSG00000151718.15	94	86	0.9
protein_coding	MINOS1	ENSG00000173436.14	176	161	0.9
protein_coding	POLR2E	ENSG00000099817.11	387	354	0.9
protein_coding	RBM15B	ENSG00000259956.1	340	311	0.9
protein_coding	H2AFY	ENSG00000113648.16	281	257	0.9
protein_coding	RPL37A	ENSG00000197756.9	6678	6106	0.9
protein_coding	FUBP3	ENSG00000107164.15	140	128	0.9
protein_coding	DVL3	ENSG00000161202.18	93	85	0.9
protein_coding	PEX19	ENSG00000162735.18	93	85	0.9
protein_coding	ELOA	ENSG00000011007.12	93	85	0.9
antisense	ZFAS1	ENSG00000177410.12	534	488	0.9
protein_coding	ARHGAP17	ENSG00000140750.16	58	53	0.9
protein_coding	TENT2	ENSG00000164329.13	58	53	0.9
protein_coding	KCTD20	ENSG00000112078.13	174	159	0.9
protein_coding	PSMG2	ENSG00000128789.20	174	159	0.9
protein_coding	PSMA3	ENSG00000100567.12	197	180	0.9
protein_coding	FMOD	ENSG00000122176.11	23	21	0.9
protein_coding	MFSD8	ENSG00000164073.10	23	21	0.9
protein_coding	ZNF254	ENSG00000213096.10	23	21	0.9

protein_coding	MEX3A	ENSG00000254726.2	23	21	0.9
protein_coding	VSIG10	ENSG00000176834.13	46	42	0.9
protein_coding	TUSC2	ENSG00000114383.9	46	42	0.9
protein_coding	PIP4K2A	ENSG00000150867.13	46	42	0.9
protein_coding	C19orf12	ENSG00000131943.17	69	63	0.9
protein_coding	IFT46	ENSG00000118096.7	69	63	0.9
protein_coding	SF3B2	ENSG00000087365.15	299	273	0.9
protein_coding	SEC61B	ENSG00000106803.9	493	450	0.9
protein_coding	YIPF5	ENSG00000145817.16	423	386	0.9
protein_coding	ASCC1	ENSG00000138303.17	57	52	0.9
protein_coding	MYOF	ENSG00000138119.16	592	540	0.9
protein_coding	NDUFA9	ENSG00000139180.10	91	83	0.9
protein_coding	GADD45A	ENSG00000116717.12	216	197	0.9
protein_coding	SNRPB2	ENSG00000125870.10	250	228	0.9
protein_coding	FANCE	ENSG00000112039.4	34	31	0.9
protein_coding	RPL30	ENSG00000156482.10	2741	2499	0.9
protein_coding	CREG1	ENSG00000143162.8	181	165	0.9
protein_coding	PRAF2	ENSG00000243279.3	113	103	0.9
protein_coding	SPATS2	ENSG00000123352.17	79	72	0.9
protein_coding	AHR	ENSG00000106546.13	304	277	0.9
protein_coding	CISD3	ENSG00000277972.1	45	41	0.9
protein_coding	MORN2	ENSG00000188010.13	45	41	0.9
protein_coding	FBXL15	ENSG00000107872.12	45	41	0.9
protein_coding	DSTYK	ENSG00000133059.16	135	123	0.9
protein_coding	MDH1	ENSG00000014641.17	292	266	0.9
protein_coding	USP36	ENSG00000055483.19	56	51	0.9
protein_coding	PTPRF	ENSG00000142949.16	179	163	0.9
protein_coding	MCRS1	ENSG00000187778.13	67	61	0.9
protein_coding	SLC10A3	ENSG00000126903.15	67	61	0.9
protein_coding	MBOAT7	ENSG00000125505.16	201	183	0.9
protein_coding	VPS53	ENSG00000141252.19	89	81	0.9
protein_coding	MTCH1	ENSG00000137409.19	856	779	0.9
protein_coding	DLG1	ENSG00000075711.20	100	91	0.9
protein_coding	COX8A	ENSG00000176340.3	333	303	0.9
protein_coding	EGFR	ENSG00000146648.17	355	323	0.9
protein_coding	PPIC	ENSG00000168938.5	199	181	0.9
protein_coding	DUT	ENSG00000128951.13	243	221	0.9
protein_coding	ETFA	ENSG00000140374.15	320	291	0.9
protein_coding	ZNF432	ENSG00000256087.6	11	10	0.9
protein_coding	ZNF234	ENSG00000263002.7	11	10	0.9
antisense	ZNF529-AS1	ENSG00000233527.8	11	10	0.9
protein_coding	DCPS	ENSG00000110063.9	11	10	0.9
protein_coding	COQ10A	ENSG00000135469.13	11	10	0.9
protein_coding	KRBOX4	ENSG00000147121.15	11	10	0.9

protein_coding	FIGNL1	ENSG00000132436.11	11	10	0.9
protein_coding	IFIH1	ENSG00000115267.6	11	10	0.9
protein_coding	ZBTB26	ENSG00000171448.8	11	10	0.9
protein_coding	KATNAL2	ENSG00000167216.16	11	10	0.9
protein_coding	ATAD2B	ENSG00000119778.14	22	20	0.9
protein_coding	INPP5F	ENSG00000198825.13	22	20	0.9
protein_coding	CHRNA1	ENSG00000138435.15	22	20	0.9
protein_coding	RBAK	ENSG00000146587.17	33	30	0.9
protein_coding	FRMD4A	ENSG00000151474.22	33	30	0.9
protein_coding	ESS2	ENSG00000100056.11	33	30	0.9
protein_coding	TATDN3	ENSG00000203705.10	33	30	0.9
protein_coding	FBXL7	ENSG00000183580.9	44	40	0.9
protein_coding	PACS2	ENSG00000179364.13	44	40	0.9
protein_coding	TECPR2	ENSG00000196663.15	55	50	0.9
protein_coding	GLIPR2	ENSG00000122694.15	55	50	0.9
protein_coding	AGGF1	ENSG00000164252.12	66	60	0.9
protein_coding	UBE3B	ENSG00000151148.13	77	70	0.9
protein_coding	SF3B6	ENSG00000115128.6	165	150	0.9
protein_coding	RPL27	ENSG00000131469.14	2022	1838	0.9
protein_coding	NECAP2	ENSG00000157191.19	131	119	0.9
protein_coding	MTPN	ENSG00000105887.10	904	821	0.9
protein_coding	ARPC2	ENSG00000163466.15	1034	939	0.9
protein_coding	TMA16	ENSG00000198498.9	76	69	0.9
protein_coding	SPSB3	ENSG00000162032.15	54	49	0.9
protein_coding	RAP1GDS1	ENSG00000138698.14	108	98	0.9
protein_coding	RAB29	ENSG00000117280.12	162	147	0.9
protein_coding	RPL7	ENSG00000147604.13	1825	1656	0.9
protein_coding	SMPD1	ENSG00000166311.9	205	186	0.9
protein_coding	TSN	ENSG00000211460.11	140	127	0.9
protein_coding	GOPC	ENSG00000047932.13	140	127	0.9
protein_coding	ECM1	ENSG00000143369.14	538	488	0.9
protein_coding	DMXL1	ENSG00000172869.14	43	39	0.9
protein_coding	RPA3	ENSG00000106399.11	43	39	0.9
protein_coding	REPIN1	ENSG00000214022.11	43	39	0.9
protein_coding	TAF6	ENSG00000106290.14	86	78	0.9
protein_coding	RPLP2	ENSG00000177600.8	3241	2939	0.9
protein_coding	DKK¬†3	ENSG00000050165.17	1105	1002	0.9
protein_coding	PIGG	ENSG00000174227.15	75	68	0.9
protein_coding	PHACTR4	ENSG00000204138.12	107	97	0.9
protein_coding	CORO1C	ENSG00000110880.10	331	300	0.9
protein_coding	VWA8	ENSG00000102763.17	32	29	0.9
protein_coding	ZNF500	ENSG00000103199.13	32	29	0.9
antisense	AC093673.1	ENSG00000232533.1	64	58	0.9
protein_coding	PDGFRA	ENSG00000134853.11	3338	3025	0.9

protein_coding	RPS8	ENSG00000142937.11	5064	4589	0.9
protein_coding	HPS1	ENSG00000107521.18	159	144	0.9
protein_coding	LAP3	ENSG00000002549.12	74	67	0.9
protein_coding	SPOCK1	ENSG00000152377.13	148	134	0.9
protein_coding	ACAA2	ENSG00000167315.17	222	201	0.9
protein_coding	HDDC2	ENSG00000111906.17	95	86	0.9
protein_coding	AKT2	ENSG00000105221.16	306	277	0.9
protein_coding	ADK	ENSG00000156110.13	116	105	0.9
protein_coding	C19orf25	ENSG00000119559.15	21	19	0.9
protein_coding	ZBED4	ENSG00000100426.6	21	19	0.9
processed_transcript	GARS-DT	ENSG00000196295.11	21	19	0.9
protein_coding	AGK	ENSG00000006530.16	21	19	0.9
protein_coding	TDRD3	ENSG00000083544.14	42	38	0.9
protein_coding	ZNF528	ENSG00000167555.13	42	38	0.9
protein_coding	ZNF622	ENSG00000173545.4	42	38	0.9
protein_coding	CDK10	ENSG00000185324.21	63	57	0.9
protein_coding	TRAPPC2	ENSG00000196459.13	63	57	0.9
protein_coding	PDPK1	ENSG00000140992.18	84	76	0.9
protein_coding	PBX3	ENSG00000167081.16	105	95	0.9
protein_coding	TULP3	ENSG00000078246.16	126	114	0.9
protein_coding	CASP4	ENSG00000196954.13	126	114	0.9
protein_coding	RNF130	ENSG00000113269.13	199	180	0.9
protein_coding	ZYG11B	ENSG00000162378.12	178	161	0.9
protein_coding	TMEM101	ENSG00000091947.9	73	66	0.9
protein_coding	PARVA	ENSG00000197702.12	552	499	0.9
protein_coding	VHL	ENSG00000134086.7	52	47	0.9
protein_coding	MRPL1	ENSG00000169288.17	52	47	0.9
protein_coding	MBOAT2	ENSG00000143797.11	52	47	0.9
protein_coding	TAF7	ENSG00000178913.7	301	272	0.9
protein_coding	MBP	ENSG00000197971.14	31	28	0.9
protein_coding	PRR14	ENSG00000156858.11	31	28	0.9
protein_coding	SUSD1	ENSG00000106868.16	31	28	0.9
protein_coding	DHX57	ENSG00000163214.20	31	28	0.9
protein_coding	TRAPPC3	ENSG00000054116.11	185	167	0.9
protein_coding	STK10	ENSG00000072786.12	41	37	0.9
protein_coding	ZNF197	ENSG00000186448.14	41	37	0.9
protein_coding	IL6ST	ENSG00000134352.19	981	885	0.9
protein_coding	SLC9A7	ENSG00000065923.9	194	175	0.9
protein_coding	NRBP1	ENSG00000115216.13	102	92	0.9
protein_coding	PSMB4	ENSG00000159377.10	652	588	0.9
protein_coding	KPNA1	ENSG00000114030.12	112	101	0.9
protein_coding	RPL21	ENSG00000122026.10	1028	927	0.9
protein_coding	ABLIM3	ENSG00000173210.19	61	55	0.9
protein_coding	SIDT2	ENSG00000149577.15	122	110	0.9

protein_coding	RBPMS	ENSG00000157110.15	254	229	0.9
protein_coding	GSR	ENSG00000104687.13	233	210	0.9
protein_coding	RANGAP1	ENSG00000100401.19	101	91	0.9
protein_coding	ZHX1	ENSG00000165156.14	161	145	0.9
protein_coding	QTRT2	ENSG00000151576.10	10	9	0.9
protein_coding	CNNM4	ENSG00000158158.11	10	9	0.9
protein_coding	UBE3D	ENSG00000118420.16	10	9	0.9
protein_coding	NR1H3	ENSG00000025434.18	10	9	0.9
protein_coding	FAM171A2	ENSG00000161682.14	10	9	0.9
protein_coding	PGBD4	ENSG00000182405.5	10	9	0.9
protein_coding	PHACTR1	ENSG00000112137.17	10	9	0.9
protein_coding	RPP40	ENSG00000124787.13	10	9	0.9
protein_coding	ANLN	ENSG00000011426.10	10	9	0.9
protein_coding	FUT4	ENSG00000196371.3	10	9	0.9
protein_coding	MST1	ENSG00000173531.15	10	9	0.9
protein_coding	CD320	ENSG00000167775.10	20	18	0.9
protein_coding	SLC25A40	ENSG00000075303.12	20	18	0.9
protein_coding	ZNF568	ENSG00000198453.12	20	18	0.9
protein_coding	PCDHB14	ENSG00000120327.6	20	18	0.9
protein_coding	MBLAC2	ENSG00000176055.9	20	18	0.9
protein_coding	PTBP2	ENSG00000117569.18	20	18	0.9
protein_coding	SPRED2	ENSG00000198369.9	30	27	0.9
protein_coding	EIF2B5	ENSG00000145191.13	30	27	0.9
protein_coding	MTRR	ENSG00000124275.14	30	27	0.9
protein_coding	ABCC10	ENSG00000124574.14	40	36	0.9
protein_coding	DDX41	ENSG00000183258.11	50	45	0.9
protein_coding	PCMTD2	ENSG00000203880.11	70	63	0.9
protein_coding	WDR33	ENSG00000136709.11	80	72	0.9
protein_coding	BTBD2	ENSG00000133243.8	90	81	0.9
protein_coding	HERPUD2	ENSG00000122557.9	90	81	0.9
protein_coding	DRAP1	ENSG00000175550.7	130	117	0.9
protein_coding	NOMO1	ENSG00000103512.14	130	117	0.9
protein_coding	DAG1	ENSG00000173402.11	200	180	0.9
protein_coding	PUM1	ENSG00000134644.15	328	295	0.9
protein_coding	RPL7A	ENSG00000148303.16	3115	2801	0.9
protein_coding	RNF20	ENSG00000155827.11	109	98	0.9
protein_coding	PPP1R12A	ENSG00000058272.18	573	515	0.9
protein_coding	SSBP3	ENSG00000157216.15	69	62	0.9
protein_coding	PRPF8	ENSG00000174231.16	197	177	0.9
protein_coding	CD164	ENSG00000135535.16	502	451	0.9
protein_coding	COL1A2	ENSG00000164692.17	30000	26950	0.9
protein_coding	MGAT2	ENSG00000168282.5	59	53	0.9
protein_coding	UQCC2	ENSG00000137288.9	59	53	0.9
protein_coding	SULF1	ENSG00000137573.13	942	846	0.9

protein_coding	HSDL1	ENSG00000103160.11	49	44	0.9
protein_coding	ABCF2	ENSG00000033050.8	49	44	0.9
protein_coding	EHBP1	ENSG00000115504.14	186	167	0.9
protein_coding	MAGOH	ENSG00000162385.10	39	35	0.9
protein_coding	WDR7	ENSG00000091157.13	39	35	0.9
protein_coding	KCTD10	ENSG00000110906.12	224	201	0.9
protein_coding	NSL1	ENSG00000117697.14	107	96	0.9
protein_coding	PPA1	ENSG00000180817.11	107	96	0.9
protein_coding	VIRMA	ENSG00000164944.11	126	113	0.9
protein_coding	ASB3	ENSG00000115239.21	29	26	0.9
protein_coding	TMEM68	ENSG00000167904.14	29	26	0.9
protein_coding	SPG11	ENSG00000104133.14	58	52	0.9
protein_coding	NCOA1	ENSG00000084676.15	87	78	0.9
protein_coding	CCNC	ENSG00000112237.12	106	95	0.9
protein_coding	STX4	ENSG00000103496.14	77	69	0.9
protein_coding	SPG7	ENSG00000197912.15	115	103	0.9
protein_coding	DDAH2	ENSG00000213722.8	182	163	0.9
protein_coding	USP42	ENSG00000106346.11	67	60	0.9
protein_coding	RPS29	ENSG00000213741.10	3836	3435	0.9
protein_coding	SBF2	ENSG00000133812.15	86	77	0.9
protein_coding	ARL¬†1	ENSG00000120805.13	200	179	0.9
protein_coding	C17orf51	ENSG00000212719.11	19	17	0.9
protein_coding	RBM19	ENSG00000122965.10	19	17	0.9
protein_coding	CIPC	ENSG00000198894.7	19	17	0.9
protein_coding	AP4B1	ENSG00000134262.12	19	17	0.9
protein_coding	ZC3H10	ENSG00000135482.6	19	17	0.9
protein_coding	ZNF248	ENSG00000198105.13	19	17	0.9
protein_coding	DPF3	ENSG00000205683.11	19	17	0.9
protein_coding	TSPAN10	ENSG00000182612.10	19	17	0.9
protein_coding	RPP14	ENSG00000163684.11	38	34	0.9
protein_coding	AP3S2	ENSG00000157823.16	38	34	0.9
protein_coding	TMEM218	ENSG00000150433.9	38	34	0.9
protein_coding	SGSM2	ENSG00000141258.12	76	68	0.9
protein_coding	ELAVL1	ENSG00000066044.14	114	102	0.9
protein_coding	DHRS1	ENSG00000157379.13	114	102	0.9
protein_coding	RNF115	ENSG00000265491.4	228	204	0.9
protein_coding	SERPINE1	ENSG00000106366.8	635	568	0.9
protein_coding	SEC11A	ENSG00000140612.13	558	499	0.9
protein_coding	URM1	ENSG00000167118.10	104	93	0.9
protein_coding	NTAN1	ENSG00000157045.8	208	186	0.9
protein_coding	VGLL3	ENSG00000206538.8	208	186	0.9
protein_coding	PSMA1	ENSG00000129084.17	293	262	0.9
protein_coding	ELK4	ENSG00000158711.13	85	76	0.9
protein_coding	RPLP1	ENSG00000137818.11	6336	5665	0.9

protein_coding	SUB1	ENSG00000113387.11	434	388	0.9
protein_coding	CFAP20	ENSG00000070761.7	47	42	0.9
protein_coding	ZDHHC17	ENSG00000186908.14	47	42	0.9
protein_coding	MRPS11	ENSG00000181991.15	47	42	0.9
protein_coding	CCDC80	ENSG00000091986.15	1645	1470	0.9
protein_coding	PRKG1	ENSG00000185532.16	225	201	0.9
protein_coding	REXO2	ENSG00000076043.9	599	535	0.9
protein_coding	METTL3	ENSG00000165819.11	28	25	0.9
protein_coding	PRR14L	ENSG00000183530.13	28	25	0.9
protein_coding	ODF2	ENSG00000136811.16	28	25	0.9
protein_coding	USP19	ENSG00000172046.18	84	75	0.9
protein_coding	COA3	ENSG00000183978.7	84	75	0.9
protein_coding	RPS23	ENSG00000186468.12	979	874	0.9
processed_transcript	SNHG5	ENSG00000203875.11	1892	1689	0.9
protein_coding	ITM2C	ENSG00000135916.15	409	365	0.9
protein_coding	RPL31	ENSG00000071082.10	3430	3061	0.9
protein_coding	RPLP0	ENSG00000089157.15	3344	2984	0.9
protein_coding	GAA	ENSG00000171298.12	102	91	0.9
protein_coding	MGMT	ENSG00000170430.9	102	91	0.9
protein_coding	ATP5F1A	ENSG00000152234.15	510	455	0.9
protein_coding	TLN1	ENSG00000137076.20	343	306	0.9
protein_coding	SUZ12	ENSG00000178691.10	139	124	0.9
protein_coding	DHX33	ENSG00000005100.12	37	33	0.9
protein_coding	TCTA	ENSG00000145022.4	37	33	0.9
protein_coding	ING4	ENSG00000111653.19	37	33	0.9
protein_coding	OGT	ENSG00000147162.13	148	132	0.9
protein_coding	NDUFB7	ENSG00000099795.6	277	247	0.9
protein_coding	EIF3G	ENSG00000130811.11	175	156	0.9
protein_coding	CSTF3	ENSG00000176102.12	92	82	0.9
protein_coding	PPARA	ENSG00000186951.16	101	90	0.9
protein_coding	CLTC	ENSG00000141367.11	1146	1021	0.9
protein_coding	TSFM	ENSG00000123297.17	55	49	0.9
protein_coding	NABP2	ENSG00000139579.12	55	49	0.9
protein_coding	CCT4	ENSG00000115484.14	348	310	0.9
protein_coding	DPY19L4	ENSG00000156162.16	128	114	0.9
protein_coding	PFN2	ENSG00000070087.14	402	358	0.9
protein_coding	CALM2	ENSG00000143933.16	904	805	0.9
protein_coding	USP4	ENSG00000114316.12	73	65	0.9
protein_coding	SIK3	ENSG00000160584.15	82	73	0.9
protein_coding	ZNF106	ENSG00000103994.17	770	685	0.9
protein_coding	RAPGEF5	ENSG00000136237.18	9	8	0.9
protein_coding	PRR7	ENSG00000131188.11	9	8	0.9
protein_coding	SIPA1	ENSG00000213445.9	9	8	0.9
antisense	MINCR	ENSG00000253716.5	9	8	0.9

protein_coding	HECW1	ENSG00000002746.14	9	8	0.9
protein_coding	RAD51B	ENSG00000182185.18	9	8	0.9
protein_coding	ETFBKMT	ENSG00000139160.13	9	8	0.9
protein_coding	PRKD2	ENSG00000105287.12	18	16	0.9
protein_coding	JMJD4	ENSG00000081692.12	18	16	0.9
protein_coding	ZKSCAN5	ENSG00000196652.11	18	16	0.9
protein_coding	NPTXR	ENSG00000221890.3	18	16	0.9
protein_coding	ATXN7L1	ENSG00000146776.14	18	16	0.9
protein_coding	ZNF16	ENSG00000170631.14	18	16	0.9
protein_coding	KIF20B	ENSG00000138182.14	27	24	0.9
protein_coding	BRF2	ENSG00000104221.12	27	24	0.9
protein_coding	SNPH	ENSG00000101298.14	27	24	0.9
protein_coding	NOM1	ENSG00000146909.7	27	24	0.9
protein_coding	ZNF688	ENSG00000229809.8	27	24	0.9
protein_coding	TAOK2	ENSG00000149930.17	36	32	0.9
protein_coding	SLF2	ENSG00000119906.12	36	32	0.9
protein_coding	RRP7A	ENSG00000189306.10	36	32	0.9
protein_coding	ABCB10	ENSG00000135776.4	36	32	0.9
protein_coding	MRPL55	ENSG00000162910.18	45	40	0.9
protein_coding	ECHDC2	ENSG00000121310.16	45	40	0.9
protein_coding	DAXX	ENSG00000204209.12	45	40	0.9
protein_coding	TINF2	ENSG00000092330.16	54	48	0.9
protein_coding	MITD1	ENSG00000158411.11	54	48	0.9
protein_coding	TMEM19	ENSG00000139291.13	63	56	0.9
protein_coding	PELI2	ENSG00000139946.9	63	56	0.9
protein_coding	BMPR1A	ENSG00000107779.13	72	64	0.9
protein_coding	CSE1L	ENSG00000124207.16	72	64	0.9
protein_coding	HMGB3	ENSG00000029993.14	81	72	0.9
protein_coding	DYM	ENSG00000141627.13	90	80	0.9
protein_coding	SMIM4	ENSG00000168273.7	126	112	0.9
protein_coding	FAM8A1	ENSG00000137414.5	144	128	0.9
protein_coding	FOXD1	ENSG00000251493.4	225	200	0.9
protein_coding	ESYT1	ENSG00000139641.12	251	223	0.9
protein_coding	ITGBL1	ENSG00000198542.13	690	613	0.9
protein_coding	RPL35A	ENSG00000182899.16	2452	2178	0.9
protein_coding	FLOT1	ENSG00000137312.14	160	142	0.9
protein_coding	MBD3	ENSG00000071655.17	71	63	0.9
protein_coding	RHOBTB2	ENSG00000008853.16	71	63	0.9
protein_coding	CIAO1	ENSG00000144021.2	62	55	0.9
protein_coding	TP53BP2	ENSG00000143514.16	62	55	0.9
protein_coding	TRAM2	ENSG00000065308.4	239	212	0.9
protein_coding	RNF11	ENSG00000123091.4	416	369	0.9
protein_coding	EHMT2	ENSG00000204371.11	53	47	0.9
protein_coding	NRIP1	ENSG00000180530.10	106	94	0.9

protein_coding	RNF141	ENSG00000110315.6	106	94	0.9
protein_coding	NEDD8	ENSG00000129559.12	344	305	0.9
protein_coding	AC118549.1	ENSG00000036549.12	97	86	0.9
protein_coding	RNF157	ENSG00000141576.15	44	39	0.9
protein_coding	GLRX3	ENSG00000108010.11	132	117	0.9
protein_coding	IGBP1	ENSG00000089289.15	264	234	0.9
protein_coding	NDUFAF8	ENSG00000224877.3	211	187	0.9
protein_coding	SEC14L1	ENSG00000129657.15	184	163	0.9
protein_coding	SLC38A5	ENSG00000017483.14	35	31	0.9
protein_coding	KIFC2	ENSG00000167702.12	35	31	0.9
protein_coding	THAP1	ENSG00000131931.8	35	31	0.9
protein_coding	FEM1A	ENSG00000141965.4	35	31	0.9
protein_coding	BTN3A2	ENSG00000186470.13	35	31	0.9
protein_coding	NIT1	ENSG00000158793.13	35	31	0.9
protein_coding	PDCD2	ENSG00000071994.10	131	116	0.9
protein_coding	GPX8	ENSG00000164294.13	323	286	0.9
protein_coding	FEZ1	ENSG00000149557.13	96	85	0.9
protein_coding	DNAJC15	ENSG00000120675.5	157	139	0.9
protein_coding	PMAIP1	ENSG00000141682.11	61	54	0.9
antisense	PSMA3-AS1	ENSG00000257621.7	61	54	0.9
protein_coding	MZT2A	ENSG00000173272.15	113	100	0.9
protein_coding	KAT6A	ENSG00000083168.10	139	123	0.9
protein_coding	ATP5F1C	ENSG00000165629.19	356	315	0.9
protein_coding	DUSP18	ENSG00000167065.13	26	23	0.9
protein_coding	PELI3	ENSG00000174516.14	26	23	0.9
protein_coding	ABHD17A	ENSG00000129968.15	26	23	0.9
protein_coding	C1orf122	ENSG00000197982.13	164	145	0.9
transcribed_unitary_pseudogene	AHSA2P	ENSG00000173209.23	69	61	0.9
protein_coding	GPALPP1	ENSG00000133114.17	69	61	0.9
protein_coding	ALG3	ENSG00000214160.9	69	61	0.9
protein_coding	COX5B	ENSG00000135940.6	336	297	0.9
protein_coding	RAP1B	ENSG00000127314.17	155	137	0.9
protein_coding	NOV	ENSG00000136999.4	86	76	0.9
protein_coding	TRA2B	ENSG00000136527.17	172	152	0.9
protein_coding	CST3	ENSG00000101439.8	361	319	0.9
protein_coding	CHMP3	ENSG00000115561.15	352	311	0.9
protein_coding	LPAR1	ENSG00000198121.13	1408	1244	0.9
protein_coding	ANKRD13C	ENSG00000118454.12	60	53	0.9
protein_coding	NOL3	ENSG00000140939.14	60	53	0.9
protein_coding	OSBPL5	ENSG00000021762.19	137	121	0.9
protein_coding	ARPC5L	ENSG00000136950.13	77	68	0.9
protein_coding	AP3M1	ENSG00000185009.12	77	68	0.9

protein_coding	CDC37	ENSG00000105401.8	503	444	0.9
protein_coding	ICMT	ENSG00000116237.15	264	233	0.9
protein_coding	LPXN	ENSG00000110031.12	17	15	0.9
protein_coding	ZNF136	ENSG00000196646.11	17	15	0.9
protein_coding	BPHL	ENSG00000137274.12	17	15	0.9
protein_coding	EEF1AKMT3	ENSG00000123427.16	17	15	0.9
protein_coding	SDHAF4	ENSG00000154079.5	17	15	0.9
protein_coding	COA7	ENSG00000162377.5	17	15	0.9
protein_coding	FUZ	ENSG00000010361.13	17	15	0.9
protein_coding	WDR77	ENSG00000116455.13	17	15	0.9
protein_coding	LYRM9	ENSG00000232859.9	17	15	0.9
protein_coding	MRPS25	ENSG00000131368.7	34	30	0.9
protein_coding	PRPSAP2	ENSG00000141127.14	34	30	0.9
protein_coding	CPNE8	ENSG00000139117.13	34	30	0.9
protein_coding	HSD17B10	ENSG00000072506.12	34	30	0.9
protein_coding	STRN	ENSG00000115808.11	51	45	0.9
protein_coding	TMX2	ENSG00000213593.9	51	45	0.9
protein_coding	COMMD1	ENSG00000173163.10	51	45	0.9
protein_coding	FNIP2	ENSG00000052795.12	51	45	0.9
protein_coding	ZNF3	ENSG00000166526.16	51	45	0.9
protein_coding	ABL2	ENSG00000143322.19	68	60	0.9
protein_coding	RTL8B	ENSG00000212747.4	119	105	0.9
protein_coding	RGCC	ENSG00000102760.12	119	105	0.9
protein_coding	RNF181	ENSG00000168894.9	204	180	0.9
protein_coding	PLIN3	ENSG00000105355.8	508	448	0.9
protein_coding	PRDX6	ENSG00000117592.8	584	515	0.9
protein_coding	PSMC6	ENSG00000100519.11	93	82	0.9
protein_coding	RPL15	ENSG00000174748.20	2357	2078	0.9
protein_coding	WIPF1	ENSG00000115935.17	152	134	0.9
protein_coding	SLC44A2	ENSG00000129353.14	228	201	0.9
protein_coding	BAG3	ENSG00000151929.9	160	141	0.9
protein_coding	CIRBP	ENSG00000099622.13	261	230	0.9
protein_coding	EIF2D	ENSG00000143486.15	143	126	0.9
protein_coding	MYL6	ENSG00000092841.18	1891	1666	0.9
protein_coding	RNF121	ENSG00000137522.17	42	37	0.9
protein_coding	CMTM3	ENSG00000140931.19	84	74	0.9
protein_coding	XRCC6	ENSG00000196419.12	134	118	0.9
protein_coding	CHCHD10	ENSG00000250479.8	268	236	0.9
protein_coding	KDELR3	ENSG00000100196.10	335	295	0.9
protein_coding	HNRNPH3	ENSG00000096746.17	226	199	0.9
protein_coding	ZFYVE28	ENSG00000159733.13	25	22	0.9
protein_coding	LIMK1	ENSG00000106683.14	50	44	0.9
protein_coding	SUPT20H	ENSG00000102710.19	75	66	0.9
protein_coding	SCP2	ENSG00000116171.17	299	263	0.9

protein_coding	SMURF2	ENSG00000108854.15	83	73	0.9
protein_coding	FLCN	ENSG00000154803.12	83	73	0.9
protein_coding	NACC2	ENSG00000148411.7	141	124	0.9
protein_coding	RAP1A	ENSG00000116473.14	257	226	0.9
protein_coding	MED8	ENSG00000159479.16	58	51	0.9
protein_coding	GPS1	ENSG00000169727.12	91	80	0.9
protein_coding	SLC35B3	ENSG00000124786.11	91	80	0.9
protein_coding	TUBB	ENSG00000196230.12	1125	989	0.9
protein_coding	PPWD1	ENSG00000113593.11	33	29	0.9
protein_coding	ZKSCAN8	ENSG00000198315.10	33	29	0.9
protein_coding	DRG1	ENSG00000185721.12	66	58	0.9
protein_coding	RAC1	ENSG00000136238.17	981	862	0.9
protein_coding	CCDC90B	ENSG00000137500.9	206	181	0.9
protein_coding	INTS13	ENSG00000064102.14	74	65	0.9
protein_coding	NOTCH2	ENSG00000134250.19	296	260	0.9
protein_coding	HLA-A	ENSG00000206503.12	935	821	0.9
protein_coding	NAA60	ENSG00000122390.18	41	36	0.9
protein_coding	TUSC3	ENSG00000104723.20	287	252	0.9
protein_coding	XBP1	ENSG00000100219.16	172	151	0.9
protein_coding	HNRNPF	ENSG00000169813.16	344	302	0.9
protein_coding	TNFRSF1A	ENSG00000067182.7	262	230	0.9
protein_coding	NAP1L4	ENSG00000205531.12	278	244	0.9
protein_coding	FAM219B	ENSG00000178761.14	49	43	0.9
protein_coding	RIOK1	ENSG00000124784.8	49	43	0.9
protein_coding	C4orf48	ENSG00000243449.6	98	86	0.9
protein_coding	SCPEP1	ENSG00000121064.12	155	136	0.9
protein_coding	STIP1	ENSG00000168439.16	106	93	0.9
protein_coding	PHLDB1	ENSG00000019144.18	163	143	0.9
protein_coding	EDF1	ENSG00000107223.12	277	243	0.9
protein_coding	UQCC1	ENSG00000101019.21	57	50	0.9
protein_coding	PIGM	ENSG00000143315.7	57	50	0.9
protein_coding	ZNF791	ENSG00000173875.13	65	57	0.9
protein_coding	FRG1	ENSG00000109536.11	138	121	0.9
protein_coding	GOLPH3	ENSG00000113384.13	284	249	0.9
protein_coding	DHX30	ENSG00000132153.14	73	64	0.9
protein_coding	NDUFB6	ENSG00000165264.10	146	128	0.9
protein_coding	TMEM245	ENSG00000106771.12	373	327	0.9
protein_coding	INPP1	ENSG00000151689.12	81	71	0.9
protein_coding	COMMD8	ENSG00000169019.10	89	78	0.9
protein_coding	CD44	ENSG00000026508.18	2466	2161	0.9
protein_coding	MAPK6	ENSG00000069956.11	97	85	0.9
protein_coding	TMEM120A	ENSG00000189077.10	97	85	0.9
protein_coding	SRGAP1	ENSG00000196935.8	194	170	0.9
protein_coding	WDFY3	ENSG00000163625.15	105	92	0.9

protein_coding	UGCG	ENSG00000148154.9	129	113	0.9
protein_coding	IQGAP1	ENSG00000140575.12	564	494	0.9
protein_coding	HCFC1	ENSG00000172534.13	153	134	0.9
protein_coding	WARS	ENSG00000140105.17	177	155	0.9
protein_coding	PTTG1IP	ENSG00000183255.11	1118	979	0.9
bidirectional_promoter_lnc RNA	TYMSOS	ENSG00000176912.3	8	7	0.9
protein_coding	ZNF384	ENSG00000126746.17	8	7	0.9
protein_coding	ZNF404	ENSG00000176222.8	8	7	0.9
protein_coding	HHEX	ENSG00000152804.10	8	7	0.9
protein_coding	EPGN	ENSG00000182585.9	8	7	0.9
protein_coding	PNMA2	ENSG00000240694.8	8	7	0.9
antisense	AF001548.2	ENSG00000263335.1	8	7	0.9
protein_coding	TYSND1	ENSG00000156521.13	8	7	0.9
protein_coding	KIRREL3	ENSG00000149571.11	8	7	0.9
protein_coding	TEX15	ENSG00000133863.8	8	7	0.9
protein_coding	TXNDC16	ENSG00000087301.8	8	7	0.9
antisense	SDCBP2-AS1	ENSG00000234684.6	8	7	0.9
protein_coding	PRDM16	ENSG00000142611.16	8	7	0.9
protein_coding	ZNF671	ENSG00000083814.13	8	7	0.9
protein_coding	TACR1	ENSG00000115353.10	8	7	0.9
protein_coding	ZNF682	ENSG00000197124.11	8	7	0.9
protein_coding	ALG6	ENSG00000088035.16	16	14	0.9
protein_coding	TTF2	ENSG00000116830.11	16	14	0.9
processed_transcript	SNHG3	ENSG00000242125.3	16	14	0.9
protein_coding	SRR	ENSG00000167720.12	16	14	0.9
protein_coding	USP30	ENSG00000135093.12	16	14	0.9
antisense	TMEM161B-	ENSG00000247828.7	16	14	0.9
protein_coding	C16orf70	ENSG00000125149.11	16	14	0.9
protein_coding	NBPF3	ENSG00000142794.18	16	14	0.9
lincRNA	AL390728.6	ENSG00000259865.1	16	14	0.9
protein_coding	UPRT	ENSG00000094841.13	24	21	0.9
unprocessed_pseudogene	AC245060.4	ENSG00000272779.1	24	21	0.9
protein_coding	RUBCN	ENSG00000145016.15	24	21	0.9
protein_coding	WDR3	ENSG00000065183.15	24	21	0.9
protein_coding	SMARCD2	ENSG00000108604.15	24	21	0.9
protein_coding	C21orf91	ENSG00000154642.10	24	21	0.9
protein_coding	METTL17	ENSG00000165792.17	24	21	0.9
protein_coding	PLCD4	ENSG00000115556.13	24	21	0.9
protein_coding	KIAA0513	ENSG00000135709.12	24	21	0.9
protein_coding	ETAA1	ENSG00000143971.8	32	28	0.9
protein_coding	RBM4	ENSG00000173933.20	32	28	0.9
protein_coding	CBFA2T2	ENSG00000078699.21	32	28	0.9

protein_coding	KDM2B	ENSG00000089094.18	32	28	0.9
protein_coding	ZNF830	ENSG00000198783.5	32	28	0.9
protein_coding	CNOT3	ENSG00000088038.18	32	28	0.9
protein_coding	HACD4	ENSG00000188921.13	40	35	0.9
protein_coding	PDSS2	ENSG00000164494.11	48	42	0.9
protein_coding	SDR39U1	ENSG00000100445.17	48	42	0.9
protein_coding	ISY1	ENSG00000240682.9	48	42	0.9
protein_coding	ATP7A	ENSG00000165240.19	56	49	0.9
protein_coding	PFKP	ENSG00000067057.16	64	56	0.9
protein_coding	DPP3	ENSG00000254986.7	64	56	0.9
protein_coding	CCDC14	ENSG00000175455.14	64	56	0.9
protein_coding	GGCX	ENSG00000115486.11	80	70	0.9
protein_coding	DERA	ENSG00000023697.12	80	70	0.9
protein_coding	WDR41	ENSG00000164253.13	112	98	0.9
protein_coding	TAF15	ENSG00000270647.5	176	154	0.9
protein_coding	MPG	ENSG00000103152.11	240	210	0.9
protein_coding	NFE2L2	ENSG00000116044.15	463	405	0.9
protein_coding	HADHB	ENSG00000138029.13	415	363	0.9
protein_coding	TOMM20	ENSG00000173726.10	303	265	0.9
protein_coding	CD82	ENSG00000085117.11	151	132	0.9
protein_coding	ENPP2	ENSG00000136960.12	270	236	0.9
protein_coding	PDLIM4	ENSG00000131435.12	103	90	0.9
protein_coding	ANAPC11	ENSG00000141552.17	292	255	0.9
protein_coding	C3orf18	ENSG00000088543.14	63	55	0.9
protein_coding	RPS25	ENSG00000118181.10	1259	1099	0.9
protein_coding	METTL26	ENSG00000130731.15	118	103	0.9
protein_coding	ZNF664	ENSG00000179195.15	173	151	0.9
protein_coding	RNF217	ENSG00000146373.16	55	48	0.9
protein_coding	NAGA	ENSG00000198951.11	55	48	0.9
protein_coding	VPS45	ENSG00000136631.14	55	48	0.9
protein_coding	PEX11B	ENSG00000131779.10	55	48	0.9
protein_coding	GOT2	ENSG00000125166.12	110	96	0.9
protein_coding	C12orf75	ENSG00000235162.8	306	267	0.9
protein_coding	DYRK2	ENSG00000127334.10	47	41	0.9
protein_coding	PERP	ENSG00000112378.11	610	532	0.9
protein_coding	PCID2	ENSG00000126226.21	78	68	0.9
protein_coding	DPP8	ENSG00000074603.18	187	163	0.9
protein_coding	FIP1L1	ENSG00000145216.15	296	258	0.9
protein_coding	UBAC1	ENSG00000130560.8	70	61	0.9
protein_coding	AP1M1	ENSG00000072958.8	70	61	0.9
protein_coding	ACOT2	ENSG00000119673.14	70	61	0.9
protein_coding	PCNP	ENSG00000081154.11	280	244	0.9
protein_coding	CKAP5	ENSG00000175216.14	101	88	0.9
protein_coding	CLIP3	ENSG00000105270.14	194	169	0.9

protein_coding	ATAD2	ENSG00000156802.12	31	27	0.9
protein_coding	PPIH	ENSG00000171960.10	31	27	0.9
protein_coding	CRKL	ENSG00000099942.12	62	54	0.9
protein_coding	KDM3B	ENSG00000120733.13	62	54	0.9
protein_coding	NUDT16	ENSG00000198585.11	62	54	0.9
protein_coding	SCAF8	ENSG00000213079.9	62	54	0.9
protein_coding	RPL29	ENSG00000162244.11	589	513	0.9
protein_coding	SNX6	ENSG00000129515.18	209	182	0.9
protein_coding	BCL7B	ENSG00000106635.7	108	94	0.9
protein_coding	ZDHHC7	ENSG00000153786.12	362	315	0.9
protein_coding	ARHGEF7	ENSG00000102606.18	77	67	0.9
protein_coding	REX1BD	ENSG00000006015.17	77	67	0.9
protein_coding	WIPF2	ENSG00000171475.13	223	194	0.9
protein_coding	NPR¬†3	ENSG00000113389.15	23	20	0.9
protein_coding	COLEC10	ENSG00000184374.2	23	20	0.9
protein_coding	MSH2	ENSG00000095002.14	23	20	0.9
protein_coding	TMEM254	ENSG00000133678.13	23	20	0.9
protein_coding	INTS8	ENSG00000164941.13	46	40	0.9
protein_coding	ARAF	ENSG00000078061.12	46	40	0.9
protein_coding	HNRNPA0	ENSG00000177733.6	467	406	0.9
protein_coding	CBR1	ENSG00000159228.12	275	239	0.9
protein_coding	IDS	ENSG00000010404.17	229	199	0.9
protein_coding	ATF6	ENSG00000118217.5	137	119	0.9
protein_coding	CORO1B	ENSG00000172725.13	175	152	0.9
protein_coding	TMEM219	ENSG00000149932.16	175	152	0.9
protein_coding	HILPDA	ENSG00000135245.9	38	33	0.9
protein_coding	ARHGEF19	ENSG00000142632.16	38	33	0.9
protein_coding	RBBP9	ENSG00000089050.15	38	33	0.9
protein_coding	RANBP9	ENSG00000010017.12	190	165	0.9
protein_coding	TGFBR3	ENSG00000069702.10	304	264	0.9
protein_coding	FAM129B	ENSG00000136830.11	1063	923	0.9
protein_coding	PHF14	ENSG00000106443.16	167	145	0.9
protein_coding	ZCCHC14	ENSG00000140948.11	53	46	0.9
protein_coding	GOLPH3L	ENSG00000143457.10	68	59	0.9
protein_coding	ADGRE5	ENSG00000123146.19	151	131	0.9
protein_coding	NAA35	ENSG00000135040.15	128	111	0.9
protein_coding	EIF3H	ENSG00000147677.10	564	489	0.9
protein_coding	RNGTT	ENSG00000111880.15	15	13	0.9
protein_coding	LAMA1	ENSG00000101680.14	15	13	0.9
protein_coding	LRRTM2	ENSG00000146006.7	15	13	0.9
protein_coding	TTC27	ENSG00000018699.12	15	13	0.9
antisense	MBNL1-AS1	ENSG00000229619.3	15	13	0.9
protein_coding	TXNRD2	ENSG00000184470.20	15	13	0.9
protein_coding	TTC30A	ENSG00000197557.6	15	13	0.9

protein_coding	TTLL1	ENSG00000100271.16	15	13	0.9
processed_pseudogene	RPL41P2	ENSG00000256338.2	15	13	0.9
protein_coding	ZSCAN9	ENSG00000137185.12	15	13	0.9
protein_coding	AKR1C2	ENSG00000151632.17	15	13	0.9
protein_coding	TOP1MT	ENSG00000184428.12	30	26	0.9
protein_coding	RFX5	ENSG00000143390.17	45	39	0.9
protein_coding	SOGA1	ENSG00000149639.14	45	39	0.9
protein_coding	LCMT1	ENSG00000205629.11	45	39	0.9
protein_coding	ORC4	ENSG00000115947.13	45	39	0.9
protein_coding	ZNHIT6	ENSG00000117174.10	75	65	0.9
protein_coding	ZDHHC4	ENSG00000136247.14	75	65	0.9
protein_coding	WIPI2	ENSG00000157954.14	165	143	0.9
protein_coding	MGST3	ENSG00000143198.12	832	721	0.9
protein_coding	CTSB	ENSG00000164733.20	5267	4564	0.9
protein_coding	METTL23	ENSG00000181038.13	67	58	0.9
protein_coding	JPT1	ENSG00000189159.15	134	116	0.9
protein_coding	SEC31A	ENSG00000138674.16	632	547	0.9
protein_coding	SNX12	ENSG00000147164.11	171	148	0.9
protein_coding	ZMIZ2	ENSG00000122515.14	52	45	0.9
protein_coding	CHERP	ENSG00000085872.14	52	45	0.9
protein_coding	ABCD4	ENSG00000119688.20	52	45	0.9
protein_coding	HIP1	ENSG00000127946.16	52	45	0.9
protein_coding	C1orf35	ENSG00000143793.12	52	45	0.9
protein_coding	DCAF10	ENSG00000122741.15	156	135	0.9
protein_coding	PRMT2	ENSG00000160310.17	208	180	0.9
protein_coding	AHCYL1	ENSG00000168710.17	765	662	0.9
protein_coding	TRIM56	ENSG00000169871.12	200	173	0.9
protein_coding	G2E3	ENSG00000092140.15	37	32	0.9
protein_coding	MFHAS1	ENSG00000147324.10	37	32	0.9
protein_coding	GSS	ENSG00000100983.10	37	32	0.9
protein_coding	UBE2L3	ENSG00000185651.14	170	147	0.9
protein_coding	TLE1	ENSG00000196781.14	133	115	0.9
protein_coding	CYFIP1	ENSG00000273749.4	155	134	0.9
protein_coding	MRPL10	ENSG00000159111.12	59	51	0.9
protein_coding	SYNPO2	ENSG00000172403.10	59	51	0.9
protein_coding	ATP2B4	ENSG00000058668.14	1111	960	0.9
protein_coding	DHCR24	ENSG00000116133.12	272	235	0.9
protein_coding	TMEM43	ENSG00000170876.7	338	292	0.9
protein_coding	TRIM47	ENSG00000132481.6	22	19	0.9
protein_coding	MARK2	ENSG00000072518.20	22	19	0.9
protein_coding	RGS19	ENSG00000171700.13	22	19	0.9
protein_coding	PROS1	ENSG00000184500.15	22	19	0.9
protein_coding	NME6	ENSG00000172113.9	22	19	0.9
protein_coding	LRRC8D	ENSG00000171492.14	44	38	0.9

protein_coding	VPS4A	ENSG00000132612.15	66	57	0.9
protein_coding	VPS39	ENSG00000166887.15	66	57	0.9
protein_coding	TMEM126B	ENSG00000171204.12	66	57	0.9
protein_coding	TULP4	ENSG00000130338.12	117	101	0.9
protein_coding	CLCN7	ENSG00000103249.17	95	82	0.9
protein_coding	TXN2	ENSG00000100348.9	95	82	0.9
protein_coding	EIF2B1	ENSG00000111361.12	95	82	0.9
protein_coding	ADAMTS1	ENSG00000154734.14	241	208	0.9
protein_coding	SLC26A11	ENSG00000181045.14	73	63	0.9
protein_coding	RING1	ENSG00000204227.4	73	63	0.9
protein_coding	COPS5	ENSG00000121022.13	146	126	0.9
protein_coding	PELO	ENSG00000152684.10	51	44	0.9
protein_coding	DCN	ENSG00000011465.16	4546	3922	0.9
protein_coding	EIF3M	ENSG00000149100.12	415	358	0.9
protein_coding	NDUFAF2	ENSG00000164182.10	80	69	0.9
transcribed_unprocessed_pseudogene	MT1L	ENSG00000260549.1	29	25	0.9
protein_coding	SCAPER	ENSG00000140386.12	29	25	0.9
protein_coding	POMGNT1	ENSG00000085998.13	58	50	0.9
protein_coding	STIM2	ENSG00000109689.15	58	50	0.9
protein_coding	LRP10	ENSG00000197324.8	862	743	0.9
protein_coding	RNF149	ENSG00000163162.8	123	106	0.9
protein_coding	RPS16	ENSG00000105193.8	2203	1898	0.9
protein_coding	SLC25A46	ENSG00000164209.16	130	112	0.9
protein_coding	ACBD6	ENSG00000230124.7	72	62	0.9
protein_coding	ASCC2	ENSG00000100325.14	72	62	0.9
protein_coding	CRTC3	ENSG00000140577.15	72	62	0.9
protein_coding	PSMB2	ENSG00000126067.11	216	186	0.9
protein_coding	DDX17	ENSG00000100201.20	792	682	0.9
protein_coding	RILPL2	ENSG00000150977.10	115	99	0.9
protein_coding	MTREX	ENSG00000039123.15	79	68	0.9
protein_coding	RBM27	ENSG00000091009.7	43	37	0.9
protein_coding	LRBA	ENSG00000198589.11	43	37	0.9
protein_coding	FOXJ2	ENSG00000065970.8	43	37	0.9
protein_coding	ACADVL	ENSG00000072778.19	315	271	0.9
protein_coding	PLEKHA4	ENSG00000105559.11	143	123	0.9
protein_coding	H2AFY2	ENSG00000099284.13	50	43	0.9
protein_coding	SRM	ENSG00000116649.9	107	92	0.9
protein_coding	COX7C	ENSG00000127184.12	798	686	0.9
protein_coding	PRSS12	ENSG00000164099.3	349	300	0.9
protein_coding	LHFPL6	ENSG00000183722.8	235	202	0.9
protein_coding	PRKAR2A	ENSG00000114302.15	277	238	0.9
protein_coding	FRYL	ENSG00000075539.14	71	61	0.9

protein_coding	OSBPL9	ENSG00000117859.18	142	122	0.9
protein_coding	DNAJC16	ENSG00000116138.12	78	67	0.9
protein_coding	CYHR1	ENSG00000187954.12	78	67	0.9
protein_coding	PLBD2	ENSG00000151176.7	312	268	0.9
protein_coding	RANBP3	ENSG00000031823.14	85	73	0.9
protein_coding	SNX17	ENSG00000115234.10	99	85	0.9
protein_coding	ATG13	ENSG00000175224.16	113	97	0.9
protein_coding	KLF10	ENSG00000155090.14	113	97	0.9
protein_coding	RASA1	ENSG00000145715.14	120	103	0.9
protein_coding	MID1	ENSG00000101871.14	120	103	0.9
protein_coding	STAT2	ENSG00000170581.13	261	224	0.9
protein_coding	RAB9A	ENSG00000123595.7	134	115	0.9
protein_coding	HNRNPK	ENSG00000165119.20	1049	900	0.9
protein_coding	PKD2	ENSG00000118762.7	176	151	0.9
protein_coding	GPRASP1	ENSG00000198932.12	7	6	0.9
protein_coding	PRDM15	ENSG00000141956.13	7	6	0.9
antisense	AC027601.4	ENSG00000276101.1	7	6	0.9
protein_coding	ZNF814	ENSG00000204514.9	7	6	0.9
protein_coding	SCUBE3	ENSG00000146197.8	7	6	0.9
protein_coding	ZNF665	ENSG00000197497.10	7	6	0.9
protein_coding	NMNAT2	ENSG00000157064.10	7	6	0.9
protein_coding	WDR31	ENSG00000148225.15	7	6	0.9
protein_coding	TM7SF2	ENSG00000149809.14	7	6	0.9
protein_coding	KRTAP1-5	ENSG00000221852.4	7	6	0.9
protein_coding	ZNF764	ENSG00000169951.9	7	6	0.9
protein_coding	ZCWPW1	ENSG00000078487.17	7	6	0.9
sense_intronic	AF131215.6	ENSG00000269918.1	7	6	0.9
protein_coding	APBB3	ENSG00000113108.19	7	6	0.9
lincRNA	AC137767.1	ENSG00000256092.2	7	6	0.9
antisense	U62317.2	ENSG00000272821.1	7	6	0.9
lincRNA	AL683813.1	ENSG00000232611.1	7	6	0.9
processed_transcript	TRIM52-AS1	ENSG00000248275.1	7	6	0.9
antisense	KCNK15-AS1	ENSG00000244558.5	7	6	0.9
protein_coding	AQP11	ENSG00000178301.3	7	6	0.9
protein_coding	THNSL2	ENSG00000144115.16	7	6	0.9
protein_coding	KANTR	ENSG00000232593.7	14	12	0.9
protein_coding	TMEM209	ENSG00000146842.16	14	12	0.9
protein_coding	LYNX1	ENSG00000180155.19	14	12	0.9
processed_pseudogene	AC005912.1	ENSG00000227081.5	14	12	0.9
protein_coding	FAM193B	ENSG00000146067.15	14	12	0.9
protein_coding	RPRD1B	ENSG00000101413.11	21	18	0.9
protein_coding	MAGI2	ENSG00000187391.20	21	18	0.9
protein_coding	INTS4	ENSG00000149262.16	21	18	0.9
protein_coding	MRPL2	ENSG00000112651.11	21	18	0.9

transcribed_unprocessed_pseudogene	CROCCP2	ENSG00000215908.10	21	18	0.9
protein_coding	ARFGAP2	ENSG00000149182.14	28	24	0.9
protein_coding	KCNE4	ENSG00000152049.6	35	30	0.9
protein_coding	MGME1	ENSG00000125871.13	35	30	0.9
protein_coding	CSNK1G1	ENSG00000169118.17	35	30	0.9
protein_coding	CNTNAP1	ENSG00000108797.11	35	30	0.9
protein_coding	PSMG1	ENSG00000183527.11	42	36	0.9
protein_coding	PSMG4	ENSG00000180822.11	42	36	0.9
protein_coding	TBC1D24	ENSG00000162065.13	42	36	0.9
protein_coding	NRDE2	ENSG00000119720.17	49	42	0.9
protein_coding	LETM1	ENSG00000168924.14	56	48	0.9
protein_coding	AKAP10	ENSG00000108599.14	56	48	0.9
protein_coding	POLD2	ENSG00000106628.10	56	48	0.9
protein_coding	FAM160B2	ENSG00000158863.21	56	48	0.9
protein_coding	MAD2L1BP	ENSG00000124688.13	63	54	0.9
protein_coding	DZIP3	ENSG00000198919.12	63	54	0.9
protein_coding	STAMBP	ENSG00000124356.15	98	84	0.9
protein_coding	MYO5A	ENSG00000197535.14	119	102	0.9
protein_coding	KHDRBS1	ENSG00000121774.17	420	360	0.9
protein_coding	PLCB4	ENSG00000101333.16	384	329	0.9
protein_coding	NDUFA11	ENSG00000174886.12	209	179	0.9
protein_coding	RWDD1	ENSG00000111832.12	473	405	0.9
protein_coding	PSMD5	ENSG00000095261.13	111	95	0.9
protein_coding	RPL22L1	ENSG00000163584.17	215	184	0.9
protein_coding	MMP24OS	ENSG00000126005.16	215	184	0.9
protein_coding	ACAT1	ENSG00000075239.13	159	136	0.9
protein_coding	CAVIN1	ENSG00000177469.12	1334	1141	0.9
protein_coding	ZNF655	ENSG00000197343.10	76	65	0.9
protein_coding	RTL8C	ENSG00000134590.13	697	596	0.9
protein_coding	GNL1	ENSG00000204590.12	138	118	0.9
protein_coding	RPS27L	ENSG00000185088.13	971	830	0.9
protein_coding	VAT1	ENSG00000108828.15	936	800	0.9
protein_coding	RBM3	ENSG00000102317.17	337	288	0.9
protein_coding	ELOF1	ENSG00000130165.10	55	47	0.9
protein_coding	RIOK2	ENSG00000058729.10	55	47	0.9
protein_coding	UBE4B	ENSG00000130939.18	151	129	0.9
protein_coding	UNG	ENSG00000076248.10	48	41	0.9
protein_coding	PJA1	ENSG00000181191.11	48	41	0.9
protein_coding	EIF4H	ENSG00000106682.14	96	82	0.9
protein_coding	EMC1	ENSG00000127463.14	96	82	0.9
protein_coding	CAMK2G	ENSG00000148660.20	185	158	0.9
protein_coding	EPB41L2	ENSG00000079819.18	226	193	0.9

protein_coding	HDAC3	ENSG00000171720.9	89	76	0.9
protein_coding	SLFN5	ENSG00000166750.9	445	380	0.9
protein_coding	LRRFIP2	ENSG00000093167.17	219	187	0.9
protein_coding	BFAR	ENSG00000103429.10	130	111	0.9
protein_coding	RWDD4	ENSG00000182552.14	41	35	0.9
protein_coding	FAM126B	ENSG00000155744.9	41	35	0.9
protein_coding	C22orf39	ENSG00000242259.8	41	35	0.9
protein_coding	BCL10	ENSG00000142867.13	82	70	0.9
protein_coding	MAP3K13	ENSG00000073803.13	75	64	0.9
protein_coding	SSU72	ENSG00000160075.11	143	122	0.9
protein_coding	PPP1CB	ENSG00000213639.9	415	354	0.9
protein_coding	LAMP1	ENSG00000185896.10	1476	1259	0.9
protein_coding	CDK2	ENSG00000123374.10	34	29	0.9
protein_coding	MAGOHB	ENSG00000111196.9	34	29	0.9
protein_coding	STX3	ENSG00000166900.16	34	29	0.9
protein_coding	GPR137	ENSG00000173264.14	34	29	0.9
protein_coding	COPS7B	ENSG00000144524.17	34	29	0.9
protein_coding	NF2	ENSG00000186575.17	68	58	0.9
protein_coding	PPP2R5C	ENSG00000078304.19	238	203	0.9
protein_coding	EPS8	ENSG00000151491.13	95	81	0.9
protein_coding	ADAMTS2	ENSG00000087116.15	251	214	0.9
protein_coding	ACIN1	ENSG00000100813.14	122	104	0.9
protein_coding	TMEM214	ENSG00000119777.19	88	75	0.9
protein_coding	ARF3	ENSG00000134287.9	203	173	0.9
protein_coding	GRN	ENSG00000030582.17	480	409	0.9
protein_coding	UBASH3B	ENSG00000154127.9	27	23	0.9
protein_coding	NCAPG2	ENSG00000146918.19	27	23	0.9
protein_coding	PAPOLG	ENSG00000115421.12	27	23	0.9
protein_coding	ELP1	ENSG00000070061.14	27	23	0.9
protein_coding	TXNRD3	ENSG00000197763.15	27	23	0.9
protein_coding	FAM76A	ENSG00000009780.15	27	23	0.9
protein_coding	GPX3	ENSG00000211445.11	27	23	0.9
protein_coding	PSENEN	ENSG00000205155.7	54	46	0.9
protein_coding	KIF16B	ENSG00000089177.18	54	46	0.9
protein_coding	FUCA1	ENSG00000179163.11	162	138	0.9
protein_coding	EPS15	ENSG00000085832.16	216	184	0.9
protein_coding	ESYT2	ENSG00000117868.15	209	178	0.9
protein_coding	CEP170B	ENSG00000099814.15	128	109	0.9
protein_coding	ARID1B	ENSG00000049618.23	256	218	0.9
protein_coding	SDC2	ENSG00000169439.11	343	292	0.9
protein_coding	EIF3E	ENSG00000104408.9	168	143	0.9
protein_coding	STMP1	ENSG00000243317.7	168	143	0.9
protein_coding	APOL2	ENSG00000128335.13	47	40	0.9
protein_coding	TMEM65	ENSG00000164983.7	161	137	0.9

protein_coding	RAB5C	ENSG00000108774.14	483	411	0.9
protein_coding	VWA5A	ENSG00000110002.15	114	97	0.9
protein_coding	CCDC50	ENSG00000152492.14	154	131	0.9
protein_coding	GLMP	ENSG00000198715.12	214	182	0.9
protein_coding	TALDO1	ENSG00000177156.10	987	839	0.9
processed_transcript	LINC-PINT	ENSG00000231721.7	20	17	0.9
antisense	TBX2-AS1	ENSG00000267280.5	20	17	0.9
protein_coding	SLC7A6OS	ENSG00000103061.12	20	17	0.9
protein_coding	FBXO34	ENSG00000178974.9	20	17	0.9
protein_coding	BBS10	ENSG00000179941.7	20	17	0.9
protein_coding	EXD3	ENSG00000187609.15	20	17	0.9
protein_coding	CMC1	ENSG00000187118.13	40	34	0.9
protein_coding	CEBPG	ENSG00000153879.8	40	34	0.9
protein_coding	CMTR1	ENSG00000137200.12	60	51	0.9
protein_coding	NDUFC1	ENSG00000109390.11	260	221	0.9
protein_coding	GBF1	ENSG00000107862.4	113	96	0.8
protein_coding	LTBP4	ENSG00000090006.17	93	79	0.8
protein_coding	AAGAB	ENSG00000103591.12	93	79	0.8
protein_coding	DRAM2	ENSG00000156171.14	93	79	0.8
protein_coding	PRMT5	ENSG00000100462.15	73	62	0.8
protein_coding	NCOA4	ENSG00000266412.5	524	445	0.8
protein_coding	S100PBP	ENSG00000116497.17	53	45	0.8
protein_coding	HMG20B	ENSG00000064961.18	86	73	0.8
protein_coding	LACTB2	ENSG00000147592.8	33	28	0.8
protein_coding	NUMBL	ENSG00000105245.9	184	156	0.8
protein_coding	TTC8	ENSG00000165533.18	59	50	0.8
protein_coding	TNKS1BP1	ENSG00000149115.13	118	100	0.8
protein_coding	MAML1	ENSG00000161021.12	72	61	0.8
protein_coding	MUT	ENSG00000146085.7	72	61	0.8
protein_coding	NSMCE2	ENSG00000156831.7	72	61	0.8
protein_coding	ZNF121	ENSG00000197961.11	72	61	0.8
protein_coding	PCMT1	ENSG00000120265.17	170	144	0.8
protein_coding	COL5A1	ENSG00000130635.15	856	725	0.8
protein_coding	SQSTM1	ENSG00000161011.19	2189	1854	0.8
protein_coding	MAPK9	ENSG00000050748.17	111	94	0.8
protein_coding	RPL12	ENSG00000197958.12	1298	1099	0.8
protein_coding	HNRNPA1	ENSG00000135486.17	306	259	0.8
transcribed_unprocessed_pseudogene	SUZ12P1	ENSG00000264538.6	13	11	0.8
protein_coding	ASPSCR1	ENSG00000169696.15	13	11	0.8
protein_coding	ZNF701	ENSG00000167562.12	13	11	0.8
protein_coding	TMEM62	ENSG00000137842.6	13	11	0.8
protein_coding	PODNL1	ENSG00000132000.12	13	11	0.8

protein_coding	FIGN	ENSG00000182263.13	13	11	0.8
protein_coding	TCF19	ENSG00000137310.11	13	11	0.8
protein_coding	TRPV4	ENSG00000111199.10	13	11	0.8
lincRNA	PAXIP1-AS1	ENSG00000273344.1	13	11	0.8
lincRNA	AP006621.3	ENSG00000255284.1	13	11	0.8
protein_coding	ACOX2	ENSG00000168306.12	26	22	0.8
protein_coding	INTS14	ENSG00000138614.14	39	33	0.8
protein_coding	BID	ENSG00000015475.18	39	33	0.8
protein_coding	UBE2O	ENSG00000175931.12	52	44	0.8
protein_coding	STAM	ENSG00000136738.14	91	77	0.8
protein_coding	FUS	ENSG00000089280.18	169	143	0.8
protein_coding	PHB2	ENSG00000215021.8	325	275	0.8
protein_coding	RBCK1	ENSG00000125826.20	214	181	0.8
protein_coding	CAP1	ENSG00000131236.16	939	794	0.8
protein_coding	CTNNAL1	ENSG00000119326.14	110	93	0.8
protein_coding	UACA	ENSG00000137831.14	375	317	0.8
protein_coding	HERPUD1	ENSG00000051108.14	239	202	0.8
protein_coding	PTDSS1	ENSG00000156471.12	155	131	0.8
protein_coding	AKR1A1	ENSG00000117448.13	142	120	0.8
protein_coding	PRDM2	ENSG00000116731.22	58	49	0.8
protein_coding	FBL	ENSG00000105202.8	116	98	0.8
protein_coding	TFDP1	ENSG00000198176.12	116	98	0.8
protein_coding	DAB2	ENSG00000153071.14	1010	853	0.8
protein_coding	NKIRAS1	ENSG00000197885.10	45	38	0.8
TEC	AC022400.7	ENSG00000279088.1	45	38	0.8
protein_coding	NUP62	ENSG00000213024.11	77	65	0.8
protein_coding	GORASP2	ENSG00000115806.12	231	195	0.8
protein_coding	HSPA4L	ENSG00000164070.11	32	27	0.8
protein_coding	RUVBL2	ENSG00000183207.13	32	27	0.8
protein_coding	CD302	ENSG00000241399.6	32	27	0.8
protein_coding	OSBPL2	ENSG00000130703.16	64	54	0.8
protein_coding	FSCN1	ENSG00000075618.17	624	526	0.8
protein_coding	MED13L	ENSG00000123066.8	140	118	0.8
protein_coding	EFEMP1	ENSG00000115380.19	140	118	0.8
protein_coding	APOL6	ENSG00000221963.5	108	91	0.8
protein_coding	APP	ENSG00000142192.20	1537	1295	0.8
protein_coding	RBPMS2	ENSG00000166831.8	19	16	0.8
protein_coding	SLC25A14	ENSG00000102078.15	19	16	0.8
protein_coding	ITPKC	ENSG00000086544.2	19	16	0.8
protein_coding	SPIN2B	ENSG00000186787.8	19	16	0.8
protein_coding	YPEL4	ENSG00000166793.10	19	16	0.8
protein_coding	POLR3D	ENSG00000168495.12	38	32	0.8
protein_coding	MBIP	ENSG00000151332.18	38	32	0.8
protein_coding	DPYSL4	ENSG00000151640.12	38	32	0.8

protein_coding	TET2	ENSG00000168769.13	38	32	0.8
protein_coding	KIZ	ENSG00000088970.15	38	32	0.8
protein_coding	NOP56	ENSG00000101361.16	76	64	0.8
protein_coding	PTRHD1	ENSG00000184924.5	76	64	0.8
protein_coding	NSMCE3	ENSG00000185115.5	95	80	0.8
protein_coding	LSS	ENSG00000160285.14	95	80	0.8
protein_coding	VAMP7	ENSG00000124333.15	101	85	0.8
protein_coding	KIFAP3	ENSG00000075945.12	101	85	0.8
protein_coding	CD109	ENSG00000156535.14	630	530	0.8
protein_coding	FH	ENSG00000091483.6	170	143	0.8
protein_coding	NAGLU	ENSG00000108784.9	69	58	0.8
protein_coding	PLAU	ENSG00000122861.15	370	311	0.8
protein_coding	C1orf54	ENSG00000118292.8	94	79	0.8
protein_coding	IFRD2	ENSG00000214706.10	25	21	0.8
protein_coding	SETD1A	ENSG00000099381.17	25	21	0.8
protein_coding	ZNF43	ENSG00000198521.11	25	21	0.8
protein_coding	FIBIN	ENSG00000176971.3	25	21	0.8
protein_coding	MSANTD4	ENSG00000170903.10	50	42	0.8
protein_coding	PSME2	ENSG00000100911.15	150	126	0.8
protein_coding	ATP5MC1	ENSG00000159199.13	81	68	0.8
protein_coding	SAP30	ENSG00000164105.3	56	47	0.8
protein_coding	BCAR3	ENSG00000137936.17	56	47	0.8
protein_coding	SUCLG1	ENSG00000163541.11	112	94	0.8
protein_coding	TRMT6	ENSG00000089195.14	31	26	0.8
protein_coding	C12orf76	ENSG00000174456.13	31	26	0.8
protein_coding	NECAP1	ENSG00000089818.17	31	26	0.8
protein_coding	ANKRD27	ENSG00000105186.15	31	26	0.8
protein_coding	PLEKHJ1	ENSG00000104886.11	31	26	0.8
sense_overlapping	AC125257.1	ENSG00000259623.1	31	26	0.8
protein_coding	COMMD9	ENSG00000110442.11	62	52	0.8
protein_coding	MTFR1L	ENSG00000117640.17	62	52	0.8
protein_coding	CNOT2	ENSG00000111596.12	155	130	0.8
protein_coding	POLR1D	ENSG00000186184.17	260	218	0.8
protein_coding	TXN	ENSG00000136810.12	2154	1806	0.8
protein_coding	MAGED2	ENSG00000102316.16	445	373	0.8
protein_coding	RTN4	ENSG00000115310.17	7134	5979	0.8
protein_coding	ZNFX1	ENSG00000124201.14	352	295	0.8
protein_coding	REEP5	ENSG00000129625.12	722	605	0.8
protein_coding	RNF24	ENSG00000101236.16	253	212	0.8
protein_coding	TENT4A	ENSG00000112941.13	37	31	0.8
protein_coding	USP40	ENSG00000085982.13	37	31	0.8
protein_coding	FAF1	ENSG00000185104.19	74	62	0.8
protein_coding	JAZF1	ENSG00000153814.12	111	93	0.8
protein_coding	PREPL	ENSG00000138078.15	148	124	0.8

protein_coding	ATOX1	ENSG00000177556.11	197	165	0.8
protein_coding	POLR3A	ENSG00000148606.12	43	36	0.8
protein_coding	ANP32A	ENSG00000140350.15	86	72	0.8
protein_coding	YTHDF2	ENSG00000198492.15	264	221	0.8
protein_coding	MSANTD3	ENSG00000066697.14	92	77	0.8
protein_coding	SH3BP1	ENSG00000100092.22	49	41	0.8
protein_coding	TRAPPC1	ENSG00000170043.11	147	123	0.8
protein_coding	CDC14B	ENSG00000081377.16	104	87	0.8
protein_coding	DELE1	ENSG00000081791.8	104	87	0.8
protein_coding	AGPAT3	ENSG00000160216.18	208	174	0.8
protein_coding	HSBP1	ENSG00000230989.6	367	307	0.8
protein_coding	SP1	ENSG00000185591.9	55	46	0.8
protein_coding	RPL8	ENSG00000161016.17	1258	1052	0.8
protein_coding	PPP2CB	ENSG00000104695.12	311	260	0.8
protein_coding	DVL2	ENSG00000004975.11	67	56	0.8
protein_coding	RIC8A	ENSG00000177963.14	140	117	0.8
protein_coding	POLR3H	ENSG00000100413.16	73	61	0.8
protein_coding	PUM2	ENSG00000055917.15	152	127	0.8
protein_coding	MAP4	ENSG00000047849.21	764	638	0.8
protein_coding	ARID1A	ENSG00000117713.19	127	106	0.8
protein_coding	PEA15	ENSG00000162734.12	532	444	0.8
protein_coding	ACO2	ENSG00000100412.15	145	121	0.8
protein_coding	PIGK	ENSG00000142892.14	145	121	0.8
protein_coding	RPL19	ENSG00000108298.11	3158	2635	0.8
protein_coding	ZSWIM6	ENSG00000130449.5	169	141	0.8
protein_coding	ZDHHC3	ENSG00000163812.13	193	161	0.8
protein_coding	FBXL17	ENSG00000145743.15	235	196	0.8
protein_coding	CHST1	ENSG00000175264.7	6	5	0.8
antisense	AC073611.1	ENSG00000257605.2	6	5	0.8
protein_coding	SPIN4	ENSG00000186767.6	6	5	0.8
lincRNA	NUP50-DT	ENSG00000226328.6	6	5	0.8
protein_coding	TCEAL7	ENSG00000182916.7	6	5	0.8
protein_coding	ZNF79	ENSG00000196152.10	6	5	0.8
protein_coding	TENM2	ENSG00000145934.16	6	5	0.8
antisense	AL118516.1	ENSG00000260708.1	6	5	0.8
processed_pseudogene	RPL18AP3	ENSG00000213442.5	6	5	0.8
protein_coding	HAGHL	ENSG00000103253.18	6	5	0.8
protein_coding	XAB2	ENSG00000076924.11	6	5	0.8
protein_coding	TELO2	ENSG00000100726.14	6	5	0.8
antisense	AL596202.1	ENSG00000235381.1	6	5	0.8
protein_coding	ZGRF1	ENSG00000138658.15	6	5	0.8
lincRNA	LYRM4-AS1	ENSG00000272142.1	6	5	0.8
protein_coding	FCRLA	ENSG00000132185.16	6	5	0.8
protein_coding	ZNF215	ENSG00000149054.15	6	5	0.8

protein_coding	XYLB	ENSG00000093217.10	6	5	0.8
protein_coding	KYAT1	ENSG00000171097.13	6	5	0.8
sense_intronic	Z83843.1	ENSG00000271533.1	6	5	0.8
protein_coding	WSCD1	ENSG00000179314.14	6	5	0.8
protein_coding	CLSPN	ENSG00000092853.13	6	5	0.8
protein_coding	HIP1R	ENSG00000130787.13	6	5	0.8
lincRNA	AL157702.2	ENSG00000227482.1	6	5	0.8
protein_coding	KIAA1328	ENSG00000150477.14	6	5	0.8
protein_coding	ABCG2	ENSG00000118777.11	6	5	0.8
protein_coding	TMEM59L	ENSG00000105696.8	6	5	0.8
snRNA	RNU6ATAC	ENSG00000221676.1	6	5	0.8
protein_coding	MX1	ENSG00000157601.13	6	5	0.8
protein_coding	GPC2	ENSG00000213420.7	6	5	0.8
protein_coding	COLGALT2	ENSG00000198756.11	6	5	0.8
protein_coding	RFXAP	ENSG00000133111.3	6	5	0.8
antisense	IDH1-AS1	ENSG00000231908.1	6	5	0.8
protein_coding	EVA1C	ENSG00000166979.12	12	10	0.8
protein_coding	PSPN	ENSG00000125650.4	12	10	0.8
protein_coding	CLEC2B	ENSG00000110852.4	12	10	0.8
protein_coding	PHLPP2	ENSG00000040199.18	12	10	0.8
protein_coding	HHAT	ENSG00000054392.12	12	10	0.8
protein_coding	ZNF821	ENSG00000102984.14	12	10	0.8
protein_coding	NPIPA1	ENSG00000183426.16	12	10	0.8
transcribed_unitary_pseudogene	CUTALP	ENSG00000226752.9	12	10	0.8
protein_coding	GPATCH3	ENSG00000198746.12	12	10	0.8
protein_coding	ACSF2	ENSG00000167107.12	12	10	0.8
protein_coding	HGH1	ENSG00000235173.6	12	10	0.8
protein_coding	SLC7A6	ENSG00000103064.14	18	15	0.8
sense_intronic	AL604028.1	ENSG00000230896.1	18	15	0.8
protein_coding	ABCA2	ENSG00000107331.16	18	15	0.8
protein_coding	PPP1R35	ENSG00000160813.6	24	20	0.8
protein_coding	ZNF160	ENSG00000170949.17	24	20	0.8
protein_coding	EIF4EBP1	ENSG00000187840.4	24	20	0.8
protein_coding	ZSCAN30	ENSG00000186814.13	24	20	0.8
protein_coding	GDF15	ENSG00000130513.6	30	25	0.8
protein_coding	SRFBP1	ENSG00000151304.5	30	25	0.8
protein_coding	TOR4A	ENSG00000198113.2	36	30	0.8
protein_coding	SGMS1	ENSG00000198964.13	36	30	0.8
protein_coding	C1QBP	ENSG00000108561.8	36	30	0.8
protein_coding	KYAT3	ENSG00000137944.17	36	30	0.8
protein_coding	ZADH2	ENSG00000180011.6	36	30	0.8
protein_coding	UBA7	ENSG00000182179.12	36	30	0.8

protein_coding	KDELC1	ENSG00000134901.12	36	30	0.8
protein_coding	WDR54	ENSG00000005448.16	42	35	0.8
protein_coding	HYI	ENSG00000178922.16	48	40	0.8
protein_coding	COX11	ENSG00000166260.11	48	40	0.8
protein_coding	BOLA3	ENSG00000163170.11	60	50	0.8
protein_coding	THOC5	ENSG00000100296.13	60	50	0.8
protein_coding	CNOT11	ENSG00000158435.7	66	55	0.8
protein_coding	VAMP4	ENSG00000117533.14	78	65	0.8
protein_coding	GNB5	ENSG00000069966.18	78	65	0.8
protein_coding	SESN1	ENSG00000080546.13	84	70	0.8
protein_coding	SLC22A17	ENSG00000092096.16	84	70	0.8
protein_coding	TOMM34	ENSG00000025772.7	96	80	0.8
protein_coding	APRT	ENSG00000198931.10	114	95	0.8
protein_coding	SRSF6	ENSG00000124193.14	126	105	0.8
protein_coding	TRIO	ENSG00000038382.19	144	120	0.8
protein_coding	UBXN1	ENSG00000162191.13	162	135	0.8
protein_coding	SEC24D	ENSG00000150961.14	192	160	0.8
protein_coding	HLA-C	ENSG00000204525.16	300	250	0.8
protein_coding	SPG21	ENSG00000090487.10	318	265	0.8
protein_coding	S100A6	ENSG00000197956.9	12736	10609	0.8
protein_coding	C8orf59	ENSG00000176731.11	185	154	0.8
protein_coding	EPN1	ENSG00000063245.14	161	134	0.8
protein_coding	RBFOX2	ENSG00000100320.22	632	526	0.8
protein_coding	PSD3	ENSG00000156011.16	435	362	0.8
protein_coding	MRPS34	ENSG00000074071.14	119	99	0.8
protein_coding	CRBN	ENSG00000113851.14	107	89	0.8
protein_coding	APH1B	ENSG00000138613.13	107	89	0.8
protein_coding	LSM¬†4	ENSG00000130520.10	107	89	0.8
protein_coding	ACAA1	ENSG00000060971.17	95	79	0.8
protein_coding	SNRNP200	ENSG00000144028.14	172	143	0.8
protein_coding	PDLIM1	ENSG00000107438.8	504	419	0.8
protein_coding	GOSR2	ENSG00000108433.16	71	59	0.8
protein_coding	KBTBD7	ENSG00000120696.8	71	59	0.8
protein_coding	RACK1	ENSG00000204628.11	1384	1150	0.8
protein_coding	SP100	ENSG00000067066.16	396	329	0.8
protein_coding	ATP10D	ENSG00000145246.13	65	54	0.8
protein_coding	FBXO3	ENSG00000110429.13	65	54	0.8
protein_coding	ARL5B	ENSG00000165997.4	59	49	0.8
protein_coding	C7orf50	ENSG00000146540.14	59	49	0.8
antisense	SNHG7	ENSG00000233016.6	171	142	0.8
protein_coding	SND1	ENSG00000197157.10	459	381	0.8
protein_coding	WDR82	ENSG00000164091.11	253	210	0.8
protein_coding	COX7A2	ENSG00000112695.11	335	278	0.8
protein_coding	STK3	ENSG00000104375.16	47	39	0.8

protein_coding	NDUFA3	ENSG00000170906.15	94	78	0.8
protein_coding	SMAP1	ENSG00000112305.14	235	195	0.8
protein_coding	SHC1	ENSG00000160691.18	370	307	0.8
protein_coding	RBMS2	ENSG00000076067.12	135	112	0.8
protein_coding	WASF2	ENSG00000158195.10	457	379	0.8
protein_coding	ACAD9	ENSG00000177646.18	41	34	0.8
protein_coding	C1QTNF1	ENSG00000173918.14	82	68	0.8
protein_coding	KLHDC10	ENSG00000128607.13	82	68	0.8
protein_coding	DPM3	ENSG00000179085.7	82	68	0.8
protein_coding	PWP1	ENSG00000136045.11	269	223	0.8
protein_coding	FNTB	ENSG00000257365.7	35	29	0.8
protein_coding	NDUFAF7	ENSG00000003509.15	35	29	0.8
protein_coding	TMUB1	ENSG00000164897.12	35	29	0.8
protein_coding	SPRTN	ENSG00000010072.15	35	29	0.8
protein_coding	PAR	ENSG00000178605.13	35	29	0.8
protein_coding	TAPBPL	ENSG00000139192.11	35	29	0.8
protein_coding	METTL25	ENSG00000127720.7	35	29	0.8
protein_coding	NUDCD3	ENSG00000015676.17	70	58	0.8
protein_coding	TBC1D14	ENSG00000132405.18	70	58	0.8
protein_coding	MRPS9	ENSG00000135972.8	70	58	0.8
protein_coding	GSK3B	ENSG00000082701.15	134	111	0.8
protein_coding	PPP1R15A	ENSG00000087074.7	64	53	0.8
protein_coding	RUVBL1	ENSG00000175792.11	64	53	0.8
protein_coding	SLC25A3	ENSG00000075415.12	1244	1030	0.8
protein_coding	PLAC9	ENSG00000189129.13	29	24	0.8
protein_coding	LETMD1	ENSG00000050426.15	58	48	0.8
protein_coding	AK6	ENSG00000085231.13	87	72	0.8
protein_coding	PRDX1	ENSG00000117450.13	1795	1485	0.8
protein_coding	RHOA	ENSG00000067560.10	1632	1350	0.8
protein_coding	MCU	ENSG00000156026.14	81	67	0.8
protein_coding	SMIM29	ENSG00000186577.13	52	43	0.8
protein_coding	BROX	ENSG00000162819.11	202	167	0.8
protein_coding	PPM1G	ENSG00000115241.10	75	62	0.8
protein_coding	RNASET2	ENSG00000026297.15	98	81	0.8
protein_coding	DCAF11	ENSG00000100897.17	121	100	0.8
protein_coding	RPS3	ENSG00000149273.14	1484	1226	0.8
protein_coding	TTC7A	ENSG00000068724.15	23	19	0.8
protein_coding	CC2D1A	ENSG00000132024.17	23	19	0.8
protein_coding	SKP2	ENSG00000145604.15	46	38	0.8
protein_coding	SYPL1	ENSG00000008282.8	230	190	0.8
protein_coding	HLA-B	ENSG00000234745.10	774	639	0.8
protein_coding	TBCB	ENSG00000105254.11	315	260	0.8
protein_coding	SSR4	ENSG00000180879.13	292	241	0.8
protein_coding	DCTN2	ENSG00000175203.15	246	203	0.8

protein_coding	MTA2	ENSG00000149480.6	80	66	0.8
protein_coding	KLHL5	ENSG00000109790.16	120	99	0.8
protein_coding	ASAP2	ENSG00000151693.10	97	80	0.8
protein_coding	HIST1H2BD	ENSG00000158373.8	57	47	0.8
protein_coding	ERAP1	ENSG00000164307.12	57	47	0.8
protein_coding	GSTA4	ENSG00000170899.10	57	47	0.8
protein_coding	DEXI	ENSG00000182108.10	171	141	0.8
protein_coding	PXDC1	ENSG00000168994.13	131	108	0.8
protein_coding	COMMD6	ENSG00000188243.12	524	432	0.8
protein_coding	AQR	ENSG00000021776.10	74	61	0.8
protein_coding	GDF11	ENSG00000135414.9	74	61	0.8
protein_coding	GID8	ENSG00000101193.7	148	122	0.8
protein_coding	STIM1	ENSG00000167323.11	165	136	0.8
protein_coding	NAA10	ENSG00000102030.15	91	75	0.8
protein_coding	NBDY	ENSG00000204272.12	273	225	0.8
protein_coding	PPP4R1	ENSG00000154845.15	125	103	0.8
protein_coding	ETFB	ENSG00000105379.9	193	159	0.8
protein_coding	TXNRD1	ENSG00000198431.15	726	598	0.8
protein_coding	YRDC	ENSG00000196449.3	17	14	0.8
protein_coding	ZBTB14	ENSG00000198081.10	17	14	0.8
protein_coding	ZNF780A	ENSG00000197782.14	17	14	0.8
protein_coding	TAB3	ENSG00000157625.15	17	14	0.8
protein_coding	TOR3A	ENSG00000186283.13	17	14	0.8
protein_coding	KANK1	ENSG00000107104.18	17	14	0.8
protein_coding	DCHS1	ENSG00000166341.7	17	14	0.8
protein_coding	NFATC3	ENSG00000072736.18	34	28	0.8
protein_coding	THAP3	ENSG00000041988.15	34	28	0.8
protein_coding	RBM7	ENSG00000076053.10	51	42	0.8
protein_coding	PHF1	ENSG00000112511.17	51	42	0.8
protein_coding	GNPDA2	ENSG00000163281.11	51	42	0.8
protein_coding	BTF3L4	ENSG00000134717.17	85	70	0.8
protein_coding	LSM14A	ENSG00000257103.8	181	149	0.8
protein_coding	CHD4	ENSG00000111642.15	181	149	0.8
protein_coding	ADH5	ENSG00000197894.10	582	479	0.8
protein_coding	TIAL1	ENSG00000151923.17	209	172	0.8
protein_coding	KDELR2	ENSG00000136240.9	836	688	0.8
protein_coding	SCAMP3	ENSG00000116521.10	96	79	0.8
protein_coding	UTP11	ENSG00000183520.11	79	65	0.8
protein_coding	MPV17	ENSG00000115204.14	141	116	0.8
protein_coding	PCNA	ENSG00000132646.10	203	167	0.8
protein_coding	ANXA6	ENSG00000197043.13	524	431	0.8
protein_coding	MALSU1	ENSG00000156928.4	107	88	0.8
protein_coding	THAP4	ENSG00000176946.11	45	37	0.8
protein_coding	COX18	ENSG00000163626.16	45	37	0.8

protein_coding	TNKS	ENSG00000173273.15	118	97	0.8
protein_coding	SLC30A7	ENSG00000162695.11	174	143	0.8
protein_coding	FKBP10	ENSG00000141756.18	409	336	0.8
protein_coding	SSX2IP	ENSG00000117155.16	28	23	0.8
protein_coding	NR2C1	ENSG00000120798.16	56	46	0.8
protein_coding	PLCD3	ENSG00000161714.11	95	78	0.8
protein_coding	CCDC115	ENSG00000136710.9	95	78	0.8
protein_coding	TMEM258	ENSG00000134825.15	469	385	0.8
protein_coding	TMEM243	ENSG00000135185.11	39	32	0.8
protein_coding	KIRREL1	ENSG00000183853.17	39	32	0.8
protein_coding	DNMBP	ENSG00000107554.16	39	32	0.8
lincRNA	SNHG9	ENSG00000255198.4	39	32	0.8
protein_coding	PLEKHG4	ENSG00000196155.12	39	32	0.8
protein_coding	ERCC1	ENSG00000012061.15	78	64	0.8
protein_coding	STX5	ENSG00000162236.11	50	41	0.8
protein_coding	EVI5L	ENSG00000142459.8	50	41	0.8
protein_coding	BAD	ENSG00000002330.13	100	82	0.8
protein_coding	MAP2K4	ENSG00000065559.14	61	50	0.8
protein_coding	EPS8L2	ENSG00000177106.15	61	50	0.8
protein_coding	SNX19	ENSG00000120451.10	133	109	0.8
protein_coding	KLF12	ENSG00000118922.17	72	59	0.8
protein_coding	TBC1D17	ENSG00000104946.12	72	59	0.8
protein_coding	ZDHHC24	ENSG00000174165.7	72	59	0.8
protein_coding	PTPRK	ENSG00000152894.14	282	231	0.8
protein_coding	FAM50A	ENSG00000071859.14	105	86	0.8
protein_coding	BMI1	ENSG00000168283.13	105	86	0.8
protein_coding	MID1IP1	ENSG00000165175.15	105	86	0.8
protein_coding	CYBRD1	ENSG00000071967.11	849	695	0.8
protein_coding	QSOX1	ENSG00000116260.16	1967	1610	0.8
protein_coding	BRK1	ENSG00000254999.3	413	338	0.8
protein_coding	HYLS1	ENSG00000198331.10	11	9	0.8
protein_coding	ZNF585B	ENSG00000245680.9	11	9	0.8
protein_coding	ZNF28	ENSG00000198538.10	11	9	0.8
protein_coding	XKR6	ENSG00000171044.10	11	9	0.8
protein_coding	ZNF674	ENSG00000251192.7	11	9	0.8
protein_coding	CPNE7	ENSG00000178773.14	11	9	0.8
protein_coding	CYB5RL	ENSG00000215883.10	11	9	0.8
protein_coding	MRPS17	ENSG00000239789.5	11	9	0.8
antisense	AC002550.2	ENSG00000276571.1	11	9	0.8
protein_coding	MPHOSPH9	ENSG00000051825.14	22	18	0.8
protein_coding	CEP135	ENSG00000174799.10	22	18	0.8
protein_coding	CARMIL1	ENSG00000079691.17	22	18	0.8
protein_coding	ABHD14A	ENSG00000248487.8	22	18	0.8
protein_coding	RAB11FIP1	ENSG00000156675.15	33	27	0.8

protein_coding	TRIM41	ENSG00000146063.19	33	27	0.8
protein_coding	PDP2	ENSG00000172840.6	44	36	0.8
protein_coding	SMIM27	ENSG00000235453.10	44	36	0.8
protein_coding	ANKDD1A	ENSG00000166839.16	44	36	0.8
protein_coding	ULK2	ENSG00000083290.19	44	36	0.8
protein_coding	USP39	ENSG00000168883.19	55	45	0.8
protein_coding	UBR7	ENSG00000012963.14	55	45	0.8
protein_coding	FDXR	ENSG00000161513.11	55	45	0.8
protein_coding	AMDHD2	ENSG00000162066.15	66	54	0.8
protein_coding	HDGFL2	ENSG00000167674.14	88	72	0.8
protein_coding	FCGRT	ENSG00000104870.12	110	90	0.8
protein_coding	MRPL3	ENSG00000114686.8	110	90	0.8
protein_coding	IGFBP3	ENSG00000146674.14	132	108	0.8
protein_coding	LSM¬†8	ENSG00000128534.7	104	85	0.8
protein_coding	GTF2IRD1	ENSG00000006704.10	60	49	0.8
protein_coding	MAP2K2	ENSG00000126934.13	169	138	0.8
protein_coding	PDE4DIP	ENSG00000178104.19	98	80	0.8
protein_coding	LRPPRC	ENSG00000138095.18	147	120	0.8
protein_coding	EXD2	ENSG00000081177.18	38	31	0.8
protein_coding	ACOT8	ENSG00000101473.16	38	31	0.8
protein_coding	MLLT1	ENSG00000130382.8	114	93	0.8
protein_coding	LACTB	ENSG00000103642.11	65	53	0.8
protein_coding	POLR2J	ENSG00000005075.15	130	106	0.8
protein_coding	GRK2	ENSG00000173020.10	27	22	0.8
processed_transcript	AC016747.1	ENSG00000212978.6	27	22	0.8
protein_coding	RCBTB2	ENSG00000136161.12	27	22	0.8
transcribed_unitary_pseudogene	CRYZL2P	ENSG00000242193.11	27	22	0.8
protein_coding	HIVEP2	ENSG00000010818.9	54	44	0.8
protein_coding	VPS36	ENSG00000136100.13	54	44	0.8
protein_coding	PATL1	ENSG00000166889.13	54	44	0.8
protein_coding	COG5	ENSG00000164597.13	54	44	0.8
protein_coding	HSPB1	ENSG00000106211.8	1803	1469	0.8
protein_coding	ANXA5	ENSG00000164111.14	2445	1992	0.8
protein_coding	TMSB4X	ENSG00000205542.10	6771	5515	0.8
protein_coding	TXNDC17	ENSG00000129235.10	199	162	0.8
protein_coding	CRTAP	ENSG00000170275.14	586	477	0.8
protein_coding	PWWP2B	ENSG00000171813.13	43	35	0.8
protein_coding	RLIM	ENSG00000131263.12	86	70	0.8
protein_coding	IFNGR2	ENSG00000159128.14	161	131	0.8
protein_coding	MPC1	ENSG00000060762.18	59	48	0.8
protein_coding	HINT3	ENSG00000111911.6	59	48	0.8
protein_coding	UQCR10	ENSG00000184076.13	193	157	0.8

protein_coding	GALNT11	ENSG00000178234.12	75	61	0.8
protein_coding	GTF3C6	ENSG00000155115.6	225	183	0.8
protein_coding	PARK7	ENSG00000116288.12	519	422	0.8
protein_coding	TRIM28	ENSG00000130726.11	123	100	0.8
protein_coding	BTF3	ENSG00000145741.15	732	595	0.8
protein_coding	FBXO45	ENSG00000174013.7	16	13	0.8
protein_coding	ZNF438	ENSG00000183621.15	16	13	0.8
processed_pseudogene	RPS13P2	ENSG00000228929.1	16	13	0.8
protein_coding	LRSAM1	ENSG00000148356.13	16	13	0.8
protein_coding	THEM4	ENSG00000159445.12	16	13	0.8
protein_coding	ZNF446	ENSG00000083838.15	16	13	0.8
protein_coding	TMEM136	ENSG00000181264.8	32	26	0.8
protein_coding	EIF1AD	ENSG00000175376.8	32	26	0.8
protein_coding	MYO19	ENSG00000278259.4	32	26	0.8
protein_coding	RAD51C	ENSG00000108384.14	32	26	0.8
protein_coding	ZZEF1	ENSG00000074755.14	32	26	0.8
protein_coding	MEGF8	ENSG00000105429.12	48	39	0.8
protein_coding	DAAM1	ENSG00000100592.15	64	52	0.8
protein_coding	NR2F6	ENSG00000160113.5	80	65	0.8
protein_coding	MED15	ENSG00000099917.17	80	65	0.8
protein_coding	ASB8	ENSG00000177981.10	80	65	0.8
protein_coding	ATP5PB	ENSG00000116459.10	282	229	0.8
protein_coding	BRI3	ENSG00000164713.9	1048	851	0.8
protein_coding	IARS	ENSG00000196305.17	138	112	0.8
protein_coding	CAPN1	ENSG00000014216.15	159	129	0.8
protein_coding	IER5	ENSG00000162783.10	212	172	0.8
protein_coding	SCD5	ENSG00000145284.11	90	73	0.8
protein_coding	FITM2	ENSG00000197296.5	37	30	0.8
protein_coding	SMG5	ENSG00000198952.8	37	30	0.8
protein_coding	MASP1	ENSG00000127241.16	317	257	0.8
transcribed_unprocessed_pseudogene	RP9P	ENSG00000205763.13	58	47	0.8
protein_coding	COX7A2L	ENSG00000115944.14	353	286	0.8
protein_coding	STARD3NL	ENSG00000010270.13	79	64	0.8
protein_coding	EWSR1	ENSG00000182944.17	158	128	0.8
protein_coding	TMEM109	ENSG00000110108.9	358	290	0.8
protein_coding	PCGF2	ENSG00000277258.4	121	98	0.8
protein_coding	COX14	ENSG00000178449.8	163	132	0.8
protein_coding	MZT2B	ENSG00000152082.13	373	302	0.8
lincRNA	LINC00324	ENSG00000178977.3	21	17	0.8
protein_coding	ZNF777	ENSG00000196453.7	21	17	0.8
protein_coding	ZNF181	ENSG00000197841.14	21	17	0.8
protein_coding	ZNF70	ENSG00000187792.4	21	17	0.8

protein_coding	MTO1	ENSG00000135297.15	21	17	0.8
protein_coding	D2HGDH	ENSG00000180902.17	42	34	0.8
protein_coding	TAB1	ENSG00000100324.13	42	34	0.8
protein_coding	MDK	ENSG00000110492.15	42	34	0.8
protein_coding	CDC42SE2	ENSG00000158985.13	63	51	0.8
protein_coding	CENPC	ENSG00000145241.10	63	51	0.8
protein_coding	COL7A1	ENSG00000114270.17	126	102	0.8
protein_coding	FAT1	ENSG00000083857.13	367	297	0.8
protein_coding	FHL2	ENSG00000115641.18	367	297	0.8
protein_coding	WDR45	ENSG00000196998.17	68	55	0.8
protein_coding	GOT1	ENSG00000120053.11	68	55	0.8
protein_coding	SART3	ENSG00000075856.11	47	38	0.8
protein_coding	MED14	ENSG00000180182.10	47	38	0.8
protein_coding	UBP1	ENSG00000153560.11	141	114	0.8
protein_coding	PHPT1	ENSG00000054148.17	120	97	0.8
protein_coding	PPP1R7	ENSG00000115685.14	99	80	0.8
protein_coding	PGAM1	ENSG00000171314.8	26	21	0.8
protein_coding	HAS2	ENSG00000170961.6	26	21	0.8
protein_coding	MCPH1	ENSG00000147316.12	26	21	0.8
protein_coding	MARCH9	ENSG00000139266.5	26	21	0.8
protein_coding	CERS5	ENSG00000139624.12	52	42	0.8
protein_coding	FNDC4	ENSG00000115226.9	52	42	0.8
protein_coding	NATD1	ENSG00000274180.1	52	42	0.8
protein_coding	TERF2	ENSG00000132604.10	78	63	0.8
protein_coding	UBE2I	ENSG00000103275.19	239	193	0.8
protein_coding	MMS19	ENSG00000155229.20	161	130	0.8
protein_coding	EIF2S3	ENSG00000130741.10	192	155	0.8
protein_coding	PIP4P2	ENSG00000155099.7	83	67	0.8
protein_coding	CHCHD1	ENSG00000172586.7	114	92	0.8
protein_coding	CTBP1	ENSG00000159692.15	404	326	0.8
protein_coding	TNS1	ENSG00000079308.18	119	96	0.8
protein_coding	OPA3	ENSG00000125741.4	31	25	0.8
protein_coding	ZNF703	ENSG00000183779.6	62	50	0.8
protein_coding	METTL14	ENSG00000145388.14	62	50	0.8
protein_coding	QKI	ENSG00000112531.16	403	325	0.8
protein_coding	ABI3BP	ENSG00000154175.16	315	254	0.8
protein_coding	BCL2L1	ENSG00000171552.12	67	54	0.8
protein_coding	EXOC4	ENSG00000131558.14	67	54	0.8
protein_coding	COX4I1	ENSG00000131143.8	1139	918	0.8
protein_coding	TMEM250	ENSG00000238227.7	103	83	0.8
protein_coding	C1S	ENSG00000182326.14	633	510	0.8
protein_coding	ATP9B	ENSG00000166377.20	36	29	0.8
protein_coding	KCTD18	ENSG00000155729.12	36	29	0.8
protein_coding	CNOT8	ENSG00000155508.13	108	87	0.8

protein_coding	S100A16	ENSG00000188643.10	180	145	0.8
protein_coding	NENF	ENSG00000117691.9	159	128	0.8
protein_coding	CNDP2	ENSG00000133313.14	41	33	0.8
protein_coding	RBFA	ENSG00000101546.12	41	33	0.8
protein_coding	GTF3C4	ENSG00000125484.11	87	70	0.8
protein_coding	RAC2	ENSG00000128340.14	46	37	0.8
protein_coding	MAPKAPK3	ENSG00000114738.10	46	37	0.8
protein_coding	MRPS26	ENSG00000125901.5	46	37	0.8
protein_coding	OST4	ENSG00000228474.5	695	559	0.8
protein_coding	UBE3A	ENSG00000114062.19	286	230	0.8
protein_coding	UQCRH	ENSG00000173660.11	454	365	0.8
protein_coding	ETFDH	ENSG00000171503.11	56	45	0.8
protein_coding	RFNG	ENSG00000169733.11	56	45	0.8
protein_coding	TMEM256	ENSG00000205544.3	61	49	0.8
protein_coding	ESD	ENSG00000139684.13	432	347	0.8
protein_coding	HMGA1	ENSG00000137309.19	386	310	0.8
protein_coding	RTRAF	ENSG00000087302.8	259	208	0.8
protein_coding	ETFRF1	ENSG00000205707.10	66	53	0.8
protein_coding	USP24	ENSG00000162402.13	71	57	0.8
protein_coding	GTF2A2	ENSG00000140307.10	157	126	0.8
protein_coding	XPNPEP1	ENSG00000108039.17	157	126	0.8
protein_coding	SIL1	ENSG00000120725.12	81	65	0.8
protein_coding	PARP1	ENSG00000143799.12	91	73	0.8
protein_coding	MRPL37	ENSG00000116221.15	96	77	0.8
protein_coding	RPS18	ENSG00000231500.6	1579	1266	0.8
protein_coding	DYNLL2	ENSG00000264364.2	126	101	0.8
protein_coding	NDUFB11	ENSG00000147123.10	136	109	0.8
protein_coding	NDUFC2	ENSG00000151366.12	251	201	0.8
protein_coding	CEP83	ENSG00000173588.14	5	4	0.8
protein_coding	PMM2	ENSG00000140650.11	5	4	0.8
protein_coding	SELENOH	ENSG00000211450.9	5	4	0.8
protein_coding	ST3GAL3	ENSG00000126091.20	5	4	0.8
protein_coding	SLC19A1	ENSG00000173638.18	5	4	0.8
protein_coding	ZNF205	ENSG00000122386.10	5	4	0.8
protein_coding	TMEM116	ENSG00000198270.12	5	4	0.8
antisense	AC114271.1	ENSG00000274425.1	5	4	0.8
protein_coding	F8A1	ENSG00000277203.1	5	4	0.8
protein_coding	ZNF888	ENSG00000213793.5	5	4	0.8
antisense	AL691432.2	ENSG00000272106.1	5	4	0.8
antisense	NDUFV2-	ENSG00000266053.2	5	4	0.8
protein_coding	ZNF501	ENSG00000186446.11	5	4	0.8
protein_coding	SMYD5	ENSG00000135632.11	5	4	0.8
protein_coding	C3orf33	ENSG00000174928.15	5	4	0.8
protein_coding	OSBPL7	ENSG00000006025.11	5	4	0.8

protein_coding	CPEB1	ENSG00000214575.9	5	4	0.8
protein_coding	BEND3	ENSG00000178409.13	5	4	0.8
protein_coding	ZNF93	ENSG00000184635.15	5	4	0.8
protein_coding	TCEANC	ENSG00000176896.8	5	4	0.8
lincRNA	OSER1-DT	ENSG00000223891.5	5	4	0.8
protein_coding	CCDC163	ENSG00000280670.2	5	4	0.8
protein_coding	ARHGAP19	ENSG00000213390.10	5	4	0.8
protein_coding	PRAG1	ENSG00000275342.4	5	4	0.8
antisense	AC005070.3	ENSG00000272918.1	5	4	0.8
transcribed_unprocessed_pseudogene	ADCY10P1	ENSG00000161912.18	5	4	0.8
antisense	AC019205.1	ENSG00000229852.2	5	4	0.8
lincRNA	ZNF674-AS1	ENSG00000230844.2	5	4	0.8
protein_coding	PDK3	ENSG00000067992.13	5	4	0.8
protein_coding	TTLL3	ENSG00000214021.15	5	4	0.8
lincRNA	AC087741.2	ENSG00000275479.1	5	4	0.8
protein_coding	ASTN2	ENSG00000148219.16	5	4	0.8
protein_coding	SPESP1	ENSG00000258484.3	5	4	0.8
processed_pseudogene	RPL37AP1	ENSG00000226243.1	5	4	0.8
protein_coding	OAZ3	ENSG00000143450.16	5	4	0.8
antisense	AC012073.1	ENSG00000271936.1	5	4	0.8
antisense	AC005076.1	ENSG00000224046.1	5	4	0.8
transcribed_unprocessed_pseudogene	CROCCP3	ENSG00000080947.14	5	4	0.8
lincRNA	LINC00663	ENSG00000266904.5	5	4	0.8
transcribed_processed_pseudogene	RPLP1P6	ENSG00000213433.5	5	4	0.8
lincRNA	AC098487.1	ENSG00000248161.5	5	4	0.8
processed_pseudogene	AC090589.1	ENSG00000243802.2	5	4	0.8
protein_coding	COBLL1	ENSG00000082438.15	5	4	0.8
protein_coding	SLC6A16	ENSG00000063127.15	5	4	0.8
protein_coding	TRAPPC3L	ENSG00000173626.9	5	4	0.8
protein_coding	KCNB1	ENSG00000158445.9	5	4	0.8
protein_coding	DPP4	ENSG00000197635.9	10	8	0.8
protein_coding	RASSF5	ENSG00000266094.7	10	8	0.8
protein_coding	ZCCHC4	ENSG00000168228.14	10	8	0.8
protein_coding	ACD	ENSG00000102977.14	10	8	0.8
protein_coding	ZRANB3	ENSG00000121988.17	10	8	0.8
protein_coding	QPCTL	ENSG00000011478.11	10	8	0.8
protein_coding	ZNF628	ENSG00000197483.9	10	8	0.8
protein_coding	PGBD1	ENSG00000137338.5	10	8	0.8
protein_coding	NAA40	ENSG00000110583.12	10	8	0.8
processed_transcript	SLC9A3-AS1	ENSG00000225138.7	10	8	0.8

protein_coding	ADAT2	ENSG00000189007.15	10	8	0.8
protein_coding	RAD51D	ENSG00000185379.20	10	8	0.8
protein_coding	LGALSL	ENSG00000119862.12	10	8	0.8
protein_coding	PNMA6A	ENSG00000235961.5	10	8	0.8
protein_coding	TRAF2	ENSG00000127191.17	10	8	0.8
protein_coding	EPOR	ENSG00000187266.13	15	12	0.8
protein_coding	LENG1	ENSG00000105617.3	15	12	0.8
protein_coding	ARHGEF28	ENSG00000214944.9	15	12	0.8
protein_coding	SMUG1	ENSG00000123415.15	15	12	0.8
protein_coding	HEXDC	ENSG00000169660.16	15	12	0.8
protein_coding	ZBTB5	ENSG00000168795.4	15	12	0.8
protein_coding	CUTC	ENSG00000119929.12	15	12	0.8
processed_transcript	ZNF433-AS1	ENSG00000219665.8	15	12	0.8
protein_coding	MAPK11	ENSG00000185386.14	15	12	0.8
protein_coding	TIGAR	ENSG00000078237.6	15	12	0.8
protein_coding	SLC37A4	ENSG00000137700.18	15	12	0.8
protein_coding	NUDT12	ENSG00000112874.9	15	12	0.8
protein_coding	KIF9	ENSG00000088727.12	20	16	0.8
protein_coding	FIG4	ENSG00000112367.10	20	16	0.8
protein_coding	PHF8	ENSG00000172943.19	20	16	0.8
protein_coding	RRP15	ENSG00000067533.5	25	20	0.8
protein_coding	NGLY1	ENSG00000151092.16	25	20	0.8
protein_coding	ATG16L2	ENSG00000168010.10	25	20	0.8
protein_coding	MANSC1	ENSG00000111261.13	25	20	0.8
protein_coding	C11orf1	ENSG00000137720.7	30	24	0.8
protein_coding	TOMM40L	ENSG00000158882.14	30	24	0.8
protein_coding	BAP1	ENSG00000163930.9	35	28	0.8
protein_coding	SLC6A8	ENSG00000130821.15	35	28	0.8
protein_coding	DLX2	ENSG00000115844.10	35	28	0.8
protein_coding	TRUB1	ENSG00000165832.5	35	28	0.8
transcribed_unitary_pseudogene	LINC02210	ENSG00000204650.14	40	32	0.8
protein_coding	USP6NL	ENSG00000148429.14	40	32	0.8
protein_coding	WRAP73	ENSG00000116213.15	40	32	0.8
protein_coding	PLPBP	ENSG00000147471.11	45	36	0.8
protein_coding	FAM136A	ENSG00000035141.7	50	40	0.8
protein_coding	MED31	ENSG00000108590.10	55	44	0.8
protein_coding	PCCB	ENSG00000114054.13	55	44	0.8
protein_coding	KLF4	ENSG00000136826.14	55	44	0.8
protein_coding	TTC9C	ENSG00000162222.13	60	48	0.8
protein_coding	TSEN15	ENSG00000198860.12	65	52	0.8
protein_coding	ARHGEF10L	ENSG00000074964.16	75	60	0.8
protein_coding	SUGCT	ENSG00000175600.15	75	60	0.8

protein_coding	TSC1	ENSG00000165699.14	80	64	0.8
protein_coding	DDHD2	ENSG00000085788.13	80	64	0.8
protein_coding	TK2	ENSG00000166548.15	90	72	0.8
protein_coding	ABR	ENSG00000159842.15	110	88	0.8
protein_coding	SPIRE1	ENSG00000134278.15	125	100	0.8
protein_coding	SMIM30	ENSG00000214194.8	140	112	0.8
protein_coding	CGGBP1	ENSG00000163320.10	160	128	0.8
protein_coding	FAM102A	ENSG00000167106.11	180	144	0.8
protein_coding	RPSA	ENSG00000168028.13	455	364	0.8
protein_coding	RPS11	ENSG00000142534.6	2190	1752	0.8
protein_coding	EMC10	ENSG00000161671.16	169	135	0.8
protein_coding	CMBL	ENSG00000164237.8	139	111	0.8
protein_coding	C1R	ENSG00000159403.16	258	206	0.8
protein_coding	SPARC	ENSG00000113140.10	9602	7664	0.8
protein_coding	PPP1R18	ENSG00000146112.11	317	253	0.8
protein_coding	RPS27	ENSG00000177954.12	1559	1244	0.8
protein_coding	TEX261	ENSG00000144043.11	173	138	0.8
protein_coding	TBL2	ENSG00000106638.16	79	63	0.8
protein_coding	CTDSP1	ENSG00000144579.7	148	118	0.8
protein_coding	RSU1	ENSG00000148484.17	439	350	0.8
protein_coding	RWDD2B	ENSG00000156253.6	69	55	0.8
protein_coding	UQCRC1	ENSG00000010256.10	123	98	0.8
protein_coding	PGLS	ENSG00000130313.6	59	47	0.8
protein_coding	ZNF131	ENSG00000172262.11	59	47	0.8
protein_coding	CNPPD1	ENSG00000115649.15	59	47	0.8
protein_coding	UBN1	ENSG00000118900.14	231	184	0.8
protein_coding	CPSF6	ENSG00000111605.17	113	90	0.8
protein_coding	DOCK11	ENSG00000147251.15	54	43	0.8
protein_coding	FBXO11	ENSG00000138081.20	162	129	0.8
protein_coding	IFI27L2	ENSG00000119632.3	157	125	0.8
protein_coding	PTCD3	ENSG00000132300.18	103	82	0.8
lincRNA	SNHG25	ENSG00000266402.3	304	242	0.8
protein_coding	DOK4	ENSG00000125170.10	49	39	0.8
protein_coding	PELP1	ENSG00000141456.14	49	39	0.8
protein_coding	CS	ENSG00000062485.18	137	109	0.8
protein_coding	SYNGR1	ENSG00000100321.14	44	35	0.8
protein_coding	AIMP2	ENSG00000106305.9	44	35	0.8
protein_coding	CD81	ENSG00000110651.11	1588	1263	0.8
protein_coding	Sep-11	ENSG00000138758.11	1985	1578	0.8
protein_coding	CRYBG3	ENSG00000080200.9	39	31	0.8
protein_coding	ADAMTS12	ENSG00000151388.10	39	31	0.8
protein_coding	C5orf30	ENSG00000181751.9	39	31	0.8
protein_coding	NUPR1	ENSG00000176046.8	507	403	0.8
protein_coding	BLMH	ENSG00000108578.14	73	58	0.8

protein_coding	RSPRY1	ENSG00000159579.13	107	85	0.8
protein_coding	PPM1M	ENSG00000164088.17	34	27	0.8
protein_coding	RHOBTB1	ENSG00000072422.16	34	27	0.8
protein_coding	ACTR10	ENSG00000131966.13	218	173	0.8
protein_coding	RNPEP	ENSG00000176393.10	150	119	0.8
protein_coding	UBB	ENSG00000170315.13	1737	1378	0.8
protein_coding	PRSS23	ENSG00000150687.11	1645	1305	0.8
protein_coding	PAR	ENSG00000002586.19	1455	1154	0.8
protein_coding	WDR70	ENSG00000082068.8	29	23	0.8
protein_coding	EVC2	ENSG00000173040.12	29	23	0.8
protein_coding	RAB30	ENSG00000137502.9	174	138	0.8
sense_intronic	AC024075.2	ENSG00000269044.2	53	42	0.8
protein_coding	NFS1	ENSG00000244005.12	53	42	0.8
protein_coding	DMAC1	ENSG00000137038.7	77	61	0.8
protein_coding	PTPA	ENSG00000119383.19	77	61	0.8
protein_coding	SH3RF3	ENSG00000172985.10	24	19	0.8
protein_coding	PRKAG2	ENSG00000106617.13	24	19	0.8
protein_coding	PIGH	ENSG00000100564.8	24	19	0.8
protein_coding	BARX1	ENSG00000131668.13	48	38	0.8
protein_coding	TPRA1	ENSG00000163870.15	48	38	0.8
protein_coding	ARL¬†15	ENSG00000185305.10	48	38	0.8
protein_coding	RPL39	ENSG00000198918.7	72	57	0.8
protein_coding	WDR1	ENSG00000071127.16	403	319	0.8
protein_coding	RPA1	ENSG00000132383.11	91	72	0.8
protein_coding	ZFYVE21	ENSG00000100711.13	134	106	0.8
protein_coding	GPANK1	ENSG00000204438.10	43	34	0.8
protein_coding	PRELID1	ENSG00000169230.9	167	132	0.8
protein_coding	CAV2	ENSG00000105971.14	167	132	0.8
protein_coding	OSTF1	ENSG00000134996.11	100	79	0.8
protein_coding	ISG20	ENSG00000172183.14	19	15	0.8
protein_coding	GNG2	ENSG00000186469.8	19	15	0.8
protein_coding	ZFYVE19	ENSG00000166140.17	19	15	0.8
protein_coding	KAT8	ENSG00000103510.19	19	15	0.8
protein_coding	OCEL1	ENSG00000099330.8	19	15	0.8
sense_intronic	AL359091.5	ENSG00000273186.1	19	15	0.8
protein_coding	PLEKHG2	ENSG00000090924.14	57	45	0.8
protein_coding	ZNF521	ENSG00000198795.10	95	75	0.8
protein_coding	EPRS	ENSG00000136628.17	256	202	0.8
protein_coding	FAM57A	ENSG00000167695.14	52	41	0.8
protein_coding	HS3ST3B1	ENSG00000125430.8	52	41	0.8
protein_coding	VAMP3	ENSG00000049245.12	274	216	0.8
protein_coding	MAU2	ENSG00000129933.20	85	67	0.8
protein_coding	OAF	ENSG00000184232.8	118	93	0.8
protein_coding	HIKESHI	ENSG00000149196.15	151	119	0.8

protein_coding	PSEN2	ENSG00000143801.16	33	26	0.8
protein_coding	UXT	ENSG00000126756.11	113	89	0.8
protein_coding	RPS15	ENSG00000115268.9	1162	915	0.8
protein_coding	RNF187	ENSG00000168159.11	301	237	0.8
protein_coding	PSMA2	ENSG00000106588.10	47	37	0.8
protein_coding	TMEM39A	ENSG00000176142.12	61	48	0.8
protein_coding	MRPL35	ENSG00000132313.14	61	48	0.8
protein_coding	VPS28	ENSG00000160948.13	136	107	0.8
protein_coding	WDR61	ENSG00000140395.8	75	59	0.8
protein_coding	RIPOR1	ENSG00000039523.19	103	81	0.8
protein_coding	UBA52	ENSG00000221983.7	1871	1471	0.8
protein_coding	SLC25A5	ENSG00000005022.5	491	386	0.8
protein_coding	LDB1	ENSG00000198728.10	14	11	0.8
protein_coding	DNAJC6	ENSG00000116675.15	14	11	0.8
protein_coding	PCYOX1L	ENSG00000145882.10	14	11	0.8
protein_coding	PPRC1	ENSG00000148840.10	14	11	0.8
protein_coding	NDUFA13	ENSG00000186010.18	14	11	0.8
protein_coding	TMEM41A	ENSG00000163900.10	28	22	0.8
protein_coding	ORAI1	ENSG00000276045.3	28	22	0.8
protein_coding	PUS7L	ENSG00000129317.14	70	55	0.8
protein_coding	PTPN23	ENSG00000076201.14	70	55	0.8
protein_coding	TYMS	ENSG00000176890.15	84	66	0.8
protein_coding	P3H4	ENSG00000141696.12	112	88	0.8
protein_coding	CTDNEP1	ENSG00000175826.11	438	344	0.8
protein_coding	ARMCX1	ENSG00000126947.12	205	161	0.8
protein_coding	CCNYL1	ENSG00000163249.11	51	40	0.8
protein_coding	C1orf52	ENSG00000162642.13	51	40	0.8
protein_coding	ECI1	ENSG00000167969.12	51	40	0.8
protein_coding	KHSRP	ENSG00000088247.17	514	403	0.8
protein_coding	EIF6	ENSG00000242372.7	134	105	0.8
protein_coding	THBS1	ENSG00000137801.10	4717	3696	0.8
protein_coding	SRSF2	ENSG00000161547.16	97	76	0.8
protein_coding	RASSF3	ENSG00000153179.12	97	76	0.8
protein_coding	KANSL1	ENSG00000120071.14	97	76	0.8
protein_coding	ZFAND3	ENSG00000156639.11	346	271	0.8
protein_coding	HSPA8	ENSG00000109971.13	378	296	0.8
protein_coding	ZNF507	ENSG00000168813.16	23	18	0.8
protein_coding	PAAF1	ENSG00000175575.12	23	18	0.8
protein_coding	ASH2L	ENSG00000129691.15	46	36	0.8
protein_coding	SUFU	ENSG00000107882.11	46	36	0.8
lincRNA	LINC01184	ENSG00000245937.7	46	36	0.8
protein_coding	RANGRF	ENSG00000108961.13	69	54	0.8
protein_coding	ITGA11	ENSG00000137809.16	179	140	0.8
protein_coding	IQSEC1	ENSG00000144711.15	78	61	0.8

protein_coding	DROSHA	ENSG00000113360.16	78	61	0.8
protein_coding	MME	ENSG00000196549.10	366	286	0.8
protein_coding	IP6K2	ENSG00000068745.14	215	168	0.8
protein_coding	PGAM5	ENSG00000247077.6	32	25	0.8
protein_coding	IER5L	ENSG00000188483.7	32	25	0.8
protein_coding	FAM168B	ENSG00000152102.17	242	189	0.8
protein_coding	MRPL18	ENSG00000112110.9	105	82	0.8
protein_coding	TSC22D2	ENSG00000196428.12	73	57	0.8
protein_coding	AP2M1	ENSG00000161203.13	579	452	0.8
protein_coding	C3orf62	ENSG00000188315.7	41	32	0.8
protein_coding	ARAP1	ENSG00000186635.14	91	71	0.8
protein_coding	ANXA7	ENSG00000138279.15	291	227	0.8
protein_coding	PEF1	ENSG00000162517.12	100	78	0.8
protein_coding	COPZ2	ENSG00000005243.9	100	78	0.8
protein_coding	TCF3	ENSG00000071564.14	168	131	0.8
protein_coding	EIF1	ENSG00000173812.10	2383	1858	0.8
protein_coding	RPS2	ENSG00000140988.15	1565	1220	0.8
protein_coding	MTMR3	ENSG00000100330.15	77	60	0.8
protein_coding	SRGAP2	ENSG00000266028.7	86	67	0.8
protein_coding	PPP1R14B	ENSG00000173457.10	95	74	0.8
protein_coding	NIT2	ENSG00000114021.11	95	74	0.8
protein_coding	POLR2L	ENSG00000177700.5	873	680	0.8
protein_coding	SPATA33	ENSG00000167523.13	9	7	0.8
protein_coding	TSEN54	ENSG00000182173.12	9	7	0.8
protein_coding	ABCB9	ENSG00000150967.17	9	7	0.8
protein_coding	PGGHG	ENSG00000142102.15	9	7	0.8
protein_coding	LRRC73	ENSG00000204052.4	9	7	0.8
lincRNA	AC147067.1	ENSG00000244459.2	9	7	0.8
protein_coding	KCTD6	ENSG00000168301.12	9	7	0.8
protein_coding	RBKS	ENSG00000171174.14	9	7	0.8
antisense	AL135905.1	ENSG00000266680.1	9	7	0.8
protein_coding	NEK8	ENSG00000160602.13	9	7	0.8
antisense	ZNF213-AS1	ENSG00000263072.6	9	7	0.8
protein_coding	ANKRD6	ENSG00000135299.16	9	7	0.8
protein_coding	BAX	ENSG00000087088.19	9	7	0.8
protein_coding	PRELID3A	ENSG00000141391.13	9	7	0.8
protein_coding	C2orf81	ENSG00000284308.1	9	7	0.8
protein_coding	EARS2	ENSG00000103356.15	18	14	0.8
protein_coding	N4BP2L1	ENSG00000139597.17	18	14	0.8
protein_coding	TRIM39	ENSG00000204599.14	18	14	0.8
protein_coding	HEATR6	ENSG00000068097.14	18	14	0.8
protein_coding	CTF1	ENSG00000150281.6	18	14	0.8
protein_coding	BTBD19	ENSG00000222009.8	18	14	0.8
protein_coding	DEPTOR	ENSG00000155792.9	18	14	0.8

protein_coding	SIRT3	ENSG00000142082.14	27	21	0.8
protein_coding	RNLS	ENSG00000184719.11	27	21	0.8
protein_coding	GOLGA1	ENSG00000136935.13	27	21	0.8
protein_coding	ACADSB	ENSG00000196177.12	45	35	0.8
protein_coding	C19orf66	ENSG00000130813.17	54	42	0.8
protein_coding	VARS	ENSG00000204394.12	54	42	0.8
protein_coding	FAM162A	ENSG00000114023.15	63	49	0.8
protein_coding	FTSJ1	ENSG00000068438.14	63	49	0.8
protein_coding	IFITM3	ENSG00000142089.15	2066	1606	0.8
protein_coding	VPS29	ENSG00000111237.18	238	185	0.8
protein_coding	TJP1	ENSG00000104067.16	175	136	0.8
protein_coding	BGN	ENSG00000182492.15	260	202	0.8
protein_coding	YTHDF1	ENSG00000149658.17	94	73	0.8
protein_coding	RPS5	ENSG00000083845.8	1068	829	0.8
protein_coding	EIF4B	ENSG00000063046.17	210	163	0.8
protein_coding	KAT5	ENSG00000172977.12	58	45	0.8
protein_coding	CERS6	ENSG00000172292.14	174	135	0.8
protein_coding	UBC	ENSG00000150991.14	1842	1429	0.8
TEC	HEIH	ENSG00000278970.1	49	38	0.8
protein_coding	CUEDC1	ENSG00000180891.12	98	76	0.8
protein_coding	TSPAN5	ENSG00000168785.7	355	275	0.8
protein_coding	EIF4A2	ENSG00000156976.16	528	409	0.8
protein_coding	PSMB5	ENSG00000100804.18	448	347	0.8
protein_coding	GNS	ENSG00000135677.10	439	340	0.8
protein_coding	TSEN34	ENSG00000170892.10	133	103	0.8
protein_coding	AXL	ENSG00000167601.11	461	357	0.8
protein_coding	CTSD	ENSG00000117984.13	656	508	0.8
protein_coding	COX6B1	ENSG00000126267.9	301	233	0.8
protein_coding	ERLIN1	ENSG00000107566.13	146	113	0.8
protein_coding	EIF3B	ENSG00000106263.17	115	89	0.8
protein_coding	CYTH2	ENSG00000105443.14	115	89	0.8
protein_coding	TSTD2	ENSG00000136925.14	53	41	0.8
protein_coding	COMMD7	ENSG00000149600.11	128	99	0.8
protein_coding	MCM3AP	ENSG00000160294.10	75	58	0.8
antisense	AL592148.3	ENSG00000272750.1	22	17	0.8
protein_coding	SPEF2	ENSG00000152582.13	22	17	0.8
protein_coding	NLRX1	ENSG00000160703.15	22	17	0.8
protein_coding	GUCY1B1	ENSG00000061918.12	22	17	0.8
sense_intronic	AL049840.4	ENSG00000269958.1	44	34	0.8
protein_coding	MRVI1	ENSG00000072952.18	44	34	0.8
protein_coding	ARMC10	ENSG00000170632.13	66	51	0.8
protein_coding	TSC22D4	ENSG00000166925.8	88	68	0.8
protein_coding	NDUFAF3	ENSG00000178057.14	136	105	0.8
protein_coding	SNRPD3	ENSG00000100028.11	149	115	0.8

protein_coding	SCN9A	ENSG00000169432.15	302	233	0.8
protein_coding	S100A11	ENSG00000163191.5	1172	904	0.8
protein_coding	SAT2	ENSG00000141504.11	131	101	0.8
protein_coding	MTG2	ENSG00000101181.17	48	37	0.8
protein_coding	LAMC1	ENSG00000135862.5	951	733	0.8
protein_coding	CUEDC2	ENSG00000107874.10	61	47	0.8
protein_coding	NAE1	ENSG00000159593.14	135	104	0.8
protein_coding	RCC1L	ENSG00000274523.4	87	67	0.8
protein_coding	IRAK1	ENSG00000184216.13	100	77	0.8
protein_coding	ABHD4	ENSG00000100439.10	100	77	0.8
protein_coding	ATP6V1H	ENSG00000047249.17	152	117	0.8
protein_coding	LASP1	ENSG00000002834.17	1124	865	0.8
protein_coding	ITGA10	ENSG00000143127.12	13	10	0.8
protein_coding	WDR24	ENSG00000127580.16	13	10	0.8
protein_coding	DDX28	ENSG00000182810.6	13	10	0.8
protein_coding	USPL1	ENSG00000132952.11	13	10	0.8
protein_coding	SLC12A7	ENSG00000113504.20	13	10	0.8
protein_coding	GCDH	ENSG00000105607.12	13	10	0.8
protein_coding	L2HGDH	ENSG00000087299.11	13	10	0.8
protein_coding	EFCAB2	ENSG00000203666.12	26	20	0.8
protein_coding	OSBPL6	ENSG00000079156.16	26	20	0.8
protein_coding	CFAP298	ENSG00000159079.18	39	30	0.8
protein_coding	PHF13	ENSG00000116273.5	65	50	0.8
protein_coding	STK17A	ENSG00000164543.6	78	60	0.8
protein_coding	LAMTOR3	ENSG00000109270.12	78	60	0.8
protein_coding	SECTM1	ENSG00000141574.7	91	70	0.8
protein_coding	TACC1	ENSG00000147526.19	403	310	0.8
protein_coding	CCNG1	ENSG00000113328.18	458	352	0.8
protein_coding	MMP2	ENSG00000087245.12	1438	1105	0.8
protein_coding	SHROOM3	ENSG00000138771.15	82	63	0.8
protein_coding	CARHSP1	ENSG00000153048.10	69	53	0.8
protein_coding	XPC	ENSG00000154767.14	181	139	0.8
protein_coding	PIAS1	ENSG00000033800.13	112	86	0.8
protein_coding	NOP10	ENSG00000182117.5	400	307	0.8
protein_coding	NOSIP	ENSG00000142546.13	43	33	0.8
protein_coding	TXNL1	ENSG00000091164.12	232	178	0.8
protein_coding	HSPB11	ENSG00000081870.11	103	79	0.8
protein_coding	NSMCE4A	ENSG00000107672.14	30	23	0.8
protein_coding	UQCC3	ENSG00000204922.4	30	23	0.8
protein_coding	IDH3B	ENSG00000101365.20	60	46	0.8
protein_coding	DTX3	ENSG00000178498.15	60	46	0.8
protein_coding	RBMX	ENSG00000147274.14	347	266	0.8
protein_coding	SLC1A1	ENSG00000106688.11	77	59	0.8
protein_coding	TOX2	ENSG00000124191.17	124	95	0.8

protein_coding	NDUFS6	ENSG00000145494.11	171	131	0.8
protein_coding	TMEM39B	ENSG00000121775.17	47	36	0.8
protein_coding	PSMB9	ENSG00000240065.7	47	36	0.8
protein_coding	FBXO9	ENSG00000112146.16	188	144	0.8
protein_coding	PI4K2A	ENSG00000155252.13	111	85	0.8
protein_coding	HEBP1	ENSG00000013583.9	388	297	0.8
protein_coding	PPP1CC	ENSG00000186298.11	247	189	0.8
protein_coding	RHOC	ENSG00000155366.16	281	215	0.8
protein_coding	LARGE1	ENSG00000133424.20	17	13	0.8
protein_coding	STRBP	ENSG00000165209.18	17	13	0.8
protein_coding	ADGRL4	ENSG00000162618.13	17	13	0.8
protein_coding	GSTZ1	ENSG00000100577.18	17	13	0.8
protein_coding	HSPBAP1	ENSG00000169087.10	17	13	0.8
protein_coding	DHX35	ENSG00000101452.14	17	13	0.8
processed_transcript	CCDC18-AS1	ENSG00000223745.7	17	13	0.8
protein_coding	ZSCAN31	ENSG00000235109.7	17	13	0.8
protein_coding	RABGEF1	ENSG00000154710.17	34	26	0.8
protein_coding	SLC35A4	ENSG00000176087.14	34	26	0.8
protein_coding	MINDY1	ENSG00000143409.15	34	26	0.8
protein_coding	CBR3	ENSG00000159231.5	34	26	0.8
protein_coding	RIOX2	ENSG00000170854.17	51	39	0.8
protein_coding	DCTN3	ENSG00000137100.15	51	39	0.8
protein_coding	COX6A1	ENSG00000111775.2	204	156	0.8
protein_coding	SVEP1	ENSG00000165124.17	212	162	0.8
protein_coding	FAM133B	ENSG00000234545.7	72	55	0.8
protein_coding	COMMD4	ENSG00000140365.15	72	55	0.8
protein_coding	PYURF	ENSG00000145337.4	148	113	0.8
protein_coding	FN1	ENSG00000115414.18	20681	15790	0.8
protein_coding	GM2A	ENSG00000196743.8	131	100	0.8
protein_coding	BTN3A1	ENSG00000026950.16	38	29	0.8
protein_coding	BCAS3	ENSG00000141376.22	38	29	0.8
protein_coding	OCIAD2	ENSG00000145247.11	97	74	0.8
protein_coding	SUGP2	ENSG00000064607.16	59	45	0.8
protein_coding	TEAD3	ENSG00000007866.20	59	45	0.8
protein_coding	QARS	ENSG00000172053.17	240	183	0.8
protein_coding	TSPYL1	ENSG00000189241.6	345	263	0.8
protein_coding	NPC2	ENSG00000119655.10	530	404	0.8
protein_coding	GSTK1	ENSG00000197448.13	227	173	0.8
protein_coding	E2F7	ENSG00000165891.15	21	16	0.8
protein_coding	TRMT2A	ENSG00000099899.14	21	16	0.8
protein_coding	CEP192	ENSG00000101639.18	21	16	0.8
protein_coding	ZFP62	ENSG00000196670.13	21	16	0.8
protein_coding	SMDT1	ENSG00000183172.8	42	32	0.8
protein_coding	NUBP1	ENSG00000103274.10	42	32	0.8

protein_coding	AMMECR1	ENSG00000101935.9	84	64	0.8
protein_coding	PRKCD	ENSG00000163932.13	67	51	0.8
protein_coding	GPR108	ENSG00000125734.15	46	35	0.8
protein_coding	KCTD5	ENSG00000167977.8	46	35	0.8
protein_coding	CCPG1	ENSG00000260916.7	209	159	0.8
protein_coding	APEH	ENSG00000164062.12	71	54	0.8
protein_coding	LZTR1	ENSG00000099949.19	71	54	0.8
protein_coding	ALDH18A1	ENSG00000059573.8	96	73	0.8
protein_coding	OTULIN	ENSG00000154124.4	25	19	0.8
protein_coding	UVSSA	ENSG00000163945.16	25	19	0.8
protein_coding	ZNF189	ENSG00000136870.10	25	19	0.8
protein_coding	RCCD1	ENSG00000166965.12	25	19	0.8
protein_coding	LRR1	ENSG00000165501.16	25	19	0.8
protein_coding	NBPF11	ENSG00000263956.6	25	19	0.8
protein_coding	IDUA	ENSG00000127415.12	25	19	0.8
protein_coding	ZWILCH	ENSG00000174442.11	50	38	0.8
protein_coding	CLUH	ENSG00000132361.16	75	57	0.8
protein_coding	CDKN1A	ENSG00000124762.13	1491	1133	0.8
protein_coding	FAM98B	ENSG00000171262.11	54	41	0.8
protein_coding	CFL2	ENSG00000165410.14	270	205	0.8
protein_coding	ALDH3B1	ENSG00000006534.15	137	104	0.8
protein_coding	AAR2	ENSG00000131043.11	83	63	0.8
protein_coding	CSK	ENSG00000103653.16	29	22	0.8
protein_coding	PTCH1	ENSG00000185920.15	29	22	0.8
protein_coding	ZNRD1	ENSG00000066379.14	29	22	0.8
protein_coding	FBXO4	ENSG00000151876.12	29	22	0.8
protein_coding	REPS1	ENSG00000135597.18	58	44	0.8
protein_coding	IDH3G	ENSG00000067829.18	62	47	0.8
protein_coding	ATP5MC2	ENSG00000135390.18	537	407	0.8
protein_coding	FBLIM1	ENSG00000162458.12	260	197	0.8
protein_coding	LPIN1	ENSG00000134324.11	99	75	0.8
protein_coding	ANKRD13A	ENSG00000076513.16	206	156	0.8
protein_coding	KIAA1217	ENSG00000120549.17	173	131	0.8
protein_coding	METTL16	ENSG00000127804.12	70	53	0.8
protein_coding	MRS2	ENSG00000124532.14	37	28	0.8
protein_coding	CAPZA2	ENSG00000198898.13	222	168	0.8
protein_coding	ARIH2	ENSG00000177479.19	189	143	0.8
protein_coding	VPS11	ENSG00000160695.14	41	31	0.8
protein_coding	AKIP1	ENSG00000166452.11	82	62	0.8
protein_coding	PSMD13	ENSG00000185627.17	123	93	0.8
protein_coding	PKP4	ENSG00000144283.21	168	127	0.8
protein_coding	ATP5MC3	ENSG00000154518.9	684	517	0.8
protein_coding	FERMT2	ENSG00000073712.14	172	130	0.8
protein_coding	SNAPIN	ENSG00000143553.10	90	68	0.8

protein_coding	FOXF2	ENSG00000137273.5	560	423	0.8
protein_coding	SKA2	ENSG00000182628.12	94	71	0.8
protein_coding	RBM22	ENSG00000086589.11	98	74	0.8
protein_coding	NME4	ENSG00000103202.12	200	151	0.8
protein_coding	S100A4	ENSG00000196154.11	2599	1962	0.8
protein_coding	CARS	ENSG00000110619.17	257	194	0.8
protein_coding	HECTD3	ENSG00000126107.14	53	40	0.8
protein_coding	METAP1	ENSG00000164024.11	106	80	0.8
protein_coding	MAPRE2	ENSG00000166974.12	57	43	0.8
protein_coding	LANCL2	ENSG00000132434.9	57	43	0.8
protein_coding	CDC16	ENSG00000130177.15	171	129	0.8
protein_coding	NDST1	ENSG00000070614.14	305	230	0.8
protein_coding	CYTH3	ENSG00000008256.15	69	52	0.8
protein_coding	COPS3	ENSG00000141030.12	85	64	0.8
protein_coding	STX7	ENSG00000079950.13	89	67	0.8
protein_coding	SLC25A6	ENSG00000169100.13	936	704	0.8
protein_coding	PDAP1	ENSG00000106244.12	177	133	0.8
protein_coding	ALDH7A1	ENSG00000164904.17	177	133	0.8
protein_coding	RRAGC	ENSG00000116954.7	197	148	0.8
protein_coding	ACTB	ENSG00000075624.14	1159	870	0.8
protein_coding	CIP2A	ENSG00000163507.13	4	3	0.8
protein_coding	DHRS13	ENSG00000167536.13	4	3	0.8
protein_coding	SIPA1L3	ENSG00000105738.10	4	3	0.8
protein_coding	NTNG1	ENSG00000162631.18	4	3	0.8
protein_coding	KNTC1	ENSG00000184445.11	4	3	0.8
protein_coding	CCNJ	ENSG00000107443.15	4	3	0.8
protein_coding	ZNF692	ENSG00000171163.15	4	3	0.8
protein_coding	GABARAP	ENSG00000170296.9	4	3	0.8
protein_coding	ABCC9	ENSG00000069431.11	4	3	0.8
protein_coding	AGAP6	ENSG00000204149.10	4	3	0.8
protein_coding	ZNF714	ENSG00000160352.15	4	3	0.8
protein_coding	ZNF684	ENSG00000117010.16	4	3	0.8
protein_coding	ZNF391	ENSG00000124613.8	4	3	0.8
protein_coding	REC8	ENSG00000100918.12	4	3	0.8
protein_coding	GLYATL2	ENSG00000156689.6	4	3	0.8
protein_coding	GINS1	ENSG00000101003.9	4	3	0.8
protein_coding	PLK4	ENSG00000142731.10	4	3	0.8
lincRNA	AC048341.2	ENSG00000275180.1	4	3	0.8
protein_coding	C2orf92	ENSG00000228486.10	4	3	0.8
sense_intronic	AC002044.1	ENSG00000272330.1	4	3	0.8
protein_coding	ZNF783	ENSG00000204946.9	4	3	0.8
processed_pseudogene	AC006978.1	ENSG00000235859.5	4	3	0.8
antisense	AL590428.1	ENSG00000231652.2	4	3	0.8
antisense	AC008105.3	ENSG00000267121.5	4	3	0.8

protein_coding	PRDM6	ENSG00000061455.10	4	3	0.8
protein_coding	CDT1	ENSG00000167513.8	4	3	0.8
protein_coding	ZWINT	ENSG00000122952.16	4	3	0.8
protein_coding	BNC1	ENSG00000169594.13	4	3	0.8
processed_pseudogene	RPS15AP10	ENSG00000225447.1	4	3	0.8
protein_coding	SIRT4	ENSG00000089163.4	4	3	0.8
protein_coding	FBXO48	ENSG00000204923.3	4	3	0.8
protein_coding	SCNN1D	ENSG00000162572.20	4	3	0.8
antisense	AC005476.2	ENSG00000259146.3	4	3	0.8
antisense	AC135507.1	ENSG00000272182.1	4	3	0.8
protein_coding	SPATC1L	ENSG00000160284.14	4	3	0.8
protein_coding	C10orf55	ENSG00000222047.8	4	3	0.8
protein_coding	CBWD5	ENSG00000147996.16	4	3	0.8
processed_pseudogene	RPS19P3	ENSG00000240463.1	4	3	0.8
protein_coding	ZNF691	ENSG00000164011.17	4	3	0.8
antisense	AP002387.1	ENSG00000254682.1	4	3	0.8
protein_coding	MORN1	ENSG00000116151.13	4	3	0.8
protein_coding	MC1R	ENSG00000258839.3	4	3	0.8
protein_coding	AC008397.2	ENSG00000285188.1	4	3	0.8
transcribed_unprocessed_pseudogene	RASA4CP	ENSG00000228903.7	4	3	0.8
protein_coding	CATSPER1	ENSG00000175294.5	4	3	0.8
antisense	AC012640.2	ENSG00000259802.1	4	3	0.8
lincRNA	AC008115.4	ENSG00000275963.1	4	3	0.8
protein_coding	HAAO	ENSG00000162882.14	4	3	0.8
protein_coding	ZNF578	ENSG00000258405.9	4	3	0.8
protein_coding	KIF17	ENSG00000117245.12	4	3	0.8
protein_coding	CACNB4	ENSG00000182389.19	4	3	0.8
protein_coding	SFI1	ENSG00000198089.15	8	6	0.8
protein_coding	CELF2	ENSG00000048740.18	8	6	0.8
protein_coding	MAPK8IP1	ENSG00000121653.11	8	6	0.8
protein_coding	METTL1	ENSG00000037897.16	8	6	0.8
protein_coding	TRIM6	ENSG00000121236.20	8	6	0.8
lincRNA	LINC01252	ENSG00000247157.6	8	6	0.8
protein_coding	HDAC10	ENSG00000100429.17	8	6	0.8
processed_transcript	C8orf31	ENSG00000177335.10	8	6	0.8
protein_coding	PRC1	ENSG00000198901.13	8	6	0.8
protein_coding	PTPRS	ENSG00000105426.16	8	6	0.8
protein_coding	TMEM81	ENSG00000174529.7	8	6	0.8
processed_pseudogene	AC114728.1	ENSG00000241612.1	8	6	0.8
antisense	AC105942.1	ENSG00000235501.5	8	6	0.8
protein_coding	ZNF17	ENSG00000186272.12	8	6	0.8
processed_pseudogene	RPL10P16	ENSG00000178464.6	8	6	0.8

protein_coding	PUS10	ENSG00000162927.13	8	6	0.8
antisense	DGUOK-AS1	ENSG00000237883.1	8	6	0.8
antisense	AL158163.2	ENSG00000278601.1	8	6	0.8
protein_coding	C8orf44	ENSG00000213865.7	8	6	0.8
protein_coding	KIAA0895L	ENSG00000196123.12	12	9	0.8
protein_coding	ACSF3	ENSG00000176715.16	12	9	0.8
protein_coding	LIMD1	ENSG00000144791.9	12	9	0.8
protein_coding	COQ3	ENSG00000132423.11	12	9	0.8
protein_coding	PCDHB10	ENSG00000120324.8	12	9	0.8
protein_coding	KIF4A	ENSG00000090889.11	12	9	0.8
antisense	AL591845.1	ENSG00000116883.8	12	9	0.8
protein_coding	NAALADL1	ENSG00000168060.15	12	9	0.8
protein_coding	VDR	ENSG00000111424.10	12	9	0.8
lincRNA	AP001330.5	ENSG00000271882.1	12	9	0.8
protein_coding	AMN1	ENSG00000151743.10	12	9	0.8
protein_coding	ZNF555	ENSG00000186300.11	12	9	0.8
transcribed_unprocessed_pseudogene	RNF216P1	ENSG00000196204.11	16	12	0.8
protein_coding	ACAN	ENSG00000157766.16	16	12	0.8
Mt_tRNA	MT-TQ	ENSG00000210107.1	16	12	0.8
protein_coding	SMG9	ENSG00000105771.13	16	12	0.8
protein_coding	MCM9	ENSG00000111877.17	16	12	0.8
protein_coding	ITGA7	ENSG00000135424.16	16	12	0.8
protein_coding	CAPS2	ENSG00000180881.19	16	12	0.8
protein_coding	PSG1	ENSG00000231924.9	16	12	0.8
protein_coding	ANGPTL1	ENSG00000116194.12	16	12	0.8
protein_coding	INAFM2	ENSG00000259330.2	20	15	0.8
protein_coding	RTKN	ENSG00000114993.16	20	15	0.8
transcribed_unprocessed_pseudogene	RPL32P3	ENSG00000251474.6	20	15	0.8
lincRNA	LINC00941	ENSG00000285517.1	24	18	0.8
lincRNA	LINC00467	ENSG00000153363.12	24	18	0.8
protein_coding	NDUFAF6	ENSG00000156170.12	24	18	0.8
protein_coding	TGIF2	ENSG00000118707.9	24	18	0.8
lincRNA	MIR34AHG	ENSG00000228526.7	28	21	0.8
protein_coding	TRMT9B	ENSG00000250305.8	28	21	0.8
protein_coding	NAV3	ENSG00000067798.15	28	21	0.8
protein_coding	ZFAND2B	ENSG00000158552.12	32	24	0.8
protein_coding	CAAP1	ENSG00000120159.12	36	27	0.8
protein_coding	RITA1	ENSG00000139405.15	36	27	0.8
protein_coding	ETV5	ENSG00000244405.7	40	30	0.8
protein_coding	ERMARD	ENSG00000130023.15	40	30	0.8
protein_coding	CEPT1	ENSG00000134255.13	44	33	0.8

protein_coding	ELP5	ENSG00000170291.14	44	33	0.8
protein_coding	CCDC85C	ENSG00000205476.8	44	33	0.8
protein_coding	GIPC1	ENSG00000123159.15	48	36	0.8
protein_coding	RNF26	ENSG00000173456.4	48	36	0.8
protein_coding	FARSA	ENSG00000179115.10	52	39	0.8
protein_coding	KIAA1522	ENSG00000162522.10	52	39	0.8
protein_coding	PID1	ENSG00000153823.18	56	42	0.8
protein_coding	S1PR1	ENSG00000170989.9	56	42	0.8
protein_coding	MLX	ENSG00000108788.11	60	45	0.8
protein_coding	CARS2	ENSG00000134905.16	80	60	0.8
protein_coding	SURF1	ENSG00000148290.9	88	66	0.8
protein_coding	YEATS2	ENSG00000163872.15	92	69	0.8
protein_coding	SAMHD1	ENSG00000101347.9	92	69	0.8
protein_coding	RIC1	ENSG00000107036.11	96	72	0.8
protein_coding	PDLIM2	ENSG00000120913.23	152	114	0.8
protein_coding	SNX29	ENSG00000048471.13	152	114	0.8
protein_coding	MAX	ENSG00000125952.18	184	138	0.8
protein_coding	STK24	ENSG00000102572.14	243	182	0.7
protein_coding	SNU13	ENSG00000100138.14	239	179	0.7
protein_coding	NQO1	ENSG00000181019.12	1764	1321	0.7
protein_coding	CDYL	ENSG00000153046.17	119	89	0.7
protein_coding	NMD3	ENSG00000169251.12	99	74	0.7
protein_coding	OSER1	ENSG00000132823.10	95	71	0.7
protein_coding	PDK2	ENSG00000005882.11	87	65	0.7
protein_coding	SLC20A1	ENSG00000144136.10	174	130	0.7
protein_coding	COPZ1	ENSG00000111481.9	174	130	0.7
protein_coding	COL6A1	ENSG00000142156.14	7349	5490	0.7
protein_coding	DPAGT1	ENSG00000172269.18	83	62	0.7
protein_coding	U2AF2	ENSG00000063244.12	71	53	0.7
protein_coding	LAMTOR1	ENSG00000149357.9	410	306	0.7
protein_coding	OXA1L	ENSG00000155463.12	185	138	0.7
protein_coding	RBBP7	ENSG00000102054.17	244	182	0.7
protein_coding	PSME1	ENSG00000092010.14	366	273	0.7
protein_coding	PEX2	ENSG00000164751.14	59	44	0.7
protein_coding	TATDN2	ENSG00000157014.10	59	44	0.7
protein_coding	KLHL36	ENSG00000135686.12	114	85	0.7
protein_coding	RPUSD4	ENSG00000165526.8	55	41	0.7
protein_coding	C1GALT1	ENSG00000106392.10	98	73	0.7
protein_coding	ZNF706	ENSG00000120963.11	94	70	0.7
protein_coding	SPATS2L	ENSG00000196141.13	1499	1116	0.7
protein_coding	CSTB	ENSG00000160213.7	579	431	0.7
protein_coding	ZNF195	ENSG00000005801.17	43	32	0.7
protein_coding	VEZT	ENSG00000028203.17	82	61	0.7
protein_coding	GPX4	ENSG00000167468.16	777	578	0.7

protein_coding	BHLHE40	ENSG00000134107.4	39	29	0.7
protein_coding	TRIT1	ENSG00000043514.16	39	29	0.7
protein_coding	CCBE1	ENSG00000183287.14	273	203	0.7
protein_coding	ALCAM	ENSG00000170017.12	1025	762	0.7
protein_coding	OLA1	ENSG00000138430.15	187	139	0.7
protein_coding	ENO1	ENSG00000074800.15	775	576	0.7
protein_coding	FBLN1	ENSG00000077942.18	2015	1497	0.7
protein_coding	PIGF	ENSG00000151665.12	35	26	0.7
protein_coding	TMEM150A	ENSG00000168890.13	35	26	0.7
protein_coding	TMEM117	ENSG00000139173.9	35	26	0.7
protein_coding	BCL9L	ENSG00000186174.12	140	104	0.7
protein_coding	HMCES	ENSG00000183624.13	66	49	0.7
protein_coding	MTX2	ENSG00000128654.13	66	49	0.7
protein_coding	TMEM60	ENSG00000135211.5	97	72	0.7
protein_coding	SPON2	ENSG00000159674.11	1160	861	0.7
protein_coding	UQCRFS1	ENSG00000169021.5	128	95	0.7
protein_coding	IFITM2	ENSG00000185201.16	469	348	0.7
protein_coding	CARD8	ENSG00000105483.17	31	23	0.7
protein_coding	SLC27A3	ENSG00000143554.13	31	23	0.7
sense_overlapping	AC073896.4	ENSG00000258199.1	93	69	0.7
protein_coding	CNRIP1	ENSG00000119865.8	124	92	0.7
protein_coding	RTN3	ENSG00000133318.13	577	428	0.7
protein_coding	TIMP1	ENSG00000102265.11	5684	4216	0.7
protein_coding	THAP11	ENSG00000168286.2	89	66	0.7
protein_coding	CTNNBIP1	ENSG00000178585.14	58	43	0.7
protein_coding	B4GALT5	ENSG00000158470.5	116	86	0.7
protein_coding	ZNF592	ENSG00000166716.9	27	20	0.7
protein_coding	SAMD14	ENSG00000167100.14	27	20	0.7
protein_coding	FGGY	ENSG00000172456.17	27	20	0.7
protein_coding	BAG6	ENSG00000204463.12	162	120	0.7
protein_coding	PLA2G15	ENSG00000103066.12	104	77	0.7
protein_coding	ARL2BP	ENSG00000102931.7	204	151	0.7
protein_coding	NIPA2	ENSG00000140157.14	127	94	0.7
protein_coding	PCYT1A	ENSG00000161217.11	142	105	0.7
processed_transcript	LINC00963	ENSG00000204054.13	69	51	0.7
protein_coding	AEBP1	ENSG00000106624.10	272	201	0.7
protein_coding	VEZF1	ENSG00000136451.8	195	144	0.7
protein_coding	A4GALT	ENSG00000128274.16	42	31	0.7
protein_coding	TTC39C	ENSG00000168234.12	84	62	0.7
protein_coding	DOCK1	ENSG00000150760.12	168	124	0.7
protein_coding	RSBN1L	ENSG00000187257.15	61	45	0.7
protein_coding	CLIP4	ENSG00000115295.19	137	101	0.7
protein_coding	GPSM2	ENSG00000121957.13	19	14	0.7
protein_coding	GTF2E1	ENSG00000153767.9	19	14	0.7

protein_coding	CDH2	ENSG00000170558.8	38	28	0.7
protein_coding	FARP2	ENSG00000006607.13	38	28	0.7
protein_coding	COG3	ENSG00000136152.14	38	28	0.7
protein_coding	PPM1F	ENSG00000100034.13	38	28	0.7
transcribed_unprocessed_pseudogene	ZNF252P	ENSG00000196922.10	57	42	0.7
protein_coding	PRMT1	ENSG00000126457.21	133	98	0.7
protein_coding	RPS28	ENSG00000233927.4	505	372	0.7
protein_coding	TOMM22	ENSG00000100216.5	148	109	0.7
protein_coding	FAM180A	ENSG00000189320.8	239	176	0.7
protein_coding	RNF139	ENSG00000170881.4	110	81	0.7
protein_coding	PRPF31	ENSG00000105618.13	91	67	0.7
protein_coding	TRIR	ENSG00000123144.10	91	67	0.7
protein_coding	ATPAF1	ENSG00000123472.12	72	53	0.7
protein_coding	EAF1	ENSG00000144597.13	53	39	0.7
protein_coding	CDK16	ENSG00000102225.15	53	39	0.7
protein_coding	POLR2I	ENSG00000105258.8	87	64	0.7
protein_coding	ZDHHC2	ENSG00000104219.12	87	64	0.7
protein_coding	PTPN4	ENSG00000088179.8	34	25	0.7
protein_coding	MRPL11	ENSG00000174547.13	68	50	0.7
protein_coding	ITGB1BP1	ENSG00000119185.12	185	136	0.7
protein_coding	RAB8B	ENSG00000166128.12	117	86	0.7
protein_coding	FAM91A1	ENSG00000176853.15	132	97	0.7
protein_coding	TARBP1	ENSG00000059588.9	15	11	0.7
protein_coding	TIFA	ENSG00000145365.10	15	11	0.7
protein_coding	TBC1D25	ENSG00000068354.15	15	11	0.7
protein_coding	PLAGL2	ENSG00000126003.6	15	11	0.7
antisense	AC009779.2	ENSG00000258056.2	15	11	0.7
protein_coding	FAM198B	ENSG00000164125.15	30	22	0.7
protein_coding	GAL3ST4	ENSG00000197093.10	45	33	0.7
protein_coding	NADK2	ENSG00000152620.12	60	44	0.7
protein_coding	GATAD2A	ENSG00000167491.17	75	55	0.7
protein_coding	SYS1	ENSG00000204070.9	90	66	0.7
protein_coding	STK25	ENSG00000115694.14	225	165	0.7
protein_coding	ACVR1	ENSG00000115170.13	281	206	0.7
protein_coding	COL16A1	ENSG00000084636.17	206	151	0.7
protein_coding	F2RL2	ENSG00000164220.6	86	63	0.7
protein_coding	ARHGAP32	ENSG00000134909.18	56	41	0.7
protein_coding	SNAPC2	ENSG00000104976.11	41	30	0.7
protein_coding	CIAPIN1	ENSG00000005194.14	41	30	0.7
protein_coding	FBXO28	ENSG00000143756.11	82	60	0.7
protein_coding	DAZAP1	ENSG00000071626.16	108	79	0.7
protein_coding	CHKA	ENSG00000110721.11	67	49	0.7

protein_coding	DPY30	ENSG00000162961.13	93	68	0.7
protein_coding	ADNP	ENSG00000101126.16	186	136	0.7
protein_coding	RRAS2	ENSG00000133818.13	171	125	0.7
protein_coding	TMEM67	ENSG00000164953.15	26	19	0.7
protein_coding	UBALD1	ENSG00000153443.12	26	19	0.7
protein_coding	ZFAT	ENSG00000066827.15	26	19	0.7
protein_coding	CBLL1	ENSG00000105879.11	52	38	0.7
protein_coding	TTBK2	ENSG00000128881.16	52	38	0.7
protein_coding	RBM5	ENSG00000003756.16	104	76	0.7
protein_coding	PCOLCE	ENSG00000106333.12	928	678	0.7
protein_coding	JTB	ENSG00000143543.14	282	206	0.7
protein_coding	UROS	ENSG00000188690.13	89	65	0.7
protein_coding	VOPP1	ENSG00000154978.12	215	157	0.7
protein_coding	TMEM161A	ENSG00000064545.14	37	27	0.7
protein_coding	TMPO	ENSG00000120802.13	37	27	0.7
protein_coding	SH3YL1	ENSG00000035115.21	37	27	0.7
protein_coding	BLVRA	ENSG00000106605.10	111	81	0.7
protein_coding	CDC25B	ENSG00000101224.17	440	321	0.7
protein_coding	NRP1	ENSG00000099250.17	532	388	0.7
protein_coding	MTCL1	ENSG00000168502.17	48	35	0.7
protein_coding	BDH2	ENSG00000164039.14	59	43	0.7
protein_coding	CARM1	ENSG00000142453.11	217	158	0.7
protein_coding	ETHE1	ENSG00000105755.7	147	107	0.7
protein_coding	MLF¬†1	ENSG00000178053.17	11	8	0.7
protein_coding	THAP10	ENSG00000129028.8	11	8	0.7
protein_coding	SLC41A2	ENSG00000136052.9	11	8	0.7
protein_coding	SMAD6	ENSG00000137834.14	11	8	0.7
processed_pseudogene	RPL17P50	ENSG00000213700.3	11	8	0.7
processed_transcript	AC090517.5	ENSG00000285331.2	11	8	0.7
protein_coding	PPP1R16A	ENSG00000160972.9	22	16	0.7
protein_coding	PSTPIP2	ENSG00000152229.18	22	16	0.7
protein_coding	C4orf33	ENSG00000151470.12	22	16	0.7
protein_coding	TIMM29	ENSG00000142444.6	33	24	0.7
protein_coding	MRPS12	ENSG00000128626.11	33	24	0.7
protein_coding	PKIA	ENSG00000171033.12	33	24	0.7
protein_coding	DNAJB5	ENSG00000137094.14	33	24	0.7
protein_coding	BIN3	ENSG00000147439.12	33	24	0.7
protein_coding	IPO13	ENSG00000117408.10	33	24	0.7
protein_coding	CUL7	ENSG00000044090.8	33	24	0.7
protein_coding	DHX16	ENSG00000204560.9	44	32	0.7
protein_coding	ZNF677	ENSG00000197928.10	44	32	0.7
protein_coding	FMNL3	ENSG00000161791.13	55	40	0.7
protein_coding	STK40	ENSG00000196182.10	55	40	0.7
protein_coding	FAM118B	ENSG00000197798.8	55	40	0.7

protein_coding	RUSC1	ENSG00000160753.15	55	40	0.7
protein_coding	OGFOD1	ENSG00000087263.16	66	48	0.7
protein_coding	G6PD	ENSG00000160211.17	286	208	0.7
protein_coding	NCBP2	ENSG00000114503.10	150	109	0.7
protein_coding	PSMD10	ENSG00000101843.18	84	61	0.7
protein_coding	COTL1	ENSG00000103187.7	460	334	0.7
protein_coding	RHOG	ENSG00000177105.9	113	82	0.7
protein_coding	CRABP2	ENSG00000143320.8	630	457	0.7
protein_coding	ITGA5	ENSG00000161638.10	142	103	0.7
protein_coding	NIPAL3	ENSG00000001461.16	171	124	0.7
protein_coding	GSDMD	ENSG00000104518.10	40	29	0.7
protein_coding	SNX7	ENSG00000162627.16	200	145	0.7
transcribed_processed_pseudogene	CENPBD1P1	ENSG00000213753.10	69	50	0.7
protein_coding	PIP5K1A	ENSG00000143398.19	98	71	0.7
protein_coding	POLR1C	ENSG00000171453.18	29	21	0.7
protein_coding	PPIE	ENSG00000084072.16	29	21	0.7
protein_coding	RPS6KA6	ENSG00000072133.10	29	21	0.7
protein_coding	STYXL1	ENSG00000127952.16	29	21	0.7
protein_coding	ABRAXAS1	ENSG00000163322.13	29	21	0.7
protein_coding	TRIP4	ENSG00000103671.9	58	42	0.7
protein_coding	BTG2	ENSG00000159388.5	58	42	0.7
lincRNA	EBLN3P	ENSG00000281649.1	87	63	0.7
protein_coding	SEC24C	ENSG00000176986.15	87	63	0.7
protein_coding	GPR176	ENSG00000166073.10	583	422	0.7
protein_coding	UBL5	ENSG00000198258.10	449	325	0.7
protein_coding	CCDC106	ENSG00000173581.7	47	34	0.7
protein_coding	COL27A1	ENSG00000196739.14	94	68	0.7
protein_coding	MORC3	ENSG00000159256.12	65	47	0.7
protein_coding	UCHL3	ENSG00000118939.17	65	47	0.7
protein_coding	IMPDH1	ENSG00000106348.17	65	47	0.7
protein_coding	FTL	ENSG00000087086.14	10148	7336	0.7
protein_coding	AIG1	ENSG00000146416.18	83	60	0.7
protein_coding	BLCAP	ENSG00000166619.13	137	99	0.7
protein_coding	COL15A1	ENSG00000204291.10	292	211	0.7
protein_coding	LRRC41	ENSG00000132128.16	317	229	0.7
protein_coding	FOSL1	ENSG00000175592.8	18	13	0.7
protein_coding	PARS2	ENSG00000162396.5	18	13	0.7
protein_coding	COQ8A	ENSG00000163050.16	36	26	0.7
TEC	AP001972.5	ENSG00000279117.1	54	39	0.7
protein_coding	RAB11FIP3	ENSG00000090565.15	54	39	0.7
protein_coding	CBY1	ENSG00000100211.10	126	91	0.7
protein_coding	RNF150	ENSG00000170153.10	392	283	0.7

protein_coding	LGALS1	ENSG00000100097.11	6961	5024	0.7
protein_coding	SERF2	ENSG00000140264.19	1566	1130	0.7
protein_coding	LMBR1	ENSG00000105983.20	79	57	0.7
protein_coding	DGKA	ENSG00000065357.19	158	114	0.7
protein_coding	ZNF330	ENSG00000109445.10	61	44	0.7
protein_coding	MAP2K5	ENSG00000137764.19	43	31	0.7
protein_coding	PHF11	ENSG00000136147.17	86	62	0.7
protein_coding	VEGFC	ENSG00000150630.3	68	49	0.7
protein_coding	PEAR1	ENSG00000187800.13	68	49	0.7
protein_coding	ITGA8	ENSG00000077943.7	1091	786	0.7
protein_coding	HK1	ENSG00000156515.23	236	170	0.7
protein_coding	TBCEL	ENSG00000154114.12	25	18	0.7
protein_coding	NUP155	ENSG00000113569.15	25	18	0.7
protein_coding	FAM219A	ENSG00000164970.14	25	18	0.7
protein_coding	ALPK1	ENSG00000073331.17	25	18	0.7
protein_coding	SETD6	ENSG00000103037.11	25	18	0.7
protein_coding	HS1BP3	ENSG00000118960.12	50	36	0.7
protein_coding	SREBF2	ENSG00000198911.11	50	36	0.7
protein_coding	EML1	ENSG00000066629.17	100	72	0.7
protein_coding	TNFSF12	ENSG00000239697.10	125	90	0.7
protein_coding	GPN1	ENSG00000198522.13	82	59	0.7
protein_coding	EPHB4	ENSG00000196411.9	203	146	0.7
protein_coding	SH3BGRL3	ENSG00000142669.14	1605	1154	0.7
protein_coding	MAPK8	ENSG00000107643.16	32	23	0.7
protein_coding	TUBG2	ENSG00000037042.8	32	23	0.7
protein_coding	KLF11	ENSG00000172059.10	32	23	0.7
protein_coding	SETDB1	ENSG00000143379.12	32	23	0.7
protein_coding	ELMOD3	ENSG00000115459.17	32	23	0.7
protein_coding	ZNF219	ENSG00000165804.15	96	69	0.7
protein_coding	YARS	ENSG00000134684.10	128	92	0.7
protein_coding	TUFM	ENSG00000178952.10	192	138	0.7
protein_coding	MTHFR	ENSG00000177000.12	71	51	0.7
protein_coding	IFT22	ENSG00000128581.15	39	28	0.7
protein_coding	VPS50	ENSG00000004766.16	39	28	0.7
protein_coding	SLC12A9	ENSG00000146828.17	39	28	0.7
protein_coding	SPTAN1	ENSG00000197694.15	163	117	0.7
protein_coding	LRRC57	ENSG00000180979.9	85	61	0.7
protein_coding	MATN2	ENSG00000132561.13	46	33	0.7
protein_coding	ARHGAP31	ENSG00000031081.10	46	33	0.7
protein_coding	TNS2	ENSG00000111077.17	152	109	0.7
protein_coding	ACOT9	ENSG00000123130.16	60	43	0.7
protein_coding	MGST2	ENSG00000085871.8	60	43	0.7
protein_coding	CDIP1	ENSG00000089486.16	60	43	0.7
protein_coding	ADD1	ENSG00000087274.16	363	260	0.7

protein_coding	NEK7	ENSG00000151414.14	257	184	0.7
protein_coding	GLYR1	ENSG00000140632.16	109	78	0.7
protein_coding	POLDIP2	ENSG00000004142.11	116	83	0.7
protein_coding	KCNMA1	ENSG00000156113.22	417	298	0.7
protein_coding	POM121	ENSG00000196313.11	7	5	0.7
processed_pseudogene	AL365357.1	ENSG00000213058.3	7	5	0.7
protein_coding	EPHA5	ENSG00000145242.13	7	5	0.7
protein_coding	ST6GALNAC	ENSG00000117069.14	7	5	0.7
sense_overlapping	AC010168.2	ENSG00000261324.2	7	5	0.7
protein_coding	CGAS	ENSG00000164430.15	7	5	0.7
protein_coding	TMEM216	ENSG00000187049.9	7	5	0.7
TEC	AC091825.1	ENSG00000280047.1	7	5	0.7
protein_coding	TXLNB	ENSG00000164440.14	7	5	0.7
lincRNA	LINC01270	ENSG00000203999.8	7	5	0.7
protein_coding	MPP2	ENSG00000108852.14	7	5	0.7
protein_coding	RBL1	ENSG00000080839.11	7	5	0.7
protein_coding	KIF21A	ENSG00000139116.18	7	5	0.7
protein_coding	SERPINB9	ENSG00000170542.5	7	5	0.7
protein_coding	SOBP	ENSG00000112320.11	7	5	0.7
protein_coding	YJU2	ENSG00000105248.15	7	5	0.7
lincRNA	AC026979.2	ENSG00000271869.1	7	5	0.7
protein_coding	LY6G5C	ENSG00000204428.12	7	5	0.7
protein_coding	CIB2	ENSG00000136425.13	7	5	0.7
sense_intronic	AC007390.2	ENSG00000272054.1	7	5	0.7
protein_coding	PLXNC1	ENSG00000136040.8	7	5	0.7
protein_coding	ZSCAN16	ENSG00000196812.4	7	5	0.7
protein_coding	SEC61A2	ENSG00000065665.20	14	10	0.7
protein_coding	ITIH5	ENSG00000123243.14	14	10	0.7
protein_coding	SLC45A4	ENSG00000022567.9	14	10	0.7
protein_coding	WDR91	ENSG00000105875.13	14	10	0.7
protein_coding	NTHL1	ENSG00000065057.7	14	10	0.7
antisense	AL451165.2	ENSG00000272288.5	14	10	0.7
protein_coding	MON1A	ENSG00000164077.14	14	10	0.7
protein_coding	KIF22	ENSG00000079616.12	14	10	0.7
transcribed_unprocessed_pseudogene	HERC2P2	ENSG00000276550.4	21	15	0.7
protein_coding	ZNF667	ENSG00000198046.11	21	15	0.7
protein_coding	TMEM132B	ENSG00000139364.10	21	15	0.7
protein_coding	TBKBP1	ENSG00000198933.9	21	15	0.7
protein_coding	ZNF496	ENSG00000162714.12	28	20	0.7
protein_coding	RREB1	ENSG00000124782.19	35	25	0.7
protein_coding	ZNF83	ENSG00000167766.18	42	30	0.7
protein_coding	LYPLAL1	ENSG00000143353.11	42	30	0.7

protein_coding	RNF135	ENSG00000181481.13	49	35	0.7
protein_coding	SLC40A1	ENSG00000138449.10	49	35	0.7
protein_coding	MED25	ENSG00000104973.17	56	40	0.7
protein_coding	TADA3	ENSG00000171148.13	63	45	0.7
protein_coding	WDR48	ENSG00000114742.13	84	60	0.7
protein_coding	RALGAPB	ENSG00000170471.14	84	60	0.7
protein_coding	PHF2	ENSG00000197724.10	84	60	0.7
protein_coding	MAP3K3	ENSG00000198909.7	91	65	0.7
protein_coding	RAD23B	ENSG00000119318.12	475	339	0.7
protein_coding	PLS3	ENSG00000102024.17	261	186	0.7
protein_coding	PFN1	ENSG00000108518.7	1272	906	0.7
protein_coding	FAM229B	ENSG00000203778.7	59	42	0.7
protein_coding	MPRIP	ENSG00000133030.20	274	195	0.7
protein_coding	XPO6	ENSG00000169180.11	149	106	0.7
protein_coding	MDH2	ENSG00000146701.11	270	192	0.7
protein_coding	ALDH9A1	ENSG00000143149.12	173	123	0.7
protein_coding	TMEM158	ENSG00000249992.1	584	415	0.7
protein_coding	DRG2	ENSG00000108591.9	38	27	0.7
protein_coding	PPIA	ENSG00000196262.13	373	265	0.7
protein_coding	FBLN5	ENSG00000140092.14	480	341	0.7
protein_coding	ARHGEF2	ENSG00000116584.17	100	71	0.7
protein_coding	ING1	ENSG00000153487.12	31	22	0.7
protein_coding	STX8	ENSG00000170310.14	62	44	0.7
protein_coding	SLC25A39	ENSG00000013306.15	93	66	0.7
protein_coding	CRELD2	ENSG00000184164.14	93	66	0.7
protein_coding	GALK2	ENSG00000156958.14	55	39	0.7
protein_coding	ANAPC7	ENSG00000196510.12	55	39	0.7
protein_coding	SNRPD2	ENSG00000125743.10	319	226	0.7
protein_coding	APIP	ENSG00000149089.12	24	17	0.7
protein_coding	PARP8	ENSG00000151883.17	24	17	0.7
protein_coding	TRAPPC9	ENSG00000167632.15	24	17	0.7
protein_coding	TNFRSF12A	ENSG00000006327.13	24	17	0.7
protein_coding	ZC3H8	ENSG00000144161.12	24	17	0.7
protein_coding	INTS1	ENSG00000164880.15	24	17	0.7
protein_coding	TDRP	ENSG00000180190.11	24	17	0.7
protein_coding	PARPBP	ENSG00000185480.11	24	17	0.7
protein_coding	ZNF606	ENSG00000166704.11	24	17	0.7
protein_coding	PPP1R37	ENSG00000104866.10	24	17	0.7
protein_coding	PLEKHF1	ENSG00000166289.5	24	17	0.7
protein_coding	COMMD10	ENSG00000145781.8	48	34	0.7
protein_coding	CRAT	ENSG00000095321.16	72	51	0.7
protein_coding	CYC1	ENSG00000179091.4	89	63	0.7
protein_coding	TMEM205	ENSG00000105518.13	65	46	0.7
protein_coding	TPCN1	ENSG00000186815.12	65	46	0.7

protein_coding	PKIG	ENSG00000168734.13	188	133	0.7
protein_coding	WDR20	ENSG00000140153.17	41	29	0.7
protein_coding	TRIM69	ENSG00000185880.12	41	29	0.7
protein_coding	NAB2	ENSG00000166886.12	82	58	0.7
protein_coding	FLNC	ENSG00000128591.15	239	169	0.7
protein_coding	MCUB	ENSG00000005059.15	58	41	0.7
protein_coding	SHKBP1	ENSG00000160410.14	58	41	0.7
protein_coding	COA1	ENSG00000106603.18	75	53	0.7
protein_coding	COL12A1	ENSG00000111799.20	1868	1319	0.7
protein_coding	LHPP	ENSG00000107902.13	17	12	0.7
protein_coding	ZNF251	ENSG00000198169.8	17	12	0.7
lincRNA	LINC01705	ENSG00000232679.1	17	12	0.7
protein_coding	IL15RA	ENSG00000134470.20	17	12	0.7
protein_coding	ERMP1	ENSG00000099219.13	17	12	0.7
protein_coding	MVB12B	ENSG00000196814.14	17	12	0.7
protein_coding	BOK	ENSG00000176720.5	17	12	0.7
protein_coding	IQSEC2	ENSG00000124313.14	34	24	0.7
protein_coding	FAM216A	ENSG00000204856.11	34	24	0.7
protein_coding	ZNF354C	ENSG00000177932.6	34	24	0.7
protein_coding	STXBP6	ENSG00000168952.15	34	24	0.7
protein_coding	PRPF19	ENSG00000110107.8	34	24	0.7
lincRNA	LINC00847	ENSG00000245060.6	34	24	0.7
protein_coding	PARP3	ENSG00000041880.14	68	48	0.7
protein_coding	FAM45A	ENSG00000119979.17	136	96	0.7
protein_coding	HTRA1	ENSG00000166033.12	849	599	0.7
protein_coding	CYB5R1	ENSG00000159348.12	190	134	0.7
protein_coding	PTPN21	ENSG00000070778.12	61	43	0.7
protein_coding	SLC2A4RG	ENSG00000125520.13	210	148	0.7
protein_coding	RAF1	ENSG00000132155.11	132	93	0.7
protein_coding	NAGK	ENSG00000124357.12	115	81	0.7
protein_coding	CSNK2B	ENSG00000204435.13	186	131	0.7
protein_coding	CALHM2	ENSG00000138172.10	71	50	0.7
protein_coding	HAX1	ENSG00000143575.14	142	100	0.7
protein_coding	SEC14L2	ENSG00000100003.17	27	19	0.7
protein_coding	TRIM52	ENSG00000183718.5	27	19	0.7
protein_coding	SLC25A42	ENSG00000181035.13	27	19	0.7
protein_coding	VPS25	ENSG00000131475.6	54	38	0.7
protein_coding	BAALC	ENSG00000164929.16	54	38	0.7
protein_coding	PAX9	ENSG00000198807.12	415	292	0.7
protein_coding	URB1	ENSG00000142207.6	37	26	0.7
protein_coding	TIMM21	ENSG00000075336.11	37	26	0.7
protein_coding	TEX10	ENSG00000136891.13	37	26	0.7
protein_coding	MRTFB	ENSG00000186260.16	37	26	0.7
protein_coding	FAN1	ENSG00000198690.9	37	26	0.7

protein_coding	GOLM1	ENSG00000135052.16	74	52	0.7
protein_coding	CAMTA1	ENSG00000171735.18	141	99	0.7
protein_coding	DNM2	ENSG00000079805.16	124	87	0.7
protein_coding	PIAS3	ENSG00000131788.15	67	47	0.7
protein_coding	SRF	ENSG00000112658.7	77	54	0.7
protein_coding	DPYSL2	ENSG00000092964.17	539	378	0.7
protein_coding	PCDH18	ENSG00000189184.11	924	648	0.7
protein_coding	LTBP2	ENSG00000119681.11	1175	824	0.7
protein_coding	HOMER3	ENSG00000051128.18	241	169	0.7
protein_coding	GPR68	ENSG00000119714.10	10	7	0.7
transcribed_unprocessed_pseudogene	AC093752.1	ENSG00000245958.6	10	7	0.7
protein_coding	RRP12	ENSG00000052749.13	10	7	0.7
protein_coding	PECR	ENSG00000115425.13	10	7	0.7
protein_coding	DOHH	ENSG00000129932.8	10	7	0.7
protein_coding	PXYLP1	ENSG00000155893.12	10	7	0.7
protein_coding	IKBKE	ENSG00000263528.7	10	7	0.7
protein_coding	SEC31B	ENSG00000075826.16	10	7	0.7
protein_coding	ZNF35	ENSG00000169981.10	10	7	0.7
protein_coding	RNF19B	ENSG00000116514.16	10	7	0.7
antisense	SNAI3-AS1	ENSG00000260630.6	10	7	0.7
protein_coding	C9orf72	ENSG00000147894.15	10	7	0.7
protein_coding	ZNF717	ENSG00000227124.9	10	7	0.7
processed_pseudogene	AL133260.1	ENSG00000219747.1	10	7	0.7
protein_coding	MCM2	ENSG00000073111.13	10	7	0.7
protein_coding	ELAC1	ENSG00000141642.8	10	7	0.7
transcribed_unprocessed_pseudogene	PAR	ENSG00000223773.7	10	7	0.7
protein_coding	PAK1IP1	ENSG00000111845.4	20	14	0.7
protein_coding	WDR90	ENSG00000161996.18	20	14	0.7
protein_coding	APOLD1	ENSG00000178878.12	20	14	0.7
protein_coding	ZNF419	ENSG00000105136.20	20	14	0.7
protein_coding	DCUN1D3	ENSG00000188215.9	30	21	0.7
protein_coding	POMGNT2	ENSG00000144647.5	30	21	0.7
protein_coding	BRIX1	ENSG00000113460.12	40	28	0.7
protein_coding	DUSP7	ENSG00000164086.9	50	35	0.7
protein_coding	FAM222B	ENSG00000173065.13	50	35	0.7
protein_coding	WDR19	ENSG00000157796.17	60	42	0.7
protein_coding	NBAS	ENSG00000151779.12	80	56	0.7
protein_coding	SHISA4	ENSG00000198892.6	90	63	0.7
protein_coding	MYL6B	ENSG00000196465.10	110	77	0.7
protein_coding	CLNS1A	ENSG00000074201.8	163	114	0.7
protein_coding	MPLKIP	ENSG00000168303.7	143	100	0.7

protein_coding	GNAQ	ENSG00000156052.10	256	179	0.7
protein_coding	HMGXB4	ENSG00000100281.13	103	72	0.7
protein_coding	FAM43A	ENSG00000185112.5	103	72	0.7
protein_coding	CAPN2	ENSG00000162909.17	850	594	0.7
protein_coding	STARD7	ENSG00000084090.13	415	290	0.7
lincRNA	TP53TG1	ENSG00000182165.17	73	51	0.7
protein_coding	GTF3C3	ENSG00000119041.10	63	44	0.7
protein_coding	FAM200B	ENSG00000237765.6	116	81	0.7
protein_coding	CD55	ENSG00000196352.15	53	37	0.7
protein_coding	ADAM12	ENSG00000148848.14	106	74	0.7
protein_coding	GTPBP8	ENSG00000163607.15	43	30	0.7
protein_coding	PIK3R3	ENSG00000117461.14	86	60	0.7
protein_coding	FLII	ENSG00000177731.15	175	122	0.7
protein_coding	EMP1	ENSG00000134531.9	1198	835	0.7
protein_coding	BORCS8	ENSG00000254901.7	33	23	0.7
protein_coding	PDRG1	ENSG00000088356.5	33	23	0.7
protein_coding	PPIL2	ENSG00000100023.18	33	23	0.7
protein_coding	TGFB1I1	ENSG00000140682.18	122	85	0.7
protein_coding	FAR1	ENSG00000197601.12	89	62	0.7
protein_coding	MAEA	ENSG00000090316.15	201	140	0.7
protein_coding	TMEM141	ENSG00000244187.7	56	39	0.7
protein_coding	PEPD	ENSG00000124299.13	227	158	0.7
protein_coding	ABI2	ENSG00000138443.15	125	87	0.7
protein_coding	UTP20	ENSG00000120800.4	23	16	0.7
protein_coding	GPAM	ENSG00000119927.13	23	16	0.7
protein_coding	ALMS1	ENSG00000116127.17	23	16	0.7
protein_coding	LPAR6	ENSG00000139679.15	23	16	0.7
protein_coding	ATG4A	ENSG00000101844.17	23	16	0.7
protein_coding	ZNF710	ENSG00000140548.9	23	16	0.7
protein_coding	COX15	ENSG00000014919.12	46	32	0.7
protein_coding	RASSF4	ENSG00000107551.20	46	32	0.7
protein_coding	RASL12	ENSG00000103710.10	46	32	0.7
protein_coding	CYB5A	ENSG00000166347.18	92	64	0.7
protein_coding	NTM	ENSG00000182667.14	118	82	0.7
protein_coding	MED29	ENSG00000063322.13	95	66	0.7
protein_coding	BDKRB1	ENSG00000100739.10	36	25	0.7
protein_coding	EIF4ENIF1	ENSG00000184708.17	36	25	0.7
protein_coding	SLC14A1	ENSG00000141469.17	324	225	0.7
protein_coding	DLC1	ENSG00000164741.14	229	159	0.7
protein_coding	COL6A2	ENSG00000142173.14	1086	754	0.7
protein_coding	AGO1	ENSG00000092847.11	85	59	0.7
protein_coding	OCRL	ENSG00000122126.16	85	59	0.7
protein_coding	TIPARP	ENSG00000163659.12	49	34	0.7
protein_coding	RAB4A	ENSG00000168118.11	98	68	0.7

protein_coding	MXD4	ENSG00000123933.16	297	206	0.7
protein_coding	SNTB2	ENSG00000168807.16	359	249	0.7
protein_coding	UST	ENSG00000111962.7	62	43	0.7
protein_coding	RP9	ENSG00000164610.8	124	86	0.7
protein_coding	AP5M1	ENSG00000053770.11	88	61	0.7
protein_coding	HIBADH	ENSG00000106049.8	176	122	0.7
protein_coding	SMAD3	ENSG00000166949.15	215	149	0.7
protein_coding	METTL2A	ENSG00000087995.15	13	9	0.7
protein_coding	WDHD1	ENSG00000198554.11	13	9	0.7
protein_coding	ZNF266	ENSG00000174652.18	13	9	0.7
protein_coding	ZNF212	ENSG00000170260.8	13	9	0.7
protein_coding	GLI2	ENSG00000074047.21	13	9	0.7
protein_coding	KIN	ENSG00000151657.11	26	18	0.7
protein_coding	RARRES3	ENSG00000133321.10	26	18	0.7
protein_coding	ZNF319	ENSG00000166188.2	26	18	0.7
protein_coding	ARHGAP22	ENSG00000128805.14	39	27	0.7
protein_coding	GEMIN8	ENSG00000046647.13	39	27	0.7
protein_coding	MOV10	ENSG00000155363.18	52	36	0.7
protein_coding	DNAJC19	ENSG00000205981.7	117	81	0.7
protein_coding	RYK	ENSG00000163785.12	130	90	0.7
protein_coding	TMEM138	ENSG00000149483.11	94	65	0.7
protein_coding	LMNA	ENSG00000160789.19	2178	1506	0.7
protein_coding	TBL1XR1	ENSG00000177565.16	505	349	0.7
protein_coding	S1PR2	ENSG00000267534.3	55	38	0.7
protein_coding	RNF38	ENSG00000137075.17	42	29	0.7
protein_coding	GFRA1	ENSG00000151892.14	29	20	0.7
protein_coding	LAGE3	ENSG00000196976.7	29	20	0.7
protein_coding	TUB	ENSG00000166402.8	29	20	0.7
protein_coding	DIP2A	ENSG00000160305.17	58	40	0.7
protein_coding	DNPEP	ENSG00000123992.19	87	60	0.7
protein_coding	LAMA4	ENSG00000112769.18	676	466	0.7
protein_coding	COL13A1	ENSG00000197467.14	45	31	0.7
protein_coding	HAUS2	ENSG00000137814.10	45	31	0.7
protein_coding	BOC	ENSG00000144857.14	45	31	0.7
protein_coding	HMOX2	ENSG00000103415.11	45	31	0.7
protein_coding	PGAP2	ENSG00000148985.19	45	31	0.7
protein_coding	PLEKHA2	ENSG00000169499.14	45	31	0.7
protein_coding	AIP	ENSG00000110711.9	61	42	0.7
protein_coding	SELENON	ENSG00000162430.16	122	84	0.7
protein_coding	SPATA13	ENSG00000182957.15	77	53	0.7
protein_coding	BEND6	ENSG00000151917.17	109	75	0.7
protein_coding	FTH1	ENSG00000167996.15	14266	9815	0.7
protein_coding	NOP16	ENSG00000048162.20	16	11	0.7
protein_coding	THUMPD2	ENSG00000138050.14	16	11	0.7

protein_coding	C8orf58	ENSG00000241852.9	16	11	0.7
protein_coding	GALNT14	ENSG00000158089.14	16	11	0.7
protein_coding	CARF	ENSG00000138380.17	16	11	0.7
antisense	MIR762HG	ENSG00000260083.1	16	11	0.7
protein_coding	ZNF548	ENSG00000188785.11	16	11	0.7
protein_coding	NUBPL	ENSG00000151413.16	32	22	0.7
protein_coding	CINP	ENSG00000100865.14	48	33	0.7
protein_coding	SLC7A11	ENSG00000151012.13	409	281	0.7
protein_coding	ZCCHC7	ENSG00000147905.17	67	46	0.7
protein_coding	ACYP2	ENSG00000170634.12	67	46	0.7
protein_coding	DDX50	ENSG00000107625.12	153	105	0.7
protein_coding	UAP1	ENSG00000117143.13	86	59	0.7
protein_coding	MDM2	ENSG00000135679.23	500	343	0.7
protein_coding	SCMH1	ENSG00000010803.16	35	24	0.7
antisense	NR2F1-AS1	ENSG00000237187.8	70	48	0.7
protein_coding	EXT2	ENSG00000151348.13	197	135	0.7
protein_coding	EMILIN1	ENSG00000138080.13	181	124	0.7
protein_coding	TP53I3	ENSG00000115129.13	165	113	0.7
protein_coding	SESN2	ENSG00000130766.4	149	102	0.7
protein_coding	FBLN2	ENSG00000163520.13	168	115	0.7
protein_coding	CNTN3	ENSG00000113805.8	19	13	0.7
TEC	AC007382.1	ENSG00000279519.1	19	13	0.7
protein_coding	ACACB	ENSG00000076555.15	19	13	0.7
protein_coding	OTUD6B	ENSG00000155100.10	19	13	0.7
protein_coding	RNF112	ENSG00000128482.15	19	13	0.7
protein_coding	EMG1	ENSG00000126749.15	38	26	0.7
protein_coding	SNRPA	ENSG00000077312.8	38	26	0.7
protein_coding	PLEKHO2	ENSG00000241839.9	57	39	0.7
protein_coding	STUB1	ENSG00000103266.10	98	67	0.7
protein_coding	DPF2	ENSG00000133884.9	98	67	0.7
protein_coding	CNP	ENSG00000173786.16	243	166	0.7
protein_coding	Sep-08	ENSG00000164402.13	142	97	0.7
protein_coding	PRCC	ENSG00000143294.14	82	56	0.7
protein_coding	CYSTM1	ENSG00000120306.10	104	71	0.7
protein_coding	GJC1	ENSG00000182963.9	63	43	0.7
protein_coding	MRPS18A	ENSG00000096080.11	22	15	0.7
protein_coding	SNUPN	ENSG00000169371.13	22	15	0.7
protein_coding	ZNF771	ENSG00000179965.11	22	15	0.7
protein_coding	PPP1R12B	ENSG00000077157.21	22	15	0.7
protein_coding	PIAS4	ENSG00000105229.6	22	15	0.7
protein_coding	ZNF229	ENSG00000278318.4	22	15	0.7
protein_coding	CDC42EP1	ENSG00000128283.6	22	15	0.7
protein_coding	GTDC1	ENSG00000121964.14	44	30	0.7
protein_coding	ELAC2	ENSG00000006744.18	69	47	0.7

protein_coding	MIB2	ENSG00000197530.12	47	32	0.7
TEC	Z99129.4	ENSG00000279453.1	47	32	0.7
protein_coding	MAN1C1	ENSG00000117643.14	119	81	0.7
protein_coding	NT5C3B	ENSG00000141698.16	72	49	0.7
protein_coding	NR2C2	ENSG00000177463.15	97	66	0.7
protein_coding	ZNF524	ENSG00000171443.6	25	17	0.7
protein_coding	DCAF4	ENSG00000119599.16	25	17	0.7
lincRNA	TRAM2-AS1	ENSG00000225791.6	25	17	0.7
protein_coding	PCSK7	ENSG00000160613.12	50	34	0.7
protein_coding	TBPL1	ENSG00000028839.9	50	34	0.7
protein_coding	CUTA	ENSG00000112514.15	278	189	0.7
protein_coding	CCT3	ENSG00000163468.14	253	172	0.7
protein_coding	BTN3A3	ENSG00000111801.15	78	53	0.7
protein_coding	ERRFI1	ENSG00000116285.12	53	36	0.7
processed_transcript	OIP5-AS1	ENSG00000247556.6	109	74	0.7
protein_coding	RPL10	ENSG00000147403.16	1043	708	0.7
protein_coding	TNPO2	ENSG00000105576.15	174	118	0.7
protein_coding	TP53INP1	ENSG00000164938.13	146	99	0.7
protein_coding	UCK1	ENSG00000130717.12	59	40	0.7
protein_coding	SPEG	ENSG00000072195.14	31	21	0.7
protein_coding	VTA1	ENSG00000009844.15	127	86	0.7
protein_coding	MED19	ENSG00000156603.17	65	44	0.7
protein_coding	USP5	ENSG00000111667.13	65	44	0.7
protein_coding	GNPDA1	ENSG00000113552.15	195	132	0.7
protein_coding	XPO5	ENSG00000124571.17	34	23	0.7
protein_coding	TTPAL	ENSG00000124120.10	34	23	0.7
protein_coding	BRPF1	ENSG00000156983.15	34	23	0.7
protein_coding	KBTBD4	ENSG00000123444.13	34	23	0.7
protein_coding	MAB21L1	ENSG00000180660.7	34	23	0.7
protein_coding	NUDT19	ENSG00000213965.3	34	23	0.7
protein_coding	SNX27	ENSG00000143376.13	136	92	0.7
protein_coding	MRPL52	ENSG00000172590.18	139	94	0.7
protein_coding	TRIM37	ENSG00000108395.13	71	48	0.7
protein_coding	SLC25A4	ENSG00000151729.10	37	25	0.7
protein_coding	SWI5	ENSG00000175854.11	37	25	0.7
protein_coding	DTYMK	ENSG00000168393.12	37	25	0.7
protein_coding	EPHX1	ENSG00000143819.12	77	52	0.7
protein_coding	WIZ	ENSG00000011451.19	117	79	0.7
protein_coding	NUP37	ENSG00000075188.8	40	27	0.7
protein_coding	MXRA5	ENSG00000101825.7	381	257	0.7
protein_coding	DPH5	ENSG00000117543.20	43	29	0.7
protein_coding	DTNBP1	ENSG00000047579.19	43	29	0.7
protein_coding	EVL	ENSG00000196405.12	46	31	0.7
protein_coding	ANXA2	ENSG00000182718.16	4594	3095	0.7

protein_coding	MED12	ENSG00000184634.15	49	33	0.7
protein_coding	LMF1	ENSG00000103227.18	55	37	0.7
protein_coding	VKORC1	ENSG00000167397.14	385	259	0.7
protein_coding	C19orf53	ENSG00000104979.8	177	119	0.7
protein_coding	SUPT5H	ENSG00000196235.13	125	84	0.7
protein_coding	GNA13	ENSG00000120063.9	67	45	0.7
protein_coding	SSH2	ENSG00000141298.18	70	47	0.7
protein_coding	PARP9	ENSG00000138496.16	73	49	0.7
protein_coding	NUDT16L1	ENSG00000168101.14	73	49	0.7
protein_coding	MRPL24	ENSG00000143314.12	85	57	0.7
protein_coding	SEC24B	ENSG00000138802.11	88	59	0.7
protein_coding	GATAD1	ENSG00000157259.7	91	61	0.7
protein_coding	CTSO	ENSG00000256043.2	185	124	0.7
protein_coding	NFKB1	ENSG00000109320.11	94	63	0.7
protein_coding	TRIM22	ENSG00000132274.15	163	109	0.7
protein_coding	CCL26	ENSG00000006606.8	3	2	0.7
protein_coding	DDX39B	ENSG00000198563.13	3	2	0.7
protein_coding	EPOP	ENSG00000273604.1	3	2	0.7
lincRNA	RALY-AS1	ENSG00000285230.1	3	2	0.7
sense_intronic	ANKRD10-	ENSG00000229152.2	3	2	0.7
misc_RNA	RN7SL37P	ENSG00000242493.3	3	2	0.7
protein_coding	TLR6	ENSG00000174130.12	3	2	0.7
protein_coding	IGSF23	ENSG00000216588.8	3	2	0.7
antisense	AC044802.2	ENSG00000258122.1	3	2	0.7
unprocessed_pseudogene	MTCO1P12	ENSG00000237973.1	3	2	0.7
protein_coding	SYTL3	ENSG00000164674.15	3	2	0.7
lincRNA	AC026979.3	ENSG00000272375.1	3	2	0.7
lincRNA	AC012615.1	ENSG00000261526.2	3	2	0.7
protein_coding	TDRD9	ENSG00000156414.18	3	2	0.7
antisense	AC073896.2	ENSG00000257303.1	3	2	0.7
protein_coding	STK26	ENSG00000134602.15	3	2	0.7
protein_coding	ZNF525	ENSG00000203326.11	3	2	0.7
protein_coding	PKP3	ENSG00000184363.9	3	2	0.7
processed_pseudogene	AC115223.1	ENSG00000243199.1	3	2	0.7
protein_coding	NCALD	ENSG00000104490.17	3	2	0.7
protein_coding	MMP15	ENSG00000102996.4	3	2	0.7
protein_coding	NALCN	ENSG00000102452.16	3	2	0.7
protein_coding	CDC7	ENSG00000097046.12	3	2	0.7
sense_overlapping	AL022311.1	ENSG00000279738.1	3	2	0.7
protein_coding	LMTK3	ENSG00000142235.9	3	2	0.7
protein_coding	GAS7	ENSG00000007237.18	3	2	0.7
transcribed_processed_pseudogene	AP002414.2	ENSG00000256616.3	3	2	0.7

antisense	SMAD5-AS1	ENSG00000164621.5	3	2	0.7
antisense	AC012510.1	ENSG00000273466.1	3	2	0.7
protein_coding	GUCY1A1	ENSG00000164116.16	3	2	0.7
protein_coding	SEMA4D	ENSG00000187764.11	3	2	0.7
protein_coding	ZSCAN20	ENSG00000121903.14	3	2	0.7
protein_coding	ZNF552	ENSG00000178935.5	3	2	0.7
protein_coding	HIST1H2BN	ENSG00000233822.4	3	2	0.7
transcribed_unprocessed_pseudogene	MRPL45P2	ENSG00000228782.7	3	2	0.7
TEC	AP000560.1	ENSG00000278879.1	3	2	0.7
processed_transcript	AC068152.1	ENSG00000262879.5	3	2	0.7
antisense	AC004812.2	ENSG00000277283.1	3	2	0.7
protein_coding	HIST1H3E	ENSG00000274750.2	3	2	0.7
protein_coding	ZNF34	ENSG00000196378.11	3	2	0.7
antisense	IER3-AS1	ENSG00000272273.1	3	2	0.7
protein_coding	ALS2CR12	ENSG00000155749.12	3	2	0.7
lincRNA	FGF14-AS2	ENSG00000272143.1	3	2	0.7
protein_coding	VAT1L	ENSG00000171724.2	3	2	0.7
protein_coding	TMEM200C	ENSG00000206432.4	3	2	0.7
protein_coding	GRIN3B	ENSG00000116032.5	3	2	0.7
protein_coding	CD274	ENSG00000120217.13	3	2	0.7
bidirectional_promoter_lncRNA	AC099343.3	ENSG00000271646.1	3	2	0.7
protein_coding	STAC	ENSG00000144681.10	3	2	0.7
antisense	AC093249.6	ENSG00000261840.2	3	2	0.7
protein_coding	ASB9	ENSG00000102048.15	3	2	0.7
sense_intronic	SOS1-IT1	ENSG00000229692.3	3	2	0.7
protein_coding	DNMT3B	ENSG00000088305.18	3	2	0.7
sense_intronic	AC087481.3	ENSG00000270015.1	3	2	0.7
protein_coding	ZNF619	ENSG00000177873.12	3	2	0.7
protein_coding	CCDC120	ENSG00000147144.12	3	2	0.7
protein_coding	ZNF442	ENSG00000198342.9	3	2	0.7
antisense	ARF4-AS1	ENSG00000272146.5	3	2	0.7
protein_coding	RHBDL1	ENSG00000103269.13	3	2	0.7
protein_coding	SLC25A35	ENSG00000125434.10	3	2	0.7
protein_coding	MLC1	ENSG00000100427.15	3	2	0.7
sense_overlapping	AL592424.1	ENSG00000261168.1	3	2	0.7
antisense	AC092123.1	ENSG00000261118.1	3	2	0.7
processed_pseudogene	RPS15AP17	ENSG00000225405.3	3	2	0.7
lincRNA	AC135048.3	ENSG00000275263.1	3	2	0.7
protein_coding	HHIPL2	ENSG00000143512.12	3	2	0.7
protein_coding	GTF2A1L	ENSG00000242441.7	3	2	0.7
protein_coding	ZNF781	ENSG00000196381.10	3	2	0.7

transcribed_unprocessed_pseudogene	ZNF883	ENSG00000228623.6	3	2	0.7
lincRNA	AC009275.1	ENSG00000273297.1	3	2	0.7
protein_coding	CARD10	ENSG00000100065.14	3	2	0.7
protein_coding	TMEM229B	ENSG00000198133.8	3	2	0.7
processed_pseudogene	AC136632.1	ENSG00000218227.3	3	2	0.7
protein_coding	RNASEK	ENSG00000219200.11	3	2	0.7
TEC	AC096720.2	ENSG00000279464.1	3	2	0.7
lincRNA	AC018638.7	ENSG00000271553.1	3	2	0.7
protein_coding	GADD45G	ENSG00000130222.10	3	2	0.7
antisense	AC072061.1	ENSG00000259826.1	3	2	0.7
processed_pseudogene	RPSAP15	ENSG00000237506.3	3	2	0.7
lincRNA	AL161937.2	ENSG00000237595.3	3	2	0.7
antisense	AC079305.1	ENSG00000222043.2	3	2	0.7
antisense	AL079303.1	ENSG00000258661.1	3	2	0.7
protein_coding	MYBPH	ENSG00000133055.8	3	2	0.7
protein_coding	AGAP9	ENSG00000204172.12	3	2	0.7
protein_coding	MROH6	ENSG00000204839.8	3	2	0.7
protein_coding	TCAF2	ENSG00000170379.20	6	4	0.7
TEC	AC007485.2	ENSG00000279059.1	6	4	0.7
protein_coding	F10	ENSG00000126218.11	6	4	0.7
protein_coding	TPD52L1	ENSG00000111907.20	6	4	0.7
protein_coding	RFC2	ENSG00000049541.10	6	4	0.7
protein_coding	DHFR	ENSG00000228716.6	6	4	0.7
processed_transcript	AC091057.1	ENSG00000187951.11	6	4	0.7
protein_coding	PGBD2	ENSG00000185220.11	6	4	0.7
lincRNA	AC009404.1	ENSG00000236255.1	6	4	0.7
antisense	FLJ37453	ENSG00000179743.4	6	4	0.7
protein_coding	RCOR2	ENSG00000167771.5	6	4	0.7
lincRNA	LINC00997	ENSG00000281332.1	6	4	0.7
protein_coding	TOR2A	ENSG00000160404.17	6	4	0.7
protein_coding	ZNF549	ENSG00000121406.8	6	4	0.7
antisense	AP001160.3	ENSG00000269176.2	6	4	0.7
protein_coding	ZNF660	ENSG00000144792.9	6	4	0.7
protein_coding	ZNF594	ENSG00000180626.9	6	4	0.7
protein_coding	TRIM3	ENSG00000110171.19	6	4	0.7
lincRNA	SNHG15	ENSG00000232956.8	6	4	0.7
protein_coding	ABCB4	ENSG00000005471.17	6	4	0.7
protein_coding	SUPT3H	ENSG00000196284.15	6	4	0.7
lincRNA	FLVCR1-DT	ENSG00000198468.7	6	4	0.7
antisense	AC093827.4	ENSG00000284968.1	6	4	0.7
antisense	AC009812.1	ENSG00000251867.4	6	4	0.7
lincRNA	AC091563.1	ENSG00000254343.2	6	4	0.7

protein_coding	ANKRD18B	ENSG00000230453.9	6	4	0.7
antisense	SGMS1-AS1	ENSG00000226200.6	6	4	0.7
protein_coding	FABP3	ENSG00000121769.7	6	4	0.7
protein_coding	HTR1F	ENSG00000179097.5	6	4	0.7
protein_coding	CTC1	ENSG00000178971.14	6	4	0.7
protein_coding	GGT7	ENSG00000131067.16	6	4	0.7
protein_coding	MOCS1	ENSG00000124615.19	9	6	0.7
protein_coding	PCSK5	ENSG00000099139.13	9	6	0.7
protein_coding	FBXW9	ENSG00000132004.12	9	6	0.7
protein_coding	ANK2	ENSG00000145362.17	9	6	0.7
protein_coding	KLHL23	ENSG00000213160.9	9	6	0.7
protein_coding	BATF3	ENSG00000123685.8	9	6	0.7
protein_coding	POLA2	ENSG00000014138.8	9	6	0.7
protein_coding	NBPF1	ENSG00000219481.10	9	6	0.7
protein_coding	NSUN3	ENSG00000178694.9	9	6	0.7
protein_coding	FOXRED1	ENSG00000110074.10	9	6	0.7
protein_coding	COQ9	ENSG00000088682.13	9	6	0.7
protein_coding	ZNF101	ENSG00000181896.11	9	6	0.7
protein_coding	ARSE	ENSG00000157399.14	9	6	0.7
protein_coding	CMTM8	ENSG00000170293.8	9	6	0.7
protein_coding	ADORA1	ENSG00000163485.16	9	6	0.7
protein_coding	HIC1	ENSG00000177374.12	12	8	0.7
processed_transcript	BACE1-AS	ENSG00000278768.2	12	8	0.7
protein_coding	OAS2	ENSG00000111335.12	12	8	0.7
protein_coding	TMEM251	ENSG00000153485.5	12	8	0.7
protein_coding	REEP2	ENSG00000132563.15	12	8	0.7
protein_coding	LRRC37B	ENSG00000185158.12	12	8	0.7
antisense	NIFK-AS1	ENSG00000236859.6	12	8	0.7
lincRNA	URB1-AS1	ENSG00000256073.3	12	8	0.7
protein_coding	COPG2	ENSG00000158623.14	12	8	0.7
processed_pseudogene	RPS3AP47	ENSG00000205871.5	12	8	0.7
protein_coding	MEIS2	ENSG00000134138.19	12	8	0.7
protein_coding	LRP3	ENSG00000130881.13	12	8	0.7
protein_coding	LRRC20	ENSG00000172731.13	12	8	0.7
protein_coding	PPAT	ENSG00000128059.8	15	10	0.7
protein_coding	CCDC22	ENSG00000101997.12	15	10	0.7
protein_coding	C14orf93	ENSG00000100802.14	15	10	0.7
protein_coding	INCENP	ENSG00000149503.12	15	10	0.7
sense_overlapping	AL596244.1	ENSG00000261534.1	15	10	0.7
protein_coding	TBC1D19	ENSG00000109680.10	15	10	0.7
antisense	LOXL1-AS1	ENSG00000261801.5	18	12	0.7
protein_coding	DXO	ENSG00000204348.9	18	12	0.7
protein_coding	ANKRD44	ENSG00000065413.19	18	12	0.7
protein_coding	ZNF71	ENSG00000197951.9	18	12	0.7

protein_coding	SELENOO	ENSG00000073169.13	18	12	0.7
lincRNA	AC026401.3	ENSG00000280206.1	18	12	0.7
protein_coding	INMT	ENSG00000241644.2	18	12	0.7
protein_coding	MTERF1	ENSG00000127989.13	18	12	0.7
protein_coding	WEE1	ENSG00000166483.10	21	14	0.7
protein_coding	ZNF7	ENSG00000147789.15	21	14	0.7
protein_coding	GRK4	ENSG00000125388.19	21	14	0.7
protein_coding	HHIPL1	ENSG00000182218.9	21	14	0.7
protein_coding	USP20	ENSG00000136878.12	21	14	0.7
protein_coding	WNT9A	ENSG00000143816.7	21	14	0.7
protein_coding	ZNF337	ENSG00000130684.13	24	16	0.7
protein_coding	DUSP23	ENSG00000158716.8	27	18	0.7
protein_coding	E4F1	ENSG00000167967.15	27	18	0.7
protein_coding	TIGD5	ENSG00000179886.5	30	20	0.7
protein_coding	BICRAL	ENSG00000112624.12	30	20	0.7
protein_coding	PIGZ	ENSG00000119227.7	30	20	0.7
transcribed_processed_pseudogene	AC098614.1	ENSG00000213846.5	30	20	0.7
processed_pseudogene	AL022718.1	ENSG00000226532.1	30	20	0.7
protein_coding	STMN3	ENSG00000197457.9	33	22	0.7
protein_coding	RPP25L	ENSG00000164967.9	33	22	0.7
antisense	ZNF436-AS1	ENSG00000249087.6	33	22	0.7
protein_coding	NAPEPLD	ENSG00000161048.11	36	24	0.7
protein_coding	HDAC11	ENSG00000163517.14	36	24	0.7
protein_coding	SLC48A1	ENSG00000211584.13	36	24	0.7
protein_coding	EPHB3	ENSG00000182580.2	36	24	0.7
protein_coding	ADPRHL2	ENSG00000116863.10	39	26	0.7
protein_coding	NIF3L1	ENSG00000196290.14	45	30	0.7
protein_coding	FAM206A	ENSG00000119328.11	48	32	0.7
protein_coding	DHDDS	ENSG00000117682.16	48	32	0.7
protein_coding	CLCA2	ENSG00000137975.7	57	38	0.7
protein_coding	POGK	ENSG00000143157.11	57	38	0.7
protein_coding	MANBAL	ENSG00000101363.12	60	40	0.7
protein_coding	RHNO1	ENSG00000171792.10	63	42	0.7
protein_coding	YY1AP1	ENSG00000163374.19	72	48	0.7
protein_coding	N4BP1	ENSG00000102921.7	93	62	0.7
protein_coding	DTD1	ENSG00000125821.12	105	70	0.7
protein_coding	BBC3	ENSG00000105327.17	244	162	0.7
protein_coding	ARSJ	ENSG00000180801.13	119	79	0.7
protein_coding	PKM	ENSG00000067225.17	3822	2537	0.7
protein_coding	SIX5	ENSG00000177045.8	107	71	0.7
protein_coding	VPS26C	ENSG00000157538.13	104	69	0.7
protein_coding	STON1	ENSG00000243244.6	101	67	0.7

protein_coding	DNPH1	ENSG00000112667.12	74	49	0.7
protein_coding	UBE2F	ENSG00000184182.18	142	94	0.7
protein_coding	LPCAT2	ENSG00000087253.12	65	43	0.7
protein_coding	RASAL2	ENSG00000075391.16	65	43	0.7
protein_coding	FKTN	ENSG00000106692.14	59	39	0.7
protein_coding	INSIG1	ENSG00000186480.12	53	35	0.7
protein_coding	MRPS2	ENSG00000122140.10	53	35	0.7
protein_coding	C6orf120	ENSG00000185127.6	53	35	0.7
protein_coding	MAPK14	ENSG00000112062.10	106	70	0.7
protein_coding	RNPEPL1	ENSG00000142327.12	50	33	0.7
protein_coding	TIAM2	ENSG00000146426.18	50	33	0.7
protein_coding	DGKZ	ENSG00000149091.15	47	31	0.7
protein_coding	MSRB1	ENSG00000198736.11	47	31	0.7
protein_coding	XPOT	ENSG00000184575.11	220	145	0.7
protein_coding	ZNHIT3	ENSG00000273611.4	85	56	0.7
protein_coding	JDP2	ENSG00000140044.12	126	83	0.7
protein_coding	VSIR	ENSG00000107738.19	41	27	0.7
protein_coding	NUPL2	ENSG00000136243.16	41	27	0.7
protein_coding	DCAKD	ENSG00000172992.11	41	27	0.7
protein_coding	NDUFA10	ENSG00000130414.11	120	79	0.7
protein_coding	H2AFX	ENSG00000188486.3	38	25	0.7
protein_coding	VEGFB	ENSG00000173511.9	190	125	0.7
protein_coding	CCDC86	ENSG00000110104.12	35	23	0.7
protein_coding	TAF1C	ENSG00000103168.16	35	23	0.7
protein_coding	JADE2	ENSG00000043143.20	32	21	0.7
protein_coding	RNF5	ENSG00000204308.7	32	21	0.7
protein_coding	DOP1A	ENSG00000083097.14	32	21	0.7
protein_coding	XPO7	ENSG00000130227.16	151	99	0.7
protein_coding	CAB39L	ENSG00000102547.18	29	19	0.7
protein_coding	ABCC3	ENSG00000108846.15	29	19	0.7
protein_coding	SNX21	ENSG00000124104.18	87	57	0.7
protein_coding	DCTD	ENSG00000129187.14	174	114	0.7
protein_coding	ZCCHC24	ENSG00000165424.6	687	450	0.7
protein_coding	ASAP1	ENSG00000153317.14	571	374	0.7
protein_coding	TPM1	ENSG00000140416.20	527	345	0.7
protein_coding	NUP43	ENSG00000120253.13	55	36	0.7
protein_coding	MAP4K5	ENSG00000012983.11	301	197	0.7
protein_coding	STAT5B	ENSG00000173757.9	26	17	0.7
protein_coding	LIFR	ENSG00000113594.9	26	17	0.7
protein_coding	ZNF32	ENSG00000169740.13	104	68	0.7
protein_coding	TAF1D	ENSG00000166012.16	312	204	0.7
protein_coding	RGS10	ENSG00000148908.14	179	117	0.7
protein_coding	F2R	ENSG00000181104.6	447	292	0.7
protein_coding	LATS1	ENSG00000131023.12	72	47	0.7

protein_coding	DUSP14	ENSG00000276023.4	141	92	0.7
protein_coding	PSPH	ENSG00000146733.13	23	15	0.7
lincRNA	AP003486.1	ENSG00000255455.2	23	15	0.7
protein_coding	STRN4	ENSG00000090372.14	23	15	0.7
protein_coding	DENND6A	ENSG00000174839.12	23	15	0.7
protein_coding	ZNF282	ENSG00000170265.11	46	30	0.7
protein_coding	IL11RA	ENSG00000137070.17	115	75	0.7
protein_coding	GSN	ENSG00000148180.19	1382	901	0.7
protein_coding	MPP6	ENSG00000105926.15	43	28	0.7
protein_coding	CHP1	ENSG00000187446.11	381	248	0.7
protein_coding	SLIT2	ENSG00000145147.19	63	41	0.7
lincRNA	SNHG8	ENSG00000269893.6	458	298	0.7
protein_coding	ITGA3	ENSG00000005884.17	83	54	0.7
protein_coding	SERPINI1	ENSG00000163536.12	20	13	0.7
protein_coding	R3HCC1L	ENSG00000166024.13	20	13	0.7
protein_coding	HSPA14	ENSG00000187522.15	20	13	0.7
protein_coding	GTPBP3	ENSG00000130299.16	20	13	0.7
protein_coding	CLCN6	ENSG00000011021.22	20	13	0.7
protein_coding	NPM3	ENSG00000107833.10	40	26	0.7
protein_coding	ZNF587	ENSG00000198466.11	40	26	0.7
protein_coding	MTAP	ENSG00000099810.19	80	52	0.7
protein_coding	TMEM64	ENSG00000180694.13	80	52	0.7
protein_coding	ENOPH1	ENSG00000145293.15	77	50	0.6
protein_coding	FZD4	ENSG00000174804.3	77	50	0.6
protein_coding	ATG101	ENSG00000123395.14	57	37	0.6
protein_coding	UBE2E2	ENSG00000182247.9	57	37	0.6
protein_coding	MRPL48	ENSG00000175581.13	57	37	0.6
protein_coding	VIPAS39	ENSG00000151445.15	37	24	0.6
protein_coding	KCNS3	ENSG00000170745.11	37	24	0.6
protein_coding	SLC15A4	ENSG00000139370.11	91	59	0.6
protein_coding	EPB41	ENSG00000159023.21	88	57	0.6
protein_coding	CYR61	ENSG00000142871.16	156	101	0.6
protein_coding	TRAPPC10	ENSG00000160218.12	17	11	0.6
protein_coding	MICB	ENSG00000204516.9	17	11	0.6
protein_coding	OSGEP	ENSG00000092094.10	17	11	0.6
protein_coding	C12orf43	ENSG00000157895.11	34	22	0.6
protein_coding	DBP	ENSG00000105516.10	34	22	0.6
protein_coding	PLSCR4	ENSG00000114698.14	116	75	0.6
protein_coding	CROT	ENSG00000005469.11	178	115	0.6
protein_coding	DOCK10	ENSG00000135905.19	79	51	0.6
protein_coding	TLDC1	ENSG00000140950.15	31	20	0.6
protein_coding	CCDC71	ENSG00000177352.9	31	20	0.6
protein_coding	MAPK7	ENSG00000166484.19	62	40	0.6
protein_coding	GGPS1	ENSG00000152904.11	93	60	0.6

protein_coding	RTCB	ENSG00000100220.11	155	100	0.6
protein_coding	RBM43	ENSG00000184898.6	107	69	0.6
protein_coding	MOSPD3	ENSG00000106330.11	76	49	0.6
protein_coding	CIC	ENSG00000079432.7	45	29	0.6
protein_coding	CAMK1	ENSG00000134072.10	45	29	0.6
protein_coding	SDC4	ENSG00000124145.6	357	230	0.6
protein_coding	NMRK1	ENSG00000106733.20	73	47	0.6
protein_coding	ZDHHC18	ENSG00000204160.11	14	9	0.6
protein_coding	PLPP4	ENSG00000203805.10	14	9	0.6
protein_coding	CNTRL	ENSG00000119397.16	14	9	0.6
protein_coding	PRDM10	ENSG00000170325.14	14	9	0.6
protein_coding	MARCH4	ENSG00000144583.4	14	9	0.6
protein_coding	YBEY	ENSG00000182362.13	14	9	0.6
protein_coding	P2RX7	ENSG00000089041.16	14	9	0.6
protein_coding	TMEM225B	ENSG00000244219.6	14	9	0.6
lincRNA	AL355338.1	ENSG00000274605.1	14	9	0.6
protein_coding	MAML3	ENSG00000196782.12	28	18	0.6
protein_coding	PTTG1	ENSG00000164611.12	28	18	0.6
protein_coding	ZNF182	ENSG00000147118.11	28	18	0.6
protein_coding	ORC3	ENSG00000135336.14	28	18	0.6
protein_coding	TRAP1	ENSG00000126602.10	28	18	0.6
protein_coding	CHIC2	ENSG00000109220.10	42	27	0.6
protein_coding	TIMM50	ENSG00000105197.10	42	27	0.6
protein_coding	MSRA	ENSG00000175806.14	70	45	0.6
protein_coding	JCAD	ENSG00000165757.8	151	97	0.6
protein_coding	PAIP1	ENSG00000172239.13	67	43	0.6
protein_coding	SNAP47	ENSG00000143740.14	67	43	0.6
protein_coding	SDC1	ENSG00000115884.10	53	34	0.6
protein_coding	PDGFRB	ENSG00000113721.13	1520	975	0.6
protein_coding	CLTB	ENSG00000175416.12	117	75	0.6
protein_coding	PSMD6	ENSG00000163636.10	142	91	0.6
protein_coding	DCTN1	ENSG00000204843.12	167	107	0.6
protein_coding	PITHD1	ENSG00000057757.9	139	89	0.6
protein_coding	ATP5S	ENSG00000125375.14	25	16	0.6
protein_coding	RAB2B	ENSG00000129472.14	75	48	0.6
protein_coding	TMEM135	ENSG00000166575.16	75	48	0.6
protein_coding	SERPINF1	ENSG00000132386.10	300	192	0.6
protein_coding	ZNF275	ENSG00000063587.14	36	23	0.6
protein_coding	EPHB2	ENSG00000133216.16	94	60	0.6
protein_coding	OGG1	ENSG00000114026.21	69	44	0.6
protein_coding	MIER2	ENSG00000105556.11	11	7	0.6
processed_pseudogene	RPL34P18	ENSG00000240509.1	11	7	0.6
protein_coding	MFSD9	ENSG00000135953.10	11	7	0.6
processed_pseudogene	RPS23P8	ENSG00000230629.2	11	7	0.6

antisense	AC107375.1	ENSG00000259891.1	11	7	0.6
protein_coding	CDPF1	ENSG00000205643.10	11	7	0.6
protein_coding	PIDD1	ENSG00000177595.17	11	7	0.6
protein_coding	TRIM45	ENSG00000134253.9	11	7	0.6
protein_coding	PPM1L	ENSG00000163590.13	11	7	0.6
protein_coding	PRX	ENSG00000105227.14	11	7	0.6
protein_coding	EPHB6	ENSG00000106123.11	11	7	0.6
protein_coding	ASTE1	ENSG00000034533.11	11	7	0.6
protein_coding	KLF16	ENSG00000129911.8	22	14	0.6
protein_coding	PI4K2B	ENSG00000038210.12	22	14	0.6
protein_coding	PLCB3	ENSG00000149782.11	22	14	0.6
protein_coding	R3HDM4	ENSG00000198858.9	22	14	0.6
protein_coding	TTI2	ENSG00000129696.12	22	14	0.6
protein_coding	DOLPP1	ENSG00000167130.17	33	21	0.6
protein_coding	DDX60	ENSG00000137628.16	33	21	0.6
protein_coding	EZH1	ENSG00000108799.12	33	21	0.6
protein_coding	PHETA2	ENSG00000177096.8	44	28	0.6
protein_coding	FAM220A	ENSG00000178397.12	55	35	0.6
protein_coding	VPS41	ENSG00000006715.15	154	98	0.6
protein_coding	ABCA1	ENSG00000165029.15	703	447	0.6
protein_coding	ARHGAP23	ENSG00000275832.4	162	103	0.6
protein_coding	PPP2R3A	ENSG00000073711.10	148	94	0.6
protein_coding	ZBTB2	ENSG00000181472.4	41	26	0.6
protein_coding	ZFYVE1	ENSG00000165861.13	82	52	0.6
protein_coding	CCDC92	ENSG00000119242.8	325	206	0.6
protein_coding	C8orf82	ENSG00000213563.6	71	45	0.6
protein_coding	UROD	ENSG00000126088.13	161	102	0.6
protein_coding	PFKFB3	ENSG00000170525.20	251	159	0.6
protein_coding	FCHSD1	ENSG00000197948.10	30	19	0.6
antisense	DNAH10OS	ENSG00000250091.3	30	19	0.6
protein_coding	JUN	ENSG00000177606.6	270	171	0.6
protein_coding	RAB32	ENSG00000118508.4	49	31	0.6
protein_coding	Sep-09	ENSG00000184640.17	1045	661	0.6
protein_coding	RPL26L1	ENSG00000037241.7	68	43	0.6
protein_coding	PML	ENSG00000140464.19	106	67	0.6
protein_coding	TMEM54	ENSG00000121900.18	19	12	0.6
protein_coding	GAREM1	ENSG00000141441.15	19	12	0.6
protein_coding	CLSTN2	ENSG00000158258.16	19	12	0.6
protein_coding	ABHD18	ENSG00000164074.15	19	12	0.6
protein_coding	CEP250	ENSG00000126001.15	19	12	0.6
protein_coding	STRIP2	ENSG00000128578.9	19	12	0.6
protein_coding	XAF1	ENSG00000132530.16	76	48	0.6
protein_coding	CBFB	ENSG00000067955.13	114	72	0.6
lincRNA	SNHG18	ENSG00000250786.1	114	72	0.6

protein_coding	MRGBP	ENSG00000101189.6	46	29	0.6
protein_coding	MTMR10	ENSG00000166912.16	46	29	0.6
protein_coding	C1orf123	ENSG00000162384.13	119	75	0.6
protein_coding	SSBP4	ENSG00000130511.15	73	46	0.6
protein_coding	MTHFD2	ENSG00000065911.11	200	126	0.6
protein_coding	LLGL1	ENSG00000131899.10	208	131	0.6
protein_coding	APCDD1L	ENSG00000198768.10	27	17	0.6
protein_coding	APOBEC3G	ENSG00000239713.8	27	17	0.6
protein_coding	CNN1	ENSG00000130176.7	27	17	0.6
protein_coding	FLYWCH2	ENSG00000162076.12	27	17	0.6
protein_coding	SLC5A3	ENSG00000198743.6	135	85	0.6
protein_coding	CPNE1	ENSG00000214078.12	259	163	0.6
protein_coding	MTHFD1	ENSG00000100714.15	62	39	0.6
antisense	NNT-AS1	ENSG00000248092.7	132	83	0.6
protein_coding	EPB41L5	ENSG00000115109.13	35	22	0.6
protein_coding	CAMSAP1	ENSG00000130559.18	43	27	0.6
antisense	THAP9-AS1	ENSG00000251022.6	43	27	0.6
protein_coding	IPP	ENSG00000197429.10	43	27	0.6
protein_coding	CDKN2A	ENSG00000147889.17	161	101	0.6
protein_coding	ACTG1	ENSG00000184009.11	7525	4720	0.6
protein_coding	POU2F2	ENSG00000028277.21	67	42	0.6
protein_coding	AKR1B1	ENSG00000085662.13	1205	755	0.6
protein_coding	RHBDF1	ENSG00000007384.15	187	117	0.6
protein_coding	ZNF100	ENSG00000197020.10	8	5	0.6
antisense	AC002074.1	ENSG00000285090.1	8	5	0.6
protein_coding	ERV3-1	ENSG00000213462.4	8	5	0.6
protein_coding	TBX19	ENSG00000143178.12	8	5	0.6
protein_coding	REXO1	ENSG00000079313.14	8	5	0.6
protein_coding	TTC30B	ENSG00000196659.9	8	5	0.6
protein_coding	MPV17L2	ENSG00000254858.9	8	5	0.6
protein_coding	ZNF865	ENSG00000261221.3	8	5	0.6
processed_pseudogene	AL079342.1	ENSG00000220130.1	8	5	0.6
protein_coding	LYL1	ENSG00000104903.4	8	5	0.6
protein_coding	WDR63	ENSG00000162643.12	8	5	0.6
antisense	AC137630.3	ENSG00000272434.1	8	5	0.6
antisense	SNAP25-AS1	ENSG00000227906.7	8	5	0.6
protein_coding	CCDC9B	ENSG00000188549.12	8	5	0.6
protein_coding	HIST1H2BG	ENSG00000273802.2	8	5	0.6
protein_coding	CABCOCO1	ENSG00000183346.7	8	5	0.6
processed_transcript	ZFPM2-AS1	ENSG00000251003.8	16	10	0.6
protein_coding	DHX58	ENSG00000108771.12	16	10	0.6
lincRNA	LINC01444	ENSG00000264301.1	16	10	0.6
protein_coding	DNASE1L1	ENSG00000013563.13	24	15	0.6
protein_coding	GRB10	ENSG00000106070.18	24	15	0.6

transcribed_processed_pseudogene	RPL9P9	ENSG00000237550.5	24	15	0.6
protein_coding	PDCD2L	ENSG00000126249.7	24	15	0.6
protein_coding	IKZF5	ENSG00000095574.11	32	20	0.6
protein_coding	DTD2	ENSG00000129480.12	40	25	0.6
protein_coding	VPS52	ENSG00000223501.8	40	25	0.6
protein_coding	NSD2	ENSG00000109685.17	48	30	0.6
protein_coding	TP53	ENSG00000141510.17	56	35	0.6
protein_coding	RHBDD1	ENSG00000144468.16	56	35	0.6
lincRNA	SNHG19	ENSG00000260260.1	80	50	0.6
protein_coding	SUMF2	ENSG00000129103.17	128	80	0.6
protein_coding	AFAP1	ENSG00000196526.10	125	78	0.6
polymorphic_pseudogene	GPX1	ENSG00000233276.4	359	224	0.6
protein_coding	MRGPRF	ENSG00000172935.8	202	126	0.6
protein_coding	TNC	ENSG00000041982.15	162	101	0.6
protein_coding	UBE2L6	ENSG00000156587.15	138	86	0.6
protein_coding	GJA1	ENSG00000152661.8	1199	747	0.6
protein_coding	CTHRC1	ENSG00000164932.12	575	358	0.6
protein_coding	DLST	ENSG00000119689.14	98	61	0.6
protein_coding	GBE1	ENSG00000114480.12	331	206	0.6
protein_coding	CLN8	ENSG00000182372.9	45	28	0.6
protein_coding	TMEM69	ENSG00000159596.6	45	28	0.6
protein_coding	RAB34	ENSG00000109113.19	267	166	0.6
protein_coding	NUDT1	ENSG00000106268.15	37	23	0.6
protein_coding	HRAS	ENSG00000174775.16	66	41	0.6
protein_coding	ATP8B1	ENSG00000081923.13	95	59	0.6
protein_coding	COG2	ENSG00000135775.13	29	18	0.6
protein_coding	TMEM119	ENSG00000183160.8	759	471	0.6
protein_coding	RPS6KB2	ENSG00000175634.14	50	31	0.6
protein_coding	UTP14A	ENSG00000156697.12	50	31	0.6
protein_coding	APOBEC3C	ENSG00000244509.3	365	226	0.6
antisense	SNHG10	ENSG00000247092.6	21	13	0.6
protein_coding	ILVBL	ENSG00000105135.15	42	26	0.6
protein_coding	LGALS3	ENSG00000131981.15	624	386	0.6
protein_coding	MLH1	ENSG00000076242.14	34	21	0.6
protein_coding	SREBF1	ENSG00000072310.16	81	50	0.6
protein_coding	HMGCS1	ENSG00000112972.14	47	29	0.6
protein_coding	POLI	ENSG00000101751.10	47	29	0.6
protein_coding	SLC9A1	ENSG00000090020.10	94	58	0.6
protein_coding	TCF7L1	ENSG00000152284.4	107	66	0.6
protein_coding	EXOSC6	ENSG00000223496.2	133	82	0.6
protein_coding	VIM	ENSG00000026025.15	66980	41249	0.6
protein_coding	LIX1L	ENSG00000271601.3	190	117	0.6

protein_coding	TET3	ENSG00000187605.15	13	8	0.6
protein_coding	SYNGAP1	ENSG00000197283.16	13	8	0.6
protein_coding	RAB27B	ENSG00000041353.9	13	8	0.6
protein_coding	ZNF284	ENSG00000186026.6	13	8	0.6
protein_coding	RPUSD2	ENSG00000166133.17	13	8	0.6
lincRNA	AC104695.2	ENSG00000270210.1	13	8	0.6
protein_coding	POLRMT	ENSG00000099821.13	13	8	0.6
protein_coding	JADE3	ENSG00000102221.13	13	8	0.6
bidirectional_promoter_lncRNA	AP001372.2	ENSG00000254837.2	13	8	0.6
protein_coding	CUBN	ENSG00000107611.15	26	16	0.6
protein_coding	DCAF17	ENSG00000115827.13	26	16	0.6
protein_coding	PINX1	ENSG00000254093.8	26	16	0.6
protein_coding	RPUSD3	ENSG00000156990.14	39	24	0.6
protein_coding	CRAMP1	ENSG00000007545.15	39	24	0.6
protein_coding	ZNF436	ENSG00000125945.14	39	24	0.6
protein_coding	TRAPPC13	ENSG00000113597.17	52	32	0.6
protein_coding	HSBP1L1	ENSG00000226742.3	78	48	0.6
protein_coding	THG1L	ENSG00000113272.13	70	43	0.6
protein_coding	TKT	ENSG00000163931.15	604	371	0.6
protein_coding	ERAP2	ENSG00000164308.16	127	78	0.6
protein_coding	SOX12	ENSG00000177732.8	145	89	0.6
protein_coding	DEDD2	ENSG00000160570.13	44	27	0.6
protein_coding	TMEM170A	ENSG00000166822.12	31	19	0.6
protein_coding	FUT8	ENSG00000033170.16	129	79	0.6
protein_coding	MCCC1	ENSG00000078070.12	49	30	0.6
protein_coding	SCN1B	ENSG00000105711.11	49	30	0.6
protein_coding	GYG1	ENSG00000163754.17	134	82	0.6
protein_coding	S100A10	ENSG00000197747.8	1760	1077	0.6
protein_coding	APOL1	ENSG00000100342.20	85	52	0.6
protein_coding	RNH1	ENSG00000023191.16	548	335	0.6
protein_coding	ADAMTSL5	ENSG00000185761.10	18	11	0.6
protein_coding	PCYT2	ENSG00000185813.10	18	11	0.6
lincRNA	LINC00702	ENSG00000233117.2	18	11	0.6
protein_coding	VCPKMT	ENSG00000100483.13	18	11	0.6
protein_coding	C2	ENSG00000166278.14	18	11	0.6
protein_coding	VPS33B	ENSG00000184056.14	18	11	0.6
protein_coding	MDGA1	ENSG00000112139.15	18	11	0.6
protein_coding	ATP8B3	ENSG00000130270.16	18	11	0.6
lincRNA	AL021368.2	ENSG00000272316.1	18	11	0.6
protein_coding	PPP3CC	ENSG00000120910.14	36	22	0.6
protein_coding	TMEM220	ENSG00000187824.8	36	22	0.6
protein_coding	MORC2	ENSG00000133422.12	54	33	0.6

protein_coding	ERMAP	ENSG00000164010.14	54	33	0.6
processed_transcript	GAS5	ENSG00000234741.7	324	198	0.6
protein_coding	DHCR7	ENSG00000172893.15	41	25	0.6
protein_coding	COG7	ENSG00000168434.12	23	14	0.6
protein_coding	EN1	ENSG00000163064.6	23	14	0.6
protein_coding	ACBD4	ENSG00000181513.14	23	14	0.6
protein_coding	MIGA2	ENSG00000148343.18	23	14	0.6
protein_coding	CAND2	ENSG00000144712.12	23	14	0.6
protein_coding	WISP2	ENSG00000064205.10	97	59	0.6
protein_coding	MRPS16	ENSG00000182180.13	171	104	0.6
protein_coding	DDX23	ENSG00000174243.9	74	45	0.6
protein_coding	ZNF430	ENSG00000118620.12	28	17	0.6
protein_coding	MAP1S	ENSG00000130479.10	28	17	0.6
protein_coding	SH3GLB2	ENSG00000148341.17	84	51	0.6
protein_coding	PCNX2	ENSG00000135749.18	61	37	0.6
protein_coding	BCL2L13	ENSG00000099968.17	226	137	0.6
protein_coding	PCK2	ENSG00000100889.11	66	40	0.6
protein_coding	FZD2	ENSG00000180340.6	165	100	0.6
protein_coding	MARCKS	ENSG00000277443.2	2095	1269	0.6
protein_coding	ANXA4	ENSG00000196975.15	421	255	0.6
protein_coding	DESI1	ENSG00000100418.7	71	43	0.6
protein_coding	DYRK4	ENSG00000010219.13	71	43	0.6
protein_coding	VPS26B	ENSG00000151502.10	109	66	0.6
protein_coding	NAA16	ENSG00000172766.18	38	23	0.6
protein_coding	CNN3	ENSG00000117519.15	927	561	0.6
protein_coding	R3HCC1	ENSG00000104679.10	43	26	0.6
protein_coding	EXOSC8	ENSG00000120699.12	48	29	0.6
protein_coding	PIP4K2B	ENSG00000276293.4	149	90	0.6
protein_coding	DDAH1	ENSG00000153904.19	207	125	0.6
protein_coding	GAR1	ENSG00000109534.16	53	32	0.6
protein_coding	CLASP2	ENSG00000163539.16	53	32	0.6
protein_coding	FIS1	ENSG00000214253.8	116	70	0.6
protein_coding	CKLF	ENSG00000217555.12	73	44	0.6
protein_coding	PDZRN3	ENSG00000121440.14	219	132	0.6
protein_coding	TRAK1	ENSG00000182606.14	93	56	0.6
protein_coding	KANK2	ENSG00000197256.10	464	279	0.6
protein_coding	CAMK2D	ENSG00000145349.16	253	152	0.6
protein_coding	PLXDC2	ENSG00000120594.16	5	3	0.6
protein_coding	USP31	ENSG00000103404.14	5	3	0.6
protein_coding	CDC25A	ENSG00000164045.11	5	3	0.6
lincRNA	AP001362.2	ENSG00000285533.1	5	3	0.6
protein_coding	RRM2	ENSG00000171848.14	5	3	0.6
lincRNA	AC018645.2	ENSG00000273014.1	5	3	0.6
antisense	MALINC1	ENSG00000245146.6	5	3	0.6

protein_coding	DAZL	ENSG00000092345.13	5	3	0.6
protein_coding	CDC6	ENSG00000094804.10	5	3	0.6
protein_coding	TIMM23B	ENSG00000204152.10	5	3	0.6
protein_coding	ZNF211	ENSG00000121417.13	5	3	0.6
protein_coding	SHCBP1	ENSG00000171241.8	5	3	0.6
protein_coding	NBPF15	ENSG00000266338.6	5	3	0.6
protein_coding	POLN	ENSG00000130997.16	5	3	0.6
processed_pseudogene	FTH1P16	ENSG00000227376.1	5	3	0.6
processed_pseudogene	RPS2P7	ENSG00000235508.3	5	3	0.6
protein_coding	UBXN11	ENSG00000158062.20	5	3	0.6
protein_coding	METAP1D	ENSG00000172878.13	5	3	0.6
protein_coding	CDON	ENSG00000064309.14	5	3	0.6
protein_coding	NACA2	ENSG00000253506.2	5	3	0.6
processed_pseudogene	AC024940.2	ENSG00000243517.1	5	3	0.6
protein_coding	HCLS1	ENSG00000180353.10	5	3	0.6
lincRNA	AC060766.4	ENSG00000267547.1	5	3	0.6
protein_coding	FZD3	ENSG00000104290.10	5	3	0.6
antisense	AC092140.2	ENSG00000274031.1	5	3	0.6
protein_coding	SMIM1	ENSG00000235169.8	5	3	0.6
sense_intronic	AC026124.1	ENSG00000250280.2	5	3	0.6
lincRNA	LINC00856	ENSG00000230417.11	5	3	0.6
protein_coding	ZNF132	ENSG00000131849.11	5	3	0.6
protein_coding	C7orf43	ENSG00000146826.16	10	6	0.6
protein_coding	POLH	ENSG00000170734.11	10	6	0.6
protein_coding	FANCG	ENSG00000221829.9	10	6	0.6
protein_coding	AKAP7	ENSG00000118507.16	10	6	0.6
protein_coding	ZNF225	ENSG00000256294.7	10	6	0.6
protein_coding	RBM38	ENSG00000132819.16	10	6	0.6
protein_coding	NDST2	ENSG00000166507.17	10	6	0.6
protein_coding	HDHD3	ENSG00000119431.9	10	6	0.6
antisense	AL353622.1	ENSG00000270605.1	10	6	0.6
protein_coding	TMEM155	ENSG00000164112.13	10	6	0.6
protein_coding	UBE2T	ENSG00000077152.10	15	9	0.6
protein_coding	OMA1	ENSG00000162600.11	15	9	0.6
protein_coding	SLC9A5	ENSG00000135740.16	15	9	0.6
protein_coding	GNAL	ENSG00000141404.15	15	9	0.6
protein_coding	RINL	ENSG00000187994.13	15	9	0.6
protein_coding	TASP1	ENSG00000089123.15	15	9	0.6
lincRNA	AP002884.1	ENSG00000250303.3	15	9	0.6
protein_coding	URGCP	ENSG00000106608.16	20	12	0.6
protein_coding	DNAJC27	ENSG00000115137.11	20	12	0.6
protein_coding	TMEM126A	ENSG00000171202.6	30	18	0.6
protein_coding	NPRL2	ENSG00000114388.12	30	18	0.6
protein_coding	TUBGCP5	ENSG00000275835.4	35	21	0.6

protein_coding	WDR34	ENSG00000119333.11	35	21	0.6
protein_coding	SCRIB	ENSG00000180900.18	45	27	0.6
protein_coding	ARV1	ENSG00000173409.13	55	33	0.6
protein_coding	EMD	ENSG00000102119.10	110	66	0.6
protein_coding	SARS	ENSG00000031698.12	220	132	0.6
protein_coding	COL1A1	ENSG00000108821.13	38804	23244	0.6
protein_coding	RRAS	ENSG00000126458.3	187	112	0.6
protein_coding	LARP6	ENSG00000166173.10	289	173	0.6
protein_coding	CIZ1	ENSG00000148337.20	226	135	0.6
protein_coding	PPP2R1A	ENSG00000105568.17	201	120	0.6
protein_coding	LSM¬†2	ENSG00000204392.10	52	31	0.6
protein_coding	ST8SIA1	ENSG00000111728.10	99	59	0.6
protein_coding	FMN2	ENSG00000155816.19	173	103	0.6
protein_coding	TP53I11	ENSG00000175274.18	341	203	0.6
protein_coding	ALDH1L2	ENSG00000136010.13	84	50	0.6
protein_coding	GMFG	ENSG00000130755.12	37	22	0.6
protein_coding	ST3GAL5	ENSG00000115525.17	37	22	0.6
lincRNA	AC005261.3	ENSG00000268713.1	37	22	0.6
protein_coding	SLFN11	ENSG00000172716.16	32	19	0.6
protein_coding	IGDCC4	ENSG00000103742.11	32	19	0.6
protein_coding	GLI3	ENSG00000106571.13	91	54	0.6
protein_coding	AMOT	ENSG00000126016.15	118	70	0.6
protein_coding	LRRC28	ENSG00000168904.14	27	16	0.6
protein_coding	TANGO2	ENSG00000183597.15	27	16	0.6
protein_coding	ZNF415	ENSG00000170954.11	27	16	0.6
protein_coding	NSMCE1	ENSG00000169189.16	54	32	0.6
protein_coding	NTPCR	ENSG00000135778.11	115	68	0.6
protein_coding	DDB2	ENSG00000134574.11	137	81	0.6
protein_coding	PCLAF	ENSG00000166803.12	22	13	0.6
protein_coding	GHDC	ENSG00000167925.15	22	13	0.6
protein_coding	CCDC51	ENSG00000164051.13	22	13	0.6
protein_coding	RPTOR	ENSG00000141564.14	22	13	0.6
TEC	AC015813.6	ENSG00000279207.1	22	13	0.6
protein_coding	SERTAD2	ENSG00000179833.4	166	98	0.6
protein_coding	SIAH1	ENSG00000196470.11	39	23	0.6
protein_coding	SAAL1	ENSG00000166788.9	39	23	0.6
protein_coding	CDH6	ENSG00000113361.12	486	286	0.6
protein_coding	RBM45	ENSG00000155636.14	17	10	0.6
protein_coding	DOCK6	ENSG00000130158.13	17	10	0.6
protein_coding	CYBA	ENSG00000051523.10	17	10	0.6
TEC	AC115618.3	ENSG00000279528.1	17	10	0.6
protein_coding	PRADC1	ENSG00000135617.3	34	20	0.6
protein_coding	PLCD1	ENSG00000187091.13	34	20	0.6
protein_coding	PYGL	ENSG00000100504.16	51	30	0.6

protein_coding	PXK	ENSG00000168297.15	165	97	0.6
protein_coding	SLC38A6	ENSG00000139974.15	97	57	0.6
protein_coding	ARHGAP1	ENSG00000175220.11	194	114	0.6
protein_coding	JAG1	ENSG00000101384.12	80	47	0.6
protein_coding	MTA3	ENSG00000057935.13	80	47	0.6
protein_coding	PRR16	ENSG00000184838.14	109	64	0.6
protein_coding	MMP19	ENSG00000123342.15	46	27	0.6
protein_coding	SAMD9L	ENSG00000177409.11	75	44	0.6
protein_coding	KDM4A	ENSG00000066135.12	133	78	0.6
protein_coding	SNAPC5	ENSG00000174446.12	29	17	0.6
antisense	AC007920.2	ENSG00000283175.1	29	17	0.6
processed_transcript	PAX8-AS1	ENSG00000189223.14	94	55	0.6
protein_coding	GCNT1	ENSG00000187210.13	77	45	0.6
protein_coding	SERPINH1	ENSG00000149257.14	1049	613	0.6
protein_coding	SP140L	ENSG00000185404.16	89	52	0.6
protein_coding	GXYLT2	ENSG00000172986.12	89	52	0.6
protein_coding	AKR1C1	ENSG00000187134.13	1296	757	0.6
protein_coding	PTK7	ENSG00000112655.15	339	198	0.6
protein_coding	UHRF1BP1	ENSG00000065060.16	12	7	0.6
protein_coding	HINFP	ENSG00000172273.12	12	7	0.6
protein_coding	KIAA1958	ENSG00000165185.14	12	7	0.6
protein_coding	P2RX6	ENSG00000099957.16	12	7	0.6
protein_coding	AGTR1	ENSG00000144891.17	12	7	0.6
protein_coding	PRICKLE1	ENSG00000139174.11	12	7	0.6
protein_coding	SELENOP	ENSG00000250722.5	12	7	0.6
transcribed_unprocessed_pseudogene	RRN3P3	ENSG00000257122.5	24	14	0.6
protein_coding	ANKRD35	ENSG00000198483.12	24	14	0.6
protein_coding	NDUFAF4	ENSG00000123545.5	36	21	0.6
protein_coding	EXOSC7	ENSG00000075914.12	55	32	0.6
protein_coding	SOCS5	ENSG00000171150.8	184	107	0.6
processed_pseudogene	TMSB4XP4	ENSG00000223551.1	43	25	0.6
protein_coding	IRF1	ENSG00000125347.13	43	25	0.6
protein_coding	SSH3	ENSG00000172830.12	43	25	0.6
protein_coding	EXOSC1	ENSG00000171311.12	86	50	0.6
protein_coding	TRIP6	ENSG00000087077.13	222	129	0.6
protein_coding	GPNMB	ENSG00000136235.16	1160	674	0.6
protein_coding	TIMM44	ENSG00000104980.7	31	18	0.6
protein_coding	OPN3	ENSG00000054277.13	31	18	0.6
protein_coding	RHBDD3	ENSG00000100263.13	31	18	0.6
protein_coding	DMAP1	ENSG00000178028.13	31	18	0.6
protein_coding	POLR3C	ENSG00000186141.8	31	18	0.6
protein_coding	BCAT2	ENSG00000105552.14	31	18	0.6

protein_coding	SYNE3	ENSG00000176438.12	81	47	0.6
protein_coding	PXMP4	ENSG00000101417.11	50	29	0.6
protein_coding	SMAGP	ENSG00000170545.16	100	58	0.6
protein_coding	ELK3	ENSG00000111145.7	169	98	0.6
protein_coding	NES	ENSG00000132688.10	119	69	0.6
protein_coding	CEP131	ENSG00000141577.13	19	11	0.6
protein_coding	ZNF862	ENSG00000106479.10	19	11	0.6
protein_coding	SLC38A9	ENSG00000177058.11	19	11	0.6
antisense	AC010642.2	ENSG00000283103.1	38	22	0.6
protein_coding	EFNA5	ENSG00000184349.12	76	44	0.6
protein_coding	GLOD4	ENSG00000167699.13	83	48	0.6
protein_coding	NOA1	ENSG00000084092.6	109	63	0.6
protein_coding	CLEC16A	ENSG00000038532.15	26	15	0.6
protein_coding	ARPIN	ENSG00000242498.7	26	15	0.6
protein_coding	ANKRD50	ENSG00000151458.11	104	60	0.6
protein_coding	CHCHD6	ENSG00000159685.10	33	19	0.6
lincRNA	LINC00839	ENSG00000185904.11	47	27	0.6
protein_coding	NABP1	ENSG00000173559.12	47	27	0.6
protein_coding	HSD3B7	ENSG00000099377.13	82	47	0.6
protein_coding	ARFIP1	ENSG00000164144.15	96	55	0.6
protein_coding	ADGRA2	ENSG00000020181.17	327	187	0.6
protein_coding	FAM167B	ENSG00000183615.5	7	4	0.6
protein_coding	SLC9A3R1	ENSG00000109062.11	7	4	0.6
protein_coding	SLC22A15	ENSG00000163393.12	7	4	0.6
protein_coding	PPP4R4	ENSG00000119698.11	7	4	0.6
antisense	UBA6-AS1	ENSG00000248049.6	7	4	0.6
protein_coding	RPL39L	ENSG00000163923.9	7	4	0.6
lincRNA	BX470102.2	ENSG00000285867.1	7	4	0.6
protein_coding	PANK4	ENSG00000157881.13	7	4	0.6
lincRNA	AC005332.5	ENSG00000277476.1	7	4	0.6
lincRNA	AL445423.1	ENSG00000272823.1	7	4	0.6
protein_coding	NTF3	ENSG00000185652.11	7	4	0.6
protein_coding	DNAJC17	ENSG00000104129.9	7	4	0.6
antisense	ZNF790-AS1	ENSG00000267254.5	7	4	0.6
protein_coding	FOXE1	ENSG00000178919.8	7	4	0.6
antisense	AC016727.1	ENSG00000270820.5	7	4	0.6
protein_coding	ZNF546	ENSG00000187187.13	7	4	0.6
processed_transcript	PPP3CB-AS1	ENSG00000221817.9	7	4	0.6
protein_coding	ESPNL	ENSG00000144488.14	7	4	0.6
protein_coding	KCNE3	ENSG00000175538.10	7	4	0.6
protein_coding	EXOSC5	ENSG00000077348.8	14	8	0.6
protein_coding	ZNF398	ENSG00000197024.8	14	8	0.6
protein_coding	IKZF2	ENSG00000030419.16	14	8	0.6
protein_coding	PROB1	ENSG00000228672.3	14	8	0.6

lincRNA	AC009414.2	ENSG00000260025.1	14	8	0.6
protein_coding	CPEB3	ENSG00000107864.14	14	8	0.6
protein_coding	CCDC102A	ENSG00000135736.5	14	8	0.6
transcribed_unprocessed_pseudogene	SEPT7P2	ENSG00000214765.8	14	8	0.6
protein_coding	MKS1	ENSG00000011143.16	14	8	0.6
protein_coding	NFKBIE	ENSG00000146232.16	14	8	0.6
protein_coding	KIAA1324	ENSG00000116299.16	14	8	0.6
protein_coding	WDR47	ENSG00000085433.15	21	12	0.6
protein_coding	PPM1H	ENSG00000111110.11	21	12	0.6
protein_coding	ABHD10	ENSG00000144827.8	35	20	0.6
protein_coding	FAM49A	ENSG00000197872.11	35	20	0.6
protein_coding	TRABD	ENSG00000170638.9	42	24	0.6
protein_coding	NMI	ENSG00000123609.10	42	24	0.6
protein_coding	CCDC102B	ENSG00000150636.17	49	28	0.6
protein_coding	INO80	ENSG00000128908.16	70	40	0.6
protein_coding	FAM171B	ENSG00000144369.12	399	228	0.6
protein_coding	MEA1	ENSG00000124733.4	163	93	0.6
protein_coding	RAMP1	ENSG00000132329.10	65	37	0.6
Mt_tRNA	MT-TL1	ENSG00000209082.1	51	29	0.6
protein_coding	ADAMTSL4	ENSG00000143382.14	44	25	0.6
protein_coding	GRPEL2	ENSG00000164284.14	30	17	0.6
protein_coding	TSHZ1	ENSG00000179981.10	23	13	0.6
protein_coding	RAB33B	ENSG00000172007.5	23	13	0.6
protein_coding	MEGF6	ENSG00000162591.15	23	13	0.6
protein_coding	SLC25A12	ENSG00000115840.13	62	35	0.6
protein_coding	ANXA11	ENSG00000122359.17	420	237	0.6
protein_coding	FARSB	ENSG00000116120.9	39	22	0.6
protein_coding	RAP2B	ENSG00000181467.4	204	115	0.6
protein_coding	IFI27L1	ENSG00000165948.10	55	31	0.6
protein_coding	CCDC146	ENSG00000135205.14	16	9	0.6
unprocessed_pseudogene	MTCO2P12	ENSG00000229344.1	16	9	0.6
protein_coding	GRIN2D	ENSG00000105464.3	16	9	0.6
protein_coding	DUS4L	ENSG00000105865.10	16	9	0.6
protein_coding	WDR27	ENSG00000184465.16	16	9	0.6
protein_coding	LRRC49	ENSG00000137821.11	16	9	0.6
protein_coding	PUS1	ENSG00000177192.13	16	9	0.6
protein_coding	PARD6G	ENSG00000178184.15	16	9	0.6
protein_coding	MPI	ENSG00000178802.17	32	18	0.6
protein_coding	SNRNP40	ENSG00000060688.12	32	18	0.6
protein_coding	MET	ENSG00000105976.14	48	27	0.6
protein_coding	IMPDH2	ENSG00000178035.11	224	126	0.6
protein_coding	GABPB1	ENSG00000104064.17	41	23	0.6

protein_coding	PMS1	ENSG00000064933.17	41	23	0.6
protein_coding	EDA2R	ENSG00000131080.14	148	83	0.6
protein_coding	EGR1	ENSG00000120738.7	116	65	0.6
protein_coding	CENPF	ENSG00000117724.12	25	14	0.6
protein_coding	RMI1	ENSG00000178966.16	25	14	0.6
protein_coding	ZSWIM1	ENSG00000168612.4	25	14	0.6
protein_coding	COL10A1	ENSG00000123500.9	25	14	0.6
protein_coding	EPSTI1	ENSG00000133106.14	25	14	0.6
protein_coding	PPM1D	ENSG00000170836.11	75	42	0.6
protein_coding	ZNF224	ENSG00000267680.5	34	19	0.6
protein_coding	RAB7B	ENSG00000276600.4	68	38	0.6
protein_coding	SDHAF2	ENSG00000167985.6	77	43	0.6
protein_coding	SUGP1	ENSG00000105705.15	43	24	0.6
protein_coding	ACTA2	ENSG00000107796.13	398	222	0.6
protein_coding	CDC42EP4	ENSG00000179604.9	131	73	0.6
protein_coding	FAM69A	ENSG00000154511.11	259	144	0.6
protein_coding	SOX9	ENSG00000125398.6	9	5	0.6
protein_coding	PARP2	ENSG00000129484.13	9	5	0.6
scaRNA	SCARNA22	ENSG00000249784.1	9	5	0.6
protein_coding	PABPC1L	ENSG00000101104.12	9	5	0.6
protein_coding	MYBL1	ENSG00000185697.16	9	5	0.6
protein_coding	DNM1	ENSG00000106976.20	9	5	0.6
protein_coding	NEURL2	ENSG00000124257.6	9	5	0.6
protein_coding	AARS2	ENSG00000124608.4	9	5	0.6
protein_coding	ZNF624	ENSG00000197566.9	9	5	0.6
protein_coding	TRIM7	ENSG00000146054.17	9	5	0.6
lincRNA	LY6E-DT	ENSG00000247317.3	9	5	0.6
protein_coding	LIPT1	ENSG00000144182.16	9	5	0.6
protein_coding	ZNF793	ENSG00000188227.12	9	5	0.6
antisense	ZNF710-AS1	ENSG00000259291.2	9	5	0.6
protein_coding	GCNT4	ENSG00000176928.5	9	5	0.6
protein_coding	TMEM169	ENSG00000163449.10	9	5	0.6
protein_coding	GCHFR	ENSG00000137880.5	9	5	0.6
protein_coding	ZNF45	ENSG00000124459.11	18	10	0.6
protein_coding	SNAPC4	ENSG00000165684.4	18	10	0.6
protein_coding	OLFM1	ENSG00000130558.19	18	10	0.6
protein_coding	TAMM41	ENSG00000144559.10	18	10	0.6
protein_coding	CNEP1R1	ENSG00000205423.11	36	20	0.6
protein_coding	RGL2	ENSG00000237441.9	54	30	0.6
protein_coding	VPS72	ENSG00000163159.12	54	30	0.6
protein_coding	MARCH8	ENSG00000165406.15	72	40	0.6
protein_coding	CDK4	ENSG00000135446.16	179	99	0.6
protein_coding	TMEM97	ENSG00000109084.13	38	21	0.6
protein_coding	TCFL5	ENSG00000101190.12	58	32	0.6

protein_coding	LDAH	ENSG00000118961.14	40	22	0.6
protein_coding	DNTTIP1	ENSG00000101457.12	40	22	0.6
protein_coding	C17orf75	ENSG00000108666.9	40	22	0.6
protein_coding	TENM3	ENSG00000218336.8	60	33	0.6
protein_coding	LAMB3	ENSG00000196878.14	80	44	0.6
protein_coding	SPECC1L	ENSG00000100014.19	71	39	0.5
protein_coding	SAE1	ENSG00000142230.11	224	123	0.5
protein_coding	GARS	ENSG00000106105.13	423	232	0.5
protein_coding	IFI44	ENSG00000137965.10	53	29	0.5
protein_coding	UGDH	ENSG00000109814.11	245	134	0.5
protein_coding	PTPN14	ENSG00000152104.11	225	123	0.5
protein_coding	CNN2	ENSG00000064666.14	386	211	0.5
protein_coding	KLHL21	ENSG00000162413.16	161	88	0.5
protein_coding	SHANK2	ENSG00000162105.18	11	6	0.5
transcribed_unprocessed_pseudogene	AC004980.1	ENSG00000205485.13	11	6	0.5
protein_coding	TNFAIP8L1	ENSG00000185361.8	11	6	0.5
protein_coding	ARRDC1	ENSG00000197070.13	11	6	0.5
protein_coding	SLC10A7	ENSG00000120519.14	11	6	0.5
protein_coding	TIGD7	ENSG00000140993.10	11	6	0.5
protein_coding	KMT5C	ENSG00000133247.13	11	6	0.5
protein_coding	ZNF718	ENSG00000250312.7	11	6	0.5
protein_coding	FKBP1B	ENSG00000119782.13	11	6	0.5
processed_pseudogene	RPL11P3	ENSG00000213613.2	11	6	0.5
transcribed_unprocessed_pseudogene	BTN2A3P	ENSG00000124549.14	11	6	0.5
protein_coding	PHEX	ENSG00000102174.8	11	6	0.5
protein_coding	AKTIP	ENSG00000166971.16	11	6	0.5
protein_coding	JMJD6	ENSG00000070495.14	22	12	0.5
protein_coding	KAZN	ENSG00000189337.16	22	12	0.5
protein_coding	SLC29A3	ENSG00000198246.8	22	12	0.5
transcribed_unprocessed_pseudogene	LRRC37BP1	ENSG00000250462.8	22	12	0.5
protein_coding	ZNF839	ENSG00000022976.15	22	12	0.5
lincRNA	Z97200.1	ENSG00000271811.1	22	12	0.5
protein_coding	GLB1L	ENSG00000163521.15	22	12	0.5
protein_coding	LRIG3	ENSG00000139263.11	33	18	0.5
protein_coding	MKKS	ENSG00000125863.19	44	24	0.5
protein_coding	DOK6	ENSG00000206052.10	77	42	0.5
protein_coding	CKS1B	ENSG00000173207.12	35	19	0.5
protein_coding	USE1	ENSG00000053501.12	35	19	0.5
protein_coding	CHIC1	ENSG00000204116.11	59	32	0.5
antisense	PCAT6	ENSG00000228288.6	24	13	0.5

protein_coding	RUNX1T1	ENSG00000079102.16	150	81	0.5
protein_coding	MKNK2	ENSG00000099875.14	254	137	0.5
protein_coding	TRIB2	ENSG00000071575.11	89	48	0.5
protein_coding	PI4KB	ENSG00000143393.16	89	48	0.5
protein_coding	LIMA1	ENSG00000050405.13	1183	638	0.5
protein_coding	SCFD2	ENSG00000184178.15	191	103	0.5
protein_coding	LAMA3	ENSG00000053747.16	13	7	0.5
protein_coding	ACP6	ENSG00000162836.11	13	7	0.5
protein_coding	ZNF471	ENSG00000196263.7	13	7	0.5
protein_coding	BOP¬†1	ENSG00000261236.7	13	7	0.5
protein_coding	MZF1	ENSG00000099326.8	13	7	0.5
protein_coding	KRBA1	ENSG00000133619.17	13	7	0.5
lincRNA	AC005332.4	ENSG00000274712.1	13	7	0.5
protein_coding	ANKS3	ENSG00000168096.14	13	7	0.5
protein_coding	PCIF1	ENSG00000100982.11	13	7	0.5
lincRNA	LINC01963	ENSG00000260804.3	13	7	0.5
lincRNA	AP003469.4	ENSG00000261087.1	13	7	0.5
protein_coding	ANKRD46	ENSG00000186106.11	26	14	0.5
protein_coding	PPFIBP2	ENSG00000166387.12	26	14	0.5
protein_coding	MYC	ENSG00000136997.17	26	14	0.5
protein_coding	CAMK1D	ENSG00000183049.12	67	36	0.5
protein_coding	NSMAF	ENSG00000035681.8	108	58	0.5
protein_coding	FAM110B	ENSG00000169122.11	41	22	0.5
protein_coding	RAB3IL1	ENSG00000167994.11	41	22	0.5
protein_coding	LOXL1	ENSG00000129038.15	371	199	0.5
lincRNA	AL035071.1	ENSG00000260257.2	28	15	0.5
protein_coding	ZC4H2	ENSG00000126970.15	28	15	0.5
protein_coding	OLFML2B	ENSG00000162745.10	140	75	0.5
protein_coding	ALKBH3	ENSG00000166199.12	43	23	0.5
protein_coding	HIST1H4H	ENSG00000158406.4	43	23	0.5
protein_coding	SLC35A3	ENSG00000117620.14	58	31	0.5
protein_coding	RGL1	ENSG00000143344.15	73	39	0.5
antisense	NUTM2A-	ENSG00000223482.7	15	8	0.5
protein_coding	TSTD3	ENSG00000228439.5	15	8	0.5
protein_coding	FEN1	ENSG00000168496.3	15	8	0.5
protein_coding	TBC1D22B	ENSG00000065491.8	15	8	0.5
protein_coding	LRRC23	ENSG00000010626.14	15	8	0.5
protein_coding	NECTIN1	ENSG00000110400.10	15	8	0.5
protein_coding	PRR11	ENSG00000068489.12	15	8	0.5
protein_coding	PER2	ENSG00000132326.11	15	8	0.5
protein_coding	ZNF414	ENSG00000133250.13	15	8	0.5
protein_coding	TADA2B	ENSG00000173011.11	30	16	0.5
protein_coding	VAMP5	ENSG00000168899.4	135	72	0.5
protein_coding	PCCA	ENSG00000175198.16	47	25	0.5

protein_coding	CTPS2	ENSG00000047230.14	32	17	0.5
protein_coding	COA6	ENSG00000168275.14	49	26	0.5
protein_coding	SHMT2	ENSG00000182199.10	149	79	0.5
protein_coding	CNST	ENSG00000162852.13	68	36	0.5
protein_coding	EBF1	ENSG00000164330.16	140	74	0.5
protein_coding	OGFRL1	ENSG00000119900.8	142	75	0.5
protein_coding	SMAD1	ENSG00000170365.9	36	19	0.5
protein_coding	NR3C1	ENSG00000113580.14	493	260	0.5
protein_coding	SCARA3	ENSG00000168077.13	55	29	0.5
protein_coding	CD4	ENSG00000010610.9	19	10	0.5
protein_coding	EVA1A	ENSG00000115363.13	19	10	0.5
protein_coding	ZNF529	ENSG00000186020.12	19	10	0.5
protein_coding	TYK2	ENSG00000105397.13	57	30	0.5
protein_coding	TPX2	ENSG00000088325.15	76	40	0.5
protein_coding	NT5DC2	ENSG00000168268.10	61	32	0.5
antisense	CTBP1-DT	ENSG00000196810.4	21	11	0.5
protein_coding	BCL2L12	ENSG00000126453.9	21	11	0.5
protein_coding	MAD1L1	ENSG00000002822.15	21	11	0.5
protein_coding	CDC42EP5	ENSG00000167617.2	42	22	0.5
protein_coding	ZNF557	ENSG00000130544.11	42	22	0.5
protein_coding	CHN1	ENSG00000128656.13	342	179	0.5
protein_coding	DCLRE1C	ENSG00000152457.17	44	23	0.5
protein_coding	PCBD1	ENSG00000166228.8	111	58	0.5
protein_coding	MAPK8IP3	ENSG00000138834.12	23	12	0.5
protein_coding	ELF4	ENSG00000102034.16	23	12	0.5
protein_coding	ZNF579	ENSG00000218891.4	23	12	0.5
protein_coding	ZNF584	ENSG00000171574.17	23	12	0.5
protein_coding	BAIAP2	ENSG00000175866.15	48	25	0.5
protein_coding	PATZ1	ENSG00000100105.17	48	25	0.5
protein_coding	BCAT1	ENSG00000060982.14	676	352	0.5
protein_coding	ZNF580	ENSG00000213015.8	25	13	0.5
protein_coding	APBA3	ENSG00000011132.11	25	13	0.5
protein_coding	RNF138	ENSG00000134758.13	25	13	0.5
protein_coding	SLC15A3	ENSG00000110446.10	25	13	0.5
protein_coding	HMGA2	ENSG00000149948.13	104	54	0.5
protein_coding	AHNAK2	ENSG00000185567.6	156	81	0.5
protein_coding	PIP5K1C	ENSG00000186111.9	79	41	0.5
protein_coding	TMCO4	ENSG00000162542.13	27	14	0.5
protein_coding	AC093323.1	ENSG00000170846.17	27	14	0.5
protein_coding	CEP78	ENSG00000148019.13	27	14	0.5
protein_coding	FILIP1L	ENSG00000168386.18	137	71	0.5
protein_coding	NEGR1	ENSG00000172260.14	85	44	0.5
protein_coding	PHYHD1	ENSG00000175287.18	29	15	0.5
protein_coding	PRIMPOL	ENSG00000164306.10	29	15	0.5

protein_coding	SERPINB6	ENSG00000124570.19	120	62	0.5
protein_coding	MMAA	ENSG00000151611.14	31	16	0.5
protein_coding	GLCCI1	ENSG00000106415.12	93	48	0.5
transcribed_processed_pseudogene	TSPY26P	ENSG00000235217.6	33	17	0.5
protein_coding	TRIM24	ENSG00000122779.17	35	18	0.5
protein_coding	PNPLA4	ENSG00000006757.11	35	18	0.5
protein_coding	EIF2B3	ENSG00000070785.16	37	19	0.5
protein_coding	AHDC1	ENSG00000126705.14	113	58	0.5
protein_coding	GSE1	ENSG00000131149.18	76	39	0.5
protein_coding	TUBE1	ENSG00000074935.13	39	20	0.5
protein_coding	RARG	ENSG00000172819.16	80	41	0.5
protein_coding	ACACA	ENSG00000278540.4	43	22	0.5
protein_coding	GSTM4	ENSG00000168765.16	43	22	0.5
protein_coding	EYA1	ENSG00000104313.18	90	46	0.5
protein_coding	KLF7	ENSG00000118263.14	47	24	0.5
protein_coding	RFXANK	ENSG00000064490.13	47	24	0.5
protein_coding	HNMT	ENSG00000150540.13	49	25	0.5
protein_coding	PRRX1	ENSG00000116132.11	2765	1410	0.5
protein_coding	NUP93	ENSG00000102900.12	53	27	0.5
protein_coding	SNRPF	ENSG00000139343.10	110	56	0.5
protein_coding	CXXC5	ENSG00000171604.11	165	84	0.5
protein_coding	ASF1A	ENSG00000111875.7	61	31	0.5
protein_coding	CREB3L1	ENSG00000157613.10	621	315	0.5
protein_coding	SH3D19	ENSG00000109686.17	211	107	0.5
protein_coding	ITPR3	ENSG00000096433.10	75	38	0.5
protein_coding	AHNAK	ENSG00000124942.13	1883	949	0.5
processed_transcript	RPARP-AS1	ENSG00000269609.5	2	1	0.5
protein_coding	BRICD5	ENSG00000182685.7	2	1	0.5
protein_coding	ESPL1	ENSG00000135476.11	2	1	0.5
protein_coding	TEC	ENSG00000135605.12	2	1	0.5
protein_coding	PCSK1N	ENSG00000102109.8	2	1	0.5
protein_coding	MAP10	ENSG00000212916.4	2	1	0.5
protein_coding	NUF2	ENSG00000143228.12	2	1	0.5
antisense	DARS-AS1	ENSG00000231890.7	2	1	0.5
antisense	JAZF1-AS1	ENSG00000234336.6	2	1	0.5
protein_coding	SEMA7A	ENSG00000138623.10	2	1	0.5
antisense	AC067750.1	ENSG00000272631.1	2	1	0.5
protein_coding	GIPC3	ENSG00000179855.6	2	1	0.5
sense_overlapping	HYMAI	ENSG00000283122.1	2	1	0.5
protein_coding	LGI4	ENSG00000153902.13	2	1	0.5
protein_coding	ACOT4	ENSG00000177465.4	2	1	0.5

bidirectional_promoter_lncRNA	AL662884.3	ENSG00000284954.1	2	1	0.5
sense_intronic	AC007216.3	ENSG00000261560.1	2	1	0.5
processed_pseudogene	AC010343.1	ENSG00000240376.1	2	1	0.5
antisense	DICER1-AS1	ENSG00000235706.7	2	1	0.5
antisense	AL022328.1	ENSG00000272836.1	2	1	0.5
protein_coding	GNLY	ENSG00000115523.16	2	1	0.5
lincRNA	AC107982.3	ENSG00000265478.2	2	1	0.5
TEC	AC006077.2	ENSG00000279799.1	2	1	0.5
protein_coding	FSD1	ENSG00000105255.10	2	1	0.5
protein_coding	SUV39H1	ENSG00000101945.16	2	1	0.5
protein_coding	HARBI1	ENSG00000180423.4	2	1	0.5
protein_coding	CELSR3	ENSG00000008300.16	2	1	0.5
processed_pseudogene	FO393411.1	ENSG00000180211.5	2	1	0.5
protein_coding	B3GNT5	ENSG00000176597.11	2	1	0.5
protein_coding	CBWD2	ENSG00000136682.14	2	1	0.5
lincRNA	AC092279.1	ENSG00000268362.5	2	1	0.5
protein_coding	FOXP2	ENSG00000128573.24	2	1	0.5
antisense	AP002748.3	ENSG00000255517.6	2	1	0.5
lincRNA	TMEM202-	ENSG00000261423.1	2	1	0.5
lincRNA	AC011815.2	ENSG00000274184.1	2	1	0.5
protein_coding	ANO7	ENSG00000146205.13	2	1	0.5
antisense	AC006116.4	ENSG00000267192.1	2	1	0.5
protein_coding	NFKBID	ENSG00000167604.14	2	1	0.5
antisense	AC121761.1	ENSG00000257497.2	2	1	0.5
antisense	AC015712.2	ENSG00000259583.2	2	1	0.5
antisense	AL391069.2	ENSG00000232671.5	2	1	0.5
protein_coding	RYR2	ENSG00000198626.15	2	1	0.5
protein_coding	RFESD	ENSG00000175449.13	2	1	0.5
TEC	AC012513.3	ENSG00000279348.1	2	1	0.5
antisense	AL162377.1	ENSG00000231856.2	2	1	0.5
processed_pseudogene	RPL13AP20	ENSG00000234498.3	2	1	0.5
protein_coding	SEMA4A	ENSG00000196189.12	2	1	0.5
antisense	AL118506.1	ENSG00000268858.2	2	1	0.5
processed_transcript	DLEU1	ENSG00000176124.12	2	1	0.5
processed_pseudogene	RPL5P34	ENSG00000234009.1	2	1	0.5
antisense	FLJ31356	ENSG00000229951.5	2	1	0.5
misc_RNA	RN7SL689P	ENSG00000263432.2	2	1	0.5
protein_coding	WBP1	ENSG00000239779.6	2	1	0.5
transcribed_processed_pseudogene	RPSAP36	ENSG00000243175.1	2	1	0.5
sense_intronic	AC026356.2	ENSG00000276115.1	2	1	0.5
lincRNA	AC006449.6	ENSG00000277969.1	2	1	0.5

antisense	FRMD6-AS1	ENSG00000273888.1	2	1	0.5
antisense	NRSN2-AS1	ENSG00000225377.5	2	1	0.5
transcribed_unitary_pseudogene	PRORSD1P	ENSG00000162997.15	2	1	0.5
protein_coding	TTYH2	ENSG00000141540.10	2	1	0.5
lincRNA	ITGB1-DT	ENSG00000229656.6	2	1	0.5
bidirectional_promoter_lncRNA	INKA2-AS1	ENSG00000227811.2	2	1	0.5
antisense	VLDLR-AS1	ENSG00000236404.9	2	1	0.5
processed_transcript	AC074212.1	ENSG00000259605.3	2	1	0.5
protein_coding	STKLD1	ENSG00000198870.7	2	1	0.5
3prime_overlapping_ncRNA	AC051619.6	ENSG00000259539.1	2	1	0.5
lincRNA	AC104653.1	ENSG00000228857.2	2	1	0.5
lincRNA	AC114689.3	ENSG00000265533.1	2	1	0.5
snoRNA	SNORD114-	ENSG00000272344.1	2	1	0.5
protein_coding	AC079594.2	ENSG00000248710.1	2	1	0.5
sense_overlapping	LINC01118	ENSG00000222005.9	2	1	0.5
sense_intronic	AC012370.1	ENSG00000232693.2	2	1	0.5
protein_coding	AC112128.1	ENSG00000285943.1	2	1	0.5
protein_coding	CENPI	ENSG00000102384.13	2	1	0.5
protein_coding	CHTF18	ENSG00000127586.16	2	1	0.5
protein_coding	NEIL3	ENSG00000109674.3	2	1	0.5
processed_pseudogene	PPP1R14BP2	ENSG00000213082.3	2	1	0.5
processed_pseudogene	RPL7P9	ENSG00000137970.7	2	1	0.5
processed_pseudogene	RPL12P4	ENSG00000185834.10	2	1	0.5
TEC	AC134407.3	ENSG00000279880.1	2	1	0.5
protein_coding	MMP28	ENSG00000271447.5	2	1	0.5
protein_coding	BBS1	ENSG00000174483.19	2	1	0.5
processed_pseudogene	AL050331.1	ENSG00000233558.1	2	1	0.5
processed_pseudogene	RPS15AP30	ENSG00000227560.1	2	1	0.5
protein_coding	UFSP1	ENSG00000176125.4	2	1	0.5
lincRNA	AL023581.2	ENSG00000227192.1	2	1	0.5
misc_RNA	RN7SL200P	ENSG00000265123.2	2	1	0.5
transcribed_unprocessed_pseudogene	SUGT1P3	ENSG00000239827.8	2	1	0.5
lincRNA	AC073335.2	ENSG00000273142.1	2	1	0.5
TEC	AC090616.6	ENSG00000278867.1	2	1	0.5
antisense	AC118553.1	ENSG00000228084.1	2	1	0.5
antisense	AL162274.2	ENSG00000277959.1	2	1	0.5
lincRNA	AC025175.1	ENSG00000272416.1	2	1	0.5
protein_coding	ROBO2	ENSG00000185008.17	2	1	0.5
antisense	AL451085.2	ENSG00000271380.1	2	1	0.5

protein_coding	DNAH1	ENSG00000114841.17	2	1	0.5
protein_coding	MMP13	ENSG00000137745.11	2	1	0.5
antisense	AC079174.2	ENSG00000277715.1	2	1	0.5
protein_coding	MCF2L	ENSG00000126217.20	2	1	0.5
lincRNA	AC080013.6	ENSG00000272440.1	2	1	0.5
antisense	AC022893.1	ENSG00000253636.1	2	1	0.5
antisense	AC002454.1	ENSG00000237819.5	2	1	0.5
protein_coding	IBSP	ENSG00000029559.6	2	1	0.5
protein_coding	CSF1R	ENSG00000182578.13	2	1	0.5
protein_coding	MYCBPAP	ENSG00000136449.13	2	1	0.5
protein_coding	SPIN2A	ENSG00000147059.8	2	1	0.5
protein_coding	SPANXB1	ENSG00000227234.1	2	1	0.5
antisense	AL139099.1	ENSG00000258377.1	2	1	0.5
protein_coding	ANKRD31	ENSG00000145700.9	2	1	0.5
protein_coding	TOMM5	ENSG00000175768.12	2	1	0.5
processed_transcript	SUGT1P4-STRA6LP	ENSG00000254876.5	2	1	0.5
antisense	AC022098.1	ENSG00000267169.1	2	1	0.5
sense_intronic	HM13-IT1	ENSG00000235313.1	2	1	0.5
protein_coding	MBNL3	ENSG00000076770.14	2	1	0.5
protein_coding	FBF1	ENSG00000188878.19	2	1	0.5
antisense	OSGEPL1-	ENSG00000253559.1	2	1	0.5
protein_coding	LRP4	ENSG00000134569.9	2	1	0.5
protein_coding	RASEF	ENSG00000165105.9	2	1	0.5
processed_transcript	[illegible] PVRIG2P- [illegible]	ENSG00000272752.6	2	1	0.5
sense_overlapping	AC108676.1	ENSG00000244675.2	2	1	0.5
sense_intronic	AL136295.7	ENSG00000278784.1	2	1	0.5
protein_coding	RNF122	ENSG00000133874.1	2	1	0.5
sense_intronic	AL513477.2	ENSG00000272420.1	2	1	0.5
antisense	SEC24B-AS1	ENSG00000247950.6	2	1	0.5
processed_pseudogene	RPL7P24	ENSG00000240003.2	2	1	0.5
protein_coding	FILIP1	ENSG00000118407.14	2	1	0.5
antisense	AC100793.2	ENSG00000267042.1	2	1	0.5
processed_pseudogene	RPL9P32	ENSG00000242100.2	2	1	0.5
protein_coding	REPS2	ENSG00000169891.17	2	1	0.5
protein_coding	JAG2	ENSG00000184916.8	2	1	0.5
antisense	AL080317.3	ENSG00000272356.1	2	1	0.5
protein_coding	TMEM144	ENSG00000164124.10	2	1	0.5
protein_coding	LUZP2	ENSG00000187398.11	2	1	0.5
transcribed_unprocessed_pseudogene	GTF2IP20	ENSG00000272645.3	2	1	0.5
lincRNA	AC027237.2	ENSG00000259215.1	2	1	0.5

processed_pseudogene	TPT1P9	ENSG00000234782.3	2	1	0.5
protein_coding	ZNF843	ENSG00000176723.9	2	1	0.5
antisense	LIFR-AS1	ENSG00000244968.6	2	1	0.5
protein_coding	SRRM3	ENSG00000177679.15	2	1	0.5
protein_coding	SLC26A4	ENSG00000091137.12	2	1	0.5
processed_pseudogene	EIF5P1	ENSG00000215319.2	2	1	0.5
antisense	AL603839.3	ENSG00000238287.1	2	1	0.5
protein_coding	MFSD2B	ENSG00000205639.10	2	1	0.5
protein_coding	B3GNT8	ENSG00000177191.2	2	1	0.5
protein_coding	ZNF491	ENSG00000177599.12	2	1	0.5
protein_coding	SULT2B1	ENSG00000088002.11	2	1	0.5
antisense	AC016252.1	ENSG00000272609.1	2	1	0.5
protein_coding	CDRT1	ENSG00000241322.10	2	1	0.5
transcribed_unprocessed_pseudogene	AC241952.1	ENSG00000275131.3	2	1	0.5
antisense	TNFRSF14-	ENSG00000238164.6	2	1	0.5
antisense	ZEB2-AS1	ENSG00000238057.9	2	1	0.5
antisense	AL136304.1	ENSG00000272223.1	2	1	0.5
antisense	AL158212.2	ENSG00000233547.1	2	1	0.5
lincRNA	BX323046.1	ENSG00000273175.1	2	1	0.5
lincRNA	LINC01934	ENSG00000234663.6	2	1	0.5
lincRNA	AC009974.1	ENSG00000272555.1	2	1	0.5
lincRNA	AC025754.2	ENSG00000271874.1	2	1	0.5
lincRNA	AC104118.1	ENSG00000271862.1	2	1	0.5
lincRNA	LINC01611	ENSG00000231776.5	2	1	0.5
lincRNA	AL109924.2	ENSG00000229720.1	2	1	0.5
lincRNA	AC061975.1	ENSG00000260777.1	2	1	0.5
processed_pseudogene	AC133965.1	ENSG00000225933.1	2	1	0.5
processed_pseudogene	Z75741.1	ENSG00000224548.1	2	1	0.5
processed_transcript	LINC01341	ENSG00000227953.6	2	1	0.5
processed_transcript	AC010913.1	ENSG00000272702.1	2	1	0.5
protein_coding	MPP4	ENSG00000082126.17	2	1	0.5
protein_coding	ACSL6	ENSG00000164398.12	2	1	0.5
protein_coding	PCDHB3	ENSG00000113205.5	2	1	0.5
protein_coding	ENDOU	ENSG00000111405.8	2	1	0.5
protein_coding	RDH5	ENSG00000135437.9	2	1	0.5
protein_coding	MMP17	ENSG00000198598.6	2	1	0.5
protein_coding	LRCOL1	ENSG00000204583.9	2	1	0.5
protein_coding	U2AF1L5	ENSG00000275895.6	2	1	0.5
protein_coding	DSCAM	ENSG00000171587.14	2	1	0.5
protein_coding	NLGN3	ENSG00000196338.12	2	1	0.5
protein_coding	PABPC5	ENSG00000174740.7	2	1	0.5
TEC	AC084876.2	ENSG00000280426.1	2	1	0.5

transcribed_unprocessed_pseudogene	ZNF300P1	ENSG00000197083.11	2	1	0.5
protein_coding	ACSM3	ENSG00000005187.11	2	1	0.5
misc_RNA	RF00019	ENSG00000207326.1	2	1	0.5
antisense	HAND2-AS1	ENSG00000237125.9	2	1	0.5
TEC	AC012676.5	ENSG00000280063.1	2	1	0.5
lincRNA	AC011450.1	ENSG00000197813.5	2	1	0.5
processed_transcript	CLLU1	ENSG00000257127.6	2	1	0.5
processed_pseudogene	AC091564.1	ENSG00000241678.1	2	1	0.5
lincRNA	LINC01715	ENSG00000229388.1	2	1	0.5
protein_coding	TEDC2	ENSG00000162062.14	2	1	0.5
transcribed_unprocessed_pseudogene	FAM86DP	ENSG00000244026.6	4	2	0.5
protein_coding	ZNF565	ENSG00000196357.11	4	2	0.5
protein_coding	FAM234B	ENSG00000084444.13	4	2	0.5
protein_coding	CD34	ENSG00000174059.16	4	2	0.5
protein_coding	ETV4	ENSG00000175832.12	4	2	0.5
lincRNA	AC013652.1	ENSG00000259345.6	4	2	0.5
protein_coding	FAM229A	ENSG00000225828.1	4	2	0.5
protein_coding	AGBL3	ENSG00000146856.14	4	2	0.5
protein_coding	UCP2	ENSG00000175567.8	4	2	0.5
protein_coding	PTGIS	ENSG00000124212.5	4	2	0.5
protein_coding	ZNF484	ENSG00000127081.13	4	2	0.5
protein_coding	SLC25A27	ENSG00000153291.15	4	2	0.5
TEC	AC006511.3	ENSG00000279865.1	4	2	0.5
protein_coding	VMAC	ENSG00000187650.3	4	2	0.5
protein_coding	ZNF789	ENSG00000198556.13	4	2	0.5
protein_coding	C18orf32	ENSG00000177576.11	4	2	0.5
lincRNA	AL645933.2	ENSG00000272221.1	4	2	0.5
processed_pseudogene	AC034236.1	ENSG00000185641.6	4	2	0.5
protein_coding	RNF166	ENSG00000158717.10	4	2	0.5
protein_coding	ICAM5	ENSG00000105376.4	4	2	0.5
processed_pseudogene	HMGB1P31	ENSG00000233266.1	4	2	0.5
protein_coding	TRPV1	ENSG00000196689.12	4	2	0.5
protein_coding	RGS20	ENSG00000147509.13	4	2	0.5
antisense	GAS5-AS1	ENSG00000270084.1	4	2	0.5
protein_coding	CYP2S1	ENSG00000167600.13	4	2	0.5
protein_coding	ACAP1	ENSG00000072818.11	4	2	0.5
antisense	FOXC2-AS1	ENSG00000260944.1	4	2	0.5
protein_coding	SIGLEC15	ENSG00000197046.11	4	2	0.5
protein_coding	ZNF782	ENSG00000196597.11	4	2	0.5
protein_coding	LDHD	ENSG00000166816.14	4	2	0.5
protein_coding	NEURL1B	ENSG00000214357.8	4	2	0.5

protein_coding	SOWAHD	ENSG00000187808.4	4	2	0.5
protein_coding	CFAP70	ENSG00000156042.17	4	2	0.5
bidirectional_promoter_lncRNA	AP002851.1	ENSG00000283959.1	4	2	0.5
protein_coding	QTRT1	ENSG00000213339.8	4	2	0.5
misc_RNA	VTRNA1-3	ENSG00000202515.1	4	2	0.5
lincRNA	LINC00886	ENSG00000240875.5	4	2	0.5
antisense	AL031775.1	ENSG00000272345.1	4	2	0.5
processed_pseudogene	AC011495.1	ENSG00000243829.1	4	2	0.5
processed_pseudogene	CDC20P1	ENSG00000231007.5	4	2	0.5
protein_coding	SCIN	ENSG00000006747.14	4	2	0.5
protein_coding	PPFIA2	ENSG00000139220.16	4	2	0.5
bidirectional_promoter_lncRNA	KDM7A-DT	ENSG00000260231.2	4	2	0.5
protein_coding	PASK	ENSG00000115687.13	4	2	0.5
protein_coding	MOB3B	ENSG00000120162.9	4	2	0.5
lincRNA	AC027307.3	ENSG00000268798.1	4	2	0.5
protein_coding	GBA	ENSG00000177628.15	4	2	0.5
antisense	LRRC8C-DT	ENSG00000231999.6	4	2	0.5
lincRNA	AC012557.1	ENSG00000271843.1	4	2	0.5
protein_coding	FAIM2	ENSG00000135472.8	4	2	0.5
antisense	AZIN1-AS1	ENSG00000253320.6	6	3	0.5
protein_coding	ZSCAN25	ENSG00000197037.10	6	3	0.5
protein_coding	MARCH3	ENSG00000173926.5	6	3	0.5
protein_coding	DACT1	ENSG00000165617.14	6	3	0.5
protein_coding	CGNL1	ENSG00000128849.10	6	3	0.5
lincRNA	AL392172.1	ENSG00000228106.5	6	3	0.5
protein_coding	ABCA9	ENSG00000154258.16	6	3	0.5
protein_coding	LEF1	ENSG00000138795.9	6	3	0.5
protein_coding	EPHX4	ENSG00000172031.6	6	3	0.5
protein_coding	TIRAP	ENSG00000150455.13	6	3	0.5
protein_coding	SPIRE2	ENSG00000204991.10	6	3	0.5
antisense	AL117336.3	ENSG00000271335.5	6	3	0.5
antisense	AC004803.1	ENSG00000250132.6	6	3	0.5
protein_coding	LZTS3	ENSG00000088899.15	6	3	0.5
protein_coding	TNIP3	ENSG00000050730.15	6	3	0.5
protein_coding	TMEM241	ENSG00000134490.13	6	3	0.5
protein_coding	NUDT7	ENSG00000140876.11	6	3	0.5
protein_coding	VWA1	ENSG00000179403.11	6	3	0.5
lincRNA	AC008393.1	ENSG00000245317.2	6	3	0.5
protein_coding	ZNF696	ENSG00000185730.7	6	3	0.5
protein_coding	GLI4	ENSG00000250571.6	6	3	0.5
protein_coding	FAM241A	ENSG00000174749.5	6	3	0.5

transcribed_unprocessed_pseudogene	AC242376.2	ENSG00000274471.2	6	3	0.5
protein_coding	OPRL1	ENSG00000125510.15	6	3	0.5
protein_coding	CCNF	ENSG00000162063.12	6	3	0.5
protein_coding	OLAH	ENSG00000152463.14	6	3	0.5
lincRNA	AC136475.5	ENSG00000255328.1	6	3	0.5
transcribed_unprocessed_pseudogene	LINC01881	ENSG00000220804.8	8	4	0.5
unprocessed_pseudogene	AC003072.1	ENSG00000250318.1	8	4	0.5
protein_coding	TMEM150C	ENSG00000249242.7	8	4	0.5
protein_coding	ZNF575	ENSG00000176472.10	8	4	0.5
protein_coding	TK1	ENSG00000167900.11	8	4	0.5
protein_coding	MEIS1	ENSG00000143995.19	8	4	0.5
protein_coding	RAB38	ENSG00000123892.11	8	4	0.5
protein_coding	HIST1H2BK	ENSG00000197903.7	8	4	0.5
protein_coding	OLFM2	ENSG00000105088.8	8	4	0.5
protein_coding	ANKRD36	ENSG00000135976.18	8	4	0.5
protein_coding	IL17RD	ENSG00000144730.17	8	4	0.5
protein_coding	KRT7	ENSG00000135480.15	8	4	0.5
protein_coding	STPG1	ENSG00000001460.17	8	4	0.5
protein_coding	TUT1	ENSG00000149016.15	8	4	0.5
protein_coding	HSPB8	ENSG00000152137.6	8	4	0.5
protein_coding	FLVCR2	ENSG00000119686.9	8	4	0.5
processed_pseudogene	AC026403.1	ENSG00000229119.3	8	4	0.5
lincRNA	SLC2A1-AS1	ENSG00000227533.5	8	4	0.5
protein_coding	OR10A6	ENSG00000279000.3	8	4	0.5
protein_coding	SLC38A4	ENSG00000139209.15	8	4	0.5
antisense	AC120053.1	ENSG00000271971.1	10	5	0.5
protein_coding	PITPNM2	ENSG00000090975.12	10	5	0.5
protein_coding	C19orf48	ENSG00000167747.14	10	5	0.5
protein_coding	VPS37D	ENSG00000176428.5	10	5	0.5
protein_coding	RTL10	ENSG00000215012.8	10	5	0.5
protein_coding	GPRIN1	ENSG00000169258.6	10	5	0.5
protein_coding	ZNF560	ENSG00000198028.3	10	5	0.5
protein_coding	CD83	ENSG00000112149.9	10	5	0.5
protein_coding	USP49	ENSG00000164663.14	10	5	0.5
protein_coding	PYROXD2	ENSG00000119943.12	10	5	0.5
protein_coding	ASIC1	ENSG00000110881.11	10	5	0.5
protein_coding	VASH2	ENSG00000143494.15	10	5	0.5
protein_coding	POU6F1	ENSG00000184271.17	12	6	0.5
protein_coding	TIGD1	ENSG00000221944.7	12	6	0.5
protein_coding	TRPV2	ENSG00000187688.14	12	6	0.5
protein_coding	SETD4	ENSG00000185917.13	12	6	0.5

protein_coding	HR	ENSG00000168453.14	12	6	0.5
protein_coding	EIF4E3	ENSG00000163412.12	12	6	0.5
processed_transcript	LINC01278	ENSG00000235437.7	12	6	0.5
protein_coding	PPP2R3B	ENSG00000167393.17	12	6	0.5
protein_coding	NAPRT	ENSG00000147813.15	12	6	0.5
antisense	MAN1B1-DT	ENSG00000268996.3	14	7	0.5
protein_coding	NARS2	ENSG00000137513.9	14	7	0.5
protein_coding	RWDD2A	ENSG00000013392.7	14	7	0.5
protein_coding	LSM¬†11	ENSG00000155858.5	14	7	0.5
antisense	PINK1-AS	ENSG00000117242.7	14	7	0.5
protein_coding	ATP6V1G2	ENSG00000213760.10	14	7	0.5
protein_coding	ABCA7	ENSG00000064687.12	14	7	0.5
protein_coding	TGFB3	ENSG00000119699.7	16	8	0.5
protein_coding	TAF4	ENSG00000130699.18	16	8	0.5
processed_pseudogene	RPL37P2	ENSG00000239559.2	16	8	0.5
protein_coding	ERCC8	ENSG00000049167.14	16	8	0.5
protein_coding	MECR	ENSG00000116353.15	16	8	0.5
protein_coding	CRACR2A	ENSG00000130038.9	18	9	0.5
protein_coding	MXD1	ENSG00000059728.10	20	10	0.5
protein_coding	AGTPBP1	ENSG00000135049.15	20	10	0.5
lincRNA	AC104986.2	ENSG00000253948.1	20	10	0.5
protein_coding	MOK	ENSG00000080823.22	22	11	0.5
protein_coding	POLR1B	ENSG00000125630.15	22	11	0.5
protein_coding	C1orf53	ENSG00000203724.10	22	11	0.5
protein_coding	SLC39A3	ENSG00000141873.10	22	11	0.5
lincRNA	EIF3J-DT	ENSG00000179523.4	24	12	0.5
protein_coding	VWCE	ENSG00000167992.12	24	12	0.5
protein_coding	LRRCC1	ENSG00000133739.15	24	12	0.5
protein_coding	SERTAD4	ENSG00000082497.11	26	13	0.5
protein_coding	ZSCAN2	ENSG00000176371.13	28	14	0.5
protein_coding	SARM1	ENSG00000004139.13	28	14	0.5
protein_coding	CTBS	ENSG00000117151.12	30	15	0.5
lincRNA	MIR193BHG	ENSG00000262454.3	34	17	0.5
protein_coding	MARCH2	ENSG00000099785.10	38	19	0.5
protein_coding	DCXR	ENSG00000169738.7	38	19	0.5
protein_coding	MAPK12	ENSG00000188130.13	40	20	0.5
protein_coding	KAZALD1	ENSG00000107821.14	42	21	0.5
protein_coding	CKB	ENSG00000166165.12	44	22	0.5
protein_coding	COQ6	ENSG00000119723.16	46	23	0.5
protein_coding	ALS2	ENSG00000003393.14	48	24	0.5
protein_coding	MTMR1	ENSG00000063601.16	48	24	0.5
protein_coding	MAMDC2	ENSG00000165072.9	58	29	0.5
protein_coding	TGFB1	ENSG00000105329.9	64	32	0.5
protein_coding	DALRD3	ENSG00000178149.16	64	32	0.5

protein_coding	CAPG	ENSG00000042493.15	86	43	0.5
protein_coding	SELENBP1	ENSG00000143416.20	94	47	0.5
protein_coding	SLC6A9	ENSG00000196517.11	110	55	0.5
protein_coding	SOD3	ENSG00000109610.5	238	119	0.5
protein_coding	UHRF2	ENSG00000147854.16	157	78	0.5
protein_coding	ANXA1	ENSG00000135046.13	865	429	0.5
protein_coding	PDGFC	ENSG00000145431.10	109	54	0.5
protein_coding	FYCO1	ENSG00000163820.14	206	102	0.5
protein_coding	DDIT3	ENSG00000175197.12	93	46	0.5
protein_coding	PXN	ENSG00000089159.16	235	116	0.5
protein_coding	PLPP1	ENSG00000067113.16	622	307	0.5
protein_coding	SSBP2	ENSG00000145687.16	75	37	0.5
protein_coding	MITF	ENSG00000187098.15	63	31	0.5
protein_coding	RCBTB1	ENSG00000136144.11	61	30	0.5
protein_coding	TNFRSF19	ENSG00000127863.15	122	60	0.5
protein_coding	ANGPTL2	ENSG00000136859.9	478	235	0.5
protein_coding	FAH	ENSG00000103876.12	55	27	0.5
protein_coding	LSM¬†7	ENSG00000130332.14	94	46	0.5
protein_coding	DTNA	ENSG00000134769.21	45	22	0.5
protein_coding	KLHDC8B	ENSG00000185909.14	43	21	0.5
protein_coding	DGLUCY	ENSG00000133943.20	43	21	0.5
protein_coding	LRRK1	ENSG00000154237.12	41	20	0.5
protein_coding	C2CD5	ENSG00000111731.12	37	18	0.5
lincRNA	APTR	ENSG00000214293.8	37	18	0.5
protein_coding	CPS1	ENSG00000021826.15	35	17	0.5
protein_coding	MYNN	ENSG00000085274.15	33	16	0.5
protein_coding	CBLN2	ENSG00000141668.9	33	16	0.5
protein_coding	IFT80	ENSG00000068885.14	66	32	0.5
antisense	DHRS4-AS1	ENSG00000215256.3	31	15	0.5
protein_coding	PLEKHF2	ENSG00000175895.3	31	15	0.5
protein_coding	ZNF766	ENSG00000196214.10	31	15	0.5
protein_coding	ZNF174	ENSG00000103343.12	31	15	0.5
protein_coding	AARS	ENSG00000090861.15	234	113	0.5
protein_coding	PTCD1	ENSG00000106246.17	29	14	0.5
protein_coding	FOXQ1	ENSG00000164379.6	58	28	0.5
protein_coding	OSBPL3	ENSG00000070882.12	110	53	0.5
protein_coding	LRRC17	ENSG00000128606.12	27	13	0.5
protein_coding	ZC3H7B	ENSG00000100403.11	389	187	0.5
protein_coding	CHST15	ENSG00000182022.17	25	12	0.5
protein_coding	SCAI	ENSG00000173611.17	25	12	0.5
protein_coding	ZMYM3	ENSG00000147130.14	75	36	0.5
protein_coding	UMPS	ENSG00000114491.13	23	11	0.5
protein_coding	ZNF250	ENSG00000196150.13	23	11	0.5
TEC	AC134043.2	ENSG00000279204.1	23	11	0.5

protein_coding	GTF2F2	ENSG00000188342.11	247	118	0.5
protein_coding	EPHA2	ENSG00000142627.12	21	10	0.5
protein_coding	ADM2	ENSG00000128165.8	21	10	0.5
protein_coding	ZNF317	ENSG00000130803.14	21	10	0.5
protein_coding	GMPR	ENSG00000137198.9	21	10	0.5
protein_coding	RFTN2	ENSG00000162944.10	42	20	0.5
protein_coding	NDUFAF1	ENSG00000137806.8	40	19	0.5
protein_coding	ZNF155	ENSG00000204920.10	19	9	0.5
protein_coding	KCNK2	ENSG00000082482.13	167	79	0.5
protein_coding	COPRS	ENSG00000172301.10	195	92	0.5
protein_coding	CAV1	ENSG00000105974.11	6285	2961	0.5
protein_coding	MAMLD1	ENSG00000013619.13	17	8	0.5
protein_coding	ZNF616	ENSG00000204611.6	17	8	0.5
protein_coding	NEK11	ENSG00000114670.13	17	8	0.5
protein_coding	PRKD1	ENSG00000184304.14	51	24	0.5
protein_coding	PARD3B	ENSG00000116117.17	85	40	0.5
protein_coding	ADGRL2	ENSG00000117114.19	66	31	0.5
protein_coding	JADE1	ENSG00000077684.15	248	116	0.5
protein_coding	MT1E	ENSG00000169715.14	154	72	0.5
protein_coding	ZNRF2	ENSG00000180233.10	15	7	0.5
protein_coding	C6orf136	ENSG00000204564.11	15	7	0.5
protein_coding	SMIM8	ENSG00000111850.10	15	7	0.5
protein_coding	ROR1	ENSG00000185483.11	15	7	0.5
protein_coding	GAS1	ENSG00000180447.6	534	249	0.5
protein_coding	ALDH3A2	ENSG00000072210.18	163	76	0.5
protein_coding	TRIM16	ENSG00000221926.12	73	34	0.5
protein_coding	DISP1	ENSG00000154309.8	58	27	0.5
protein_coding	FHL3	ENSG00000183386.9	56	26	0.5
protein_coding	APBB2	ENSG00000163697.16	93	43	0.5
protein_coding	PPP1R3C	ENSG00000119938.8	119	55	0.5
protein_coding	THAP8	ENSG00000161277.10	13	6	0.5
lincRNA	PVT1	ENSG00000249859.9	13	6	0.5
protein_coding	NXT1	ENSG00000132661.3	13	6	0.5
protein_coding	KRT34	ENSG00000131737.5	13	6	0.5
TEC	AP001528.3	ENSG00000280339.1	13	6	0.5
protein_coding	ATP23	ENSG00000166896.8	13	6	0.5
protein_coding	JRK	ENSG00000234616.8	26	12	0.5
protein_coding	GEMIN7	ENSG00000142252.10	26	12	0.5
protein_coding	ATP6V0E2	ENSG00000171130.18	26	12	0.5
protein_coding	DNAJC4	ENSG00000110011.13	39	18	0.5
protein_coding	NRP2	ENSG00000118257.16	78	36	0.5
protein_coding	STEAP3	ENSG00000115107.19	109	50	0.5
protein_coding	FBXO2	ENSG00000116661.10	24	11	0.5
protein_coding	BTBD3	ENSG00000132640.14	24	11	0.5

protein_coding	ALPK2	ENSG00000198796.6	96	44	0.5
protein_coding	SLC29A1	ENSG00000112759.17	35	16	0.5
protein_coding	GEMIN4	ENSG00000179409.10	35	16	0.5
protein_coding	FIZ1	ENSG00000179943.7	11	5	0.5
protein_coding	C19orf54	ENSG00000188493.14	11	5	0.5
protein_coding	ZNF519	ENSG00000175322.11	11	5	0.5
protein_coding	PTGER4	ENSG00000171522.5	11	5	0.5
TEC	AC008537.3	ENSG00000279108.1	11	5	0.5
protein_coding	UBOX5	ENSG00000185019.16	11	5	0.5
protein_coding	MLST8	ENSG00000167965.17	33	15	0.5
protein_coding	APOE	ENSG00000130203.9	33	15	0.5
protein_coding	CNOT10	ENSG00000182973.18	44	20	0.5
protein_coding	KCTD1	ENSG00000134504.13	44	20	0.5
protein_coding	TARSL2	ENSG00000185418.15	119	54	0.5
protein_coding	SYNC	ENSG00000162520.14	73	33	0.5
protein_coding	ARG2	ENSG00000081181.7	20	9	0.5
processed_pseudogene	AC073861.1	ENSG00000242299.1	20	9	0.5
protein_coding	MTHFSD	ENSG00000103248.18	20	9	0.5
protein_coding	TMCC1	ENSG00000172765.17	60	27	0.5
protein_coding	AMOTL2	ENSG00000114019.14	60	27	0.5
protein_coding	CASP8	ENSG00000064012.21	49	22	0.4
protein_coding	NYNRIN	ENSG00000205978.5	58	26	0.4
protein_coding	ARHGAP42	ENSG00000165895.18	38	17	0.4
protein_coding	AP4M1	ENSG00000221838.9	47	21	0.4
protein_coding	THSD4	ENSG00000187720.14	188	84	0.4
protein_coding	PLPP3	ENSG00000162407.8	3736	1669	0.4
protein_coding	FBXO17	ENSG00000269190.5	92	41	0.4
protein_coding	FZD8	ENSG00000177283.7	9	4	0.4
protein_coding	ALKBH8	ENSG00000137760.14	9	4	0.4
protein_coding	CDKN3	ENSG00000100526.19	9	4	0.4
antisense	AP003068.2	ENSG00000254614.2	9	4	0.4
protein_coding	ZKSCAN7	ENSG00000196345.12	9	4	0.4
protein_coding	MPZ	ENSG00000158887.15	9	4	0.4
protein_coding	ZNF574	ENSG00000105732.12	9	4	0.4
protein_coding	FAM227B	ENSG00000166262.15	9	4	0.4
transcribed_unprocessed_pseudogene	NMRAL2P	ENSG00000171658.8	9	4	0.4
protein_coding	CYP¬†20	ENSG00000130649.9	9	4	0.4
antisense	AC009318.2	ENSG00000273680.1	9	4	0.4
protein_coding	CTSH	ENSG00000103811.16	9	4	0.4
protein_coding	HEPH	ENSG00000089472.16	9	4	0.4
sense_overlapping	SNHG20	ENSG00000234912.12	9	4	0.4
protein_coding	MAP2K6	ENSG00000108984.14	9	4	0.4

protein_coding	RRP1	ENSG00000160214.12	18	8	0.4
protein_coding	TRDMT1	ENSG00000107614.21	18	8	0.4
protein_coding	PARP12	ENSG00000059378.12	18	8	0.4
protein_coding	IFI35	ENSG00000068079.7	18	8	0.4
protein_coding	C2orf76	ENSG00000186132.14	18	8	0.4
protein_coding	ATF7	ENSG00000170653.18	27	12	0.4
protein_coding	NEFM	ENSG00000104722.13	27	12	0.4
protein_coding	ELOVL6	ENSG00000170522.9	36	16	0.4
protein_coding	ICK	ENSG00000112144.15	63	28	0.4
protein_coding	TCEA2	ENSG00000171703.16	72	32	0.4
protein_coding	SGMS2	ENSG00000164023.14	90	40	0.4
protein_coding	LUM	ENSG00000139329.4	577	255	0.4
protein_coding	PIR	ENSG00000087842.10	34	15	0.4
protein_coding	EPS15L1	ENSG00000127527.13	25	11	0.4
protein_coding	FRMD6	ENSG00000139926.15	297	130	0.4
protein_coding	C2CD3	ENSG00000168014.16	16	7	0.4
protein_coding	TFAP4	ENSG00000090447.11	16	7	0.4
protein_coding	TBXA2R	ENSG00000006638.11	16	7	0.4
protein_coding	TCN2	ENSG00000185339.8	16	7	0.4
protein_coding	IMP4	ENSG00000136718.9	39	17	0.4
protein_coding	PMVK	ENSG00000163344.5	39	17	0.4
protein_coding	CHRM2	ENSG00000181072.11	62	27	0.4
protein_coding	S100A3	ENSG00000188015.9	23	10	0.4
protein_coding	ADCY7	ENSG00000121281.12	23	10	0.4
protein_coding	NRG1	ENSG00000157168.18	53	23	0.4
protein_coding	GFOD2	ENSG00000141098.12	30	13	0.4
protein_coding	RARA	ENSG00000131759.17	67	29	0.4
protein_coding	B3GALT6	ENSG00000176022.5	95	41	0.4
protein_coding	PAK1	ENSG00000149269.9	51	22	0.4
protein_coding	MRPS6	ENSG00000243927.5	144	62	0.4
protein_coding	SIPA1L2	ENSG00000116991.10	93	40	0.4
protein_coding	EME2	ENSG00000197774.12	7	3	0.4
protein_coding	C3orf67	ENSG00000163689.20	7	3	0.4
protein_coding	ZSCAN12	ENSG00000158691.14	7	3	0.4
lincRNA	AC040977.1	ENSG00000262089.1	7	3	0.4
protein_coding	SNCA	ENSG00000145335.15	7	3	0.4
protein_coding	FUK	ENSG00000157353.16	7	3	0.4
protein_coding	LURAP1	ENSG00000171357.5	7	3	0.4
protein_coding	GBGT1	ENSG00000148288.12	7	3	0.4
antisense	AP001160.2	ENSG00000267811.1	7	3	0.4
protein_coding	CCHCR1	ENSG00000204536.13	7	3	0.4
protein_coding	APOC1	ENSG00000130208.9	7	3	0.4
protein_coding	TRAM1L1	ENSG00000174599.5	7	3	0.4
antisense	RBM26-AS1	ENSG00000227354.6	7	3	0.4

antisense	AL358472.4	ENSG00000282386.1	7	3	0.4
protein_coding	ARRB1	ENSG00000137486.16	14	6	0.4
protein_coding	PHLPP1	ENSG00000081913.13	14	6	0.4
protein_coding	ARVCF	ENSG00000099889.13	14	6	0.4
protein_coding	PCDH9	ENSG00000184226.14	14	6	0.4
protein_coding	ZNF276	ENSG00000158805.11	14	6	0.4
protein_coding	ZNF425	ENSG00000204947.8	14	6	0.4
protein_coding	INKA1	ENSG00000185614.4	14	6	0.4
protein_coding	IL20RB	ENSG00000174564.12	14	6	0.4
protein_coding	NUDT11	ENSG00000196368.4	21	9	0.4
protein_coding	SLC25A51	ENSG00000122696.13	21	9	0.4
protein_coding	MLPH	ENSG00000115648.13	21	9	0.4
protein_coding	MAN2C1	ENSG00000140400.16	28	12	0.4
protein_coding	ETNK2	ENSG00000143845.14	28	12	0.4
protein_coding	SCN8A	ENSG00000196876.15	56	24	0.4
sense_overlapping	AP003071.4	ENSG00000261625.1	56	24	0.4
protein_coding	TRIM26	ENSG00000234127.8	70	30	0.4
protein_coding	IFT52	ENSG00000101052.12	70	30	0.4
protein_coding	PDP1	ENSG00000164951.15	77	33	0.4
protein_coding	PHGDH	ENSG00000092621.12	110	47	0.4
protein_coding	COL3A1	ENSG00000168542.14	6263	2661	0.4
protein_coding	TMEM171	ENSG00000157111.12	73	31	0.4
protein_coding	TRNAU1AP	ENSG00000180098.9	33	14	0.4
protein_coding	PPM1K	ENSG00000163644.14	33	14	0.4
protein_coding	ZSCAN26	ENSG00000197062.11	33	14	0.4
protein_coding	PEX3	ENSG00000034693.14	26	11	0.4
protein_coding	PAWR	ENSG00000177425.10	149	63	0.4
protein_coding	NRM	ENSG00000137404.14	19	8	0.4
protein_coding	ZNF672	ENSG00000171161.12	19	8	0.4
protein_coding	MPP1	ENSG00000130830.14	50	21	0.4
protein_coding	GPHN	ENSG00000171723.15	43	18	0.4
protein_coding	TRIM65	ENSG00000141569.11	43	18	0.4
protein_coding	SLC12A8	ENSG00000221955.10	110	46	0.4
protein_coding	AK5	ENSG00000154027.18	230	96	0.4
protein_coding	SLC4A7	ENSG00000033867.16	367	153	0.4
protein_coding	TRMT61A	ENSG00000166166.12	12	5	0.4
protein_coding	FP565260.6	ENSG00000280433.1	12	5	0.4
protein_coding	AGFG2	ENSG00000106351.12	12	5	0.4
protein_coding	FBXO5	ENSG00000112029.9	12	5	0.4
protein_coding	KLHDC4	ENSG00000104731.13	12	5	0.4
protein_coding	FOXM1	ENSG00000111206.12	12	5	0.4
protein_coding	ZNF92	ENSG00000146757.13	12	5	0.4
protein_coding	EEF1A2	ENSG00000101210.11	12	5	0.4
protein_coding	TTLL4	ENSG00000135912.10	12	5	0.4

protein_coding	PSMD9	ENSG00000110801.13	12	5	0.4
protein_coding	AXIN2	ENSG00000168646.12	12	5	0.4
Mt_tRNA	MT-TM	ENSG00000210112.1	24	10	0.4
protein_coding	ADA	ENSG00000196839.12	36	15	0.4
protein_coding	IFIT1	ENSG00000185745.9	48	20	0.4
protein_coding	MYLIP	ENSG00000007944.14	161	67	0.4
protein_coding	USP32	ENSG00000170832.12	41	17	0.4
protein_coding	BLVRB	ENSG00000090013.10	99	41	0.4
transcribed_unprocessed_pseudogene	RPL23AP82	ENSG00000184319.15	29	12	0.4
protein_coding	SETBP1	ENSG00000152217.18	75	31	0.4
protein_coding	RELB	ENSG00000104856.13	17	7	0.4
protein_coding	PCDHB7	ENSG00000113212.6	17	7	0.4
protein_coding	ZNF804A	ENSG00000170396.7	17	7	0.4
protein_coding	MAB21L2	ENSG00000181541.5	34	14	0.4
protein_coding	KCNJ6	ENSG00000157542.10	34	14	0.4
protein_coding	XRRA1	ENSG00000166435.15	34	14	0.4
protein_coding	INPP5B	ENSG00000204084.12	34	14	0.4
protein_coding	RGS17	ENSG00000091844.7	34	14	0.4
protein_coding	MGARP	ENSG00000137463.4	51	21	0.4
protein_coding	LRRN4CL	ENSG00000177363.4	119	49	0.4
antisense	AL160006.1	ENSG00000258634.3	22	9	0.4
protein_coding	RNF144B	ENSG00000137393.9	22	9	0.4
protein_coding	BCL7C	ENSG00000099385.11	49	20	0.4
protein_coding	ANKRD33B	ENSG00000164236.11	49	20	0.4
protein_coding	TEX30	ENSG00000151287.16	27	11	0.4
protein_coding	ZNF581	ENSG00000171425.9	59	24	0.4
protein_coding	MYLK	ENSG00000065534.18	716	288	0.4
protein_coding	BBS9	ENSG00000122507.20	112	45	0.4
protein_coding	CILP2	ENSG00000160161.9	5	2	0.4
protein_coding	SFR1	ENSG00000156384.14	5	2	0.4
lincRNA	AC132872.1	ENSG00000260563.3	5	2	0.4
processed_pseudogene	AC091042.1	ENSG00000265936.1	5	2	0.4
protein_coding	MANEAL	ENSG00000185090.14	5	2	0.4
protein_coding	ITGB3	ENSG00000259207.7	5	2	0.4
protein_coding	SINHCAF	ENSG00000139146.13	5	2	0.4
protein_coding	ADAM8	ENSG00000151651.15	5	2	0.4
antisense	VIM-AS1	ENSG00000229124.6	5	2	0.4
lincRNA	LINC00882	ENSG00000242759.6	5	2	0.4
protein_coding	PRELID2	ENSG00000186314.11	5	2	0.4
protein_coding	ZBTB49	ENSG00000168826.15	5	2	0.4
protein_coding	PDIK1L	ENSG00000175087.9	5	2	0.4
antisense	NR2F2-AS1	ENSG00000247809.7	5	2	0.4

protein_coding	ALG10	ENSG00000139133.6	5	2	0.4
antisense	B4GALT1-	ENSG00000233554.5	5	2	0.4
protein_coding	ZNF530	ENSG00000183647.10	5	2	0.4
processed_pseudogene	EEF1DP2	ENSG00000226721.2	5	2	0.4
lincRNA	AC069234.5	ENSG00000277423.1	5	2	0.4
protein_coding	CDKL1	ENSG00000100490.9	5	2	0.4
protein_coding	EID2B	ENSG00000176401.5	5	2	0.4
protein_coding	TFEB	ENSG00000112561.17	5	2	0.4
lincRNA	LINC01686	ENSG00000261504.1	5	2	0.4
lincRNA	AL928921.2	ENSG00000284693.1	5	2	0.4
protein_coding	NKX6-1	ENSG00000163623.9	5	2	0.4
sense_intronic	AC127502.2	ENSG00000270055.1	5	2	0.4
protein_coding	ZNF805	ENSG00000204524.6	5	2	0.4
lincRNA	AC068338.2	ENSG00000260274.1	5	2	0.4
antisense	IPO9-AS1	ENSG00000231871.5	5	2	0.4
protein_coding	RFLNA	ENSG00000178882.14	5	2	0.4
protein_coding	IL32	ENSG00000008517.16	5	2	0.4
protein_coding	SRGAP2B	ENSG00000196369.11	5	2	0.4
protein_coding	RGS7	ENSG00000182901.16	10	4	0.4
protein_coding	SUOX	ENSG00000139531.12	10	4	0.4
protein_coding	MED30	ENSG00000164758.7	10	4	0.4
protein_coding	DUSP28	ENSG00000188542.9	10	4	0.4
protein_coding	ZNF324	ENSG00000083812.11	10	4	0.4
protein_coding	FLRT3	ENSG00000125848.9	10	4	0.4
protein_coding	MAP3K7CL	ENSG00000156265.15	10	4	0.4
protein_coding	ZNF607	ENSG00000198182.12	10	4	0.4
protein_coding	ZKSCAN2	ENSG00000155592.15	10	4	0.4
protein_coding	LMCD1	ENSG00000071282.11	10	4	0.4
protein_coding	PFAS	ENSG00000178921.13	10	4	0.4
lincRNA	LINC01003	ENSG00000261455.1	10	4	0.4
protein_coding	ZNF365	ENSG00000138311.16	10	4	0.4
protein_coding	ORC6	ENSG00000091651.8	15	6	0.4
protein_coding	MAPRE3	ENSG00000084764.11	15	6	0.4
protein_coding	PIP	ENSG00000159763.3	15	6	0.4
protein_coding	P2RX4	ENSG00000135124.14	15	6	0.4
protein_coding	SLC4A11	ENSG00000088836.13	20	8	0.4
protein_coding	KIAA1671	ENSG00000197077.13	20	8	0.4
protein_coding	CIART	ENSG00000159208.15	35	14	0.4
protein_coding	CYP26B1	ENSG00000003137.8	55	22	0.4
protein_coding	PAG1	ENSG00000076641.4	60	24	0.4
protein_coding	CAPN5	ENSG00000149260.16	75	30	0.4
protein_coding	PDE5A	ENSG00000138735.15	299	119	0.4
protein_coding	CNKSR3	ENSG00000153721.18	63	25	0.4
protein_coding	DUSP6	ENSG00000139318.7	96	38	0.4

protein_coding	EPHA4	ENSG00000116106.11	28	11	0.4
protein_coding	TBC1D2	ENSG00000095383.19	23	9	0.4
protein_coding	TNFAIP8	ENSG00000145779.7	23	9	0.4
protein_coding	EEF1AKMT1	ENSG00000150456.10	18	7	0.4
protein_coding	THRB	ENSG00000151090.18	18	7	0.4
protein_coding	SNX11	ENSG00000002919.14	18	7	0.4
protein_coding	GPER1	ENSG00000164850.14	18	7	0.4
protein_coding	PQLC2L	ENSG00000174899.10	36	14	0.4
lincRNA	AF127577.4	ENSG00000235609.7	31	12	0.4
antisense	AC090152.1	ENSG00000167912.5	31	12	0.4
protein_coding	CDC42EP3	ENSG00000163171.7	210	81	0.4
protein_coding	C2orf68	ENSG00000168887.10	13	5	0.4
processed_pseudogene	RPL23AP2	ENSG00000225067.4	13	5	0.4
protein_coding	NUDT18	ENSG00000275074.1	13	5	0.4
lincRNA	AL355001.2	ENSG00000275964.1	13	5	0.4
protein_coding	IGHMBP2	ENSG00000132740.8	13	5	0.4
protein_coding	ZNF440	ENSG00000171295.12	13	5	0.4
protein_coding	ALDH5A1	ENSG00000112294.12	13	5	0.4
protein_coding	SAMD12	ENSG00000177570.14	13	5	0.4
protein_coding	LOXL4	ENSG00000138131.3	39	15	0.4
protein_coding	CDKN2C	ENSG00000123080.10	73	28	0.4
protein_coding	AJUBA	ENSG00000129474.15	47	18	0.4
protein_coding	ROBO3	ENSG00000154134.14	34	13	0.4
protein_coding	ALKBH2	ENSG00000189046.10	29	11	0.4
antisense	DNM3OS	ENSG00000230630.5	29	11	0.4
protein_coding	SMARCD1	ENSG00000066117.14	45	17	0.4
protein_coding	MORC4	ENSG00000133131.14	61	23	0.4
protein_coding	NEDD4L	ENSG00000049759.17	223	84	0.4
protein_coding	PURG	ENSG00000172733.11	8	3	0.4
transcribed_unitary_pseudogene	WFDC21P	ENSG00000261040.7	8	3	0.4
protein_coding	THOC6	ENSG00000131652.13	8	3	0.4
protein_coding	ESM1	ENSG00000164283.12	8	3	0.4
protein_coding	BIRC5	ENSG00000089685.14	8	3	0.4
protein_coding	BORA	ENSG00000136122.15	8	3	0.4
protein_coding	FCRLB	ENSG00000162746.14	8	3	0.4
lincRNA	CH17-	ENSG00000197180.2	8	3	0.4
processed_pseudogene	RPL5P4	ENSG00000229994.1	8	3	0.4
sense_overlapping	AL031985.3	ENSG00000260920.2	8	3	0.4
protein_coding	SENP3	ENSG00000161956.12	8	3	0.4
sense_intronic	DGCR11	ENSG00000273311.1	8	3	0.4
protein_coding	ZNF461	ENSG00000197808.11	8	3	0.4

transcribed_unprocessed_pseudogene	SDHAP1	ENSG00000185485.14	8	3	0.4
protein_coding	NXPH4	ENSG00000182379.9	8	3	0.4
antisense	PRKCZ-AS1	ENSG00000182873.5	8	3	0.4
protein_coding	APOD	ENSG00000189058.8	8	3	0.4
protein_coding	LRRC8B	ENSG00000197147.13	8	3	0.4
antisense	ZFHX4-AS1	ENSG00000253661.1	8	3	0.4
protein_coding	APLP1	ENSG00000105290.11	16	6	0.4
protein_coding	F8	ENSG00000185010.14	16	6	0.4
protein_coding	PSCA	ENSG00000167653.4	16	6	0.4
protein_coding	GLRB	ENSG00000109738.10	32	12	0.4
protein_coding	POLR1A	ENSG00000068654.15	48	18	0.4
protein_coding	CHMP6	ENSG00000176108.9	27	10	0.4
protein_coding	TRIM59	ENSG00000213186.7	46	17	0.4
protein_coding	BNC2	ENSG00000173068.17	130	48	0.4
protein_coding	HECW2	ENSG00000138411.12	103	38	0.4
protein_coding	ZNF680	ENSG00000173041.11	19	7	0.4
protein_coding	ST6GAL1	ENSG00000073849.14	19	7	0.4
protein_coding	FGF1	ENSG00000113578.17	19	7	0.4
protein_coding	PRIM2	ENSG00000146143.17	38	14	0.4
protein_coding	ZNF329	ENSG00000181894.14	30	11	0.4
protein_coding	CRYBG1	ENSG00000112297.14	30	11	0.4
protein_coding	SLC22A18	ENSG00000110628.14	52	19	0.4
protein_coding	EVI2B	ENSG00000185862.6	11	4	0.4
antisense	AC084036.1	ENSG00000272990.1	11	4	0.4
protein_coding	CCDC142	ENSG00000135637.13	11	4	0.4
antisense	AC009831.1	ENSG00000263823.1	11	4	0.4
antisense	AL121832.2	ENSG00000273619.1	11	4	0.4
lincRNA	ZNF582-AS1	ENSG00000267454.5	11	4	0.4
protein_coding	CD68	ENSG00000129226.13	11	4	0.4
protein_coding	FBXO27	ENSG00000161243.8	22	8	0.4
protein_coding	OLFML2A	ENSG00000185585.19	33	12	0.4
protein_coding	POSTN	ENSG00000133110.14	884	321	0.4
protein_coding	FAM129A	ENSG00000135842.16	237	86	0.4
protein_coding	BMP4	ENSG00000125378.15	122	44	0.4
protein_coding	PTPN18	ENSG00000072135.12	25	9	0.4
protein_coding	PPIL1	ENSG00000137168.7	25	9	0.4
protein_coding	PARP11	ENSG00000111224.13	25	9	0.4
protein_coding	BCL11A	ENSG00000119866.21	25	9	0.4
protein_coding	TRIM16L	ENSG00000108448.21	67	24	0.4
lincRNA	ILF3-DT	ENSG00000267100.1	14	5	0.4
protein_coding	TADA1	ENSG00000152382.5	14	5	0.4
protein_coding	VAMP1	ENSG00000139190.16	14	5	0.4

protein_coding	ZNF333	ENSG00000160961.11	14	5	0.4
protein_coding	C19orf18	ENSG00000177025.3	14	5	0.4
protein_coding	ALDH1A3	ENSG00000184254.16	28	10	0.4
protein_coding	SLC46A3	ENSG00000139508.14	45	16	0.4
protein_coding	PAMR1	ENSG00000149090.11	82	29	0.4
protein_coding	OXSM	ENSG00000151093.7	17	6	0.4
protein_coding	KCTD15	ENSG00000153885.14	17	6	0.4
protein_coding	NCKAP5L	ENSG00000167566.16	17	6	0.4
protein_coding	DHPS	ENSG00000095059.15	17	6	0.4
protein_coding	C20orf96	ENSG00000196476.11	17	6	0.4
protein_coding	CHAC1	ENSG00000128965.12	17	6	0.4
protein_coding	CPEB2	ENSG00000137449.15	68	24	0.4
protein_coding	RGMB	ENSG00000174136.11	564	199	0.4
protein_coding	KBTBD3	ENSG00000182359.14	20	7	0.4
protein_coding	ZNF85	ENSG00000105750.14	20	7	0.4
protein_coding	NDE1	ENSG00000072864.14	40	14	0.4
protein_coding	SEMA3C	ENSG00000075223.13	103	36	0.3
protein_coding	PTCHD4	ENSG00000244694.7	23	8	0.3
protein_coding	NEK10	ENSG00000163491.16	23	8	0.3
protein_coding	HDDC3	ENSG00000184508.11	23	8	0.3
protein_coding	ZNF385D	ENSG00000151789.11	29	10	0.3
protein_coding	RRNAD1	ENSG00000143303.11	29	10	0.3
lincRNA	LNCTAM34	ENSG00000234546.3	29	10	0.3
protein_coding	MIOS	ENSG00000164654.15	32	11	0.3
protein_coding	CTSK	ENSG00000143387.12	1128	382	0.3
protein_coding	OLFML3	ENSG00000116774.11	101	34	0.3
protein_coding	PTX3	ENSG00000163661.3	247	83	0.3
processed_pseudogene	RPL15P3	ENSG00000212802.4	3	1	0.3
protein_coding	TSGA10	ENSG00000135951.14	3	1	0.3
protein_coding	PIGU	ENSG00000101464.10	3	1	0.3
antisense	LINC01004	ENSG00000228393.3	3	1	0.3
protein_coding	FAM122C	ENSG00000156500.15	3	1	0.3
snoRNA	SNORA12	ENSG00000212464.1	3	1	0.3
processed_transcript	ARHGAP27P1-BPTFP1-KPNA2P3	ENSG00000215769.8	3	1	0.3
protein_coding	RELL2	ENSG00000164620.8	3	1	0.3
protein_coding	ENPP4	ENSG00000001561.6	3	1	0.3
antisense	PAN3-AS1	ENSG00000261485.1	3	1	0.3
protein_coding	ZNF540	ENSG00000171817.16	3	1	0.3
protein_coding	CFAP299	ENSG00000197826.11	3	1	0.3
protein_coding	SSTR2	ENSG00000180616.8	3	1	0.3
protein_coding	CCDC159	ENSG00000183401.11	3	1	0.3

antisense	ADNP-AS1	ENSG00000259456.3	3	1	0.3
protein_coding	KIF18B	ENSG00000186185.13	3	1	0.3
protein_coding	GINS3	ENSG00000181938.13	3	1	0.3
antisense	SCARNA9	ENSG00000254911.3	3	1	0.3
protein_coding	HES6	ENSG00000144485.10	3	1	0.3
lincRNA	AL359513.1	ENSG00000278238.1	3	1	0.3
protein_coding	DYNC1I1	ENSG00000158560.14	3	1	0.3
protein_coding	LRRC37A3	ENSG00000176809.10	3	1	0.3
processed_transcript	AC017104.1	ENSG00000224376.1	3	1	0.3
lincRNA	AC012313.5	ENSG00000268912.1	3	1	0.3
protein_coding	NEFH	ENSG00000100285.9	3	1	0.3
transcribed_unitary_pseudogene	CMAHP	ENSG00000168405.17	3	1	0.3
antisense	SPRY4-AS1	ENSG00000231185.6	3	1	0.3
protein_coding	SLC9B1	ENSG00000164037.16	3	1	0.3
processed_pseudogene	AL161787.1	ENSG00000219928.2	3	1	0.3
protein_coding	HSF4	ENSG00000102878.16	3	1	0.3
protein_coding	PPP5D1	ENSG00000230510.6	3	1	0.3
lincRNA	AC004801.6	ENSG00000276814.1	3	1	0.3
processed_pseudogene	RPL7AP6	ENSG00000242071.3	3	1	0.3
protein_coding	CDC20	ENSG00000117399.13	3	1	0.3
protein_coding	SLC27A5	ENSG00000083807.9	3	1	0.3
antisense	PRKAR2A-	ENSG00000224424.7	3	1	0.3
bidirectional_promoter_lncRNA	AL591485.1	ENSG00000285219.1	3	1	0.3
protein_coding	ETV7	ENSG00000010030.13	3	1	0.3
lincRNA	AL356019.2	ENSG00000258768.2	3	1	0.3
protein_coding	THSD1	ENSG00000136114.16	3	1	0.3
protein_coding	PAPLN	ENSG00000100767.16	3	1	0.3
protein_coding	NANOS1	ENSG00000188613.6	3	1	0.3
protein_coding	CXCL14	ENSG00000145824.12	3	1	0.3
antisense	AL008729.1	ENSG00000215022.7	3	1	0.3
processed_transcript	STARD7-AS1	ENSG00000204685.6	3	1	0.3
lincRNA	AC005224.3	ENSG00000266378.1	3	1	0.3
protein_coding	MCOLN2	ENSG00000153898.12	3	1	0.3
lincRNA	AC092903.2	ENSG00000248787.2	3	1	0.3
protein_coding	PPFIA3	ENSG00000177380.13	3	1	0.3
protein_coding	NBEAL2	ENSG00000160796.16	3	1	0.3
protein_coding	ZNF256	ENSG00000152454.3	3	1	0.3
sense_intronic	AC007996.1	ENSG00000277534.1	3	1	0.3
protein_coding	PARD6B	ENSG00000124171.8	3	1	0.3
protein_coding	NR6A1	ENSG00000148200.16	3	1	0.3
processed_pseudogene	RPL39P3	ENSG00000235174.1	3	1	0.3

misc_RNA	RNY1P9	ENSG00000255156.1	3	1	0.3
protein_coding	TMOD1	ENSG00000136842.13	3	1	0.3
antisense	TMEM147-	ENSG00000236144.6	3	1	0.3
protein_coding	CLYBL	ENSG00000125246.15	3	1	0.3
processed_transcript	CBR3-AS1	ENSG00000236830.6	3	1	0.3
lincRNA	PURPL	ENSG00000250337.5	3	1	0.3
lincRNA	AC005618.1	ENSG00000272070.1	3	1	0.3
lincRNA	LINC01134	ENSG00000236423.5	3	1	0.3
lincRNA	AC068473.5	ENSG00000274828.1	3	1	0.3
antisense	AC008691.1	ENSG00000249738.10	3	1	0.3
antisense	AC009005.1	ENSG00000267751.5	3	1	0.3
lincRNA	AL360219.1	ENSG00000273264.1	3	1	0.3
lincRNA	LINC00664	ENSG00000268658.5	3	1	0.3
sense_intronic	AC006566.1	ENSG00000273368.1	3	1	0.3
transcribed_unprocessed_pseudogene	PLAC9P1	ENSG00000214100.8	3	1	0.3
transcribed_unprocessed_pseudogene	BX322639.1	ENSG00000215146.5	3	1	0.3
protein_coding	SCN4B	ENSG00000177098.8	3	1	0.3
antisense	AC048382.6	ENSG00000276278.1	3	1	0.3
protein_coding	CDHR3	ENSG00000128536.15	3	1	0.3
protein_coding	NRL	ENSG00000129535.12	3	1	0.3
antisense	MCM3AP-	ENSG00000215424.9	3	1	0.3
protein_coding	ACP5	ENSG00000102575.11	3	1	0.3
lincRNA	AC107214.2	ENSG00000272744.1	3	1	0.3
protein_coding	BTC	ENSG00000174808.11	3	1	0.3
processed_pseudogene	AC117382.1	ENSG00000240695.1	3	1	0.3
antisense	NKILA	ENSG00000278709.1	3	1	0.3
processed_pseudogene	AL354714.2	ENSG00000213080.3	3	1	0.3
protein_coding	GPR63	ENSG00000112218.8	3	1	0.3
protein_coding	NPL	ENSG00000135838.13	3	1	0.3
sense_intronic	AC079414.3	ENSG00000276007.1	3	1	0.3
antisense	LBX2-AS1	ENSG00000257702.3	3	1	0.3
lincRNA	AC092119.2	ENSG00000274460.1	3	1	0.3
protein_coding	KLLN	ENSG00000227268.3	3	1	0.3
protein_coding	TMEM37	ENSG00000171227.6	3	1	0.3
protein_coding	C3	ENSG00000125730.16	3	1	0.3
protein_coding	CHRNE	ENSG00000108556.8	3	1	0.3
antisense	NALT1	ENSG00000237886.1	3	1	0.3
antisense	AC114760.2	ENSG00000272211.1	3	1	0.3
antisense	MATN1-AS1	ENSG00000186056.10	3	1	0.3
antisense	AL136040.1	ENSG00000273783.1	3	1	0.3
processed_pseudogene	RPL17P36	ENSG00000236058.3	3	1	0.3

protein_coding	POLD4	ENSG00000175482.8	3	1	0.3
antisense	CYB561D2	ENSG00000271858.5	3	1	0.3
antisense	PLCE1-AS1	ENSG00000268894.6	3	1	0.3
antisense	AP001783.1	ENSG00000254607.2	3	1	0.3
processed_pseudogene	TVP23CP1	ENSG00000223584.1	3	1	0.3
processed_pseudogene	RPL30P14	ENSG00000243256.1	3	1	0.3
protein_coding	TDO2	ENSG00000151790.8	3	1	0.3
protein_coding	ZP3	ENSG00000188372.14	3	1	0.3
protein_coding	AL117339.5	ENSG00000283930.1	3	1	0.3
protein_coding	FES	ENSG00000182511.11	3	1	0.3
protein_coding	KCNS1	ENSG00000124134.8	3	1	0.3
lincRNA	AL121583.1	ENSG00000277692.1	3	1	0.3
protein_coding	SHBG	ENSG00000129214.14	3	1	0.3
processed_pseudogene	AL078604.1	ENSG00000216480.2	3	1	0.3
antisense	SENCR	ENSG00000254703.2	3	1	0.3
protein_coding	POC1A	ENSG00000164087.7	6	2	0.3
protein_coding	EBLN2	ENSG00000255423.1	6	2	0.3
misc_RNA	RN7SL2	ENSG00000274012.1	6	2	0.3
protein_coding	PLA2G6	ENSG00000184381.18	6	2	0.3
protein_coding	MAPK10	ENSG00000109339.21	6	2	0.3
processed_pseudogene	AC099789.1	ENSG00000225475.1	6	2	0.3
protein_coding	ANKMY1	ENSG00000144504.15	6	2	0.3
protein_coding	ZNF792	ENSG00000180884.9	6	2	0.3
processed_pseudogene	RPL26P6	ENSG00000229659.1	6	2	0.3
sense_overlapping	AC021016.2	ENSG00000261338.2	6	2	0.3
protein_coding	TG	ENSG00000042832.11	6	2	0.3
protein_coding	PLPPR4	ENSG00000117600.12	6	2	0.3
protein_coding	RAPGEF3	ENSG00000079337.15	6	2	0.3
protein_coding	GGACT	ENSG00000134864.10	6	2	0.3
protein_coding	CRTAM	ENSG00000109943.8	6	2	0.3
transcribed_unprocessed_pseudogene	CFL1P1	ENSG00000223820.5	6	2	0.3
protein_coding	PPCDC	ENSG00000138621.11	6	2	0.3
antisense	AL080317.1	ENSG00000230177.1	6	2	0.3
antisense	ASH1L-AS1	ENSG00000235919.4	6	2	0.3
protein_coding	ZNF662	ENSG00000182983.14	6	2	0.3
protein_coding	SHF	ENSG00000138606.19	6	2	0.3
unprocessed_pseudogene	FRG1JP	ENSG00000215548.2	6	2	0.3
protein_coding	ZNF630	ENSG00000221994.10	6	2	0.3
antisense	AC046143.1	ENSG00000229334.1	6	2	0.3
antisense	LINC01137	ENSG00000233621.1	6	2	0.3
protein_coding	PKDCC	ENSG00000162878.12	6	2	0.3
protein_coding	TUFT1	ENSG00000143367.15	9	3	0.3

protein_coding	FKBPL	ENSG00000204315.3	9	3	0.3
protein_coding	ALPL	ENSG00000162551.13	9	3	0.3
processed_transcript	LINC01138	ENSG00000274020.2	9	3	0.3
protein_coding	MFSD13A	ENSG00000138111.14	9	3	0.3
protein_coding	CORO2B	ENSG00000103647.12	9	3	0.3
transcribed_unprocessed_pseudogene	SCAND2P	ENSG00000176700.20	9	3	0.3
protein_coding	ZMAT1	ENSG00000166432.14	9	3	0.3
protein_coding	ZNF185	ENSG00000147394.18	12	4	0.3
protein_coding	PIANP	ENSG00000139200.13	12	4	0.3
protein_coding	ZNF213	ENSG00000085644.13	12	4	0.3
protein_coding	PLEKHG5	ENSG00000171680.21	18	6	0.3
protein_coding	INTS7	ENSG00000143493.12	18	6	0.3
transcribed_processed_pseudogene	SDAD1P1	ENSG00000228451.4	21	7	0.3
protein_coding	C1orf56	ENSG00000143443.9	24	8	0.3
lincRNA	MIRLET7BH	ENSG00000197182.14	27	9	0.3
protein_coding	HSD17B14	ENSG00000087076.8	27	9	0.3
protein_coding	RSAD1	ENSG00000136444.9	42	14	0.3
protein_coding	EFNB1	ENSG00000090776.5	69	23	0.3
protein_coding	SIX1	ENSG00000126778.9	790	260	0.3
protein_coding	AKAP6	ENSG00000151320.10	31	10	0.3
protein_coding	ARHGAP20	ENSG00000137727.12	31	10	0.3
protein_coding	AAAS	ENSG00000094914.12	22	7	0.3
lincRNA	FAM111A-	ENSG00000245571.6	22	7	0.3
protein_coding	TRMT13	ENSG00000122435.9	19	6	0.3
protein_coding	TMEM51	ENSG00000171729.13	128	40	0.3
protein_coding	GPR1	ENSG00000183671.12	29	9	0.3
protein_coding	GUCY1A2	ENSG00000152402.10	55	17	0.3
protein_coding	CSKMT	ENSG00000214756.7	13	4	0.3
antisense	CKMT2-AS1	ENSG00000247572.7	13	4	0.3
protein_coding	TLE2	ENSG00000065717.14	13	4	0.3
protein_coding	IFI44L	ENSG00000137959.15	26	8	0.3
protein_coding	FOXC2	ENSG00000176692.6	36	11	0.3
protein_coding	SRC	ENSG00000197122.11	36	11	0.3
protein_coding	AKR1C3	ENSG00000196139.13	122	37	0.3
antisense	AP001528.2	ENSG00000255471.1	33	10	0.3
protein_coding	INTU	ENSG00000164066.12	10	3	0.3
protein_coding	ZNF283	ENSG00000167637.16	10	3	0.3
protein_coding	ISYNA1	ENSG00000105655.18	10	3	0.3
protein_coding	PAXX	ENSG00000148362.10	10	3	0.3
protein_coding	WDR4	ENSG00000160193.11	10	3	0.3
processed_transcript	TTC28-AS1	ENSG00000235954.6	10	3	0.3

antisense	RBMS3-AS3	ENSG00000235904.2	10	3	0.3
transcribed_processed_pseudogene	AC098847.1	ENSG00000267669.1	10	3	0.3
protein_coding	SENP8	ENSG00000166192.14	10	3	0.3
protein_coding	GCNT3	ENSG00000140297.12	10	3	0.3
protein_coding	THEM6	ENSG00000130193.7	20	6	0.3
protein_coding	OSGIN1	ENSG00000140961.12	20	6	0.3
protein_coding	ZNF74	ENSG00000185252.18	30	9	0.3
protein_coding	PER3	ENSG00000049246.14	40	12	0.3
protein_coding	ABHD14B	ENSG00000114779.19	27	8	0.3
protein_coding	LGALS3BP	ENSG00000108679.12	98	29	0.3
protein_coding	LIMK2	ENSG00000182541.17	17	5	0.3
protein_coding	LRMDA	ENSG00000148655.14	17	5	0.3
protein_coding	TRIB3	ENSG00000101255.10	34	10	0.3
protein_coding	KHDRBS3	ENSG00000131773.13	101	29	0.3
antisense	AC124798.1	ENSG00000260196.1	7	2	0.3
protein_coding	CDK5R1	ENSG00000176749.8	7	2	0.3
protein_coding	C21orf58	ENSG00000160298.17	7	2	0.3
processed_pseudogene	RPL13P12	ENSG00000215030.5	7	2	0.3
processed_pseudogene	RPL10AP6	ENSG00000226360.5	7	2	0.3
protein_coding	TMEM182	ENSG00000170417.15	7	2	0.3
antisense	AC009318.3	ENSG00000274315.1	7	2	0.3
protein_coding	C1orf112	ENSG00000000460.16	7	2	0.3
protein_coding	EIF4A1	ENSG00000161960.14	7	2	0.3
protein_coding	TMEM38A	ENSG00000072954.6	7	2	0.3
lincRNA	LINC00520	ENSG00000258791.8	7	2	0.3
protein_coding	GDPD1	ENSG00000153982.10	7	2	0.3
antisense	AL138724.1	ENSG00000272269.1	7	2	0.3
lincRNA	AC007114.1	ENSG00000263004.1	7	2	0.3
protein_coding	GALNT16	ENSG00000100626.16	7	2	0.3
lincRNA	FLJ37035	ENSG00000224023.10	7	2	0.3
protein_coding	IL18BP	ENSG00000137496.17	7	2	0.3
protein_coding	ZNF385A	ENSG00000161642.17	7	2	0.3
protein_coding	ARHGEF4	ENSG00000136002.18	7	2	0.3
protein_coding	DNAAF4	ENSG00000256061.7	7	2	0.3
protein_coding	PRDM11	ENSG00000019485.13	7	2	0.3
transcribed_unprocessed_pseudogene	AC138207.8	ENSG00000266865.6	7	2	0.3
protein_coding	GNRH1	ENSG00000147437.9	7	2	0.3
lincRNA	AC087477.2	ENSG00000259275.2	7	2	0.3
lincRNA	U47924.3	ENSG00000275703.1	7	2	0.3
protein_coding	FMO5	ENSG00000131781.12	7	2	0.3
protein_coding	NLE1	ENSG00000073536.17	14	4	0.3

protein_coding	C8orf34	ENSG00000165084.15	14	4	0.3
transcribed_unprocessed_pseudogene	AL109918.1	ENSG00000216775.3	14	4	0.3
protein_coding	ZNF48	ENSG00000180035.12	21	6	0.3
protein_coding	PSAT1	ENSG00000135069.13	204	58	0.3
processed_transcript	AC012146.1	ENSG00000234327.7	18	5	0.3
protein_coding	RPIA	ENSG00000153574.8	18	5	0.3
protein_coding	TIMM17B	ENSG00000126768.12	18	5	0.3
protein_coding	KITLG	ENSG00000049130.15	360	100	0.3
protein_coding	STK32B	ENSG00000152953.12	40	11	0.3
protein_coding	LIN9	ENSG00000183814.15	11	3	0.3
lincRNA	LINC01119	ENSG00000239332.5	11	3	0.3
protein_coding	ZNF551	ENSG00000204519.10	11	3	0.3
protein_coding	DCLRE1A	ENSG00000198924.7	11	3	0.3
protein_coding	FAM185A	ENSG00000222011.8	11	3	0.3
protein_coding	ZFP3	ENSG00000180787.5	11	3	0.3
lincRNA	LINC01133	ENSG00000224259.6	11	3	0.3
lincRNA	AC010618.3	ENSG00000269439.5	11	3	0.3
protein_coding	IL1RAP	ENSG00000196083.9	22	6	0.3
protein_coding	SYNPO	ENSG00000171992.12	99	27	0.3
protein_coding	EVI2A	ENSG00000126860.11	63	17	0.3
protein_coding	ZNF2	ENSG00000275111.4	15	4	0.3
protein_coding	TYW1B	ENSG00000277149.4	15	4	0.3
protein_coding	OXTR	ENSG00000180914.10	75	20	0.3
protein_coding	CDCA4	ENSG00000170779.10	19	5	0.3
protein_coding	ZDHHC1	ENSG00000159714.11	19	5	0.3
antisense	AC093157.1	ENSG00000233184.6	19	5	0.3
protein_coding	NMB	ENSG00000197696.9	85	22	0.3
protein_coding	KIT	ENSG00000157404.15	4	1	0.3
protein_coding	C1orf159	ENSG00000131591.17	4	1	0.3
snoRNA	RF01241	ENSG00000238390.1	4	1	0.3
antisense	GNAS-AS1	ENSG00000235590.7	4	1	0.3
protein_coding	EID3	ENSG00000255150.2	4	1	0.3
antisense	DDN-AS1	ENSG00000257913.2	4	1	0.3
protein_coding	CGREF1	ENSG00000138028.15	4	1	0.3
protein_coding	ZNF124	ENSG00000196418.12	4	1	0.3
protein_coding	ATRNL1	ENSG00000107518.17	4	1	0.3
lincRNA	TEX41	ENSG00000226674.9	4	1	0.3
antisense	AC023157.3	ENSG00000276900.1	4	1	0.3
protein_coding	ARMC4	ENSG00000169126.15	4	1	0.3
processed_pseudogene	AC010468.1	ENSG00000214784.4	4	1	0.3
processed_pseudogene	RPL23AP32	ENSG00000237887.1	4	1	0.3
processed_pseudogene	RPL34P6	ENSG00000231333.2	4	1	0.3

transcribed_unitary_pseudogene	SRP54-AS1	ENSG00000258704.6	4	1	0.3
antisense	SH3BP5-AS1	ENSG00000224660.1	4	1	0.3
antisense	ZNF460-AS1	ENSG00000267871.5	4	1	0.3
processed_pseudogene	AL162578.1	ENSG00000218596.2	4	1	0.3
protein_coding	TRIM46	ENSG00000163462.17	4	1	0.3
protein_coding	FPGT-	ENSG00000259030.7	4	1	0.3
protein_coding	CDX1	ENSG00000113722.16	4	1	0.3
sense_intronic	AC012676.1	ENSG00000262712.1	4	1	0.3
protein_coding	ADGRE2	ENSG00000127507.17	4	1	0.3
protein_coding	TPK1	ENSG00000196511.14	4	1	0.3
protein_coding	GPSM3	ENSG00000213654.9	4	1	0.3
protein_coding	ERICH2	ENSG00000204334.7	4	1	0.3
protein_coding	KRT19	ENSG00000171345.13	4	1	0.3
protein_coding	SLC25A53	ENSG00000269743.2	4	1	0.3
protein_coding	PIK3C2B	ENSG00000133056.13	4	1	0.3
protein_coding	GDF5	ENSG00000125965.8	4	1	0.3
protein_coding	C7	ENSG00000112936.18	4	1	0.3
protein_coding	DMKN	ENSG00000161249.20	4	1	0.3
processed_transcript	DUXAP8	ENSG00000206195.10	4	1	0.3
lincRNA	LINC01006	ENSG00000182648.11	4	1	0.3
antisense	AC099778.1	ENSG00000260236.1	4	1	0.3
processed_pseudogene	RPS19P1	ENSG00000214612.3	4	1	0.3
processed_pseudogene	RPS15AP1	ENSG00000214535.3	4	1	0.3
antisense	AC062017.1	ENSG00000222020.2	4	1	0.3
protein_coding	FBXL8	ENSG00000135722.8	4	1	0.3
protein_coding	DDX43	ENSG00000080007.7	4	1	0.3
protein_coding	GVQW2	ENSG00000279968.2	4	1	0.3
protein_coding	CAMKK1	ENSG00000004660.14	4	1	0.3
protein_coding	NME5	ENSG00000112981.4	4	1	0.3
antisense	FOXN3-AS1	ENSG00000258920.1	4	1	0.3
processed_pseudogene	BEND7P1	ENSG00000230320.1	4	1	0.3
protein_coding	RASL10B	ENSG00000270885.1	4	1	0.3
protein_coding	PPP1R14A	ENSG00000167641.10	4	1	0.3
antisense	AP000866.1	ENSG00000245498.6	4	1	0.3
lincRNA	AC127070.2	ENSG00000250790.4	4	1	0.3
processed_transcript	FLJ46284	ENSG00000248858.7	4	1	0.3
protein_coding	SFXN2	ENSG00000156398.12	4	1	0.3
antisense	CASC2	ENSG00000177640.15	4	1	0.3
protein_coding	GNB1L	ENSG00000185838.13	4	1	0.3
protein_coding	LAMC2	ENSG00000058085.14	4	1	0.3
protein_coding	MSS51	ENSG00000166343.9	4	1	0.3
protein_coding	STON2	ENSG00000140022.11	4	1	0.3

processed_pseudogene	RPL4P3	ENSG00000230364.1	4	1	0.3
protein_coding	CFAP57	ENSG00000243710.7	4	1	0.3
lincRNA	AL358072.1	ENSG00000271427.1	4	1	0.3
lincRNA	AC233266.2	ENSG00000261600.1	4	1	0.3
antisense	AP001437.1	ENSG00000273210.1	4	1	0.3
protein_coding	PDZK1	ENSG00000174827.13	4	1	0.3
antisense	AP001596.1	ENSG00000232692.1	4	1	0.3
lincRNA	GAPLINC	ENSG00000266835.5	4	1	0.3
protein_coding	ASPRV1	ENSG00000244617.2	4	1	0.3
sense_intronic	AP003117.1	ENSG00000270077.1	4	1	0.3
processed_pseudogene	RPSAP61	ENSG00000214016.3	4	1	0.3
protein_coding	NEURL1	ENSG00000107954.10	4	1	0.3
lincRNA	LINC02019	ENSG00000273356.1	8	2	0.3
protein_coding	POLR3G	ENSG00000113356.11	8	2	0.3
lincRNA	GMDS-DT	ENSG00000250903.8	8	2	0.3
protein_coding	HSD17B7	ENSG00000132196.14	8	2	0.3
processed_pseudogene	FTH1P8	ENSG00000219507.4	8	2	0.3
protein_coding	DUS3L	ENSG00000141994.15	8	2	0.3
protein_coding	CLBA1	ENSG00000140104.13	8	2	0.3
transcribed_unprocessed_pseudogene	WASH2P	ENSG00000146556.14	8	2	0.3
protein_coding	RSPH9	ENSG00000172426.15	8	2	0.3
protein_coding	PSG9	ENSG00000183668.17	8	2	0.3
protein_coding	SPAG1	ENSG00000104450.12	8	2	0.3
protein_coding	C11orf45	ENSG00000174370.9	8	2	0.3
protein_coding	ARHGEF10	ENSG00000104728.15	8	2	0.3
transcribed_processed_pseudogene	RPL23AP53	ENSG00000223508.5	8	2	0.3
transcribed_processed_pseudogene	ZNF702P	ENSG00000242779.6	8	2	0.3
lincRNA	HRAT17	ENSG00000234520.5	8	2	0.3
protein_coding	ANK1	ENSG00000029534.20	12	3	0.3
protein_coding	CDAN1	ENSG00000140326.13	12	3	0.3
protein_coding	TIGD2	ENSG00000180346.3	12	3	0.3
protein_coding	RAB40C	ENSG00000197562.9	12	3	0.3
unprocessed_pseudogene	NBPF8	ENSG00000270231.3	12	3	0.3
lincRNA	MIR222HG	ENSG00000270069.1	12	3	0.3
protein_coding	DCK	ENSG00000156136.9	16	4	0.3
lincRNA	AC092807.3	ENSG00000282057.1	16	4	0.3
protein_coding	KIAA1549L	ENSG00000110427.15	16	4	0.3
protein_coding	ZNF573	ENSG00000189144.13	24	6	0.3
protein_coding	PRELP	ENSG00000188783.5	32	8	0.3
protein_coding	MIF4GD	ENSG00000125457.14	37	9	0.2

protein_coding	C15orf39	ENSG00000167173.18	25	6	0.2
protein_coding	DCLK2	ENSG00000170390.15	25	6	0.2
protein_coding	C4orf46	ENSG00000205208.4	46	11	0.2
protein_coding	F3	ENSG00000117525.13	21	5	0.2
protein_coding	CRYL1	ENSG00000165475.14	55	13	0.2
protein_coding	PFKFB4	ENSG00000114268.11	17	4	0.2
processed_transcript	CTC-	ENSG00000233937.6	17	4	0.2
protein_coding	NGF	ENSG00000134259.3	17	4	0.2
protein_coding	ENOX1	ENSG00000120658.13	17	4	0.2
lincRNA	LINC00968	ENSG00000246430.6	17	4	0.2
protein_coding	CLDN11	ENSG00000013297.11	523	121	0.2
protein_coding	SNRPA1	ENSG00000131876.16	13	3	0.2
protein_coding	LDB2	ENSG00000169744.12	83	19	0.2
protein_coding	PTGFR	ENSG00000122420.9	584	133	0.2
protein_coding	SIRT7	ENSG00000187531.13	22	5	0.2
transcribed_unprocessed_pseudogene	AMZ2P1	ENSG00000214174.8	22	5	0.2
protein_coding	TOX	ENSG00000198846.5	119	27	0.2
protein_coding	DOP1B	ENSG00000142197.12	31	7	0.2
protein_coding	PSD	ENSG00000059915.16	9	2	0.2
protein_coding	VIT	ENSG00000205221.12	9	2	0.2
protein_coding	FAM110A	ENSG00000125898.12	9	2	0.2
protein_coding	CDK1	ENSG00000170312.15	9	2	0.2
lincRNA	FLJ42351	ENSG00000237753.1	9	2	0.2
protein_coding	TRIL	ENSG00000255690.2	9	2	0.2
protein_coding	C15orf65	ENSG00000261652.2	14	3	0.2
protein_coding	APOO	ENSG00000184831.13	14	3	0.2
protein_coding	CABLES2	ENSG00000149679.11	14	3	0.2
protein_coding	DRP2	ENSG00000102385.12	14	3	0.2
lincRNA	LINC02482	ENSG00000251580.2	14	3	0.2
protein_coding	AKAP8L	ENSG00000011243.17	29	6	0.2
protein_coding	LAMA5	ENSG00000130702.15	73	15	0.2
protein_coding	APCDD1	ENSG00000154856.12	190	39	0.2
protein_coding	PRTG	ENSG00000166450.12	5	1	0.2
lincRNA	LINC01772	ENSG00000226029.1	5	1	0.2
protein_coding	MUTYH	ENSG00000132781.17	5	1	0.2
protein_coding	RNF208	ENSG00000212864.3	5	1	0.2
protein_coding	NCAPG	ENSG00000109805.9	5	1	0.2
antisense	AC073073.2	ENSG00000272604.1	5	1	0.2
protein_coding	RIPPLY3	ENSG00000183145.8	5	1	0.2
protein_coding	ZNF280B	ENSG00000275004.3	5	1	0.2
protein_coding	PLD6	ENSG00000179598.5	5	1	0.2
sense_intronic	AC083799.1	ENSG00000203644.3	5	1	0.2

lincRNA	AC079322.1	ENSG00000261187.1	5	1	0.2
protein_coding	CCDC148	ENSG00000153237.17	5	1	0.2
transcribed_processed_pseudogene	RPS20P22	ENSG00000239218.2	5	1	0.2
protein_coding	HAUS4	ENSG00000092036.18	5	1	0.2
protein_coding	ZNF823	ENSG00000197933.12	5	1	0.2
protein_coding	C17orf100	ENSG00000256806.5	5	1	0.2
protein_coding	ANKRD65	ENSG00000235098.8	5	1	0.2
antisense	AC096667.1	ENSG00000283839.1	5	1	0.2
transcribed_unprocessed_pseudogene	AC141586.1	ENSG00000215154.6	5	1	0.2
protein_coding	EPHX2	ENSG00000120915.13	5	1	0.2
sense_intronic	AC022613.3	ENSG00000259644.1	5	1	0.2
protein_coding	GABRB3	ENSG00000166206.14	5	1	0.2
lincRNA	AP001527.2	ENSG00000277459.1	5	1	0.2
protein_coding	LRRC4	ENSG00000128594.7	5	1	0.2
antisense	DNAJC9-AS1	ENSG00000227540.1	5	1	0.2
lincRNA	AC026367.3	ENSG00000275759.1	5	1	0.2
protein_coding	APOM	ENSG00000204444.10	5	1	0.2
protein_coding	NPAS3	ENSG00000151322.18	5	1	0.2
lincRNA	LINC02447	ENSG00000245468.3	5	1	0.2
protein_coding	RXFP1	ENSG00000171509.15	5	1	0.2
protein_coding	FAM90A1	ENSG00000171847.10	5	1	0.2
protein_coding	VMO1	ENSG00000182853.11	5	1	0.2
antisense	AC087392.1	ENSG00000262003.1	5	1	0.2
antisense	TMEM44-	ENSG00000231770.5	5	1	0.2
protein_coding	IRF9	ENSG00000213928.8	5	1	0.2
lincRNA	AC078850.1	ENSG00000248187.1	5	1	0.2
protein_coding	PTH1R	ENSG00000160801.13	5	1	0.2
protein_coding	CD72	ENSG00000137101.12	5	1	0.2
protein_coding	MYLK4	ENSG00000145949.10	5	1	0.2
protein_coding	JAM2	ENSG00000154721.14	10	2	0.2
protein_coding	NOL12	ENSG00000273899.4	10	2	0.2
lincRNA	AC011445.2	ENSG00000269246.1	10	2	0.2
protein_coding	CHRNB1	ENSG00000170175.10	10	2	0.2
antisense	AC087741.1	ENSG00000262580.5	10	2	0.2
antisense	AC097359.2	ENSG00000270194.1	10	2	0.2
protein_coding	MT1X	ENSG00000187193.8	10	2	0.2
protein_coding	PFKFB2	ENSG00000123836.14	10	2	0.2
protein_coding	GEMIN2	ENSG00000092208.17	10	2	0.2
protein_coding	SOSTDC1	ENSG00000171243.7	10	2	0.2
protein_coding	PRKCZ	ENSG00000067606.16	15	3	0.2
protein_coding	MCM7	ENSG00000166508.17	30	6	0.2

protein_coding	NHSL2	ENSG00000204131.9	40	8	0.2
protein_coding	HES1	ENSG00000114315.3	88	17	0.2
protein_coding	ASPN	ENSG00000106819.11	69	13	0.2
protein_coding	MREG	ENSG00000118242.15	16	3	0.2
protein_coding	RCAN2	ENSG00000172348.14	27	5	0.2
antisense	AC108673.2	ENSG00000273174.1	11	2	0.2
protein_coding	PEX16	ENSG00000121680.15	17	3	0.2
protein_coding	ECSIT	ENSG00000130159.13	23	4	0.2
lincRNA	MIAT	ENSG00000225783.7	35	6	0.2
protein_coding	ZNF799	ENSG00000196466.10	6	1	0.2
protein_coding	ARL17B	ENSG00000228696.8	6	1	0.2
protein_coding	NFE2L3	ENSG00000050344.8	6	1	0.2
lincRNA	AL136038.5	ENSG00000274015.1	6	1	0.2
processed_transcript	HAS2-AS1	ENSG00000248690.7	6	1	0.2
protein_coding	CTSS	ENSG00000163131.10	6	1	0.2
sense_intronic	DLEU2L	ENSG00000116652.6	6	1	0.2
protein_coding	MUC12	ENSG00000205277.9	6	1	0.2
protein_coding	PCOLCE2	ENSG00000163710.8	6	1	0.2
antisense	SERTAD4-	ENSG00000203706.8	6	1	0.2
protein_coding	L1CAM	ENSG00000198910.13	6	1	0.2
antisense	AL021707.2	ENSG00000228274.3	6	1	0.2
antisense	AC007686.3	ENSG00000273729.1	6	1	0.2
antisense	AC034243.1	ENSG00000253404.1	6	1	0.2
antisense	AC089983.1	ENSG00000257732.1	6	1	0.2
lincRNA	LINC02154	ENSG00000235385.1	6	1	0.2
protein_coding	PLXNA2	ENSG00000076356.6	12	2	0.2
antisense	Z97989.1	ENSG00000255389.1	12	2	0.2
protein_coding	INHBE	ENSG00000139269.2	12	2	0.2
protein_coding	PLXDC1	ENSG00000161381.13	61	10	0.2
protein_coding	MAFB	ENSG00000204103.3	19	3	0.2
protein_coding	MFAP4	ENSG00000166482.11	181	28	0.2
protein_coding	MINDY4	ENSG00000106125.14	13	2	0.2
protein_coding	SLC49A3	ENSG00000169026.12	13	2	0.2
protein_coding	CCR10	ENSG00000184451.5	13	2	0.2
protein_coding	BOLA1	ENSG00000178096.8	33	5	0.2
protein_coding	P2RY11	ENSG00000244165.1	7	1	0.1
protein_coding	PYCR3	ENSG00000104524.13	7	1	0.1
processed_pseudogene	RPS29P16	ENSG00000235354.1	7	1	0.1
protein_coding	SLITRK1	ENSG00000178235.7	7	1	0.1
protein_coding	RAD9A	ENSG00000172613.7	7	1	0.1
protein_coding	ECM2	ENSG00000106823.12	7	1	0.1
protein_coding	TSLP	ENSG00000145777.14	7	1	0.1
protein_coding	ISLR	ENSG00000129009.12	7	1	0.1
protein_coding	FBXO36	ENSG00000153832.11	7	1	0.1

sense_intronic	AC011477.2	ENSG00000267481.1	14	2	0.1
Mt_tRNA	MT-TV	ENSG00000210077.1	44	6	0.1
protein_coding	AKR1B10	ENSG00000198074.9	15	2	0.1
protein_coding	CCR1	ENSG00000163823.3	31	4	0.1
lincRNA	AL359764.1	ENSG00000225554.1	39	5	0.1
protein_coding	KDM8	ENSG00000155666.11	8	1	0.1
protein_coding	DNAH5	ENSG00000039139.9	8	1	0.1
antisense	ALOX12-AS1	ENSG00000215067.9	8	1	0.1
antisense	AL450384.2	ENSG00000240291.1	8	1	0.1
protein_coding	MAP3K14	ENSG00000006062.15	8	1	0.1
protein_coding	TRMT44	ENSG00000155275.18	8	1	0.1
lincRNA	AC091182.2	ENSG00000253746.1	8	1	0.1
protein_coding	TTC12	ENSG00000149292.16	8	1	0.1
protein_coding	MCOLN3	ENSG00000055732.12	8	1	0.1
protein_coding	RAB42	ENSG00000188060.7	9	1	0.1
protein_coding	AURKB	ENSG00000178999.12	9	1	0.1
protein_coding	UCN2	ENSG00000145040.3	9	1	0.1
protein_coding	RAP1GAP2	ENSG00000132359.14	9	1	0.1
protein_coding	ARHGAP28	ENSG00000088756.12	9	1	0.1
protein_coding	ADH1B	ENSG00000196616.13	72	8	0.1
misc_RNA	RF00019	ENSG00000200834.1	10	1	0.1
protein_coding	RNFT1	ENSG00000189050.15	10	1	0.1
protein_coding	DISP2	ENSG00000140323.5	10	1	0.1
protein_coding	FCGR2A	ENSG00000143226.13	10	1	0.1
protein_coding	KHDC1	ENSG00000135314.12	10	1	0.1
protein_coding	PRKG2	ENSG00000138669.9	10	1	0.1
protein_coding	PDE1A	ENSG00000115252.18	10	1	0.1
protein_coding	INTS9	ENSG00000104299.14	11	1	0.1
protein_coding	WDR76	ENSG00000092470.11	11	1	0.1
protein_coding	TENT5C	ENSG00000183508.4	11	1	0.1
protein_coding	TMEM201	ENSG00000188807.12	12	1	0.1
protein_coding	SPATA24	ENSG00000170469.10	12	1	0.1
protein_coding	ADCK1	ENSG00000063761.15	12	1	0.1
protein_coding	CYS1	ENSG00000205795.4	13	1	0.1
transcribed_unprocessed_pseudogene	CASP17P	ENSG00000235505.7	13	1	0.1
protein_coding	SPRY3	ENSG00000168939.11	13	1	0.1
protein_coding	TLR3	ENSG00000164342.12	14	1	0.1
protein_coding	LAMP5	ENSG00000125869.9	14	1	0.1
protein_coding	PPL	ENSG00000118898.15	50	3	0.1
protein_coding	PRKAR1B	ENSG00000188191.14	17	1	0.1
protein_coding	OSR1	ENSG00000143867.6	52	3	0.1
protein_coding	HSPB6	ENSG00000004776.12	18	1	0.1

protein_coding	CENPP	ENSG00000188312.13	21	1	0
protein_coding	OMD	ENSG00000127083.7	39	1	0
sense_intronic	AC008147.2	ENSG00000257298.1	1	0	0
protein_coding	BAIAP2L2	ENSG00000128298.16	1	0	0
TEC	AC009951.1	ENSG00000279166.1	1	0	0
protein_coding	NIM1K	ENSG00000177453.7	1	0	0
transcribed_unprocessed_pseudogene	NSUN5P1	ENSG00000223705.9	1	0	0
transcribed_processed_pseudogene	CR382285.1	ENSG00000270533.2	1	0	0
protein_coding	ANKRD36B	ENSG00000196912.12	1	0	0
protein_coding	SLC2A9	ENSG00000109667.11	1	0	0
antisense	AC090198.1	ENSG00000253106.1	1	0	0
sense_intronic	BACH1-IT1	ENSG00000248476.1	1	0	0
lincRNA	AC119674.1	ENSG00000260971.4	1	0	0
protein_coding	SCART1	ENSG00000214279.13	1	0	0
protein_coding	TMCC3	ENSG00000057704.12	1	0	0
protein_coding	CXorf40A	ENSG00000197620.10	1	0	0
protein_coding	ANKAR	ENSG00000151687.14	1	0	0
protein_coding	EDN1	ENSG00000078401.6	1	0	0
antisense	AC004908.3	ENSG00000273402.1	1	0	0
protein_coding	KCNF1	ENSG00000162975.4	1	0	0
protein_coding	VPREB3	ENSG00000128218.7	1	0	0
antisense	AC074032.1	ENSG00000272368.2	1	0	0
lincRNA	AC007389.5	ENSG00000281920.1	1	0	0
protein_coding	ATAD5	ENSG00000176208.8	1	0	0
protein_coding	RAD51AP1	ENSG00000111247.14	1	0	0
protein_coding	ZNF324B	ENSG00000249471.7	1	0	0
protein_coding	SGO1	ENSG00000129810.14	1	0	0
snRNA	RNU7-41P	ENSG00000251726.1	1	0	0
lincRNA	DANT2	ENSG00000235244.3	1	0	0
antisense	PSMD6-AS2	ENSG00000239653.1	1	0	0
protein_coding	IGF2BP1	ENSG00000159217.9	1	0	0
lincRNA	AC069503.1	ENSG00000255856.2	1	0	0
processed_transcript	MIR17HG	ENSG00000215417.12	1	0	0
protein_coding	MRPL46	ENSG00000259494.1	1	0	0
protein_coding	PSMA6	ENSG00000100902.10	1	0	0
protein_coding	SCHIP1	ENSG00000151967.18	1	0	0
misc_RNA	RN7SL452P	ENSG00000243103.3	1	0	0
misc_RNA	RN7SL574P	ENSG00000266075.2	1	0	0
antisense	AC097662.1	ENSG00000236432.7	1	0	0
protein_coding	SYCE1L	ENSG00000205078.5	1	0	0
antisense	AC127070.1	ENSG00000236617.2	1	0	0

lincRNA	BX005019.1	ENSG00000259946.1	1	0	0
lincRNA	AL078604.2	ENSG00000237927.1	1	0	0
protein_coding	HRH2	ENSG00000113749.7	1	0	0
snoRNA	SNORD114-	ENSG00000201247.1	1	0	0
protein_coding	NYAP1	ENSG00000166924.8	1	0	0
protein_coding	FBXL14	ENSG00000171823.6	1	0	0
protein_coding	IL16	ENSG00000172349.17	1	0	0
processed_pseudogene	AC073052.1	ENSG00000228446.2	1	0	0
protein_coding	AUNIP	ENSG00000127423.10	1	0	0
antisense	TMPO-AS1	ENSG00000257167.2	1	0	0
protein_coding	GINS2	ENSG00000131153.8	1	0	0
protein_coding	FAM83D	ENSG00000101447.14	1	0	0
protein_coding	MRPS28	ENSG00000147586.9	1	0	0
processed_pseudogene	AC023161.2	ENSG00000258357.1	1	0	0
protein_coding	RNFT2	ENSG00000135119.14	1	0	0
protein_coding	LPAR2	ENSG00000064547.13	1	0	0
antisense	ATP2C2-AS1	ENSG00000261286.1	1	0	0
protein_coding	RPS6KA1	ENSG00000117676.13	1	0	0
protein_coding	ACSL5	ENSG00000197142.10	1	0	0
protein_coding	PDE9A	ENSG00000160191.17	1	0	0
protein_coding	GNG7	ENSG00000176533.12	1	0	0
protein_coding	CFAP206	ENSG00000272514.5	1	0	0
antisense	AC009509.4	ENSG00000276261.1	1	0	0
sense_intronic	AC124319.1	ENSG00000262979.1	1	0	0
TEC	AC008895.1	ENSG00000279948.1	1	0	0
processed_pseudogene	NDUFA9P1	ENSG00000237406.1	1	0	0
processed_pseudogene	RPL10P6	ENSG00000230076.1	1	0	0
protein_coding	EGFL8	ENSG00000241404.6	1	0	0
lincRNA	AC011815.1	ENSG00000268573.1	1	0	0
transcribed_unitary_pseudogene	DBIL5P	ENSG00000231784.8	1	0	0
protein_coding	RIMS1	ENSG00000079841.18	1	0	0
transcribed_processed_pseudogene	ZNF321P	ENSG00000213801.4	1	0	0
misc_RNA	RF00019	ENSG00000202382.1	1	0	0
sense_intronic	AL391001.1	ENSG00000269887.1	1	0	0
sense_intronic	AC125437.1	ENSG00000267655.1	1	0	0
lincRNA	AL445472.1	ENSG00000227486.1	1	0	0
protein_coding	PPP1R32	ENSG00000162148.10	1	0	0
protein_coding	ZNF490	ENSG00000188033.9	1	0	0
protein_coding	RSPH14	ENSG00000100218.11	1	0	0
processed_pseudogene	AC024995.1	ENSG00000253945.1	1	0	0
TEC	AC073130.3	ENSG00000279086.1	1	0	0

lincRNA	AC055764.2	ENSG00000273375.1	1	0	0
lincRNA	AC005050.1	ENSG00000267052.2	1	0	0
protein_coding	SLC6A17	ENSG00000197106.6	1	0	0
protein_coding	EFHB	ENSG00000163576.17	1	0	0
protein_coding	PCDHGA11	ENSG00000253873.5	1	0	0
protein_coding	SLC13A4	ENSG00000164707.15	1	0	0
protein_coding	MYRF	ENSG00000124920.13	1	0	0
protein_coding	HCRT	ENSG00000161610.1	1	0	0
protein_coding	SPTBN4	ENSG00000160460.15	1	0	0
protein_coding	GATD3B	ENSG00000280071.3	1	0	0
snoRNA	SNORD114-	ENSG00000202048.1	1	0	0
lincRNA	AC009171.2	ENSG00000263105.1	1	0	0
processed_pseudogene	RPL19P16	ENSG00000236762.1	1	0	0
snRNA	RF00004	ENSG00000278774.1	1	0	0
antisense	AC017083.2	ENSG00000273275.1	1	0	0
sense_intronic	AL049840.5	ENSG00000270108.1	1	0	0
processed_transcript	Z92544.2	ENSG00000261659.2	1	0	0
miRNA	MIR103A2	ENSG00000199024.1	1	0	0
protein_coding	TBC1D30	ENSG00000111490.13	1	0	0
protein_coding	SCML2	ENSG00000102098.17	1	0	0
sense_overlapping	AC015849.4	ENSG00000270894.1	1	0	0
misc_RNA	RF00019	ENSG00000200170.1	1	0	0
processed_pseudogene	EEF1B2P6	ENSG00000213261.3	1	0	0
protein_coding	PRR19	ENSG00000188368.9	1	0	0
processed_pseudogene	RPL5P8	ENSG00000213917.2	1	0	0
protein_coding	TIAM1	ENSG00000156299.13	1	0	0
processed_transcript	AC100861.1	ENSG00000246582.2	1	0	0
protein_coding	PIMREG	ENSG00000129195.15	1	0	0
protein_coding	SMG8	ENSG00000167447.12	1	0	0
processed_pseudogene	RPS7P15	ENSG00000237170.3	1	0	0
protein_coding	SMKR1	ENSG00000240204.2	1	0	0
lincRNA	AP000350.6	ENSG00000273295.1	1	0	0
antisense	AC016394.1	ENSG00000272599.2	1	0	0
antisense	AC064807.1	ENSG00000228801.5	1	0	0
processed_pseudogene	AC098591.2	ENSG00000250568.1	1	0	0
protein_coding	GCH1	ENSG00000131979.18	1	0	0
protein_coding	TSPAN1	ENSG00000117472.9	1	0	0
lincRNA	AC073534.1	ENSG00000276030.1	1	0	0
processed_pseudogene	RPS28P7	ENSG00000227097.5	1	0	0
sense_intronic	AC048344.4	ENSG00000277342.1	1	0	0
transcribed_unprocessed_pseudogene	LRRC37A4P	ENSG00000214425.7	1	0	0
protein_coding	ABCA10	ENSG00000154263.17	1	0	0

protein_coding	STXBP2	ENSG00000076944.15	1	0	0
protein_coding	VGF	ENSG00000128564.6	1	0	0
protein_coding	TNIK	ENSG00000154310.16	1	0	0
antisense	AL162258.2	ENSG00000272030.1	1	0	0
antisense	AC009061.2	ENSG00000270049.2	1	0	0
lincRNA	AC007405.3	ENSG00000239467.5	1	0	0
protein_coding	CCDC81	ENSG00000149201.9	1	0	0
transcribed_processed_pseudogene	SLC44A3-AS1	ENSG00000224081.8	1	0	0
lincRNA	LINC01483	ENSG00000227517.6	1	0	0
processed_pseudogene	H3F3AP4	ENSG00000235655.3	1	0	0
protein_coding	CLIC2	ENSG00000155962.12	1	0	0
processed_pseudogene	RPS7P10	ENSG00000226525.5	1	0	0
TEC	AC018628.1	ENSG00000279133.1	1	0	0
processed_pseudogene	AC007182.2	ENSG00000242951.1	1	0	0
protein_coding	ABCG1	ENSG00000160179.18	1	0	0
TEC	AC022413.1	ENSG00000280161.1	1	0	0
processed_pseudogene	MTND5P26	ENSG00000234886.1	1	0	0
TEC	AC069528.2	ENSG00000279320.1	1	0	0
protein_coding	SPAG8	ENSG00000137098.13	1	0	0
antisense	AC044849.1	ENSG00000272256.1	1	0	0
transcribed_unitary_pseudogene	CRYM-AS1	ENSG00000189149.12	1	0	0
protein_coding	HOXC6	ENSG00000197757.7	1	0	0
transcribed_processed_pseudogene	AC079416.1	ENSG00000261056.2	1	0	0
lincRNA	LINC01556	ENSG00000204709.4	1	0	0
protein_coding	ADGRV1	ENSG00000164199.17	1	0	0
protein_coding	RSAD2	ENSG00000134321.11	1	0	0
sense_intronic	AC011503.2	ENSG00000269397.1	1	0	0
antisense	AC026801.2	ENSG00000272323.1	1	0	0
lincRNA	AC004951.1	ENSG00000228434.1	1	0	0
antisense	AC120114.1	ENSG00000247735.2	1	0	0
transcribed_unprocessed_pseudogene	PMS2P3	ENSG00000127957.17	1	0	0
miRNA	MIR1254-1	ENSG00000221184.1	1	0	0
antisense	AL513550.1	ENSG00000228506.1	1	0	0
protein_coding	TCHH	ENSG00000159450.12	1	0	0
lincRNA	AC009159.3	ENSG00000278058.1	1	0	0
antisense	AC009148.1	ENSG00000260495.1	1	0	0
protein_coding	KCNAB3	ENSG00000170049.9	1	0	0
sense_intronic	ACAP2-IT1	ENSG00000229325.1	1	0	0

transcribed_unprocessed_pseudogene	FAM238C	ENSG00000283709.1	1	0	0
antisense	AL358852.1	ENSG00000278899.1	1	0	0
lincRNA	AL139246.5	ENSG00000272449.2	1	0	0
protein_coding	DOCK3	ENSG00000088538.12	1	0	0
protein_coding	CPNE5	ENSG00000124772.11	1	0	0
antisense	HCG14	ENSG00000224157.1	1	0	0
antisense	AC106782.6	ENSG00000274653.1	1	0	0
lincRNA	AC007611.1	ENSG00000260086.2	1	0	0
processed_pseudogene	AC132942.1	ENSG00000240898.1	1	0	0
processed_transcript	SRD5A3-AS1	ENSG00000249700.8	1	0	0
protein_coding	CNTN2	ENSG00000184144.11	1	0	0
protein_coding	IGF1	ENSG00000017427.16	1	0	0
antisense	AC079807.1	ENSG00000233230.1	1	0	0
antisense	IGFBP7-AS1	ENSG00000245067.6	1	0	0
antisense	AP000781.1	ENSG00000254662.1	1	0	0
antisense	Z98885.3	ENSG00000279345.1	1	0	0
bidirectional_promoter_lncRNA	AC104971.1	ENSG00000267226.2	1	0	0
lincRNA	AL031281.3	ENSG00000285752.1	1	0	0
lincRNA	AL451074.6	ENSG00000273365.1	1	0	0
lincRNA	AC133528.1	ENSG00000273113.1	1	0	0
lincRNA	AC008467.1	ENSG00000249476.1	1	0	0
lincRNA	AC022915.1	ENSG00000253455.1	1	0	0
lincRNA	AL162412.1	ENSG00000261447.1	1	0	0
miRNA	MIR199A1	ENSG00000207752.1	1	0	0
misc_RNA	RF00017	ENSG00000277001.1	1	0	0
protein_coding	EMCN	ENSG00000164035.9	1	0	0
protein_coding	CYP7A1	ENSG00000167910.3	1	0	0
protein_coding	KL	ENSG00000133116.7	1	0	0
protein_coding	COX8C	ENSG00000187581.2	1	0	0
protein_coding	CCDC154	ENSG00000197599.12	1	0	0
protein_coding	CD19	ENSG00000177455.12	1	0	0
protein_coding	APLN	ENSG00000171388.11	1	0	0
protein_coding	XPNPEP2	ENSG00000122121.10	1	0	0
scaRNA	SCARNA21	ENSG00000252835.1	1	0	0
sense_intronic	AC009878.1	ENSG00000278840.1	1	0	0
sense_intronic	AL031651.2	ENSG00000277829.1	1	0	0
snoRNA	SNORD116-	ENSG00000207460.1	1	0	0
TEC	AP001148.1	ENSG00000278989.1	1	0	0
TEC	AC025809.2	ENSG00000278875.1	1	0	0
transcribed_unprocessed_pseudogene	POM121L9P	ENSG00000128262.8	1	0	0

protein_coding	U2AF1L4	ENSG00000161265.14	1	0	0
snoRNA	SNORD3A	ENSG00000263934.4	1	0	0
unprocessed_pseudogene	DPY19L1P1	ENSG00000229358.3	1	0	0
processed_pseudogene	AL161909.1	ENSG00000232176.1	1	0	0
protein_coding	LRRD1	ENSG00000240720.8	1	0	0
sense_intronic	AC022558.1	ENSG00000259767.1	1	0	0
sense_intronic	AC022150.4	ENSG00000269825.1	1	0	0
lincRNA	ERVK9-11	ENSG00000269486.2	1	0	0
processed_pseudogene	SNRPEP2	ENSG00000256968.1	1	0	0
processed_pseudogene	TWF1P1	ENSG00000178082.6	1	0	0
processed_pseudogene	EIF4A1P10	ENSG00000229132.2	1	0	0
protein_coding	TRPM8	ENSG00000144481.16	1	0	0
protein_coding	FIGNL2	ENSG00000261308.2	1	0	0
sense_intronic	AC008750.5	ENSG00000268520.1	1	0	0
snoRNA	SNORD123	ENSG00000239112.1	1	0	0
TEC	AC067931.1	ENSG00000279766.1	1	0	0
transcribed_processed_pseudogene	TPI1P2	ENSG00000230359.5	1	0	0
processed_pseudogene	BRD7P2	ENSG00000184100.6	1	0	0
protein_coding	GLDC	ENSG00000178445.9	1	0	0
protein_coding	MAGIX	ENSG00000269313.5	1	0	0
processed_pseudogene	AL136380.1	ENSG00000226499.1	1	0	0
processed_pseudogene	AL359263.1	ENSG00000234130.2	1	0	0
protein_coding	ATXN7	ENSG00000163635.18	1	0	0
protein_coding	CDH24	ENSG00000139880.19	1	0	0
protein_coding	SH2D5	ENSG00000189410.11	1	0	0
antisense	AP001318.2	ENSG00000255062.1	1	0	0
TEC	AC007342.7	ENSG00000279722.1	1	0	0
antisense	AL049795.1	ENSG00000224066.1	1	0	0
lincRNA	AL133215.2	ENSG00000273162.1	1	0	0
protein_coding	NAT8L	ENSG00000185818.7	1	0	0
antisense	PRRT3-AS1	ENSG00000230082.1	1	0	0
protein_coding	GPR19	ENSG00000183150.7	1	0	0
protein_coding	EXO1	ENSG00000174371.16	1	0	0
protein_coding	LYPD6	ENSG00000187123.14	1	0	0
protein_coding	UBE2C	ENSG00000175063.16	1	0	0
protein_coding	MEST	ENSG00000106484.15	1	0	0
protein_coding	NCAPH	ENSG00000121152.9	1	0	0
antisense	AC009118.2	ENSG00000276131.1	1	0	0
protein_coding	ANKRD22	ENSG00000152766.5	1	0	0
protein_coding	RNASEH2A	ENSG00000104889.6	1	0	0
protein_coding	SAPCD2	ENSG00000186193.8	1	0	0
processed_pseudogene	SEC14L1P1	ENSG00000213693.4	1	0	0

protein_coding	FGF12	ENSG00000114279.13	1	0	0
transcribed_unprocessed_pseudogene	FKBP9P1	ENSG00000176826.15	1	0	0
protein_coding	BUB1B	ENSG00000156970.12	1	0	0
sense_intronic	AC004241.3	ENSG00000276390.1	1	0	0
protein_coding	KLK7	ENSG00000169035.11	1	0	0
lincRNA	AP003086.1	ENSG00000251323.2	1	0	0
antisense	AC022079.2	ENSG00000278733.1	1	0	0
protein_coding	NXF3	ENSG00000147206.16	1	0	0
protein_coding	GJB3	ENSG00000188910.7	1	0	0
protein_coding	SBK1	ENSG00000188322.4	1	0	0
lincRNA	AC098614.4	ENSG00000271943.1	1	0	0
sense_overlapping	AL035458.2	ENSG00000250917.1	1	0	0
transcribed_unprocessed_pseudogene	CYP2B7P	ENSG00000256612.7	1	0	0
protein_coding	ZNF658	ENSG00000274349.4	1	0	0
protein_coding	TINAGL1	ENSG00000142910.15	1	0	0
protein_coding	TTC9	ENSG00000133985.2	1	0	0
protein_coding	LRRIQ1	ENSG00000133640.19	1	0	0
sense_intronic	AC026124.2	ENSG00000276853.1	1	0	0
processed_pseudogene	RPS29P5	ENSG00000230777.1	1	0	0
protein_coding	P2RX5-TAX1BP3	ENSG00000257950.3	1	0	0
lincRNA	LINC01637	ENSG00000237476.1	1	0	0
processed_pseudogene	RPS21P4	ENSG00000242358.1	1	0	0
processed_pseudogene	AL355032.1	ENSG00000241494.1	1	0	0
protein_coding	PARM1	ENSG00000169116.11	1	0	0
protein_coding	ZCCHC18	ENSG00000166707.10	1	0	0
processed_pseudogene	AC007256.1	ENSG00000213090.2	1	0	0
antisense	AL136531.1	ENSG00000229728.1	1	0	0
protein_coding	ST8SIA4	ENSG00000113532.12	1	0	0
protein_coding	RTN4R	ENSG00000040608.13	1	0	0
sense_overlapping	AC019080.1	ENSG00000213963.6	1	0	0
lincRNA	AC137630.4	ENSG00000273211.1	1	0	0
sense_intronic	AC139100.2	ENSG00000278000.1	1	0	0
protein_coding	RAB4B	ENSG00000167578.17	1	0	0
bidirectional_promoter_lncRNA	AC104162.2	ENSG00000285399.2	1	0	0
processed_transcript	AC004477.1	ENSG00000263412.1	1	0	0
processed_pseudogene	RPL10P9	ENSG00000233913.7	1	0	0
antisense	DIAPH2-AS1	ENSG00000236256.9	1	0	0
antisense	RGS5	ENSG00000232995.7	1	0	0
protein_coding	KCNJ15	ENSG00000157551.18	1	0	0

protein_coding	ZIC2	ENSG00000043355.11	1	0	0
protein_coding	S1PR5	ENSG00000180739.13	1	0	0
protein_coding	ENPP5	ENSG00000112796.9	1	0	0
processed_transcript	LINC01232	ENSG00000280734.2	1	0	0
protein_coding	TBX18	ENSG00000112837.16	1	0	0
protein_coding	P2RY6	ENSG00000171631.14	1	0	0
protein_coding	FAM53A	ENSG00000174137.12	1	0	0
lincRNA	AL035563.1	ENSG00000273148.1	1	0	0
protein_coding	FOXA1	ENSG00000129514.5	1	0	0
lincRNA	LINC01990	ENSG00000273125.1	1	0	0
lincRNA	AC023813.3	ENSG00000260052.1	1	0	0
processed_pseudogene	RPL13AP7	ENSG00000213885.3	1	0	0
snRNA	RNU6-665P	ENSG00000207369.1	1	0	0
protein_coding	SLC16A13	ENSG00000174327.6	1	0	0
antisense	AC084125.4	ENSG00000265393.1	1	0	0
protein_coding	ARIH2OS	ENSG00000221883.3	1	0	0
sense_intronic	AC068790.3	ENSG00000269997.1	1	0	0
antisense	AL662844.4	ENSG00000272501.1	1	0	0
protein_coding	NDOR1	ENSG00000188566.13	1	0	0
TEC	AL353763.1	ENSG00000279456.1	1	0	0
lincRNA	AL080250.1	ENSG00000225793.2	1	0	0
protein_coding	C2orf73	ENSG00000177994.15	1	0	0
transcribed_unprocessed_pseudogene	FAM86JP	ENSG00000171084.15	1	0	0
protein_coding	ENTPD2	ENSG00000054179.11	1	0	0
protein_coding	MAST1	ENSG00000105613.9	1	0	0
protein_coding	TACSTD2	ENSG00000184292.6	1	0	0
lincRNA	AL354707.1	ENSG00000225489.6	1	0	0
lincRNA	LINC00910	ENSG00000188825.13	1	0	0
protein_coding	FGFR2	ENSG00000066468.22	1	0	0
protein_coding	PLEKHG6	ENSG00000008323.15	1	0	0
lincRNA	MIR181A1H	ENSG00000229989.3	1	0	0
protein_coding	SFN	ENSG00000175793.11	1	0	0
protein_coding	RHOV	ENSG00000104140.6	1	0	0
protein_coding	AC118553.2	ENSG00000283761.1	1	0	0
processed_pseudogene	NONOP2	ENSG00000237522.1	1	0	0
protein_coding	RALGPS1	ENSG00000136828.18	1	0	0
protein_coding	SORCS2	ENSG00000184985.16	1	0	0
protein_coding	SLC52A1	ENSG00000132517.15	1	0	0
protein_coding	C17orf99	ENSG00000187997.11	1	0	0
protein_coding	SDK2	ENSG00000069188.16	1	0	0
protein_coding	KBTBD8	ENSG00000163376.11	1	0	0
protein_coding	SAA1	ENSG00000173432.11	1	0	0

transcribed_unprocessed_pseudogene	ABCC6P2	ENSG00000255277.3	1	0	0
bidirectional_promoter_lncRNA	AL355499.1	ENSG00000232234.3	1	0	0
antisense	AC009318.4	ENSG00000275476.1	1	0	0
protein_coding	NOD2	ENSG00000167207.13	1	0	0
sense_intronic	AC018809.1	ENSG00000269894.1	1	0	0
sense_intronic	AC091769.2	ENSG00000282915.1	1	0	0
snRNA	RNU7-40P	ENSG00000252206.1	1	0	0
protein_coding	SCN2A	ENSG00000136531.15	1	0	0
protein_coding	ELF3	ENSG00000163435.15	1	0	0
antisense	AL365181.3	ENSG00000272405.1	1	0	0
lincRNA	AC110769.2	ENSG00000270019.1	1	0	0
lincRNA	AC008764.6	ENSG00000269399.2	1	0	0
antisense	AL136038.3	ENSG00000261242.1	1	0	0
protein_coding	CEL	ENSG00000170835.14	1	0	0
lincRNA	AP000911.1	ENSG00000204241.7	1	0	0
antisense	MGC16275	ENSG00000246731.2	1	0	0
protein_coding	C19orf57	ENSG00000132016.11	1	0	0
lincRNA	AL022344.2	ENSG00000259869.2	1	0	0
processed_pseudogene	GMPSP1	ENSG00000250471.2	1	0	0
protein_coding	MYO7A	ENSG00000137474.20	1	0	0
protein_coding	NHLH2	ENSG00000177551.5	1	0	0
lincRNA	LINC01550	ENSG00000246223.8	1	0	0
antisense	KCTD21-AS1	ENSG00000246174.7	1	0	0
protein_coding	KLHL6	ENSG00000172578.11	1	0	0
lincRNA	AL121895.2	ENSG00000278035.1	1	0	0
protein_coding	FUT2	ENSG00000176920.11	1	0	0
antisense	AL121845.1	ENSG00000229299.2	1	0	0
lincRNA	AL161756.3	ENSG00000284664.1	1	0	0
processed_pseudogene	AL355802.1	ENSG00000219470.1	1	0	0
protein_coding	AKR1B15	ENSG00000227471.8	1	0	0
protein_coding	SMPDL3B	ENSG00000130768.14	1	0	0
lincRNA	AC106782.2	ENSG00000260219.2	1	0	0
protein_coding	RPEL1	ENSG00000235376.5	1	0	0
sense_intronic	AC079336.2	ENSG00000265222.1	1	0	0
sense_intronic	AC132938.2	ENSG00000264812.1	1	0	0
lincRNA	AC026904.2	ENSG00000253688.2	1	0	0
lincRNA	AC023310.4	ENSG00000278626.1	1	0	0
lincRNA	AL121601.1	ENSG00000232412.1	1	0	0
processed_pseudogene	AC092757.1	ENSG00000241640.2	1	0	0
processed_pseudogene	AC026271.1	ENSG00000174977.8	1	0	0
processed_pseudogene	PTP4A2P1	ENSG00000267185.1	1	0	0

processed_pseudogene	AL355309.1	ENSG00000214748.2	1	0	0
protein_coding	CACNA1D	ENSG00000157388.16	1	0	0
protein_coding	AP000646.1	ENSG00000285509.1	1	0	0
antisense	AL158196.1	ENSG00000276968.1	1	0	0
lincRNA	AC012065.4	ENSG00000270100.1	1	0	0
processed_pseudogene	AC010319.1	ENSG00000254503.1	1	0	0
protein_coding	PLEKHD1	ENSG00000175985.9	1	0	0
protein_coding	AC008758.4	ENSG00000268744.1	1	0	0
sense_intronic	AC089999.2	ENSG00000277566.1	1	0	0
antisense	NPHP3-AS1	ENSG00000248724.6	1	0	0
antisense	AC019077.1	ENSG00000214559.3	1	0	0
lincRNA	AC006033.2	ENSG00000272908.1	1	0	0
lincRNA	AC099518.6	ENSG00000278389.1	1	0	0
processed_pseudogene	AL451074.4	ENSG00000230175.1	1	0	0
processed_pseudogene	RPS26P13	ENSG00000227887.1	1	0	0
processed_pseudogene	RPS3AP5	ENSG00000178429.9	1	0	0
processed_pseudogene	AC079601.2	ENSG00000257376.1	1	0	0
processed_pseudogene	AL365475.1	ENSG00000261208.1	1	0	0
processed_pseudogene	AC145285.1	ENSG00000240634.1	1	0	0
processed_pseudogene	AC024619.4	ENSG00000263781.3	1	0	0
processed_pseudogene	RPL23P2	ENSG00000176054.6	1	0	0
processed_transcript	AL031282.2	ENSG00000268575.1	1	0	0
processed_transcript	BMS1P4	ENSG00000271816.1	1	0	0
protein_coding	SLC25A31	ENSG00000151475.5	1	0	0
protein_coding	KCNJ5	ENSG00000120457.11	1	0	0
protein_coding	SPNS2	ENSG00000183018.8	1	0	0
sense_intronic	AC099343.2	ENSG00000270426.1	1	0	0
sense_intronic	AC087301.1	ENSG00000265010.1	1	0	0
TEC	AC015813.5	ENSG00000279069.1	1	0	0
protein_coding	VWA7	ENSG00000204396.10	1	0	0
protein_coding	ANGPT2	ENSG00000091879.13	1	0	0
processed_pseudogene	RPS29P9	ENSG00000223433.1	1	0	0
processed_pseudogene	AC025458.1	ENSG00000249774.1	1	0	0
processed_pseudogene	AC018644.1	ENSG00000229677.1	1	0	0
processed_pseudogene	ATP5PDP4	ENSG00000234925.2	1	0	0
protein_coding	MLXIPL	ENSG00000009950.15	1	0	0
sense_intronic	AC007671.1	ENSG00000274624.1	1	0	0
snRNA	RNU2-70P	ENSG00000222650.1	1	0	0
snRNA	RNU7-47P	ENSG00000251787.1	1	0	0
antisense	AC013468.1	ENSG00000273240.1	1	0	0
antisense	AC109347.1	ENSG00000260526.1	1	0	0
antisense	HCG25	ENSG00000232940.5	1	0	0
antisense	AL356215.1	ENSG00000255521.1	1	0	0
antisense	AC025034.1	ENSG00000258302.2	1	0	0

antisense	AC103740.1	ENSG00000259370.2	1	0	0
lincRNA	AL590644.1	ENSG00000204362.6	1	0	0
lincRNA	AC008972.1	ENSG00000271926.1	1	0	0
lincRNA	AL442128.2	ENSG00000277767.1	1	0	0
lincRNA	AC009093.6	ENSG00000277999.1	1	0	0
misc_RNA	VTRNA1-1	ENSG00000199990.1	1	0	0
processed_pseudogene	AC016700.2	ENSG00000231414.1	1	0	0
processed_pseudogene	RPL9P18	ENSG00000213309.3	1	0	0
processed_pseudogene	AC104843.1	ENSG00000225416.1	1	0	0
processed_pseudogene	AC093014.1	ENSG00000258090.1	1	0	0
protein_coding	ARMH1	ENSG00000198520.11	1	0	0
protein_coding	CDHR4	ENSG00000187492.8	1	0	0
protein_coding	NKX3-2	ENSG00000109705.7	1	0	0
protein_coding	CARD9	ENSG00000187796.14	1	0	0
protein_coding	ZNF233	ENSG00000159915.12	1	0	0
protein_coding	CACNG8	ENSG00000142408.4	1	0	0
protein_coding	ZSCAN5B	ENSG00000197213.9	1	0	0
protein_coding	KLHDC7B	ENSG00000130487.7	1	0	0
sense_intronic	AF230666.2	ENSG00000270137.1	1	0	0
snRNA	RNU6-16P	ENSG00000207113.1	1	0	0
antisense	AL031727.2	ENSG00000235185.1	1	0	0
antisense	AL354864.1	ENSG00000232335.1	1	0	0
antisense	AL590133.1	ENSG00000231073.1	1	0	0
antisense	AL445493.3	ENSG00000275392.1	1	0	0
antisense	AC092809.4	ENSG00000237101.1	1	0	0
antisense	AC013403.2	ENSG00000272148.1	1	0	0
antisense	PKP4-AS1	ENSG00000204380.4	1	0	0
antisense	AC010680.3	ENSG00000271011.1	1	0	0
antisense	AC079354.1	ENSG00000222035.3	1	0	0
antisense	AC097461.1	ENSG00000261428.2	1	0	0
antisense	AC019068.1	ENSG00000233611.3	1	0	0
antisense	DENND6A-	ENSG00000239801.1	1	0	0
antisense	IL12A-AS1	ENSG00000244040.6	1	0	0
antisense	AC116651.1	ENSG00000273133.1	1	0	0
antisense	AC009570.1	ENSG00000272986.1	1	0	0
antisense	AC012640.1	ENSG00000248968.1	1	0	0
antisense	AC116366.1	ENSG00000234290.2	1	0	0
antisense	AC116312.1	ENSG00000272411.1	1	0	0
antisense	AL512380.2	ENSG00000283480.1	1	0	0
antisense	FOXP4-AS1	ENSG00000234753.5	1	0	0
antisense	AC233992.3	ENSG00000272115.1	1	0	0
antisense	AL162231.2	ENSG00000230074.1	1	0	0
antisense	AL161729.2	ENSG00000271314.1	1	0	0
antisense	AL731537.1	ENSG00000223502.1	1	0	0

antisense	AC100771.2	ENSG00000254862.5	1	0	0
antisense	USP30-AS1	ENSG00000256262.1	1	0	0
antisense	PRC1-AS1	ENSG00000258725.1	1	0	0
antisense	AC002550.1	ENSG00000261312.1	1	0	0
antisense	AC018553.2	ENSG00000283689.1	1	0	0
antisense	AC020763.1	ENSG00000260156.1	1	0	0
antisense	AC010536.3	ENSG00000277504.1	1	0	0
antisense	TTC39C-AS1	ENSG00000264745.1	1	0	0
antisense	CACTIN-AS1	ENSG00000226800.5	1	0	0
antisense	AC007292.1	ENSG00000267980.1	1	0	0
antisense	AC011471.2	ENSG00000267563.1	1	0	0
antisense	AC022098.3	ENSG00000267783.1	1	0	0
antisense	AD000090.1	ENSG00000283907.1	1	0	0
antisense	AC010247.2	ENSG00000259436.1	1	0	0
antisense	GEMIN7-AS1	ENSG00000267348.2	1	0	0
antisense	AC005757.1	ENSG00000267044.1	1	0	0
antisense	AL133492.1	ENSG00000275139.1	1	0	0
lincRNA	AL139246.3	ENSG00000228037.1	1	0	0
lincRNA	AC098484.2	ENSG00000234917.2	1	0	0
lincRNA	AL603840.1	ENSG00000234810.3	1	0	0
lincRNA	AC092807.1	ENSG00000230285.1	1	0	0
lincRNA	LINC01750	ENSG00000231437.3	1	0	0
lincRNA	LINC01765	ENSG00000233730.1	1	0	0
lincRNA	AL023495.1	ENSG00000235303.1	1	0	0
lincRNA	AL445228.2	ENSG00000271387.1	1	0	0
lincRNA	AL513283.1	ENSG00000228536.2	1	0	0
lincRNA	AL591885.1	ENSG00000226828.1	1	0	0
lincRNA	AC114810.1	ENSG00000271868.1	1	0	0
lincRNA	AC010904.2	ENSG00000272002.1	1	0	0
lincRNA	AC012508.2	ENSG00000260634.1	1	0	0
lincRNA	LINC01182	ENSG00000250634.5	1	0	0
lincRNA	AC024132.3	ENSG00000251325.1	1	0	0
lincRNA	LINC02511	ENSG00000248869.5	1	0	0
lincRNA	AC034236.3	ENSG00000272265.1	1	0	0
lincRNA	LINC01933	ENSG00000254226.5	1	0	0
lincRNA	LINC01962	ENSG00000248473.1	1	0	0
lincRNA	AL139330.1	ENSG00000285642.1	1	0	0
lincRNA	LINC02519	ENSG00000226194.5	1	0	0
lincRNA	AC009276.1	ENSG00000224375.1	1	0	0
lincRNA	AC023202.1	ENSG00000253891.1	1	0	0
lincRNA	AC016813.1	ENSG00000254321.1	1	0	0
lincRNA	AL353613.1	ENSG00000232211.1	1	0	0
lincRNA	AL138781.2	ENSG00000275329.1	1	0	0
lincRNA	LINC00703	ENSG00000224382.1	1	0	0

lincRNA	AC124276.2	ENSG00000255400.1	1	0	0
lincRNA	AC009549.1	ENSG00000270607.1	1	0	0
lincRNA	AC092111.1	ENSG00000275367.1	1	0	0
lincRNA	AC053513.1	ENSG00000256973.1	1	0	0
lincRNA	AC012464.3	ENSG00000271382.1	1	0	0
lincRNA	AL136295.6	ENSG00000276698.1	1	0	0
lincRNA	LINC01551	ENSG00000186960.10	1	0	0
lincRNA	AL135818.2	ENSG00000260810.1	1	0	0
lincRNA	AC022706.1	ENSG00000267364.1	1	0	0
lincRNA	LINC01970	ENSG00000265692.1	1	0	0
lincRNA	LINC01903	ENSG00000265555.1	1	0	0
lincRNA	LINC00683	ENSG00000266256.1	1	0	0
lincRNA	ZNF236-DT	ENSG00000264278.1	1	0	0
lincRNA	AC007785.3	ENSG00000268401.1	1	0	0
lincRNA	AC005498.1	ENSG00000267224.1	1	0	0
lincRNA	AL139351.1	ENSG00000276923.1	1	0	0
lincRNA	AP001065.1	ENSG00000274225.1	1	0	0
lincRNA	Z95114.4	ENSG00000279927.1	1	0	0
lincRNA	PAR	ENSG00000237531.6	1	0	0
miRNA	MIR571	ENSG00000207642.1	1	0	0
miRNA	MIR4766	ENSG00000266594.1	1	0	0
misc_RNA	RN7SKP247	ENSG00000223026.1	1	0	0
misc_RNA	RF00017	ENSG00000277668.1	1	0	0
misc_RNA	RF00019	ENSG00000207091.1	1	0	0
processed_pseudogene	AL606517.2	ENSG00000234784.2	1	0	0
processed_pseudogene	AL031864.1	ENSG00000213060.4	1	0	0
processed_pseudogene	BX248409.1	ENSG00000225591.2	1	0	0
processed_pseudogene	AL359837.1	ENSG00000229652.1	1	0	0
processed_pseudogene	AL591846.1	ENSG00000224114.1	1	0	0
processed_pseudogene	AL512343.1	ENSG00000270598.1	1	0	0
processed_pseudogene	RPS7P5	ENSG00000217327.3	1	0	0
processed_pseudogene	AL358176.3	ENSG00000228844.1	1	0	0
processed_pseudogene	KRT18P52	ENSG00000237007.4	1	0	0
processed_pseudogene	ASS1P2	ENSG00000223922.1	1	0	0
processed_pseudogene	AC012354.2	ENSG00000231848.1	1	0	0
processed_pseudogene	TXNP5	ENSG00000228236.2	1	0	0
processed_pseudogene	RPL21P32	ENSG00000224019.1	1	0	0
processed_pseudogene	PSMB3P2	ENSG00000235444.2	1	0	0
processed_pseudogene	RPL7P15	ENSG00000240622.1	1	0	0
processed_pseudogene	HADHAP1	ENSG00000251596.1	1	0	0
processed_pseudogene	SERBP1P6	ENSG00000248873.1	1	0	0
processed_pseudogene	AC109486.1	ENSG00000213896.4	1	0	0
processed_pseudogene	RPL3P6	ENSG00000213891.3	1	0	0
processed_pseudogene	AC068657.1	ENSG00000223908.5	1	0	0

processed_pseudogene	RPL5P20	ENSG00000218643.2	1	0	0
processed_pseudogene	AL450405.1	ENSG00000230202.1	1	0	0
processed_pseudogene	RPL36AP26	ENSG00000235828.5	1	0	0
processed_pseudogene	AC020983.1	ENSG00000243099.1	1	0	0
processed_pseudogene	AL354707.3	ENSG00000232946.1	1	0	0
processed_pseudogene	RPS20P24	ENSG00000236862.1	1	0	0
processed_pseudogene	AL353705.4	ENSG00000234819.1	1	0	0
processed_pseudogene	AL137074.1	ENSG00000213669.2	1	0	0
processed_pseudogene	BTBD7P1	ENSG00000203414.2	1	0	0
processed_pseudogene	RPL7AP53	ENSG00000235962.5	1	0	0
processed_pseudogene	PDCL2P2	ENSG00000255058.1	1	0	0
processed_pseudogene	NACAP8	ENSG00000257210.1	1	0	0
processed_pseudogene	AC126615.1	ENSG00000237774.3	1	0	0
processed_pseudogene	AC093023.1	ENSG00000258142.2	1	0	0
processed_pseudogene	RPL31P49	ENSG00000241680.1	1	0	0
processed_pseudogene	RPL23AP67	ENSG00000240991.3	1	0	0
processed_pseudogene	NAP1L4P3	ENSG00000234145.1	1	0	0
processed_pseudogene	ZDHHC20P4	ENSG00000232380.1	1	0	0
processed_pseudogene	RPL18P11	ENSG00000213307.4	1	0	0
processed_pseudogene	AC110588.1	ENSG00000259340.2	1	0	0
processed_pseudogene	RPS3P7	ENSG00000243101.1	1	0	0
processed_pseudogene	AC015911.1	ENSG00000242660.1	1	0	0
processed_pseudogene	MINOS1P2	ENSG00000270714.1	1	0	0
processed_pseudogene	AC011933.1	ENSG00000228007.1	1	0	0
processed_pseudogene	AP000894.1	ENSG00000264179.1	1	0	0
processed_pseudogene	UQCRFS1P1	ENSG00000226085.3	1	0	0
processed_transcript	AC060234.2	ENSG00000233665.8	1	0	0
processed_transcript	AC127526.2	ENSG00000255367.3	1	0	0
processed_transcript	AC129507.1	ENSG00000262061.5	1	0	0
processed_transcript	SOX9-AS1	ENSG00000234899.9	1	0	0
processed_transcript	AC012313.3	ENSG00000268230.5	1	0	0
protein_coding	TNFRSF18	ENSG00000186891.13	1	0	0
protein_coding	C1QTNF12	ENSG00000184163.3	1	0	0
protein_coding	CCDC27	ENSG00000162592.9	1	0	0
protein_coding	SLC25A34	ENSG00000162461.7	1	0	0
protein_coding	CD52	ENSG00000169442.8	1	0	0
protein_coding	RSPO1	ENSG00000169218.13	1	0	0
protein_coding	C1orf162	ENSG00000143110.11	1	0	0
protein_coding	FAM19A3	ENSG00000184599.13	1	0	0
protein_coding	RIIAD1	ENSG00000178796.12	1	0	0
protein_coding	OR10J1	ENSG00000196184.9	1	0	0
protein_coding	CD247	ENSG00000198821.10	1	0	0
protein_coding	MROH9	ENSG00000117501.14	1	0	0
protein_coding	FMO1	ENSG00000010932.16	1	0	0

protein_coding	CRB1	ENSG00000134376.15	1	0	0
protein_coding	ATP6V1G3	ENSG00000151418.11	1	0	0
protein_coding	STON1-GTF2A1L	ENSG00000068781.21	1	0	0
protein_coding	ANKRD53	ENSG00000144031.11	1	0	0
protein_coding	CCDC74B	ENSG00000152076.18	1	0	0
protein_coding	SLC4A10	ENSG00000144290.16	1	0	0
protein_coding	FGD5	ENSG00000154783.11	1	0	0
protein_coding	LRRC3B	ENSG00000179796.12	1	0	0
protein_coding	ASB14	ENSG00000239388.8	1	0	0
protein_coding	MUC20	ENSG00000176945.16	1	0	0
protein_coding	HSD17B13	ENSG00000170509.11	1	0	0
protein_coding	NDST3	ENSG00000164100.8	1	0	0
protein_coding	MYOZ2	ENSG00000172399.5	1	0	0
protein_coding	FSTL5	ENSG00000168843.13	1	0	0
protein_coding	PRLR	ENSG00000113494.16	1	0	0
protein_coding	DMGDH	ENSG00000132837.14	1	0	0
protein_coding	GPR150	ENSG00000178015.4	1	0	0
protein_coding	PLAC8L1	ENSG00000173261.8	1	0	0
protein_coding	SOX30	ENSG00000039600.10	1	0	0
protein_coding	SCGB3A1	ENSG00000161055.3	1	0	0
protein_coding	ZBED9	ENSG00000232040.3	1	0	0
protein_coding	TNXB	ENSG00000168477.19	1	0	0
protein_coding	BMP5	ENSG00000112175.7	1	0	0
protein_coding	CFTR	ENSG00000001626.15	1	0	0
protein_coding	FAM71F1	ENSG00000135248.15	1	0	0
protein_coding	KEL	ENSG00000197993.8	1	0	0
protein_coding	SLCO5A1	ENSG00000137571.10	1	0	0
protein_coding	PGM5	ENSG00000154330.12	1	0	0
protein_coding	MUSK	ENSG00000030304.13	1	0	0
protein_coding	OR1N2	ENSG00000171501.9	1	0	0
protein_coding	MALRD1	ENSG00000204740.10	1	0	0
protein_coding	OGDHL	ENSG00000197444.9	1	0	0
protein_coding	CYP2C8	ENSG00000138115.13	1	0	0
protein_coding	PLEKHS1	ENSG00000148735.14	1	0	0
protein_coding	HBD	ENSG00000223609.9	1	0	0
protein_coding	OR5P2	ENSG00000183303.2	1	0	0
protein_coding	LGALS12	ENSG00000133317.14	1	0	0
protein_coding	ROBO4	ENSG00000154133.14	1	0	0
protein_coding	DDX47	ENSG00000213782.7	1	0	0
protein_coding	TESPA1	ENSG00000135426.15	1	0	0
protein_coding	OTOGL	ENSG00000165899.11	1	0	0
protein_coding	CCDC62	ENSG00000130783.13	1	0	0

protein_coding	KCNH5	ENSG00000140015.19	1	0	0
protein_coding	ADAM20	ENSG00000134007.3	1	0	0
protein_coding	RGS6	ENSG00000182732.17	1	0	0
protein_coding	ESRRB	ENSG00000119715.15	1	0	0
protein_coding	INSYN1	ENSG00000205363.5	1	0	0
protein_coding	AC004754.1	ENSG00000269881.1	1	0	0
protein_coding	EIF3CL	ENSG00000205609.12	1	0	0
protein_coding	ZNF423	ENSG00000102935.11	1	0	0
protein_coding	KCTD19	ENSG00000168676.10	1	0	0
protein_coding	TSNAXIP1	ENSG00000102904.14	1	0	0
protein_coding	TRPV3	ENSG00000167723.14	1	0	0
protein_coding	CRYBA1	ENSG00000108255.7	1	0	0
protein_coding	DCC	ENSG00000187323.11	1	0	0
protein_coding	TMPRSS9	ENSG00000178297.13	1	0	0
protein_coding	ICAM4	ENSG00000105371.9	1	0	0
protein_coding	CCDC151	ENSG00000198003.11	1	0	0
protein_coding	PSG11	ENSG00000243130.7	1	0	0
protein_coding	HSPA12B	ENSG00000132622.10	1	0	0
protein_coding	REM1	ENSG00000088320.3	1	0	0
protein_coding	TLDC2	ENSG00000101342.9	1	0	0
protein_coding	KCNK15	ENSG00000124249.6	1	0	0
protein_coding	CASS4	ENSG00000087589.16	1	0	0
protein_coding	FTCD	ENSG00000160282.13	1	0	0
protein_coding	CCDC116	ENSG00000161180.10	1	0	0
protein_coding	SMC1B	ENSG00000077935.16	1	0	0
protein_coding	KLHL34	ENSG00000185915.5	1	0	0
protein_coding	FOXP3	ENSG00000049768.14	1	0	0
rRNA_pseudogene	RNA5SP464	ENSG00000252060.1	1	0	0
sense_intronic	AC026316.3	ENSG00000244738.1	1	0	0
sense_intronic	AF131215.5	ENSG00000255310.2	1	0	0
sense_intronic	AC104232.2	ENSG00000254330.1	1	0	0
sense_intronic	AC073569.3	ENSG00000277130.1	1	0	0
sense_intronic	AL138955.1	ENSG00000276809.1	1	0	0
sense_intronic	AC124312.4	ENSG00000270704.3	1	0	0
sense_intronic	AC012676.4	ENSG00000277440.1	1	0	0
sense_intronic	AC009090.1	ENSG00000260038.1	1	0	0
sense_intronic	AC092332.1	ENSG00000260064.1	1	0	0
sense_intronic	AC099811.1	ENSG00000236194.3	1	0	0
sense_intronic	AC090236.2	ENSG00000267504.1	1	0	0
sense_intronic	AC006213.5	ENSG00000278492.1	1	0	0
sense_intronic	AC093503.1	ENSG00000268423.4	1	0	0
snRNA	RNU6-360P	ENSG00000212246.1	1	0	0
snRNA	RNU7-110P	ENSG00000252700.1	1	0	0
snRNA	RNU6-32P	ENSG00000206675.1	1	0	0

snRNA	RNU6-388P	ENSG00000252821.1	1	0	0
snRNA	RNU6-79P	ENSG00000199381.1	1	0	0
snRNA	RNU6-30P	ENSG00000207291.1	1	0	0
TEC	AL356289.3	ENSG00000279096.2	1	0	0
TEC	AC012020.2	ENSG00000284828.1	1	0	0
TEC	AC041040.1	ENSG00000279881.1	1	0	0
TEC	AC016397.2	ENSG00000279822.1	1	0	0
TEC	AC020951.1	ENSG00000278897.1	1	0	0
TEC	AC093503.3	ENSG00000279161.1	1	0	0
transcribed_processed_pseudogene	SELENOKP3	ENSG00000225549.3	1	0	0
transcribed_processed_pseudogene	ATF4P4	ENSG00000256167.1	1	0	0
transcribed_processed_pseudogene	MAP2K4P1	ENSG00000269904.2	1	0	0
transcribed_unprocessed_pseudogene	TFP1	ENSG00000242337.5	1	0	0
transcribed_unprocessed_pseudogene	MRPS31P5	ENSG00000243406.6	1	0	0
transcribed_unprocessed_pseudogene	METTL21EP	ENSG00000250878.3	1	0	0
transcribed_unprocessed_pseudogene	ALOX15P1	ENSG00000274114.2	1	0	0
unprocessed_pseudogene	SUGT1P1	ENSG00000226823.1	1	0	0
unprocessed_pseudogene	CR848007.1	ENSG00000170165.5	1	0	0
protein_coding	STATH	ENSG00000126549.9	1	0	0
protein_coding	PCDHB4	ENSG00000081818.3	1	0	0
protein_coding	CLN3	ENSG00000188603.19	1	0	0
antisense	AC012358.3	ENSG00000240401.8	1	0	0
antisense	AL136131.3	ENSG00000272114.1	1	0	0
antisense	AC034111.1	ENSG00000260093.1	1	0	0
antisense	AC007485.1	ENSG00000275710.1	1	0	0
lincRNA	LINC02256	ENSG00000261064.1	1	0	0
processed_pseudogene	SETP14	ENSG00000240489.1	1	0	0
processed_pseudogene	AC009220.1	ENSG00000228360.1	1	0	0
processed_pseudogene	MRPL40P1	ENSG00000256037.1	1	0	0
protein_coding	SLC22A1	ENSG00000175003.13	1	0	0
protein_coding	XKR9	ENSG00000221947.7	1	0	0
snRNA	RNU6-925P	ENSG00000207359.1	1	0	0
antisense	AC008937.3	ENSG00000271828.1	1	0	0
antisense	AL606469.1	ENSG00000224215.1	1	0	0
antisense	AC006942.1	ENSG00000269194.1	1	0	0
lincRNA	AC005592.1	ENSG00000261757.1	1	0	0

lincRNA	AC083805.1	ENSG00000245651.2	1	0	0
processed_pseudogene	AL445433.1	ENSG00000227034.1	1	0	0
processed_pseudogene	AC104333.2	ENSG00000236317.1	1	0	0
processed_pseudogene	AC113367.1	ENSG00000244192.1	1	0	0
processed_pseudogene	CBX3P9	ENSG00000217241.1	1	0	0
processed_pseudogene	ARL6IP1P2	ENSG00000225355.1	1	0	0
processed_pseudogene	NDUFB8P2	ENSG00000270264.1	1	0	0
processed_pseudogene	RPL9P7	ENSG00000238103.4	1	0	0
processed_pseudogene	GNG5P2	ENSG00000133136.4	1	0	0
processed_pseudogene	XRCC6P2	ENSG00000234825.3	1	0	0
protein_coding	PRKN	ENSG00000185345.20	1	0	0
protein_coding	AJM1	ENSG00000232434.2	1	0	0
protein_coding	SPTBN5	ENSG00000137877.9	1	0	0
protein_coding	CPT1B	ENSG00000205560.12	1	0	0
sense_intronic	AC068790.5	ENSG00000270061.1	1	0	0
sense_intronic	AP000864.1	ENSG00000272788.1	1	0	0
TEC	AC092692.1	ENSG00000279349.1	1	0	0
unprocessed_pseudogene	EIF1P5	ENSG00000266563.1	1	0	0
misc_RNA	RF00017	ENSG00000275070.1	1	0	0
protein_coding	FBXL6	ENSG00000182325.10	1	0	0
protein_coding	NR2E3	ENSG00000278570.4	1	0	0
TEC	AL132780.5	ENSG00000280129.1	1	0	0
antisense	AC093157.2	ENSG00000235795.1	1	0	0
antisense	RASGRF2-	ENSG00000251450.1	1	0	0
antisense	AP001468.1	ENSG00000228404.1	1	0	0
lincRNA	AP003392.4	ENSG00000255121.2	1	0	0
processed_pseudogene	AC008065.1	ENSG00000224553.1	1	0	0
protein_coding	PTPRC	ENSG00000081237.19	1	0	0
protein_coding	C10orf67	ENSG00000179133.13	1	0	0
sense_overlapping	AC008770.3	ENSG00000267274.1	1	0	0
TEC	AC020917.4	ENSG00000280332.1	1	0	0
protein_coding	ZBTB8B	ENSG00000273274.1	1	0	0
transcribed_unprocessed_pseudogene	STAG3L5P	ENSG00000242294.6	1	0	0
antisense	AL512408.1	ENSG00000260063.1	1	0	0
transcribed_unprocessed_pseudogene	AC012618.3	ENSG00000234773.7	1	0	0
protein_coding	RET	ENSG00000165731.18	1	0	0
sense_intronic	AF129075.1	ENSG00000231125.2	1	0	0
transcribed_processed_pseudogene	RPL26P30	ENSG00000236264.5	1	0	0
protein_coding	SH3GL3	ENSG00000140600.16	1	0	0
antisense	AC231981.1	ENSG00000235078.1	1	0	0

lincRNA	HCG27	ENSG00000206344.7	1	0	0
protein_coding	TRAIP	ENSG00000183763.8	1	0	0
protein_coding	ZNF235	ENSG00000159917.16	2	0	0
protein_coding	DENND2C	ENSG00000175984.14	2	0	0
antisense	C1RL-AS1	ENSG00000205885.7	2	0	0
lincRNA	PCF11-AS1	ENSG00000269939.1	2	0	0
protein_coding	PIGA	ENSG00000165195.15	2	0	0
antisense	ADAMTS9-	ENSG00000241684.6	2	0	0
antisense	AL441883.1	ENSG00000261071.1	2	0	0
protein_coding	DTL	ENSG00000143476.17	2	0	0
antisense	AC007666.1	ENSG00000236754.5	2	0	0
protein_coding	SCUBE2	ENSG00000175356.13	2	0	0
protein_coding	XRCC3	ENSG00000126215.13	2	0	0
protein_coding	POLD1	ENSG00000062822.13	2	0	0
protein_coding	ARHGAP44	ENSG00000006740.16	2	0	0
protein_coding	OAS1	ENSG00000089127.12	2	0	0
processed_pseudogene	AC006042.3	ENSG00000233264.2	2	0	0
protein_coding	PCSK1	ENSG00000175426.10	2	0	0
transcribed_processed_pseudogene	FTH1P10	ENSG00000223361.5	2	0	0
protein_coding	FTCDNL1	ENSG00000226124.7	2	0	0
protein_coding	AC098650.1	ENSG00000283563.1	2	0	0
sense_overlapping	AC005674.2	ENSG00000261490.1	2	0	0
sense_intronic	AC026356.1	ENSG00000274964.1	2	0	0
processed_pseudogene	AL035458.1	ENSG00000236456.1	2	0	0
protein_coding	ZNF443	ENSG00000180855.15	2	0	0
antisense	BDNF-AS	ENSG00000245573.7	2	0	0
protein_coding	SLC12A5	ENSG00000124140.13	2	0	0
processed_pseudogene	CKS1BP1	ENSG00000258788.3	2	0	0
protein_coding	KNDC1	ENSG00000171798.17	2	0	0
lincRNA	AC022929.2	ENSG00000274281.1	2	0	0
protein_coding	KRT33B	ENSG00000131738.10	2	0	0
protein_coding	ZSCAN1	ENSG00000152467.9	2	0	0
protein_coding	NDUFV2	ENSG00000178127.12	2	0	0
lincRNA	AC129492.4	ENSG00000266824.1	2	0	0
processed_pseudogene	DDX10P1	ENSG00000237135.1	2	0	0
protein_coding	NPIPB4	ENSG00000185864.16	2	0	0
antisense	AC091133.1	ENSG00000230532.1	2	0	0
protein_coding	GPR39	ENSG00000183840.6	2	0	0
misc_RNA	RF00017	ENSG00000277233.1	2	0	0
protein_coding	LCA5L	ENSG00000157578.13	2	0	0
protein_coding	CCZ1B	ENSG00000146574.15	2	0	0
antisense	FUT8-AS1	ENSG00000276116.2	2	0	0

processed_transcript	FAM106A	ENSG00000273018.6	2	0	0
TEC	AL928654.4	ENSG00000279495.1	2	0	0
transcribed_unprocessed_pseudogene	AC138393.1	ENSG00000185495.10	2	0	0
sense_overlapping	AC007066.2	ENSG00000261094.2	2	0	0
miRNA	MIR320D1	ENSG00000211491.2	2	0	0
lincRNA	AC015712.1	ENSG00000232386.9	2	0	0
protein_coding	NPAS1	ENSG00000130751.9	2	0	0
protein_coding	TXK	ENSG00000074966.10	2	0	0
TEC	AC026471.6	ENSG00000280132.1	2	0	0
antisense	AL109741.1	ENSG00000225938.1	2	0	0
protein_coding	UBQLNL	ENSG00000175518.6	2	0	0
protein_coding	LINC00672	ENSG00000263874.2	2	0	0
protein_coding	HES7	ENSG00000179111.8	2	0	0
lincRNA	AC116447.1	ENSG00000274918.1	2	0	0
protein_coding	CORIN	ENSG00000145244.11	2	0	0
protein_coding	NDNF	ENSG00000173376.13	2	0	0
protein_coding	NRCAM	ENSG00000091129.19	2	0	0
processed_pseudogene	EEF1A1P29	ENSG00000215093.3	2	0	0
protein_coding	SULT1A1	ENSG00000196502.11	2	0	0
protein_coding	RPL17	ENSG00000265681.7	2	0	0
antisense	MED14OS	ENSG00000234636.2	2	0	0
protein_coding	TCP10L	ENSG00000242220.6	2	0	0
antisense	AC127024.6	ENSG00000276250.1	2	0	0
antisense	AC022916.1	ENSG00000266947.1	2	0	0
lincRNA	LINC00460	ENSG00000233532.6	2	0	0
processed_pseudogene	COX20P1	ENSG00000213025.2	2	0	0
protein_coding	RAD54L	ENSG00000085999.11	2	0	0
processed_pseudogene	AL031729.1	ENSG00000235912.1	2	0	0
protein_coding	ZNF485	ENSG00000198298.12	2	0	0
protein_coding	TIMP4	ENSG00000157150.4	2	0	0
protein_coding	RAD54B	ENSG00000197275.13	2	0	0
protein_coding	TNFSF15	ENSG00000181634.7	2	0	0
TEC	AC005831.1	ENSG00000280202.1	2	0	0
antisense	PTOV1-AS1	ENSG00000268006.1	2	0	0
processed_pseudogene	AC000089.1	ENSG00000235776.2	2	0	0
TEC	AC011498.7	ENSG00000280239.1	2	0	0
protein_coding	TNFRSF25	ENSG00000215788.10	2	0	0
protein_coding	DEC1	ENSG00000173077.15	2	0	0
processed_pseudogene	AC084824.1	ENSG00000257511.1	2	0	0
protein_coding	FOXL2	ENSG00000183770.6	2	0	0
TEC	AC002044.2	ENSG00000279591.1	2	0	0
protein_coding	POMZP3	ENSG00000146707.14	2	0	0

protein_coding	VWF	ENSG00000110799.13	2	0	0
protein_coding	ZNF774	ENSG00000196391.10	2	0	0
protein_coding	DTWD2	ENSG00000169570.9	2	0	0
snRNA	RNU1-67P	ENSG00000207175.1	2	0	0
antisense	AC016590.3	ENSG00000276846.1	2	0	0
processed_pseudogene	RPSAP54	ENSG00000213621.3	2	0	0
protein_coding	MYLPF	ENSG00000180209.11	2	0	0
transcribed_unprocessed_pseudogene	AC068620.3	ENSG00000283156.1	2	0	0
protein_coding	ZNF214	ENSG00000149050.9	2	0	0
antisense	AC022075.1	ENSG00000245648.1	2	0	0
protein_coding	NECTIN4	ENSG00000143217.8	2	0	0
protein_coding	CSRNP3	ENSG00000178662.15	2	0	0
antisense	AC126118.1	ENSG00000271993.1	2	0	0
lincRNA	AC135803.1	ENSG00000272864.1	2	0	0
lincRNA	AC092653.1	ENSG00000273245.1	2	0	0
lincRNA	AC026367.1	ENSG00000270482.1	2	0	0
processed_pseudogene	FAM133DP	ENSG00000230562.3	2	0	0
lincRNA	AC005899.6	ENSG00000274341.1	2	0	0
transcribed_unprocessed_pseudogene	HERC2P3	ENSG00000180229.12	2	0	0
processed_pseudogene	AC013701.1	ENSG00000233109.4	2	0	0
lincRNA	AC091057.4	ENSG00000269974.1	2	0	0
protein_coding	RWDD3	ENSG00000122481.16	2	0	0
protein_coding	CRYM	ENSG00000103316.10	2	0	0
antisense	UPK1A-AS1	ENSG00000226510.1	2	0	0
unprocessed_pseudogene	SMG1P2	ENSG00000205534.6	2	0	0
sense_intronic	AC020915.2	ENSG00000268201.1	2	0	0
unprocessed_pseudogene	MTND2P41	ENSG00000283131.1	2	0	0
antisense	AC234582.1	ENSG00000231064.7	2	0	0
antisense	AL513217.1	ENSG00000223774.5	2	0	0
antisense	AC026992.2	ENSG00000261634.3	2	0	0
lincRNA	AL136382.1	ENSG00000285210.1	2	0	0
lincRNA	AC122136.1	ENSG00000227824.1	2	0	0
lincRNA	LINC02458	ENSG00000246363.2	2	0	0
lincRNA	AC004156.1	ENSG00000267666.2	2	0	0
processed_pseudogene	AC019206.1	ENSG00000275381.1	2	0	0
protein_coding	PPFIA4	ENSG00000143847.15	2	0	0
protein_coding	CD74	ENSG00000019582.14	2	0	0
protein_coding	OR1Q1	ENSG00000165202.3	2	0	0
protein_coding	HCN2	ENSG00000099822.2	2	0	0
protein_coding	ANKLE1	ENSG00000160117.14	2	0	0
protein_coding	ZNF285	ENSG00000267508.5	2	0	0

protein_coding	KIAA1755	ENSG00000149633.11	2	0	0
protein_coding	SYN1	ENSG00000008056.13	2	0	0
sense_intronic	AC026271.3	ENSG00000264885.1	2	0	0
TEC	AC068880.4	ENSG00000279858.1	2	0	0
sense_intronic	N4BP2L2-IT2	ENSG00000281026.1	2	0	0
protein_coding	ACY1	ENSG00000243989.8	2	0	0
protein_coding	DNASE1L2	ENSG00000167968.12	2	0	0
sense_intronic	WWTR1-IT1	ENSG00000241985.1	2	0	0
lincRNA	AL359397.2	ENSG00000259071.1	2	0	0
protein_coding	TPPP3	ENSG00000159713.10	2	0	0
lincRNA	AC104836.1	ENSG00000273487.1	2	0	0
processed_pseudogene	EIF2S2P3	ENSG00000236493.2	2	0	0
transcribed_processed_pseudogene	AF274858.1	ENSG00000260081.2	2	0	0
antisense	AC093388.1	ENSG00000272979.1	2	0	0
protein_coding	ZXDA	ENSG00000198205.6	2	0	0
protein_coding	CD177	ENSG00000204936.9	2	0	0
antisense	AC004839.1	ENSG00000272854.1	2	0	0
protein_coding	PCDHGB2	ENSG00000253910.2	2	0	0
protein_coding	ARFGEF3	ENSG00000112379.8	2	0	0
protein_coding	LETM2	ENSG00000165046.12	2	0	0
processed_pseudogene	AC093663.2	ENSG00000243280.2	2	0	0
protein_coding	GRHL1	ENSG00000134317.17	2	0	0
protein_coding	CBX2	ENSG00000173894.10	2	0	0
protein_coding	ST3GAL6	ENSG00000064225.12	2	0	0
protein_coding	UBE2S	ENSG00000108106.13	2	0	0
processed_transcript	IPO5P1	ENSG00000284428.1	2	0	0
sense_intronic	AC022306.3	ENSG00000276593.1	2	0	0
transcribed_processed_pseudogene	AP000919.1	ENSG00000263606.1	2	0	0
lincRNA	AC110285.2	ENSG00000262877.4	2	0	0
processed_pseudogene	AC099336.2	ENSG00000236439.4	2	0	0
processed_transcript	AP005212.4	ENSG00000283294.1	2	0	0
protein_coding	RBP7	ENSG00000162444.11	2	0	0
protein_coding	WAS	ENSG00000015285.10	2	0	0
protein_coding	DAND5	ENSG00000179284.5	2	0	0
sense_overlapping	AL390195.1	ENSG00000243960.1	2	0	0
protein_coding	SH3RF2	ENSG00000156463.17	2	0	0
antisense	HOTAIR	ENSG00000228630.5	2	0	0
protein_coding	LMO2	ENSG00000135363.11	2	0	0
protein_coding	FAM183A	ENSG00000186973.10	2	0	0
processed_pseudogene	SNRPEP4	ENSG00000233270.1	2	0	0
protein_coding	SCAMP5	ENSG00000198794.11	2	0	0

protein_coding	CNTN1	ENSG00000018236.14	2	0	0
protein_coding	CDK5R2	ENSG00000171450.5	2	0	0
protein_coding	CDA	ENSG00000158825.5	2	0	0
protein_coding	FRMD5	ENSG00000171877.20	2	0	0
protein_coding	NDRG2	ENSG00000165795.23	2	0	0
protein_coding	AL136295.5	ENSG00000259529.1	2	0	0
processed_transcript	AC068733.3	ENSG00000282556.2	2	0	0
processed_pseudogene	AL033519.2	ENSG00000219023.1	2	0	0
protein_coding	LRP2BP	ENSG00000109771.15	2	0	0
transcribed_unprocessed_pseudogene	AL133216.2	ENSG00000276805.2	2	0	0
protein_coding	FAM81A	ENSG00000157470.11	2	0	0
protein_coding	AC106886.5	ENSG00000282034.1	2	0	0
protein_coding	CRISPLD1	ENSG00000121005.8	2	0	0
protein_coding	SYCP2L	ENSG00000153157.12	2	0	0
sense_intronic	AC009996.1	ENSG00000259659.1	2	0	0
antisense	SRRM2-AS1	ENSG00000205913.6	2	0	0
antisense	AC009113.1	ENSG00000259877.2	2	0	0
protein_coding	FAM129C	ENSG00000167483.17	2	0	0
antisense	AL021392.1	ENSG00000234869.1	2	0	0
protein_coding	GPLD1	ENSG00000112293.14	2	0	0
transcribed_unprocessed_pseudogene	GTF2IP13	ENSG00000272556.2	2	0	0
antisense	AC074117.1	ENSG00000234072.1	2	0	0
lincRNA	AC022034.1	ENSG00000237807.3	2	0	0
protein_coding	RND1	ENSG00000172602.10	2	0	0
protein_coding	AL139353.1	ENSG00000203546.7	2	0	0
protein_coding	SP6	ENSG00000189120.4	2	0	0
antisense	AC138123.1	ENSG00000257322.5	2	0	0
lincRNA	AC026250.1	ENSG00000245522.2	2	0	0
protein_coding	PTPRH	ENSG00000080031.9	2	0	0
protein_coding	CPLANE2	ENSG00000132881.11	2	0	0
protein_coding	MAP7D2	ENSG00000184368.15	2	0	0
protein_coding	ZNF286B	ENSG00000249459.8	2	0	0
protein_coding	KSR2	ENSG00000171435.13	2	0	0
lincRNA	SMC2-AS1	ENSG00000270332.1	2	0	0
processed_pseudogene	NPM1P40	ENSG00000236523.2	2	0	0
protein_coding	CFAP43	ENSG00000197748.12	2	0	0
protein_coding	MMP9	ENSG00000100985.7	2	0	0
antisense	SEPT4-AS1	ENSG00000264672.5	2	0	0
lincRNA	AC002470.1	ENSG00000272829.1	2	0	0
processed_pseudogene	NTAN1P2	ENSG00000250569.1	2	0	0
antisense	LENG8-AS1	ENSG00000226696.5	2	0	0

antisense	FAM66C	ENSG00000226711.6	2	0	0
miRNA	MIR7851	ENSG00000278520.1	2	0	0
antisense	LINC00412	ENSG00000234772.1	2	0	0
antisense	AP000350.5	ENSG00000272973.1	2	0	0
lincRNA	AC010997.3	ENSG00000272692.1	2	0	0
lincRNA	LINC00592	ENSG00000258279.3	2	0	0
lincRNA	AC110048.2	ENSG00000277152.1	2	0	0
protein_coding	B3GALT1	ENSG00000172318.5	2	0	0
protein_coding	ACKR2	ENSG00000144648.15	2	0	0
snRNA	RNU6-482P	ENSG00000212370.1	2	0	0
antisense	FAM13A-	ENSG00000248019.2	2	0	0
lincRNA	AC211433.2	ENSG00000273069.1	2	0	0
antisense	AC004877.1	ENSG00000273419.1	2	0	0
antisense	AL020996.1	ENSG00000228172.5	2	0	0
antisense	AC022211.2	ENSG00000263843.1	2	0	0
lincRNA	AC004223.2	ENSG00000267457.1	2	0	0
processed_pseudogene	RPL4P5	ENSG00000230207.1	2	0	0
protein_coding	ZMYND10	ENSG00000004838.13	2	0	0
protein_coding	IFIT1B	ENSG00000204010.3	2	0	0
protein_coding	CSRP3	ENSG00000129170.9	2	0	0
transcribed_unprocessed_pseudogene	PPIEL	ENSG00000243970.3	2	0	0
antisense	LINC01389	ENSG00000225762.1	2	0	0
antisense	SPATA42	ENSG00000203897.3	2	0	0
antisense	AL591846.2	ENSG00000237605.1	2	0	0
antisense	LGALS8-AS1	ENSG00000223776.5	2	0	0
antisense	AC007743.1	ENSG00000233251.7	2	0	0
antisense	UBE2E1-AS1	ENSG00000223791.1	2	0	0
antisense	NECTIN3-	ENSG00000242242.5	2	0	0
antisense	AC117490.2	ENSG00000273013.1	2	0	0
antisense	AC107464.3	ENSG00000272927.1	2	0	0
antisense	AC008522.1	ENSG00000279232.2	2	0	0
antisense	AL032821.1	ENSG00000234484.1	2	0	0
antisense	AP000880.1	ENSG00000255129.5	2	0	0
antisense	AC083805.3	ENSG00000273805.1	2	0	0
antisense	AL355102.4	ENSG00000258793.1	2	0	0
antisense	AC022613.1	ENSG00000256802.2	2	0	0
antisense	AC092376.3	ENSG00000285918.1	2	0	0
antisense	AC125257.2	ENSG00000274630.1	2	0	0
lincRNA	AC082651.1	ENSG00000240687.1	2	0	0
lincRNA	LINC01238	ENSG00000237940.3	2	0	0
lincRNA	LINC02030	ENSG00000240708.1	2	0	0
lincRNA	LINC02021	ENSG00000249846.6	2	0	0

lincRNA	AC109779.1	ENSG00000223930.5	2	0	0
lincRNA	LMNB1-DT	ENSG00000251072.2	2	0	0
lincRNA	AC104109.2	ENSG00000270177.1	2	0	0
lincRNA	AL390955.2	ENSG00000271040.1	2	0	0
lincRNA	LINC01510	ENSG00000231210.3	2	0	0
lincRNA	AL359182.2	ENSG00000230013.1	2	0	0
lincRNA	AL136141.1	ENSG00000270755.1	2	0	0
lincRNA	AC117498.2	ENSG00000257660.5	2	0	0
lincRNA	AC020637.1	ENSG00000258231.5	2	0	0
lincRNA	LMO7DN	ENSG00000178734.5	2	0	0
lincRNA	AC021755.3	ENSG00000275636.1	2	0	0
lincRNA	EWSAT1	ENSG00000212766.9	2	0	0
lincRNA	AC013391.3	ENSG00000261478.1	2	0	0
lincRNA	AC009108.3	ENSG00000270020.1	2	0	0
lincRNA	PPP4R1-AS1	ENSG00000263627.1	2	0	0
lincRNA	AC010624.2	ENSG00000267890.1	2	0	0
lincRNA	LINC01203	ENSG00000226985.6	2	0	0
lincRNA	SPANXA2-	ENSG00000277215.1	2	0	0
processed_pseudogene	AL020997.1	ENSG00000231344.1	2	0	0
processed_pseudogene	AC010731.1	ENSG00000213083.3	2	0	0
processed_pseudogene	FTH1P23	ENSG00000242960.1	2	0	0
processed_pseudogene	AC106872.5	ENSG00000248632.1	2	0	0
processed_pseudogene	AC026444.1	ENSG00000251585.1	2	0	0
processed_pseudogene	AL445529.1	ENSG00000214641.3	2	0	0
processed_pseudogene	RPS27AP13	ENSG00000244159.1	2	0	0
processed_pseudogene	EEF1A1P5	ENSG00000196205.8	2	0	0
processed_pseudogene	DUSP8P5	ENSG00000235316.1	2	0	0
processed_pseudogene	RPL21P106	ENSG00000239201.1	2	0	0
protein_coding	TAS1R3	ENSG00000169962.4	2	0	0
protein_coding	RHCE	ENSG00000188672.18	2	0	0
protein_coding	DMRTA2	ENSG00000142700.11	2	0	0
protein_coding	SHISAL2A	ENSG00000182183.14	2	0	0
protein_coding	NTRK1	ENSG00000198400.11	2	0	0
protein_coding	RGS1	ENSG00000090104.11	2	0	0
protein_coding	B3GALT2	ENSG00000162630.5	2	0	0
protein_coding	SLC30A3	ENSG00000115194.10	2	0	0
protein_coding	TRIM54	ENSG00000138100.13	2	0	0
protein_coding	PLCH1	ENSG00000114805.17	2	0	0
protein_coding	ART3	ENSG00000156219.16	2	0	0
protein_coding	FBLL1	ENSG00000188573.7	2	0	0
protein_coding	TLX3	ENSG00000164438.5	2	0	0
protein_coding	FGF18	ENSG00000156427.7	2	0	0
protein_coding	TMEM244	ENSG00000203756.7	2	0	0
protein_coding	VNN¬†1	ENSG00000112299.7	2	0	0

protein_coding	IPCEF1	ENSG00000074706.13	2	0	0
protein_coding	LRRC4C	ENSG00000148948.7	2	0	0
protein_coding	SPI1	ENSG00000066336.11	2	0	0
protein_coding	AMHR2	ENSG00000135409.10	2	0	0
protein_coding	ANKS1B	ENSG00000185046.18	2	0	0
protein_coding	PDIA2	ENSG00000185615.15	2	0	0
protein_coding	OMG	ENSG00000126861.4	2	0	0
protein_coding	ABI3	ENSG00000108798.8	2	0	0
protein_coding	ZGLP1	ENSG00000220201.7	2	0	0
protein_coding	SCUBE1	ENSG00000159307.18	2	0	0
protein_coding	MAGEA12	ENSG00000213401.9	2	0	0
sense_intronic	AC012653.2	ENSG00000274719.1	2	0	0
sense_overlapping	AP000662.1	ENSG00000254602.1	2	0	0
snoRNA	RF00438	ENSG00000201827.1	2	0	0
snoRNA	RF00012	ENSG00000212539.2	2	0	0
TEC	AC011008.2	ENSG00000280294.1	2	0	0
transcribed_processed_pseudogene	AC006116.2	ENSG00000266932.1	2	0	0
transcribed_unprocessed_pseudogene	CCNYL2	ENSG00000182632.15	2	0	0
transcribed_unprocessed_pseudogene	FAM21FP	ENSG00000237840.6	2	0	0
transcribed_unprocessed_pseudogene	SORD2P	ENSG00000259479.6	2	0	0
transcribed_unprocessed_pseudogene	ABCC6P1	ENSG00000256340.8	2	0	0
transcribed_unprocessed_pseudogene	SMG1P1	ENSG00000237296.9	2	0	0
unprocessed_pseudogene	AC008752.3	ENSG00000267370.1	2	0	0
processed_pseudogene	AC002367.1	ENSG00000226436.1	2	0	0
protein_coding	UPK2	ENSG00000110375.2	2	0	0
processed_pseudogene	RPS27AP11	ENSG00000218208.1	2	0	0
antisense	AC009630.1	ENSG00000253133.1	2	0	0
antisense	AC090673.1	ENSG00000197301.7	2	0	0
lincRNA	AL358472.2	ENSG00000272654.1	2	0	0
processed_pseudogene	AC113398.1	ENSG00000242814.2	2	0	0
antisense	AC109361.1	ENSG00000249635.1	2	0	0
antisense	AC010173.1	ENSG00000258101.2	2	0	0
polymorphic_pseudogene	PKD1L2	ENSG00000166473.17	2	0	0
processed_pseudogene	ST13P15	ENSG00000243759.1	2	0	0
processed_pseudogene	AC020898.1	ENSG00000213757.3	2	0	0
processed_pseudogene	AC005099.1	ENSG00000226624.1	2	0	0
protein_coding	SSC4D	ENSG00000146700.8	2	0	0

protein_coding	DLX6	ENSG00000006377.10	2	0	0
unprocessed_pseudogene	SALL4P5	ENSG00000231915.2	2	0	0
unprocessed_pseudogene	OR7E7P	ENSG00000238228.1	2	0	0
processed_pseudogene	AC002075.2	ENSG00000237729.2	2	0	0
antisense	AL031665.2	ENSG00000276026.1	2	0	0
lincRNA	MIR3681HG	ENSG00000224184.5	2	0	0
misc_RNA	RF00019	ENSG00000222614.1	2	0	0
protein_coding	C20orf144	ENSG00000149609.5	2	0	0
unprocessed_pseudogene	NCOR1P2	ENSG00000265019.1	2	0	0
lincRNA	LINC01315	ENSG00000229891.1	2	0	0
antisense	AC006329.1	ENSG00000232445.1	2	0	0
protein_coding	ITGAX	ENSG00000140678.16	2	0	0
antisense	AC073957.3	ENSG00000273151.1	2	0	0
protein_coding	CLMN	ENSG00000165959.11	3	0	0
lincRNA	AL390957.1	ENSG00000285280.1	3	0	0
protein_coding	CXorf40B	ENSG00000197021.8	3	0	0
protein_coding	ZNF30	ENSG00000168661.14	3	0	0
protein_coding	ANXA3	ENSG00000138772.12	3	0	0
sense_overlapping	AC011468.1	ENSG00000260160.1	3	0	0
protein_coding	ZNF341	ENSG00000131061.13	3	0	0
protein_coding	RAB24	ENSG00000169228.13	3	0	0
transcribed_unprocessed_pseudogene	TPTEP2	ENSG00000244627.5	3	0	0
TEC	AL078621.3	ENSG00000279267.1	3	0	0
protein_coding	CPNE4	ENSG00000196353.11	3	0	0
protein_coding	SSTR1	ENSG00000139874.5	3	0	0
protein_coding	DDIAS	ENSG00000165490.12	3	0	0
protein_coding	C5orf34	ENSG00000172244.8	3	0	0
protein_coding	SGSM3	ENSG00000100359.20	3	0	0
protein_coding	MECOM	ENSG00000085276.17	3	0	0
antisense	SLC25A25-	ENSG00000234771.3	3	0	0
protein_coding	LNX1	ENSG00000072201.13	3	0	0
snRNA	RNU4-2	ENSG00000202538.1	3	0	0
protein_coding	GSTM1	ENSG00000134184.12	3	0	0
protein_coding	KCNQ5	ENSG00000185760.15	3	0	0
protein_coding	BHLHE41	ENSG00000123095.5	3	0	0
processed_pseudogene	FAM3C2	ENSG00000174028.6	3	0	0
protein_coding	FAXC	ENSG00000146267.11	3	0	0
protein_coding	ZNF460	ENSG00000197714.8	3	0	0
antisense	AC024060.1	ENSG00000271870.1	3	0	0
protein_coding	CELSR1	ENSG00000075275.16	3	0	0
protein_coding	DOK3	ENSG00000146094.14	3	0	0
protein_coding	KLRG1	ENSG00000139187.9	3	0	0

sense_intronic	AL122023.1	ENSG00000278396.1	3	0	0
TEC	AC079416.2	ENSG00000279589.1	3	0	0
protein_coding	PIWIL2	ENSG00000197181.11	3	0	0
antisense	TOLLIP-AS1	ENSG00000255153.1	3	0	0
snRNA	RNU5F-1	ENSG00000199377.1	3	0	0
antisense	AC010226.1	ENSG00000249249.1	3	0	0
protein_coding	CHI3L1	ENSG00000133048.12	3	0	0
protein_coding	EGFLAM	ENSG00000164318.17	3	0	0
antisense	AL354811.1	ENSG00000278177.1	3	0	0
bidirectional_promoter_lncRNA	AL359762.1	ENSG00000284882.1	3	0	0
processed_pseudogene	RPS7P1	ENSG00000263266.2	3	0	0
protein_coding	PLCE1	ENSG00000138193.15	3	0	0
protein_coding	NOG	ENSG00000183691.4	3	0	0
antisense	AC096677.1	ENSG00000224536.2	3	0	0
protein_coding	LPAR4	ENSG00000147145.12	3	0	0
antisense	DHCR24-DT	ENSG00000233203.6	3	0	0
sense_intronic	FOXP1-IT1	ENSG00000242094.1	3	0	0
antisense	AL513165.1	ENSG00000234160.1	3	0	0
TEC	AP001273.1	ENSG00000279696.1	3	0	0
protein_coding	MAPT	ENSG00000186868.15	3	0	0
processed_transcript	ADIRF-AS1	ENSG00000272734.1	3	0	0
protein_coding	IGSF10	ENSG00000152580.8	3	0	0
antisense	STX18-AS1	ENSG00000247708.7	3	0	0
processed_pseudogene	YBX1P2	ENSG00000231167.3	3	0	0
antisense	SLC25A21-	ENSG00000258708.1	3	0	0
misc_RNA	RF00019	ENSG00000200090.1	3	0	0
protein_coding	MATR3	ENSG00000015479.18	3	0	0
protein_coding	PNPLA7	ENSG00000130653.15	3	0	0
protein_coding	GGT1	ENSG00000100031.18	3	0	0
lincRNA	AC105277.1	ENSG00000232453.6	3	0	0
lincRNA	LINC01191	ENSG00000234199.2	3	0	0
protein_coding	SCN3A	ENSG00000153253.16	3	0	0
protein_coding	CADPS	ENSG00000163618.17	3	0	0
protein_coding	PPIL6	ENSG00000185250.15	3	0	0
protein_coding	LRGUK	ENSG00000155530.3	3	0	0
protein_coding	CHRDL1	ENSG00000101938.14	3	0	0
transcribed_unprocessed_pseudogene	GTF2IRD2P1	ENSG00000214544.7	3	0	0
unprocessed_pseudogene	DDX11L2	ENSG00000236397.3	3	0	0
lincRNA	AC021851.1	ENSG00000272800.1	3	0	0
TEC	AL133243.4	ENSG00000279544.1	3	0	0
processed_pseudogene	AC073072.2	ENSG00000234513.1	3	0	0

unprocessed_pseudogene	AL353807.3	ENSG00000246203.2	3	0	0
sense_intronic	AC131009.3	ENSG00000273568.1	3	0	0
sense_intronic	AC006538.1	ENSG00000261342.1	3	0	0
processed_transcript	AC004967.2	ENSG00000285725.1	3	0	0
lincRNA	AC015912.3	ENSG00000274213.1	3	0	0
protein_coding	ZNF726	ENSG00000213967.10	3	0	0
transcribed_unprocessed_pseudogene	AP000347.1	ENSG00000272578.5	3	0	0
protein_coding	LRP5L	ENSG00000100068.12	3	0	0
sense_overlapping	AC104083.1	ENSG00000260244.1	3	0	0
snRNA	RNU6-681P	ENSG00000200882.1	3	0	0
protein_coding	TMCO6	ENSG00000113119.12	3	0	0
protein_coding	OTX1	ENSG00000115507.9	3	0	0
protein_coding	UBE2V1	ENSG00000244687.11	3	0	0
transcribed_processed_pseudogene	AC097376.1	ENSG00000248863.2	3	0	0
processed_transcript	ITGA9-AS1	ENSG00000235257.8	3	0	0
protein_coding	KHDC1L	ENSG00000256980.4	3	0	0
antisense	AL391069.3	ENSG00000237976.1	3	0	0
antisense	AC008906.1	ENSG00000248734.2	3	0	0
protein_coding	PEX11G	ENSG00000104883.7	3	0	0
protein_coding	RAB11FIP4	ENSG00000131242.17	3	0	0
protein_coding	ACSS1	ENSG00000154930.14	3	0	0
protein_coding	MACROD2	ENSG00000172264.17	3	0	0
TEC	AC090181.3	ENSG00000278991.1	3	0	0
protein_coding	TMC4	ENSG00000167608.11	3	0	0
protein_coding	CYYR1	ENSG00000166265.11	3	0	0
protein_coding	ALDH1A1	ENSG00000165092.12	3	0	0
scaRNA	SCARNA13	ENSG00000252481.1	3	0	0
processed_transcript	SLC25A5-	ENSG00000224281.4	3	0	0
processed_pseudogene	AP002381.1	ENSG00000234751.1	3	0	0
protein_coding	HIST1H4I	ENSG00000276180.1	3	0	0
lincRNA	AP001462.1	ENSG00000269038.1	3	0	0
antisense	AL161729.4	ENSG00000271659.1	3	0	0
protein_coding	ZSCAN23	ENSG00000187987.9	3	0	0
protein_coding	BLNK	ENSG00000095585.16	3	0	0
lincRNA	BX649601.1	ENSG00000273473.1	3	0	0
antisense	AL592429.2	ENSG00000226571.2	3	0	0
processed_transcript	AC103760.1	ENSG00000254231.2	3	0	0
protein_coding	MAP4K1	ENSG00000104814.12	3	0	0
antisense	AC021016.3	ENSG00000273361.1	3	0	0
processed_pseudogene	AL353705.1	ENSG00000228781.1	3	0	0
protein_coding	NMNAT3	ENSG00000163864.16	3	0	0

antisense	AC106820.3	ENSG00000260095.1	3	0	0
lincRNA	LINC01358	ENSG00000237352.3	3	0	0
protein_coding	TMEM61	ENSG00000143001.4	3	0	0
processed_pseudogene	AC005005.2	ENSG00000235573.2	3	0	0
antisense	AC007570.1	ENSG00000255655.1	3	0	0
lincRNA	LINC00691	ENSG00000224074.3	3	0	0
lincRNA	AC007228.1	ENSG00000268568.1	3	0	0
protein_coding	CHL1	ENSG00000134121.9	3	0	0
protein_coding	TRAPPC5	ENSG00000181029.8	3	0	0
protein_coding	NTN5	ENSG00000142233.11	3	0	0
antisense	FAM3D-AS1	ENSG00000244383.2	3	0	0
antisense	AC097634.3	ENSG00000277855.1	3	0	0
antisense	AC024243.1	ENSG00000272969.1	3	0	0
antisense	AC068305.2	ENSG00000257443.1	3	0	0
antisense	TSC22D1-	ENSG00000278156.1	3	0	0
antisense	AC104938.1	ENSG00000260037.5	3	0	0
lincRNA	AL353708.1	ENSG00000260360.1	3	0	0
lincRNA	Z99289.3	ENSG00000271208.1	3	0	0
processed_pseudogene	AL389915.1	ENSG00000236005.1	3	0	0
processed_transcript	AC010247.1	ENSG00000254887.1	3	0	0
protein_coding	TNNI3K	ENSG00000116783.14	3	0	0
protein_coding	CPN2	ENSG00000178772.6	3	0	0
protein_coding	CC2D2B	ENSG00000188649.13	3	0	0
protein_coding	HPX	ENSG00000110169.10	3	0	0
protein_coding	KRTAP5-8	ENSG00000241233.3	3	0	0
protein_coding	PLA2G1B	ENSG00000170890.13	3	0	0
protein_coding	MC4R	ENSG00000166603.5	3	0	0
protein_coding	NUDT10	ENSG00000122824.10	3	0	0
protein_coding	BX276092.9	ENSG00000283599.2	3	0	0
sense_intronic	AL157762.1	ENSG00000277246.1	3	0	0
snRNA	RNU7-50P	ENSG00000238304.1	3	0	0
protein_coding	NR3C2	ENSG00000151623.14	3	0	0
antisense	RASSF1-AS1	ENSG00000281358.1	3	0	0
antisense	AL031775.2	ENSG00000272402.1	3	0	0
lincRNA	AC092919.1	ENSG00000240497.2	3	0	0
processed_pseudogene	AC009362.1	ENSG00000233287.1	3	0	0
protein_coding	FDX2	ENSG00000267673.6	3	0	0
transcribed_unprocessed_pseudogene	AL772337.3	ENSG00000234460.2	3	0	0
transcribed_unprocessed_pseudogene	CLCN3P1	ENSG00000232000.2	3	0	0
TEC	AC083843.3	ENSG00000279518.1	3	0	0
protein_coding	SLC4A3	ENSG00000114923.16	4	0	0

protein_coding	HMCN1	ENSG00000143341.11	4	0	0
protein_coding	KIAA1024	ENSG00000169330.8	4	0	0
protein_coding	MCTP1	ENSG00000175471.19	4	0	0
protein_coding	ITPKA	ENSG00000137825.10	4	0	0
processed_pseudogene	HMGB1P49	ENSG00000230519.2	4	0	0
protein_coding	YY2	ENSG00000230797.2	4	0	0
protein_coding	LRWD1	ENSG00000161036.12	4	0	0
protein_coding	MTG1	ENSG00000148824.18	4	0	0
protein_coding	ZBTB42	ENSG00000179627.9	4	0	0
protein_coding	LFNG	ENSG00000106003.12	4	0	0
lincRNA	AC009041.2	ENSG00000260807.6	4	0	0
protein_coding	FAM228A	ENSG00000186453.12	4	0	0
protein_coding	FAM107A	ENSG00000168309.17	4	0	0
antisense	ERICH6-AS1	ENSG00000240137.5	4	0	0
lincRNA	AC008736.1	ENSG00000267475.1	4	0	0
sense_intronic	AC011379.1	ENSG00000250069.1	4	0	0
protein_coding	NBPF14	ENSG00000270629.5	4	0	0
antisense	JMJD1C-AS1	ENSG00000272767.1	4	0	0
protein_coding	GSG1	ENSG00000111305.18	4	0	0
processed_pseudogene	PFN1P1	ENSG00000233328.3	4	0	0
protein_coding	GAL	ENSG00000069482.6	4	0	0
lincRNA	ERICD	ENSG00000280303.2	4	0	0
TEC	AC009303.4	ENSG00000279227.1	4	0	0
antisense	TMEM92-	ENSG00000251179.1	4	0	0
antisense	AD000671.3	ENSG00000267439.1	4	0	0
protein_coding	ADRA1D	ENSG00000171873.7	4	0	0
transcribed_unprocessed_pseudogene	FAM86EP	ENSG00000251669.5	4	0	0
protein_coding	C5	ENSG00000106804.7	4	0	0
protein_coding	CENPM	ENSG00000100162.14	4	0	0
protein_coding	DET1	ENSG00000140543.14	4	0	0
lincRNA	LINC00342	ENSG00000232931.5	4	0	0
TEC	AC145098.2	ENSG00000279821.1	4	0	0
lincRNA	AC125494.2	ENSG00000269892.1	4	0	0
TEC	AC022417.1	ENSG00000279923.1	4	0	0
protein_coding	ZSWIM5	ENSG00000162415.6	4	0	0
processed_pseudogene	IRS3P	ENSG00000184414.2	4	0	0
lincRNA	AC009093.1	ENSG00000259807.1	4	0	0
protein_coding	GAB3	ENSG00000160219.11	4	0	0
processed_transcript	LINC01121	ENSG00000205054.7	4	0	0
protein_coding	FER1L6	ENSG00000214814.7	4	0	0
protein_coding	OLFML1	ENSG00000183801.7	4	0	0
processed_pseudogene	PPIAP22	ENSG00000198618.5	4	0	0

protein_coding	HLA-DMA	ENSG00000204257.14	4	0	0
protein_coding	LZTS1	ENSG00000061337.15	4	0	0
transcribed_processed_pseudogene	RPSAP52	ENSG00000241749.4	4	0	0
TEC	AC016542.2	ENSG00000279502.1	4	0	0
lincRNA	AL359715.1	ENSG00000233967.6	4	0	0
antisense	ENO1-AS1	ENSG00000230679.1	4	0	0
antisense	AL138789.1	ENSG00000233589.1	4	0	0
processed_transcript	ATP1A1-AS1	ENSG00000203865.9	4	0	0
protein_coding	TMEM92	ENSG00000167105.7	4	0	0
protein_coding	GCSH	ENSG00000140905.10	4	0	0
sense_intronic	AC118344.1	ENSG00000205041.1	4	0	0
protein_coding	IL21R	ENSG00000103522.15	4	0	0
protein_coding	SLC44A5	ENSG00000137968.16	4	0	0
protein_coding	KCNAB1	ENSG00000169282.17	4	0	0
unprocessed_pseudogene	AL592293.1	ENSG00000215237.6	4	0	0
antisense	BHLHE40-	ENSG00000235831.6	4	0	0
lincRNA	AC068282.1	ENSG00000236682.1	4	0	0
protein_coding	FAM19A2	ENSG00000198673.10	4	0	0
lincRNA	AL589826.2	ENSG00000283010.1	4	0	0
antisense	AC010894.3	ENSG00000236449.1	4	0	0
antisense	AP001453.1	ENSG00000256116.1	4	0	0
lincRNA	AC026355.1	ENSG00000228561.2	4	0	0
lincRNA	AC087672.2	ENSG00000254288.1	4	0	0
lincRNA	Z69706.1	ENSG00000268836.1	4	0	0
lincRNA	HAR1B	ENSG00000231133.6	4	0	0
processed_pseudogene	AP000942.1	ENSG00000243777.1	4	0	0
protein_coding	RNF175	ENSG00000145428.14	4	0	0
protein_coding	FOXS1	ENSG00000179772.7	4	0	0
protein_coding	ZNF334	ENSG00000198185.11	4	0	0
processed_pseudogene	RPL29P19	ENSG00000224594.2	4	0	0
processed_pseudogene	AC079203.1	ENSG00000243094.1	4	0	0
lincRNA	AC108488.1	ENSG00000242282.6	4	0	0
protein_coding	PLEKHG3	ENSG00000126822.16	5	0	0
protein_coding	CPAMD8	ENSG00000160111.12	5	0	0
protein_coding	SMPD2	ENSG00000135587.8	5	0	0
protein_coding	DZANK1	ENSG00000089091.16	5	0	0
protein_coding	DNM3	ENSG00000197959.13	5	0	0
transcribed_unprocessed_pseudogene	ZNF767P	ENSG00000133624.13	5	0	0
TEC	AC010435.1	ENSG00000279557.1	5	0	0
protein_coding	OPLAH	ENSG00000178814.16	5	0	0
protein_coding	MT1M	ENSG00000205364.3	5	0	0

protein_coding	DHODH	ENSG00000102967.11	5	0	0
lincRNA	AP000766.1	ENSG00000261098.1	5	0	0
protein_coding	FBXO41	ENSG00000163013.11	5	0	0
protein_coding	ABCC2	ENSG00000023839.11	5	0	0
transcribed_unprocessed_pseudogene	LSP1P4	ENSG00000143429.10	5	0	0
antisense	AL731569.1	ENSG00000227896.2	5	0	0
antisense	AC109322.1	ENSG00000255224.1	5	0	0
antisense	ITFG2-AS1	ENSG00000258325.2	5	0	0
protein_coding	ADH1C	ENSG00000248144.5	5	0	0
protein_coding	ANKRD1	ENSG00000148677.6	5	0	0
lincRNA	MYOSLID	ENSG00000229647.1	5	0	0
protein_coding	ALDH3A1	ENSG00000108602.17	5	0	0
antisense	AL158151.1	ENSG00000204055.4	5	0	0
protein_coding	IRF6	ENSG00000117595.11	5	0	0
lincRNA	LINC00346	ENSG00000255874.2	5	0	0
protein_coding	SNCG	ENSG00000173267.13	5	0	0
processed_transcript	LINC00173	ENSG00000196668.3	5	0	0
lincRNA	AC136443.3	ENSG00000257264.5	5	0	0
lincRNA	AC121247.1	ENSG00000225399.4	5	0	0
protein_coding	SUCNR1	ENSG00000198829.6	5	0	0
protein_coding	OGN	ENSG00000106809.10	5	0	0
protein_coding	NR0B1	ENSG00000169297.7	5	0	0
processed_transcript	AC092902.2	ENSG00000241288.7	6	0	0
protein_coding	SLC25A19	ENSG00000125454.11	6	0	0
protein_coding	FDXACB1	ENSG00000255561.6	6	0	0
antisense	AL161772.1	ENSG00000278291.1	6	0	0
lincRNA	AC016957.2	ENSG00000276136.1	6	0	0
protein_coding	IQGAP3	ENSG00000183856.10	6	0	0
transcribed_unprocessed_pseudogene	SMG1P7	ENSG00000261556.9	6	0	0
protein_coding	PLEKHA6	ENSG00000143850.14	6	0	0
processed_pseudogene	RPL23AP97	ENSG00000260615.1	6	0	0
protein_coding	PTPN20	ENSG00000204179.10	6	0	0
protein_coding	ZNF586	ENSG00000083828.15	6	0	0
antisense	AL354920.1	ENSG00000254473.1	6	0	0
protein_coding	MYO3B	ENSG00000071909.18	6	0	0
sense_intronic	AC133552.5	ENSG00000275494.1	6	0	0
protein_coding	DPY19L2	ENSG00000177990.11	7	0	0
protein_coding	SYDE2	ENSG00000097096.8	7	0	0
protein_coding	PCSK4	ENSG00000115257.15	7	0	0
protein_coding	CLN6	ENSG00000128973.12	7	0	0
protein_coding	PPM1J	ENSG00000155367.15	7	0	0

lincRNA	AC008703.1	ENSG00000283413.1	7	0	0
sense_intronic	AC009268.2	ENSG00000278472.1	7	0	0
protein_coding	ACE	ENSG00000159640.15	8	0	0
protein_coding	SLC17A7	ENSG00000104888.9	8	0	0
protein_coding	HEY1	ENSG00000164683.16	8	0	0
antisense	AL034346.1	ENSG00000261730.1	8	0	0
antisense	AL355073.2	ENSG00000275569.1	8	0	0
protein_coding	RAB33A	ENSG00000134594.4	8	0	0
protein_coding	NUAK2	ENSG00000163545.8	8	0	0
protein_coding	ADRA2A	ENSG00000150594.6	8	0	0
lincRNA	LINC01204	ENSG00000229563.6	8	0	0
antisense	ST7-AS1	ENSG00000227199.1	9	0	0
lincRNA	AL138995.1	ENSG00000277763.1	9	0	0
protein_coding	HOGA1	ENSG00000241935.8	9	0	0
protein_coding	GDPD3	ENSG00000102886.14	9	0	0
protein_coding	SLC7A14	ENSG00000013293.5	14	0	0
protein_coding	IL24	ENSG00000162892.15	0	224	
protein_coding	MTRNR2L10	ENSG00000256045.2	0	184	
protein_coding	PRND	ENSG00000171864.4	0	30	
protein_coding	CMYA5	ENSG00000164309.14	0	27	
protein_coding	CREB5	ENSG00000146592.16	0	24	
protein_coding	EREG	ENSG00000124882.3	0	21	
protein_coding	C2CD4A	ENSG00000198535.5	0	21	
protein_coding	RIPOR2	ENSG00000111913.18	0	21	
protein_coding	PDE10A	ENSG00000112541.14	0	19	
protein_coding	NTN1	ENSG00000065320.8	0	16	
protein_coding	CCR7	ENSG00000126353.3	0	16	
protein_coding	IL1RL2	ENSG00000115598.9	0	13	
protein_coding	LRRC1	ENSG00000137269.14	0	11	
protein_coding	MTUS2	ENSG00000132938.19	0	11	
protein_coding	BEST1	ENSG00000167995.15	0	11	
protein_coding	CKAP2L	ENSG00000169607.12	0	10	
protein_coding	EEF2KMT	ENSG00000118894.14	0	10	
protein_coding	GBP5	ENSG00000154451.14	0	10	
protein_coding	PHACTR3	ENSG00000087495.16	0	10	
protein_coding	ADAM28	ENSG00000042980.12	0	9	
protein_coding	GNGT1	ENSG00000127928.12	0	9	
protein_coding	FAM124A	ENSG00000150510.16	0	9	
protein_coding	YPEL1	ENSG00000100027.14	0	8	
protein_coding	ZNF69	ENSG00000198429.9	0	8	
protein_coding	TMEM71	ENSG00000165071.14	0	8	
protein_coding	REP15	ENSG00000174236.3	0	8	
protein_coding	BCAS1	ENSG00000064787.13	0	8	
protein_coding	MTRNR2L6	ENSG00000270672.1	0	8	

protein_coding	RHBDL2	ENSG00000158315.10	0	7
protein_coding	GAS2	ENSG00000148935.11	0	7
protein_coding	ENPEP	ENSG00000138792.9	0	7
protein_coding	ZNF345	ENSG00000251247.10	0	7
protein_coding	PALMD	ENSG00000099260.10	0	7
protein_coding	MUC13	ENSG00000173702.7	0	7
protein_coding	ACKR4	ENSG00000129048.6	0	7
protein_coding	AC011043.1	ENSG00000276256.1	0	6
protein_coding	ZFP69	ENSG00000187815.9	0	6
protein_coding	BTBD11	ENSG00000151136.14	0	6
protein_coding	ZDHHC23	ENSG00000184307.14	0	6
protein_coding	BX255925.3	ENSG00000284976.1	0	6
protein_coding	C7orf31	ENSG00000153790.11	0	6
protein_coding	CCT6B	ENSG00000132141.13	0	6
protein_coding	ZNF396	ENSG00000186496.11	0	6
protein_coding	C10orf90	ENSG00000154493.18	0	6
protein_coding	ADAM32	ENSG00000197140.14	0	6
protein_coding	PCDHA10	ENSG00000250120.6	0	6
protein_coding	MMP8	ENSG00000118113.11	0	6
protein_coding	CCDC87	ENSG00000182791.4	0	6
protein_coding	MTRNR2L7	ENSG00000256892.2	0	6
protein_coding	MYEOV	ENSG00000172927.7	0	6
protein_coding	SLC25A15	ENSG00000102743.14	0	6
protein_coding	HAS3	ENSG00000103044.10	0	5
protein_coding	SLC39A8	ENSG00000138821.12	0	5
protein_coding	NEMP2	ENSG00000189362.11	0	5
protein_coding	HMMR	ENSG00000072571.19	0	5
protein_coding	GALNT3	ENSG00000115339.13	0	5
protein_coding	IQANK1	ENSG00000203499.11	0	5
protein_coding	LLGL2	ENSG00000073350.13	0	5
protein_coding	PCDHGB6	ENSG00000253305.2	0	5
protein_coding	ASAH2B	ENSG00000204147.10	0	5
protein_coding	LTB4R	ENSG00000213903.8	0	5
protein_coding	SDK1	ENSG00000146555.18	0	5
protein_coding	ANKRD30B	ENSG00000180777.13	0	5
protein_coding	FNBP1L	ENSG00000137942.16	0	5
protein_coding	NOX4	ENSG00000086991.12	0	5
protein_coding	DNAJC12	ENSG00000108176.14	0	5
protein_coding	CCRL2	ENSG00000121797.9	0	5
protein_coding	CFAP46	ENSG00000171811.13	0	5
protein_coding	IL18R1	ENSG00000115604.10	0	5
protein_coding	PALM2	ENSG00000243444.7	0	5
protein_coding	CDCA7	ENSG00000144354.13	0	4
protein_coding	IL1B	ENSG00000125538.11	0	4

protein_coding	BDH1	ENSG00000161267.11	0	4
protein_coding	SPC24	ENSG00000161888.11	0	4
protein_coding	LRRC34	ENSG00000171757.15	0	4
protein_coding	ZNF81	ENSG00000197779.13	0	4
protein_coding	TAS2R14	ENSG00000212127.5	0	4
protein_coding	TAF6L	ENSG00000162227.7	0	4
protein_coding	ADAP1	ENSG00000105963.14	0	4
protein_coding	KRT8	ENSG00000170421.12	0	4
protein_coding	AL031281.2	ENSG00000285721.1	0	4
protein_coding	CCND2	ENSG00000118971.7	0	4
protein_coding	CYP2R1	ENSG00000186104.10	0	4
protein_coding	CCDC157	ENSG00000187860.10	0	4
protein_coding	HAUS5	ENSG00000249115.8	0	4
protein_coding	PCDHGA5	ENSG00000253485.2	0	4
protein_coding	TVP23C	ENSG00000175106.16	0	4
protein_coding	ZNF543	ENSG00000178229.7	0	4
protein_coding	SKIDA1	ENSG00000180592.16	0	4
protein_coding	RIMS2	ENSG00000176406.22	0	4
protein_coding	ARMC7	ENSG00000125449.6	0	4
protein_coding	FIBCD1	ENSG00000130720.12	0	4
protein_coding	OSBP2	ENSG00000184792.15	0	4
protein_coding	C1orf115	ENSG00000162817.6	0	4
protein_coding	NR5A2	ENSG00000116833.13	0	4
protein_coding	CCDC3	ENSG00000151468.10	0	4
protein_coding	ADAMTS15	ENSG00000166106.3	0	4
protein_coding	WNT3	ENSG00000108379.9	0	4
protein_coding	USF1	ENSG00000158773.14	0	4
protein_coding	PPARGC1A	ENSG00000109819.8	0	4
protein_coding	OXCT2	ENSG00000198754.5	0	4
protein_coding	HTR2B	ENSG00000135914.5	0	4
protein_coding	RSPO3	ENSG00000146374.13	0	4
protein_coding	ANGPTL5	ENSG00000187151.7	0	4
protein_coding	CCDC13	ENSG00000244607.6	0	4
protein_coding	FREM3	ENSG00000183090.5	0	4
protein_coding	PRL	ENSG00000172179.11	0	4
protein_coding	ZC3H12D	ENSG00000178199.13	0	4
protein_coding	RIPK3	ENSG00000129465.15	0	4
protein_coding	PCSK2	ENSG00000125851.9	0	4
protein_coding	GRIP2	ENSG00000144596.12	0	4
protein_coding	DUSP27	ENSG00000198842.9	0	4
protein_coding	PALM	ENSG00000099864.17	0	4
protein_coding	COL23A1	ENSG00000050767.16	0	4
protein_coding	SFRP2	ENSG00000145423.4	0	4
protein_coding	EFCAB1	ENSG00000034239.10	0	4

protein_coding	TICRR	ENSG00000140534.13	0	3
protein_coding	HIVEP3	ENSG00000127124.15	0	3
protein_coding	RPA4	ENSG00000204086.4	0	3
protein_coding	CCDC15	ENSG00000149548.14	0	3
protein_coding	HIST1H1D	ENSG00000124575.6	0	3
protein_coding	ARHGEF39	ENSG00000137135.17	0	3
protein_coding	CDCA3	ENSG00000111665.11	0	3
protein_coding	KIF15	ENSG00000163808.16	0	3
protein_coding	KIFC1	ENSG00000237649.7	0	3
protein_coding	DENND2D	ENSG00000162777.16	0	3
protein_coding	PSD4	ENSG00000125637.15	0	3
protein_coding	ANXA10	ENSG00000109511.11	0	3
protein_coding	AURKC	ENSG00000105146.12	0	3
protein_coding	HNRNPA1L2	ENSG00000139675.12	0	3
protein_coding	ATP8A2	ENSG00000132932.16	0	3
protein_coding	BMP6	ENSG00000153162.8	0	3
protein_coding	H3F3A	ENSG00000163041.9	0	3
protein_coding	AP002495.1	ENSG00000254469.7	0	3
protein_coding	DEPDC4	ENSG00000166153.16	0	3
protein_coding	ZBED8	ENSG00000221886.3	0	3
protein_coding	AL136454.1	ENSG00000231767.4	0	3
protein_coding	PPIP5K1	ENSG00000168781.22	0	3
protein_coding	CCDC189	ENSG00000196118.11	0	3
protein_coding	TMEM30B	ENSG00000182107.6	0	3
protein_coding	LBX2	ENSG00000179528.15	0	3
protein_coding	CCDC24	ENSG00000159214.12	0	3
protein_coding	ZNF850	ENSG00000267041.5	0	3
protein_coding	ATF6B	ENSG00000213676.12	0	3
protein_coding	SLC10A5	ENSG00000253598.1	0	3
protein_coding	TAF1A	ENSG00000143498.17	0	3
protein_coding	CERS4	ENSG00000090661.11	0	3
protein_coding	EPCAM	ENSG00000119888.10	0	3
protein_coding	NINJ2	ENSG00000171840.11	0	3
protein_coding	KCNRG	ENSG00000198553.8	0	3
protein_coding	ARMC2	ENSG00000118690.12	0	3
protein_coding	SYBU	ENSG00000147642.16	0	3
protein_coding	PPP1R1C	ENSG00000150722.10	0	3
protein_coding	OASL	ENSG00000135114.12	0	3
protein_coding	HELB	ENSG00000127311.9	0	3
protein_coding	LRRIQ3	ENSG00000162620.15	0	3
protein_coding	ACPP	ENSG00000014257.15	0	3
protein_coding	TBCE	ENSG00000284770.1	0	3
protein_coding	IQCA1	ENSG00000132321.16	0	3
protein_coding	PROX1	ENSG00000117707.15	0	3

protein_coding	SALL4	ENSG00000101115.12	0	3
protein_coding	COL4A4	ENSG00000081052.12	0	3
protein_coding	FAM174B	ENSG00000185442.12	0	3
protein_coding	C19orf73	ENSG00000221916.3	0	3
protein_coding	KCNH3	ENSG00000135519.7	0	3
protein_coding	RNF25	ENSG00000163481.7	0	3
protein_coding	AC099489.1	ENSG00000188897.9	0	3
protein_coding	MTUS1	ENSG00000129422.14	0	3
protein_coding	BEST4	ENSG00000142959.4	0	3
protein_coding	BCO2	ENSG00000197580.11	0	3
protein_coding	CHADL	ENSG00000100399.15	0	3
protein_coding	IFI27	ENSG00000165949.12	0	3
protein_coding	SAMD11	ENSG00000187634.11	0	3
protein_coding	BEGAIN	ENSG00000183092.16	0	3
protein_coding	PRSS27	ENSG00000172382.9	0	3
protein_coding	CYTIP	ENSG00000115165.9	0	3
protein_coding	MSMB	ENSG00000263639.5	0	3
protein_coding	ACTC1	ENSG00000159251.7	0	3
protein_coding	KRBA2	ENSG00000184619.5	0	3
protein_coding	RARRES1	ENSG00000118849.9	0	3
protein_coding	CCDC8	ENSG00000169515.6	0	3
protein_coding	PTPRN	ENSG00000054356.13	0	3
protein_coding	CHRD	ENSG00000090539.15	0	3
protein_coding	PCDHB6	ENSG00000113211.5	0	3
protein_coding	ITK	ENSG00000113263.12	0	3
protein_coding	GRIK4	ENSG00000149403.12	0	3
protein_coding	CACNA2D4	ENSG00000151062.14	0	3
protein_coding	USP44	ENSG00000136014.11	0	3
protein_coding	RHCG	ENSG00000140519.13	0	3
protein_coding	LBP	ENSG00000129988.5	0	3
protein_coding	CLIC6	ENSG00000159212.12	0	3
protein_coding	SEC14L6	ENSG00000214491.8	0	3
protein_coding	ATP2B3	ENSG00000067842.17	0	3
protein_coding	KLRD1	ENSG00000134539.16	0	3
protein_coding	DNAH12	ENSG00000174844.14	0	3
protein_coding	ADAM11	ENSG00000073670.13	0	3
protein_coding	TXNDC2	ENSG00000168454.11	0	3
protein_coding	ZNF737	ENSG00000237440.8	0	3
protein_coding	ZNF418	ENSG00000196724.12	0	3
protein_coding	TM4SF20	ENSG00000168955.3	0	3
protein_coding	NRROS	ENSG00000174004.5	0	3
protein_coding	TRPC3	ENSG00000138741.10	0	3
protein_coding	PDE3B	ENSG00000152270.8	0	3
protein_coding	BLID	ENSG00000259571.1	0	3

protein_coding	CDO1	ENSG00000129596.4	0	3
protein_coding	VCAM1	ENSG00000162692.11	0	3
protein_coding	GLYATL1	ENSG00000166840.13	0	3
protein_coding	SALL1	ENSG00000103449.11	0	3
protein_coding	IL34	ENSG00000157368.10	0	3
protein_coding	CHGB	ENSG00000089199.9	0	3
protein_coding	CACNB2	ENSG00000165995.19	0	3
protein_coding	TRABD2B	ENSG00000269113.3	0	3
protein_coding	C2CD4B	ENSG00000205502.3	0	3
protein_coding	RNF180	ENSG00000164197.11	0	3
protein_coding	NAPSA	ENSG00000131400.7	0	3
protein_coding	TRPM3	ENSG00000083067.22	0	3
protein_coding	MAPK8IP2	ENSG00000008735.13	0	3
protein_coding	C17orf53	ENSG00000125319.14	0	3
protein_coding	CYP27B1	ENSG00000111012.9	0	3
protein_coding	CXCL3	ENSG00000163734.4	0	3
protein_coding	ZNF547	ENSG00000152433.14	0	3
protein_coding	SCLY	ENSG00000132330.16	0	3
protein_coding	E2F1	ENSG00000101412.12	0	2
protein_coding	MND1	ENSG00000121211.7	0	2
protein_coding	CCNE2	ENSG00000175305.17	0	2
protein_coding	KIAA0040	ENSG00000235750.9	0	2
protein_coding	CXCL8	ENSG00000169429.10	0	2
protein_coding	CHAF1B	ENSG00000159259.7	0	2
protein_coding	IL33	ENSG00000137033.11	0	2
protein_coding	SHISA2	ENSG00000180730.4	0	2
protein_coding	PTK6	ENSG00000101213.6	0	2
protein_coding	TRIP13	ENSG00000071539.13	0	2
protein_coding	MTFR2	ENSG00000146410.11	0	2
protein_coding	RUNDC3B	ENSG00000105784.15	0	2
protein_coding	PRIM1	ENSG00000198056.13	0	2
protein_coding	SYT16	ENSG00000139973.16	0	2
protein_coding	PDSS1	ENSG00000148459.15	0	2
protein_coding	GPR137C	ENSG00000180998.11	0	2
protein_coding	ADTRP	ENSG00000111863.12	0	2
protein_coding	PLEKHH2	ENSG00000152527.13	0	2
protein_coding	P2RX5	ENSG00000083454.21	0	2
protein_coding	IL1R2	ENSG00000115590.13	0	2
protein_coding	TMEM154	ENSG00000170006.11	0	2
protein_coding	TCTE3	ENSG00000184786.5	0	2
protein_coding	MTCP1	ENSG00000214827.9	0	2
protein_coding	PLEKHG1	ENSG00000120278.16	0	2
protein_coding	KRT32	ENSG00000108759.3	0	2
protein_coding	ZNF681	ENSG00000196172.9	0	2

protein_coding	FAAH	ENSG00000117480.15	0	2
protein_coding	DOCK2	ENSG00000134516.16	0	2
protein_coding	PCDHGB4	ENSG00000253953.2	0	2
protein_coding	LRRC24	ENSG00000254402.6	0	2
protein_coding	IL1RN	ENSG00000136689.18	0	2
protein_coding	FAM83A	ENSG00000147689.16	0	2
protein_coding	ZNF165	ENSG00000197279.3	0	2
protein_coding	CENPA	ENSG00000115163.14	0	2
protein_coding	PRSS16	ENSG00000112812.15	0	2
protein_coding	SLC37A2	ENSG00000134955.11	0	2
protein_coding	TJP2	ENSG00000119139.19	0	2
protein_coding	HYKK	ENSG00000188266.13	0	2
protein_coding	EPN3	ENSG00000049283.17	0	2
protein_coding	TMC7	ENSG00000170537.12	0	2
protein_coding	WRAP53	ENSG00000141499.16	0	2
protein_coding	GTF2H2C	ENSG00000183474.15	0	2
protein_coding	NSUN7	ENSG00000179299.16	0	2
protein_coding	GPX2	ENSG00000176153.11	0	2
protein_coding	TNS4	ENSG00000131746.12	0	2
protein_coding	IFNLR1	ENSG00000185436.11	0	2
protein_coding	GIMAP2	ENSG00000106560.10	0	2
protein_coding	TMTC2	ENSG00000179104.8	0	2
protein_coding	HIST1H2BE	ENSG00000274290.2	0	2
protein_coding	ANKRD23	ENSG00000163126.14	0	2
protein_coding	PLEKHB1	ENSG00000021300.13	0	2
protein_coding	DDN	ENSG00000181418.7	0	2
protein_coding	SRRM5	ENSG00000226763.4	0	2
protein_coding	RELN	ENSG00000189056.13	0	2
protein_coding	CLSTN3	ENSG00000139182.14	0	2
protein_coding	KCNMB3	ENSG00000171121.16	0	2
protein_coding	FAM149A	ENSG00000109794.13	0	2
protein_coding	EFCAB5	ENSG00000176927.15	0	2
protein_coding	ZNF841	ENSG00000197608.11	0	2
protein_coding	PPP6R1	ENSG00000105063.18	0	2
protein_coding	CORO2A	ENSG00000106789.12	0	2
protein_coding	PODXL	ENSG00000128567.16	0	2
protein_coding	CFAP410	ENSG00000160226.15	0	2
protein_coding	FAM86C1	ENSG00000158483.15	0	2
protein_coding	STIMATE	ENSG00000213533.11	0	2
protein_coding	CCL28	ENSG00000151882.11	0	2
protein_coding	RAB17	ENSG00000124839.12	0	2
protein_coding	HES4	ENSG00000188290.10	0	2
protein_coding	TSNARE1	ENSG00000171045.14	0	2
protein_coding	FYB1	ENSG00000082074.16	0	2

protein_coding	CCDC110	ENSG00000168491.9	0	2
protein_coding	CCDC88B	ENSG00000168071.21	0	2
protein_coding	B3GNT4	ENSG00000176383.8	0	2
protein_coding	WNT10B	ENSG00000169884.13	0	2
protein_coding	KRTCAP2	ENSG00000163463.11	0	2
protein_coding	CCDC173	ENSG00000154479.12	0	2
protein_coding	LRRC39	ENSG00000122477.12	0	2
protein_coding	BCL11B	ENSG00000127152.17	0	2
protein_coding	ZBTB46	ENSG00000130584.10	0	2
protein_coding	ESR1	ENSG00000091831.23	0	2
protein_coding	TMEM79	ENSG00000163472.18	0	2
protein_coding	RNF32	ENSG00000105982.16	0	2
protein_coding	TMIGD1	ENSG00000182271.12	0	2
protein_coding	MGAT4C	ENSG00000182050.13	0	2
protein_coding	TBCE	ENSG00000285053.1	0	2
protein_coding	CPE	ENSG00000109472.13	0	2
protein_coding	RDH12	ENSG00000139988.9	0	2
protein_coding	TTC4	ENSG00000243725.6	0	2
protein_coding	SLC29A2	ENSG00000174669.11	0	2
protein_coding	STK19	ENSG00000204344.14	0	2
protein_coding	RNF207	ENSG00000158286.12	0	2
protein_coding	GATA2	ENSG00000179348.11	0	2
protein_coding	ARSI	ENSG00000183876.8	0	2
protein_coding	RCSD1	ENSG00000198771.10	0	2
protein_coding	GALNT13	ENSG00000144278.14	0	2
protein_coding	AOC2	ENSG00000131480.8	0	2
protein_coding	ADAM21	ENSG00000139985.6	0	2
protein_coding	DUSP8	ENSG00000184545.10	0	2
protein_coding	PAK5	ENSG00000101349.16	0	2
protein_coding	GSTM2	ENSG00000213366.12	0	2
protein_coding	SFTPB	ENSG00000168878.16	0	2
protein_coding	STAR	ENSG00000147465.11	0	2
protein_coding	NEK5	ENSG00000197168.12	0	2
protein_coding	PATL2	ENSG00000229474.6	0	2
protein_coding	ZMYND15	ENSG00000141497.13	0	2
protein_coding	AC011511.1	ENSG00000167807.15	0	2
protein_coding	C19orf38	ENSG00000214212.8	0	2
protein_coding	HIPK4	ENSG00000160396.8	0	2
protein_coding	MAN2B1	ENSG00000104774.12	0	2
protein_coding	UPK1A	ENSG00000105668.7	0	2
protein_coding	B3GNT10	ENSG00000214654.8	0	2
protein_coding	CATSPERD	ENSG00000174898.15	0	2
protein_coding	PIK3CG	ENSG00000105851.10	0	2
protein_coding	HLA-DOB	ENSG00000241106.7	0	2

protein_coding	TFAP2E	ENSG00000116819.7	0	2
protein_coding	BCAN	ENSG00000132692.18	0	2
protein_coding	CX3CR1	ENSG00000168329.13	0	2
protein_coding	LMOD3	ENSG00000163380.15	0	2
protein_coding	EXOC1L	ENSG00000250821.2	0	2
protein_coding	STBD1	ENSG00000118804.8	0	2
protein_coding	PCDHGA3	ENSG00000254245.2	0	2
protein_coding	HIST1H3B	ENSG00000274267.1	0	2
protein_coding	PRPH2	ENSG00000112619.7	0	2
protein_coding	GPR22	ENSG00000172209.5	0	2
protein_coding	ODF1	ENSG00000155087.3	0	2
protein_coding	FAM69B	ENSG00000165716.10	0	2
protein_coding	PTPRO	ENSG00000151490.13	0	2
protein_coding	CCDC184	ENSG00000177875.4	0	2
protein_coding	METTL7B	ENSG00000170439.6	0	2
protein_coding	RUBCNL	ENSG00000102445.18	0	2
protein_coding	TEX22	ENSG00000226174.6	0	2
protein_coding	EXD1	ENSG00000178997.11	0	2
protein_coding	PLIN1	ENSG00000166819.11	0	2
protein_coding	ABCC6	ENSG00000091262.15	0	2
protein_coding	Sep-04	ENSG00000108387.14	0	2
protein_coding	RDH8	ENSG00000080511.3	0	2
protein_coding	TSPAN16	ENSG00000130167.13	0	2
protein_coding	SBSN	ENSG00000189001.10	0	2
protein_coding	GUCY2F	ENSG00000101890.4	0	2
protein_coding	PLXNB3	ENSG00000198753.11	0	2
protein_coding	SRPK3	ENSG00000184343.10	0	2
protein_coding	IL20	ENSG00000162891.10	0	2
protein_coding	CLEC3B	ENSG00000163815.5	0	2
protein_coding	SLC7A2	ENSG00000003989.17	0	2
protein_coding	DPYS	ENSG00000147647.12	0	2
protein_coding	AARD	ENSG00000205002.3	0	2
protein_coding	ANO5	ENSG00000171714.11	0	2
protein_coding	CES4A	ENSG00000172824.15	0	2
protein_coding	EDC4	ENSG00000038358.14	0	2
protein_coding	NECAB2	ENSG00000103154.9	0	2
protein_coding	VTN	ENSG00000109072.13	0	2
protein_coding	KRTAP2-3	ENSG00000212724.3	0	2
protein_coding	NANOS3	ENSG00000187556.7	0	2
protein_coding	ITPRIPL1	ENSG00000198885.9	0	2
protein_coding	CALB2	ENSG00000172137.18	0	2
protein_coding	GIPR	ENSG00000010310.8	0	2
protein_coding	CACNG7	ENSG00000105605.7	0	2
protein_coding	IGFBP1	ENSG00000146678.9	0	2

protein_coding	GBX2	ENSG00000168505.6	0	2
protein_coding	LEP	ENSG00000174697.4	0	2
protein_coding	CD7	ENSG00000173762.7	0	2
protein_coding	ZP1	ENSG00000149506.11	0	2
protein_coding	NDP	ENSG00000124479.9	0	2
protein_coding	AC090360.1	ENSG00000267127.7	0	2
protein_coding	GRIN2A	ENSG00000183454.16	0	2
protein_coding	SPNS1	ENSG00000169682.17	0	2
protein_coding	FBXW10	ENSG00000171931.12	0	2
protein_coding	TSACC	ENSG00000163467.11	0	2
protein_coding	ELMO3	ENSG00000102890.14	0	2
protein_coding	NDUFA7	ENSG00000267855.5	0	2
protein_coding	CDC42	ENSG00000070831.15	0	2
protein_coding	FP565260.1	ENSG00000275464.4	0	2
protein_coding	LAPTM5	ENSG00000162511.7	0	1
protein_coding	MYBL2	ENSG00000101057.15	0	1
protein_coding	CDCA5	ENSG00000146670.9	0	1
protein_coding	ASF1B	ENSG00000105011.8	0	1
protein_coding	CCDC89	ENSG00000179071.4	0	1
protein_coding	TDRKH	ENSG00000182134.15	0	1
protein_coding	GIMAP8	ENSG00000171115.3	0	1
protein_coding	WDR62	ENSG00000075702.17	0	1
protein_coding	TMEM145	ENSG00000167619.11	0	1
protein_coding	STIL	ENSG00000123473.15	0	1
protein_coding	ZNF699	ENSG00000196110.7	0	1
protein_coding	TONSL	ENSG00000160949.16	0	1
protein_coding	CA9	ENSG00000107159.12	0	1
protein_coding	TAF4B	ENSG00000141384.12	0	1
protein_coding	MAP3K21	ENSG00000143674.10	0	1
protein_coding	MYO5C	ENSG00000128833.12	0	1
protein_coding	CHRNA5	ENSG00000169684.13	0	1
protein_coding	THBS4	ENSG00000113296.14	0	1
protein_coding	PAPPA2	ENSG00000116183.10	0	1
protein_coding	C6orf163	ENSG00000203872.6	0	1
protein_coding	SMN2	ENSG00000205571.13	0	1
protein_coding	NEK2	ENSG00000117650.12	0	1
protein_coding	NIPAL1	ENSG00000163293.11	0	1
protein_coding	CEP128	ENSG00000100629.16	0	1
protein_coding	FAM169A	ENSG00000198780.11	0	1
protein_coding	MT1F	ENSG00000198417.6	0	1
protein_coding	C1orf226	ENSG00000239887.4	0	1
protein_coding	SGK494	ENSG00000167524.14	0	1
protein_coding	RHEBL1	ENSG00000167550.10	0	1
protein_coding	PMEL	ENSG00000185664.14	0	1

protein_coding	WDR83	ENSG00000123154.11	0	1
protein_coding	TAS2R30	ENSG00000256188.3	0	1
protein_coding	NOS1AP	ENSG00000198929.12	0	1
protein_coding	PTPRB	ENSG00000127329.15	0	1
protein_coding	CHD7	ENSG00000171316.11	0	1
protein_coding	PITPNM3	ENSG00000091622.15	0	1
protein_coding	SPTBN2	ENSG00000173898.12	0	1
protein_coding	S100P	ENSG00000163993.6	0	1
protein_coding	EMB	ENSG00000170571.11	0	1
protein_coding	ALPK3	ENSG00000136383.6	0	1
protein_coding	NPIPB15	ENSG00000196436.8	0	1
protein_coding	SSUH2	ENSG00000125046.14	0	1
protein_coding	ZNF497	ENSG00000174586.10	0	1
protein_coding	MED12L	ENSG00000144893.12	0	1
protein_coding	SAMD15	ENSG00000100583.4	0	1
protein_coding	CRLF1	ENSG00000006016.10	0	1
protein_coding	GCNT2	ENSG00000111846.17	0	1
protein_coding	SDR42E1	ENSG00000184860.9	0	1
protein_coding	NCAM2	ENSG00000154654.14	0	1
protein_coding	TMEM238	ENSG00000233493.3	0	1
protein_coding	AFAP1L2	ENSG00000169129.14	0	1
protein_coding	POLR2J3	ENSG00000285437.1	0	1
protein_coding	ZFP69B	ENSG00000187801.14	0	1
protein_coding	NSG1	ENSG00000168824.14	0	1
protein_coding	GNA15	ENSG00000060558.3	0	1
protein_coding	SLITRK6	ENSG00000184564.9	0	1
protein_coding	ABCA13	ENSG00000179869.14	0	1
protein_coding	TSPAN15	ENSG00000099282.9	0	1
protein_coding	PADI1	ENSG00000142623.10	0	1
protein_coding	TC2N	ENSG00000165929.12	0	1
protein_coding	TMEM184A	ENSG00000164855.15	0	1
protein_coding	RASSF9	ENSG00000198774.4	0	1
protein_coding	SEMA3E	ENSG00000170381.13	0	1
protein_coding	SUSD4	ENSG00000143502.14	0	1
protein_coding	DLK2	ENSG00000171462.14	0	1
protein_coding	MPZL3	ENSG00000160588.9	0	1
protein_coding	NPM2	ENSG00000158806.13	0	1
protein_coding	KMO	ENSG00000117009.11	0	1
protein_coding	IRGM	ENSG00000237693.4	0	1
protein_coding	WISP3	ENSG00000112761.20	0	1
protein_coding	IL7	ENSG00000104432.13	0	1
protein_coding	SCARF1	ENSG00000074660.15	0	1
protein_coding	JPH2	ENSG00000149596.6	0	1
protein_coding	ELMO1	ENSG00000155849.15	0	1

protein_coding	DNAAF3	ENSG00000167646.13	0	1
protein_coding	RASGRP3	ENSG00000152689.17	0	1
protein_coding	LIPT2	ENSG00000175536.6	0	1
protein_coding	FAM222A	ENSG00000139438.5	0	1
protein_coding	NAP1L2	ENSG00000186462.8	0	1
protein_coding	FAM177B	ENSG00000197520.10	0	1
protein_coding	RRAGD	ENSG00000025039.14	0	1
protein_coding	CCDC9	ENSG00000105321.13	0	1
protein_coding	ZNF517	ENSG00000197363.9	0	1
protein_coding	HOXC8	ENSG00000037965.5	0	1
protein_coding	ADCY1	ENSG00000164742.15	0	1
protein_coding	C3orf52	ENSG00000114529.12	0	1
protein_coding	DHX34	ENSG00000134815.18	0	1
protein_coding	SLC1A3	ENSG00000079215.13	0	1
protein_coding	LPAR3	ENSG00000171517.5	0	1
protein_coding	EML6	ENSG00000214595.11	0	1
protein_coding	TNFSF9	ENSG00000125657.4	0	1
protein_coding	TLR5	ENSG00000187554.12	0	1
protein_coding	TP63	ENSG00000073282.12	0	1
protein_coding	TENM4	ENSG00000149256.15	0	1
protein_coding	CLEC18B	ENSG00000140839.11	0	1
protein_coding	C11orf80	ENSG00000173715.16	0	1
protein_coding	TBX1	ENSG00000184058.14	0	1
protein_coding	RTN1	ENSG00000139970.16	0	1
protein_coding	ATAD3C	ENSG00000215915.9	0	1
protein_coding	ATF7IP2	ENSG00000166669.13	0	1
protein_coding	HERC6	ENSG00000138642.14	0	1
protein_coding	AC068946.1	ENSG00000280537.2	0	1
protein_coding	TAF10	ENSG00000166337.9	0	1
protein_coding	USP2	ENSG00000036672.15	0	1
protein_coding	INAVA	ENSG00000163362.10	0	1
protein_coding	RIPK4	ENSG00000183421.11	0	1
protein_coding	MAB21L3	ENSG00000173212.4	0	1
protein_coding	FAM78A	ENSG00000126882.12	0	1
protein_coding	AP001267.5	ENSG00000285827.1	0	1
protein_coding	RAPGEF4	ENSG00000091428.17	0	1
protein_coding	KCNMB4	ENSG00000135643.4	0	1
protein_coding	CAPN12	ENSG00000182472.8	0	1
protein_coding	CYSLTR1	ENSG00000173198.5	0	1
protein_coding	FGFBP3	ENSG00000174721.9	0	1
protein_coding	POU3F1	ENSG00000185668.7	0	1
protein_coding	SMIM10L2A	ENSG00000178947.8	0	1
protein_coding	IL17RE	ENSG00000163701.18	0	1
protein_coding	MGAT4A	ENSG00000071073.12	0	1

protein_coding	APC2	ENSG00000115266.11	0	1
protein_coding	RIBC1	ENSG00000158423.16	0	1
protein_coding	HES2	ENSG00000069812.11	0	1
protein_coding	FABP5	ENSG00000164687.10	0	1
protein_coding	GPRC5C	ENSG00000170412.16	0	1
protein_coding	EML5	ENSG00000165521.15	0	1
protein_coding	ABCB1	ENSG00000085563.14	0	1
protein_coding	RBM11	ENSG00000185272.13	0	1
protein_coding	EFNA1	ENSG00000169242.11	0	1
protein_coding	SPATA17	ENSG00000162814.10	0	1
protein_coding	BEX2	ENSG00000133134.11	0	1
protein_coding	NRG4	ENSG00000169752.16	0	1
protein_coding	PKP1	ENSG00000081277.12	0	1
protein_coding	ADAP2	ENSG00000184060.10	0	1
protein_coding	FRRS1	ENSG00000156869.13	0	1
protein_coding	WDR66	ENSG00000158023.9	0	1
protein_coding	EFHC2	ENSG00000183690.12	0	1
protein_coding	DLGAP1	ENSG00000170579.16	0	1
protein_coding	OLFM4	ENSG00000102837.6	0	1
protein_coding	ANXA2R	ENSG00000177721.4	0	1
protein_coding	RASD2	ENSG00000100302.6	0	1
protein_coding	STK32A	ENSG00000169302.15	0	1
protein_coding	SLC6A20	ENSG00000163817.15	0	1
protein_coding	KCNT2	ENSG00000162687.17	0	1
protein_coding	CCDC30	ENSG00000186409.15	0	1
protein_coding	MYO15A	ENSG00000091536.17	0	1
protein_coding	ZNF653	ENSG00000161914.9	0	1
protein_coding	IL23A	ENSG00000110944.8	0	1
protein_coding	MYO16	ENSG00000041515.15	0	1
protein_coding	A1BG	ENSG00000121410.11	0	1
protein_coding	UNC79	ENSG00000133958.13	0	1
protein_coding	FSBP	ENSG00000265817.2	0	1
protein_coding	CBWD6	ENSG00000215126.10	0	1
protein_coding	COL4A3	ENSG00000169031.19	0	1
protein_coding	LRRC46	ENSG00000141294.9	0	1
protein_coding	TF	ENSG00000091513.15	0	1
protein_coding	EIF5AL1	ENSG00000253626.3	0	1
protein_coding	ACAD11	ENSG00000240303.7	0	1
protein_coding	ADGRG2	ENSG00000173698.17	0	1
protein_coding	NBPF9	ENSG00000269713.7	0	1
protein_coding	POPDC2	ENSG00000121577.13	0	1
protein_coding	LECT2	ENSG00000145826.8	0	1
protein_coding	PIWIL1	ENSG00000125207.7	0	1
protein_coding	RYR3	ENSG00000198838.13	0	1

protein_coding	EIF3C	ENSG00000184110.14	0	1
protein_coding	FADS6	ENSG00000172782.11	0	1
protein_coding	NLRP12	ENSG00000142405.21	0	1
protein_coding	WFDC10B	ENSG00000182931.9	0	1
protein_coding	FOXD4	ENSG00000170122.5	0	1
protein_coding	C6orf48	ENSG00000204387.13	0	1
protein_coding	ARF5	ENSG00000004059.10	0	1
protein_coding	PCDHGA9	ENSG00000261934.2	0	1
protein_coding	SLC19A3	ENSG00000135917.14	0	1
protein_coding	GREB1	ENSG00000196208.13	0	1
protein_coding	AC092835.1	ENSG00000233757.6	0	1
protein_coding	LYPD6B	ENSG00000150556.16	0	1
protein_coding	OR2C3	ENSG00000196242.9	0	1
protein_coding	TMSB15B	ENSG00000158427.14	0	1
protein_coding	ZBTB12	ENSG00000204366.3	0	1
protein_coding	INSL4	ENSG00000120211.4	0	1
protein_coding	ARHGAP11B	ENSG00000285077.1	0	1
protein_coding	CD37	ENSG00000104894.11	0	1
protein_coding	ST6GALNAC	ENSG00000184005.10	0	1
protein_coding	HSPA6	ENSG00000173110.7	0	1
protein_coding	TMIE	ENSG00000181585.4	0	1
protein_coding	LST1	ENSG00000204482.10	0	1
protein_coding	NOTCH4	ENSG00000204301.6	0	1
protein_coding	ADGRF5	ENSG00000069122.18	0	1
protein_coding	DDO	ENSG00000203797.9	0	1
protein_coding	ORM1	ENSG00000229314.5	0	1
protein_coding	CABP1	ENSG00000157782.9	0	1
protein_coding	TMED6	ENSG00000157315.4	0	1
protein_coding	DEFB118	ENSG00000131068.3	0	1
protein_coding	EXTL1	ENSG00000158008.9	0	1
protein_coding	BMP8A	ENSG00000183682.7	0	1
protein_coding	FOXE3	ENSG00000186790.5	0	1
protein_coding	DAB1	ENSG00000173406.15	0	1
protein_coding	KCNA3	ENSG00000177272.8	0	1
protein_coding	SYT6	ENSG00000134207.16	0	1
protein_coding	S100A5	ENSG00000196420.7	0	1
protein_coding	NUP210L	ENSG00000143552.9	0	1
protein_coding	OR10K2	ENSG00000180708.5	0	1
protein_coding	PYHIN1	ENSG00000163564.14	0	1
protein_coding	SLAMF8	ENSG00000158714.10	0	1
protein_coding	SELL	ENSG00000188404.8	0	1
protein_coding	FMO3	ENSG00000007933.12	0	1
protein_coding	OCLM	ENSG00000262180.1	0	1
protein_coding	KISS1	ENSG00000170498.8	0	1

protein_coding	MIXL1	ENSG00000185155.11	0	1
protein_coding	COLEC11	ENSG00000118004.17	0	1
protein_coding	C2orf16	ENSG00000221843.4	0	1
protein_coding	SLC11A1	ENSG00000018280.16	0	1
protein_coding	C2orf72	ENSG00000204128.5	0	1
protein_coding	GPR35	ENSG00000178623.11	0	1
protein_coding	LHFPL4	ENSG00000156959.8	0	1
protein_coding	XCR1	ENSG00000173578.7	0	1
protein_coding	SEMA3G	ENSG00000010319.6	0	1
protein_coding	FAM184B	ENSG00000047662.4	0	1
protein_coding	THEGL	ENSG00000249693.2	0	1
protein_coding	CXCL5	ENSG00000163735.6	0	1
protein_coding	COL25A1	ENSG00000188517.15	0	1
protein_coding	TTC29	ENSG00000137473.17	0	1
protein_coding	FAM218A	ENSG00000250486.4	0	1
protein_coding	ADCY2	ENSG00000078295.16	0	1
protein_coding	TSSK1B	ENSG00000212122.3	0	1
protein_coding	PCDHB5	ENSG00000113209.8	0	1
protein_coding	MYOZ3	ENSG00000164591.13	0	1
protein_coding	HUS1B	ENSG00000188996.4	0	1
protein_coding	C4A	ENSG00000244731.7	0	1
protein_coding	PTCRA	ENSG00000171611.9	0	1
protein_coding	LGSN	ENSG00000146166.16	0	1
protein_coding	AL355312.5	ENSG00000285991.1	0	1
protein_coding	PRR18	ENSG00000176381.5	0	1
protein_coding	AL159163.1	ENSG00000249141.1	0	1
protein_coding	NPC1L1	ENSG00000015520.14	0	1
protein_coding	C7orf57	ENSG00000164746.13	0	1
protein_coding	FKBP6	ENSG00000077800.12	0	1
protein_coding	CYP3A43	ENSG00000021461.16	0	1
protein_coding	MOGAT3	ENSG00000106384.11	0	1
protein_coding	EFCAB10	ENSG00000185055.10	0	1
protein_coding	PPP1R3A	ENSG00000154415.7	0	1
protein_coding	NOS3	ENSG00000164867.10	0	1
protein_coding	XKR5	ENSG00000275591.4	0	1
protein_coding	LPL	ENSG00000175445.15	0	1
protein_coding	DOK2	ENSG00000147443.12	0	1
protein_coding	NECAB1	ENSG00000123119.11	0	1
protein_coding	ERICH5	ENSG00000177459.10	0	1
protein_coding	SLC25A32	ENSG00000164933.11	0	1
protein_coding	RSPO2	ENSG00000147655.10	0	1
protein_coding	ARC	ENSG00000198576.3	0	1
protein_coding	TRPM6	ENSG00000119121.21	0	1
protein_coding	C9orf153	ENSG00000187753.13	0	1

protein_coding	HEMGN	ENSG00000136929.12	0	1
protein_coding	LAMC3	ENSG00000050555.17	0	1
protein_coding	SARDH	ENSG00000123453.17	0	1
protein_coding	CNGA4	ENSG00000132259.12	0	1
protein_coding	ANO3	ENSG00000134343.12	0	1
protein_coding	GNG3	ENSG00000162188.5	0	1
protein_coding	SNX32	ENSG00000172803.17	0	1
protein_coding	FOLR1	ENSG00000110195.12	0	1
protein_coding	APOA1	ENSG00000118137.9	0	1
protein_coding	ADAMTS8	ENSG00000134917.9	0	1
protein_coding	AC005833.1	ENSG00000255639.3	0	1
protein_coding	BIN2	ENSG00000110934.10	0	1
protein_coding	RAD9B	ENSG00000151164.18	0	1
protein_coding	HPD	ENSG00000158104.11	0	1
protein_coding	ADGRD1	ENSG00000111452.12	0	1
protein_coding	RASL11A	ENSG00000122035.6	0	1
protein_coding	F7	ENSG00000057593.13	0	1
protein_coding	RPGRIP1	ENSG00000092200.12	0	1
protein_coding	CIDEB	ENSG00000136305.11	0	1
protein_coding	NRXN3	ENSG00000021645.18	0	1
protein_coding	SERPINA3	ENSG00000196136.17	0	1
protein_coding	SNURF	ENSG00000273173.5	0	1
protein_coding	CHRNA7	ENSG00000175344.17	0	1
protein_coding	AC090517.4	ENSG00000285253.1	0	1
protein_coding	AQP9	ENSG00000103569.9	0	1
protein_coding	C16orf96	ENSG00000205832.7	0	1
protein_coding	OTOA	ENSG00000155719.17	0	1
protein_coding	BOLA2-	ENSG00000261740.6	0	1
protein_coding	CMTM1	ENSG00000089505.17	0	1
protein_coding	LRRC36	ENSG00000159708.17	0	1
protein_coding	CCL13	ENSG00000181374.7	0	1
protein_coding	SRCIN1	ENSG00000277363.4	0	1
protein_coding	KLHL10	ENSG00000161594.6	0	1
protein_coding	WNK4	ENSG00000126562.16	0	1
protein_coding	NOTUM	ENSG00000185269.11	0	1
protein_coding	FN3K	ENSG00000167363.13	0	1
protein_coding	SERPINB12	ENSG00000166634.6	0	1
protein_coding	CDH7	ENSG00000081138.13	0	1
protein_coding	NETO1	ENSG00000166342.18	0	1
protein_coding	GALR1	ENSG00000166573.5	0	1
protein_coding	SLC25A41	ENSG00000181240.13	0	1
protein_coding	PRR36	ENSG00000183248.11	0	1
protein_coding	AC010422.8	ENSG00000285589.1	0	1
protein_coding	F2RL3	ENSG00000127533.3	0	1

protein_coding	FXYD7	ENSG00000221946.7	0	1
protein_coding	GNG8	ENSG00000167414.4	0	1
protein_coding	IZUMO1	ENSG00000182264.8	0	1
protein_coding	RIMS4	ENSG00000101098.12	0	1
protein_coding	MRAP	ENSG00000170262.12	0	1
protein_coding	KCNE1	ENSG00000180509.11	0	1
protein_coding	GATD3A	ENSG00000160221.17	0	1
protein_coding	CCDC188	ENSG00000234409.6	0	1
protein_coding	FAM9C	ENSG00000187268.11	0	1
protein_coding	RAI2	ENSG00000131831.17	0	1
protein_coding	MAP3K15	ENSG00000180815.14	0	1
protein_coding	DCAF8L2	ENSG00000189186.10	0	1
protein_coding	WNK3	ENSG00000196632.10	0	1
protein_coding	MTMR8	ENSG00000102043.15	0	1
protein_coding	TAF7L	ENSG00000102387.15	0	1
protein_coding	RAB40A	ENSG00000172476.4	0	1
protein_coding	FRMD7	ENSG00000165694.9	0	1
protein_coding	IFITM1	ENSG00000185885.15	0	1
protein_coding	TMEM74B	ENSG00000125895.5	0	1
protein_coding	ST6GALNAC	ENSG00000160408.14	0	1
protein_coding	KLF8	ENSG00000102349.17	0	1
protein_coding	GPR3	ENSG00000181773.6	0	1
protein_coding	GJA8	ENSG00000121634.5	0	1
protein_coding	MOBP	ENSG00000168314.17	0	1
protein_coding	CCK	ENSG00000187094.11	0	1
protein_coding	EFCAB12	ENSG00000172771.11	0	1
protein_coding	TM4SF4	ENSG00000169903.6	0	1
protein_coding	GABRB1	ENSG00000163288.13	0	1
protein_coding	RASL11B	ENSG00000128045.6	0	1
protein_coding	ASB5	ENSG00000164122.8	0	1
protein_coding	MEGF10	ENSG00000145794.16	0	1
protein_coding	PPT2-EGFL8	ENSG00000258388.7	0	1
protein_coding	GUCA1A	ENSG00000048545.13	0	1
protein_coding	GFRA2	ENSG00000168546.10	0	1
protein_coding	SAA2	ENSG00000134339.8	0	1
protein_coding	CCDC177	ENSG00000267909.2	0	1
protein_coding	BAIAP3	ENSG00000007516.13	0	1
protein_coding	AC025283.2	ENSG00000262621.5	0	1
protein_coding	NPIPA5	ENSG00000183793.13	0	1
protein_coding	FAM131C	ENSG00000185519.8	0	1
protein_coding	DPT	ENSG00000143196.4	0	1
protein_coding	KIAA2012	ENSG00000182329.12	0	1
protein_coding	CLGN	ENSG00000153132.12	0	1
protein_coding	ISM2	ENSG00000100593.17	0	1

protein_coding	BCL2L10	ENSG00000137875.4	0	1
protein_coding	RND2	ENSG00000108830.9	0	1
protein_coding	GFAP	ENSG00000131095.12	0	1
protein_coding	CFP	ENSG00000126759.13	0	1
protein_coding	HIST1H4J	ENSG00000197238.4	0	1
protein_coding	NPIPB2	ENSG00000234719.8	0	1
protein_coding	CCIN	ENSG00000185972.5	0	1
protein_coding	BRINP1	ENSG00000078725.12	0	1
protein_coding	RASGRP2	ENSG00000068831.18	0	1
protein_coding	NTRK3	ENSG00000140538.16	0	1
protein_coding	SOX8	ENSG00000005513.9	0	1
protein_coding	TNFRSF17	ENSG00000048462.10	0	1
protein_coding	PTCHD1	ENSG00000165186.11	0	1
protein_coding	ZNF221	ENSG00000159905.14	0	1
protein_coding	SHC2	ENSG00000129946.10	0	1
protein_coding	SYT5	ENSG00000129990.14	0	1
protein_coding	CXCL2	ENSG00000081041.8	0	1
protein_coding	AMZ1	ENSG00000174945.13	0	1
protein_coding	GPR4	ENSG00000177464.4	0	1
protein_coding	NPPC	ENSG00000163273.3	0	1
protein_coding	GALR2	ENSG00000182687.3	0	1
protein_coding	ESYT3	ENSG00000158220.13	0	1
protein_coding	PCDHGB3	ENSG00000262209.2	0	1
protein_coding	HIST1H2AB	ENSG00000278463.1	0	1
protein_coding	KCTD14	ENSG00000151364.16	0	1
protein_coding	PRCD	ENSG00000214140.10	0	1
protein_coding	TNNI3	ENSG00000129991.12	0	1
protein_coding	CRACR2B	ENSG00000177685.16	0	1
protein_coding	TCAP	ENSG00000173991.5	0	1
protein_coding	KANK3	ENSG00000186994.11	0	1
protein_coding	EIF3L	ENSG00000100129.17	0	1
protein_coding	PPP1R42	ENSG00000178125.14	0	1
protein_coding	TECTA	ENSG00000109927.10	0	1
protein_coding	IQCN	ENSG00000130518.16	0	1
protein_coding	TMEM240	ENSG00000205090.8	0	1
protein_coding	STX11	ENSG00000135604.9	0	1
protein_coding	NBPF10	ENSG00000271425.7	0	1
protein_coding	HAVCR2	ENSG00000135077.8	0	1
protein_coding	RPP25	ENSG00000178718.6	0	1
protein_coding	SLA	ENSG00000155926.13	0	1
protein_coding	PTPN22	ENSG00000134242.15	0	1
protein_coding	TGM1	ENSG00000092295.11	0	1
protein_coding	ADM5	ENSG00000224420.3	0	1
protein_coding	CNGB3	ENSG00000170289.12	0	1

protein_coding	SULT1B1	ENSG00000173597.8	0	1
protein_coding	SUMO4	ENSG00000177688.6	0	1
protein_coding	Sep-14	ENSG00000154997.8	0	1
protein_coding	CNIH2	ENSG00000174871.10	0	1
protein_coding	NKD1	ENSG00000140807.6	0	1
protein_coding	AC092718.3	ENSG00000260643.2	0	1
protein_coding	UNC5D	ENSG00000156687.10	0	1
protein_coding	ZNF852	ENSG00000178917.16	0	1
protein_coding	ADRA2C	ENSG00000184160.7	0	1
protein_coding	C8G	ENSG00000176919.12	0	1
protein_coding	USP18	ENSG00000184979.9	0	1
protein_coding	ANAPC2	ENSG00000176248.8	0	1
protein_coding	NRTN	ENSG00000171119.2	0	1
protein_coding	FP565260.3	ENSG00000277117.4	0	1
protein_coding	PNMA3	ENSG00000183837.9	0	1
protein_coding	MFAP3L	ENSG00000198948.11	0	1
protein_coding	DMC1	ENSG00000100206.9	0	1
protein_coding	C7orf25	ENSG00000136197.12	0	1
protein_coding	KHK	ENSG00000138030.12	0	1
protein_coding	DYSF	ENSG00000135636.13	0	1
protein_coding	DLG2	ENSG00000150672.17	0	1
protein_coding	ITGB7	ENSG00000139626.15	0	1
protein_coding	SH3TC2	ENSG00000169247.12	0	1
protein_coding	FAM111B	ENSG00000189057.10	0	0
protein_coding	MCM10	ENSG00000065328.16	0	0
protein_coding	JPH1	ENSG00000104369.4	0	0
protein_coding	XRCC2	ENSG00000196584.2	0	0
protein_coding	GPAT3	ENSG00000138678.10	0	0
protein_coding	NTSR1	ENSG00000101188.4	0	0
protein_coding	POLE2	ENSG00000100479.12	0	0
protein_coding	KRT6B	ENSG00000185479.5	0	0
protein_coding	SAMD10	ENSG00000130590.13	0	0
protein_coding	CAMP	ENSG00000164047.4	0	0
protein_coding	RIBC2	ENSG00000128408.8	0	0
protein_coding	E2F8	ENSG00000129173.12	0	0
protein_coding	CHAC2	ENSG00000143942.4	0	0
protein_coding	NME2	ENSG00000243678.11	0	0
protein_coding	HPSE	ENSG00000173083.14	0	0
protein_coding	TNFRSF11A	ENSG00000141655.16	0	0
protein_coding	B4GALT6	ENSG00000118276.11	0	0
protein_coding	SPTSSB	ENSG00000196542.8	0	0
protein_coding	ARHGAP25	ENSG00000163219.11	0	0
protein_coding	TGFA	ENSG00000163235.15	0	0
protein_coding	PPARGC1B	ENSG00000155846.16	0	0

protein_coding	CPA6	ENSG00000165078.12	0	0
protein_coding	TM4SF19	ENSG00000145107.15	0	0
protein_coding	HIST1H1A	ENSG00000124610.4	0	0
protein_coding	ESCO2	ENSG00000171320.14	0	0
protein_coding	KRT18	ENSG00000111057.10	0	0
protein_coding	TPRG1	ENSG00000188001.9	0	0
protein_coding	PKMYT1	ENSG00000127564.16	0	0
protein_coding	PLLP	ENSG00000102934.9	0	0
protein_coding	CEACAM6	ENSG00000086548.8	0	0
protein_coding	KREMEN2	ENSG00000131650.13	0	0
protein_coding	EN2	ENSG00000164778.4	0	0
protein_coding	POU3F2	ENSG00000184486.9	0	0
protein_coding	DOK7	ENSG00000175920.17	0	0
protein_coding	CYP4F11	ENSG00000171903.16	0	0
protein_coding	ANKRD7	ENSG00000106013.14	0	0
protein_coding	EME1	ENSG00000154920.14	0	0
protein_coding	POLQ	ENSG00000051341.13	0	0
protein_coding	UHRF1	ENSG00000276043.4	0	0
protein_coding	ERCC6L	ENSG00000186871.6	0	0
protein_coding	SKA1	ENSG00000154839.9	0	0
protein_coding	HKDC1	ENSG00000156510.12	0	0
protein_coding	MYZAP	ENSG00000263155.5	0	0
protein_coding	KRT80	ENSG00000167767.13	0	0
protein_coding	ENHO	ENSG00000168913.6	0	0
protein_coding	PLAC8	ENSG00000145287.10	0	0
protein_coding	CARD11	ENSG00000198286.9	0	0
protein_coding	B3GNT3	ENSG00000179913.10	0	0
protein_coding	HS3ST1	ENSG00000002587.9	0	0
protein_coding	ICAM2	ENSG00000108622.10	0	0
protein_coding	NUP210	ENSG00000132182.11	0	0
protein_coding	RNF165	ENSG00000141622.13	0	0
protein_coding	KIF21B	ENSG00000116852.14	0	0
protein_coding	DMRTA1	ENSG00000176399.3	0	0
protein_coding	IL22RA1	ENSG00000142677.3	0	0
protein_coding	TFCP2L1	ENSG00000115112.7	0	0
protein_coding	SHH	ENSG00000164690.7	0	0
protein_coding	RDM1	ENSG00000278023.5	0	0
protein_coding	SPC25	ENSG00000152253.8	0	0
protein_coding	IL1A	ENSG00000115008.5	0	0
protein_coding	UGT8	ENSG00000174607.10	0	0
protein_coding	XDH	ENSG00000158125.9	0	0
protein_coding	S100A9	ENSG00000163220.10	0	0
protein_coding	ST6GALNAC	ENSG00000070526.14	0	0
protein_coding	HOXA11	ENSG00000005073.5	0	0

protein_coding	ERVMER34-1	ENSG00000226887.7	0	0
protein_coding	AMH	ENSG00000104899.7	0	0
protein_coding	SCNN1A	ENSG00000111319.12	0	0
protein_coding	SLC6A15	ENSG00000072041.16	0	0
protein_coding	TRPM2	ENSG00000142185.16	0	0
protein_coding	ELFN2	ENSG00000166897.14	0	0
protein_coding	ADGRF4	ENSG00000153294.11	0	0
protein_coding	CYP2B6	ENSG00000197408.9	0	0
protein_coding	AGR2	ENSG00000106541.11	0	0
protein_coding	CXCL11	ENSG00000169248.12	0	0
protein_coding	ACP7	ENSG00000183760.10	0	0
protein_coding	ABCB11	ENSG00000073734.9	0	0
protein_coding	RPS17	ENSG00000182774.12	0	0
protein_coding	ALDH1L1	ENSG00000144908.13	0	0
protein_coding	ISL1	ENSG00000016082.14	0	0
protein_coding	RP1	ENSG00000104237.9	0	0
protein_coding	SNX31	ENSG00000174226.8	0	0
protein_coding	KRT6C	ENSG00000170465.9	0	0
protein_coding	HASPIN	ENSG00000177602.5	0	0
protein_coding	U2AF1	ENSG00000160201.11	0	0
protein_coding	ZNF410	ENSG00000119725.18	0	0
protein_coding	TAS2R4	ENSG00000127364.3	0	0
protein_coding	FABP6	ENSG00000170231.15	0	0
protein_coding	CXCL1	ENSG00000163739.4	0	0
protein_coding	RELT	ENSG00000054967.12	0	0
protein_coding	CYP1A1	ENSG00000140465.13	0	0
protein_coding	FOXA2	ENSG00000125798.14	0	0
protein_coding	ZBED2	ENSG00000177494.5	0	0
protein_coding	ADGRF1	ENSG00000153292.15	0	0
protein_coding	MACC1	ENSG00000183742.12	0	0
protein_coding	HOXA13	ENSG00000106031.8	0	0
protein_coding	SH2D3A	ENSG00000125731.12	0	0
protein_coding	EOMES	ENSG00000163508.12	0	0
protein_coding	DMRT2	ENSG00000173253.15	0	0
protein_coding	C9orf84	ENSG00000165181.16	0	0
protein_coding	MYB	ENSG00000118513.18	0	0
protein_coding	ATP6V0D2	ENSG00000147614.3	0	0
protein_coding	PBK	ENSG00000168078.9	0	0
protein_coding	ARHGDIB	ENSG00000111348.8	0	0
protein_coding	CSMD3	ENSG00000164796.17	0	0
protein_coding	SOX21	ENSG00000125285.5	0	0
protein_coding	MNX1	ENSG00000130675.14	0	0
protein_coding	HCAR2	ENSG00000182782.7	0	0
protein_coding	LYPD5	ENSG00000159871.14	0	0

protein_coding	WNT7A	ENSG00000154764.5	0	0
protein_coding	SLC16A9	ENSG00000165449.11	0	0
protein_coding	DHRS9	ENSG00000073737.16	0	0
protein_coding	ANKRD18A	ENSG00000180071.19	0	0
protein_coding	VAX1	ENSG00000148704.12	0	0
protein_coding	FBXL13	ENSG00000161040.16	0	0
protein_coding	FCHO1	ENSG00000130475.14	0	0
protein_coding	ASRGL1	ENSG00000162174.12	0	0
protein_coding	ANKFN1	ENSG00000153930.11	0	0
protein_coding	SMIM24	ENSG00000095932.6	0	0
protein_coding	FAM72D	ENSG00000215784.5	0	0
protein_coding	ILDR2	ENSG00000143195.12	0	0
protein_coding	SULT1C2	ENSG00000198203.9	0	0
protein_coding	LHFPL1	ENSG00000182508.13	0	0
protein_coding	BNIPL	ENSG00000163141.19	0	0
protein_coding	MEI1	ENSG00000167077.12	0	0
protein_coding	CSPG5	ENSG00000114646.9	0	0
protein_coding	IGFL1	ENSG00000188293.5	0	0
protein_coding	AGAP2	ENSG00000135439.11	0	0
protein_coding	PAX5	ENSG00000196092.12	0	0
protein_coding	PRR15	ENSG00000176532.3	0	0
protein_coding	MAP3K9	ENSG00000006432.15	0	0
protein_coding	PKP2	ENSG00000057294.14	0	0
protein_coding	TMPRSS4	ENSG00000137648.17	0	0
protein_coding	REG1A	ENSG00000115386.5	0	0
protein_coding	TMEM156	ENSG00000121895.7	0	0
protein_coding	GJB5	ENSG00000189280.3	0	0
protein_coding	UGT1A1	ENSG00000241635.7	0	0
protein_coding	IL20RA	ENSG00000016402.13	0	0
protein_coding	GSDMC	ENSG00000147697.8	0	0
protein_coding	SYNDIG1	ENSG00000101463.5	0	0
protein_coding	DRC3	ENSG00000171962.17	0	0
protein_coding	TMPRSS7	ENSG00000176040.13	0	0
protein_coding	PITX2	ENSG00000164093.16	0	0
protein_coding	AIF1L	ENSG00000126878.12	0	0
protein_coding	CALB1	ENSG00000104327.7	0	0
protein_coding	GNG4	ENSG00000168243.10	0	0
protein_coding	SNX10	ENSG00000086300.15	0	0
protein_coding	PLS1	ENSG00000120756.12	0	0
protein_coding	NELL2	ENSG00000184613.10	0	0
protein_coding	SP5	ENSG00000204335.3	0	0
protein_coding	SEMA5B	ENSG00000082684.15	0	0
protein_coding	GAST	ENSG00000184502.3	0	0
protein_coding	BRSK2	ENSG00000174672.15	0	0

protein_coding	AKAP5	ENSG00000179841.8	0	0
protein_coding	ACOXL	ENSG00000153093.18	0	0
protein_coding	RNF182	ENSG00000180537.12	0	0
protein_coding	MYH15	ENSG00000144821.9	0	0
protein_coding	RAP1GAP	ENSG00000076864.19	0	0
protein_coding	ARHGEF26	ENSG00000114790.12	0	0
protein_coding	CLCA4	ENSG00000016602.9	0	0
protein_coding	COL9A3	ENSG00000092758.17	0	0
protein_coding	MST1R	ENSG00000164078.12	0	0
protein_coding	PTPRZ1	ENSG00000106278.11	0	0
protein_coding	ZNF488	ENSG00000265763.3	0	0
protein_coding	ZG16B	ENSG00000162078.11	0	0
protein_coding	FGFBP1	ENSG00000137440.4	0	0
protein_coding	HSD17B8	ENSG00000204228.3	0	0
protein_coding	CAMK2N2	ENSG00000163888.3	0	0
protein_coding	CDX2	ENSG00000165556.9	0	0
protein_coding	ONECUT2	ENSG00000119547.5	0	0
protein_coding	F2RL1	ENSG00000164251.4	0	0
protein_coding	NUP62CL	ENSG00000198088.10	0	0
protein_coding	INPP5D	ENSG00000168918.13	0	0
protein_coding	ZNF860	ENSG00000197385.5	0	0
protein_coding	RNF39	ENSG00000204618.8	0	0
protein_coding	SLCO1A2	ENSG00000084453.16	0	0
protein_coding	LAD1	ENSG00000159166.13	0	0
protein_coding	CP	ENSG00000047457.13	0	0
protein_coding	OVOL2	ENSG00000125850.10	0	0
protein_coding	MARK1	ENSG00000116141.15	0	0
protein_coding	SPINK5	ENSG00000133710.15	0	0
protein_coding	BARX2	ENSG00000043039.6	0	0
protein_coding	GRHL2	ENSG00000083307.11	0	0
protein_coding	ATP2C2	ENSG00000064270.12	0	0
protein_coding	DAPP1	ENSG00000070190.12	0	0
protein_coding	PLCH2	ENSG00000149527.17	0	0
protein_coding	GJB4	ENSG00000189433.5	0	0
protein_coding	BTBD16	ENSG00000138152.8	0	0
protein_coding	SLC5A12	ENSG00000148942.14	0	0
protein_coding	FBXL22	ENSG00000197361.7	0	0
protein_coding	RASGEF1B	ENSG00000138670.17	0	0
protein_coding	NOCT	ENSG00000151014.5	0	0
protein_coding	HIST1H4D	ENSG00000277157.1	0	0
protein_coding	GRM4	ENSG00000124493.13	0	0
protein_coding	DRD4	ENSG00000069696.6	0	0
protein_coding	TPH1	ENSG00000129167.9	0	0
protein_coding	SDR42E2	ENSG00000183921.7	0	0

protein_coding	BOLA2B	ENSG00000169627.7	0	0
protein_coding	C2CD4C	ENSG00000183186.7	0	0
protein_coding	SHE	ENSG00000169291.9	0	0
protein_coding	INHA	ENSG00000123999.4	0	0
protein_coding	SNORC	ENSG00000182600.9	0	0
protein_coding	CXCL9	ENSG00000138755.5	0	0
protein_coding	HIST1H2BF	ENSG00000277224.2	0	0
protein_coding	CNTN5	ENSG00000149972.10	0	0
protein_coding	KLRF1	ENSG00000150045.11	0	0
protein_coding	KLHL11	ENSG00000178502.5	0	0
protein_coding	RNF125	ENSG00000101695.8	0	0
protein_coding	TMEM191C	ENSG00000206140.10	0	0
protein_coding	P2RY10	ENSG00000078589.12	0	0
protein_coding	TMEM253	ENSG00000232070.8	0	0
protein_coding	FAM72C	ENSG00000263513.5	0	0
protein_coding	AGER	ENSG00000204305.13	0	0
protein_coding	SYCE3	ENSG00000217442.3	0	0
protein_coding	ZSCAN4	ENSG00000180532.10	0	0
protein_coding	AC007325.4	ENSG00000278817.1	0	0
protein_coding	HOXB5	ENSG00000120075.5	0	0
protein_coding	XK	ENSG00000047597.6	0	0
protein_coding	MDH1B	ENSG00000138400.12	0	0
protein_coding	SCEL	ENSG00000136155.16	0	0
protein_coding	GLRA3	ENSG00000145451.12	0	0
protein_coding	CCDC78	ENSG00000162004.16	0	0
protein_coding	AL031708.1	ENSG00000261732.1	0	0
protein_coding	GRAMD2A	ENSG00000175318.11	0	0
protein_coding	CEACAM1	ENSG00000079385.21	0	0
protein_coding	NIPAL4	ENSG00000172548.14	0	0
protein_coding	PIFO	ENSG00000173947.13	0	0
protein_coding	ARHGEF35	ENSG00000213214.4	0	0
protein_coding	TMC5	ENSG00000103534.16	0	0
protein_coding	GIMAP6	ENSG00000133561.15	0	0
protein_coding	CDK18	ENSG00000117266.15	0	0
protein_coding	LCN2	ENSG00000148346.11	0	0
protein_coding	SLC52A3	ENSG00000101276.15	0	0
protein_coding	DHRS2	ENSG00000100867.14	0	0
protein_coding	ZIC5	ENSG00000139800.8	0	0
protein_coding	CD1D	ENSG00000158473.6	0	0
protein_coding	HOOK1	ENSG00000134709.10	0	0
protein_coding	CXorf57	ENSG00000147231.13	0	0
protein_coding	HS6ST2	ENSG00000171004.18	0	0
protein_coding	HOXA1	ENSG00000105991.8	0	0
protein_coding	PDGFB	ENSG00000100311.16	0	0

protein_coding	PDLIM3	ENSG00000154553.14	0	0
protein_coding	MUCL1	ENSG00000172551.10	0	0
protein_coding	ESRP2	ENSG00000103067.13	0	0
protein_coding	IL18	ENSG00000150782.11	0	0
protein_coding	SMIM22	ENSG00000267795.5	0	0
protein_coding	NRARP	ENSG00000198435.3	0	0
protein_coding	S100A8	ENSG00000143546.9	0	0
protein_coding	LHX1	ENSG00000273706.4	0	0
protein_coding	FGF9	ENSG00000102678.6	0	0
protein_coding	ISL2	ENSG00000159556.9	0	0
protein_coding	TNNT3	ENSG00000130595.18	0	0
protein_coding	CNTNAP3B	ENSG00000154529.14	0	0
protein_coding	RAB25	ENSG00000132698.14	0	0
protein_coding	FAM181B	ENSG00000182103.4	0	0
protein_coding	MFSD2A	ENSG00000168389.17	0	0
protein_coding	PAOX	ENSG00000148832.15	0	0
protein_coding	PLAC1	ENSG00000170965.9	0	0
protein_coding	WDR72	ENSG00000166415.14	0	0
protein_coding	KRT6A	ENSG00000205420.10	0	0
protein_coding	HIST1H3G	ENSG00000273983.1	0	0
protein_coding	WDR17	ENSG00000150627.15	0	0
protein_coding	ADAMTS16	ENSG00000145536.15	0	0
protein_coding	RIPPLY2	ENSG00000203877.8	0	0
protein_coding	GAP43	ENSG00000172020.12	0	0
protein_coding	PLA2G10	ENSG00000069764.9	0	0
protein_coding	SOX7	ENSG00000171056.7	0	0
protein_coding	HOXA10	ENSG00000253293.4	0	0
protein_coding	CLDN4	ENSG00000189143.9	0	0
protein_coding	EVPL	ENSG00000167880.7	0	0
protein_coding	TRHDE	ENSG00000072657.8	0	0
protein_coding	SLC16A10	ENSG00000112394.16	0	0
protein_coding	GTSF1	ENSG00000170627.10	0	0
protein_coding	GPR37	ENSG00000170775.2	0	0
protein_coding	ADAM22	ENSG00000008277.14	0	0
protein_coding	HOXB7	ENSG00000260027.4	0	0
protein_coding	LPAR5	ENSG00000184574.9	0	0
protein_coding	FUT3	ENSG00000171124.13	0	0
protein_coding	ENTPD3	ENSG00000168032.9	0	0
protein_coding	RTKN2	ENSG00000182010.10	0	0
protein_coding	HORMAD1	ENSG00000143452.15	0	0
protein_coding	LARGE2	ENSG00000165905.17	0	0
protein_coding	SPARCL1	ENSG00000152583.12	0	0
protein_coding	FA2H	ENSG00000103089.8	0	0
protein_coding	TREM1	ENSG00000124731.12	0	0

protein_coding	LINGO2	ENSG00000174482.10	0	0
protein_coding	SIRPB1	ENSG00000101307.15	0	0
protein_coding	PAQR6	ENSG00000160781.16	0	0
protein_coding	TMEM63C	ENSG00000165548.10	0	0
protein_coding	ABO	ENSG00000175164.14	0	0
protein_coding	FGF13	ENSG00000129682.15	0	0
protein_coding	VSIG1	ENSG00000101842.13	0	0
protein_coding	NKX1-2	ENSG00000229544.8	0	0
protein_coding	RLN2	ENSG00000107014.8	0	0
protein_coding	PHF21B	ENSG00000056487.15	0	0
protein_coding	C12orf56	ENSG00000185306.12	0	0
protein_coding	MAGEB2	ENSG00000099399.5	0	0
protein_coding	PAK6	ENSG00000137843.11	0	0
protein_coding	PLCXD2	ENSG00000240891.7	0	0
protein_coding	SOX2	ENSG00000181449.3	0	0
protein_coding	SERPINB5	ENSG00000206075.13	0	0
protein_coding	TMC6	ENSG00000141524.15	0	0
protein_coding	LCP1	ENSG00000136167.13	0	0
protein_coding	NKX2-8	ENSG00000136327.6	0	0
protein_coding	ARHGEF16	ENSG00000130762.14	0	0
protein_coding	TPD52	ENSG00000076554.15	0	0
protein_coding	CTSV	ENSG00000136943.10	0	0
protein_coding	PTAFR	ENSG00000169403.11	0	0
protein_coding	AP1M2	ENSG00000129354.11	0	0
protein_coding	PCDH1	ENSG00000156453.13	0	0
protein_coding	KLK10	ENSG00000129451.11	0	0
protein_coding	PLXNA4	ENSG00000221866.9	0	0
protein_coding	GPR27	ENSG00000170837.2	0	0
protein_coding	CPVL	ENSG00000106066.14	0	0
protein_coding	UNC13D	ENSG00000092929.11	0	0
protein_coding	MAL2	ENSG00000147676.13	0	0
protein_coding	EPHA1	ENSG00000146904.8	0	0
protein_coding	TREM2	ENSG00000095970.16	0	0
protein_coding	S100A14	ENSG00000189334.8	0	0
protein_coding	KRT17	ENSG00000128422.16	0	0
protein_coding	MARVELD3	ENSG00000140832.9	0	0
protein_coding	GJB2	ENSG00000165474.6	0	0
protein_coding	ELOVL7	ENSG00000164181.13	0	0
protein_coding	C6orf141	ENSG00000197261.11	0	0
protein_coding	TNNI2	ENSG00000130598.15	0	0
protein_coding	MDFI	ENSG00000112559.13	0	0
protein_coding	RHPN1	ENSG00000158106.13	0	0
protein_coding	ANKRD2	ENSG00000165887.11	0	0
protein_coding	IL13RA2	ENSG00000123496.7	0	0

protein_coding	LANCL3	ENSG00000147036.11	0	0
protein_coding	ERG	ENSG00000157554.18	0	0
protein_coding	SLC27A2	ENSG00000140284.10	0	0
protein_coding	BEX5	ENSG00000184515.10	0	0
protein_coding	CXADR	ENSG00000154639.18	0	0
protein_coding	RNF43	ENSG00000108375.12	0	0
protein_coding	FBXO16	ENSG00000214050.7	0	0
protein_coding	ELAVL2	ENSG00000107105.14	0	0
protein_coding	C19orf33	ENSG00000167644.11	0	0
protein_coding	IGSF11	ENSG00000144847.12	0	0
protein_coding	STOX1	ENSG00000165730.15	0	0
protein_coding	POU4F1	ENSG00000152192.7	0	0
protein_coding	C12orf54	ENSG00000177627.10	0	0
protein_coding	DSG2	ENSG00000046604.12	0	0
protein_coding	HOXC4	ENSG00000198353.7	0	0
protein_coding	PADI3	ENSG00000142619.4	0	0
protein_coding	EDDM13	ENSG00000267710.8	0	0
protein_coding	HOXD13	ENSG00000128714.5	0	0
protein_coding	FAM83B	ENSG00000168143.8	0	0
protein_coding	VSNL1	ENSG00000163032.11	0	0
protein_coding	GRB7	ENSG00000141738.13	0	0
protein_coding	OOEP	ENSG00000203907.9	0	0
protein_coding	COL17A1	ENSG00000065618.19	0	0
protein_coding	C2orf15	ENSG00000273045.6	0	0
protein_coding	DNAH11	ENSG00000105877.17	0	0
protein_coding	NKX2-5	ENSG00000183072.9	0	0
protein_coding	KCNB2	ENSG00000182674.5	0	0
protein_coding	NKX2-1	ENSG00000136352.17	0	0
protein_coding	IQCC	ENSG00000160051.11	0	0
protein_coding	PDZD2	ENSG00000133401.15	0	0
protein_coding	P2RY2	ENSG00000175591.11	0	0
protein_coding	CHRM3	ENSG00000133019.11	0	0
protein_coding	C16orf54	ENSG00000185905.3	0	0
protein_coding	EAF2	ENSG00000145088.8	0	0
protein_coding	SYK	ENSG00000165025.14	0	0
protein_coding	KRT222	ENSG00000213424.8	0	0
protein_coding	EPHA7	ENSG00000135333.13	0	0
protein_coding	IL31RA	ENSG00000164509.13	0	0
protein_coding	HOXA5	ENSG00000106004.4	0	0
protein_coding	CDC25C	ENSG00000158402.19	0	0
protein_coding	IRX1	ENSG00000170549.3	0	0
protein_coding	ESRP1	ENSG00000104413.16	0	0
protein_coding	HOXC10	ENSG00000180818.4	0	0
protein_coding	CRB3	ENSG00000130545.15	0	0

protein_coding	DNHD1	ENSG00000179532.12	0	0
protein_coding	SYT17	ENSG00000103528.16	0	0
protein_coding	WNT7B	ENSG00000188064.9	0	0
protein_coding	CLDN8	ENSG00000156284.5	0	0
protein_coding	ST14	ENSG00000149418.10	0	0
protein_coding	CACNG4	ENSG00000075461.5	0	0
protein_coding	SPICE1	ENSG00000163611.11	0	0
protein_coding	ANXA8	ENSG00000265190.6	0	0
protein_coding	HOXA3	ENSG00000105997.22	0	0
protein_coding	CDH3	ENSG00000062038.13	0	0
protein_coding	DMTN	ENSG00000158856.18	0	0
protein_coding	C1orf116	ENSG00000182795.12	0	0
protein_coding	HTR2C	ENSG00000147246.9	0	0
protein_coding	PPP2R2C	ENSG00000074211.13	0	0
protein_coding	KLHL4	ENSG00000102271.13	0	0
protein_coding	MISP	ENSG00000099812.8	0	0
protein_coding	PIK3C2G	ENSG00000139144.9	0	0
protein_coding	TENM1	ENSG00000009694.13	0	0
protein_coding	SEMA6A	ENSG00000092421.16	0	0
protein_coding	SPOCK3	ENSG00000196104.10	0	0
protein_coding	KDF1	ENSG00000175707.8	0	0
protein_coding	POF1B	ENSG00000124429.17	0	0
protein_coding	SLCO1B3	ENSG00000111700.12	0	0
protein_coding	KLK11	ENSG00000167757.13	0	0
protein_coding	SV2C	ENSG00000122012.13	0	0
protein_coding	CD70	ENSG00000125726.10	0	0
protein_coding	TCN1	ENSG00000134827.7	0	0
protein_coding	CTXN3	ENSG00000205279.8	0	0
protein_coding	HOXB3	ENSG00000120093.11	0	0
protein_coding	ZNF20	ENSG00000132010.15	0	0
protein_coding	FAM241B	ENSG00000171224.8	0	0
protein_coding	C11orf65	ENSG00000166323.12	0	0
protein_coding	EVPLL	ENSG00000214860.4	0	0
protein_coding	HOXB4	ENSG00000182742.5	0	0
protein_coding	GATM	ENSG00000171766.15	0	0
protein_coding	KCP	ENSG00000135253.13	0	0
protein_coding	C9orf66	ENSG00000183784.7	0	0
protein_coding	BICDL2	ENSG00000162069.15	0	0
protein_coding	LYG1	ENSG00000144214.9	0	0
protein_coding	POU5F2	ENSG00000248483.6	0	0
protein_coding	HOXA2	ENSG00000105996.6	0	0
protein_coding	MAT1A	ENSG00000151224.12	0	0
protein_coding	LSP1	ENSG00000130592.15	0	0
protein_coding	TBX6	ENSG00000149922.10	0	0

protein_coding	PRSS8	ENSG00000052344.15	0	0
protein_coding	TEX45	ENSG00000198723.10	0	0
protein_coding	CRHBP	ENSG00000145708.10	0	0
protein_coding	OTUD7A	ENSG00000169918.9	0	0
protein_coding	CYP4F12	ENSG00000186204.14	0	0
protein_coding	RBBP8NL	ENSG00000130701.3	0	0
protein_coding	MAK	ENSG00000111837.11	0	0
protein_coding	AOC1	ENSG00000002726.20	0	0
protein_coding	PTPRN2	ENSG00000155093.18	0	0
protein_coding	TH	ENSG00000180176.14	0	0
protein_coding	AC104389.4	ENSG00000239920.2	0	0
protein_coding	TMEM45B	ENSG00000151715.7	0	0
protein_coding	HIST4H4	ENSG00000197837.3	0	0
protein_coding	PABPC3	ENSG00000151846.8	0	0
protein_coding	LRRC9	ENSG00000131951.11	0	0
protein_coding	KRT31	ENSG00000094796.4	0	0
protein_coding	ANGPTL6	ENSG00000130812.10	0	0
protein_coding	RTBDN	ENSG00000132026.13	0	0
protein_coding	FAM83C	ENSG00000125998.7	0	0
protein_coding	ALPP	ENSG00000163283.6	0	0
protein_coding	MCMDC2	ENSG00000178460.17	0	0
protein_coding	LRMP	ENSG00000118308.15	0	0
protein_coding	KRTAP4-1	ENSG00000198443.6	0	0
protein_coding	KLRG2	ENSG00000188883.4	0	0
protein_coding	MMP7	ENSG00000137673.8	0	0
protein_coding	SEMA6B	ENSG00000167680.15	0	0
protein_coding	TTC34	ENSG00000215912.12	0	0
protein_coding	FAM72A	ENSG00000196550.10	0	0
protein_coding	HOXD1	ENSG00000128645.14	0	0
protein_coding	UGT1A6	ENSG00000167165.18	0	0
protein_coding	SPATA12	ENSG00000186451.1	0	0
protein_coding	PEX5L	ENSG00000114757.18	0	0
protein_coding	SULT1E1	ENSG00000109193.11	0	0
protein_coding	EGF	ENSG00000138798.11	0	0
protein_coding	TIGD4	ENSG00000169989.2	0	0
protein_coding	CDH12	ENSG00000154162.14	0	0
protein_coding	FSTL4	ENSG00000053108.16	0	0
protein_coding	GFRA3	ENSG00000146013.10	0	0
protein_coding	PRRT4	ENSG00000224940.8	0	0
protein_coding	RP1L1	ENSG00000183638.5	0	0
protein_coding	AK8	ENSG00000165695.9	0	0
protein_coding	RNF224	ENSG00000233198.3	0	0
protein_coding	PATE2	ENSG00000196844.8	0	0
protein_coding	UBE2L5	ENSG00000236444.4	0	0

protein_coding	DEGS2	ENSG00000168350.7	0	0
protein_coding	AC091057.6	ENSG00000284906.1	0	0
protein_coding	TMEM266	ENSG00000169758.12	0	0
protein_coding	AARSD1	ENSG00000266967.6	0	0
protein_coding	SGCA	ENSG00000108823.15	0	0
protein_coding	LRG1	ENSG00000171236.9	0	0
protein_coding	AIFM3	ENSG00000183773.15	0	0
protein_coding	EIF1AY	ENSG00000198692.9	0	0
protein_coding	CFHR4	ENSG00000134365.13	0	0
protein_coding	LAMP3	ENSG00000078081.7	0	0
protein_coding	NPY1R	ENSG00000164128.6	0	0
protein_coding	HOXB9	ENSG00000170689.9	0	0
protein_coding	CNGA1	ENSG00000198515.13	0	0
protein_coding	FOXO6	ENSG00000204060.7	0	0
protein_coding	GRIA2	ENSG00000120251.19	0	0
protein_coding	DBF4B	ENSG00000161692.17	0	0
protein_coding	CASQ2	ENSG00000118729.11	0	0
protein_coding	ASTN1	ENSG00000152092.15	0	0
protein_coding	CYTH4	ENSG00000100055.20	0	0
protein_coding	KLK1	ENSG00000167748.10	0	0
protein_coding	PGBD5	ENSG00000177614.10	0	0
protein_coding	FAM133A	ENSG00000179083.6	0	0
protein_coding	RNASE1	ENSG00000129538.13	0	0
protein_coding	IZUMO4	ENSG00000099840.13	0	0
protein_coding	CECR2	ENSG00000099954.18	0	0
protein_coding	CPXCR1	ENSG00000147183.9	0	0
protein_coding	SAMD13	ENSG00000203943.8	0	0
protein_coding	GREB1L	ENSG00000141449.14	0	0
protein_coding	CNKSR1	ENSG00000142675.17	0	0
protein_coding	CNTNAP3	ENSG00000106714.17	0	0
protein_coding	AGMO	ENSG00000187546.13	0	0
protein_coding	LIPH	ENSG00000163898.9	0	0
protein_coding	VSIG8	ENSG00000243284.1	0	0
protein_coding	RGS9BP	ENSG00000186326.3	0	0
protein_coding	PI3	ENSG00000124102.4	0	0
protein_coding	PDE11A	ENSG00000128655.17	0	0
protein_coding	CD226	ENSG00000150637.8	0	0
protein_coding	LMNTD2	ENSG00000185522.8	0	0
protein_coding	NCAM1	ENSG00000149294.16	0	0
protein_coding	NTRK2	ENSG00000148053.15	0	0
protein_coding	ARHGAP45	ENSG00000180448.10	0	0
protein_coding	EFCAB6	ENSG00000186976.14	0	0
protein_coding	DHDH	ENSG00000104808.7	0	0
protein_coding	ACER2	ENSG00000177076.5	0	0

protein_coding	ZNF169	ENSG00000175787.16	0	0
protein_coding	MTRNR2L1	ENSG00000256618.2	0	0
protein_coding	CDH1	ENSG00000039068.18	0	0
protein_coding	CHMP4C	ENSG00000164695.4	0	0
protein_coding	FAM84B	ENSG00000168672.3	0	0
protein_coding	TP73	ENSG00000078900.14	0	0
protein_coding	ANO9	ENSG00000185101.12	0	0
protein_coding	HLA-DQB1	ENSG00000179344.16	0	0
protein_coding	CNTNAP2	ENSG00000174469.21	0	0
protein_coding	ERBB3	ENSG00000065361.15	0	0
protein_coding	CCDC73	ENSG00000186714.12	0	0
protein_coding	SFMBT2	ENSG00000198879.11	0	0
protein_coding	EHF	ENSG00000135373.12	0	0
protein_coding	TSTD1	ENSG00000215845.10	0	0
protein_coding	SYT8	ENSG00000149043.16	0	0
protein_coding	PLEKHH1	ENSG00000054690.13	0	0
protein_coding	RAG1	ENSG00000166349.9	0	0
protein_coding	SPINT1	ENSG00000166145.14	0	0
protein_coding	STK31	ENSG00000196335.12	0	0
protein_coding	IGSF9	ENSG00000085552.16	0	0
protein_coding	DEF6	ENSG00000023892.10	0	0
protein_coding	HOXA7	ENSG00000122592.7	0	0
protein_coding	TMEM40	ENSG00000088726.15	0	0
protein_coding	FUT1	ENSG00000174951.11	0	0
protein_coding	DOCK8	ENSG00000107099.15	0	0
protein_coding	TRIM36	ENSG00000152503.9	0	0
protein_coding	PTPN6	ENSG00000111679.16	0	0
protein_coding	PCLO	ENSG00000186472.19	0	0
protein_coding	ZNF713	ENSG00000178665.15	0	0
protein_coding	BICDL1	ENSG00000135127.11	0	0
protein_coding	HOXC11	ENSG00000123388.4	0	0
protein_coding	NRN1	ENSG00000124785.8	0	0
protein_coding	PRRG2	ENSG00000126460.10	0	0
protein_coding	CCNO	ENSG00000152669.8	0	0
protein_coding	ABCA12	ENSG00000144452.14	0	0
protein_coding	GPR87	ENSG00000138271.5	0	0
protein_coding	CAPNS2	ENSG00000256812.1	0	0
protein_coding	IFNE	ENSG00000184995.7	0	0
protein_coding	CBLC	ENSG00000142273.12	0	0
protein_coding	COL4A5	ENSG00000188153.13	0	0
protein_coding	WFDC2	ENSG00000101443.17	0	0
protein_coding	TMPRSS15	ENSG00000154646.8	0	0
protein_coding	C17orf64	ENSG00000141371.12	0	0
protein_coding	NGFR	ENSG00000064300.8	0	0

protein_coding	PRKCQ	ENSG00000065675.14	0	0
protein_coding	TSPAN7	ENSG00000156298.12	0	0
protein_coding	KRT14	ENSG00000186847.5	0	0
protein_coding	KLK6	ENSG00000167755.14	0	0
protein_coding	GCA	ENSG00000115271.10	0	0
protein_coding	KRT5	ENSG00000186081.11	0	0
protein_coding	PAIP2B	ENSG00000124374.8	0	0
protein_coding	CAGE1	ENSG00000164304.15	0	0
protein_coding	CYP4F3	ENSG00000186529.15	0	0
protein_coding	NCF2	ENSG00000116701.14	0	0
protein_coding	LRRC7	ENSG00000033122.18	0	0
protein_coding	HS3ST2	ENSG00000122254.6	0	0
protein_coding	MMRN2	ENSG00000173269.13	0	0
protein_coding	HOXD11	ENSG00000128713.13	0	0
protein_coding	F11R	ENSG00000158769.17	0	0
protein_coding	RBP1	ENSG00000114115.9	0	0
protein_coding	FAM110C	ENSG00000184731.5	0	0
protein_coding	P2RY1	ENSG00000169860.6	0	0
protein_coding	HERC5	ENSG00000138646.8	0	0
protein_coding	FOXB1	ENSG00000171956.6	0	0
protein_coding	PNLIPRP3	ENSG00000203837.4	0	0
protein_coding	CCNA1	ENSG00000133101.9	0	0
protein_coding	SCN3B	ENSG00000166257.8	0	0
protein_coding	RASGRF1	ENSG00000058335.15	0	0
protein_coding	PRRG4	ENSG00000135378.3	0	0
protein_coding	EDAR	ENSG00000135960.9	0	0
protein_coding	COL4A6	ENSG00000197565.15	0	0
protein_coding	SCGB1A1	ENSG00000149021.6	0	0
protein_coding	CDC42BPG	ENSG00000171219.8	0	0
protein_coding	HOXC9	ENSG00000180806.4	0	0
protein_coding	SYTL1	ENSG00000142765.17	0	0
protein_coding	CA2	ENSG00000104267.9	0	0
protein_coding	TMEM105	ENSG00000185332.7	0	0
protein_coding	NAP1L3	ENSG00000186310.9	0	0
protein_coding	CDS1	ENSG00000163624.5	0	0
protein_coding	PPP1R14C	ENSG00000198729.4	0	0
protein_coding	CKMT1A	ENSG00000223572.9	0	0
protein_coding	LRRC61	ENSG00000127399.14	0	0
protein_coding	EPHA3	ENSG00000044524.10	0	0
protein_coding	CRYBG2	ENSG00000176092.15	0	0
protein_coding	CCDC68	ENSG00000166510.13	0	0
protein_coding	STOX2	ENSG00000173320.11	0	0
protein_coding	GJA3	ENSG00000121743.3	0	0
protein_coding	XCL1	ENSG00000143184.4	0	0

protein_coding	HOXB13	ENSG00000159184.7	0	0
protein_coding	TMC8	ENSG00000167895.14	0	0
protein_coding	PCDHB12	ENSG00000120328.6	0	0
protein_coding	RDH16	ENSG00000139547.7	0	0
protein_coding	NR2E1	ENSG00000112333.11	0	0
protein_coding	ZBED6CL	ENSG00000188707.5	0	0
protein_coding	CAPN6	ENSG00000077274.8	0	0
protein_coding	ZYG11A	ENSG00000203995.9	0	0
protein_coding	SLCO1B1	ENSG00000134538.2	0	0
protein_coding	B4GALNT3	ENSG00000139044.11	0	0
protein_coding	COL2A1	ENSG00000139219.18	0	0
protein_coding	GRHL3	ENSG00000158055.15	0	0
protein_coding	ISLR2	ENSG00000167178.15	0	0
protein_coding	IQCD	ENSG00000166578.9	0	0
protein_coding	TRIM29	ENSG00000137699.16	0	0
protein_coding	MPZL2	ENSG00000149573.8	0	0
protein_coding	GATA3	ENSG00000107485.16	0	0
protein_coding	C5orf38	ENSG00000186493.12	0	0
protein_coding	SPAG17	ENSG00000155761.13	0	0
protein_coding	ADRB1	ENSG00000043591.5	0	0
protein_coding	IRX4	ENSG00000113430.9	0	0
protein_coding	CEBPA	ENSG00000245848.2	0	0
protein_coding	NLGN4X	ENSG00000146938.15	0	0
protein_coding	ITGB6	ENSG00000115221.11	0	0
protein_coding	FHIT	ENSG00000189283.9	0	0
protein_coding	TTC22	ENSG00000006555.10	0	0
protein_coding	MARCH1	ENSG00000145416.13	0	0
protein_coding	SERPINB3	ENSG00000057149.15	0	0
protein_coding	RASSF6	ENSG00000169435.13	0	0
protein_coding	EPB41L4A	ENSG00000129595.12	0	0
protein_coding	CLDN7	ENSG00000181885.18	0	0
protein_coding	GPR158	ENSG00000151025.10	0	0
protein_coding	HOXA4	ENSG00000197576.13	0	0
protein_coding	BANK1	ENSG00000153064.11	0	0
protein_coding	BIK	ENSG00000100290.2	0	0
protein_coding	ARAP2	ENSG00000047365.11	0	0
protein_coding	SLC15A2	ENSG00000163406.10	0	0
protein_coding	ANK3	ENSG00000151150.21	0	0
protein_coding	HOXD9	ENSG00000128709.12	0	0
protein_coding	ADRB2	ENSG00000169252.5	0	0
protein_coding	MYCL	ENSG00000116990.10	0	0
protein_coding	IQCH	ENSG00000103599.19	0	0
protein_coding	RPS4Y1	ENSG00000129824.15	0	0
protein_coding	FAHD2B	ENSG00000144199.11	0	0

protein_coding	DOC2A	ENSG00000149927.17	0	0
protein_coding	LCE5A	ENSG00000186207.4	0	0
protein_coding	LHX9	ENSG00000143355.15	0	0
protein_coding	WNT6	ENSG00000115596.3	0	0
protein_coding	MKRN2OS	ENSG00000225526.4	0	0
protein_coding	ENO4	ENSG00000188316.13	0	0
protein_coding	REEP1	ENSG00000068615.18	0	0
protein_coding	PLA2G4D	ENSG00000159337.6	0	0
protein_coding	SOX5	ENSG00000134532.16	0	0
protein_coding	REM2	ENSG00000139890.9	0	0
protein_coding	PANO1	ENSG00000274897.2	0	0
protein_coding	POLR2J3	ENSG00000168255.20	0	0
protein_coding	KLK5	ENSG00000167754.12	0	0
protein_coding	CD24	ENSG00000272398.5	0	0
protein_coding	CD86	ENSG00000114013.15	0	0
protein_coding	VIPR1	ENSG00000114812.12	0	0
protein_coding	CDH8	ENSG00000150394.13	0	0
protein_coding	IGSF3	ENSG00000143061.17	0	0
protein_coding	WNT10A	ENSG00000135925.8	0	0
protein_coding	HOXD10	ENSG00000128710.5	0	0
protein_coding	ZNF704	ENSG00000164684.13	0	0
protein_coding	CERS3	ENSG00000154227.13	0	0
protein_coding	FAM83F	ENSG00000133477.16	0	0
protein_coding	HEY2	ENSG00000135547.8	0	0
protein_coding	PLEKHA7	ENSG00000166689.15	0	0
protein_coding	C4orf47	ENSG00000205129.8	0	0
protein_coding	SPRR1B	ENSG00000169469.8	0	0
protein_coding	RORB	ENSG00000198963.10	0	0
protein_coding	SUN3	ENSG00000164744.12	0	0
protein_coding	RNF223	ENSG00000237330.2	0	0
protein_coding	PLPPR1	ENSG00000148123.14	0	0
protein_coding	RASSF10	ENSG00000189431.7	0	0
protein_coding	HAP1	ENSG00000173805.15	0	0
protein_coding	NTF4	ENSG00000225950.8	0	0
protein_coding	STX19	ENSG00000178750.2	0	0
protein_coding	FAM160A1	ENSG00000164142.15	0	0
protein_coding	CAMSAP3	ENSG00000076826.9	0	0
protein_coding	DQX1	ENSG00000144045.13	0	0
protein_coding	NOXA1	ENSG00000188747.8	0	0
protein_coding	CFHR3	ENSG00000116785.13	0	0
protein_coding	SPERT	ENSG00000174015.9	0	0
protein_coding	CYP2J2	ENSG00000134716.10	0	0
protein_coding	CASC1	ENSG00000118307.18	0	0
protein_coding	RLN1	ENSG00000107018.7	0	0

protein_coding	RNF128	ENSG00000133135.13	0	0
protein_coding	HOXD8	ENSG00000175879.8	0	0
protein_coding	STRA6	ENSG00000137868.18	0	0
protein_coding	VANGL2	ENSG00000162738.5	0	0
protein_coding	FAT2	ENSG00000086570.12	0	0
protein_coding	ANXA8L1	ENSG00000264230.8	0	0
protein_coding	DUSP9	ENSG00000130829.17	0	0
protein_coding	CGN	ENSG00000143375.14	0	0
protein_coding	PRB3	ENSG00000197870.12	0	0
protein_coding	CYLC1	ENSG00000183035.12	0	0
protein_coding	LHX5	ENSG00000089116.3	0	0
protein_coding	ESPN	ENSG00000187017.16	0	0
protein_coding	AADAC	ENSG00000114771.13	0	0
protein_coding	PCDH7	ENSG00000169851.15	0	0
protein_coding	RETREG1	ENSG00000154153.13	0	0
protein_coding	UGT2B7	ENSG00000171234.13	0	0
protein_coding	ADGRG6	ENSG00000112414.14	0	0
protein_coding	PLCG2	ENSG00000197943.9	0	0
protein_coding	CLIC3	ENSG00000169583.12	0	0
protein_coding	PRMT8	ENSG00000111218.11	0	0
protein_coding	FGFR3	ENSG00000068078.18	0	0
protein_coding	SPATA6L	ENSG00000106686.16	0	0
protein_coding	NMU	ENSG00000109255.11	0	0
protein_coding	PABPC4L	ENSG00000254535.3	0	0
protein_coding	LGR6	ENSG00000133067.17	0	0
protein_coding	CDKL2	ENSG00000138769.10	0	0
protein_coding	ALG1L	ENSG00000189366.9	0	0
protein_coding	DSG3	ENSG00000134757.4	0	0
protein_coding	NUPR2	ENSG00000185290.3	0	0
protein_coding	MYO5B	ENSG00000167306.19	0	0
protein_coding	DSC3	ENSG00000134762.16	0	0
protein_coding	ZNF711	ENSG00000147180.16	0	0
protein_coding	DUOX1	ENSG00000137857.17	0	0
protein_coding	GJB6	ENSG00000121742.17	0	0
protein_coding	CLTRN	ENSG00000147003.6	0	0
protein_coding	GFI1	ENSG00000162676.11	0	0
protein_coding	WFDC3	ENSG00000124116.18	0	0
protein_coding	DLX4	ENSG00000108813.10	0	0
protein_coding	PTPRR	ENSG00000153233.12	0	0
protein_coding	AQP10	ENSG00000143595.12	0	0
protein_coding	DISP3	ENSG00000204624.7	0	0
protein_coding	CD96	ENSG00000153283.12	0	0
protein_coding	TRIM58	ENSG00000162722.8	0	0
protein_coding	PCDH11X	ENSG00000102290.22	0	0

protein_coding	ABLIM2	ENSG00000163995.19	0	0
protein_coding	CAMK2A	ENSG00000070808.15	0	0
protein_coding	ODF3L1	ENSG00000182950.2	0	0
protein_coding	TAGLN3	ENSG00000144834.13	0	0
protein_coding	DUOX2	ENSG00000140279.12	0	0
protein_coding	HRASLS2	ENSG00000133328.3	0	0
protein_coding	CORO1A	ENSG00000102879.15	0	0
protein_coding	KCNH4	ENSG00000089558.8	0	0
protein_coding	MCF2L2	ENSG00000053524.12	0	0
protein_coding	FAM153A	ENSG00000170074.19	0	0
protein_coding	AC013470.2	ENSG00000226690.8	0	0
protein_coding	GDA	ENSG00000119125.16	0	0
protein_coding	KRT9	ENSG00000171403.9	0	0
protein_coding	ZFY	ENSG00000067646.11	0	0
protein_coding	DPPA2	ENSG00000163530.4	0	0
protein_coding	ASIC3	ENSG00000213199.7	0	0
protein_coding	SLITRK5	ENSG00000165300.7	0	0
protein_coding	PMFBP1	ENSG00000118557.15	0	0
protein_coding	PLIN4	ENSG00000167676.4	0	0
protein_coding	PLCXD3	ENSG00000182836.9	0	0
protein_coding	HIST1H4E	ENSG00000276966.2	0	0
protein_coding	CTNND2	ENSG00000169862.18	0	0
protein_coding	RAET1E	ENSG00000164520.11	0	0
protein_coding	TSPAN2	ENSG00000134198.9	0	0
protein_coding	NXPH3	ENSG00000182575.7	0	0
protein_coding	BMP7	ENSG00000101144.12	0	0
protein_coding	PRSS22	ENSG00000005001.9	0	0
protein_coding	DSC2	ENSG00000134755.15	0	0
protein_coding	ANOS1	ENSG00000011201.11	0	0
protein_coding	COL28A1	ENSG00000215018.9	0	0
protein_coding	TMPRSS13	ENSG00000137747.15	0	0
protein_coding	HOXC13	ENSG00000123364.4	0	0
protein_coding	ARL¬†14	ENSG00000179674.3	0	0
protein_coding	HOXB6	ENSG00000108511.9	0	0
protein_coding	NEFL	ENSG00000277586.2	0	0
protein_coding	GRIP1	ENSG00000155974.12	0	0
protein_coding	CASP14	ENSG00000105141.5	0	0
protein_coding	ARL14EPL	ENSG00000268223.5	0	0
protein_coding	CKMT1B	ENSG00000237289.9	0	0
protein_coding	PCYT1B	ENSG00000102230.13	0	0
protein_coding	ACTR3C	ENSG00000106526.10	0	0
protein_coding	FAAH2	ENSG00000165591.6	0	0
protein_coding	CCL5	ENSG00000271503.5	0	0
protein_coding	PGLYRP4	ENSG00000163218.14	0	0

protein_coding	DEFB1	ENSG00000164825.3	0	0
protein_coding	BCHE	ENSG00000114200.9	0	0
protein_coding	TPPP	ENSG00000171368.11	0	0
protein_coding	CCDC180	ENSG00000197816.13	0	0
protein_coding	MFNG	ENSG00000100060.17	0	0
protein_coding	MYH14	ENSG00000105357.16	0	0
protein_coding	NEBL	ENSG00000078114.18	0	0
protein_coding	SERPINB4	ENSG00000206073.10	0	0
protein_coding	TP53AIP1	ENSG00000120471.15	0	0
protein_coding	KIAA1211L	ENSG00000196872.11	0	0
protein_coding	GABRA3	ENSG00000011677.12	0	0
protein_coding	ROPN1L	ENSG00000145491.11	0	0
protein_coding	CREG2	ENSG00000175874.9	0	0
protein_coding	MUM1L1	ENSG00000157502.13	0	0
protein_coding	BST2	ENSG00000130303.12	0	0
protein_coding	MAP2	ENSG00000078018.19	0	0
protein_coding	ANO1	ENSG00000131620.17	0	0
protein_coding	TLL1	ENSG00000038295.7	0	0
protein_coding	SERPINB13	ENSG00000197641.11	0	0
protein_coding	AC015802.6	ENSG00000284526.1	0	0
protein_coding	KPNA7	ENSG00000185467.7	0	0
protein_coding	LY6D	ENSG00000167656.4	0	0
protein_coding	KLC3	ENSG00000104892.16	0	0
protein_coding	ZNF114	ENSG00000178150.9	0	0
protein_coding	CLDN16	ENSG00000113946.3	0	0
protein_coding	ELMOD1	ENSG00000110675.12	0	0
protein_coding	SERPINF2	ENSG00000167711.13	0	0
protein_coding	USH1G	ENSG00000182040.8	0	0
protein_coding	RNASE7	ENSG00000165799.4	0	0
protein_coding	ZNF467	ENSG00000181444.12	0	0
protein_coding	CD101	ENSG00000134256.12	0	0
protein_coding	CARD14	ENSG00000141527.17	0	0
protein_coding	EDA	ENSG00000158813.17	0	0
protein_coding	GCSAM	ENSG00000174500.12	0	0
protein_coding	ILDR1	ENSG00000145103.13	0	0
protein_coding	TUBAL3	ENSG00000178462.11	0	0
protein_coding	GCOM1	ENSG00000137878.17	0	0
protein_coding	MB	ENSG00000198125.12	0	0
protein_coding	PCP4L1	ENSG00000248485.1	0	0
protein_coding	ODAM	ENSG00000109205.16	0	0
protein_coding	CLPSL2	ENSG00000196748.9	0	0
protein_coding	AL354822.1	ENSG00000278384.1	0	0
protein_coding	SLAMF7	ENSG00000026751.16	0	0
protein_coding	CHRNB4	ENSG00000117971.11	0	0

protein_coding	TEX19	ENSG00000182459.4	0	0
protein_coding	PKD1L1	ENSG00000158683.8	0	0
protein_coding	TAS2R13	ENSG00000212128.2	0	0
protein_coding	VAV3	ENSG00000134215.15	0	0
protein_coding	OLR1	ENSG00000173391.8	0	0
protein_coding	EPPK1	ENSG00000261150.2	0	0
protein_coding	GBP6	ENSG00000183347.14	0	0
protein_coding	MPPED2	ENSG00000066382.16	0	0
protein_coding	TMEM125	ENSG00000179178.10	0	0
protein_coding	CCDC33	ENSG00000140481.14	0	0
protein_coding	PCDH10	ENSG00000138650.8	0	0
protein_coding	C1orf210	ENSG00000253313.5	0	0
protein_coding	CMPK2	ENSG00000134326.11	0	0
protein_coding	TMPRSS11E	ENSG00000087128.9	0	0
protein_coding	ALDH3B2	ENSG00000132746.14	0	0
protein_coding	HPGD	ENSG00000164120.13	0	0
protein_coding	KRT13	ENSG00000171401.14	0	0
protein_coding	TLL2	ENSG00000095587.8	0	0
protein_coding	OVOL1	ENSG00000172818.9	0	0
protein_coding	TMEM52B	ENSG00000165685.8	0	0
protein_coding	IRF5	ENSG00000128604.19	0	0
protein_coding	HIST1H2BH	ENSG00000275713.2	0	0
protein_coding	LMX1B	ENSG00000136944.17	0	0
protein_coding	DAPL1	ENSG00000163331.11	0	0
protein_coding	THSD7A	ENSG00000005108.15	0	0
protein_coding	FAM71E1	ENSG00000142530.10	0	0
protein_coding	MOGAT1	ENSG00000124003.12	0	0
protein_coding	RNF17	ENSG00000132972.18	0	0
protein_coding	ITM2A	ENSG00000078596.10	0	0
protein_coding	SLURP1	ENSG00000126233.1	0	0
protein_coding	WDR93	ENSG00000140527.14	0	0
protein_coding	RSPH1	ENSG00000160188.9	0	0
protein_coding	RIMS3	ENSG00000117016.9	0	0
protein_coding	AC068580.4	ENSG00000250644.3	0	0
protein_coding	WNT4	ENSG00000162552.14	0	0
protein_coding	AMDHD1	ENSG00000139344.7	0	0
protein_coding	FABP4	ENSG00000170323.8	0	0
protein_coding	ALOX5AP	ENSG00000132965.9	0	0
protein_coding	IQGAP2	ENSG00000145703.15	0	0
protein_coding	CASZ1	ENSG00000130940.14	0	0
protein_coding	AMTN	ENSG00000187689.9	0	0
protein_coding	HRK	ENSG00000135116.9	0	0
protein_coding	C1QTNF3	ENSG00000082196.20	0	0
protein_coding	SEC14L4	ENSG00000133488.14	0	0

protein_coding	PARD6A	ENSG00000102981.9	0	0
protein_coding	CLEC7A	ENSG00000172243.17	0	0
protein_coding	CYP39A1	ENSG00000146233.7	0	0
protein_coding	DUOXA1	ENSG00000140254.12	0	0
protein_coding	EGLN3	ENSG00000129521.13	0	0
protein_coding	DLX3	ENSG00000064195.7	0	0
protein_coding	KANK4	ENSG00000132854.18	0	0
protein_coding	CFI	ENSG00000205403.13	0	0
protein_coding	ICA1	ENSG00000003147.18	0	0
protein_coding	KRT16	ENSG00000186832.8	0	0
protein_coding	ZNF750	ENSG00000141579.6	0	0
protein_coding	FOXN1	ENSG00000109101.7	0	0
protein_coding	IQCM	ENSG00000234828.8	0	0
protein_coding	PROC	ENSG00000115718.17	0	0
protein_coding	GNAO1	ENSG00000087258.14	0	0
protein_coding	TRIML2	ENSG00000179046.8	0	0
protein_coding	KRT1	ENSG00000167768.4	0	0
protein_coding	BMP2	ENSG00000125845.6	0	0
protein_coding	SLC2A5	ENSG00000142583.17	0	0
protein_coding	PLEKHG4B	ENSG00000153404.14	0	0
protein_coding	POU2AF1	ENSG00000110777.11	0	0
protein_coding	FGD3	ENSG00000127084.18	0	0
protein_coding	MAFA	ENSG00000182759.3	0	0
protein_coding	DIO2	ENSG00000211448.11	0	0
protein_coding	TINCR	ENSG00000223573.7	0	0
protein_coding	SLC47A2	ENSG00000180638.17	0	0
protein_coding	WFDC5	ENSG00000175121.11	0	0
protein_coding	VTCN1	ENSG00000134258.16	0	0
protein_coding	LMO1	ENSG00000166407.13	0	0
protein_coding	NWD1	ENSG00000188039.14	0	0
protein_coding	PCDH15	ENSG00000150275.18	0	0
protein_coding	C10orf99	ENSG00000188373.4	0	0
protein_coding	SLC6A2	ENSG00000103546.18	0	0
protein_coding	TCHHL1	ENSG00000182898.3	0	0
protein_coding	RERG	ENSG00000134533.6	0	0
protein_coding	GPM6A	ENSG00000150625.16	0	0
protein_coding	CDH18	ENSG00000145526.11	0	0
protein_coding	SLC39A2	ENSG00000165794.9	0	0
protein_coding	KIAA1257	ENSG00000114656.11	0	0
protein_coding	POU2F3	ENSG00000137709.9	0	0
protein_coding	NCBP2L	ENSG00000170935.7	0	0
protein_coding	GSTA3	ENSG00000174156.14	0	0
protein_coding	OPRM1	ENSG00000112038.17	0	0
protein_coding	AC068896.1	ENSG00000258539.1	0	0

protein_coding	TENT5B	ENSG00000158246.7	0	0
protein_coding	ADD2	ENSG00000075340.22	0	0
protein_coding	GFI1B	ENSG00000165702.14	0	0
protein_coding	GPRIN3	ENSG00000185477.4	0	0
protein_coding	GABRG1	ENSG00000163285.7	0	0
protein_coding	ZFP57	ENSG00000204644.9	0	0
protein_coding	SMOC2	ENSG00000112562.18	0	0
protein_coding	LCE1C	ENSG00000197084.5	0	0
protein_coding	ERP27	ENSG00000139055.6	0	0
protein_coding	ARHGAP30	ENSG00000186517.13	0	0
protein_coding	OSTN	ENSG00000188729.6	0	0
protein_coding	VNN¬†2	ENSG00000112303.13	0	0
protein_coding	MMP20	ENSG00000137674.3	0	0
protein_coding	TEX14	ENSG00000121101.15	0	0
protein_coding	DSC1	ENSG00000134765.9	0	0
protein_coding	SATL1	ENSG00000184788.13	0	0
protein_coding	WNT3A	ENSG00000154342.5	0	0
protein_coding	PCDHB8	ENSG00000120322.3	0	0
protein_coding	SNAI3	ENSG00000185669.5	0	0
protein_coding	ANKRD34B	ENSG00000189127.7	0	0
protein_coding	HEPHL1	ENSG00000181333.11	0	0
protein_coding	EYS	ENSG00000188107.14	0	0
protein_coding	PRR29	ENSG00000224383.7	0	0
protein_coding	IQUB	ENSG00000164675.10	0	0
protein_coding	RARB	ENSG00000077092.18	0	0
protein_coding	POU5F1	ENSG00000204531.17	0	0
protein_coding	HOXA9	ENSG00000078399.17	0	0
protein_coding	CLEC1A	ENSG00000150048.10	0	0
protein_coding	FXYD3	ENSG00000089356.18	0	0
protein_coding	CYP2D6	ENSG00000100197.21	0	0
protein_coding	CXorf58	ENSG00000165182.11	0	0
protein_coding	ZMYND12	ENSG00000066185.12	0	0
protein_coding	AMY2B	ENSG00000240038.6	0	0
protein_coding	LAX1	ENSG00000122188.12	0	0
protein_coding	LRAT	ENSG00000121207.11	0	0
protein_coding	GABRG2	ENSG00000113327.16	0	0
protein_coding	TFEC	ENSG00000105967.15	0	0
protein_coding	DUSP13	ENSG00000079393.20	0	0
protein_coding	MOGAT2	ENSG00000166391.14	0	0
protein_coding	SCIMP	ENSG00000161929.14	0	0
protein_coding	ITGA2B	ENSG00000005961.18	0	0
protein_coding	SERPINB10	ENSG00000242550.5	0	0
protein_coding	FUT6	ENSG00000156413.13	0	0
protein_coding	FAT3	ENSG00000165323.15	0	0

protein_coding	LGALS9C	ENSG00000171916.16	0	0
protein_coding	IL17RC	ENSG00000163702.19	0	0
protein_coding	ADGRE1	ENSG00000174837.14	0	0
protein_coding	SPINK4	ENSG00000122711.8	0	0
protein_coding	BBOX1	ENSG00000129151.8	0	0
protein_coding	RPRM	ENSG00000177519.3	0	0
protein_coding	RBM20	ENSG00000203867.7	0	0
protein_coding	HPCAL4	ENSG00000116983.12	0	0
protein_coding	CXCR2	ENSG00000180871.7	0	0
protein_coding	PPP2R2B	ENSG00000156475.18	0	0
protein_coding	ADAM18	ENSG00000168619.15	0	0
protein_coding	ANXA9	ENSG00000143412.9	0	0
protein_coding	KCNN3	ENSG00000143603.18	0	0
protein_coding	GPR37L1	ENSG00000170075.8	0	0
protein_coding	FAM124B	ENSG00000124019.9	0	0
protein_coding	SLC34A1	ENSG00000131183.10	0	0
protein_coding	NKAIN2	ENSG00000188580.14	0	0
protein_coding	PACRG	ENSG00000112530.11	0	0
protein_coding	HEPN1	ENSG00000221932.6	0	0
protein_coding	ADAMTS20	ENSG00000173157.16	0	0
protein_coding	TJP3	ENSG00000105289.14	0	0
protein_coding	SYCP1	ENSG00000198765.11	0	0
protein_coding	GPR75	ENSG00000119737.5	0	0
protein_coding	FAM237A	ENSG00000235118.8	0	0
protein_coding	ASB18	ENSG00000182177.14	0	0
protein_coding	SLC9C1	ENSG00000172139.14	0	0
protein_coding	HTR3C	ENSG00000178084.1	0	0
protein_coding	DRD5	ENSG00000169676.5	0	0
protein_coding	TECRL	ENSG00000205678.7	0	0
protein_coding	HTN3	ENSG00000205649.7	0	0
protein_coding	ADH6	ENSG00000172955.17	0	0
protein_coding	POU4F3	ENSG00000091010.5	0	0
protein_coding	HLA-DQA2	ENSG00000237541.3	0	0
protein_coding	FABP12	ENSG00000197416.4	0	0
protein_coding	CCDC187	ENSG00000260220.6	0	0
protein_coding	CALHM3	ENSG00000183128.7	0	0
protein_coding	OR52I1	ENSG00000232268.6	0	0
protein_coding	HBB	ENSG00000244734.4	0	0
protein_coding	GRM5	ENSG00000168959.14	0	0
protein_coding	AICDA	ENSG00000111732.10	0	0
protein_coding	FAM186B	ENSG00000135436.8	0	0
protein_coding	KRT4	ENSG00000170477.12	0	0
protein_coding	HAL	ENSG00000084110.10	0	0
protein_coding	SYCP3	ENSG00000139351.14	0	0

protein_coding	FREM2	ENSG00000150893.10	0	0
protein_coding	ARL¬†11	ENSG00000152213.3	0	0
protein_coding	GOLGA6L7	ENSG00000261649.6	0	0
protein_coding	SKOR1	ENSG00000188779.10	0	0
protein_coding	SCNN1G	ENSG00000166828.2	0	0
protein_coding	OR3A2	ENSG00000221882.3	0	0
protein_coding	NAPA	ENSG00000105402.7	0	0
protein_coding	ZNF320	ENSG00000182986.13	0	0
protein_coding	CSTL1	ENSG00000125823.12	0	0
protein_coding	MTRNR2L3	ENSG00000256222.2	0	0
protein_coding	CDH26	ENSG00000124215.16	0	0
protein_coding	ARX	ENSG00000004848.7	0	0
protein_coding	TENT5D	ENSG00000174016.11	0	0
protein_coding	AGTR2	ENSG00000180772.6	0	0
protein_coding	CDH4	ENSG00000179242.15	0	0
protein_coding	SLC23A3	ENSG00000213901.10	0	0
protein_coding	ZC3HAV1L	ENSG00000146858.7	0	0
protein_coding	UCP3	ENSG00000175564.12	0	0
protein_coding	CDK5RAP3	ENSG00000108465.14	0	0
protein_coding	GOLGA8N	ENSG00000232653.8	0	0
protein_coding	ASGR1	ENSG00000141505.11	0	0
protein_coding	ANKHD1-EIF4EBP3	ENSG00000254996.5	0	0
protein_coding	FAM189A2	ENSG00000135063.19	0	0
protein_coding	DACH1	ENSG00000276644.4	0	0
protein_coding	IL12RB1	ENSG00000096996.15	0	0
protein_coding	CD8A	ENSG00000153563.15	0	0
protein_coding	TMED7-	ENSG00000251201.8	0	0
protein_coding	GRAP	ENSG00000154016.13	0	0
protein_coding	ONECUT3	ENSG00000205922.4	0	0
protein_coding	SOX18	ENSG00000203883.6	0	0
protein_coding	SPRR3	ENSG00000163209.14	0	0
protein_coding	ACADL	ENSG00000115361.7	0	0
protein_coding	ERBB4	ENSG00000178568.14	0	0
protein_coding	DNASE1L3	ENSG00000163687.13	0	0
protein_coding	CRISP3	ENSG00000096006.11	0	0
protein_coding	SLC18A2	ENSG00000165646.13	0	0
protein_coding	ACY3	ENSG00000132744.7	0	0
protein_coding	TSPAN19	ENSG00000231738.10	0	0
protein_coding	TGM5	ENSG00000104055.15	0	0
protein_coding	PSTPIP1	ENSG00000140368.12	0	0
protein_coding	CIITA	ENSG00000179583.19	0	0
protein_coding	LGALS9B	ENSG00000170298.15	0	0

protein_coding	XKRX	ENSG00000182489.8	0	0
protein_coding	PADI4	ENSG00000159339.13	0	0
protein_coding	HTR1D	ENSG00000179546.4	0	0
protein_coding	AHSG	ENSG00000145192.12	0	0
protein_coding	UGT2B15	ENSG00000196620.9	0	0
protein_coding	TREML1	ENSG00000161911.11	0	0
protein_coding	RAET1L	ENSG00000155918.7	0	0
protein_coding	KIF25	ENSG00000125337.17	0	0
protein_coding	HTRA4	ENSG00000169495.4	0	0
protein_coding	C9orf24	ENSG00000164972.12	0	0
protein_coding	TMEM236	ENSG00000148483.8	0	0
protein_coding	ATOH7	ENSG00000179774.8	0	0
protein_coding	SPOCK2	ENSG00000107742.12	0	0
protein_coding	KRT74	ENSG00000170484.9	0	0
protein_coding	NDUFA4L2	ENSG00000185633.10	0	0
protein_coding	RNF212B	ENSG00000215277.8	0	0
protein_coding	KCNK13	ENSG00000152315.4	0	0
protein_coding	CATSPERB	ENSG00000133962.7	0	0
protein_coding	ASPG	ENSG00000166183.15	0	0
protein_coding	DNAH17	ENSG00000187775.16	0	0
protein_coding	ZBTB7C	ENSG00000184828.9	0	0
protein_coding	HMSD	ENSG00000221887.5	0	0
protein_coding	IFI30	ENSG00000216490.3	0	0
protein_coding	TMEM255A	ENSG00000125355.15	0	0
protein_coding	C1orf127	ENSG00000175262.14	0	0
protein_coding	TSSK3	ENSG00000162526.6	0	0
protein_coding	DMBX1	ENSG00000197587.10	0	0
protein_coding	CYP4B1	ENSG00000142973.13	0	0
protein_coding	CYP4X1	ENSG00000186377.7	0	0
protein_coding	AGBL4	ENSG00000186094.16	0	0
protein_coding	MROH7	ENSG00000184313.19	0	0
protein_coding	IL12RB2	ENSG00000081985.11	0	0
protein_coding	CA14	ENSG00000118298.11	0	0
protein_coding	SYT2	ENSG00000143858.11	0	0
protein_coding	CHIT1	ENSG00000133063.15	0	0
protein_coding	REN	ENSG00000143839.14	0	0
protein_coding	LEMD1	ENSG00000186007.9	0	0
protein_coding	BECN2	ENSG00000196289.7	0	0
protein_coding	ADGRF3	ENSG00000173567.14	0	0
protein_coding	PCARE	ENSG00000179270.6	0	0
protein_coding	PLEK	ENSG00000115956.9	0	0
protein_coding	CLEC4F	ENSG00000152672.7	0	0
protein_coding	XIRP2	ENSG00000163092.19	0	0
protein_coding	UNC80	ENSG00000144406.18	0	0

protein_coding	CNTN4	ENSG00000144619.14	0	0
protein_coding	SLC6A1	ENSG00000157103.11	0	0
protein_coding	KCNH8	ENSG00000183960.8	0	0
protein_coding	ZNF620	ENSG00000177842.12	0	0
protein_coding	CADM2	ENSG00000175161.13	0	0
protein_coding	EPHA6	ENSG00000080224.17	0	0
protein_coding	ROPN1B	ENSG00000114547.9	0	0
protein_coding	PLSCR5	ENSG00000231213.6	0	0
protein_coding	AC104472.3	ENSG00000284952.1	0	0
protein_coding	LRRIQ4	ENSG00000188306.6	0	0
protein_coding	CRYGS	ENSG00000213139.7	0	0
protein_coding	MUC4	ENSG00000145113.21	0	0
protein_coding	SLC26A1	ENSG00000145217.13	0	0
protein_coding	CFAP99	ENSG00000206113.10	0	0
protein_coding	CRMP1	ENSG00000072832.14	0	0
protein_coding	KCNIP4	ENSG00000185774.15	0	0
protein_coding	KCTD8	ENSG00000183783.6	0	0
protein_coding	GABRA2	ENSG00000151834.15	0	0
protein_coding	SPINK2	ENSG00000128040.10	0	0
protein_coding	HHIP	ENSG00000164161.9	0	0
protein_coding	TKTL2	ENSG00000151005.4	0	0
protein_coding	TRIM60	ENSG00000176979.13	0	0
protein_coding	UGT3A2	ENSG00000168671.9	0	0
protein_coding	ADAMTS19	ENSG00000145808.9	0	0
protein_coding	PKD2L2	ENSG00000078795.16	0	0
protein_coding	GABRB2	ENSG00000145864.12	0	0
protein_coding	MBOAT1	ENSG00000172197.10	0	0
protein_coding	DCDC2	ENSG00000146038.11	0	0
protein_coding	HIST1H4B	ENSG00000278705.1	0	0
protein_coding	MPIG6B	ENSG00000204420.9	0	0
protein_coding	LY6G6C	ENSG00000204421.2	0	0
protein_coding	FGD2	ENSG00000146192.14	0	0
protein_coding	DNAH8	ENSG00000124721.17	0	0
protein_coding	GNMT	ENSG00000124713.5	0	0
protein_coding	TTBK1	ENSG00000146216.12	0	0
protein_coding	CLIC5	ENSG00000112782.16	0	0
protein_coding	IL17A	ENSG00000112115.6	0	0
protein_coding	KLHL31	ENSG00000124743.5	0	0
protein_coding	CGA	ENSG00000135346.8	0	0
protein_coding	CALHM4	ENSG00000164451.13	0	0
protein_coding	TRDN	ENSG00000186439.12	0	0
protein_coding	TAGAP	ENSG00000164691.17	0	0
protein_coding	SP8	ENSG00000164651.16	0	0
protein_coding	AOAH	ENSG00000136250.11	0	0

protein_coding	POU6F2	ENSG00000106536.19	0	0
protein_coding	CAMK2B	ENSG00000058404.19	0	0
protein_coding	COBL	ENSG00000106078.18	0	0
protein_coding	UPK3B	ENSG00000243566.6	0	0
protein_coding	CYP3A5	ENSG00000106258.14	0	0
protein_coding	SLC26A3	ENSG00000091138.12	0	0
protein_coding	TMEM213	ENSG00000214128.10	0	0
protein_coding	TMEM139	ENSG00000178826.10	0	0
protein_coding	SGCZ	ENSG00000185053.13	0	0
protein_coding	KCNU1	ENSG00000215262.7	0	0
protein_coding	RALYL	ENSG00000184672.11	0	0
protein_coding	DCAF4L2	ENSG00000176566.4	0	0
protein_coding	FAM135B	ENSG00000147724.11	0	0
protein_coding	GPR20	ENSG00000204882.3	0	0
protein_coding	HSD17B3	ENSG00000130948.10	0	0
protein_coding	CRB2	ENSG00000148204.11	0	0
protein_coding	AGAP4	ENSG00000188234.13	0	0
protein_coding	KCNIP2	ENSG00000120049.19	0	0
protein_coding	TRIM34	ENSG00000258659.6	0	0
protein_coding	OR2AG2	ENSG00000188124.3	0	0
protein_coding	MUC15	ENSG00000169550.13	0	0
protein_coding	OR4C11	ENSG00000172188.5	0	0
protein_coding	OR4C6	ENSG00000181903.5	0	0
protein_coding	OR9Q1	ENSG00000186509.4	0	0
protein_coding	CABP4	ENSG00000175544.13	0	0
protein_coding	CCDC83	ENSG00000150676.12	0	0
protein_coding	COLCA2	ENSG00000214290.8	0	0
protein_coding	C11orf52	ENSG00000149300.9	0	0
protein_coding	ANKK1	ENSG00000170209.4	0	0
protein_coding	FGF23	ENSG00000118972.2	0	0
protein_coding	ACRBP	ENSG00000111644.7	0	0
protein_coding	KLRC1	ENSG00000134545.13	0	0
protein_coding	TAS2R43	ENSG00000255374.3	0	0
protein_coding	PRPH	ENSG00000135406.13	0	0
protein_coding	WSCD2	ENSG00000075035.9	0	0
protein_coding	CFAP73	ENSG00000186710.11	0	0
protein_coding	NOS1	ENSG00000089250.18	0	0
protein_coding	DIABLO	ENSG00000184047.17	0	0
protein_coding	TMEM132D	ENSG00000151952.15	0	0
protein_coding	PDX1	ENSG00000139515.5	0	0
protein_coding	SOHLH2	ENSG00000120669.15	0	0
protein_coding	C13orf42	ENSG00000226792.7	0	0
protein_coding	OR4Q3	ENSG00000182652.3	0	0
protein_coding	RNASE6	ENSG00000169413.2	0	0

protein_coding	INSM2	ENSG00000168348.3	0	0
protein_coding	SLC24A4	ENSG00000140090.17	0	0
protein_coding	EXOC3L4	ENSG00000205436.7	0	0
protein_coding	TMEM179	ENSG00000258986.6	0	0
protein_coding	ACSBG1	ENSG00000103740.9	0	0
protein_coding	GOLGA6L10	ENSG00000278662.4	0	0
protein_coding	MTRNR2L4	ENSG00000232196.3	0	0
protein_coding	CLEC19A	ENSG00000261210.7	0	0
protein_coding	SCNN1B	ENSG00000168447.10	0	0
protein_coding	SULT1A2	ENSG00000197165.10	0	0
protein_coding	MT1B	ENSG00000169688.10	0	0
protein_coding	CNGB1	ENSG00000070729.13	0	0
protein_coding	BEAN1	ENSG00000166546.13	0	0
protein_coding	AC040162.1	ENSG00000261884.2	0	0
protein_coding	SMTNL2	ENSG00000188176.11	0	0
protein_coding	IKZF3	ENSG00000161405.16	0	0
protein_coding	LRRC37A	ENSG00000176681.14	0	0
protein_coding	TSPOAP1	ENSG00000005379.16	0	0
protein_coding	DNAI2	ENSG00000171595.13	0	0
protein_coding	SMIM6	ENSG00000259120.2	0	0
protein_coding	ST6GALNAC	ENSG00000070731.10	0	0
protein_coding	DSG1	ENSG00000134760.5	0	0
protein_coding	MADCAM1	ENSG00000099866.14	0	0
protein_coding	S1PR4	ENSG00000125910.5	0	0
protein_coding	ARHGEF18	ENSG00000104880.17	0	0
protein_coding	ELAVL3	ENSG00000196361.9	0	0
protein_coding	CYP4F8	ENSG00000186526.12	0	0
protein_coding	SLC5A5	ENSG00000105641.3	0	0
protein_coding	KCNN1	ENSG00000105642.15	0	0
protein_coding	PIK3R2	ENSG00000105647.16	0	0
protein_coding	SCGB2B2	ENSG00000205209.7	0	0
protein_coding	CCER2	ENSG00000262484.1	0	0
protein_coding	NKPD1	ENSG00000179846.9	0	0
protein_coding	ZNF534	ENSG00000198633.10	0	0
protein_coding	TTYH1	ENSG00000167614.13	0	0
protein_coding	NLRP9	ENSG00000185792.9	0	0
protein_coding	SAMSN1	ENSG00000155307.18	0	0
protein_coding	KRTAP19-1	ENSG00000184351.7	0	0
protein_coding	TMPRSS3	ENSG00000160183.14	0	0
protein_coding	ADA2	ENSG00000093072.16	0	0
protein_coding	TOP3B	ENSG00000100038.19	0	0
protein_coding	C22orf31	ENSG00000100249.4	0	0
protein_coding	EMID1	ENSG00000186998.15	0	0
protein_coding	IL2RB	ENSG00000100385.13	0	0

protein_coding	Z82206.1	ENSG00000225528.3	0	0
protein_coding	PRR5-ARHGAP8	ENSG00000248405.10	0	0
protein_coding	PKDREJ	ENSG00000130943.6	0	0
protein_coding	PAR	ENSG00000198223.16	0	0
protein_coding	ASB11	ENSG00000165192.13	0	0
protein_coding	ZNF157	ENSG00000147117.7	0	0
protein_coding	PFKFB1	ENSG00000158571.10	0	0
protein_coding	H2BFWT	ENSG00000123569.8	0	0
protein_coding	SPANXN3	ENSG00000189252.4	0	0
protein_coding	MAGEA10	ENSG00000124260.11	0	0
protein_coding	MAGEA3	ENSG00000221867.8	0	0
protein_coding	USP9Y	ENSG00000114374.12	0	0
protein_coding	DNAH3	ENSG00000158486.13	0	0
protein_coding	ARHGAP4	ENSG00000089820.15	0	0
protein_coding	BCO1	ENSG00000135697.9	0	0
protein_coding	FGF22	ENSG00000070388.11	0	0
protein_coding	HPCA	ENSG00000121905.9	0	0
protein_coding	GPR84	ENSG00000139572.3	0	0
protein_coding	TSHR	ENSG00000165409.17	0	0
protein_coding	FAM57B	ENSG00000149926.13	0	0
protein_coding	CHST6	ENSG00000183196.9	0	0
protein_coding	NOVA2	ENSG00000104967.6	0	0
protein_coding	CTCFL	ENSG00000124092.12	0	0
protein_coding	MGAT3	ENSG00000128268.11	0	0
protein_coding	TMPRSS5	ENSG00000166682.11	0	0
protein_coding	C2orf74	ENSG00000237651.6	0	0
protein_coding	LDB3	ENSG00000122367.19	0	0
protein_coding	MYO1F	ENSG00000142347.17	0	0
protein_coding	RBM44	ENSG00000177483.11	0	0
protein_coding	TFR2	ENSG00000106327.12	0	0
protein_coding	UGT2B11	ENSG00000213759.9	0	0
protein_coding	PDE8B	ENSG00000113231.13	0	0
protein_coding	GNA14	ENSG00000156049.6	0	0
protein_coding	C7orf61	ENSG00000185955.4	0	0
protein_coding	RAC3	ENSG00000169750.8	0	0
protein_coding	CXCL10	ENSG00000169245.5	0	0
protein_coding	DPP6	ENSG00000130226.16	0	0
protein_coding	KISS1R	ENSG00000116014.9	0	0
protein_coding	MYOM3	ENSG00000142661.18	0	0
protein_coding	LDLRAD1	ENSG00000203985.10	0	0
protein_coding	C1orf68	ENSG00000198854.5	0	0
protein_coding	EGR4	ENSG00000135625.7	0	0

protein_coding	HTN1	ENSG00000126550.8	0	0
protein_coding	GDF9	ENSG00000164404.8	0	0
protein_coding	MZB1	ENSG00000170476.15	0	0
protein_coding	PCDHGC3	ENSG00000240184.6	0	0
protein_coding	FGF20	ENSG00000078579.8	0	0
protein_coding	NPFFR1	ENSG00000148734.7	0	0
protein_coding	FXYD2	ENSG00000137731.13	0	0
protein_coding	PPM1E	ENSG00000175175.5	0	0
protein_coding	MAPK4	ENSG00000141639.11	0	0
protein_coding	ZNF66	ENSG00000160229.11	0	0
protein_coding	IGSF1	ENSG00000147255.18	0	0
protein_coding	CAPN8	ENSG00000203697.11	0	0
protein_coding	KRTCAP3	ENSG00000157992.12	0	0
protein_coding	AC073082.1	ENSG00000279956.1	0	0
protein_coding	TEX37	ENSG00000172073.3	0	0
protein_coding	AC092587.1	ENSG00000273155.1	0	0
protein_coding	CNTN6	ENSG00000134115.12	0	0
protein_coding	SLITRK3	ENSG00000121871.3	0	0
protein_coding	TLR1	ENSG00000174125.7	0	0
protein_coding	UGT2A1	ENSG00000173610.12	0	0
protein_coding	ODAPH	ENSG00000174792.10	0	0
protein_coding	FAM47E	ENSG00000189157.13	0	0
protein_coding	PCDHGB1	ENSG00000254221.2	0	0
protein_coding	INSYN2B	ENSG00000204767.3	0	0
protein_coding	ATP6V0A4	ENSG00000105929.16	0	0
protein_coding	FBP1	ENSG00000165140.10	0	0
protein_coding	RIC3	ENSG00000166405.14	0	0
protein_coding	TBX5	ENSG00000089225.19	0	0
protein_coding	RPL3L	ENSG00000140986.7	0	0
protein_coding	OR1F1	ENSG00000168124.2	0	0
protein_coding	CLUL1	ENSG00000079101.16	0	0
protein_coding	JSRP1	ENSG00000167476.10	0	0
protein_coding	GRIK1	ENSG00000171189.17	0	0
protein_coding	PNCK	ENSG00000130822.15	0	0
protein_coding	DRAXIN	ENSG00000162490.6	0	0
protein_coding	PRAMEF18	ENSG00000279804.2	0	0
protein_coding	FNDC5	ENSG00000160097.17	0	0
protein_coding	FYB2	ENSG00000187889.12	0	0
protein_coding	IL23R	ENSG00000162594.15	0	0
protein_coding	GIPC2	ENSG00000137960.5	0	0
protein_coding	GNAT2	ENSG00000134183.11	0	0
protein_coding	CD53	ENSG00000143119.13	0	0
protein_coding	SPRR1A	ENSG00000169474.4	0	0
protein_coding	ATP1A4	ENSG00000132681.16	0	0

protein_coding	FAM78B	ENSG00000188859.6	0	0
protein_coding	ZC3H11A	ENSG00000058673.16	0	0
protein_coding	MYT1L	ENSG00000186487.19	0	0
protein_coding	CTNNA2	ENSG00000066032.18	0	0
protein_coding	NEURL3	ENSG00000163121.9	0	0
protein_coding	ST6GAL2	ENSG00000144057.15	0	0
protein_coding	RGPD8	ENSG00000169629.11	0	0
protein_coding	RAB6C	ENSG00000222014.5	0	0
protein_coding	LRP1B	ENSG00000168702.17	0	0
protein_coding	HOXD3	ENSG00000128652.11	0	0
protein_coding	ZNF385B	ENSG00000144331.19	0	0
protein_coding	AC090004.1	ENSG00000268279.4	0	0
protein_coding	CCR9	ENSG00000173585.15	0	0
protein_coding	HYAL1	ENSG00000114378.16	0	0
protein_coding	HGD	ENSG00000113924.11	0	0
protein_coding	GRK7	ENSG00000114124.2	0	0
protein_coding	SERPINI2	ENSG00000114204.14	0	0
protein_coding	TMEM271	ENSG00000273238.2	0	0
protein_coding	PTTG2	ENSG00000250254.1	0	0
protein_coding	KLB	ENSG00000134962.6	0	0
protein_coding	UGT2B4	ENSG00000156096.13	0	0
protein_coding	FDCSP	ENSG00000181617.5	0	0
protein_coding	MTTP	ENSG00000138823.13	0	0
protein_coding	NPNT	ENSG00000168743.12	0	0
protein_coding	GALNTL6	ENSG00000174473.15	0	0
protein_coding	SPATA4	ENSG00000150628.6	0	0
protein_coding	PRDM9	ENSG00000164256.10	0	0
protein_coding	SHISAL2B	ENSG00000145642.11	0	0
protein_coding	FAM81B	ENSG00000153347.9	0	0
protein_coding	GABRP	ENSG00000094755.16	0	0
protein_coding	ZFP2	ENSG00000198939.7	0	0
protein_coding	HIST1H3I	ENSG00000275379.1	0	0
protein_coding	MEP1A	ENSG00000112818.9	0	0
protein_coding	KHDRBS2	ENSG00000112232.8	0	0
protein_coding	GJB7	ENSG00000164411.11	0	0
protein_coding	ZNF716	ENSG00000182111.8	0	0
protein_coding	MUC3A	ENSG00000169894.17	0	0
protein_coding	SLC26A5	ENSG00000170615.14	0	0
protein_coding	ASB15	ENSG00000146809.12	0	0
protein_coding	SVOPL	ENSG00000157703.15	0	0
protein_coding	WEE2	ENSG00000214102.7	0	0
protein_coding	FGF17	ENSG00000158815.10	0	0
protein_coding	DUSP26	ENSG00000133878.8	0	0
protein_coding	IDO1	ENSG00000131203.12	0	0

protein_coding	KCNQ3	ENSG00000184156.16	0	0
protein_coding	TYRP1	ENSG00000107165.12	0	0
protein_coding	FREM1	ENSG00000164946.19	0	0
protein_coding	HINT2	ENSG00000137133.10	0	0
protein_coding	SPAAR	ENSG00000235387.3	0	0
protein_coding	CBWD3	ENSG00000196873.15	0	0
protein_coding	LCN12	ENSG00000184925.11	0	0
protein_coding	DYDC2	ENSG00000133665.12	0	0
protein_coding	MUC2	ENSG00000198788.8	0	0
protein_coding	ASCL2	ENSG00000183734.4	0	0
protein_coding	HBG2	ENSG00000196565.14	0	0
protein_coding	C11orf94	ENSG00000234776.4	0	0
protein_coding	MS4A12	ENSG00000071203.9	0	0
protein_coding	LBHD1	ENSG00000162194.12	0	0
protein_coding	KCNK7	ENSG00000173338.12	0	0
protein_coding	SLN	ENSG00000170290.3	0	0
protein_coding	DRD2	ENSG00000149295.13	0	0
protein_coding	AC011604.2	ENSG00000257046.5	0	0
protein_coding	STAC3	ENSG00000185482.7	0	0
protein_coding	PAH	ENSG00000171759.9	0	0
protein_coding	C12orf42	ENSG00000179088.14	0	0
protein_coding	CCDC63	ENSG00000173093.12	0	0
protein_coding	CLDN10	ENSG00000134873.9	0	0
protein_coding	HS6ST3	ENSG00000185352.8	0	0
protein_coding	CCDC168	ENSG00000175820.3	0	0
protein_coding	COCH	ENSG00000100473.16	0	0
protein_coding	TTC6	ENSG00000139865.16	0	0
protein_coding	ZDHHC22	ENSG00000177108.5	0	0
protein_coding	DLL4	ENSG00000128917.7	0	0
protein_coding	ELL3	ENSG00000128886.11	0	0
protein_coding	KBTBD13	ENSG00000234438.4	0	0
protein_coding	UBE2Q2L	ENSG00000259511.2	0	0
protein_coding	ADAMTS17	ENSG00000140470.13	0	0
protein_coding	HS3ST6	ENSG00000162040.6	0	0
protein_coding	PAM16	ENSG00000217930.7	0	0
protein_coding	SEC14L5	ENSG00000103184.11	0	0
protein_coding	ANKS4B	ENSG00000175311.6	0	0
protein_coding	ZG16	ENSG00000174992.7	0	0
protein_coding	AC009119.2	ENSG00000260300.5	0	0
protein_coding	JPH3	ENSG00000154118.12	0	0
protein_coding	TMEM88	ENSG00000167874.6	0	0
protein_coding	AC011195.2	ENSG00000266826.2	0	0
protein_coding	AC113554.1	ENSG00000264813.6	0	0
protein_coding	CD300A	ENSG00000167851.14	0	0

protein_coding	HRH4	ENSG00000134489.6	0	0
protein_coding	ASXL3	ENSG00000141431.11	0	0
protein_coding	EBI3	ENSG00000105246.5	0	0
protein_coding	OR7C1	ENSG00000127530.3	0	0
protein_coding	KRTDAP	ENSG00000188508.10	0	0
protein_coding	CATSPERG	ENSG00000099338.22	0	0
protein_coding	IFNL1	ENSG00000182393.2	0	0
protein_coding	SELENOV	ENSG00000186838.13	0	0
protein_coding	ATP1A3	ENSG00000105409.18	0	0
protein_coding	CXCL17	ENSG00000189377.8	0	0
protein_coding	BHMG1	ENSG00000237452.2	0	0
protein_coding	AC011473.4	ENSG00000269741.5	0	0
protein_coding	PAX1	ENSG00000125813.13	0	0
protein_coding	CST8	ENSG00000125815.8	0	0
protein_coding	ARHGAP40	ENSG00000124143.10	0	0
protein_coding	FAM209A	ENSG00000124103.8	0	0
protein_coding	NKAIN4	ENSG00000101198.14	0	0
protein_coding	TFF1	ENSG00000160182.2	0	0
protein_coding	Sep-05	ENSG00000184702.19	0	0
protein_coding	DRICH1	ENSG00000189269.12	0	0
protein_coding	SGSM1	ENSG00000167037.18	0	0
protein_coding	RASL10A	ENSG00000100276.9	0	0
protein_coding	MPPED1	ENSG00000186732.13	0	0
protein_coding	IL1RAPL1	ENSG00000169306.9	0	0
protein_coding	TBX22	ENSG00000122145.14	0	0
protein_coding	OR4F5	ENSG00000186092.6	0	0
protein_coding	OR4F29	ENSG00000284733.1	0	0
protein_coding	OR4F16	ENSG00000284662.1	0	0
protein_coding	TTLL10	ENSG00000162571.13	0	0
protein_coding	TNFRSF4	ENSG00000186827.10	0	0
protein_coding	TMEM88B	ENSG00000205116.3	0	0
protein_coding	MMP23B	ENSG00000189409.13	0	0
protein_coding	CALML6	ENSG00000169885.9	0	0
protein_coding	CFAP74	ENSG00000142609.18	0	0
protein_coding	GABRD	ENSG00000187730.8	0	0
protein_coding	HES5	ENSG00000197921.5	0	0
protein_coding	ACTRT2	ENSG00000169717.6	0	0
protein_coding	AJAP1	ENSG00000196581.10	0	0
protein_coding	AL031847.2	ENSG00000285629.1	0	0
protein_coding	HES3	ENSG00000173673.7	0	0
protein_coding	TAS1R1	ENSG00000173662.20	0	0
protein_coding	UTS2	ENSG00000049247.13	0	0
protein_coding	TNFRSF9	ENSG00000049249.8	0	0
protein_coding	SLC2A7	ENSG00000197241.3	0	0

protein_coding	CENPS-	ENSG00000251503.8	0	0
protein_coding	CORT	ENSG00000241563.3	0	0
protein_coding	AL109811.4	ENSG00000277726.4	0	0
protein_coding	MASP2	ENSG00000009724.16	0	0
protein_coding	ANGPTL7	ENSG00000171819.4	0	0
protein_coding	NPPA	ENSG00000175206.10	0	0
protein_coding	NPPB	ENSG00000120937.8	0	0
protein_coding	TNFRSF8	ENSG00000120949.14	0	0
protein_coding	AADACL4	ENSG00000204518.2	0	0
protein_coding	AADACL3	ENSG00000188984.11	0	0
protein_coding	C1orf158	ENSG00000157330.9	0	0
protein_coding	PRAMEF12	ENSG00000116726.4	0	0
protein_coding	PRAMEF1	ENSG00000116721.9	0	0
protein_coding	PRAMEF11	ENSG00000239810.3	0	0
protein_coding	HNRNPCL1	ENSG00000179172.9	0	0
protein_coding	PRAMEF2	ENSG00000120952.4	0	0
protein_coding	PRAMEF4	ENSG00000243073.3	0	0
protein_coding	PRAMEF10	ENSG00000187545.5	0	0
protein_coding	PRAMEF7	ENSG00000204510.5	0	0
protein_coding	PRAMEF6	ENSG00000232423.6	0	0
protein_coding	PRAMEF27	ENSG00000274764.5	0	0
protein_coding	HNRNPCL3	ENSG00000277058.2	0	0
protein_coding	PRAMEF25	ENSG00000229571.7	0	0
protein_coding	HNRNPCL2	ENSG00000275774.2	0	0
protein_coding	PRAMEF26	ENSG00000280267.4	0	0
protein_coding	HNRNPCL4	ENSG00000179412.10	0	0
protein_coding	PRAMEF9	ENSG00000204505.4	0	0
protein_coding	PRAMEF13	ENSG00000279169.2	0	0
protein_coding	PRAMEF5	ENSG00000270601.4	0	0
protein_coding	PRAMEF8	ENSG00000182330.10	0	0
protein_coding	PRAMEF33	ENSG00000237700.2	0	0
protein_coding	PRAMEF15	ENSG00000204501.7	0	0
protein_coding	PRAMEF14	ENSG00000204481.7	0	0
protein_coding	PRAMEF19	ENSG00000204480.8	0	0
protein_coding	PRAMEF17	ENSG00000204479.4	0	0
protein_coding	PRAMEF20	ENSG00000204478.9	0	0
protein_coding	LRRC38	ENSG00000162494.5	0	0
protein_coding	CTRC	ENSG00000162438.11	0	0
protein_coding	CELA2A	ENSG00000142615.7	0	0
protein_coding	CELA2B	ENSG00000215704.9	0	0
protein_coding	RSC1A1	ENSG00000215695.1	0	0
protein_coding	TMEM82	ENSG00000162460.6	0	0
protein_coding	SRARP	ENSG00000183888.4	0	0
protein_coding	CLCNKA	ENSG00000186510.11	0	0

protein_coding	CLCNKB	ENSG00000184908.17	0	0
protein_coding	SPATA21	ENSG00000187144.11	0	0
protein_coding	PADI6	ENSG00000276747.1	0	0
protein_coding	ACTL8	ENSG00000117148.7	0	0
protein_coding	IGSF21	ENSG00000117154.11	0	0
protein_coding	KLHDC7A	ENSG00000179023.8	0	0
protein_coding	PAX7	ENSG00000009709.11	0	0
protein_coding	TAS1R2	ENSG00000179002.5	0	0
protein_coding	AL080251.1	ENSG00000255275.3	0	0
protein_coding	MINOS1-	ENSG00000270136.6	0	0
protein_coding	HTR6	ENSG00000158748.3	0	0
protein_coding	RNF186	ENSG00000178828.6	0	0
protein_coding	PLA2G2E	ENSG00000188784.4	0	0
protein_coding	PLA2G2A	ENSG00000188257.11	0	0
protein_coding	PLA2G2D	ENSG00000117215.14	0	0
protein_coding	PLA2G2F	ENSG00000158786.4	0	0
protein_coding	PLA2G2C	ENSG00000187980.6	0	0
protein_coding	UBXN10	ENSG00000162543.5	0	0
protein_coding	VWA5B1	ENSG00000158816.15	0	0
protein_coding	FAM43B	ENSG00000183114.7	0	0
protein_coding	LDLRAD2	ENSG00000187942.11	0	0
protein_coding	CELA3B	ENSG00000219073.7	0	0
protein_coding	CELA3A	ENSG00000142789.19	0	0
protein_coding	EPHA8	ENSG00000070886.11	0	0
protein_coding	C1QA	ENSG00000173372.16	0	0
protein_coding	C1QC	ENSG00000159189.11	0	0
protein_coding	C1QB	ENSG00000173369.15	0	0
protein_coding	LACTBL1	ENSG00000215906.8	0	0
protein_coding	TEX46	ENSG00000227868.5	0	0
protein_coding	CNR2	ENSG00000188822.7	0	0
protein_coding	NCMAP	ENSG00000184454.6	0	0
protein_coding	RHD	ENSG00000187010.20	0	0
protein_coding	AL020996.2	ENSG00000255054.3	0	0
protein_coding	SLC30A2	ENSG00000158014.14	0	0
protein_coding	TRIM63	ENSG00000158022.6	0	0
protein_coding	FAM110D	ENSG00000197245.5	0	0
protein_coding	C1orf232	ENSG00000282872.1	0	0
protein_coding	AL391650.1	ENSG00000236782.7	0	0
protein_coding	CATSPER4	ENSG00000188782.8	0	0
protein_coding	ZNF683	ENSG00000176083.17	0	0
protein_coding	LIN28A	ENSG00000131914.10	0	0
protein_coding	NR0B2	ENSG00000131910.4	0	0
protein_coding	FCN3	ENSG00000142748.12	0	0
protein_coding	CD164L2	ENSG00000174950.10	0	0

protein_coding	FGR	ENSG00000000938.12	0	0
protein_coding	OPRD1	ENSG00000116329.10	0	0
protein_coding	MATN1	ENSG00000162510.5	0	0
protein_coding	NKAIN1	ENSG00000084628.9	0	0
protein_coding	HCRTR1	ENSG00000121764.11	0	0
protein_coding	DCDC2B	ENSG00000222046.2	0	0
protein_coding	AL033529.1	ENSG00000254553.1	0	0
protein_coding	A3GALT2	ENSG00000184389.9	0	0
protein_coding	HMGB4	ENSG00000176256.10	0	0
protein_coding	C1orf94	ENSG00000142698.14	0	0
protein_coding	GJA4	ENSG00000187513.8	0	0
protein_coding	DLGAP3	ENSG00000116544.11	0	0
protein_coding	AC114490.2	ENSG00000271741.1	0	0
protein_coding	TMEM35B	ENSG00000243749.1	0	0
protein_coding	TEKT2	ENSG00000092850.11	0	0
protein_coding	CSF3R	ENSG00000119535.17	0	0
protein_coding	GRIK3	ENSG00000163873.9	0	0
protein_coding	EPHA10	ENSG00000183317.16	0	0
protein_coding	GJA9-	ENSG00000274944.4	0	0
protein_coding	GJA9	ENSG00000131233.9	0	0
protein_coding	NT5C1A	ENSG00000116981.3	0	0
protein_coding	TMCO2	ENSG00000188800.5	0	0
protein_coding	KCNQ4	ENSG00000117013.15	0	0
protein_coding	SLFNL1	ENSG00000171790.15	0	0
protein_coding	EDN2	ENSG00000127129.9	0	0
protein_coding	AC119676.1	ENSG00000284895.1	0	0
protein_coding	GUCA2B	ENSG00000044012.3	0	0
protein_coding	GUCA2A	ENSG00000197273.3	0	0
protein_coding	AC098484.3	ENSG00000283580.3	0	0
protein_coding	TIE1	ENSG00000066056.13	0	0
protein_coding	MPL	ENSG00000117400.17	0	0
protein_coding	AL451062.3	ENSG00000284989.1	0	0
protein_coding	KLF18	ENSG00000283039.1	0	0
protein_coding	TCTEX1D4	ENSG00000188396.3	0	0
protein_coding	HPDL	ENSG00000186603.5	0	0
protein_coding	CCDC17	ENSG00000159588.14	0	0
protein_coding	AL358075.4	ENSG00000278139.1	0	0
protein_coding	AL672043.1	ENSG00000250719.1	0	0
protein_coding	AL136373.1	ENSG00000282881.2	0	0
protein_coding	KNCN	ENSG00000162456.9	0	0
protein_coding	TEX38	ENSG00000186118.8	0	0
protein_coding	CYP4A11	ENSG00000187048.12	0	0
protein_coding	CYP4Z1	ENSG00000186160.4	0	0
protein_coding	CYP4A22	ENSG00000162365.11	0	0

protein_coding	PDZK1IP1	ENSG00000162366.7	0	0
protein_coding	TAL1	ENSG00000162367.11	0	0
protein_coding	SLC5A9	ENSG00000117834.12	0	0
protein_coding	BEND5	ENSG00000162373.12	0	0
protein_coding	C1orf185	ENSG00000204006.9	0	0
protein_coding	AL445685.3	ENSG00000285839.1	0	0
protein_coding	SLC1A7	ENSG00000162383.12	0	0
protein_coding	DMRTB1	ENSG00000143006.7	0	0
protein_coding	DIO1	ENSG00000211452.10	0	0
protein_coding	AL357673.1	ENSG00000256407.2	0	0
protein_coding	CDCP2	ENSG00000157211.11	0	0
protein_coding	FAM151A	ENSG00000162391.11	0	0
protein_coding	MROH7-	ENSG00000271723.5	0	0
protein_coding	LEXM	ENSG00000162398.11	0	0
protein_coding	BSND	ENSG00000162399.7	0	0
protein_coding	C8A	ENSG00000157131.10	0	0
protein_coding	C8B	ENSG00000021852.12	0	0
protein_coding	L1TD1	ENSG00000240563.1	0	0
protein_coding	ANGPTL3	ENSG00000132855.4	0	0
protein_coding	UBE2U	ENSG00000177414.13	0	0
protein_coding	C1orf141	ENSG00000203963.11	0	0
protein_coding	RPE65	ENSG00000116745.6	0	0
protein_coding	MSH4	ENSG00000057468.6	0	0
protein_coding	ASB17	ENSG00000154007.6	0	0
protein_coding	DNASE2B	ENSG00000137976.7	0	0
protein_coding	CLCA1	ENSG00000016490.15	0	0
protein_coding	AC093155.3	ENSG00000267561.2	0	0
protein_coding	AC093423.3	ENSG00000271949.1	0	0
protein_coding	BARHL2	ENSG00000143032.7	0	0
protein_coding	HFM1	ENSG00000162669.15	0	0
protein_coding	BRDT	ENSG00000137948.18	0	0
protein_coding	SETSIP	ENSG00000230667.5	0	0
protein_coding	C1orf146	ENSG00000203910.8	0	0
protein_coding	ABCA4	ENSG00000198691.12	0	0
protein_coding	GPR88	ENSG00000181656.6	0	0
protein_coding	OLFM3	ENSG00000118733.16	0	0
protein_coding	AMY2A	ENSG00000243480.7	0	0
protein_coding	AMY1A	ENSG00000237763.9	0	0
protein_coding	AMY1B	ENSG00000174876.16	0	0
protein_coding	AMY1C	ENSG00000187733.6	0	0
protein_coding	NBPF4	ENSG00000196427.13	0	0
protein_coding	NBPF6	ENSG00000186086.18	0	0
protein_coding	FNDC7	ENSG00000143107.8	0	0
protein_coding	AL449266.1	ENSG00000274068.1	0	0

protein_coding	C1orf194	ENSG00000179902.12	0	0
protein_coding	GPR61	ENSG00000156097.12	0	0
protein_coding	EPS8L3	ENSG00000198758.10	0	0
protein_coding	ALX3	ENSG00000156150.7	0	0
protein_coding	UBL4B	ENSG00000186150.4	0	0
protein_coding	PROK1	ENSG00000143125.5	0	0
protein_coding	KCNA10	ENSG00000143105.7	0	0
protein_coding	KCNA2	ENSG00000177301.14	0	0
protein_coding	CHI3L2	ENSG00000064886.13	0	0
protein_coding	CHIA	ENSG00000134216.18	0	0
protein_coding	TMIGD3	ENSG00000121933.18	0	0
protein_coding	ADORA3	ENSG00000282608.1	0	0
protein_coding	INKA2	ENSG00000197852.10	0	0
protein_coding	AL049557.1	ENSG00000284755.1	0	0
protein_coding	AL603832.3	ENSG00000271810.5	0	0
protein_coding	BCL2L15	ENSG00000188761.12	0	0
protein_coding	AMPD1	ENSG00000116748.21	0	0
protein_coding	TSHB	ENSG00000134200.3	0	0
protein_coding	CD2	ENSG00000116824.4	0	0
protein_coding	TBX15	ENSG00000092607.14	0	0
protein_coding	HAO2	ENSG00000116882.14	0	0
protein_coding	HSD3B2	ENSG00000203859.9	0	0
protein_coding	HSD3B1	ENSG00000203857.9	0	0
protein_coding	HMGCS2	ENSG00000134240.11	0	0
protein_coding	REG4	ENSG00000134193.14	0	0
protein_coding	ADAM30	ENSG00000134249.6	0	0
protein_coding	PPIAL4A	ENSG00000263353.3	0	0
protein_coding	FCGR1B	ENSG00000198019.12	0	0
protein_coding	FAM72B	ENSG00000188610.12	0	0
protein_coding	HIST2H3PS2	ENSG00000203818.7	0	0
protein_coding	PPIAL4E	ENSG00000271567.1	0	0
protein_coding	PPIAL4F	ENSG00000279782.1	0	0
protein_coding	PPIAL4D	ENSG00000256374.2	0	0
protein_coding	CD160	ENSG00000117281.15	0	0
protein_coding	AC243547.3	ENSG00000280778.1	0	0
protein_coding	HJV	ENSG00000168509.19	0	0
protein_coding	AC239799.1	ENSG00000213240.8	0	0
protein_coding	AC243756.1	ENSG00000270339.3	0	0
protein_coding	GJA5	ENSG00000265107.2	0	0
protein_coding	PPIAL4G	ENSG00000236334.2	0	0
protein_coding	NUDT4B	ENSG00000177144.7	0	0
protein_coding	PPIAL4C	ENSG00000263464.2	0	0
protein_coding	FCGR1A	ENSG00000150337.13	0	0
protein_coding	HIST2H3D	ENSG00000183598.3	0	0

protein_coding	HIST2H3C	ENSG00000203811.1	0	0
protein_coding	HIST2H2AA	ENSG00000203812.2	0	0
protein_coding	HIST2H2AA	ENSG00000272196.2	0	0
protein_coding	HIST2H3A	ENSG00000203852.3	0	0
protein_coding	HIST2H4B	ENSG00000270276.2	0	0
protein_coding	HIST2H2AB	ENSG00000184270.4	0	0
protein_coding	CTXND2	ENSG00000283324.1	0	0
protein_coding	TNFAIP8L2	ENSG00000163154.5	0	0
protein_coding	TMOD4	ENSG00000163157.14	0	0
protein_coding	CELF3	ENSG00000159409.14	0	0
protein_coding	LINGO4	ENSG00000213171.2	0	0
protein_coding	RORC	ENSG00000143365.17	0	0
protein_coding	THEM5	ENSG00000196407.11	0	0
protein_coding	RPTN	ENSG00000215853.3	0	0
protein_coding	HRNR	ENSG00000197915.5	0	0
protein_coding	FLG	ENSG00000143631.10	0	0
protein_coding	FLG2	ENSG00000143520.6	0	0
protein_coding	CRNN	ENSG00000143536.7	0	0
protein_coding	CRCT1	ENSG00000169509.5	0	0
protein_coding	LCE3E	ENSG00000185966.3	0	0
protein_coding	LCE3D	ENSG00000163202.4	0	0
protein_coding	LCE3C	ENSG00000244057.4	0	0
protein_coding	LCE3B	ENSG00000187238.5	0	0
protein_coding	LCE3A	ENSG00000185962.1	0	0
protein_coding	LCE2D	ENSG00000187223.3	0	0
protein_coding	LCE2C	ENSG00000187180.3	0	0
protein_coding	LCE2B	ENSG00000159455.8	0	0
protein_coding	LCE2A	ENSG00000187173.3	0	0
protein_coding	LCE4A	ENSG00000187170.4	0	0
protein_coding	KPRP	ENSG00000203786.6	0	0
protein_coding	LCE1F	ENSG00000240386.3	0	0
protein_coding	LCE1E	ENSG00000186226.8	0	0
protein_coding	LCE1D	ENSG00000172155.9	0	0
protein_coding	LCE1B	ENSG00000196734.8	0	0
protein_coding	LCE1A	ENSG00000186844.5	0	0
protein_coding	LCE6A	ENSG00000235942.2	0	0
protein_coding	AL162596.1	ENSG00000285946.1	0	0
protein_coding	SMCP	ENSG00000163206.5	0	0
protein_coding	SPRR5	ENSG00000283227.1	0	0
protein_coding	SPRR4	ENSG00000184148.3	0	0
protein_coding	SPRR2D	ENSG00000163216.6	0	0
protein_coding	SPRR2A	ENSG00000241794.1	0	0
protein_coding	SPRR2B	ENSG00000196805.7	0	0
protein_coding	SPRR2E	ENSG00000203785.8	0	0

protein_coding	SPRR2F	ENSG00000244094.1	0	0
protein_coding	SPRR2G	ENSG00000159516.8	0	0
protein_coding	LELP1	ENSG00000203784.2	0	0
protein_coding	PRR9	ENSG00000203783.4	0	0
protein_coding	LOR	ENSG00000203782.5	0	0
protein_coding	PGLYRP3	ENSG00000159527.3	0	0
protein_coding	S100A12	ENSG00000163221.8	0	0
protein_coding	S100A7A	ENSG00000184330.11	0	0
protein_coding	S100A7L2	ENSG00000197364.7	0	0
protein_coding	S100A7	ENSG00000143556.8	0	0
protein_coding	S100A1	ENSG00000160678.11	0	0
protein_coding	NPR¬†1	ENSG00000169418.9	0	0
protein_coding	SLC39A1	ENSG00000143570.17	0	0
protein_coding	AL358472.7	ENSG00000285779.1	0	0
protein_coding	AL358472.6	ENSG00000285641.1	0	0
protein_coding	C1orf189	ENSG00000163263.6	0	0
protein_coding	TDRD10	ENSG00000163239.12	0	0
protein_coding	CHRNB2	ENSG00000160716.5	0	0
protein_coding	LENEP	ENSG00000163352.5	0	0
protein_coding	DCST1	ENSG00000163357.10	0	0
protein_coding	AL691442.1	ENSG00000251246.1	0	0
protein_coding	AL713999.1	ENSG00000273088.1	0	0
protein_coding	MTX1	ENSG00000173171.14	0	0
protein_coding	PKLR	ENSG00000143627.19	0	0
protein_coding	RXFP4	ENSG00000173080.5	0	0
protein_coding	PMF1-	ENSG00000260238.6	0	0
protein_coding	BGLAP	ENSG00000242252.1	0	0
protein_coding	VHLL	ENSG00000189030.9	0	0
protein_coding	RHBG	ENSG00000132677.12	0	0
protein_coding	C1orf61	ENSG00000125462.16	0	0
protein_coding	TTC24	ENSG00000187862.11	0	0
protein_coding	HAPLN2	ENSG00000132702.12	0	0
protein_coding	INSRR	ENSG00000027644.4	0	0
protein_coding	LRRC71	ENSG00000160838.13	0	0
protein_coding	ETV3L	ENSG00000253831.1	0	0
protein_coding	FCRL5	ENSG00000143297.18	0	0
protein_coding	FCRL4	ENSG00000163518.10	0	0
protein_coding	FCRL3	ENSG00000160856.20	0	0
protein_coding	FCRL2	ENSG00000132704.15	0	0
protein_coding	FCRL1	ENSG00000163534.14	0	0
protein_coding	CD5L	ENSG00000073754.5	0	0
protein_coding	CD1A	ENSG00000158477.6	0	0
protein_coding	CD1C	ENSG00000158481.12	0	0
protein_coding	CD1B	ENSG00000158485.10	0	0

protein_coding	CD1E	ENSG00000158488.15	0	0
protein_coding	OR10T2	ENSG00000186306.1	0	0
protein_coding	OR10K1	ENSG00000173285.4	0	0
protein_coding	OR10R2	ENSG00000198965.4	0	0
protein_coding	OR6Y1	ENSG00000197532.2	0	0
protein_coding	OR6P1	ENSG00000186440.2	0	0
protein_coding	OR10X1	ENSG00000279111.2	0	0
protein_coding	OR10Z1	ENSG00000198967.4	0	0
protein_coding	SPTA1	ENSG00000163554.13	0	0
protein_coding	OR6K2	ENSG00000196171.3	0	0
protein_coding	OR6K3	ENSG00000203757.2	0	0
protein_coding	OR6N1	ENSG00000197403.4	0	0
protein_coding	OR6K6	ENSG00000180433.5	0	0
protein_coding	OR6N2	ENSG00000188340.2	0	0
protein_coding	MNDA	ENSG00000163563.7	0	0
protein_coding	CADM3	ENSG00000162706.12	0	0
protein_coding	ACKR1	ENSG00000213088.10	0	0
protein_coding	FCER1A	ENSG00000179639.10	0	0
protein_coding	OR10J5	ENSG00000184155.8	0	0
protein_coding	APCS	ENSG00000132703.3	0	0
protein_coding	CRP	ENSG00000132693.12	0	0
protein_coding	FCRL6	ENSG00000181036.13	0	0
protein_coding	SNHG28	ENSG00000256029.6	0	0
protein_coding	KCNJ10	ENSG00000177807.9	0	0
protein_coding	KCNJ9	ENSG00000162728.4	0	0
protein_coding	ATP1A2	ENSG00000018625.14	0	0
protein_coding	CASQ1	ENSG00000143318.12	0	0
protein_coding	AL139011.2	ENSG00000258465.7	0	0
protein_coding	NHLH1	ENSG00000171786.5	0	0
protein_coding	SLAMF6	ENSG00000162739.13	0	0
protein_coding	CD84	ENSG00000066294.14	0	0
protein_coding	SLAMF1	ENSG00000117090.14	0	0
protein_coding	CD48	ENSG00000117091.9	0	0
protein_coding	LY9	ENSG00000122224.17	0	0
protein_coding	CD244	ENSG00000122223.12	0	0
protein_coding	ITLN1	ENSG00000179914.4	0	0
protein_coding	ITLN2	ENSG00000158764.6	0	0
protein_coding	AL591806.3	ENSG00000270149.5	0	0
protein_coding	APOA2	ENSG00000158874.11	0	0
protein_coding	CFAP126	ENSG00000188931.3	0	0
protein_coding	FCGR3A	ENSG00000203747.10	0	0
protein_coding	FCGR3B	ENSG00000162747.10	0	0
protein_coding	FCGR2B	ENSG00000072694.20	0	0
protein_coding	AL512785.2	ENSG00000254706.2	0	0

protein_coding	SPATA46	ENSG00000171722.12	0	0
protein_coding	SH2D1B	ENSG00000198574.5	0	0
protein_coding	LMX1A	ENSG00000162761.14	0	0
protein_coding	RXRG	ENSG00000143171.12	0	0
protein_coding	LRRC52	ENSG00000162763.3	0	0
protein_coding	MAEL	ENSG00000143194.12	0	0
protein_coding	GPA33	ENSG00000143167.11	0	0
protein_coding	ADCY10	ENSG00000143199.17	0	0
protein_coding	XCL2	ENSG00000143185.3	0	0
protein_coding	CCDC181	ENSG00000117477.12	0	0
protein_coding	F5	ENSG00000198734.10	0	0
protein_coding	SELP	ENSG00000174175.16	0	0
protein_coding	SELE	ENSG00000007908.15	0	0
protein_coding	METTL11B	ENSG00000203740.3	0	0
protein_coding	FMO2	ENSG00000094963.13	0	0
protein_coding	C1orf105	ENSG00000180999.10	0	0
protein_coding	FASLG	ENSG00000117560.7	0	0
protein_coding	SLC9C2	ENSG00000162753.12	0	0
protein_coding	AL139142.2	ENSG00000285777.1	0	0
protein_coding	ANKRD45	ENSG00000183831.6	0	0
protein_coding	TEX50	ENSG00000232113.2	0	0
protein_coding	GPR52	ENSG00000203737.3	0	0
protein_coding	TNN	ENSG00000120332.15	0	0
protein_coding	TNR	ENSG00000116147.16	0	0
protein_coding	BRINP2	ENSG00000198797.6	0	0
protein_coding	SEC16B	ENSG00000120341.18	0	0
protein_coding	CLEC20A	ENSG00000188585.9	0	0
protein_coding	TEX35	ENSG00000240021.9	0	0
protein_coding	AXDND1	ENSG00000162779.21	0	0
protein_coding	NPHS2	ENSG00000116218.12	0	0
protein_coding	TDRD5	ENSG00000162782.15	0	0
protein_coding	FAM163A	ENSG00000143340.6	0	0
protein_coding	CACNA1E	ENSG00000198216.11	0	0
protein_coding	ZNF648	ENSG00000179930.5	0	0
protein_coding	TEDDM1	ENSG00000203730.2	0	0
protein_coding	RGSL1	ENSG00000121446.19	0	0
protein_coding	SHCBP1L	ENSG00000157060.15	0	0
protein_coding	APOBEC4	ENSG00000173627.7	0	0
protein_coding	PRG4	ENSG00000116690.12	0	0
protein_coding	BRINP3	ENSG00000162670.10	0	0
protein_coding	RGS21	ENSG00000253148.1	0	0
protein_coding	RGS13	ENSG00000127074.14	0	0
protein_coding	CFHR1	ENSG00000244414.6	0	0
protein_coding	CFHR2	ENSG00000080910.12	0	0

protein_coding	CFHR5	ENSG00000134389.9	0	0
protein_coding	F13B	ENSG00000143278.4	0	0
protein_coding	GPR25	ENSG00000170128.4	0	0
protein_coding	CACNA1S	ENSG00000081248.10	0	0
protein_coding	ASCL5	ENSG00000232237.3	0	0
protein_coding	IGFN1	ENSG00000163395.16	0	0
protein_coding	TNNT2	ENSG00000118194.18	0	0
protein_coding	TNNI1	ENSG00000159173.18	0	0
protein_coding	PTPN7	ENSG00000143851.15	0	0
protein_coding	MYOG	ENSG00000122180.4	0	0
protein_coding	OPTC	ENSG00000188770.9	0	0
protein_coding	ZBED6	ENSG00000257315.2	0	0
protein_coding	GOLT1A	ENSG00000174567.7	0	0
protein_coding	LRRN2	ENSG00000170382.11	0	0
protein_coding	KLHDC8A	ENSG00000162873.14	0	0
protein_coding	MFSD4A	ENSG00000174514.12	0	0
protein_coding	PM20D1	ENSG00000162877.12	0	0
protein_coding	SLC26A9	ENSG00000174502.18	0	0
protein_coding	CTSE	ENSG00000196188.10	0	0
protein_coding	RHEX	ENSG00000263961.7	0	0
protein_coding	AVPR1B	ENSG00000198049.6	0	0
protein_coding	IL10	ENSG00000136634.6	0	0
protein_coding	IL19	ENSG00000142224.15	0	0
protein_coding	PIGR	ENSG00000162896.5	0	0
protein_coding	FCAMR	ENSG00000162897.14	0	0
protein_coding	C4BPB	ENSG00000123843.12	0	0
protein_coding	C4BPA	ENSG00000123838.10	0	0
protein_coding	CR2	ENSG00000117322.17	0	0
protein_coding	CR1	ENSG00000203710.11	0	0
protein_coding	CR1L	ENSG00000197721.16	0	0
protein_coding	CAMK1G	ENSG00000008118.9	0	0
protein_coding	TRAF3IP3	ENSG00000009790.14	0	0
protein_coding	KCNH1	ENSG00000143473.12	0	0
protein_coding	AL590132.1	ENSG00000284299.1	0	0
protein_coding	AC092017.3	ENSG00000283952.1	0	0
protein_coding	RD3	ENSG00000198570.5	0	0
protein_coding	FAM71A	ENSG00000162771.7	0	0
protein_coding	USH2A	ENSG00000042781.12	0	0
protein_coding	ESRRG	ENSG00000196482.16	0	0
protein_coding	SLC30A10	ENSG00000196660.10	0	0
protein_coding	CCDC185	ENSG00000178395.6	0	0
protein_coding	LEFTY1	ENSG00000243709.1	0	0
protein_coding	AL117348.2	ENSG00000255835.1	0	0
protein_coding	LEFTY2	ENSG00000143768.12	0	0

protein_coding	STUM	ENSG00000203685.9	0	0
protein_coding	PRSS38	ENSG00000185888.5	0	0
protein_coding	TRIM17	ENSG00000162931.11	0	0
protein_coding	HIST3H3	ENSG00000168148.3	0	0
protein_coding	SPHAR	ENSG00000213029.3	0	0
protein_coding	ACTA1	ENSG00000143632.14	0	0
protein_coding	CAPN9	ENSG00000135773.12	0	0
protein_coding	TRIM67	ENSG00000119283.15	0	0
protein_coding	TSNAX-	ENSG00000270106.6	0	0
protein_coding	ACTN2	ENSG00000077522.12	0	0
protein_coding	MT1HL1	ENSG00000244020.2	0	0
protein_coding	ZP4	ENSG00000116996.9	0	0
protein_coding	MTRNR2L11	ENSG00000270188.1	0	0
protein_coding	WDR64	ENSG00000162843.17	0	0
protein_coding	MAP1LC3C	ENSG00000197769.5	0	0
protein_coding	PLD5	ENSG00000180287.16	0	0
protein_coding	C1orf100	ENSG00000173728.10	0	0
protein_coding	AL451007.3	ENSG00000284188.2	0	0
protein_coding	ZNF670-ZNF695	ENSG00000135747.11	0	0
protein_coding	NLRP3	ENSG00000162711.17	0	0
protein_coding	OR2B11	ENSG00000177535.8	0	0
protein_coding	GCSAML	ENSG00000169224.12	0	0
protein_coding	GCSAML-	ENSG00000284824.1	0	0
protein_coding	OR2G2	ENSG00000177489.1	0	0
protein_coding	OR2G3	ENSG00000177476.3	0	0
protein_coding	OR13G1	ENSG00000197437.4	0	0
protein_coding	OR6F1	ENSG00000169214.4	0	0
protein_coding	AC118470.1	ENSG00000239395.2	0	0
protein_coding	OR14A2	ENSG00000241128.2	0	0
protein_coding	OR14K1	ENSG00000153230.4	0	0
protein_coding	OR1C1	ENSG00000221888.4	0	0
protein_coding	OR14A16	ENSG00000196772.4	0	0
protein_coding	OR11L1	ENSG00000197591.3	0	0
protein_coding	OR2W3	ENSG00000238243.3	0	0
protein_coding	OR2T8	ENSG00000177462.7	0	0
protein_coding	OR2AJ1	ENSG00000177275.4	0	0
protein_coding	OR2L8	ENSG00000279263.1	0	0
protein_coding	OR2AK2	ENSG00000187080.9	0	0
protein_coding	OR2L5	ENSG00000197454.2	0	0
protein_coding	OR2L2	ENSG00000203663.4	0	0
protein_coding	OR2L3	ENSG00000198128.4	0	0
protein_coding	OR2L13	ENSG00000196071.5	0	0

protein_coding	OR2M5	ENSG00000162727.3	0	0
protein_coding	OR2M2	ENSG00000198601.3	0	0
protein_coding	OR2M3	ENSG00000228198.3	0	0
protein_coding	OR2M4	ENSG00000171180.2	0	0
protein_coding	OR2T33	ENSG00000177212.4	0	0
protein_coding	OR2T12	ENSG00000177201.2	0	0
protein_coding	OR2M7	ENSG00000177186.3	0	0
protein_coding	OR14C36	ENSG00000177174.1	0	0
protein_coding	OR2T4	ENSG00000196944.4	0	0
protein_coding	OR2T6	ENSG00000198104.3	0	0
protein_coding	OR2T1	ENSG00000175143.4	0	0
protein_coding	OR2T2	ENSG00000196240.4	0	0
protein_coding	OR2T3	ENSG00000196539.3	0	0
protein_coding	OR2T5	ENSG00000203661.4	0	0
protein_coding	OR2G6	ENSG00000188558.6	0	0
protein_coding	OR2T29	ENSG00000182783.5	0	0
protein_coding	OR2T34	ENSG00000183310.3	0	0
protein_coding	OR2T10	ENSG00000184022.4	0	0
protein_coding	OR2T11	ENSG00000279301.4	0	0
protein_coding	OR2T35	ENSG00000177151.4	0	0
protein_coding	OR2T27	ENSG00000187701.4	0	0
protein_coding	OR14I1	ENSG00000189181.4	0	0
protein_coding	LYPD8	ENSG00000259823.5	0	0
protein_coding	ALKAL2	ENSG00000189292.15	0	0
protein_coding	SNTG2	ENSG00000172554.11	0	0
protein_coding	TPO	ENSG00000115705.21	0	0
protein_coding	AC108488.2	ENSG00000255767.1	0	0
protein_coding	ALLC	ENSG00000151360.9	0	0
protein_coding	DCDC2C	ENSG00000214866.8	0	0
protein_coding	SOX11	ENSG00000176887.6	0	0
protein_coding	C2orf50	ENSG00000150873.11	0	0
protein_coding	NTSR2	ENSG00000169006.6	0	0
protein_coding	MYCN	ENSG00000134323.11	0	0
protein_coding	MSGN1	ENSG00000151379.3	0	0
protein_coding	NT5C1B-	ENSG00000250741.6	0	0
protein_coding	NT5C1B	ENSG00000185013.16	0	0
protein_coding	APOB	ENSG00000084674.14	0	0
protein_coding	TDRD15	ENSG00000218819.4	0	0
protein_coding	AC008073.3	ENSG00000276087.2	0	0
protein_coding	DRC1	ENSG00000157856.11	0	0
protein_coding	OTOF	ENSG00000115155.17	0	0
protein_coding	CIB4	ENSG00000157884.10	0	0
protein_coding	DPYSL5	ENSG00000157851.16	0	0
protein_coding	ABHD1	ENSG00000143994.13	0	0

protein_coding	PRR30	ENSG00000186143.11	0	0
protein_coding	TCF23	ENSG00000163792.6	0	0
protein_coding	DNAJC5G	ENSG00000163793.12	0	0
protein_coding	ALK	ENSG00000171094.17	0	0
protein_coding	CAPN13	ENSG00000162949.16	0	0
protein_coding	SRD5A2	ENSG00000277893.1	0	0
protein_coding	NLRC4	ENSG00000091106.18	0	0
protein_coding	SULT6B1	ENSG00000138068.10	0	0
protein_coding	KCNG3	ENSG00000171126.7	0	0
protein_coding	C1GALT1C1	ENSG00000223658.7	0	0
protein_coding	ABCG5	ENSG00000138075.12	0	0
protein_coding	ABCG8	ENSG00000143921.7	0	0
protein_coding	AC013717.1	ENSG00000285542.1	0	0
protein_coding	SLC3A1	ENSG00000138079.13	0	0
protein_coding	TMEM247	ENSG00000284701.1	0	0
protein_coding	STPG4	ENSG00000239605.10	0	0
protein_coding	AC073283.3	ENSG00000273269.3	0	0
protein_coding	LHCGR	ENSG00000138039.14	0	0
protein_coding	FSHR	ENSG00000170820.11	0	0
protein_coding	NRXN1	ENSG00000179915.22	0	0
protein_coding	GPR75-ASB3	ENSG00000270898.5	0	0
protein_coding	TSPYL6	ENSG00000178021.10	0	0
protein_coding	CCDC85A	ENSG00000055813.5	0	0
protein_coding	PROKR1	ENSG00000169618.6	0	0
protein_coding	BMP10	ENSG00000163217.1	0	0
protein_coding	FIGLA	ENSG00000183733.6	0	0
protein_coding	CD207	ENSG00000116031.8	0	0
protein_coding	ATP6V1B1	ENSG00000116039.12	0	0
protein_coding	EMX1	ENSG00000135638.13	0	0
protein_coding	NOTO	ENSG00000214513.3	0	0
protein_coding	NAT8	ENSG00000144035.3	0	0
protein_coding	C2orf78	ENSG00000187833.7	0	0
protein_coding	AC006030.1	ENSG00000264324.1	0	0
protein_coding	AC005041.1	ENSG00000159239.13	0	0
protein_coding	INO80B-	ENSG00000274049.4	0	0
protein_coding	TLX2	ENSG00000115297.10	0	0
protein_coding	M1AP	ENSG00000159374.17	0	0
protein_coding	LRRTM4	ENSG00000176204.13	0	0
protein_coding	REG3G	ENSG00000143954.12	0	0
protein_coding	REG1B	ENSG00000172023.7	0	0
protein_coding	REG3A	ENSG00000172016.15	0	0
protein_coding	LRRTM1	ENSG00000162951.10	0	0
protein_coding	RNF103-	ENSG00000249884.8	0	0
protein_coding	CD8B	ENSG00000172116.21	0	0

protein_coding	RGPD1	ENSG00000187627.15	0	0
protein_coding	PLGLB1	ENSG00000183281.14	0	0
protein_coding	PLGLB2	ENSG00000125551.18	0	0
protein_coding	RGPD2	ENSG00000185304.14	0	0
protein_coding	SMYD1	ENSG00000115593.14	0	0
protein_coding	FABP1	ENSG00000163586.9	0	0
protein_coding	FOXI3	ENSG00000214336.4	0	0
protein_coding	AL845331.2	ENSG00000259916.1	0	0
protein_coding	TEKT4	ENSG00000163060.7	0	0
protein_coding	MAL	ENSG00000172005.10	0	0
protein_coding	TRIM43B	ENSG00000144010.9	0	0
protein_coding	TRIM43	ENSG00000144015.4	0	0
protein_coding	ADRA2B	ENSG00000274286.1	0	0
protein_coding	ASTL	ENSG00000188886.3	0	0
protein_coding	DUSP2	ENSG00000158050.4	0	0
protein_coding	FER1L5	ENSG00000249715.11	0	0
protein_coding	ZAP70	ENSG00000115085.13	0	0
protein_coding	VWA3B	ENSG00000168658.18	0	0
protein_coding	CNGA3	ENSG00000144191.11	0	0
protein_coding	LYG2	ENSG00000185674.9	0	0
protein_coding	LONRF2	ENSG00000170500.12	0	0
protein_coding	NMS	ENSG00000204640.1	0	0
protein_coding	IL18RAP	ENSG00000115607.9	0	0
protein_coding	SLC9A4	ENSG00000180251.4	0	0
protein_coding	SLC9A2	ENSG00000115616.2	0	0
protein_coding	POU3F3	ENSG00000198914.3	0	0
protein_coding	GPR45	ENSG00000135973.2	0	0
protein_coding	C2orf40	ENSG00000119147.9	0	0
protein_coding	RGPD3	ENSG00000153165.18	0	0
protein_coding	CD8B2	ENSG00000254126.7	0	0
protein_coding	RGPD4	ENSG00000196862.9	0	0
protein_coding	SLC5A7	ENSG00000115665.8	0	0
protein_coding	SULT1C3	ENSG00000196228.3	0	0
protein_coding	SULT1C4	ENSG00000198075.9	0	0
protein_coding	RGPD5	ENSG00000015568.12	0	0
protein_coding	LIMS3	ENSG00000256977.12	0	0
protein_coding	LIMS3-LOC440895	ENSG00000284337.1	0	0
protein_coding	MALL	ENSG00000144063.3	0	0
protein_coding	AC112229.3	ENSG00000257207.5	0	0
protein_coding	LIMS4	ENSG00000256671.6	0	0
protein_coding	RGPD6	ENSG00000183054.11	0	0
protein_coding	IL37	ENSG00000125571.9	0	0

protein_coding	IL36A	ENSG00000136694.8	0	0
protein_coding	IL36B	ENSG00000136696.10	0	0
protein_coding	IL1F10	ENSG00000136697.12	0	0
protein_coding	FOXD4L1	ENSG00000184492.6	0	0
protein_coding	DPP10	ENSG00000175497.16	0	0
protein_coding	MARCO	ENSG00000019169.10	0	0
protein_coding	C1QL2	ENSG00000144119.3	0	0
protein_coding	SCTR	ENSG00000080293.9	0	0
protein_coding	CFAP221	ENSG00000163075.12	0	0
protein_coding	CNTNAP5	ENSG00000155052.14	0	0
protein_coding	TEX51	ENSG00000237524.9	0	0
protein_coding	MYO7B	ENSG00000169994.18	0	0
protein_coding	GPR17	ENSG00000144230.16	0	0
protein_coding	POTEF	ENSG00000196604.12	0	0
protein_coding	TUBA3E	ENSG00000152086.8	0	0
protein_coding	POTEI	ENSG00000196834.11	0	0
protein_coding	CFC1B	ENSG00000152093.7	0	0
protein_coding	CFC1	ENSG00000136698.8	0	0
protein_coding	POTEJ	ENSG00000222038.3	0	0
protein_coding	GPR148	ENSG00000173302.5	0	0
protein_coding	AMER3	ENSG00000178171.10	0	0
protein_coding	AC009477.2	ENSG00000284479.1	0	0
protein_coding	POTEE	ENSG00000188219.14	0	0
protein_coding	RAB6D	ENSG00000233087.7	0	0
protein_coding	TUBA3D	ENSG00000075886.10	0	0
protein_coding	ANKRD30BL	ENSG00000163046.15	0	0
protein_coding	TMEM163	ENSG00000152128.13	0	0
protein_coding	ACMSD	ENSG00000153086.13	0	0
protein_coding	MAP3K19	ENSG00000176601.12	0	0
protein_coding	LCT	ENSG00000115850.9	0	0
protein_coding	THSD7B	ENSG00000144229.11	0	0
protein_coding	NXPH2	ENSG00000144227.4	0	0
protein_coding	ARHGAP15	ENSG00000075884.13	0	0
protein_coding	NEB	ENSG00000183091.19	0	0
protein_coding	AC068547.1	ENSG00000283228.1	0	0
protein_coding	KCNJ3	ENSG00000162989.4	0	0
protein_coding	UPP2	ENSG00000007001.12	0	0
protein_coding	LY75-CD302	ENSG00000248672.5	0	0
protein_coding	TBR1	ENSG00000136535.14	0	0
protein_coding	GCG	ENSG00000115263.14	0	0
protein_coding	KCNH7	ENSG00000184611.11	0	0
protein_coding	SLC38A11	ENSG00000169507.9	0	0
protein_coding	SCN1A	ENSG00000144285.19	0	0
protein_coding	SCN7A	ENSG00000136546.14	0	0

protein_coding	NOSTRIN	ENSG00000163072.15	0	0
protein_coding	G6PC2	ENSG00000152254.10	0	0
protein_coding	LRP2	ENSG00000081479.13	0	0
protein_coding	AC093899.2	ENSG00000251569.1	0	0
protein_coding	EVX2	ENSG00000174279.4	0	0
protein_coding	HOXD12	ENSG00000170178.6	0	0
protein_coding	AC009336.2	ENSG00000278500.1	0	0
protein_coding	HOXD4	ENSG00000170166.5	0	0
protein_coding	PDE11A	ENSG00000284741.1	0	0
protein_coding	TTN	ENSG00000155657.26	0	0
protein_coding	CCDC141	ENSG00000163492.15	0	0
protein_coding	CERKL	ENSG00000188452.13	0	0
protein_coding	NEUROD1	ENSG00000162992.3	0	0
protein_coding	FSIP2	ENSG00000188738.14	0	0
protein_coding	ZSWIM2	ENSG00000163012.3	0	0
protein_coding	C2orf66	ENSG00000187944.2	0	0
protein_coding	HSPE1-	ENSG00000270757.1	0	0
protein_coding	BOLL	ENSG00000152430.17	0	0
protein_coding	PLCL1	ENSG00000115896.15	0	0
protein_coding	CTLA4	ENSG00000163599.15	0	0
protein_coding	ICOS	ENSG00000163600.12	0	0
protein_coding	DYTN	ENSG00000232125.3	0	0
protein_coding	CPO	ENSG00000144410.4	0	0
protein_coding	CRYGD	ENSG00000118231.4	0	0
protein_coding	CRYGC	ENSG00000163254.4	0	0
protein_coding	CRYGB	ENSG00000182187.3	0	0
protein_coding	CRYGA	ENSG00000168582.4	0	0
protein_coding	C2orf80	ENSG00000188674.10	0	0
protein_coding	PTH2R	ENSG00000144407.9	0	0
protein_coding	MYL1	ENSG00000168530.15	0	0
protein_coding	VWC2L	ENSG00000174453.9	0	0
protein_coding	TNP1	ENSG00000118245.2	0	0
protein_coding	RUFY4	ENSG00000188282.12	0	0
protein_coding	CXCR1	ENSG00000163464.7	0	0
protein_coding	GPBAR1	ENSG00000179921.14	0	0
protein_coding	CATIP	ENSG00000158428.3	0	0
protein_coding	VIL1	ENSG00000127831.10	0	0
protein_coding	PRKAG3	ENSG00000115592.11	0	0
protein_coding	FEV	ENSG00000163497.2	0	0
protein_coding	CFAP65	ENSG00000181378.13	0	0
protein_coding	IHH	ENSG00000163501.6	0	0
protein_coding	NHEJ1	ENSG00000187736.12	0	0
protein_coding	ABCB6	ENSG00000115657.13	0	0
protein_coding	AC068946.2	ENSG00000284820.1	0	0

protein_coding	TUBA4B	ENSG00000243910.7	0	0
protein_coding	RESP18	ENSG00000182698.11	0	0
protein_coding	DES	ENSG00000175084.11	0	0
protein_coding	ASIC4	ENSG00000072182.12	0	0
protein_coding	PAX3	ENSG00000135903.19	0	0
protein_coding	CCDC140	ENSG00000163081.3	0	0
protein_coding	SGPP2	ENSG00000163082.9	0	0
protein_coding	CCDC195	ENSG00000283428.1	0	0
protein_coding	NYAP2	ENSG00000144460.12	0	0
protein_coding	SCYGR1	ENSG00000284629.1	0	0
protein_coding	SCYGR2	ENSG00000284643.1	0	0
protein_coding	C2orf83	ENSG00000042304.11	0	0
protein_coding	SCYGR3	ENSG00000284704.1	0	0
protein_coding	SCYGR4	ENSG00000284631.1	0	0
protein_coding	SCYGR5	ENSG00000284667.1	0	0
protein_coding	SCYGR6	ENSG00000284725.1	0	0
protein_coding	SCYGR7	ENSG00000284718.1	0	0
protein_coding	SCYGR8	ENSG00000284635.1	0	0
protein_coding	DAW1	ENSG00000123977.9	0	0
protein_coding	SPHKAP	ENSG00000153820.12	0	0
protein_coding	SP140	ENSG00000079263.18	0	0
protein_coding	GPR55	ENSG00000135898.9	0	0
protein_coding	SPATA3	ENSG00000173699.16	0	0
protein_coding	B3GNT7	ENSG00000156966.6	0	0
protein_coding	TEX44	ENSG00000177673.3	0	0
protein_coding	ALPG	ENSG00000163286.8	0	0
protein_coding	ALPI	ENSG00000163295.4	0	0
protein_coding	ECEL1	ENSG00000171551.11	0	0
protein_coding	PRSS56	ENSG00000237412.6	0	0
protein_coding	CHRND	ENSG00000135902.9	0	0
protein_coding	CHRNG	ENSG00000196811.11	0	0
protein_coding	KCNJ13	ENSG00000115474.6	0	0
protein_coding	NEU2	ENSG00000115488.3	0	0
protein_coding	SAG	ENSG00000130561.16	0	0
protein_coding	UGT1A8	ENSG00000242366.3	0	0
protein_coding	UGT1A10	ENSG00000242515.5	0	0
protein_coding	UGT1A9	ENSG00000241119.1	0	0
protein_coding	UGT1A5	ENSG00000240224.1	0	0
protein_coding	UGT1A4	ENSG00000244474.5	0	0
protein_coding	UGT1A3	ENSG00000243135.6	0	0
protein_coding	MROH2A	ENSG00000185038.14	0	0
protein_coding	PRLH	ENSG00000071677.1	0	0
protein_coding	UBE2F-SCLY	ENSG00000258984.5	0	0
protein_coding	OR6B2	ENSG00000182083.7	0	0

protein_coding	OR6B3	ENSG00000178586.5	0	0
protein_coding	OTOS	ENSG00000178602.7	0	0
protein_coding	AQP12B	ENSG00000185176.12	0	0
protein_coding	AQP12A	ENSG00000184945.13	0	0
protein_coding	AGXT	ENSG00000172482.4	0	0
protein_coding	MAB21L4	ENSG00000172478.17	0	0
protein_coding	CROCC2	ENSG00000226321.5	0	0
protein_coding	GAL3ST2	ENSG00000154252.11	0	0
protein_coding	NEU4	ENSG00000204099.11	0	0
protein_coding	PDCD1	ENSG00000188389.10	0	0
protein_coding	RTP5	ENSG00000188011.5	0	0
protein_coding	AC131097.2	ENSG00000216921.8	0	0
protein_coding	IL5RA	ENSG00000091181.19	0	0
protein_coding	LRRN1	ENSG00000175928.5	0	0
protein_coding	GRM7	ENSG00000196277.15	0	0
protein_coding	CAV3	ENSG00000182533.6	0	0
protein_coding	CPNE9	ENSG00000144550.12	0	0
protein_coding	ARPC4-	ENSG00000250151.9	0	0
protein_coding	CIDEC	ENSG00000187288.10	0	0
protein_coding	FANCD2OS	ENSG00000163705.12	0	0
protein_coding	AC022384.1	ENSG00000272410.5	0	0
protein_coding	GHRL	ENSG00000157017.15	0	0
protein_coding	ATP2B2	ENSG00000157087.18	0	0
protein_coding	SLC6A11	ENSG00000132164.9	0	0
protein_coding	C3orf20	ENSG00000131379.9	0	0
protein_coding	PP2D1	ENSG00000183977.13	0	0
protein_coding	GADL1	ENSG00000144644.14	0	0
protein_coding	TRIM71	ENSG00000206557.5	0	0
protein_coding	CCR4	ENSG00000183813.6	0	0
protein_coding	ARPP21	ENSG00000172995.16	0	0
protein_coding	DCLK3	ENSG00000163673.7	0	0
protein_coding	ITGA9	ENSG00000144668.11	0	0
protein_coding	DLEC1	ENSG00000008226.19	0	0
protein_coding	SLC22A13	ENSG00000172940.12	0	0
protein_coding	SCN10A	ENSG00000185313.7	0	0
protein_coding	XIRP1	ENSG00000168334.8	0	0
protein_coding	CCR8	ENSG00000179934.6	0	0
protein_coding	MYRIP	ENSG00000170011.13	0	0
protein_coding	LYZL4	ENSG00000157093.8	0	0
protein_coding	KLHL40	ENSG00000157119.11	0	0
protein_coding	HHATL	ENSG00000010282.14	0	0
protein_coding	AC006059.2	ENSG00000280571.2	0	0
protein_coding	AC092042.3	ENSG00000273291.5	0	0
protein_coding	CYP8B1	ENSG00000180432.5	0	0

protein_coding	TOPAZ1	ENSG00000173769.4	0	0
protein_coding	TGM4	ENSG00000163810.11	0	0
protein_coding	CXCR6	ENSG00000172215.5	0	0
protein_coding	CCR2	ENSG00000121807.5	0	0
protein_coding	CCR5	ENSG00000160791.13	0	0
protein_coding	LTF	ENSG00000012223.12	0	0
protein_coding	RTP3	ENSG00000163825.3	0	0
protein_coding	TDGF1	ENSG00000241186.9	0	0
protein_coding	AC104304.1	ENSG00000283877.1	0	0
protein_coding	FAM240A	ENSG00000283473.2	0	0
protein_coding	PRSS50	ENSG00000283706.1	0	0
protein_coding	PRSS50	ENSG00000206549.13	0	0
protein_coding	PRSS45	ENSG00000188086.13	0	0
protein_coding	AC109583.3	ENSG00000284672.1	0	0
protein_coding	PRSS42	ENSG00000178055.11	0	0
protein_coding	MYL3	ENSG00000160808.9	0	0
protein_coding	SPINK8	ENSG00000229453.2	0	0
protein_coding	FBXW12	ENSG00000164049.14	0	0
protein_coding	TREX1	ENSG00000213689.13	0	0
protein_coding	TMEM89	ENSG00000183396.3	0	0
protein_coding	C3orf84	ENSG00000236980.9	0	0
protein_coding	AC104452.1	ENSG00000283189.2	0	0
protein_coding	NICN1	ENSG00000145029.13	0	0
protein_coding	BSN	ENSG00000164061.4	0	0
protein_coding	AMIGO3	ENSG00000176020.8	0	0
protein_coding	CAMKV	ENSG00000164076.16	0	0
protein_coding	GNAT1	ENSG00000114349.9	0	0
protein_coding	LSMEM2	ENSG00000179564.3	0	0
protein_coding	Z84492.1	ENSG00000272104.1	0	0
protein_coding	GRM2	ENSG00000164082.14	0	0
protein_coding	IQCF6	ENSG00000214686.5	0	0
protein_coding	AC097636.1	ENSG00000285749.1	0	0
protein_coding	IQCF3	ENSG00000229972.8	0	0
protein_coding	IQCF2	ENSG00000184345.4	0	0
protein_coding	IQCF5	ENSG00000214681.3	0	0
protein_coding	IQCF1	ENSG00000173389.15	0	0
protein_coding	GPR62	ENSG00000180929.5	0	0
protein_coding	ABHD14A-ACY1	ENSG00000114786.16	0	0
protein_coding	TLR9	ENSG00000239732.3	0	0
protein_coding	AC097637.1	ENSG00000173366.11	0	0
protein_coding	TNNC1	ENSG00000114854.7	0	0
protein_coding	STAB1	ENSG00000010327.10	0	0

protein_coding	ITIH1	ENSG00000055957.10	0	0
protein_coding	ITIH3	ENSG00000162267.12	0	0
protein_coding	ITIH4	ENSG00000055955.15	0	0
protein_coding	AC006254.1	ENSG00000243696.4	0	0
protein_coding	MUSTN1	ENSG00000272573.5	0	0
protein_coding	TMEM110-MUSTN1	ENSG00000248592.7	0	0
protein_coding	AC096887.1	ENSG00000272305.5	0	0
protein_coding	LRTM1	ENSG00000144771.7	0	0
protein_coding	HESX1	ENSG00000163666.9	0	0
protein_coding	HTD2	ENSG00000255154.7	0	0
protein_coding	FAM3D	ENSG00000198643.6	0	0
protein_coding	FEZF2	ENSG00000153266.12	0	0
protein_coding	SYNPR	ENSG00000163630.10	0	0
protein_coding	SNTN	ENSG00000188817.7	0	0
protein_coding	C3orf49	ENSG00000163632.13	0	0
protein_coding	FAM19A1	ENSG00000183662.10	0	0
protein_coding	FAM19A4	ENSG00000163377.15	0	0
protein_coding	MDFIC2	ENSG00000242120.3	0	0
protein_coding	AC097634.4	ENSG00000285708.1	0	0
protein_coding	PROK2	ENSG00000163421.8	0	0
protein_coding	FRG2C	ENSG00000172969.7	0	0
protein_coding	POU1F1	ENSG00000064835.10	0	0
protein_coding	CSNKA2IP	ENSG00000283434.1	0	0
protein_coding	GABRR3	ENSG00000183185.9	0	0
protein_coding	OR5AC2	ENSG00000196578.4	0	0
protein_coding	OR5H1	ENSG00000231192.2	0	0
protein_coding	OR5H14	ENSG00000236032.3	0	0
protein_coding	OR5H15	ENSG00000233412.5	0	0
protein_coding	OR5H6	ENSG00000230301.6	0	0
protein_coding	OR5H2	ENSG00000197938.5	0	0
protein_coding	OR5K4	ENSG00000196098.2	0	0
protein_coding	OR5K3	ENSG00000206536.1	0	0
protein_coding	OR5K1	ENSG00000232382.2	0	0
protein_coding	OR5K2	ENSG00000231861.2	0	0
protein_coding	AC021660.3	ENSG00000285635.1	0	0
protein_coding	GPR15	ENSG00000154165.4	0	0
protein_coding	CCDC54	ENSG00000138483.2	0	0
protein_coding	HHLA2	ENSG00000114455.13	0	0
protein_coding	RETNLB	ENSG00000163515.6	0	0
protein_coding	TRAT1	ENSG00000163519.13	0	0
protein_coding	GUCA1C	ENSG00000138472.10	0	0
protein_coding	MORC1	ENSG00000114487.9	0	0

protein_coding	C3orf85	ENSG00000241224.7	0	0
protein_coding	DPPA4	ENSG00000121570.12	0	0
protein_coding	CD200	ENSG00000091972.18	0	0
protein_coding	CD200R1L	ENSG00000206531.10	0	0
protein_coding	CD200R1	ENSG00000163606.10	0	0
protein_coding	DRD3	ENSG00000151577.12	0	0
protein_coding	ZNF80	ENSG00000174255.6	0	0
protein_coding	TIGIT	ENSG00000181847.11	0	0
protein_coding	C3orf30	ENSG00000163424.8	0	0
protein_coding	AC083800.1	ENSG00000251012.2	0	0
protein_coding	CD80	ENSG00000121594.11	0	0
protein_coding	PLA1A	ENSG00000144837.8	0	0
protein_coding	MAATS1	ENSG00000183833.16	0	0
protein_coding	AC069444.2	ENSG00000285585.1	0	0
protein_coding	NR1I2	ENSG00000144852.17	0	0
protein_coding	STXBP5L	ENSG00000145087.12	0	0
protein_coding	ARGFX	ENSG00000186103.3	0	0
protein_coding	FBXO40	ENSG00000163833.7	0	0
protein_coding	CASR	ENSG00000036828.16	0	0
protein_coding	PARP15	ENSG00000173200.12	0	0
protein_coding	ROPN1	ENSG00000065371.17	0	0
protein_coding	CFAP100	ENSG00000163885.11	0	0
protein_coding	UROC1	ENSG00000159650.8	0	0
protein_coding	CHST13	ENSG00000180767.9	0	0
protein_coding	C3orf22	ENSG00000180697.8	0	0
protein_coding	C3orf56	ENSG00000214324.5	0	0
protein_coding	PRR20G	ENSG00000239620.2	0	0
protein_coding	PODXL2	ENSG00000114631.10	0	0
protein_coding	KBTBD12	ENSG00000187715.13	0	0
protein_coding	DNAJB8	ENSG00000179407.3	0	0
protein_coding	EFCC1	ENSG00000114654.7	0	0
protein_coding	GP9	ENSG00000169704.4	0	0
protein_coding	RAB43	ENSG00000172780.16	0	0
protein_coding	ISY1-RAB43	ENSG00000261796.1	0	0
protein_coding	RHO	ENSG00000163914.4	0	0
protein_coding	H1FOO	ENSG00000178804.7	0	0
protein_coding	TRH	ENSG00000170893.3	0	0
protein_coding	ALG1L2	ENSG00000251287.8	0	0
protein_coding	COL6A5	ENSG00000172752.14	0	0
protein_coding	COL6A6	ENSG00000206384.10	0	0
protein_coding	NPHP3-ACAD11	ENSG00000274810.4	0	0
protein_coding	BFSP2	ENSG00000170819.4	0	0

protein_coding	SLCO2A1	ENSG00000174640.13	0	0
protein_coding	KY	ENSG00000174611.11	0	0
protein_coding	SOX14	ENSG00000168875.2	0	0
protein_coding	CLDN18	ENSG00000066405.12	0	0
protein_coding	A4GNT	ENSG00000118017.3	0	0
protein_coding	PRR23A	ENSG00000206260.3	0	0
protein_coding	PRR23B	ENSG00000184814.5	0	0
protein_coding	PRR23C	ENSG00000233701.3	0	0
protein_coding	RBP2	ENSG00000114113.6	0	0
protein_coding	TRIM42	ENSG00000155890.3	0	0
protein_coding	SPSB4	ENSG00000175093.4	0	0
protein_coding	AC112504.2	ENSG00000285558.1	0	0
protein_coding	CPB1	ENSG00000153002.11	0	0
protein_coding	CPA3	ENSG00000163751.3	0	0
protein_coding	TM4SF18	ENSG00000163762.6	0	0
protein_coding	MINDY4B	ENSG00000214237.10	0	0
protein_coding	AC020636.2	ENSG00000260234.5	0	0
protein_coding	CLRN1	ENSG00000163646.11	0	0
protein_coding	GPR171	ENSG00000174946.6	0	0
protein_coding	P2RY14	ENSG00000174944.8	0	0
protein_coding	P2RY13	ENSG00000181631.6	0	0
protein_coding	P2RY12	ENSG00000169313.9	0	0
protein_coding	AADACL2	ENSG00000197953.5	0	0
protein_coding	GPR149	ENSG00000174948.5	0	0
protein_coding	DWORF	ENSG00000240045.1	0	0
protein_coding	IQCJ-SCHIP1	ENSG00000283154.2	0	0
protein_coding	IQCJ	ENSG00000214216.10	0	0
protein_coding	OTOL1	ENSG00000182447.4	0	0
protein_coding	SI	ENSG00000090402.7	0	0
protein_coding	ZBBX	ENSG00000169064.12	0	0
protein_coding	WDR49	ENSG00000174776.11	0	0
protein_coding	LRRC31	ENSG00000114248.9	0	0
protein_coding	SAMD7	ENSG00000187033.9	0	0
protein_coding	AC026316.5	ENSG00000285218.1	0	0
protein_coding	SLC2A2	ENSG00000163581.13	0	0
protein_coding	TMEM212	ENSG00000186329.9	0	0
protein_coding	GHSR	ENSG00000121853.3	0	0
protein_coding	SPATA16	ENSG00000144962.6	0	0
protein_coding	KCNMB2	ENSG00000197584.11	0	0
protein_coding	AC117457.1	ENSG00000275163.1	0	0
protein_coding	CCDC39	ENSG00000284862.1	0	0
protein_coding	CCDC39	ENSG00000145075.12	0	0
protein_coding	AC131160.1	ENSG00000283765.1	0	0
protein_coding	MAP6D1	ENSG00000180834.7	0	0

protein_coding	HTR3D	ENSG00000186090.10	0	0
protein_coding	HTR3E	ENSG00000186038.9	0	0
protein_coding	VWA5B2	ENSG00000145198.14	0	0
protein_coding	EEF1AKMT4-ECE2	ENSG00000284917.1	0	0
protein_coding	ECE2	ENSG00000145194.18	0	0
protein_coding	THPO	ENSG00000090534.19	0	0
protein_coding	C3orf70	ENSG00000187068.2	0	0
protein_coding	AC068631.2	ENSG00000283149.1	0	0
protein_coding	FETUB	ENSG00000090512.11	0	0
protein_coding	KNG1	ENSG00000113889.13	0	0
protein_coding	ADIPOQ	ENSG00000181092.9	0	0
protein_coding	SST	ENSG00000157005.3	0	0
protein_coding	RTP2	ENSG00000198471.1	0	0
protein_coding	TMEM207	ENSG00000198398.2	0	0
protein_coding	GMNC	ENSG00000205835.8	0	0
protein_coding	PYDC2	ENSG00000253548.1	0	0
protein_coding	HRASLS	ENSG00000127252.5	0	0
protein_coding	ATP13A4	ENSG00000127249.14	0	0
protein_coding	GP5	ENSG00000178732.5	0	0
protein_coding	ZDHHC19	ENSG00000163958.13	0	0
protein_coding	SLC51A	ENSG00000163959.9	0	0
protein_coding	AC069257.3	ENSG00000272741.1	0	0
protein_coding	TM4SF19-TCTEX1D2	ENSG00000273331.1	0	0
protein_coding	SMCO1	ENSG00000214097.4	0	0
protein_coding	ZNF732	ENSG00000186777.11	0	0
protein_coding	NKX1-1	ENSG00000235608.1	0	0
protein_coding	HGFAC	ENSG00000109758.8	0	0
protein_coding	OTOP1	ENSG00000163982.5	0	0
protein_coding	C4orf50	ENSG00000181215.15	0	0
protein_coding	JAKMIP1	ENSG00000152969.19	0	0
protein_coding	AC092442.1	ENSG00000284684.1	0	0
protein_coding	PSAPL1	ENSG00000178597.6	0	0
protein_coding	GPR78	ENSG00000155269.11	0	0
protein_coding	HMX1	ENSG00000215612.7	0	0
protein_coding	FAM90A26	ENSG00000229924.3	0	0
protein_coding	USP17L10	ENSG00000231396.2	0	0
protein_coding	USP17L11	ENSG00000233136.2	0	0
protein_coding	USP17L12	ENSG00000227551.1	0	0
protein_coding	USP17L13	ENSG00000232399.3	0	0
protein_coding	USP17L15	ENSG00000223569.6	0	0
protein_coding	USP17L17	ENSG00000249104.2	0	0

protein_coding	USP17L18	ENSG00000250844.2	0	0
protein_coding	USP17L19	ENSG00000248920.2	0	0
protein_coding	USP17L20	ENSG00000250745.2	0	0
protein_coding	USP17L21	ENSG00000249811.2	0	0
protein_coding	USP17L22	ENSG00000248933.2	0	0
protein_coding	USP17L23	ENSG00000250913.2	0	0
protein_coding	USP17L24	ENSG00000232264.4	0	0
protein_coding	USP17L25	ENSG00000230430.4	0	0
protein_coding	USP17L26	ENSG00000229579.4	0	0
protein_coding	USP17L5	ENSG00000227140.3	0	0
protein_coding	USP17L27	ENSG00000235780.3	0	0
protein_coding	USP17L28	ENSG00000231051.3	0	0
protein_coding	USP17L29	ENSG00000231637.3	0	0
protein_coding	USP17L30	ENSG00000228856.3	0	0
protein_coding	DEFB131A	ENSG00000186146.1	0	0
protein_coding	CLNK	ENSG00000109684.14	0	0
protein_coding	C1QTNF7	ENSG00000163145.12	0	0
protein_coding	CD38	ENSG00000004468.12	0	0
protein_coding	FGFBP2	ENSG00000137441.7	0	0
protein_coding	PROM1	ENSG00000007062.11	0	0
protein_coding	CLRN2	ENSG00000249581.2	0	0
protein_coding	LGI2	ENSG00000153012.11	0	0
protein_coding	AC104662.2	ENSG00000281028.1	0	0
protein_coding	SLC34A2	ENSG00000157765.12	0	0
protein_coding	CCKAR	ENSG00000163394.5	0	0
protein_coding	DTHD1	ENSG00000197057.9	0	0
protein_coding	NWD2	ENSG00000174145.7	0	0
protein_coding	C4orf19	ENSG00000154274.14	0	0
protein_coding	TLR10	ENSG00000174123.10	0	0
protein_coding	PHOX2B	ENSG00000109132.6	0	0
protein_coding	BEND4	ENSG00000188848.15	0	0
protein_coding	SHISA3	ENSG00000178343.4	0	0
protein_coding	GRXCR1	ENSG00000215203.2	0	0
protein_coding	YIPF7	ENSG00000177752.14	0	0
protein_coding	COX7B2	ENSG00000170516.16	0	0
protein_coding	GABRA4	ENSG00000109158.10	0	0
protein_coding	SLC10A4	ENSG00000145248.6	0	0
protein_coding	ZAR¬†1	ENSG00000182223.7	0	0
protein_coding	CWH43	ENSG00000109182.11	0	0
protein_coding	AC058822.1	ENSG00000282278.1	0	0
protein_coding	GSX2	ENSG00000180613.10	0	0
protein_coding	KDR	ENSG00000128052.9	0	0
protein_coding	PDCL2	ENSG00000163440.11	0	0
protein_coding	KIAA1211	ENSG00000109265.14	0	0

protein_coding	HOPX	ENSG00000171476.21	0	0
protein_coding	ADGRL3	ENSG00000150471.16	0	0
protein_coding	STAP1	ENSG00000035720.7	0	0
protein_coding	GNRHR	ENSG00000109163.6	0	0
protein_coding	TMPRSS11D	ENSG00000153802.11	0	0
protein_coding	TMPRSS11A	ENSG00000187054.15	0	0
protein_coding	TMPRSS11F	ENSG00000198092.5	0	0
protein_coding	TMPRSS11B	ENSG00000185873.7	0	0
protein_coding	UGT2B17	ENSG00000197888.2	0	0
protein_coding	UGT2B10	ENSG00000109181.11	0	0
protein_coding	UGT2A3	ENSG00000135220.10	0	0
protein_coding	UGT2B28	ENSG00000135226.17	0	0
protein_coding	UGT2A2	ENSG00000271271.5	0	0
protein_coding	AC108941.2	ENSG00000284695.1	0	0
protein_coding	CSN1S1	ENSG00000126545.13	0	0
protein_coding	CSN2	ENSG00000135222.6	0	0
protein_coding	PRR27	ENSG00000187533.13	0	0
protein_coding	CSN3	ENSG00000171209.3	0	0
protein_coding	CABS1	ENSG00000145309.5	0	0
protein_coding	SMR3A	ENSG00000109208.4	0	0
protein_coding	SMR3B	ENSG00000171201.10	0	0
protein_coding	OPRPN	ENSG00000171199.10	0	0
protein_coding	MUC7	ENSG00000171195.10	0	0
protein_coding	AMBN	ENSG00000178522.14	0	0
protein_coding	ENAM	ENSG00000132464.11	0	0
protein_coding	GC	ENSG00000145321.12	0	0
protein_coding	NPFFR2	ENSG00000056291.17	0	0
protein_coding	ALB	ENSG00000163631.16	0	0
protein_coding	AFP	ENSG00000081051.7	0	0
protein_coding	AFM	ENSG00000079557.4	0	0
protein_coding	CXCL6	ENSG00000124875.9	0	0
protein_coding	PF4V1	ENSG00000109272.3	0	0
protein_coding	PPBP	ENSG00000163736.3	0	0
protein_coding	PPEF2	ENSG00000156194.17	0	0
protein_coding	FAM47E-	ENSG00000272414.5	0	0
protein_coding	CCDC158	ENSG00000163749.17	0	0
protein_coding	SOWAHB	ENSG00000186212.3	0	0
protein_coding	CXCL13	ENSG00000156234.7	0	0
protein_coding	NAA11	ENSG00000156269.4	0	0
protein_coding	GK2	ENSG00000196475.5	0	0
protein_coding	SLC10A6	ENSG00000145283.7	0	0
protein_coding	C4orf36	ENSG00000163633.11	0	0
protein_coding	AC093827.5	ENSG00000285458.1	0	0
protein_coding	DSPP	ENSG00000152591.13	0	0

protein_coding	DMP1	ENSG00000152592.13	0	0
protein_coding	MEPE	ENSG00000152595.16	0	0
protein_coding	SPP1	ENSG00000118785.13	0	0
protein_coding	PIGY	ENSG00000255072.1	0	0
protein_coding	AC098582.1	ENSG00000285385.1	0	0
protein_coding	MMRN1	ENSG00000138722.9	0	0
protein_coding	GRID2	ENSG00000152208.12	0	0
protein_coding	HPGDS	ENSG00000163106.10	0	0
protein_coding	UNC5C	ENSG00000182168.14	0	0
protein_coding	PDHA2	ENSG00000163114.5	0	0
protein_coding	STPG2	ENSG00000163116.9	0	0
protein_coding	ADH4	ENSG00000198099.8	0	0
protein_coding	ADH1A	ENSG00000187758.7	0	0
protein_coding	C4orf17	ENSG00000138813.9	0	0
protein_coding	C4orf54	ENSG00000248713.1	0	0
protein_coding	TACR3	ENSG00000169836.4	0	0
protein_coding	CXXC4	ENSG00000168772.10	0	0
protein_coding	ARHGEF38	ENSG00000236699.8	0	0
protein_coding	GIMD1	ENSG00000250298.4	0	0
protein_coding	ETNPPL	ENSG00000164089.8	0	0
protein_coding	AC126283.2	ENSG00000285330.1	0	0
protein_coding	LRIT3	ENSG00000183423.11	0	0
protein_coding	NEUROG2	ENSG00000178403.3	0	0
protein_coding	NDST4	ENSG00000138653.9	0	0
protein_coding	MTRNR2L13	ENSG00000270394.4	0	0
protein_coding	FABP2	ENSG00000145384.3	0	0
protein_coding	QRFPR	ENSG00000186867.10	0	0
protein_coding	ADAD1	ENSG00000164113.10	0	0
protein_coding	IL2	ENSG00000109471.4	0	0
protein_coding	IL21	ENSG00000138684.8	0	0
protein_coding	MGAT4D	ENSG00000205301.11	0	0
protein_coding	UCP1	ENSG00000109424.3	0	0
protein_coding	GYPE	ENSG00000197465.13	0	0
protein_coding	GYPB	ENSG00000250361.8	0	0
protein_coding	GYPA	ENSG00000170180.21	0	0
protein_coding	AC098588.1	ENSG00000285713.1	0	0
protein_coding	C4orf51	ENSG00000237136.7	0	0
protein_coding	REELD1	ENSG00000250673.2	0	0
protein_coding	POU4F2	ENSG00000151615.3	0	0
protein_coding	PRSS48	ENSG00000189099.11	0	0
protein_coding	FGB	ENSG00000171564.11	0	0
protein_coding	FGA	ENSG00000171560.14	0	0
protein_coding	FGG	ENSG00000171557.16	0	0
protein_coding	RBM46	ENSG00000151962.7	0	0

protein_coding	NPY2R	ENSG00000185149.5	0	0
protein_coding	ASIC5	ENSG00000256394.2	0	0
protein_coding	C4orf45	ENSG00000164123.6	0	0
protein_coding	NPY5R	ENSG00000164129.11	0	0
protein_coding	SMIM31	ENSG00000248771.5	0	0
protein_coding	APELA	ENSG00000248329.5	0	0
protein_coding	TRIM61	ENSG00000183439.7	0	0
protein_coding	TRIM75P	ENSG00000250374.4	0	0
protein_coding	GK3P	ENSG00000229894.4	0	0
protein_coding	SCRG1	ENSG00000164106.7	0	0
protein_coding	HAND2	ENSG00000164107.8	0	0
protein_coding	ADAM29	ENSG00000168594.15	0	0
protein_coding	CLDN22	ENSG00000177300.6	0	0
protein_coding	CLDN24	ENSG00000185758.9	0	0
protein_coding	ENPP6	ENSG00000164303.10	0	0
protein_coding	HELT	ENSG00000187821.8	0	0
protein_coding	KLKB1	ENSG00000164344.15	0	0
protein_coding	F11	ENSG00000088926.13	0	0
protein_coding	AC018709.1	ENSG00000272297.2	0	0
protein_coding	MTNR1A	ENSG00000168412.6	0	0
protein_coding	ZFP42	ENSG00000179059.9	0	0
protein_coding	TRIML1	ENSG00000184108.7	0	0
protein_coding	FRG2	ENSG00000205097.6	0	0
protein_coding	DUX4	ENSG00000260596.5	0	0
protein_coding	LRRC14B	ENSG00000185028.3	0	0
protein_coding	SLC9A3	ENSG00000066230.11	0	0
protein_coding	ZDHHC11B	ENSG00000206077.10	0	0
protein_coding	NKD2	ENSG00000145506.13	0	0
protein_coding	SLC6A19	ENSG00000174358.15	0	0
protein_coding	SLC6A18	ENSG00000164363.9	0	0
protein_coding	TERT	ENSG00000164362.19	0	0
protein_coding	SLC6A3	ENSG00000142319.17	0	0
protein_coding	TAS2R1	ENSG00000169777.6	0	0
protein_coding	LINC02218	ENSG00000249662.6	0	0
protein_coding	AC106774.4	ENSG00000268799.3	0	0
protein_coding	TAF11L2	ENSG00000284373.1	0	0
protein_coding	TAF11L3	ENSG00000284439.1	0	0
protein_coding	TAF11L4	ENSG00000284283.1	0	0
protein_coding	TAF11L5	ENSG00000284234.1	0	0
protein_coding	TAF11L6	ENSG00000284042.1	0	0
protein_coding	TAF11L7	ENSG00000284465.1	0	0
protein_coding	TAF11L8	ENSG00000283967.1	0	0
protein_coding	TAF11L9	ENSG00000283988.1	0	0
protein_coding	TAF11L10	ENSG00000284356.1	0	0

protein_coding	TAF11L11	ENSG00000283740.1	0	0
protein_coding	TAF11L12	ENSG00000249156.2	0	0
protein_coding	TAF11L13	ENSG00000283776.1	0	0
protein_coding	TAF11L14	ENSG00000250782.1	0	0
protein_coding	H3.Y	ENSG00000269466.3	0	0
protein_coding	CDH9	ENSG00000113100.9	0	0
protein_coding	RXFP3	ENSG00000182631.6	0	0
protein_coding	SLC45A2	ENSG00000164175.14	0	0
protein_coding	C1QTNF3-AMACR	ENSG00000273294.1	0	0
protein_coding	TTC23L	ENSG00000205838.14	0	0
protein_coding	AGXT2	ENSG00000113492.13	0	0
protein_coding	CAPSL	ENSG00000152611.11	0	0
protein_coding	UGT3A1	ENSG00000145626.11	0	0
protein_coding	C9	ENSG00000113600.10	0	0
protein_coding	MROH2B	ENSG00000171495.16	0	0
protein_coding	C6	ENSG00000039537.13	0	0
protein_coding	FGF10	ENSG00000070193.4	0	0
protein_coding	GZMK	ENSG00000113088.5	0	0
protein_coding	GZMA	ENSG00000145649.7	0	0
protein_coding	DDX4	ENSG00000152670.18	0	0
protein_coding	ANKRD55	ENSG00000164512.17	0	0
protein_coding	C5orf67	ENSG00000225940.6	0	0
protein_coding	ACTBL2	ENSG00000169067.3	0	0
protein_coding	GAPT	ENSG00000175857.8	0	0
protein_coding	RAB3C	ENSG00000152932.7	0	0
protein_coding	LRRC70	ENSG00000186105.7	0	0
protein_coding	HTR1A	ENSG00000178394.4	0	0
protein_coding	RGS7BP	ENSG00000186479.4	0	0
protein_coding	CD180	ENSG00000134061.5	0	0
protein_coding	SERF1B	ENSG00000205572.9	0	0
protein_coding	SERF1A	ENSG00000172058.15	0	0
protein_coding	NAIP	ENSG00000249437.7	0	0
protein_coding	CARTPT	ENSG00000164326.4	0	0
protein_coding	ZNF366	ENSG00000178175.11	0	0
protein_coding	TMEM174	ENSG00000164325.7	0	0
protein_coding	S100Z	ENSG00000171643.13	0	0
protein_coding	AC008581.2	ENSG00000285000.1	0	0
protein_coding	AC022414.1	ENSG00000284762.1	0	0
protein_coding	OTP	ENSG00000171540.7	0	0
protein_coding	BHMT	ENSG00000145692.14	0	0
protein_coding	SPZ1	ENSG00000164299.6	0	0
protein_coding	CKMT2	ENSG00000131730.15	0	0

protein_coding	ACOT12	ENSG00000172497.8	0	0
protein_coding	LIX1	ENSG00000145721.11	0	0
protein_coding	SLCO4C1	ENSG00000173930.8	0	0
protein_coding	AC008575.1	ENSG00000258864.1	0	0
protein_coding	TICAM2	ENSG00000243414.5	0	0
protein_coding	LVRN	ENSG00000172901.19	0	0
protein_coding	FAM170A	ENSG00000164334.15	0	0
protein_coding	FTMT	ENSG00000181867.2	0	0
protein_coding	ZNF474	ENSG00000164185.5	0	0
protein_coding	AC010255.3	ENSG00000250803.6	0	0
protein_coding	SLC27A6	ENSG00000113396.12	0	0
protein_coding	KIAA1024L	ENSG00000186367.6	0	0
protein_coding	AC008695.1	ENSG00000273217.1	0	0
protein_coding	MEIKIN	ENSG00000239642.5	0	0
protein_coding	AC026398.1	ENSG00000281938.1	0	0
protein_coding	IL3	ENSG00000164399.4	0	0
protein_coding	CSF2	ENSG00000164400.5	0	0
protein_coding	AC116366.3	ENSG00000283782.2	0	0
protein_coding	IL5	ENSG00000113525.9	0	0
protein_coding	IL13	ENSG00000169194.9	0	0
protein_coding	IL4	ENSG00000113520.10	0	0
protein_coding	CCNI2	ENSG00000205089.7	0	0
protein_coding	AC104109.3	ENSG00000272772.1	0	0
protein_coding	DCANP1	ENSG00000251380.3	0	0
protein_coding	TIFAB	ENSG00000255833.1	0	0
protein_coding	NEUROG1	ENSG00000181965.5	0	0
protein_coding	SLC25A48	ENSG00000145832.14	0	0
protein_coding	IL9	ENSG00000145839.1	0	0
protein_coding	SMIM32	ENSG00000271824.1	0	0
protein_coding	TRPC7	ENSG00000069018.18	0	0
protein_coding	MYOT	ENSG00000120729.9	0	0
protein_coding	WNT8A	ENSG00000061492.11	0	0
protein_coding	MATR3	ENSG00000280987.4	0	0
protein_coding	SLC23A1	ENSG00000170482.16	0	0
protein_coding	AC142391.1	ENSG00000279686.1	0	0
protein_coding	ECSCR	ENSG00000249751.3	0	0
protein_coding	SMIM33	ENSG00000283288.1	0	0
protein_coding	PSD2	ENSG00000146005.3	0	0
protein_coding	SLC4A9	ENSG00000113073.14	0	0
protein_coding	EIF4EBP3	ENSG00000243056.1	0	0
protein_coding	DND1	ENSG00000256453.1	0	0
protein_coding	PCDHA1	ENSG00000204970.9	0	0
protein_coding	PCDHA2	ENSG00000204969.6	0	0
protein_coding	PCDHA3	ENSG00000255408.3	0	0

protein_coding	PCDHA5	ENSG00000204965.8	0	0
protein_coding	PCDHA6	ENSG00000081842.17	0	0
protein_coding	PCDHA8	ENSG00000204962.5	0	0
protein_coding	PCDHA9	ENSG00000204961.6	0	0
protein_coding	PCDHA11	ENSG00000249158.6	0	0
protein_coding	PCDHA12	ENSG00000251664.3	0	0
protein_coding	PCDHA13	ENSG00000239389.7	0	0
protein_coding	PCDHAC1	ENSG00000248383.4	0	0
protein_coding	PCDHAC2	ENSG00000243232.4	0	0
protein_coding	PCDHB1	ENSG00000171815.5	0	0
protein_coding	AC244517.10	ENSG00000279983.1	0	0
protein_coding	SLC25A2	ENSG00000120329.6	0	0
protein_coding	PCDHGC4	ENSG00000242419.5	0	0
protein_coding	PCDHGC5	ENSG00000240764.3	0	0
protein_coding	PCDH12	ENSG00000113555.5	0	0
protein_coding	HMHB1	ENSG00000158497.3	0	0
protein_coding	AC091959.3	ENSG00000275740.1	0	0
protein_coding	GPR151	ENSG00000173250.2	0	0
protein_coding	SPINK1	ENSG00000164266.10	0	0
protein_coding	SCGB3A2	ENSG00000164265.8	0	0
protein_coding	SPINK14	ENSG00000196800.6	0	0
protein_coding	SPINK6	ENSG00000178172.6	0	0
protein_coding	MARCOL	ENSG00000248109.2	0	0
protein_coding	SPINK13	ENSG00000214510.9	0	0
protein_coding	SPINK9	ENSG00000204909.7	0	0
protein_coding	HTR4	ENSG00000164270.17	0	0
protein_coding	IL17B	ENSG00000127743.5	0	0
protein_coding	PDE6A	ENSG00000132915.10	0	0
protein_coding	SLC6A7	ENSG00000011083.8	0	0
protein_coding	SLC36A3	ENSG00000186334.9	0	0
protein_coding	SLC36A2	ENSG00000186335.8	0	0
protein_coding	GLRA1	ENSG00000145888.10	0	0
protein_coding	GRIA1	ENSG00000155511.17	0	0
protein_coding	HAND1	ENSG00000113196.2	0	0
protein_coding	KIF4B	ENSG00000226650.5	0	0
protein_coding	TIMD4	ENSG00000145850.8	0	0
protein_coding	HAVCR1	ENSG00000113249.12	0	0
protein_coding	FAM71B	ENSG00000170613.4	0	0
protein_coding	AC008676.3	ENSG00000285868.1	0	0
protein_coding	FNDC9	ENSG00000172568.4	0	0
protein_coding	C5orf52	ENSG00000187658.6	0	0
protein_coding	IL12B	ENSG00000113302.4	0	0
protein_coding	ADRA1B	ENSG00000170214.4	0	0
protein_coding	ATP10B	ENSG00000118322.13	0	0

protein_coding	GABRA6	ENSG00000145863.10	0	0
protein_coding	GABRA1	ENSG00000022355.16	0	0
protein_coding	FOXI1	ENSG00000168269.9	0	0
protein_coding	C5orf58	ENSG00000234511.9	0	0
protein_coding	LCP2	ENSG00000043462.11	0	0
protein_coding	KCNIP1	ENSG00000182132.13	0	0
protein_coding	SMIM23	ENSG00000185662.9	0	0
protein_coding	EFCAB9	ENSG00000214360.4	0	0
protein_coding	C5orf47	ENSG00000185056.9	0	0
protein_coding	NSG2	ENSG00000170091.10	0	0
protein_coding	CPLX2	ENSG00000145920.14	0	0
protein_coding	FAM153B	ENSG00000182230.12	0	0
protein_coding	AC139491.7	ENSG00000285476.1	0	0
protein_coding	CDHR2	ENSG00000074276.10	0	0
protein_coding	SNCB	ENSG00000074317.10	0	0
protein_coding	EIF4E1B	ENSG00000175766.12	0	0
protein_coding	UNC5A	ENSG00000113763.11	0	0
protein_coding	HK3	ENSG00000160883.10	0	0
protein_coding	FGFR4	ENSG00000160867.14	0	0
protein_coding	PFN3	ENSG00000196570.2	0	0
protein_coding	PROP1	ENSG00000175325.2	0	0
protein_coding	FAM153C	ENSG00000204677.10	0	0
protein_coding	GMCL2	ENSG00000244234.2	0	0
protein_coding	AC113348.1	ENSG00000285891.1	0	0
protein_coding	AC113348.2	ENSG00000285978.1	0	0
protein_coding	GRM6	ENSG00000113262.15	0	0
protein_coding	C5orf60	ENSG00000204661.9	0	0
protein_coding	CBY3	ENSG00000204659.4	0	0
protein_coding	LTC4S	ENSG00000213316.9	0	0
protein_coding	RASGEF1C	ENSG00000146090.15	0	0
protein_coding	FLT4	ENSG00000037280.15	0	0
protein_coding	OR2Y1	ENSG00000174339.2	0	0
protein_coding	BTNL8	ENSG00000113303.11	0	0
protein_coding	BTNL3	ENSG00000168903.8	0	0
protein_coding	BTNL9	ENSG00000165810.16	0	0
protein_coding	OR2V1	ENSG00000185372.3	0	0
protein_coding	OR2V2	ENSG00000182613.2	0	0
protein_coding	OR4F3	ENSG00000230178.1	0	0
protein_coding	FAM217A	ENSG00000145975.14	0	0
protein_coding	C6orf201	ENSG00000185689.16	0	0
protein_coding	F13A1	ENSG00000124491.15	0	0
protein_coding	LY86	ENSG00000112799.8	0	0
protein_coding	TXNDC5	ENSG00000239264.8	0	0

protein_coding	BLOC1S5-TXNDC5	ENSG00000259040.5	0	0
protein_coding	EEF1E1-BLOC1S5	ENSG00000265818.1	0	0
protein_coding	C6orf52	ENSG00000137434.11	0	0
protein_coding	AL024498.2	ENSG00000272162.1	0	0
protein_coding	GCM2	ENSG00000124827.6	0	0
protein_coding	ERVFRD-1	ENSG00000244476.2	0	0
protein_coding	STMND1	ENSG00000230873.8	0	0
protein_coding	HDGFL1	ENSG00000112273.6	0	0
protein_coding	NRSN1	ENSG00000152954.11	0	0
protein_coding	KAAG1	ENSG00000146049.1	0	0
protein_coding	ARMH2	ENSG00000260286.3	0	0
protein_coding	AL512428.1	ENSG00000282804.1	0	0
protein_coding	SCGN	ENSG00000079689.13	0	0
protein_coding	HIST1H2AA	ENSG00000164508.4	0	0
protein_coding	HIST1H2BA	ENSG00000146047.6	0	0
protein_coding	SLC17A4	ENSG00000146039.10	0	0
protein_coding	SLC17A1	ENSG00000124568.10	0	0
protein_coding	SLC17A3	ENSG00000124564.17	0	0
protein_coding	SLC17A2	ENSG00000112337.10	0	0
protein_coding	HIST1H3A	ENSG00000275714.1	0	0
protein_coding	HIST1H4A	ENSG00000278637.1	0	0
protein_coding	HIST1H2BB	ENSG00000276410.3	0	0
protein_coding	HIST1H1T	ENSG00000187475.5	0	0
protein_coding	HIST1H3D	ENSG00000197409.7	0	0
protein_coding	HIST1H2AD	ENSG00000196866.2	0	0
protein_coding	HIST1H4F	ENSG00000274618.1	0	0
protein_coding	HIST1H4G	ENSG00000275663.1	0	0
protein_coding	HIST1H3F	ENSG00000277775.1	0	0
protein_coding	HIST1H2BI	ENSG00000278588.1	0	0
protein_coding	BTN1A1	ENSG00000124557.12	0	0
protein_coding	POM121L2	ENSG00000158553.4	0	0
protein_coding	HIST1H2BL	ENSG00000185130.5	0	0
protein_coding	HIST1H2AI	ENSG00000196747.4	0	0
protein_coding	HIST1H2BM	ENSG00000273703.1	0	0
protein_coding	HIST1H4K	ENSG00000273542.1	0	0
protein_coding	HIST1H2AK	ENSG00000275221.1	0	0
protein_coding	HIST1H2AL	ENSG00000276903.1	0	0
protein_coding	HIST1H4L	ENSG00000275126.1	0	0
protein_coding	HIST1H3J	ENSG00000197153.4	0	0
protein_coding	HIST1H2AM	ENSG00000278677.1	0	0
protein_coding	OR2B2	ENSG00000168131.4	0	0

protein_coding	AL021997.3	ENSG00000276302.1	0	0
protein_coding	GPX6	ENSG00000198704.9	0	0
protein_coding	GPX5	ENSG00000224586.6	0	0
protein_coding	OR2W1	ENSG00000204704.2	0	0
protein_coding	OR2B3	ENSG00000204703.5	0	0
protein_coding	OR2J1	ENSG00000204702.5	0	0
protein_coding	OR2J3	ENSG00000204701.2	0	0
protein_coding	OR2J2	ENSG00000204700.5	0	0
protein_coding	OR14J1	ENSG00000204695.4	0	0
protein_coding	OR5V1	ENSG00000243729.4	0	0
protein_coding	OR12D3	ENSG00000112462.8	0	0
protein_coding	OR12D2	ENSG00000280236.3	0	0
protein_coding	OR11A1	ENSG00000204694.11	0	0
protein_coding	OR10C1	ENSG00000206474.8	0	0
protein_coding	OR2H1	ENSG00000204688.9	0	0
protein_coding	MAS1L	ENSG00000204687.4	0	0
protein_coding	OR2I1P	ENSG00000237988.5	0	0
protein_coding	UBD	ENSG00000213886.3	0	0
protein_coding	OR2H2	ENSG00000204657.4	0	0
protein_coding	MOG	ENSG00000204655.11	0	0
protein_coding	HLA-G	ENSG00000204632.11	0	0
protein_coding	TRIM31	ENSG00000204616.10	0	0
protein_coding	TRIM40	ENSG00000204614.8	0	0
protein_coding	TRIM10	ENSG00000204613.10	0	0
protein_coding	TRIM15	ENSG00000204610.12	0	0
protein_coding	TRIM39-	ENSG00000248167.7	0	0
protein_coding	RPP21	ENSG00000241370.5	0	0
protein_coding	GTF2H4	ENSG00000213780.10	0	0
protein_coding	SFTA2	ENSG00000196260.4	0	0
protein_coding	MUCL3	ENSG00000168631.12	0	0
protein_coding	MUC21	ENSG00000204544.5	0	0
protein_coding	MUC22	ENSG00000261272.1	0	0
protein_coding	C6orf15	ENSG00000204542.2	0	0
protein_coding	PSORS1C2	ENSG00000204538.3	0	0
protein_coding	MCCD1	ENSG00000204511.2	0	0
protein_coding	ATP6V1G2-DDX39B	ENSG00000254870.5	0	0
protein_coding	LTA	ENSG00000226979.8	0	0
protein_coding	TNF	ENSG00000232810.3	0	0
protein_coding	NCR3	ENSG00000204475.9	0	0
protein_coding	AIF1	ENSG00000204472.12	0	0
protein_coding	AL662899.2	ENSG00000263020.6	0	0
protein_coding	LY6G5B	ENSG00000240053.8	0	0

protein_coding	ABHD16A	ENSG00000204427.11	0	0
protein_coding	AL662899.1	ENSG00000204422.7	0	0
protein_coding	LY6G6F	ENSG00000204424.9	0	0
protein_coding	LY6G6F-<br>LY6G6D	ENSG00000250641.1	0	0
protein_coding	LY6G6E	ENSG00000255552.7	0	0
protein_coding	LY6G6D	ENSG00000244355.7	0	0
protein_coding	MSH5	ENSG00000204410.14	0	0
protein_coding	MSH5-	ENSG00000255152.8	0	0
protein_coding	SAPCD1	ENSG00000228727.8	0	0
protein_coding	SLC44A4	ENSG00000204385.11	0	0
protein_coding	AL645922.1	ENSG00000244255.5	0	0
protein_coding	CFB	ENSG00000243649.8	0	0
protein_coding	C4B	ENSG00000224389.9	0	0
protein_coding	CYP21A2	ENSG00000231852.7	0	0
protein_coding	AL662884.4	ENSG00000285085.1	0	0
protein_coding	C6orf10	ENSG00000204296.11	0	0
protein_coding	BTNL2	ENSG00000204290.10	0	0
protein_coding	HLA-DRA	ENSG00000204287.13	0	0
protein_coding	HLA-DRB5	ENSG00000198502.5	0	0
protein_coding	HLA-DQA1	ENSG00000196735.11	0	0
protein_coding	HLA-DQB2	ENSG00000232629.8	0	0
protein_coding	AL669918.1	ENSG00000250264.1	0	0
protein_coding	AL645941.2	ENSG00000248993.1	0	0
protein_coding	HLA-DOA	ENSG00000204252.13	0	0
protein_coding	AL662820.1	ENSG00000285064.1	0	0
protein_coding	ZBTB9	ENSG00000213588.5	0	0
protein_coding	IP6K3	ENSG00000161896.11	0	0
protein_coding	MLN	ENSG00000096395.10	0	0
protein_coding	RPS10-	ENSG00000270800.3	0	0
protein_coding	PACSIN1	ENSG00000124507.10	0	0
protein_coding	TCP11	ENSG00000124678.17	0	0
protein_coding	TULP1	ENSG00000112041.12	0	0
protein_coding	CLPSL1	ENSG00000204140.9	0	0
protein_coding	CLPS	ENSG00000137392.9	0	0
protein_coding	SLC26A8	ENSG00000112053.13	0	0
protein_coding	PNPLA1	ENSG00000180316.12	0	0
protein_coding	C6orf222	ENSG00000189325.6	0	0
protein_coding	RAB44	ENSG00000255587.8	0	0
protein_coding	PI16	ENSG00000164530.14	0	0
protein_coding	GLP1R	ENSG00000112164.5	0	0
protein_coding	KCNK5	ENSG00000164626.8	0	0
protein_coding	KCNK17	ENSG00000124780.13	0	0

protein_coding	KCNK16	ENSG00000095981.10	0	0
protein_coding	KIF6	ENSG00000164627.17	0	0
protein_coding	LRFN2	ENSG00000156564.8	0	0
protein_coding	TSPO2	ENSG00000112212.11	0	0
protein_coding	APOBEC2	ENSG00000124701.5	0	0
protein_coding	TREML2	ENSG00000112195.8	0	0
protein_coding	TREML4	ENSG00000188056.11	0	0
protein_coding	NCR2	ENSG00000096264.13	0	0
protein_coding	PGC	ENSG00000096088.16	0	0
protein_coding	PRICKLE4	ENSG00000278224.6	0	0
protein_coding	TOMM6	ENSG00000214736.7	0	0
protein_coding	AL096814.1	ENSG00000214732.2	0	0
protein_coding	GUCA1B	ENSG00000112599.8	0	0
protein_coding	SLC22A7	ENSG00000137204.14	0	0
protein_coding	CRIP3	ENSG00000146215.13	0	0
protein_coding	C6orf223	ENSG00000181577.15	0	0
protein_coding	CAPN11	ENSG00000137225.12	0	0
protein_coding	MYMX	ENSG00000262179.2	0	0
protein_coding	TMEM151B	ENSG00000178233.17	0	0
protein_coding	AL353588.1	ENSG00000272442.2	0	0
protein_coding	SPATS1	ENSG00000249481.6	0	0
protein_coding	TDRD6	ENSG00000180113.15	0	0
protein_coding	PLA2G7	ENSG00000146070.16	0	0
protein_coding	ANKRD66	ENSG00000230062.5	0	0
protein_coding	ADGRF2	ENSG00000164393.8	0	0
protein_coding	OPN5	ENSG00000124818.15	0	0
protein_coding	GLYATL3	ENSG00000203972.9	0	0
protein_coding	RHAG	ENSG00000112077.16	0	0
protein_coding	CRISP2	ENSG00000124490.13	0	0
protein_coding	PGK¬†2	ENSG00000170950.5	0	0
protein_coding	CRISP1	ENSG00000124812.14	0	0
protein_coding	DEFB133	ENSG00000214643.6	0	0
protein_coding	DEFB114	ENSG00000177684.3	0	0
protein_coding	DEFB113	ENSG00000214642.1	0	0
protein_coding	DEFB110	ENSG00000203970.3	0	0
protein_coding	DEFB112	ENSG00000180872.4	0	0
protein_coding	TFAP2D	ENSG00000008197.4	0	0
protein_coding	TFAP2B	ENSG00000008196.12	0	0
protein_coding	PKHD1	ENSG00000170927.14	0	0
protein_coding	IL17F	ENSG00000112116.9	0	0
protein_coding	GSTA2	ENSG00000244067.2	0	0
protein_coding	GSTA1	ENSG00000243955.5	0	0
protein_coding	GSTA5	ENSG00000182793.11	0	0
protein_coding	GCM1	ENSG00000137270.10	0	0

protein_coding	MLIP	ENSG00000146147.14	0	0
protein_coding	TINAG	ENSG00000137251.15	0	0
protein_coding	HCRTR2	ENSG00000137252.9	0	0
protein_coding	GFRAL	ENSG00000187871.2	0	0
protein_coding	HMGCLL1	ENSG00000146151.13	0	0
protein_coding	COL21A1	ENSG00000124749.16	0	0
protein_coding	FKBP1C	ENSG00000198225.5	0	0
protein_coding	ADGRB3	ENSG00000135298.13	0	0
protein_coding	COL19A1	ENSG00000082293.12	0	0
protein_coding	COL9A1	ENSG00000112280.16	0	0
protein_coding	B3GAT2	ENSG00000112309.10	0	0
protein_coding	AL365232.1	ENSG00000243501.5	0	0
protein_coding	DPPA5	ENSG00000203909.3	0	0
protein_coding	KHDC3L	ENSG00000203908.3	0	0
protein_coding	IMPG1	ENSG00000112706.11	0	0
protein_coding	HTR1B	ENSG00000135312.6	0	0
protein_coding	MEI4	ENSG00000269964.3	0	0
protein_coding	SNAP91	ENSG00000065609.14	0	0
protein_coding	AL589666.1	ENSG00000271793.1	0	0
protein_coding	HTR1E	ENSG00000168830.7	0	0
protein_coding	AL049697.1	ENSG00000213204.8	0	0
protein_coding	SPACA1	ENSG00000118434.8	0	0
protein_coding	CNR1	ENSG00000118432.12	0	0
protein_coding	SRSF12	ENSG00000154548.8	0	0
protein_coding	GABRR1	ENSG00000146276.11	0	0
protein_coding	GABRR2	ENSG00000111886.10	0	0
protein_coding	GJA10	ENSG00000135355.4	0	0
protein_coding	FUT9	ENSG00000172461.10	0	0
protein_coding	FHL5	ENSG00000112214.10	0	0
protein_coding	KLHL32	ENSG00000186231.16	0	0
protein_coding	PRDM13	ENSG00000112238.11	0	0
protein_coding	MCHR2	ENSG00000152034.10	0	0
protein_coding	SIM1	ENSG00000112246.9	0	0
protein_coding	LIN28B	ENSG00000187772.7	0	0
protein_coding	SCML4	ENSG00000146285.13	0	0
protein_coding	GPR6	ENSG00000146360.8	0	0
protein_coding	METTL24	ENSG00000053328.8	0	0
protein_coding	SLC22A16	ENSG00000004809.13	0	0
protein_coding	AL365214.3	ENSG00000281613.2	0	0
protein_coding	RFPL4B	ENSG00000251258.1	0	0
protein_coding	Z84488.2	ENSG00000285446.1	0	0
protein_coding	CALHM6	ENSG00000188820.12	0	0
protein_coding	RSPH4A	ENSG00000111834.12	0	0
protein_coding	GPRC6A	ENSG00000173612.9	0	0

protein_coding	RFX6	ENSG00000185002.9	0	0
protein_coding	VGLL2	ENSG00000170162.13	0	0
protein_coding	ROS1	ENSG00000047936.10	0	0
protein_coding	AL132671.2	ENSG00000282218.1	0	0
protein_coding	PLN	ENSG00000198523.5	0	0
protein_coding	FAM184A	ENSG00000111879.19	0	0
protein_coding	FABP7	ENSG00000164434.11	0	0
protein_coding	CLVS2	ENSG00000146352.12	0	0
protein_coding	AL096711.2	ENSG00000255330.9	0	0
protein_coding	SOGA3	ENSG00000214338.10	0	0
protein_coding	C6orf58	ENSG00000184530.8	0	0
protein_coding	THEMIS	ENSG00000172673.10	0	0
protein_coding	SMLR1	ENSG00000256162.2	0	0
protein_coding	ARG1	ENSG00000118520.14	0	0
protein_coding	ENPP3	ENSG00000154269.14	0	0
protein_coding	OR2A4	ENSG00000180658.4	0	0
protein_coding	CTAGE9	ENSG00000236761.5	0	0
protein_coding	TAAR9	ENSG00000237110.2	0	0
protein_coding	TAAR8	ENSG00000146385.1	0	0
protein_coding	TAAR6	ENSG00000146383.7	0	0
protein_coding	TAAR5	ENSG00000135569.4	0	0
protein_coding	TAAR2	ENSG00000146378.6	0	0
protein_coding	TAAR1	ENSG00000146399.1	0	0
protein_coding	VNN¬†3	ENSG00000093134.14	0	0
protein_coding	SLC35D3	ENSG00000182747.4	0	0
protein_coding	IL22RA2	ENSG00000164485.14	0	0
protein_coding	OLIG3	ENSG00000177468.6	0	0
protein_coding	PBOV1	ENSG00000254440.3	0	0
protein_coding	SMIM28	ENSG00000262543.1	0	0
protein_coding	ECT2L	ENSG00000203734.11	0	0
protein_coding	NMBR	ENSG00000135577.4	0	0
protein_coding	GJE1	ENSG00000203733.5	0	0
protein_coding	AL049844.1	ENSG00000257065.1	0	0
protein_coding	AL049844.3	ENSG00000280148.1	0	0
protein_coding	ZC2HC1B	ENSG00000118491.9	0	0
protein_coding	GRM1	ENSG00000152822.13	0	0
protein_coding	ADGB	ENSG00000118492.16	0	0
protein_coding	ULBP3	ENSG00000131019.10	0	0
protein_coding	MYCT1	ENSG00000120279.6	0	0
protein_coding	VIP	ENSG00000146469.12	0	0
protein_coding	CLDN20	ENSG00000171217.5	0	0
protein_coding	NOX3	ENSG00000074771.3	0	0
protein_coding	SOD2	ENSG00000285441.1	0	0
protein_coding	PNLDC1	ENSG00000146453.12	0	0

protein_coding	MAS1	ENSG00000130368.5	0	0
protein_coding	SLC22A2	ENSG00000112499.12	0	0
protein_coding	SLC22A3	ENSG00000146477.5	0	0
protein_coding	LPA	ENSG00000198670.11	0	0
protein_coding	PLG	ENSG00000122194.18	0	0
protein_coding	TBXT	ENSG00000164458.9	0	0
protein_coding	RAMACL	ENSG00000235272.2	0	0
protein_coding	CCR6	ENSG00000112486.16	0	0
protein_coding	TCP10L2	ENSG00000166984.11	0	0
protein_coding	GPR31	ENSG00000120436.3	0	0
protein_coding	UNC93A	ENSG00000112494.9	0	0
protein_coding	TTLL2	ENSG00000120440.14	0	0
protein_coding	TCP10	ENSG00000203690.12	0	0
protein_coding	FRMD1	ENSG00000153303.17	0	0
protein_coding	DACT2	ENSG00000164488.11	0	0
protein_coding	AL031315.1	ENSG00000285733.1	0	0
protein_coding	AC187653.1	ENSG00000248767.2	0	0
protein_coding	GET4	ENSG00000239857.6	0	0
protein_coding	CYP2W1	ENSG00000073067.13	0	0
protein_coding	UNCX	ENSG00000164853.8	0	0
protein_coding	GRIFIN	ENSG00000275572.1	0	0
protein_coding	PAPOLB	ENSG00000218823.1	0	0
protein_coding	MMD2	ENSG00000136297.14	0	0
protein_coding	RBAK-	ENSG00000272968.5	0	0
protein_coding	OCM	ENSG00000122543.10	0	0
protein_coding	RSPH10B	ENSG00000155026.16	0	0
protein_coding	ANKRD61	ENSG00000157999.5	0	0
protein_coding	GRID2IP	ENSG00000215045.8	0	0
protein_coding	RSPH10B2	ENSG00000169402.15	0	0
protein_coding	NXPH1	ENSG00000122584.12	0	0
protein_coding	VWDE	ENSG00000146530.13	0	0
protein_coding	DGKB	ENSG00000136267.13	0	0
protein_coding	MEOX2	ENSG00000106511.5	0	0
protein_coding	LRRC72	ENSG00000205858.9	0	0
protein_coding	AC019117.2	ENSG00000283321.1	0	0
protein_coding	PRPS1L1	ENSG00000229937.6	0	0
protein_coding	FERD3L	ENSG00000146618.3	0	0
protein_coding	TMEM196	ENSG00000173452.13	0	0
protein_coding	ABCB5	ENSG00000004846.16	0	0
protein_coding	NPY	ENSG00000122585.7	0	0
protein_coding	NPVF	ENSG00000105954.2	0	0
protein_coding	AC004080.3	ENSG00000257184.3	0	0
protein_coding	EVX1	ENSG00000106038.12	0	0
protein_coding	AC004593.3	ENSG00000285162.1	0	0

protein_coding	AC006978.2	ENSG00000281593.1	0	0
protein_coding	AC005154.5	ENSG00000281039.1	0	0
protein_coding	CRHR2	ENSG00000106113.18	0	0
protein_coding	INMT-	ENSG00000254959.6	0	0
protein_coding	AC004691.2	ENSG00000250424.4	0	0
protein_coding	GHRHR	ENSG00000106128.18	0	0
protein_coding	ADCYAP1R1	ENSG00000078549.14	0	0
protein_coding	NEUROD6	ENSG00000164600.6	0	0
protein_coding	ITPRID1	ENSG00000180347.13	0	0
protein_coding	PPP1R17	ENSG00000106341.10	0	0
protein_coding	NPSR1	ENSG00000187258.13	0	0
protein_coding	TBX20	ENSG00000164532.10	0	0
protein_coding	GPR141	ENSG00000187037.8	0	0
protein_coding	NME8	ENSG00000086288.11	0	0
protein_coding	AC010132.3	ENSG00000256646.7	0	0
protein_coding	MRPS24	ENSG00000062582.13	0	0
protein_coding	URGCP-MRPS24	ENSG00000270617.1	0	0
protein_coding	SPDYE1	ENSG00000136206.3	0	0
protein_coding	PGAM2	ENSG00000164708.5	0	0
protein_coding	MYL7	ENSG00000106631.8	0	0
protein_coding	MYO1G	ENSG00000136286.15	0	0
protein_coding	RAMP3	ENSG00000122679.8	0	0
protein_coding	AC096582.3	ENSG00000283247.2	0	0
protein_coding	CDC14C	ENSG00000218305.4	0	0
protein_coding	VWC2	ENSG00000188730.4	0	0
protein_coding	ZPBP	ENSG00000042813.7	0	0
protein_coding	SPATA48	ENSG00000164500.6	0	0
protein_coding	IKZF1	ENSG00000185811.18	0	0
protein_coding	DDC	ENSG00000132437.17	0	0
protein_coding	POM121L12	ENSG00000221900.5	0	0
protein_coding	VSTM2A	ENSG00000170419.10	0	0
protein_coding	AC092647.5	ENSG00000249773.3	0	0
protein_coding	ZNF479	ENSG00000185177.12	0	0
protein_coding	AC115220.1	ENSG00000241149.3	0	0
protein_coding	ZNF727	ENSG00000214652.5	0	0
protein_coding	ZNF735	ENSG00000223614.5	0	0
protein_coding	ZNF679	ENSG00000197123.9	0	0
protein_coding	AC068533.4	ENSG00000249319.2	0	0
protein_coding	AC027644.4	ENSG00000284461.2	0	0
protein_coding	CALN1	ENSG00000183166.10	0	0
protein_coding	TRIM74	ENSG00000155428.12	0	0
protein_coding	TRIM50	ENSG00000146755.10	0	0

protein_coding	NCF1	ENSG00000158517.13	0	0
protein_coding	CASTOR2	ENSG00000274070.1	0	0
protein_coding	TRIM73	ENSG00000178809.11	0	0
protein_coding	SPDYE5	ENSG00000170092.14	0	0
protein_coding	CCL24	ENSG00000106178.6	0	0
protein_coding	SPDYE16	ENSG00000185040.13	0	0
protein_coding	GNAT3	ENSG00000214415.3	0	0
protein_coding	GRM3	ENSG00000198822.10	0	0
protein_coding	ZNF804B	ENSG00000182348.6	0	0
protein_coding	TEX47	ENSG00000164645.2	0	0
protein_coding	FAM237B	ENSG00000283267.1	0	0
protein_coding	CYP51A1	ENSG00000001630.16	0	0
protein_coding	AC000120.2	ENSG00000285772.1	0	0
protein_coding	AC000120.3	ENSG00000285953.1	0	0
protein_coding	ERVW-1	ENSG00000242950.6	0	0
protein_coding	HEPACAM2	ENSG00000188175.9	0	0
protein_coding	CALCR	ENSG00000004948.14	0	0
protein_coding	PPP1R9A	ENSG00000158528.11	0	0
protein_coding	PON3	ENSG00000105852.10	0	0
protein_coding	ASB4	ENSG00000005981.12	0	0
protein_coding	TAC1	ENSG00000006128.11	0	0
protein_coding	OCM2	ENSG00000135175.5	0	0
protein_coding	BHLHA15	ENSG00000180535.3	0	0
protein_coding	NPTX2	ENSG00000106236.3	0	0
protein_coding	ATP5MF-	ENSG00000248919.7	0	0
protein_coding	AC005020.2	ENSG00000272647.3	0	0
protein_coding	CYP3A7	ENSG00000160870.13	0	0
protein_coding	CYP3A7-<br>CYP3A51P	ENSG00000282301.2	0	0
protein_coding	CYP3A4	ENSG00000160868.14	0	0
protein_coding	OR2AE1	ENSG00000244623.1	0	0
protein_coding	AZGP1	ENSG00000160862.12	0	0
protein_coding	STAG3	ENSG00000066923.17	0	0
protein_coding	PVRIG	ENSG00000213413.2	0	0
protein_coding	SPDYE3	ENSG00000214300.7	0	0
protein_coding	SAP25	ENSG00000205307.11	0	0
protein_coding	LRCH4	ENSG00000077454.15	0	0
protein_coding	ACTL6B	ENSG00000077080.9	0	0
protein_coding	EPO	ENSG00000130427.2	0	0
protein_coding	ACHE	ENSG00000087085.14	0	0
protein_coding	MUC17	ENSG00000169876.13	0	0
protein_coding	NAT16	ENSG00000167011.8	0	0
protein_coding	COL26A1	ENSG00000160963.13	0	0

protein_coding	SPDYE6	ENSG00000260097.2	0	0
protein_coding	RASA4B	ENSG00000170667.14	0	0
protein_coding	AC093668.2	ENSG00000272949.1	0	0
protein_coding	AC093668.3	ENSG00000284981.1	0	0
protein_coding	AC093668.1	ENSG00000270249.1	0	0
protein_coding	SPDYE2	ENSG00000205238.9	0	0
protein_coding	RASA4	ENSG00000105808.17	0	0
protein_coding	AC105052.1	ENSG00000205236.6	0	0
protein_coding	UPK3BL1	ENSG00000267368.1	0	0
protein_coding	AC105052.3	ENSG00000267645.5	0	0
protein_coding	SPDYE2B	ENSG00000173678.14	0	0
protein_coding	NFE4	ENSG00000230257.2	0	0
protein_coding	LHFPL3	ENSG00000187416.11	0	0
protein_coding	LAMB4	ENSG00000091128.12	0	0
protein_coding	FEZF1	ENSG00000128610.11	0	0
protein_coding	RNF133	ENSG00000188050.2	0	0
protein_coding	RNF148	ENSG00000235631.1	0	0
protein_coding	TAS2R16	ENSG00000128519.3	0	0
protein_coding	SLC13A1	ENSG00000081800.8	0	0
protein_coding	LMOD2	ENSG00000170807.11	0	0
protein_coding	HYAL4	ENSG00000106302.9	0	0
protein_coding	SPAM1	ENSG00000106304.15	0	0
protein_coding	TMEM229A	ENSG00000234224.2	0	0
protein_coding	SSU72P8	ENSG00000230268.3	0	0
protein_coding	C7orf77	ENSG00000275356.4	0	0
protein_coding	GRM8	ENSG00000179603.17	0	0
protein_coding	FSCN3	ENSG00000106328.9	0	0
protein_coding	PAX4	ENSG00000106331.15	0	0
protein_coding	OPN1SW	ENSG00000128617.2	0	0
protein_coding	AC011005.1	ENSG00000230626.4	0	0
protein_coding	TSPAN33	ENSG00000158457.5	0	0
protein_coding	SSMEM1	ENSG00000165120.4	0	0
protein_coding	CPA2	ENSG00000158516.11	0	0
protein_coding	CPA5	ENSG00000158525.15	0	0
protein_coding	CPA1	ENSG00000091704.9	0	0
protein_coding	TSGA13	ENSG00000213265.8	0	0
protein_coding	STRA8	ENSG00000146857.3	0	0
protein_coding	LUZP6	ENSG00000267697.1	0	0
protein_coding	AKR1D1	ENSG00000122787.14	0	0
protein_coding	C7orf55-LUC7L2	ENSG00000269955.2	0	0
protein_coding	CLEC2L	ENSG00000236279.6	0	0
protein_coding	RAB19	ENSG00000146955.10	0	0

protein_coding	TAS2R3	ENSG00000127362.2	0	0
protein_coding	TAS2R5	ENSG00000127366.5	0	0
protein_coding	PRSS37	ENSG00000165076.13	0	0
protein_coding	OR9A4	ENSG00000258083.2	0	0
protein_coding	CLEC5A	ENSG00000258227.6	0	0
protein_coding	TAS2R38	ENSG00000257138.1	0	0
protein_coding	MGAM2	ENSG00000257743.8	0	0
protein_coding	PRSS58	ENSG00000258223.6	0	0
protein_coding	PRSS1	ENSG00000204983.13	0	0
protein_coding	PRSS2	ENSG00000275896.5	0	0
protein_coding	TRPV6	ENSG00000165125.19	0	0
protein_coding	TRPV5	ENSG00000127412.6	0	0
protein_coding	LLCFC1	ENSG00000165131.6	0	0
protein_coding	OR9A2	ENSG00000179468.5	0	0
protein_coding	OR6V1	ENSG00000225781.1	0	0
protein_coding	TAS2R39	ENSG00000236398.2	0	0
protein_coding	TAS2R40	ENSG00000221937.4	0	0
protein_coding	CLCN1	ENSG00000188037.11	0	0
protein_coding	TAS2R60	ENSG00000185899.1	0	0
protein_coding	TAS2R41	ENSG00000221855.1	0	0
protein_coding	CTAGE15	ENSG00000271079.1	0	0
protein_coding	TCAF2C	ENSG00000283528.2	0	0
protein_coding	CTAGE6	ENSG00000271321.1	0	0
protein_coding	OR2F2	ENSG00000221910.2	0	0
protein_coding	OR2F1	ENSG00000213215.5	0	0
protein_coding	OR6B1	ENSG00000221813.4	0	0
protein_coding	OR2A5	ENSG00000221836.3	0	0
protein_coding	OR2A25	ENSG00000221933.3	0	0
protein_coding	OR2A12	ENSG00000221858.3	0	0
protein_coding	OR2A2	ENSG00000221989.2	0	0
protein_coding	OR2A14	ENSG00000221938.5	0	0
protein_coding	CTAGE4	ENSG00000225932.3	0	0
protein_coding	OR2A42	ENSG00000212807.2	0	0
protein_coding	CTAGE8	ENSG00000244693.1	0	0
protein_coding	OR2A1	ENSG00000221970.2	0	0
protein_coding	NOBOX	ENSG00000106410.15	0	0
protein_coding	C7orf33	ENSG00000170279.2	0	0
protein_coding	SSPO	ENSG00000197558.11	0	0
protein_coding	AC073111.3	ENSG00000284041.1	0	0
protein_coding	GIMAP1	ENSG00000213203.2	0	0
protein_coding	GIMAP1-GIMAP5	ENSG00000281887.3	0	0
protein_coding	GIMAP5	ENSG00000196329.11	0	0

protein_coding	TMEM176B	ENSG00000106565.17	0	0
protein_coding	TMEM176A	ENSG00000002933.8	0	0
protein_coding	KCNH2	ENSG00000055118.14	0	0
protein_coding	ATG9B	ENSG00000181652.19	0	0
protein_coding	GBX1	ENSG00000164900.4	0	0
protein_coding	ASB10	ENSG00000146926.10	0	0
protein_coding	IQCA1L	ENSG00000278685.4	0	0
protein_coding	AC021097.3	ENSG00000285480.1	0	0
protein_coding	AC021097.2	ENSG00000285292.1	0	0
protein_coding	WDR86	ENSG00000187260.15	0	0
protein_coding	CRYGN	ENSG00000127377.9	0	0
protein_coding	GALNTL5	ENSG00000106648.13	0	0
protein_coding	HTR5A	ENSG00000157219.4	0	0
protein_coding	VIPR2	ENSG00000106018.13	0	0
protein_coding	OR4F21	ENSG00000176269.3	0	0
protein_coding	DLGAP2	ENSG00000198010.12	0	0
protein_coding	KBTBD11-	ENSG00000283239.1	0	0
protein_coding	DEFA6	ENSG00000164822.4	0	0
protein_coding	DEFA4	ENSG00000164821.4	0	0
protein_coding	DEFA1	ENSG00000206047.2	0	0
protein_coding	DEFA1B	ENSG00000240247.7	0	0
protein_coding	DEFA3	ENSG00000239839.6	0	0
protein_coding	DEFA5	ENSG00000164816.7	0	0
protein_coding	USP17L1	ENSG00000230549.3	0	0
protein_coding	USP17L4	ENSG00000236125.3	0	0
protein_coding	ZNF705G	ENSG00000215372.6	0	0
protein_coding	DEFB4B	ENSG00000177257.2	0	0
protein_coding	DEFB103B	ENSG00000177243.3	0	0
protein_coding	SPAG11B	ENSG00000164871.17	0	0
protein_coding	DEFB104B	ENSG00000177023.2	0	0
protein_coding	DEFB106B	ENSG00000187082.2	0	0
protein_coding	DEFB105B	ENSG00000186599.7	0	0
protein_coding	DEFB107B	ENSG00000198129.2	0	0
protein_coding	PRR23D1	ENSG00000255251.1	0	0
protein_coding	AC134684.11	ENSG00000285975.1	0	0
protein_coding	AC134684.8	ENSG00000285687.1	0	0
protein_coding	AC134684.9	ENSG00000285765.1	0	0
protein_coding	AC084121.9	ENSG00000285814.1	0	0
protein_coding	AC084121.11	ENSG00000285913.1	0	0
protein_coding	AC084121.6	ENSG00000285620.1	0	0
protein_coding	AC084121.12	ENSG00000285937.1	0	0
protein_coding	AC084121.8	ENSG00000285720.1	0	0
protein_coding	AC084121.7	ENSG00000285657.1	0	0
protein_coding	AC084121.5	ENSG00000285607.1	0	0

protein_coding	AC084121.13	ENSG00000285950.1	0	0
protein_coding	PRR23D2	ENSG00000255378.1	0	0
protein_coding	DEFB107A	ENSG00000186572.2	0	0
protein_coding	DEFB105A	ENSG00000186562.7	0	0
protein_coding	DEFB106A	ENSG00000186579.2	0	0
protein_coding	DEFB104A	ENSG00000176782.2	0	0
protein_coding	SPAG11A	ENSG00000178287.18	0	0
protein_coding	DEFB103A	ENSG00000176797.3	0	0
protein_coding	DEFB4A	ENSG00000171711.2	0	0
protein_coding	ZNF705B	ENSG00000215356.4	0	0
protein_coding	USP17L8	ENSG00000237038.5	0	0
protein_coding	USP17L3	ENSG00000225327.3	0	0
protein_coding	PRSS51	ENSG00000253649.5	0	0
protein_coding	PRSS55	ENSG00000184647.10	0	0
protein_coding	PINX1	ENSG00000258724.1	0	0
protein_coding	SLC35G5	ENSG00000177710.5	0	0
protein_coding	BLK	ENSG00000136573.13	0	0
protein_coding	GATA4	ENSG00000136574.17	0	0
protein_coding	DEFB136	ENSG00000205884.2	0	0
protein_coding	DEFB135	ENSG00000205883.2	0	0
protein_coding	DEFB134	ENSG00000205882.8	0	0
protein_coding	DEFB130B	ENSG00000233050.1	0	0
protein_coding	ZNF705D	ENSG00000215343.7	0	0
protein_coding	USP17L7	ENSG00000226430.6	0	0
protein_coding	USP17L2	ENSG00000223443.2	0	0
protein_coding	FAM86B1	ENSG00000186523.14	0	0
protein_coding	DEFB130A	ENSG00000232948.1	0	0
protein_coding	FAM86B2	ENSG00000145002.12	0	0
protein_coding	MTMR7	ENSG00000003987.13	0	0
protein_coding	NAT2	ENSG00000156006.4	0	0
protein_coding	SLC18A1	ENSG00000036565.14	0	0
protein_coding	LGI3	ENSG00000168481.8	0	0
protein_coding	SFTPC	ENSG00000168484.12	0	0
protein_coding	AC037459.1	ENSG00000248235.6	0	0
protein_coding	PEBP4	ENSG00000134020.7	0	0
protein_coding	AC107959.5	ENSG00000284956.1	0	0
protein_coding	NKX2-6	ENSG00000180053.7	0	0
protein_coding	ADAMDEC1	ENSG00000134028.14	0	0
protein_coding	ADAM7	ENSG00000069206.15	0	0
protein_coding	EBF2	ENSG00000221818.8	0	0
protein_coding	ADRA1A	ENSG00000120907.17	0	0
protein_coding	STMN4	ENSG00000015592.16	0	0
protein_coding	CHRNA2	ENSG00000120903.12	0	0
protein_coding	NUGGC	ENSG00000189233.11	0	0

protein_coding	PNOC	ENSG00000168081.8	0	0
protein_coding	MBOAT4	ENSG00000177669.3	0	0
protein_coding	SMIM18	ENSG00000253457.2	0	0
protein_coding	GOT1L1	ENSG00000169154.6	0	0
protein_coding	AC144573.1	ENSG00000285880.1	0	0
protein_coding	ADRB3	ENSG00000188778.5	0	0
protein_coding	C8orf86	ENSG00000196166.4	0	0
protein_coding	ADAM2	ENSG00000104755.14	0	0
protein_coding	IDO2	ENSG00000188676.13	0	0
protein_coding	NKX6-3	ENSG00000165066.12	0	0
protein_coding	DKK¬†4	ENSG00000104371.4	0	0
protein_coding	CHRNB3	ENSG00000147432.6	0	0
protein_coding	CHRNA6	ENSG00000147434.8	0	0
protein_coding	AC110275.1	ENSG00000254673.1	0	0
protein_coding	PPDPFL	ENSG00000168333.13	0	0
protein_coding	PXDNL	ENSG00000147485.12	0	0
protein_coding	ST18	ENSG00000147488.11	0	0
protein_coding	ALKAL1	ENSG00000196711.8	0	0
protein_coding	NPBWR1	ENSG00000183729.3	0	0
protein_coding	OPRK1	ENSG00000082556.10	0	0
protein_coding	SOX17	ENSG00000164736.5	0	0
protein_coding	XKR4	ENSG00000206579.8	0	0
protein_coding	MOS	ENSG00000172680.1	0	0
protein_coding	CA8	ENSG00000178538.9	0	0
protein_coding	CLVS1	ENSG00000177182.10	0	0
protein_coding	NKAIN3	ENSG00000185942.11	0	0
protein_coding	BHLHE22	ENSG00000180828.2	0	0
protein_coding	CYP7B1	ENSG00000172817.3	0	0
protein_coding	DNAJC5B	ENSG00000147570.9	0	0
protein_coding	TRIM55	ENSG00000147573.16	0	0
protein_coding	CRH	ENSG00000147571.4	0	0
protein_coding	AC009879.2	ENSG00000285655.1	0	0
protein_coding	AC009879.3	ENSG00000285791.1	0	0
protein_coding	VXN	ENSG00000169085.12	0	0
protein_coding	PREX2	ENSG00000046889.18	0	0
protein_coding	PRDM14	ENSG00000147596.3	0	0
protein_coding	SBSPON	ENSG00000164764.10	0	0
protein_coding	C8orf89	ENSG00000274443.4	0	0
protein_coding	AC022826.2	ENSG00000258677.2	0	0
protein_coding	PI15	ENSG00000137558.8	0	0
protein_coding	STMN2	ENSG00000104435.13	0	0
protein_coding	AC036214.3	ENSG00000276418.5	0	0
protein_coding	PMP2	ENSG00000147588.6	0	0
protein_coding	FABP9	ENSG00000205186.2	0	0

protein_coding	CA1	ENSG00000133742.13	0	0
protein_coding	CA3	ENSG00000164879.6	0	0
protein_coding	PSKH2	ENSG00000147613.7	0	0
protein_coding	SLC7A13	ENSG00000164893.8	0	0
protein_coding	CNBD1	ENSG00000176571.11	0	0
protein_coding	SLC26A7	ENSG00000147606.8	0	0
protein_coding	CDH17	ENSG00000079112.9	0	0
protein_coding	GDF6	ENSG00000156466.9	0	0
protein_coding	KCNS2	ENSG00000156486.7	0	0
protein_coding	RGS22	ENSG00000132554.19	0	0
protein_coding	FBXO43	ENSG00000156509.13	0	0
protein_coding	AC012213.5	ENSG00000285982.1	0	0
protein_coding	ABRA	ENSG00000174429.3	0	0
protein_coding	TMEM74	ENSG00000164841.4	0	0
protein_coding	TRHR	ENSG00000174417.2	0	0
protein_coding	PKHD1L1	ENSG00000205038.11	0	0
protein_coding	KCNV1	ENSG00000164794.8	0	0
protein_coding	SLC30A8	ENSG00000164756.12	0	0
protein_coding	KLHL38	ENSG00000175946.8	0	0
protein_coding	ADCY8	ENSG00000155897.9	0	0
protein_coding	OC90	ENSG00000253117.4	0	0
protein_coding	AC100868.1	ENSG00000258417.3	0	0
protein_coding	HHLA1	ENSG00000132297.11	0	0
protein_coding	COL22A1	ENSG00000169436.16	0	0
protein_coding	AC138647.1	ENSG00000226490.2	0	0
protein_coding	ADGRB1	ENSG00000181790.11	0	0
protein_coding	LYPD2	ENSG00000197353.3	0	0
protein_coding	LYNX1-	ENSG00000284505.1	0	0
protein_coding	SLURP2	ENSG00000283992.1	0	0
protein_coding	GML	ENSG00000104499.6	0	0
protein_coding	CYP11B1	ENSG00000160882.11	0	0
protein_coding	CYP11B2	ENSG00000179142.2	0	0
protein_coding	LY6L	ENSG00000261667.1	0	0
protein_coding	LY6H	ENSG00000176956.12	0	0
protein_coding	GPIHBP1	ENSG00000277494.1	0	0
protein_coding	AC138696.1	ENSG00000264668.1	0	0
protein_coding	CCDC166	ENSG00000255181.3	0	0
protein_coding	MAPK15	ENSG00000181085.14	0	0
protein_coding	SPATC1	ENSG00000186583.11	0	0
protein_coding	WDR97	ENSG00000179698.13	0	0
protein_coding	SCRT1	ENSG00000261678.2	0	0
protein_coding	TMEM249	ENSG00000261587.2	0	0
protein_coding	AC233992.2	ENSG00000271698.1	0	0
protein_coding	FOXH1	ENSG00000160973.7	0	0

protein_coding	GPT	ENSG00000167701.13	0	0
protein_coding	DMRT1	ENSG00000137090.11	0	0
protein_coding	DMRT3	ENSG00000064218.4	0	0
protein_coding	KCNV2	ENSG00000168263.8	0	0
protein_coding	INSL6	ENSG00000120210.7	0	0
protein_coding	MLANA	ENSG00000120215.9	0	0
protein_coding	TPD52L3	ENSG00000170777.10	0	0
protein_coding	CER1	ENSG00000147869.4	0	0
protein_coding	C9orf92	ENSG00000205549.9	0	0
protein_coding	SH3GL2	ENSG00000107295.9	0	0
protein_coding	SLC24A2	ENSG00000155886.11	0	0
protein_coding	IFNB1	ENSG00000171855.6	0	0
protein_coding	IFNW1	ENSG00000177047.6	0	0
protein_coding	IFNA21	ENSG00000137080.4	0	0
protein_coding	IFNA4	ENSG00000236637.4	0	0
protein_coding	IFNA7	ENSG00000214042.1	0	0
protein_coding	IFNA10	ENSG00000186803.3	0	0
protein_coding	IFNA16	ENSG00000147885.4	0	0
protein_coding	IFNA17	ENSG00000234829.4	0	0
protein_coding	IFNA14	ENSG00000228083.2	0	0
protein_coding	IFNA5	ENSG00000147873.5	0	0
protein_coding	IFNA6	ENSG00000120235.4	0	0
protein_coding	IFNA13	ENSG00000233816.3	0	0
protein_coding	IFNA2	ENSG00000188379.6	0	0
protein_coding	IFNA8	ENSG00000120242.3	0	0
protein_coding	IFNA1	ENSG00000197919.5	0	0
protein_coding	AL359922.1	ENSG00000264545.1	0	0
protein_coding	IZUMO3	ENSG00000205442.12	0	0
protein_coding	LRRC19	ENSG00000184434.7	0	0
protein_coding	TEK	ENSG00000120156.20	0	0
protein_coding	EQTN	ENSG00000120160.10	0	0
protein_coding	IFNK	ENSG00000147896.3	0	0
protein_coding	TAF1L	ENSG00000122728.6	0	0
protein_coding	TMEM215	ENSG00000188133.5	0	0
protein_coding	AQP7	ENSG00000165269.12	0	0
protein_coding	DNAI1	ENSG00000122735.15	0	0
protein_coding	CNTFR	ENSG00000122756.14	0	0
protein_coding	ARID3C	ENSG00000205143.2	0	0
protein_coding	AL162231.3	ENSG00000258728.1	0	0
protein_coding	CCL27	ENSG00000213927.3	0	0
protein_coding	AL162231.1	ENSG00000187186.14	0	0
protein_coding	CCL19	ENSG00000172724.11	0	0
protein_coding	CCL21	ENSG00000137077.7	0	0
protein_coding	FAM205A	ENSG00000205108.5	0	0

protein_coding	FAM205C	ENSG00000187791.12	0	0
protein_coding	C9orf131	ENSG00000174038.12	0	0
protein_coding	SIT¬†1	ENSG00000137078.8	0	0
protein_coding	MSMP	ENSG00000215183.4	0	0
protein_coding	AL133410.3	ENSG00000285645.1	0	0
protein_coding	FAM221B	ENSG00000204930.9	0	0
protein_coding	OR13J1	ENSG00000168828.5	0	0
protein_coding	OR2S2	ENSG00000278889.4	0	0
protein_coding	AL513165.2	ENSG00000256966.6	0	0
protein_coding	AL138752.2	ENSG00000255872.3	0	0
protein_coding	FRMPD1	ENSG00000070601.9	0	0
protein_coding	IGFBPL1	ENSG00000137142.4	0	0
protein_coding	FAM95C	ENSG00000283486.2	0	0
protein_coding	FAM240B	ENSG00000283329.1	0	0
protein_coding	SPATA31A1	ENSG00000204849.7	0	0
protein_coding	ANKRD20A2	ENSG00000183148.6	0	0
protein_coding	FOXD4L6	ENSG00000273514.1	0	0
protein_coding	SPATA31A6	ENSG00000185775.9	0	0
protein_coding	SPATA31A5	ENSG00000276581.2	0	0
protein_coding	SPATA31A7	ENSG00000276040.4	0	0
protein_coding	CNTNAP3C	ENSG00000283378.1	0	0
protein_coding	ANKRD20A4	ENSG00000172014.12	0	0
protein_coding	FOXD4L5	ENSG00000204779.2	0	0
protein_coding	FOXD4L4	ENSG00000184659.5	0	0
protein_coding	ANKRD20A3	ENSG00000276203.4	0	0
protein_coding	SPATA31A3	ENSG00000275969.2	0	0
protein_coding	ANKRD20A1	ENSG00000260691.5	0	0
protein_coding	FOXD4L3	ENSG00000187559.5	0	0
protein_coding	TMEM252	ENSG00000181778.4	0	0
protein_coding	PIP5K1B	ENSG00000107242.17	0	0
protein_coding	PRKACG	ENSG00000165059.7	0	0
protein_coding	AL358113.1	ENSG00000285130.1	0	0
protein_coding	C9orf135	ENSG00000204711.8	0	0
protein_coding	C9orf57	ENSG00000204669.9	0	0
protein_coding	FOXB2	ENSG00000204612.1	0	0
protein_coding	SPATA31D4	ENSG00000189357.8	0	0
protein_coding	SPATA31D3	ENSG00000186788.13	0	0
protein_coding	SPATA31D1	ENSG00000214929.3	0	0
protein_coding	SPATA31E1	ENSG00000177992.9	0	0
protein_coding	AL353572.3	ENSG00000283205.1	0	0
protein_coding	DIRAS2	ENSG00000165023.6	0	0
protein_coding	WNK2	ENSG00000165238.16	0	0
protein_coding	C9orf129	ENSG00000204352.3	0	0
protein_coding	NUTM2F	ENSG00000130950.13	0	0

protein_coding	FBP2	ENSG00000130957.4	0	0
protein_coding	AL160269.1	ENSG00000285269.2	0	0
protein_coding	NUTM2G	ENSG00000188152.12	0	0
protein_coding	MSANTD3-TMEFF1	ENSG00000251349.3	0	0
protein_coding	TMEFF1	ENSG00000241697.4	0	0
protein_coding	BAAT	ENSG00000136881.11	0	0
protein_coding	ALDOB	ENSG00000136872.18	0	0
protein_coding	GRIN3A	ENSG00000198785.4	0	0
protein_coding	PPP3R2	ENSG00000188386.6	0	0
protein_coding	CYLC2	ENSG00000155833.14	0	0
protein_coding	OR13F1	ENSG00000186881.3	0	0
protein_coding	OR13C4	ENSG00000148136.5	0	0
protein_coding	OR13C3	ENSG00000204246.3	0	0
protein_coding	OR13C8	ENSG00000186943.1	0	0
protein_coding	OR13C5	ENSG00000277556.1	0	0
protein_coding	OR13C2	ENSG00000276119.1	0	0
protein_coding	OR13C9	ENSG00000136839.1	0	0
protein_coding	OR13D1	ENSG00000179055.7	0	0
protein_coding	TAL2	ENSG00000186051.6	0	0
protein_coding	ACTL7B	ENSG00000148156.7	0	0
protein_coding	ACTL7A	ENSG00000187003.6	0	0
protein_coding	PALM2-	ENSG00000157654.17	0	0
protein_coding	AKAP2	ENSG00000241978.9	0	0
protein_coding	C9orf152	ENSG00000188959.9	0	0
protein_coding	TXNDC8	ENSG00000204193.10	0	0
protein_coding	OR2K2	ENSG00000171133.3	0	0
protein_coding	DNAJC25-GNG10	ENSG00000244115.1	0	0
protein_coding	GNG10	ENSG00000242616.3	0	0
protein_coding	ZNF883	ENSG00000285447.1	0	0
protein_coding	RNF183	ENSG00000165188.13	0	0
protein_coding	BSPRY	ENSG00000119411.10	0	0
protein_coding	AMBP	ENSG00000106927.11	0	0
protein_coding	KIF12	ENSG00000136883.14	0	0
protein_coding	ORM2	ENSG00000228278.3	0	0
protein_coding	TEX53	ENSG00000230054.2	0	0
protein_coding	TEX48	ENSG00000230601.6	0	0
protein_coding	TNFSF8	ENSG00000106952.7	0	0
protein_coding	AL160272.2	ENSG00000285082.1	0	0
protein_coding	MORN5	ENSG00000185681.12	0	0
protein_coding	LHX6	ENSG00000106852.15	0	0
protein_coding	OR1J1	ENSG00000136834.3	0	0

protein_coding	OR1J2	ENSG00000197233.7	0	0
protein_coding	OR1J4	ENSG00000239590.1	0	0
protein_coding	OR1N1	ENSG00000171505.5	0	0
protein_coding	OR1L8	ENSG00000171496.4	0	0
protein_coding	OR1B1	ENSG00000280094.2	0	0
protein_coding	OR1L1	ENSG00000173679.2	0	0
protein_coding	OR1L3	ENSG00000171481.4	0	0
protein_coding	OR1L4	ENSG00000136939.1	0	0
protein_coding	OR1L6	ENSG00000171459.4	0	0
protein_coding	OR5C1	ENSG00000148215.4	0	0
protein_coding	OR1K1	ENSG00000165204.3	0	0
protein_coding	GPR21	ENSG00000188394.6	0	0
protein_coding	ADGRD2	ENSG00000180264.11	0	0
protein_coding	NR5A1	ENSG00000136931.9	0	0
protein_coding	WDR38	ENSG00000136918.7	0	0
protein_coding	CFAP157	ENSG00000160401.14	0	0
protein_coding	TTC16	ENSG00000167094.15	0	0
protein_coding	AL157935.2	ENSG00000257524.6	0	0
protein_coding	AL672142.1	ENSG00000251184.1	0	0
protein_coding	C9orf50	ENSG00000179058.6	0	0
protein_coding	HMCN2	ENSG00000148357.16	0	0
protein_coding	QRFP	ENSG00000188710.2	0	0
protein_coding	PRRT1B	ENSG00000283526.1	0	0
protein_coding	CFAP77	ENSG00000188523.8	0	0
protein_coding	BARHL1	ENSG00000125492.9	0	0
protein_coding	AL162417.1	ENSG00000285245.1	0	0
protein_coding	OBP2B	ENSG00000171102.14	0	0
protein_coding	MYMK	ENSG00000187616.4	0	0
protein_coding	ADAMTSL2	ENSG00000197859.9	0	0
protein_coding	FAM163B	ENSG00000196990.8	0	0
protein_coding	DBH	ENSG00000123454.11	0	0
protein_coding	FCN2	ENSG00000160339.15	0	0
protein_coding	FCN1	ENSG00000085265.10	0	0
protein_coding	LCN1	ENSG00000160349.9	0	0
protein_coding	OBP2A	ENSG00000122136.13	0	0
protein_coding	PAEP	ENSG00000122133.16	0	0
protein_coding	AL354761.1	ENSG00000236543.2	0	0
protein_coding	GLT6D1	ENSG00000204007.6	0	0
protein_coding	LCN9	ENSG00000148386.9	0	0
protein_coding	SOHLH1	ENSG00000165643.10	0	0
protein_coding	KCNT1	ENSG00000107147.12	0	0
protein_coding	LHX3	ENSG00000107187.16	0	0
protein_coding	DNLZ	ENSG00000213221.4	0	0
protein_coding	LCN10	ENSG00000187922.13	0	0

protein_coding	AL355987.1	ENSG00000204003.8	0	0
protein_coding	LCN6	ENSG00000267206.5	0	0
protein_coding	LCN8	ENSG00000204001.9	0	0
protein_coding	LCN15	ENSG00000177984.6	0	0
protein_coding	AL355987.3	ENSG00000272896.1	0	0
protein_coding	PTGDS	ENSG00000107317.12	0	0
protein_coding	AL807752.7	ENSG00000284341.1	0	0
protein_coding	LCNL1	ENSG00000214402.6	0	0
protein_coding	FUT7	ENSG00000180549.7	0	0
protein_coding	AL807752.6	ENSG00000279073.3	0	0
protein_coding	GRIN1	ENSG00000176884.14	0	0
protein_coding	LRRC26	ENSG00000184709.7	0	0
protein_coding	AL929554.1	ENSG00000261793.1	0	0
protein_coding	TMEM210	ENSG00000185863.7	0	0
protein_coding	SLC34A3	ENSG00000198569.9	0	0
protein_coding	FAM166A	ENSG00000188163.7	0	0
protein_coding	STPG3	ENSG00000197768.10	0	0
protein_coding	ENTPD8	ENSG00000188833.9	0	0
protein_coding	CACNA1B	ENSG00000148408.12	0	0
protein_coding	TUBB8	ENSG00000261456.5	0	0
protein_coding	PRR26	ENSG00000180525.11	0	0
protein_coding	IDI2	ENSG00000148377.5	0	0
protein_coding	AKR1C8P	ENSG00000264006.8	0	0
protein_coding	UCN3	ENSG00000178473.6	0	0
protein_coding	CALML3	ENSG00000178363.4	0	0
protein_coding	IL2RA	ENSG00000134460.17	0	0
protein_coding	ITIH2	ENSG00000151655.18	0	0
protein_coding	UCMA	ENSG00000165623.9	0	0
protein_coding	AL157392.5	ENSG00000282246.1	0	0
protein_coding	C1QL3	ENSG00000165985.9	0	0
protein_coding	ST8SIA6	ENSG00000148488.16	0	0
protein_coding	MRC1	ENSG00000260314.2	0	0
protein_coding	SLC39A12	ENSG00000148482.11	0	0
protein_coding	C10orf113	ENSG00000204683.11	0	0
protein_coding	EBLN1	ENSG00000223601.2	0	0
protein_coding	COMMD3-	ENSG00000269897.5	0	0
protein_coding	SPAG6	ENSG00000077327.15	0	0
protein_coding	ARMC3	ENSG00000165309.13	0	0
protein_coding	PTF1A	ENSG00000168267.6	0	0
protein_coding	MYO3A	ENSG00000095777.15	0	0
protein_coding	GAD2	ENSG00000136750.12	0	0
protein_coding	LYZL1	ENSG00000120563.9	0	0
protein_coding	LYZL2	ENSG00000151033.9	0	0
protein_coding	GJD4	ENSG00000177291.3	0	0

protein_coding	ANKRD30A	ENSG00000148513.17	0	0
protein_coding	C10orf142	ENSG00000277288.4	0	0
protein_coding	TMEM72	ENSG00000187783.11	0	0
protein_coding	OR13A1	ENSG00000256574.7	0	0
protein_coding	ALOX5	ENSG00000012779.10	0	0
protein_coding	ANTXRL	ENSG00000274209.4	0	0
protein_coding	NPY4R	ENSG00000204174.7	0	0
protein_coding	GPRIN2	ENSG00000204175.5	0	0
protein_coding	SYT15	ENSG00000204176.13	0	0
protein_coding	GDF10	ENSG00000266524.2	0	0
protein_coding	GDF2	ENSG00000263761.2	0	0
protein_coding	RBP3	ENSG00000265203.1	0	0
protein_coding	FAM25G	ENSG00000189090.7	0	0
protein_coding	FO681492.1	ENSG00000277758.4	0	0
protein_coding	NPY4R2	ENSG00000264717.5	0	0
protein_coding	FAM25C	ENSG00000276430.2	0	0
protein_coding	FRMPD2	ENSG00000170324.20	0	0
protein_coding	WDFY4	ENSG00000128815.19	0	0
protein_coding	LRRC18	ENSG00000165383.11	0	0
protein_coding	FAM170B	ENSG00000172538.6	0	0
protein_coding	TMEM273	ENSG00000204161.13	0	0
protein_coding	C10orf71	ENSG00000177354.11	0	0
protein_coding	DRGX	ENSG00000165606.8	0	0
protein_coding	CHAT	ENSG00000070748.18	0	0
protein_coding	SLC18A3	ENSG00000187714.6	0	0
protein_coding	C10orf53	ENSG00000178645.12	0	0
protein_coding	A1CF	ENSG00000148584.14	0	0
protein_coding	MBL2	ENSG00000165471.6	0	0
protein_coding	MTRNR2L5	ENSG00000249860.3	0	0
protein_coding	MRLN	ENSG00000227877.6	0	0
protein_coding	AC067752.1	ENSG00000285551.1	0	0
protein_coding	CTNNA3	ENSG00000183230.16	0	0
protein_coding	LRRTM3	ENSG00000198739.10	0	0
protein_coding	TACR2	ENSG00000075073.14	0	0
protein_coding	NEUROG3	ENSG00000122859.4	0	0
protein_coding	NODAL	ENSG00000156574.9	0	0
protein_coding	PRF1	ENSG00000180644.7	0	0
protein_coding	TBATA	ENSG00000166220.12	0	0
protein_coding	OIT3	ENSG00000138315.12	0	0
protein_coding	PLA2G12B	ENSG00000138308.5	0	0
protein_coding	SYNPO2L	ENSG00000166317.11	0	0
protein_coding	AGAP5	ENSG00000172650.13	0	0
protein_coding	AC022400.6	ENSG00000272916.5	0	0
protein_coding	DUPD1	ENSG00000188716.5	0	0

protein_coding	SFTPA2	ENSG00000185303.16	0	0
protein_coding	SFTPA1	ENSG00000122852.14	0	0
protein_coding	NUTM2B	ENSG00000188199.10	0	0
protein_coding	NUTM2E	ENSG00000228570.7	0	0
protein_coding	SFTPD	ENSG00000133661.15	0	0
protein_coding	DYDC1	ENSG00000170788.13	0	0
protein_coding	SH2D4B	ENSG00000178217.14	0	0
protein_coding	NRG3	ENSG00000185737.12	0	0
protein_coding	CDHR1	ENSG00000148600.14	0	0
protein_coding	LRIT2	ENSG00000204033.9	0	0
protein_coding	LRIT1	ENSG00000148602.5	0	0
protein_coding	RGR	ENSG00000148604.13	0	0
protein_coding	GRID1	ENSG00000182771.18	0	0
protein_coding	OPN4	ENSG00000122375.11	0	0
protein_coding	FAM25A	ENSG00000188100.8	0	0
protein_coding	NUTM2A	ENSG00000184923.12	0	0
protein_coding	NUTM2D	ENSG00000214562.14	0	0
protein_coding	LIPJ	ENSG00000204022.9	0	0
protein_coding	LIPF	ENSG00000182333.14	0	0
protein_coding	LIPK	ENSG00000204021.3	0	0
protein_coding	LIPN	ENSG00000204020.5	0	0
protein_coding	LIPM	ENSG00000173239.13	0	0
protein_coding	SLC16A12	ENSG00000152779.13	0	0
protein_coding	CYP26C1	ENSG00000187553.8	0	0
protein_coding	CYP26A1	ENSG00000095596.11	0	0
protein_coding	FFAR4	ENSG00000186188.10	0	0
protein_coding	RBP4	ENSG00000138207.13	0	0
protein_coding	PDE6C	ENSG00000095464.9	0	0
protein_coding	LGI1	ENSG00000108231.12	0	0
protein_coding	CYP2C18	ENSG00000108242.12	0	0
protein_coding	AL583836.1	ENSG00000276490.1	0	0
protein_coding	CYP2C19	ENSG00000165841.10	0	0
protein_coding	CYP2C9	ENSG00000138109.10	0	0
protein_coding	ACSM6	ENSG00000173124.14	0	0
protein_coding	AL365273.2	ENSG00000270099.1	0	0
protein_coding	DNTT	ENSG00000107447.7	0	0
protein_coding	OPALIN	ENSG00000197430.10	0	0
protein_coding	PIK3AP1	ENSG00000155629.14	0	0
protein_coding	SLIT1	ENSG00000187122.16	0	0
protein_coding	ARHGAP19-SLIT1	ENSG00000269891.2	0	0
protein_coding	AL355315.1	ENSG00000249967.1	0	0
protein_coding	C10orf62	ENSG00000203942.4	0	0

protein_coding	SFRP5	ENSG00000120057.4	0	0
protein_coding	CRTAC1	ENSG00000095713.13	0	0
protein_coding	HPSE2	ENSG00000172987.12	0	0
protein_coding	NKX2-3	ENSG00000119919.10	0	0
protein_coding	AL133353.2	ENSG00000285932.1	0	0
protein_coding	CPN1	ENSG00000120054.11	0	0
protein_coding	PKD2L1	ENSG00000107593.16	0	0
protein_coding	WNT8B	ENSG00000075290.7	0	0
protein_coding	AL133352.1	ENSG00000255339.6	0	0
protein_coding	PAX2	ENSG00000075891.21	0	0
protein_coding	TLX1	ENSG00000107807.12	0	0
protein_coding	LBX1	ENSG00000138136.6	0	0
protein_coding	FGF8	ENSG00000107831.12	0	0
protein_coding	CYP17A1	ENSG00000148795.6	0	0
protein_coding	BORCS7-	ENSG00000270316.1	0	0
protein_coding	AS3MT	ENSG00000214435.7	0	0
protein_coding	CALHM1	ENSG00000185933.6	0	0
protein_coding	SORCS3	ENSG00000156395.12	0	0
protein_coding	TECTB	ENSG00000119913.5	0	0
protein_coding	HABP2	ENSG00000148702.14	0	0
protein_coding	NRAP	ENSG00000197893.13	0	0
protein_coding	CCDC172	ENSG00000182645.5	0	0
protein_coding	PNLIP	ENSG00000175535.6	0	0
protein_coding	KCNK18	ENSG00000186795.1	0	0
protein_coding	PRLHR	ENSG00000119973.5	0	0
protein_coding	ARMS2	ENSG00000254636.1	0	0
protein_coding	DMBT1	ENSG00000187908.17	0	0
protein_coding	C10orf120	ENSG00000183559.11	0	0
protein_coding	FAM24A	ENSG00000203795.2	0	0
protein_coding	HMX3	ENSG00000188620.9	0	0
protein_coding	HMX2	ENSG00000188816.3	0	0
protein_coding	GPR26	ENSG00000154478.3	0	0
protein_coding	TEX36	ENSG00000175018.12	0	0
protein_coding	MMP21	ENSG00000154485.4	0	0
protein_coding	NPS	ENSG00000214285.2	0	0
protein_coding	FOXI2	ENSG00000186766.7	0	0
protein_coding	CLRN3	ENSG00000180745.4	0	0
protein_coding	TCERG1L	ENSG00000176769.9	0	0
protein_coding	NKX6-2	ENSG00000148826.8	0	0
protein_coding	ADGRA1	ENSG00000197177.15	0	0
protein_coding	UTF1	ENSG00000171794.3	0	0
protein_coding	VENTX	ENSG00000151650.7	0	0
protein_coding	ZNF511-	ENSG00000283496.1	0	0
protein_coding	CALY	ENSG00000130643.8	0	0

protein_coding	PRAP1	ENSG00000165828.14	0	0
protein_coding	AL360181.3	ENSG00000254536.1	0	0
protein_coding	FRG2B	ENSG00000225899.7	0	0
protein_coding	SCGB1C1	ENSG00000188076.2	0	0
protein_coding	ODF3	ENSG00000177947.13	0	0
protein_coding	NLRP6	ENSG00000174885.12	0	0
protein_coding	IFITM5	ENSG00000206013.2	0	0
protein_coding	CDHR5	ENSG00000099834.18	0	0
protein_coding	SCT	ENSG00000070031.3	0	0
protein_coding	MUC6	ENSG00000184956.15	0	0
protein_coding	MUC5AC	ENSG00000215182.8	0	0
protein_coding	MUC5B	ENSG00000117983.17	0	0
protein_coding	KRTAP5-1	ENSG00000205869.2	0	0
protein_coding	KRTAP5-3	ENSG00000196224.7	0	0
protein_coding	KRTAP5-4	ENSG00000241598.5	0	0
protein_coding	KRTAP5-5	ENSG00000185940.10	0	0
protein_coding	KRTAP5-6	ENSG00000205864.1	0	0
protein_coding	PRR33	ENSG00000283787.1	0	0
protein_coding	IGF2	ENSG00000167244.19	0	0
protein_coding	IGF2	ENSG00000284779.1	0	0
protein_coding	INS-IGF2	ENSG00000129965.15	0	0
protein_coding	INS	ENSG00000254647.6	0	0
protein_coding	C11orf21	ENSG00000110665.11	0	0
protein_coding	TSPAN32	ENSG00000064201.15	0	0
protein_coding	TRPM5	ENSG00000070985.13	0	0
protein_coding	KCNQ1	ENSG00000053918.16	0	0
protein_coding	MRGPRG	ENSG00000182170.3	0	0
protein_coding	MRGPRE	ENSG00000184350.9	0	0
protein_coding	ART1	ENSG00000129744.2	0	0
protein_coding	SSU72P5	ENSG00000284018.1	0	0
protein_coding	SSU72P2	ENSG00000284306.1	0	0
protein_coding	SSU72P4	ENSG00000283873.1	0	0
protein_coding	SSU72P3	ENSG00000284546.1	0	0
protein_coding	SSU72P7	ENSG00000284438.1	0	0
protein_coding	OR52B4	ENSG00000221996.6	0	0
protein_coding	OR52K2	ENSG00000181963.5	0	0
protein_coding	OR52K1	ENSG00000196778.3	0	0
protein_coding	OR52M1	ENSG00000197790.1	0	0
protein_coding	C11orf40	ENSG00000171987.1	0	0
protein_coding	OR52I2	ENSG00000226288.2	0	0
protein_coding	OR51E1	ENSG00000180785.9	0	0
protein_coding	OR51E2	ENSG00000167332.8	0	0
protein_coding	OR51C1P	ENSG00000197674.7	0	0
protein_coding	MMP26	ENSG00000167346.7	0	0

protein_coding	OR51F1	ENSG00000280021.1	0	0
protein_coding	OR52R1	ENSG00000279270.2	0	0
protein_coding	OR51F2	ENSG00000176925.7	0	0
protein_coding	OR51S1	ENSG00000176922.4	0	0
protein_coding	OR51T1	ENSG00000176900.2	0	0
protein_coding	OR51A7	ENSG00000176895.9	0	0
protein_coding	OR51G2	ENSG00000176893.5	0	0
protein_coding	OR51G1	ENSG00000278870.2	0	0
protein_coding	OR51A4	ENSG00000205497.4	0	0
protein_coding	OR51A2	ENSG00000205496.1	0	0
protein_coding	OR51L1	ENSG00000176798.2	0	0
protein_coding	OR52J3	ENSG00000205495.1	0	0
protein_coding	OR52E2	ENSG00000176787.2	0	0
protein_coding	OR52A5	ENSG00000171944.2	0	0
protein_coding	OR52A1	ENSG00000182070.5	0	0
protein_coding	OR51V1	ENSG00000176742.2	0	0
protein_coding	HBG1	ENSG00000213934.8	0	0
protein_coding	AC104389.5	ENSG00000284931.1	0	0
protein_coding	HBE1	ENSG00000213931.6	0	0
protein_coding	OR51B4	ENSG00000183251.4	0	0
protein_coding	OR51B2	ENSG00000279012.2	0	0
protein_coding	OR51B6	ENSG00000176239.7	0	0
protein_coding	OR51M1	ENSG00000184698.5	0	0
protein_coding	OR51Q1	ENSG00000167360.6	0	0
protein_coding	OR51I1	ENSG00000167359.8	0	0
protein_coding	OR51I2	ENSG00000187918.5	0	0
protein_coding	OR52D1	ENSG00000181609.5	0	0
protein_coding	UBQLN3	ENSG00000175520.8	0	0
protein_coding	OR52H1	ENSG00000181616.9	0	0
protein_coding	OR52B6	ENSG00000187747.2	0	0
protein_coding	TRIM6-	ENSG00000258588.3	0	0
protein_coding	OR56B1	ENSG00000181023.7	0	0
protein_coding	OR52N4	ENSG00000181074.4	0	0
protein_coding	OR52N5	ENSG00000181009.5	0	0
protein_coding	OR52N1	ENSG00000181001.2	0	0
protein_coding	OR52N2	ENSG00000180988.2	0	0
protein_coding	OR52E6	ENSG00000205409.3	0	0
protein_coding	OR52E8	ENSG00000183269.5	0	0
protein_coding	OR52E4	ENSG00000180974.4	0	0
protein_coding	OR52E5	ENSG00000277932.2	0	0
protein_coding	OR56A3	ENSG00000184478.7	0	0
protein_coding	OR56A5	ENSG00000188691.5	0	0
protein_coding	OR52L1	ENSG00000183313.4	0	0
protein_coding	OR56A4	ENSG00000183389.5	0	0

protein_coding	OR56A1	ENSG00000180934.6	0	0
protein_coding	OR56B4	ENSG00000180919.3	0	0
protein_coding	OR52B2	ENSG00000255307.1	0	0
protein_coding	OR52W1	ENSG00000175485.2	0	0
protein_coding	C11orf42	ENSG00000180878.2	0	0
protein_coding	CCKBR	ENSG00000110148.9	0	0
protein_coding	AC084337.2	ENSG00000283977.1	0	0
protein_coding	OR2AG1	ENSG00000279486.5	0	0
protein_coding	OR6A2	ENSG00000184933.5	0	0
protein_coding	OR10A5	ENSG00000166363.5	0	0
protein_coding	OR10A2	ENSG00000170790.5	0	0
protein_coding	OR10A4	ENSG00000170782.3	0	0
protein_coding	OR2D2	ENSG00000166368.2	0	0
protein_coding	OR2D3	ENSG00000178358.4	0	0
protein_coding	NLRP14	ENSG00000158077.4	0	0
protein_coding	SYT9	ENSG00000170743.16	0	0
protein_coding	OVCH2	ENSG00000183378.11	0	0
protein_coding	OR5P3	ENSG00000182334.2	0	0
protein_coding	OR10A3	ENSG00000170683.6	0	0
protein_coding	STK33	ENSG00000130413.15	0	0
protein_coding	C11orf16	ENSG00000176029.13	0	0
protein_coding	ASCL3	ENSG00000176009.3	0	0
protein_coding	LYVE1	ENSG00000133800.8	0	0
protein_coding	PTH	ENSG00000152266.6	0	0
protein_coding	AC018523.2	ENSG00000256206.2	0	0
protein_coding	CALCB	ENSG00000175868.13	0	0
protein_coding	CALCA	ENSG00000110680.12	0	0
protein_coding	KCNJ11	ENSG00000187486.5	0	0
protein_coding	ABCC8	ENSG00000006071.13	0	0
protein_coding	USH1C	ENSG00000006611.15	0	0
protein_coding	OTOG	ENSG00000188162.10	0	0
protein_coding	MYOD1	ENSG00000129152.3	0	0
protein_coding	MRGPRX3	ENSG00000179826.6	0	0
protein_coding	MRGPRX4	ENSG00000179817.5	0	0
protein_coding	SAA4	ENSG00000148965.9	0	0
protein_coding	SAA2-SAA4	ENSG00000255071.3	0	0
protein_coding	LDHC	ENSG00000166796.11	0	0
protein_coding	LDHAL6A	ENSG00000166800.9	0	0
protein_coding	IGSF22	ENSG00000179057.13	0	0
protein_coding	PTPN5	ENSG00000110786.17	0	0
protein_coding	MRGPRX1	ENSG00000170255.7	0	0
protein_coding	MRGPRX2	ENSG00000183695.2	0	0
protein_coding	DBX1	ENSG00000109851.6	0	0
protein_coding	SLC6A5	ENSG00000165970.11	0	0

protein_coding	NELL1	ENSG00000165973.18	0	0
protein_coding	SLC17A6	ENSG00000091664.8	0	0
protein_coding	CCDC179	ENSG00000255359.2	0	0
protein_coding	KCNA4	ENSG00000182255.6	0	0
protein_coding	FSHB	ENSG00000131808.10	0	0
protein_coding	DCDC1	ENSG00000170959.14	0	0
protein_coding	PAX6	ENSG00000007372.22	0	0
protein_coding	AL035078.4	ENSG00000285283.1	0	0
protein_coding	C11orf91	ENSG00000205177.6	0	0
protein_coding	AL049629.2	ENSG00000284969.1	0	0
protein_coding	ELF5	ENSG00000135374.9	0	0
protein_coding	RAG2	ENSG00000175097.7	0	0
protein_coding	ACCSL	ENSG00000205126.2	0	0
protein_coding	SYT13	ENSG00000019505.7	0	0
protein_coding	CHRM4	ENSG00000180720.7	0	0
protein_coding	F2	ENSG00000180210.14	0	0
protein_coding	MYBPC3	ENSG00000134571.10	0	0
protein_coding	RAPSN	ENSG00000165917.9	0	0
protein_coding	FAM180B	ENSG00000196666.4	0	0
protein_coding	C1QTNF4	ENSG00000172247.3	0	0
protein_coding	OR4B1	ENSG00000175619.3	0	0
protein_coding	OR4X2	ENSG00000172208.6	0	0
protein_coding	OR4X1	ENSG00000176567.1	0	0
protein_coding	OR4S1	ENSG00000176555.1	0	0
protein_coding	OR4C3	ENSG00000176547.9	0	0
protein_coding	OR4C5	ENSG00000176540.3	0	0
protein_coding	OR4A47	ENSG00000237388.2	0	0
protein_coding	TRIM51GP	ENSG00000220948.5	0	0
protein_coding	TRIM49B	ENSG00000182053.12	0	0
protein_coding	TRIM64C	ENSG00000214891.9	0	0
protein_coding	FOLH1	ENSG00000086205.17	0	0
protein_coding	OR4C13	ENSG00000258817.1	0	0
protein_coding	OR4C12	ENSG00000221954.2	0	0
protein_coding	OR4C46	ENSG00000185926.1	0	0
protein_coding	OR4A5	ENSG00000221840.4	0	0
protein_coding	OR4A16	ENSG00000181961.4	0	0
protein_coding	OR4A15	ENSG00000181958.4	0	0
protein_coding	OR4C15	ENSG00000181939.3	0	0
protein_coding	OR4C16	ENSG00000279514.2	0	0
protein_coding	OR4P4	ENSG00000181927.3	0	0
protein_coding	OR4S2	ENSG00000174982.3	0	0
protein_coding	OR5D3P	ENSG00000186886.8	0	0
protein_coding	OR5D13	ENSG00000279761.2	0	0
protein_coding	OR5D14	ENSG00000186113.1	0	0

protein_coding	OR5L1	ENSG00000279395.3	0	0
protein_coding	OR5D18	ENSG00000186119.8	0	0
protein_coding	OR5L2	ENSG00000205030.1	0	0
protein_coding	OR5D16	ENSG00000205029.1	0	0
protein_coding	TRIM51	ENSG00000124900.12	0	0
protein_coding	OR5W2	ENSG00000187612.1	0	0
protein_coding	OR5I1	ENSG00000167825.3	0	0
protein_coding	OR10AG1	ENSG00000174970.5	0	0
protein_coding	OR5F1	ENSG00000149133.1	0	0
protein_coding	OR5AS1	ENSG00000181785.2	0	0
protein_coding	OR8I2	ENSG00000172154.10	0	0
protein_coding	OR8H2	ENSG00000181767.3	0	0
protein_coding	OR8H3	ENSG00000181761.5	0	0
protein_coding	OR8J3	ENSG00000167822.2	0	0
protein_coding	OR8K5	ENSG00000181752.3	0	0
protein_coding	OR5J2	ENSG00000174957.1	0	0
protein_coding	OR5T2	ENSG00000181718.6	0	0
protein_coding	OR5T3	ENSG00000172489.6	0	0
protein_coding	OR5T1	ENSG00000181698.4	0	0
protein_coding	OR8H1	ENSG00000181693.8	0	0
protein_coding	OR8K3	ENSG00000280314.3	0	0
protein_coding	OR8K1	ENSG00000150261.4	0	0
protein_coding	OR8J1	ENSG00000172487.4	0	0
protein_coding	OR8U1	ENSG00000172199.1	0	0
protein_coding	OR5R1	ENSG00000279961.2	0	0
protein_coding	AP002512.3	ENSG00000284732.1	0	0
protein_coding	OR5M9	ENSG00000150269.1	0	0
protein_coding	OR5M3	ENSG00000174937.4	0	0
protein_coding	OR5M8	ENSG00000181371.4	0	0
protein_coding	OR5M11	ENSG00000255223.4	0	0
protein_coding	OR5M10	ENSG00000254834.4	0	0
protein_coding	OR5M1	ENSG00000255012.2	0	0
protein_coding	OR5AP2	ENSG00000172464.3	0	0
protein_coding	OR5AR1	ENSG00000172459.4	0	0
protein_coding	OR9G1	ENSG00000174914.2	0	0
protein_coding	OR9G4	ENSG00000172457.6	0	0
protein_coding	OR5AK2	ENSG00000181273.2	0	0
protein_coding	LRRC55	ENSG00000183908.5	0	0
protein_coding	APLNR	ENSG00000134817.10	0	0
protein_coding	P2RX3	ENSG00000109991.8	0	0
protein_coding	PRG3	ENSG00000156575.2	0	0
protein_coding	PRG2	ENSG00000186652.9	0	0
protein_coding	AP000781.2	ENSG00000254979.5	0	0
protein_coding	RTN4RL2	ENSG00000186907.7	0	0

protein_coding	SMTNL1	ENSG00000214872.8	0	0
protein_coding	TMX2-	ENSG00000254462.1	0	0
protein_coding	AP001931.1	ENSG00000254732.1	0	0
protein_coding	BTBD18	ENSG00000233436.7	0	0
protein_coding	OR6Q1	ENSG00000279051.4	0	0
protein_coding	OR9I1	ENSG00000172377.2	0	0
protein_coding	OR9Q2	ENSG00000186513.3	0	0
protein_coding	OR1S2	ENSG00000197887.4	0	0
protein_coding	OR1S1	ENSG00000280204.3	0	0
protein_coding	OR10Q1	ENSG00000180475.4	0	0
protein_coding	OR10W1	ENSG00000172772.3	0	0
protein_coding	OR5B17	ENSG00000197786.3	0	0
protein_coding	OR5B3	ENSG00000172769.3	0	0
protein_coding	OR5B2	ENSG00000172365.3	0	0
protein_coding	OR5B12	ENSG00000172362.3	0	0
protein_coding	OR5B21	ENSG00000198283.2	0	0
protein_coding	ZFP91-CNTF	ENSG00000255073.8	0	0
protein_coding	GLYAT	ENSG00000149124.10	0	0
protein_coding	GLYATL1B	ENSG00000255151.2	0	0
protein_coding	MPEG1	ENSG00000197629.5	0	0
protein_coding	OR5AN1	ENSG00000176495.3	0	0
protein_coding	OR5A2	ENSG00000172324.5	0	0
protein_coding	OR5A1	ENSG00000172320.3	0	0
protein_coding	OR4D6	ENSG00000166884.2	0	0
protein_coding	OR4D10	ENSG00000254466.2	0	0
protein_coding	OR4D11	ENSG00000176200.1	0	0
protein_coding	OR4D9	ENSG00000172742.5	0	0
protein_coding	OR10V1	ENSG00000172289.3	0	0
protein_coding	GIF	ENSG00000134812.7	0	0
protein_coding	OOSP3	ENSG00000285231.1	0	0
protein_coding	OOSP1	ENSG00000284873.1	0	0
protein_coding	OOSP4A	ENSG00000285010.1	0	0
protein_coding	OOSP4B	ENSG00000255393.2	0	0
protein_coding	OOSP2	ENSG00000149507.6	0	0
protein_coding	MS4A3	ENSG00000149516.13	0	0
protein_coding	MS4A2	ENSG00000149534.8	0	0
protein_coding	MS4A6A	ENSG00000110077.14	0	0
protein_coding	MS4A4A	ENSG00000110079.17	0	0
protein_coding	MS4A4E	ENSG00000214787.9	0	0
protein_coding	MS4A6E	ENSG00000166926.8	0	0
protein_coding	MS4A7	ENSG00000166927.12	0	0
protein_coding	MS4A14	ENSG00000166928.10	0	0
protein_coding	MS4A5	ENSG00000166930.6	0	0
protein_coding	MS4A1	ENSG00000156738.17	0	0

protein_coding	MS4A13	ENSG00000204979.7	0	0
protein_coding	MS4A8	ENSG00000166959.7	0	0
protein_coding	MS4A18	ENSG00000214782.7	0	0
protein_coding	MS4A15	ENSG00000166961.14	0	0
protein_coding	MS4A10	ENSG00000172689.1	0	0
protein_coding	PTGDR2	ENSG00000183134.4	0	0
protein_coding	CD5	ENSG00000110448.10	0	0
protein_coding	PGA3	ENSG00000229859.9	0	0
protein_coding	PGA4	ENSG00000229183.8	0	0
protein_coding	SCGB1D1	ENSG00000168515.3	0	0
protein_coding	SCGB2A1	ENSG00000124939.5	0	0
protein_coding	SCGB1D2	ENSG00000124935.3	0	0
protein_coding	SCGB2A2	ENSG00000110484.6	0	0
protein_coding	SCGB1D4	ENSG00000197745.2	0	0
protein_coding	EEF1G	ENSG00000254772.9	0	0
protein_coding	AP002990.1	ENSG00000255508.7	0	0
protein_coding	AP001458.2	ENSG00000255432.1	0	0
protein_coding	C11orf98	ENSG00000278615.4	0	0
protein_coding	BSCL2	ENSG00000168000.14	0	0
protein_coding	HNRNPUL2-BSCL2	ENSG00000234857.2	0	0
protein_coding	TEX54	ENSG00000283268.1	0	0
protein_coding	CHRM1	ENSG00000168539.3	0	0
protein_coding	SLC22A6	ENSG00000197901.11	0	0
protein_coding	SLC22A8	ENSG00000149452.15	0	0
protein_coding	SLC22A24	ENSG00000197658.9	0	0
protein_coding	SLC22A10	ENSG00000184999.11	0	0
protein_coding	SLC22A25	ENSG00000196600.12	0	0
protein_coding	SLC22A9	ENSG00000149742.9	0	0
protein_coding	HRASLS5	ENSG00000168004.9	0	0
protein_coding	AP000721.1	ENSG00000256100.1	0	0
protein_coding	FLRT1	ENSG00000126500.3	0	0
protein_coding	KCNK4	ENSG00000182450.12	0	0
protein_coding	CATSPERZ	ENSG00000219435.5	0	0
protein_coding	SLC22A11	ENSG00000168065.15	0	0
protein_coding	SLC22A12	ENSG00000197891.11	0	0
protein_coding	PYGM	ENSG00000068976.13	0	0
protein_coding	GPHA2	ENSG00000149735.6	0	0
protein_coding	MAJIN	ENSG00000168070.11	0	0
protein_coding	ARL2-SNX15	ENSG00000273003.1	0	0
protein_coding	SNX15	ENSG00000110025.12	0	0
protein_coding	TMEM262	ENSG00000187066.8	0	0
protein_coding	SPDYC	ENSG00000204710.2	0	0

protein_coding	AP000944.2	ENSG00000285816.1	0	0
protein_coding	CDC42EP2	ENSG00000149798.4	0	0
protein_coding	CTSW	ENSG00000172543.7	0	0
protein_coding	TSGA10IP	ENSG00000175513.9	0	0
protein_coding	GAL3ST3	ENSG00000175229.6	0	0
protein_coding	TMEM151A	ENSG00000179292.4	0	0
protein_coding	NPAS4	ENSG00000174576.9	0	0
protein_coding	AP002748.4	ENSG00000256349.1	0	0
protein_coding	ACTN3	ENSG00000248746.5	0	0
protein_coding	AP003419.1	ENSG00000256514.1	0	0
protein_coding	TBC1D10C	ENSG00000175463.11	0	0
protein_coding	PTPRCAP	ENSG00000213402.2	0	0
protein_coding	GPR152	ENSG00000175514.2	0	0
protein_coding	CABP2	ENSG00000167791.13	0	0
protein_coding	TBX10	ENSG00000167800.9	0	0
protein_coding	MRGPRD	ENSG00000172938.3	0	0
protein_coding	AP003071.5	ENSG00000284713.1	0	0
protein_coding	FGF4	ENSG00000075388.3	0	0
protein_coding	FGF3	ENSG00000186895.3	0	0
protein_coding	KRTAP5-7	ENSG00000244411.3	0	0
protein_coding	KRTAP5-9	ENSG00000254997.3	0	0
protein_coding	KRTAP5-10	ENSG00000204572.9	0	0
protein_coding	KRTAP5-11	ENSG00000204571.6	0	0
protein_coding	ZNF705E	ENSG00000214534.5	0	0
protein_coding	DEFB108B	ENSG00000184276.2	0	0
protein_coding	DEFB131B	ENSG00000225805.4	0	0
protein_coding	AP000812.5	ENSG00000284922.1	0	0
protein_coding	AP000812.4	ENSG00000284844.1	0	0
protein_coding	FOLR3	ENSG00000110203.8	0	0
protein_coding	FOLR2	ENSG00000165457.13	0	0
protein_coding	PHOX2A	ENSG00000165462.5	0	0
protein_coding	PDE2A	ENSG00000186642.15	0	0
protein_coding	OR2AT4	ENSG00000171561.4	0	0
protein_coding	SLCO2B1	ENSG00000137491.14	0	0
protein_coding	TPBGL	ENSG00000261594.3	0	0
protein_coding	WNT11	ENSG00000085741.12	0	0
protein_coding	B3GNT6	ENSG00000198488.10	0	0
protein_coding	OMP	ENSG00000254550.1	0	0
protein_coding	GDPD4	ENSG00000178795.9	0	0
protein_coding	NDUFC2-KCTD14	ENSG00000259112.2	0	0
protein_coding	THRSP	ENSG00000151365.2	0	0
protein_coding	TYR	ENSG00000077498.8	0	0

protein_coding	TRIM77	ENSG00000214414.9	0	0
protein_coding	TRIM49	ENSG00000168930.13	0	0
protein_coding	TRIM64B	ENSG00000189253.7	0	0
protein_coding	TRIM49D1	ENSG00000223417.8	0	0
protein_coding	TRIM49D2	ENSG00000233802.8	0	0
protein_coding	TRIM64	ENSG00000204450.7	0	0
protein_coding	TRIM49C	ENSG00000204449.3	0	0
protein_coding	UBTFL1	ENSG00000255009.4	0	0
protein_coding	NAALAD2	ENSG00000077616.10	0	0
protein_coding	MTNR1B	ENSG00000134640.2	0	0
protein_coding	DEUP1	ENSG00000165325.13	0	0
protein_coding	AP001273.2	ENSG00000284057.1	0	0
protein_coding	VSTM5	ENSG00000214376.5	0	0
protein_coding	IZUMO1R	ENSG00000183560.9	0	0
protein_coding	GPR83	ENSG00000123901.8	0	0
protein_coding	KDM4E	ENSG00000235268.2	0	0
protein_coding	KDM4F	ENSG00000255855.2	0	0
protein_coding	DDI1	ENSG00000170967.4	0	0
protein_coding	CASP5	ENSG00000137757.10	0	0
protein_coding	CARD17	ENSG00000255221.3	0	0
protein_coding	CARD18	ENSG00000255501.2	0	0
protein_coding	GRIA4	ENSG00000152578.12	0	0
protein_coding	RAB39A	ENSG00000179331.2	0	0
protein_coding	C11orf53	ENSG00000150750.7	0	0
protein_coding	BTG4	ENSG00000137707.13	0	0
protein_coding	C11orf88	ENSG00000183644.13	0	0
protein_coding	AP001781.2	ENSG00000258529.5	0	0
protein_coding	HSPB2-	ENSG00000254445.1	0	0
protein_coding	AP002884.3	ENSG00000255292.8	0	0
protein_coding	PLET1	ENSG00000188771.4	0	0
protein_coding	CLDN25	ENSG00000228607.2	0	0
protein_coding	HTR3B	ENSG00000149305.6	0	0
protein_coding	HTR3A	ENSG00000166736.11	0	0
protein_coding	ZBTB16	ENSG00000109906.13	0	0
protein_coding	AP002373.1	ENSG00000255663.1	0	0
protein_coding	NXPE1	ENSG00000095110.8	0	0
protein_coding	NXPE4	ENSG00000137634.9	0	0
protein_coding	NXPE2	ENSG00000204361.8	0	0
protein_coding	APOA5	ENSG00000110243.11	0	0
protein_coding	APOA4	ENSG00000110244.6	0	0
protein_coding	APOC3	ENSG00000110245.11	0	0
protein_coding	DSCAML1	ENSG00000177103.13	0	0
protein_coding	FXYD6-	ENSG00000255245.4	0	0
protein_coding	FXYD6	ENSG00000137726.16	0	0

protein_coding	IL10RA	ENSG00000110324.10	0	0
protein_coding	SMIM35	ENSG00000255274.9	0	0
protein_coding	JAML	ENSG00000160593.18	0	0
protein_coding	CD3E	ENSG00000198851.9	0	0
protein_coding	CD3D	ENSG00000167286.9	0	0
protein_coding	CD3G	ENSG00000160654.10	0	0
protein_coding	TTC36	ENSG00000172425.10	0	0
protein_coding	TREH	ENSG00000118094.11	0	0
protein_coding	CXCR5	ENSG00000160683.4	0	0
protein_coding	FOXR1	ENSG00000176302.12	0	0
protein_coding	ABCG4	ENSG00000172350.9	0	0
protein_coding	PDZD3	ENSG00000172367.15	0	0
protein_coding	C1QTNF5	ENSG00000223953.5	0	0
protein_coding	MFRP	ENSG00000235718.8	0	0
protein_coding	BSX	ENSG00000188909.4	0	0
protein_coding	OR6X1	ENSG00000221931.2	0	0
protein_coding	OR6M1	ENSG00000196099.4	0	0
protein_coding	TMEM225	ENSG00000204300.6	0	0
protein_coding	OR8D4	ENSG00000181518.4	0	0
protein_coding	OR4D5	ENSG00000171014.2	0	0
protein_coding	OR6T1	ENSG00000181499.2	0	0
protein_coding	OR10S1	ENSG00000196248.5	0	0
protein_coding	OR10G6	ENSG00000198674.3	0	0
protein_coding	OR10G4	ENSG00000254737.2	0	0
protein_coding	OR10G9	ENSG00000236981.1	0	0
protein_coding	OR10G8	ENSG00000234560.4	0	0
protein_coding	OR10G7	ENSG00000182634.8	0	0
protein_coding	OR10D3	ENSG00000197309.3	0	0
protein_coding	OR8G1	ENSG00000197849.6	0	0
protein_coding	OR8G5	ENSG00000255298.3	0	0
protein_coding	OR8D1	ENSG00000196341.3	0	0
protein_coding	OR8D2	ENSG00000279116.2	0	0
protein_coding	OR8B2	ENSG00000284680.1	0	0
protein_coding	OR8B3	ENSG00000284609.1	0	0
protein_coding	OR8B4	ENSG00000280090.2	0	0
protein_coding	OR8B8	ENSG00000197125.3	0	0
protein_coding	OR8B12	ENSG00000170953.3	0	0
protein_coding	PANX3	ENSG00000154143.2	0	0
protein_coding	VSIG2	ENSG00000019102.11	0	0
protein_coding	HEPACAM	ENSG00000165478.6	0	0
protein_coding	PKNOX2	ENSG00000165495.15	0	0
protein_coding	ACRV1	ENSG00000134940.13	0	0
protein_coding	PATE1	ENSG00000171053.9	0	0
protein_coding	PATE3	ENSG00000236027.2	0	0

protein_coding	PATE4	ENSG00000237353.6	0	0
protein_coding	VSIG10L2	ENSG00000283703.2	0	0
protein_coding	KCNJ1	ENSG00000151704.15	0	0
protein_coding	OPCML	ENSG00000183715.13	0	0
protein_coding	SPATA19	ENSG00000166118.7	0	0
protein_coding	IGSF9B	ENSG00000080854.14	0	0
protein_coding	GLB1L3	ENSG00000166105.15	0	0
protein_coding	GLB1L2	ENSG00000149328.14	0	0
protein_coding	B3GAT1	ENSG00000109956.12	0	0
protein_coding	SLC6A13	ENSG00000010379.15	0	0
protein_coding	LRTM2	ENSG00000166159.10	0	0
protein_coding	NRIP2	ENSG00000053702.14	0	0
protein_coding	TEX52	ENSG00000283297.1	0	0
protein_coding	AC008012.1	ENSG00000285901.1	0	0
protein_coding	FGF6	ENSG00000111241.2	0	0
protein_coding	AC005832.4	ENSG00000272921.1	0	0
protein_coding	GALNT8	ENSG00000130035.7	0	0
protein_coding	KCNA6	ENSG00000151079.7	0	0
protein_coding	KCNA1	ENSG00000111262.6	0	0
protein_coding	KCNA5	ENSG00000130037.4	0	0
protein_coding	CD27	ENSG00000139193.3	0	0
protein_coding	NOP2	ENSG00000111641.11	0	0
protein_coding	AC006064.6	ENSG00000285238.2	0	0
protein_coding	LAG3	ENSG00000089692.8	0	0
protein_coding	RBP5	ENSG00000139194.7	0	0
protein_coding	ACSM4	ENSG00000215009.5	0	0
protein_coding	CD163	ENSG00000177575.12	0	0
protein_coding	APOBEC1	ENSG00000111701.6	0	0
protein_coding	GDF3	ENSG00000184344.3	0	0
protein_coding	DPPA3	ENSG00000187569.2	0	0
protein_coding	CLEC4C	ENSG00000198178.10	0	0
protein_coding	NANOGNB	ENSG00000205857.2	0	0
protein_coding	NANOG	ENSG00000111704.10	0	0
protein_coding	SLC2A14	ENSG00000173262.11	0	0
protein_coding	CLEC4A	ENSG00000111729.14	0	0
protein_coding	AC092111.3	ENSG00000284393.1	0	0
protein_coding	ZNF705A	ENSG00000196946.10	0	0
protein_coding	CLEC6A	ENSG00000205846.3	0	0
protein_coding	CLEC4D	ENSG00000166527.7	0	0
protein_coding	CLEC4E	ENSG00000166523.7	0	0
protein_coding	A2ML1	ENSG00000166535.19	0	0
protein_coding	A2M	ENSG00000175899.14	0	0
protein_coding	PZP	ENSG00000126838.9	0	0
protein_coding	KLRB1	ENSG00000111796.3	0	0

protein_coding	CLECL1	ENSG00000184293.7	0	0
protein_coding	CD69	ENSG00000110848.8	0	0
protein_coding	KLRF2	ENSG00000256797.1	0	0
protein_coding	CLEC2A	ENSG00000188393.8	0	0
protein_coding	CLEC12A	ENSG00000172322.13	0	0
protein_coding	CLEC1B	ENSG00000165682.14	0	0
protein_coding	CLEC12B	ENSG00000256660.5	0	0
protein_coding	CLEC9A	ENSG00000197992.6	0	0
protein_coding	KLRK1	ENSG00000213809.8	0	0
protein_coding	KLRC4-	ENSG00000255819.7	0	0
protein_coding	KLRC4	ENSG00000183542.5	0	0
protein_coding	KLRC3	ENSG00000205810.8	0	0
protein_coding	AC068775.1	ENSG00000255641.1	0	0
protein_coding	KLRC2	ENSG00000205809.9	0	0
protein_coding	EIF2S3B	ENSG00000180574.3	0	0
protein_coding	TAS2R7	ENSG00000121377.2	0	0
protein_coding	TAS2R8	ENSG00000121314.2	0	0
protein_coding	TAS2R9	ENSG00000121381.4	0	0
protein_coding	TAS2R10	ENSG00000121318.2	0	0
protein_coding	PRR4	ENSG00000111215.12	0	0
protein_coding	PRH1-PRR4	ENSG00000275778.2	0	0
protein_coding	AC006518.7	ENSG00000284826.1	0	0
protein_coding	PRH2	ENSG00000134551.12	0	0
protein_coding	TAS2R50	ENSG00000212126.3	0	0
protein_coding	TAS2R31	ENSG00000256436.1	0	0
protein_coding	TAS2R46	ENSG00000226761.3	0	0
protein_coding	TAS2R42	ENSG00000186136.1	0	0
protein_coding	PRB4	ENSG00000230657.6	0	0
protein_coding	PRB1	ENSG00000251655.6	0	0
protein_coding	PRB2	ENSG00000121335.11	0	0
protein_coding	GPRC5D	ENSG00000111291.8	0	0
protein_coding	GRIN2B	ENSG00000273079.5	0	0
protein_coding	GUCY2C	ENSG00000070019.4	0	0
protein_coding	SMCO3	ENSG00000179256.2	0	0
protein_coding	ART4	ENSG00000111339.11	0	0
protein_coding	PDE6H	ENSG00000139053.2	0	0
protein_coding	SLC15A5	ENSG00000188991.3	0	0
protein_coding	RERGL	ENSG00000111404.6	0	0
protein_coding	PLCZ1	ENSG00000139151.15	0	0
protein_coding	SLCO1C1	ENSG00000139155.8	0	0
protein_coding	SLCO1B7	ENSG00000205754.11	0	0
protein_coding	AC022335.1	ENSG00000257062.6	0	0
protein_coding	IAPP	ENSG00000121351.7	0	0
protein_coding	GYS2	ENSG00000111713.2	0	0

protein_coding	AC010197.2	ENSG00000285854.1	0	0
protein_coding	LMNTD1	ENSG00000152936.10	0	0
protein_coding	C12orf71	ENSG00000214700.5	0	0
protein_coding	MANSC4	ENSG00000205693.3	0	0
protein_coding	SYT10	ENSG00000110975.8	0	0
protein_coding	ABCD2	ENSG00000173208.3	0	0
protein_coding	C12orf40	ENSG00000180116.15	0	0
protein_coding	MUC19	ENSG00000205592.14	0	0
protein_coding	PDZRN4	ENSG00000165966.15	0	0
protein_coding	DBX2	ENSG00000185610.6	0	0
protein_coding	OR10AD1	ENSG00000172640.3	0	0
protein_coding	H1FNT	ENSG00000187166.1	0	0
protein_coding	ANP32D	ENSG00000139223.2	0	0
protein_coding	OR8S1	ENSG00000284723.1	0	0
protein_coding	OR5BS1P	ENSG00000198678.5	0	0
protein_coding	LALBA	ENSG00000167531.6	0	0
protein_coding	TEX49	ENSG00000257987.5	0	0
protein_coding	AC073610.3	ENSG00000272822.1	0	0
protein_coding	AC073610.2	ENSG00000255863.2	0	0
protein_coding	WNT1	ENSG00000125084.11	0	0
protein_coding	DHH	ENSG00000139549.3	0	0
protein_coding	C1QL4	ENSG00000186897.4	0	0
protein_coding	AQP2	ENSG00000167580.7	0	0
protein_coding	AQP5	ENSG00000161798.6	0	0
protein_coding	AQP6	ENSG00000086159.12	0	0
protein_coding	GPD1	ENSG00000167588.12	0	0
protein_coding	FAM186A	ENSG00000185958.9	0	0
protein_coding	TMPRSS12	ENSG00000186452.10	0	0
protein_coding	HIGD1C	ENSG00000214511.3	0	0
protein_coding	CELA1	ENSG00000139610.1	0	0
protein_coding	ANKRD33	ENSG00000167612.12	0	0
protein_coding	OR7E47P	ENSG00000284791.1	0	0
protein_coding	KRT83	ENSG00000170523.3	0	0
protein_coding	KRT85	ENSG00000135443.8	0	0
protein_coding	KRT84	ENSG00000161849.3	0	0
protein_coding	KRT82	ENSG00000161850.2	0	0
protein_coding	KRT75	ENSG00000170454.5	0	0
protein_coding	KRT71	ENSG00000139648.6	0	0
protein_coding	KRT72	ENSG00000170486.10	0	0
protein_coding	KRT73	ENSG00000186049.8	0	0
protein_coding	KRT2	ENSG00000172867.3	0	0
protein_coding	KRT77	ENSG00000189182.9	0	0
protein_coding	KRT76	ENSG00000185069.2	0	0
protein_coding	KRT3	ENSG00000186442.7	0	0

protein_coding	KRT79	ENSG00000185640.5	0	0
protein_coding	KRT78	ENSG00000170423.12	0	0
protein_coding	AC021072.1	ENSG00000283536.2	0	0
protein_coding	SP7	ENSG00000170374.5	0	0
protein_coding	AC023509.3	ENSG00000267281.2	0	0
protein_coding	NPFF	ENSG00000139574.8	0	0
protein_coding	HOXC12	ENSG00000123407.3	0	0
protein_coding	AC012531.2	ENSG00000273049.1	0	0
protein_coding	HOXC5	ENSG00000172789.3	0	0
protein_coding	NCKAP1L	ENSG00000123338.12	0	0
protein_coding	PDE1B	ENSG00000123360.11	0	0
protein_coding	PPP1R1A	ENSG00000135447.16	0	0
protein_coding	LACRT	ENSG00000135413.8	0	0
protein_coding	DCD	ENSG00000161634.11	0	0
protein_coding	NEUROD4	ENSG00000123307.3	0	0
protein_coding	OR9K2	ENSG00000170605.6	0	0
protein_coding	OR10A7	ENSG00000179919.3	0	0
protein_coding	OR6C74	ENSG00000197706.3	0	0
protein_coding	OR6C6	ENSG00000188324.4	0	0
protein_coding	OR6C1	ENSG00000205330.4	0	0
protein_coding	OR6C3	ENSG00000205329.2	0	0
protein_coding	OR6C75	ENSG00000187857.4	0	0
protein_coding	OR6C65	ENSG00000205328.2	0	0
protein_coding	OR6C76	ENSG00000185821.3	0	0
protein_coding	OR6C2	ENSG00000179695.2	0	0
protein_coding	OR6C70	ENSG00000184954.4	0	0
protein_coding	OR6C68	ENSG00000205327.3	0	0
protein_coding	OR6C4	ENSG00000179626.4	0	0
protein_coding	OR2AP1	ENSG00000179615.2	0	0
protein_coding	OR10P1	ENSG00000175398.2	0	0
protein_coding	BLOC1S1-	ENSG00000258311.5	0	0
protein_coding	AC023055.1	ENSG00000257390.5	0	0
protein_coding	AC034102.2	ENSG00000257411.1	0	0
protein_coding	SLC39A5	ENSG00000139540.11	0	0
protein_coding	AC073896.1	ENSG00000144785.8	0	0
protein_coding	APOF	ENSG00000175336.9	0	0
protein_coding	MIP	ENSG00000135517.7	0	0
protein_coding	AC097104.1	ENSG00000285528.1	0	0
protein_coding	AC117378.1	ENSG00000285625.1	0	0
protein_coding	SDR9C7	ENSG00000170426.1	0	0
protein_coding	GPR182	ENSG00000166856.2	0	0
protein_coding	MYO1A	ENSG00000166866.12	0	0
protein_coding	AC137834.1	ENSG00000258830.1	0	0
protein_coding	INHBC	ENSG00000175189.3	0	0

protein_coding	ARHGAP9	ENSG00000123329.17	0	0
protein_coding	AC022506.1	ENSG00000285133.1	0	0
protein_coding	AC025165.3	ENSG00000257921.5	0	0
protein_coding	WIF1	ENSG00000156076.9	0	0
protein_coding	AC078927.1	ENSG00000228144.2	0	0
protein_coding	IFNG	ENSG00000111537.4	0	0
protein_coding	IL26	ENSG00000111536.4	0	0
protein_coding	IL22	ENSG00000127318.11	0	0
protein_coding	LYZ	ENSG00000090382.6	0	0
protein_coding	LRRC10	ENSG00000198812.4	0	0
protein_coding	AC025263.2	ENSG00000258052.1	0	0
protein_coding	MYRFL	ENSG00000166268.10	0	0
protein_coding	AC073612.1	ENSG00000258064.1	0	0
protein_coding	TPH2	ENSG00000139287.12	0	0
protein_coding	KCNC2	ENSG00000166006.13	0	0
protein_coding	GLIPR1L1	ENSG00000173401.9	0	0
protein_coding	MYF6	ENSG00000111046.3	0	0
protein_coding	MYF5	ENSG00000111049.3	0	0
protein_coding	C12orf50	ENSG00000165805.9	0	0
protein_coding	GALNT4	ENSG00000257594.3	0	0
protein_coding	POC1B-GALNT4	ENSG00000259075.6	0	0
protein_coding	CCER1	ENSG00000197651.4	0	0
protein_coding	EPYC	ENSG00000083782.7	0	0
protein_coding	KERA	ENSG00000139330.5	0	0
protein_coding	PLEKHG7	ENSG00000187510.9	0	0
protein_coding	CCDC38	ENSG00000165972.12	0	0
protein_coding	FAM71C	ENSG00000180219.1	0	0
protein_coding	SLC17A8	ENSG00000179520.10	0	0
protein_coding	NR1H4	ENSG00000012504.14	0	0
protein_coding	ANO4	ENSG00000151572.17	0	0
protein_coding	SLC5A8	ENSG00000256870.2	0	0
protein_coding	SPIC	ENSG00000166211.7	0	0
protein_coding	MYBPC1	ENSG00000196091.13	0	0
protein_coding	ASCL1	ENSG00000139352.3	0	0
protein_coding	STAB2	ENSG00000136011.14	0	0
protein_coding	RFX4	ENSG00000111783.12	0	0
protein_coding	ASCL4	ENSG00000187855.5	0	0
protein_coding	DAO	ENSG00000110887.7	0	0
protein_coding	SVOP	ENSG00000166111.9	0	0
protein_coding	FOXN4	ENSG00000139445.17	0	0
protein_coding	MYO1H	ENSG00000174527.9	0	0
protein_coding	MYL2	ENSG00000111245.14	0	0

protein_coding	CUX2	ENSG00000111249.13	0	0
protein_coding	AC002996.1	ENSG00000257767.2	0	0
protein_coding	RPH3A	ENSG00000089169.14	0	0
protein_coding	DTX1	ENSG00000135144.7	0	0
protein_coding	RASAL1	ENSG00000111344.11	0	0
protein_coding	SDS	ENSG00000135094.10	0	0
protein_coding	TESC	ENSG00000088992.17	0	0
protein_coding	SRRM4	ENSG00000139767.9	0	0
protein_coding	CCDC60	ENSG00000183273.6	0	0
protein_coding	TMEM233	ENSG00000224982.3	0	0
protein_coding	MSI1	ENSG00000135097.6	0	0
protein_coding	AL021546.1	ENSG00000111780.8	0	0
protein_coding	HNF1A	ENSG00000135100.17	0	0
protein_coding	AC069503.2	ENSG00000256950.2	0	0
protein_coding	IL31	ENSG00000204671.1	0	0
protein_coding	DIABLO	ENSG00000284934.1	0	0
protein_coding	AC048338.1	ENSG00000256861.1	0	0
protein_coding	ARL6IP4	ENSG00000182196.13	0	0
protein_coding	AC068790.8	ENSG00000274874.1	0	0
protein_coding	TMEM132C	ENSG00000181234.9	0	0
protein_coding	GLT1D1	ENSG00000151948.11	0	0
protein_coding	FZD10	ENSG00000111432.4	0	0
protein_coding	RIMBP2	ENSG00000060709.15	0	0
protein_coding	GALNT9	ENSG00000182870.12	0	0
protein_coding	P2RX2	ENSG00000187848.13	0	0
protein_coding	AC026786.1	ENSG00000256825.4	0	0
protein_coding	ANHX	ENSG00000227059.6	0	0
protein_coding	TUBA3C	ENSG00000198033.11	0	0
protein_coding	TPTE2	ENSG00000132958.17	0	0
protein_coding	SGCG	ENSG00000102683.7	0	0
protein_coding	C1QTNF9B	ENSG00000205863.10	0	0
protein_coding	AL359736.1	ENSG00000273167.1	0	0
protein_coding	C1QTNF9	ENSG00000240654.6	0	0
protein_coding	ATP12A	ENSG00000075673.11	0	0
protein_coding	AMER2	ENSG00000165566.12	0	0
protein_coding	GPR12	ENSG00000132975.7	0	0
protein_coding	GSX1	ENSG00000169840.4	0	0
protein_coding	URAD	ENSG00000183463.5	0	0
protein_coding	FLT3	ENSG00000122025.14	0	0
protein_coding	TEX26	ENSG00000175664.9	0	0
protein_coding	RXFP2	ENSG00000133105.7	0	0
protein_coding	ZAR1L	ENSG00000189167.11	0	0

protein_coding	CCDC169-SOHLH2	ENSG00000250709.1	0	0
protein_coding	SERTM1	ENSG00000180440.3	0	0
protein_coding	CSNK1A1L	ENSG00000180138.7	0	0
protein_coding	STOML3	ENSG00000133115.11	0	0
protein_coding	TNFSF11	ENSG00000120659.14	0	0
protein_coding	FAM216B	ENSG00000179813.6	0	0
protein_coding	AL512506.3	ENSG00000281883.1	0	0
protein_coding	SMIM2	ENSG00000139656.6	0	0
protein_coding	ERICH6B	ENSG00000165837.11	0	0
protein_coding	SIAH3	ENSG00000215475.4	0	0
protein_coding	CPB2	ENSG00000080618.14	0	0
protein_coding	LRRC63	ENSG00000173988.12	0	0
protein_coding	CYSLTR2	ENSG00000152207.7	0	0
protein_coding	MLNR	ENSG00000102539.5	0	0
protein_coding	DLEU7	ENSG00000186047.9	0	0
protein_coding	SERPINE3	ENSG00000253309.6	0	0
protein_coding	AL162377.3	ENSG00000285444.1	0	0
protein_coding	TMEM272	ENSG00000281106.3	0	0
protein_coding	CCDC70	ENSG00000123171.6	0	0
protein_coding	CNMD	ENSG00000136110.12	0	0
protein_coding	PCDH8	ENSG00000136099.13	0	0
protein_coding	PRR20A	ENSG00000204919.1	0	0
protein_coding	PRR20C	ENSG00000229665.8	0	0
protein_coding	PRR20B	ENSG00000204918.3	0	0
protein_coding	PRR20D	ENSG00000227151.4	0	0
protein_coding	PRR20E	ENSG00000234278.3	0	0
protein_coding	PCDH17	ENSG00000118946.11	0	0
protein_coding	AL592490.1	ENSG00000197991.11	0	0
protein_coding	AL445989.1	ENSG00000237378.3	0	0
protein_coding	AL445238.1	ENSG00000285566.1	0	0
protein_coding	KLHL1	ENSG00000150361.11	0	0
protein_coding	ACOD1	ENSG00000102794.9	0	0
protein_coding	AC001226.2	ENSG00000283208.1	0	0
protein_coding	SLAIN1	ENSG00000139737.21	0	0
protein_coding	GPC5	ENSG00000179399.14	0	0
protein_coding	DCT	ENSG00000080166.15	0	0
protein_coding	OXGR1	ENSG00000165621.8	0	0
protein_coding	RNF113B	ENSG00000139797.7	0	0
protein_coding	SLC15A1	ENSG00000088386.16	0	0
protein_coding	GPR18	ENSG00000125245.12	0	0
protein_coding	FGF14	ENSG00000102466.15	0	0
protein_coding	METTL21C	ENSG00000139780.7	0	0

protein_coding	BIVM-ERCC5	ENSG00000270181.3	0	0
protein_coding	SLC10A2	ENSG00000125255.6	0	0
protein_coding	DAOA	ENSG00000182346.19	0	0
protein_coding	SOX1	ENSG00000182968.4	0	0
protein_coding	SPACA7	ENSG00000153498.11	0	0
protein_coding	PROZ	ENSG00000126231.13	0	0
protein_coding	ATP4B	ENSG00000186009.4	0	0
protein_coding	GRK1	ENSG00000185974.6	0	0
protein_coding	C13orf46	ENSG00000283199.2	0	0
protein_coding	CFAP97D2	ENSG00000283361.2	0	0
protein_coding	OR11H12	ENSG00000257115.1	0	0
protein_coding	POTEM	ENSG00000222036.7	0	0
protein_coding	POTEG	ENSG00000187537.13	0	0
protein_coding	OR11H2	ENSG00000258453.3	0	0
protein_coding	OR4N2	ENSG00000176294.5	0	0
protein_coding	OR4M1	ENSG00000176299.5	0	0
protein_coding	OR4K2	ENSG00000165762.3	0	0
protein_coding	OR4K5	ENSG00000176281.4	0	0
protein_coding	OR4K1	ENSG00000155249.5	0	0
protein_coding	OR4K15	ENSG00000169488.6	0	0
protein_coding	OR4K14	ENSG00000169484.3	0	0
protein_coding	OR4K13	ENSG00000176253.3	0	0
protein_coding	OR4L1	ENSG00000176246.1	0	0
protein_coding	OR4K17	ENSG00000176230.6	0	0
protein_coding	OR4N5	ENSG00000184394.2	0	0
protein_coding	OR11H6	ENSG00000176219.3	0	0
protein_coding	OR11H4	ENSG00000176198.3	0	0
protein_coding	KLHL33	ENSG00000185271.8	0	0
protein_coding	RNASE10	ENSG00000182545.6	0	0
protein_coding	RNASE9	ENSG00000188655.10	0	0
protein_coding	AL163195.3	ENSG00000259060.7	0	0
protein_coding	RNASE11	ENSG00000173464.14	0	0
protein_coding	RNASE12	ENSG00000258436.1	0	0
protein_coding	OR6S1	ENSG00000181803.3	0	0
protein_coding	AL163636.2	ENSG00000259171.1	0	0
protein_coding	EDDM3A	ENSG00000181562.4	0	0
protein_coding	EDDM3B	ENSG00000181552.3	0	0
protein_coding	RNASE3	ENSG00000169397.3	0	0
protein_coding	RNASE2	ENSG00000169385.2	0	0
protein_coding	TPPP2	ENSG00000179636.14	0	0
protein_coding	RNASE13	ENSG00000206150.3	0	0
protein_coding	RNASE8	ENSG00000173431.2	0	0
protein_coding	OR5AU1	ENSG00000169327.5	0	0
protein_coding	OR10G3	ENSG00000169208.2	0	0

protein_coding	OR10G2	ENSG00000255582.1	0	0
protein_coding	OR4E2	ENSG00000221977.2	0	0
protein_coding	OR4E1	ENSG00000276240.2	0	0
protein_coding	OR6J1	ENSG00000255804.2	0	0
protein_coding	AL132780.3	ENSG00000259132.1	0	0
protein_coding	PSMB11	ENSG00000222028.3	0	0
protein_coding	LMLN2	ENSG00000283654.2	0	0
protein_coding	CEBPE	ENSG00000092067.5	0	0
protein_coding	BCL2L2-PABPN1	ENSG00000258643.5	0	0
protein_coding	IL25	ENSG00000166090.8	0	0
protein_coding	CMTM5	ENSG00000166091.20	0	0
protein_coding	MYH6	ENSG00000197616.11	0	0
protein_coding	MYH7	ENSG00000092054.12	0	0
protein_coding	THTPA	ENSG00000259431.5	0	0
protein_coding	JPH4	ENSG00000092051.16	0	0
protein_coding	CPNE6	ENSG00000100884.9	0	0
protein_coding	AL136295.3	ENSG00000259371.2	0	0
protein_coding	RNF31	ENSG00000092098.16	0	0
protein_coding	AL136295.4	ENSG00000259522.3	0	0
protein_coding	AL136295.1	ENSG00000254692.1	0	0
protein_coding	TSSK4	ENSG00000139908.14	0	0
protein_coding	MDP1	ENSG00000213920.8	0	0
protein_coding	NEDD8-	ENSG00000255526.6	0	0
protein_coding	LTB4R2	ENSG00000213906.9	0	0
protein_coding	AL096870.1	ENSG00000258973.1	0	0
protein_coding	CMA1	ENSG00000092009.10	0	0
protein_coding	CTSG	ENSG00000100448.3	0	0
protein_coding	GZMH	ENSG00000100450.12	0	0
protein_coding	FOXG1	ENSG00000176165.10	0	0
protein_coding	GPR33	ENSG00000214943.4	0	0
protein_coding	SFTA3	ENSG00000229415.9	0	0
protein_coding	CLEC14A	ENSG00000176435.6	0	0
protein_coding	LRFN5	ENSG00000165379.13	0	0
protein_coding	FSCB	ENSG00000189139.5	0	0
protein_coding	RPL10L	ENSG00000165496.4	0	0
protein_coding	MDGA2	ENSG00000139915.19	0	0
protein_coding	ABHD12B	ENSG00000131969.14	0	0
protein_coding	PTGDR	ENSG00000168229.3	0	0
protein_coding	TBPL2	ENSG00000182521.5	0	0
protein_coding	OTX2	ENSG00000165588.16	0	0
protein_coding	CCDC198	ENSG00000100557.9	0	0
protein_coding	SLC35F4	ENSG00000151812.14	0	0

protein_coding	TOMM20L	ENSG00000196860.7	0	0
protein_coding	CCDC175	ENSG00000151838.11	0	0
protein_coding	SIX6	ENSG00000184302.6	0	0
protein_coding	AL355916.2	ENSG00000258989.1	0	0
protein_coding	RHOJ	ENSG00000126785.12	0	0
protein_coding	GPHB5	ENSG00000179600.3	0	0
protein_coding	SPTB	ENSG00000070182.20	0	0
protein_coding	CHURC1-	ENSG00000125954.12	0	0
protein_coding	CCDC196	ENSG00000196553.15	0	0
protein_coding	AL049779.1	ENSG00000258466.5	0	0
protein_coding	SLC10A1	ENSG00000100652.4	0	0
protein_coding	SLC8A3	ENSG00000100678.18	0	0
protein_coding	SYNJ2BP-COX16	ENSG00000258644.5	0	0
protein_coding	HEATR4	ENSG00000187105.8	0	0
protein_coding	ACOT1	ENSG00000184227.7	0	0
protein_coding	ACOT6	ENSG00000205669.3	0	0
protein_coding	AC005520.1	ENSG00000258653.3	0	0
protein_coding	VSX2	ENSG00000119614.2	0	0
protein_coding	VRTN	ENSG00000133980.4	0	0
protein_coding	SYNDIG1L	ENSG00000183379.8	0	0
protein_coding	PROX2	ENSG00000119608.12	0	0
protein_coding	LRRC74A	ENSG00000100565.15	0	0
protein_coding	AC007375.1	ENSG00000259164.1	0	0
protein_coding	NGB	ENSG00000165553.4	0	0
protein_coding	NOXRED1	ENSG00000165555.9	0	0
protein_coding	C14orf178	ENSG00000197734.9	0	0
protein_coding	GPR65	ENSG00000140030.5	0	0
protein_coding	KCNK10	ENSG00000100433.15	0	0
protein_coding	CHGA	ENSG00000100604.12	0	0
protein_coding	AL110118.2	ENSG00000259066.5	0	0
protein_coding	PRIMA1	ENSG00000175785.12	0	0
protein_coding	FAM181A	ENSG00000140067.6	0	0
protein_coding	CCDC197	ENSG00000175699.14	0	0
protein_coding	SERPINA10	ENSG00000140093.9	0	0
protein_coding	SERPINA1	ENSG00000197249.13	0	0
protein_coding	SERPINA11	ENSG00000186910.3	0	0
protein_coding	SERPINA9	ENSG00000170054.14	0	0
protein_coding	SERPINA12	ENSG00000165953.9	0	0
protein_coding	SERPINA4	ENSG00000100665.11	0	0
protein_coding	SERPINA5	ENSG00000188488.13	0	0
protein_coding	AL049839.2	ENSG00000273259.3	0	0
protein_coding	TCL1B	ENSG00000213231.12	0	0

protein_coding	TCL1A	ENSG00000100721.10	0	0
protein_coding	TUNAR	ENSG00000250366.2	0	0
protein_coding	AL355102.2	ENSG00000258691.1	0	0
protein_coding	C14orf177	ENSG00000176605.8	0	0
protein_coding	SLC25A47	ENSG00000140107.10	0	0
protein_coding	DLK1	ENSG00000185559.14	0	0
protein_coding	RTL1	ENSG00000254656.2	0	0
protein_coding	LBHD2	ENSG00000283071.1	0	0
protein_coding	AL139300.1	ENSG00000256500.5	0	0
protein_coding	RD3L	ENSG00000227729.4	0	0
protein_coding	KIF26A	ENSG00000066735.14	0	0
protein_coding	C14orf180	ENSG00000184601.10	0	0
protein_coding	PLD4	ENSG00000166428.13	0	0
protein_coding	GPR132	ENSG00000183484.11	0	0
protein_coding	CRIP1	ENSG00000213145.9	0	0
protein_coding	AL928654.3	ENSG00000257341.5	0	0
protein_coding	GOLGA6L6	ENSG00000277322.1	0	0
protein_coding	POTEB2	ENSG00000230031.10	0	0
protein_coding	POTEB3	ENSG00000278522.4	0	0
protein_coding	LINC02203	ENSG00000280709.2	0	0
protein_coding	AC135068.1	ENSG00000182974.3	0	0
protein_coding	AC135068.3	ENSG00000279408.3	0	0
protein_coding	POTEB	ENSG00000233917.8	0	0
protein_coding	AC134980.3	ENSG00000285472.1	0	0
protein_coding	OR4M2	ENSG00000274102.2	0	0
protein_coding	OR4N4	ENSG00000183706.5	0	0
protein_coding	GOLGA6L22	ENSG00000277865.4	0	0
protein_coding	GOLGA6L1	ENSG00000273976.1	0	0
protein_coding	GOLGA8S	ENSG00000261739.2	0	0
protein_coding	GOLGA6L2	ENSG00000174450.11	0	0
protein_coding	MKRN3	ENSG00000179455.8	0	0
protein_coding	MAGEL2	ENSG00000254585.3	0	0
protein_coding	NPAP1	ENSG00000185823.3	0	0
protein_coding	AC124312.1	ENSG00000214265.11	0	0
protein_coding	GABRA5	ENSG00000186297.11	0	0
protein_coding	GABRG3	ENSG00000182256.12	0	0
protein_coding	OCA2	ENSG00000104044.15	0	0
protein_coding	GOLGA8F	ENSG00000153684.15	0	0
protein_coding	GOLGA8G	ENSG00000183629.13	0	0
protein_coding	GOLGA8M	ENSG00000188626.6	0	0
protein_coding	FAM189A1	ENSG00000104059.4	0	0
protein_coding	GOLGA8J	ENSG00000179938.12	0	0
protein_coding	GOLGA8T	ENSG00000261247.1	0	0
protein_coding	CHRFAM7A	ENSG00000166664.13	0	0

protein_coding	GOLGA8R	ENSG00000186399.10	0	0
protein_coding	GOLGA8Q	ENSG00000178115.11	0	0
protein_coding	GOLGA8H	ENSG00000261794.1	0	0
protein_coding	TRPM1	ENSG00000134160.13	0	0
protein_coding	GOLGA8K	ENSG00000249931.4	0	0
protein_coding	GOLGA8O	ENSG00000206127.10	0	0
protein_coding	CHRM5	ENSG00000184984.9	0	0
protein_coding	NUTM1	ENSG00000184507.15	0	0
protein_coding	GJD2	ENSG00000159248.4	0	0
protein_coding	NANOGP8	ENSG00000255192.5	0	0
protein_coding	TMCO5A	ENSG00000166069.13	0	0
protein_coding	RASGRP1	ENSG00000172575.11	0	0
protein_coding	C15orf53	ENSG00000175779.2	0	0
protein_coding	BUB1B-PAK6	ENSG00000259288.6	0	0
protein_coding	ANKRD63	ENSG00000230778.1	0	0
protein_coding	PHGR1	ENSG00000233041.8	0	0
protein_coding	AC012476.1	ENSG00000285396.1	0	0
protein_coding	PPP1R14D	ENSG00000166143.9	0	0
protein_coding	AC087721.2	ENSG00000285920.1	0	0
protein_coding	JMJD7	ENSG00000243789.10	0	0
protein_coding	JMJD7-	ENSG00000168970.22	0	0
protein_coding	PLA2G4B	ENSG00000243708.10	0	0
protein_coding	PLA2G4E	ENSG00000188089.13	0	0
protein_coding	PLA2G4F	ENSG00000168907.13	0	0
protein_coding	AC012651.1	ENSG00000258461.5	0	0
protein_coding	CAPN3	ENSG00000092529.23	0	0
protein_coding	AC018362.3	ENSG00000285942.1	0	0
protein_coding	EPB42	ENSG00000166947.13	0	0
protein_coding	TGM7	ENSG00000159495.7	0	0
protein_coding	STRC	ENSG00000242866.10	0	0
protein_coding	AC011330.3	ENSG00000284772.1	0	0
protein_coding	AC018512.1	ENSG00000262560.1	0	0
protein_coding	SERINC4	ENSG00000184716.13	0	0
protein_coding	TERB2	ENSG00000167014.10	0	0
protein_coding	DUOXA2	ENSG00000140274.13	0	0
protein_coding	SLC28A2	ENSG00000137860.11	0	0
protein_coding	AC090527.2	ENSG00000260170.1	0	0
protein_coding	SLC24A5	ENSG00000188467.10	0	0
protein_coding	CTXN2	ENSG00000233932.5	0	0
protein_coding	SLC12A1	ENSG00000074803.18	0	0
protein_coding	HDC	ENSG00000140287.10	0	0
protein_coding	SCG3	ENSG00000104112.8	0	0
protein_coding	ONECUT1	ENSG00000169856.8	0	0
protein_coding	UNC13C	ENSG00000137766.17	0	0

protein_coding	ALDH1A2	ENSG00000128918.14	0	0
protein_coding	LDHAL6B	ENSG00000171989.5	0	0
protein_coding	AC087632.1	ENSG00000259316.11	0	0
protein_coding	AC069368.1	ENSG00000249240.2	0	0
protein_coding	SLC51B	ENSG00000186198.3	0	0
protein_coding	CILP	ENSG00000138615.5	0	0
protein_coding	IGDCC3	ENSG00000174498.13	0	0
protein_coding	NOX5	ENSG00000255346.9	0	0
protein_coding	CT62	ENSG00000225362.9	0	0
protein_coding	AC009690.3	ENSG00000273025.1	0	0
protein_coding	CELF6	ENSG00000140488.15	0	0
protein_coding	AC009690.1	ENSG00000260729.1	0	0
protein_coding	TMEM202	ENSG00000187806.8	0	0
protein_coding	GOLGA6B	ENSG00000215186.6	0	0
protein_coding	HIGD2B	ENSG00000175202.4	0	0
protein_coding	HCN4	ENSG00000138622.3	0	0
protein_coding	REC114	ENSG00000183324.10	0	0
protein_coding	TBC1D21	ENSG00000167139.8	0	0
protein_coding	GOLGA6A	ENSG00000159289.6	0	0
protein_coding	CYP11A1	ENSG00000140459.17	0	0
protein_coding	CYP1A2	ENSG00000140505.6	0	0
protein_coding	LMAN1L	ENSG00000140506.16	0	0
protein_coding	CPLX3	ENSG00000213578.5	0	0
protein_coding	GOLGA6C	ENSG00000167195.7	0	0
protein_coding	GOLGA6D	ENSG00000140478.15	0	0
protein_coding	LINGO1	ENSG00000169783.12	0	0
protein_coding	SH2D7	ENSG00000183476.12	0	0
protein_coding	CRABP1	ENSG00000166426.7	0	0
protein_coding	CHRNA3	ENSG00000080644.15	0	0
protein_coding	ANKRD34C	ENSG00000235711.4	0	0
protein_coding	ST20-MTHFS	ENSG00000259332.3	0	0
protein_coding	TMC3	ENSG00000188869.12	0	0
protein_coding	AC245033.1	ENSG00000260836.2	0	0
protein_coding	AP3B2	ENSG00000103723.13	0	0
protein_coding	TM6SF1	ENSG00000136404.15	0	0
protein_coding	ADAMTSL3	ENSG00000156218.12	0	0
protein_coding	GOLGA6L4	ENSG00000184206.11	0	0
protein_coding	SLC28A1	ENSG00000156222.11	0	0
protein_coding	AGBL1	ENSG00000273540.3	0	0
protein_coding	AC013489.1	ENSG00000173867.10	0	0
protein_coding	RLBP1	ENSG00000140522.11	0	0
protein_coding	MESP1	ENSG00000166823.5	0	0
protein_coding	MESP2	ENSG00000188095.5	0	0
protein_coding	C15orf38-	ENSG00000250021.7	0	0

protein_coding	AC091167.7	ENSG00000284626.1	0	0
protein_coding	TTLL13P	ENSG00000213471.10	0	0
protein_coding	AC091167.2	ENSG00000261147.1	0	0
protein_coding	AC091167.6	ENSG00000275674.1	0	0
protein_coding	AC068831.7	ENSG00000284946.1	0	0
protein_coding	SV2B	ENSG00000185518.11	0	0
protein_coding	SPATA8	ENSG00000185594.5	0	0
protein_coding	PGPEP1L	ENSG00000183571.10	0	0
protein_coding	OR4F6	ENSG00000184140.6	0	0
protein_coding	OR4F15	ENSG00000182854.8	0	0
protein_coding	OR4F4	ENSG00000177693.4	0	0
protein_coding	HBZ	ENSG00000130656.4	0	0
protein_coding	HBM	ENSG00000206177.6	0	0
protein_coding	HBA2	ENSG00000188536.13	0	0
protein_coding	HBA1	ENSG00000206172.8	0	0
protein_coding	HBQ1	ENSG00000086506.2	0	0
protein_coding	ARHGDIG	ENSG00000242173.8	0	0
protein_coding	PRR35	ENSG00000161992.5	0	0
protein_coding	NHLRC4	ENSG00000257108.1	0	0
protein_coding	WFIKKN1	ENSG00000127578.6	0	0
protein_coding	FBXL16	ENSG00000127585.11	0	0
protein_coding	MSLN	ENSG00000102854.15	0	0
protein_coding	MSLNL	ENSG00000162006.9	0	0
protein_coding	GNG13	ENSG00000127588.4	0	0
protein_coding	SSTR5	ENSG00000162009.8	0	0
protein_coding	C1QTNF8	ENSG00000184471.7	0	0
protein_coding	CACNA1H	ENSG00000196557.12	0	0
protein_coding	TPSG1	ENSG00000116176.6	0	0
protein_coding	TPSB2	ENSG00000197253.13	0	0
protein_coding	TPSAB1	ENSG00000172236.16	0	0
protein_coding	TPSD1	ENSG00000095917.13	0	0
protein_coding	AL032819.3	ENSG00000284395.1	0	0
protein_coding	PTX4	ENSG00000251692.7	0	0
protein_coding	IGFALS	ENSG00000099769.5	0	0
protein_coding	MEIOB	ENSG00000162039.15	0	0
protein_coding	RNF151	ENSG00000179580.9	0	0
protein_coding	NOXO1	ENSG00000196408.11	0	0
protein_coding	CASKIN1	ENSG00000167971.15	0	0
protein_coding	NTN3	ENSG00000162068.1	0	0
protein_coding	AC093525.2	ENSG00000260272.1	0	0
protein_coding	ATP6V0C	ENSG00000185883.11	0	0
protein_coding	AC093525.1	ENSG00000259784.1	0	0
protein_coding	CEMP1	ENSG00000205923.3	0	0
protein_coding	PRSS33	ENSG00000103355.13	0	0

protein_coding	PRSS41	ENSG00000215148.8	0	0
protein_coding	PRSS21	ENSG00000007038.10	0	0
protein_coding	AC004233.2	ENSG00000270168.2	0	0
protein_coding	CLDN9	ENSG00000213937.3	0	0
protein_coding	CLDN6	ENSG00000184697.6	0	0
protein_coding	MMP25	ENSG00000008516.16	0	0
protein_coding	ZSCAN10	ENSG00000130182.7	0	0
protein_coding	MEFV	ENSG00000103313.12	0	0
protein_coding	OR2C1	ENSG00000168158.3	0	0
protein_coding	AC025283.3	ENSG00000285329.1	0	0
protein_coding	C16orf90	ENSG00000215131.10	0	0
protein_coding	NLRC3	ENSG00000167984.17	0	0
protein_coding	SRL	ENSG00000185739.13	0	0
protein_coding	CORO7-	ENSG00000103426.12	0	0
protein_coding	Sep-12	ENSG00000140623.13	0	0
protein_coding	C16orf89	ENSG00000153446.15	0	0
protein_coding	RBFOX1	ENSG00000078328.20	0	0
protein_coding	TMEM114	ENSG00000232258.6	0	0
protein_coding	AC022167.5	ENSG00000283516.1	0	0
protein_coding	TEKT5	ENSG00000153060.7	0	0
protein_coding	TVP23A	ENSG00000166676.15	0	0
protein_coding	TNP2	ENSG00000178279.3	0	0
protein_coding	PRM3	ENSG00000178257.3	0	0
protein_coding	PRM2	ENSG00000122304.10	0	0
protein_coding	PRM1	ENSG00000175646.3	0	0
protein_coding	NPIPA3	ENSG00000224712.12	0	0
protein_coding	NPIPA2	ENSG00000254852.8	0	0
protein_coding	AC140504.1	ENSG00000261130.5	0	0
protein_coding	NOMO3	ENSG00000103226.17	0	0
protein_coding	PKD1P1	ENSG00000183889.12	0	0
protein_coding	NPIPA7	ENSG00000214967.5	0	0
protein_coding	NPIPA8	ENSG00000214940.8	0	0
protein_coding	AC126755.2	ENSG00000233024.7	0	0
protein_coding	NOMO2	ENSG00000185164.14	0	0
protein_coding	AC138811.2	ENSG00000260342.2	0	0
protein_coding	GPR139	ENSG00000180269.7	0	0
protein_coding	GP2	ENSG00000169347.16	0	0
protein_coding	UMOD	ENSG00000169344.15	0	0
protein_coding	PDILT	ENSG00000169340.9	0	0
protein_coding	ACSM5	ENSG00000183549.10	0	0
protein_coding	ACSM2A	ENSG00000183747.11	0	0
protein_coding	ACSM2B	ENSG00000066813.14	0	0
protein_coding	ACSM1	ENSG00000166743.9	0	0
protein_coding	ZP2	ENSG00000103310.10	0	0

protein_coding	NPIPB3	ENSG00000169246.16	0	0
protein_coding	IGSF6	ENSG00000140749.8	0	0
protein_coding	PDZD9	ENSG00000155714.13	0	0
protein_coding	VWA3A	ENSG00000175267.14	0	0
protein_coding	NPIPB5	ENSG00000243716.10	0	0
protein_coding	ERN¬†2	ENSG00000134398.14	0	0
protein_coding	CHP2	ENSG00000166869.2	0	0
protein_coding	PRKCB	ENSG00000166501.13	0	0
protein_coding	CACNG3	ENSG00000006116.3	0	0
protein_coding	SLC5A11	ENSG00000158865.12	0	0
protein_coding	AQP8	ENSG00000103375.10	0	0
protein_coding	HS3ST4	ENSG00000182601.6	0	0
protein_coding	GSG1L	ENSG00000169181.12	0	0
protein_coding	NPIPB6	ENSG00000198156.10	0	0
protein_coding	AC138894.1	ENSG00000261832.6	0	0
protein_coding	NPIPB7	ENSG00000233232.6	0	0
protein_coding	APOBR	ENSG00000184730.10	0	0
protein_coding	IL27	ENSG00000197272.2	0	0
protein_coding	NPIPB8	ENSG00000255524.7	0	0
protein_coding	NPIPB9	ENSG00000196993.8	0	0
protein_coding	ATP2A1	ENSG00000196296.13	0	0
protein_coding	LAT	ENSG00000213658.11	0	0
protein_coding	NPIPB11	ENSG00000254206.5	0	0
protein_coding	BOLA2	ENSG00000183336.8	0	0
protein_coding	SLX1B	ENSG00000181625.17	0	0
protein_coding	SULT1A4	ENSG00000213648.10	0	0
protein_coding	NPIPB12	ENSG00000169203.16	0	0
protein_coding	AC009086.2	ENSG00000277669.1	0	0
protein_coding	AC009133.6	ENSG00000280893.1	0	0
protein_coding	PAGR1	ENSG00000280789.1	0	0
protein_coding	AC120114.4	ENSG00000281348.1	0	0
protein_coding	C16orf92	ENSG00000167194.7	0	0
protein_coding	AC093512.2	ENSG00000285043.1	0	0
protein_coding	ALDOA	ENSG00000149925.18	0	0
protein_coding	SLX1A	ENSG00000132207.17	0	0
protein_coding	SULT1A3	ENSG00000261052.5	0	0
protein_coding	NPIPB13	ENSG00000198064.13	0	0
protein_coding	ITGAL	ENSG00000005844.17	0	0
protein_coding	AC002310.5	ENSG00000261459.1	0	0
protein_coding	AC002310.4	ENSG00000260869.1	0	0
protein_coding	TMEM265	ENSG00000281991.1	0	0
protein_coding	STX1B	ENSG00000099365.10	0	0
protein_coding	PRSS53	ENSG00000151006.7	0	0
protein_coding	AC135050.2	ENSG00000255439.6	0	0

protein_coding	TRIM72	ENSG00000177238.13	0	0
protein_coding	PYDC1	ENSG00000169900.7	0	0
protein_coding	ITGAD	ENSG00000156886.11	0	0
protein_coding	COX6A2	ENSG00000156885.5	0	0
protein_coding	SLC5A2	ENSG00000140675.12	0	0
protein_coding	AHSP	ENSG00000169877.9	0	0
protein_coding	TP53TG3D	ENSG00000205456.11	0	0
protein_coding	TP53TG3	ENSG00000183632.14	0	0
protein_coding	TP53TG3C	ENSG00000205457.11	0	0
protein_coding	TP53TG3E	ENSG00000275034.2	0	0
protein_coding	TP53TG3B	ENSG00000261509.6	0	0
protein_coding	TP53TG3F	ENSG00000278848.2	0	0
protein_coding	AC136428.1	ENSG00000259680.5	0	0
protein_coding	ABCC12	ENSG00000140798.16	0	0
protein_coding	CBLN1	ENSG00000102924.11	0	0
protein_coding	C16orf78	ENSG00000166152.3	0	0
protein_coding	SNX20	ENSG00000167208.14	0	0
protein_coding	TOX3	ENSG00000103460.16	0	0
protein_coding	IRX6	ENSG00000159387.7	0	0
protein_coding	CES1	ENSG00000198848.12	0	0
protein_coding	CES5A	ENSG00000159398.15	0	0
protein_coding	MT4	ENSG00000102891.3	0	0
protein_coding	MT3	ENSG00000087250.8	0	0
protein_coding	MT1G	ENSG00000125144.13	0	0
protein_coding	MT1H	ENSG00000205358.3	0	0
protein_coding	SLC12A3	ENSG00000070915.9	0	0
protein_coding	CETP	ENSG00000087237.11	0	0
protein_coding	CX3CL1	ENSG00000006210.6	0	0
protein_coding	ADGRG5	ENSG00000159618.15	0	0
protein_coding	ADGRG3	ENSG00000182885.16	0	0
protein_coding	DRC7	ENSG00000159625.14	0	0
protein_coding	TEPP	ENSG00000159648.11	0	0
protein_coding	PRSS54	ENSG00000103023.11	0	0
protein_coding	AC018554.3	ENSG00000278499.2	0	0
protein_coding	CDH5	ENSG00000179776.18	0	0
protein_coding	AC010542.3	ENSG00000260851.6	0	0
protein_coding	CKLF-	ENSG00000254788.7	0	0
protein_coding	CMTM2	ENSG00000140932.9	0	0
protein_coding	TERB1	ENSG00000249961.9	0	0
protein_coding	CA7	ENSG00000168748.13	0	0
protein_coding	CDH16	ENSG00000166589.12	0	0
protein_coding	CES3	ENSG00000172828.12	0	0
protein_coding	AC074143.1	ENSG00000265690.7	0	0
protein_coding	EXOC3L1	ENSG00000179044.15	0	0

protein_coding	AGRP	ENSG00000159723.4	0	0
protein_coding	CARMIL2	ENSG00000159753.13	0	0
protein_coding	NRN1L	ENSG00000188038.7	0	0
protein_coding	CTRL	ENSG00000141086.17	0	0
protein_coding	DPEP3	ENSG00000141096.4	0	0
protein_coding	DPEP2	ENSG00000167261.13	0	0
protein_coding	DPEP2NB	ENSG00000263201.1	0	0
protein_coding	AC026464.4	ENSG00000260914.3	0	0
protein_coding	PDF	ENSG00000258429.1	0	0
protein_coding	AC026464.6	ENSG00000272617.3	0	0
protein_coding	AC026464.3	ENSG00000260371.1	0	0
protein_coding	AC026464.1	ENSG00000259900.5	0	0
protein_coding	CLEC18A	ENSG00000157322.17	0	0
protein_coding	CLEC18C	ENSG00000157335.20	0	0
protein_coding	AC012184.2	ENSG00000260537.2	0	0
protein_coding	TLE7	ENSG00000260734.5	0	0
protein_coding	ZNF23	ENSG00000167377.18	0	0
protein_coding	AC010547.4	ENSG00000261611.6	0	0
protein_coding	CHST4	ENSG00000140835.9	0	0
protein_coding	TAT	ENSG00000198650.10	0	0
protein_coding	HP	ENSG00000257017.8	0	0
protein_coding	HPR	ENSG00000261701.7	0	0
protein_coding	CTRB2	ENSG00000168928.12	0	0
protein_coding	CTRB1	ENSG00000168925.11	0	0
protein_coding	AC009163.4	ENSG00000261717.5	0	0
protein_coding	AC009163.2	ENSG00000260092.1	0	0
protein_coding	CHST5	ENSG00000135702.14	0	0
protein_coding	AC025287.4	ENSG00000284484.1	0	0
protein_coding	DUXB	ENSG00000282757.3	0	0
protein_coding	CPHXL	ENSG00000283755.1	0	0
protein_coding	CNTNAP4	ENSG00000152910.18	0	0
protein_coding	AC104151.1	ENSG00000261833.2	0	0
protein_coding	ADAMTS18	ENSG00000140873.15	0	0
protein_coding	CLEC3A	ENSG00000166509.10	0	0
protein_coding	DYNLRB2	ENSG00000168589.14	0	0
protein_coding	AC092718.8	ENSG00000284512.1	0	0
protein_coding	DNAAF1	ENSG00000154099.17	0	0
protein_coding	ADAD2	ENSG00000140955.10	0	0
protein_coding	KCNG4	ENSG00000168418.7	0	0
protein_coding	FAM92B	ENSG00000153789.12	0	0
protein_coding	IRF8	ENSG00000140968.10	0	0
protein_coding	AC010531.1	ENSG00000131152.4	0	0
protein_coding	CA5A	ENSG00000174990.5	0	0
protein_coding	IL17C	ENSG00000124391.4	0	0

protein_coding	PABPN1L	ENSG00000205022.9	0	0
protein_coding	CBFA2T3	ENSG00000129993.14	0	0
protein_coding	SLC22A31	ENSG00000259803.6	0	0
protein_coding	DPEP1	ENSG00000015413.9	0	0
protein_coding	AC092143.1	ENSG00000198211.8	0	0
protein_coding	TUBB3	ENSG00000258947.6	0	0
protein_coding	PRDM7	ENSG00000126856.14	0	0
protein_coding	SCGB1C2	ENSG00000268320.3	0	0
protein_coding	DOC2B	ENSG00000272636.3	0	0
protein_coding	BHLHA9	ENSG00000205899.3	0	0
protein_coding	TRARG1	ENSG00000184811.3	0	0
protein_coding	OVCA2	ENSG00000262664.2	0	0
protein_coding	OR1D5	ENSG00000262628.1	0	0
protein_coding	OR1D2	ENSG00000184166.2	0	0
protein_coding	OR1G1	ENSG00000183024.3	0	0
protein_coding	OR1A2	ENSG00000172150.4	0	0
protein_coding	OR1A1	ENSG00000172146.2	0	0
protein_coding	OR3A1	ENSG00000180090.5	0	0
protein_coding	AC087498.1	ENSG00000180042.5	0	0
protein_coding	OR1E1	ENSG00000180016.2	0	0
protein_coding	OR3A3	ENSG00000159961.2	0	0
protein_coding	OR1E2	ENSG00000127780.3	0	0
protein_coding	SPATA22	ENSG00000141255.12	0	0
protein_coding	AC027796.3	ENSG00000262304.2	0	0
protein_coding	SPNS3	ENSG00000182557.7	0	0
protein_coding	ALOX15	ENSG00000161905.12	0	0
protein_coding	TM4SF5	ENSG00000142484.6	0	0
protein_coding	GLTPD2	ENSG00000182327.7	0	0
protein_coding	GP1BA	ENSG00000185245.7	0	0
protein_coding	AC007846.2	ENSG00000285471.1	0	0
protein_coding	SLC13A5	ENSG00000141485.16	0	0
protein_coding	FBXO39	ENSG00000177294.6	0	0
protein_coding	TEKT1	ENSG00000167858.12	0	0
protein_coding	ALOX12	ENSG00000108839.11	0	0
protein_coding	RNASEK-C17orf49	ENSG00000161939.19	0	0
protein_coding	C17orf49	ENSG00000258315.5	0	0
protein_coding	BCL6B	ENSG00000161940.10	0	0
protein_coding	CLEC10A	ENSG00000132514.13	0	0
protein_coding	ASGR2	ENSG00000161944.16	0	0
protein_coding	AC120057.2	ENSG00000262526.2	0	0
protein_coding	AC003688.1	ENSG00000262302.1	0	0
protein_coding	YBX2	ENSG00000006047.12	0	0

protein_coding	AC026954.2	ENSG00000261915.6	0	0
protein_coding	NEURL4	ENSG00000215041.9	0	0
protein_coding	TMEM95	ENSG00000182896.12	0	0
protein_coding	PLSCR3	ENSG00000187838.16	0	0
protein_coding	TMEM256-PLSCR3	ENSG00000262481.5	0	0
protein_coding	SPEM1	ENSG00000181323.7	0	0
protein_coding	SPEM2	ENSG00000184560.7	0	0
protein_coding	SPEM3	ENSG00000283439.2	0	0
protein_coding	TMEM102	ENSG00000181284.2	0	0
protein_coding	FGF11	ENSG00000161958.10	0	0
protein_coding	SLC35G6	ENSG00000259224.2	0	0
protein_coding	TNFSF12-TNFSF13	ENSG00000248871.1	0	0
protein_coding	TNFSF13	ENSG00000161955.16	0	0
protein_coding	SENP3-	ENSG00000277957.1	0	0
protein_coding	CYB5D1	ENSG00000182224.11	0	0
protein_coding	AC104581.2	ENSG00000262730.1	0	0
protein_coding	GUCY2D	ENSG00000132518.6	0	0
protein_coding	ALOX12B	ENSG00000179477.10	0	0
protein_coding	ALOXE3	ENSG00000179148.9	0	0
protein_coding	AC129492.3	ENSG00000263620.1	0	0
protein_coding	ARHGEF15	ENSG00000198844.11	0	0
protein_coding	ODF4	ENSG00000184650.10	0	0
protein_coding	AC135178.2	ENSG00000263809.1	0	0
protein_coding	CCDC42	ENSG00000161973.10	0	0
protein_coding	SPDYE4	ENSG00000183318.11	0	0
protein_coding	MFSD6L	ENSG00000185156.5	0	0
protein_coding	PIK3R6	ENSG00000276231.4	0	0
protein_coding	PIK3R5	ENSG00000141506.13	0	0
protein_coding	DHRS7C	ENSG00000184544.11	0	0
protein_coding	GSG1L2	ENSG00000214978.7	0	0
protein_coding	RCVRN	ENSG00000109047.7	0	0
protein_coding	MYH13	ENSG00000006788.13	0	0
protein_coding	MYH8	ENSG00000133020.4	0	0
protein_coding	MYH4	ENSG00000264424.1	0	0
protein_coding	MYH1	ENSG00000109061.9	0	0
protein_coding	MYH2	ENSG00000125414.18	0	0
protein_coding	TMEM238L	ENSG00000263429.3	0	0
protein_coding	PIRT	ENSG00000233670.6	0	0
protein_coding	SHISA6	ENSG00000188803.14	0	0
protein_coding	DNAH9	ENSG00000007174.17	0	0
protein_coding	CDRT15	ENSG00000223510.6	0	0

protein_coding	TEKT3	ENSG00000125409.12	0	0
protein_coding	TVP23C-	ENSG00000259024.6	0	0
protein_coding	AC005324.3	ENSG00000251537.4	0	0
protein_coding	AC005324.4	ENSG00000255104.8	0	0
protein_coding	TBC1D26	ENSG00000214946.14	0	0
protein_coding	AC098850.3	ENSG00000266302.6	0	0
protein_coding	TNFRSF13B	ENSG00000240505.8	0	0
protein_coding	AC055811.2	ENSG00000264187.1	0	0
protein_coding	TBC1D28	ENSG00000189375.10	0	0
protein_coding	SLC5A10	ENSG00000154025.15	0	0
protein_coding	GRAPL	ENSG00000189152.10	0	0
protein_coding	CDRT15L2	ENSG00000214819.1	0	0
protein_coding	CCDC144NL	ENSG00000205212.4	0	0
protein_coding	KCNJ18	ENSG00000260458.3	0	0
protein_coding	AC015688.4	ENSG00000266728.5	0	0
protein_coding	NOS2	ENSG00000007171.17	0	0
protein_coding	AC005697.1	ENSG00000266202.1	0	0
protein_coding	SEBOX	ENSG00000274529.5	0	0
protein_coding	AC002094.3	ENSG00000273171.1	0	0
protein_coding	SLC13A2	ENSG00000007216.14	0	0
protein_coding	SEZ6	ENSG00000063015.19	0	0
protein_coding	SLC6A4	ENSG00000108576.9	0	0
protein_coding	TBC1D29	ENSG00000266733.6	0	0
protein_coding	AC134669.1	ENSG00000265118.5	0	0
protein_coding	ASIC2	ENSG00000108684.14	0	0
protein_coding	CCL11	ENSG00000172156.3	0	0
protein_coding	CCL1	ENSG00000108702.3	0	0
protein_coding	TMEM132E	ENSG00000181291.7	0	0
protein_coding	RAD51L3-	ENSG00000267618.5	0	0
protein_coding	FNDC8	ENSG00000073598.5	0	0
protein_coding	UNC45B	ENSG00000141161.11	0	0
protein_coding	SLC35G3	ENSG00000164729.7	0	0
protein_coding	SLFN13	ENSG00000154760.13	0	0
protein_coding	SLFN14	ENSG00000236320.3	0	0
protein_coding	GAS2L2	ENSG00000270765.5	0	0
protein_coding	C17orf50	ENSG00000270806.1	0	0
protein_coding	HEATR9	ENSG00000270379.5	0	0
protein_coding	LYZL6	ENSG00000275722.4	0	0
protein_coding	CCL16	ENSG00000275152.4	0	0
protein_coding	CCL14	ENSG00000276409.4	0	0
protein_coding	CCL15-	ENSG00000275688.4	0	0
protein_coding	CCL15	ENSG00000275718.1	0	0
protein_coding	CCL23	ENSG00000274736.4	0	0
protein_coding	CCL18	ENSG00000275385.1	0	0

protein_coding	CCL3	ENSG00000277632.1	0	0
protein_coding	CCL4	ENSG00000275302.1	0	0
protein_coding	TBC1D3B	ENSG00000274808.5	0	0
protein_coding	CCL3L1	ENSG00000276085.1	0	0
protein_coding	CCL4L2	ENSG00000276070.4	0	0
protein_coding	TBC1D3G	ENSG00000260287.4	0	0
protein_coding	TBC1D3H	ENSG00000274226.5	0	0
protein_coding	TBC1D3F	ENSG00000275954.5	0	0
protein_coding	C17orf78	ENSG00000278505.4	0	0
protein_coding	HNF1B	ENSG00000275410.4	0	0
protein_coding	TBC1D3K	ENSG00000273513.1	0	0
protein_coding	TBC1D3L	ENSG00000274512.5	0	0
protein_coding	TBC1D3D	ENSG00000274419.6	0	0
protein_coding	TBC1D3C	ENSG00000278299.5	0	0
protein_coding	TBC1D3E	ENSG00000278599.5	0	0
protein_coding	TBC1D3	ENSG00000274611.3	0	0
protein_coding	GPR179	ENSG00000277399.4	0	0
protein_coding	C17orf98	ENSG00000275489.1	0	0
protein_coding	FBXO47	ENSG00000204952.2	0	0
protein_coding	ARL5C	ENSG00000141748.12	0	0
protein_coding	PPP1R1B	ENSG00000131771.13	0	0
protein_coding	PNMT	ENSG00000141744.3	0	0
protein_coding	ZPBP2	ENSG00000186075.12	0	0
protein_coding	LRRC3C	ENSG00000204913.5	0	0
protein_coding	CSF3	ENSG00000108342.12	0	0
protein_coding	GJD3	ENSG00000183153.6	0	0
protein_coding	AC073508.2	ENSG00000264058.2	0	0
protein_coding	KRT24	ENSG00000167916.4	0	0
protein_coding	KRT25	ENSG00000204897.6	0	0
protein_coding	KRT26	ENSG00000186393.5	0	0
protein_coding	KRT27	ENSG00000171446.6	0	0
protein_coding	KRT28	ENSG00000173908.8	0	0
protein_coding	KRT12	ENSG00000187242.5	0	0
protein_coding	KRT23	ENSG00000108244.16	0	0
protein_coding	KRT39	ENSG00000196859.7	0	0
protein_coding	KRT40	ENSG00000204889.10	0	0
protein_coding	KRTAP3-3	ENSG00000212899.2	0	0
protein_coding	KRTAP3-2	ENSG00000212900.2	0	0
protein_coding	KRTAP3-1	ENSG00000212901.3	0	0
protein_coding	KRTAP1-4	ENSG00000204887.4	0	0
protein_coding	KRTAP1-3	ENSG00000221880.3	0	0
protein_coding	KRTAP1-1	ENSG00000188581.8	0	0
protein_coding	KRTAP2-1	ENSG00000212725.3	0	0
protein_coding	KRTAP2-2	ENSG00000214518.3	0	0

protein_coding	KRTAP2-4	ENSG00000213417.3	0	0
protein_coding	KRTAP4-7	ENSG00000240871.5	0	0
protein_coding	KRTAP4-8	ENSG00000204880.7	0	0
protein_coding	KRTAP4-16	ENSG00000241241.1	0	0
protein_coding	KRTAP4-9	ENSG00000212722.7	0	0
protein_coding	KRTAP4-11	ENSG00000212721.3	0	0
protein_coding	KRTAP4-12	ENSG00000213416.3	0	0
protein_coding	KRTAP4-6	ENSG00000198090.3	0	0
protein_coding	KRTAP4-5	ENSG00000198271.4	0	0
protein_coding	KRTAP4-4	ENSG00000171396.11	0	0
protein_coding	KRTAP4-3	ENSG00000196156.4	0	0
protein_coding	KRTAP4-2	ENSG00000244537.2	0	0
protein_coding	KRTAP9-1	ENSG00000240542.4	0	0
protein_coding	KRTAP9-2	ENSG00000239886.5	0	0
protein_coding	KRTAP9-3	ENSG00000204873.4	0	0
protein_coding	KRTAP9-8	ENSG00000187272.6	0	0
protein_coding	KRTAP9-4	ENSG00000241595.2	0	0
protein_coding	KRTAP9-9	ENSG00000198083.9	0	0
protein_coding	KRTAP9-6	ENSG00000212659.1	0	0
protein_coding	KRTAP9-7	ENSG00000180386.7	0	0
protein_coding	KRTAP29-1	ENSG00000212658.1	0	0
protein_coding	KRTAP16-1	ENSG00000212657.1	0	0
protein_coding	KRTAP17-1	ENSG00000186860.4	0	0
protein_coding	KRT33A	ENSG00000006059.3	0	0
protein_coding	KRT37	ENSG00000108417.3	0	0
protein_coding	KRT38	ENSG00000171360.3	0	0
protein_coding	KRT35	ENSG00000197079.8	0	0
protein_coding	KRT36	ENSG00000126337.13	0	0
protein_coding	AC099811.2	ENSG00000267261.5	0	0
protein_coding	HSPB9	ENSG00000260325.1	0	0
protein_coding	RAMP2	ENSG00000131477.10	0	0
protein_coding	CNTD1	ENSG00000176563.9	0	0
protein_coding	G6PC	ENSG00000131482.9	0	0
protein_coding	PTGES3L-AARSD1	ENSG00000108825.17	0	0
protein_coding	CCDC200	ENSG00000236383.8	0	0
protein_coding	MEOX1	ENSG00000005102.12	0	0
protein_coding	SOST	ENSG00000167941.2	0	0
protein_coding	CFAP97D1	ENSG00000231256.7	0	0
protein_coding	CD300LG	ENSG00000161649.12	0	0
protein_coding	PPY	ENSG00000108849.7	0	0
protein_coding	PYY	ENSG00000131096.10	0	0
protein_coding	ASB16	ENSG00000161664.6	0	0

protein_coding	SLC4A1	ENSG00000004939.14	0	0
protein_coding	HIGD1B	ENSG00000131097.6	0	0
protein_coding	CCDC103	ENSG00000167131.16	0	0
protein_coding	FAM187A	ENSG00000214447.4	0	0
protein_coding	SPATA32	ENSG00000184361.12	0	0
protein_coding	LINC02210-CRHR1	ENSG00000263715.7	0	0
protein_coding	CRHR1	ENSG00000120088.14	0	0
protein_coding	SPPL2C	ENSG00000185294.6	0	0
protein_coding	STH	ENSG00000256762.1	0	0
protein_coding	ARL17A	ENSG00000185829.17	0	0
protein_coding	WNT9B	ENSG00000158955.10	0	0
protein_coding	AC005670.2	ENSG00000262633.2	0	0
protein_coding	RPRML	ENSG00000179673.4	0	0
protein_coding	MYL4	ENSG00000198336.9	0	0
protein_coding	AC068234.1	ENSG00000259753.1	0	0
protein_coding	TBX21	ENSG00000073861.2	0	0
protein_coding	PRR15L	ENSG00000167183.2	0	0
protein_coding	SKAP1	ENSG00000141293.15	0	0
protein_coding	HOXB1	ENSG00000120094.7	0	0
protein_coding	HOXB2	ENSG00000173917.10	0	0
protein_coding	HOXB8	ENSG00000120068.6	0	0
protein_coding	PRAC1	ENSG00000159182.4	0	0
protein_coding	PRAC2	ENSG00000229637.3	0	0
protein_coding	TTLL6	ENSG00000170703.15	0	0
protein_coding	GIP	ENSG00000159224.4	0	0
protein_coding	B4GALNT2	ENSG00000167080.8	0	0
protein_coding	PHOSPHO1	ENSG00000173868.11	0	0
protein_coding	TAC4	ENSG00000176358.15	0	0
protein_coding	CHAD	ENSG00000136457.9	0	0
protein_coding	ANKRD40CL	ENSG00000167117.9	0	0
protein_coding	WFIKKN2	ENSG00000173714.7	0	0
protein_coding	NME1-NME2	ENSG00000011052.21	0	0
protein_coding	CA10	ENSG00000154975.13	0	0
protein_coding	KIF2B	ENSG00000141200.7	0	0
protein_coding	HLF	ENSG00000108924.13	0	0
protein_coding	SMIM36	ENSG00000261873.2	0	0
protein_coding	CCDC182	ENSG00000166329.2	0	0
protein_coding	AC015813.2	ENSG00000266086.2	0	0
protein_coding	OR4D1	ENSG00000141194.6	0	0
protein_coding	OR4D2	ENSG00000255713.2	0	0
protein_coding	EPX	ENSG00000121053.5	0	0
protein_coding	LPO	ENSG00000167419.10	0	0

protein_coding	MPO	ENSG00000005381.7	0	0
protein_coding	SUPT4H1	ENSG00000213246.6	0	0
protein_coding	AC004687.2	ENSG00000285897.1	0	0
protein_coding	HSF5	ENSG00000176160.10	0	0
protein_coding	C17orf47	ENSG00000181013.3	0	0
protein_coding	AC099850.2	ENSG00000265303.1	0	0
protein_coding	AC005702.1	ENSG00000267318.1	0	0
protein_coding	CA4	ENSG00000167434.9	0	0
protein_coding	EFCAB3	ENSG00000172421.9	0	0
protein_coding	MARCH10	ENSG00000173838.11	0	0
protein_coding	KCNH6	ENSG00000173826.14	0	0
protein_coding	AC046185.1	ENSG00000125695.12	0	0
protein_coding	CSH2	ENSG00000213218.10	0	0
protein_coding	GH2	ENSG00000136487.17	0	0
protein_coding	CSH1	ENSG00000136488.14	0	0
protein_coding	CSHL1	ENSG00000204414.12	0	0
protein_coding	GH1	ENSG00000259384.6	0	0
protein_coding	AC127029.3	ENSG00000285947.1	0	0
protein_coding	CD79B	ENSG00000007312.12	0	0
protein_coding	SCN4A	ENSG00000007314.12	0	0
protein_coding	AC004805.1	ENSG00000266076.1	0	0
protein_coding	APOH	ENSG00000091583.10	0	0
protein_coding	CACNG5	ENSG00000075429.8	0	0
protein_coding	CACNG1	ENSG00000108878.4	0	0
protein_coding	KCNJ16	ENSG00000153822.13	0	0
protein_coding	CPSF4L	ENSG00000187959.9	0	0
protein_coding	KIF19	ENSG00000196169.14	0	0
protein_coding	BTBD17	ENSG00000204347.3	0	0
protein_coding	GPR142	ENSG00000257008.6	0	0
protein_coding	CD300LB	ENSG00000178789.8	0	0
protein_coding	CD300C	ENSG00000167850.3	0	0
protein_coding	CD300LD	ENSG00000204345.1	0	0
protein_coding	CD300E	ENSG00000186407.6	0	0
protein_coding	CD300LF	ENSG00000186074.18	0	0
protein_coding	OTOP2	ENSG00000183034.12	0	0
protein_coding	OTOP3	ENSG00000182938.5	0	0
protein_coding	MYO15B	ENSG00000266714.8	0	0
protein_coding	SMIM5	ENSG00000204323.5	0	0
protein_coding	MRPL38	ENSG00000204316.12	0	0
protein_coding	AC087289.3	ENSG00000267426.5	0	0
protein_coding	TEN1	ENSG00000257949.6	0	0
protein_coding	TEN1-CDK3	ENSG00000261408.7	0	0
protein_coding	CDK3	ENSG00000250506.7	0	0
protein_coding	ZACN	ENSG00000186919.12	0	0

protein_coding	FOXJ1	ENSG00000129654.7	0	0
protein_coding	AANAT	ENSG00000129673.9	0	0
protein_coding	AC005837.2	ENSG00000267168.1	0	0
protein_coding	TMEM235	ENSG00000204278.12	0	0
protein_coding	CEP295NL	ENSG00000178404.9	0	0
protein_coding	RBFOX3	ENSG00000167281.19	0	0
protein_coding	ENPP7	ENSG00000182156.9	0	0
protein_coding	PVALEF	ENSG00000225180.7	0	0
protein_coding	FSCN2	ENSG00000186765.11	0	0
protein_coding	AC139530.2	ENSG00000262660.1	0	0
protein_coding	GCGR	ENSG00000215644.9	0	0
protein_coding	PPP1R27	ENSG00000182676.4	0	0
protein_coding	MYADML2	ENSG00000185105.5	0	0
protein_coding	UTS2R	ENSG00000181408.3	0	0
protein_coding	TUBB8P12	ENSG00000173213.9	0	0
protein_coding	CETN1	ENSG00000177143.4	0	0
protein_coding	ADCYAP1	ENSG00000141433.12	0	0
protein_coding	AKAIN1	ENSG00000231824.3	0	0
protein_coding	EPB41L3	ENSG00000082397.17	0	0
protein_coding	L3MBTL4	ENSG00000154655.15	0	0
protein_coding	LRRC30	ENSG00000206422.2	0	0
protein_coding	SLC35G4	ENSG00000236396.8	0	0
protein_coding	ANKRD62	ENSG00000181626.11	0	0
protein_coding	CIDEA	ENSG00000176194.17	0	0
protein_coding	MC5R	ENSG00000176136.6	0	0
protein_coding	MC2R	ENSG00000185231.4	0	0
protein_coding	POTEC	ENSG00000183206.17	0	0
protein_coding	CTAGE1	ENSG00000212710.4	0	0
protein_coding	PSMA8	ENSG00000154611.14	0	0
protein_coding	AQP4	ENSG00000171885.14	0	0
protein_coding	CHST9	ENSG00000154080.13	0	0
protein_coding	DSG4	ENSG00000175065.11	0	0
protein_coding	TTR	ENSG00000118271.10	0	0
protein_coding	SLC25A52	ENSG00000141437.8	0	0
protein_coding	MEP1B	ENSG00000141434.11	0	0
protein_coding	KLHL14	ENSG00000197705.9	0	0
protein_coding	CCDC178	ENSG00000166960.16	0	0
protein_coding	AC007998.2	ENSG00000267140.1	0	0
protein_coding	CELF4	ENSG00000101489.19	0	0
protein_coding	RIT2	ENSG00000152214.13	0	0
protein_coding	SYT4	ENSG00000132872.11	0	0
protein_coding	SLC14A2	ENSG00000132874.14	0	0
protein_coding	LOXHD1	ENSG00000167210.17	0	0
protein_coding	ST8SIA5	ENSG00000101638.13	0	0

protein_coding	ELOA3D	ENSG00000274744.1	0	0
protein_coding	ELOA3C	ENSG00000275553.3	0	0
protein_coding	ELOA3B	ENSG00000278674.1	0	0
protein_coding	ELOA3	ENSG00000183791.4	0	0
protein_coding	ELOA2	ENSG00000206181.5	0	0
protein_coding	AC012254.2	ENSG00000267228.7	0	0
protein_coding	SKOR2	ENSG00000215474.7	0	0
protein_coding	RPL17-	ENSG00000215472.10	0	0
protein_coding	AC090227.1	ENSG00000266997.2	0	0
protein_coding	MRO	ENSG00000134042.12	0	0
protein_coding	AC091551.1	ENSG00000267699.2	0	0
protein_coding	STARD6	ENSG00000174448.8	0	0
protein_coding	DYNAP	ENSG00000178690.3	0	0
protein_coding	BOD1L2	ENSG00000228075.4	0	0
protein_coding	ST8SIA3	ENSG00000177511.5	0	0
protein_coding	GRP	ENSG00000134443.9	0	0
protein_coding	RAX	ENSG00000134438.9	0	0
protein_coding	CPLX4	ENSG00000166569.8	0	0
protein_coding	CDH20	ENSG00000101542.9	0	0
protein_coding	SERPINB11	ENSG00000206072.12	0	0
protein_coding	CDH19	ENSG00000071991.8	0	0
protein_coding	C18orf63	ENSG00000206043.6	0	0
protein_coding	FAM69C	ENSG00000187773.8	0	0
protein_coding	CNDP1	ENSG00000150656.14	0	0
protein_coding	SMIM21	ENSG00000206026.7	0	0
protein_coding	SALL3	ENSG00000256463.8	0	0
protein_coding	KCNG2	ENSG00000178342.4	0	0
protein_coding	OR4F17	ENSG00000176695.8	0	0
protein_coding	THEG	ENSG00000105549.10	0	0
protein_coding	GZMM	ENSG00000197540.7	0	0
protein_coding	PRSS57	ENSG00000185198.11	0	0
protein_coding	PLPPR3	ENSG00000129951.18	0	0
protein_coding	AZU1	ENSG00000172232.9	0	0
protein_coding	PRTN3	ENSG00000196415.9	0	0
protein_coding	EFNA2	ENSG00000099617.3	0	0
protein_coding	PLK5	ENSG00000185988.13	0	0
protein_coding	AC005943.1	ENSG00000267059.2	0	0
protein_coding	ADAT3	ENSG00000213638.5	0	0
protein_coding	AC005258.1	ENSG00000273734.1	0	0
protein_coding	PEAK3	ENSG00000188305.5	0	0
protein_coding	LINGO3	ENSG00000220008.3	0	0
protein_coding	AC006538.2	ENSG00000267001.1	0	0
protein_coding	AC005551.1	ENSG00000284638.1	0	0
protein_coding	C19orf71	ENSG00000183397.5	0	0

protein_coding	RAX2	ENSG00000173976.15	0	0
protein_coding	MATK	ENSG00000007264.14	0	0
protein_coding	ZFR2	ENSG00000105278.10	0	0
protein_coding	ATCAY	ENSG00000167654.17	0	0
protein_coding	NMRK2	ENSG00000077009.13	0	0
protein_coding	CREB3L3	ENSG00000060566.13	0	0
protein_coding	ANKRD24	ENSG00000089847.12	0	0
protein_coding	SHD	ENSG00000105251.10	0	0
protein_coding	TMIGD2	ENSG00000167664.8	0	0
protein_coding	PLIN5	ENSG00000214456.8	0	0
protein_coding	AC011498.4	ENSG00000267385.1	0	0
protein_coding	ARRDC5	ENSG00000205784.2	0	0
protein_coding	ZNRF4	ENSG00000105428.5	0	0
protein_coding	PRR22	ENSG00000212123.3	0	0
protein_coding	AC011499.1	ENSG00000267157.1	0	0
protein_coding	FUT5	ENSG00000130383.7	0	0
protein_coding	AC024592.3	ENSG00000267740.5	0	0
protein_coding	AC104532.1	ENSG00000267314.1	0	0
protein_coding	ACSBG2	ENSG00000130377.13	0	0
protein_coding	ACER1	ENSG00000167769.4	0	0
protein_coding	DENND1C	ENSG00000205744.9	0	0
protein_coding	TUBB4A	ENSG00000104833.11	0	0
protein_coding	MBD3L2B	ENSG00000196589.6	0	0
protein_coding	MBD3L5	ENSG00000237247.6	0	0
protein_coding	MBD3L4	ENSG00000205718.9	0	0
protein_coding	MBD3L2	ENSG00000230522.5	0	0
protein_coding	MBD3L3	ENSG00000182315.9	0	0
protein_coding	AC119396.1	ENSG00000263264.1	0	0
protein_coding	AC008878.3	ENSG00000268861.6	0	0
protein_coding	AC008878.1	ENSG00000267952.1	0	0
protein_coding	AC008878.2	ENSG00000268614.1	0	0
protein_coding	AC008763.2	ENSG00000268400.5	0	0
protein_coding	PCP2	ENSG00000174788.9	0	0
protein_coding	RETN	ENSG00000104918.7	0	0
protein_coding	AC008763.3	ENSG00000269711.1	0	0
protein_coding	FCER2	ENSG00000104921.14	0	0
protein_coding	CLEC4G	ENSG00000182566.13	0	0
protein_coding	CD209	ENSG00000090659.17	0	0
protein_coding	CLEC4M	ENSG00000104938.16	0	0
protein_coding	CCL25	ENSG00000131142.13	0	0
protein_coding	FBN3	ENSG00000142449.12	0	0
protein_coding	AC010323.1	ENSG00000167774.2	0	0
protein_coding	PRAM1	ENSG00000133246.11	0	0
protein_coding	ACTL9	ENSG00000181786.4	0	0

protein_coding	OR2Z1	ENSG00000181733.3	0	0
protein_coding	MBD3L1	ENSG00000170948.3	0	0
protein_coding	OR1M1	ENSG00000170929.6	0	0
protein_coding	OR7G2	ENSG00000170923.3	0	0
protein_coding	OR7G1	ENSG00000161807.3	0	0
protein_coding	OR7G3	ENSG00000170920.2	0	0
protein_coding	OR7D2	ENSG00000188000.4	0	0
protein_coding	OR7D4	ENSG00000174667.4	0	0
protein_coding	OR7E24	ENSG00000237521.3	0	0
protein_coding	ZNF559-ZNF177	ENSG00000270011.7	0	0
protein_coding	ZNF177	ENSG00000188629.12	0	0
protein_coding	PPAN-	ENSG00000243207.6	0	0
protein_coding	AC011511.4	ENSG00000267303.1	0	0
protein_coding	ANGPTL8	ENSG00000130173.13	0	0
protein_coding	AC008481.3	ENSG00000267477.1	0	0
protein_coding	AC008770.2	ENSG00000267179.1	0	0
protein_coding	AC008770.1	ENSG00000257355.1	0	0
protein_coding	ZNF878	ENSG00000257446.3	0	0
protein_coding	ZNF625-	ENSG00000213297.8	0	0
protein_coding	AC008758.5	ENSG00000268870.1	0	0
protein_coding	AC008758.6	ENSG00000269755.1	0	0
protein_coding	AC008758.1	ENSG00000196826.7	0	0
protein_coding	AC010422.6	ENSG00000269693.1	0	0
protein_coding	AC010422.3	ENSG00000269242.1	0	0
protein_coding	AC010422.5	ENSG00000269590.1	0	0
protein_coding	WDR83OS	ENSG00000105583.10	0	0
protein_coding	GNG14	ENSG00000283980.1	0	0
protein_coding	BEST2	ENSG00000039987.6	0	0
protein_coding	THSD8	ENSG00000284491.2	0	0
protein_coding	KLF1	ENSG00000105610.5	0	0
protein_coding	RLN3	ENSG00000171136.6	0	0
protein_coding	MISP3	ENSG00000141854.9	0	0
protein_coding	C19orf67	ENSG00000188032.9	0	0
protein_coding	CLEC17A	ENSG00000187912.11	0	0
protein_coding	ADGRE3	ENSG00000131355.14	0	0
protein_coding	OR7A5	ENSG00000188269.9	0	0
protein_coding	OR7A10	ENSG00000127515.2	0	0
protein_coding	OR7A17	ENSG00000185385.4	0	0
protein_coding	OR7C2	ENSG00000127529.7	0	0
protein_coding	SLC1A6	ENSG00000105143.12	0	0
protein_coding	CCDC105	ENSG00000160994.3	0	0
protein_coding	EPHX3	ENSG00000105131.7	0	0

protein_coding	RASAL3	ENSG00000105122.12	0	0
protein_coding	PGLYRP2	ENSG00000161031.12	0	0
protein_coding	CYP4F22	ENSG00000171954.12	0	0
protein_coding	OR10H2	ENSG00000171942.4	0	0
protein_coding	OR10H3	ENSG00000171936.2	0	0
protein_coding	OR10H1	ENSG00000186723.4	0	0
protein_coding	CYP4F2	ENSG00000186115.12	0	0
protein_coding	OR10H4	ENSG00000176231.2	0	0
protein_coding	HSH2D	ENSG00000196684.12	0	0
protein_coding	CIB3	ENSG00000141977.9	0	0
protein_coding	CALR3	ENSG00000269058.5	0	0
protein_coding	AC008764.1	ENSG00000141979.4	0	0
protein_coding	AC008764.4	ENSG00000268790.5	0	0
protein_coding	AC010646.1	ENSG00000269095.1	0	0
protein_coding	USHBP1	ENSG00000130307.11	0	0
protein_coding	AC010463.1	ENSG00000269307.1	0	0
protein_coding	PLVAP	ENSG00000130300.8	0	0
protein_coding	CCDC194	ENSG00000269720.2	0	0
protein_coding	AC010319.2	ENSG00000269035.1	0	0
protein_coding	NXNL1	ENSG00000171773.2	0	0
protein_coding	UNC13A	ENSG00000130477.15	0	0
protein_coding	INSL3	ENSG00000248099.3	0	0
protein_coding	AC007192.1	ENSG00000268173.3	0	0
protein_coding	PDE4C	ENSG00000105650.22	0	0
protein_coding	AC008397.1	ENSG00000284797.1	0	0
protein_coding	LRRC25	ENSG00000175489.9	0	0
protein_coding	COMP	ENSG00000105664.10	0	0
protein_coding	GDF1	ENSG00000130283.8	0	0
protein_coding	CERS1	ENSG00000223802.7	0	0
protein_coding	AC002985.1	ENSG00000268193.5	0	0
protein_coding	BORCS8-	ENSG00000064489.22	0	0
protein_coding	MEF2B	ENSG00000213999.15	0	0
protein_coding	NCAN	ENSG00000130287.13	0	0
protein_coding	HAPLN4	ENSG00000187664.8	0	0
protein_coding	AC011448.1	ENSG00000258674.5	0	0
protein_coding	AC010615.4	ENSG00000269237.1	0	0
protein_coding	ZNF208	ENSG00000160321.14	0	0
protein_coding	ZNF676	ENSG00000196109.7	0	0
protein_coding	ZNF729	ENSG00000196350.8	0	0
protein_coding	ZNF98	ENSG00000197360.9	0	0
protein_coding	ZNF492	ENSG00000229676.2	0	0
protein_coding	ZNF99	ENSG00000213973.8	0	0
protein_coding	ZNF723	ENSG00000268696.2	0	0
protein_coding	ZNF728	ENSG00000269067.1	0	0

protein_coding	ZNF730	ENSG00000183850.13	0	0
protein_coding	AC092329.3	ENSG00000283201.1	0	0
protein_coding	VSTM2B	ENSG00000187135.7	0	0
protein_coding	ZNF536	ENSG00000198597.8	0	0
protein_coding	SLC7A9	ENSG00000021488.12	0	0
protein_coding	WDR88	ENSG00000166359.10	0	0
protein_coding	SLC7A10	ENSG00000130876.11	0	0
protein_coding	AC092073.1	ENSG00000266953.6	0	0
protein_coding	HPN	ENSG00000105707.13	0	0
protein_coding	AC020907.6	ENSG00000285526.1	0	0
protein_coding	FXYD1	ENSG00000266964.5	0	0
protein_coding	FAM187B	ENSG00000177558.3	0	0
protein_coding	HAMP	ENSG00000105697.8	0	0
protein_coding	MAG	ENSG00000105695.14	0	0
protein_coding	CD22	ENSG00000012124.16	0	0
protein_coding	FFAR1	ENSG00000126266.3	0	0
protein_coding	FFAR3	ENSG00000185897.6	0	0
protein_coding	GPR42	ENSG00000126251.6	0	0
protein_coding	FFAR2	ENSG00000126262.4	0	0
protein_coding	GAPDHS	ENSG00000105679.8	0	0
protein_coding	ATP4A	ENSG00000105675.8	0	0
protein_coding	PMIS2	ENSG00000283758.2	0	0
protein_coding	ZBTB32	ENSG00000011590.13	0	0
protein_coding	AD000671.2	ENSG00000267120.3	0	0
protein_coding	AD000671.1	ENSG00000188223.9	0	0
protein_coding	LIN37	ENSG00000267796.7	0	0
protein_coding	PRODH2	ENSG00000250799.9	0	0
protein_coding	NPHS1	ENSG00000161270.19	0	0
protein_coding	KIRREL2	ENSG00000126259.19	0	0
protein_coding	TYROBP	ENSG00000011600.11	0	0
protein_coding	SYNE4	ENSG00000181392.15	0	0
protein_coding	OVOL3	ENSG00000105261.7	0	0
protein_coding	AC012309.1	ENSG00000267360.6	0	0
protein_coding	AC093227.2	ENSG00000267552.6	0	0
protein_coding	WDR87	ENSG00000171804.10	0	0
protein_coding	AC011479.1	ENSG00000267748.4	0	0
protein_coding	RASGRP4	ENSG00000171777.15	0	0
protein_coding	RYR1	ENSG00000196218.12	0	0
protein_coding	LGALS7	ENSG00000205076.4	0	0
protein_coding	LGALS7B	ENSG00000178934.4	0	0
protein_coding	AC008982.1	ENSG00000268083.5	0	0
protein_coding	AC011455.2	ENSG00000269547.1	0	0
protein_coding	NCCRP1	ENSG00000188505.4	0	0
protein_coding	SYCN	ENSG00000179751.6	0	0

protein_coding	IFNL3	ENSG00000197110.8	0	0
protein_coding	IFNL2	ENSG00000183709.7	0	0
protein_coding	LGALS13	ENSG00000105198.10	0	0
protein_coding	LGALS16	ENSG00000249861.3	0	0
protein_coding	LGALS14	ENSG00000006659.12	0	0
protein_coding	CLC	ENSG00000105205.6	0	0
protein_coding	LEUTX	ENSG00000213921.7	0	0
protein_coding	FCGBP	ENSG00000275395.5	0	0
protein_coding	TTC9B	ENSG00000174521.7	0	0
protein_coding	MIA	ENSG00000261857.6	0	0
protein_coding	MIA-RAB4B	ENSG00000268975.2	0	0
protein_coding	RAB4B-	ENSG00000171570.11	0	0
protein_coding	EGLN2	ENSG00000269858.5	0	0
protein_coding	AC008537.1	ENSG00000268797.1	0	0
protein_coding	CYP2A6	ENSG00000255974.7	0	0
protein_coding	CYP2A7	ENSG00000198077.10	0	0
protein_coding	CYP2A13	ENSG00000197838.4	0	0
protein_coding	CYP2F1	ENSG00000197446.8	0	0
protein_coding	AC011462.1	ENSG00000255730.5	0	0
protein_coding	ERICH4	ENSG00000204978.2	0	0
protein_coding	CEACAM21	ENSG00000007129.17	0	0
protein_coding	CEACAM4	ENSG00000105352.10	0	0
protein_coding	CEACAM7	ENSG00000007306.14	0	0
protein_coding	CEACAM5	ENSG00000105388.15	0	0
protein_coding	AC243967.1	ENSG00000267881.1	0	0
protein_coding	CEACAM3	ENSG00000170956.16	0	0
protein_coding	LYPD4	ENSG00000273111.5	0	0
protein_coding	DMRTC2	ENSG00000142025.15	0	0
protein_coding	AC010616.1	ENSG00000268041.2	0	0
protein_coding	AC010616.2	ENSG00000285505.1	0	0
protein_coding	GRIK5	ENSG00000105737.9	0	0
protein_coding	AC006486.1	ENSG00000268643.1	0	0
protein_coding	CEACAM8	ENSG00000124469.11	0	0
protein_coding	PSG8	ENSG00000124467.18	0	0
protein_coding	PSG6	ENSG00000170848.15	0	0
protein_coding	PSG7	ENSG00000221878.12	0	0
protein_coding	TEX101	ENSG00000131126.18	0	0
protein_coding	L34079.1	ENSG00000268361.1	0	0
protein_coding	IRGC	ENSG00000124449.6	0	0
protein_coding	AC067968.1	ENSG00000267022.1	0	0
protein_coding	AC245748.1	ENSG00000267173.1	0	0
protein_coding	CEACAM20	ENSG00000273777.4	0	0
protein_coding	CEACAM16	ENSG00000213892.11	0	0
protein_coding	APOC4	ENSG00000267467.3	0	0

protein_coding	APOC4-	ENSG00000224916.9	0	0
protein_coding	APOC2	ENSG00000234906.9	0	0
protein_coding	AC005779.2	ENSG00000267545.1	0	0
protein_coding	EXOC3L2	ENSG00000283632.2	0	0
protein_coding	CKM	ENSG00000104879.4	0	0
protein_coding	AC011530.1	ENSG00000268434.5	0	0
protein_coding	RSPH6A	ENSG00000104941.7	0	0
protein_coding	FOXA3	ENSG00000170608.2	0	0
protein_coding	NANOS2	ENSG00000188425.3	0	0
protein_coding	PGLYRP1	ENSG00000008438.4	0	0
protein_coding	IGFL4	ENSG00000204869.8	0	0
protein_coding	IGFL3	ENSG00000188624.2	0	0
protein_coding	IGFL2	ENSG00000204866.8	0	0
protein_coding	HIF3A	ENSG00000124440.15	0	0
protein_coding	PNMA8C	ENSG00000277531.2	0	0
protein_coding	SLC8A2	ENSG00000118160.13	0	0
protein_coding	ZNF541	ENSG00000118156.12	0	0
protein_coding	TPRX1	ENSG00000178928.8	0	0
protein_coding	CRX	ENSG00000105392.15	0	0
protein_coding	BSPH1	ENSG00000188334.3	0	0
protein_coding	ELSPBP1	ENSG00000169393.8	0	0
protein_coding	CABP5	ENSG00000105507.2	0	0
protein_coding	CCDC114	ENSG00000105479.15	0	0
protein_coding	SYNGR4	ENSG00000105467.8	0	0
protein_coding	AC008403.1	ENSG00000268465.1	0	0
protein_coding	SPACA4	ENSG00000177202.2	0	0
protein_coding	FGF21	ENSG00000105550.9	0	0
protein_coding	TULP2	ENSG00000104804.7	0	0
protein_coding	LHB	ENSG00000104826.13	0	0
protein_coding	AC008687.4	ENSG00000268655.2	0	0
protein_coding	CGB3	ENSG00000104827.12	0	0
protein_coding	AC008687.1	ENSG00000267335.2	0	0
protein_coding	CGB2	ENSG00000104818.14	0	0
protein_coding	CGB1	ENSG00000267631.4	0	0
protein_coding	CGB5	ENSG00000189052.6	0	0
protein_coding	CGB8	ENSG00000213030.5	0	0
protein_coding	CGB7	ENSG00000196337.11	0	0
protein_coding	AC008687.8	ENSG00000283663.1	0	0
protein_coding	KCNA7	ENSG00000104848.1	0	0
protein_coding	HRC	ENSG00000130528.11	0	0
protein_coding	DKKL1	ENSG00000104901.6	0	0
protein_coding	CCDC155	ENSG00000161609.9	0	0
protein_coding	PTH2	ENSG00000142538.1	0	0
protein_coding	GFY	ENSG00000261949.5	0	0

protein_coding	AC010619.1	ENSG00000269469.1	0	0
protein_coding	TSKS	ENSG00000126467.10	0	0
protein_coding	IL4I1	ENSG00000104951.15	0	0
protein_coding	AC011452.1	ENSG00000269179.1	0	0
protein_coding	SIGLEC11	ENSG00000161640.15	0	0
protein_coding	IZUMO2	ENSG00000161652.12	0	0
protein_coding	AC020909.1	ENSG00000142539.9	0	0
protein_coding	SPIB	ENSG00000269404.6	0	0
protein_coding	MYBPC2	ENSG00000086967.9	0	0
protein_coding	ASPDH	ENSG00000204653.9	0	0
protein_coding	LRRC4B	ENSG00000131409.12	0	0
protein_coding	C19orf81	ENSG00000235034.6	0	0
protein_coding	SHANK1	ENSG00000161681.15	0	0
protein_coding	GPR32	ENSG00000142511.4	0	0
protein_coding	AC010325.1	ENSG00000261341.6	0	0
protein_coding	ACP4	ENSG00000142513.5	0	0
protein_coding	KLK15	ENSG00000174562.13	0	0
protein_coding	KLK3	ENSG00000142515.14	0	0
protein_coding	KLK2	ENSG00000167751.12	0	0
protein_coding	KLK4	ENSG00000167749.11	0	0
protein_coding	KLK8	ENSG00000129455.15	0	0
protein_coding	KLK9	ENSG00000213022.5	0	0
protein_coding	KLK12	ENSG00000186474.15	0	0
protein_coding	KLK13	ENSG00000167759.12	0	0
protein_coding	KLK14	ENSG00000129437.10	0	0
protein_coding	SIGLEC9	ENSG00000129450.8	0	0
protein_coding	SIGLEC7	ENSG00000168995.13	0	0
protein_coding	SIGLECL1	ENSG00000179213.13	0	0
protein_coding	IGLON5	ENSG00000142549.9	0	0
protein_coding	AC008750.8	ENSG00000269403.1	0	0
protein_coding	NKG7	ENSG00000105374.9	0	0
protein_coding	LIM2	ENSG00000105370.7	0	0
protein_coding	C19orf84	ENSG00000262874.1	0	0
protein_coding	SIGLEC10	ENSG00000142512.14	0	0
protein_coding	SIGLEC8	ENSG00000105366.15	0	0
protein_coding	CEACAM18	ENSG00000213822.6	0	0
protein_coding	SIGLEC12	ENSG00000254521.6	0	0
protein_coding	SIGLEC6	ENSG00000105492.15	0	0
protein_coding	SIGLEC5	ENSG00000105501.12	0	0
protein_coding	AC018755.2	ENSG00000268500.5	0	0
protein_coding	SIGLEC14	ENSG00000254415.3	0	0
protein_coding	FPR2	ENSG00000171049.8	0	0
protein_coding	FPR3	ENSG00000187474.4	0	0
protein_coding	AC010487.3	ENSG00000283088.1	0	0

protein_coding	ZNF816-ZNF321P	ENSG00000221874.4	0	0
protein_coding	ERVV-1	ENSG00000269526.1	0	0
protein_coding	ERVV-2	ENSG00000268964.1	0	0
protein_coding	VN1R2	ENSG00000196131.6	0	0
protein_coding	VN1R4	ENSG00000228567.3	0	0
protein_coding	BIRC8	ENSG00000163098.4	0	0
protein_coding	DPRX	ENSG00000204595.1	0	0
protein_coding	PRKCG	ENSG00000126583.10	0	0
protein_coding	VSTM1	ENSG00000189068.10	0	0
protein_coding	TARM1	ENSG00000248385.7	0	0
protein_coding	LILRB3	ENSG00000204577.11	0	0
protein_coding	LILRA6	ENSG00000244482.10	0	0
protein_coding	LILRB5	ENSG00000105609.16	0	0
protein_coding	LILRB2	ENSG00000131042.14	0	0
protein_coding	LILRA5	ENSG00000187116.13	0	0
protein_coding	LILRA4	ENSG00000239961.2	0	0
protein_coding	LAIR1	ENSG00000167613.15	0	0
protein_coding	LAIR2	ENSG00000167618.9	0	0
protein_coding	LILRA1	ENSG00000104974.11	0	0
protein_coding	LILRB1	ENSG00000104972.15	0	0
protein_coding	LILRB4	ENSG00000186818.12	0	0
protein_coding	KIR3DL3	ENSG00000242019.1	0	0
protein_coding	KIR2DL3	ENSG00000243772.7	0	0
protein_coding	KIR2DL1	ENSG00000125498.19	0	0
protein_coding	KIR2DL4	ENSG00000189013.14	0	0
protein_coding	KIR3DL1	ENSG00000167633.18	0	0
protein_coding	KIR3DL2	ENSG00000240403.5	0	0
protein_coding	FCAR	ENSG00000186431.18	0	0
protein_coding	NCR1	ENSG00000189430.12	0	0
protein_coding	NLRP7	ENSG00000167634.12	0	0
protein_coding	NLRP2	ENSG00000022556.15	0	0
protein_coding	GP6	ENSG00000088053.11	0	0
protein_coding	AC010327.1	ENSG00000267110.1	0	0
protein_coding	TMEM150B	ENSG00000180061.9	0	0
protein_coding	COX6B2	ENSG00000160471.12	0	0
protein_coding	AC020922.1	ENSG00000267706.3	0	0
protein_coding	FAM71E2	ENSG00000180043.11	0	0
protein_coding	TMEM190	ENSG00000160472.4	0	0
protein_coding	SHISA7	ENSG00000187902.11	0	0
protein_coding	C19orf85	ENSG00000283567.1	0	0
protein_coding	SBK2	ENSG00000187550.8	0	0
protein_coding	SBK3	ENSG00000231274.5	0	0

protein_coding	RFPL4A	ENSG00000223638.3	0	0
protein_coding	RFPL4AL1	ENSG00000229292.1	0	0
protein_coding	NLRP11	ENSG00000179873.14	0	0
protein_coding	NLRP4	ENSG00000160505.15	0	0
protein_coding	NLRP13	ENSG00000173572.11	0	0
protein_coding	NLRP8	ENSG00000179709.8	0	0
protein_coding	NLRP5	ENSG00000171487.14	0	0
protein_coding	GALP	ENSG00000197487.8	0	0
protein_coding	ZSCAN5C	ENSG00000204532.6	0	0
protein_coding	SMIM17	ENSG00000268182.5	0	0
protein_coding	ZNF835	ENSG00000127903.13	0	0
protein_coding	ZIM2	ENSG00000269699.6	0	0
protein_coding	PEG3	ENSG00000198300.13	0	0
protein_coding	USP29	ENSG00000131864.10	0	0
protein_coding	ZIM3	ENSG00000141946.1	0	0
protein_coding	DUXA	ENSG00000258873.2	0	0
protein_coding	AC003002.1	ENSG00000268133.1	0	0
protein_coding	AC003002.3	ENSG00000269533.5	0	0
protein_coding	AC003002.2	ENSG00000268533.1	0	0
protein_coding	AC004076.1	ENSG00000268163.1	0	0
protein_coding	AC003005.1	ENSG00000268107.5	0	0
protein_coding	AC003006.1	ENSG00000269026.2	0	0
protein_coding	AC010522.1	ENSG00000268750.6	0	0
protein_coding	AC010326.2	ENSG00000269476.1	0	0
protein_coding	AC020915.5	ENSG00000283515.1	0	0
protein_coding	RNF225	ENSG00000269855.2	0	0
protein_coding	DEFB125	ENSG00000178591.6	0	0
protein_coding	DEFB126	ENSG00000125788.5	0	0
protein_coding	DEFB127	ENSG00000088782.4	0	0
protein_coding	DEFB128	ENSG00000185982.6	0	0
protein_coding	DEFB129	ENSG00000125903.4	0	0
protein_coding	DEFB132	ENSG00000186458.4	0	0
protein_coding	TCF15	ENSG00000125878.6	0	0
protein_coding	SRXN1	ENSG00000271303.1	0	0
protein_coding	AL121758.1	ENSG00000270299.1	0	0
protein_coding	SCRT2	ENSG00000215397.3	0	0
protein_coding	ANGPT4	ENSG00000101280.7	0	0
protein_coding	RAD21L1	ENSG00000244588.5	0	0
protein_coding	AL136531.2	ENSG00000274322.1	0	0
protein_coding	SIRPD	ENSG00000125900.12	0	0
protein_coding	AL049634.2	ENSG00000260861.6	0	0
protein_coding	SIRPG	ENSG00000089012.14	0	0
protein_coding	PDYN	ENSG00000101327.8	0	0
protein_coding	TGM3	ENSG00000125780.11	0	0

protein_coding	TGM6	ENSG00000166948.9	0	0
protein_coding	AL049650.1	ENSG00000256566.1	0	0
protein_coding	TMC2	ENSG00000149488.13	0	0
protein_coding	CPXM1	ENSG00000088882.7	0	0
protein_coding	AL035460.1	ENSG00000241690.3	0	0
protein_coding	C20orf141	ENSG00000258713.2	0	0
protein_coding	TMEM239	ENSG00000198326.9	0	0
protein_coding	GNRH2	ENSG00000125787.11	0	0
protein_coding	AVP	ENSG00000101200.5	0	0
protein_coding	GFRA4	ENSG00000125861.14	0	0
protein_coding	SIGLEC1	ENSG00000088827.12	0	0
protein_coding	PROKR2	ENSG00000101292.7	0	0
protein_coding	LRRN4	ENSG00000125872.7	0	0
protein_coding	HAO1	ENSG00000101323.4	0	0
protein_coding	AL034430.1	ENSG00000285508.1	0	0
protein_coding	AL034430.2	ENSG00000285723.1	0	0
protein_coding	SEL1L2	ENSG00000101251.12	0	0
protein_coding	OTOR	ENSG00000125879.4	0	0
protein_coding	BANF2	ENSG00000125888.14	0	0
protein_coding	AL121900.2	ENSG00000284776.1	0	0
protein_coding	SCP2D1	ENSG00000132631.5	0	0
protein_coding	CFAP61	ENSG00000089101.17	0	0
protein_coding	INSM1	ENSG00000173404.4	0	0
protein_coding	NKX2-4	ENSG00000125816.4	0	0
protein_coding	NKX2-2	ENSG00000125820.5	0	0
protein_coding	AL121722.1	ENSG00000283932.1	0	0
protein_coding	SSTR4	ENSG00000132671.5	0	0
protein_coding	CD93	ENSG00000125810.9	0	0
protein_coding	CST11	ENSG00000125831.9	0	0
protein_coding	CST9L	ENSG00000101435.4	0	0
protein_coding	CST4	ENSG00000101441.4	0	0
protein_coding	CST5	ENSG00000170367.4	0	0
protein_coding	GGTLC1	ENSG00000149435.12	0	0
protein_coding	CST7	ENSG00000077984.5	0	0
protein_coding	VSX1	ENSG00000100987.14	0	0
protein_coding	DEFB115	ENSG00000215547.1	0	0
protein_coding	DEFB116	ENSG00000215545.1	0	0
protein_coding	DEFB119	ENSG00000180483.6	0	0
protein_coding	DEFB121	ENSG00000204548.3	0	0
protein_coding	DEFB123	ENSG00000180424.6	0	0
protein_coding	DEFB124	ENSG00000180383.3	0	0
protein_coding	MCTS2P	ENSG00000101898.6	0	0
protein_coding	COX4I2	ENSG00000131055.4	0	0
protein_coding	MYLK2	ENSG00000101306.10	0	0

protein_coding	DUSP15	ENSG00000149599.15	0	0
protein_coding	TTLL9	ENSG00000131044.17	0	0
protein_coding	XKR7	ENSG00000260903.2	0	0
protein_coding	CCM2L	ENSG00000101331.15	0	0
protein_coding	HCK	ENSG00000101336.13	0	0
protein_coding	C20orf203	ENSG00000198547.8	0	0
protein_coding	FO393400.1	ENSG00000285382.1	0	0
protein_coding	EFCAB8	ENSG00000215529.12	0	0
protein_coding	SUN5	ENSG00000167098.11	0	0
protein_coding	BPIFB2	ENSG00000078898.6	0	0
protein_coding	BPIFB6	ENSG00000167104.11	0	0
protein_coding	BPIFB3	ENSG00000186190.7	0	0
protein_coding	BPIFB4	ENSG00000186191.7	0	0
protein_coding	BPIFA2	ENSG00000131050.10	0	0
protein_coding	BPIFA3	ENSG00000131059.11	0	0
protein_coding	BPIFA1	ENSG00000198183.11	0	0
protein_coding	BPIFB1	ENSG00000125999.10	0	0
protein_coding	ASIP	ENSG00000101440.9	0	0
protein_coding	MYH7B	ENSG00000078814.15	0	0
protein_coding	AL121753.1	ENSG00000261582.1	0	0
protein_coding	C20orf173	ENSG00000125975.13	0	0
protein_coding	AL109827.1	ENSG00000272897.5	0	0
protein_coding	TGIF2-	ENSG00000259399.1	0	0
protein_coding	SLA2	ENSG00000101082.13	0	0
protein_coding	GHRH	ENSG00000118702.9	0	0
protein_coding	NNAT	ENSG00000053438.9	0	0
protein_coding	BPI	ENSG00000101425.13	0	0
protein_coding	ADIG	ENSG00000182035.11	0	0
protein_coding	SLC32A1	ENSG00000101438.3	0	0
protein_coding	PPP1R16B	ENSG00000101445.9	0	0
protein_coding	EMILIN3	ENSG00000183798.4	0	0
protein_coding	PTPRT	ENSG00000196090.12	0	0
protein_coding	Z98752.3	ENSG00000277611.1	0	0
protein_coding	GTSF1L	ENSG00000124196.5	0	0
protein_coding	GDAP1L1	ENSG00000124194.16	0	0
protein_coding	R3HDML	ENSG00000101074.3	0	0
protein_coding	WFDC12	ENSG00000168703.5	0	0
protein_coding	SEMG1	ENSG00000124233.11	0	0
protein_coding	SEMG2	ENSG00000124157.6	0	0
protein_coding	RBPJL	ENSG00000124232.10	0	0
protein_coding	SYS1-	ENSG00000254806.5	0	0
protein_coding	TP53TG5	ENSG00000124251.10	0	0
protein_coding	DBNDD2	ENSG00000244274.7	0	0
protein_coding	SPINT3	ENSG00000101446.7	0	0

protein_coding	WFDC6	ENSG00000243543.8	0	0
protein_coding	EPPIN-	ENSG00000249139.1	0	0
protein_coding	EPPIN	ENSG00000101448.13	0	0
protein_coding	WFDC8	ENSG00000158901.11	0	0
protein_coding	WFDC9	ENSG00000180205.3	0	0
protein_coding	WFDC10A	ENSG00000180305.4	0	0
protein_coding	WFDC11	ENSG00000180083.10	0	0
protein_coding	WFDC13	ENSG00000168634.4	0	0
protein_coding	SPINT4	ENSG00000149651.3	0	0
protein_coding	SPATA25	ENSG00000149634.4	0	0
protein_coding	CDH22	ENSG00000149654.9	0	0
protein_coding	OCSTAMP	ENSG00000149635.2	0	0
protein_coding	TMEM189-<br>UBE2V1	ENSG00000124208.16	0	0
protein_coding	CBLN4	ENSG00000054803.3	0	0
protein_coding	MC3R	ENSG00000124089.4	0	0
protein_coding	GCNT7	ENSG00000124091.9	0	0
protein_coding	FAM209B	ENSG00000213714.1	0	0
protein_coding	SPO11	ENSG00000054796.12	0	0
protein_coding	PCK1	ENSG00000124253.10	0	0
protein_coding	ZBP1	ENSG00000124256.14	0	0
protein_coding	C20orf85	ENSG00000124237.5	0	0
protein_coding	ANKRD60	ENSG00000124227.5	0	0
protein_coding	STX16-	ENSG00000254995.4	0	0
protein_coding	TUBB1	ENSG00000101162.3	0	0
protein_coding	ZNF831	ENSG00000124203.6	0	0
protein_coding	EDN3	ENSG00000124205.16	0	0
protein_coding	HRH3	ENSG00000101180.15	0	0
protein_coding	GATA5	ENSG00000130700.6	0	0
protein_coding	BHLHE23	ENSG00000125533.5	0	0
protein_coding	BIRC7	ENSG00000101197.12	0	0
protein_coding	CHRNA4	ENSG00000101204.16	0	0
protein_coding	SRMS	ENSG00000125508.3	0	0
protein_coding	RTEL1	ENSG00000258366.8	0	0
protein_coding	RTEL1-<br>TNFRSF6B	ENSG00000026036.22	0	0
protein_coding	TNFRSF6B	ENSG00000243509.5	0	0
protein_coding	AL121845.3	ENSG00000273154.3	0	0
protein_coding	LIME1	ENSG00000203896.9	0	0
protein_coding	AL121845.2	ENSG00000273047.1	0	0
protein_coding	ABHD16B	ENSG00000183260.7	0	0
protein_coding	C20orf204	ENSG00000196421.8	0	0
protein_coding	LKAAEAR1	ENSG00000171695.10	0	0

protein_coding	MYT1	ENSG00000196132.12	0	0
protein_coding	NPBWR2	ENSG00000125522.3	0	0
protein_coding	FP565260.4	ENSG00000279493.1	0	0
protein_coding	FP565260.2	ENSG00000276612.3	0	0
protein_coding	H2BFS	ENSG00000274559.3	0	0
protein_coding	CBSL	ENSG00000274276.4	0	0
protein_coding	CRYAA2	ENSG00000276076.4	0	0
protein_coding	SMIM11B	ENSG00000273590.4	0	0
protein_coding	FAM243B	ENSG00000277277.3	0	0
protein_coding	SMIM34B	ENSG00000278961.2	0	0
protein_coding	KCNE1B	ENSG00000276289.4	0	0
protein_coding	TPTE	ENSG00000274391.4	0	0
protein_coding	POTED	ENSG00000166351.10	0	0
protein_coding	LIPI	ENSG00000188992.11	0	0
protein_coding	CHODL	ENSG00000154645.13	0	0
protein_coding	KRTAP24-1	ENSG00000188694.5	0	0
protein_coding	KRTAP25-1	ENSG00000232263.1	0	0
protein_coding	KRTAP26-1	ENSG00000197683.4	0	0
protein_coding	KRTAP27-1	ENSG00000206107.2	0	0
protein_coding	KRTAP23-1	ENSG00000186980.6	0	0
protein_coding	KRTAP13-2	ENSG00000182816.8	0	0
protein_coding	KRTAP13-1	ENSG00000198390.4	0	0
protein_coding	KRTAP13-3	ENSG00000240432.3	0	0
protein_coding	KRTAP13-4	ENSG00000186971.3	0	0
protein_coding	KRTAP15-1	ENSG00000186970.4	0	0
protein_coding	KRTAP19-2	ENSG00000186965.4	0	0
protein_coding	KRTAP19-3	ENSG00000244025.4	0	0
protein_coding	KRTAP19-4	ENSG00000186967.6	0	0
protein_coding	KRTAP19-5	ENSG00000186977.2	0	0
protein_coding	KRTAP19-6	ENSG00000186925.6	0	0
protein_coding	KRTAP19-7	ENSG00000244362.3	0	0
protein_coding	KRTAP22-2	ENSG00000206106.2	0	0
protein_coding	KRTAP6-3	ENSG00000212938.3	0	0
protein_coding	KRTAP6-2	ENSG00000186930.4	0	0
protein_coding	KRTAP22-1	ENSG00000186924.3	0	0
protein_coding	KRTAP6-1	ENSG00000184724.5	0	0
protein_coding	KRTAP20-1	ENSG00000244624.3	0	0
protein_coding	KRTAP20-4	ENSG00000206105.2	0	0
protein_coding	KRTAP20-2	ENSG00000184032.2	0	0
protein_coding	KRTAP20-3	ENSG00000206104.2	0	0
protein_coding	KRTAP21-3	ENSG00000231068.1	0	0
protein_coding	KRTAP21-2	ENSG00000187026.2	0	0
protein_coding	KRTAP21-1	ENSG00000187005.4	0	0
protein_coding	KRTAP8-1	ENSG00000183640.5	0	0

protein_coding	KRTAP7-1	ENSG00000274749.1	0	0
protein_coding	KRTAP11-1	ENSG00000182591.5	0	0
protein_coding	KRTAP19-8	ENSG00000206102.2	0	0
protein_coding	AP000275.2	ENSG00000265590.9	0	0
protein_coding	C21orf62	ENSG00000205929.10	0	0
protein_coding	OLIG2	ENSG00000205927.4	0	0
protein_coding	OLIG1	ENSG00000184221.12	0	0
protein_coding	AP000295.1	ENSG00000249624.9	0	0
protein_coding	AP000311.1	ENSG00000249209.2	0	0
protein_coding	KCNE2	ENSG00000159197.3	0	0
protein_coding	SMIM11A	ENSG00000205670.10	0	0
protein_coding	FAM243A	ENSG00000222018.1	0	0
protein_coding	SMIM34A	ENSG00000243627.4	0	0
protein_coding	CLDN14	ENSG00000159261.10	0	0
protein_coding	WRB-	ENSG00000285815.1	0	0
protein_coding	SH3BGR	ENSG00000185437.13	0	0
protein_coding	B3GALT5	ENSG00000183778.17	0	0
protein_coding	IGSF5	ENSG00000183067.5	0	0
protein_coding	PCP4	ENSG00000183036.10	0	0
protein_coding	FAM3B	ENSG00000183844.16	0	0
protein_coding	TMPRSS2	ENSG00000184012.11	0	0
protein_coding	UMODL1	ENSG00000177398.18	0	0
protein_coding	TFF3	ENSG00000160180.15	0	0
protein_coding	TFF2	ENSG00000160181.8	0	0
protein_coding	UBASH3A	ENSG00000160185.14	0	0
protein_coding	CBS	ENSG00000160200.17	0	0
protein_coding	CRYAA	ENSG00000160202.7	0	0
protein_coding	SIK1	ENSG00000142178.8	0	0
protein_coding	H2BFS	ENSG00000234289.5	0	0
protein_coding	DNMT3L	ENSG00000142182.8	0	0
protein_coding	TSPEAR	ENSG00000175894.16	0	0
protein_coding	KRTAP10-1	ENSG00000215455.4	0	0
protein_coding	KRTAP10-2	ENSG00000205445.3	0	0
protein_coding	KRTAP10-3	ENSG00000212935.1	0	0
protein_coding	KRTAP10-4	ENSG00000215454.6	0	0
protein_coding	KRTAP10-5	ENSG00000241123.1	0	0
protein_coding	KRTAP10-6	ENSG00000188155.11	0	0
protein_coding	KRTAP10-7	ENSG00000272804.3	0	0
protein_coding	KRTAP10-8	ENSG00000187766.1	0	0
protein_coding	KRTAP10-9	ENSG00000221837.5	0	0
protein_coding	KRTAP10-10	ENSG00000221859.2	0	0
protein_coding	KRTAP10-11	ENSG00000243489.4	0	0
protein_coding	KRTAP12-4	ENSG00000212933.1	0	0
protein_coding	KRTAP12-3	ENSG00000205439.10	0	0

protein_coding	KRTAP12-2	ENSG00000221864.4	0	0
protein_coding	KRTAP12-1	ENSG00000187175.5	0	0
protein_coding	KRTAP10-12	ENSG00000189169.7	0	0
protein_coding	S100B	ENSG00000160307.9	0	0
protein_coding	OR11H1	ENSG00000130538.5	0	0
protein_coding	POTEH	ENSG00000198062.14	0	0
protein_coding	CCT8L2	ENSG00000198445.4	0	0
protein_coding	XKR3	ENSG00000172967.7	0	0
protein_coding	GAB4	ENSG00000215568.8	0	0
protein_coding	SLC25A18	ENSG00000182902.13	0	0
protein_coding	TUBA8	ENSG00000183785.14	0	0
protein_coding	GGTLC3	ENSG00000274252.1	0	0
protein_coding	TMEM191B	ENSG00000278558.4	0	0
protein_coding	RIMBP3	ENSG00000275793.1	0	0
protein_coding	DGCR6	ENSG00000183628.12	0	0
protein_coding	AC007326.4	ENSG00000283809.1	0	0
protein_coding	PRODH	ENSG00000100033.16	0	0
protein_coding	TSSK2	ENSG00000206203.4	0	0
protein_coding	GSC2	ENSG00000063515.2	0	0
protein_coding	CLDN5	ENSG00000184113.9	0	0
protein_coding	SEPT5-	ENSG00000284874.1	0	0
protein_coding	GP1BB	ENSG00000203618.5	0	0
protein_coding	USP41	ENSG00000161133.16	0	0
protein_coding	AC007731.5	ENSG00000277971.1	0	0
protein_coding	SLC7A4	ENSG00000099960.12	0	0
protein_coding	GGT2	ENSG00000133475.17	0	0
protein_coding	RIMBP3B	ENSG00000274600.1	0	0
protein_coding	VPREB1	ENSG00000169575.4	0	0
protein_coding	ZNF280A	ENSG00000169548.3	0	0
protein_coding	PRAME	ENSG00000185686.17	0	0
protein_coding	GGTLC2	ENSG00000100121.12	0	0
protein_coding	IGLL5	ENSG00000254709.7	0	0
protein_coding	IGLL1	ENSG00000128322.6	0	0
protein_coding	RGL4	ENSG00000159496.14	0	0
protein_coding	C22orf15	ENSG00000169314.14	0	0
protein_coding	DERL3	ENSG00000099958.14	0	0
protein_coding	AP000350.4	ENSG00000251357.4	0	0
protein_coding	DDTL	ENSG00000099974.7	0	0
protein_coding	DDT	ENSG00000099977.14	0	0
protein_coding	AC253536.7	ENSG00000285762.1	0	0
protein_coding	GSTT4	ENSG00000276950.5	0	0
protein_coding	SUSD2	ENSG00000099994.10	0	0
protein_coding	GGT5	ENSG00000099998.17	0	0

protein_coding	SPECC1L-ADORA2A	ENSG00000258555.6	0	0
protein_coding	ADORA2A	ENSG00000128271.21	0	0
protein_coding	UPB1	ENSG00000100024.14	0	0
protein_coding	PIWIL3	ENSG00000184571.13	0	0
protein_coding	TMEM211	ENSG00000206069.6	0	0
protein_coding	CRYBB3	ENSG00000100053.9	0	0
protein_coding	CRYBB2	ENSG00000244752.2	0	0
protein_coding	MYO18B	ENSG00000133454.15	0	0
protein_coding	SEZ6L	ENSG00000100095.18	0	0
protein_coding	CRYBB1	ENSG00000100122.6	0	0
protein_coding	CRYBA4	ENSG00000196431.3	0	0
protein_coding	RFPL1	ENSG00000128250.5	0	0
protein_coding	CABP7	ENSG00000100314.3	0	0
protein_coding	HORMAD2	ENSG00000176635.17	0	0
protein_coding	OSM	ENSG00000099985.3	0	0
protein_coding	AC004997.1	ENSG00000248751.6	0	0
protein_coding	AC004832.3	ENSG00000249590.7	0	0
protein_coding	MTFP1	ENSG00000242114.5	0	0
protein_coding	SEC14L3	ENSG00000100012.11	0	0
protein_coding	GAL3ST1	ENSG00000128242.12	0	0
protein_coding	INPP5J	ENSG00000185133.13	0	0
protein_coding	PLA2G3	ENSG00000100078.3	0	0
protein_coding	Z82190.2	ENSG00000285404.1	0	0
protein_coding	SLC5A1	ENSG00000100170.9	0	0
protein_coding	C22orf42	ENSG00000205856.3	0	0
protein_coding	RFPL2	ENSG00000128253.13	0	0
protein_coding	SLC5A4	ENSG00000100191.5	0	0
protein_coding	RFPL3	ENSG00000128276.10	0	0
protein_coding	BPIFC	ENSG00000184459.8	0	0
protein_coding	SYN3	ENSG00000185666.14	0	0
protein_coding	ISX	ENSG00000175329.12	0	0
protein_coding	APOL5	ENSG00000128313.2	0	0
protein_coding	APOL4	ENSG00000100336.17	0	0
protein_coding	CACNG2	ENSG00000166862.6	0	0
protein_coding	PVALB	ENSG00000100362.12	0	0
protein_coding	CSF2RB	ENSG00000100368.13	0	0
protein_coding	TEX33	ENSG00000185264.11	0	0
protein_coding	TMPRSS6	ENSG00000187045.18	0	0
protein_coding	SSTR3	ENSG00000278195.1	0	0
protein_coding	LGALS2	ENSG00000100079.6	0	0
protein_coding	Z83844.3	ENSG00000285304.1	0	0
protein_coding	PDXP	ENSG00000241360.1	0	0

protein_coding	Z83844.1	ENSG00000100101.15	0	0
protein_coding	GALR3	ENSG00000128310.2	0	0
protein_coding	C22orf23	ENSG00000128346.10	0	0
protein_coding	SOX10	ENSG00000100146.16	0	0
protein_coding	SLC16A8	ENSG00000100156.10	0	0
protein_coding	TPTEP2-CSNK1E	ENSG00000283900.1	0	0
protein_coding	KCNJ4	ENSG00000168135.4	0	0
protein_coding	APOBEC3A	ENSG00000128383.12	0	0
protein_coding	APOBEC3B	ENSG00000179750.15	0	0
protein_coding	AL022318.4	ENSG00000284554.2	0	0
protein_coding	APOBEC3D	ENSG00000243811.9	0	0
protein_coding	AL022312.1	ENSG00000285025.1	0	0
protein_coding	CACNA1I	ENSG00000100346.17	0	0
protein_coding	ENTHD1	ENSG00000176177.9	0	0
protein_coding	GRAP2	ENSG00000100351.16	0	0
protein_coding	AL022238.4	ENSG00000284431.1	0	0
protein_coding	DNAJB7	ENSG00000172404.4	0	0
protein_coding	SHISA8	ENSG00000234965.2	0	0
protein_coding	SULT4A1	ENSG00000130540.13	0	0
protein_coding	PNPLA5	ENSG00000100341.11	0	0
protein_coding	PARVG	ENSG00000138964.16	0	0
protein_coding	UPK3A	ENSG00000100373.9	0	0
protein_coding	FAM19A5	ENSG00000219438.8	0	0
protein_coding	IL17REL	ENSG00000188263.10	0	0
protein_coding	TTLL8	ENSG00000138892.11	0	0
protein_coding	SCO2	ENSG00000130489.14	0	0
protein_coding	SCO2	ENSG00000284194.1	0	0
protein_coding	CHKB-	ENSG00000254413.8	0	0
protein_coding	ACR	ENSG00000100312.10	0	0
protein_coding	SHOX	ENSG00000185960.14	0	0
protein_coding	PAR	ENSG00000185291.11	0	0
protein_coding	PAR	ENSG00000182162.10	0	0
protein_coding	PAR	ENSG00000196433.12	0	0
protein_coding	ARSH	ENSG00000205667.2	0	0
protein_coding	ARSF	ENSG00000062096.14	0	0
protein_coding	VCX3A	ENSG00000169059.12	0	0
protein_coding	VCX	ENSG00000182583.12	0	0
protein_coding	VCX2	ENSG00000177504.10	0	0
protein_coding	VCX3B	ENSG00000205642.9	0	0
protein_coding	FAM9A	ENSG00000183304.10	0	0
protein_coding	CLDN34	ENSG00000234469.4	0	0
protein_coding	AMELX	ENSG00000125363.14	0	0

protein_coding	TLR7	ENSG00000196664.4	0	0
protein_coding	TLR8	ENSG00000101916.11	0	0
protein_coding	ATXN3L	ENSG00000123594.5	0	0
protein_coding	EGFL6	ENSG00000198759.11	0	0
protein_coding	GLRA2	ENSG00000101958.13	0	0
protein_coding	BMX	ENSG00000102010.14	0	0
protein_coding	ACE2	ENSG00000130234.10	0	0
protein_coding	AC097625.1	ENSG00000285602.1	0	0
protein_coding	GRPR	ENSG00000126010.5	0	0
protein_coding	MAGEB17	ENSG00000182798.10	0	0
protein_coding	S100G	ENSG00000169906.5	0	0
protein_coding	BEND2	ENSG00000177324.13	0	0
protein_coding	RS1	ENSG00000102104.8	0	0
protein_coding	PPEF1	ENSG00000086717.18	0	0
protein_coding	CNKSR2	ENSG00000149970.15	0	0
protein_coding	SMPX	ENSG00000091482.6	0	0
protein_coding	CBLL2	ENSG00000175809.5	0	0
protein_coding	DDX53	ENSG00000184735.6	0	0
protein_coding	MAGEB18	ENSG00000176774.5	0	0
protein_coding	MAGEB6B	ENSG00000232030.3	0	0
protein_coding	MAGEB6	ENSG00000176746.6	0	0
protein_coding	MAGEB5	ENSG00000188408.5	0	0
protein_coding	PPP4R3C	ENSG00000224960.4	0	0
protein_coding	DCAF8L1	ENSG00000226372.3	0	0
protein_coding	MAGEB3	ENSG00000198798.5	0	0
protein_coding	MAGEB4	ENSG00000120289.11	0	0
protein_coding	MAGEB1	ENSG00000214107.7	0	0
protein_coding	CXorf21	ENSG00000120280.5	0	0
protein_coding	FTHL17	ENSG00000132446.6	0	0
protein_coding	FAM47A	ENSG00000185448.10	0	0
protein_coding	FAM47B	ENSG00000189132.6	0	0
protein_coding	MAGEB16	ENSG00000189023.10	0	0
protein_coding	CFAP47	ENSG00000165164.13	0	0
protein_coding	FAM47C	ENSG00000198173.4	0	0
protein_coding	AF241726.2	ENSG00000250349.3	0	0
protein_coding	HYPM	ENSG00000187516.6	0	0
protein_coding	AL121578.2	ENSG00000229674.2	0	0
protein_coding	OTC	ENSG00000036473.7	0	0
protein_coding	MPC1L	ENSG00000238205.3	0	0
protein_coding	NYX	ENSG00000188937.5	0	0
protein_coding	GPR34	ENSG00000171659.14	0	0
protein_coding	PPP1R2C	ENSG00000102055.6	0	0
protein_coding	DUSP21	ENSG00000189037.7	0	0
protein_coding	CXorf36	ENSG00000147113.16	0	0

protein_coding	SPACA5	ENSG00000171489.10	0	0
protein_coding	SPACA5B	ENSG00000171478.7	0	0
protein_coding	SSX5	ENSG00000165583.14	0	0
protein_coding	SSX1	ENSG00000126752.7	0	0
protein_coding	SSX3	ENSG00000165584.15	0	0
protein_coding	SSX4	ENSG00000268009.5	0	0
protein_coding	SSX4B	ENSG00000269791.5	0	0
protein_coding	GLOD5	ENSG00000171433.11	0	0
protein_coding	GATA1	ENSG00000102145.13	0	0
protein_coding	ERAS	ENSG00000187682.2	0	0
protein_coding	CACNA1F	ENSG00000102001.12	0	0
protein_coding	GAGE10	ENSG00000215274.5	0	0
protein_coding	GAGE12J	ENSG00000224659.2	0	0
protein_coding	GAGE13	ENSG00000274274.1	0	0
protein_coding	GAGE2E	ENSG00000275113.1	0	0
protein_coding	GAGE12B	ENSG00000236737.1	0	0
protein_coding	GAGE12C	ENSG00000237671.3	0	0
protein_coding	GAGE12D	ENSG00000227488.2	0	0
protein_coding	GAGE12F	ENSG00000236362.8	0	0
protein_coding	GAGE12E	ENSG00000216649.3	0	0
protein_coding	GAGE12G	ENSG00000215269.5	0	0
protein_coding	GAGE12H	ENSG00000224902.5	0	0
protein_coding	GAGE1	ENSG00000205777.17	0	0
protein_coding	GAGE2A	ENSG00000189064.8	0	0
protein_coding	PAGE1	ENSG00000068985.4	0	0
protein_coding	PAGE4	ENSG00000101951.16	0	0
protein_coding	AKAP4	ENSG00000147081.14	0	0
protein_coding	CCNB3	ENSG00000147082.17	0	0
protein_coding	DGKK	ENSG00000274588.1	0	0
protein_coding	BMP15	ENSG00000130385.5	0	0
protein_coding	CXorf67	ENSG00000187690.3	0	0
protein_coding	CENPVL3	ENSG00000224109.3	0	0
protein_coding	CENPVL2	ENSG00000283093.1	0	0
protein_coding	CENPVL1	ENSG00000223591.5	0	0
protein_coding	MAGED4B	ENSG00000187243.16	0	0
protein_coding	XAGE2	ENSG00000155622.6	0	0
protein_coding	XAGE1A	ENSG00000204379.10	0	0
protein_coding	XAGE1B	ENSG00000204382.11	0	0
protein_coding	SSX7	ENSG00000187754.8	0	0
protein_coding	SSX2	ENSG00000241476.8	0	0
protein_coding	SSX2B	ENSG00000268447.5	0	0
protein_coding	SPANXN5	ENSG00000204363.4	0	0
protein_coding	XAGE5	ENSG00000171405.12	0	0
protein_coding	XAGE3	ENSG00000171402.14	0	0

protein_coding	FAM156B	ENSG00000179304.16	0	0
protein_coding	FAM156A	ENSG00000268350.7	0	0
protein_coding	ITIH6	ENSG00000102313.8	0	0
protein_coding	ALAS2	ENSG00000158578.20	0	0
protein_coding	PAGE2B	ENSG00000238269.8	0	0
protein_coding	PAGE2	ENSG00000234068.6	0	0
protein_coding	PAGE5	ENSG00000158639.12	0	0
protein_coding	PAGE3	ENSG00000204279.7	0	0
protein_coding	FOXR2	ENSG00000189299.6	0	0
protein_coding	NLRP2B	ENSG00000215174.2	0	0
protein_coding	ASB12	ENSG00000198881.9	0	0
protein_coding	VSIG4	ENSG00000155659.14	0	0
protein_coding	FAM155B	ENSG00000130054.4	0	0
protein_coding	AWAT2	ENSG00000147160.9	0	0
protein_coding	OTUD6A	ENSG00000189401.2	0	0
protein_coding	DGAT2L6	ENSG00000184210.5	0	0
protein_coding	AWAT1	ENSG00000204195.3	0	0
protein_coding	P2RY4	ENSG00000186912.6	0	0
protein_coding	ARR3	ENSG00000120500.17	0	0
protein_coding	RAB41	ENSG00000147127.8	0	0
protein_coding	TEX11	ENSG00000120498.13	0	0
protein_coding	SLC7A3	ENSG00000165349.11	0	0
protein_coding	CXorf65	ENSG00000204165.5	0	0
protein_coding	AL590764.2	ENSG00000285171.1	0	0
protein_coding	IL2RG	ENSG00000147168.12	0	0
protein_coding	GJB1	ENSG00000169562.10	0	0
protein_coding	CXCR3	ENSG00000186810.7	0	0
protein_coding	CXorf49	ENSG00000215115.6	0	0
protein_coding	CXorf49B	ENSG00000215113.6	0	0
protein_coding	CITED1	ENSG00000125931.10	0	0
protein_coding	AL133500.1	ENSG00000285547.1	0	0
protein_coding	DMRTC1B	ENSG00000184911.14	0	0
protein_coding	FAM236B	ENSG00000268994.3	0	0
protein_coding	FAM236D	ENSG00000225396.5	0	0
protein_coding	DMRTC1	ENSG00000269502.5	0	0
protein_coding	FAM236C	ENSG00000283594.1	0	0
protein_coding	FAM236A	ENSG00000275520.1	0	0
protein_coding	PABPC1L2B	ENSG00000184388.5	0	0
protein_coding	PABPC1L2A	ENSG00000186288.5	0	0
protein_coding	CDX4	ENSG00000131264.3	0	0
protein_coding	ZCCHC13	ENSG00000187969.5	0	0
protein_coding	NEXMIF	ENSG00000050030.14	0	0
protein_coding	ZDHHC15	ENSG00000102383.13	0	0
protein_coding	MAGEE2	ENSG00000186675.6	0	0

protein_coding	FGF16	ENSG00000196468.7	0	0
protein_coding	PGAM4	ENSG00000226784.2	0	0
protein_coding	RTL3	ENSG00000179300.3	0	0
protein_coding	GPR174	ENSG00000147138.2	0	0
protein_coding	POU3F4	ENSG00000196767.7	0	0
protein_coding	DACH2	ENSG00000126733.20	0	0
protein_coding	TGIF2LX	ENSG00000153779.10	0	0
protein_coding	TNMD	ENSG00000000005.5	0	0
protein_coding	NOX1	ENSG00000007952.17	0	0
protein_coding	ARL13A	ENSG00000174225.14	0	0
protein_coding	BTK	ENSG00000010671.15	0	0
protein_coding	RPL36A-HNRNPH2	ENSG00000257529.5	0	0
protein_coding	NXF5	ENSG00000126952.16	0	0
protein_coding	TCEAL6	ENSG00000204071.10	0	0
protein_coding	TCP11X1	ENSG00000268235.7	0	0
protein_coding	NXF2	ENSG00000269405.6	0	0
protein_coding	AC235565.2	ENSG00000284800.1	0	0
protein_coding	NXF2B	ENSG00000269437.7	0	0
protein_coding	TCP11X2	ENSG00000215029.9	0	0
protein_coding	TMSB15A	ENSG00000158164.6	0	0
protein_coding	RAB40AL	ENSG00000102128.8	0	0
protein_coding	TCEAL5	ENSG00000204065.2	0	0
protein_coding	GLRA4	ENSG00000188828.11	0	0
protein_coding	PLP1	ENSG00000123560.13	0	0
protein_coding	TMSB15B	ENSG00000269226.7	0	0
protein_coding	IL1RAPL2	ENSG00000189108.12	0	0
protein_coding	TEX13A	ENSG00000268629.6	0	0
protein_coding	NRK	ENSG00000123572.16	0	0
protein_coding	SERPINA7	ENSG00000123561.14	0	0
protein_coding	RIPPLY1	ENSG00000147223.5	0	0
protein_coding	CLDN2	ENSG00000165376.10	0	0
protein_coding	PIH1D3	ENSG00000080572.12	0	0
protein_coding	TEX13B	ENSG00000170925.3	0	0
protein_coding	AL035425.2	ENSG00000260548.1	0	0
protein_coding	IRS4	ENSG00000133124.11	0	0
protein_coding	DCX	ENSG00000077279.18	0	0
protein_coding	SERTM2	ENSG00000260802.1	0	0
protein_coding	TRPC5	ENSG00000072315.3	0	0
protein_coding	TRPC5OS	ENSG00000204025.7	0	0
protein_coding	RTL4	ENSG00000187823.3	0	0
protein_coding	RBMXL3	ENSG00000175718.9	0	0
protein_coding	LUZP4	ENSG00000102021.10	0	0

protein_coding	SLC6A14	ENSG00000268104.2	0	0
protein_coding	CT83	ENSG00000204019.4	0	0
protein_coding	LONRF3	ENSG00000175556.16	0	0
protein_coding	AL772284.2	ENSG00000277535.2	0	0
protein_coding	KIAA1210	ENSG00000250423.2	0	0
protein_coding	AKAP14	ENSG00000186471.12	0	0
protein_coding	RHOXF2B	ENSG00000203989.4	0	0
protein_coding	RHOXF2	ENSG00000131721.5	0	0
protein_coding	ATP1B4	ENSG00000101892.11	0	0
protein_coding	CT47B1	ENSG00000236446.3	0	0
protein_coding	AC008162.2	ENSG00000278646.1	0	0
protein_coding	CT47A12	ENSG00000226685.4	0	0
protein_coding	CT47A11	ENSG00000226929.3	0	0
protein_coding	CT47A10	ENSG00000224089.3	0	0
protein_coding	CT47A9	ENSG00000226600.3	0	0
protein_coding	CT47A8	ENSG00000230347.5	0	0
protein_coding	CT47A7	ENSG00000228517.2	0	0
protein_coding	CT47A6	ENSG00000226023.6	0	0
protein_coding	CT47A5	ENSG00000237957.6	0	0
protein_coding	CT47A4	ENSG00000230594.3	0	0
protein_coding	CT47A3	ENSG00000236126.3	0	0
protein_coding	CT47A2	ENSG00000242362.2	0	0
protein_coding	CT47A1	ENSG00000236371.5	0	0
protein_coding	SH2D1A	ENSG00000183918.16	0	0
protein_coding	TEX13D	ENSG00000282419.2	0	0
protein_coding	TEX13C	ENSG00000282815.1	0	0
protein_coding	DCAF12L2	ENSG00000198354.6	0	0
protein_coding	DCAF12L1	ENSG00000198889.4	0	0
protein_coding	PRR32	ENSG00000183631.4	0	0
protein_coding	ACTRT1	ENSG00000123165.8	0	0
protein_coding	SASH3	ENSG00000122122.9	0	0
protein_coding	GPR119	ENSG00000147262.3	0	0
protein_coding	ARHGAP36	ENSG00000147256.11	0	0
protein_coding	OR13H1	ENSG00000171054.8	0	0
protein_coding	USP26	ENSG00000134588.12	0	0
protein_coding	TFDP3	ENSG00000183434.9	0	0
protein_coding	ETDB	ENSG00000224107.5	0	0
protein_coding	CT55	ENSG00000169551.12	0	0
protein_coding	ETDA	ENSG00000238210.3	0	0
protein_coding	ETDC	ENSG00000283644.1	0	0
protein_coding	CT45A1	ENSG00000268940.5	0	0
protein_coding	CT45A3	ENSG00000269096.6	0	0
protein_coding	CT45A5	ENSG00000228836.8	0	0
protein_coding	CT45A6	ENSG00000278289.4	0	0

protein_coding	CT45A2	ENSG00000271449.7	0	0
protein_coding	CT45A7	ENSG00000273696.4	0	0
protein_coding	CT45A8	ENSG00000278085.4	0	0
protein_coding	CT45A9	ENSG00000270946.5	0	0
protein_coding	CT45A10	ENSG00000269586.7	0	0
protein_coding	SAGE1	ENSG00000181433.9	0	0
protein_coding	ADGRG4	ENSG00000156920.10	0	0
protein_coding	BRS3	ENSG00000102239.4	0	0
protein_coding	CD40LG	ENSG00000102245.7	0	0
protein_coding	GPR101	ENSG00000165370.1	0	0
protein_coding	ZIC3	ENSG00000156925.11	0	0
protein_coding	F9	ENSG00000101981.11	0	0
protein_coding	MCF¬†2	ENSG00000101977.20	0	0
protein_coding	CXorf66	ENSG00000203933.2	0	0
protein_coding	SOX3	ENSG00000134595.8	0	0
protein_coding	CDR1	ENSG00000184258.7	0	0
protein_coding	SPANXC	ENSG00000198573.6	0	0
protein_coding	SPANXA1	ENSG00000198021.7	0	0
protein_coding	SPANXA2	ENSG00000203926.4	0	0
protein_coding	SPANXD	ENSG00000196406.4	0	0
protein_coding	MAGEC3	ENSG00000165509.13	0	0
protein_coding	MAGEC1	ENSG00000155495.8	0	0
protein_coding	SPANXN4	ENSG00000189326.4	0	0
protein_coding	SLITRK4	ENSG00000179542.15	0	0
protein_coding	SPANXN2	ENSG00000268988.1	0	0
protein_coding	SPANXN1	ENSG00000203923.4	0	0
protein_coding	SLITRK2	ENSG00000185985.9	0	0
protein_coding	CXorf51B	ENSG00000235699.2	0	0
protein_coding	CXorf51A	ENSG00000224440.1	0	0
protein_coding	FMR1NB	ENSG00000176988.8	0	0
protein_coding	AFF2	ENSG00000155966.13	0	0
protein_coding	AC244197.3	ENSG00000241489.7	0	0
protein_coding	HSFX3	ENSG00000283697.2	0	0
protein_coding	MAGEA9B	ENSG00000267978.5	0	0
protein_coding	HSFX2	ENSG00000268738.2	0	0
protein_coding	MAGEA11	ENSG00000185247.14	0	0
protein_coding	HSFX1	ENSG00000171116.7	0	0
protein_coding	MAGEA9	ENSG00000123584.7	0	0
protein_coding	MAGEA8	ENSG00000156009.9	0	0
protein_coding	HSFX4	ENSG00000283463.1	0	0
protein_coding	GPR50	ENSG00000102195.9	0	0
protein_coding	PASD1	ENSG00000166049.10	0	0
protein_coding	PRRG3	ENSG00000130032.15	0	0
protein_coding	FATE1	ENSG00000147378.11	0	0

protein_coding	CNGA2	ENSG00000183862.5	0	0
protein_coding	MAGEA4	ENSG00000147381.11	0	0
protein_coding	GABRQ	ENSG00000268089.2	0	0
protein_coding	CSAG2	ENSG00000268902.3	0	0
protein_coding	MAGEA2B	ENSG00000183305.13	0	0
protein_coding	CSAG1	ENSG00000198930.12	0	0
protein_coding	MAGEA2	ENSG00000268606.5	0	0
protein_coding	CSAG3	ENSG00000268916.6	0	0
protein_coding	MAGEA6	ENSG00000197172.10	0	0
protein_coding	PNMA5	ENSG00000198883.11	0	0
protein_coding	MAGEA1	ENSG00000198681.6	0	0
protein_coding	PNMA6F	ENSG00000225110.2	0	0
protein_coding	PNMA6E	ENSG00000214897.4	0	0
protein_coding	TREX2	ENSG00000183479.12	0	0
protein_coding	PDZD4	ENSG00000067840.12	0	0
protein_coding	U52112.1	ENSG00000284987.1	0	0
protein_coding	AVPR2	ENSG00000126895.14	0	0
protein_coding	OPN1LW	ENSG00000102076.9	0	0
protein_coding	OPN1MW	ENSG00000268221.5	0	0
protein_coding	OPN1MW2	ENSG00000166160.9	0	0
protein_coding	OPN1MW3	ENSG00000269433.3	0	0
protein_coding	TEX28	ENSG00000278057.4	0	0
protein_coding	TKTL1	ENSG00000007350.16	0	0
protein_coding	IKBKG	ENSG00000269335.5	0	0
protein_coding	CTAG1A	ENSG00000268651.2	0	0
protein_coding	CTAG1B	ENSG00000184033.13	0	0
protein_coding	CTAG2	ENSG00000126890.13	0	0
protein_coding	SMIM9	ENSG00000203870.5	0	0
protein_coding	H2AFB1	ENSG00000274183.1	0	0
protein_coding	CMC4	ENSG00000182712.15	0	0
protein_coding	RAB39B	ENSG00000155961.4	0	0
protein_coding	H2AFB2	ENSG00000277858.1	0	0
protein_coding	F8A2	ENSG00000274791.1	0	0
protein_coding	F8A3	ENSG00000277150.1	0	0
protein_coding	H2AFB3	ENSG00000277745.1	0	0
protein_coding	PAR	ENSG00000124334.17	0	0
protein_coding	SRY	ENSG00000184895.7	0	0
protein_coding	TGIF2LY	ENSG00000176679.8	0	0
protein_coding	PCDH11Y	ENSG00000099715.14	0	0
protein_coding	TSPY2	ENSG00000168757.12	0	0
protein_coding	AMELY	ENSG00000099721.13	0	0
protein_coding	TBL1Y	ENSG00000092377.13	0	0
protein_coding	TSPY4	ENSG00000233803.9	0	0
protein_coding	TSPY8	ENSG00000229549.9	0	0

protein_coding	TSPY3	ENSG00000228927.10	0	0
protein_coding	TSPY1	ENSG00000258992.6	0	0
protein_coding	TSPY10	ENSG00000236424.7	0	0
protein_coding	DDX3Y	ENSG00000067048.16	0	0
protein_coding	UTY	ENSG00000183878.15	0	0
protein_coding	TMSB4Y	ENSG00000154620.5	0	0
protein_coding	VCY	ENSG00000129864.6	0	0
protein_coding	VCY1B	ENSG00000129862.6	0	0
protein_coding	CDY2B	ENSG00000129873.7	0	0
protein_coding	CDY2A	ENSG00000182415.9	0	0
protein_coding	HSFY1	ENSG00000172468.13	0	0
protein_coding	HSFY2	ENSG00000169953.11	0	0
protein_coding	KDM5D	ENSG00000012817.15	0	0
protein_coding	RPS4Y2	ENSG00000280969.1	0	0
protein_coding	PRORY	ENSG00000183146.4	0	0
protein_coding	RBMY1B	ENSG00000242875.6	0	0
protein_coding	RBMY1A1	ENSG00000234414.7	0	0
protein_coding	RBMY1D	ENSG00000244395.6	0	0
protein_coding	RBMY1E	ENSG00000242389.8	0	0
protein_coding	PRY2	ENSG00000169807.10	0	0
protein_coding	RBMY1F	ENSG00000169800.13	0	0
protein_coding	RBMY1J	ENSG00000226941.8	0	0
protein_coding	PRY	ENSG00000169789.10	0	0
protein_coding	BPY2	ENSG00000183753.10	0	0
protein_coding	DAZ1	ENSG00000188120.14	0	0
protein_coding	DAZ2	ENSG00000205944.11	0	0
protein_coding	CDY1B	ENSG00000172352.5	0	0
protein_coding	BPY2B	ENSG00000183795.8	0	0
protein_coding	DAZ3	ENSG00000187191.14	0	0
protein_coding	DAZ4	ENSG00000205916.11	0	0
protein_coding	BPY2C	ENSG00000185894.8	0	0
protein_coding	CDY1	ENSG00000172288.7	0	0
protein_coding	BX004987.1	ENSG00000278704.1	0	0
protein_coding	AC145212.1	ENSG00000277400.1	0	0
protein_coding	MAFIP	ENSG00000274847.1	0	0
protein_coding	AC011043.2	ENSG00000278198.1	0	0
protein_coding	AC011841.1	ENSG00000273496.1	0	0
protein_coding	BX072566.1	ENSG00000277630.4	0	0
protein_coding	AC213203.2	ENSG00000277475.1	0	0
protein_coding	AC213203.1	ENSG00000268674.2	0	0
protein_coding	AC004556.1	ENSG00000276345.1	0	0
protein_coding	AC233755.2	ENSG00000277856.1	0	0
protein_coding	AC233755.1	ENSG00000275063.1	0	0
protein_coding	AC136352.3	ENSG00000277666.1	0	0

protein_coding	AC136352.2	ENSG00000276760.4	0	0
protein_coding	AC171558.3	ENSG00000275249.1	0	0
protein_coding	AC171558.1	ENSG00000274792.1	0	0
protein_coding	AC133551.1	ENSG00000274175.1	0	0
protein_coding	AC136612.1	ENSG00000275869.1	0	0
protein_coding	AC136616.1	ENSG00000273554.4	0	0
protein_coding	AC136616.3	ENSG00000278782.1	0	0
protein_coding	AC136616.2	ENSG00000277761.1	0	0
protein_coding	AC141272.1	ENSG00000277836.1	0	0
protein_coding	AC023491.2	ENSG00000278633.1	0	0
protein_coding	AC007325.1	ENSG00000276017.1	0	0
		ENSG00000228572.7_PA	0	0
		ENSG00000182378.13_I	0	0
		ENSG00000178605.13_I	0	0
		ENSG00000226179.6_PA	0	0
		ENSG00000167393.17_I	0	0
		ENSG00000281849.3_PA	0	0
		ENSG00000275287.5_PA	0	0
		ENSG00000280767.3_PA	0	0
		ENSG00000234958.6_PA	0	0
		ENSG00000229232.6_PA	0	0
		ENSG00000185960.14_I	0	0
		ENSG00000237531.6_PA	0	0
		ENSG00000225661.7_PA	0	0
		ENSG00000205755.11_I	0	0
		ENSG00000198223.16_I	0	0
		ENSG00000265658.6_PA	0	0
		ENSG00000223274.6_PA	0	0
		ENSG00000185291.11_I	0	0
		ENSG00000169100.13_I	0	0
		ENSG00000236871.7_PA	0	0
		ENSG00000236017.8_PA	0	0
		ENSG00000169093.15_I	0	0
		ENSG00000182162.10_I	0	0
		ENSG00000197976.11_I	0	0
		ENSG00000196433.12_I	0	0
		ENSG00000223511.7_PA	0	0
		ENSG00000234622.6_PA	0	0
		ENSG00000169084.13_I	0	0
		ENSG00000223571.6_PA	0	0
		ENSG00000214717.11_I	0	0
		ENSG00000277120.5_PA	0	0
		ENSG00000223773.7_PA	0	0
		ENSG00000230542.6_PA	0	0

		ENSG00000002586.19_P	0	0
		ENSG00000168939.11_P	0	0
		ENSG00000237801.6_PA	0	0
		ENSG00000237040.6_PA	0	0
		ENSG00000124333.15_P	0	0
		ENSG00000228410.6_PA	0	0
		ENSG00000223484.7_PA	0	0
		ENSG00000124334.17_P	0	0
		ENSG00000270726.6_PA	0	0
		ENSG00000185203.12_P	0	0
		ENSG00000182484.15_P	0	0
		ENSG00000227159.8_PA	0	0
protein_coding	LCK	ENSG00000182866.16	0	0
protein_coding	TAC3	ENSG00000166863.11	0	0
protein_coding	IGFLR1	ENSG00000126246.9	0	0
protein_coding	AMT	ENSG00000145020.15	0	0
protein_coding	ADGRG7	ENSG00000144820.7	0	0
protein_coding	PITX3	ENSG00000107859.9	0	0
protein_coding	C10orf82	ENSG00000165863.16	0	0
protein_coding	GLS2	ENSG00000135423.12	0	0
protein_coding	PTPRQ	ENSG00000139304.13	0	0
protein_coding	OGFOD2	ENSG00000111325.16	0	0
protein_coding	AC107871.1	ENSG00000260007.3	0	0
protein_coding	FSD2	ENSG00000186628.12	0	0
protein_coding	HSPB7	ENSG00000173641.17	0	0
protein_coding	TCTEX1D1	ENSG00000152760.9	0	0
protein_coding	INSL5	ENSG00000172410.4	0	0
protein_coding	LRRC53	ENSG00000162621.6	0	0
protein_coding	ERICH3	ENSG00000178965.13	0	0
protein_coding	DCST2	ENSG00000163354.14	0	0
protein_coding	MYOC	ENSG00000034971.16	0	0
protein_coding	LHX4	ENSG00000121454.5	0	0
protein_coding	POMC	ENSG00000115138.10	0	0
protein_coding	CDKL4	ENSG00000205111.8	0	0
protein_coding	C2orf91	ENSG00000205086.7	0	0
protein_coding	OXER1	ENSG00000162881.6	0	0
protein_coding	SLC4A5	ENSG00000188687.17	0	0
protein_coding	GPAT2	ENSG00000186281.12	0	0
protein_coding	AC079447.1	ENSG00000241962.9	0	0
protein_coding	CXCR4	ENSG00000121966.6	0	0
protein_coding	CD28	ENSG00000178562.17	0	0
protein_coding	COLQ	ENSG00000206561.12	0	0
protein_coding	SLC22A14	ENSG00000144671.10	0	0
protein_coding	SLC38A3	ENSG00000188338.14	0	0

protein_coding	CACNA2D2	ENSG00000007402.11	0	0
protein_coding	PLSCR2	ENSG00000163746.11	0	0
protein_coding	ZIC4	ENSG00000174963.17	0	0
protein_coding	RTP1	ENSG00000175077.5	0	0
protein_coding	PDE6B	ENSG00000133256.12	0	0
protein_coding	RHOH	ENSG00000168421.12	0	0
protein_coding	RRH	ENSG00000180245.4	0	0
protein_coding	UBE2QL1	ENSG00000215218.3	0	0
protein_coding	MARCH11	ENSG00000183654.8	0	0
protein_coding	HCN1	ENSG00000164588.6	0	0
protein_coding	CDC20B	ENSG00000164287.12	0	0
protein_coding	PCDHA4	ENSG00000204967.10	0	0
protein_coding	PCDHA7	ENSG00000204963.5	0	0
protein_coding	DRD1	ENSG00000184845.3	0	0
protein_coding	ZNF454	ENSG00000178187.7	0	0
protein_coding	IRF4	ENSG00000137265.14	0	0
protein_coding	PXT1	ENSG00000179165.10	0	0
protein_coding	TCTE1	ENSG00000146221.9	0	0
protein_coding	KIAA0408	ENSG00000189367.14	0	0
protein_coding	SAMD3	ENSG00000164483.16	0	0
protein_coding	FAM221A	ENSG00000188732.10	0	0
protein_coding	CLDN3	ENSG00000165215.6	0	0
protein_coding	STEAP4	ENSG00000127954.12	0	0
protein_coding	AC004922.1	ENSG00000284292.1	0	0
protein_coding	FBXO24	ENSG00000106336.12	0	0
protein_coding	ASZ1	ENSG00000154438.7	0	0
protein_coding	TNFRSF10C	ENSG00000173535.14	0	0
protein_coding	DCSTAMP	ENSG00000164935.6	0	0
protein_coding	SAXO1	ENSG00000155875.14	0	0
protein_coding	PRSS3	ENSG00000010438.16	0	0
protein_coding	PKN3	ENSG00000160447.6	0	0
protein_coding	RASGEF1A	ENSG00000198915.11	0	0
protein_coding	C10orf105	ENSG00000214688.5	0	0
protein_coding	SPRN	ENSG00000203772.7	0	0
protein_coding	KCNC1	ENSG00000129159.7	0	0
protein_coding	TRIM48	ENSG00000150244.11	0	0
protein_coding	LRRC10B	ENSG00000204950.3	0	0
protein_coding	C11orf86	ENSG00000173237.4	0	0
protein_coding	OR8A1	ENSG00000196119.7	0	0
protein_coding	OVCH1	ENSG00000187950.8	0	0
protein_coding	AC068987.5	ENSG00000284730.1	0	0
protein_coding	AVPR1A	ENSG00000166148.3	0	0
protein_coding	LGR5	ENSG00000139292.12	0	0
protein_coding	CLLU1OS	ENSG00000205057.5	0	0

protein_coding	CCDC169	ENSG00000242715.7	0	0
protein_coding	ERCC5	ENSG00000134899.19	0	0
protein_coding	CARMIL3	ENSG00000186648.14	0	0
protein_coding	AL121594.1	ENSG00000258790.1	0	0
protein_coding	SERPINA6	ENSG00000170099.5	0	0
protein_coding	LTK	ENSG00000062524.15	0	0
protein_coding	USP50	ENSG00000170236.14	0	0
protein_coding	CTXND1	ENSG00000259417.2	0	0
protein_coding	NPW	ENSG00000183971.7	0	0
protein_coding	PKD1L3	ENSG00000277481.1	0	0
protein_coding	STAC2	ENSG00000141750.6	0	0
protein_coding	GSDMA	ENSG00000167914.11	0	0
protein_coding	GNGT2	ENSG00000167083.6	0	0
protein_coding	NPB	ENSG00000183979.7	0	0
protein_coding	LIPG	ENSG00000101670.11	0	0
protein_coding	ODF3L2	ENSG00000181781.9	0	0
protein_coding	CELF5	ENSG00000161082.12	0	0
protein_coding	OR1I1	ENSG00000094661.3	0	0
protein_coding	FLT3LG	ENSG00000090554.12	0	0
protein_coding	KCNC3	ENSG00000131398.13	0	0
protein_coding	SYT3	ENSG00000213023.10	0	0
protein_coding	CACNG6	ENSG00000130433.7	0	0
protein_coding	MATN4	ENSG00000124159.15	0	0
protein_coding	SERPIND1	ENSG00000099937.10	0	0
protein_coding	GSTT2B	ENSG00000133433.10	0	0
protein_coding	NFAM1	ENSG00000235568.6	0	0
protein_coding	PAR	ENSG00000205755.11	0	0
protein_coding	FAM9B	ENSG00000177138.15	0	0
protein_coding	MAGEB10	ENSG00000177689.10	0	0
protein_coding	GPR82	ENSG00000171657.5	0	0
protein_coding	STARD8	ENSG00000130052.13	0	0
protein_coding	TCEAL2	ENSG00000184905.8	0	0
protein_coding	TMEM31	ENSG00000179363.6	0	0
protein_coding	RIMBP3C	ENSG00000183246.6	0	0
protein_coding	SLC35F3	ENSG00000183780.12	0	0
protein_coding	CST2	ENSG00000170369.3	0	0
protein_coding	MFAP2	ENSG00000117122.13	0	0
protein_coding	CLDN19	ENSG00000164007.10	0	0
protein_coding	GBP7	ENSG00000213512.2	0	0
protein_coding	AC017083.3	ENSG00000273398.6	0	0
protein_coding	SH2D6	ENSG00000152292.16	0	0
protein_coding	FAM178B	ENSG00000168754.14	0	0
protein_coding	MERTK	ENSG00000153208.16	0	0
protein_coding	NMUR1	ENSG00000171596.6	0	0

protein_coding	ADCY5	ENSG00000173175.14	0	0
protein_coding	ERICH6	ENSG00000163645.14	0	0
protein_coding	ANKDD1B	ENSG00000189045.13	0	0
protein_coding	PHF24	ENSG00000122733.12	0	0
protein_coding	PALD1	ENSG00000107719.8	0	0
protein_coding	INSYN2	ENSG00000188916.8	0	0
protein_coding	MMP27	ENSG00000137675.4	0	0
protein_coding	ESAM	ENSG00000149564.11	0	0
protein_coding	SLC6A12	ENSG00000111181.12	0	0
protein_coding	RPS6KL1	ENSG00000198208.11	0	0
protein_coding	MEGF11	ENSG00000157890.17	0	0
protein_coding	SMPD3	ENSG00000103056.11	0	0
protein_coding	SLC16A11	ENSG00000174326.11	0	0
protein_coding	SPACA3	ENSG00000141316.12	0	0
protein_coding	CACNA1G	ENSG00000006283.17	0	0
protein_coding	LDLRAD4	ENSG00000168675.18	0	0
protein_coding	LRFN1	ENSG00000128011.4	0	0
protein_coding	VN1R1	ENSG00000178201.4	0	0
protein_coding	EBF4	ENSG00000088881.20	0	0
protein_coding	CST1	ENSG00000170373.8	0	0
protein_coding	ICOSLG	ENSG00000160223.17	0	0
protein_coding	AIRE	ENSG00000160224.16	0	0
protein_coding	ATP1B2	ENSG00000129244.8	0	0
protein_coding	SAXO2	ENSG00000188659.9	0	0
protein_coding	IMPG2	ENSG00000081148.11	0	0
protein_coding	JAKMIP2	ENSG00000176049.15	0	0
protein_coding	DIO3	ENSG00000197406.7	0	0
protein_coding	NEUROD2	ENSG00000171532.4	0	0
protein_coding	MEIOC	ENSG00000180336.17	0	0
protein_coding	GRIN2C	ENSG00000161509.13	0	0
protein_coding	PSG3	ENSG00000221826.9	0	0
protein_coding	PRR34	ENSG00000182257.7	0	0
protein_coding	RTL9	ENSG00000243978.8	0	0
protein_coding	RENBP	ENSG00000102032.12	0	0
protein_coding	JCHAIN	ENSG00000132465.10	0	0
protein_coding	CATSPER3	ENSG00000152705.7	0	0
protein_coding	GLP2R	ENSG00000065325.12	0	0
protein_coding	HAS1	ENSG00000105509.10	0	0
protein_coding	EFCAB13	ENSG00000178852.15	0	0
protein_coding	PCDHGA4	ENSG00000262576.2	0	0
protein_coding	BMP3	ENSG00000152785.6	0	0
protein_coding	ESR2	ENSG00000140009.18	0	0
protein_coding	SLC38A8	ENSG00000166558.10	0	0
protein_coding	ZCCHC12	ENSG00000174460.3	0	0

protein_coding	CCL7	ENSG00000108688.11	0	0
protein_coding	EGR2	ENSG00000122877.15	0	0
protein_coding	DCHS2	ENSG00000197410.13	0	0
protein_coding	ASNS	ENSG00000070669.16	0	0
protein_coding	NT5DC4	ENSG00000144130.11	0	0
protein_coding	TLR2	ENSG00000137462.7	0	0
protein_coding	PKIB	ENSG00000135549.14	0	0
protein_coding	POMK	ENSG00000185900.9	0	0
protein_coding	PHYHIPL	ENSG00000165443.11	0	0
protein_coding	PNLIPRP1	ENSG00000187021.14	0	0
protein_coding	GGT6	ENSG00000167741.10	0	0
protein_coding	KCNJ12	ENSG00000184185.9	0	0
protein_coding	FAM83E	ENSG00000105523.3	0	0
protein_coding	ZNF749	ENSG00000186230.6	0	0
protein_coding	TMEM52	ENSG00000178821.12	0	0
protein_coding	BMP8B	ENSG00000116985.11	0	0
protein_coding	MYBPHL	ENSG00000221986.6	0	0
protein_coding	SLAMF9	ENSG00000162723.9	0	0
protein_coding	RAD51AP2	ENSG00000214842.5	0	0
protein_coding	TOGARAM2	ENSG00000189350.12	0	0
protein_coding	DCAF4L1	ENSG00000182308.6	0	0
protein_coding	SLCO6A1	ENSG00000205359.9	0	0
protein_coding	MGAM	ENSG00000257335.8	0	0
protein_coding	KCNK9	ENSG00000169427.7	0	0
protein_coding	FAM166B	ENSG00000215187.10	0	0
protein_coding	C10orf95	ENSG00000120055.7	0	0
protein_coding	VWA2	ENSG00000165816.12	0	0
protein_coding	JAKMIP3	ENSG00000188385.11	0	0
protein_coding	TAS2R19	ENSG00000212124.2	0	0
protein_coding	BCL2L14	ENSG00000121380.12	0	0
protein_coding	KRT86	ENSG00000170442.11	0	0
protein_coding	ITGAM	ENSG00000169896.17	0	0
protein_coding	CCL17	ENSG00000102970.10	0	0
protein_coding	C17orf113	ENSG00000267221.2	0	0
protein_coding	MYOM1	ENSG00000101605.12	0	0
protein_coding	MUC16	ENSG00000181143.15	0	0
protein_coding	LRRC74B	ENSG00000187905.10	0	0
protein_coding	GDPD2	ENSG00000130055.13	0	0
protein_coding	ESX1	ENSG00000123576.5	0	0
protein_coding	MMEL1	ENSG00000142606.15	0	0
protein_coding	CHD5	ENSG00000116254.17	0	0
protein_coding	CA6	ENSG00000131686.14	0	0
protein_coding	C1orf167	ENSG00000215910.7	0	0
protein_coding	ELAVL4	ENSG00000162374.16	0	0

protein_coding	TXNDC12	ENSG00000117862.12	0	0
protein_coding	FOXD3	ENSG00000187140.5	0	0
protein_coding	NR1I3	ENSG00000143257.11	0	0
protein_coding	CCDC190	ENSG00000185860.13	0	0
protein_coding	MYOCOS	ENSG00000283683.1	0	0
protein_coding	PDC	ENSG00000116703.13	0	0
protein_coding	SPATA45	ENSG00000185523.6	0	0
protein_coding	C2orf70	ENSG00000173557.14	0	0
protein_coding	GKN2	ENSG00000183607.9	0	0
protein_coding	GKN1	ENSG00000169605.5	0	0
protein_coding	MRPL53	ENSG00000204822.6	0	0
protein_coding	DNAH6	ENSG00000115423.18	0	0
protein_coding	ATG9A	ENSG00000198925.11	0	0
protein_coding	UGT1A7	ENSG00000244122.2	0	0
protein_coding	KIF1A	ENSG00000130294.16	0	0
protein_coding	CCR3	ENSG00000183625.14	0	0
protein_coding	ZPLD1	ENSG00000170044.8	0	0
protein_coding	BTLA	ENSG00000186265.9	0	0
protein_coding	UPK1B	ENSG00000114638.7	0	0
protein_coding	FOXL2NB	ENSG00000206262.8	0	0
protein_coding	ZIC1	ENSG00000152977.9	0	0
protein_coding	HRG	ENSG00000113905.4	0	0
protein_coding	ATP13A5	ENSG00000187527.10	0	0
protein_coding	CPLX1	ENSG00000168993.14	0	0
protein_coding	MSANTD1	ENSG00000188981.10	0	0
protein_coding	CPZ	ENSG00000109625.18	0	0
protein_coding	CCSER1	ENSG00000184305.15	0	0
protein_coding	ATOH1	ENSG00000172238.4	0	0
protein_coding	CDH10	ENSG00000040731.10	0	0
protein_coding	MCIDAS	ENSG00000234602.7	0	0
protein_coding	TEX43	ENSG00000196900.4	0	0
protein_coding	GRXCR2	ENSG00000204928.2	0	0
protein_coding	SPINK7	ENSG00000145879.10	0	0
protein_coding	NMUR2	ENSG00000132911.4	0	0
protein_coding	PPP1R2B	ENSG00000231989.4	0	0
protein_coding	KIAA0319	ENSG00000137261.13	0	0
protein_coding	HIST1H3C	ENSG00000278272.1	0	0
protein_coding	OR2B6	ENSG00000124657.1	0	0
protein_coding	LTB	ENSG00000227507.2	0	0
protein_coding	HLA-DRB1	ENSG00000196126.11	0	0
protein_coding	HLA-DMB	ENSG00000242574.8	0	0
protein_coding	UNC5CL	ENSG00000124602.9	0	0
protein_coding	PTP4A1	ENSG00000112245.11	0	0
protein_coding	SLC35F1	ENSG00000196376.10	0	0

protein_coding	IYD	ENSG00000009765.14	0	0
protein_coding	HOXA6	ENSG00000106006.6	0	0
protein_coding	GALNT17	ENSG00000185274.11	0	0
protein_coding	PON1	ENSG00000005421.8	0	0
protein_coding	GJC3	ENSG00000176402.5	0	0
protein_coding	ZAN	ENSG00000146839.18	0	0
protein_coding	MYL10	ENSG00000106436.6	0	0
protein_coding	POLR2J2	ENSG00000228049.7	0	0
protein_coding	KLF14	ENSG00000266265.3	0	0
protein_coding	C8orf74	ENSG00000171060.10	0	0
protein_coding	FGL1	ENSG00000104760.16	0	0
protein_coding	ZMAT4	ENSG00000165061.14	0	0
protein_coding	SNTG1	ENSG00000147481.15	0	0
protein_coding	TTPA	ENSG00000137561.4	0	0
protein_coding	ANXA13	ENSG00000104537.16	0	0
protein_coding	POU5F1B	ENSG00000212993.5	0	0
protein_coding	PRDM12	ENSG00000130711.3	0	0
protein_coding	AKR1C4	ENSG00000198610.10	0	0
protein_coding	CALML5	ENSG00000178372.7	0	0
protein_coding	ACBD7	ENSG00000176244.6	0	0
protein_coding	FXYD4	ENSG00000150201.14	0	0
protein_coding	MYOZ1	ENSG00000177791.11	0	0
protein_coding	CUZD1	ENSG00000138161.13	0	0
protein_coding	KRTAP5-2	ENSG00000205867.3	0	0
protein_coding	RBMXL2	ENSG00000170748.6	0	0
protein_coding	CNTF	ENSG00000242689.2	0	0
protein_coding	CD6	ENSG00000013725.14	0	0
protein_coding	FGF19	ENSG00000162344.3	0	0
protein_coding	TEX12	ENSG00000150783.9	0	0
protein_coding	DDX25	ENSG00000109832.13	0	0
protein_coding	IQSEC3	ENSG00000120645.11	0	0
protein_coding	TAS2R20	ENSG00000255837.1	0	0
protein_coding	SPX	ENSG00000134548.10	0	0
protein_coding	CCDC65	ENSG00000139537.10	0	0
protein_coding	ALX1	ENSG00000180318.3	0	0
protein_coding	NTS	ENSG00000133636.10	0	0
protein_coding	MAP1LC3B2	ENSG00000258102.3	0	0
protein_coding	PCDH20	ENSG00000280165.1	0	0
protein_coding	RNASE4	ENSG00000258818.3	0	0
protein_coding	GZMB	ENSG00000100453.12	0	0
protein_coding	RAD51	ENSG00000051180.16	0	0
protein_coding	C15orf62	ENSG00000188277.9	0	0
protein_coding	SNX22	ENSG00000157734.13	0	0
protein_coding	CALML4	ENSG00000129007.14	0	0

protein_coding	CFAP161	ENSG00000156206.13	0	0
protein_coding	SPN	ENSG00000197471.11	0	0
protein_coding	MYLK3	ENSG00000140795.12	0	0
protein_coding	ABCC11	ENSG00000121270.15	0	0
protein_coding	HYDIN	ENSG00000157423.17	0	0
protein_coding	C16orf95	ENSG00000260456.6	0	0
protein_coding	DPH1	ENSG00000108963.17	0	0
protein_coding	P2RX1	ENSG00000108405.3	0	0
protein_coding	AIPL1	ENSG00000129221.15	0	0
protein_coding	RNF222	ENSG00000189051.5	0	0
protein_coding	TBC1D3I	ENSG00000274933.5	0	0
protein_coding	ZNF385C	ENSG00000187595.16	0	0
protein_coding	PTGES3L	ENSG00000267060.5	0	0
protein_coding	NOL4	ENSG00000101746.15	0	0
protein_coding	VAV1	ENSG00000141968.7	0	0
protein_coding	ZNF625	ENSG00000257591.5	0	0
protein_coding	SYCE2	ENSG00000161860.7	0	0
protein_coding	PALM3	ENSG00000187867.8	0	0
protein_coding	OR10H5	ENSG00000172519.9	0	0
protein_coding	CHST8	ENSG00000124302.12	0	0
protein_coding	LGALS4	ENSG00000171747.8	0	0
protein_coding	CD33	ENSG00000105383.14	0	0
protein_coding	LILRA2	ENSG00000239998.5	0	0
protein_coding	RSPO4	ENSG00000101282.8	0	0
protein_coding	C20orf202	ENSG00000215595.1	0	0
protein_coding	SPEF1	ENSG00000101222.12	0	0
protein_coding	HNF4A	ENSG00000101076.16	0	0
protein_coding	COL20A1	ENSG00000101203.16	0	0
protein_coding	SIK1B	ENSG00000275993.2	0	0
protein_coding	NCF4	ENSG00000100365.14	0	0
protein_coding	MIOX	ENSG00000100253.12	0	0
protein_coding	CYBB	ENSG00000165168.7	0	0
protein_coding	ITGB1BP2	ENSG00000147166.10	0	0
protein_coding	FRMPD3	ENSG00000147234.10	0	0
protein_coding	RHOXF1	ENSG00000101883.4	0	0
protein_coding	GLUD2	ENSG00000182890.4	0	0
protein_coding	MAGEC2	ENSG00000046774.9	0	0
protein_coding	HAUS7	ENSG00000213397.10	0	0
protein_coding	AC007325.2	ENSG00000277196.4	0	0
protein_coding	TMEM232	ENSG00000186952.14	0	0
protein_coding	LSM¬†12	ENSG00000161654.9	0	0
protein_coding	RIMKLA	ENSG00000177181.14	0	0
protein_coding	LAT2	ENSG00000086730.16	0	0
protein_coding	FITM1	ENSG00000139914.6	0	0

protein_coding	SYN2	ENSG00000157152.16	0	0
protein_coding	CCDC160	ENSG00000203952.9	0	0
protein_coding	GPR156	ENSG00000175697.10	0	0
protein_coding	UTS2B	ENSG00000188958.9	0	0
protein_coding	AL365205.1	ENSG00000124593.16	0	0
protein_coding	HS3ST5	ENSG00000249853.7	0	0
protein_coding	ZNF483	ENSG00000173258.12	0	0
protein_coding	CHRNA10	ENSG00000129749.3	0	0
protein_coding	SHISA9	ENSG00000237515.8	0	0
protein_coding	USP6	ENSG00000129204.16	0	0
protein_coding	RAB37	ENSG00000172794.19	0	0
protein_coding	ARMCX6	ENSG00000198960.10	0	0
protein_coding	PAK3	ENSG00000077264.14	0	0
protein_coding	NLGN4Y	ENSG00000165246.14	0	0
protein_coding	SLFN12L	ENSG00000205045.8	0	0
protein_coding	TNFSF18	ENSG00000120337.8	0	0
protein_coding	SPP2	ENSG00000072080.10	0	0
protein_coding	ANKUB1	ENSG00000206199.10	0	0
protein_coding	ZDHHC11	ENSG00000188818.12	0	0
protein_coding	AKR1E2	ENSG00000165568.17	0	0
protein_coding	C14orf39	ENSG00000179008.8	0	0
protein_coding	AC240274.1	ENSG00000271254.6	0	0
protein_coding	RANBP3L	ENSG00000164188.8	0	0
protein_coding	AK7	ENSG00000140057.8	0	0
protein_coding	KLF17	ENSG00000171872.4	0	0
protein_coding	FAM169B	ENSG00000283597.2	0	0
protein_coding	ZNF695	ENSG00000197472.14	0	0
protein_coding	ASB2	ENSG00000100628.11	0	0
protein_coding	MCEMP1	ENSG00000183019.7	0	0
protein_coding	MFSD14A	ENSG00000156875.13	0	0
protein_coding	SOWAHA	ENSG00000198944.5	0	0
protein_coding	TMEM270	ENSG00000175877.3	0	0
protein_coding	ENKUR	ENSG00000151023.16	0	0
protein_coding	DNAJB13	ENSG00000187726.8	0	0
protein_coding	AMN	ENSG00000166126.10	0	0
protein_coding	AC013394.1	ENSG00000279765.3	0	0
protein_coding	CCL22	ENSG00000102962.4	0	0
protein_coding	ALOX15B	ENSG00000179593.15	0	0
protein_coding	TNFSF14	ENSG00000125735.10	0	0
protein_coding	RAVER1	ENSG00000161847.13	0	0
protein_coding	SULT2A1	ENSG00000105398.3	0	0
protein_coding	CST9	ENSG00000173335.4	0	0
protein_coding	TMEM121B	ENSG00000183307.3	0	0
protein_coding	GPR143	ENSG00000101850.12	0	0

protein_coding	TMEM56-RWDD3	ENSG00000271092.5	0	0
protein_coding	C2CD4D	ENSG00000225556.1	0	0
protein_coding	RGS8	ENSG00000135824.12	0	0
protein_coding	HIST3H2BB	ENSG00000196890.4	0	0
protein_coding	AC007240.1	ENSG00000284681.2	0	0
protein_coding	ATP6V1C2	ENSG00000143882.11	0	0
protein_coding	SPDYA	ENSG00000163806.15	0	0
protein_coding	CAPN14	ENSG00000214711.9	0	0
protein_coding	NME9	ENSG00000181322.13	0	0
protein_coding	PAQR9	ENSG00000188582.8	0	0
protein_coding	ADH7	ENSG00000196344.11	0	0
protein_coding	OCLN	ENSG00000197822.10	0	0
protein_coding	KCNN2	ENSG00000080709.15	0	0
protein_coding	HIST1H2AH	ENSG00000274997.1	0	0
protein_coding	HIST1H2BO	ENSG00000274641.1	0	0
protein_coding	CDSN	ENSG00000204539.3	0	0
protein_coding	LHFPL5	ENSG00000197753.9	0	0
protein_coding	AGR3	ENSG00000173467.8	0	0
protein_coding	WIPF3	ENSG00000122574.10	0	0
protein_coding	METTL27	ENSG00000165171.10	0	0
protein_coding	GIMAP7	ENSG00000179144.4	0	0
protein_coding	CNPY1	ENSG00000146910.12	0	0
protein_coding	HNF4G	ENSG00000164749.11	0	0
protein_coding	LHX2	ENSG00000106689.10	0	0
protein_coding	ADARB2	ENSG00000185736.15	0	0
protein_coding	TDRD1	ENSG00000095627.9	0	0
protein_coding	OR51D1	ENSG00000197428.3	0	0
protein_coding	WT1	ENSG00000184937.13	0	0
protein_coding	RBM14-	ENSG00000248643.5	0	0
protein_coding	C11orf97	ENSG00000257057.2	0	0
protein_coding	LMO3	ENSG00000048540.14	0	0
protein_coding	CAPZA3	ENSG00000177938.4	0	0
protein_coding	H3F3C	ENSG00000188375.4	0	0
protein_coding	KRT81	ENSG00000205426.10	0	0
protein_coding	BEST3	ENSG00000127325.18	0	0
protein_coding	TSPAN8	ENSG00000127324.8	0	0
protein_coding	HYPK	ENSG00000242028.6	0	0
protein_coding	PSMB10	ENSG00000205220.11	0	0
protein_coding	CCDC144A	ENSG00000170160.17	0	0
protein_coding	KRT20	ENSG00000171431.3	0	0
protein_coding	RUNDC3A	ENSG00000108309.13	0	0
protein_coding	BCKDHA	ENSG00000248098.11	0	0

protein_coding	CD79A	ENSG00000105369.9	0	0
protein_coding	KCNQ2	ENSG00000075043.18	0	0
protein_coding	H2BFM	ENSG00000101812.12	0	0
protein_coding	TIGD3	ENSG00000173825.6	0	0
protein_coding	TLE6	ENSG00000104953.19	0	0
protein_coding	CDK11A	ENSG00000008128.22	0	0
protein_coding	LEAP2	ENSG00000164406.7	0	0
protein_coding	MYH3	ENSG00000109063.14	0	0
protein_coding	HIST2H2BF	ENSG00000203814.6	0	0
protein_coding	ALDH8A1	ENSG00000118514.13	0	0
protein_coding	KLHL30	ENSG00000168427.8	0	0
protein_coding	ARHGAP8	ENSG00000241484.9	0	0
protein_coding	PADI2	ENSG00000117115.12	0	0
protein_coding	IL36G	ENSG00000136688.10	0	0
protein_coding	CHN2	ENSG00000106069.22	0	0
protein_coding	DCLK1	ENSG00000133083.14	0	0
protein_coding	SIRPB2	ENSG00000196209.12	0	0
protein_coding	IL1RL1	ENSG00000115602.16	0	0
protein_coding	IL17RB	ENSG00000056736.9	0	0
protein_coding	SORBS1	ENSG00000095637.22	0	0
protein_coding	OR51B5	ENSG00000167355.8	0	0
protein_coding	HSD17B2	ENSG00000086696.10	0	0
protein_coding	KIF5C	ENSG00000168280.16	0	0
protein_coding	CCDC192	ENSG00000230561.4	0	0
protein_coding	C5orf46	ENSG00000178776.4	0	0
protein_coding	HIST1H2AJ	ENSG00000276368.1	0	0
protein_coding	OR2A7	ENSG00000243896.4	0	0
protein_coding	TCF24	ENSG00000261787.1	0	0
protein_coding	SORCS1	ENSG00000108018.15	0	0
protein_coding	MGP	ENSG00000111341.9	0	0
protein_coding	PLCB2	ENSG00000137841.11	0	0
protein_coding	ZGPAT	ENSG00000197114.11	0	0
protein_coding	CLDN17	ENSG00000156282.4	0	0
protein_coding	VGLL1	ENSG00000102243.12	0	0
protein_coding	ZFP92	ENSG00000189420.8	0	0
protein_coding	EFR3B	ENSG00000084710.13	0	0
protein_coding	CRYBA2	ENSG00000163499.11	0	0
protein_coding	C6orf118	ENSG00000112539.14	0	0
protein_coding	EMX2	ENSG00000170370.11	0	0
protein_coding	CCL20	ENSG00000115009.12	0	0
protein_coding	SIDT1	ENSG00000072858.10	0	0
protein_coding	AC007040.2	ENSG00000258881.6	0	0
protein_coding	CDH23	ENSG00000107736.20	0	0
protein_coding	NT5M	ENSG00000205309.13	0	0

protein_coding	TNFRSF13C	ENSG00000159958.6	0	0
protein_coding	ZNF564	ENSG00000249709.7	0	0
protein_coding	C3orf80	ENSG00000180044.5	0	0
protein_coding	CFAP54	ENSG00000188596.10	0	0
protein_coding	AP003108.2	ENSG00000256591.5	0	0
protein_coding	SIM2	ENSG00000159263.15	0	0
protein_coding	GIMAP4	ENSG00000133574.9	0	0
protein_coding	PF4	ENSG00000163737.3	0	0
protein_coding	PLPPR5	ENSG00000117598.11	0	0
protein_coding	IVL	ENSG00000163207.6	0	0
protein_coding	HIST1H1B	ENSG00000184357.4	0	0
protein_coding	HSD11B2	ENSG00000176387.6	0	0
protein_coding	SLC22A18AS	ENSG00000254827.5	0	0
protein_coding	IL36RN	ENSG00000136695.14	0	0
protein_coding	FHAD1	ENSG00000142621.19	0	0
protein_coding	HCAR3	ENSG00000255398.2	0	0
protein_coding	GOLGA7B	ENSG00000155265.10	0	0
protein_coding	PCSK6	ENSG00000140479.16	0	0
protein_coding	ZNF257	ENSG00000197134.11	0	0
protein_coding	ZNF296	ENSG00000170684.8	0	0
protein_coding	TRIM9	ENSG00000100505.13	0	0
protein_coding	SPDEF	ENSG00000124664.10	0	0
protein_coding	CYP24A1	ENSG00000019186.9	0	0
protein_coding	EEF1AKMT4	ENSG00000284753.1	0	0
protein_coding	SDR16C5	ENSG00000170786.12	0	0
protein_coding	E2F2	ENSG00000007968.6	0	0
3prime_overlapping_ncRNA	HYI-AS1	ENSG00000229348.1	0	0
3prime_overlapping_ncRNA	AC096677.2	ENSG00000224818.1	0	0
3prime_overlapping_ncRNA	TGFB2-OT1	ENSG00000281453.1	0	0
3prime_overlapping_ncRNA	AL160004.1	ENSG00000226920.1	0	0
3prime_overlapping_ncRNA	AC108025.2	ENSG00000242540.2	0	0
3prime_overlapping_ncRNA	AC010733.2	ENSG00000267520.2	0	0
3prime_overlapping_ncRNA	AC012442.2	ENSG00000243389.1	0	0
3prime_overlapping_ncRNA	AC092620.2	ENSG00000241772.2	0	0

3prime_overlapping_ncRNA	AC009487.2	ENSG00000251621.1	0	0
3prime_overlapping_ncRNA	AC007283.1	ENSG00000234431.2	0	0
3prime_overlapping_ncRNA	AC064852.1	ENSG00000241409.1	0	0
3prime_overlapping_ncRNA	AC093010.3	ENSG00000259976.3	0	0
3prime_overlapping_ncRNA	PP7080	ENSG00000188242.4	0	0
3prime_overlapping_ncRNA	AL662884.1	ENSG00000273333.2	0	0
3prime_overlapping_ncRNA	KHDRBS2-OT	ENSG00000250686.2	0	0
3prime_overlapping_ncRNA	AC234917.1	ENSG00000255343.1	0	0
3prime_overlapping_ncRNA	AL355140.1	ENSG00000243888.1	0	0
3prime_overlapping_ncRNA	AC132217.1	ENSG00000240801.1	0	0
3prime_overlapping_ncRNA	AC055720.2	ENSG00000256185.1	0	0
3prime_overlapping_ncRNA	AC024940.5	ENSG00000275097.1	0	0
3prime_overlapping_ncRNA	AC087190.3	ENSG00000263244.2	0	0
3prime_overlapping_ncRNA	AC127459.1	ENSG00000260566.2	0	0
3prime_overlapping_ncRNA	AC008870.1	ENSG00000260482.3	0	0
3prime_overlapping_ncRNA	AC026464.2	ENSG00000260108.1	0	0
3prime_overlapping_ncRNA	AC012321.1	ENSG00000260772.1	0	0
3prime_overlapping_ncRNA	AC008738.7	ENSG00000273420.1	0	0
3prime_overlapping_ncRNA	LINC00846	ENSG00000186842.4	0	0
3prime_overlapping_ncRNA	AL023653.1	ENSG00000240143.1	0	0
antisense	AL627309.2	ENSG00000239906.1	0	0
antisense	AL669831.2	ENSG00000229905.1	0	0
antisense	AL645608.1	ENSG00000224969.1	0	0

antisense	AL390719.3	ENSG00000285812.1	0	0
antisense	TTLL10-AS1	ENSG00000205231.1	0	0
antisense	AL391244.2	ENSG00000225905.1	0	0
antisense	FO704657.1	ENSG00000272004.1	0	0
antisense	AL391845.2	ENSG00000233542.1	0	0
antisense	AL391845.1	ENSG00000226969.1	0	0
antisense	AL590822.2	ENSG00000271806.1	0	0
antisense	AL139246.1	ENSG00000224387.1	0	0
antisense	AL139246.4	ENSG00000229393.1	0	0
antisense	AL831784.1	ENSG00000237058.1	0	0
antisense	AC242022.1	ENSG00000283259.1	0	0
antisense	LINC00982	ENSG00000177133.10	0	1
antisense	AL008733.1	ENSG00000226286.1	0	0
antisense	AL513320.1	ENSG00000238260.1	0	0
antisense	AL136528.1	ENSG00000227589.1	0	0
antisense	AL136528.2	ENSG00000235131.1	0	0
antisense	AL365330.1	ENSG00000272153.1	0	0
antisense	AL031848.2	ENSG00000271746.1	0	0
antisense	AL031848.1	ENSG00000231868.1	0	0
antisense	AL512330.1	ENSG00000237365.1	0	0
antisense	Z97987.1	ENSG00000237728.2	0	0
antisense	AL365194.1	ENSG00000225126.1	0	0
antisense	Z98884.1	ENSG00000236266.1	0	0
antisense	AL034417.4	ENSG00000284747.1	0	0
antisense	AL096855.1	ENSG00000232912.5	0	0
antisense	PIK3CD-AS1	ENSG00000179840.5	0	0
antisense	AL357140.5	ENSG00000285701.1	0	0
antisense	AL139424.1	ENSG00000271989.1	0	0
antisense	AL354956.1	ENSG00000203469.2	0	0
antisense	AL139423.1	ENSG00000272078.1	0	0
antisense	AL109811.3	ENSG00000271895.2	0	0
antisense	MTOR-AS1	ENSG00000225602.5	0	0
antisense	AL590989.1	ENSG00000285833.1	0	0
antisense	AL031731.1	ENSG00000284708.1	0	0
antisense	AL953897.1	ENSG00000177553.6	0	0
antisense	AL357835.1	ENSG00000285604.1	0	0
antisense	AL354712.1	ENSG00000259961.1	0	0
antisense	TMEM51-	ENSG00000175147.12	0	0
antisense	AL031283.2	ENSG00000233485.1	0	0
antisense	AL031283.3	ENSG00000236045.1	0	0
antisense	AL121992.3	ENSG00000272510.1	0	0
antisense	AL450998.2	ENSG00000237938.5	0	0
antisense	SLC25A34-	ENSG00000224459.1	0	0
antisense	AL355994.3	ENSG00000233078.1	0	0

antisense	AL451042.2	ENSG00000227959.1	0	0
antisense	AL451042.1	ENSG00000224621.1	0	0
antisense	ARHGEF19-	ENSG00000234166.1	0	0
antisense	AL049569.1	ENSG00000226526.1	0	0
antisense	AC004824.1	ENSG00000227751.1	0	0
antisense	IGSF21-AS1	ENSG00000230035.2	0	0
antisense	AL391883.1	ENSG00000235434.1	0	0
antisense	UBXN10-AS1	ENSG00000225986.1	0	0
antisense	AL020998.1	ENSG00000226664.1	0	0
antisense	AL663074.1	ENSG00000233069.1	0	0
antisense	AL031005.1	ENSG00000236936.1	0	0
antisense	AL031728.1	ENSG00000231105.1	0	0
antisense	LINC02596	ENSG00000233431.1	0	0
antisense	AL512444.1	ENSG00000225952.1	0	0
antisense	AL031428.1	ENSG00000240553.1	0	0
antisense	AL590609.3	ENSG00000232557.3	0	0
antisense	AL591178.1	ENSG00000225315.2	0	0
antisense	AL591178.2	ENSG00000230703.1	0	0
antisense	AL138902.1	ENSG00000232298.2	0	0
antisense	AL445471.1	ENSG00000229162.1	0	0
antisense	AL031280.1	ENSG00000233478.1	0	0
antisense	AL020996.3	ENSG00000272478.1	0	0
antisense	AL033528.2	ENSG00000236528.1	0	0
antisense	AL513365.2	ENSG00000225891.1	0	0
antisense	AL034380.1	ENSG00000226698.1	0	0
antisense	AL590640.1	ENSG00000224311.1	0	0
antisense	AL445490.1	ENSG00000225886.3	0	0
antisense	AL512288.1	ENSG00000227050.1	0	0
antisense	AL009181.1	ENSG00000233427.1	0	0
antisense	AL590729.1	ENSG00000237934.1	0	0
antisense	AL445235.1	ENSG00000235143.1	0	0
antisense	AL451070.1	ENSG00000229044.1	0	0
antisense	AC114488.1	ENSG00000229167.1	0	0
antisense	AC114488.2	ENSG00000235790.7	0	0
antisense	AC114488.3	ENSG00000264078.1	0	1
antisense	AL354919.2	ENSG00000254545.1	0	0
antisense	AL445248.1	ENSG00000203325.3	0	0
antisense	AL020995.1	ENSG00000236065.2	0	0
antisense	AL513327.1	ENSG00000225313.5	0	0
antisense	AL513327.2	ENSG00000233246.1	0	0
antisense	CSMD2-AS1	ENSG00000231163.5	0	0
antisense	AL121988.1	ENSG00000255811.1	0	0
antisense	AL122010.1	ENSG00000230163.1	0	0
antisense	ZMYM4-AS1	ENSG00000227409.1	0	0

antisense	AL138787.2	ENSG00000271554.1	0	0
antisense	AC117945.1	ENSG00000234481.1	0	0
antisense	AL929472.3	ENSG00000233728.1	0	0
antisense	AL929472.2	ENSG00000230955.1	0	0
antisense	AL442071.1	ENSG00000226438.1	0	0
antisense	AL365277.1	ENSG00000228060.2	0	0
antisense	AL035404.2	ENSG00000225903.1	0	0
antisense	AL033527.3	ENSG00000261798.1	0	0
antisense	AL033527.2	ENSG00000236546.1	0	0
antisense	AL603839.1	ENSG00000227278.1	0	0
antisense	AL603839.2	ENSG00000238186.1	0	0
antisense	NFYC-AS1	ENSG00000272145.1	0	0
antisense	AC119677.1	ENSG00000229528.1	0	0
antisense	SLFNL1-AS1	ENSG00000281207.1	0	0
antisense	AC093151.2	ENSG00000229901.1	0	1
antisense	AL445933.2	ENSG00000230881.1	0	0
antisense	AC098484.4	ENSG00000285728.1	0	0
antisense	AC098484.1	ENSG00000228452.1	0	0
antisense	AL512353.1	ENSG00000228192.7	0	1
antisense	AL139289.1	ENSG00000229431.1	0	0
antisense	SZT2-AS1	ENSG00000229372.1	0	0
antisense	AL451062.1	ENSG00000229444.1	0	1
antisense	AL357079.2	ENSG00000285649.1	0	0
antisense	AL358075.1	ENSG00000226957.1	0	0
antisense	MKNK1-AS1	ENSG00000269956.1	0	0
antisense	EFCAB14-	ENSG00000228237.5	0	0
antisense	AL135960.1	ENSG00000226252.1	0	0
antisense	AL109659.2	ENSG00000272491.1	0	0
antisense	AC099788.1	ENSG00000229846.2	0	0
antisense	AL590432.1	ENSG00000230114.1	0	0
antisense	AL592182.2	ENSG00000237337.1	0	0
antisense	AL592182.1	ENSG00000233407.1	0	0
antisense	AL049637.1	ENSG00000225767.1	0	0
antisense	AL162430.2	ENSG00000236434.2	0	0
antisense	TTC39A-AS1	ENSG00000261664.5	0	0
antisense	AC104170.2	ENSG00000238140.1	0	0
antisense	AC104170.1	ENSG00000227070.1	0	0
antisense	AL050343.1	ENSG00000266993.3	0	1
antisense	AL445685.1	ENSG00000223390.1	0	0
antisense	TXNDC12-	ENSG00000228369.2	0	0
antisense	AL513218.1	ENSG00000272100.1	0	0
antisense	AC099677.4	ENSG00000242391.2	0	0
antisense	AL445183.1	ENSG00000232993.1	0	0
antisense	AL445183.2	ENSG00000235563.1	0	0

antisense	AL606760.2	ENSG00000236723.2	0	0
antisense	AL355483.4	ENSG00000234578.1	0	0
antisense	AL355483.2	ENSG00000228838.1	0	0
antisense	AL355483.3	ENSG00000232762.1	0	0
antisense	AL357673.2	ENSG00000280425.2	0	0
antisense	SSBP3-AS1	ENSG00000198711.5	0	0
antisense	AL161644.1	ENSG00000225632.1	0	0
antisense	AC096536.1	ENSG00000237453.1	0	0
antisense	AC096536.2	ENSG00000242396.1	0	0
antisense	AL590440.1	ENSG00000233271.1	0	0
antisense	AL360295.1	ENSG00000229913.1	0	0
antisense	AL161740.1	ENSG00000236341.1	0	0
antisense	DAB1-AS1	ENSG00000226759.7	0	0
antisense	AL035416.1	ENSG00000226883.1	0	0
antisense	NFIA-AS2	ENSG00000237928.5	0	0
antisense	ROR1-AS1	ENSG00000223949.6	0	0
antisense	AL139294.1	ENSG00000229294.1	0	0
antisense	AL590783.1	ENSG00000227466.1	0	0
antisense	AL592161.1	ENSG00000231080.1	0	0
antisense	GNG12-AS1	ENSG00000232284.7	0	0
antisense	DEPDC1-AS1	ENSG00000234264.1	0	0
antisense	AL158840.1	ENSG00000237919.5	0	0
antisense	AL353771.1	ENSG00000228988.1	0	0
antisense	AL158839.1	ENSG00000226088.1	0	0
antisense	AL031429.1	ENSG00000235782.1	0	0
antisense	ZRANB2-	ENSG00000235079.1	0	0
antisense	AL354949.1	ENSG00000231985.1	0	0
antisense	AC093158.1	ENSG00000237324.3	0	0
antisense	AC105271.1	ENSG00000233894.1	0	0
antisense	ERICH3-AS1	ENSG00000234497.5	0	0
antisense	AL035409.1	ENSG00000230498.1	0	0
antisense	AC095030.1	ENSG00000233099.1	0	0
antisense	AL138799.4	ENSG00000234953.2	0	0
antisense	AC117944.1	ENSG00000236676.1	0	0
antisense	AL356270.1	ENSG00000236915.2	0	0
antisense	AL121989.1	ENSG00000267734.1	0	0
antisense	AC099063.4	ENSG00000284734.1	0	0
antisense	AC099063.1	ENSG00000237568.1	0	0
antisense	AL109613.1	ENSG00000224093.5	0	0
antisense	AC093579.1	ENSG00000233482.1	0	0
antisense	AC095033.1	ENSG00000230427.1	0	0
antisense	AC092802.2	ENSG00000228852.6	0	0
antisense	DPYD-AS1	ENSG00000232878.3	0	0
antisense	DPYD-AS2	ENSG00000235777.1	0	0

antisense	AL445433.2	ENSG00000232825.2	0	0
antisense	AC093019.2	ENSG00000241073.1	0	0
antisense	AL445928.2	ENSG00000285530.1	0	0
antisense	AL589990.1	ENSG00000228086.1	0	0
antisense	VAV3-AS1	ENSG00000230489.1	0	0
antisense	AL359258.2	ENSG00000260879.1	0	0
antisense	AL591719.2	ENSG00000232971.2	0	0
antisense	AL355310.3	ENSG00000254942.1	0	0
antisense	AL355310.2	ENSG00000228703.1	0	0
antisense	AC000032.1	ENSG00000260246.1	0	0
antisense	AL158847.1	ENSG00000241720.2	0	0
antisense	AL450468.1	ENSG00000235005.1	0	0
antisense	LINC01397	ENSG00000258673.1	0	0
antisense	AL355990.1	ENSG00000227091.1	0	0
antisense	AL355990.2	ENSG00000235526.1	0	0
antisense	LINC02586	ENSG00000224965.1	0	0
antisense	RBM15-AS1	ENSG00000227963.1	0	0
antisense	AL358215.1	ENSG00000270380.1	0	0
antisense	AL360270.1	ENSG00000232811.1	0	0
antisense	AL355816.1	ENSG00000272982.1	0	0
antisense	AL356387.1	ENSG00000229283.1	0	0
antisense	KCND3-AS1	ENSG00000237556.1	0	0
antisense	AL603832.1	ENSG00000225075.1	0	0
antisense	AL603832.2	ENSG00000261595.1	0	0
antisense	AL137856.1	ENSG00000231128.5	0	0
antisense	AP4B1-AS1	ENSG00000226167.1	0	0
antisense	HIPK1-AS1	ENSG00000235527.6	0	0
antisense	NGF-AS1	ENSG00000228035.1	0	0
antisense	AL365318.1	ENSG00000237993.1	0	0
antisense	AL445231.1	ENSG00000236137.1	0	0
antisense	AL157902.1	ENSG00000236866.5	0	0
antisense	WARS2-IT1	ENSG00000224238.2	0	0
antisense	AC244453.3	ENSG00000234998.1	0	0
antisense	AC244453.2	ENSG00000233029.3	0	1
antisense	SRGAP2-AS1	ENSG00000230806.1	0	0
antisense	AC246680.1	ENSG00000230186.3	0	0
antisense	AC243547.1	ENSG00000244619.2	0	1
antisense	AC243547.2	ENSG00000278431.1	0	0
antisense	AC242426.2	ENSG00000237188.4	0	0
antisense	LINC00624	ENSG00000278811.4	0	0
antisense	AC241644.3	ENSG00000274415.1	0	0
antisense	AC239802.1	ENSG00000254913.1	0	0
antisense	AC239802.2	ENSG00000255148.2	0	0
antisense	AC239868.1	ENSG00000264207.1	0	0

antisense	AC242988.1	ENSG00000276110.1	0	0
antisense	AL356356.1	ENSG00000237781.3	0	0
antisense	AL590133.2	ENSG00000259357.2	0	0
antisense	AL391069.1	ENSG00000224645.1	0	0
antisense	AL391335.1	ENSG00000250734.2	0	0
antisense	AL589765.1	ENSG00000227045.1	0	0
antisense	AL589765.7	ENSG00000269621.1	0	0
antisense	AL589765.4	ENSG00000249602.1	0	0
antisense	AL589765.5	ENSG00000268288.1	0	0
antisense	TDRKH-AS1	ENSG00000203288.3	0	0
antisense	AL450992.2	ENSG00000234614.1	0	0
antisense	AL450992.3	ENSG00000285651.1	0	0
antisense	AL450992.1	ENSG00000229021.2	0	0
antisense	AL589986.1	ENSG00000226716.1	0	0
antisense	LINC01527	ENSG00000224308.1	0	0
antisense	BX470102.1	ENSG00000238279.1	0	0
antisense	AL513523.4	ENSG00000243613.1	0	0
antisense	AL513523.3	ENSG00000233222.2	0	0
antisense	AL358472.5	ENSG00000284738.1	0	0
antisense	AL358472.3	ENSG00000273026.1	0	0
antisense	IL6R-AS1	ENSG00000228013.1	0	0
antisense	AL162591.2	ENSG00000273110.1	0	2
antisense	UBE2Q1-AS1	ENSG00000229780.1	0	0
antisense	AL592078.1	ENSG00000233875.1	0	0
antisense	AC234582.2	ENSG00000236263.1	0	0
antisense	AL353807.2	ENSG00000232519.2	0	2
antisense	AL162734.1	ENSG00000227673.1	0	0
antisense	AL355388.2	ENSG00000273002.1	0	0
antisense	AL355388.1	ENSG00000224276.1	0	0
antisense	AL355388.3	ENSG00000285677.1	0	0
antisense	AL590666.2	ENSG00000229953.1	0	0
antisense	AL590666.4	ENSG00000285570.1	0	0
antisense	AL590666.1	ENSG00000223356.1	0	0
antisense	AL356276.1	ENSG00000227217.1	0	0
antisense	AL365440.2	ENSG00000236656.1	0	0
antisense	CADM3-AS1	ENSG00000225670.4	0	0
antisense	AL513323.1	ENSG00000228560.1	0	0
antisense	AL121987.1	ENSG00000225279.1	0	0
antisense	AL121987.2	ENSG00000227741.1	0	2
antisense	AL138930.1	ENSG00000234425.1	0	0
antisense	AL354714.1	ENSG00000198358.4	0	0
antisense	AL590385.1	ENSG00000224515.1	0	0
antisense	AL451067.1	ENSG00000234211.2	0	0
antisense	AL359541.1	ENSG00000226889.3	0	0

antisense	AL450163.1	ENSG00000285636.1	0	0
antisense	AL512785.1	ENSG00000227094.1	0	0
antisense	AL596325.1	ENSG00000259788.1	0	0
antisense	AL499616.1	ENSG00000232892.1	0	0
antisense	AL357568.1	ENSG00000233693.1	0	0
antisense	AL390730.1	ENSG00000224702.1	0	0
antisense	AL390730.2	ENSG00000238022.1	0	0
antisense	AL157714.2	ENSG00000237463.5	0	0
antisense	AL358115.1	ENSG00000236364.3	0	0
antisense	AL626787.1	ENSG00000203307.2	0	0
antisense	AL158837.1	ENSG00000227907.1	0	0
antisense	LINC01363	ENSG00000231605.5	0	0
antisense	AL031733.2	ENSG00000241666.2	0	0
antisense	Z99943.1	ENSG00000232194.1	0	0
antisense	AL031726.1	ENSG00000237707.1	0	0
antisense	Z99758.1	ENSG00000235575.1	0	0
antisense	AL356475.1	ENSG00000232959.1	0	0
antisense	AL021026.1	ENSG00000225243.5	0	0
antisense	AL445673.1	ENSG00000231424.2	0	0
antisense	AL139142.1	ENSG00000238272.1	0	0
antisense	Z99127.1	ENSG00000229531.5	0	1
antisense	Z94057.1	ENSG00000260990.1	0	0
antisense	AL590723.1	ENSG00000228686.2	0	0
antisense	AL359265.3	ENSG00000236021.1	0	0
antisense	AL160286.3	ENSG00000261250.1	0	0
antisense	AL359853.1	ENSG00000229407.5	0	0
antisense	LAMC1-AS1	ENSG00000224468.3	0	0
antisense	AL590422.1	ENSG00000227554.1	0	0
antisense	AL133553.1	ENSG00000224691.1	0	0
antisense	AL596220.1	ENSG00000229739.2	0	0
antisense	AL354771.1	ENSG00000225811.1	0	0
antisense	AL136322.1	ENSG00000230260.1	0	0
antisense	AL365258.1	ENSG00000224901.1	0	0
antisense	AL157402.2	ENSG00000261573.1	0	0
antisense	AL358473.1	ENSG00000229191.1	0	0
antisense	AL358473.2	ENSG00000234132.2	0	0
antisense	AC103925.1	ENSG00000229821.1	0	0
antisense	AC119427.1	ENSG00000282221.1	0	0
antisense	AC092800.1	ENSG00000236390.1	0	0
antisense	AC104463.2	ENSG00000226862.1	0	0
antisense	AC098934.4	ENSG00000260021.1	0	0
antisense	AC105940.2	ENSG00000234775.1	0	0
antisense	AC105940.1	ENSG00000224671.2	0	0
antisense	AL592146.1	ENSG00000261065.1	0	0

antisense	ERLNC1	ENSG00000230550.1	0	0
antisense	AL592114.3	ENSG00000231691.1	0	0
antisense	AL606489.1	ENSG00000226330.1	0	0
antisense	AL512306.3	ENSG00000240710.1	0	0
antisense	AC093422.2	ENSG00000225063.1	0	0
antisense	LEMD1-AS1	ENSG00000226235.1	0	0
antisense	AL713965.1	ENSG00000227687.1	0	0
antisense	AC244035.1	ENSG00000226780.1	0	0
antisense	C1orf147	ENSG00000162888.4	0	0
antisense	AL137789.1	ENSG00000236911.6	0	0
antisense	AL137789.2	ENSG00000285239.1	0	0
antisense	AL356275.2	ENSG00000285719.1	0	0
antisense	AL031316.1	ENSG00000227591.5	0	0
antisense	AL445488.1	ENSG00000229258.5	0	0
antisense	AL360091.3	ENSG00000234915.1	0	0
antisense	AL590648.2	ENSG00000235862.2	0	0
antisense	AL606537.1	ENSG00000272167.2	0	0
antisense	AL358452.1	ENSG00000229242.1	0	0
antisense	AC093581.1	ENSG00000233620.5	0	0
antisense	AC138024.1	ENSG00000236292.1	0	0
antisense	SPATA17-	ENSG00000234070.1	0	0
antisense	TGFB2-AS1	ENSG00000232480.1	0	1
antisense	LYPLAL1-DT	ENSG00000228063.1	0	0
antisense	HLX-AS1	ENSG00000257551.1	0	0
antisense	AL596330.1	ENSG00000229400.1	0	0
antisense	AL391811.1	ENSG00000225334.1	0	2
antisense	AC099066.2	ENSG00000227496.1	0	1
antisense	AL512343.2	ENSG00000272562.1	0	0
antisense	ACBD3-AS1	ENSG00000234478.1	0	0
antisense	ITPKB-AS1	ENSG00000228548.1	0	0
antisense	AL353689.2	ENSG00000233706.1	0	0
antisense	AL451047.1	ENSG00000228625.1	0	0
antisense	SNAP47-AS1	ENSG00000230005.2	0	0
antisense	AL353593.2	ENSG00000269934.1	0	0
antisense	AL353593.3	ENSG00000270110.1	0	0
antisense	AL670729.2	ENSG00000270094.1	0	0
antisense	AL670729.1	ENSG00000231563.1	0	0
antisense	AL162595.2	ENSG00000233920.1	0	0
antisense	AL117350.1	ENSG00000237481.1	0	0
antisense	AL121990.1	ENSG00000223635.1	0	0
antisense	AL136988.2	ENSG00000227006.1	0	0
antisense	AL136988.1	ENSG00000224407.1	0	0
antisense	AL512328.1	ENSG00000244137.1	0	0
antisense	AL118511.1	ENSG00000223393.1	0	2

antisense	LINC00582	ENSG00000229228.1	0	0
antisense	AL122008.3	ENSG00000233332.1	0	0
antisense	AL122008.4	ENSG00000236244.1	0	0
antisense	AL355472.3	ENSG00000236358.1	0	0
antisense	AL355472.4	ENSG00000273367.1	0	0
antisense	AL160408.2	ENSG00000228830.1	0	0
antisense	AL357556.4	ENSG00000285177.1	0	0
antisense	LYST-AS1	ENSG00000229463.2	0	0
antisense	AL359924.1	ENSG00000237250.3	0	0
antisense	CHRM3-AS2	ENSG00000233355.6	0	0
antisense	CHRM3-AS1	ENSG00000234601.1	0	0
antisense	AL359918.2	ENSG00000233735.5	0	0
antisense	AL590490.1	ENSG00000233519.1	0	0
antisense	AL358176.1	ENSG00000224359.2	0	0
antisense	AL365184.1	ENSG00000226919.2	0	0
antisense	AL591686.2	ENSG00000272865.1	0	0
antisense	AL606534.1	ENSG00000227230.1	0	0
antisense	AL606534.2	ENSG00000232085.1	0	0
antisense	AL591721.1	ENSG00000236031.1	0	0
antisense	AL645465.1	ENSG00000240963.1	0	0
antisense	AL356512.1	ENSG00000272195.1	0	2
antisense	KIF26B-AS1	ENSG00000232192.1	0	0
antisense	AC104462.2	ENSG00000238224.1	0	0
antisense	AC104462.1	ENSG00000231612.1	0	0
antisense	AC118555.1	ENSG00000235096.2	0	0
antisense	AC092801.1	ENSG00000226876.2	0	0
antisense	AL606804.1	ENSG00000236817.5	0	0
antisense	AL390860.1	ENSG00000235749.2	0	0
antisense	AC098483.1	ENSG00000224521.1	0	0
antisense	AL672291.1	ENSG00000227237.1	0	0
antisense	AC079779.1	ENSG00000227061.1	0	0
antisense	AC092159.2	ENSG00000233296.1	0	0
antisense	AC092159.3	ENSG00000233970.1	0	0
antisense	AC113607.1	ENSG00000234796.1	0	0
antisense	AC116614.1	ENSG00000235688.2	0	0
antisense	AC114808.1	ENSG00000235403.1	0	0
antisense	AC114808.2	ENSG00000236665.1	0	0
antisense	AC108462.1	ENSG00000233553.1	0	0
antisense	AC141930.1	ENSG00000228613.1	0	0
antisense	AC093390.2	ENSG00000284600.1	0	0
antisense	AC093390.1	ENSG00000232057.1	0	0
antisense	MYT1L-AS1	ENSG00000225619.1	0	0
antisense	TRAPPC12-	ENSG00000225234.1	0	0
antisense	AC010907.2	ENSG00000237370.1	0	0

antisense	AC010907.1	ENSG00000224661.1	0	0
antisense	LINC01248	ENSG00000224128.1	0	0
antisense	NRIR	ENSG00000225964.5	0	0
antisense	AC068481.1	ENSG00000223884.6	0	0
antisense	AC080162.1	ENSG00000239300.5	0	0
antisense	AC010969.1	ENSG00000188525.3	0	0
antisense	AC104794.4	ENSG00000271787.1	0	0
antisense	AC007314.1	ENSG00000234818.1	0	0
antisense	AC012456.1	ENSG00000203643.3	0	0
antisense	AC012456.2	ENSG00000230790.2	0	0
antisense	AC008278.2	ENSG00000234022.1	0	0
antisense	AC008278.1	ENSG00000224194.1	0	0
antisense	MYCNOS	ENSG00000233718.7	0	1
antisense	AC104623.1	ENSG00000237633.3	0	0
antisense	AC079145.1	ENSG00000227210.1	0	0
antisense	AC011239.1	ENSG00000224361.1	0	0
antisense	AC008073.2	ENSG00000232642.1	0	0
antisense	AC008073.1	ENSG00000223754.1	0	0
antisense	DNAJC27-	ENSG00000224165.5	0	2
antisense	LINC01381	ENSG00000230452.1	0	0
antisense	AC104699.1	ENSG00000224220.1	0	0
antisense	AC015977.2	ENSG00000225378.1	0	0
antisense	AC013472.2	ENSG00000230286.1	0	0
antisense	AGBL5-AS1	ENSG00000231636.1	0	0
antisense	AC013472.3	ENSG00000272056.1	0	0
antisense	GTF3C2-AS1	ENSG00000234945.7	0	1
antisense	AC093690.1	ENSG00000223522.1	0	0
antisense	AC074011.1	ENSG00000230730.1	0	0
antisense	AC092164.1	ENSG00000226833.5	0	1
antisense	AC105398.1	ENSG00000229224.1	0	0
antisense	AC106870.2	ENSG00000230737.1	0	0
antisense	AC106870.1	ENSG00000197644.2	0	0
antisense	AC016907.2	ENSG00000233862.5	0	0
antisense	AC009305.1	ENSG00000234579.1	0	0
antisense	AL133247.1	ENSG00000228563.1	0	0
antisense	BIRC6-AS1	ENSG00000230046.1	0	0
antisense	AC019127.1	ENSG00000285577.1	0	0
antisense	AC020594.1	ENSG00000237133.1	0	0
antisense	AC007378.1	ENSG00000273090.1	0	0
antisense	AC007391.2	ENSG00000285925.1	0	0
antisense	AC006369.1	ENSG00000236213.1	0	0
antisense	RMDN2-AS1	ENSG00000235848.4	0	0
antisense	AC011247.2	ENSG00000235586.1	0	0
antisense	AC074366.1	ENSG00000232518.3	0	0

antisense	AC018693.1	ENSG00000225284.1	0	0
antisense	AC013480.1	ENSG00000226398.1	0	0
antisense	AC083949.1	ENSG00000224875.2	0	0
antisense	AC010883.1	ENSG00000234936.1	0	0
antisense	AC019129.2	ENSG00000273106.1	0	0
antisense	U51244.1	ENSG00000228481.1	0	0
antisense	AC017006.2	ENSG00000232696.1	0	0
antisense	AC017006.1	ENSG00000231336.1	0	0
antisense	AC018682.2	ENSG00000253515.1	0	0
antisense	AC018682.1	ENSG00000250116.2	0	0
antisense	AC016722.2	ENSG00000228925.1	0	0
antisense	AC093732.2	ENSG00000272814.1	0	0
antisense	AC093732.1	ENSG00000233845.1	0	0
antisense	AC073283.1	ENSG00000225187.1	0	0
antisense	HCG2040054	ENSG00000235760.4	0	0
antisense	AC068725.1	ENSG00000285548.1	0	0
antisense	AC009234.1	ENSG00000283058.1	0	0
antisense	AC092839.1	ENSG00000228108.1	0	0
antisense	AC092839.2	ENSG00000234943.2	0	0
antisense	AC093110.1	ENSG00000238018.2	0	0
antisense	AC104781.1	ENSG00000231334.1	0	0
antisense	AC104781.2	ENSG00000285519.1	0	0
antisense	AC012358.1	ENSG00000203327.2	0	0
antisense	AC007250.1	ENSG00000273063.1	0	0
antisense	AC009970.1	ENSG00000233953.1	0	0
antisense	AC016727.3	ENSG00000285857.1	0	0
antisense	AC107081.2	ENSG00000236498.1	0	0
antisense	AC018462.1	ENSG00000229839.6	0	0
antisense	AC092155.1	ENSG00000226622.5	0	0
antisense	AC092567.1	ENSG00000226605.1	0	0
antisense	AC007098.1	ENSG00000231609.6	0	0
antisense	LINC02576	ENSG00000232613.6	0	0
antisense	MEIS1-AS3	ENSG00000226819.1	0	0
antisense	MEIS1-AS2	ENSG00000230749.4	0	0
antisense	AC017083.1	ENSG00000273064.1	0	0
antisense	AC015969.1	ENSG00000203395.2	0	0
antisense	LINC01888	ENSG00000237576.1	0	0
antisense	AC022201.2	ENSG00000233849.1	0	0
antisense	AC022201.1	ENSG00000229229.1	0	0
antisense	AC005234.1	ENSG00000235035.1	0	0
antisense	ATP6V1B1-	ENSG00000239322.1	0	0
antisense	AC007040.1	ENSG00000228384.4	0	0
antisense	AC007881.2	ENSG00000236469.1	0	0
antisense	AC012366.1	ENSG00000278060.1	0	0

antisense	DCTN1-AS1	ENSG00000237737.5	0	0
antisense	AC005041.3	ENSG00000272183.1	0	0
antisense	AC007681.1	ENSG00000270571.2	0	0
antisense	AC007099.2	ENSG00000237293.1	0	0
antisense	AC007099.1	ENSG00000231172.2	0	0
antisense	AC005034.2	ENSG00000270462.1	0	0
antisense	AC079117.1	ENSG00000234653.1	0	0
antisense	AC010975.1	ENSG00000229385.1	0	0
antisense	AC016716.2	ENSG00000224731.1	0	0
antisense	AC008067.1	ENSG00000237031.7	0	0
antisense	AC062037.2	ENSG00000273196.1	0	0
antisense	AC105053.1	ENSG00000229498.1	0	0
antisense	AC015971.1	ENSG00000228363.2	0	0
antisense	AC104134.1	ENSG00000225420.1	0	0
antisense	AC103563.7	ENSG00000233850.1	0	0
antisense	AC103563.2	ENSG00000231062.1	0	0
antisense	AC021188.1	ENSG00000230747.1	0	0
antisense	AC013270.1	ENSG00000235480.1	0	0
antisense	AC092675.1	ENSG00000222000.7	0	0
antisense	AC092667.1	ENSG00000230393.1	0	0
antisense	AC016738.2	ENSG00000230140.5	0	0
antisense	AC016738.1	ENSG00000223947.1	0	0
antisense	IL1R1-AS1	ENSG00000226925.1	0	0
antisense	AC018730.1	ENSG00000239587.1	0	0
antisense	AC010884.1	ENSG00000224509.2	0	0
antisense	UTAT33	ENSG00000231851.5	0	0
antisense	AC108058.1	ENSG00000238273.3	0	0
antisense	AC010978.1	ENSG00000235522.6	0	0
antisense	AC018878.1	ENSG00000233339.1	0	0
antisense	AC016994.1	ENSG00000227294.1	0	0
antisense	LINC01594	ENSG00000225328.1	0	0
antisense	GCC2-AS1	ENSG00000214184.3	0	1
antisense	LIMS1-AS1	ENSG00000228763.1	0	0
antisense	AC112229.2	ENSG00000227574.2	0	0
antisense	AC226101.1	ENSG00000231536.1	0	0
antisense	ACOXL-AS1	ENSG00000204581.2	0	0
antisense	AC016683.1	ENSG00000234174.1	0	0
antisense	AC016745.1	ENSG00000234997.1	0	0
antisense	DPP10-AS3	ENSG00000231538.1	0	0
antisense	DPP10-AS2	ENSG00000235717.1	0	0
antisense	DPP10-AS1	ENSG00000235026.5	0	0
antisense	AC009312.1	ENSG00000238207.1	0	0
antisense	AC009303.2	ENSG00000235066.7	0	0
antisense	AC009303.3	ENSG00000272895.1	0	0

antisense	AC013457.1	ENSG00000259094.1	0	0
antisense	STEAP3-AS1	ENSG00000229867.1	0	1
antisense	AC013275.1	ENSG00000231013.1	0	0
antisense	AC012363.1	ENSG00000224789.1	0	0
antisense	AC012447.1	ENSG00000265451.1	0	0
antisense	AC079154.1	ENSG00000228400.1	0	0
antisense	AC010976.1	ENSG00000231731.7	0	0
antisense	AC010976.2	ENSG00000272789.1	0	0
antisense	AC018865.1	ENSG00000224087.1	0	0
antisense	AC013269.2	ENSG00000225819.1	0	0
antisense	AC013269.1	ENSG00000225341.1	0	0
antisense	AC140481.2	ENSG00000232408.1	0	0
antisense	AC140481.1	ENSG00000229797.1	0	0
antisense	AC133785.1	ENSG00000233221.6	0	0
antisense	AC073869.5	ENSG00000283303.1	0	0
antisense	AC010974.1	ENSG00000230065.1	0	0
antisense	AC016909.2	ENSG00000233729.1	0	0
antisense	AC010890.1	ENSG00000226953.7	0	0
antisense	CCNT2-AS1	ENSG00000224043.7	0	1
antisense	AC011893.1	ENSG00000226806.1	0	0
antisense	AC013437.1	ENSG00000257284.1	0	0
antisense	AC096558.1	ENSG00000228655.6	0	0
antisense	AC096558.2	ENSG00000257640.1	0	0
antisense	AC079793.1	ENSG00000258268.1	0	0
antisense	AC092652.1	ENSG00000231758.2	0	0
antisense	AC092652.2	ENSG00000257277.1	0	0
antisense	AC079584.2	ENSG00000257226.1	0	0
antisense	AC016910.1	ENSG00000232377.1	0	1
antisense	AC009951.2	ENSG00000283118.1	0	0
antisense	AC009480.1	ENSG00000223911.1	0	0
antisense	AC105402.3	ENSG00000231079.7	0	0
antisense	AC023469.2	ENSG00000228064.1	0	0
antisense	AC079790.1	ENSG00000225214.1	0	0
antisense	AC009227.1	ENSG00000224675.1	0	0
antisense	AC061961.1	ENSG00000235949.1	0	0
antisense	CCDC148-	ENSG00000227480.2	0	0
antisense	AC008277.1	ENSG00000223642.7	0	0
antisense	AC009313.1	ENSG00000224467.1	0	0
antisense	AC009299.3	ENSG00000235724.8	0	0
antisense	AC009487.1	ENSG00000224076.5	0	0
antisense	AC008063.1	ENSG00000230918.1	0	0
antisense	AC007750.1	ENSG00000236841.7	0	0
antisense	AC011900.1	ENSG00000237750.2	0	0
antisense	AC019197.1	ENSG00000236283.4	0	0

antisense	AC010127.1	ENSG00000236107.8	0	0
antisense	XIRP2-AS1	ENSG00000254552.1	0	0
antisense	AC016723.1	ENSG00000235335.2	0	0
antisense	AC007556.1	ENSG00000235321.1	0	0
antisense	AC012594.1	ENSG00000231898.8	0	0
antisense	AC007277.1	ENSG00000213981.8	0	0
antisense	AC007405.2	ENSG00000235934.1	0	0
antisense	AC078883.2	ENSG00000226963.1	0	0
antisense	MAP3K20-	ENSG00000238133.6	0	0
antisense	AC010894.4	ENSG00000237798.1	0	0
antisense	AC096649.1	ENSG00000229750.1	0	0
antisense	AC016751.2	ENSG00000235047.1	0	0
antisense	HOXD-AS2	ENSG00000237380.6	0	0
antisense	AC009336.1	ENSG00000272729.1	0	0
antisense	AC079305.3	ENSG00000229337.1	0	0
antisense	AC019080.3	ENSG00000271825.1	0	0
antisense	AC019080.4	ENSG00000271996.1	0	0
antisense	AC073834.1	ENSG00000237655.1	0	0
antisense	AC012499.1	ENSG00000229941.5	0	0
antisense	AC009948.4	ENSG00000270956.1	0	0
antisense	AC009948.3	ENSG00000270277.1	0	0
antisense	AC010680.2	ENSG00000270574.1	0	0
antisense	AC010680.1	ENSG00000267784.1	0	0
antisense	AC104076.1	ENSG00000236153.1	0	0
antisense	AC013733.2	ENSG00000234595.1	0	0
antisense	AC064871.1	ENSG00000224643.5	0	0
antisense	AC007966.1	ENSG00000226747.6	0	0
antisense	FSIP2-AS1	ENSG00000231646.5	0	0
antisense	AC017101.1	ENSG00000227227.1	0	0
antisense	AC007319.1	ENSG00000224063.5	0	1
antisense	AC133106.1	ENSG00000228073.1	0	0
antisense	AC006460.1	ENSG00000228509.5	0	0
antisense	AC005540.1	ENSG00000235852.1	0	0
antisense	AC067945.2	ENSG00000230686.1	0	0
antisense	AC098872.1	ENSG00000225884.2	0	0
antisense	AC098617.1	ENSG00000233766.7	0	0
antisense	AC068544.1	ENSG00000282836.1	0	0
antisense	AC013264.1	ENSG00000231621.1	0	0
antisense	AC010746.1	ENSG00000225979.1	0	0
antisense	AC011997.1	ENSG00000222017.1	0	0
antisense	AC016746.1	ENSG00000257045.1	0	0
antisense	SATB2-AS1	ENSG00000225953.2	0	0
antisense	AC007163.1	ENSG00000230408.3	0	0
antisense	AC005037.1	ENSG00000183308.6	0	2

antisense	CFLAR-AS1	ENSG00000226312.7	0	0
antisense	AC080075.1	ENSG00000256458.1	0	0
antisense	AC007362.1	ENSG00000225216.6	0	0
antisense	AC007679.1	ENSG00000225610.1	0	0
antisense	AC007383.3	ENSG00000231955.1	0	0
antisense	AC010731.2	ENSG00000228577.1	0	0
antisense	AC010731.3	ENSG00000231653.1	0	0
antisense	AC009226.1	ENSG00000223725.6	0	0
antisense	LANCL1-	ENSG00000234281.5	0	0
antisense	AC068051.1	ENSG00000197585.9	0	0
antisense	AC072062.1	ENSG00000227769.7	0	0
antisense	AC012462.3	ENSG00000230695.1	0	0
antisense	AC012513.1	ENSG00000231092.1	0	0
antisense	AC069155.1	ENSG00000233581.1	0	0
antisense	AC098820.2	ENSG00000235042.1	0	0
antisense	LINC01280	ENSG00000224391.1	0	0
antisense	AC007563.2	ENSG00000236886.2	0	0
antisense	AC007557.2	ENSG00000231597.1	0	0
antisense	AC007557.4	ENSG00000237479.1	0	0
antisense	AC010136.1	ENSG00000223923.1	0	0
antisense	CATIP-AS2	ENSG00000237281.1	0	0
antisense	LINC01494	ENSG00000228135.1	0	0
antisense	AC097468.2	ENSG00000235024.1	0	0
antisense	LINC00608	ENSG00000236445.4	0	0
antisense	AC097468.1	ENSG00000224090.1	0	0
antisense	AC053503.2	ENSG00000229525.1	0	0
antisense	AC053503.4	ENSG00000234638.1	0	0
antisense	AC053503.1	ENSG00000227432.1	0	0
antisense	AC053503.5	ENSG00000268603.1	0	0
antisense	AC009955.3	ENSG00000268896.1	0	0
antisense	AC009955.4	ENSG00000269068.1	0	0
antisense	AC009955.1	ENSG00000228973.1	0	0
antisense	AC013476.1	ENSG00000234193.1	0	0
antisense	AC073641.1	ENSG00000228802.1	0	0
antisense	AC073052.2	ENSG00000274629.1	0	0
antisense	AC064853.1	ENSG00000236116.1	0	0
antisense	AC009950.1	ENSG00000225963.7	0	0
antisense	AC010149.1	ENSG00000235419.5	0	0
antisense	AC012507.2	ENSG00000232520.1	0	0
antisense	AC012507.1	ENSG00000230385.1	0	0
antisense	AC012507.4	ENSG00000283164.1	0	0
antisense	AC019130.1	ENSG00000227033.1	0	0
antisense	AC068134.1	ENSG00000224516.5	0	0
antisense	AC073254.1	ENSG00000237126.8	0	0

antisense	AC013726.1	ENSG00000259793.1	0	0
antisense	AC114812.2	ENSG00000224814.1	0	0
antisense	AC005538.1	ENSG00000237581.1	0	0
antisense	AC064874.1	ENSG00000222007.6	0	0
antisense	AC093915.1	ENSG00000270540.1	0	0
antisense	IQCA1-AS1	ENSG00000232893.1	0	0
antisense	AC104667.2	ENSG00000234949.2	0	0
antisense	AC012485.3	ENSG00000283635.1	0	0
antisense	AC016999.1	ENSG00000229915.1	0	0
antisense	CAPN10-DT	ENSG00000260942.1	0	0
antisense	AC104809.1	ENSG00000223991.1	0	0
antisense	AC093585.1	ENSG00000229996.1	0	0
antisense	AC005237.1	ENSG00000225521.1	0	0
antisense	AC005104.1	ENSG00000223374.1	0	0
antisense	BOK-AS1	ENSG00000234235.1	0	0
antisense	AC114730.3	ENSG00000235351.1	0	0
antisense	AC114730.1	ENSG00000215692.2	0	0
antisense	AC114730.2	ENSG00000234793.1	0	0
antisense	AC131097.1	ENSG00000215023.2	0	0
antisense	AC131097.3	ENSG00000224272.2	0	0
antisense	LINC01238	ENSG00000261186.2	0	0
antisense	CHL1-AS1	ENSG00000234661.2	0	0
antisense	CNTN4-AS1	ENSG00000237990.3	0	0
antisense	ITPR1-DT	ENSG00000231249.1	0	0
antisense	AC018816.1	ENSG00000235978.6	0	0
antisense	AC026202.2	ENSG00000233912.1	0	0
antisense	AC026202.3	ENSG00000268509.2	0	0
antisense	GRM7-AS2	ENSG00000237665.1	0	0
antisense	GRM7-AS1	ENSG00000236202.1	0	0
antisense	AC068313.1	ENSG00000270207.1	0	0
antisense	SRGAP3-AS1	ENSG00000224808.1	0	0
antisense	SRGAP3-AS2	ENSG00000228723.6	0	0
antisense	SRGAP3-AS3	ENSG00000227929.4	0	0
antisense	AC026191.1	ENSG00000254485.5	0	0
antisense	AC018809.2	ENSG00000269982.1	0	0
antisense	SLC6A1-AS1	ENSG00000232287.2	0	0
antisense	AC022001.2	ENSG00000271716.1	0	0
antisense	AC022001.3	ENSG00000272483.1	0	0
antisense	HDAC11-	ENSG00000244502.2	0	0
antisense	LINC00620	ENSG00000224514.2	0	0
antisense	AC093495.1	ENSG00000228242.6	0	0
antisense	AC090952.1	ENSG00000235629.1	0	0
antisense	AC090957.1	ENSG00000230172.1	0	0
antisense	EAF1-AS1	ENSG00000249786.7	0	0

antisense	PLCL2-AS1	ENSG00000226441.2	0	0
antisense	ZNF385D-	ENSG00000225542.1	0	0
antisense	UBE2E2-AS1	ENSG00000233153.5	0	0
antisense	RBMS3-AS2	ENSG00000203506.5	0	0
antisense	RBMS3-AS1	ENSG00000235593.1	0	0
antisense	CNOT10-AS1	ENSG00000251224.1	0	0
antisense	AC112211.1	ENSG00000272149.1	0	0
antisense	ARPP21-AS1	ENSG00000230830.1	0	0
antisense	ACVR2B-AS1	ENSG00000229589.1	0	0
antisense	AC116038.1	ENSG00000231243.1	0	0
antisense	AC092053.3	ENSG00000284669.1	0	1
antisense	AC092053.2	ENSG00000283849.1	0	0
antisense	AC092058.1	ENSG00000285885.1	0	0
antisense	AC018358.1	ENSG00000281160.1	0	0
antisense	VIPR1-AS1	ENSG00000232354.8	0	0
antisense	HHATL-AS1	ENSG00000230970.3	0	0
antisense	CCDC13-AS1	ENSG00000173811.10	0	0
antisense	LINC02158	ENSG00000225611.1	0	0
antisense	AC099329.1	ENSG00000235288.3	0	1
antisense	KRBOX1-AS1	ENSG00000206552.4	0	0
antisense	SNRK-AS1	ENSG00000234617.2	0	2
antisense	ZKSCAN7-	ENSG00000236869.1	0	0
antisense	ZNF197-AS1	ENSG00000233509.2	0	0
antisense	AC098649.1	ENSG00000235845.1	0	0
antisense	LARS2-AS1	ENSG00000232455.2	0	0
antisense	AC099782.2	ENSG00000285788.1	0	0
antisense	AC098613.1	ENSG00000223552.1	0	0
antisense	LRRC2-AS1	ENSG00000268324.2	0	0
antisense	KIF9-AS1	ENSG00000227398.3	0	4
antisense	AC134772.1	ENSG00000244380.1	0	0
antisense	LINC02585	ENSG00000228350.1	0	0
antisense	AC137630.2	ENSG00000235236.1	0	0
antisense	AC137630.1	ENSG00000223343.1	0	0
antisense	BSN-AS1	ENSG00000235120.1	0	0
antisense	AC105935.2	ENSG00000230698.1	0	0
antisense	AC105935.1	ENSG00000228008.1	0	0
antisense	RBM5-AS1	ENSG00000281691.1	0	0
antisense	SEMA3F-AS1	ENSG00000235016.1	0	0
antisense	SEMA3B-AS1	ENSG00000232352.1	0	0
antisense	ZMYND10-	ENSG00000235058.1	0	0
antisense	IQCF5-AS1	ENSG00000235455.1	0	0
antisense	ITIH4-AS1	ENSG00000239799.1	0	0
antisense	AC012467.1	ENSG00000271916.1	0	0

antisense	CACNA2D3-AS1	ENSG00000243715.1	0	0
antisense	WNT5A-AS1	ENSG00000244586.1	0	0
antisense	ARHGEF3-	ENSG00000240198.5	0	0
antisense	AC097358.2	ENSG00000272202.1	0	0
antisense	FLNB-AS1	ENSG00000244161.1	0	0
antisense	AC119424.1	ENSG00000243384.1	0	0
antisense	C3orf67-AS1	ENSG00000242428.5	0	0
antisense	SYNPR-AS1	ENSG00000241359.1	0	0
antisense	THOC7-AS1	ENSG00000240549.2	0	0
antisense	SCAANT1	ENSG00000280620.1	0	0
antisense	AC012557.2	ENSG00000272181.1	0	2
antisense	PRICKLE2-	ENSG00000241111.1	0	0
antisense	PRICKLE2-	ENSG00000241572.1	0	0
antisense	PRICKLE2-	ENSG00000241101.1	0	0
antisense	PRICKLE2-	ENSG00000226017.2	0	0
antisense	MAGI1-AS1	ENSG00000240175.1	0	0
antisense	AC109587.1	ENSG00000244513.6	0	2
antisense	FOXP1-AS1	ENSG00000244203.2	0	0
antisense	AC097634.2	ENSG00000274387.1	0	0
antisense	PDZRN3-AS1	ENSG00000239677.6	0	0
antisense	CADM2-AS2	ENSG00000241648.1	0	0
antisense	CADM2-AS1	ENSG00000239519.1	0	0
antisense	AC109129.1	ENSG00000285780.1	0	0
antisense	AC110491.3	ENSG00000282527.1	0	0
antisense	AC110491.2	ENSG00000279658.1	0	0
antisense	AC117460.1	ENSG00000249225.1	0	0
antisense	AC021660.2	ENSG00000248839.1	0	0
antisense	AC091212.1	ENSG00000239462.1	0	0
antisense	AC020651.1	ENSG00000249474.1	0	0
antisense	MORC1-AS1	ENSG00000239314.1	0	0
antisense	PLCXD2-AS1	ENSG00000240766.1	0	0
antisense	AC078785.1	ENSG00000240057.5	0	0
antisense	CFAP44-AS1	ENSG00000243849.1	0	0
antisense	SIDT1-AS1	ENSG00000239453.1	0	0
antisense	AC128687.2	ENSG00000273394.1	0	0
antisense	AC092896.2	ENSG00000285836.1	0	0
antisense	AC093010.2	ENSG00000241490.1	0	0
antisense	ZBTB20-AS1	ENSG00000241560.5	0	0
antisense	LSAMP-AS1	ENSG00000240922.1	0	0
antisense	LINC00903	ENSG00000241397.1	0	0
antisense	TUSC7	ENSG00000243197.7	0	0
antisense	LINC00901	ENSG00000242385.1	0	0

antisense	IGSF11-AS1	ENSG00000239877.2	0	0
antisense	B4GALT4-	ENSG00000240254.1	0	0
antisense	ARHGAP31-AS1	ENSG00000241155.1	0	0
antisense	AC073352.2	ENSG00000272967.1	0	0
antisense	AC069444.1	ENSG00000239994.2	0	0
antisense	MYLK-AS2	ENSG00000250174.5	0	0
antisense	AC020634.1	ENSG00000273123.1	0	0
antisense	ITGB5-AS1	ENSG00000244286.1	0	0
antisense	AC117422.1	ENSG00000248607.1	0	0
antisense	AC079848.1	ENSG00000250012.1	0	0
antisense	ALDH1L1-	ENSG00000250218.1	0	0
antisense	ALDH1L1-	ENSG00000246022.2	0	0
antisense	CCDC37-DT	ENSG00000249833.5	0	0
antisense	AC023593.1	ENSG00000285600.1	0	0
antisense	RUVBL1-AS1	ENSG00000239608.1	0	0
antisense	DNAJB8-AS1	ENSG00000242049.1	0	0
antisense	AC112484.1	ENSG00000231305.3	0	0
antisense	AC083906.3	ENSG00000250643.1	0	0
antisense	AC055733.2	ENSG00000250592.1	0	0
antisense	AC116424.1	ENSG00000250129.5	0	0
antisense	AC107027.1	ENSG00000248468.1	0	0
antisense	TMEM108-	ENSG00000251011.5	0	0
antisense	BFSP2-AS1	ENSG00000249993.1	0	0
antisense	AC080128.2	ENSG00000285908.1	0	0
antisense	AC092969.1	ENSG00000240086.6	0	0
antisense	IL20RB-AS1	ENSG00000249407.1	0	0
antisense	AC097103.1	ENSG00000248790.1	0	0
antisense	AC010181.1	ENSG00000249290.5	0	0
antisense	AC010181.2	ENSG00000251058.1	0	0
antisense	CLSTN2-AS1	ENSG00000250433.1	0	0
antisense	AC108727.2	ENSG00000251270.1	0	0
antisense	AC117383.1	ENSG00000249417.1	0	0
antisense	ATP1B3-AS1	ENSG00000244124.1	0	0
antisense	PLS1-AS1	ENSG00000239641.1	0	0
antisense	AC021074.3	ENSG00000243818.4	0	0
antisense	PAQR9-AS1	ENSG00000241570.8	0	0
antisense	SLC9A9-AS1	ENSG00000240012.1	0	0
antisense	SLC9A9-AS2	ENSG00000244493.1	0	0
antisense	AC107021.1	ENSG00000243415.2	0	0
antisense	LNCSRLR	ENSG00000240032.1	0	1
antisense	PLSCR5-AS1	ENSG00000241457.1	0	0
antisense	AC092957.1	ENSG00000243620.1	0	0

antisense	ZIC4-AS1	ENSG00000241202.1	0	0
antisense	AC092979.1	ENSG00000240521.1	0	0
antisense	HLTF-AS1	ENSG00000239718.1	0	0
antisense	AC093001.1	ENSG00000244468.1	0	0
antisense	WWTR1-AS1	ENSG00000241313.2	0	0
antisense	AC117386.2	ENSG00000243944.5	0	0
antisense	SIAH2-AS1	ENSG00000244265.1	0	0
antisense	CLRN1-AS1	ENSG00000239265.5	0	0
antisense	AC020636.1	ENSG00000243273.1	0	0
antisense	AADACL2-	ENSG00000242908.6	0	0
antisense	AC026347.1	ENSG00000243305.1	0	0
antisense	AC117394.2	ENSG00000244268.1	0	0
antisense	MME-AS1	ENSG00000240666.2	0	0
antisense	PLCH1-AS1	ENSG00000239508.1	0	0
antisense	PLCH1-AS2	ENSG00000242925.1	0	0
antisense	KCNAB1-	ENSG00000240596.1	0	0
antisense	KCNAB1-	ENSG00000242370.1	0	0
antisense	AC104411.1	ENSG00000241770.1	0	0
antisense	AC080013.5	ENSG00000272247.1	0	0
antisense	IQCJ-SCHIP1-AS1	ENSG00000241211.1	0	0
antisense	AC074033.1	ENSG00000241479.1	0	0
antisense	AC078795.1	ENSG00000269984.1	0	0
antisense	AC078795.3	ENSG00000270135.1	0	0
antisense	AC008040.1	ENSG00000239219.2	0	0
antisense	SEC62-AS1	ENSG00000240373.1	0	0
antisense	NLGN1-AS1	ENSG00000228213.5	0	0
antisense	NAALADL2-AS2	ENSG00000226779.1	0	0
antisense	TBL1XR1-	ENSG00000231310.3	0	0
antisense	AC007620.3	ENSG00000272699.1	0	0
antisense	AC090425.2	ENSG00000272910.1	0	2
antisense	PEX5L-AS1	ENSG00000243799.1	0	0
antisense	PEX5L-AS2	ENSG00000244302.1	0	0
antisense	CCDC39-AS1	ENSG00000243187.1	0	0
antisense	MCCC1-AS1	ENSG00000243368.2	0	0
antisense	ABCC5-AS1	ENSG00000223882.1	0	0
antisense	HTR3E-AS1	ENSG00000238020.1	0	0
antisense	AC131235.3	ENSG00000272721.5	0	0
antisense	LINC01840	ENSG00000230215.1	0	0
antisense	EHHADH-	ENSG00000223358.5	0	0
antisense	IGF2BP2-AS1	ENSG00000163915.7	0	0
antisense	ETV5-AS1	ENSG00000234197.1	0	0

antisense	AC068631.1	ENSG00000197099.8	0	0
antisense	AC112907.3	ENSG00000263826.1	0	0
antisense	AC112907.1	ENSG00000231724.1	0	0
antisense	ADIPOQ-	ENSG00000226482.1	0	0
antisense	AC007920.1	ENSG00000198491.3	0	0
antisense	AC072022.1	ENSG00000228804.5	0	0
antisense	AC072022.2	ENSG00000285938.1	0	0
antisense	LPP-AS1	ENSG00000224563.1	0	0
antisense	TPRG1-AS2	ENSG00000230115.1	0	0
antisense	P3H2-AS1	ENSG00000225764.1	0	0
antisense	OSTN-AS1	ENSG00000233308.1	0	1
antisense	FGF12-AS1	ENSG00000231383.2	0	0
antisense	FGF12-AS2	ENSG00000230126.1	0	0
antisense	FGF12-AS3	ENSG00000226709.1	0	0
antisense	ATP13A5-	ENSG00000236508.1	0	0
antisense	ATP13A4-	ENSG00000225473.1	0	0
antisense	OPA1-AS1	ENSG00000224855.5	0	0
antisense	AC080129.2	ENSG00000232874.1	0	0
antisense	XXYLT1-AS1	ENSG00000233303.1	0	0
antisense	XXYLT1-AS2	ENSG00000230266.1	0	0
antisense	AC069213.1	ENSG00000223711.1	0	0
antisense	TNK2-AS1	ENSG00000224614.1	0	0
antisense	AC069257.1	ENSG00000228028.2	0	0
antisense	UBXN7-AS1	ENSG00000225822.4	0	0
antisense	LINC01063	ENSG00000232065.1	0	0
antisense	NCBP2-AS1	ENSG00000225578.1	0	0
antisense	AC055764.1	ENSG00000234136.1	0	0
antisense	LMLN-AS1	ENSG00000232832.1	0	0
antisense	AC092574.2	ENSG00000281016.1	0	0
antisense	AC107464.1	ENSG00000242686.4	0	0
antisense	AC107464.2	ENSG00000248416.1	0	0
antisense	AC139887.4	ENSG00000272588.1	0	0
antisense	AC139887.1	ENSG00000233799.1	0	0
antisense	AC092535.3	ENSG00000251652.1	0	0
antisense	AC092535.1	ENSG00000227189.2	0	0
antisense	AC092535.4	ENSG00000273179.1	0	0
antisense	CTBP1-AS	ENSG00000280927.1	0	0
antisense	AL136360.2	ENSG00000250623.1	0	0
antisense	AL158068.2	ENSG00000251148.1	0	0
antisense	HTT-AS	ENSG00000251075.1	0	0
antisense	AC097382.2	ENSG00000245748.1	0	0
antisense	AFAP1-AS1	ENSG00000272620.1	0	0
antisense	AC097381.3	ENSG00000273267.1	0	0
antisense	AC097381.2	ENSG00000251460.1	0	0

antisense	AC105345.2	ENSG00000251186.1	0	0
antisense	AC108199.1	ENSG00000249219.1	0	0
antisense	AC005674.1	ENSG00000250413.1	0	0
antisense	AC098829.1	ENSG00000249252.5	0	0
antisense	AC099550.1	ENSG00000251379.1	0	0
antisense	AC108063.1	ENSG00000249234.1	0	0
antisense	TAPT1-AS1	ENSG00000263327.6	0	0
antisense	AC097515.1	ENSG00000248138.5	0	0
antisense	AC006160.1	ENSG00000249502.1	0	0
antisense	AC097505.1	ENSG00000272995.1	0	1
antisense	AC110296.1	ENSG00000250243.2	0	0
antisense	AC096576.3	ENSG00000250092.2	0	0
antisense	AC096576.2	ENSG00000248343.1	0	0
antisense	AC096719.1	ENSG00000250039.3	0	0
antisense	AC093607.1	ENSG00000250137.1	0	0
antisense	AC092834.1	ENSG00000249453.1	0	0
antisense	AC092436.4	ENSG00000250541.1	0	0
antisense	AC106047.1	ENSG00000240005.5	0	1
antisense	AC097716.1	ENSG00000249678.1	0	0
antisense	AC104078.1	ENSG00000247193.2	0	0
antisense	AC104078.2	ENSG00000251438.1	0	0
antisense	AC027607.1	ENSG00000248936.1	0	0
antisense	AC079921.1	ENSG00000249207.1	0	0
antisense	UGDH-AS1	ENSG00000249348.1	0	0
antisense	AC098869.2	ENSG00000250893.1	0	0
antisense	AC131953.1	ENSG00000250906.1	0	0
antisense	UCHL1-AS1	ENSG00000251173.1	0	0
antisense	AC105389.2	ENSG00000249216.1	0	0
antisense	AC105389.3	ENSG00000250467.1	0	0
antisense	AC024022.1	ENSG00000250781.1	0	0
antisense	AC096734.2	ENSG00000260519.1	0	0
antisense	AC096586.2	ENSG00000273369.1	0	0
antisense	AC095060.1	ENSG00000249330.1	0	0
antisense	AC107398.2	ENSG00000248254.1	0	0
antisense	AC107068.2	ENSG00000282917.1	0	0
antisense	OCIAD1-AS1	ENSG00000248256.1	0	0
antisense	AC023154.1	ENSG00000248115.1	0	0
antisense	LNX1-AS1	ENSG00000250930.5	0	0
antisense	AC110792.2	ENSG00000269506.2	0	0
antisense	AC111194.2	ENSG00000250646.1	0	0
antisense	ADGRL3-	ENSG00000248692.5	0	0
antisense	AC111000.4	ENSG00000250696.5	0	0
antisense	AC095056.1	ENSG00000250877.1	0	0
antisense	AC053527.1	ENSG00000250220.1	0	0

antisense	AC021180.1	ENSG00000250532.1	0	0
antisense	AC239584.1	ENSG00000249942.1	0	0
antisense	AC110760.2	ENSG00000249717.1	0	0
antisense	AC110760.1	ENSG00000248165.1	0	0
antisense	AC110615.1	ENSG00000229717.2	0	0
antisense	AC112719.2	ENSG00000245928.2	0	0
antisense	AC107072.2	ENSG00000233860.1	0	0
antisense	AC107072.1	ENSG00000224218.1	0	0
antisense	AC098818.2	ENSG00000260278.1	0	1
antisense	AC021127.1	ENSG00000248719.1	0	0
antisense	AC139722.1	ENSG00000251059.5	0	0
antisense	WDFY3-AS1	ENSG00000251260.1	0	0
antisense	AC104827.1	ENSG00000250062.5	0	0
antisense	AC097478.2	ENSG00000274238.1	0	0
antisense	AC097478.4	ENSG00000277695.1	0	0
antisense	AC097478.3	ENSG00000276542.1	0	2
antisense	SNCA-AS1	ENSG00000247775.2	0	0
antisense	AC004054.1	ENSG00000248984.1	0	0
antisense	AC074124.1	ENSG00000249049.1	0	0
antisense	AC095059.1	ENSG00000248627.1	0	0
antisense	AC110800.1	ENSG00000250908.1	0	0
antisense	AC106881.1	ENSG00000271474.1	0	0
antisense	STPG2-AS1	ENSG00000251620.1	0	0
antisense	AC108159.1	ENSG00000251523.1	0	0
antisense	AC019131.1	ENSG00000263923.1	0	0
antisense	AC083902.1	ENSG00000248676.1	0	0
antisense	AC097460.1	ENSG00000245322.6	0	0
antisense	AP001961.1	ENSG00000249710.1	0	0
antisense	AP002075.1	ENSG00000251309.1	0	0
antisense	AC105460.2	ENSG00000251577.5	0	0
antisense	CXXC4-AS1	ENSG00000245384.1	0	0
antisense	TET2-AS1	ENSG00000251586.1	0	0
antisense	AC004066.2	ENSG00000250522.1	0	0
antisense	AC008243.1	ENSG00000251175.5	0	0
antisense	AC109361.2	ENSG00000250740.1	0	0
antisense	AC096564.1	ENSG00000245293.2	0	0
antisense	AC096564.2	ENSG00000249604.1	0	0
antisense	AC098798.1	ENSG00000250511.1	0	0
antisense	AC023886.1	ENSG00000249509.1	0	0
antisense	AC106864.1	ENSG00000249532.5	0	0
antisense	AC017007.5	ENSG00000251126.2	0	0
antisense	AC093879.1	ENSG00000248152.1	0	0
antisense	AC093879.2	ENSG00000249373.2	0	0
antisense	AC104779.1	ENSG00000249304.1	0	0

antisense	PP12613	ENSG00000226757.2	0	0
antisense	IL21-AS1	ENSG00000227145.1	0	0
antisense	AC021205.3	ENSG00000273007.1	0	0
antisense	AC093591.2	ENSG00000261668.1	0	0
antisense	AC109927.1	ENSG00000250195.1	0	0
antisense	AC112236.2	ENSG00000272717.1	0	2
antisense	AC131182.1	ENSG00000250698.1	0	0
antisense	AC096733.1	ENSG00000248335.1	0	0
antisense	AC104596.1	ENSG00000250326.1	0	0
antisense	SMARCA5-	ENSG00000245112.2	0	0
antisense	HHIP-AS1	ENSG00000248890.1	0	0
antisense	SMAD1-AS2	ENSG00000250582.1	0	0
antisense	SMAD1-AS1	ENSG00000250902.1	0	0
antisense	AC093864.1	ENSG00000248356.1	0	0
antisense	AC104791.1	ENSG00000251687.1	0	0
antisense	AC097372.1	ENSG00000251010.1	0	0
antisense	AC092435.2	ENSG00000248764.1	0	0
antisense	AC093835.1	ENSG00000251298.1	0	0
antisense	AC069272.1	ENSG00000250354.1	0	0
antisense	AC110813.1	ENSG00000249690.1	0	0
antisense	AC095055.1	ENSG00000270681.1	0	0
antisense	AC092611.2	ENSG00000251603.1	0	0
antisense	FBXW7-AS1	ENSG00000270751.1	0	0
antisense	AC106882.1	ENSG00000248571.1	0	0
antisense	AC020703.1	ENSG00000249309.1	0	0
antisense	AC079298.3	ENSG00000280241.3	0	0
antisense	AC079298.1	ENSG00000278981.1	0	0
antisense	AC079298.2	ENSG00000280005.1	0	0
antisense	AC009567.1	ENSG00000249041.1	0	0
antisense	AC097467.3	ENSG00000250910.7	0	0
antisense	AC104407.1	ENSG00000250538.5	0	0
antisense	AC096736.2	ENSG00000249479.1	0	0
antisense	AC092608.1	ENSG00000248629.1	0	0
antisense	AC098679.1	ENSG00000250604.1	0	0
antisense	AC121161.2	ENSG00000249901.1	0	0
antisense	AC104793.1	ENSG00000249568.1	0	0
antisense	AC023136.1	ENSG00000249419.1	0	0
antisense	AC022272.1	ENSG00000250027.1	0	0
antisense	AC116634.1	ENSG00000248601.1	0	0
antisense	AC079858.1	ENSG00000249623.1	0	0
antisense	AC080188.1	ENSG00000249609.1	0	0
antisense	AC021151.1	ENSG00000251445.1	0	0
antisense	AC096741.1	ENSG00000251171.1	0	0
antisense	AC084866.2	ENSG00000251200.1	0	0

antisense	AC084866.1	ENSG00000249955.1	0	0
antisense	AC097534.1	ENSG00000248774.1	0	0
antisense	AC105914.2	ENSG00000250431.1	0	0
antisense	AC097537.1	ENSG00000249106.1	0	0
antisense	AC019163.1	ENSG00000248980.1	0	0
antisense	AC093801.1	ENSG00000248388.5	0	0
antisense	AC027627.1	ENSG00000249084.1	0	0
antisense	AC078881.1	ENSG00000250131.1	0	0
antisense	AC098864.1	ENSG00000177822.7	0	0
antisense	AC079226.2	ENSG00000248816.1	0	0
antisense	AC079226.1	ENSG00000248694.1	0	0
antisense	WWC2-AS1	ENSG00000251128.1	0	0
antisense	AC084871.1	ENSG00000251139.2	0	0
antisense	AC112722.1	ENSG00000250410.1	0	0
antisense	AC093797.1	ENSG00000233110.1	0	0
antisense	AC108472.1	ENSG00000235902.1	0	0
antisense	F11-AS1	ENSG00000251165.5	0	0
antisense	AC138781.1	ENSG00000247130.2	0	0
antisense	HRAT5	ENSG00000248925.1	0	0
antisense	AC106772.2	ENSG00000250385.1	0	0
antisense	AC106772.1	ENSG00000249650.1	0	0
antisense	AC116351.1	ENSG00000215246.5	0	0
antisense	CTD-	ENSG00000249201.2	0	0
antisense	AC025183.2	ENSG00000249966.1	0	0
antisense	CTD-	ENSG00000249326.1	0	0
antisense	AC016595.1	ENSG00000259603.1	0	0
antisense	CTD-	ENSG00000250579.1	0	0
antisense	AC022424.1	ENSG00000250866.1	0	0
antisense	AC027343.2	ENSG00000249865.1	0	0
antisense	AC093305.1	ENSG00000250761.2	0	0
antisense	AC021088.1	ENSG00000250619.1	0	0
antisense	AC091906.1	ENSG00000248537.1	0	0
antisense	ROPN1L-AS1	ENSG00000250600.1	0	0
antisense	LINC01513	ENSG00000246016.2	0	0
antisense	ANKRD33B-	ENSG00000250106.1	0	0
antisense	AC012629.2	ENSG00000272324.5	0	0
antisense	AC016576.1	ENSG00000251423.2	0	0
antisense	AC010638.1	ENSG00000250250.1	0	0
antisense	AC016650.1	ENSG00000250981.1	0	0
antisense	AC092335.1	ENSG00000250448.1	0	0
antisense	AC020980.1	ENSG00000249737.1	0	0
antisense	AC022113.1	ENSG00000246214.1	0	0
antisense	BASP1-AS1	ENSG00000215196.4	0	0
antisense	AC091946.1	ENSG00000253766.1	0	0

antisense	AC091885.2	ENSG00000251294.1	0	0
antisense	AC113386.1	ENSG00000254138.1	0	0
antisense	AC025178.1	ENSG00000250764.1	0	0
antisense	AC025754.1	ENSG00000250234.1	0	0
antisense	AC137810.1	ENSG00000248969.1	0	0
antisense	AC112204.2	ENSG00000249748.1	0	0
antisense	NADK2-AS1	ENSG00000245711.2	0	0
antisense	AC008957.2	ENSG00000274441.1	0	0
antisense	AC008957.1	ENSG00000250155.1	0	0
antisense	EGFLAM-	ENSG00000248730.1	0	0
antisense	EGFLAM-	ENSG00000249071.2	0	0
antisense	EGFLAM-	ENSG00000248572.5	0	0
antisense	EGFLAM-	ENSG00000249491.1	0	0
antisense	AC010457.1	ENSG00000251257.2	0	0
antisense	AC091435.2	ENSG00000250629.1	0	0
antisense	AC008945.1	ENSG00000272234.1	0	0
antisense	AC025171.3	ENSG00000251131.1	0	0
antisense	AC114947.2	ENSG00000261604.1	0	0
antisense	AC114956.2	ENSG00000248554.1	0	0
antisense	FGF10-AS1	ENSG00000248464.1	0	0
antisense	AC022126.1	ENSG00000248898.1	0	0
antisense	AC025180.1	ENSG00000249899.5	0	0
antisense	AC026704.1	ENSG00000251307.1	0	0
antisense	AC008914.1	ENSG00000262211.1	0	0
antisense	FLJ31104	ENSG00000227908.3	0	1
antisense	AC008937.2	ENSG00000237705.1	0	0
antisense	AC008937.1	ENSG00000225230.1	0	0
antisense	AC016644.1	ENSG00000235635.1	0	0
antisense	AC025470.2	ENSG00000250961.1	0	2
antisense	AC016642.1	ENSG00000248475.5	0	0
antisense	AC008852.1	ENSG00000248733.1	0	0
antisense	AC092343.1	ENSG00000247345.2	0	0
antisense	AC034234.1	ENSG00000248935.1	0	0
antisense	AC022445.1	ENSG00000233847.1	0	0
antisense	SMIM15-AS1	ENSG00000251279.1	0	0
antisense	AC026746.1	ENSG00000248529.5	0	0
antisense	AC025442.1	ENSG00000253744.1	0	0
antisense	AC010273.1	ENSG00000248664.1	0	0
antisense	AC145146.1	ENSG00000249295.1	0	0
antisense	AC140134.2	ENSG00000285151.1	0	0
antisense	AC139834.1	ENSG00000285204.1	0	0
antisense	AC093218.1	ENSG00000285804.1	0	2
antisense	AC035140.1	ENSG00000249085.1	0	0
antisense	AC116345.3	ENSG00000251599.1	0	0

antisense	LINC01386	ENSG00000251324.1	0	0
antisense	AC091868.2	ENSG00000249293.1	0	0
antisense	AC010501.1	ENSG00000271714.1	0	0
antisense	AC010245.1	ENSG00000248881.1	0	0
antisense	AC113404.1	ENSG00000250348.1	0	0
antisense	AC026725.1	ENSG00000249713.1	0	0
antisense	AC025188.1	ENSG00000225407.3	0	0
antisense	AC008581.1	ENSG00000250615.1	0	0
antisense	AC008496.2	ENSG00000250258.1	0	0
antisense	LINC01455	ENSG00000250888.1	0	0
antisense	CTD-	ENSG00000249825.5	0	0
antisense	AC010260.1	ENSG00000251675.1	0	0
antisense	AC026427.1	ENSG00000249772.1	0	0
antisense	ATG10-AS1	ENSG00000248192.1	0	0
antisense	VCAN-AS1	ENSG00000249835.2	0	0
antisense	AC018754.1	ENSG00000285190.1	0	0
antisense	MEF2C-AS2	ENSG00000245864.2	0	0
antisense	AC008525.2	ENSG00000250555.2	0	0
antisense	AC074132.1	ENSG00000285618.1	0	1
antisense	AC008799.2	ENSG00000241059.2	0	0
antisense	AC008534.1	ENSG00000249175.1	0	0
antisense	AC012312.1	ENSG00000249545.1	0	0
antisense	AC008840.1	ENSG00000250240.5	0	0
antisense	AC104123.1	ENSG00000251314.2	0	0
antisense	AC020900.1	ENSG00000249180.1	0	0
antisense	AC008906.2	ENSG00000272109.1	0	0
antisense	AC008883.1	ENSG00000248758.1	0	0
antisense	RGMB-AS1	ENSG00000246763.6	0	1
antisense	AC094108.1	ENSG00000249017.1	0	0
antisense	AC011362.1	ENSG00000248203.1	0	0
antisense	AC008871.1	ENSG00000250383.1	0	0
antisense	AC008572.1	ENSG00000253613.2	0	0
antisense	AC010468.2	ENSG00000249318.1	0	0
antisense	AC010275.1	ENSG00000248268.1	0	0
antisense	NREP-AS1	ENSG00000250095.5	0	0
antisense	AC010261.1	ENSG00000250882.1	0	0
antisense	AC010261.2	ENSG00000251187.5	0	0
antisense	AC104126.1	ENSG00000251076.1	0	0
antisense	AC079465.1	ENSG00000232633.4	0	0
antisense	AC010230.1	ENSG00000246316.7	0	0
antisense	AC008494.2	ENSG00000249791.1	0	0
antisense	SEMA6A-	ENSG00000248445.5	0	0
antisense	AC008629.1	ENSG00000249494.5	0	0
antisense	AC010255.1	ENSG00000247311.2	0	0

antisense	AC010255.2	ENSG00000249610.1	0	0
antisense	AC113349.2	ENSG00000272139.1	0	2
antisense	AC119150.1	ENSG00000249916.1	0	0
antisense	AC106786.2	ENSG00000249996.1	0	0
antisense	AC106786.1	ENSG00000223652.2	0	0
antisense	AC112196.1	ENSG00000249112.1	0	0
antisense	AC093535.1	ENSG00000250602.6	0	0
antisense	AC022118.1	ENSG00000248799.1	0	0
antisense	ADAMTS19-	ENSG00000249421.1	0	0
antisense	AC008591.1	ENSG00000251680.5	0	0
antisense	AC034228.3	ENSG00000234758.1	0	0
antisense	AC034228.1	ENSG00000223548.1	0	0
antisense	AC034228.2	ENSG00000231585.1	0	0
antisense	P4HA2-AS1	ENSG00000237714.1	0	0
antisense	AC116366.2	ENSG00000238160.1	0	0
antisense	TH2LCRR	ENSG00000223442.1	0	0
antisense	AC004039.1	ENSG00000230612.3	0	0
antisense	AC010307.2	ENSG00000248245.1	0	0
antisense	AC010307.3	ENSG00000249478.1	0	0
antisense	AC010307.4	ENSG00000250244.5	0	0
antisense	AC010608.1	ENSG00000250409.1	0	0
antisense	AC008406.3	ENSG00000277619.1	0	0
antisense	AC026691.1	ENSG00000270021.1	0	0
antisense	AC022092.1	ENSG00000249639.1	0	0
antisense	AC034206.1	ENSG00000250167.1	0	0
antisense	TRPC7-AS1	ENSG00000248211.1	0	0
antisense	TRPC7-AS2	ENSG00000250947.1	0	0
antisense	AC113382.1	ENSG00000246323.2	0	0
antisense	AC104116.1	ENSG00000249971.1	0	0
antisense	AC011405.1	ENSG00000249593.6	0	0
antisense	AC135457.1	ENSG00000272742.1	0	0
antisense	AC008667.2	ENSG00000250692.1	0	0
antisense	AC008438.2	ENSG00000253965.1	0	0
antisense	AC005609.5	ENSG00000279726.1	0	0
antisense	AC005609.2	ENSG00000278907.1	0	0
antisense	AC005609.4	ENSG00000278946.1	0	0
antisense	AC005609.3	ENSG00000278915.1	0	0
antisense	AC005609.1	ENSG00000278901.1	0	0
antisense	AC010223.1	ENSG00000279028.1	0	0
antisense	AC244517.5	ENSG00000279047.3	0	0
antisense	AC244517.2	ENSG00000272154.4	0	0
antisense	AC244517.4	ENSG00000278936.1	0	0
antisense	AC244517.7	ENSG00000279375.1	0	0
antisense	AC005618.3	ENSG00000280026.1	0	0

antisense	AC005618.2	ENSG00000279855.1	0	0
antisense	AC008781.2	ENSG00000246422.2	0	0
antisense	AC008781.1	ENSG00000228737.2	0	0
antisense	AC005740.3	ENSG00000254099.1	0	0
antisense	ARHGAP26-AS1	ENSG00000226272.5	0	0
antisense	AC091887.1	ENSG00000251556.1	0	0
antisense	AC011396.2	ENSG00000250025.2	0	0
antisense	AC008728.1	ENSG00000250407.1	0	0
antisense	STK32A-AS1	ENSG00000250343.1	0	0
antisense	AC011373.1	ENSG00000272239.1	0	0
antisense	JAKMIP2-	ENSG00000280780.2	0	0
antisense	AC011352.1	ENSG00000248362.1	0	0
antisense	AC011352.3	ENSG00000251320.1	0	0
antisense	AC091948.1	ENSG00000247199.4	0	0
antisense	AC012613.1	ENSG00000248647.2	0	0
antisense	AC012613.2	ENSG00000253406.1	0	0
antisense	GRPEL2-AS1	ENSG00000253618.1	0	0
antisense	AC131025.1	ENSG00000253865.1	0	0
antisense	AC011383.1	ENSG00000253852.1	0	0
antisense	AC008453.1	ENSG00000250309.2	0	0
antisense	CLMAT3	ENSG00000249035.6	0	0
antisense	AC091982.1	ENSG00000253921.1	0	0
antisense	SAP30L-AS1	ENSG00000245275.7	0	1
antisense	AC025434.1	ENSG00000254163.1	0	0
antisense	AC011377.1	ENSG00000254246.1	0	0
antisense	AC010609.1	ENSG00000253980.1	0	0
antisense	AC009185.1	ENSG00000253653.1	0	0
antisense	AC008676.1	ENSG00000248544.2	0	0
antisense	AC106801.1	ENSG00000253519.1	0	0
antisense	AC136424.2	ENSG00000253811.1	0	0
antisense	AC136424.1	ENSG00000253456.1	0	0
antisense	LINC01847	ENSG00000253311.2	0	0
antisense	AC008609.1	ENSG00000247699.2	0	0
antisense	AC008456.1	ENSG00000253687.1	0	0
antisense	AC011363.1	ENSG00000254391.1	0	0
antisense	AC091944.1	ENSG00000253403.1	0	0
antisense	AC113414.1	ENSG00000254186.2	0	0
antisense	HMMR-AS1	ENSG00000251018.2	0	0
antisense	AC091820.2	ENSG00000254365.1	0	0
antisense	AC091820.1	ENSG00000254297.1	0	0
antisense	AC008601.1	ENSG00000253527.1	0	0
antisense	AC008708.2	ENSG00000254187.1	0	0

antisense	AC008464.1	ENSG00000253660.1	0	0
antisense	AC008705.1	ENSG00000253947.1	0	0
antisense	AC011369.1	ENSG00000253925.1	0	0
antisense	AC011365.2	ENSG00000254192.1	0	0
antisense	AC011365.1	ENSG00000254042.1	0	0
antisense	AC011389.2	ENSG00000248965.1	0	0
antisense	AC011389.1	ENSG00000248222.5	0	0
antisense	AC008680.1	ENSG00000253269.1	0	0
antisense	CTD-	ENSG00000253647.1	0	0
antisense	AC008619.1	ENSG00000253858.1	0	0
antisense	AC027312.1	ENSG00000253591.1	0	0
antisense	AC008514.1	ENSG00000253348.1	0	0
antisense	AC091980.2	ENSG00000275038.2	0	0
antisense	AC027309.1	ENSG00000253445.1	0	0
antisense	AC022217.2	ENSG00000253295.1	0	0
antisense	AC022217.3	ENSG00000253736.2	0	0
antisense	AC008378.1	ENSG00000253172.1	0	0
antisense	AC008663.2	ENSG00000253955.1	0	0
antisense	AC113423.1	ENSG00000253244.1	0	0
antisense	AC113423.2	ENSG00000253447.6	0	0
antisense	AC138965.1	ENSG00000250801.1	0	0
antisense	AC139491.3	ENSG00000250992.1	0	0
antisense	AC139491.1	ENSG00000248469.1	0	0
antisense	AC138956.1	ENSG00000250909.1	0	0
antisense	AC138956.2	ENSG00000251414.1	0	0
antisense	AC145098.1	ENSG00000248996.1	0	0
antisense	AC138819.1	ENSG00000249849.1	0	0
antisense	AC140125.2	ENSG00000249109.1	0	0
antisense	AC140125.3	ENSG00000249186.1	0	0
antisense	AC136601.1	ENSG00000253698.1	0	0
antisense	AC104117.3	ENSG00000254035.1	0	0
antisense	AC136604.2	ENSG00000244945.1	0	0
antisense	AC136604.3	ENSG00000250999.1	0	0
antisense	AC010285.1	ENSG00000249412.1	0	0
antisense	AC010285.3	ENSG00000285865.1	0	0
antisense	AC008443.5	ENSG00000250900.6	0	0
antisense	AC008443.3	ENSG00000248514.1	0	0
antisense	AC008443.1	ENSG00000247049.2	0	0
antisense	AL031770.1	ENSG00000230433.1	0	0
antisense	AL035693.1	ENSG00000285603.1	0	0
antisense	AL031963.1	ENSG00000228170.1	0	0
antisense	AL445309.1	ENSG00000272320.1	0	0
antisense	AL138831.1	ENSG00000230648.1	0	0
antisense	AL136309.4	ENSG00000254821.1	0	0

antisense	AL356747.1	ENSG00000284823.1	0	0
antisense	AL022725.1	ENSG00000236336.1	0	0
antisense	AL021328.1	ENSG00000269985.1	0	0
antisense	AL022097.1	ENSG00000270174.1	0	0
antisense	LY86-AS1	ENSG00000216863.9	0	0
antisense	AL139095.4	ENSG00000238221.1	0	0
antisense	AL139039.3	ENSG00000237685.1	0	0
antisense	AL358777.1	ENSG00000285763.1	0	0
antisense	AL357497.1	ENSG00000235051.3	0	0
antisense	ELOVL2-AS1	ENSG00000230314.6	0	0
antisense	AL139807.1	ENSG00000247925.2	0	0
antisense	AL157373.1	ENSG00000225102.1	0	0
antisense	GFOD1-AS1	ENSG00000237786.1	0	0
antisense	JARID2-AS1	ENSG00000235488.1	0	0
antisense	AL009031.1	ENSG00000282024.1	0	0
antisense	AL137003.1	ENSG00000229931.1	0	0
antisense	AL158198.1	ENSG00000227803.2	0	0
antisense	AL513188.1	ENSG00000233848.1	0	0
antisense	AL133268.3	ENSG00000229313.1	0	0
antisense	AL133268.4	ENSG00000285801.1	0	0
antisense	U91328.2	ENSG00000272558.1	0	0
antisense	AL353759.1	ENSG00000283064.1	0	0
antisense	AL021918.4	ENSG00000285849.1	0	0
antisense	AL049543.1	ENSG00000246350.1	0	0
antisense	HCG15	ENSG00000227214.2	0	0
antisense	AL662791.1	ENSG00000244349.1	0	0
antisense	AL662791.2	ENSG00000277661.4	0	0
antisense	TRIM31-AS1	ENSG00000231226.1	0	0
antisense	LINC02569	ENSG00000235781.1	0	0
antisense	MDC1-AS1	ENSG00000224328.1	0	0
antisense	AL662797.1	ENSG00000272540.1	0	0
antisense	DDR1-DT	ENSG00000237775.1	0	0
antisense	HCG21	ENSG00000233529.1	0	0
antisense	DDX39B-AS1	ENSG00000234006.1	0	0
antisense	C6orf47-AS1	ENSG00000227198.1	0	0
antisense	SAPCD1-AS1	ENSG00000235663.1	0	0
antisense	EHMT2-AS1	ENSG00000237080.3	0	0
antisense	C2-AS1	ENSG00000281756.1	0	0
antisense	C4A-AS1	ENSG00000233627.2	0	0
antisense	C4B-AS1	ENSG00000229776.1	0	0
antisense	HCG23	ENSG00000225914.2	0	0
antisense	HLA-DQB1-	ENSG00000223534.1	0	0
antisense	AL645941.3	ENSG00000263756.1	0	0
antisense	HCG24	ENSG00000230313.1	0	0

antisense	AL157823.2	ENSG00000232909.1	0	0
antisense	Z84484.1	ENSG00000224666.3	0	0
antisense	AL121574.1	ENSG00000225945.1	0	0
antisense	BTBD9-AS1	ENSG00000226533.1	0	0
antisense	AL034345.2	ENSG00000231150.5	0	0
antisense	AL590999.1	ENSG00000235033.7	0	0
antisense	TDRG1	ENSG00000204091.7	0	0
antisense	AL035588.1	ENSG00000231102.1	0	0
antisense	AL035587.1	ENSG00000231113.2	0	1
antisense	AL355385.1	ENSG00000272170.1	0	0
antisense	AL133375.1	ENSG00000245261.1	0	0
antisense	AL355802.2	ENSG00000271754.1	0	0
antisense	POLH-AS1	ENSG00000203362.2	0	0
antisense	AL035701.1	ENSG00000231769.2	0	0
antisense	AL359633.2	ENSG00000236466.1	0	0
antisense	AL591242.1	ENSG00000242973.6	0	0
antisense	AL096772.1	ENSG00000225730.1	0	0
antisense	AL121974.1	ENSG00000235122.3	0	0
antisense	AL355997.1	ENSG00000228689.8	0	0
antisense	AL033397.2	ENSG00000249379.1	0	0
antisense	AL033384.2	ENSG00000271218.1	0	0
antisense	MLIP-AS1	ENSG00000235050.6	0	0
antisense	AL589946.1	ENSG00000228231.2	0	0
antisense	AL512422.1	ENSG00000231441.1	0	0
antisense	ZNF451-AS1	ENSG00000226803.8	0	1
antisense	AL354719.2	ENSG00000236345.1	0	0
antisense	AL357375.1	ENSG00000232120.1	0	0
antisense	AL160262.1	ENSG00000253809.1	0	0
antisense	KCNQ5-AS1	ENSG00000229154.1	0	0
antisense	OOEP-AS1	ENSG00000231332.2	0	0
antisense	AL603910.1	ENSG00000223821.1	0	0
antisense	AL121972.1	ENSG00000229862.5	0	0
antisense	AL445465.1	ENSG00000237174.7	0	0
antisense	AL355613.1	ENSG00000227215.1	0	0
antisense	TBX18-AS1	ENSG00000228290.6	0	0
antisense	AL139274.2	ENSG00000272008.1	0	0
antisense	AL353135.1	ENSG00000271931.1	0	0
antisense	AL159174.1	ENSG00000237027.1	0	0
antisense	AL096678.1	ENSG00000228124.1	0	0
antisense	AL121787.1	ENSG00000226455.1	0	0
antisense	UFL1-AS1	ENSG00000233797.1	0	0
antisense	AL033379.1	ENSG00000224384.1	0	0
antisense	AL137784.2	ENSG00000272017.1	0	1
antisense	MCHR2-AS1	ENSG00000229315.6	0	0

antisense	Z86062.2	ENSG00000228082.1	0	0
antisense	BVES-AS1	ENSG00000203808.11	0	1
antisense	AL133406.2	ENSG00000231628.1	0	0
antisense	AL356859.1	ENSG00000233941.1	0	0
antisense	AL109920.1	ENSG00000282408.1	0	0
antisense	AL121957.1	ENSG00000234206.5	0	0
antisense	ARMC2-AS1	ENSG00000230290.1	0	0
antisense	AL390208.1	ENSG00000271730.1	0	1
antisense	AL109947.1	ENSG00000223537.2	0	0
antisense	AC002464.1	ENSG00000260188.1	0	0
antisense	AL360227.1	ENSG00000231046.1	0	0
antisense	Z99289.1	ENSG00000237234.7	0	0
antisense	AL365214.2	ENSG00000226440.7	0	0
antisense	FLJ34503	ENSG00000175967.3	0	0
antisense	AL050331.2	ENSG00000236326.1	0	0
antisense	Z84488.1	ENSG00000244158.1	0	0
antisense	AL445224.1	ENSG00000234117.2	0	0
antisense	AL132671.1	ENSG00000226181.1	0	0
antisense	TRDN-AS1	ENSG00000235535.7	0	0
antisense	AL445259.1	ENSG00000285691.1	0	0
antisense	AL133257.1	ENSG00000285941.1	0	0
antisense	AL365259.1	ENSG00000237742.6	0	0
antisense	NCOA7-AS1	ENSG00000232131.1	0	0
antisense	AL590006.1	ENSG00000227945.1	0	0
antisense	AL034349.1	ENSG00000224733.1	0	0
antisense	AL356124.2	ENSG00000233351.1	0	2
antisense	AL355581.1	ENSG00000227678.7	0	0
antisense	AL024497.1	ENSG00000223542.1	0	0
antisense	AL445190.1	ENSG00000240056.2	0	0
antisense	AL353596.1	ENSG00000232876.1	0	0
antisense	MYB-AS1	ENSG00000236703.1	0	0
antisense	AL023693.1	ENSG00000224374.1	0	0
antisense	AL049552.1	ENSG00000234084.1	0	0
antisense	AL138828.1	ENSG00000237596.6	0	0
antisense	AL024508.1	ENSG00000234263.1	0	0
antisense	AL135902.1	ENSG00000235399.1	0	0
antisense	NMBR-AS1	ENSG00000236822.1	0	0
antisense	AL023584.1	ENSG00000233138.1	0	0
antisense	AL023581.1	ENSG00000225752.1	0	0
antisense	PHACTR2-	ENSG00000235740.1	0	0
antisense	AL024474.2	ENSG00000225311.1	0	0
antisense	AL513164.1	ENSG00000224658.1	0	0
antisense	UST-AS1	ENSG00000227660.1	0	0
antisense	AL357992.1	ENSG00000236591.1	0	0

antisense	AL078581.3	ENSG00000281021.1	0	0
antisense	AL355312.3	ENSG00000273132.1	0	0
antisense	RAET1E-AS1	ENSG00000268592.3	0	0
antisense	RAET1E-AS1	ENSG00000223701.3	0	0
antisense	AL133260.2	ENSG00000232290.1	0	0
antisense	AL138733.1	ENSG00000223598.1	0	0
antisense	AL356311.1	ENSG00000233823.1	0	0
antisense	SYNE1-AS1	ENSG00000234577.1	0	0
antisense	AL049548.1	ENSG00000226193.1	0	0
antisense	NANOGP11	ENSG00000233464.2	0	0
antisense	AL080276.2	ENSG00000227627.2	0	0
antisense	AL355297.4	ENSG00000271551.2	0	2
antisense	AL355297.2	ENSG00000270487.1	0	0
antisense	AL049820.1	ENSG00000233044.1	0	0
antisense	AL391863.2	ENSG00000234361.1	0	0
antisense	AL391863.1	ENSG00000229502.5	0	0
antisense	AL035634.1	ENSG00000236324.1	0	0
antisense	AL360169.1	ENSG00000238019.1	0	0
antisense	EZR-AS1	ENSG00000233893.2	0	0
antisense	AL035530.2	ENSG00000271913.6	0	1
antisense	AL356417.2	ENSG00000233682.3	0	13
antisense	AIRN	ENSG00000268257.2	0	0
antisense	AL139393.1	ENSG00000231863.1	0	0
antisense	AL590286.2	ENSG00000285610.1	0	0
antisense	PACRG-AS2	ENSG00000225437.5	0	0
antisense	PACRG-AS3	ENSG00000225683.5	0	0
antisense	PACRG-AS1	ENSG00000281692.1	0	0
antisense	AL590302.2	ENSG00000236627.1	0	0
antisense	AL022069.1	ENSG00000261420.1	0	0
antisense	RPS6KA2-	ENSG00000231654.1	0	0
antisense	AL354892.2	ENSG00000232640.1	0	0
antisense	AL354892.1	ENSG00000227704.1	0	0
antisense	AL596442.1	ENSG00000232197.1	0	0
antisense	AL109910.1	ENSG00000230960.1	0	0
antisense	AC093627.3	ENSG00000240093.1	0	0
antisense	AC147651.1	ENSG00000229380.1	0	0
antisense	AC073957.1	ENSG00000225146.1	0	0
antisense	AC073957.2	ENSG00000257607.1	0	0
antisense	AC091729.2	ENSG00000226291.1	0	0
antisense	AC102953.1	ENSG00000225981.1	0	0
antisense	AC093734.1	ENSG00000231927.1	0	0
antisense	AC004906.1	ENSG00000237286.1	0	0
antisense	AC073316.3	ENSG00000236708.1	0	0
antisense	AC011284.1	ENSG00000236510.1	0	0

antisense	AC017000.1	ENSG00000283991.1	0	0
antisense	AC093620.1	ENSG00000241269.1	0	0
antisense	AC092171.1	ENSG00000188365.3	0	0
antisense	AC006483.2	ENSG00000272719.1	0	0
antisense	AC079742.1	ENSG00000232581.1	0	0
antisense	AC073343.2	ENSG00000228010.5	0	0
antisense	AC006042.4	ENSG00000234141.1	0	0
antisense	AC006042.2	ENSG00000233108.1	0	0
antisense	AC006042.1	ENSG00000227719.1	0	0
antisense	AC007009.1	ENSG00000244239.1	0	0
antisense	AC007128.1	ENSG00000229970.3	0	0
antisense	AC004160.2	ENSG00000230435.1	0	0
antisense	AC005281.1	ENSG00000225606.1	0	0
antisense	AC006150.1	ENSG00000226323.1	0	0
antisense	AC005550.2	ENSG00000237070.1	0	0
antisense	ISPD-AS1	ENSG00000229688.7	0	1
antisense	AC073333.1	ENSG00000235837.1	0	0
antisense	AC010082.1	ENSG00000225000.1	0	0
antisense	AC004994.1	ENSG00000226522.1	0	0
antisense	AC003986.1	ENSG00000229533.1	0	0
antisense	MACC1-AS1	ENSG00000228598.1	0	0
antisense	AC002480.1	ENSG00000232759.1	0	1
antisense	AC002480.2	ENSG00000232949.1	0	0
antisense	AC073072.1	ENSG00000179428.2	0	0
antisense	AC005082.2	ENSG00000285926.1	0	0
antisense	AC006026.3	ENSG00000234286.1	0	0
antisense	AC004485.1	ENSG00000228944.1	0	0
antisense	AC004540.2	ENSG00000225792.1	0	0
antisense	HOXA-AS3	ENSG00000254369.6	0	0
antisense	HOXA10-AS	ENSG00000253187.2	0	0
antisense	HOXA11-AS	ENSG00000240990.10	0	0
antisense	HOTTIP	ENSG00000243766.8	0	0
antisense	EVX1-AS	ENSG00000253405.1	0	0
antisense	AC005091.1	ENSG00000229893.2	0	0
antisense	AC005162.2	ENSG00000233517.1	0	0
antisense	AC005162.1	ENSG00000229452.1	0	0
antisense	AC005232.1	ENSG00000285412.1	0	0
antisense	AC004593.1	ENSG00000235669.1	0	0
antisense	AC007255.1	ENSG00000223813.2	0	0
antisense	AC007285.1	ENSG00000227014.6	0	0
antisense	AC007285.2	ENSG00000231519.1	0	0
antisense	AC004691.1	ENSG00000229263.1	0	0
antisense	AC008080.2	ENSG00000227481.1	0	0
antisense	AC007327.2	ENSG00000229679.1	0	0

antisense	AC006960.3	ENSG00000260070.1	0	0
antisense	AC006960.2	ENSG00000237400.1	0	0
antisense	AC007349.2	ENSG00000230831.1	0	0
antisense	ELMO1-AS1	ENSG00000224101.1	0	0
antisense	POU6F2-AS2	ENSG00000233854.1	0	0
antisense	POU6F2-AS1	ENSG00000224122.1	0	0
antisense	AC011290.1	ENSG00000227172.2	0	0
antisense	AC004837.3	ENSG00000231951.1	0	0
antisense	AC004837.2	ENSG00000228554.1	0	0
antisense	AC004988.1	ENSG00000203446.2	0	0
antisense	AC004692.2	ENSG00000228680.1	0	0
antisense	AC013436.1	ENSG00000228596.1	0	0
antisense	LINC01952	ENSG00000234183.1	0	0
antisense	LINC00525	ENSG00000146666.5	0	0
antisense	C7orf69	ENSG00000136275.10	0	3
antisense	AC020743.1	ENSG00000228005.1	0	0
antisense	AC124014.1	ENSG00000285165.1	0	0
antisense	DDC-AS1	ENSG00000226122.1	0	0
antisense	AC004830.1	ENSG00000228204.2	0	0
antisense	AC074351.1	ENSG00000234707.2	0	0
antisense	EGFR-AS1	ENSG00000224057.1	0	0
antisense	AC099681.1	ENSG00000223475.1	0	0
antisense	AC073349.1	ENSG00000189316.3	0	0
antisense	AC068533.3	ENSG00000234185.2	0	0
antisense	ELN-AS1	ENSG00000232415.1	0	0
antisense	AC211486.5	ENSG00000277675.1	0	0
antisense	AC211486.2	ENSG00000263081.1	0	0
antisense	AC005522.1	ENSG00000250614.1	0	0
antisense	AC098851.1	ENSG00000250990.1	0	0
antisense	AC004990.1	ENSG00000232756.1	0	0
antisense	MAGI2-AS1	ENSG00000251276.1	0	0
antisense	MAGI2-AS2	ENSG00000226978.1	0	0
antisense	AC006355.2	ENSG00000281120.1	0	0
antisense	AC004862.1	ENSG00000232667.10	0	0
antisense	AC006145.1	ENSG00000223770.5	0	0
antisense	AC079799.1	ENSG00000235503.2	0	0
antisense	AC005009.1	ENSG00000231255.1	0	0
antisense	AC005009.2	ENSG00000233073.1	0	0
antisense	AC003991.2	ENSG00000254003.1	0	0
antisense	AC003991.1	ENSG00000228113.7	0	0
antisense	AC002064.2	ENSG00000234459.1	0	0
antisense	CYP51A1-	ENSG00000188693.7	0	0
antisense	AC007566.1	ENSG00000244055.1	0	4
antisense	AC006378.1	ENSG00000236861.6	0	0

antisense	AC002429.2	ENSG00000236197.3	0	1
antisense	AC005021.1	ENSG00000273138.1	0	0
antisense	AC002451.1	ENSG00000231170.5	0	0
antisense	AC004834.1	ENSG00000284523.1	0	0
antisense	AC069294.1	ENSG00000273407.1	0	0
antisense	AC004522.2	ENSG00000237640.1	0	0
antisense	AC073842.2	ENSG00000242798.1	0	0
antisense	AC073842.1	ENSG00000235077.1	0	0
antisense	AC092849.2	ENSG00000241357.1	0	0
antisense	AC092849.1	ENSG00000240211.1	0	0
antisense	AC254629.1	ENSG00000274993.1	0	0
antisense	AC005096.1	ENSG00000259294.1	0	0
antisense	AC005086.1	ENSG00000261535.1	0	0
antisense	AC073517.1	ENSG00000239480.1	0	0
antisense	AC073127.1	ENSG00000236226.1	0	0
antisense	AC005064.1	ENSG00000234715.1	0	0
antisense	KMT2E-AS1	ENSG00000239569.3	0	0
antisense	AC004884.2	ENSG00000242154.1	0	0
antisense	AC007032.1	ENSG00000273320.1	0	0
antisense	SLC26A4-	ENSG00000233705.6	0	0
antisense	DOCK4-AS1	ENSG00000225572.1	0	0
antisense	AC004112.1	ENSG00000226851.1	0	0
antisense	AC073626.1	ENSG00000224595.1	0	0
antisense	AC073130.2	ENSG00000243243.5	0	0
antisense	AC006159.1	ENSG00000235427.1	0	0
antisense	AC006159.2	ENSG00000243220.1	0	0
antisense	AC106873.1	ENSG00000228368.1	0	0
antisense	ST7-AS2	ENSG00000226367.5	0	0
antisense	AC002465.1	ENSG00000238202.1	0	0
antisense	CFTR-AS1	ENSG00000232661.1	0	0
antisense	AC000061.1	ENSG00000083622.8	0	0
antisense	AC004888.1	ENSG00000231295.1	0	0
antisense	FEZF1-AS1	ENSG00000230316.6	0	0
antisense	AC004594.1	ENSG00000240499.7	0	0
antisense	AC073323.1	ENSG00000232524.1	0	0
antisense	AC006333.1	ENSG00000230442.5	0	0
antisense	AC004690.2	ENSG00000241345.1	0	0
antisense	AC006148.1	ENSG00000242593.5	0	0
antisense	POT1-AS1	ENSG00000224897.6	0	0
antisense	AC000372.1	ENSG00000241921.1	0	0
antisense	AC000099.1	ENSG00000236340.5	0	0
antisense	AC000123.1	ENSG00000224138.1	0	0
antisense	AC073934.1	ENSG00000240790.1	0	0
antisense	FLNC-AS1	ENSG00000242902.1	0	0

antisense	AC011005.4	ENSG00000243230.1	0	0
antisense	AC073320.1	ENSG00000244036.3	0	0
antisense	AC087071.1	ENSG00000229196.3	0	0
antisense	AC087071.2	ENSG00000240571.1	0	0
antisense	MESTIT1	ENSG00000272701.3	0	0
antisense	AC007938.3	ENSG00000270953.1	0	0
antisense	AC008264.2	ENSG00000273489.1	0	0
antisense	AC018643.1	ENSG00000225144.2	0	0
antisense	AC011625.1	ENSG00000223436.1	0	0
antisense	AC009365.1	ENSG00000225881.1	0	0
antisense	AC009365.2	ENSG00000227197.1	0	0
antisense	AC008154.2	ENSG00000231098.1	0	0
antisense	AC009542.1	ENSG00000231794.5	0	0
antisense	AC091736.1	ENSG00000273219.1	0	0
antisense	AC015987.1	ENSG00000224746.1	0	0
antisense	AC083880.1	ENSG00000273391.1	0	0
antisense	AC005692.2	ENSG00000261629.1	0	0
antisense	AC073878.1	ENSG00000261570.1	0	0
antisense	AC245427.1	ENSG00000224970.2	0	0
antisense	AC073342.2	ENSG00000268170.2	0	0
antisense	AC073342.1	ENSG00000231840.1	0	0
antisense	EPHA1-AS1	ENSG00000229153.5	0	0
antisense	AC004889.1	ENSG00000244198.6	0	0
antisense	AC074386.1	ENSG00000284644.1	0	0
antisense	OR2A1-AS1	ENSG00000244479.7	0	0
antisense	AC006004.1	ENSG00000236795.1	0	0
antisense	AC004941.1	ENSG00000228151.1	0	0
antisense	ATP6V0E2-	ENSG00000204934.10	0	0
antisense	AC005586.1	ENSG00000240449.1	0	0
antisense	AC073111.1	ENSG00000239377.1	0	0
antisense	AC010973.1	ENSG00000243433.1	0	0
antisense	AC010973.2	ENSG00000244151.1	0	0
antisense	AC005486.1	ENSG00000243018.1	0	0
antisense	AC005996.1	ENSG00000241456.1	0	0
antisense	AC074257.1	ENSG00000227562.1	0	0
antisense	AC006017.1	ENSG00000229591.1	0	0
antisense	AC006019.3	ENSG00000270114.1	0	0
antisense	AC006019.1	ENSG00000203335.4	0	0
antisense	AC006019.2	ENSG00000233363.1	0	0
antisense	AC073336.1	ENSG00000236408.1	0	0
antisense	AC093726.1	ENSG00000272760.1	0	0
antisense	HTR5A-AS1	ENSG00000220575.7	0	0
antisense	AC008060.2	ENSG00000227365.1	0	0
antisense	MNX1-AS2	ENSG00000235029.1	0	0

antisense	AC005481.1	ENSG00000222012.1	0	0
antisense	AC011899.2	ENSG00000233038.6	0	0
antisense	AC011899.3	ENSG00000272839.1	0	0
antisense	AC011899.4	ENSG00000285821.1	0	0
antisense	AC011899.1	ENSG00000231980.1	0	0
antisense	AC078942.1	ENSG00000225365.1	0	0
antisense	LINC01022	ENSG00000232715.1	0	0
antisense	AC004908.1	ENSG00000272240.1	0	0
antisense	DLGAP2-AS1	ENSG00000253267.5	0	0
antisense	AC019257.7	ENSG00000285957.1	0	0
antisense	AC026991.2	ENSG00000285861.1	0	0
antisense	AC022068.1	ENSG00000285977.1	0	0
antisense	MCPH1-AS1	ENSG00000249898.7	0	0
antisense	AF233439.1	ENSG00000254625.1	0	0
antisense	FAM85B	ENSG00000253893.2	0	0
antisense	AC022784.5	ENSG00000254340.1	0	0
antisense	AC023385.1	ENSG00000285675.1	0	0
antisense	AC079200.1	ENSG00000285606.1	0	0
antisense	AC105001.1	ENSG00000248896.2	0	0
antisense	AC011008.1	ENSG00000253695.1	0	0
antisense	AF131216.1	ENSG00000246477.3	0	0
antisense	AF131216.3	ENSG00000255020.1	0	0
antisense	FAM167A-	ENSG00000184608.8	0	0
antisense	AC022239.1	ENSG00000269954.2	0	0
antisense	FAM66D	ENSG00000255052.4	0	0
antisense	AC145124.1	ENSG00000255495.1	0	0
antisense	AC019270.1	ENSG00000253932.1	0	0
antisense	AC124069.1	ENSG00000253168.1	0	0
antisense	AC027117.2	ENSG00000253944.1	0	0
antisense	AC087273.1	ENSG00000253215.1	0	0
antisense	AC100800.1	ENSG00000187229.3	0	0
antisense	AC009884.1	ENSG00000253335.1	0	0
antisense	AC105206.3	ENSG00000285881.1	0	0
antisense	AC105206.2	ENSG00000254064.1	0	0
antisense	AC037459.4	ENSG00000254230.1	0	0
antisense	AC037441.1	ENSG00000248738.6	0	0
antisense	AC107959.1	ENSG00000245025.2	0	0
antisense	AC107959.2	ENSG00000246130.1	0	0
antisense	AC107959.3	ENSG00000253616.5	0	0
antisense	TNFRSF10A-AS1	ENSG00000253930.1	0	0
antisense	AC100861.2	ENSG00000250714.3	0	0
antisense	AC120193.1	ENSG00000253535.5	0	0

antisense	AC024958.1	ENSG00000253643.5	0	0
antisense	AC090150.1	ENSG00000253888.1	0	0
antisense	AC013643.2	ENSG00000253875.1	0	0
antisense	AC069113.2	ENSG00000253397.1	0	0
antisense	AC021678.2	ENSG00000253690.1	0	0
antisense	AC025871.1	ENSG00000253567.1	0	0
antisense	INTS9-AS1	ENSG00000254034.2	0	0
antisense	AC108449.3	ENSG00000259607.1	0	0
antisense	AC108449.1	ENSG00000254129.1	0	0
antisense	AC026979.1	ENSG00000253708.1	0	0
antisense	AC102945.1	ENSG00000253112.1	0	0
antisense	AC009563.1	ENSG00000253961.1	0	0
antisense	AC137579.1	ENSG00000253344.1	0	0
antisense	AC138356.3	ENSG00000253181.1	0	0
antisense	AC138356.1	ENSG00000183154.1	0	0
antisense	AC084024.3	ENSG00000253356.1	0	0
antisense	AC087362.2	ENSG00000255487.1	0	0
antisense	AC087362.1	ENSG00000254898.1	0	0
antisense	AC087623.3	ENSG00000272159.1	0	1
antisense	AC087623.1	ENSG00000255201.1	0	0
antisense	AC067817.2	ENSG00000253829.1	0	0
antisense	AC108863.1	ENSG00000253645.1	0	0
antisense	AC007991.3	ENSG00000253939.1	0	0
antisense	AC007991.4	ENSG00000254287.1	0	0
antisense	AC009630.2	ENSG00000253174.2	0	0
antisense	AC009630.3	ENSG00000264578.1	0	0
antisense	AC113133.1	ENSG00000253389.2	0	0
antisense	AC103724.3	ENSG00000261449.1	0	0
antisense	AC103843.1	ENSG00000255101.1	0	0
antisense	AC022915.2	ENSG00000253608.1	0	0
antisense	AC012413.1	ENSG00000253664.1	0	0
antisense	AC021915.2	ENSG00000254314.1	0	0
antisense	AC021915.1	ENSG00000253551.1	0	0
antisense	AC009646.2	ENSG00000254687.1	0	0
antisense	AC113194.1	ENSG00000272457.1	0	0
antisense	AC090200.1	ENSG00000254357.1	0	0
antisense	CERNA3	ENSG00000253603.1	0	0
antisense	AC012349.1	ENSG00000254254.5	0	0
antisense	AC113143.1	ENSG00000254432.1	0	0
antisense	AC023866.2	ENSG00000254222.1	0	0
antisense	AC023866.1	ENSG00000253711.1	0	0
antisense	AC018861.2	ENSG00000254050.1	0	0
antisense	AC120042.1	ENSG00000240915.2	0	0
antisense	AC120042.2	ENSG00000253121.1	0	0

antisense	AC090136.3	ENSG00000254102.1	0	0
antisense	AC022874.1	ENSG00000255130.1	0	0
antisense	AC011853.2	ENSG00000255206.1	0	0
antisense	C8orf34-AS1	ENSG00000248801.6	0	0
antisense	AC091047.1	ENSG00000254557.1	0	0
antisense	AC079089.1	ENSG00000246528.3	0	0
antisense	LACTB2-AS1	ENSG00000246366.6	0	0
antisense	AC022858.1	ENSG00000254031.5	0	0
antisense	AC022893.3	ENSG00000272254.1	0	0
antisense	RDH10-AS1	ENSG00000250295.6	0	0
antisense	STAU2-AS1	ENSG00000253302.1	0	1
antisense	AC087627.1	ENSG00000253983.2	0	0
antisense	AC103952.1	ENSG00000253596.1	0	0
antisense	AC115837.2	ENSG00000254080.1	0	0
antisense	AC100782.1	ENSG00000254238.1	0	0
antisense	AC036214.4	ENSG00000285758.1	0	0
antisense	AC009686.1	ENSG00000254205.1	0	0
antisense	AC018616.1	ENSG00000253859.2	0	0
antisense	AC023644.1	ENSG00000253374.5	0	0
antisense	AC132219.1	ENSG00000253334.1	0	0
antisense	AC011773.4	ENSG00000260493.1	0	0
antisense	AC011773.1	ENSG00000254208.1	0	0
antisense	AC011773.3	ENSG00000258256.1	0	0
antisense	AC011773.2	ENSG00000257962.1	0	0
antisense	AC023194.3	ENSG00000253675.1	0	0
antisense	AC084128.1	ENSG00000253699.1	0	0
antisense	AC090572.3	ENSG00000254115.1	0	0
antisense	AF121898.1	ENSG00000253500.5	0	0
antisense	AC037450.1	ENSG00000253171.1	0	0
antisense	AC090578.1	ENSG00000253553.6	0	0
antisense	AC106038.1	ENSG00000246792.2	0	1
antisense	AC103770.1	ENSG00000254251.1	0	0
antisense	AC087439.1	ENSG00000253358.1	0	0
antisense	AF181450.1	ENSG00000253576.1	0	0
antisense	AC117834.1	ENSG00000253197.5	0	0
antisense	LINC00535	ENSG00000246662.6	0	1
antisense	AC010834.3	ENSG00000253854.1	0	0
antisense	AC010834.1	ENSG00000253722.1	0	0
antisense	AC010834.2	ENSG00000253848.1	0	2
antisense	AC023632.5	ENSG00000254315.1	0	0
antisense	AP003692.1	ENSG00000254307.2	0	0
antisense	MIR3150BHG	ENSG00000245080.7	0	0
antisense	AC068189.1	ENSG00000254248.1	0	0
antisense	AP003465.1	ENSG00000254224.1	0	0

antisense	AP003117.2	ENSG00000272249.1	0	0
antisense	AP003352.1	ENSG00000245970.2	0	1
antisense	AP003355.2	ENSG00000272321.1	0	0
antisense	AP003467.1	ENSG00000253911.1	0	0
antisense	AC107909.2	ENSG00000253562.1	0	0
antisense	AC018442.2	ENSG00000253539.1	0	0
antisense	AP000424.1	ENSG00000253666.1	0	0
antisense	AP000424.2	ENSG00000254364.1	0	0
antisense	AP001205.1	ENSG00000253740.5	0	0
antisense	AP000426.1	ENSG00000253629.1	0	2
antisense	UBR5-AS1	ENSG00000246263.2	0	5
antisense	AP002907.1	ENSG00000272037.1	0	0
antisense	AP003550.1	ENSG00000254236.1	0	0
antisense	LINC01181	ENSG00000250929.2	0	0
antisense	BAALC-AS2	ENSG00000236939.2	0	0
antisense	AC025370.1	ENSG00000253851.1	0	0
antisense	AC012213.1	ENSG00000253477.5	0	0
antisense	AC012564.1	ENSG00000283157.1	0	0
antisense	AC021546.1	ENSG00000254041.1	0	0
antisense	AC103853.1	ENSG00000253420.1	0	0
antisense	AC087620.1	ENSG00000253754.1	0	0
antisense	AC104248.1	ENSG00000253796.1	0	0
antisense	AC021237.1	ENSG00000250267.2	0	0
antisense	AC079061.1	ENSG00000248050.1	0	0
antisense	AC027451.1	ENSG00000255402.1	0	0
antisense	AF178030.1	ENSG00000227170.1	0	0
antisense	AC023590.1	ENSG00000225885.7	0	0
antisense	AC107953.2	ENSG00000254278.1	0	0
antisense	AC021733.1	ENSG00000253398.1	0	0
antisense	AC104958.1	ENSG00000248318.1	0	0
antisense	AC104316.1	ENSG00000253607.1	0	0
antisense	AC068228.1	ENSG00000253258.1	0	0
antisense	FAM83A-	ENSG00000204949.8	0	0
antisense	AC090193.1	ENSG00000253286.5	0	0
antisense	FER1L6-AS1	ENSG00000181171.5	0	0
antisense	AC100871.2	ENSG00000254249.1	0	0
antisense	FER1L6-AS2	ENSG00000253868.3	0	0
antisense	AC009908.1	ENSG00000253513.1	0	0
antisense	WASHC5-	ENSG00000253167.1	0	0
antisense	AC084083.1	ENSG00000254431.1	0	0
antisense	AC022973.4	ENSG00000254317.1	0	0
antisense	AC103726.2	ENSG00000253992.1	0	0
antisense	AF230666.1	ENSG00000223697.3	0	0
antisense	ZFAT-AS1	ENSG00000248492.1	0	0

antisense	AC040914.1	ENSG00000254372.1	0	0
antisense	AC021744.1	ENSG00000253574.5	0	0
antisense	AC011676.1	ENSG00000253307.1	0	0
antisense	AC011676.2	ENSG00000254019.1	0	0
antisense	AC011676.4	ENSG00000254291.1	0	0
antisense	LINC01300	ENSG00000253595.5	0	0
antisense	AC100803.1	ENSG00000244998.1	0	0
antisense	AC100803.3	ENSG00000271959.1	0	0
antisense	LINC00051	ENSG00000254008.1	0	0
antisense	AC134682.1	ENSG00000261693.1	0	0
antisense	AP006547.1	ENSG00000261044.1	0	0
antisense	AC108002.2	ENSG00000277332.1	0	0
antisense	LNCOC1	ENSG00000253741.1	0	0
antisense	AC108002.1	ENSG00000253806.1	0	0
antisense	AC083841.1	ENSG00000253196.1	0	0
antisense	AC083841.2	ENSG00000253715.1	0	0
antisense	AC083841.3	ENSG00000253728.5	0	0
antisense	RHPN1-AS1	ENSG00000254389.3	0	0
antisense	AC105118.1	ENSG00000253931.1	0	0
antisense	AC067930.1	ENSG00000254144.3	0	0
antisense	AC067930.5	ENSG00000255050.1	0	0
antisense	AC067930.2	ENSG00000254741.1	0	0
antisense	AC067930.3	ENSG00000254812.1	0	0
antisense	AC105219.2	ENSG00000254574.1	0	0
antisense	AC105219.1	ENSG00000254548.1	0	0
antisense	AC234917.2	ENSG00000285599.1	0	0
antisense	AC233992.1	ENSG00000254690.1	0	0
antisense	TONSL-AS1	ENSG00000232600.3	0	0
antisense	AC084125.1	ENSG00000254578.1	0	0
antisense	AC084125.3	ENSG00000255456.1	0	0
antisense	AF235103.1	ENSG00000255164.1	0	0
antisense	ZNF252P-	ENSG00000255559.1	0	0
antisense	AL928970.1	ENSG00000227518.5	0	0
antisense	AL158832.1	ENSG00000235880.1	0	0
antisense	AL161725.1	ENSG00000227155.7	0	0
antisense	AL136979.1	ENSG00000227914.3	0	0
antisense	AL359076.1	ENSG00000236199.1	0	0
antisense	AL137071.1	ENSG00000236724.1	0	0
antisense	GLIS3-AS1	ENSG00000237009.2	0	0
antisense	AL162419.1	ENSG00000228322.1	0	0
antisense	AL158147.1	ENSG00000228165.1	0	0
antisense	AL136980.1	ENSG00000225408.1	0	0
antisense	AL133480.1	ENSG00000233367.1	0	0
antisense	LURAP1L-	ENSG00000235448.2	0	0

antisense	AL136366.1	ENSG00000225472.1	0	0
antisense	AL512643.1	ENSG00000283647.1	0	0
antisense	AL449983.1	ENSG00000231756.1	0	0
antisense	AL450003.1	ENSG00000230694.1	0	0
antisense	AL161909.2	ENSG00000232978.2	0	0
antisense	AL359922.2	ENSG00000265194.1	0	0
antisense	CDKN2A-DT	ENSG00000224854.3	0	0
antisense	IFT74-AS1	ENSG00000234676.1	0	0
antisense	AL163192.1	ENSG00000237734.1	0	0
antisense	AL451123.1	ENSG00000285103.2	0	0
antisense	AL589642.1	ENSG00000223440.1	0	0
antisense	AL356218.1	ENSG00000223678.1	0	0
antisense	AL139008.2	ENSG00000228072.1	0	0
antisense	PTENP1-AS	ENSG00000281128.1	0	0
antisense	LINC01251	ENSG00000233776.5	0	0
antisense	UBE2R2-AS1	ENSG00000235481.2	0	0
antisense	AL354989.1	ENSG00000228352.2	0	0
antisense	AL160270.1	ENSG00000230729.1	0	0
antisense	CNTFR-AS1	ENSG00000237159.5	0	0
antisense	AL353795.2	ENSG00000234181.1	0	0
antisense	AL357874.2	ENSG00000231393.1	0	0
antisense	AL357874.1	ENSG00000227933.1	0	0
antisense	AL133410.2	ENSG00000228843.2	0	0
antisense	AL450267.1	ENSG00000258885.1	0	0
antisense	AL161781.2	ENSG00000250850.2	0	0
antisense	AL512604.2	ENSG00000233242.2	0	0
antisense	BX005040.3	ENSG00000277412.1	0	0
antisense	AL353608.2	ENSG00000226904.1	0	0
antisense	PGM5-AS1	ENSG00000224958.5	0	0
antisense	AL161457.2	ENSG00000233178.7	0	0
antisense	AL354794.1	ENSG00000236733.1	0	0
antisense	AL353693.1	ENSG00000229312.2	0	0
antisense	AL159990.2	ENSG00000232086.1	0	0
antisense	AL159990.1	ENSG00000223966.1	0	0
antisense	RORB-AS1	ENSG00000224825.2	0	0
antisense	C9orf41-AS1	ENSG00000203321.2	0	0
antisense	PCA3	ENSG00000225937.2	0	0
antisense	VPS13A-AS1	ENSG00000232998.2	0	0
antisense	GNA14-AS1	ENSG00000231373.1	0	0
antisense	AL137847.1	ENSG00000229109.1	0	0
antisense	AL137847.2	ENSG00000237529.2	0	0
antisense	AL354733.1	ENSG00000226877.8	0	0
antisense	AL354733.2	ENSG00000231616.8	0	0
antisense	AL354733.3	ENSG00000235298.1	0	0

antisense	AL356134.1	ENSG00000233262.1	0	0
antisense	AL157886.1	ENSG00000285987.1	0	0
antisense	AL772337.2	ENSG00000233884.1	0	0
antisense	AL136097.2	ENSG00000236115.1	0	0
antisense	AL157827.2	ENSG00000285650.1	0	0
antisense	AL451065.1	ENSG00000223446.1	0	0
antisense	AL583839.1	ENSG00000237385.1	0	0
antisense	AL353768.2	ENSG00000285807.1	0	0
antisense	AL161729.1	ENSG00000271155.1	0	0
antisense	HSD17B3-	ENSG00000232283.1	0	0
antisense	AL162385.1	ENSG00000228174.7	0	0
antisense	AL162385.2	ENSG00000231521.1	0	0
antisense	AL354726.1	ENSG00000236896.1	0	0
antisense	AL136084.2	ENSG00000267026.5	0	0
antisense	AL136084.3	ENSG00000270412.1	0	0
antisense	TMEM246-	ENSG00000225376.5	0	0
antisense	AL450426.1	ENSG00000283001.1	0	0
antisense	AL359182.1	ENSG00000226334.1	0	0
antisense	AL158070.1	ENSG00000228317.1	0	0
antisense	AL807761.4	ENSG00000230782.1	0	0
antisense	AL158829.1	ENSG00000232939.1	0	0
antisense	AL135787.1	ENSG00000224644.1	0	0
antisense	C9orf147	ENSG00000230185.4	0	0
antisense	AL390067.1	ENSG00000226609.1	0	0
antisense	AL139041.1	ENSG00000271631.1	0	0
antisense	AL162727.2	ENSG00000237073.1	0	0
antisense	AL691420.1	ENSG00000228714.3	0	0
antisense	PAPPA-AS2	ENSG00000226604.2	0	0
antisense	AL137024.1	ENSG00000244757.1	0	0
antisense	PAPPA-AS1	ENSG00000256040.2	0	0
antisense	ASTN2-AS1	ENSG00000229105.1	0	0
antisense	AL133284.1	ENSG00000230894.1	0	0
antisense	AL513122.2	ENSG00000239593.1	0	0
antisense	GSN-AS1	ENSG00000235865.2	0	0
antisense	AL359644.1	ENSG00000227355.2	0	0
antisense	AL357936.1	ENSG00000229854.1	0	0
antisense	AL359636.1	ENSG00000229245.2	0	0
antisense	AL359636.2	ENSG00000231465.2	0	0
antisense	AL445489.1	ENSG00000237336.1	0	0
antisense	AL390774.2	ENSG00000230826.1	0	0
antisense	AC006450.3	ENSG00000236668.2	0	0
antisense	AC006450.2	ENSG00000234921.1	0	0
antisense	AL162724.2	ENSG00000235204.1	0	0
antisense	AL354928.1	ENSG00000236643.1	0	0

antisense	AL354710.2	ENSG00000239705.1	0	0
antisense	AL162584.1	ENSG00000227068.1	0	0
antisense	AL450263.1	ENSG00000228487.2	0	0
antisense	AL157935.1	ENSG00000227218.7	0	0
antisense	AL360268.2	ENSG00000230848.1	0	0
antisense	AL360268.1	ENSG00000230536.1	0	0
antisense	ODF2-AS1	ENSG00000225951.1	0	0
antisense	AL356481.3	ENSG00000280758.1	0	0
antisense	AL158151.2	ENSG00000234055.1	0	0
antisense	AL590369.1	ENSG00000234789.1	0	0
antisense	PRRX2-AS1	ENSG00000236024.1	0	0
antisense	AL158207.2	ENSG00000230684.1	0	0
antisense	AL161733.1	ENSG00000236658.1	0	0
antisense	AL157938.2	ENSG00000236986.6	0	1
antisense	AL157938.3	ENSG00000246851.1	0	0
antisense	AL358781.1	ENSG00000176868.2	0	0
antisense	AL358781.2	ENSG00000230289.1	0	0
antisense	AC002101.1	ENSG00000261018.1	0	0
antisense	DBH-AS1	ENSG00000225756.1	0	0
antisense	AL445931.1	ENSG00000235138.1	0	0
antisense	BX649632.1	ENSG00000273249.1	0	1
antisense	COL5A1-AS1	ENSG00000204011.4	0	0
antisense	AL161452.1	ENSG00000226706.1	0	0
antisense	AL355574.1	ENSG00000238058.1	0	0
antisense	CR392000.2	ENSG00000283769.1	0	0
antisense	AL592301.1	ENSG00000227512.1	0	0
antisense	HSPC324	ENSG00000228401.4	0	0
antisense	CCDC183-	ENSG00000228544.1	0	0
antisense	C9orf139	ENSG00000180539.7	0	0
antisense	AL807752.4	ENSG00000236394.2	0	0
antisense	AL807752.2	ENSG00000229257.2	0	0
antisense	AL807752.3	ENSG00000231864.2	0	0
antisense	STPG3-AS1	ENSG00000275549.1	0	0
antisense	AL772363.1	ENSG00000203987.2	0	0
antisense	AL669841.1	ENSG00000233021.2	0	0
antisense	AL359878.2	ENSG00000229869.1	0	0
antisense	IDI2-AS1	ENSG00000232656.7	0	0
antisense	ADARB2-	ENSG00000205696.4	0	0
antisense	AL451164.3	ENSG00000278419.1	0	0
antisense	PITRM1-AS1	ENSG00000237399.7	0	0
antisense	AL450322.2	ENSG00000229672.2	0	0
antisense	AL450322.1	ENSG00000228353.1	0	0
antisense	AL391427.1	ENSG00000224251.6	0	0
antisense	CALML3-	ENSG00000205488.8	0	0

antisense	AL732437.1	ENSG00000256462.1	0	0
antisense	AL365356.1	ENSG00000226647.2	0	0
antisense	AL137186.1	ENSG00000229664.1	0	0
antisense	AL445070.1	ENSG00000230121.1	0	0
antisense	AL136369.1	ENSG00000228027.1	0	0
antisense	CELF2-AS2	ENSG00000237986.3	0	0
antisense	AL136320.1	ENSG00000230322.1	0	0
antisense	CELF2-AS1	ENSG00000181800.5	0	0
antisense	AL512631.2	ENSG00000271360.1	0	0
antisense	PROSER2-	ENSG00000225778.5	0	0
antisense	AL512770.1	ENSG00000228302.2	0	0
antisense	AL353586.1	ENSG00000285520.1	0	0
antisense	AL355870.1	ENSG00000233256.1	0	0
antisense	AL590677.1	ENSG00000227175.1	0	0
antisense	AL157392.1	ENSG00000225112.1	0	0
antisense	AL157392.2	ENSG00000234091.1	0	0
antisense	AC044781.1	ENSG00000229751.1	0	0
antisense	AL157896.1	ENSG00000235410.1	0	0
antisense	AL158168.1	ENSG00000236495.2	0	0
antisense	AL607028.1	ENSG00000232739.1	0	0
antisense	AL133415.1	ENSG00000234961.1	0	0
antisense	ST8SIA6-AS1	ENSG00000204832.9	0	0
antisense	STAM-AS1	ENSG00000260589.1	0	0
antisense	AC069542.1	ENSG00000229190.1	0	0
antisense	AC069023.1	ENSG00000235637.1	0	0
antisense	SLC39A12-	ENSG00000226083.5	0	0
antisense	AL450384.1	ENSG00000225527.1	0	0
antisense	AL590378.1	ENSG00000227734.1	0	0
antisense	AL157895.1	ENSG00000233968.6	0	0
antisense	AC069549.1	ENSG00000238246.1	0	0
antisense	NEBL-AS1	ENSG00000231920.1	0	0
antisense	GPR158-AS1	ENSG00000233642.1	0	0
antisense	AL358612.1	ENSG00000226304.1	0	0
antisense	AL390961.2	ENSG00000235843.1	0	0
antisense	AL353796.2	ENSG00000285824.1	0	0
antisense	AL158834.2	ENSG00000227253.3	0	0
antisense	AL158834.1	ENSG00000226842.1	0	0
antisense	AL391839.2	ENSG00000233825.1	0	0
antisense	AL391839.1	ENSG00000229327.1	0	0
antisense	AL121748.2	ENSG00000238258.1	0	0
antisense	AL121749.1	ENSG00000273312.2	0	1
antisense	AL135791.1	ENSG00000236514.1	0	1
antisense	ZNF32-AS3	ENSG00000223910.1	0	0
antisense	ZNF32-AS1	ENSG00000226245.1	0	0

antisense	ZNF32-AS2	ENSG00000230565.1	0	0
antisense	TMEM72-	ENSG00000224812.2	0	0
antisense	AL353801.2	ENSG00000234504.2	0	0
antisense	AL731567.1	ENSG00000231964.1	0	0
antisense	AL356056.2	ENSG00000231187.2	0	0
antisense	AL591684.1	ENSG00000224919.1	0	0
antisense	AC074325.1	ENSG00000285786.1	0	0
antisense	AC060234.1	ENSG00000226576.1	0	0
antisense	FAM170B-	ENSG00000234736.5	0	0
antisense	C10orf71-AS1	ENSG00000236208.1	0	0
antisense	AL138760.1	ENSG00000235939.1	0	0
antisense	AL442003.1	ENSG00000285803.1	0	0
antisense	AL117341.1	ENSG00000225303.2	0	0
antisense	AC022537.1	ENSG00000231132.1	0	0
antisense	PRKG1-AS1	ENSG00000236671.8	0	0
antisense	AL353784.1	ENSG00000234173.1	0	0
antisense	AC025038.1	ENSG00000236556.1	0	0
antisense	AL592430.1	ENSG00000232682.2	0	0
antisense	AL592430.2	ENSG00000282121.1	0	0
antisense	TMEM26-	ENSG00000237233.2	0	0
antisense	AL451049.1	ENSG00000272592.1	0	0
antisense	AC067751.1	ENSG00000238280.2	0	0
antisense	AL590502.1	ENSG00000224301.1	0	0
antisense	AC022017.1	ENSG00000273360.1	0	0
antisense	AL139240.1	ENSG00000225299.1	0	0
antisense	AL596223.1	ENSG00000229261.1	0	0
antisense	AL596223.2	ENSG00000231748.1	0	0
antisense	AL450311.2	ENSG00000236154.1	0	0
antisense	CDH23-AS1	ENSG00000223817.1	0	0
antisense	DDIT4-AS1	ENSG00000269926.1	0	0
antisense	AL513185.1	ENSG00000226163.1	0	0
antisense	AL731563.2	ENSG00000237768.2	0	0
antisense	DNAJC9-AS1	ENSG00000236756.4	0	0
antisense	AL512656.1	ENSG00000233144.1	0	0
antisense	AC073389.1	ENSG00000268584.1	0	0
antisense	AC073389.2	ENSG00000271848.1	0	0
antisense	ZSWIM8-AS1	ENSG00000272589.1	0	0
antisense	AC022400.1	ENSG00000224195.1	0	0
antisense	AC022400.2	ENSG00000229990.3	0	0
antisense	AC022540.1	ENSG00000232342.7	0	0
antisense	AC018511.2	ENSG00000234149.1	0	0
antisense	AC018511.1	ENSG00000227186.1	0	0
antisense	AC018511.5	ENSG00000285810.1	0	0
antisense	AC010997.2	ENSG00000236842.1	0	0

antisense	AC013286.1	ENSG00000230575.1	0	0
antisense	AC024603.1	ENSG00000269256.1	0	0
antisense	KCNMA1-	ENSG00000236467.8	0	0
antisense	KCNMA1-	ENSG00000225497.5	0	0
antisense	AL731575.1	ENSG00000224500.1	0	0
antisense	KCNMA1-	ENSG00000225652.1	0	0
antisense	AL391421.1	ENSG00000204049.1	0	0
antisense	AL133481.1	ENSG00000235426.2	0	0
antisense	SFTPD-AS1	ENSG00000273372.1	0	0
antisense	TMEM254-	ENSG00000230091.6	0	0
antisense	AC021028.1	ENSG00000226659.1	0	0
antisense	AC010157.2	ENSG00000285739.1	0	0
antisense	AC010157.1	ENSG00000229458.2	0	0
antisense	AL603756.1	ENSG00000271933.1	0	2
antisense	GRID1-AS1	ENSG00000234942.2	0	0
antisense	AC022028.2	ENSG00000270002.1	0	0
antisense	AL136982.7	ENSG00000273413.1	0	0
antisense	AL136982.2	ENSG00000229969.1	0	0
antisense	AL138767.1	ENSG00000196566.2	0	0
antisense	ACTA2-AS1	ENSG00000180139.11	0	0
antisense	AL353751.1	ENSG00000232110.7	0	0
antisense	SLC16A12-	ENSG00000234452.5	0	0
antisense	TNKS2-AS1	ENSG00000228701.1	0	0
antisense	AL359198.1	ENSG00000272817.1	0	0
antisense	AL358613.1	ENSG00000226425.1	0	0
antisense	AL358154.1	ENSG00000280485.1	0	0
antisense	PLCE1-AS2	ENSG00000232913.8	0	0
antisense	AL389885.1	ENSG00000228553.1	0	0
antisense	AL157834.2	ENSG00000234026.1	0	0
antisense	AL136181.1	ENSG00000229418.2	0	0
antisense	SLIT1-AS1	ENSG00000234855.1	0	0
antisense	AL355490.2	ENSG00000231970.1	0	0
antisense	AL139241.1	ENSG00000230928.1	0	0
antisense	DNMBP-AS1	ENSG00000227695.5	0	0
antisense	AL138921.2	ENSG00000236308.1	0	0
antisense	AL133215.1	ENSG00000236662.1	0	0
antisense	KCNIP2-AS1	ENSG00000226009.1	0	0
antisense	AL121928.1	ENSG00000273262.1	0	0
antisense	AL358790.1	ENSG00000282772.1	0	0
antisense	AL139339.1	ENSG00000234699.1	0	0
antisense	NEURL1-AS1	ENSG00000235470.5	0	0
antisense	AL121929.2	ENSG00000273108.1	0	0
antisense	AL162742.2	ENSG00000270075.1	0	0
antisense	CFAP58-DT	ENSG00000231233.1	0	0

antisense	SORCS3-AS1	ENSG00000226387.2	0	0
antisense	AL360182.2	ENSG00000228417.1	0	1
antisense	AL158163.1	ENSG00000270589.1	0	0
antisense	AL157786.1	ENSG00000232934.7	0	0
antisense	AL139120.1	ENSG00000233340.1	0	0
antisense	AL158212.5	ENSG00000285676.1	0	0
antisense	AL592546.1	ENSG00000234393.1	0	0
antisense	AC005383.1	ENSG00000275024.1	0	0
antisense	AC016042.1	ENSG00000285582.1	0	0
antisense	AC016825.1	ENSG00000232767.1	0	0
antisense	AL731557.1	ENSG00000225936.1	0	0
antisense	EMX2OS	ENSG00000229847.8	0	0
antisense	AC022395.1	ENSG00000231104.8	0	0
antisense	AL157788.1	ENSG00000229272.1	0	0
antisense	AL583824.1	ENSG00000236426.5	0	0
antisense	WDR11-AS1	ENSG00000227165.8	0	0
antisense	AC010998.2	ENSG00000271670.1	0	0
antisense	AC025947.1	ENSG00000224250.1	0	0
antisense	AL731566.1	ENSG00000273891.1	0	0
antisense	AC063960.1	ENSG00000285715.1	0	0
antisense	AC063960.2	ENSG00000285973.1	0	1
antisense	BX842242.1	ENSG00000285955.1	0	0
antisense	AC009987.1	ENSG00000231138.1	0	0
antisense	AL513190.1	ENSG00000278831.1	0	0
antisense	FAM53B-AS1	ENSG00000233334.3	0	0
antisense	AL731577.1	ENSG00000226899.1	0	0
antisense	AL731571.1	ENSG00000273599.1	0	1
antisense	TEX36-AS1	ENSG00000237675.5	0	0
antisense	AL158835.3	ENSG00000280561.1	0	0
antisense	EDRF1-AS1	ENSG00000236991.6	0	0
antisense	FANK1-AS1	ENSG00000233409.1	0	0
antisense	AL359094.1	ENSG00000223528.7	0	5
antisense	AL359094.2	ENSG00000232935.2	0	0
antisense	AL157832.2	ENSG00000237224.4	0	0
antisense	AL354950.2	ENSG00000275327.1	0	0
antisense	AL354950.1	ENSG00000275005.1	0	0
antisense	TCERG1L-	ENSG00000230098.1	0	0
antisense	AL162274.1	ENSG00000273521.1	0	0
antisense	AL512622.1	ENSG00000235010.1	0	1
antisense	AL451069.3	ENSG00000234311.1	0	0
antisense	LINC01165	ENSG00000229081.1	0	0
antisense	ADGRA1-	ENSG00000256925.2	0	0
antisense	MIR202HG	ENSG00000166917.10	0	0
antisense	AL360181.1	ENSG00000226699.1	0	0

antisense	AL161645.1	ENSG00000278518.1	0	0
antisense	AC069287.3	ENSG00000255229.1	0	0
antisense	AC069287.2	ENSG00000254559.1	0	0
antisense	AC136475.3	ENSG00000255026.1	0	0
antisense	AC136475.2	ENSG00000254910.1	0	0
antisense	AC136475.1	ENSG00000251661.3	0	0
antisense	AC136475.4	ENSG00000255089.1	0	0
antisense	AC138230.1	ENSG00000255237.1	0	0
antisense	AC137894.1	ENSG00000254739.1	0	0
antisense	AC131934.1	ENSG00000255158.1	0	0
antisense	AP006621.4	ENSG00000269915.1	0	0
antisense	AP006621.1	ENSG00000255108.1	0	0
antisense	MUC5B-AS1	ENSG00000255177.2	0	0
antisense	KRTAP5-AS1	ENSG00000233930.3	0	0
antisense	FAM99B	ENSG00000205865.4	0	0
antisense	AC068580.3	ENSG00000235027.1	0	0
antisense	MRPL23-AS1	ENSG00000226416.1	0	0
antisense	IGF2-AS	ENSG00000099869.7	0	0
antisense	CD81-AS1	ENSG00000238184.1	0	0
antisense	AC124057.1	ENSG00000230483.1	0	0
antisense	KCNQ1-AS1	ENSG00000229414.2	0	0
antisense	KCNQ1DN	ENSG00000237941.2	0	0
antisense	AC131971.1	ENSG00000183562.3	0	0
antisense	CARS-AS1	ENSG00000247473.2	0	0
antisense	AC108448.3	ENSG00000285644.1	0	0
antisense	AC108448.1	ENSG00000234791.1	0	0
antisense	MRGPRG-	ENSG00000236301.5	0	0
antisense	AC109309.1	ENSG00000224513.2	0	0
antisense	AC123788.1	ENSG00000254592.1	0	0
antisense	AC090587.1	ENSG00000228661.1	0	0
antisense	RRM1-AS1	ENSG00000255276.1	0	0
antisense	AC104389.1	ENSG00000224091.1	0	0
antisense	AC091564.2	ENSG00000254400.1	0	0
antisense	AC091564.6	ENSG00000255680.1	0	0
antisense	AC091564.4	ENSG00000255390.1	0	0
antisense	AC091564.5	ENSG00000255410.1	0	0
antisense	AC107884.1	ENSG00000251364.6	0	0
antisense	AC107884.2	ENSG00000254864.1	0	0
antisense	AC104237.1	ENSG00000254707.1	0	0
antisense	CASC23	ENSG00000255420.1	0	0
antisense	AC116456.1	ENSG00000254921.1	0	0
antisense	AC091053.2	ENSG00000254900.1	0	0
antisense	AC091053.1	ENSG00000254665.1	0	0
antisense	AC026894.1	ENSG00000255159.1	0	0

antisense	TMEM9B-	ENSG00000254860.5	0	0
antisense	AC079296.1	ENSG00000253973.2	0	0
antisense	AC132192.1	ENSG00000254397.1	0	0
antisense	AC100763.1	ENSG00000254865.1	0	0
antisense	AC080023.1	ENSG00000254554.1	0	0
antisense	MRVI1-AS1	ENSG00000177112.7	0	0
antisense	AC023946.1	ENSG00000255351.1	0	0
antisense	AC079329.1	ENSG00000254680.1	0	0
antisense	AC013549.2	ENSG00000254688.1	0	0
antisense	AC013549.1	ENSG00000203258.3	0	0
antisense	SPON1-AS1	ENSG00000254418.1	0	0
antisense	AC103794.1	ENSG00000254878.1	0	0
antisense	AC124798.2	ENSG00000285545.1	0	0
antisense	AC124301.2	ENSG00000255335.1	0	0
antisense	AC103974.1	ENSG00000254966.1	0	0
antisense	AC023078.5	ENSG00000255244.1	0	0
antisense	NAV2-AS5	ENSG00000255043.1	0	0
antisense	NAV2-AS4	ENSG00000254622.1	0	0
antisense	NAV2-AS3	ENSG00000254542.1	0	1
antisense	NAV2-AS2	ENSG00000254453.1	0	1
antisense	NAV2-AS1	ENSG00000254894.1	0	0
antisense	AC090707.1	ENSG00000254906.1	0	0
antisense	AC090857.2	ENSG00000255167.1	0	0
antisense	AC107886.1	ENSG00000255372.1	0	0
antisense	AC104009.1	ENSG00000254768.5	0	0
antisense	AC040936.1	ENSG00000254540.1	0	0
antisense	AC006299.1	ENSG00000246225.6	0	0
antisense	AL353699.1	ENSG00000255480.1	0	0
antisense	MPPED2-	ENSG00000254489.1	0	0
antisense	AL137804.1	ENSG00000255525.1	0	0
antisense	AC131571.1	ENSG00000228061.6	0	0
antisense	AL035078.1	ENSG00000254584.1	0	0
antisense	AL035078.3	ENSG00000254836.1	0	0
antisense	AL078612.1	ENSG00000255252.3	0	0
antisense	AL049629.1	ENSG00000255202.1	0	0
antisense	FBXO3-DT	ENSG00000254508.5	0	0
antisense	AC113192.5	ENSG00000255272.1	0	0
antisense	AC132216.1	ENSG00000184566.3	0	0
antisense	CD44-AS1	ENSG00000255443.1	0	2
antisense	AL133330.2	ENSG00000255004.1	0	0
antisense	AC090625.2	ENSG00000255542.1	0	0
antisense	AC090692.1	ENSG00000254919.1	0	0
antisense	AC009656.1	ENSG00000255060.1	0	0
antisense	AC087276.2	ENSG00000254907.1	0	0

antisense	AC087276.3	ENSG00000255340.1	0	0
antisense	AC087521.2	ENSG00000246250.2	0	0
antisense	AC087521.4	ENSG00000283375.1	0	0
antisense	ALKBH3-	ENSG00000244926.6	0	0
antisense	AC010768.1	ENSG00000254693.1	0	0
antisense	AC010768.2	ENSG00000255092.1	0	0
antisense	AC103681.2	ENSG00000254664.1	0	0
antisense	AC103681.1	ENSG00000254497.1	0	0
antisense	AC087442.1	ENSG00000255091.1	0	0
antisense	AC068385.1	ENSG00000255498.1	0	1
antisense	LINC02489	ENSG00000255007.1	0	0
antisense	AC127035.1	ENSG00000285658.1	0	1
antisense	AC090589.2	ENSG00000255520.1	0	0
antisense	AC018410.2	ENSG00000256897.1	0	0
antisense	AC018410.1	ENSG00000256746.5	0	0
antisense	AC090559.1	ENSG00000255197.5	0	0
antisense	KF459542.1	ENSG00000231880.2	0	0
antisense	PTPRJ-AS1	ENSG00000254879.1	0	0
antisense	AP002893.1	ENSG00000255301.1	0	0
antisense	AP001350.1	ENSG00000269570.2	0	0
antisense	AP001652.1	ENSG00000255523.1	0	0
antisense	AP001636.3	ENSG00000255240.5	0	0
antisense	AP000777.1	ENSG00000255845.1	0	0
antisense	AP000777.2	ENSG00000255959.1	0	0
antisense	AP000777.3	ENSG00000256813.1	0	0
antisense	AP003721.4	ENSG00000257052.1	0	0
antisense	AP003721.3	ENSG00000256944.1	0	0
antisense	AP003721.1	ENSG00000256196.1	0	0
antisense	AP003306.1	ENSG00000254404.1	0	0
antisense	AP003306.2	ENSG00000255118.1	0	0
antisense	AP003064.1	ENSG00000255126.1	0	0
antisense	AP003064.2	ENSG00000255446.1	0	0
antisense	AP001363.1	ENSG00000250659.2	0	0
antisense	AP001363.2	ENSG00000257058.1	0	0
antisense	AP001458.1	ENSG00000254964.1	0	0
antisense	AP000438.1	ENSG00000257002.1	0	0
antisense	AP000753.1	ENSG00000203520.3	0	0
antisense	AP000753.2	ENSG00000256789.1	0	0
antisense	AP000721.2	ENSG00000256824.1	0	0
antisense	AP006333.2	ENSG00000256481.1	0	0
antisense	AP006333.1	ENSG00000256341.1	0	0
antisense	AP001092.1	ENSG00000237410.1	0	0
antisense	AP003068.1	ENSG00000254501.1	0	0
antisense	AP003068.3	ENSG00000255173.1	0	0

antisense	AP000944.1	ENSG00000255478.1	0	0
antisense	OVOL1-AS1	ENSG00000255120.5	0	0
antisense	AP001266.1	ENSG00000255404.1	0	0
antisense	AP001107.4	ENSG00000254461.1	0	0
antisense	AP001107.8	ENSG00000254855.1	0	0
antisense	AP001107.7	ENSG00000254762.1	0	0
antisense	AP001107.2	ENSG00000254452.1	0	0
antisense	AP001107.3	ENSG00000254458.1	0	0
antisense	AP001107.6	ENSG00000254756.1	0	2
antisense	RPS6KB2-	ENSG00000255949.1	0	0
antisense	AP003385.3	ENSG00000255119.1	0	0
antisense	AC004923.4	ENSG00000255306.1	0	0
antisense	AP002992.1	ENSG00000255236.2	0	0
antisense	AP000808.2	ENSG00000255741.1	0	0
antisense	AP003071.3	ENSG00000261276.1	0	0
antisense	LINC02584	ENSG00000254417.1	0	0
antisense	ANO1-AS1	ENSG00000254902.1	0	0
antisense	AP000879.1	ENSG00000254721.1	0	0
antisense	AP002336.2	ENSG00000254604.1	0	0
antisense	AP002336.3	ENSG00000255539.1	0	0
antisense	AP000487.2	ENSG00000254495.1	0	0
antisense	SHANK2-	ENSG00000226627.1	0	0
antisense	SHANK2-	ENSG00000236262.1	0	0
antisense	SHANK2-	ENSG00000171671.6	0	0
antisense	AP002490.1	ENSG00000251143.1	0	0
antisense	AP000812.1	ENSG00000204971.3	0	0
antisense	AP000593.3	ENSG00000255843.1	0	0
antisense	AP002892.2	ENSG00000256739.1	0	0
antisense	AP002892.1	ENSG00000255672.1	0	0
antisense	AP003785.1	ENSG00000256403.1	0	0
antisense	AP005019.1	ENSG00000256633.1	0	0
antisense	AP003065.1	ENSG00000255808.1	0	0
antisense	ARAP1-AS1	ENSG00000256007.1	0	0
antisense	ARAP1-AS2	ENSG00000245148.2	0	0
antisense	AP002761.1	ENSG00000215841.3	0	0
antisense	AP002761.3	ENSG00000257038.1	0	0
antisense	AP000763.4	ENSG00000256928.1	0	0
antisense	AP000763.3	ENSG00000256448.5	0	0
antisense	AP002993.1	ENSG00000255928.1	0	0
antisense	AP002770.1	ENSG00000256034.1	0	0
antisense	AP003717.1	ENSG00000255847.5	0	0
antisense	AP001372.3	ENSG00000254928.1	0	0
antisense	AP001972.4	ENSG00000255395.1	0	0
antisense	AP001972.1	ENSG00000254429.1	0	0

antisense	AP001972.2	ENSG00000254963.1	0	0
antisense	AP001922.5	ENSG00000255326.1	0	0
antisense	AP001922.1	ENSG00000247867.2	0	0
antisense	AP003031.1	ENSG00000254814.1	0	0
antisense	UVRAG-DT	ENSG00000255507.5	0	2
antisense	AP002340.1	ENSG00000255421.1	0	0
antisense	AP000785.1	ENSG00000254933.1	0	0
antisense	AP001189.1	ENSG00000236304.1	0	0
antisense	AP003119.1	ENSG00000254632.1	0	0
antisense	AP002498.1	ENSG00000254988.1	0	0
antisense	AP003680.1	ENSG00000268635.2	0	0
antisense	AP002812.2	ENSG00000254459.1	0	0
antisense	AP002812.3	ENSG00000254691.1	0	0
antisense	AP002812.5	ENSG00000255449.1	0	0
antisense	AP003032.2	ENSG00000254829.1	0	0
antisense	AP003032.1	ENSG00000254675.1	0	0
antisense	AP003086.2	ENSG00000254420.1	0	0
antisense	AP003086.3	ENSG00000254649.1	0	0
antisense	AP003110.1	ENSG00000255084.1	0	0
antisense	AP002768.1	ENSG00000254563.1	0	0
antisense	AP000873.3	ENSG00000254551.1	0	0
antisense	AP000446.1	ENSG00000255234.5	0	0
antisense	AP002370.2	ENSG00000255311.5	0	0
antisense	AP001825.1	ENSG00000254787.1	0	0
antisense	AP000857.2	ENSG00000255555.1	0	0
antisense	AP003128.1	ENSG00000255005.1	0	0
antisense	AP001831.1	ENSG00000254733.1	0	0
antisense	AP005436.2	ENSG00000255241.1	0	0
antisense	GRM5-AS1	ENSG00000255082.1	0	0
antisense	AP000722.1	ENSG00000254705.1	0	0
antisense	AP003718.1	ENSG00000255506.1	0	0
antisense	AP000786.1	ENSG00000255893.1	0	0
antisense	AP000943.3	ENSG00000255929.5	0	0
antisense	AP002383.2	ENSG00000256469.1	0	0
antisense	AP001351.1	ENSG00000248027.1	0	0
antisense	AP000942.2	ENSG00000254422.1	0	0
antisense	AP001830.2	ENSG00000255482.1	0	0
antisense	AP000851.1	ENSG00000256916.1	0	0
antisense	AP003043.1	ENSG00000255548.1	0	0
antisense	AP000813.1	ENSG00000285813.1	0	0
antisense	AP001001.1	ENSG00000254433.1	0	0
antisense	AP002433.2	ENSG00000285696.1	0	0
antisense	AP003049.2	ENSG00000255028.5	0	0
antisense	COLCA1	ENSG00000196167.9	0	0

antisense	AP002008.1	ENSG00000254980.1	0	0
antisense	AP002884.2	ENSG00000254638.1	0	0
antisense	AP002884.4	ENSG00000268472.2	0	3
antisense	AP000802.1	ENSG00000247416.3	0	0
antisense	NCAM1-AS1	ENSG00000227487.3	0	0
antisense	AP002840.1	ENSG00000256757.1	0	0
antisense	AP003170.3	ENSG00000256452.1	0	0
antisense	AP002518.2	ENSG00000256947.1	0	0
antisense	AP002518.1	ENSG00000256195.2	0	0
antisense	AP006216.1	ENSG00000226645.1	0	0
antisense	AP006216.2	ENSG00000236267.1	0	0
antisense	AP006216.3	ENSG00000285513.1	0	0
antisense	APOA1-AS	ENSG00000235910.1	0	0
antisense	AP000936.1	ENSG00000224077.1	0	0
antisense	AP000892.1	ENSG00000250699.1	0	0
antisense	AP000757.2	ENSG00000254844.4	0	0
antisense	AP000757.1	ENSG00000254528.7	0	0
antisense	AP001267.1	ENSG00000254873.1	0	0
antisense	AP001267.3	ENSG00000255435.6	0	0
antisense	AP000941.1	ENSG00000255176.1	0	0
antisense	AP002954.1	ENSG00000255422.3	0	0
antisense	AP004609.1	ENSG00000245869.2	0	0
antisense	AP003392.1	ENSG00000254428.1	0	1
antisense	AP003392.3	ENSG00000255114.1	0	0
antisense	AP003396.1	ENSG00000245385.2	0	0
antisense	AP003396.3	ENSG00000254740.2	0	0
antisense	USP2-AS1	ENSG00000245248.7	0	0
antisense	AP003393.1	ENSG00000254561.3	0	0
antisense	AP003390.1	ENSG00000254854.1	0	0
antisense	AP000679.1	ENSG00000176984.6	0	0
antisense	AP001150.1	ENSG00000259541.5	0	0
antisense	AP004147.1	ENSG00000250493.2	0	0
antisense	AP000977.1	ENSG00000246790.2	0	0
antisense	AP000755.2	ENSG00000255219.1	0	0
antisense	AP002762.1	ENSG00000285909.1	0	0
antisense	AP001970.1	ENSG00000254710.1	0	0
antisense	AP000866.2	ENSG00000250073.2	0	0
antisense	AP003501.1	ENSG00000254568.1	0	0
antisense	AP003501.2	ENSG00000254943.1	0	0
antisense	AP001007.1	ENSG00000254932.2	0	0
antisense	AP000842.2	ENSG00000255027.2	0	0
antisense	AP001318.1	ENSG00000254905.1	0	0
antisense	KIRREL3-	ENSG00000257271.1	0	0
antisense	KIRREL3-	ENSG00000254960.2	0	0

antisense	AP002833.2	ENSG00000255317.1	0	0
antisense	KIRREL3-	ENSG00000218109.5	0	0
antisense	ETS1-AS1	ENSG00000254588.1	0	0
antisense	AP003025.1	ENSG00000237654.5	0	0
antisense	AP003025.2	ENSG00000285980.1	0	0
antisense	AP000844.2	ENSG00000238117.1	0	0
antisense	AP000844.1	ENSG00000224700.2	0	0
antisense	AP001775.2	ENSG00000255348.1	0	0
antisense	AC026369.3	ENSG00000256948.1	0	0
antisense	AC026369.1	ENSG00000249695.6	0	0
antisense	AC026369.2	ENSG00000256694.1	0	0
antisense	AC007406.1	ENSG00000255671.1	0	0
antisense	AC007406.4	ENSG00000256577.2	0	0
antisense	AC006205.1	ENSG00000255825.1	0	0
antisense	AC004765.1	ENSG00000285704.1	0	0
antisense	AC004672.1	ENSG00000249028.2	0	0
antisense	AC005343.1	ENSG00000285627.1	0	0
antisense	AC005342.2	ENSG00000256706.1	0	0
antisense	AC005342.1	ENSG00000203593.3	0	2
antisense	AC005344.1	ENSG00000278255.1	0	0
antisense	CACNA1C-	ENSG00000256025.1	0	0
antisense	AC005293.1	ENSG00000285555.1	0	0
antisense	AC005414.1	ENSG00000285734.1	0	0
antisense	CACNA1C-	ENSG00000256769.1	0	0
antisense	CACNA1C-	ENSG00000256271.1	0	0
antisense	CACNA1C-	ENSG00000246627.6	0	0
antisense	AC005841.1	ENSG00000258092.1	0	0
antisense	AC005908.2	ENSG00000256691.1	0	0
antisense	AC006207.1	ENSG00000283138.1	0	0
antisense	AC005842.1	ENSG00000256862.2	0	0
antisense	AC006064.3	ENSG00000255966.1	0	0
antisense	AC006064.4	ENSG00000269968.1	0	0
antisense	AC006064.1	ENSG00000245667.2	0	0
antisense	AC125494.3	ENSG00000270068.1	0	0
antisense	U47924.1	ENSG00000271969.1	0	0
antisense	AC018653.3	ENSG00000256967.1	0	0
antisense	AC018653.4	ENSG00000285770.1	0	0
antisense	AC018653.1	ENSG00000255572.1	0	0
antisense	AC092490.2	ENSG00000255829.1	0	0
antisense	A2ML1-AS1	ENSG00000256661.1	0	0
antisense	A2ML1-AS2	ENSG00000256904.1	0	0
antisense	AC006581.2	ENSG00000282022.1	0	0
antisense	AC006581.1	ENSG00000257105.1	0	0
antisense	AC010186.1	ENSG00000256442.1	0	0

antisense	AC091814.1	ENSG00000255882.1	0	0
antisense	AC024224.2	ENSG00000256803.1	0	0
antisense	AC115676.1	ENSG00000255958.1	0	0
antisense	AC008115.1	ENSG00000256658.1	0	0
antisense	AC007688.2	ENSG00000256011.1	0	0
antisense	AC008114.1	ENSG00000255649.1	0	0
antisense	PLBD1-AS1	ENSG00000256751.5	0	0
antisense	AC007655.1	ENSG00000256339.1	0	0
antisense	AC007552.2	ENSG00000256564.1	0	0
antisense	AC087242.1	ENSG00000255648.1	0	0
antisense	AC129102.1	ENSG00000256879.1	0	0
antisense	AC010197.1	ENSG00000256615.1	0	0
antisense	AC010185.1	ENSG00000255644.1	0	0
antisense	AC008250.1	ENSG00000257022.1	0	0
antisense	AC087241.3	ENSG00000257023.1	0	0
antisense	AC087260.1	ENSG00000256473.1	0	0
antisense	AC023796.2	ENSG00000256482.1	0	0
antisense	AC026310.2	ENSG00000255921.1	0	0
antisense	AC023510.1	ENSG00000258449.1	0	0
antisense	AC023510.2	ENSG00000276842.1	0	0
antisense	AC092794.2	ENSG00000275197.1	0	0
antisense	AC022509.3	ENSG00000256894.1	0	0
antisense	AC022509.2	ENSG00000256234.1	0	0
antisense	AC022509.1	ENSG00000255750.5	0	0
antisense	AC022509.4	ENSG00000278095.1	0	0
antisense	AC024145.1	ENSG00000255968.1	0	0
antisense	AC023051.1	ENSG00000234428.2	0	0
antisense	AC024896.1	ENSG00000247903.1	0	0
antisense	ARNTL2-AS1	ENSG00000245311.2	0	0
antisense	AC009509.1	ENSG00000256377.5	0	0
antisense	AC009511.2	ENSG00000256747.1	0	0
antisense	AC009511.1	ENSG00000256512.1	0	0
antisense	AC012150.2	ENSG00000275278.1	0	0
antisense	OVCH1-AS1	ENSG00000257599.2	0	0
antisense	AC009320.1	ENSG00000257456.1	0	0
antisense	DENND5B-	ENSG00000255867.1	0	0
antisense	AC048344.1	ENSG00000257530.1	0	0
antisense	AC046130.1	ENSG00000245482.2	0	0
antisense	CPNE8-AS1	ENSG00000257718.1	0	0
antisense	AC121334.3	ENSG00000285732.1	0	0
antisense	AC079630.1	ENSG00000225342.2	0	0
antisense	AC107023.1	ENSG00000258167.1	0	0
antisense	AC090531.1	ENSG00000257228.5	0	0
antisense	AC079601.1	ENSG00000257225.1	0	0

antisense	AC025030.2	ENSG00000257947.1	0	0
antisense	AC008127.1	ENSG00000257319.1	0	0
antisense	AC025031.2	ENSG00000258096.1	0	0
antisense	AC008083.1	ENSG00000257925.1	0	0
antisense	AC004466.1	ENSG00000268069.2	0	0
antisense	AC004466.3	ENSG00000276691.1	0	0
antisense	AC121338.1	ENSG00000205537.2	0	0
antisense	AC004801.4	ENSG00000257985.1	0	0
antisense	AC074029.3	ENSG00000274124.1	0	0
antisense	AC024257.3	ENSG00000269514.2	0	0
antisense	AC090115.1	ENSG00000257735.1	0	0
antisense	AC089987.2	ENSG00000258121.1	0	0
antisense	AC117498.1	ENSG00000257653.1	0	0
antisense	AC011603.3	ENSG00000258283.1	0	0
antisense	AC011603.1	ENSG00000257346.1	0	0
antisense	AC011603.2	ENSG00000258017.1	0	0
antisense	AC125611.3	ENSG00000258232.2	0	0
antisense	AC125611.4	ENSG00000258334.1	0	0
antisense	AC020612.3	ENSG00000257964.1	0	0
antisense	AC025154.2	ENSG00000257588.1	0	0
antisense	AC025154.1	ENSG00000257378.1	0	0
antisense	AC008147.1	ENSG00000257256.1	0	0
antisense	AC008121.3	ENSG00000274797.1	0	0
antisense	AC107031.1	ENSG00000271065.1	0	0
antisense	AC025259.1	ENSG00000257663.1	0	0
antisense	KRT7-AS	ENSG00000257671.1	0	0
antisense	AC021066.1	ENSG00000257830.1	0	0
antisense	AC121757.1	ENSG00000257829.1	0	0
antisense	AC078865.1	ENSG00000258253.1	0	0
antisense	AC055736.1	ENSG00000257500.1	0	0
antisense	KRT73-AS1	ENSG00000257495.5	0	0
antisense	AC055716.3	ENSG00000257700.1	0	0
antisense	AC023509.2	ENSG00000257550.1	0	0
antisense	AC023509.6	ENSG00000285692.1	0	0
antisense	HOXC-AS1	ENSG00000250451.5	0	0
antisense	AC023794.1	ENSG00000248576.1	0	0
antisense	AC078778.1	ENSG00000257596.1	0	0
antisense	AC079313.2	ENSG00000258137.5	0	0
antisense	AC079313.1	ENSG00000258086.1	0	0
antisense	AC068789.1	ENSG00000257824.1	0	0
antisense	AC009779.4	ENSG00000258921.1	0	0
antisense	AC025162.1	ENSG00000258554.1	0	0
antisense	AC034102.8	ENSG00000273890.1	0	0
antisense	AC034102.3	ENSG00000257449.1	0	0

antisense	AC034102.4	ENSG00000257553.1	0	0
antisense	AC034102.7	ENSG00000258345.1	0	0
antisense	AC034102.6	ENSG00000258317.1	0	0
antisense	AC034102.5	ENSG00000257809.1	0	0
antisense	AC073896.5	ENSG00000258260.1	0	0
antisense	LRP1-AS	ENSG00000259125.1	0	0
antisense	AC137834.2	ENSG00000276727.1	0	0
antisense	AC025165.1	ENSG00000224713.4	0	0
antisense	AC025165.2	ENSG00000257342.1	0	0
antisense	AC083805.2	ENSG00000257953.1	0	0
antisense	AC079035.1	ENSG00000257568.1	0	0
antisense	AC084357.2	ENSG00000249753.2	0	0
antisense	RXYLT1-AS1	ENSG00000255850.2	0	0
antisense	AC020611.2	ENSG00000255886.1	0	0
antisense	AC025576.2	ENSG00000255817.1	0	0
antisense	AC025576.1	ENSG00000255629.1	0	0
antisense	AC078962.2	ENSG00000256199.1	0	0
antisense	AC078962.4	ENSG00000256670.1	0	0
antisense	AC078962.3	ENSG00000256314.1	0	0
antisense	AC025262.1	ENSG00000215159.3	0	0
antisense	AC135895.1	ENSG00000198671.3	0	0
antisense	AC090673.2	ENSG00000256083.1	0	0
antisense	LLPH-DT	ENSG00000239335.4	0	0
antisense	AC078889.1	ENSG00000256072.1	0	0
antisense	AC078777.1	ENSG00000235872.2	0	0
antisense	IFNG-AS1	ENSG00000255733.5	0	0
antisense	AC025423.4	ENSG00000257181.1	0	0
antisense	AC020656.1	ENSG00000257764.2	0	0
antisense	AC025263.1	ENSG00000247131.5	0	0
antisense	AC078922.1	ENSG00000257241.1	0	0
antisense	AC083809.1	ENSG00000277247.1	0	0
antisense	AC078860.1	ENSG00000257761.1	0	0
antisense	AC090109.1	ENSG00000258115.1	0	0
antisense	AC025257.1	ENSG00000257386.1	0	0
antisense	AC073525.1	ENSG00000257434.1	0	0
antisense	AC091534.1	ENSG00000254451.2	0	0
antisense	AC011611.3	ENSG00000257453.1	0	0
antisense	AC011611.4	ENSG00000257839.1	0	0
antisense	AC124784.1	ENSG00000257910.1	0	0
antisense	AC073571.1	ENSG00000258225.1	0	0
antisense	AC090709.1	ENSG00000257191.1	0	0
antisense	AC073569.1	ENSG00000258044.1	0	0
antisense	AC074031.1	ENSG00000257429.1	0	0
antisense	AC078955.1	ENSG00000258026.1	0	0

antisense	AC069228.1	ENSG00000258162.2	0	0
antisense	AC024941.2	ENSG00000281333.1	0	0
antisense	AC024909.2	ENSG00000274021.1	0	2
antisense	AC063949.2	ENSG00000266923.1	0	0
antisense	AC124947.1	ENSG00000257252.5	0	0
antisense	AC012085.2	ENSG00000258274.1	0	0
antisense	AC012464.1	ENSG00000257283.1	0	0
antisense	AC123567.2	ENSG00000258035.1	0	0
antisense	AC073655.1	ENSG00000258172.1	0	0
antisense	AC090001.1	ENSG00000258343.1	0	0
antisense	AC007298.2	ENSG00000257878.1	0	0
antisense	AC007298.1	ENSG00000257715.1	0	0
antisense	AC008149.1	ENSG00000258177.1	0	0
antisense	AC069437.1	ENSG00000257458.1	0	0
antisense	AC117377.1	ENSG00000258039.2	0	0
antisense	AC010203.2	ENSG00000257696.1	0	0
antisense	AC138360.1	ENSG00000257325.1	0	0
antisense	AC063947.1	ENSG00000258007.1	0	0
antisense	AC117505.1	ENSG00000257514.5	0	0
antisense	AC084398.2	ENSG00000257202.1	0	0
antisense	AC025265.2	ENSG00000257737.1	0	0
antisense	AC025265.3	ENSG00000257766.1	0	0
antisense	AC025265.1	ENSG00000257681.1	0	1
antisense	AC012555.2	ENSG00000257754.1	0	0
antisense	AC016257.1	ENSG00000257999.1	0	0
antisense	KCCAT198	ENSG00000257642.1	0	0
antisense	AC011595.1	ENSG00000257438.1	0	0
antisense	AC079385.1	ENSG00000257545.4	0	0
antisense	AC079385.2	ENSG00000257711.1	0	0
antisense	AC079385.3	ENSG00000257918.1	0	0
antisense	AC078929.1	ENSG00000257548.1	0	0
antisense	AC007540.1	ENSG00000257579.1	0	0
antisense	AC007622.2	ENSG00000258136.1	0	0
antisense	LINC01498	ENSG00000247213.6	0	0
antisense	AC007569.1	ENSG00000257221.3	0	0
antisense	FAM222A-	ENSG00000255650.5	0	0
antisense	AC084876.1	ENSG00000277299.1	0	0
antisense	ATXN2-AS	ENSG00000258099.1	0	0
antisense	AC073575.2	ENSG00000274227.1	0	0
antisense	AC073575.1	ENSG00000258323.1	0	0
antisense	AC004217.1	ENSG00000257494.1	0	0
antisense	AC004551.1	ENSG00000257452.1	0	0
antisense	AC089999.1	ENSG00000257286.1	0	0
antisense	LHX5-AS1	ENSG00000257935.2	0	0

antisense	AC073863.1	ENSG00000257359.1	0	0
antisense	TBX5-AS1	ENSG00000255399.3	0	0
antisense	AC012157.1	ENSG00000258034.1	0	0
antisense	AC083806.2	ENSG00000274554.1	0	0
antisense	AC127164.1	ENSG00000257279.1	0	0
antisense	AC084291.1	ENSG00000256071.1	0	0
antisense	AC131159.2	ENSG00000277873.1	0	0
antisense	AC131159.1	ENSG00000276292.1	0	0
antisense	AC131238.1	ENSG00000274859.1	0	0
antisense	AC084361.1	ENSG00000257095.1	0	0
antisense	AC084880.4	ENSG00000256884.1	0	0
antisense	AC084880.3	ENSG00000256609.1	0	0
antisense	AC084880.2	ENSG00000256311.1	0	0
antisense	AC002563.1	ENSG00000255692.1	0	0
antisense	AC004263.1	ENSG00000275936.1	0	0
antisense	AC125616.1	ENSG00000256008.2	0	0
antisense	AC069234.2	ENSG00000256364.1	0	0
antisense	AC069234.3	ENSG00000256569.1	0	0
antisense	AC069234.1	ENSG00000255946.1	0	0
antisense	AC079360.1	ENSG00000256811.1	0	0
antisense	AC068768.2	ENSG00000269980.1	0	0
antisense	THRIL	ENSG00000280634.1	0	0
antisense	AC137590.1	ENSG00000257000.1	0	0
antisense	AC148477.3	ENSG00000256943.1	0	0
antisense	AC148477.2	ENSG00000256542.2	0	0
antisense	AC148477.1	ENSG00000255916.1	0	0
antisense	AC026786.2	ENSG00000271963.1	0	0
antisense	LINC00442	ENSG00000232685.4	0	0
antisense	AL137001.1	ENSG00000226619.1	0	0
antisense	PSPC1-AS2	ENSG00000226352.2	0	1
antisense	AL355001.1	ENSG00000223576.2	0	0
antisense	LINC00556	ENSG00000260131.1	0	0
antisense	AL590096.1	ENSG00000277020.4	0	0
antisense	LATS2-AS1	ENSG00000233851.1	0	0
antisense	SPATA13-	ENSG00000227213.1	0	0
antisense	C1QTNF9-	ENSG00000240868.1	0	0
antisense	LINC01053	ENSG00000238169.1	0	0
antisense	WASF3-AS1	ENSG00000237001.6	0	0
antisense	AL159978.1	ENSG00000285935.1	0	0
antisense	USP12-AS1	ENSG00000232162.1	0	0
antisense	MTUS2-AS2	ENSG00000236758.5	0	0
antisense	MTUS2-AS1	ENSG00000179141.9	0	0
antisense	LINC00572	ENSG00000224405.1	0	0
antisense	AL353648.1	ENSG00000285840.1	0	0

antisense	AL353680.1	ENSG00000238185.1	0	0
antisense	FRY-AS1	ENSG00000237637.1	0	0
antisense	AL138999.2	ENSG00000285621.1	0	0
antisense	SPART-AS1	ENSG00000120664.10	0	0
antisense	FREM2-AS1	ENSG00000225350.1	0	0
antisense	AL157932.1	ENSG00000231530.1	0	0
antisense	ENOX1-AS2	ENSG00000238189.2	0	0
antisense	AL138963.3	ENSG00000273149.1	0	0
antisense	HTR2A-AS1	ENSG00000224517.6	0	0
antisense	SUCLA2-AS1	ENSG00000227848.1	0	0
antisense	MED4-AS1	ENSG00000229111.1	0	0
antisense	AL392048.1	ENSG00000277684.1	0	0
antisense	DLEU7-AS1	ENSG00000237152.3	0	0
antisense	AL139082.1	ENSG00000273523.1	0	0
antisense	AL445288.1	ENSG00000278722.1	0	0
antisense	DIAPH3-AS1	ENSG00000227528.5	0	0
antisense	DIAPH3-AS2	ENSG00000223815.1	0	0
antisense	LINC00395	ENSG00000231061.1	0	0
antisense	PCDH9-AS1	ENSG00000234527.1	0	0
antisense	PCDH9-AS2	ENSG00000228842.3	0	0
antisense	PCDH9-AS3	ENSG00000225263.1	0	0
antisense	PCDH9-AS4	ENSG00000233840.1	0	0
antisense	AL160254.1	ENSG00000285588.1	0	0
antisense	ATXN8OS	ENSG00000230223.6	0	0
antisense	AL139230.1	ENSG00000225203.2	0	0
antisense	SCEL-AS1	ENSG00000224347.6	0	0
antisense	RNF219-AS1	ENSG00000234377.7	0	0
antisense	AL445209.1	ENSG00000271776.1	0	0
antisense	NDFIP2-AS1	ENSG00000232132.1	0	0
antisense	AL355481.1	ENSG00000285680.1	0	0
antisense	GPC5-AS2	ENSG00000232885.1	0	0
antisense	GPC5-AS1	ENSG00000235984.5	0	0
antisense	GPC6-AS2	ENSG00000224394.1	0	0
antisense	GPC6-AS1	ENSG00000236520.2	0	0
antisense	CLDN10-AS1	ENSG00000223392.1	0	0
antisense	AL161896.1	ENSG00000269599.1	0	0
antisense	FARP1-AS1	ENSG00000231194.1	0	0
antisense	DOCK9-AS1	ENSG00000229918.1	0	0
antisense	LINC00449	ENSG00000203441.2	0	0
antisense	CLYBL-AS2	ENSG00000227659.1	0	0
antisense	CLYBL-AS1	ENSG00000234303.2	0	0
antisense	LINC00554	ENSG00000260738.1	0	0
antisense	PCCA-AS1	ENSG00000234650.5	0	0
antisense	AL136526.1	ENSG00000280169.1	0	0

antisense	NALCN-AS1	ENSG00000233009.1	0	0
antisense	FGF14-AS1	ENSG00000234445.1	0	0
antisense	LINC00555	ENSG00000261057.1	0	0
antisense	LINC00283	ENSG00000231633.1	0	0
antisense	DAOA-AS1	ENSG00000232307.1	0	0
antisense	AL138689.2	ENSG00000284966.2	0	0
antisense	MYO16-AS2	ENSG00000229938.1	0	0
antisense	MYO16-AS1	ENSG00000236242.1	0	0
antisense	LINC00676	ENSG00000234854.1	0	0
antisense	AL162497.1	ENSG00000275741.1	0	0
antisense	COL4A2-AS2	ENSG00000224821.5	0	0
antisense	COL4A2-AS1	ENSG00000232814.2	0	0
antisense	ARHGEF7-	ENSG00000235875.3	0	0
antisense	ARHGEF7-	ENSG00000227352.1	0	0
antisense	AL353704.1	ENSG00000285856.1	0	0
antisense	ATP11A-AS1	ENSG00000232684.1	0	0
antisense	MCF2L-AS1	ENSG00000235280.2	0	0
antisense	F10-AS1	ENSG00000231882.1	0	0
antisense	AL137002.2	ENSG00000283828.1	0	0
antisense	AL137002.1	ENSG00000269125.1	0	0
antisense	GRTP1-AS1	ENSG00000225083.1	0	0
antisense	DCUN1D2-	ENSG00000233613.5	0	0
antisense	AL160396.1	ENSG00000283347.1	0	0
antisense	LINC01054	ENSG00000229723.1	0	0
antisense	CR383656.10	ENSG00000257959.1	0	0
antisense	AL929601.3	ENSG00000275563.1	0	0
antisense	AL512624.2	ENSG00000276888.1	0	0
antisense	AL356019.1	ENSG00000258459.1	0	0
antisense	AL355075.4	ENSG00000259001.3	0	0
antisense	AL355075.2	ENSG00000258515.1	0	0
antisense	AL163195.2	ENSG00000258573.5	0	0
antisense	AL163636.1	ENSG00000258451.1	0	0
antisense	AL133371.2	ENSG00000258810.1	0	0
antisense	AL133371.1	ENSG00000258642.1	0	0
antisense	AL161668.3	ENSG00000258471.2	0	0
antisense	AL161668.2	ENSG00000255472.1	0	0
antisense	AL135744.1	ENSG00000260830.1	0	0
antisense	AL161747.2	ENSG00000257096.1	0	0
antisense	AC243965.2	ENSG00000275552.1	0	0
antisense	PRMT5-AS1	ENSG00000237054.9	0	0
antisense	AL132780.1	ENSG00000257285.5	0	0
antisense	AL049829.2	ENSG00000259018.1	0	0
antisense	AL132855.1	ENSG00000258444.1	0	0
antisense	AL135999.2	ENSG00000273618.1	0	0

antisense	AL132800.1	ENSG00000258744.1	0	1
antisense	AL136018.1	ENSG00000258657.5	0	0
antisense	LINC02588	ENSG00000257842.5	0	0
antisense	FOXG1-AS1	ENSG00000257126.5	0	0
antisense	AL049777.1	ENSG00000274762.1	0	0
antisense	AL356756.1	ENSG00000257120.1	0	0
antisense	AL133372.3	ENSG00000257904.1	0	0
antisense	G2E3-AS1	ENSG00000257636.6	0	0
antisense	AL121852.1	ENSG00000258558.1	0	0
antisense	AL136418.1	ENSG00000257831.1	0	0
antisense	AL163973.3	ENSG00000258196.1	0	0
antisense	AL163973.2	ENSG00000257155.1	0	0
antisense	AL161665.1	ENSG00000285608.1	0	0
antisense	AL136298.1	ENSG00000258580.1	0	0
antisense	EGLN3-AS1	ENSG00000258897.1	0	0
antisense	AL445363.2	ENSG00000259135.1	0	0
antisense	AL162311.3	ENSG00000258938.1	0	1
antisense	NKX2-1-AS1	ENSG00000253563.2	0	0
antisense	AL162464.2	ENSG00000258690.1	0	0
antisense	AL162464.1	ENSG00000258601.1	0	0
antisense	AL359233.1	ENSG00000258696.1	0	0
antisense	AL392023.2	ENSG00000259048.1	0	0
antisense	AL132639.3	ENSG00000259083.1	0	0
antisense	AL356022.1	ENSG00000258747.1	0	0
antisense	AL049870.2	ENSG00000258633.1	0	0
antisense	AL049870.3	ENSG00000258949.1	0	0
antisense	AL121809.1	ENSG00000249163.3	0	0
antisense	AL133485.1	ENSG00000258843.1	0	0
antisense	AL358334.1	ENSG00000258687.1	0	0
antisense	AL358334.3	ENSG00000258745.1	0	0
antisense	AL591770.1	ENSG00000259055.1	0	0
antisense	AL358333.2	ENSG00000258928.1	0	0
antisense	AL358333.3	ENSG00000259007.1	0	0
antisense	AL358333.1	ENSG00000258854.1	0	0
antisense	AL139317.5	ENSG00000285664.1	0	0
antisense	AL352979.2	ENSG00000258985.1	0	0
antisense	AL356020.1	ENSG00000258731.1	0	1
antisense	AL158801.3	ENSG00000258455.1	0	0
antisense	AL161757.2	ENSG00000258428.5	0	0
antisense	AL161757.5	ENSG00000259133.5	0	0
antisense	AL161804.1	ENSG00000259039.2	0	0
antisense	AL139021.2	ENSG00000258658.1	0	0
antisense	AL139021.1	ENSG00000258378.1	0	0
antisense	AL133299.1	ENSG00000261120.1	0	0

antisense	AL157756.1	ENSG00000254718.6	0	2
antisense	AL049874.3	ENSG00000258670.1	0	0
antisense	AL355916.1	ENSG00000258926.1	0	0
antisense	AL122035.1	ENSG00000258824.2	0	1
antisense	AL049869.3	ENSG00000259116.2	0	0
antisense	AL049869.2	ENSG00000259076.1	0	0
antisense	AL139022.2	ENSG00000272158.1	0	1
antisense	AL139022.1	ENSG00000259118.5	0	0
antisense	AL049835.1	ENSG00000258490.1	0	0
antisense	AL133370.1	ENSG00000258837.1	0	1
antisense	AL121820.2	ENSG00000259038.1	0	0
antisense	AL121820.1	ENSG00000258623.1	0	0
antisense	ACTN1-AS1	ENSG00000259062.2	0	0
antisense	AL160191.1	ENSG00000258422.5	0	0
antisense	AC004974.1	ENSG00000285612.1	0	0
antisense	AC004900.1	ENSG00000285518.1	0	0
antisense	AL392024.1	ENSG00000285936.1	0	0
antisense	AL442663.3	ENSG00000258813.2	0	2
antisense	AC005225.3	ENSG00000258695.2	0	0
antisense	AC005225.2	ENSG00000258603.3	0	0
antisense	AC006146.1	ENSG00000258660.1	0	0
antisense	AC005520.2	ENSG00000259065.1	0	2
antisense	AC005480.1	ENSG00000258891.1	0	0
antisense	AL049780.2	ENSG00000259138.1	0	0
antisense	AF111167.1	ENSG00000258820.5	0	0
antisense	AC007182.1	ENSG00000224721.1	0	0
antisense	AF107885.2	ENSG00000259103.2	0	0
antisense	AC016526.4	ENSG00000283629.1	0	0
antisense	AF111169.3	ENSG00000259081.1	0	0
antisense	AC007375.2	ENSG00000269883.1	0	0
antisense	AF099810.1	ENSG00000258723.1	0	0
antisense	AC009396.2	ENSG00000258719.1	0	0
antisense	AC009396.3	ENSG00000258874.1	0	0
antisense	AC009396.1	ENSG00000258478.1	0	0
antisense	AC026888.1	ENSG00000258829.1	0	0
antisense	AC022469.2	ENSG00000258662.1	0	0
antisense	AC008056.2	ENSG00000259106.1	0	0
antisense	AC008056.1	ENSG00000258637.1	0	0
antisense	DIO2-AS1	ENSG00000258766.1	0	0
antisense	AC007262.2	ENSG00000284959.1	0	0
antisense	AL157955.1	ENSG00000258407.1	0	0
antisense	AL162171.2	ENSG00000258983.2	0	0
antisense	AL162171.1	ENSG00000258789.1	0	0
antisense	AL357093.2	ENSG00000258752.1	0	0

antisense	AL357093.1	ENSG00000258699.1	0	0
antisense	AL356805.1	ENSG00000258380.1	0	0
antisense	FOXN3-AS2	ENSG00000259073.1	0	0
antisense	AL512791.1	ENSG00000258424.1	0	0
antisense	AL096869.2	ENSG00000259163.1	0	0
antisense	AL139193.1	ENSG00000258437.1	0	0
antisense	AL139193.2	ENSG00000258716.1	0	0
antisense	ITPK1-AS1	ENSG00000258730.1	0	0
antisense	FAM181A-	ENSG00000258584.2	0	0
antisense	AL132642.1	ENSG00000258987.1	0	0
antisense	AL132708.1	ENSG00000256357.1	0	0
antisense	AL139020.1	ENSG00000257275.6	0	0
antisense	AL355102.5	ENSG00000259036.1	0	0
antisense	AL162151.1	ENSG00000229402.1	0	0
antisense	AL109767.1	ENSG00000235785.1	0	0
antisense	AL110504.1	ENSG00000258749.1	0	0
antisense	AL157912.1	ENSG00000258560.1	0	0
antisense	AL133523.1	ENSG00000258982.1	0	0
antisense	AL157871.4	ENSG00000258666.1	0	0
antisense	AL157871.2	ENSG00000258521.1	0	0
antisense	AL117190.2	ENSG00000258663.1	0	0
antisense	AL137779.2	ENSG00000259088.1	0	0
antisense	AL137779.1	ENSG00000256705.3	0	0
antisense	AL117209.1	ENSG00000259515.1	0	0
antisense	AL138976.2	ENSG00000259775.1	0	2
antisense	AL139300.2	ENSG00000258851.1	0	0
antisense	AL049840.1	ENSG00000246451.2	0	0
antisense	AL359399.1	ENSG00000258748.1	0	0
antisense	BX927359.1	ENSG00000259037.1	0	0
antisense	AL583722.4	ENSG00000258858.1	0	0
antisense	AL583722.2	ENSG00000258430.1	0	0
antisense	AL928654.2	ENSG00000257270.1	0	0
antisense	AC126407.1	ENSG00000260978.1	0	0
antisense	GABRG3-	ENSG00000228740.2	0	0
antisense	AC104002.1	ENSG00000258970.1	0	0
antisense	AC104002.3	ENSG00000259168.1	0	0
antisense	AC090696.1	ENSG00000232394.1	0	0
antisense	AC127522.1	ENSG00000259277.1	0	0
antisense	AC022613.2	ENSG00000259523.1	0	0
antisense	AC026150.3	ENSG00000270016.1	0	0
antisense	AC091057.2	ENSG00000247728.2	0	0
antisense	AC009562.1	ENSG00000259720.1	0	0
antisense	AC123768.5	ENSG00000285948.1	0	0
antisense	AC123768.1	ENSG00000241818.1	0	0

antisense	AC055874.1	ENSG00000259446.5	0	0
antisense	AC010809.3	ENSG00000276702.1	0	0
antisense	AC010809.1	ENSG00000259287.2	0	0
antisense	AC079203.2	ENSG00000259468.1	0	0
antisense	AC087457.1	ENSG00000250007.6	0	0
antisense	AC013640.1	ENSG00000261191.1	0	0
antisense	AC116158.1	ENSG00000259326.1	0	0
antisense	AC116158.2	ENSG00000259598.1	0	0
antisense	AC037198.2	ENSG00000278621.1	0	0
antisense	BMF-AS1	ENSG00000259409.1	0	0
antisense	PLCB2-AS1	ENSG00000259307.1	0	0
antisense	AC013356.4	ENSG00000259368.1	0	0
antisense	AC013356.3	ENSG00000259364.1	0	0
antisense	AC013356.2	ENSG00000259211.1	0	0
antisense	AC091045.1	ENSG00000259536.5	0	0
antisense	AC020661.3	ENSG00000259617.1	0	0
antisense	AC020661.2	ENSG00000259463.1	0	0
antisense	INO80-AS1	ENSG00000259521.1	0	0
antisense	AC020659.2	ENSG00000250379.1	0	0
antisense	AC020659.1	ENSG00000174171.5	0	0
antisense	EHD4-AS1	ENSG00000259883.1	0	0
antisense	PLA2G4E-	ENSG00000246740.2	0	0
antisense	AC039056.1	ENSG00000257797.1	0	0
antisense	AC036103.1	ENSG00000261002.5	0	0
antisense	AC018362.1	ENSG00000261684.2	0	0
antisense	AC018362.2	ENSG00000261822.1	0	0
antisense	AC090510.3	ENSG00000278769.1	0	0
antisense	AC090510.2	ENSG00000274403.1	0	0
antisense	AC068724.3	ENSG00000285080.1	0	0
antisense	AC068724.1	ENSG00000261687.1	0	0
antisense	AC009852.1	ENSG00000260406.1	0	0
antisense	AC025043.1	ENSG00000259595.1	0	0
antisense	AC025430.1	ENSG00000259563.1	0	0
antisense	AC091117.2	ENSG00000259418.1	0	0
antisense	AC091117.1	ENSG00000259352.1	0	0
antisense	AC051619.4	ENSG00000259519.1	0	0
antisense	AC025580.3	ENSG00000275672.1	0	0
antisense	AC025580.1	ENSG00000259342.1	0	0
antisense	AC025580.2	ENSG00000259354.5	0	0
antisense	AC023905.1	ENSG00000259221.5	0	0
antisense	AC009558.1	ENSG00000259360.1	0	0
antisense	AC012050.1	ENSG00000259588.1	0	0
antisense	AC066612.2	ENSG00000259385.1	0	0
antisense	AC023355.1	ENSG00000259488.2	0	0

antisense	AC012379.1	ENSG00000259670.1	0	0
antisense	AC091073.1	ENSG00000259602.1	0	0
antisense	AC025040.1	ENSG00000259188.5	0	1
antisense	AC012170.2	ENSG00000259298.1	0	0
antisense	AC084756.1	ENSG00000259684.1	0	0
antisense	AC012100.2	ENSG00000259773.1	0	0
antisense	AC066613.2	ENSG00000259678.1	0	0
antisense	AC020892.2	ENSG00000259296.1	0	0
antisense	AC090971.2	ENSG00000259201.1	0	0
antisense	AC090971.1	ENSG00000259185.1	0	0
antisense	AC023906.5	ENSG00000259712.1	0	0
antisense	AC023906.4	ENSG00000259709.1	0	0
antisense	AC025917.1	ENSG00000260618.1	0	0
antisense	AC012378.2	ENSG00000285805.1	0	0
antisense	AC012378.1	ENSG00000259180.1	0	0
antisense	AC025431.1	ENSG00000259285.1	0	0
antisense	LIPC-AS1	ENSG00000259293.1	0	0
antisense	AC018904.1	ENSG00000259250.1	0	0
antisense	AC090515.4	ENSG00000259353.1	0	0
antisense	AC025918.1	ENSG00000225798.1	0	0
antisense	RORA-AS1	ENSG00000245534.6	0	0
antisense	AC107241.1	ENSG00000259274.1	0	0
antisense	RORA-AS2	ENSG00000259482.1	0	0
antisense	AC022898.1	ENSG00000259591.1	0	0
antisense	AC012404.1	ENSG00000259481.1	0	0
antisense	AC012404.2	ENSG00000259575.1	0	0
antisense	AC009554.1	ENSG00000259564.2	0	0
antisense	AC009554.2	ENSG00000285863.1	0	0
antisense	MGC15885	ENSG00000259458.1	0	0
antisense	AC100839.1	ENSG00000259756.1	0	0
antisense	TPM1-AS	ENSG00000259498.1	0	0
antisense	AC079328.2	ENSG00000259627.1	0	0
antisense	AC073167.1	ENSG00000259589.2	0	0
antisense	AC015914.1	ENSG00000259351.1	0	0
antisense	AC100830.2	ENSG00000264937.1	0	0
antisense	AC100830.1	ENSG00000259635.1	0	0
antisense	AC103691.2	ENSG00000275332.1	0	0
antisense	AC013553.3	ENSG00000277351.1	0	0
antisense	AC011939.3	ENSG00000275638.1	0	0
antisense	AC055855.2	ENSG00000261318.1	0	0
antisense	AC055855.1	ENSG00000260773.1	0	0
antisense	AC087482.1	ENSG00000259347.6	0	0
antisense	AC012568.1	ENSG00000259202.1	0	0
antisense	AC087639.2	ENSG00000285919.1	0	0

antisense	AC027088.3	ENSG00000259265.1	0	0
antisense	AC027237.3	ENSG00000259426.5	0	0
antisense	AC009269.3	ENSG00000259624.1	0	0
antisense	THSD4-AS1	ENSG00000259964.6	0	0
antisense	AC108861.1	ENSG00000278408.1	0	0
antisense	AC020779.2	ENSG00000260173.1	0	0
antisense	AC009690.2	ENSG00000261460.1	0	0
antisense	AC068397.1	ENSG00000259528.1	0	0
antisense	AC068397.2	ENSG00000259650.1	0	0
antisense	AC018943.1	ENSG00000260624.1	0	0
antisense	AC010931.3	ENSG00000261543.1	0	0
antisense	AC023300.2	ENSG00000277749.1	0	0
antisense	AC100835.1	ENSG00000260919.1	0	0
antisense	AC113208.3	ENSG00000261779.1	0	0
antisense	AC105020.3	ENSG00000260235.1	0	0
antisense	AC105020.2	ENSG00000260206.1	0	0
antisense	AC105020.5	ENSG00000274515.1	0	0
antisense	AC105020.4	ENSG00000260892.1	0	0
antisense	DNM1P35	ENSG00000246877.1	0	0
antisense	AC091100.1	ENSG00000259422.1	0	0
antisense	AC027243.1	ENSG00000259514.1	0	0
antisense	LINGO1-AS1	ENSG00000259666.2	0	0
antisense	LINGO1-AS2	ENSG00000259281.1	0	0
antisense	AC105133.1	ENSG00000259213.1	0	0
antisense	AC104758.2	ENSG00000259792.1	0	0
antisense	AC090607.3	ENSG00000259708.1	0	0
antisense	AC090607.1	ENSG00000259322.1	0	0
antisense	AC090607.5	ENSG00000277482.1	0	0
antisense	AC027228.1	ENSG00000259474.1	0	0
antisense	AC027228.2	ENSG00000261762.1	0	0
antisense	AC067863.1	ENSG00000259555.1	0	0
antisense	AC011944.1	ENSG00000177699.4	0	0
antisense	AC016705.1	ENSG00000258010.4	0	0
antisense	AC108451.1	ENSG00000259175.1	0	0
antisense	AC027808.1	ENSG00000259546.1	0	0
antisense	AC027808.2	ENSG00000259649.4	0	0
antisense	TMC3-AS1	ENSG00000259343.6	0	0
antisense	AC026624.1	ENSG00000285974.1	0	0
antisense	CPEB1-AS1	ENSG00000259462.2	0	2
antisense	AC105339.3	ENSG00000259442.1	0	0
antisense	SNHG21	ENSG00000250988.7	0	0
antisense	AC022558.2	ENSG00000259805.1	0	0
antisense	AC024270.4	ENSG00000260608.1	0	0
antisense	AC024270.3	ENSG00000260579.1	0	0

antisense	AC103876.1	ENSG00000259986.1	0	0
antisense	AC048382.5	ENSG00000275120.2	0	0
antisense	AC048382.1	ENSG00000256278.1	0	0
antisense	AC115102.1	ENSG00000259654.1	0	0
antisense	AC087286.3	ENSG00000259375.1	0	0
antisense	AC087286.2	ENSG00000259367.1	0	0
antisense	AC087286.1	ENSG00000259276.1	0	0
antisense	AC021739.4	ENSG00000259544.1	0	0
antisense	AC021739.2	ENSG00000259407.1	0	0
antisense	AC021739.5	ENSG00000259762.1	0	0
antisense	AC021739.3	ENSG00000259416.2	0	0
antisense	LINC01584	ENSG00000260477.7	0	0
antisense	AGBL1-AS1	ENSG00000260125.1	0	0
antisense	AC012229.1	ENSG00000259734.1	0	0
antisense	AC016987.1	ENSG00000259620.1	0	0
antisense	AC013565.3	ENSG00000261407.1	0	0
antisense	AC013391.1	ENSG00000259615.1	0	0
antisense	AC018946.1	ENSG00000259177.1	0	0
antisense	AC103739.1	ENSG00000259212.1	0	0
antisense	AC103739.2	ENSG00000259314.1	0	0
antisense	CRTC3-AS1	ENSG00000259736.1	0	0
antisense	AC103739.3	ENSG00000285560.1	0	0
antisense	AC068831.4	ENSG00000259661.1	0	0
antisense	AC068831.1	ENSG00000258384.1	0	0
antisense	AC116903.2	ENSG00000260661.1	0	0
antisense	AC116903.1	ENSG00000258761.1	0	0
antisense	AC091544.5	ENSG00000271763.1	0	0
antisense	AC091544.7	ENSG00000275965.1	0	0
antisense	SPATA8-AS1	ENSG00000259282.5	0	0
antisense	AC069029.1	ENSG00000259621.1	0	0
antisense	AC036108.1	ENSG00000259475.1	0	0
antisense	AC036108.2	ENSG00000261054.1	0	0
antisense	AC036108.3	ENSG00000261616.1	0	0
antisense	AC015660.2	ENSG00000259760.1	0	0
antisense	AC084855.1	ENSG00000254744.3	0	0
antisense	AC084855.2	ENSG00000259219.1	0	0
antisense	AC022710.1	ENSG00000259356.1	0	0
antisense	CERS3-AS1	ENSG00000259430.1	0	0
antisense	AC027020.1	ENSG00000259540.1	0	0
antisense	AC015712.7	ENSG00000278456.1	0	0
antisense	AC090907.1	ENSG00000259376.1	0	0
antisense	AC090907.2	ENSG00000259755.1	0	0
antisense	AC023024.1	ENSG00000259172.1	0	0
antisense	PCSK6-AS1	ENSG00000259764.1	0	0

antisense	Z69720.1	ENSG00000269482.1	0	0
antisense	Z69666.1	ENSG00000228779.1	0	0
antisense	Z97986.1	ENSG00000261691.1	0	0
antisense	AL022341.1	ENSG00000228201.1	0	0
antisense	AL022341.2	ENSG00000262528.2	0	0
antisense	Z92544.1	ENSG00000260394.2	0	0
antisense	AL031716.1	ENSG00000260022.1	0	0
antisense	LMF1-AS1	ENSG00000260439.1	0	0
antisense	AC120498.3	ENSG00000260403.1	0	0
antisense	AC120498.1	ENSG00000259910.1	0	0
antisense	AC120498.6	ENSG00000261294.1	0	0
antisense	AC120498.10	ENSG00000277010.1	0	0
antisense	AC120498.9	ENSG00000274751.1	0	1
antisense	AL031714.1	ENSG00000261505.1	0	0
antisense	AL031709.1	ENSG00000260425.1	0	0
antisense	AL032819.1	ENSG00000260132.1	0	0
antisense	AL031600.3	ENSG00000261641.2	0	0
antisense	AL031600.1	ENSG00000260051.1	0	0
antisense	AL133297.2	ENSG00000260989.1	0	0
antisense	Z97652.1	ENSG00000226890.1	0	0
antisense	AL031710.1	ENSG00000261399.1	0	0
antisense	AL031717.1	ENSG00000261207.1	0	0
antisense	AC009065.6	ENSG00000261240.1	0	0
antisense	AC009065.3	ENSG00000260447.1	0	0
antisense	AC009065.8	ENSG00000261663.1	0	0
antisense	AC106820.6	ENSG00000285970.1	0	0
antisense	AC106820.2	ENSG00000259895.1	0	0
antisense	AC093525.6	ENSG00000261613.2	0	0
antisense	AC093525.7	ENSG00000269937.1	0	0
antisense	AC093525.4	ENSG00000261140.1	0	0
antisense	AC093525.3	ENSG00000260436.1	0	0
antisense	AC141586.3	ENSG00000261093.1	0	0
antisense	AC003965.2	ENSG00000263325.1	0	0
antisense	AC004034.1	ENSG00000262482.1	0	0
antisense	AC108134.1	ENSG00000205890.3	0	0
antisense	AJ003147.2	ENSG00000262668.1	0	0
antisense	AC004494.1	ENSG00000262312.2	0	0
antisense	AC005736.2	ENSG00000263159.1	0	0
antisense	AC023830.2	ENSG00000261789.1	0	0
antisense	AC023830.1	ENSG00000261442.1	0	0
antisense	AC020663.2	ENSG00000267077.1	0	0
antisense	LINC01570	ENSG00000260338.1	0	0
antisense	AC005774.1	ENSG00000261198.1	0	0
antisense	AC022167.1	ENSG00000259939.1	0	0

antisense	AC022167.4	ENSG00000261481.1	0	2
antisense	AC087190.1	ENSG00000260349.1	0	0
antisense	AC007218.2	ENSG00000283221.1	0	0
antisense	AC133565.1	ENSG00000261810.1	0	0
antisense	AC027277.1	ENSG00000256013.1	0	0
antisense	AC007595.1	ENSG00000263279.1	0	0
antisense	AC133065.1	ENSG00000262151.1	0	0
antisense	AC007014.1	ENSG00000262020.1	0	0
antisense	AC009121.1	ENSG00000262703.1	0	0
antisense	AC099489.3	ENSG00000262999.1	0	0
antisense	AC007613.1	ENSG00000262420.3	0	0
antisense	AC007216.4	ENSG00000263307.1	0	0
antisense	AC007216.2	ENSG00000261216.1	0	0
antisense	AC007216.1	ENSG00000260488.1	0	0
antisense	AC007601.1	ENSG00000175604.2	0	0
antisense	AC007601.2	ENSG00000261293.1	0	0
antisense	AC007598.2	ENSG00000261394.1	0	0
antisense	AC010333.1	ENSG00000259876.1	0	0
antisense	AC010333.2	ENSG00000259899.1	0	0
antisense	AC109597.1	ENSG00000260378.1	0	0
antisense	AC109597.2	ENSG00000261158.1	0	0
antisense	AC009134.1	ENSG00000262116.1	0	0
antisense	AC003009.1	ENSG00000262011.1	0	0
antisense	AC010401.1	ENSG00000262732.1	0	0
antisense	AC130650.1	ENSG00000262529.1	0	0
antisense	AC009167.1	ENSG00000261523.1	0	0
antisense	AC136443.4	ENSG00000261695.1	0	0
antisense	AC138932.5	ENSG00000275910.1	0	1
antisense	AC026401.1	ENSG00000257769.1	0	0
antisense	AC130651.1	ENSG00000262171.1	0	0
antisense	AC136624.1	ENSG00000262332.1	0	0
antisense	AC136624.2	ENSG00000262848.1	0	0
antisense	AC109446.3	ENSG00000261448.1	0	0
antisense	AC138811.1	ENSG00000260017.1	0	0
antisense	AC092287.1	ENSG00000260352.1	0	0
antisense	AC099518.5	ENSG00000261759.1	0	0
antisense	AC130456.4	ENSG00000260681.1	0	0
antisense	AC130456.5	ENSG00000260934.1	0	0
antisense	AC027130.1	ENSG00000261195.1	0	0
antisense	AC106796.1	ENSG00000263237.1	0	0
antisense	AC137056.1	ENSG00000262995.1	0	0
antisense	AC004381.1	ENSG00000260510.1	0	0
antisense	AC008551.1	ENSG00000263331.1	0	0
antisense	AF001550.1	ENSG00000262983.1	0	0

antisense	AC092119.3	ENSG00000275445.1	0	0
antisense	AC009019.1	ENSG00000260277.1	0	0
antisense	AC009034.1	ENSG00000261113.1	0	0
antisense	AC092338.1	ENSG00000260635.1	0	0
antisense	AC092338.2	ENSG00000260790.1	0	0
antisense	AC099482.1	ENSG00000260741.1	0	0
antisense	AC008870.4	ENSG00000261723.1	0	0
antisense	AC008870.3	ENSG00000261266.2	0	0
antisense	AC012317.1	ENSG00000261583.1	0	0
antisense	AC008731.1	ENSG00000261669.1	0	0
antisense	LCMT1-AS2	ENSG00000260034.1	0	0
antisense	AC008741.1	ENSG00000259955.1	0	1
antisense	AC092725.1	ENSG00000261482.1	0	0
antisense	AC109449.1	ENSG00000259940.2	0	1
antisense	AC106739.1	ENSG00000274092.1	0	2
antisense	IL21R-AS1	ENSG00000259954.1	0	0
antisense	AC016597.1	ENSG00000261329.5	0	0
antisense	AC133550.1	ENSG00000260570.1	0	0
antisense	AC109460.2	ENSG00000260853.1	0	0
antisense	AC109460.1	ENSG00000260367.2	0	1
antisense	AC109460.4	ENSG00000261552.1	0	0
antisense	AC009133.3	ENSG00000260719.1	0	0
antisense	AC009133.4	ENSG00000275857.1	0	0
antisense	AC009133.2	ENSG00000259952.1	0	0
antisense	AC120114.2	ENSG00000278713.1	0	0
antisense	AC093512.1	ENSG00000274904.1	0	0
antisense	AC012645.1	ENSG00000250616.2	0	2
antisense	AC012645.2	ENSG00000261367.1	0	0
antisense	AC012645.4	ENSG00000275371.1	0	0
antisense	AC012645.3	ENSG00000261416.1	0	0
antisense	AC116348.3	ENSG00000261346.1	0	0
antisense	AC116348.1	ENSG00000260487.1	0	0
antisense	AC116348.2	ENSG00000261332.1	0	0
antisense	AC002310.2	ENSG00000239791.1	0	0
antisense	AC093249.2	ENSG00000260167.1	0	0
antisense	AC135050.4	ENSG00000261124.1	0	0
antisense	AC009088.3	ENSG00000261385.1	0	0
antisense	AC009088.2	ENSG00000260304.1	0	0
antisense	AC009088.1	ENSG00000260060.1	0	0
antisense	AC026471.5	ENSG00000277543.1	0	0
antisense	AC026471.3	ENSG00000260740.2	0	0
antisense	AC026471.2	ENSG00000260625.2	0	0
antisense	AC074050.3	ENSG00000261731.2	0	0
antisense	AC133485.3	ENSG00000260575.1	0	0

antisense	AC138907.3	ENSG00000260974.1	0	0
antisense	AC138869.2	ENSG00000260419.1	0	0
antisense	ITFG1-AS1	ENSG00000260281.5	0	0
antisense	AC007494.2	ENSG00000260744.1	0	0
antisense	AC007533.1	ENSG00000261369.1	0	0
antisense	AC026470.2	ENSG00000261267.1	0	0
antisense	AC007614.1	ENSG00000279249.2	0	0
antisense	AC007610.1	ENSG00000259843.2	0	0
antisense	AC007610.2	ENSG00000260381.2	0	0
antisense	AC007493.2	ENSG00000261393.1	0	0
antisense	AC007608.2	ENSG00000260029.2	0	0
antisense	AC007608.1	ENSG00000205414.1	0	0
antisense	AC007608.3	ENSG00000260249.2	0	0
antisense	AC007728.2	ENSG00000261644.2	0	0
antisense	AC007728.1	ENSG00000260616.6	0	0
antisense	AC009166.1	ENSG00000261238.1	0	0
antisense	AC009039.1	ENSG00000261470.1	0	0
antisense	AC007496.2	ENSG00000261630.1	0	0
antisense	AC007496.1	ENSG00000260194.1	0	0
antisense	AC007347.1	ENSG00000261049.2	0	0
antisense	AC109462.1	ENSG00000259283.2	0	0
antisense	DKFZP434H1	ENSG00000261439.1	0	0
antisense	AC009102.2	ENSG00000272372.1	0	0
antisense	AC092140.1	ENSG00000260621.1	0	0
antisense	AC106779.1	ENSG00000261302.5	0	0
antisense	AC023825.2	ENSG00000260145.1	0	0
antisense	AC009090.2	ENSG00000260148.1	0	0
antisense	AC009090.6	ENSG00000285979.1	0	0
antisense	AC009090.3	ENSG00000276663.1	0	0
antisense	AC018552.3	ENSG00000261633.1	0	0
antisense	AC018552.2	ENSG00000260467.1	0	0
antisense	AC092118.2	ENSG00000276166.1	0	0
antisense	AC026771.1	ENSG00000260545.1	0	0
antisense	AC009107.2	ENSG00000260927.1	0	0
antisense	AC009107.1	ENSG00000260867.1	0	0
antisense	AC009118.1	ENSG00000261078.1	0	0
antisense	AC012174.1	ENSG00000260115.1	0	0
antisense	BEAN1-AS1	ENSG00000261656.5	0	0
antisense	AC010542.1	ENSG00000260650.1	0	0
antisense	AC018557.1	ENSG00000260465.1	0	0
antisense	AC027682.5	ENSG00000261396.1	0	0
antisense	AC027682.2	ENSG00000259945.1	0	0
antisense	AC027682.3	ENSG00000260894.1	0	0
antisense	AC027682.1	ENSG00000259804.1	0	0

antisense	AC009095.1	ENSG00000237718.2	0	0
antisense	AC020978.3	ENSG00000260891.1	0	0
antisense	AC020978.2	ENSG00000260441.5	0	0
antisense	AC126773.1	ENSG00000260084.1	0	0
antisense	AC099314.1	ENSG00000260798.1	0	0
antisense	AC092115.2	ENSG00000261602.1	0	0
antisense	AC092115.3	ENSG00000262136.1	0	0
antisense	AC009060.1	ENSG00000247228.2	0	0
antisense	AC012184.4	ENSG00000285710.1	0	0
antisense	AC012184.1	ENSG00000260111.1	0	0
antisense	AC138625.2	ENSG00000259833.5	0	0
antisense	AC138625.1	ENSG00000259798.1	0	0
antisense	AC010547.1	ENSG00000247324.2	0	0
antisense	AC010547.3	ENSG00000260520.1	0	0
antisense	TAT-AS1	ENSG00000260886.1	0	0
antisense	AC009097.2	ENSG00000260593.1	0	0
antisense	AC009097.1	ENSG00000260185.1	0	0
antisense	AC009097.4	ENSG00000261513.1	0	0
antisense	AC009087.1	ENSG00000260252.1	0	0
antisense	AC009053.3	ENSG00000261170.1	0	0
antisense	AC099508.1	ENSG00000247033.1	0	0
antisense	AC009163.1	ENSG00000203472.3	0	0
antisense	AC009139.2	ENSG00000261063.1	0	0
antisense	AC009139.1	ENSG00000260922.2	0	0
antisense	AC025284.1	ENSG00000260701.1	0	0
antisense	AC092134.1	ENSG00000261707.1	0	0
antisense	AC079414.1	ENSG00000261540.1	0	0
antisense	WWOX-AS1	ENSG00000260969.1	0	0
antisense	AC046158.1	ENSG00000260733.1	0	0
antisense	AC009145.3	ENSG00000260694.1	0	0
antisense	AC108097.1	ENSG00000260183.1	0	0
antisense	AC099313.1	ENSG00000260594.1	0	0
antisense	AC092718.5	ENSG00000261141.1	0	0
antisense	AC092142.1	ENSG00000261235.1	0	0
antisense	AC125793.1	ENSG00000260832.1	0	0
antisense	AC009142.1	ENSG00000261410.1	0	0
antisense	AC009063.3	ENSG00000261103.1	0	0
antisense	AC009063.2	ENSG00000260788.5	0	0
antisense	AC009063.1	ENSG00000259914.1	0	0
antisense	AC009119.1	ENSG00000260228.5	0	0
antisense	AC009119.3	ENSG00000260932.1	0	0
antisense	AC040169.4	ENSG00000285792.1	0	0
antisense	AC010551.2	ENSG00000260530.1	0	0
antisense	AC022165.1	ENSG00000261243.1	0	0

antisense	AC092145.1	ENSG00000261471.1	0	0
antisense	AC025280.3	ENSG00000285848.1	0	0
antisense	AC025280.2	ENSG00000279622.2	0	0
antisense	AC018695.2	ENSG00000269898.1	0	0
antisense	AC018695.4	ENSG00000270184.1	0	0
antisense	AC018695.3	ENSG00000270159.1	0	0
antisense	AC136285.2	ENSG00000261651.1	0	0
antisense	AC010531.7	ENSG00000270082.1	0	0
antisense	AC010531.5	ENSG00000269901.1	0	0
antisense	AC010531.3	ENSG00000261592.1	0	0
antisense	AC092720.2	ENSG00000269935.1	0	0
antisense	AC126696.2	ENSG00000260466.1	0	0
antisense	AC116552.1	ENSG00000261744.1	0	0
antisense	AC138028.3	ENSG00000259813.1	0	0
antisense	AC138028.6	ENSG00000278341.1	0	0
antisense	AC138028.4	ENSG00000260121.1	0	0
antisense	AC138028.2	ENSG00000224888.4	0	1
antisense	AC138028.5	ENSG00000260617.1	0	0
antisense	AC138028.1	ENSG00000182376.2	0	0
antisense	AC092384.2	ENSG00000259881.1	0	0
antisense	AC092384.1	ENSG00000205018.2	0	0
antisense	AC135782.3	ENSG00000261546.1	0	0
antisense	AC009113.2	ENSG00000260659.1	0	0
antisense	AC137932.3	ENSG00000268218.1	0	0
antisense	AC137932.1	ENSG00000260279.3	0	0
antisense	VPS9D1-AS1	ENSG00000261373.1	0	0
antisense	AC092143.2	ENSG00000259006.1	0	0
antisense	GAS8-AS1	ENSG00000221819.6	0	0
antisense	AC129507.4	ENSG00000262920.5	0	0
antisense	AC129507.2	ENSG00000262294.1	0	0
antisense	AC129507.3	ENSG00000262558.1	0	0
antisense	AC015853.2	ENSG00000263015.1	0	0
antisense	AC015853.3	ENSG00000263300.1	0	0
antisense	AC015853.1	ENSG00000262905.1	0	0
antisense	AC087392.4	ENSG00000262434.1	0	0
antisense	AC087392.2	ENSG00000262133.1	0	0
antisense	AC016292.1	ENSG00000235361.1	0	0
antisense	AC032044.1	ENSG00000262777.1	0	0
antisense	AC130343.1	ENSG00000262791.1	0	0
antisense	AC099684.2	ENSG00000262445.3	0	0
antisense	AC099684.1	ENSG00000228133.2	0	0
antisense	AC090617.3	ENSG00000262533.1	0	0
antisense	AC090617.4	ENSG00000262810.1	0	0
antisense	AC090617.2	ENSG00000236838.2	0	0

antisense	AL450226.1	ENSG00000225084.1	0	0
antisense	AC006435.3	ENSG00000274758.1	0	0
antisense	AC006435.2	ENSG00000263345.1	0	0
antisense	AC006435.1	ENSG00000262456.1	0	0
antisense	AC005696.2	ENSG00000272770.1	0	0
antisense	AC005696.1	ENSG00000262050.1	0	0
antisense	AC015921.1	ENSG00000262884.1	0	0
antisense	AC027796.4	ENSG00000262903.1	0	2
antisense	AC116914.2	ENSG00000262692.1	0	0
antisense	AC116914.1	ENSG00000262358.1	0	0
antisense	AC087292.1	ENSG00000263165.1	0	0
antisense	AC127521.1	ENSG00000262823.1	0	2
antisense	AC118754.1	ENSG00000229782.1	0	0
antisense	AC091153.2	ENSG00000235085.3	0	0
antisense	AC091153.4	ENSG00000261898.2	0	0
antisense	AC004771.4	ENSG00000262429.1	0	1
antisense	AC004771.2	ENSG00000234203.1	0	0
antisense	AC004771.5	ENSG00000262678.1	0	0
antisense	AC004771.3	ENSG00000262227.1	0	0
antisense	AC012146.3	ENSG00000262693.1	0	0
antisense	AC040977.2	ENSG00000267047.1	0	0
antisense	MIR497HG	ENSG00000267532.5	0	3
antisense	AC003688.2	ENSG00000263342.1	0	0
antisense	AC026954.3	ENSG00000263171.1	0	0
antisense	AC113189.1	ENSG00000262624.1	0	0
antisense	AC113189.3	ENSG00000263301.1	0	0
antisense	AC016876.1	ENSG00000233223.2	0	2
antisense	AC129492.1	ENSG00000214999.3	0	0
antisense	AC135178.1	ENSG00000226871.1	0	0
antisense	AC135178.3	ENSG00000265749.5	0	0
antisense	AC005695.1	ENSG00000262966.2	0	0
antisense	AC005695.2	ENSG00000263708.2	0	0
antisense	AC087501.1	ENSG00000225751.2	0	0
antisense	AC087501.2	ENSG00000262815.1	0	0
antisense	AC087501.3	ENSG00000263051.1	0	0
antisense	AC118755.2	ENSG00000265349.1	0	0
antisense	AC118755.1	ENSG00000262296.1	0	0
antisense	AC027045.3	ENSG00000283025.1	0	0
antisense	AC005291.2	ENSG00000273388.1	0	0
antisense	AC005291.1	ENSG00000264067.1	0	0
antisense	AC005323.2	ENSG00000272736.5	0	0
antisense	MYHAS	ENSG00000272975.1	0	0
antisense	AC005323.1	ENSG00000214970.8	0	0
antisense	AC002347.2	ENSG00000263388.1	0	0

antisense	TMEM220-	ENSG00000263400.7	0	0
antisense	AC005548.1	ENSG00000285541.1	0	0
antisense	AC005209.1	ENSG00000263684.1	0	0
antisense	AC005410.2	ENSG00000266368.1	0	0
antisense	AC005358.1	ENSG00000227274.1	0	0
antisense	AC005358.2	ENSG00000265489.1	0	0
antisense	AC005277.2	ENSG00000277621.1	0	0
antisense	AC005277.1	ENSG00000263707.1	0	0
antisense	AC005703.3	ENSG00000265445.1	0	0
antisense	AC005324.5	ENSG00000266261.1	0	0
antisense	AC005324.1	ENSG00000233002.2	0	0
antisense	AC093484.3	ENSG00000265401.1	0	0
antisense	AC093484.2	ENSG00000264739.1	0	0
antisense	AC098850.1	ENSG00000264673.1	0	0
antisense	MPRIP-AS1	ENSG00000225442.2	0	0
antisense	AC055811.1	ENSG00000263624.1	0	0
antisense	AC020558.2	ENSG00000265511.1	0	0
antisense	AC020558.1	ENSG00000264666.1	0	2
antisense	RAI1-AS1	ENSG00000237328.1	0	0
antisense	AC122129.1	ENSG00000197815.4	0	0
antisense	AC087164.1	ENSG00000266677.1	0	3
antisense	AL353997.2	ENSG00000264177.1	0	0
antisense	AC107982.2	ENSG00000264273.1	0	0
antisense	AC090286.1	ENSG00000196893.3	0	0
antisense	AC007952.6	ENSG00000262319.1	0	0
antisense	AC007952.1	ENSG00000197665.7	0	0
antisense	EPN2-AS1	ENSG00000235397.1	0	0
antisense	AC004448.3	ENSG00000265126.1	0	0
antisense	AC025627.1	ENSG00000262769.1	0	0
antisense	AC115989.1	ENSG00000264932.3	0	0
antisense	AC005722.3	ENSG00000264785.1	0	0
antisense	AC005730.2	ENSG00000261033.1	0	0
antisense	CCDC144NL-AS1	ENSG00000233098.8	0	0
antisense	AC107926.1	ENSG00000264660.2	0	0
antisense	AC087393.2	ENSG00000263986.1	0	0
antisense	AC087294.1	ENSG00000235530.6	0	0
antisense	AC233702.7	ENSG00000266466.1	0	0
antisense	AC132825.2	ENSG00000243655.2	0	0
antisense	AC015688.6	ENSG00000266872.1	0	0
antisense	AC005697.2	ENSG00000266527.1	0	0
antisense	AC002094.2	ENSG00000265618.1	0	0
antisense	AC005726.4	ENSG00000265168.1	0	0

antisense	AC005726.2	ENSG00000264044.1	0	0
antisense	AC010761.3	ENSG00000265205.1	0	0
antisense	AC010761.1	ENSG00000264577.1	0	0
antisense	AC010761.6	ENSG00000267729.1	0	0
antisense	AC010761.2	ENSG00000265073.1	0	0
antisense	AC010761.4	ENSG00000265474.1	0	0
antisense	AC010761.5	ENSG00000265840.1	0	0
antisense	AC024267.3	ENSG00000264304.1	0	0
antisense	AC024267.6	ENSG00000266642.2	0	0
antisense	AC024267.4	ENSG00000265845.2	0	0
antisense	AC024267.5	ENSG00000265908.1	0	0
antisense	AC024619.2	ENSG00000263613.1	0	0
antisense	AC024619.3	ENSG00000263709.1	0	0
antisense	AC068025.1	ENSG00000264808.1	0	0
antisense	ABHD15-AS1	ENSG00000264031.1	0	0
antisense	AC104564.4	ENSG00000264647.1	0	0
antisense	AC104564.1	ENSG00000263370.1	0	0
antisense	AC023389.1	ENSG00000263657.1	0	0
antisense	AC104982.1	ENSG00000263477.1	0	2
antisense	AC104996.1	ENSG00000265289.1	0	0
antisense	AC104984.2	ENSG00000265394.1	0	0
antisense	AC104984.3	ENSG00000265739.1	0	0
antisense	AC104984.6	ENSG00000266987.1	0	0
antisense	AC104984.1	ENSG00000264125.1	0	0
antisense	AC104984.4	ENSG00000266120.1	0	0
antisense	AC011840.4	ENSG00000266775.1	0	0
antisense	AC130324.2	ENSG00000265334.1	0	0
antisense	AC138207.1	ENSG00000230113.1	0	0
antisense	AC026620.1	ENSG00000263674.1	0	0
antisense	AC005899.8	ENSG00000279762.3	0	0
antisense	AC079336.5	ENSG00000266718.2	0	1
antisense	AC079336.4	ENSG00000266599.1	0	0
antisense	AC025211.1	ENSG00000264458.1	0	0
antisense	AC084809.1	ENSG00000226377.1	0	0
antisense	AC008133.1	ENSG00000266535.1	0	0
antisense	AC011824.3	ENSG00000265697.1	0	0
antisense	AC011824.2	ENSG00000265125.5	0	0
antisense	AC011824.1	ENSG00000265115.1	0	0
antisense	AC024610.1	ENSG00000263435.1	0	0
antisense	AC004147.3	ENSG00000264791.1	0	0
antisense	AC022903.2	ENSG00000285559.1	0	0
antisense	AC004223.3	ENSG00000273687.1	0	0
antisense	AC022916.4	ENSG00000267782.1	0	0
antisense	AC015911.5	ENSG00000267349.1	0	0

antisense	AC015911.6	ENSG00000267359.1	0	0
antisense	AC015849.1	ENSG00000270240.2	0	0
antisense	AC244100.2	ENSG00000275431.1	0	0
antisense	AC244100.3	ENSG00000275944.1	0	0
antisense	AC243829.4	ENSG00000277089.4	0	0
antisense	AC243829.2	ENSG00000276241.1	0	0
antisense	AC243773.1	ENSG00000276707.1	0	0
antisense	AC244093.5	ENSG00000278638.1	0	0
antisense	AC243654.1	ENSG00000273965.1	0	0
antisense	AC243585.2	ENSG00000277688.1	0	0
antisense	AC243571.2	ENSG00000277501.1	0	0
antisense	AC006449.1	ENSG00000274996.1	0	0
antisense	AC006441.1	ENSG00000263466.1	0	0
antisense	AC006441.3	ENSG00000265784.1	0	0
antisense	AC004408.1	ENSG00000266101.1	0	0
antisense	AC005288.1	ENSG00000266469.1	0	2
antisense	AC080112.1	ENSG00000266208.1	0	0
antisense	AC004231.3	ENSG00000265359.1	0	0
antisense	AC004231.1	ENSG00000234477.1	0	1
antisense	AC099811.4	ENSG00000267758.1	0	0
antisense	AC100793.3	ENSG00000267765.1	0	0
antisense	AC003098.1	ENSG00000267604.1	0	0
antisense	AC007993.3	ENSG00000282199.1	0	0
antisense	AC023855.1	ENSG00000267638.1	0	0
antisense	AC004596.1	ENSG00000267394.1	0	0
antisense	MAP3K14-	ENSG00000267278.5	0	0
antisense	AC003070.1	ENSG00000267344.1	0	0
antisense	AC091132.2	ENSG00000236234.1	0	0
antisense	MAPT-AS1	ENSG00000264589.3	0	0
antisense	CR936218.2	ENSG00000262881.1	0	0
antisense	CR936218.1	ENSG00000262372.1	0	0
antisense	AC002558.2	ENSG00000261872.1	0	0
antisense	AC068234.2	ENSG00000276790.1	0	0
antisense	THCAT158	ENSG00000263293.2	0	0
antisense	AC040934.1	ENSG00000253347.1	0	0
antisense	AC025682.1	ENSG00000263766.5	0	0
antisense	AC015674.1	ENSG00000264558.1	0	0
antisense	AC018521.6	ENSG00000266601.1	0	0
antisense	AC018521.2	ENSG00000264019.1	0	0
antisense	AC018521.4	ENSG00000264701.1	0	0
antisense	AC004477.2	ENSG00000266341.1	0	0
antisense	SKAP1-AS1	ENSG00000263787.1	0	0
antisense	THRA1/BTR	ENSG00000235300.4	0	0
antisense	HOXB-AS1	ENSG00000230148.8	0	0

antisense	HOXB-AS3	ENSG00000233101.10	0	0
antisense	HOXB-AS2	ENSG00000239552.2	0	0
antisense	HOXB-AS4	ENSG00000242207.1	0	0
antisense	AC091133.2	ENSG00000250838.1	0	0
antisense	AC091180.5	ENSG00000262039.1	0	0
antisense	AC091180.4	ENSG00000250948.1	0	0
antisense	AC091180.3	ENSG00000250186.3	0	0
antisense	AC006487.1	ENSG00000249906.1	0	0
antisense	AC006487.2	ENSG00000250310.2	0	0
antisense	AC015795.1	ENSG00000250751.1	0	0
antisense	FLJ45513	ENSG00000204584.1	0	1
antisense	AC002401.3	ENSG00000275025.1	0	0
antisense	AC002401.2	ENSG00000250282.1	0	0
antisense	AC002401.1	ENSG00000236472.1	0	0
antisense	AC015909.2	ENSG00000253730.1	0	0
antisense	AC015909.3	ENSG00000261959.1	0	0
antisense	AC004707.1	ENSG00000253102.1	0	0
antisense	CACNA1G-	ENSG00000250107.1	0	0
antisense	AC021491.3	ENSG00000250976.1	0	0
antisense	AC004590.1	ENSG00000251239.1	0	0
antisense	AC005921.2	ENSG00000262967.1	0	0
antisense	AC091062.1	ENSG00000261976.2	0	0
antisense	AC005920.2	ENSG00000249870.1	0	0
antisense	AC034268.2	ENSG00000285939.1	0	0
antisense	AC007638.2	ENSG00000263096.1	0	0
antisense	AC015912.1	ENSG00000262112.1	0	0
antisense	AC004584.1	ENSG00000262298.1	0	0
antisense	AC007114.2	ENSG00000263089.1	0	0
antisense	AC007431.2	ENSG00000266100.1	0	0
antisense	AC007431.1	ENSG00000263499.1	0	0
antisense	AC015845.1	ENSG00000264914.1	0	0
antisense	TSPOAP1-	ENSG00000265148.5	0	0
antisense	AC004687.1	ENSG00000265206.5	0	0
antisense	AC099850.1	ENSG00000224738.1	0	0
antisense	AC099850.3	ENSG00000265415.1	0	0
antisense	AC091059.1	ENSG00000266002.1	0	0
antisense	AC011921.1	ENSG00000259349.1	0	0
antisense	AC005884.1	ENSG00000267207.1	0	0
antisense	AC005884.2	ENSG00000267449.1	0	0
antisense	AC005856.1	ENSG00000267667.1	0	0
antisense	AC005746.3	ENSG00000273982.1	0	0
antisense	AC005746.2	ENSG00000267131.1	0	0
antisense	AC005901.1	ENSG00000267137.1	0	0
antisense	AC018628.2	ENSG00000285879.1	0	0

antisense	AC005821.1	ENSG00000265702.1	0	0
antisense	AC006270.1	ENSG00000264513.1	0	0
antisense	AC015923.1	ENSG00000233635.2	0	0
antisense	AC005828.2	ENSG00000263501.1	0	0
antisense	AC005828.1	ENSG00000226797.1	0	0
antisense	AC005828.3	ENSG00000263644.1	0	0
antisense	AC103810.2	ENSG00000265218.1	0	0
antisense	AC103810.5	ENSG00000266644.1	0	0
antisense	AC037487.2	ENSG00000266598.1	0	0
antisense	PRKCA-AS1	ENSG00000264630.5	0	0
antisense	AC005544.1	ENSG00000264491.1	0	0
antisense	AC005544.2	ENSG00000265664.1	0	0
antisense	AC007448.2	ENSG00000264421.1	0	0
antisense	AC007448.4	ENSG00000285877.1	0	3
antisense	AC134407.1	ENSG00000266717.1	0	0
antisense	AC005332.1	ENSG00000265100.1	0	0
antisense	AC005495.1	ENSG00000285931.1	0	0
antisense	AC005208.1	ENSG00000230258.6	0	0
antisense	AC011120.1	ENSG00000264196.1	0	0
antisense	AC097641.2	ENSG00000277728.1	0	0
antisense	AC124804.1	ENSG00000264985.3	0	0
antisense	AC032019.1	ENSG00000264750.1	0	0
antisense	AC100786.1	ENSG00000264272.1	0	0
antisense	AC103809.1	ENSG00000266106.1	0	0
antisense	C17orf77	ENSG00000182352.8	0	0
antisense	AC064805.2	ENSG00000264659.1	0	0
antisense	AC016888.1	ENSG00000266036.1	0	0
antisense	HID1-AS1	ENSG00000263586.1	0	0
antisense	AC011933.4	ENSG00000265987.1	0	0
antisense	AC087749.2	ENSG00000264829.1	0	0
antisense	AC087749.1	ENSG00000264270.1	0	0
antisense	AC087289.1	ENSG00000266980.1	0	0
antisense	AC087289.5	ENSG00000267801.1	0	0
antisense	AC087289.2	ENSG00000267342.1	0	0
antisense	AC087289.4	ENSG00000267615.1	0	0
antisense	RNF157-AS1	ENSG00000267128.1	0	0
antisense	AC015802.1	ENSG00000267078.1	0	0
antisense	AC005837.3	ENSG00000277382.1	0	0
antisense	AC111182.1	ENSG00000266998.1	0	0
antisense	AC111170.1	ENSG00000267016.1	0	0
antisense	AC022966.1	ENSG00000267601.1	0	0
antisense	AC100788.1	ENSG00000267491.1	0	0
antisense	C1QTNF1-	ENSG00000265096.1	0	0
antisense	AC021534.1	ENSG00000266711.1	0	0

antisense	AC100791.2	ENSG00000262768.2	0	0
antisense	AC100791.3	ENSG00000275516.1	0	0
antisense	AC116025.1	ENSG00000261978.1	0	0
antisense	AC124319.3	ENSG00000276863.1	0	1
antisense	AC016245.1	ENSG00000262833.1	0	0
antisense	AC127496.3	ENSG00000262313.1	0	0
antisense	AC127496.4	ENSG00000262662.1	0	0
antisense	AC127496.1	ENSG00000261924.1	0	0
antisense	AC127496.6	ENSG00000263218.2	0	0
antisense	AC115099.1	ENSG00000263167.1	0	0
antisense	AC027601.2	ENSG00000262115.1	0	0
antisense	AC027601.1	ENSG00000260005.6	0	0
antisense	AC110285.4	ENSG00000263271.1	0	0
antisense	AC139149.1	ENSG00000229848.1	0	0
antisense	AC145207.3	ENSG00000262831.1	0	0
antisense	AC145207.2	ENSG00000262413.1	0	0
antisense	AC145207.8	ENSG00000264769.1	0	0
antisense	AC145207.4	ENSG00000263585.1	0	0
antisense	AC145207.1	ENSG00000235296.1	0	0
antisense	AC137723.1	ENSG00000264569.1	0	0
antisense	AC129510.1	ENSG00000265678.1	0	0
antisense	AC129510.2	ENSG00000266654.1	0	0
antisense	NARF-AS1	ENSG00000266445.1	0	0
antisense	AC124283.2	ENSG00000262147.1	0	0
antisense	AC124283.1	ENSG00000261845.2	0	0
antisense	AC068014.1	ENSG00000263098.1	0	0
antisense	AC130371.1	ENSG00000262339.5	0	0
antisense	AP001178.3	ENSG00000266456.1	0	0
antisense	AP001178.2	ENSG00000265490.1	0	0
antisense	AP001020.3	ENSG00000266171.1	0	0
antisense	AP001020.1	ENSG00000264339.1	0	0
antisense	AP000894.3	ENSG00000265671.1	0	0
antisense	AP001011.1	ENSG00000266049.1	0	0
antisense	AP000919.4	ENSG00000272625.1	0	0
antisense	AP000919.2	ENSG00000265907.1	0	0
antisense	AP000919.3	ENSG00000266397.1	0	0
antisense	AP005329.2	ENSG00000265399.1	0	0
antisense	DLGAP1-AS5	ENSG00000261520.5	0	0
antisense	AP001496.4	ENSG00000285575.1	0	0
antisense	AP005059.2	ENSG00000265316.1	0	0
antisense	AP005059.1	ENSG00000264000.1	0	0
antisense	AP005433.1	ENSG00000264449.5	0	0
antisense	AP001021.1	ENSG00000265487.1	0	0
antisense	AP001021.2	ENSG00000266846.1	0	0

antisense	AP005205.2	ENSG00000266441.1	0	0
antisense	AP005210.1	ENSG00000263797.1	0	0
antisense	AP002409.1	ENSG00000265069.1	0	0
antisense	AP005062.1	ENSG00000264475.1	0	0
antisense	AP000897.2	ENSG00000266767.1	0	0
antisense	AP001094.3	ENSG00000266149.1	0	0
antisense	AP001094.1	ENSG00000263970.1	0	0
antisense	AP001793.1	ENSG00000266708.1	0	0
antisense	AP005899.1	ENSG00000263847.1	0	0
antisense	AP001381.1	ENSG00000266541.1	0	0
antisense	AC006238.2	ENSG00000285653.1	0	0
antisense	AP001180.1	ENSG00000260779.1	0	0
antisense	AP001180.4	ENSG00000264843.1	0	0
antisense	AP005120.1	ENSG00000263952.5	0	0
antisense	AP005137.1	ENSG00000267455.1	0	0
antisense	CHMP1B-	ENSG00000267165.1	0	1
antisense	AP005137.2	ENSG00000272703.1	0	0
antisense	AP001269.4	ENSG00000273141.1	0	0
antisense	AP001269.1	ENSG00000266955.1	0	0
antisense	AP001542.3	ENSG00000267480.1	0	0
antisense	AP002449.1	ENSG00000266969.1	0	0
antisense	AP001198.1	ENSG00000267239.1	0	0
antisense	AP002505.1	ENSG00000267177.1	0	0
antisense	AP002439.1	ENSG00000267393.1	0	0
antisense	LDLRAD4-	ENSG00000267690.1	0	0
antisense	AP005131.5	ENSG00000267694.1	0	0
antisense	AP005131.2	ENSG00000267366.1	0	0
antisense	AP005131.1	ENSG00000267136.1	0	0
antisense	AP005131.3	ENSG00000267503.1	0	0
antisense	AP001010.1	ENSG00000266954.1	0	0
antisense	AC006557.1	ENSG00000267150.1	0	0
antisense	AC006557.3	ENSG00000267356.1	0	0
antisense	AP006565.1	ENSG00000265737.1	0	0
antisense	AC015878.1	ENSG00000265751.1	0	0
antisense	AC106037.2	ENSG00000265656.1	0	0
antisense	MIR133A1H	ENSG00000265142.8	0	0
antisense	GATA6-AS1	ENSG00000266010.2	0	0
antisense	AC091588.1	ENSG00000264012.1	0	0
antisense	AC091588.3	ENSG00000266283.1	0	0
antisense	AC090912.1	ENSG00000265943.1	0	0
antisense	AC090912.2	ENSG00000266850.1	0	0
antisense	AC011731.1	ENSG00000266495.1	0	0
antisense	AC090772.2	ENSG00000265204.1	0	0
antisense	AC090772.1	ENSG00000264924.1	0	0

antisense	AC023983.1	ENSG00000264365.1	0	0
antisense	AC105114.2	ENSG00000285595.1	0	0
antisense	AC110603.1	ENSG00000264434.2	0	0
antisense	DSCAS	ENSG00000265888.1	0	0
antisense	AC012417.1	ENSG00000263698.1	0	0
antisense	DSG1-AS1	ENSG00000266729.5	0	0
antisense	DSG2-AS1	ENSG00000264859.5	0	1
antisense	AC017100.1	ENSG00000259985.1	0	0
antisense	AC022960.1	ENSG00000263924.1	0	0
antisense	AC011825.4	ENSG00000265008.1	0	0
antisense	AC011825.2	ENSG00000263393.1	0	0
antisense	AC015563.2	ENSG00000264982.1	0	0
antisense	AC025887.2	ENSG00000285095.1	0	2
antisense	AC012123.1	ENSG00000228835.1	0	0
antisense	AC090371.2	ENSG00000263765.5	0	0
antisense	AC090371.1	ENSG00000263450.1	0	0
antisense	AC104985.1	ENSG00000267746.1	0	0
antisense	AC022601.1	ENSG00000268873.1	0	0
antisense	AC016493.1	ENSG00000267039.1	0	0
antisense	AC015961.2	ENSG00000267707.2	0	0
antisense	AC090386.1	ENSG00000267202.5	0	0
antisense	AC009899.1	ENSG00000285940.1	0	0
antisense	SLC14A2-	ENSG00000267097.1	0	0
antisense	AC023421.2	ENSG00000267193.5	0	0
antisense	AC018931.1	ENSG00000285993.1	0	0
antisense	AC012254.1	ENSG00000266957.1	0	0
antisense	AC012254.3	ENSG00000267724.1	0	0
antisense	AC120349.1	ENSG00000269365.2	0	0
antisense	AC048380.1	ENSG00000267762.1	0	0
antisense	AC093567.1	ENSG00000267764.1	0	0
antisense	AC016866.2	ENSG00000265128.1	0	0
antisense	AC044840.1	ENSG00000266696.1	0	0
antisense	AC100778.3	ENSG00000265496.5	0	0
antisense	SNHG22	ENSG00000267322.2	0	0
antisense	AC093462.1	ENSG00000277324.1	0	0
antisense	AC098848.1	ENSG00000267112.1	0	0
antisense	TCF4-AS1	ENSG00000267028.1	0	0
antisense	TCF4-AS2	ENSG00000267402.1	0	0
antisense	AC012301.1	ENSG00000267146.1	0	0
antisense	AC107896.1	ENSG00000267743.5	0	0
antisense	AC090236.1	ENSG00000267396.1	0	0
antisense	AC105105.1	ENSG00000267257.1	0	0
antisense	AC104971.2	ENSG00000267579.1	0	0
antisense	AC104365.1	ENSG00000267476.1	0	0

antisense	AC016229.1	ENSG00000267000.1	0	0
antisense	AC090213.1	ENSG00000267061.1	0	0
antisense	AC010776.1	ENSG00000267269.1	0	0
antisense	AC091576.1	ENSG00000285681.1	0	0
antisense	AC027514.2	ENSG00000267560.1	0	0
antisense	AC100843.1	ENSG00000267341.1	0	0
antisense	AC064801.1	ENSG00000278017.1	0	1
antisense	AC022726.2	ENSG00000283125.1	0	0
antisense	AC009802.1	ENSG00000283667.1	0	0
antisense	AC110597.3	ENSG00000263424.1	0	0
antisense	AC022035.1	ENSG00000263637.1	0	0
antisense	AC096708.2	ENSG00000264472.4	0	0
antisense	AC119868.2	ENSG00000265643.1	0	0
antisense	AC068254.1	ENSG00000266840.1	0	0
antisense	AC009704.1	ENSG00000264340.1	0	0
antisense	AC116003.1	ENSG00000264116.5	0	0
antisense	AC009716.1	ENSG00000263982.1	0	0
antisense	AC093330.1	ENSG00000266844.1	0	0
antisense	AC018529.1	ENSG00000275178.1	0	0
antisense	AC018529.2	ENSG00000278330.1	0	0
antisense	AC104423.1	ENSG00000267628.1	0	0
antisense	AC023090.1	ENSG00000267015.1	0	0
antisense	AC018445.1	ENSG00000275186.1	0	0
antisense	AC068473.2	ENSG00000266901.1	0	0
antisense	AC139100.1	ENSG00000267251.2	0	0
antisense	AC016588.1	ENSG00000267124.2	0	0
antisense	AC005775.1	ENSG00000266933.2	0	0
antisense	AC005379.1	ENSG00000267159.2	0	0
antisense	AC004528.2	ENSG00000274177.1	0	0
antisense	CIRBP-AS1	ENSG00000267493.3	0	0
antisense	AC005329.2	ENSG00000267755.1	0	0
antisense	AC005329.3	ENSG00000280486.1	0	0
antisense	AC005329.1	ENSG00000248015.6	0	0
antisense	AC027307.1	ENSG00000267092.2	0	0
antisense	AC012615.2	ENSG00000267007.1	0	0
antisense	AC012615.3	ENSG00000267125.2	0	0
antisense	AC012615.4	ENSG00000267141.1	0	0
antisense	CSNK1G2-	ENSG00000180846.8	0	0
antisense	AC004490.1	ENSG00000267122.1	0	0
antisense	AC092068.1	ENSG00000267214.1	0	0
antisense	AC006130.1	ENSG00000267063.1	0	0
antisense	AC005944.1	ENSG00000267469.1	0	0
antisense	AC005262.2	ENSG00000267139.1	0	0
antisense	AC005264.1	ENSG00000267551.3	0	0

antisense	AC010649.1	ENSG00000267448.5	0	1
antisense	AC005786.3	ENSG00000267436.1	0	0
antisense	AC004637.1	ENSG00000267304.1	0	0
antisense	AC005954.2	ENSG00000267205.1	0	0
antisense	AC104521.1	ENSG00000269425.1	0	0
antisense	AC007292.2	ENSG00000269318.1	0	0
antisense	AC011498.3	ENSG00000267255.1	0	0
antisense	AC011498.2	ENSG00000267030.1	0	0
antisense	AC011498.6	ENSG00000267769.1	0	0
antisense	AC005339.1	ENSG00000268565.1	0	0
antisense	DPP9-AS1	ENSG00000205790.1	0	0
antisense	AC027319.1	ENSG00000267484.1	0	0
antisense	AC022517.1	ENSG00000267550.1	0	0
antisense	AC024592.2	ENSG00000267709.1	0	0
antisense	AC024592.1	ENSG00000266941.1	0	0
antisense	AC104532.2	ENSG00000267571.1	0	0
antisense	AC011444.2	ENSG00000267262.1	0	0
antisense	AC011444.3	ENSG00000267299.1	0	0
antisense	AC011491.3	ENSG00000269802.1	0	0
antisense	AC010503.2	ENSG00000268203.1	0	0
antisense	AC010503.1	ENSG00000268191.1	0	0
antisense	AC025278.1	ENSG00000268845.1	0	0
antisense	AC119396.2	ENSG00000267852.1	0	0
antisense	AC008878.4	ENSG00000269371.1	0	0
antisense	AC008763.1	ENSG00000268204.1	0	0
antisense	AC010336.1	ENSG00000214248.2	0	0
antisense	AC010336.4	ENSG00000268149.1	0	0
antisense	AC010336.2	ENSG00000260500.1	0	0
antisense	AC010336.6	ENSG00000269813.1	0	0
antisense	AC022146.2	ENSG00000271717.1	0	0
antisense	AC136469.1	ENSG00000268931.1	0	0
antisense	AC092316.1	ENSG00000268618.1	0	0
antisense	AC130469.1	ENSG00000267986.1	0	0
antisense	AC008734.1	ENSG00000269300.1	0	0
antisense	AC011451.1	ENSG00000267510.1	0	0
antisense	AC008742.1	ENSG00000267650.1	0	0
antisense	AC020931.1	ENSG00000267387.1	0	0
antisense	AC011511.2	ENSG00000266978.1	0	0
antisense	AC011511.3	ENSG00000267105.1	0	0
antisense	AC011511.5	ENSG00000267607.1	0	0
antisense	AC011442.1	ENSG00000266936.1	0	0
antisense	AC011472.1	ENSG00000267082.1	0	0
antisense	AC024575.1	ENSG00000267277.1	0	0
antisense	AC008543.5	ENSG00000267646.1	0	0

antisense	AC008758.2	ENSG00000234848.1	0	0
antisense	AC010422.7	ENSG00000285583.1	0	0
antisense	AC018761.4	ENSG00000267791.1	0	0
antisense	AC018761.2	ENSG00000267062.1	0	0
antisense	AC020934.1	ENSG00000267424.1	0	0
antisense	AC020934.2	ENSG00000267735.1	0	0
antisense	FARSA-AS1	ENSG00000266975.1	0	0
antisense	AC138474.1	ENSG00000267417.1	0	0
antisense	AC007787.1	ENSG00000267610.2	0	0
antisense	AC011446.1	ENSG00000267512.1	0	0
antisense	AC011446.2	ENSG00000267598.1	0	0
antisense	AC008686.1	ENSG00000267633.1	0	0
antisense	AC020916.2	ENSG00000267582.1	0	0
antisense	AC022098.4	ENSG00000275091.1	0	0
antisense	AC022098.2	ENSG00000267670.1	0	0
antisense	AC008569.1	ENSG00000267379.1	0	0
antisense	AC003956.1	ENSG00000268564.1	0	0
antisense	AC008894.2	ENSG00000269243.1	0	0
antisense	AC020911.1	ENSG00000267033.1	0	0
antisense	AC020911.2	ENSG00000267275.1	0	0
antisense	AC020917.2	ENSG00000267703.1	0	0
antisense	AC008764.3	ENSG00000268309.1	0	0
antisense	AC008764.5	ENSG00000269085.1	0	0
antisense	AC024075.3	ENSG00000269427.1	0	0
antisense	AC020908.2	ENSG00000268985.1	0	0
antisense	AC020908.1	ENSG00000268289.1	0	0
antisense	AC020908.3	ENSG00000269066.1	0	0
antisense	AC020913.1	ENSG00000268056.5	0	0
antisense	AC020913.3	ENSG00000269836.1	0	0
antisense	AC020913.2	ENSG00000269480.1	0	0
antisense	AC010463.2	ENSG00000269350.1	0	0
antisense	AC010319.4	ENSG00000269481.1	0	0
antisense	AC010319.3	ENSG00000269053.1	0	0
antisense	AC010618.2	ENSG00000269161.1	0	0
antisense	AC008761.2	ENSG00000269752.1	0	0
antisense	AC008761.1	ENSG00000268112.1	0	0
antisense	AC007192.2	ENSG00000269145.2	0	0
antisense	AC005759.1	ENSG00000268650.3	0	2
antisense	AC010335.1	ENSG00000268199.2	0	0
antisense	AC005387.1	ENSG00000268938.2	0	0
antisense	AC005387.2	ENSG00000269191.1	0	0
antisense	AC005253.1	ENSG00000268030.1	0	0
antisense	AC005253.2	ENSG00000268983.1	0	0
antisense	AC005197.1	ENSG00000269694.1	0	0

antisense	AC092067.1	ENSG00000267234.1	0	0
antisense	AC002306.1	ENSG00000259242.2	0	0
antisense	AC011477.3	ENSG00000267565.1	0	0
antisense	AC011447.3	ENSG00000267383.6	0	3
antisense	AC123912.1	ENSG00000268081.1	0	0
antisense	AC024563.1	ENSG00000268981.5	0	0
antisense	AC010300.1	ENSG00000267934.1	0	0
antisense	AC011503.1	ENSG00000269289.5	0	0
antisense	AC011474.2	ENSG00000266248.1	0	0
antisense	AC005597.1	ENSG00000267223.1	0	0
antisense	AC025809.1	ENSG00000267662.1	0	0
antisense	AC008474.1	ENSG00000267557.1	0	0
antisense	AC008805.2	ENSG00000267555.1	0	0
antisense	AC008738.4	ENSG00000267714.1	0	0
antisense	AC008738.3	ENSG00000267580.1	0	0
antisense	AC008738.5	ENSG00000267727.1	0	0
antisense	AC010504.1	ENSG00000267219.1	0	0
antisense	AC008747.1	ENSG00000267024.1	0	0
antisense	AC020907.3	ENSG00000269303.1	0	0
antisense	AC020907.4	ENSG00000271032.1	0	0
antisense	HPN-AS1	ENSG00000227392.1	0	0
antisense	AC002128.2	ENSG00000271366.1	0	0
antisense	AC002398.2	ENSG00000267328.1	0	0
antisense	AC002398.1	ENSG00000267049.1	0	0
antisense	AC002116.2	ENSG00000267698.1	0	0
antisense	AD001527.1	ENSG00000270760.1	0	0
antisense	AC092296.1	ENSG00000266973.1	0	0
antisense	AC092295.2	ENSG00000267309.1	0	0
antisense	AC092295.1	ENSG00000228629.2	0	0
antisense	AC010632.1	ENSG00000267345.1	0	1
antisense	AC012309.2	ENSG00000267437.1	0	0
antisense	AC011465.1	ENSG00000268764.1	0	0
antisense	AC005625.1	ENSG00000266963.1	0	0
antisense	AC005789.1	ENSG00000267090.1	0	0
antisense	AC067969.2	ENSG00000269445.1	0	0
antisense	AC067969.1	ENSG00000268055.1	0	0
antisense	AC008649.1	ENSG00000267291.1	0	0
antisense	AC008649.2	ENSG00000267375.1	0	0
antisense	AC104534.1	ENSG00000268756.1	0	0
antisense	AC011455.1	ENSG00000269050.1	0	0
antisense	AC010605.1	ENSG00000267992.1	0	0
antisense	AC011443.1	ENSG00000269172.1	0	0
antisense	AC011500.3	ENSG00000269792.1	0	0
antisense	AC005614.1	ENSG00000269296.1	0	0

antisense	AC010271.1	ENSG00000268366.1	0	0
antisense	AC008537.4	ENSG00000282951.1	0	0
antisense	AC011510.1	ENSG00000269652.1	0	0
antisense	AC011462.2	ENSG00000268475.1	0	0
antisense	AC243967.2	ENSG00000268833.1	0	0
antisense	AC004784.1	ENSG00000282943.1	0	0
antisense	AC005392.3	ENSG00000272396.1	0	0
antisense	L34079.2	ENSG00000269177.1	0	0
antisense	L34079.3	ENSG00000269583.1	0	0
antisense	AC006213.3	ENSG00000267191.1	0	0
antisense	AC243964.2	ENSG00000266903.1	0	0
antisense	AC011481.2	ENSG00000267282.1	0	0
antisense	AC005779.1	ENSG00000266958.1	0	0
antisense	DM1-AS	ENSG00000267395.5	0	2
antisense	AC092301.1	ENSG00000269148.1	0	0
antisense	AC006262.3	ENSG00000269745.1	0	0
antisense	AC007193.2	ENSG00000269124.1	0	0
antisense	AC007193.1	ENSG00000268810.1	0	0
antisense	AC007193.3	ENSG00000269151.1	0	0
antisense	AC011484.1	ENSG00000204850.4	0	0
antisense	AC093503.2	ENSG00000269292.1	0	0
antisense	DACT3-AS1	ENSG00000245598.6	0	0
antisense	AC008635.1	ENSG00000269487.1	0	0
antisense	AC010331.1	ENSG00000277383.1	0	0
antisense	BICRA-AS1	ENSG00000269806.1	0	0
antisense	NOP53-AS1	ENSG00000269656.1	0	0
antisense	PLA2G4C-	ENSG00000269420.5	0	0
antisense	AC011466.3	ENSG00000269534.5	0	0
antisense	AC011466.2	ENSG00000269321.1	0	0
antisense	AC008403.2	ENSG00000268530.5	0	0
antisense	AC022154.1	ENSG00000268093.1	0	1
antisense	NUCB1-AS1	ENSG00000235191.1	0	0
antisense	AC008687.2	ENSG00000268108.1	0	0
antisense	AC010524.1	ENSG00000268157.1	0	2
antisense	AC010643.1	ENSG00000268686.1	0	0
antisense	AC011495.2	ENSG00000268636.1	0	0
antisense	AC011495.3	ENSG00000268677.1	0	0
antisense	AC018766.1	ENSG00000268047.1	0	0
antisense	PTOV1-AS2	ENSG00000269352.1	0	0
antisense	AC010624.3	ENSG00000269091.5	0	0
antisense	AC008655.1	ENSG00000267815.1	0	0
antisense	AC008655.2	ENSG00000269392.1	0	0
antisense	AC020909.3	ENSG00000268854.1	0	0
antisense	AC011523.1	ENSG00000267968.1	0	0

antisense	AC011483.2	ENSG00000269495.1	0	0
antisense	AC011483.1	ENSG00000267879.1	0	0
antisense	AC011473.2	ENSG00000268739.1	0	0
antisense	AC011473.3	ENSG00000268906.1	0	0
antisense	AC063977.6	ENSG00000269072.1	0	0
antisense	AC063977.3	ENSG00000268595.1	0	0
antisense	AC008750.3	ENSG00000267905.5	0	0
antisense	AC008750.4	ENSG00000267984.1	0	0
antisense	AC008750.1	ENSG00000254760.1	0	0
antisense	AC008750.2	ENSG00000255441.1	0	0
antisense	AC020914.1	ENSG00000268777.1	0	0
antisense	ZNF649-AS1	ENSG00000268095.1	0	0
antisense	ZNF350-AS1	ENSG00000269235.1	0	0
antisense	AC010320.1	ENSG00000267927.1	0	0
antisense	AC010320.2	ENSG00000268015.1	0	0
antisense	AC010320.3	ENSG00000269102.1	0	0
antisense	AC010320.4	ENSG00000269535.1	0	0
antisense	AC022150.3	ENSG00000269349.1	0	0
antisense	AC022150.1	ENSG00000268886.1	0	0
antisense	AC092070.1	ENSG00000268842.1	0	0
antisense	AC092070.3	ENSG00000269288.1	0	0
antisense	AC008440.1	ENSG00000228323.2	0	0
antisense	AC008440.2	ENSG00000232220.2	0	0
antisense	AC245052.4	ENSG00000237017.1	0	0
antisense	AC245884.1	ENSG00000227407.1	0	0
antisense	LILRB1-AS1	ENSG00000224730.1	0	0
antisense	AC011476.3	ENSG00000267265.5	0	0
antisense	AC020922.2	ENSG00000268729.1	0	0
antisense	AC020922.3	ENSG00000269275.1	0	0
antisense	AC008735.3	ENSG00000269859.1	0	0
antisense	AC008735.4	ENSG00000276488.1	0	0
antisense	AC008735.2	ENSG00000267523.1	0	0
antisense	AC010525.1	ENSG00000267117.1	0	0
antisense	AC006116.8	ENSG00000267549.5	0	0
antisense	AC005498.3	ENSG00000269696.1	0	2
antisense	AC006115.3	ENSG00000284729.1	0	0
antisense	ZIM2-AS1	ENSG00000269793.6	0	0
antisense	AC005261.2	ENSG00000268678.1	0	0
antisense	AC004076.2	ENSG00000276449.1	0	2
antisense	AC012313.2	ENSG00000268049.1	0	0
antisense	AC012313.6	ENSG00000269054.1	0	0
antisense	AC016629.2	ENSG00000269600.1	0	0
antisense	SIRPG-AS1	ENSG00000237914.5	0	0
antisense	AL117335.1	ENSG00000276649.1	0	0

antisense	PDYN-AS1	ENSG00000233896.1	0	0
antisense	AL049712.1	ENSG00000228293.1	0	0
antisense	UBOX5-AS1	ENSG00000235958.5	0	0
antisense	AL031670.1	ENSG00000275582.1	0	0
antisense	LINC01433	ENSG00000230176.2	0	0
antisense	AL109935.1	ENSG00000235820.1	0	0
antisense	MCM8-AS1	ENSG00000278719.1	0	0
antisense	AL021396.1	ENSG00000229766.6	0	0
antisense	LAMP5-AS1	ENSG00000225988.1	0	0
antisense	AL353612.1	ENSG00000232738.1	0	0
antisense	AL050403.2	ENSG00000270792.5	0	0
antisense	AL035448.1	ENSG00000236526.1	0	0
antisense	ISM1-AS1	ENSG00000226263.1	0	0
antisense	AL050320.1	ENSG00000225956.1	0	0
antisense	MACROD2-	ENSG00000235914.1	0	0
antisense	AL138808.1	ENSG00000237832.1	0	0
antisense	AL121584.1	ENSG00000225181.1	0	0
antisense	AL160411.1	ENSG00000229262.1	0	0
antisense	AL049646.2	ENSG00000237259.1	0	0
antisense	LINC00851	ENSG00000237282.3	0	0
antisense	AL121900.1	ENSG00000233993.1	0	0
antisense	AL136090.1	ENSG00000235996.1	0	0
antisense	AL049647.1	ENSG00000179447.2	0	0
antisense	AL035454.1	ENSG00000241054.1	0	0
antisense	AL049648.1	ENSG00000225417.1	0	0
antisense	AL121721.1	ENSG00000243810.1	0	0
antisense	AL121759.1	ENSG00000228604.3	0	0
antisense	KIZ-AS1	ENSG00000232712.6	0	0
antisense	AL158013.1	ENSG00000225280.6	0	0
antisense	NKX2-2-AS1	ENSG00000258197.1	0	0
antisense	AL049651.1	ENSG00000230492.1	0	0
antisense	AL096677.1	ENSG00000234832.1	0	0
antisense	AL591074.1	ENSG00000230908.1	0	0
antisense	AL157413.1	ENSG00000228539.1	0	0
antisense	AL121772.2	ENSG00000274507.1	0	0
antisense	FAM242A	ENSG00000231934.1	0	0
antisense	HM13-AS1	ENSG00000230613.1	0	0
antisense	AL117381.1	ENSG00000236559.1	0	0
antisense	ABALON	ENSG00000281376.1	0	0
antisense	AL031658.1	ENSG00000226239.1	0	0
antisense	AL034550.1	ENSG00000236772.1	0	0
antisense	AL034550.2	ENSG00000277301.1	0	0
antisense	AL133343.2	ENSG00000233293.1	0	0
antisense	AL133343.1	ENSG00000228156.1	0	0

antisense	AL035071.2	ENSG00000260536.1	0	0
antisense	AL121901.1	ENSG00000224876.1	0	0
antisense	AL121906.1	ENSG00000271803.1	0	0
antisense	AL050349.1	ENSG00000229188.2	0	0
antisense	ZNF341-AS1	ENSG00000230753.5	0	0
antisense	ITCH-AS1	ENSG00000236388.1	0	0
antisense	AL356652.1	ENSG00000278367.1	0	0
antisense	FAM83C-AS1	ENSG00000235214.1	0	0
antisense	GDF5OS	ENSG00000204183.1	0	0
antisense	FO393401.1	ENSG00000230155.6	0	0
antisense	AL121895.1	ENSG00000232406.6	0	0
antisense	DLGAP4-AS1	ENSG00000232907.7	0	0
antisense	AL136172.1	ENSG00000269846.1	0	0
antisense	AL359555.3	ENSG00000285144.1	0	0
antisense	AL359555.4	ENSG00000285347.1	0	1
antisense	AL359555.1	ENSG00000206249.3	0	0
antisense	AL049812.2	ENSG00000225458.1	0	0
antisense	AL031656.1	ENSG00000229042.2	0	0
antisense	AL031676.1	ENSG00000227599.1	0	0
antisense	Z98752.1	ENSG00000226143.1	0	0
antisense	AL117382.1	ENSG00000226812.2	0	0
antisense	HNF4A-AS1	ENSG00000229005.2	0	0
antisense	AL117382.2	ENSG00000233376.1	0	0
antisense	AL031663.2	ENSG00000237464.1	0	0
antisense	AL008726.1	ENSG00000271984.1	0	0
antisense	AL031666.2	ENSG00000267882.2	0	0
antisense	AL031666.1	ENSG00000231119.2	0	0
antisense	AL354813.1	ENSG00000255438.2	0	0
antisense	CSE1L-AS1	ENSG00000227431.5	0	0
antisense	CEBPB-AS1	ENSG00000277449.1	0	0
antisense	AL133230.1	ENSG00000232043.1	0	0
antisense	AL353653.1	ENSG00000234693.1	0	1
antisense	AL050404.1	ENSG00000232358.1	0	0
antisense	AL391097.1	ENSG00000223492.1	0	0
antisense	AL391097.2	ENSG00000227705.1	0	0
antisense	AL354993.1	ENSG00000231703.2	0	0
antisense	AL354993.2	ENSG00000259723.1	0	0
antisense	AC005220.1	ENSG00000236352.1	0	0
antisense	BMP7-AS1	ENSG00000235032.1	0	0
antisense	AL109955.1	ENSG00000218018.2	0	0
antisense	AL121917.2	ENSG00000270951.1	0	0
antisense	AL121908.1	ENSG00000238194.1	0	0
antisense	AL162457.1	ENSG00000179253.3	0	0
antisense	AL162457.2	ENSG00000225106.1	0	0

antisense	LAMA5-AS1	ENSG00000228812.7	0	0
antisense	AL121832.1	ENSG00000233017.2	0	0
antisense	MIR1-1HG-	ENSG00000174403.15	0	0
antisense	MIR1-1HG	ENSG00000174407.13	0	0
antisense	AL450469.2	ENSG00000232121.1	0	0
antisense	AL450469.1	ENSG00000229882.1	0	0
antisense	AL357033.1	ENSG00000167046.4	0	1
antisense	AL357033.3	ENSG00000276317.1	0	0
antisense	SLCO4A1-	ENSG00000232803.1	0	0
antisense	AL357033.2	ENSG00000223669.1	0	0
antisense	LINC00659	ENSG00000228705.1	0	0
antisense	OGFR-AS1	ENSG00000229873.1	0	0
antisense	AL121827.1	ENSG00000203900.2	0	0
antisense	AL353658.2	ENSG00000280936.1	0	0
antisense	AL353658.1	ENSG00000226390.1	0	0
antisense	AL121829.1	ENSG00000230226.1	0	0
antisense	AL121829.2	ENSG00000275812.1	0	2
antisense	ZBTB46-AS1	ENSG00000231208.4	0	0
antisense	UCKL1-AS1	ENSG00000280213.1	0	0
antisense	AL355803.1	ENSG00000237371.1	0	0
antisense	FP565260.5	ENSG00000279687.1	0	0
antisense	SAMSN1-	ENSG00000223662.1	0	0
antisense	AF127577.3	ENSG00000235277.1	0	0
antisense	AF127577.1	ENSG00000229047.1	0	0
antisense	AF127577.2	ENSG00000231201.1	0	0
antisense	AL109761.1	ENSG00000244676.5	0	0
antisense	AP001136.1	ENSG00000226771.1	0	0
antisense	LINC00515	ENSG00000260583.1	0	1
antisense	AP001439.1	ENSG00000224541.1	0	0
antisense	CYYR1-AS1	ENSG00000197934.8	0	1
antisense	AP001599.1	ENSG00000223563.1	0	0
antisense	AF124730.1	ENSG00000224649.1	0	0
antisense	AP000238.1	ENSG00000273464.1	0	0
antisense	GRIK1-AS1	ENSG00000174680.9	0	0
antisense	HUNK-AS1	ENSG00000237138.1	0	0
antisense	MIS18A-AS1	ENSG00000227256.1	0	0
antisense	AP000266.1	ENSG00000232623.1	0	0
antisense	AP000281.2	ENSG00000232360.1	0	0
antisense	AP000282.1	ENSG00000227757.3	0	0
antisense	AP000290.1	ENSG00000226433.1	0	0
antisense	IL10RB-DT	ENSG00000223799.1	0	0
antisense	AP000302.1	ENSG00000231355.1	0	0
antisense	AP000320.1	ENSG00000225555.1	0	0
antisense	LINC01426	ENSG00000234380.1	0	1

antisense	AP000688.4	ENSG00000236677.1	0	0
antisense	AP000695.1	ENSG00000230479.1	0	0
antisense	AP000697.1	ENSG00000224269.1	0	0
antisense	TTC3-AS1	ENSG00000228677.1	0	0
antisense	AF129408.1	ENSG00000272991.1	0	0
antisense	B3GALT5-	ENSG00000184809.12	0	0
antisense	DSCAM-AS1	ENSG00000235123.5	0	0
antisense	LINC00323	ENSG00000226496.2	0	0
antisense	PLAC4	ENSG00000280109.3	0	0
antisense	BACE2-IT1	ENSG00000224388.1	0	0
antisense	AP001610.1	ENSG00000228318.3	0	0
antisense	LINC00112	ENSG00000232401.1	0	0
antisense	AP001615.1	ENSG00000236883.1	0	0
antisense	AP001619.2	ENSG00000236545.1	0	0
antisense	AP001625.2	ENSG00000235772.1	0	0
antisense	AP001626.1	ENSG00000235023.1	0	0
antisense	AP001627.1	ENSG00000225731.1	0	0
antisense	AP001630.1	ENSG00000224100.1	0	0
antisense	FRGCA	ENSG00000236663.1	0	0
antisense	AP001059.1	ENSG00000232010.1	0	0
antisense	AP001062.2	ENSG00000232969.1	0	0
antisense	LRRC3-DT	ENSG00000229356.1	0	0
antisense	TSPEAR-AS1	ENSG00000235890.2	0	0
antisense	TSPEAR-AS2	ENSG00000182912.6	0	0
antisense	LINC01424	ENSG00000236519.1	0	0
antisense	ITGB2-AS1	ENSG00000227039.6	0	0
antisense	AL844908.1	ENSG00000272825.1	0	0
antisense	BX322559.1	ENSG00000228355.1	0	2
antisense	COL18A1-	ENSG00000224574.1	0	0
antisense	COL18A1-	ENSG00000183535.9	0	0
antisense	AL592528.1	ENSG00000205424.1	0	0
antisense	AP001471.1	ENSG00000227438.1	0	0
antisense	FTCD-AS1	ENSG00000237338.1	0	0
antisense	AP001469.1	ENSG00000223901.2	0	0
antisense	AP001469.2	ENSG00000228137.1	0	0
antisense	AP000534.1	ENSG00000233408.1	0	0
antisense	POTEH-AS1	ENSG00000236666.1	0	0
antisense	HDHD5-AS1	ENSG00000185837.3	0	3
antisense	AC008079.1	ENSG00000280007.1	0	0
antisense	LINC02592	ENSG00000187979.4	0	0
antisense	AC004471.1	ENSG00000223461.1	0	0
antisense	AC000068.3	ENSG00000273300.1	0	0
antisense	AC000068.2	ENSG00000273212.1	0	0
antisense	AC006547.1	ENSG00000236540.7	0	0

antisense	AC006547.3	ENSG00000268292.1	0	0
antisense	AC006547.2	ENSG00000243762.1	0	0
antisense	AC007663.2	ENSG00000249923.1	0	0
antisense	AC007731.1	ENSG00000188280.11	0	0
antisense	AC007731.4	ENSG00000236003.1	0	0
antisense	AC007308.1	ENSG00000272600.1	0	0
antisense	AC002472.1	ENSG00000226872.1	0	0
antisense	LL22NC03-63E9.3	ENSG00000220891.1	0	0
antisense	AP000345.1	ENSG00000224277.5	0	0
antisense	AP000350.7	ENSG00000279467.1	0	0
antisense	ADORA2A-	ENSG00000178803.10	0	0
antisense	AP000355.1	ENSG00000228923.1	0	0
antisense	AP000356.2	ENSG00000284070.1	0	0
antisense	AL022323.3	ENSG00000279085.1	0	0
antisense	AL022323.5	ENSG00000279548.1	0	0
antisense	AL022323.2	ENSG00000279006.1	0	0
antisense	AL022323.4	ENSG00000279110.1	0	0
antisense	AL022323.1	ENSG00000203280.4	0	0
antisense	AL022329.1	ENSG00000234884.1	0	0
antisense	AL022329.2	ENSG00000237387.1	0	0
antisense	AL022329.3	ENSG00000278960.1	0	0
antisense	Z98949.1	ENSG00000231933.7	0	0
antisense	Z98949.3	ENSG00000232464.1	0	0
antisense	AL022337.1	ENSG00000229770.1	0	0
antisense	AL080273.1	ENSG00000224192.2	0	0
antisense	AL121885.1	ENSG00000226169.1	0	0
antisense	ZNRF3-AS1	ENSG00000177993.3	0	0
antisense	AL021393.1	ENSG00000226772.1	0	0
antisense	AL031186.1	ENSG00000237015.1	0	0
antisense	RFPL1S	ENSG00000225465.8	0	3
antisense	AC000035.1	ENSG00000282816.1	0	0
antisense	AC004882.1	ENSG00000232396.2	0	0
antisense	AC004882.2	ENSG00000239446.1	0	0
antisense	HORMAD2-	ENSG00000227117.6	0	0
antisense	AC002378.1	ENSG00000225676.1	0	0
antisense	LIF-AS1	ENSG00000232530.1	0	0
antisense	AC004832.1	ENSG00000181123.8	0	0
antisense	MORC2-AS1	ENSG00000235989.3	0	0
antisense	AC005005.3	ENSG00000273387.1	0	0
antisense	RNF185-AS1	ENSG00000254835.1	0	0
antisense	PIK3IP1-AS1	ENSG00000228839.5	0	0
antisense	SLC5A4-AS1	ENSG00000242082.1	0	0

antisense	AL021937.3	ENSG00000234626.1	0	0
antisense	Z82246.1	ENSG00000236054.1	0	1
antisense	Z73495.1	ENSG00000229673.1	0	0
antisense	Z82198.2	ENSG00000232073.1	0	0
antisense	Z82173.1	ENSG00000230741.1	0	0
antisense	Z73429.1	ENSG00000273082.1	0	0
antisense	AL008635.1	ENSG00000273176.1	0	0
antisense	AL022313.3	ENSG00000229971.1	0	0
antisense	AL049749.1	ENSG00000234688.1	0	0
antisense	Z82185.1	ENSG00000234979.1	0	0
antisense	NCF4-AS1	ENSG00000183822.3	0	0
antisense	LL22NC01-81G9.3	ENSG00000215403.1	0	0
antisense	AL022314.1	ENSG00000231467.2	0	0
antisense	Z82188.2	ENSG00000235237.1	0	0
antisense	Z94160.2	ENSG00000272694.1	0	0
antisense	AL022315.1	ENSG00000225867.1	0	0
antisense	Z83844.2	ENSG00000233360.4	0	0
antisense	AL031587.1	ENSG00000222044.1	0	0
antisense	AL031587.2	ENSG00000233739.1	0	0
antisense	AL022322.2	ENSG00000279080.1	0	0
antisense	AL020993.1	ENSG00000235246.1	0	0
antisense	Z97056.1	ENSG00000228620.1	0	0
antisense	AL021707.4	ENSG00000230912.1	0	0
antisense	AL021707.5	ENSG00000244491.1	0	0
antisense	AL021707.3	ENSG00000230149.2	0	0
antisense	AL021707.1	ENSG00000225450.1	0	0
antisense	APOBEC3B-	ENSG00000249310.2	0	0
antisense	AL031590.1	ENSG00000284633.1	0	0
antisense	MGAT3-AS1	ENSG00000227188.1	0	0
antisense	AL022238.2	ENSG00000229999.6	0	0
antisense	AL022238.3	ENSG00000272834.1	0	0
antisense	MRTFA-AS1	ENSG00000232564.3	0	0
antisense	AL035658.1	ENSG00000232754.1	0	0
antisense	EP300-AS1	ENSG00000231993.1	0	0
antisense	SREBF2-AS1	ENSG00000184068.2	0	0
antisense	Z99716.1	ENSG00000224883.1	0	0
antisense	AL022476.1	ENSG00000230319.1	0	2
antisense	Z82214.2	ENSG00000236272.1	0	0
antisense	Z82214.1	ENSG00000234892.1	0	0
antisense	Z99756.1	ENSG00000203527.2	0	0
antisense	EFCAB6-AS1	ENSG00000223843.4	0	0
antisense	AL031595.2	ENSG00000280011.1	0	1

antisense	AL021391.1	ENSG00000232363.1	0	0
antisense	LINC01589	ENSG00000238120.1	0	0
antisense	BX324167.2	ENSG00000279954.1	0	0
antisense	TBC1D22A-	ENSG00000280080.2	0	0
antisense	AL022328.3	ENSG00000273188.1	0	0
antisense	AL022328.2	ENSG00000273137.1	0	0
antisense	CR559946.1	ENSG00000227484.1	0	0
antisense	CR559946.2	ENSG00000279182.1	0	0
antisense	AC002056.2	ENSG00000254499.1	0	0
antisense	PAR	ENSG00000281849.3	0	0
antisense	GYG2-AS1	ENSG00000235483.1	0	0
antisense	ARSD-AS1	ENSG00000229851.1	0	0
antisense	PRKX-AS1	ENSG00000236188.1	0	0
antisense	WWC3-AS1	ENSG00000225076.1	0	0
antisense	FRMPD4-	ENSG00000223487.1	0	0
antisense	TLR8-AS1	ENSG00000233338.1	0	0
antisense	GS1-600G8.3	ENSG00000231216.1	0	0
antisense	INE2	ENSG00000281371.1	0	0
antisense	AC078993.1	ENSG00000238178.6	0	0
antisense	NHS-AS1	ENSG00000230020.1	0	0
antisense	PPEF1-AS1	ENSG00000237221.1	0	0
antisense	PHKA2-AS1	ENSG00000237836.5	0	0
antisense	EIF1AX-AS1	ENSG00000225037.1	0	0
antisense	PHEX-AS1	ENSG00000224204.1	0	0
antisense	AC131011.1	ENSG00000233785.1	0	0
antisense	ZFX-AS1	ENSG00000234230.1	0	0
antisense	PCYT1B-AS1	ENSG00000236836.1	0	0
antisense	GK-AS1	ENSG00000243055.1	0	0
antisense	TAB3-AS1	ENSG00000231542.1	0	0
antisense	TAB3-AS2	ENSG00000235512.1	0	0
antisense	AC079177.1	ENSG00000285900.1	0	0
antisense	DMD-AS3	ENSG00000236828.1	0	0
antisense	AL606516.1	ENSG00000226484.2	0	0
antisense	AL606748.1	ENSG00000226679.1	0	0
antisense	MID1IP1-AS1	ENSG00000238123.1	0	0
antisense	CASK-AS1	ENSG00000233033.1	0	0
antisense	NDP-AS1	ENSG00000236276.1	0	0
antisense	AC136489.1	ENSG00000229491.5	0	0
antisense	UXT-AS1	ENSG00000267064.1	0	0
antisense	ZNF630-AS1	ENSG00000277541.1	0	0
antisense	AC115618.1	ENSG00000204620.3	0	0
antisense	AC115618.2	ENSG00000228343.1	0	0
antisense	AC231533.1	ENSG00000232828.1	0	0
antisense	SYP-AS1	ENSG00000237341.1	0	0

antisense	AC233728.1	ENSG00000233250.1	0	0
antisense	AL139397.1	ENSG00000224799.1	0	0
antisense	AL022157.1	ENSG00000226310.1	0	0
antisense	SPIN4-AS1	ENSG00000233661.1	0	0
antisense	AL034397.2	ENSG00000237311.1	0	0
antisense	AL034397.3	ENSG00000274536.6	0	0
antisense	IGBP1-AS2	ENSG00000220925.2	0	0
antisense	IGBP1-AS1	ENSG00000203588.3	0	0
antisense	AL139398.1	ENSG00000284391.1	0	0
antisense	DLG3-AS1	ENSG00000231651.1	0	0
antisense	AL590764.1	ENSG00000228427.1	0	0
antisense	PHKA1-AS1	ENSG00000231944.1	0	0
antisense	PABPC1L2B-AS1	ENSG00000226725.2	0	0
antisense	AL662864.1	ENSG00000231963.1	0	0
antisense	PABPC5-AS1	ENSG00000234161.1	0	0
antisense	ARMCX3-	ENSG00000228275.1	0	0
antisense	AL035427.1	ENSG00000270050.1	0	0
antisense	Z95624.1	ENSG00000234050.1	0	0
antisense	Z69733.1	ENSG00000234405.1	0	0
antisense	TCEAL3-AS1	ENSG00000224031.1	0	0
antisense	TMSB15B-	ENSG00000231728.3	0	0
antisense	FRMPD3-	ENSG00000227610.1	0	0
antisense	AL035425.1	ENSG00000237863.2	0	0
antisense	ALG13-AS1	ENSG00000229487.1	0	0
antisense	DANT1	ENSG00000229335.1	0	0
antisense	AC004973.1	ENSG00000226661.1	0	0
antisense	RHOXF1-AS1	ENSG00000258545.5	0	0
antisense	XIAP-AS1	ENSG00000237331.1	0	0
antisense	AL034405.1	ENSG00000235189.1	0	0
antisense	LINC01201	ENSG00000228659.1	0	0
antisense	HS6ST2-AS1	ENSG00000235849.1	0	0
antisense	INTS6L-AS1	ENSG00000225235.1	0	1
antisense	AL035443.1	ENSG00000224765.1	0	0
antisense	AL031386.1	ENSG00000232183.1	0	0
antisense	FGF13-AS1	ENSG00000226031.5	0	0
antisense	AC239727.1	ENSG00000227303.1	0	0
antisense	AL713923.1	ENSG00000234712.1	0	0
antisense	L29074.1	ENSG00000227083.1	0	0
antisense	AC002368.1	ENSG00000237741.1	0	0
antisense	MAGEA8-	ENSG00000230899.1	0	0
antisense	GPR50-AS1	ENSG00000234696.1	0	0
antisense	MAGEA4-	ENSG00000229967.1	0	0

antisense	AC116666.1	ENSG00000231937.1	0	0
antisense	AC244102.1	ENSG00000231447.1	0	0
antisense	U52111.1	ENSG00000232725.1	0	0
antisense	HCFC1-AS1	ENSG00000235802.1	0	0
antisense	AC245140.1	ENSG00000231830.1	0	0
antisense	AC245140.3	ENSG00000285018.1	0	0
antisense	AC234781.1	ENSG00000224216.1	0	0
antisense	TMLHE-AS1	ENSG00000224533.4	0	0
antisense	BX571846.1	ENSG00000225393.1	0	0
antisense	WASIR1	ENSG00000185203.12	0	0
antisense	ZFY-AS1	ENSG00000233070.1	0	0
antisense	TTTY20	ENSG00000232808.1	0	0
antisense	NLGN4Y-	ENSG00000228787.1	0	0
antisense	TTTY9B	ENSG00000131007.9	0	0
antisense	TTTY6B	ENSG00000131548.7	0	0
bidirectional_promoter_lncRNA	AL445253.1	ENSG00000285873.1	0	0
bidirectional_promoter_lncRNA	AL391650.2	ENSG00000284309.1	0	0
bidirectional_promoter_lncRNA	AL513220.1	ENSG00000284748.1	0	0
bidirectional_promoter_lncRNA	AL049637.2	ENSG00000284700.1	0	0
bidirectional_promoter_lncRNA	AL590440.2	ENSG00000284601.1	0	0
bidirectional_promoter_lncRNA	AL049796.1	ENSG00000260464.1	0	0
bidirectional_promoter_lncRNA	AC095032.1	ENSG00000224613.6	0	0
bidirectional_promoter_lncRNA	AL513013.1	ENSG00000285910.1	0	0
bidirectional_promoter_lncRNA	AC119673.1	ENSG00000285521.1	0	0
bidirectional_promoter_lncRNA	AL162595.1	ENSG00000177788.5	0	0
bidirectional_promoter_lncRNA	AC013400.1	ENSG00000271991.1	0	0
bidirectional_promoter_lncRNA	LGALSL-DT	ENSG00000223935.2	0	0
bidirectional_promoter_lncRNA	AC074008.2	ENSG00000285068.1	0	0
bidirectional_promoter_lncRNA	AC006994.2	ENSG00000279317.2	0	0

bidirectional_promoter_lncRNA	LIMD1-AS1	ENSG00000230530.1	0	0
bidirectional_promoter_lncRNA	AC107398.4	ENSG00000282904.1	0	0
bidirectional_promoter_lncRNA	AC131025.3	ENSG00000285736.1	0	0
bidirectional_promoter_lncRNA	AL355499.2	ENSG00000285216.1	0	0
bidirectional_promoter_lncRNA	DINOL	ENSG00000285244.1	0	0
bidirectional_promoter_lncRNA	AL591428.1	ENSG00000285652.1	0	0
bidirectional_promoter_lncRNA	Z94721.1	ENSG00000227598.1	0	0
bidirectional_promoter_lncRNA	Z94721.3	ENSG00000285730.1	0	0
bidirectional_promoter_lncRNA	AC084262.2	ENSG00000285601.1	0	0
bidirectional_promoter_lncRNA	AC084024.4	ENSG00000285632.1	0	0
bidirectional_promoter_lncRNA	AC083837.2	ENSG00000285744.1	0	0
bidirectional_promoter_lncRNA	LINC02235	ENSG00000254689.2	0	0
bidirectional_promoter_lncRNA	AL391834.3	ENSG00000285911.1	0	0
bidirectional_promoter_lncRNA	AL807776.1	ENSG00000285706.1	0	0
bidirectional_promoter_lncRNA	AL353147.1	ENSG00000285852.1	0	0
bidirectional_promoter_lncRNA	AL022345.4	ENSG00000285884.1	0	0
bidirectional_promoter_lncRNA	AL355490.1	ENSG00000225850.3	0	0
bidirectional_promoter_lncRNA	AL133353.1	ENSG00000229278.2	0	0
bidirectional_promoter_lncRNA	WT1-AS	ENSG00000183242.11	0	0
bidirectional_promoter_lncRNA	AP000708.1	ENSG00000255537.1	0	1
bidirectional_promoter_lncRNA	AP001122.1	ENSG00000245008.4	0	0

bidirectional_promoter_lncRNA	AC125494.1	ENSG00000219410.6	0	0
bidirectional_promoter_lncRNA	AL121612.2	ENSG00000285205.2	0	0
bidirectional_promoter_lncRNA	C17orf102	ENSG00000197322.3	0	0
IG_C_gene	IGKC	ENSG00000211592.8	0	0
IG_C_gene	IGHA2	ENSG00000211890.4	0	0
IG_C_gene	IGHE	ENSG00000211891.6	0	0
IG_C_gene	IGHG4	ENSG00000211892.4	0	0
IG_C_gene	IGHG2	ENSG00000211893.4	0	0
IG_C_gene	IGHA1	ENSG00000211895.5	0	0
IG_C_gene	IGHG1	ENSG00000211896.7	0	0
IG_C_gene	IGHG3	ENSG00000211897.9	0	0
IG_C_gene	IGHD	ENSG00000211898.7	0	0
IG_C_gene	IGHM	ENSG00000211899.10	0	0
IG_C_gene	IGLC1	ENSG00000211675.2	0	0
IG_C_gene	IGLC2	ENSG00000211677.2	0	0
IG_C_gene	IGLC3	ENSG00000211679.2	0	0
IG_C_gene	IGLC7	ENSG00000211685.3	0	0
IG_C_pseudogene	IGHEP2	ENSG00000254017.1	0	0
IG_C_pseudogene	IGHGP	ENSG00000253755.1	0	0
IG_C_pseudogene	IGHEP1	ENSG00000253692.3	0	0
IG_C_pseudogene	IGLJCOR18	ENSG00000265243.1	0	0
IG_C_pseudogene	IGLC4	ENSG00000254029.1	0	0
IG_C_pseudogene	IGLC5	ENSG00000254030.1	0	0
IG_C_pseudogene	IGLC6	ENSG00000222037.5	0	0
IG_C_pseudogene	IGLCOR22-1	ENSG00000254127.1	0	0
IG_C_pseudogene	IGLCOR22-2	ENSG00000271093.1	0	0
IG_D_gene	IGHD7-27	ENSG00000236597.1	0	0
IG_D_gene	IGHD1-26	ENSG00000211907.1	0	0
IG_D_gene	IGHD6-25	ENSG00000225825.1	0	0
IG_D_gene	IGHD5-24	ENSG00000211909.1	0	0
IG_D_gene	IGHD4-23	ENSG00000227196.1	0	0
IG_D_gene	IGHD3-22	ENSG00000211911.1	0	0
IG_D_gene	IGHD2-21	ENSG00000211912.1	0	0
IG_D_gene	IGHD1-20	ENSG00000237020.1	0	0
IG_D_gene	IGHD6-19	ENSG00000211914.1	0	0
IG_D_gene	IGHD5-18	ENSG00000211915.1	0	0
IG_D_gene	IGHD4-17	ENSG00000227800.1	0	0
IG_D_gene	IGHD3-16	ENSG00000211917.1	0	0
IG_D_gene	IGHD2-15	ENSG00000211918.1	0	0
IG_D_gene	IGHD1-14	ENSG00000227108.1	0	0

IG_D_gene	IGHD6-13	ENSG00000211920.1	0	1
IG_D_gene	IGHD5-12	ENSG00000211921.1	0	0
IG_D_gene	IGHD4-11	ENSG00000232543.2	0	0
IG_D_gene	IGHD3-10	ENSG00000211923.1	0	0
IG_D_gene	IGHD3-9	ENSG00000211924.1	0	0
IG_D_gene	IGHD2-8	ENSG00000211925.1	0	0
IG_D_gene	IGHD1-7	ENSG00000237197.1	0	0
IG_D_gene	IGHD6-6	ENSG00000228131.1	0	0
IG_D_gene	IGHD5-5	ENSG00000211928.1	0	0
IG_D_gene	IGHD4-4	ENSG00000233655.1	0	0
IG_D_gene	IGHD3-3	ENSG00000211930.1	0	0
IG_D_gene	IGHD2-2	ENSG00000211931.1	0	0
IG_D_gene	IGHD1-1	ENSG00000236170.1	0	0
IG_D_gene	IGHD5OR15-	ENSG00000270961.1	0	0
IG_D_gene	IGHD4OR15-	ENSG00000271317.1	0	0
IG_D_gene	IGHD3OR15-	ENSG00000282520.1	0	0
IG_D_gene	IGHD2OR15-	ENSG00000282599.1	0	0
IG_D_gene	IGHD1OR15-	ENSG00000271336.1	0	0
IG_D_gene	IGHD5OR15-	ENSG00000270824.1	0	0
IG_D_gene	IGHD4OR15-	ENSG00000270451.1	0	0
IG_D_gene	IGHD3OR15-	ENSG00000282089.1	0	0
IG_D_gene	IGHD2OR15-	ENSG00000282268.1	0	0
IG_D_gene	IGHD1OR15-	ENSG00000270185.1	0	0
IG_J_gene	IGKJ5	ENSG00000211593.2	0	0
IG_J_gene	IGKJ4	ENSG00000211594.2	0	0
IG_J_gene	IGKJ3	ENSG00000211595.2	0	0
IG_J_gene	IGKJ2	ENSG00000211596.3	0	0
IG_J_gene	IGKJ1	ENSG00000211597.2	0	0
IG_J_gene	IGHJ6	ENSG00000211900.2	0	0
IG_J_gene	IGHJ5	ENSG00000242472.1	0	0
IG_J_gene	IGHJ4	ENSG00000240041.1	0	0
IG_J_gene	IGHJ3	ENSG00000242887.1	0	0
IG_J_gene	IGHJ2	ENSG00000211904.2	0	0
IG_J_gene	IGHJ1	ENSG00000211905.1	0	0
IG_J_gene	IGLJ1	ENSG00000211674.2	0	0
IG_J_gene	IGLJ2	ENSG00000211676.2	0	0
IG_J_gene	IGLJ3	ENSG00000211678.2	0	0
IG_J_gene	IGLJ4	ENSG00000211680.2	0	0
IG_J_gene	IGLJ5	ENSG00000211681.2	0	0
IG_J_gene	IGLJ6	ENSG00000211682.2	0	0
IG_J_gene	IGLJ7	ENSG00000211684.2	0	0
IG_J_pseudogene	IGHJ3P	ENSG00000237111.1	0	0
IG_J_pseudogene	IGHJ2P	ENSG00000237547.1	0	0
IG_J_pseudogene	IGHJ1P	ENSG00000227335.1	0	0

IG_pseudogene	AC245369.1	ENSG00000283571.1	0	0
IG_V_gene	IGKV3OR2-	ENSG00000233999.3	0	0
IG_V_gene	IGKV4-1	ENSG00000211598.2	0	0
IG_V_gene	IGKV5-2	ENSG00000211599.2	0	0
IG_V_gene	IGKV1-5	ENSG00000243466.1	0	0
IG_V_gene	IGKV1-6	ENSG00000239855.1	0	0
IG_V_gene	IGKV3-7	ENSG00000243063.1	0	0
IG_V_gene	IGKV1-8	ENSG00000240671.4	0	0
IG_V_gene	IGKV1-9	ENSG00000241755.1	0	0
IG_V_gene	IGKV3-11	ENSG00000241351.3	0	0
IG_V_gene	IGKV1-12	ENSG00000243290.3	0	0
IG_V_gene	IGKV3-15	ENSG00000244437.1	0	0
IG_V_gene	IGKV1-16	ENSG00000240864.3	0	0
IG_V_gene	IGKV1-17	ENSG00000240382.3	0	0
IG_V_gene	IGKV3-20	ENSG00000239951.1	0	0
IG_V_gene	IGKV6-21	ENSG00000211611.2	0	0
IG_V_gene	IGKV2-24	ENSG00000241294.1	0	0
IG_V_gene	IGKV1-27	ENSG00000244575.3	0	0
IG_V_gene	IGKV2-28	ENSG00000244116.3	0	0
IG_V_gene	IGKV2-30	ENSG00000243238.1	0	0
IG_V_gene	IGKV1-33	ENSG00000242076.2	0	0
IG_V_gene	IGKV1-37	ENSG00000239862.1	0	0
IG_V_gene	IGKV1-39	ENSG00000242371.1	0	0
IG_V_gene	IGKV2-40	ENSG00000273962.1	0	0
IG_V_gene	IGKV2D-40	ENSG00000251039.2	0	0
IG_V_gene	IGKV1D-39	ENSG00000251546.1	0	0
IG_V_gene	IGKV1D-37	ENSG00000250036.1	0	0
IG_V_gene	IGKV1D-33	ENSG00000239975.2	0	0
IG_V_gene	IGKV2D-30	ENSG00000239571.1	0	0
IG_V_gene	IGKV2D-29	ENSG00000243264.2	0	0
IG_V_gene	IGKV2D-28	ENSG00000242534.2	0	0
IG_V_gene	IGKV2D-26	ENSG00000211623.2	0	0
IG_V_gene	IGKV2D-24	ENSG00000241566.1	0	0
IG_V_gene	IGKV6D-21	ENSG00000225523.2	0	0
IG_V_gene	IGKV3D-20	ENSG00000211625.2	0	0
IG_V_gene	IGKV1D-17	ENSG00000242766.1	0	0
IG_V_gene	IGKV1D-16	ENSG00000241244.1	0	0
IG_V_gene	IGKV3D-15	ENSG00000224041.3	0	0
IG_V_gene	IGKV1D-13	ENSG00000276566.1	0	0
IG_V_gene	IGKV1D-12	ENSG00000278857.1	0	0
IG_V_gene	IGKV3D-11	ENSG00000211632.4	0	0
IG_V_gene	IGKV1D-42	ENSG00000211633.3	0	0
IG_V_gene	IGKV1D-43	ENSG00000242580.1	0	0
IG_V_gene	IGKV1D-8	ENSG00000239819.2	0	0

IG_V_gene	IGKV3D-7	ENSG00000228325.5	0	0
IG_V_gene	IGKV1OR2-	ENSG00000231292.6	0	0
IG_V_gene	IGHV6-1	ENSG00000211933.2	0	0
IG_V_gene	IGHV1-2	ENSG00000211934.3	0	0
IG_V_gene	IGHV1-3	ENSG00000211935.3	0	0
IG_V_gene	IGHV4-4	ENSG00000276775.1	0	0
IG_V_gene	IGHV7-4-1	ENSG00000282122.1	0	0
IG_V_gene	IGHV2-5	ENSG00000211937.3	0	0
IG_V_gene	IGHV3-7	ENSG00000211938.2	0	0
IG_V_gene	IGHV3-64D	ENSG00000282639.1	0	0
IG_V_gene	IGHV5-10-1	ENSG00000282651.2	0	0
IG_V_gene	IGHV3-11	ENSG00000211941.3	0	0
IG_V_gene	IGHV3-13	ENSG00000211942.3	0	0
IG_V_gene	IGHV3-15	ENSG00000211943.2	0	0
IG_V_gene	IGHV3-16	ENSG00000211944.2	0	0
IG_V_gene	IGHV1-18	ENSG00000211945.2	0	0
IG_V_gene	IGHV3-20	ENSG00000211946.3	0	0
IG_V_gene	IGHV3-21	ENSG00000211947.2	0	0
IG_V_gene	IGHV3-23	ENSG00000211949.3	0	0
IG_V_gene	IGHV1-24	ENSG00000211950.2	0	0
IG_V_gene	IGHV2-26	ENSG00000211951.2	0	0
IG_V_gene	IGHV4-28	ENSG00000211952.3	0	0
IG_V_gene	IGHV3-30	ENSG00000270550.1	0	0
IG_V_gene	IGHV4-31	ENSG00000231475.3	0	0
IG_V_gene	IGHV3-33	ENSG00000211955.2	0	0
IG_V_gene	IGHV4-34	ENSG00000211956.2	0	0
IG_V_gene	IGHV3-35	ENSG00000211957.2	0	0
IG_V_gene	IGHV3-38	ENSG00000211958.2	0	0
IG_V_gene	IGHV4-39	ENSG00000211959.2	0	0
IG_V_gene	IGHV3-43	ENSG00000232216.1	0	0
IG_V_gene	IGHV1-45	ENSG00000211961.3	0	0
IG_V_gene	IGHV1-46	ENSG00000211962.2	0	0
IG_V_gene	IGHV3-48	ENSG00000211964.3	0	0
IG_V_gene	IGHV3-49	ENSG00000211965.4	0	0
IG_V_gene	IGHV5-51	ENSG00000211966.2	0	0
IG_V_gene	IGHV3-53	ENSG00000211967.3	0	0
IG_V_gene	IGHV1-58	ENSG00000211968.3	0	0
IG_V_gene	IGHV4-59	ENSG00000224373.3	0	0
IG_V_gene	IGHV4-61	ENSG00000211970.3	0	0
IG_V_gene	IGHV3-64	ENSG00000223648.4	0	0
IG_V_gene	IGHV3-66	ENSG00000211972.2	0	0
IG_V_gene	IGHV1-69	ENSG00000211973.2	0	0
IG_V_gene	IGHV2-70D	ENSG00000211974.3	0	0
IG_V_gene	IGHV1-69-2	ENSG00000281990.1	0	0

IG_V_gene	IGHV1-69D	ENSG00000280411.1	0	0
IG_V_gene	IGHV2-70	ENSG00000274576.2	0	0
IG_V_gene	IGHV3-72	ENSG00000225698.3	0	0
IG_V_gene	IGHV3-73	ENSG00000211976.2	0	0
IG_V_gene	IGHV3-74	ENSG00000224650.2	0	0
IG_V_gene	IGHV7-81	ENSG00000211979.2	0	0
IG_V_gene	IGHV1OR15-	ENSG00000188403.7	0	0
IG_V_gene	IGHV3OR15-	ENSG00000259490.2	0	0
IG_V_gene	AC135068.8	ENSG00000281179.1	0	0
IG_V_gene	AC135068.2	ENSG00000278263.2	0	0
IG_V_gene	IGHV1OR15-	ENSG00000270505.1	0	0
IG_V_gene	IGHV4OR15-	ENSG00000259261.2	0	0
IG_V_gene	IGHV3OR16-	ENSG00000270472.2	0	0
IG_V_gene	IGHV2OR16-	ENSG00000259303.5	0	0
IG_V_gene	IGHV3OR16-	ENSG00000233732.5	0	0
IG_V_gene	IGHV3OR16-	ENSG00000271130.1	0	0
IG_V_gene	IGHV3OR16-	ENSG00000270467.1	0	0
IG_V_gene	IGHV3OR16-	ENSG00000271178.1	0	0
IG_V_gene	IGHV1OR21-	ENSG00000277282.1	0	0
IG_V_gene	IGLV4-69	ENSG00000211637.2	0	0
IG_V_gene	IGLV10-54	ENSG00000211642.3	0	0
IG_V_gene	IGLV8-61	ENSG00000211638.2	0	0
IG_V_gene	IGLV4-60	ENSG00000211639.2	0	0
IG_V_gene	IGLV6-57	ENSG00000211640.4	0	0
IG_V_gene	IGLV11-55	ENSG00000211641.3	0	0
IG_V_gene	IGLV5-52	ENSG00000211643.2	0	0
IG_V_gene	IGLV1-51	ENSG00000211644.3	0	0
IG_V_gene	IGLV1-50	ENSG00000211645.2	0	0
IG_V_gene	IGLV9-49	ENSG00000223350.2	0	0
IG_V_gene	IGLV5-48	ENSG00000211647.1	0	0
IG_V_gene	IGLV1-47	ENSG00000211648.2	0	0
IG_V_gene	IGLV7-46	ENSG00000211649.3	0	0
IG_V_gene	IGLV5-45	ENSG00000211650.2	0	0
IG_V_gene	IGLV1-44	ENSG00000211651.3	0	0
IG_V_gene	IGLV7-43	ENSG00000211652.2	0	0
IG_V_gene	IGLV1-40	ENSG00000211653.2	0	0
IG_V_gene	IGLV5-37	ENSG00000211654.2	0	0
IG_V_gene	IGLV1-36	ENSG00000211655.3	0	0
IG_V_gene	IGLV2-33	ENSG00000211656.3	0	0
IG_V_gene	IGLV3-32	ENSG00000211657.3	0	0
IG_V_gene	IGLV3-27	ENSG00000211658.2	0	0
IG_V_gene	IGLV3-25	ENSG00000211659.2	0	0
IG_V_gene	IGLV2-23	ENSG00000211660.3	0	0
IG_V_gene	IGLV3-22	ENSG00000211661.2	0	0

IG_V_gene	IGLV3-21	ENSG00000211662.2	0	0
IG_V_gene	IGLV3-19	ENSG00000211663.2	0	0
IG_V_gene	IGLV2-18	ENSG00000211664.3	0	0
IG_V_gene	IGLV3-16	ENSG00000211665.3	0	0
IG_V_gene	IGLV2-14	ENSG00000211666.2	0	0
IG_V_gene	IGLV3-12	ENSG00000211667.3	0	0
IG_V_gene	IGLV2-11	ENSG00000211668.2	0	0
IG_V_gene	IGLV3-10	ENSG00000211669.3	0	0
IG_V_gene	IGLV3-9	ENSG00000211670.2	0	0
IG_V_gene	IGLV2-8	ENSG00000278196.3	0	0
IG_V_gene	IGLV4-3	ENSG00000211672.2	0	0
IG_V_gene	IGLV3-1	ENSG00000211673.2	0	0
IG_V_pseudogene	IGKV1OR1-1	ENSG00000276674.1	0	0
IG_V_pseudogene	IGKV7-3	ENSG00000197794.2	0	0
IG_V_pseudogene	IGKV2-4	ENSG00000253435.1	0	0
IG_V_pseudogene	IGKV2-10	ENSG00000253278.1	0	0
IG_V_pseudogene	IGKV1-13	ENSG00000253497.1	0	0
IG_V_pseudogene	IGKV2-14	ENSG00000253265.1	0	0
IG_V_pseudogene	IGKV2-18	ENSG00000254157.1	0	0
IG_V_pseudogene	IGKV2-19	ENSG00000253732.1	0	0
IG_V_pseudogene	IGKV1-22	ENSG00000253578.1	0	0
IG_V_pseudogene	IGKV2-23	ENSG00000253625.1	0	0
IG_V_pseudogene	IGKV3-25	ENSG00000253202.1	0	0
IG_V_pseudogene	IGKV2-26	ENSG00000254098.1	0	0
IG_V_pseudogene	IGKV2-29	ENSG00000253998.3	0	0
IG_V_pseudogene	IGKV3-31	ENSG00000253158.1	0	0
IG_V_pseudogene	IGKV1-32	ENSG00000253870.1	0	0
IG_V_pseudogene	IGKV3-34	ENSG00000253860.1	0	0
IG_V_pseudogene	IGKV1-35	ENSG00000253461.1	0	0
IG_V_pseudogene	IGKV2-36	ENSG00000253487.1	0	0
IG_V_pseudogene	IGKV2-38	ENSG00000253592.1	0	0
IG_V_pseudogene	IGKV2D-38	ENSG00000254009.1	0	0
IG_V_pseudogene	IGKV2D-36	ENSG00000253127.1	0	0
IG_V_pseudogene	IGKV1D-35	ENSG00000232747.2	0	0
IG_V_pseudogene	IGKV3D-34	ENSG00000253501.1	0	0
IG_V_pseudogene	IGKV1D-32	ENSG00000253191.1	0	0
IG_V_pseudogene	IGKV3D-31	ENSG00000253999.1	0	0
IG_V_pseudogene	IGKV1D-27	ENSG00000224607.4	0	0
IG_V_pseudogene	IGKV3D-25	ENSG00000254097.1	0	0
IG_V_pseudogene	IGKV2D-23	ENSG00000254345.1	0	0
IG_V_pseudogene	IGKV1D-22	ENSG00000253365.1	0	0
IG_V_pseudogene	IGKV2D-19	ENSG00000253765.1	0	0
IG_V_pseudogene	IGKV2D-18	ENSG00000254220.1	0	0
IG_V_pseudogene	IGKV2D-14	ENSG00000254292.1	0	0

IG_V_pseudogene	IGKV2D-10	ENSG00000253906.1	0	0
IG_V_pseudogene	IGKV1OR2-	ENSG00000270999.1	0	0
IG_V_pseudogene	IGKV1OR2-1	ENSG00000235235.6	0	0
IG_V_pseudogene	IGKV2OR2-1	ENSG00000270748.1	0	0
IG_V_pseudogene	IGKV2OR2-2	ENSG00000271402.1	0	0
IG_V_pseudogene	IGKV1OR2-3	ENSG00000204670.7	0	0
IG_V_pseudogene	IGKV1OR2-9	ENSG00000271351.1	0	0
IG_V_pseudogene	IGKV2OR2-	ENSG00000276050.1	0	0
IG_V_pseudogene	IGKV2OR2-	ENSG00000271015.1	0	0
IG_V_pseudogene	IGKV3OR2-5	ENSG00000270252.1	0	0
IG_V_pseudogene	IGKV1OR2-6	ENSG00000271569.1	0	0
IG_V_pseudogene	IGKV2OR2-7	ENSG00000270450.1	0	0
IG_V_pseudogene	IGKV2OR2-8	ENSG00000278537.1	0	0
IG_V_pseudogene	IGKV1OR2-	ENSG00000270187.1	0	0
IG_V_pseudogene	IGLV8OR8-1	ENSG00000215177.3	0	0
IG_V_pseudogene	IGKV1OR9-2	ENSG00000225380.4	0	0
IG_V_pseudogene	IGKV1OR-2	ENSG00000156755.10	0	0
IG_V_pseudogene	IGKV1OR9-1	ENSG00000204780.5	0	0
IG_V_pseudogene	IGKV1OR-3	ENSG00000204776.6	0	0
IG_V_pseudogene	IGKV1OR10-	ENSG00000237592.2	0	0
IG_V_pseudogene	IGHVII-1-1	ENSG00000254279.1	0	0
IG_V_pseudogene	IGHVIII-2-1	ENSG00000253780.1	0	0
IG_V_pseudogene	IGHVIII-5-1	ENSG00000253387.1	0	0
IG_V_pseudogene	IGHVIII-5-2	ENSG00000254053.1	0	0
IG_V_pseudogene	IGHV3-6	ENSG00000253763.1	0	0
IG_V_pseudogene	IGHVIII-11-1	ENSG00000254215.1	0	0
IG_V_pseudogene	IGHV1-12	ENSG00000254174.1	0	0
IG_V_pseudogene	IGHVIII-13-1	ENSG00000253412.1	0	0
IG_V_pseudogene	IGHV1-14	ENSG00000253709.1	0	0
IG_V_pseudogene	IGHVII-15-1	ENSG00000253458.1	0	0
IG_V_pseudogene	IGHVIII-16-1	ENSG00000283948.1	0	0
IG_V_pseudogene	IGHV1-17	ENSG00000254046.1	0	0
IG_V_pseudogene	IGHV3-19	ENSG00000253883.1	0	0
IG_V_pseudogene	IGHV3-22	ENSG00000253957.1	0	0
IG_V_pseudogene	IGHVII-22-1	ENSG00000253345.1	0	0
IG_V_pseudogene	IGHVIII-22-2	ENSG00000254045.1	0	0
IG_V_pseudogene	IGHV3-25	ENSG00000253441.1	0	0
IG_V_pseudogene	IGHVIII-25-1	ENSG00000253367.1	0	0
IG_V_pseudogene	IGHVIII-26-1	ENSG00000253462.1	0	0
IG_V_pseudogene	IGHVII-26-2	ENSG00000253482.1	0	0
IG_V_pseudogene	IGHV7-27	ENSG00000254326.1	0	0
IG_V_pseudogene	IGHVII-28-1	ENSG00000253149.3	0	0
IG_V_pseudogene	IGHV3-32	ENSG00000254289.2	0	0
IG_V_pseudogene	IGHVII-30-1	ENSG00000253491.2	0	0

IG_V_pseudogene	IGHV3-30-2	ENSG00000253587.2	0	0
IG_V_pseudogene	IGHVII-30-21	ENSG00000283607.2	0	0
IG_V_pseudogene	IGHV3-29	ENSG00000270474.2	0	0
IG_V_pseudogene	IGHVII-33-1	ENSG00000254203.1	0	0
IG_V_pseudogene	IGHV3-33-2	ENSG00000253440.1	0	0
IG_V_pseudogene	IGHV7-34-1	ENSG00000253325.1	0	0
IG_V_pseudogene	IGHV3-36	ENSG00000253240.1	0	0
IG_V_pseudogene	IGHV3-37	ENSG00000253359.1	0	0
IG_V_pseudogene	IGHVIII-38-1	ENSG00000253989.2	0	0
IG_V_pseudogene	IGHV7-40	ENSG00000253467.2	0	0
IG_V_pseudogene	IGHVII-40-1	ENSG00000253294.1	0	0
IG_V_pseudogene	IGHV3-41	ENSG00000278473.1	0	0
IG_V_pseudogene	IGHV3-42	ENSG00000254228.2	0	0
IG_V_pseudogene	IGHVII-43-1	ENSG00000283195.1	0	0
IG_V_pseudogene	IGHVIII-44	ENSG00000283562.1	0	0
IG_V_pseudogene	AC244452.3	ENSG00000283464.1	0	0
IG_V_pseudogene	IGHVIV-44-1	ENSG00000253465.1	0	0
IG_V_pseudogene	IGHVII-44-2	ENSG00000253895.1	0	0
IG_V_pseudogene	IGHVII-46-1	ENSG00000253808.1	0	0
IG_V_pseudogene	IGHV3-47	ENSG00000229092.2	0	0
IG_V_pseudogene	IGHVIII-47-1	ENSG00000253862.1	0	0
IG_V_pseudogene	IGHVII-49-1	ENSG00000253386.1	0	0
IG_V_pseudogene	IGHV3-50	ENSG00000253241.1	0	0
IG_V_pseudogene	IGHVIII-51-1	ENSG00000254167.1	0	0
IG_V_pseudogene	IGHVII-51-2	ENSG00000253941.1	0	0
IG_V_pseudogene	IGHV3-52	ENSG00000253545.1	0	0
IG_V_pseudogene	IGHVII-53-1	ENSG00000253714.1	0	0
IG_V_pseudogene	IGHV3-54	ENSG00000275475.1	0	0
IG_V_pseudogene	IGHV4-55	ENSG00000254395.1	0	0
IG_V_pseudogene	IGHV7-56	ENSG00000253131.1	0	0
IG_V_pseudogene	IGHV3-57	ENSG00000253759.1	0	0
IG_V_pseudogene	IGHV3-60	ENSG00000253742.1	0	0
IG_V_pseudogene	IGHVII-60-1	ENSG00000254329.1	0	0
IG_V_pseudogene	IGHV3-62	ENSG00000253132.1	0	0
IG_V_pseudogene	IGHVII-62-1	ENSG00000253747.1	0	0
IG_V_pseudogene	IGHV3-63	ENSG00000253936.1	0	0
IG_V_pseudogene	IGHV3-65	ENSG00000253209.1	0	0
IG_V_pseudogene	IGHVII-65-1	ENSG00000253169.2	0	0
IG_V_pseudogene	IGHV1-67	ENSG00000253274.1	0	0
IG_V_pseudogene	IGHVII-67-1	ENSG00000253820.1	0	0
IG_V_pseudogene	IGHVIII-67-2	ENSG00000254036.1	0	0
IG_V_pseudogene	IGHVIII-67-3	ENSG00000253635.1	0	0
IG_V_pseudogene	IGHVIII-67-4	ENSG00000254052.1	0	0
IG_V_pseudogene	IGHV1-68	ENSG00000253703.2	0	0

IG_V_pseudogene	IGHV3-69-1	ENSG00000282600.2	0	0
IG_V_pseudogene	IGHV3-71	ENSG00000254056.1	0	0
IG_V_pseudogene	IGHVII-74-1	ENSG00000254134.1	0	0
IG_V_pseudogene	IGHV3-75	ENSG00000254176.1	0	0
IG_V_pseudogene	IGHV3-76	ENSG00000253247.1	0	0
IG_V_pseudogene	IGHVIII-76-1	ENSG00000253310.1	0	0
IG_V_pseudogene	IGHV5-78	ENSG00000211978.2	0	0
IG_V_pseudogene	IGHVII-78-1	ENSG00000253674.1	0	0
IG_V_pseudogene	IGHV3-79	ENSG00000275747.1	0	0
IG_V_pseudogene	IGHV4-80	ENSG00000283906.1	0	0
IG_V_pseudogene	IGHVIII-82	ENSG00000253303.1	0	0
IG_V_pseudogene	IGHV1OR15-	ENSG00000259337.4	0	0
IG_V_pseudogene	IGHV1OR15-	ENSG00000270685.1	0	0
IG_V_pseudogene	AC135068.9	ENSG00000281550.1	0	0
IG_V_pseudogene	AC134981.1	ENSG00000280655.1	0	0
IG_V_pseudogene	IGHV1OR15-	ENSG00000271288.1	0	0
IG_V_pseudogene	IGHV1OR15-	ENSG00000270356.1	0	0
IG_V_pseudogene	IGHV1OR16-	ENSG00000261704.2	0	0
IG_V_pseudogene	IGHV1OR16-	ENSG00000260048.2	0	0
IG_V_pseudogene	IGHV3OR16-	ENSG00000261834.2	0	0
IG_V_pseudogene	IGHV3OR16-	ENSG00000270864.1	0	0
IG_V_pseudogene	IGHV1OR16-	ENSG00000259852.2	0	0
IG_V_pseudogene	IGHV1OR16-	ENSG00000259997.2	0	0
IG_V_pseudogene	IGHV3OR16-	ENSG00000270318.1	0	0
IG_V_pseudogene	IGHV3OR16-	ENSG00000271620.1	0	0
IG_V_pseudogene	IGKV1OR22-	ENSG00000230481.2	0	0
IG_V_pseudogene	IGKV2OR22-	ENSG00000253691.2	0	0
IG_V_pseudogene	IGKV2OR22-	ENSG00000253460.2	0	0
IG_V_pseudogene	IGKV3OR22-	ENSG00000254264.2	0	0
IG_V_pseudogene	IGKV1OR22-	ENSG00000253481.2	0	0
IG_V_pseudogene	IGLVI-70	ENSG00000253239.1	0	0
IG_V_pseudogene	IGLVI-68	ENSG00000254355.1	0	0
IG_V_pseudogene	IGLV10-67	ENSG00000253794.1	0	0
IG_V_pseudogene	IGLVIV-66-1	ENSG00000253874.1	0	0
IG_V_pseudogene	IGLVV-66	ENSG00000254075.1	0	0
IG_V_pseudogene	IGLVIV-65	ENSG00000254161.1	0	0
IG_V_pseudogene	IGLVIV-64	ENSG00000253242.1	0	0
IG_V_pseudogene	IGLVI-63	ENSG00000253752.1	0	0
IG_V_pseudogene	IGLV1-62	ENSG00000253823.1	0	0
IG_V_pseudogene	IGLVIV-59	ENSG00000254308.1	0	0
IG_V_pseudogene	IGLVV-58	ENSG00000253637.1	0	0
IG_V_pseudogene	IGLVI-56	ENSG00000253126.1	0	0
IG_V_pseudogene	IGLVIV-53	ENSG00000253935.1	0	0
IG_V_pseudogene	IGLVI-42	ENSG00000254175.1	0	0

IG_V_pseudogene	IGLVVII-41-1	ENSG00000254073.1	0	0
IG_V_pseudogene	IGLV1-41	ENSG00000253818.1	0	0
IG_V_pseudogene	IGLVI-38	ENSG00000253889.1	0	0
IG_V_pseudogene	IGLV7-35	ENSG00000253631.1	0	0
IG_V_pseudogene	IGLV2-34	ENSG00000253120.1	0	0
IG_V_pseudogene	IGLV3-31	ENSG00000253920.1	0	0
IG_V_pseudogene	IGLV3-30	ENSG00000253329.1	0	0
IG_V_pseudogene	IGLV3-29	ENSG00000253338.1	0	0
IG_V_pseudogene	IGLV2-28	ENSG00000253451.1	0	0
IG_V_pseudogene	IGLV3-26	ENSG00000253913.1	0	0
IG_V_pseudogene	IGLVVI-25-1	ENSG00000253779.1	0	0
IG_V_pseudogene	IGLV3-24	ENSG00000253822.1	0	0
IG_V_pseudogene	IGLVVI-22-1	ENSG00000253546.1	0	0
IG_V_pseudogene	IGLVI-20	ENSG00000254240.1	0	0
IG_V_pseudogene	IGLV3-17	ENSG00000253152.1	0	0
IG_V_pseudogene	IGLV3-15	ENSG00000253786.1	0	0
IG_V_pseudogene	IGLV3-13	ENSG00000253590.1	0	0
IG_V_pseudogene	IGLV3-7	ENSG00000254077.1	0	0
IG_V_pseudogene	IGLV3-6	ENSG00000253448.1	0	0
IG_V_pseudogene	IGLV2-5	ENSG00000253234.1	0	0
IG_V_pseudogene	IGLV3-4	ENSG00000226420.1	0	0
IG_V_pseudogene	IGLV3-2	ENSG00000253963.1	0	0
IG_V_pseudogene	IGLVIVOR22-	ENSG00000271138.1	0	0
IG_V_pseudogene	IGLVIVOR22-	ENSG00000243519.1	0	0
lincRNA	MIR1302-	ENSG00000243485.5	0	0
lincRNA	FAM138A	ENSG00000237613.2	0	0
lincRNA	AL627309.1	ENSG00000238009.6	0	0
lincRNA	AL627309.3	ENSG00000239945.1	0	0
lincRNA	AL627309.4	ENSG00000241599.1	0	0
lincRNA	AL732372.1	ENSG00000236601.2	0	0
lincRNA	AC114498.1	ENSG00000235146.2	0	0
lincRNA	FAM87B	ENSG00000177757.2	0	1
lincRNA	FAM41C	ENSG00000230368.2	0	0
lincRNA	AL645608.6	ENSG00000272438.1	0	0
lincRNA	AL645608.4	ENSG00000241180.1	0	0
lincRNA	AL645608.8	ENSG00000273443.1	0	0
lincRNA	LINC01342	ENSG00000223823.1	0	0
lincRNA	LINC01786	ENSG00000230415.1	0	0
lincRNA	LINC01770	ENSG00000225285.1	0	0
lincRNA	AL590822.1	ENSG00000234396.3	0	0
lincRNA	AC242022.2	ENSG00000285945.1	0	0
lincRNA	AL592464.2	ENSG00000233234.1	0	0
lincRNA	AL592464.1	ENSG00000231630.1	0	0
lincRNA	AL589702.1	ENSG00000284745.1	0	0

lincRNA	AL590438.1	ENSG00000272235.1	0	0
lincRNA	AL512413.1	ENSG00000272088.1	0	0
lincRNA	LINC01346	ENSG00000233304.6	0	0
lincRNA	LINC01345	ENSG00000226374.2	0	0
lincRNA	AL805961.1	ENSG00000284668.1	0	0
lincRNA	AL805961.2	ENSG00000284703.1	0	0
lincRNA	LINC01777	ENSG00000235054.3	0	0
lincRNA	AL355602.1	ENSG00000284694.1	0	0
lincRNA	Z98747.1	ENSG00000227169.2	0	0
lincRNA	LINC01646	ENSG00000232596.2	0	0
lincRNA	BX005132.2	ENSG00000284739.1	0	0
lincRNA	BX005132.1	ENSG00000284674.1	0	0
lincRNA	AL691497.1	ENSG00000231510.1	0	0
lincRNA	AL139823.1	ENSG00000284616.1	0	0
lincRNA	Z98259.3	ENSG00000284692.2	0	0
lincRNA	Z98259.2	ENSG00000284666.1	0	0
lincRNA	Z98259.1	ENSG00000260972.1	0	0
lincRNA	AL365255.1	ENSG00000236948.2	0	0
lincRNA	LINC00337	ENSG00000225077.3	0	0
lincRNA	LINC01672	ENSG00000228750.6	0	0
lincRNA	AL591163.1	ENSG00000284744.1	0	0
lincRNA	AL359881.3	ENSG00000270171.1	0	0
lincRNA	AL359881.2	ENSG00000270035.1	0	0
lincRNA	AL359881.1	ENSG00000269978.1	0	0
lincRNA	AL034417.3	ENSG00000284716.1	0	0
lincRNA	AL034417.2	ENSG00000238290.1	0	1
lincRNA	LINC01714	ENSG00000227634.2	0	0
lincRNA	AL358876.2	ENSG00000233645.1	0	0
lincRNA	BX323043.1	ENSG00000284652.1	0	0
lincRNA	AL928921.1	ENSG00000235263.1	0	0
lincRNA	AL139424.3	ENSG00000284735.1	0	0
lincRNA	AL031291.1	ENSG00000284646.1	0	0
lincRNA	LINC01647	ENSG00000235643.1	0	0
lincRNA	AL021155.2	ENSG00000285646.1	0	0
lincRNA	AC243836.1	ENSG00000229484.1	0	0
lincRNA	LINC01784	ENSG00000228338.1	0	0
lincRNA	AC245056.1	ENSG00000233336.1	0	0
lincRNA	AC243961.1	ENSG00000228229.2	0	0
lincRNA	AL359771.1	ENSG00000237445.2	0	0
lincRNA	AL357873.1	ENSG00000231606.1	0	0
lincRNA	KAZN-AS1	ENSG00000234593.2	0	0
lincRNA	C1orf195	ENSG00000204464.7	0	0
lincRNA	AL031283.1	ENSG00000228140.1	0	0
lincRNA	AL121992.2	ENSG00000271742.1	0	0

lincRNA	AL137802.2	ENSG00000261135.1	0	0
lincRNA	AL137802.3	ENSG00000285853.1	0	0
lincRNA	AL137802.1	ENSG00000224174.2	0	0
lincRNA	LINC01783	ENSG00000233421.4	0	0
lincRNA	AL137798.1	ENSG00000271732.1	0	0
lincRNA	AL137798.2	ENSG00000282143.1	0	0
lincRNA	AL021920.1	ENSG00000279151.1	0	0
lincRNA	AL021920.3	ENSG00000282740.1	0	0
lincRNA	BX284668.1	ENSG00000223643.6	0	0
lincRNA	BX284668.2	ENSG00000228549.3	0	0
lincRNA	BX284668.6	ENSG00000272426.1	0	0
lincRNA	AL590644.3	ENSG00000282843.1	0	0
lincRNA	AL136529.1	ENSG00000236648.1	0	0
lincRNA	AL357509.1	ENSG00000284653.1	0	0
lincRNA	LINC01654	ENSG00000261781.1	0	0
lincRNA	AL591896.1	ENSG00000225387.1	0	0
lincRNA	Z98257.1	ENSG00000227066.1	0	0
lincRNA	LINC01757	ENSG00000228105.1	0	0
lincRNA	Z98257.2	ENSG00000284710.1	0	0
lincRNA	LINC01141	ENSG00000236963.6	0	0
lincRNA	AL139254.1	ENSG00000226487.1	0	0
lincRNA	AL139254.2	ENSG00000284641.1	0	0
lincRNA	AL139254.3	ENSG00000284743.1	0	0
lincRNA	AL592309.2	ENSG00000236009.1	0	0
lincRNA	LINC01635	ENSG00000228397.2	0	0
lincRNA	AL109936.6	ENSG00000284726.1	0	0
lincRNA	AL109936.2	ENSG00000271420.1	0	2
lincRNA	AL590683.1	ENSG00000230023.2	0	0
lincRNA	AL445686.2	ENSG00000264443.1	0	0
lincRNA	AL445648.1	ENSG00000284699.1	0	0
lincRNA	AL445471.2	ENSG00000261025.1	0	0
lincRNA	AL050344.2	ENSG00000285823.1	0	0
lincRNA	AL050344.1	ENSG00000233755.1	0	0
lincRNA	FO393419.2	ENSG00000241169.1	0	0
lincRNA	LINC02574	ENSG00000233975.1	0	0
lincRNA	AL353622.2	ENSG00000271398.1	0	0
lincRNA	AL360012.1	ENSG00000270103.3	0	0
lincRNA	AL357500.1	ENSG00000225750.1	0	0
lincRNA	LINC01756	ENSG00000230523.1	0	0
lincRNA	AC092265.1	ENSG00000228176.1	0	0
lincRNA	AL645944.2	ENSG00000284676.1	0	0
lincRNA	LINC01648	ENSG00000233399.2	0	0
lincRNA	AL137076.1	ENSG00000233372.1	0	0
lincRNA	AL161638.2	ENSG00000236335.1	0	0

lincRNA	AL161638.1	ENSG00000231949.1	0	0
lincRNA	AL137027.1	ENSG00000229607.1	0	0
lincRNA	AL354919.1	ENSG00000203620.2	0	0
lincRNA	AL136115.2	ENSG00000269967.1	0	0
lincRNA	AL136115.1	ENSG00000228634.1	0	0
lincRNA	AL136115.3	ENSG00000284702.1	0	0
lincRNA	AL109945.1	ENSG00000233775.1	0	0
lincRNA	AL662907.3	ENSG00000284721.1	0	0
lincRNA	AL513327.3	ENSG00000270115.1	0	0
lincRNA	AL590434.1	ENSG00000284640.1	0	0
lincRNA	AL139286.2	ENSG00000271914.1	0	0
lincRNA	AL596257.1	ENSG00000284705.1	0	0
lincRNA	AC117945.2	ENSG00000284720.1	0	0
lincRNA	AL031430.1	ENSG00000284650.1	0	0
lincRNA	AL353604.1	ENSG00000223944.1	0	0
lincRNA	AL139158.2	ENSG00000224592.5	0	0
lincRNA	AL139158.3	ENSG00000284623.1	0	0
lincRNA	LINC01343	ENSG00000237290.2	0	0
lincRNA	LINC01685	ENSG00000238063.2	0	0
lincRNA	AL354702.2	ENSG00000284632.1	0	0
lincRNA	AL033527.6	ENSG00000285905.1	0	0
lincRNA	AL033527.5	ENSG00000284719.1	0	0
lincRNA	AL033527.4	ENSG00000284678.1	0	0
lincRNA	AL031985.4	ENSG00000284677.1	0	0
lincRNA	AL031289.1	ENSG00000237899.1	0	0
lincRNA	AC093151.3	ENSG00000235358.1	0	0
lincRNA	AC099795.1	ENSG00000283973.1	0	0
lincRNA	LINC01144	ENSG00000281912.1	0	0
lincRNA	FOXD2-AS1	ENSG00000237424.1	0	3
lincRNA	LINC01738	ENSG00000227947.1	0	0
lincRNA	AL691459.1	ENSG00000223814.1	0	0
lincRNA	AC096541.1	ENSG00000225028.1	0	0
lincRNA	AL356289.1	ENSG00000226133.6	0	0
lincRNA	AL356289.2	ENSG00000231413.1	0	0
lincRNA	AL109659.1	ENSG00000223720.2	0	0
lincRNA	AL596275.2	ENSG00000284696.1	0	0
lincRNA	AL592182.3	ENSG00000284645.1	0	0
lincRNA	LINC01562	ENSG00000203356.2	0	0
lincRNA	AL591167.1	ENSG00000272371.1	0	0
lincRNA	AC119428.3	ENSG00000285954.1	0	0
lincRNA	AC119428.1	ENSG00000226938.1	0	0
lincRNA	AC119428.2	ENSG00000230138.1	0	0
lincRNA	AL035415.1	ENSG00000232245.1	0	0
lincRNA	AC099796.1	ENSG00000235741.1	0	0

lincRNA	MIR4422HG	ENSG00000231090.2	0	0
lincRNA	LINC01755	ENSG00000233079.2	0	0
lincRNA	LINC01753	ENSG00000230250.2	0	0
lincRNA	AL353681.1	ENSG00000235612.1	0	0
lincRNA	LINC01767	ENSG00000223956.2	0	0
lincRNA	AL390243.1	ENSG00000227935.1	0	0
lincRNA	AL445193.2	ENSG00000235038.1	0	0
lincRNA	LINC01135	ENSG00000234807.6	0	0
lincRNA	AL136985.2	ENSG00000272226.1	0	0
lincRNA	AL136985.1	ENSG00000231740.1	0	0
lincRNA	AL592431.2	ENSG00000241042.1	0	0
lincRNA	AL592431.1	ENSG00000235215.2	0	0
lincRNA	AC093424.1	ENSG00000270457.1	0	0
lincRNA	AC097066.1	ENSG00000238242.1	0	0
lincRNA	LINC01748	ENSG00000226476.3	0	0
lincRNA	AC099792.1	ENSG00000231252.2	0	0
lincRNA	AC099791.3	ENSG00000284808.1	0	0
lincRNA	AC099794.1	ENSG00000234318.1	0	0
lincRNA	LINC01739	ENSG00000229537.6	0	0
lincRNA	AL162400.1	ENSG00000227485.1	0	0
lincRNA	AL162400.2	ENSG00000229225.1	0	0
lincRNA	AC096543.1	ENSG00000203605.3	0	0
lincRNA	AC096543.2	ENSG00000228734.2	0	0
lincRNA	AL445205.1	ENSG00000284928.1	0	0
lincRNA	AL357078.3	ENSG00000272506.1	0	0
lincRNA	AL357078.1	ENSG00000231485.1	0	0
lincRNA	AL513493.1	ENSG00000285079.1	0	0
lincRNA	LINC01702	ENSG00000235200.2	0	0
lincRNA	AL035412.1	ENSG00000285041.1	0	0
lincRNA	AL691520.1	ENSG00000285473.1	0	0
lincRNA	LINC01707	ENSG00000223883.1	0	0
lincRNA	AL512271.1	ENSG00000235446.2	0	0
lincRNA	LINC01758	ENSG00000229639.1	0	0
lincRNA	AL354872.2	ENSG00000271992.1	0	4
lincRNA	LINC01788	ENSG00000229051.1	0	0
lincRNA	AL513285.1	ENSG00000226208.1	0	0
lincRNA	AL583808.2	ENSG00000227016.3	0	0
lincRNA	AL583808.3	ENSG00000285808.1	0	0
lincRNA	LINC02238	ENSG00000223479.3	0	0
lincRNA	AL591463.1	ENSG00000285778.1	0	0
lincRNA	AC133865.1	ENSG00000229943.1	0	0
lincRNA	AC092813.1	ENSG00000225605.2	0	0
lincRNA	AC092813.2	ENSG00000230027.1	0	0
lincRNA	AC104458.1	ENSG00000272855.1	0	0

lincRNA	LINC02567	ENSG00000237552.1	0	0
lincRNA	AC103591.4	ENSG00000285928.1	0	0
lincRNA	AL353651.2	ENSG00000282898.1	0	0
lincRNA	AL353651.1	ENSG00000235011.2	0	0
lincRNA	AL353651.3	ENSG00000285462.1	0	0
lincRNA	AC099671.1	ENSG00000285409.1	0	0
lincRNA	AC098657.2	ENSG00000260322.1	0	0
lincRNA	AL606519.1	ENSG00000225598.1	0	0
lincRNA	AL136234.1	ENSG00000224326.1	0	0
lincRNA	AC093154.1	ENSG00000285179.1	0	0
lincRNA	AL157944.1	ENSG00000233290.2	0	0
lincRNA	LINC01362	ENSG00000230817.5	0	0
lincRNA	LINC01361	ENSG00000236268.5	0	0
lincRNA	AC116099.1	ENSG00000285782.1	0	0
lincRNA	LINC01712	ENSG00000231364.2	0	0
lincRNA	AL035706.1	ENSG00000237076.1	0	0
lincRNA	AC104454.2	ENSG00000285201.1	0	0
lincRNA	AL844170.1	ENSG00000285361.1	0	0
lincRNA	AL359762.2	ENSG00000285374.1	0	0
lincRNA	AC104169.1	ENSG00000285325.1	0	0
lincRNA	AC092807.2	ENSG00000272691.1	0	0
lincRNA	AL355981.1	ENSG00000284846.1	0	0
lincRNA	LINC01140	ENSG00000267272.5	0	0
lincRNA	AC099062.1	ENSG00000284240.1	0	0
lincRNA	LINC01364	ENSG00000227290.1	0	0
lincRNA	AC099568.2	ENSG00000272931.1	0	1
lincRNA	AL391497.1	ENSG00000272672.1	0	0
lincRNA	AC092783.1	ENSG00000229201.1	0	0
lincRNA	AC091614.2	ENSG00000285532.1	0	0
lincRNA	LINC01763	ENSG00000225446.2	0	0
lincRNA	AC097059.1	ENSG00000226835.2	0	1
lincRNA	AC097059.2	ENSG00000236098.1	0	0
lincRNA	AC093117.1	ENSG00000223675.1	0	0
lincRNA	LINC01760	ENSG00000228504.1	0	0
lincRNA	LINC01650	ENSG00000230434.2	0	0
lincRNA	AL445218.1	ENSG00000237954.7	0	0
lincRNA	LINC01761	ENSG00000233907.1	0	0
lincRNA	AL356479.1	ENSG00000228971.2	0	0
lincRNA	AL683887.1	ENSG00000271252.1	0	0
lincRNA	AC099566.1	ENSG00000237435.9	0	1
lincRNA	LINC01787	ENSG00000231987.1	0	0
lincRNA	MIR137HG	ENSG00000225206.9	0	3
lincRNA	AC104453.1	ENSG00000285922.1	0	0
lincRNA	LINC01708	ENSG00000224445.3	0	0

lincRNA	AC104457.2	ENSG00000223906.1	0	0
lincRNA	LINC01349	ENSG00000230402.2	0	0
lincRNA	AC099670.3	ENSG00000285525.1	0	0
lincRNA	LINC01307	ENSG00000231671.1	0	0
lincRNA	LINC01709	ENSG00000226715.3	0	0
lincRNA	AC114485.1	ENSG00000233359.1	0	0
lincRNA	AC099567.1	ENSG00000230864.1	0	0
lincRNA	AC095032.2	ENSG00000230759.1	0	0
lincRNA	AL591888.1	ENSG00000285981.1	0	0
lincRNA	LINC01676	ENSG00000230768.8	0	0
lincRNA	LINC01677	ENSG00000233047.6	0	0
lincRNA	AL355306.2	ENSG00000237480.2	0	0
lincRNA	LINC01661	ENSG00000237460.2	0	0
lincRNA	AL390036.1	ENSG00000226822.2	0	0
lincRNA	AL359258.1	ENSG00000238122.1	0	0
lincRNA	AL390038.1	ENSG00000224698.1	0	0
lincRNA	AL356488.2	ENSG00000270066.3	0	0
lincRNA	LINC01768	ENSG00000228420.1	0	0
lincRNA	AL450468.2	ENSG00000261055.1	0	2
lincRNA	AL358215.2	ENSG00000282887.2	0	0
lincRNA	CYMP-AS1	ENSG00000235407.1	0	0
lincRNA	AL365361.1	ENSG00000259834.1	0	0
lincRNA	LINC01160	ENSG00000231346.5	0	0
lincRNA	AL445426.1	ENSG00000231246.1	0	0
lincRNA	LINC01356	ENSG00000215866.7	0	0
lincRNA	AL121999.1	ENSG00000232895.2	0	0
lincRNA	AL049825.1	ENSG00000285698.1	0	0
lincRNA	LINC01649	ENSG00000228127.2	0	0
lincRNA	LINC01779	ENSG00000235933.1	0	0
lincRNA	LINC01762	ENSG00000233154.5	0	0
lincRNA	AL136376.1	ENSG00000230381.2	0	0
lincRNA	AL390066.1	ENSG00000224950.2	0	0
lincRNA	C1orf137	ENSG00000203864.3	0	0
lincRNA	LINC01525	ENSG00000235202.1	0	0
lincRNA	AL139420.1	ENSG00000226172.2	0	0
lincRNA	AL139420.2	ENSG00000239216.1	0	0
lincRNA	AL359915.1	ENSG00000227712.1	0	0
lincRNA	LINC01780	ENSG00000232650.5	0	0
lincRNA	AC245008.1	ENSG00000273406.1	0	0
lincRNA	AC244453.1	ENSG00000227193.2	0	0
lincRNA	AC244021.1	ENSG00000227082.2	0	0
lincRNA	AL592494.1	ENSG00000228826.2	0	0
lincRNA	LINC01691	ENSG00000224857.1	0	0
lincRNA	AC239859.3	ENSG00000235887.2	0	0

lincRNA	AC239800.2	ENSG00000232721.2	0	0
lincRNA	LINC02591	ENSG00000263590.2	0	0
lincRNA	AC239798.2	ENSG00000234571.1	0	0
lincRNA	AC245595.1	ENSG00000232527.7	0	0
lincRNA	AC246785.3	ENSG00000237343.1	0	0
lincRNA	LINC01632	ENSG00000277199.1	0	0
lincRNA	FP700111.1	ENSG00000224363.2	0	0
lincRNA	AC245014.3	ENSG00000276216.1	0	0
lincRNA	AC245014.1	ENSG00000236140.1	0	0
lincRNA	LINC01719	ENSG00000233396.7	0	0
lincRNA	AC244394.2	ENSG00000234225.2	0	0
lincRNA	AC241644.1	ENSG00000227139.1	0	0
lincRNA	AC241644.2	ENSG00000234190.1	0	0
lincRNA	AC239803.2	ENSG00000273059.1	0	0
lincRNA	AC239803.3	ENSG00000274372.4	0	0
lincRNA	AC239809.3	ENSG00000227733.9	0	0
lincRNA	AC239803.1	ENSG00000232265.7	0	0
lincRNA	LINC01731	ENSG00000234283.1	0	0
lincRNA	AC245100.6	ENSG00000238107.1	0	0
lincRNA	AC245100.1	ENSG00000224481.2	0	0
lincRNA	AC239804.1	ENSG00000254539.1	0	0
lincRNA	AC245297.3	ENSG00000274265.4	0	0
lincRNA	AC245297.2	ENSG00000272755.1	0	3
lincRNA	AC242988.2	ENSG00000285554.1	0	0
lincRNA	FALEC	ENSG00000228126.1	0	1
lincRNA	AL589765.6	ENSG00000269489.1	0	0
lincRNA	AL161636.1	ENSG00000229699.1	0	0
lincRNA	AL451085.1	ENSG00000270361.1	0	0
lincRNA	AL139130.1	ENSG00000237390.1	0	0
lincRNA	AL365181.1	ENSG00000260460.1	0	0
lincRNA	AL365181.4	ENSG00000272971.1	0	0
lincRNA	AL365181.2	ENSG00000272068.1	0	0
lincRNA	AL590666.3	ENSG00000237588.2	0	0
lincRNA	AL138900.1	ENSG00000228239.1	0	0
lincRNA	AL138900.3	ENSG00000271736.1	0	0
lincRNA	AL138900.2	ENSG00000237189.1	0	0
lincRNA	AL138899.1	ENSG00000176320.2	0	0
lincRNA	AL663023.1	ENSG00000224943.1	0	0
lincRNA	SNHG28	ENSG00000188004.10	0	0
lincRNA	AL354714.3	ENSG00000232188.1	0	0
lincRNA	AL592295.4	ENSG00000283696.1	0	0
lincRNA	AL592295.3	ENSG00000283360.1	0	0
lincRNA	AL831711.1	ENSG00000283317.1	0	0
lincRNA	AL590408.1	ENSG00000227818.1	0	0

lincRNA	AL592435.1	ENSG00000237756.1	0	0
lincRNA	AL356441.1	ENSG00000236206.1	0	0
lincRNA	AL390115.1	ENSG00000229588.1	0	0
lincRNA	AL583804.1	ENSG00000225325.1	0	0
lincRNA	LINC01675	ENSG00000234142.1	0	0
lincRNA	AL359962.2	ENSG00000273160.1	0	0
lincRNA	AL022100.2	ENSG00000283255.1	0	0
lincRNA	AL031275.1	ENSG00000235736.1	0	0
lincRNA	LINC00970	ENSG00000203601.3	0	0
lincRNA	AL021940.1	ENSG00000230704.1	0	0
lincRNA	LINC01681	ENSG00000233985.1	0	0
lincRNA	LINC01142	ENSG00000224286.5	0	0
lincRNA	AL162399.1	ENSG00000225545.1	0	0
lincRNA	AL031599.1	ENSG00000224228.2	0	0
lincRNA	Z97198.1	ENSG00000224000.1	0	0
lincRNA	AL022310.1	ENSG00000226375.1	0	0
lincRNA	AL121983.1	ENSG00000224977.1	0	0
lincRNA	AL008626.1	ENSG00000230687.1	0	0
lincRNA	LINC01657	ENSG00000224718.1	0	0
lincRNA	LINC01645	ENSG00000224968.1	0	0
lincRNA	AL122019.1	ENSG00000227579.5	0	0
lincRNA	LINC01741	ENSG00000236720.1	0	0
lincRNA	C1orf220	ENSG00000213057.5	0	3
lincRNA	AL137796.1	ENSG00000273384.1	0	0
lincRNA	AL359853.3	ENSG00000243062.5	0	0
lincRNA	AL359853.2	ENSG00000231966.1	0	0
lincRNA	AL353708.2	ENSG00000261831.2	0	0
lincRNA	AL390718.1	ENSG00000261817.1	0	0
lincRNA	OVAAL	ENSG00000236719.3	0	0
lincRNA	AL162431.1	ENSG00000225857.5	0	0
lincRNA	LINC01732	ENSG00000237292.1	0	0
lincRNA	AL358393.1	ENSG00000225359.1	0	0
lincRNA	LINC01699	ENSG00000179452.2	0	0
lincRNA	AL161734.1	ENSG00000272880.1	0	0
lincRNA	AL355482.1	ENSG00000224810.1	0	0
lincRNA	AL355482.2	ENSG00000225982.1	0	0
lincRNA	AL390856.1	ENSG00000232309.2	0	0
lincRNA	LINC01344	ENSG00000228918.3	0	0
lincRNA	LINC00272	ENSG00000203729.8	0	0
lincRNA	LINC01688	ENSG00000227181.1	0	0
lincRNA	AL445228.1	ENSG00000231791.1	0	0
lincRNA	AL713852.1	ENSG00000285847.1	0	0
lincRNA	LINC01350	ENSG00000228309.6	0	0
lincRNA	AL133383.1	ENSG00000261729.1	0	0

lincRNA	AL096803.3	ENSG00000273198.1	0	0
lincRNA	PACERR	ENSG00000273129.1	0	1
lincRNA	ERVMER61-1	ENSG00000230426.3	0	0
lincRNA	AL136372.2	ENSG00000285894.1	0	0
lincRNA	AL596211.1	ENSG00000261182.1	0	0
lincRNA	AL929288.1	ENSG00000225006.1	0	0
lincRNA	AL691515.1	ENSG00000237283.1	0	0
lincRNA	AL691515.2	ENSG00000238054.2	0	0
lincRNA	LINC01035	ENSG00000226486.1	0	0
lincRNA	LINC01701	ENSG00000232212.1	0	0
lincRNA	AL591504.1	ENSG00000238270.1	0	0
lincRNA	LINC01351	ENSG00000237457.6	0	0
lincRNA	AL391645.1	ENSG00000241505.1	0	0
lincRNA	LINC01720	ENSG00000231175.1	0	0
lincRNA	AL138927.1	ENSG00000285638.1	0	0
lincRNA	AL713866.1	ENSG00000261642.1	0	0
lincRNA	LINC01680	ENSG00000233882.2	0	0
lincRNA	AL359081.1	ENSG00000223344.1	0	0
lincRNA	AL954650.1	ENSG00000228215.2	0	0
lincRNA	AL136987.1	ENSG00000236069.2	0	0
lincRNA	LINC01031	ENSG00000232077.1	0	0
lincRNA	AL357793.1	ENSG00000226640.2	0	0
lincRNA	AL357793.2	ENSG00000237011.1	0	0
lincRNA	AL356272.1	ENSG00000227240.1	0	0
lincRNA	AL513348.1	ENSG00000231714.1	0	0
lincRNA	AL353072.2	ENSG00000285718.1	0	0
lincRNA	LINC01724	ENSG00000227421.1	0	0
lincRNA	AL157402.1	ENSG00000223881.1	0	0
lincRNA	AC096631.1	ENSG00000225172.5	0	0
lincRNA	LINC01222	ENSG00000233410.1	0	0
lincRNA	LINC01221	ENSG00000235492.1	0	0
lincRNA	AC099673.1	ENSG00000231718.1	0	0
lincRNA	LINC00862	ENSG00000203721.6	0	1
lincRNA	AC097065.2	ENSG00000273093.1	0	0
lincRNA	AL359834.1	ENSG00000282849.1	0	0
lincRNA	AL445483.1	ENSG00000260088.1	0	0
lincRNA	AC104463.1	ENSG00000225620.1	0	0
lincRNA	LINC01353	ENSG00000231507.1	0	0
lincRNA	LINC01136	ENSG00000233791.5	0	0
lincRNA	LINC00303	ENSG00000176754.12	0	0
lincRNA	LINC00628	ENSG00000280924.1	0	0
lincRNA	AL512306.2	ENSG00000240219.1	0	0
lincRNA	AL161793.1	ENSG00000228153.1	0	0
lincRNA	AC098936.1	ENSG00000224717.1	0	0

lincRNA	AC244035.2	ENSG00000236889.1	0	0
lincRNA	AC244034.2	ENSG00000261000.1	0	0
lincRNA	AL445493.2	ENSG00000243636.1	0	0
lincRNA	AL596218.1	ENSG00000237074.2	0	0
lincRNA	AL391597.1	ENSG00000283044.1	0	0
lincRNA	AL356275.1	ENSG00000284237.1	0	0
lincRNA	LINC01735	ENSG00000261453.1	0	0
lincRNA	AC099793.1	ENSG00000259815.1	0	0
lincRNA	LINC01717	ENSG00000232812.1	0	0
lincRNA	LINC01774	ENSG00000236950.1	0	0
lincRNA	AC092810.3	ENSG00000232537.1	0	0
lincRNA	LINC01696	ENSG00000227940.1	0	0
lincRNA	AC105275.1	ENSG00000223649.1	0	0
lincRNA	AL356310.1	ENSG00000228792.2	0	0
lincRNA	AC096637.1	ENSG00000226868.1	0	0
lincRNA	AL606468.1	ENSG00000229983.1	0	0
lincRNA	AC092803.1	ENSG00000224535.1	0	0
lincRNA	LINC01740	ENSG00000228067.1	0	0
lincRNA	AC092803.2	ENSG00000260805.2	0	0
lincRNA	AL590648.3	ENSG00000237980.1	0	0
lincRNA	AC104333.4	ENSG00000282718.1	0	0
lincRNA	AL592402.1	ENSG00000225233.1	0	0
lincRNA	AC096639.1	ENSG00000228255.1	0	0
lincRNA	LINC00538	ENSG00000281664.1	0	0
lincRNA	AL592287.1	ENSG00000283518.1	0	0
lincRNA	AL450990.1	ENSG00000282265.1	0	0
lincRNA	LINC00210	ENSG00000231814.2	0	0
lincRNA	AL355526.1	ENSG00000230714.1	0	0
lincRNA	LINC01653	ENSG00000232100.1	0	0
lincRNA	C1orf143	ENSG00000228208.6	0	0
lincRNA	LINC01710	ENSG00000225561.1	0	0
lincRNA	AL360093.1	ENSG00000223842.1	0	0
lincRNA	AL356364.1	ENSG00000238232.1	0	0
lincRNA	AL451081.1	ENSG00000226927.1	0	0
lincRNA	AC096644.4	ENSG00000260505.1	0	0
lincRNA	AC096644.1	ENSG00000231529.1	0	0
lincRNA	RNU6ATAC3	ENSG00000221571.3	0	1
lincRNA	LINC01352	ENSG00000238078.1	0	0
lincRNA	C1orf140	ENSG00000234754.1	0	0
lincRNA	AL360013.2	ENSG00000232436.1	0	0
lincRNA	LINC02257	ENSG00000238042.5	0	3
lincRNA	LINC02474	ENSG00000228437.5	0	0
lincRNA	AL356108.1	ENSG00000236230.1	0	0
lincRNA	AL513314.1	ENSG00000226643.2	0	0

lincRNA	AL513314.2	ENSG00000276997.4	0	0
lincRNA	AC093152.1	ENSG00000278610.1	0	0
lincRNA	AL359979.2	ENSG00000236846.1	0	0
lincRNA	AL359979.1	ENSG00000226601.1	0	0
lincRNA	AC138393.3	ENSG00000278467.1	0	1
lincRNA	AC092811.2	ENSG00000282418.1	0	0
lincRNA	LINC01641	ENSG00000234277.2	0	0
lincRNA	AL136379.1	ENSG00000276255.2	0	0
lincRNA	IBA57-DT	ENSG00000203684.5	0	0
lincRNA	AL670729.3	ENSG00000270104.1	0	0
lincRNA	AL137793.2	ENSG00000282111.1	0	0
lincRNA	AL137793.1	ENSG00000236975.1	0	0
lincRNA	TMEM78	ENSG00000177800.2	0	0
lincRNA	LINC01682	ENSG00000227043.1	0	0
lincRNA	LINC01736	ENSG00000228058.1	0	0
lincRNA	AL133516.1	ENSG00000282564.1	0	0
lincRNA	LINC01737	ENSG00000232964.2	0	0
lincRNA	AL118511.2	ENSG00000225656.1	0	0
lincRNA	AL109810.2	ENSG00000235710.4	0	0
lincRNA	AL353052.1	ENSG00000235152.1	0	0
lincRNA	AL353052.2	ENSG00000236372.1	0	0
lincRNA	LINC01745	ENSG00000225919.1	0	0
lincRNA	LINC01744	ENSG00000236497.1	0	0
lincRNA	LINC01354	ENSG00000231768.1	0	0
lincRNA	AL161640.1	ENSG00000230404.1	0	0
lincRNA	LINC00184	ENSG00000224939.1	0	2
lincRNA	AL160408.5	ENSG00000272362.1	0	0
lincRNA	AL160408.3	ENSG00000230628.1	0	0
lincRNA	AL160408.6	ENSG00000282097.1	0	0
lincRNA	LINC01132	ENSG00000227630.3	0	0
lincRNA	AL360294.1	ENSG00000279261.2	0	0
lincRNA	AL391832.1	ENSG00000237520.1	0	1
lincRNA	AL391832.3	ENSG00000258082.1	0	0
lincRNA	LINC01348	ENSG00000280587.1	0	0
lincRNA	AL139161.2	ENSG00000237845.1	0	0
lincRNA	AL139161.1	ENSG00000229291.1	0	0
lincRNA	AL356010.2	ENSG00000234464.1	0	0
lincRNA	LINC01139	ENSG00000215808.3	0	0
lincRNA	AL359551.1	ENSG00000231877.1	0	0
lincRNA	AL356361.2	ENSG00000231979.1	0	0
lincRNA	AL606534.3	ENSG00000234116.1	0	0
lincRNA	AL590483.1	ENSG00000229960.1	0	0
lincRNA	AL590483.2	ENSG00000237759.1	0	0
lincRNA	AL451007.2	ENSG00000282317.1	0	1

lincRNA	LINC01743	ENSG00000242042.1	0	0
lincRNA	AL591848.3	ENSG00000260698.1	0	0
lincRNA	AL591848.2	ENSG00000235021.1	0	0
lincRNA	C1orf229	ENSG00000221953.5	0	0
lincRNA	AL390728.5	ENSG00000232347.1	0	0
lincRNA	AC138089.1	ENSG00000229255.1	0	0
lincRNA	AC079779.3	ENSG00000235779.7	0	0
lincRNA	AC079779.4	ENSG00000277997.1	0	0
lincRNA	LINC01865	ENSG00000233684.2	0	0
lincRNA	AC105393.2	ENSG00000236856.1	0	0
lincRNA	AC105393.1	ENSG00000226277.1	0	0
lincRNA	LINC01874	ENSG00000223985.1	0	0
lincRNA	AC093326.2	ENSG00000284452.1	0	0
lincRNA	LINC01875	ENSG00000225942.1	0	0
lincRNA	AC093326.1	ENSG00000233633.1	0	0
lincRNA	AC092159.1	ENSG00000227713.1	0	0
lincRNA	LINC01115	ENSG00000272342.1	0	0
lincRNA	AC116609.2	ENSG00000231173.1	0	0
lincRNA	AC116609.1	ENSG00000223751.1	0	0
lincRNA	LINC01115	ENSG00000237667.6	0	0
lincRNA	LINC01939	ENSG00000228799.5	0	0
lincRNA	AC105450.1	ENSG00000231482.3	0	0
lincRNA	AC141930.2	ENSG00000283766.1	0	0
lincRNA	AC144450.1	ENSG00000203635.2	0	0
lincRNA	AC018685.2	ENSG00000234929.1	0	0
lincRNA	AC018685.1	ENSG00000227364.1	0	0
lincRNA	AC011995.2	ENSG00000237720.1	0	0
lincRNA	AC011995.1	ENSG00000228391.1	0	0
lincRNA	LINC01250	ENSG00000234423.1	0	0
lincRNA	AC019118.2	ENSG00000236760.1	0	0
lincRNA	AC019118.1	ENSG00000226649.1	0	0
lincRNA	LINC01304	ENSG00000237401.6	0	2
lincRNA	AC012445.1	ENSG00000229550.1	0	0
lincRNA	AC107057.1	ENSG00000232835.1	0	0
lincRNA	AC010729.1	ENSG00000236106.1	0	0
lincRNA	LINC01810	ENSG00000237266.1	0	0
lincRNA	AC017053.1	ENSG00000234275.2	0	0
lincRNA	LINC01247	ENSG00000227007.1	0	0
lincRNA	LINC01824	ENSG00000226488.1	0	0
lincRNA	MIR7515HG	ENSG00000236172.6	0	0
lincRNA	LINC00487	ENSG00000205837.7	0	0
lincRNA	AC017076.1	ENSG00000271947.1	0	0
lincRNA	AC092580.2	ENSG00000230515.1	0	0
lincRNA	LINC01871	ENSG00000235576.1	0	0

lincRNA	AC007463.1	ENSG00000226506.5	0	0
lincRNA	LINC00298	ENSG00000235665.5	0	0
lincRNA	LINC00299	ENSG00000236790.5	0	0
lincRNA	U91324.1	ENSG00000229740.1	0	0
lincRNA	AC092617.1	ENSG00000285591.1	0	0
lincRNA	LINC01814	ENSG00000236008.2	0	0
lincRNA	AC011747.1	ENSG00000231083.1	0	0
lincRNA	AC093904.3	ENSG00000244310.1	0	0
lincRNA	AC093904.1	ENSG00000240980.1	0	0
lincRNA	AC073195.1	ENSG00000271855.1	0	1
lincRNA	AC082651.4	ENSG00000244260.1	0	0
lincRNA	AC082651.3	ENSG00000243491.1	0	0
lincRNA	AC104794.3	ENSG00000260476.1	0	0
lincRNA	AC104794.1	ENSG00000233502.1	0	0
lincRNA	C2orf48	ENSG00000163009.9	0	0
lincRNA	AC007249.2	ENSG00000257135.5	0	0
lincRNA	AC007249.1	ENSG00000217258.2	0	0
lincRNA	AC092687.2	ENSG00000236162.1	0	0
lincRNA	AC092687.1	ENSG00000232056.1	0	0
lincRNA	LINC01954	ENSG00000271952.1	0	0
lincRNA	FLJ33534	ENSG00000145063.14	0	0
lincRNA	AC099344.1	ENSG00000230952.1	0	0
lincRNA	AC099344.2	ENSG00000231403.1	0	0
lincRNA	LINC00570	ENSG00000224177.6	0	0
lincRNA	AC099344.3	ENSG00000285569.1	0	0
lincRNA	AC106875.2	ENSG00000276411.1	0	0
lincRNA	AC096559.1	ENSG00000223360.1	0	0
lincRNA	AC009486.1	ENSG00000261117.1	0	0
lincRNA	AC093912.1	ENSG00000285876.1	0	0
lincRNA	AC073062.1	ENSG00000229370.1	0	0
lincRNA	LINC00276	ENSG00000230448.5	0	0
lincRNA	AC016730.1	ENSG00000227718.1	0	0
lincRNA	AC011897.1	ENSG00000228505.5	0	0
lincRNA	AC068286.1	ENSG00000235127.1	0	0
lincRNA	LINC01804	ENSG00000231031.6	0	0
lincRNA	AC113608.2	ENSG00000237326.1	0	0
lincRNA	MYCNUT	ENSG00000223850.1	0	0
lincRNA	AC010145.1	ENSG00000226764.2	0	0
lincRNA	GACAT3	ENSG00000236289.2	0	0
lincRNA	AC130710.1	ENSG00000236989.5	0	0
lincRNA	AC010745.1	ENSG00000226041.1	0	0
lincRNA	AC010745.2	ENSG00000228876.3	0	0
lincRNA	AC010745.3	ENSG00000231266.1	0	0
lincRNA	AC010745.5	ENSG00000285354.1	0	0

lincRNA	AC010745.4	ENSG00000232444.1	0	0
lincRNA	AC010880.1	ENSG00000224400.5	0	0
lincRNA	AC008164.1	ENSG00000223536.5	0	0
lincRNA	LINC01866	ENSG00000237670.1	0	0
lincRNA	AC079148.1	ENSG00000260331.1	0	0
lincRNA	AC106053.1	ENSG00000224626.1	0	0
lincRNA	LINC01376	ENSG00000236204.6	0	0
lincRNA	AC010096.1	ENSG00000234597.1	0	0
lincRNA	LINC01808	ENSG00000237992.2	0	0
lincRNA	AC019055.1	ENSG00000235911.1	0	0
lincRNA	LINC00954	ENSG00000228784.8	0	0
lincRNA	AC098828.2	ENSG00000223734.2	0	0
lincRNA	AC098828.3	ENSG00000234378.1	0	0
lincRNA	AC023137.1	ENSG00000228950.1	0	0
lincRNA	AC012065.1	ENSG00000227047.1	0	0
lincRNA	AC012065.3	ENSG00000269976.1	0	1
lincRNA	AC012361.1	ENSG00000236436.1	0	0
lincRNA	AC115619.1	ENSG00000261012.2	0	0
lincRNA	AC018742.1	ENSG00000233005.1	0	0
lincRNA	AC011752.1	ENSG00000231204.6	0	0
lincRNA	AC009411.2	ENSG00000235537.2	0	0
lincRNA	AC009411.1	ENSG00000228538.6	0	0
lincRNA	LINC01822	ENSG00000229621.1	0	0
lincRNA	AC096570.1	ENSG00000228999.3	0	0
lincRNA	AC096570.2	ENSG00000234207.1	0	0
lincRNA	LINC01884	ENSG00000233587.1	0	0
lincRNA	AC018467.1	ENSG00000232451.1	0	0
lincRNA	AC012506.1	ENSG00000223530.1	0	0
lincRNA	AC012506.3	ENSG00000233714.1	0	0
lincRNA	AC012506.2	ENSG00000223634.1	0	0
lincRNA	AC012506.4	ENSG00000235497.5	0	0
lincRNA	AC009242.1	ENSG00000283031.1	0	0
lincRNA	AC012074.1	ENSG00000235072.2	0	1
lincRNA	LINC01460	ENSG00000205334.2	0	0
lincRNA	AC104695.1	ENSG00000227938.1	0	0
lincRNA	AC097724.1	ENSG00000273233.1	0	0
lincRNA	LINC01936	ENSG00000235997.2	0	0
lincRNA	AC009301.1	ENSG00000285984.1	0	1
lincRNA	LINC01946	ENSG00000223647.1	0	0
lincRNA	AL121652.1	ENSG00000273165.1	0	0
lincRNA	AL121658.1	ENSG00000272716.1	0	2
lincRNA	LINC00486	ENSG00000230876.7	0	0
lincRNA	AL121656.1	ENSG00000236854.1	0	0
lincRNA	LINC01320	ENSG00000228262.9	0	0

lincRNA	LINC01318	ENSG00000237790.1	0	0
lincRNA	AC073218.2	ENSG00000272027.2	0	0
lincRNA	AC073218.1	ENSG00000232153.2	0	0
lincRNA	AC012593.1	ENSG00000226994.7	0	0
lincRNA	AC007391.1	ENSG00000232028.1	0	0
lincRNA	AC006369.2	ENSG00000236572.1	0	0
lincRNA	LINC00211	ENSG00000237803.5	0	0
lincRNA	AC009229.2	ENSG00000229160.1	0	0
lincRNA	AC009229.1	ENSG00000227292.1	0	0
lincRNA	AC011247.1	ENSG00000231367.5	0	0
lincRNA	LINC01883	ENSG00000235009.1	0	0
lincRNA	AC007317.1	ENSG00000285898.1	0	0
lincRNA	LINC01794	ENSG00000233128.1	0	0
lincRNA	LINC01913	ENSG00000214691.8	0	0
lincRNA	LINC01914	ENSG00000234362.5	0	1
lincRNA	AC016735.1	ENSG00000224739.2	0	0
lincRNA	LINC01819	ENSG00000231826.5	0	0
lincRNA	LINC02590	ENSG00000233978.1	0	0
lincRNA	LINC02580	ENSG00000230587.1	0	0
lincRNA	LINC01126	ENSG00000279873.2	0	0
lincRNA	LINC01833	ENSG00000259439.2	0	1
lincRNA	SIX3-AS1	ENSG00000236502.1	0	1
lincRNA	AC012354.1	ENSG00000225156.2	0	0
lincRNA	AC093702.1	ENSG00000231156.1	0	5
lincRNA	AC093833.1	ENSG00000231054.1	0	1
lincRNA	LINC01820	ENSG00000228100.1	0	1
lincRNA	LINC02583	ENSG00000187600.14	0	0
lincRNA	AC016722.1	ENSG00000226548.1	0	0
lincRNA	AC073283.2	ENSG00000226087.1	0	0
lincRNA	AC092650.1	ENSG00000230773.7	0	0
lincRNA	AC093635.1	ENSG00000272663.1	0	0
lincRNA	AC009975.1	ENSG00000282890.1	0	0
lincRNA	AC009975.2	ENSG00000282998.1	0	0
lincRNA	AC009971.1	ENSG00000282828.1	0	0
lincRNA	AC007402.1	ENSG00000231918.1	0	0
lincRNA	LINC01867	ENSG00000225444.1	0	0
lincRNA	AC139712.3	ENSG00000236837.1	0	0
lincRNA	AC010967.1	ENSG00000228033.5	0	0
lincRNA	AC010967.2	ENSG00000232604.1	0	0
lincRNA	AC011306.1	ENSG00000272180.1	0	0
lincRNA	MIR217HG	ENSG00000226702.1	0	0
lincRNA	LINC01813	ENSG00000229805.1	0	0
lincRNA	AC132153.1	ENSG00000285755.1	0	0
lincRNA	LINC01795	ENSG00000225226.1	0	1

lincRNA	LINC01122	ENSG00000233723.8	0	0
lincRNA	AC007092.1	ENSG00000285673.1	0	0
lincRNA	LINC01793	ENSG00000222030.1	0	0
lincRNA	AC007100.1	ENSG00000271955.2	0	0
lincRNA	AC007179.2	ENSG00000233891.7	0	0
lincRNA	AC007179.1	ENSG00000231815.2	0	0
lincRNA	AC007132.1	ENSG00000285611.1	0	0
lincRNA	MIR4432HG	ENSG00000228590.2	0	0
lincRNA	LINC01185	ENSG00000228414.6	0	0
lincRNA	AC016747.2	ENSG00000271889.1	0	0
lincRNA	AC093159.1	ENSG00000228541.1	0	0
lincRNA	AC012368.2	ENSG00000228079.2	0	0
lincRNA	LINC00309	ENSG00000230923.2	0	0
lincRNA	AC114752.1	ENSG00000238012.1	0	0
lincRNA	AC114752.2	ENSG00000238201.1	0	0
lincRNA	LINC01805	ENSG00000223863.1	0	0
lincRNA	LINC02579	ENSG00000233694.5	0	0
lincRNA	AC007365.1	ENSG00000226756.1	0	0
lincRNA	LINC01800	ENSG00000234572.1	0	0
lincRNA	LINC02245	ENSG00000237638.1	0	0
lincRNA	AC012370.2	ENSG00000234255.8	0	0
lincRNA	AC007389.3	ENSG00000235725.1	0	0
lincRNA	AC118345.1	ENSG00000227293.1	0	0
lincRNA	AC092669.1	ENSG00000225815.2	0	0
lincRNA	LINC01797	ENSG00000237179.5	0	0
lincRNA	LINC01799	ENSG00000230525.5	0	0
lincRNA	LINC01628	ENSG00000232688.1	0	0
lincRNA	AC007403.1	ENSG00000223859.1	0	0
lincRNA	LINC01828	ENSG00000235885.7	0	0
lincRNA	LINC01829	ENSG00000236780.5	0	7
lincRNA	AC023115.1	ENSG00000236605.1	0	0
lincRNA	AC007422.1	ENSG00000224173.1	0	0
lincRNA	AC007422.2	ENSG00000235495.1	0	0
lincRNA	LINC01812	ENSG00000237013.1	0	0
lincRNA	LINC01890	ENSG00000228329.1	0	0
lincRNA	AC092431.1	ENSG00000231024.1	0	0
lincRNA	AC016700.3	ENSG00000233060.1	0	0
lincRNA	LINC01143	ENSG00000237751.2	0	0
lincRNA	AC073046.1	ENSG00000235499.1	0	0
lincRNA	AC073263.1	ENSG00000217702.2	0	0
lincRNA	AC073263.2	ENSG00000279070.2	0	0
lincRNA	AC104135.1	ENSG00000236209.1	0	0
lincRNA	AC005034.4	ENSG00000270996.1	0	0
lincRNA	AC084149.1	ENSG00000227088.1	0	0

lincRNA	AC084149.2	ENSG00000230968.1	0	0
lincRNA	LINC01851	ENSG00000233605.2	0	0
lincRNA	AC012494.1	ENSG00000229494.1	0	0
lincRNA	AC092660.1	ENSG00000234877.2	0	0
lincRNA	AC012355.1	ENSG00000230975.1	0	0
lincRNA	LINC01815	ENSG00000235519.2	0	0
lincRNA	AC079896.1	ENSG00000231781.1	0	0
lincRNA	AC010105.1	ENSG00000237498.2	0	0
lincRNA	LINC01809	ENSG00000232548.1	0	0
lincRNA	AC106874.1	ENSG00000228272.1	0	0
lincRNA	LINC01964	ENSG00000260840.1	0	0
lincRNA	ST3GAL5-	ENSG00000232504.4	0	0
lincRNA	AC012511.1	ENSG00000272564.1	0	0
lincRNA	LINC01955	ENSG00000281477.1	0	0
lincRNA	AC233263.6	ENSG00000281904.1	0	0
lincRNA	AC009237.15	ENSG00000273305.1	0	1
lincRNA	AC008268.1	ENSG00000235584.2	0	0
lincRNA	AC012307.1	ENSG00000228873.1	0	0
lincRNA	CNNM3-DT	ENSG00000273265.1	0	0
lincRNA	AC159540.2	ENSG00000277701.4	0	0
lincRNA	AC159540.1	ENSG00000237837.1	0	0
lincRNA	AC092683.1	ENSG00000230606.10	0	0
lincRNA	AC092683.2	ENSG00000278766.2	0	0
lincRNA	AC109826.1	ENSG00000226791.6	0	0
lincRNA	LINC01104	ENSG00000232084.5	0	0
lincRNA	LINC01849	ENSG00000234988.1	0	0
lincRNA	LINC01868	ENSG00000234169.1	0	0
lincRNA	AC092168.1	ENSG00000228488.1	0	0
lincRNA	LINC01870	ENSG00000223826.1	0	0
lincRNA	LINC01127	ENSG00000281162.2	0	0
lincRNA	LINC01796	ENSG00000277527.1	0	0
lincRNA	LINC01935	ENSG00000224095.1	0	0
lincRNA	AC108051.1	ENSG00000227680.1	0	0
lincRNA	AC073987.1	ENSG00000229209.1	0	0
lincRNA	AC013727.1	ENSG00000232597.5	0	0
lincRNA	AC013727.2	ENSG00000236141.1	0	0
lincRNA	LINC01965	ENSG00000256637.6	0	0
lincRNA	AC096554.1	ENSG00000228968.1	0	1
lincRNA	AC068535.1	ENSG00000227157.1	0	0
lincRNA	LINC01831	ENSG00000225765.1	0	0
lincRNA	LINC01102	ENSG00000235597.1	0	0
lincRNA	LINC01103	ENSG00000234781.2	0	0
lincRNA	AC013402.2	ENSG00000231626.1	0	0
lincRNA	AC013402.3	ENSG00000236109.1	0	0

lincRNA	AC013402.1	ENSG00000230690.1	0	0
lincRNA	AC068057.1	ENSG00000228528.1	0	0
lincRNA	LINC01114	ENSG00000234177.5	0	0
lincRNA	LINC01918	ENSG00000226508.1	0	0
lincRNA	AC012360.2	ENSG00000272861.1	0	0
lincRNA	AC012360.1	ENSG00000235319.1	0	0
lincRNA	AC009505.1	ENSG00000234162.1	0	0
lincRNA	AC108868.1	ENSG00000231505.1	0	0
lincRNA	LINC01789	ENSG00000229457.1	0	0
lincRNA	AC096669.1	ENSG00000225588.2	0	0
lincRNA	LINC01885	ENSG00000237880.1	0	0
lincRNA	LINC01886	ENSG00000224568.1	0	0
lincRNA	GACAT1	ENSG00000232991.5	0	0
lincRNA	RGPD4-AS1	ENSG00000230651.7	0	0
lincRNA	LINC01593	ENSG00000231221.1	0	0
lincRNA	SH3RF3-AS1	ENSG00000259863.1	0	0
lincRNA	AC074387.1	ENSG00000282033.1	0	0
lincRNA	LINC01123	ENSG00000204588.5	0	0
lincRNA	LINC01106	ENSG00000175772.10	0	0
lincRNA	AC114776.1	ENSG00000235881.2	0	0
lincRNA	AC108463.2	ENSG00000230499.1	0	0
lincRNA	AC108463.3	ENSG00000271590.1	0	2
lincRNA	AC068491.2	ENSG00000229118.1	0	0
lincRNA	AC068491.3	ENSG00000270190.1	0	0
lincRNA	AC017002.5	ENSG00000280878.1	0	0
lincRNA	AC017002.1	ENSG00000224959.1	0	0
lincRNA	AC017002.2	ENSG00000225744.1	0	0
lincRNA	AC016745.2	ENSG00000272563.1	0	0
lincRNA	LINC01961	ENSG00000234148.2	0	0
lincRNA	PGM5P4-AS1	ENSG00000231943.7	0	0
lincRNA	FAM138B	ENSG00000226516.7	0	0
lincRNA	AC017074.2	ENSG00000233479.1	0	0
lincRNA	AC017074.1	ENSG00000227359.1	0	0
lincRNA	AC104653.2	ENSG00000244063.1	0	0
lincRNA	AC110769.1	ENSG00000243179.1	0	0
lincRNA	AC010982.1	ENSG00000235242.1	0	0
lincRNA	THORLNC	ENSG00000226856.6	0	0
lincRNA	AC012363.2	ENSG00000235840.1	0	0
lincRNA	AC073257.2	ENSG00000237614.1	0	0
lincRNA	AC073257.1	ENSG00000232140.1	0	0
lincRNA	AC018866.1	ENSG00000229774.1	0	0
lincRNA	AC017033.1	ENSG00000271709.1	0	0
lincRNA	AC067960.1	ENSG00000234455.1	0	0
lincRNA	AC079988.1	ENSG00000236145.1	0	0

lincRNA	LINC01823	ENSG00000224655.5	0	0
lincRNA	AC062020.1	ENSG00000237856.1	0	0
lincRNA	LINC01826	ENSG00000232740.1	0	0
lincRNA	LINC01889	ENSG00000235491.1	0	0
lincRNA	LINC01941	ENSG00000226813.2	0	0
lincRNA	AC023347.1	ENSG00000235774.1	0	0
lincRNA	AC012508.1	ENSG00000260163.1	0	0
lincRNA	AC012306.2	ENSG00000272667.1	0	0
lincRNA	AC012451.1	ENSG00000237532.1	0	0
lincRNA	AC068483.1	ENSG00000238277.1	0	0
lincRNA	LINC01854	ENSG00000204460.3	0	0
lincRNA	LINC02572	ENSG00000229536.2	0	0
lincRNA	LINC01856	ENSG00000237574.1	0	0
lincRNA	LINC01120	ENSG00000223631.1	0	0
lincRNA	AC073869.3	ENSG00000273073.1	0	0
lincRNA	LINC01087	ENSG00000224559.2	0	0
lincRNA	C2orf27B	ENSG00000186825.5	0	0
lincRNA	AC103564.1	ENSG00000229203.1	0	0
lincRNA	AJ239322.2	ENSG00000244337.2	0	0
lincRNA	AJ239322.1	ENSG00000235615.2	0	0
lincRNA	LINC01945	ENSG00000236485.1	0	0
lincRNA	AC097532.1	ENSG00000230803.1	0	0
lincRNA	AC097532.2	ENSG00000272769.1	0	0
lincRNA	AC011243.1	ENSG00000223430.1	0	0
lincRNA	AC068492.1	ENSG00000237262.1	0	0
lincRNA	LINC01832	ENSG00000224231.1	0	0
lincRNA	AC114763.2	ENSG00000230569.1	0	0
lincRNA	AC092620.3	ENSG00000260059.1	0	0
lincRNA	AC092620.1	ENSG00000237772.1	0	0
lincRNA	AC062021.1	ENSG00000226939.1	0	0
lincRNA	AC016710.1	ENSG00000229131.1	0	0
lincRNA	AC078851.1	ENSG00000223554.1	0	0
lincRNA	LINC01853	ENSG00000224028.1	0	0
lincRNA	AC079584.1	ENSG00000236356.1	0	0
lincRNA	LINC01412	ENSG00000232606.1	0	0
lincRNA	AC023128.1	ENSG00000234940.1	0	0
lincRNA	LINC01966	ENSG00000268580.1	0	0
lincRNA	AC096666.1	ENSG00000235435.1	0	0
lincRNA	AC092484.1	ENSG00000225107.1	0	0
lincRNA	AC079163.1	ENSG00000226218.1	0	0
lincRNA	AC062031.1	ENSG00000233842.1	0	0
lincRNA	LINC01911	ENSG00000231040.1	0	0
lincRNA	AC062032.1	ENSG00000227902.2	0	0
lincRNA	LINC01931	ENSG00000162947.4	0	0

lincRNA	LINC01818	ENSG00000230645.1	0	0
lincRNA	LINC01817	ENSG00000231420.1	0	0
lincRNA	LINC01920	ENSG00000236049.1	0	0
lincRNA	AC104777.3	ENSG00000237220.1	0	0
lincRNA	AC104777.2	ENSG00000232359.1	0	0
lincRNA	AC104777.1	ENSG00000224048.5	0	0
lincRNA	AC023469.1	ENSG00000222031.1	0	0
lincRNA	AC018731.1	ENSG00000236885.1	0	0
lincRNA	AC079150.1	ENSG00000224612.1	0	0
lincRNA	LINC01850	ENSG00000227708.1	0	0
lincRNA	AC009227.2	ENSG00000238004.1	0	0
lincRNA	AC092625.1	ENSG00000230991.1	0	0
lincRNA	AC074099.1	ENSG00000282440.1	0	0
lincRNA	LINC01958	ENSG00000283436.1	0	0
lincRNA	AC019186.1	ENSG00000234584.1	0	0
lincRNA	AC091488.1	ENSG00000271320.1	0	2
lincRNA	AC005042.2	ENSG00000228586.1	0	0
lincRNA	LINC02478	ENSG00000285373.1	0	1
lincRNA	AC008063.2	ENSG00000233397.2	0	0
lincRNA	AC016766.1	ENSG00000237844.1	0	0
lincRNA	AC009495.3	ENSG00000235192.1	0	0
lincRNA	AC009495.1	ENSG00000229195.1	0	0
lincRNA	AC009495.2	ENSG00000232411.1	0	0
lincRNA	AC073050.1	ENSG00000228222.1	0	0
lincRNA	AC007405.1	ENSG00000234350.4	0	0
lincRNA	DLX2-DT	ENSG00000236651.1	0	0
lincRNA	AC104088.1	ENSG00000232555.1	0	0
lincRNA	AC106900.1	ENSG00000224638.1	0	0
lincRNA	AC016737.2	ENSG00000273258.1	0	0
lincRNA	LINC01960	ENSG00000260868.1	0	0
lincRNA	AC093459.1	ENSG00000229066.1	0	0
lincRNA	AC016751.1	ENSG00000229779.1	0	0
lincRNA	AC017048.1	ENSG00000229434.1	0	0
lincRNA	AC017048.2	ENSG00000236231.1	0	0
lincRNA	AC092162.2	ENSG00000230552.5	0	0
lincRNA	AC073636.1	ENSG00000227098.1	0	0
lincRNA	AC074286.1	ENSG00000236501.5	0	0
lincRNA	AC010680.4	ENSG00000271141.1	0	0
lincRNA	AC010680.5	ENSG00000271401.1	0	0
lincRNA	AC093911.1	ENSG00000237477.1	0	0
lincRNA	AC009478.1	ENSG00000225258.1	0	0
lincRNA	SCHLAP1	ENSG00000281131.1	0	0
lincRNA	AC068196.1	ENSG00000238171.1	0	0
lincRNA	AC020595.1	ENSG00000226681.1	0	0

lincRNA	AC013733.1	ENSG00000225570.1	0	0
lincRNA	AC093639.1	ENSG00000234172.1	0	0
lincRNA	LINC01090	ENSG00000231689.2	0	0
lincRNA	AC092614.1	ENSG00000227542.1	0	0
lincRNA	AC062039.1	ENSG00000229395.1	0	0
lincRNA	AC068135.2	ENSG00000260142.1	0	0
lincRNA	LINC01821	ENSG00000225539.5	0	0
lincRNA	LINC01790	ENSG00000230173.1	0	0
lincRNA	AC010983.1	ENSG00000235056.1	0	0
lincRNA	AC104823.1	ENSG00000224099.1	0	0
lincRNA	AC064834.1	ENSG00000271893.1	0	0
lincRNA	LINC01825	ENSG00000223466.1	0	0
lincRNA	LINC01827	ENSG00000234919.1	0	0
lincRNA	AC020719.1	ENSG00000237035.1	0	0
lincRNA	LINC01923	ENSG00000236653.2	0	0
lincRNA	AC019330.1	ENSG00000225421.2	0	0
lincRNA	AC020718.1	ENSG00000231557.1	0	0
lincRNA	LINC01877	ENSG00000238217.5	0	0
lincRNA	AC007272.1	ENSG00000232719.1	0	0
lincRNA	AC069148.1	ENSG00000273209.1	0	0
lincRNA	AC064836.2	ENSG00000272966.1	0	0
lincRNA	AC009965.1	ENSG00000234714.1	0	0
lincRNA	AC009498.1	ENSG00000237271.1	0	0
lincRNA	AC016903.1	ENSG00000236634.1	0	0
lincRNA	AC016903.2	ENSG00000237843.1	0	0
lincRNA	AC008269.2	ENSG00000260171.1	0	0
lincRNA	AC008269.1	ENSG00000229321.1	0	0
lincRNA	AC007879.1	ENSG00000224342.1	0	0
lincRNA	AC007879.2	ENSG00000225916.1	0	0
lincRNA	AC007879.4	ENSG00000240440.1	0	0
lincRNA	LINC01802	ENSG00000225064.1	0	0
lincRNA	LINC01857	ENSG00000224137.1	0	0
lincRNA	LINC01878	ENSG00000234308.2	0	0
lincRNA	AC108066.1	ENSG00000223373.1	0	0
lincRNA	AC079610.2	ENSG00000270659.1	0	0
lincRNA	LINC01953	ENSG00000236193.3	0	0
lincRNA	AC079610.3	ENSG00000272519.1	0	0
lincRNA	AC079610.1	ENSG00000196096.3	0	0
lincRNA	AC073284.1	ENSG00000237571.1	0	0
lincRNA	AC012462.1	ENSG00000225166.1	0	1
lincRNA	AC012462.2	ENSG00000228618.1	0	0
lincRNA	AC012668.2	ENSG00000234938.2	0	0
lincRNA	AC012668.3	ENSG00000237525.6	0	0
lincRNA	AC012668.1	ENSG00000227981.2	0	0

lincRNA	LINC01614	ENSG00000230838.1	0	0
lincRNA	AC093382.1	ENSG00000226276.1	0	0
lincRNA	AC073321.1	ENSG00000225794.1	0	0
lincRNA	AC007563.1	ENSG00000229352.1	0	0
lincRNA	AC007557.1	ENSG00000227021.1	0	0
lincRNA	AC007557.3	ENSG00000236295.1	0	0
lincRNA	LINC01921	ENSG00000223874.1	0	0
lincRNA	AC007749.1	ENSG00000259855.1	0	0
lincRNA	DIRC3-AS1	ENSG00000233143.1	0	0
lincRNA	AC009955.2	ENSG00000267919.1	0	0
lincRNA	LINC01803	ENSG00000228909.1	0	0
lincRNA	AC019211.1	ENSG00000239498.1	0	0
lincRNA	AC093083.1	ENSG00000235337.1	0	0
lincRNA	AC093843.1	ENSG00000224819.1	0	0
lincRNA	AC068489.1	ENSG00000234446.2	0	0
lincRNA	AC010980.2	ENSG00000267034.1	0	0
lincRNA	AC013448.1	ENSG00000231189.1	0	0
lincRNA	AC013448.2	ENSG00000235610.1	0	0
lincRNA	AC062015.1	ENSG00000235070.3	0	0
lincRNA	AC010735.2	ENSG00000272622.1	0	0
lincRNA	LINC01807	ENSG00000232023.2	0	0
lincRNA	AC012070.1	ENSG00000228226.1	0	0
lincRNA	LINC01907	ENSG00000226125.1	0	0
lincRNA	SPATA3-AS1	ENSG00000238062.5	0	0
lincRNA	AC017104.3	ENSG00000233538.1	0	0
lincRNA	AC068134.2	ENSG00000237087.1	0	0
lincRNA	AC006037.1	ENSG00000226442.2	0	0
lincRNA	AC122134.1	ENSG00000261298.1	0	0
lincRNA	AC097713.1	ENSG00000228162.1	0	0
lincRNA	LINC01891	ENSG00000231682.1	0	0
lincRNA	LINC01173	ENSG00000280744.1	0	0
lincRNA	AC114814.1	ENSG00000226542.1	0	0
lincRNA	AC114814.3	ENSG00000235293.1	0	0
lincRNA	AC084030.1	ENSG00000232328.1	0	0
lincRNA	AC107079.1	ENSG00000224844.1	0	0
lincRNA	AC112715.1	ENSG00000224132.2	0	0
lincRNA	AC112721.1	ENSG00000222022.1	0	0
lincRNA	AC112721.2	ENSG00000222032.1	0	0
lincRNA	AC104667.1	ENSG00000227107.1	0	0
lincRNA	AC012485.2	ENSG00000232306.2	0	0
lincRNA	LINC01107	ENSG00000225493.1	0	0
lincRNA	LINC01937	ENSG00000234279.1	0	0
lincRNA	AC145625.1	ENSG00000226992.1	0	2
lincRNA	LINC01940	ENSG00000227744.4	0	0

lincRNA	AC079612.1	ENSG00000196758.3	0	0
lincRNA	AC079612.2	ENSG00000227459.1	0	0
lincRNA	AC093802.2	ENSG00000259889.1	0	0
lincRNA	AC093802.1	ENSG00000220256.3	0	0
lincRNA	AC124861.1	ENSG00000227479.1	0	0
lincRNA	AC011298.1	ENSG00000219159.4	0	0
lincRNA	AC131097.4	ENSG00000235151.1	0	0
lincRNA	AC093642.1	ENSG00000226423.1	0	0
lincRNA	LINC01880	ENSG00000232002.1	0	0
lincRNA	LINC01986	ENSG00000223587.1	0	0
lincRNA	AC066595.1	ENSG00000224918.1	0	0
lincRNA	LINC01266	ENSG00000224957.5	0	0
lincRNA	AC090044.1	ENSG00000224239.1	0	0
lincRNA	AC018814.1	ENSG00000225044.1	0	0
lincRNA	CNTN4-AS2	ENSG00000227588.2	0	0
lincRNA	AC034195.1	ENSG00000223727.6	0	0
lincRNA	EGOT	ENSG00000235947.1	0	0
lincRNA	AC026202.1	ENSG00000230944.1	0	0
lincRNA	AC087857.1	ENSG00000229642.1	0	0
lincRNA	AC026167.1	ENSG00000226022.1	0	0
lincRNA	GRM7-AS3	ENSG00000226258.6	0	0
lincRNA	AC018832.1	ENSG00000228351.2	0	0
lincRNA	AC023481.1	ENSG00000231401.1	0	0
lincRNA	AC034187.1	ENSG00000224884.1	0	0
lincRNA	SRGAP3-AS4	ENSG00000235830.1	0	0
lincRNA	LINC00606	ENSG00000226567.1	0	0
lincRNA	AC018495.1	ENSG00000230599.2	0	0
lincRNA	LINC02022	ENSG00000232746.1	0	0
lincRNA	AC093496.1	ENSG00000255021.1	0	0
lincRNA	LINC01267	ENSG00000251576.1	0	0
lincRNA	AC090952.2	ENSG00000283392.1	0	0
lincRNA	AC087591.1	ENSG00000285969.1	0	0
lincRNA	LINC02011	ENSG00000260902.1	0	0
lincRNA	AC090953.2	ENSG00000285654.1	0	0
lincRNA	AC010139.1	ENSG00000269391.1	0	0
lincRNA	LINC00690	ENSG00000233570.1	0	0
lincRNA	AC091493.1	ENSG00000229271.1	0	0
lincRNA	AC090644.1	ENSG00000285731.1	0	0
lincRNA	AC132807.1	ENSG00000226238.1	0	0
lincRNA	AC132807.2	ENSG00000274840.4	0	0
lincRNA	AC116096.1	ENSG00000261734.1	0	0
lincRNA	AC099753.1	ENSG00000282987.1	0	0
lincRNA	ZNF385D-	ENSG00000223351.5	0	0
lincRNA	AC012087.1	ENSG00000272554.1	0	0

lincRNA	AC099754.1	ENSG00000225386.1	0	0
lincRNA	LINC02084	ENSG00000272282.1	0	0
lincRNA	LINC01981	ENSG00000229243.1	0	0
lincRNA	LINC01985	ENSG00000227260.1	0	0
lincRNA	AC116035.1	ENSG00000227549.1	0	0
lincRNA	LINC01811	ENSG00000226320.5	0	0
lincRNA	AC123023.1	ENSG00000236452.1	0	0
lincRNA	AC018359.1	ENSG00000227498.2	0	0
lincRNA	LINC02033	ENSG00000279727.1	0	0
lincRNA	AC011816.2	ENSG00000272334.1	0	0
lincRNA	AC097359.3	ENSG00000271653.1	0	0
lincRNA	AC122683.1	ENSG00000226302.1	0	0
lincRNA	AC099560.1	ENSG00000231873.1	0	0
lincRNA	AC099541.1	ENSG00000233096.1	0	0
lincRNA	AC009743.1	ENSG00000233919.1	0	0
lincRNA	AC092042.1	ENSG00000227245.1	0	0
lincRNA	AC092042.4	ENSG00000285766.1	0	0
lincRNA	AC006055.1	ENSG00000235886.1	0	0
lincRNA	AC006058.2	ENSG00000271192.1	0	0
lincRNA	AC006058.3	ENSG00000272121.1	0	0
lincRNA	LINC01988	ENSG00000283036.2	0	0
lincRNA	AC141002.1	ENSG00000271973.1	0	0
lincRNA	BSN-DT	ENSG00000226913.1	0	0
lincRNA	AC096920.1	ENSG00000280382.1	0	0
lincRNA	AC115284.3	ENSG00000285685.1	0	0
lincRNA	AC092059.1	ENSG00000239991.1	0	0
lincRNA	LINC02017	ENSG00000243572.1	0	0
lincRNA	AC121764.2	ENSG00000242568.1	0	0
lincRNA	AC121764.3	ENSG00000242775.1	0	0
lincRNA	AC121764.1	ENSG00000242317.1	0	0
lincRNA	AC025572.1	ENSG00000240777.2	0	0
lincRNA	DENND6A-	ENSG00000241933.1	0	0
lincRNA	AC137936.1	ENSG00000283148.1	0	0
lincRNA	AC116036.2	ENSG00000272360.1	0	0
lincRNA	AC114400.1	ENSG00000243295.1	0	0
lincRNA	AC138057.1	ENSG00000243903.1	0	0
lincRNA	AC126121.2	ENSG00000242545.1	0	0
lincRNA	LINC00698	ENSG00000244342.5	0	0
lincRNA	LINC00994	ENSG00000189196.4	0	0
lincRNA	AC096888.1	ENSG00000244564.1	0	0
lincRNA	LINC02040	ENSG00000243149.1	0	0
lincRNA	AC098969.1	ENSG00000285738.1	0	0
lincRNA	AC114401.1	ENSG00000241884.2	0	0
lincRNA	AC099328.2	ENSG00000240842.1	0	0

lincRNA	AC104445.1	ENSG00000241667.1	0	0
lincRNA	SAMMSON	ENSG00000240405.6	0	0
lincRNA	LINC00877	ENSG00000241163.7	0	0
lincRNA	AC105265.3	ENSG00000273461.5	0	0
lincRNA	AC105265.4	ENSG00000276471.1	0	0
lincRNA	LINC00870	ENSG00000243083.6	0	1
lincRNA	AC112219.2	ENSG00000272774.1	0	0
lincRNA	AC112219.1	ENSG00000242618.1	0	0
lincRNA	AC134508.1	ENSG00000260670.1	0	0
lincRNA	AC104435.2	ENSG00000244345.1	0	0
lincRNA	LINC02005	ENSG00000242741.1	0	0
lincRNA	LINC02047	ENSG00000239454.1	0	0
lincRNA	LINC02077	ENSG00000244461.1	0	0
lincRNA	AC117462.1	ENSG00000240573.1	0	0
lincRNA	AC108752.1	ENSG00000240241.5	0	0
lincRNA	AC068756.1	ENSG00000242828.1	0	0
lincRNA	LINC02050	ENSG00000242781.1	0	0
lincRNA	LINC02027	ENSG00000243694.2	0	0
lincRNA	AC099542.2	ENSG00000242145.1	0	0
lincRNA	AC099542.1	ENSG00000241593.5	0	0
lincRNA	AC017015.2	ENSG00000242190.1	0	0
lincRNA	AC129807.1	ENSG00000242009.1	0	0
lincRNA	LINC02008	ENSG00000239440.5	0	0
lincRNA	LINC00971	ENSG00000242641.5	0	0
lincRNA	LINC02025	ENSG00000242339.1	0	0
lincRNA	AC108733.1	ENSG00000239767.1	0	0
lincRNA	LINC02070	ENSG00000241328.1	0	0
lincRNA	LINC00506	ENSG00000281392.1	0	0
lincRNA	AC108749.1	ENSG00000239572.2	0	0
lincRNA	LINC00879	ENSG00000239589.5	0	0
lincRNA	AC110491.1	ENSG00000261292.2	0	0
lincRNA	AC026100.1	ENSG00000285614.1	0	0
lincRNA	AC117473.1	ENSG00000251088.1	0	0
lincRNA	AC078828.1	ENSG00000243296.1	0	0
lincRNA	AC107029.1	ENSG00000243089.1	0	0
lincRNA	AC107029.2	ENSG00000244464.1	0	0
lincRNA	AC069222.1	ENSG00000273374.1	0	0
lincRNA	LINC02085	ENSG00000214407.3	0	0
lincRNA	AC106712.1	ENSG00000241280.1	0	0
lincRNA	AC108748.1	ENSG00000241754.1	0	0
lincRNA	AC063944.1	ENSG00000239828.6	0	0
lincRNA	LINC01215	ENSG00000271856.1	0	0
lincRNA	AC063923.2	ENSG00000261364.1	0	0
lincRNA	LINC01205	ENSG00000228980.3	0	0

lincRNA	AC078980.1	ENSG00000242029.1	0	0
lincRNA	AC117430.1	ENSG00000240895.1	0	0
lincRNA	AC117402.1	ENSG00000206532.2	0	0
lincRNA	AC092916.1	ENSG00000239311.1	0	0
lincRNA	AC112487.1	ENSG00000239482.6	0	0
lincRNA	AC092894.1	ENSG00000243081.2	0	0
lincRNA	LINC02042	ENSG00000240893.1	0	0
lincRNA	AC078785.2	ENSG00000241219.1	0	0
lincRNA	LINC02044	ENSG00000243795.1	0	0
lincRNA	AC108693.2	ENSG00000242659.1	0	0
lincRNA	ZBTB20-AS5	ENSG00000242290.2	0	0
lincRNA	ZBTB20-AS2	ENSG00000241295.1	0	2
lincRNA	ZBTB20-AS3	ENSG00000239946.1	0	0
lincRNA	ZBTB20-AS4	ENSG00000242767.1	0	1
lincRNA	AC046136.1	ENSG00000242880.1	0	0
lincRNA	AC092691.1	ENSG00000239268.2	0	0
lincRNA	LINC02024	ENSG00000241213.1	0	0
lincRNA	AC092691.3	ENSG00000242816.1	0	0
lincRNA	AC068633.1	ENSG00000243276.5	0	0
lincRNA	AC063952.4	ENSG00000282950.1	0	0
lincRNA	LINC02049	ENSG00000243593.1	0	0
lincRNA	AC112503.2	ENSG00000273454.1	0	0
lincRNA	AC092902.3	ENSG00000272840.1	0	0
lincRNA	AC016924.1	ENSG00000250934.1	0	0
lincRNA	AC063919.1	ENSG00000251448.1	0	0
lincRNA	LINC02016	ENSG00000244215.1	0	0
lincRNA	LINC01471	ENSG00000239921.3	0	0
lincRNA	AC016968.1	ENSG00000240562.1	0	0
lincRNA	LINC02034	ENSG00000282975.1	0	0
lincRNA	AC084035.1	ENSG00000282860.1	0	0
lincRNA	AL449214.1	ENSG00000285619.1	0	0
lincRNA	LINC01565	ENSG00000198685.3	0	0
lincRNA	AC112484.3	ENSG00000261159.1	0	0
lincRNA	LINC02014	ENSG00000248243.1	0	0
lincRNA	AC097105.1	ENSG00000285631.1	0	0
lincRNA	AC079942.1	ENSG00000249725.1	0	0
lincRNA	AC022296.3	ENSG00000272832.1	0	0
lincRNA	LINC02000	ENSG00000243832.1	0	0
lincRNA	LINC02004	ENSG00000240006.1	0	0
lincRNA	AC010207.1	ENSG00000260633.1	0	0
lincRNA	LINC01210	ENSG00000239513.5	0	0
lincRNA	AC007159.1	ENSG00000261146.1	0	0
lincRNA	BPESC1	ENSG00000232416.3	0	0
lincRNA	PISRT1	ENSG00000281473.1	0	0

lincRNA	AC110716.2	ENSG00000261763.1	0	0
lincRNA	AC110716.1	ENSG00000250543.1	0	0
lincRNA	AC016933.1	ENSG00000251471.1	0	0
lincRNA	AC121333.1	ENSG00000261826.1	0	0
lincRNA	AC018450.2	ENSG00000243733.1	0	0
lincRNA	LINC02010	ENSG00000242671.1	0	0
lincRNA	AC092958.1	ENSG00000239922.1	0	0
lincRNA	AC092958.4	ENSG00000285798.1	0	0
lincRNA	LINC02032	ENSG00000241131.1	0	0
lincRNA	AC092958.3	ENSG00000285660.1	0	0
lincRNA	AC092958.2	ENSG00000241792.1	0	0
lincRNA	AC025566.1	ENSG00000244650.2	0	0
lincRNA	LINC02045	ENSG00000243347.1	0	0
lincRNA	LINC02046	ENSG00000242440.1	0	0
lincRNA	AC069410.1	ENSG00000285557.1	0	0
lincRNA	AC108751.4	ENSG00000243885.1	0	0
lincRNA	AC117395.1	ENSG00000242791.2	0	1
lincRNA	AC117386.1	ENSG00000241048.1	0	0
lincRNA	LINC01214	ENSG00000243550.2	0	0
lincRNA	LINC02066	ENSG00000241151.1	0	0
lincRNA	AC108718.1	ENSG00000239941.1	0	0
lincRNA	AC092924.1	ENSG00000244545.1	0	0
lincRNA	AC078788.1	ENSG00000241220.1	0	0
lincRNA	AC078788.2	ENSG00000241912.1	0	0
lincRNA	C3orf79	ENSG00000237787.5	0	0
lincRNA	AC068985.1	ENSG00000243486.1	0	0
lincRNA	AC092994.1	ENSG00000243861.1	0	0
lincRNA	AC073359.2	ENSG00000243969.1	0	0
lincRNA	AC073359.1	ENSG00000243953.1	0	0
lincRNA	LINC01487	ENSG00000241336.1	0	0
lincRNA	AC104831.1	ENSG00000242790.1	0	0
lincRNA	LINC00880	ENSG00000243629.1	0	0
lincRNA	LINC02029	ENSG00000241544.1	0	1
lincRNA	LINC00881	ENSG00000241135.5	0	0
lincRNA	AC079943.1	ENSG00000239716.1	0	0
lincRNA	AC079943.2	ENSG00000242536.1	0	0
lincRNA	AC080013.4	ENSG00000272087.1	0	0
lincRNA	LINC01100	ENSG00000242107.2	0	0
lincRNA	LINC02067	ENSG00000240567.1	0	0
lincRNA	AC112491.1	ENSG00000269888.1	0	0
lincRNA	AC131211.1	ENSG00000240354.1	0	0
lincRNA	AC128685.1	ENSG00000241168.1	0	0
lincRNA	LINC01192	ENSG00000241369.5	0	0
lincRNA	AC079910.1	ENSG00000240792.1	0	0

lincRNA	LINC01323	ENSG00000241636.1	0	0
lincRNA	LINC01324	ENSG00000241767.2	0	0
lincRNA	LINC02023	ENSG00000239205.1	0	0
lincRNA	LINC01326	ENSG00000244321.1	0	0
lincRNA	LINC01327	ENSG00000241669.1	0	0
lincRNA	AC026353.1	ENSG00000244706.2	0	0
lincRNA	LINC02082	ENSG00000242268.2	0	0
lincRNA	AC092954.1	ENSG00000272797.1	0	0
lincRNA	AC092954.2	ENSG00000273177.1	0	0
lincRNA	LINC01997	ENSG00000241383.1	0	0
lincRNA	TERC	ENSG00000270141.3	0	0
lincRNA	AC073288.1	ENSG00000239628.1	0	0
lincRNA	AC073288.2	ENSG00000242578.1	0	2
lincRNA	AC026316.2	ENSG00000239739.1	0	0
lincRNA	TMEM212-	ENSG00000234717.1	0	0
lincRNA	AC068759.1	ENSG00000237473.1	0	0
lincRNA	AC104640.1	ENSG00000260217.1	0	0
lincRNA	LINC01208	ENSG00000223715.1	0	0
lincRNA	AC092920.1	ENSG00000232461.1	0	0
lincRNA	LINC01209	ENSG00000228308.1	0	0
lincRNA	LINC00501	ENSG00000203645.2	0	0
lincRNA	AC117465.1	ENSG00000226782.2	0	0
lincRNA	AC026355.2	ENSG00000236385.1	0	0
lincRNA	AC007953.1	ENSG00000225790.1	0	0
lincRNA	AC076966.1	ENSG00000229102.1	0	0
lincRNA	AC007823.1	ENSG00000260743.1	0	0
lincRNA	AC007687.1	ENSG00000270178.1	0	1
lincRNA	LINC02053	ENSG00000241696.1	0	0
lincRNA	AC125618.1	ENSG00000239774.1	0	0
lincRNA	AC108734.4	ENSG00000285336.1	0	0
lincRNA	AC022294.1	ENSG00000241231.1	0	0
lincRNA	AC125613.1	ENSG00000239381.6	0	0
lincRNA	AC012081.1	ENSG00000240478.1	0	0
lincRNA	AC084211.1	ENSG00000242012.1	0	0
lincRNA	LINC01994	ENSG00000241098.1	0	0
lincRNA	LINC01995	ENSG00000244247.1	0	0
lincRNA	LINC02031	ENSG00000238837.3	0	0
lincRNA	AC083801.2	ENSG00000260377.1	0	0
lincRNA	AC131235.4	ENSG00000273181.1	0	1
lincRNA	LINC02054	ENSG00000224153.3	0	0
lincRNA	LINC01839	ENSG00000227509.1	0	0
lincRNA	AC107294.1	ENSG00000272922.1	0	0
lincRNA	LINC02069	ENSG00000229433.1	0	0
lincRNA	LINC02020	ENSG00000234548.5	0	0

lincRNA	LINC02052	ENSG00000224406.5	0	0
lincRNA	LINC02051	ENSG00000226859.1	0	0
lincRNA	LINC02043	ENSG00000232233.1	0	0
lincRNA	LINC02041	ENSG00000228952.1	0	0
lincRNA	AC108681.1	ENSG00000224049.1	0	0
lincRNA	AC068295.1	ENSG00000236864.1	0	0
lincRNA	LINC01991	ENSG00000224187.1	0	0
lincRNA	AC092941.1	ENSG00000225058.1	0	0
lincRNA	AC092941.2	ENSG00000236412.1	0	0
lincRNA	AC022498.1	ENSG00000234238.1	0	0
lincRNA	TPRG1-AS1	ENSG00000234076.1	0	0
lincRNA	AC108747.1	ENSG00000273370.1	0	0
lincRNA	LINC02013	ENSG00000228730.1	0	0
lincRNA	AC073365.1	ENSG00000223812.6	0	0
lincRNA	AC026320.1	ENSG00000232353.5	0	0
lincRNA	AC092966.1	ENSG00000232130.1	0	0
lincRNA	LINC02038	ENSG00000229155.1	0	0
lincRNA	LINC02026	ENSG00000214146.2	0	0
lincRNA	AC024559.1	ENSG00000238043.1	0	0
lincRNA	AC024559.2	ENSG00000276407.4	0	0
lincRNA	AC080129.1	ENSG00000229964.1	0	0
lincRNA	LINC02036	ENSG00000225742.5	0	1
lincRNA	LINC02037	ENSG00000238097.1	0	0
lincRNA	LINC02048	ENSG00000228271.1	0	0
lincRNA	AC106706.1	ENSG00000233987.1	0	0
lincRNA	LINC01968	ENSG00000237222.3	0	0
lincRNA	LINC01972	ENSG00000230401.1	0	0
lincRNA	AC090505.2	ENSG00000238031.1	0	0
lincRNA	LINC01983	ENSG00000223783.1	0	0
lincRNA	AC128709.2	ENSG00000235126.1	0	0
lincRNA	AC128709.1	ENSG00000229912.1	0	0
lincRNA	AC128709.3	ENSG00000237167.1	0	0
lincRNA	LINC02012	ENSG00000272989.1	0	0
lincRNA	AC024560.3	ENSG00000273308.1	0	0
lincRNA	AC024560.1	ENSG00000236833.1	0	0
lincRNA	AC139887.3	ENSG00000260262.1	0	0
lincRNA	AC019103.1	ENSG00000250259.1	0	0
lincRNA	AC147067.2	ENSG00000272783.1	0	0
lincRNA	AC016773.1	ENSG00000270195.1	0	0
lincRNA	AL590235.1	ENSG00000270090.5	0	0
lincRNA	AL121796.1	ENSG00000250681.1	0	0
lincRNA	LINC02171	ENSG00000250632.1	0	0
lincRNA	LINC02600	ENSG00000250986.1	0	0
lincRNA	AC105415.1	ENSG00000248516.1	0	0

lincRNA	LINC01396	ENSG00000273396.1	0	0
lincRNA	AC113615.1	ENSG00000251408.1	0	0
lincRNA	AC097381.1	ENSG00000228919.5	0	0
lincRNA	LINC02517	ENSG00000249145.2	0	0
lincRNA	AC105345.1	ENSG00000205959.3	0	0
lincRNA	AC209005.1	ENSG00000250915.1	0	0
lincRNA	AC084048.1	ENSG00000249334.1	0	0
lincRNA	LINC02498	ENSG00000283083.1	0	0
lincRNA	LINC02360	ENSG00000248300.1	0	0
lincRNA	AC108037.1	ENSG00000248262.1	0	0
lincRNA	LINC02270	ENSG00000251210.5	0	0
lincRNA	AC007370.2	ENSG00000250371.1	0	0
lincRNA	AC007370.1	ENSG00000250098.1	0	0
lincRNA	LINC01097	ENSG00000281202.2	0	0
lincRNA	LINC01096	ENSG00000246095.2	0	0
lincRNA	AC007126.1	ENSG00000250497.1	0	0
lincRNA	AC073848.1	ENSG00000251679.1	0	0
lincRNA	AC092546.1	ENSG00000249988.1	0	0
lincRNA	AC006296.2	ENSG00000251412.1	0	0
lincRNA	AC006296.1	ENSG00000248425.1	0	0
lincRNA	LINC00504	ENSG00000248360.7	0	0
lincRNA	CPEB2-DT	ENSG00000247624.6	0	0
lincRNA	AC106894.1	ENSG00000249998.1	0	0
lincRNA	LINC02493	ENSG00000250819.1	0	0
lincRNA	AC093871.1	ENSG00000251048.1	0	0
lincRNA	LINC02438	ENSG00000248238.1	0	0
lincRNA	AC024230.1	ENSG00000248515.1	0	0
lincRNA	AC110767.1	ENSG00000249441.1	0	0
lincRNA	KCNIP4-IT1	ENSG00000280650.1	0	0
lincRNA	AC097512.1	ENSG00000248837.5	0	0
lincRNA	AC114912.1	ENSG00000249645.1	0	0
lincRNA	AC092440.1	ENSG00000249547.1	0	0
lincRNA	ERVH-1	ENSG00000251292.1	0	0
lincRNA	LINC02473	ENSG00000261121.1	0	0
lincRNA	AC104662.1	ENSG00000271172.1	0	0
lincRNA	AC092436.2	ENSG00000248545.2	0	0
lincRNA	AC133961.1	ENSG00000251009.2	0	0
lincRNA	LINC02357	ENSG00000283314.1	0	0
lincRNA	AC024132.2	ENSG00000251080.1	0	0
lincRNA	AC024132.1	ENSG00000205830.1	0	0
lincRNA	LINC02261	ENSG00000249699.1	0	0
lincRNA	AC007106.1	ENSG00000251410.1	0	0
lincRNA	AC109588.1	ENSG00000250038.5	0	0
lincRNA	AC097480.1	ENSG00000250064.1	0	0

lincRNA	LINC02364	ENSG00000250190.1	0	0
lincRNA	AC068944.1	ENSG00000249228.1	0	0
lincRNA	AC109349.1	ENSG00000248176.1	0	0
lincRNA	LINC02472	ENSG00000248338.1	0	0
lincRNA	LINC02497	ENSG00000251182.1	0	0
lincRNA	AC104071.1	ENSG00000251434.1	0	0
lincRNA	LINC02506	ENSG00000251129.1	0	0
lincRNA	LINC02353	ENSG00000251329.1	0	0
lincRNA	AC097535.1	ENSG00000248417.1	0	0
lincRNA	AC016687.3	ENSG00000250954.5	0	0
lincRNA	AC016687.2	ENSG00000250723.5	0	0
lincRNA	LINC02484	ENSG00000248685.5	0	0
lincRNA	AC093689.1	ENSG00000250597.5	0	0
lincRNA	LINC02505	ENSG00000248215.5	0	0
lincRNA	AL136537.1	ENSG00000247810.6	0	0
lincRNA	AC098680.1	ENSG00000251642.1	0	0
lincRNA	LINC02513	ENSG00000248227.1	0	0
lincRNA	LINC01258	ENSG00000249534.1	0	0
lincRNA	LINC01259	ENSG00000249667.1	0	0
lincRNA	LINC02278	ENSG00000251635.1	0	0
lincRNA	AC079921.2	ENSG00000249685.1	0	0
lincRNA	AC095057.2	ENSG00000250338.1	0	0
lincRNA	LINC02265	ENSG00000249241.1	0	0
lincRNA	AC105389.1	ENSG00000249122.1	0	0
lincRNA	LINC00682	ENSG00000245870.3	0	0
lincRNA	AC108210.1	ENSG00000249771.1	0	0
lincRNA	AC106052.1	ENSG00000272862.1	0	0
lincRNA	AC111006.1	ENSG00000285454.1	0	0
lincRNA	AC096734.1	ENSG00000251517.1	0	0
lincRNA	LINC02383	ENSG00000248143.1	0	0
lincRNA	AC110799.1	ENSG00000250863.1	0	0
lincRNA	AC108467.1	ENSG00000248744.1	0	0
lincRNA	AC027271.1	ENSG00000272576.1	0	0
lincRNA	LINC02480	ENSG00000251040.1	0	0
lincRNA	AC104066.2	ENSG00000260120.1	0	0
lincRNA	LINC01618	ENSG00000250302.1	0	0
lincRNA	LINC02283	ENSG00000248184.1	0	0
lincRNA	AC098587.1	ENSG00000248518.1	0	0
lincRNA	AC098868.1	ENSG00000251264.1	0	0
lincRNA	LINC02260	ENSG00000250456.1	0	0
lincRNA	AC097494.1	ENSG00000249727.1	0	0
lincRNA	LINC02358	ENSG00000251256.1	0	0
lincRNA	AC111194.1	ENSG00000248317.1	0	0
lincRNA	AC069200.1	ENSG00000273257.1	0	0

lincRNA	AC068620.1	ENSG00000269921.1	0	0
lincRNA	AC068620.2	ENSG00000270147.1	0	0
lincRNA	AC105390.1	ENSG00000250192.1	0	0
lincRNA	LINC02380	ENSG00000248505.1	0	0
lincRNA	AC013724.1	ENSG00000251459.1	0	0
lincRNA	AC107396.1	ENSG00000251049.2	0	0
lincRNA	AC093725.2	ENSG00000250333.1	0	0
lincRNA	AC093725.1	ENSG00000249831.2	0	0
lincRNA	LINC02494	ENSG00000250340.1	0	0
lincRNA	AC019133.1	ENSG00000250078.1	0	0
lincRNA	AC017091.1	ENSG00000251339.5	0	0
lincRNA	LINC02429	ENSG00000251266.1	0	0
lincRNA	AC097501.2	ENSG00000250375.2	0	0
lincRNA	AC108517.1	ENSG00000249111.1	0	0
lincRNA	AC096588.1	ENSG00000249392.1	0	0
lincRNA	AC093857.1	ENSG00000249892.1	0	0
lincRNA	LINC02496	ENSG00000249066.1	0	0
lincRNA	LINC02271	ENSG00000240152.2	0	0
lincRNA	AC020741.1	ENSG00000205682.2	0	0
lincRNA	AC097491.1	ENSG00000251055.2	0	0
lincRNA	AC093730.1	ENSG00000250775.1	0	0
lincRNA	LINC02232	ENSG00000250125.2	0	0
lincRNA	AC018683.1	ENSG00000285711.1	0	0
lincRNA	AC104137.1	ENSG00000248479.1	0	0
lincRNA	AC097110.1	ENSG00000251527.1	0	0
lincRNA	AC116049.2	ENSG00000249413.2	0	0
lincRNA	AC096721.1	ENSG00000272304.1	0	0
lincRNA	AC104806.2	ENSG00000250075.5	0	0
lincRNA	AC021146.12	ENSG00000272626.1	0	0
lincRNA	AC068721.1	ENSG00000248567.1	0	0
lincRNA	LINC02499	ENSG00000250436.1	0	0
lincRNA	AC112518.1	ENSG00000228277.1	0	0
lincRNA	AC025244.1	ENSG00000251185.1	0	0
lincRNA	AC025244.2	ENSG00000251454.1	0	0
lincRNA	LINC02483	ENSG00000251383.1	0	0
lincRNA	AC096759.1	ENSG00000248646.1	0	0
lincRNA	AC096759.2	ENSG00000250735.5	0	0
lincRNA	AC092674.1	ENSG00000249036.1	0	0
lincRNA	LINC00989	ENSG00000250334.5	0	0
lincRNA	AC092542.1	ENSG00000251399.1	0	0
lincRNA	LINC02469	ENSG00000248408.1	0	0
lincRNA	PCAT4	ENSG00000251321.1	0	0
lincRNA	LINC00575	ENSG00000231782.6	0	0
lincRNA	AC114781.2	ENSG00000250057.1	0	0

lincRNA	AC021192.1	ENSG00000249171.1	0	0
lincRNA	AC104063.1	ENSG00000248749.1	0	0
lincRNA	AC108516.1	ENSG00000250572.1	0	0
lincRNA	AC112250.2	ENSG00000272856.1	0	0
lincRNA	AC107067.1	ENSG00000246375.2	0	0
lincRNA	AC083829.2	ENSG00000285122.1	0	2
lincRNA	AC104785.1	ENSG00000270720.1	0	1
lincRNA	AC093810.1	ENSG00000249052.1	0	0
lincRNA	LNCPRESS2	ENSG00000249152.1	0	0
lincRNA	AC112695.1	ENSG00000248511.1	0	0
lincRNA	AC096746.1	ENSG00000246541.2	0	0
lincRNA	AC108067.1	ENSG00000249951.1	0	0
lincRNA	BMPR1B-DT	ENSG00000249599.1	0	0
lincRNA	LINC02267	ENSG00000248510.2	0	0
lincRNA	AC108515.1	ENSG00000253170.1	0	0
lincRNA	AC105460.1	ENSG00000250920.1	0	0
lincRNA	AC004052.1	ENSG00000251170.5	0	0
lincRNA	AC004063.1	ENSG00000250670.1	0	0
lincRNA	AC004053.1	ENSG00000248242.1	0	0
lincRNA	AC096577.1	ENSG00000248373.5	0	0
lincRNA	AC004069.1	ENSG00000251259.1	0	0
lincRNA	LINC02173	ENSG00000249613.1	0	0
lincRNA	AC104663.1	ENSG00000251081.1	0	0
lincRNA	RPL34-AS1	ENSG00000234492.4	0	2
lincRNA	ZCCHC23	ENSG00000246774.1	0	0
lincRNA	AC126283.1	ENSG00000272795.1	0	0
lincRNA	PANCR	ENSG00000250103.2	0	0
lincRNA	LINC01438	ENSG00000249519.1	0	0
lincRNA	AC083795.2	ENSG00000248656.1	0	0
lincRNA	AC139718.2	ENSG00000250855.1	0	0
lincRNA	AC139718.1	ENSG00000248432.1	0	0
lincRNA	AC004704.1	ENSG00000249815.1	0	0
lincRNA	AC109347.2	ENSG00000272567.1	0	0
lincRNA	AC106864.2	ENSG00000250046.3	0	0
lincRNA	AC106892.1	ENSG00000250791.1	0	0
lincRNA	LINC02262	ENSG00000224932.1	0	0
lincRNA	LINC02263	ENSG00000228358.5	0	0
lincRNA	LINC01378	ENSG00000236922.9	0	0
lincRNA	LINC02264	ENSG00000229565.1	0	0
lincRNA	AC093752.3	ENSG00000260091.1	0	0
lincRNA	AC093752.2	ENSG00000250950.1	0	0
lincRNA	LINC01365	ENSG00000250772.5	0	0
lincRNA	LINC02502	ENSG00000250392.2	0	0
lincRNA	AC097173.2	ENSG00000253825.1	0	0

lincRNA	AC025741.1	ENSG00000249409.1	0	0
lincRNA	AC105254.1	ENSG00000250344.1	0	0
lincRNA	AC079341.1	ENSG00000261501.1	0	0
lincRNA	AC093821.1	ENSG00000249125.1	0	0
lincRNA	LINC02435	ENSG00000251526.1	0	0
lincRNA	AC096732.1	ENSG00000226655.1	0	0
lincRNA	AC121154.1	ENSG00000249837.1	0	0
lincRNA	LINC02516	ENSG00000261083.1	0	0
lincRNA	AC105919.1	ENSG00000250149.1	0	0
lincRNA	LINC02379	ENSG00000251649.1	0	0
lincRNA	AC096773.1	ENSG00000250945.1	0	1
lincRNA	AC093772.1	ENSG00000248491.5	0	4
lincRNA	AC110609.1	ENSG00000273077.1	0	0
lincRNA	AC078850.2	ENSG00000248802.1	0	0
lincRNA	JADRR	ENSG00000280711.1	0	0
lincRNA	AC082650.1	ENSG00000250193.1	0	0
lincRNA	LINC02465	ENSG00000249618.5	0	0
lincRNA	LINC02479	ENSG00000251326.1	0	0
lincRNA	AC093831.1	ENSG00000250503.1	0	0
lincRNA	SNHG27	ENSG00000251676.1	0	0
lincRNA	AC096711.2	ENSG00000251555.1	0	0
lincRNA	AC096711.3	ENSG00000251598.1	0	0
lincRNA	AC096711.1	ENSG00000249463.1	0	0
lincRNA	AC114741.1	ENSG00000248369.1	0	0
lincRNA	LINC01256	ENSG00000251398.1	0	0
lincRNA	AC115622.1	ENSG00000251488.1	0	0
lincRNA	AC110751.1	ENSG00000249513.2	0	0
lincRNA	AC105383.1	ENSG00000250241.5	0	0
lincRNA	AC079380.1	ENSG00000251388.1	0	0
lincRNA	AC109830.1	ENSG00000251199.5	0	0
lincRNA	LINC02462	ENSG00000251291.1	0	0
lincRNA	AC093722.1	ENSG00000249000.1	0	0
lincRNA	AC104619.2	ENSG00000249847.1	0	0
lincRNA	AC107463.1	ENSG00000248434.1	0	0
lincRNA	LINC02485	ENSG00000248456.1	0	0
lincRNA	AC105362.1	ENSG00000250865.1	0	0
lincRNA	LINC00613	ENSG00000248330.5	0	0
lincRNA	AC073429.2	ENSG00000261166.1	0	0
lincRNA	AC018680.1	ENSG00000251567.5	0	0
lincRNA	AC073429.1	ENSG00000250665.1	0	0
lincRNA	LINC02510	ENSG00000250341.1	0	0
lincRNA	LINC02172	ENSG00000251632.1	0	2
lincRNA	AC116563.1	ENSG00000250126.1	0	2
lincRNA	AC131956.2	ENSG00000250777.1	0	0

lincRNA	AC131956.1	ENSG00000250034.1	0	0
lincRNA	LINC00616	ENSG00000248307.5	0	0
lincRNA	AC093903.1	ENSG00000249091.1	0	0
lincRNA	LINC00498	ENSG00000248397.1	0	0
lincRNA	LINC00499	ENSG00000251372.5	0	0
lincRNA	LINC00500	ENSG00000249381.1	0	0
lincRNA	AC098859.2	ENSG00000250977.1	0	0
lincRNA	AC093766.1	ENSG00000250501.2	0	0
lincRNA	AC109927.2	ENSG00000250577.1	0	0
lincRNA	AC096733.2	ENSG00000273472.1	0	0
lincRNA	AC107220.1	ENSG00000248747.1	0	0
lincRNA	LINC02432	ENSG00000248810.1	0	0
lincRNA	AC097504.2	ENSG00000272632.1	0	0
lincRNA	LINC02276	ENSG00000250141.1	0	0
lincRNA	AC139720.2	ENSG00000251248.1	0	0
lincRNA	AC139720.1	ENSG00000249806.1	0	0
lincRNA	AC097658.2	ENSG00000250969.1	0	0
lincRNA	AC106871.1	ENSG00000261129.1	0	0
lincRNA	AC109811.1	ENSG00000250406.1	0	0
lincRNA	LINC02266	ENSG00000272727.1	0	0
lincRNA	AC108206.1	ENSG00000260303.1	0	0
lincRNA	AC097372.2	ENSG00000251024.1	0	0
lincRNA	LINC01095	ENSG00000248809.5	0	0
lincRNA	AC092435.1	ENSG00000248346.1	0	0
lincRNA	AC092435.3	ENSG00000260090.1	0	0
lincRNA	LINC02507	ENSG00000251688.1	0	0
lincRNA	AC108935.1	ENSG00000250357.1	0	0
lincRNA	AC108156.1	ENSG00000249752.1	0	0
lincRNA	LINC02430	ENSG00000250252.1	0	0
lincRNA	LINC02355	ENSG00000248210.1	0	0
lincRNA	AC093893.1	ENSG00000250905.1	0	0
lincRNA	AC105343.1	ENSG00000250704.1	0	0
lincRNA	AC104819.3	ENSG00000270265.1	0	0
lincRNA	FAM160A1-	ENSG00000251611.1	0	0
lincRNA	AC107074.1	ENSG00000249184.1	0	0
lincRNA	AC112242.1	ENSG00000249708.1	0	0
lincRNA	AC097375.2	ENSG00000249818.1	0	0
lincRNA	AC097375.1	ENSG00000248991.1	0	0
lincRNA	AC079340.1	ENSG00000250706.1	0	0
lincRNA	AC079340.2	ENSG00000251377.1	0	0
lincRNA	AC080078.1	ENSG00000270302.1	0	0
lincRNA	AC080078.2	ENSG00000270883.1	0	0
lincRNA	AC093599.1	ENSG00000249022.1	0	0
lincRNA	AC097467.1	ENSG00000249924.2	0	0

lincRNA	AC107208.1	ENSG00000251244.1	0	0
lincRNA	AC096736.3	ENSG00000251511.1	0	0
lincRNA	LINC02272	ENSG00000251283.1	0	0
lincRNA	AC096736.1	ENSG00000248912.1	0	0
lincRNA	LINC02433	ENSG00000234111.6	0	0
lincRNA	AC093817.1	ENSG00000249275.1	0	0
lincRNA	AC074344.2	ENSG00000250180.1	0	0
lincRNA	LINC02233	ENSG00000250488.1	0	0
lincRNA	LINC02477	ENSG00000249425.1	0	0
lincRNA	AC005150.1	ENSG00000248431.1	0	0
lincRNA	AC106872.13	ENSG00000285563.1	0	0
lincRNA	AC106872.10	ENSG00000250887.1	0	0
lincRNA	AC097487.1	ENSG00000249675.1	0	0
lincRNA	AC079349.1	ENSG00000250150.1	0	0
lincRNA	LINC02275	ENSG00000248319.1	0	0
lincRNA	LINC02512	ENSG00000251061.2	0	0
lincRNA	LINC02382	ENSG00000250968.1	0	0
lincRNA	LINC02431	ENSG00000248872.1	0	0
lincRNA	LINC02504	ENSG00000248339.1	0	0
lincRNA	AC074254.1	ENSG00000251176.1	0	0
lincRNA	LINC02174	ENSG00000249297.1	0	0
lincRNA	AC093849.1	ENSG00000261646.1	0	0
lincRNA	AC106895.1	ENSG00000250043.1	0	0
lincRNA	LINC02269	ENSG00000250708.1	0	0
lincRNA	AC106895.2	ENSG00000251216.1	0	0
lincRNA	LINC02268	ENSG00000248174.5	0	0
lincRNA	AC012055.1	ENSG00000250957.1	0	0
lincRNA	AC012055.2	ENSG00000251213.1	0	0
lincRNA	AC116616.1	ENSG00000249875.1	0	0
lincRNA	AC095050.1	ENSG00000248551.1	0	0
lincRNA	AC131094.1	ENSG00000249945.1	0	0
lincRNA	AC092673.1	ENSG00000249458.1	0	0
lincRNA	LINC02509	ENSG00000250263.1	0	0
lincRNA	AC097518.1	ENSG00000249008.1	0	0
lincRNA	AC103879.1	ENSG00000251416.1	0	0
lincRNA	LINC01098	ENSG00000231171.3	0	0
lincRNA	LINC01099	ENSG00000251504.1	0	0
lincRNA	AC104803.1	ENSG00000249883.1	0	0
lincRNA	LINC00290	ENSG00000248197.1	0	0
lincRNA	LINC02500	ENSG00000249460.1	0	0
lincRNA	TEMN3-AS1	ENSG00000251336.1	0	0
lincRNA	AC079766.1	ENSG00000272646.1	0	0
lincRNA	AC019193.3	ENSG00000273180.1	0	0
lincRNA	AC019193.2	ENSG00000272784.1	0	0

lincRNA	AC108477.1	ENSG00000283383.1	0	0
lincRNA	LINC02363	ENSG00000180712.3	0	0
lincRNA	LINC02362	ENSG00000249096.6	0	0
lincRNA	LINC02427	ENSG00000271538.5	0	0
lincRNA	LINC02365	ENSG00000254233.1	0	0
lincRNA	MIR3945HG	ENSG00000251230.5	0	0
lincRNA	LINC01093	ENSG00000249173.5	0	0
lincRNA	LINC02437	ENSG00000248517.1	0	0
lincRNA	LINC02436	ENSG00000250754.5	0	0
lincRNA	AC096659.1	ENSG00000235149.1	0	0
lincRNA	AC108865.1	ENSG00000250829.2	0	0
lincRNA	AC110772.1	ENSG00000249742.1	0	0
lincRNA	AC108474.1	ENSG00000250971.1	0	0
lincRNA	LINC02374	ENSG00000249747.1	0	0
lincRNA	LINC02514	ENSG00000250042.1	0	0
lincRNA	LINC02515	ENSG00000250620.1	0	0
lincRNA	AC093763.1	ENSG00000251490.1	0	0
lincRNA	LINC02492	ENSG00000250590.5	0	0
lincRNA	AC097521.1	ENSG00000249642.1	0	0
lincRNA	LINC01060	ENSG00000249378.5	0	0
lincRNA	AC093909.2	ENSG00000249269.1	0	0
lincRNA	LINC02508	ENSG00000251619.1	0	0
lincRNA	AC093909.4	ENSG00000250626.2	0	0
lincRNA	AC122138.1	ENSG00000250024.1	0	0
lincRNA	AC107391.1	ENSG00000251310.1	0	0
lincRNA	AC105924.1	ENSG00000249877.1	0	0
lincRNA	LINC01262	ENSG00000250739.1	0	0
lincRNA	FRG1-DT	ENSG00000245685.6	0	0
lincRNA	LINC01596	ENSG00000250666.1	0	0
lincRNA	DBET	ENSG00000281591.1	0	0
lincRNA	AC122719.1	ENSG00000248949.1	0	0
lincRNA	AC122719.2	ENSG00000250173.1	0	0
lincRNA	AC116351.2	ENSG00000272347.1	0	0
lincRNA	LINC01511	ENSG00000250584.2	0	0
lincRNA	AC091849.2	ENSG00000251532.1	0	0
lincRNA	AC026412.3	ENSG00000271119.1	0	0
lincRNA	AC112176.1	ENSG00000260066.1	0	0
lincRNA	LINC02116	ENSG00000250417.1	0	0
lincRNA	AC126768.2	ENSG00000248994.1	0	0
lincRNA	AC126768.1	ENSG00000248597.1	0	0
lincRNA	AC126768.3	ENSG00000249731.1	0	0
lincRNA	LSINCT5	ENSG00000281560.1	0	0
lincRNA	AC091891.1	ENSG00000259757.1	0	0
lincRNA	AC094105.1	ENSG00000248137.1	0	0

lincRNA	AC112192.1	ENSG00000248736.1	0	0
lincRNA	LINC01377	ENSG00000249808.2	0	0
lincRNA	LINC01019	ENSG00000248118.1	0	0
lincRNA	LINC02162	ENSG00000249830.1	0	0
lincRNA	LINC01017	ENSG00000250716.1	0	0
lincRNA	AC025773.1	ENSG00000248962.1	0	0
lincRNA	AC025187.1	ENSG00000249994.1	0	0
lincRNA	LINC02063	ENSG00000250921.1	0	0
lincRNA	AC106799.3	ENSG00000260763.1	0	0
lincRNA	AC106799.2	ENSG00000248973.1	0	0
lincRNA	AC106799.1	ENSG00000231291.3	0	0
lincRNA	AC010634.1	ENSG00000251371.1	0	0
lincRNA	LINC02114	ENSG00000249521.1	0	0
lincRNA	AC026719.1	ENSG00000248311.1	0	0
lincRNA	AC026719.2	ENSG00000248467.1	0	0
lincRNA	AC026415.1	ENSG00000249941.1	0	0
lincRNA	AC010451.3	ENSG00000251506.1	0	0
lincRNA	AC010451.1	ENSG00000250481.1	0	0
lincRNA	LINC01020	ENSG00000215231.7	0	0
lincRNA	AC010451.2	ENSG00000251426.1	0	0
lincRNA	LINC02121	ENSG00000250529.1	0	0
lincRNA	AC010266.2	ENSG00000261037.1	0	0
lincRNA	AC010266.1	ENSG00000259861.1	0	0
lincRNA	LINC02145	ENSG00000250490.1	0	0
lincRNA	LINC01018	ENSG00000250056.5	0	0
lincRNA	AC122710.2	ENSG00000251365.2	0	0
lincRNA	LINC02196	ENSG00000250974.2	0	0
lincRNA	AC122710.1	ENSG00000250060.1	0	0
lincRNA	AC091951.4	ENSG00000285689.1	0	0
lincRNA	AC027343.3	ENSG00000251678.1	0	0
lincRNA	LINC02123	ENSG00000250668.1	0	0
lincRNA	AC027343.1	ENSG00000249638.1	0	0
lincRNA	LINC02142	ENSG00000223597.2	0	0
lincRNA	AC025174.1	ENSG00000251168.1	0	0
lincRNA	LINC02226	ENSG00000245729.2	0	0
lincRNA	AC091965.1	ENSG00000249159.6	0	0
lincRNA	AC027328.1	ENSG00000249782.1	0	0
lincRNA	LINC02199	ENSG00000250198.1	0	0
lincRNA	SEMA5A-	ENSG00000251370.1	0	0
lincRNA	LINC02112	ENSG00000249781.6	0	0
lincRNA	LINC02221	ENSG00000250814.1	0	0
lincRNA	AC034229.1	ENSG00000249807.1	0	0
lincRNA	AC034229.2	ENSG00000260515.1	0	0
lincRNA	AC034229.3	ENSG00000271998.1	0	0

lincRNA	AC012640.3	ENSG00000271715.1	0	0
lincRNA	AC092336.1	ENSG00000249166.1	0	0
lincRNA	LINC02212	ENSG00000249396.1	0	0
lincRNA	LINC02213	ENSG00000249160.4	0	0
lincRNA	AC012629.1	ENSG00000250576.1	0	0
lincRNA	AC106771.1	ENSG00000248783.1	0	0
lincRNA	LINC01194	ENSG00000248131.5	0	0
lincRNA	LINC02220	ENSG00000248457.1	0	0
lincRNA	AC016553.1	ENSG00000248145.1	0	0
lincRNA	AC016575.1	ENSG00000272057.1	0	0
lincRNA	AC016651.1	ENSG00000249332.1	0	0
lincRNA	LINC02149	ENSG00000248486.1	0	0
lincRNA	LINC02150	ENSG00000248150.1	0	0
lincRNA	AC022113.2	ENSG00000250415.1	0	0
lincRNA	AC026790.1	ENSG00000271892.1	0	0
lincRNA	AC026785.2	ENSG00000248223.1	0	0
lincRNA	AC026785.3	ENSG00000249199.1	0	0
lincRNA	LINC02111	ENSG00000250822.1	0	0
lincRNA	LINC02217	ENSG00000248455.5	0	0
lincRNA	LINC02223	ENSG00000249937.6	0	0
lincRNA	LINC02100	ENSG00000248693.1	0	0
lincRNA	AC106744.1	ENSG00000249174.1	0	0
lincRNA	AC106744.2	ENSG00000251487.1	0	0
lincRNA	CDH18-AS1	ENSG00000249854.1	0	0
lincRNA	LINC02241	ENSG00000251629.6	0	0
lincRNA	LINC02146	ENSG00000249490.1	0	0
lincRNA	AC010445.1	ENSG00000249404.1	0	0
lincRNA	AC010387.1	ENSG00000250694.1	0	0
lincRNA	LINC02239	ENSG00000248908.1	0	0
lincRNA	AC106821.1	ENSG00000250524.1	0	0
lincRNA	LINC02228	ENSG00000251273.3	0	0
lincRNA	AC099499.2	ENSG00000285042.1	0	0
lincRNA	AC099499.1	ENSG00000251654.2	0	0
lincRNA	AC022140.1	ENSG00000248605.5	0	0
lincRNA	AC025475.1	ENSG00000249099.1	0	0
lincRNA	AC113355.1	ENSG00000248490.1	0	0
lincRNA	LINC02103	ENSG00000249785.1	0	0
lincRNA	AC008825.1	ENSG00000250453.1	0	0
lincRNA	LINC02109	ENSG00000259786.6	0	0
lincRNA	LINC02064	ENSG00000248391.5	0	0
lincRNA	AC022447.3	ENSG00000248813.1	0	0
lincRNA	AC022447.1	ENSG00000248378.1	0	0
lincRNA	AC022447.7	ENSG00000250164.1	0	0
lincRNA	LINC02061	ENSG00000250885.1	0	0

lincRNA	LINC02120	ENSG00000248279.5	0	0
lincRNA	AC034223.1	ENSG00000249102.1	0	0
lincRNA	LINC02160	ENSG00000251443.1	0	0
lincRNA	AC034232.2	ENSG00000250583.1	0	0
lincRNA	AC139792.1	ENSG00000271771.1	0	0
lincRNA	AC026741.1	ENSG00000272103.2	0	0
lincRNA	LINC02117	ENSG00000248539.1	0	0
lincRNA	AC008869.1	ENSG00000250032.1	0	0
lincRNA	LINC02110	ENSG00000251518.1	0	0
lincRNA	AC034226.1	ENSG00000248533.1	0	0
lincRNA	LINC02107	ENSG00000250003.2	0	0
lincRNA	LINC02119	ENSG00000248461.1	0	0
lincRNA	AC010425.1	ENSG00000251189.1	0	0
lincRNA	LINC00603	ENSG00000250048.2	0	0
lincRNA	LINC00604	ENSG00000250585.2	0	1
lincRNA	AC093277.1	ENSG00000283286.1	0	0
lincRNA	AC114977.1	ENSG00000285552.1	0	0
lincRNA	AC114977.2	ENSG00000285616.1	0	0
lincRNA	AC108099.1	ENSG00000260786.1	0	0
lincRNA	AC008875.1	ENSG00000271788.1	0	0
lincRNA	AC025171.5	ENSG00000272382.1	0	0
lincRNA	AC025171.4	ENSG00000272144.1	0	0
lincRNA	AC104119.1	ENSG00000249276.1	0	0
lincRNA	LINC02224	ENSG00000249203.1	0	0
lincRNA	AC093292.1	ENSG00000250418.1	0	0
lincRNA	AC093297.1	ENSG00000248779.1	0	0
lincRNA	AC114954.1	ENSG00000248148.1	0	0
lincRNA	AC122694.1	ENSG00000250122.1	0	0
lincRNA	AC112187.3	ENSG00000271752.1	0	0
lincRNA	AC022441.1	ENSG00000249405.1	0	0
lincRNA	AC022441.2	ENSG00000251601.1	0	0
lincRNA	AC008808.2	ENSG00000250360.1	0	0
lincRNA	LINC02106	ENSG00000251573.2	0	0
lincRNA	AC008808.1	ENSG00000248918.2	0	0
lincRNA	AC091860.2	ENSG00000251125.1	0	0
lincRNA	LINC02118	ENSG00000248789.5	0	0
lincRNA	AC027329.1	ENSG00000285831.1	0	0
lincRNA	LINC02105	ENSG00000250447.5	0	0
lincRNA	LINC01033	ENSG00000249069.7	0	0
lincRNA	AC112198.3	ENSG00000249016.1	0	0
lincRNA	AC034238.3	ENSG00000251376.1	0	0
lincRNA	AC034238.2	ENSG00000248029.2	0	0
lincRNA	AC008892.1	ENSG00000249236.2	0	0
lincRNA	AC034245.1	ENSG00000249697.1	0	0

lincRNA	LINC01948	ENSG00000248727.5	0	0
lincRNA	AC008940.1	ENSG00000228650.2	0	0
lincRNA	AC008780.1	ENSG00000248294.1	0	0
lincRNA	AC008780.2	ENSG00000249436.1	0	0
lincRNA	AC106822.1	ENSG00000249198.1	0	0
lincRNA	LINC02225	ENSG00000249584.1	0	0
lincRNA	LINC02101	ENSG00000248132.2	0	0
lincRNA	LINC02108	ENSG00000250127.1	0	0
lincRNA	C5orf64-AS1	ENSG00000251575.2	0	0
lincRNA	AC026434.1	ENSG00000248199.1	0	0
lincRNA	AC092354.2	ENSG00000272370.1	0	0
lincRNA	AC025176.1	ENSG00000250081.1	0	0
lincRNA	LINC02065	ENSG00000248846.2	0	0
lincRNA	AC113378.1	ENSG00000251391.4	0	0
lincRNA	LINC02229	ENSG00000250313.2	0	0
lincRNA	AC010420.1	ENSG00000251206.1	0	0
lincRNA	AC112206.2	ENSG00000249364.5	0	0
lincRNA	LINC02242	ENSG00000249736.1	0	0
lincRNA	AC106798.1	ENSG00000250421.5	0	0
lincRNA	LINC02219	ENSG00000253787.1	0	0
lincRNA	AC024579.1	ENSG00000251044.2	0	0
lincRNA	AC010280.3	ENSG00000249588.1	0	0
lincRNA	AC010280.1	ENSG00000248359.1	0	0
lincRNA	AC093523.1	ENSG00000249335.1	0	0
lincRNA	AC093248.1	ENSG00000250066.1	0	0
lincRNA	LINC02198	ENSG00000249352.4	0	0
lincRNA	AC146944.4	ENSG00000269983.1	0	0
lincRNA	LINC02197	ENSG00000250387.2	0	0
lincRNA	AC145141.2	ENSG00000253985.1	0	0
lincRNA	AC145141.1	ENSG00000249981.1	0	0
lincRNA	LINC02056	ENSG00000248371.5	0	0
lincRNA	AC063979.2	ENSG00000251613.3	0	0
lincRNA	AC008972.2	ENSG00000272081.1	0	1
lincRNA	AC116345.1	ENSG00000249743.5	0	0
lincRNA	LINC02230	ENSG00000251543.1	0	0
lincRNA	FOXD1-AS1	ENSG00000247993.2	0	0
lincRNA	LINC01385	ENSG00000251604.1	0	0
lincRNA	AC093283.1	ENSG00000259968.3	0	0
lincRNA	LINC01335	ENSG00000248942.1	0	0
lincRNA	AC106732.1	ENSG00000251041.1	0	0
lincRNA	LINC01333	ENSG00000249343.1	0	0
lincRNA	LINC01332	ENSG00000250446.1	0	0
lincRNA	LINC01331	ENSG00000248673.1	0	0
lincRNA	AC116337.3	ENSG00000249856.1	0	0

lincRNA	LINC01336	ENSG00000250889.2	0	0
lincRNA	AC026774.1	ENSG00000248127.1	0	0
lincRNA	AC108482.1	ENSG00000253572.2	0	0
lincRNA	LINC01337	ENSG00000251221.1	0	0
lincRNA	AC026726.1	ENSG00000249483.1	0	0
lincRNA	AC026782.1	ENSG00000248393.1	0	0
lincRNA	AC026782.2	ENSG00000248870.1	0	0
lincRNA	AC008885.2	ENSG00000249100.1	0	0
lincRNA	LINC01338	ENSG00000281327.1	0	0
lincRNA	AC108174.1	ENSG00000248112.1	0	0
lincRNA	AC027338.2	ENSG00000249857.1	0	0
lincRNA	AC027338.1	ENSG00000249664.1	0	0
lincRNA	AC114928.1	ENSG00000249349.1	0	0
lincRNA	AC117522.2	ENSG00000249792.1	0	0
lincRNA	AC010486.1	ENSG00000248667.1	0	0
lincRNA	AC010486.2	ENSG00000249023.1	0	0
lincRNA	AC010486.3	ENSG00000249842.1	0	0
lincRNA	AC026414.1	ENSG00000249153.1	0	0
lincRNA	AC016550.1	ENSG00000248195.1	0	0
lincRNA	AC016550.2	ENSG00000248363.5	0	0
lincRNA	AC016550.3	ENSG00000250124.1	0	0
lincRNA	LINC02059	ENSG00000250544.1	0	0
lincRNA	AC008539.1	ENSG00000248701.1	0	0
lincRNA	AC109492.1	ENSG00000249061.1	0	0
lincRNA	LINC01949	ENSG00000233828.3	0	2
lincRNA	LINC02488	ENSG00000249362.1	0	0
lincRNA	LINC02144	ENSG00000248708.1	0	0
lincRNA	LINC02060	ENSG00000250156.3	0	0
lincRNA	AC091826.2	ENSG00000271904.1	0	0
lincRNA	AC008525.1	ENSG00000250377.1	0	0
lincRNA	AC074131.1	ENSG00000250831.1	0	0
lincRNA	LINC02161	ENSG00000250437.1	0	0
lincRNA	AC008448.1	ENSG00000214942.4	0	0
lincRNA	AC113167.1	ENSG00000249265.1	0	0
lincRNA	AC099554.1	ENSG00000253321.1	0	0
lincRNA	AC093281.1	ENSG00000212930.3	0	0
lincRNA	AC093281.2	ENSG00000251093.1	0	0
lincRNA	AC123595.1	ENSG00000234292.3	0	0
lincRNA	AC114316.2	ENSG00000250049.5	0	0
lincRNA	AC114316.1	ENSG00000248864.1	0	0
lincRNA	AC124854.1	ENSG00000249776.5	0	0
lincRNA	AC026780.1	ENSG00000249169.1	0	0
lincRNA	AC026780.2	ENSG00000249984.1	0	0
lincRNA	AC008517.1	ENSG00000248588.1	0	0

lincRNA	LINC02058	ENSG00000248528.1	0	0
lincRNA	AC012625.1	ENSG00000251361.1	0	0
lincRNA	AC108102.1	ENSG00000272406.1	0	0
lincRNA	AC008592.4	ENSG00000251409.1	0	0
lincRNA	AC008592.5	ENSG00000272021.1	0	0
lincRNA	LINC01554	ENSG00000236882.7	0	1
lincRNA	AC008592.2	ENSG00000250955.1	0	0
lincRNA	MIR583HG	ENSG00000250551.1	0	0
lincRNA	AC099509.1	ENSG00000249746.1	0	0
lincRNA	AC099509.2	ENSG00000250158.1	0	0
lincRNA	LIX1-AS1	ENSG00000251513.2	0	0
lincRNA	AC008883.2	ENSG00000251054.1	0	0
lincRNA	LINC02234	ENSG00000248202.1	0	0
lincRNA	LINC01846	ENSG00000248901.1	0	0
lincRNA	LINC02062	ENSG00000248489.1	0	0
lincRNA	LINC02113	ENSG00000249515.1	0	0
lincRNA	AC113385.1	ENSG00000249787.1	0	0
lincRNA	AC027315.1	ENSG00000250806.2	0	0
lincRNA	AC008948.1	ENSG00000248261.1	0	0
lincRNA	LINC00492	ENSG00000250958.1	0	0
lincRNA	AC099487.1	ENSG00000247402.2	0	0
lincRNA	AC010406.1	ENSG00000250567.1	0	0
lincRNA	LINC02115	ENSG00000248757.2	0	0
lincRNA	AC099520.1	ENSG00000251574.6	0	0
lincRNA	AC099520.2	ENSG00000253776.1	0	0
lincRNA	AC091987.1	ENSG00000253584.1	0	0
lincRNA	LINC01950	ENSG00000251027.1	0	0
lincRNA	AC024587.2	ENSG00000249959.5	0	0
lincRNA	AC091917.3	ENSG00000251367.1	0	0
lincRNA	AC091917.2	ENSG00000248876.1	0	0
lincRNA	AC091917.1	ENSG00000248440.1	0	0
lincRNA	LINC01848	ENSG00000254106.1	0	0
lincRNA	AC010395.1	ENSG00000248428.1	0	0
lincRNA	LINC02200	ENSG00000250358.5	0	0
lincRNA	LINC01957	ENSG00000251542.1	0	0
lincRNA	AC094104.2	ENSG00000251132.1	0	0
lincRNA	AC094104.1	ENSG00000250242.1	0	0
lincRNA	AC008549.2	ENSG00000249021.1	0	0
lincRNA	AC008549.1	ENSG00000248709.2	0	0
lincRNA	AC018752.1	ENSG00000250015.1	0	0
lincRNA	SEMA6A-	ENSG00000249167.1	0	0
lincRNA	LINC02214	ENSG00000251311.1	0	0
lincRNA	AC010267.1	ENSG00000249150.1	0	0
lincRNA	AC093534.2	ENSG00000250949.1	0	0

lincRNA	AC093295.1	ENSG00000285809.1	0	0
lincRNA	LINC02147	ENSG00000249797.1	0	0
lincRNA	LINC02148	ENSG00000250427.1	0	0
lincRNA	LINC02208	ENSG00000250891.1	0	0
lincRNA	LINC02216	ENSG00000249551.1	0	0
lincRNA	LINC02215	ENSG00000249128.1	0	0
lincRNA	AC008574.1	ENSG00000251293.1	0	0
lincRNA	AC113418.1	ENSG00000261036.1	0	0
lincRNA	AC008568.1	ENSG00000229855.8	0	0
lincRNA	AC113349.1	ENSG00000249621.1	0	0
lincRNA	LINC02201	ENSG00000251538.6	0	0
lincRNA	AC016556.1	ENSG00000251421.2	0	0
lincRNA	AC025465.2	ENSG00000248600.1	0	0
lincRNA	AC025465.4	ENSG00000251214.1	0	0
lincRNA	AC025465.3	ENSG00000250438.1	0	0
lincRNA	AC025465.1	ENSG00000248296.1	0	0
lincRNA	LINC02240	ENSG00000260192.2	0	0
lincRNA	AC109464.1	ENSG00000248443.1	0	0
lincRNA	AC109464.2	ENSG00000249261.1	0	0
lincRNA	AC109471.1	ENSG00000250402.2	0	0
lincRNA	AC010235.1	ENSG00000248752.1	0	0
lincRNA	LINC02039	ENSG00000248107.1	0	0
lincRNA	AC010424.2	ENSG00000271766.1	0	0
lincRNA	AC010424.3	ENSG00000282925.1	0	0
lincRNA	AC068658.1	ENSG00000250603.1	0	0
lincRNA	AC063976.1	ENSG00000224015.1	0	0
lincRNA	AC004775.1	ENSG00000272203.1	0	0
lincRNA	AC010240.3	ENSG00000272023.1	0	0
lincRNA	AC113410.2	ENSG00000251616.1	0	0
lincRNA	WSPAR	ENSG00000249073.1	0	0
lincRNA	AC005355.1	ENSG00000250994.1	0	0
lincRNA	LINC01843	ENSG00000251169.2	0	0
lincRNA	C5orf66-AS1	ENSG00000249082.2	0	0
lincRNA	AC008406.2	ENSG00000248753.1	0	0
lincRNA	AC008406.1	ENSG00000248482.1	0	0
lincRNA	C5orf66-AS2	ENSG00000249647.2	0	0
lincRNA	LINC01959	ENSG00000251045.1	0	0
lincRNA	AC002428.2	ENSG00000254239.1	0	0
lincRNA	AC002428.1	ENSG00000253927.1	0	0
lincRNA	AC063980.1	ENSG00000279240.1	0	0
lincRNA	AC112178.1	ENSG00000249803.5	0	0
lincRNA	AC109439.2	ENSG00000250284.2	0	0
lincRNA	AC106791.2	ENSG00000270697.1	0	0
lincRNA	AC113382.2	ENSG00000250260.1	0	0

lincRNA	AC113361.1	ENSG00000272255.1	0	0
lincRNA	CXXC5-AS1	ENSG00000250635.1	0	0
lincRNA	AC008667.1	ENSG00000249526.1	0	0
lincRNA	PSD2-AS1	ENSG00000249131.1	0	0
lincRNA	AC008667.3	ENSG00000251387.1	0	0
lincRNA	AC008667.4	ENSG00000271792.1	0	0
lincRNA	AC244517.9	ENSG00000279558.1	0	0
lincRNA	AC244517.12	ENSG00000280336.1	0	0
lincRNA	AC005753.1	ENSG00000278925.1	0	0
lincRNA	LINC01844	ENSG00000236714.1	0	2
lincRNA	AC016598.1	ENSG00000249429.1	0	0
lincRNA	AC016598.2	ENSG00000251205.2	0	1
lincRNA	AC109441.1	ENSG00000285605.1	0	0
lincRNA	AC132803.1	ENSG00000251031.1	0	0
lincRNA	AC137770.1	ENSG00000250842.1	0	0
lincRNA	AC008700.1	ENSG00000248125.1	0	0
lincRNA	AC011396.1	ENSG00000248842.1	0	0
lincRNA	SH3TC2-DT	ENSG00000250072.5	0	0
lincRNA	AC131025.2	ENSG00000275871.1	0	0
lincRNA	NDST1-AS1	ENSG00000254333.1	0	0
lincRNA	AC008641.1	ENSG00000254298.1	0	0
lincRNA	AC091982.3	ENSG00000275765.5	0	0
lincRNA	LINC01470	ENSG00000249484.8	0	0
lincRNA	LINC01861	ENSG00000251183.1	0	0
lincRNA	AC091962.1	ENSG00000261382.1	0	0
lincRNA	AC026688.2	ENSG00000254293.1	0	0
lincRNA	AC025437.5	ENSG00000253792.1	0	0
lincRNA	AC025437.4	ENSG00000253673.1	0	0
lincRNA	AC025437.3	ENSG00000253449.1	0	0
lincRNA	AC025437.2	ENSG00000253424.1	0	0
lincRNA	AC025437.1	ENSG00000253134.1	0	0
lincRNA	LINC02227	ENSG00000276778.1	0	0
lincRNA	AC091979.1	ENSG00000254350.1	0	0
lincRNA	AC091979.2	ENSG00000272085.1	0	0
lincRNA	AC091939.1	ENSG00000254135.2	0	0
lincRNA	LINC01845	ENSG00000253298.1	0	0
lincRNA	LINC02159	ENSG00000253417.5	0	0
lincRNA	LINC01202	ENSG00000280776.1	0	0
lincRNA	AC010291.1	ENSG00000285590.1	0	0
lincRNA	AC008662.1	ENSG00000253331.1	0	0
lincRNA	AC008662.2	ENSG00000253538.1	0	0
lincRNA	LINC02143	ENSG00000253236.1	0	0
lincRNA	AC091973.1	ENSG00000285549.1	0	0
lincRNA	LINC01938	ENSG00000254066.1	0	0

lincRNA	AC008415.1	ENSG00000253693.1	0	0
lincRNA	AC114321.1	ENSG00000254171.1	0	0
lincRNA	LINC01947	ENSG00000254130.1	0	0
lincRNA	AC091819.2	ENSG00000253713.1	0	0
lincRNA	AC091819.1	ENSG00000253469.1	0	0
lincRNA	AC034199.1	ENSG00000250274.1	0	0
lincRNA	LINC01366	ENSG00000235172.7	0	0
lincRNA	AC022440.1	ENSG00000285914.1	0	0
lincRNA	AC011410.1	ENSG00000253110.1	0	0
lincRNA	LINC01944	ENSG00000254299.1	0	0
lincRNA	AC110011.1	ENSG00000253628.1	0	0
lincRNA	AC008429.3	ENSG00000254295.1	0	0
lincRNA	AC016573.1	ENSG00000253968.1	0	0
lincRNA	AC008632.1	ENSG00000253141.1	0	0
lincRNA	AC008663.1	ENSG00000253768.1	0	0
lincRNA	AC008663.3	ENSG00000254164.1	0	0
lincRNA	LINC01863	ENSG00000253959.1	0	0
lincRNA	LINC01942	ENSG00000253428.1	0	0
lincRNA	LINC01484	ENSG00000253686.1	0	0
lincRNA	LINC01485	ENSG00000254211.5	0	0
lincRNA	AC025752.1	ENSG00000249722.1	0	0
lincRNA	LINC01411	ENSG00000249306.5	0	1
lincRNA	AC108112.1	ENSG00000250256.1	0	0
lincRNA	AC113346.2	ENSG00000251670.1	0	0
lincRNA	AC113346.1	ENSG00000251144.1	0	0
lincRNA	LINC01951	ENSG00000204754.3	0	0
lincRNA	AC008413.2	ENSG00000249403.1	0	0
lincRNA	AC139491.4	ENSG00000251458.1	0	0
lincRNA	AC113391.1	ENSG00000248484.1	0	0
lincRNA	AC113391.2	ENSG00000251446.1	0	0
lincRNA	LINC01574	ENSG00000248859.2	0	0
lincRNA	AC140125.1	ENSG00000248943.1	0	0
lincRNA	AC106795.3	ENSG00000250101.1	0	0
lincRNA	AC106795.5	ENSG00000280207.1	0	1
lincRNA	AC104117.5	ENSG00000272431.1	0	0
lincRNA	AC109479.3	ENSG00000253979.1	0	0
lincRNA	AC136628.1	ENSG00000250674.1	0	0
lincRNA	AC136628.4	ENSG00000253652.1	0	0
lincRNA	AC034213.1	ENSG00000250509.1	0	0
lincRNA	LINC02222	ENSG00000248717.1	0	0
lincRNA	AC008443.2	ENSG00000248103.1	0	0
lincRNA	AC138035.1	ENSG00000238035.8	0	0
lincRNA	AC138035.2	ENSG00000250765.6	0	0
lincRNA	AL035696.3	ENSG00000263667.1	0	0

lincRNA	AL035696.4	ENSG00000285578.1	0	0
lincRNA	AL035696.1	ENSG00000205653.2	0	0
lincRNA	AL357054.4	ENSG00000272463.1	0	3
lincRNA	AL357054.2	ENSG00000271727.1	0	0
lincRNA	AL357054.3	ENSG00000271911.1	0	1
lincRNA	AL392183.1	ENSG00000272485.1	0	0
lincRNA	AL356130.1	ENSG00000276687.1	0	0
lincRNA	AL356130.2	ENSG00000278343.1	0	0
lincRNA	AL033381.2	ENSG00000270937.1	0	1
lincRNA	AL033381.1	ENSG00000176515.1	0	0
lincRNA	FOXCUT	ENSG00000280916.1	0	0
lincRNA	LINC02521	ENSG00000228718.2	0	0
lincRNA	LINC01600	ENSG00000164385.8	0	0
lincRNA	AL133351.2	ENSG00000228365.1	0	0
lincRNA	LINC01011	ENSG00000244041.7	0	1
lincRNA	AL031963.2	ENSG00000229282.2	0	2
lincRNA	LINC02525	ENSG00000230269.6	0	0
lincRNA	AL138881.1	ENSG00000228793.1	0	0
lincRNA	AL391422.2	ENSG00000233068.3	0	0
lincRNA	AL136309.2	ENSG00000234817.2	0	2
lincRNA	AL136309.3	ENSG00000237716.1	0	0
lincRNA	AL162718.1	ENSG00000285424.1	0	0
lincRNA	AL159166.1	ENSG00000231811.2	0	0
lincRNA	AL162718.2	ENSG00000285694.1	0	0
lincRNA	LINC02533	ENSG00000260239.1	0	0
lincRNA	AL034376.1	ENSG00000260673.1	0	0
lincRNA	KU-MEL-3	ENSG00000205444.3	0	0
lincRNA	AL136307.1	ENSG00000233064.2	0	0
lincRNA	AL162719.1	ENSG00000285717.1	0	0
lincRNA	AL136361.1	ENSG00000224532.2	0	0
lincRNA	AL158817.1	ENSG00000223342.2	0	0
lincRNA	AL139390.1	ENSG00000203498.2	0	0
lincRNA	AL139095.5	ENSG00000285771.1	0	0
lincRNA	AL359378.1	ENSG00000234763.1	0	0
lincRNA	AL137220.1	ENSG00000225775.1	0	0
lincRNA	AL138885.2	ENSG00000228478.1	0	0
lincRNA	AL138885.3	ENSG00000285759.1	0	0
lincRNA	LINC00518	ENSG00000183674.11	0	0
lincRNA	MIR5689HG	ENSG00000229401.1	0	0
lincRNA	LINC02522	ENSG00000231056.1	0	0
lincRNA	AL024498.1	ENSG00000272097.1	0	0
lincRNA	AL445430.1	ENSG00000233656.1	0	0
lincRNA	AL357518.1	ENSG00000271897.1	0	0
lincRNA	AL022724.1	ENSG00000234427.1	0	0

lincRNA	AL157373.2	ENSG00000229896.2	0	0
lincRNA	LINC02530	ENSG00000234015.1	0	0
lincRNA	AL008729.2	ENSG00000272379.1	0	1
lincRNA	AL023583.1	ENSG00000272209.1	0	3
lincRNA	AL022396.1	ENSG00000233981.1	0	0
lincRNA	AL133259.1	ENSG00000285639.1	0	0
lincRNA	AL353152.1	ENSG00000230631.1	0	0
lincRNA	LINC01108	ENSG00000226673.2	0	0
lincRNA	AL080313.2	ENSG00000237346.1	0	0
lincRNA	AL080313.1	ENSG00000234540.1	0	0
lincRNA	AL109914.1	ENSG00000229646.2	0	0
lincRNA	AL138720.1	ENSG00000234261.3	0	0
lincRNA	LINC02543	ENSG00000232947.1	0	0
lincRNA	MIR548A1H	ENSG00000283408.1	0	0
lincRNA	AL357052.1	ENSG00000231662.1	0	0
lincRNA	AL031767.1	ENSG00000227089.2	0	0
lincRNA	LINC00581	ENSG00000280989.1	0	0
lincRNA	AL512380.1	ENSG00000231754.2	0	0
lincRNA	AL359694.2	ENSG00000261568.1	0	0
lincRNA	AL033539.1	ENSG00000228772.2	0	0
lincRNA	AL035401.1	ENSG00000233358.2	0	0
lincRNA	AL139231.1	ENSG00000235743.1	0	0
lincRNA	AL133264.2	ENSG00000224164.1	0	0
lincRNA	AL133268.2	ENSG00000223623.1	0	0
lincRNA	U91328.3	ENSG00000272810.1	0	0
lincRNA	AL121936.1	ENSG00000284607.1	0	1
lincRNA	AL513548.3	ENSG00000275846.1	0	0
lincRNA	AL513548.4	ENSG00000285571.1	0	0
lincRNA	AL513548.1	ENSG00000261584.1	0	0
lincRNA	LINC00240	ENSG00000224843.6	0	0
lincRNA	AL133255.1	ENSG00000272312.1	0	0
lincRNA	AL021918.3	ENSG00000285703.1	0	0
lincRNA	LINC01012	ENSG00000281706.2	0	0
lincRNA	AL358933.1	ENSG00000261839.1	0	0
lincRNA	LINC00533	ENSG00000235570.1	0	0
lincRNA	AL662890.1	ENSG00000225173.1	0	0
lincRNA	LINC01623	ENSG00000225595.2	0	0
lincRNA	AL645937.2	ENSG00000227206.1	0	0
lincRNA	AL662860.1	ENSG00000229274.1	0	0
lincRNA	AL645939.4	ENSG00000272236.1	0	0
lincRNA	AL645939.5	ENSG00000285761.1	0	0
lincRNA	HCG9	ENSG00000204625.10	0	0
lincRNA	HCG20	ENSG00000228022.5	0	0
lincRNA	LINC02570	ENSG00000237923.1	0	0

lincRNA	HCG22	ENSG00000228789.7	0	0
lincRNA	AL662844.3	ENSG00000271821.1	0	0
lincRNA	LINC02571	ENSG00000256166.1	0	0
lincRNA	AL671883.3	ENSG00000285647.1	0	0
lincRNA	LINC01149	ENSG00000230174.1	0	0
lincRNA	AL645940.1	ENSG00000272217.1	0	0
lincRNA	LINC00336	ENSG00000197251.3	0	0
lincRNA	LINC01016	ENSG00000249346.6	0	0
lincRNA	AL138889.1	ENSG00000233183.2	0	0
lincRNA	Z97832.2	ENSG00000272374.1	0	0
lincRNA	AL033519.3	ENSG00000228559.1	0	0
lincRNA	PANDAR	ENSG00000281450.1	0	0
lincRNA	Z85996.2	ENSG00000285888.1	0	0
lincRNA	AL122034.1	ENSG00000232598.2	0	0
lincRNA	LINC02520	ENSG00000204110.6	0	0
lincRNA	AL353597.1	ENSG00000227920.2	0	0
lincRNA	AL391415.1	ENSG00000236355.2	0	0
lincRNA	AL139275.2	ENSG00000227131.1	0	0
lincRNA	LINC00951	ENSG00000204092.2	0	0
lincRNA	AL139275.1	ENSG00000226070.1	0	0
lincRNA	AL583854.1	ENSG00000237947.1	0	0
lincRNA	LINC01276	ENSG00000226917.5	0	0
lincRNA	AL365205.3	ENSG00000269387.1	0	0
lincRNA	AL160163.1	ENSG00000227516.1	0	0
lincRNA	AL513008.1	ENSG00000223946.1	0	0
lincRNA	AL136131.2	ENSG00000236961.1	0	0
lincRNA	AL157371.2	ENSG00000283573.1	0	0
lincRNA	LINC02537	ENSG00000283215.2	0	0
lincRNA	AL157371.1	ENSG00000223469.1	0	0
lincRNA	AL109615.2	ENSG00000231881.1	0	0
lincRNA	AL136140.1	ENSG00000237530.1	0	0
lincRNA	RUNX2-AS1	ENSG00000261080.1	0	0
lincRNA	AL161618.1	ENSG00000236164.1	0	0
lincRNA	AL356421.2	ENSG00000226594.1	0	0
lincRNA	AL359458.1	ENSG00000226707.1	0	0
lincRNA	AL138826.1	ENSG00000226733.3	0	0
lincRNA	AL360175.1	ENSG00000233470.1	0	0
lincRNA	AL135908.1	ENSG00000230472.5	0	0
lincRNA	LINCMD1	ENSG00000225613.2	0	0
lincRNA	AL033397.1	ENSG00000231683.6	0	0
lincRNA	AL033384.1	ENSG00000236740.6	0	0
lincRNA	FO680682.1	ENSG00000266579.1	0	0
lincRNA	AL021368.3	ENSG00000272541.1	0	0
lincRNA	AL021368.1	ENSG00000271761.1	0	0

lincRNA	AL590227.1	ENSG00000275773.1	0	0
lincRNA	AL713998.1	ENSG00000227706.3	0	0
lincRNA	AL646090.1	ENSG00000223504.1	0	0
lincRNA	LINC02549	ENSG00000226497.1	0	0
lincRNA	AL365226.2	ENSG00000237643.1	0	1
lincRNA	AL583856.2	ENSG00000271967.1	0	0
lincRNA	AL136164.2	ENSG00000269966.1	0	0
lincRNA	AL136164.1	ENSG00000230597.1	0	0
lincRNA	LINC01626	ENSG00000223765.2	0	0
lincRNA	AL035467.2	ENSG00000285963.1	0	0
lincRNA	AL357507.1	ENSG00000223786.1	0	0
lincRNA	AL356277.3	ENSG00000272243.5	0	0
lincRNA	AL356277.2	ENSG00000224583.1	0	0
lincRNA	AL445465.2	ENSG00000238156.1	0	0
lincRNA	LINC02540	ENSG00000236635.2	0	0
lincRNA	AL591030.1	ENSG00000280511.1	0	0
lincRNA	AL590426.2	ENSG00000271945.1	0	0
lincRNA	AL121718.1	ENSG00000230309.1	0	0
lincRNA	AL450327.1	ENSG00000229495.1	0	0
lincRNA	AL078601.2	ENSG00000231533.1	0	0
lincRNA	LINC01621	ENSG00000235357.1	0	0
lincRNA	AL132875.3	ENSG00000285586.1	0	0
lincRNA	AL132875.2	ENSG00000233835.1	0	0
lincRNA	AL359693.1	ENSG00000277797.1	0	0
lincRNA	LINC01526	ENSG00000224995.1	0	0
lincRNA	AL360157.1	ENSG00000260574.1	0	0
lincRNA	AL136972.1	ENSG00000237874.2	0	0
lincRNA	AL034347.1	ENSG00000228679.1	0	0
lincRNA	AL161621.1	ENSG00000230852.2	0	0
lincRNA	LINC02535	ENSG00000234155.1	0	0
lincRNA	AL139042.1	ENSG00000234426.2	0	0
lincRNA	AL132996.1	ENSG00000260271.2	0	0
lincRNA	AL080284.1	ENSG00000224605.1	0	0
lincRNA	AL590814.1	ENSG00000285961.1	0	0
lincRNA	AL133457.1	ENSG00000261038.1	0	0
lincRNA	LINC02531	ENSG00000220908.2	0	0
lincRNA	AL138731.1	ENSG00000235099.1	0	0
lincRNA	AL391336.1	ENSG00000229600.1	0	0
lincRNA	AL157378.1	ENSG00000231143.1	0	0
lincRNA	AL589740.1	ENSG00000271860.6	0	0
lincRNA	AL080316.1	ENSG00000226207.1	0	0
lincRNA	AL590727.1	ENSG00000236920.2	0	0
lincRNA	LIN28B-AS1	ENSG00000203809.6	0	0
lincRNA	Z97206.2	ENSG00000237567.1	0	0

lincRNA	AL591518.1	ENSG00000284999.1	0	0
lincRNA	LINC02526	ENSG00000229654.1	0	0
lincRNA	LINC02532	ENSG00000235142.8	0	0
lincRNA	Z98200.1	ENSG00000271734.1	0	0
lincRNA	OSTM1-AS1	ENSG00000225174.1	0	0
lincRNA	Z98742.1	ENSG00000230248.1	0	0
lincRNA	LINC00222	ENSG00000203801.8	0	0
lincRNA	AL512303.1	ENSG00000232311.1	0	0
lincRNA	LINC02527	ENSG00000227012.2	0	0
lincRNA	Z99289.2	ENSG00000270661.1	0	0
lincRNA	AL357514.1	ENSG00000232299.1	0	0
lincRNA	AL513123.1	ENSG00000236347.1	0	0
lincRNA	FO393415.2	ENSG00000231912.2	0	0
lincRNA	LINC01268	ENSG00000227502.2	0	1
lincRNA	AL138830.1	ENSG00000226079.1	0	0
lincRNA	LINC02534	ENSG00000228777.1	0	0
lincRNA	AL450332.1	ENSG00000226409.2	0	0
lincRNA	LINC02523	ENSG00000224506.2	0	0
lincRNA	LINC02536	ENSG00000281248.1	0	0
lincRNA	AL356124.1	ENSG00000226149.5	0	0
lincRNA	AL583803.1	ENSG00000229923.2	0	0
lincRNA	AL117378.1	ENSG00000236673.5	0	0
lincRNA	AL021408.1	ENSG00000236166.1	0	0
lincRNA	LINC01013	ENSG00000228495.1	0	2
lincRNA	LINC00326	ENSG00000231023.6	0	0
lincRNA	LINC01312	ENSG00000223586.5	0	0
lincRNA	AL078590.2	ENSG00000231971.5	0	2
lincRNA	LINC01010	ENSG00000236700.5	0	0
lincRNA	AL078590.3	ENSG00000232310.7	0	0
lincRNA	AL078590.1	ENSG00000229722.1	0	0
lincRNA	AL596188.1	ENSG00000227723.1	0	0
lincRNA	AL121970.1	ENSG00000236389.1	0	0
lincRNA	LINC00271	ENSG00000231028.8	0	0
lincRNA	AL035604.1	ENSG00000233534.2	0	0
lincRNA	AL133319.1	ENSG00000236378.1	0	0
lincRNA	NHEG1	ENSG00000225391.5	0	0
lincRNA	AL356234.2	ENSG00000230533.3	0	0
lincRNA	AL356234.3	ENSG00000283265.1	0	0
lincRNA	LINC02539	ENSG00000234956.6	0	0
lincRNA	AL591468.1	ENSG00000226004.1	0	0
lincRNA	LINC02528	ENSG00000229922.5	0	0
lincRNA	LINC01625	ENSG00000238099.2	0	0
lincRNA	FILNC1	ENSG00000231426.6	0	0
lincRNA	AL050338.1	ENSG00000225148.1	0	0

lincRNA	AL357146.1	ENSG00000236013.6	0	0
lincRNA	AL035446.2	ENSG00000285875.1	0	0
lincRNA	AL355596.1	ENSG00000259828.1	0	0
lincRNA	AL360007.1	ENSG00000237494.1	0	0
lincRNA	AL359313.1	ENSG00000236366.2	0	0
lincRNA	AL023584.2	ENSG00000237851.1	0	0
lincRNA	AL355304.1	ENSG00000232618.1	0	0
lincRNA	AL023283.1	ENSG00000285598.1	0	0
lincRNA	AL023806.1	ENSG00000270638.1	0	0
lincRNA	AL359547.1	ENSG00000237468.2	0	0
lincRNA	AL138916.1	ENSG00000227748.1	0	0
lincRNA	AL138916.2	ENSG00000272397.1	0	0
lincRNA	LUADT1	ENSG00000196634.3	0	0
lincRNA	AL033504.1	ENSG00000227681.5	0	0
lincRNA	AL365271.1	ENSG00000226249.1	0	0
lincRNA	AL445123.1	ENSG00000234675.1	0	0
lincRNA	AL359382.1	ENSG00000230205.1	0	0
lincRNA	AL031056.1	ENSG00000228408.6	0	0
lincRNA	TAB2-AS1	ENSG00000225924.2	0	0
lincRNA	AL031056.2	ENSG00000283608.1	0	0
lincRNA	AL078581.2	ENSG00000235168.1	0	0
lincRNA	AL355497.2	ENSG00000235972.1	0	0
lincRNA	AL450344.2	ENSG00000226599.1	0	0
lincRNA	AL138733.2	ENSG00000231883.1	0	0
lincRNA	AL160162.1	ENSG00000224893.5	0	0
lincRNA	AL590867.1	ENSG00000213121.2	0	0
lincRNA	AL590867.3	ENSG00000237312.1	0	0
lincRNA	AL031773.1	ENSG00000232529.1	0	0
lincRNA	AL512658.2	ENSG00000285797.1	0	0
lincRNA	AL512658.1	ENSG00000227360.1	0	0
lincRNA	AL355297.3	ENSG00000271265.1	0	1
lincRNA	AL627422.2	ENSG00000285159.1	0	0
lincRNA	AL356417.3	ENSG00000285492.1	0	0
lincRNA	AL356417.1	ENSG00000224478.2	0	0
lincRNA	AL357832.1	ENSG00000231178.2	0	0
lincRNA	LINC02529	ENSG00000234777.2	0	0
lincRNA	SOD2-OT1	ENSG00000285427.1	0	0
lincRNA	AL035691.1	ENSG00000236823.1	0	0
lincRNA	AL162582.1	ENSG00000230234.1	0	0
lincRNA	AL109933.1	ENSG00000224477.5	0	0
lincRNA	AL391361.2	ENSG00000224371.6	0	0
lincRNA	AL391361.3	ENSG00000233342.1	0	0
lincRNA	AL137182.1	ENSG00000285553.1	0	0
lincRNA	DKFZp451B0	ENSG00000285564.1	0	0

lincRNA	AL031121.2	ENSG00000285726.1	0	0
lincRNA	CAHM	ENSG00000270419.1	0	2
lincRNA	AL445307.1	ENSG00000228692.2	0	0
lincRNA	AL137005.1	ENSG00000235538.1	0	0
lincRNA	AL137005.2	ENSG00000285858.1	0	0
lincRNA	Z97205.2	ENSG00000260422.1	0	0
lincRNA	Z97205.1	ENSG00000230627.1	0	0
lincRNA	AL358972.1	ENSG00000226739.1	0	0
lincRNA	LINC00602	ENSG00000281832.1	0	0
lincRNA	AL627443.1	ENSG00000231297.3	0	0
lincRNA	AL121956.1	ENSG00000233365.2	0	0
lincRNA	AL121956.4	ENSG00000280850.1	0	0
lincRNA	AL121935.1	ENSG00000284825.1	0	0
lincRNA	AL121935.2	ENSG00000284914.1	0	0
lincRNA	HPAT5	ENSG00000280707.2	0	0
lincRNA	AL353747.3	ENSG00000231720.1	0	0
lincRNA	AL353747.2	ENSG00000228648.1	0	0
lincRNA	LINC02538	ENSG00000272549.1	0	0
lincRNA	LINC02487	ENSG00000203688.5	0	0
lincRNA	LINC01558	ENSG00000146521.9	0	0
lincRNA	AL009178.2	ENSG00000269155.2	0	0
lincRNA	AFDN-DT	ENSG00000198221.8	0	0
lincRNA	KIF25-AS1	ENSG00000229921.6	0	0
lincRNA	AL611929.1	ENSG00000234768.1	0	0
lincRNA	AL606970.4	ENSG00000237987.1	0	0
lincRNA	AL606970.2	ENSG00000228361.2	0	0
lincRNA	AL606970.3	ENSG00000234647.2	0	0
lincRNA	AL138918.1	ENSG00000260771.1	0	0
lincRNA	AL136099.1	ENSG00000235815.1	0	0
lincRNA	AL109924.1	ENSG00000225879.1	0	0
lincRNA	AL109924.5	ENSG00000285917.1	0	0
lincRNA	AL109924.3	ENSG00000234519.1	0	0
lincRNA	LINC02544	ENSG00000261039.2	0	1
lincRNA	AL049612.1	ENSG00000236173.1	0	0
lincRNA	AL596442.2	ENSG00000273100.1	0	0
lincRNA	AL109910.2	ENSG00000271820.1	0	0
lincRNA	LINC01624	ENSG00000227508.6	0	0
lincRNA	AL078605.1	ENSG00000271234.1	0	0
lincRNA	AL731661.2	ENSG00000234986.1	0	0
lincRNA	AL731661.1	ENSG00000213863.2	0	0
lincRNA	AL731684.2	ENSG00000230423.1	0	0
lincRNA	AL731684.1	ENSG00000225532.1	0	0
lincRNA	AC215522.3	ENSG00000283061.1	0	0
lincRNA	AC093627.1	ENSG00000232325.4	0	0

lincRNA	AC093627.4	ENSG00000240859.2	0	6
lincRNA	AC093627.7	ENSG00000261795.1	0	0
lincRNA	AC093627.2	ENSG00000239715.1	0	0
lincRNA	AC147651.2	ENSG00000234471.1	0	0
lincRNA	AC073094.1	ENSG00000233082.1	0	0
lincRNA	AC074389.2	ENSG00000231476.1	0	0
lincRNA	AC074389.3	ENSG00000234977.1	0	0
lincRNA	AC074389.1	ENSG00000205971.3	0	0
lincRNA	ELFN1-AS1	ENSG00000236081.1	0	0
lincRNA	AC024028.1	ENSG00000228334.1	0	0
lincRNA	AC073316.1	ENSG00000217455.8	0	0
lincRNA	AC073316.2	ENSG00000231892.1	0	0
lincRNA	RBAKDN	ENSG00000273313.1	0	0
lincRNA	AC092171.2	ENSG00000230733.2	0	0
lincRNA	AC004895.1	ENSG00000231704.5	0	0
lincRNA	AC004982.1	ENSG00000272732.1	0	0
lincRNA	AC007128.2	ENSG00000272328.1	0	0
lincRNA	AC004852.2	ENSG00000278254.4	0	0
lincRNA	AC060834.2	ENSG00000234710.1	0	0
lincRNA	AC004936.1	ENSG00000235431.1	0	0
lincRNA	AC004949.1	ENSG00000283117.1	0	0
lincRNA	AC004415.1	ENSG00000236414.1	0	0
lincRNA	AC007029.1	ENSG00000271185.1	0	0
lincRNA	AC011287.1	ENSG00000229618.2	0	0
lincRNA	AC011287.2	ENSG00000237713.1	0	0
lincRNA	AC005019.2	ENSG00000224330.1	0	0
lincRNA	AC006458.1	ENSG00000227489.1	0	0
lincRNA	LINC02587	ENSG00000229108.1	0	0
lincRNA	AC005550.1	ENSG00000225974.1	0	0
lincRNA	AC006041.1	ENSG00000229379.1	0	0
lincRNA	AC005014.3	ENSG00000272537.1	0	1
lincRNA	KCCAT333	ENSG00000236318.2	0	0
lincRNA	AC019117.1	ENSG00000236039.2	0	0
lincRNA	AC006482.1	ENSG00000228675.1	0	0
lincRNA	AC007091.1	ENSG00000223838.1	0	0
lincRNA	AC004543.1	ENSG00000237921.2	0	0
lincRNA	AC007001.1	ENSG00000267055.1	0	0
lincRNA	AC099342.1	ENSG00000226097.5	0	0
lincRNA	LINC01162	ENSG00000232790.2	0	0
lincRNA	AC080068.1	ENSG00000285592.1	0	0
lincRNA	KLHL7-DT	ENSG00000230658.1	0	0
lincRNA	AC005082.1	ENSG00000226816.3	0	0
lincRNA	AC003087.1	ENSG00000227930.1	0	0
lincRNA	AC004129.3	ENSG00000285716.1	0	0

lincRNA	AC003985.1	ENSG00000233824.1	0	0
lincRNA	AC003985.2	ENSG00000260951.1	0	0
lincRNA	AC005100.1	ENSG00000285960.1	0	0
lincRNA	AC091705.1	ENSG00000227386.1	0	0
lincRNA	AC005165.1	ENSG00000223561.6	0	0
lincRNA	AC010677.2	ENSG00000285860.1	0	0
lincRNA	AC004540.1	ENSG00000214870.8	0	0
lincRNA	KIAA0087	ENSG00000122548.5	0	0
lincRNA	AC004947.1	ENSG00000233760.1	0	0
lincRNA	C7orf71	ENSG00000222004.7	0	0
lincRNA	AC004080.2	ENSG00000253508.1	0	0
lincRNA	AC004080.1	ENSG00000253308.2	0	0
lincRNA	AC004009.1	ENSG00000224322.1	0	0
lincRNA	AC005013.1	ENSG00000228421.2	0	0
lincRNA	AC007036.1	ENSG00000227017.1	0	0
lincRNA	AC005154.4	ENSG00000264520.1	0	0
lincRNA	AC005154.3	ENSG00000263683.1	0	0
lincRNA	AC005090.1	ENSG00000231418.1	0	0
lincRNA	AC018637.1	ENSG00000284839.1	0	0
lincRNA	AC018648.1	ENSG00000272905.1	0	2
lincRNA	AC008080.1	ENSG00000173862.3	0	0
lincRNA	AC008080.4	ENSG00000236494.1	0	0
lincRNA	AC008080.3	ENSG00000233219.1	0	0
lincRNA	AC009262.1	ENSG00000236212.1	0	0
lincRNA	AC009531.1	ENSG00000226063.1	0	0
lincRNA	AC007652.1	ENSG00000235464.1	0	0
lincRNA	AC018647.1	ENSG00000227544.8	0	0
lincRNA	SEPT7-AS1	ENSG00000228878.7	0	0
lincRNA	AC083864.2	ENSG00000232930.1	0	0
lincRNA	AC083864.4	ENSG00000285573.1	0	0
lincRNA	AC083864.1	ENSG00000205745.2	0	0
lincRNA	AC083864.3	ENSG00000261184.2	0	0
lincRNA	AC007349.4	ENSG00000272984.1	0	0
lincRNA	AC007349.1	ENSG00000229424.1	0	0
lincRNA	AC007349.3	ENSG00000235728.1	0	0
lincRNA	LINC00265	ENSG00000188185.11	0	0
lincRNA	LINC01450	ENSG00000232458.1	0	0
lincRNA	LINC01449	ENSG00000224017.1	0	0
lincRNA	LINC01448	ENSG00000238284.1	0	0
lincRNA	AC010132.1	ENSG00000231539.1	0	0
lincRNA	AC073325.1	ENSG00000225537.1	0	0
lincRNA	AC073115.2	ENSG00000237471.1	0	0
lincRNA	AC073115.1	ENSG00000229628.1	0	0
lincRNA	AC023669.1	ENSG00000229459.1	0	0

lincRNA	AC023669.2	ENSG00000237760.1	0	0
lincRNA	AC004869.2	ENSG00000240355.1	0	0
lincRNA	AC004869.1	ENSG00000230680.2	0	0
lincRNA	AC004870.4	ENSG00000232072.1	0	0
lincRNA	AC004870.3	ENSG00000229192.6	0	0
lincRNA	C7orf65	ENSG00000221845.4	0	0
lincRNA	AC091770.1	ENSG00000228173.1	0	0
lincRNA	AC091770.2	ENSG00000285536.1	0	0
lincRNA	AC091730.1	ENSG00000234686.1	0	0
lincRNA	AC020743.2	ENSG00000231681.2	0	0
lincRNA	AC020743.3	ENSG00000235620.1	0	0
lincRNA	AC004920.1	ENSG00000236046.1	0	0
lincRNA	AC005999.2	ENSG00000285741.1	0	0
lincRNA	AC005999.1	ENSG00000229403.1	0	0
lincRNA	AC079763.1	ENSG00000233960.1	0	0
lincRNA	AC009468.2	ENSG00000234105.1	0	0
lincRNA	AC009468.1	ENSG00000228627.1	0	0
lincRNA	LINC01446	ENSG00000205628.2	0	0
lincRNA	AC073521.1	ENSG00000238131.1	0	0
lincRNA	AC092848.1	ENSG00000280920.1	0	0
lincRNA	LINC01445	ENSG00000231427.5	0	0
lincRNA	AC011228.1	ENSG00000228735.1	0	0
lincRNA	ELDR	ENSG00000280890.1	0	0
lincRNA	AC099681.3	ENSG00000233977.1	0	0
lincRNA	AC099681.2	ENSG00000231394.1	0	0
lincRNA	AC006970.3	ENSG00000285670.1	0	0
lincRNA	AC092447.4	ENSG00000225488.1	0	0
lincRNA	AC092447.8	ENSG00000261275.1	0	0
lincRNA	AC092447.5	ENSG00000233288.1	0	0
lincRNA	AC118758.3	ENSG00000279072.1	0	0
lincRNA	AC099654.1	ENSG00000234089.1	0	0
lincRNA	AC237221.1	ENSG00000260653.5	0	0
lincRNA	AC069285.1	ENSG00000227148.1	0	0
lincRNA	AC006455.1	ENSG00000226587.1	0	0
lincRNA	AC006455.4	ENSG00000229301.1	0	0
lincRNA	AC073188.3	ENSG00000230600.1	0	0
lincRNA	AC073188.4	ENSG00000232817.1	0	0
lincRNA	AC073188.2	ENSG00000227305.1	0	0
lincRNA	AC073188.5	ENSG00000234387.1	0	0
lincRNA	AC092634.5	ENSG00000236299.7	0	0
lincRNA	AC092634.3	ENSG00000226581.1	0	0
lincRNA	LINC01005	ENSG00000228564.2	0	0
lincRNA	AC091685.1	ENSG00000229881.1	0	0
lincRNA	AC104073.4	ENSG00000282381.1	0	0

lincRNA	AC114501.3	ENSG00000238124.1	0	0
lincRNA	AC092637.1	ENSG00000225209.1	0	0
lincRNA	AC006013.1	ENSG00000236531.2	0	0
lincRNA	AC093655.1	ENSG00000226829.1	0	0
lincRNA	AC104688.1	ENSG00000233689.1	0	0
lincRNA	AC069280.1	ENSG00000236839.1	0	0
lincRNA	AC092100.1	ENSG00000225718.1	0	0
lincRNA	AC006012.1	ENSG00000234215.2	0	0
lincRNA	AC211476.6	ENSG00000285886.1	0	0
lincRNA	AC099398.1	ENSG00000261467.2	0	0
lincRNA	AC006972.1	ENSG00000285666.1	0	0
lincRNA	AC114737.1	ENSG00000225703.1	0	0
lincRNA	AC004921.1	ENSG00000273341.1	0	0
lincRNA	AC003988.1	ENSG00000234223.2	0	0
lincRNA	AC005008.2	ENSG00000237896.6	0	0
lincRNA	AC003984.1	ENSG00000235139.2	0	0
lincRNA	AC074183.1	ENSG00000232019.1	0	0
lincRNA	LINC00972	ENSG00000225128.1	0	0
lincRNA	AC004023.1	ENSG00000261462.1	0	0
lincRNA	AC002127.2	ENSG00000233420.1	0	0
lincRNA	AC002383.1	ENSG00000227863.2	0	0
lincRNA	AC004969.1	ENSG00000238358.2	0	0
lincRNA	AC002064.1	ENSG00000225498.1	0	0
lincRNA	AC079760.2	ENSG00000243144.6	0	0
lincRNA	AC079760.1	ENSG00000235450.1	0	0
lincRNA	AC000058.1	ENSG00000223665.1	0	0
lincRNA	AC003092.2	ENSG00000236938.1	0	0
lincRNA	AC004012.1	ENSG00000233942.1	0	0
lincRNA	AC069281.1	ENSG00000225807.1	0	0
lincRNA	LNCPRESS1	ENSG00000232301.2	0	0
lincRNA	LINC01007	ENSG00000233123.1	0	0
lincRNA	AC007384.1	ENSG00000237513.1	0	0
lincRNA	AC005487.1	ENSG00000225647.1	0	0
lincRNA	C7orf66	ENSG00000205174.4	0	0
lincRNA	AC004014.1	ENSG00000229603.1	0	0
lincRNA	AC002386.1	ENSG00000230941.1	0	0
lincRNA	AC073071.1	ENSG00000234273.1	0	0
lincRNA	AC092167.1	ENSG00000226965.1	0	0
lincRNA	AC002463.1	ENSG00000223646.1	0	0
lincRNA	LINC01392	ENSG00000233607.1	0	0
lincRNA	LINC01393	ENSG00000225535.6	0	0
lincRNA	AC092590.1	ENSG00000242072.1	0	0
lincRNA	AC093714.1	ENSG00000240973.1	0	0
lincRNA	AC093714.2	ENSG00000243345.1	0	0

lincRNA	AC003084.1	ENSG00000234826.1	0	0
lincRNA	LINC02476	ENSG00000225546.5	0	0
lincRNA	AC004872.1	ENSG00000233417.1	0	0
lincRNA	AC004946.1	ENSG00000233969.1	0	0
lincRNA	AC004875.1	ENSG00000227743.2	0	0
lincRNA	AC015983.2	ENSG00000234418.1	0	0
lincRNA	AC073311.1	ENSG00000226680.2	0	0
lincRNA	AC006148.2	ENSG00000243574.1	0	0
lincRNA	AC004930.1	ENSG00000241324.1	0	0
lincRNA	AC019155.3	ENSG00000224899.1	0	0
lincRNA	AC019155.2	ENSG00000219445.2	0	0
lincRNA	AC073486.1	ENSG00000227869.1	0	0
lincRNA	AC003975.1	ENSG00000197462.3	0	0
lincRNA	AC000124.1	ENSG00000226770.1	0	0
lincRNA	AC018635.2	ENSG00000272915.1	0	0
lincRNA	AC084864.1	ENSG00000242078.1	0	0
lincRNA	AC007938.1	ENSG00000259920.1	0	0
lincRNA	AC016831.6	ENSG00000285106.1	0	0
lincRNA	LINC00513	ENSG00000233559.1	0	1
lincRNA	AC058791.1	ENSG00000273319.1	0	1
lincRNA	AC009518.1	ENSG00000224865.7	0	0
lincRNA	FLJ40288	ENSG00000183470.9	0	1
lincRNA	AC008154.1	ENSG00000229177.1	0	0
lincRNA	AC078842.2	ENSG00000231114.1	0	0
lincRNA	AC078842.1	ENSG00000228031.3	0	0
lincRNA	AC022173.1	ENSG00000237243.1	0	0
lincRNA	AC083867.1	ENSG00000225559.1	0	0
lincRNA	AC006452.1	ENSG00000285904.1	0	0
lincRNA	AC005692.3	ENSG00000285841.1	0	0
lincRNA	AC005692.1	ENSG00000204990.3	0	0
lincRNA	AC073878.2	ENSG00000261797.1	0	0
lincRNA	AC245088.1	ENSG00000241881.1	0	0
lincRNA	TRBV11-2	ENSG00000241657.1	0	0
lincRNA	AC093673.2	ENSG00000272619.1	0	1
lincRNA	AC006007.1	ENSG00000230746.1	0	0
lincRNA	AC006974.1	ENSG00000283504.1	0	0
lincRNA	AC006974.2	ENSG00000283648.1	0	0
lincRNA	AC073314.1	ENSG00000261842.1	0	0
lincRNA	AC093458.1	ENSG00000273293.1	0	0
lincRNA	AC092681.3	ENSG00000273011.1	0	0
lincRNA	AC092681.2	ENSG00000260555.1	0	0
lincRNA	AC092666.1	ENSG00000241449.5	0	0
lincRNA	LINC00996	ENSG00000242258.1	0	0
lincRNA	LINC01287	ENSG00000234722.4	0	0

lincRNA	AC073236.1	ENSG00000285524.1	0	0
lincRNA	AC099552.1	ENSG00000217825.2	0	0
lincRNA	AC099552.3	ENSG00000228521.2	0	0
lincRNA	AC099552.2	ENSG00000225612.1	0	0
lincRNA	BLACE	ENSG00000204960.6	0	0
lincRNA	AC008060.1	ENSG00000218672.1	0	0
lincRNA	AC008060.3	ENSG00000236544.1	0	0
lincRNA	AC073133.1	ENSG00000228569.1	0	0
lincRNA	AC073133.2	ENSG00000233878.1	0	0
lincRNA	LINC00244	ENSG00000279418.1	0	0
lincRNA	AC006357.1	ENSG00000230033.1	0	0
lincRNA	AC006967.2	ENSG00000236102.2	0	0
lincRNA	AC006967.3	ENSG00000269446.2	0	0
lincRNA	AC004975.2	ENSG00000229660.1	0	0
lincRNA	AC006372.3	ENSG00000234210.1	0	0
lincRNA	AC006372.1	ENSG00000223872.1	0	0
lincRNA	AC144568.2	ENSG00000253896.3	0	0
lincRNA	AC136777.1	ENSG00000249868.5	0	0
lincRNA	AC100797.4	ENSG00000282375.1	0	0
lincRNA	AC026950.2	ENSG00000282759.1	0	0
lincRNA	AC129915.3	ENSG00000282692.1	0	0
lincRNA	AC110288.1	ENSG00000254160.1	0	0
lincRNA	AF067845.2	ENSG00000275427.1	0	0
lincRNA	AF067845.1	ENSG00000260721.1	0	0
lincRNA	AF067845.5	ENSG00000282160.1	0	0
lincRNA	AF067845.4	ENSG00000281961.1	0	0
lincRNA	AC100810.3	ENSG00000282021.1	0	0
lincRNA	AC019257.1	ENSG00000253764.2	0	0
lincRNA	KBTBD11-	ENSG00000253696.2	0	0
lincRNA	AC019257.2	ENSG00000270988.1	0	0
lincRNA	AC245164.1	ENSG00000253805.1	0	0
lincRNA	AC245519.1	ENSG00000253444.1	0	0
lincRNA	AC245519.2	ENSG00000282686.1	0	0
lincRNA	AC245187.2	ENSG00000282142.1	0	0
lincRNA	AC245187.1	ENSG00000253855.1	0	0
lincRNA	AC245123.1	ENSG00000277526.4	0	0
lincRNA	AC246817.2	ENSG00000254319.5	0	0
lincRNA	AC246817.1	ENSG00000253853.1	0	0
lincRNA	AC004944.1	ENSG00000253510.1	0	0
lincRNA	AC084768.1	ENSG00000254007.1	0	0
lincRNA	AC079054.1	ENSG00000253189.1	0	0
lincRNA	AC009435.1	ENSG00000253880.1	0	0
lincRNA	AF287957.1	ENSG00000271743.1	0	0
lincRNA	GS1-24F4.2	ENSG00000245857.2	0	0

lincRNA	FAM66B	ENSG00000215374.5	0	0
lincRNA	FAM66E	ENSG00000225725.4	0	0
lincRNA	AC068020.1	ENSG00000268955.2	0	0
lincRNA	AC103957.1	ENSG00000253505.2	0	0
lincRNA	AC103957.2	ENSG00000254153.1	0	0
lincRNA	AC114550.2	ENSG00000253343.1	0	0
lincRNA	AC114550.3	ENSG00000253774.1	0	0
lincRNA	AC114550.1	ENSG00000253130.1	0	0
lincRNA	AC087269.1	ENSG00000254367.5	0	0
lincRNA	AC022784.1	ENSG00000248538.7	0	0
lincRNA	AC022784.6	ENSG00000279949.1	0	0
lincRNA	AC022784.2	ENSG00000253252.2	0	0
lincRNA	AC022784.3	ENSG00000253887.1	0	0
lincRNA	AC021736.1	ENSG00000253735.1	0	0
lincRNA	AC021242.2	ENSG00000254237.5	0	0
lincRNA	LINC00599	ENSG00000253230.8	0	0
lincRNA	LINCR-0001	ENSG00000253641.5	0	0
lincRNA	AC104964.1	ENSG00000253678.2	0	0
lincRNA	AC104964.3	ENSG00000272505.1	0	1
lincRNA	AF131215.3	ENSG00000254839.1	0	0
lincRNA	LINC00529	ENSG00000236827.1	0	0
lincRNA	AF131216.5	ENSG00000284957.1	0	0
lincRNA	LINC00208	ENSG00000170983.3	0	0
lincRNA	C8orf49	ENSG00000255394.4	0	0
lincRNA	FAM66A	ENSG00000227888.4	0	0
lincRNA	AC087203.3	ENSG00000270074.1	0	0
lincRNA	AC130352.1	ENSG00000270154.1	0	0
lincRNA	AC123777.1	ENSG00000254813.5	0	0
lincRNA	LINC00681	ENSG00000255494.1	0	0
lincRNA	AC022690.2	ENSG00000254753.1	0	0
lincRNA	AC018437.2	ENSG00000253648.1	0	0
lincRNA	AC011586.2	ENSG00000253496.3	0	0
lincRNA	AC011586.1	ENSG00000253184.1	0	0
lincRNA	AC079193.2	ENSG00000249258.2	0	1
lincRNA	AC087273.2	ENSG00000254054.2	0	0
lincRNA	AC025062.3	ENSG00000285624.1	0	0
lincRNA	AC100849.1	ENSG00000253557.5	0	0
lincRNA	AC100849.2	ENSG00000254242.1	0	0
lincRNA	AC068880.3	ENSG00000254201.1	0	0
lincRNA	AC068880.2	ENSG00000253280.1	0	0
lincRNA	AC090541.1	ENSG00000253270.1	0	0
lincRNA	AC100802.1	ENSG00000253775.2	0	0
lincRNA	LZTS1-AS1	ENSG00000253733.3	0	0
lincRNA	AC023403.1	ENSG00000253164.5	0	0

lincRNA	AC018541.1	ENSG00000253300.1	0	0
lincRNA	AC015468.4	ENSG00000254260.1	0	0
lincRNA	AC015468.3	ENSG00000254092.1	0	0
lincRNA	AC015468.1	ENSG00000253147.5	0	0
lincRNA	AC015468.2	ENSG00000254026.1	0	0
lincRNA	LINC02153	ENSG00000253199.1	0	0
lincRNA	AC103719.1	ENSG00000254040.1	0	0
lincRNA	AC021355.1	ENSG00000254334.1	0	0
lincRNA	AC104561.1	ENSG00000253390.1	0	0
lincRNA	AC104561.3	ENSG00000253986.1	0	0
lincRNA	AC012574.1	ENSG00000253471.1	0	0
lincRNA	AC012574.2	ENSG00000254002.1	0	0
lincRNA	AC107373.1	ENSG00000253832.1	0	0
lincRNA	AC091185.1	ENSG00000253476.1	0	0
lincRNA	AC009623.1	ENSG00000253100.1	0	0
lincRNA	AC009623.2	ENSG00000253342.1	0	0
lincRNA	AC009623.3	ENSG00000285890.1	0	0
lincRNA	AC090103.1	ENSG00000254178.1	0	0
lincRNA	AC090150.2	ENSG00000253903.2	0	0
lincRNA	AC084026.2	ENSG00000253632.1	0	0
lincRNA	AC084026.1	ENSG00000253182.1	0	0
lincRNA	LINC00589	ENSG00000251191.7	0	1
lincRNA	LINC02099	ENSG00000253490.5	0	0
lincRNA	AC131254.3	ENSG00000254086.1	0	0
lincRNA	AC131254.2	ENSG00000253604.1	0	0
lincRNA	LINC02209	ENSG00000253279.5	0	0
lincRNA	AC068672.1	ENSG00000253275.1	0	0
lincRNA	AF279873.3	ENSG00000253642.5	0	0
lincRNA	AF279873.4	ENSG00000254194.5	0	0
lincRNA	AC087855.2	ENSG00000254302.1	0	0
lincRNA	AC090993.1	ENSG00000253108.1	0	0
lincRNA	AC087855.1	ENSG00000254033.1	0	0
lincRNA	LINC01288	ENSG00000254344.2	0	0
lincRNA	AC100818.1	ENSG00000253452.5	0	0
lincRNA	AC124290.1	ENSG00000253524.1	0	0
lincRNA	AC090809.1	ENSG00000253363.5	0	0
lincRNA	AC092818.1	ENSG00000254038.1	0	0
lincRNA	LINC01605	ENSG00000253161.5	0	0
lincRNA	AC124067.1	ENSG00000253388.1	0	0
lincRNA	AC124067.3	ENSG00000254111.2	0	0
lincRNA	AC124067.4	ENSG00000254290.1	0	0
lincRNA	AC137579.2	ENSG00000254306.1	0	0
lincRNA	AP006545.2	ENSG00000272128.1	0	0
lincRNA	AC069120.1	ENSG00000253361.2	0	0

lincRNA	AC087518.1	ENSG00000253381.1	0	0
lincRNA	AC022733.1	ENSG00000253665.1	0	0
lincRNA	AC022733.2	ENSG00000254143.1	0	0
lincRNA	AC105999.1	ENSG00000253354.1	0	0
lincRNA	AC010857.1	ENSG00000272479.1	0	0
lincRNA	AC048387.1	ENSG00000254383.1	0	0
lincRNA	AC016868.1	ENSG00000253396.1	0	0
lincRNA	AC027702.1	ENSG00000260588.1	0	0
lincRNA	LINC00293	ENSG00000253314.5	0	0
lincRNA	AC091163.1	ENSG00000253745.1	0	0
lincRNA	AC091163.2	ENSG00000253782.1	0	0
lincRNA	AC120036.5	ENSG00000285992.1	0	0
lincRNA	AC120036.4	ENSG00000255366.1	0	2
lincRNA	AC026904.1	ENSG00000253140.1	0	0
lincRNA	LINC02599	ENSG00000233858.4	0	0
lincRNA	AC026904.3	ENSG00000253702.1	0	0
lincRNA	AC022915.3	ENSG00000253892.1	0	0
lincRNA	AC090155.2	ENSG00000253380.1	0	0
lincRNA	AC090155.1	ENSG00000253206.1	0	0
lincRNA	AC113145.1	ENSG00000253484.1	0	0
lincRNA	AC023762.1	ENSG00000271148.1	0	0
lincRNA	AC064807.2	ENSG00000253844.1	0	0
lincRNA	AC064807.3	ENSG00000254136.1	0	0
lincRNA	AC103831.1	ENSG00000253924.1	0	0
lincRNA	AC131902.2	ENSG00000260484.1	0	0
lincRNA	AC131902.1	ENSG00000253369.1	0	0
lincRNA	AC022034.3	ENSG00000254204.1	0	0
lincRNA	AC022679.2	ENSG00000253976.1	0	1
lincRNA	AC022679.1	ENSG00000253857.1	0	0
lincRNA	AC107952.2	ENSG00000272343.1	0	0
lincRNA	AC013644.1	ENSG00000253139.1	0	0
lincRNA	AC009597.1	ENSG00000254055.1	0	0
lincRNA	LINC01606	ENSG00000253301.5	0	0
lincRNA	AC025674.2	ENSG00000253614.1	0	0
lincRNA	LINC00588	ENSG00000215117.5	0	0
lincRNA	AC068075.1	ENSG00000253871.1	0	0
lincRNA	AC090796.1	ENSG00000253821.1	0	0
lincRNA	AC104051.1	ENSG00000253322.1	0	0
lincRNA	LINC01602	ENSG00000205293.4	0	0
lincRNA	AC012103.1	ENSG00000253376.1	0	0
lincRNA	AC104350.1	ENSG00000253523.1	0	0
lincRNA	AC027698.1	ENSG00000253116.1	0	0
lincRNA	AC087664.2	ENSG00000253879.1	0	0
lincRNA	AC021393.1	ENSG00000254775.1	0	0

lincRNA	LINC01301	ENSG00000251396.6	0	0
lincRNA	AC068389.1	ENSG00000228862.4	0	0
lincRNA	AC068389.2	ENSG00000255289.1	0	0
lincRNA	AC022182.1	ENSG00000254777.5	0	0
lincRNA	AC022182.2	ENSG00000254802.1	0	0
lincRNA	AC091173.1	ENSG00000251127.2	0	0
lincRNA	AC025524.2	ENSG00000254119.5	0	0
lincRNA	AC025524.1	ENSG00000253834.1	0	0
lincRNA	LINC02155	ENSG00000254120.1	0	0
lincRNA	AC023095.1	ENSG00000285971.1	0	0
lincRNA	NKAIN3-IT1	ENSG00000274956.2	0	0
lincRNA	AC011124.1	ENSG00000253205.5	0	0
lincRNA	AC011124.2	ENSG00000253894.1	0	0
lincRNA	AC018953.1	ENSG00000253583.1	0	0
lincRNA	AC022639.1	ENSG00000253554.6	0	0
lincRNA	AC069133.1	ENSG00000253762.1	0	0
lincRNA	LINC01289	ENSG00000253734.1	0	0
lincRNA	MIR124-2HG	ENSG00000254377.5	0	0
lincRNA	AC104232.1	ENSG00000254006.5	0	0
lincRNA	LINC00251	ENSG00000280725.2	0	0
lincRNA	LINC01299	ENSG00000254081.1	0	0
lincRNA	AC084082.1	ENSG00000253190.3	0	1
lincRNA	LINC00967	ENSG00000253138.5	0	0
lincRNA	RRS1-AS1	ENSG00000246145.1	0	0
lincRNA	AC083967.1	ENSG00000254337.1	0	0
lincRNA	LINC01592	ENSG00000253658.5	0	0
lincRNA	AC021785.1	ENSG00000253373.1	0	0
lincRNA	LINC01603	ENSG00000253479.5	0	0
lincRNA	AC022730.3	ENSG00000253143.3	0	0
lincRNA	AC022730.4	ENSG00000253967.1	0	0
lincRNA	AC009446.1	ENSG00000254277.1	0	0
lincRNA	AC104012.1	ENSG00000253287.1	0	0
lincRNA	AC078906.1	ENSG00000253317.1	0	1
lincRNA	AC011131.1	ENSG00000254347.1	0	0
lincRNA	AC090735.1	ENSG00000253784.1	0	0
lincRNA	AC022893.2	ENSG00000260838.3	0	0
lincRNA	AC111149.2	ENSG00000253339.1	0	0
lincRNA	AC111149.1	ENSG00000253235.1	0	0
lincRNA	AC027018.1	ENSG00000254538.1	0	0
lincRNA	AC087672.1	ENSG00000253115.1	0	0
lincRNA	MIR2052HG	ENSG00000254349.5	0	0
lincRNA	AC011632.2	ENSG00000254043.1	0	0
lincRNA	AC022274.1	ENSG00000253712.1	0	0
lincRNA	AC067773.1	ENSG00000270866.1	0	0

lincRNA	LINC01109	ENSG00000271167.1	0	0
lincRNA	LINC01111	ENSG00000254300.1	0	1
lincRNA	AC023200.1	ENSG00000253416.1	0	0
lincRNA	AC062004.1	ENSG00000254366.6	0	0
lincRNA	AC084706.1	ENSG00000254001.5	0	0
lincRNA	PKIA-AS1	ENSG00000254266.5	0	0
lincRNA	AC083837.1	ENSG00000261618.1	0	0
lincRNA	AC138646.1	ENSG00000253659.1	0	0
lincRNA	AC036214.1	ENSG00000249328.2	0	0
lincRNA	AC034114.2	ENSG00000253238.2	0	0
lincRNA	AC009812.3	ENSG00000254162.1	0	0
lincRNA	AC022778.1	ENSG00000254060.1	0	0
lincRNA	AC079209.2	ENSG00000254177.1	0	0
lincRNA	AC079209.1	ENSG00000253214.1	0	0
lincRNA	AC009902.3	ENSG00000272425.1	0	0
lincRNA	AC027701.1	ENSG00000254123.1	0	0
lincRNA	AC060765.1	ENSG00000253503.5	0	0
lincRNA	AC105031.2	ENSG00000253836.1	0	0
lincRNA	AC105031.3	ENSG00000254394.1	0	0
lincRNA	LINC01419	ENSG00000253898.1	0	0
lincRNA	AC015522.1	ENSG00000254202.1	0	0
lincRNA	AC093331.2	ENSG00000254380.1	0	0
lincRNA	AC232323.2	ENSG00000285768.1	0	0
lincRNA	AC090572.2	ENSG00000253778.1	0	0
lincRNA	AB015752.1	ENSG00000254180.1	0	0
lincRNA	LINC01030	ENSG00000253799.1	0	0
lincRNA	AC103409.1	ENSG00000253901.1	0	0
lincRNA	AC022695.2	ENSG00000253682.1	0	0
lincRNA	AC022695.3	ENSG00000253749.1	0	0
lincRNA	AC091096.1	ENSG00000253634.2	0	0
lincRNA	AC104211.1	ENSG00000253177.1	0	0
lincRNA	AC104211.3	ENSG00000254020.1	0	0
lincRNA	AC104211.2	ENSG00000253577.1	0	0
lincRNA	C8orf87	ENSG00000279847.3	0	0
lincRNA	AC016885.1	ENSG00000253960.1	0	0
lincRNA	AC016885.2	ENSG00000254089.1	0	0
lincRNA	AC084346.1	ENSG00000254057.1	0	0
lincRNA	AC084346.2	ENSG00000264448.5	0	0
lincRNA	AP003351.1	ENSG00000253585.1	0	0
lincRNA	AC023632.2	ENSG00000253704.1	0	0
lincRNA	C8orf37-AS1	ENSG00000253773.2	0	0
lincRNA	LINC01298	ENSG00000253351.1	0	0
lincRNA	AC083836.1	ENSG00000253872.1	0	0
lincRNA	AC012339.1	ENSG00000248762.2	0	0

lincRNA	AP003465.2	ENSG00000270131.1	0	0
lincRNA	AP003548.1	ENSG00000253105.5	0	0
lincRNA	AP003115.1	ENSG00000260640.1	0	0
lincRNA	AC016877.1	ENSG00000229625.3	0	0
lincRNA	AP001574.1	ENSG00000253217.1	0	0
lincRNA	AP003472.1	ENSG00000253824.1	0	0
lincRNA	AP003472.2	ENSG00000253842.1	0	0
lincRNA	AP003469.2	ENSG00000253395.1	0	0
lincRNA	AP001330.2	ENSG00000253679.1	0	0
lincRNA	AP001330.1	ENSG00000253282.1	0	0
lincRNA	AP001208.1	ENSG00000253644.1	0	0
lincRNA	AP001208.2	ENSG00000254084.1	0	0
lincRNA	AP003696.1	ENSG00000253385.1	0	0
lincRNA	AP003354.1	ENSG00000253263.1	0	0
lincRNA	AC012213.3	ENSG00000266289.1	0	0
lincRNA	AC012213.2	ENSG00000261670.1	0	1
lincRNA	AC012213.4	ENSG00000271830.1	0	0
lincRNA	AC090802.1	ENSG00000253526.1	0	0
lincRNA	AC027031.1	ENSG00000254021.1	0	0
lincRNA	AC022634.2	ENSG00000253949.1	0	0
lincRNA	AC073023.1	ENSG00000253956.1	0	0
lincRNA	AC025366.1	ENSG00000253122.1	0	0
lincRNA	AP005357.1	ENSG00000253717.1	0	0
lincRNA	LINC01608	ENSG00000253877.5	0	0
lincRNA	LINC01609	ENSG00000253103.1	0	0
lincRNA	AC009930.1	ENSG00000253489.1	0	0
lincRNA	LINC02237	ENSG00000253434.7	0	0
lincRNA	AC103993.1	ENSG00000253207.3	0	0
lincRNA	AC064802.1	ENSG00000254339.5	0	0
lincRNA	LINC00536	ENSG00000249917.2	0	0
lincRNA	AC105177.1	ENSG00000253672.1	0	0
lincRNA	MAL2-AS1	ENSG00000253972.5	0	0
lincRNA	AP005717.1	ENSG00000245330.4	0	0
lincRNA	AC068413.1	ENSG00000253619.1	0	0
lincRNA	AC104233.1	ENSG00000254209.1	0	0
lincRNA	AC037486.1	ENSG00000254303.1	0	0
lincRNA	SMILR	ENSG00000255364.1	0	0
lincRNA	LINC01151	ENSG00000253819.1	0	0
lincRNA	AC100872.1	ENSG00000263443.1	0	0
lincRNA	AC016405.3	ENSG00000272384.1	0	0
lincRNA	AC016405.2	ENSG00000272043.1	0	0
lincRNA	AC104316.2	ENSG00000259631.1	0	0
lincRNA	AC090193.2	ENSG00000254113.1	0	0
lincRNA	AC090753.1	ENSG00000254000.1	0	0

lincRNA	AC090921.1	ENSG00000214803.3	0	2
lincRNA	AC100858.3	ENSG00000255491.2	0	0
lincRNA	AC100858.2	ENSG00000255313.1	0	0
lincRNA	AC091114.1	ENSG00000253111.2	0	0
lincRNA	AC016074.1	ENSG00000253470.1	0	0
lincRNA	LINC00861	ENSG00000245164.6	0	0
lincRNA	AC024681.2	ENSG00000254227.1	0	0
lincRNA	AC087667.1	ENSG00000244791.2	0	0
lincRNA	AC084116.2	ENSG00000253427.1	0	0
lincRNA	AC084116.1	ENSG00000253220.2	0	0
lincRNA	AC084116.3	ENSG00000253530.1	0	0
lincRNA	AC024382.1	ENSG00000253573.1	0	0
lincRNA	CCAT2	ENSG00000280997.1	0	0
lincRNA	AC011257.1	ENSG00000253926.1	0	0
lincRNA	AC103718.1	ENSG00000285108.1	0	0
lincRNA	AC022973.3	ENSG00000254263.1	0	0
lincRNA	AC022973.2	ENSG00000253720.1	0	0
lincRNA	AC090987.1	ENSG00000253656.1	0	0
lincRNA	AC087341.1	ENSG00000253407.1	0	0
lincRNA	AC104257.1	ENSG00000253507.5	0	0
lincRNA	HPYR1	ENSG00000253521.2	0	0
lincRNA	AC133634.1	ENSG00000253561.1	0	0
lincRNA	AC090821.1	ENSG00000253165.1	0	0
lincRNA	AC110741.1	ENSG00000253593.2	0	0
lincRNA	AC105180.1	ENSG00000253627.1	0	0
lincRNA	AC087045.2	ENSG00000272456.1	0	0
lincRNA	AC083843.1	ENSG00000254028.1	0	0
lincRNA	NCRNA0025	ENSG00000253433.1	0	0
lincRNA	AC103764.1	ENSG00000251218.1	0	0
lincRNA	LINC01591	ENSG00000254083.5	0	0
lincRNA	LINC02055	ENSG00000254101.6	0	0
lincRNA	AC023781.1	ENSG00000285683.1	0	0
lincRNA	AC105213.1	ENSG00000285817.1	0	0
lincRNA	AC046195.1	ENSG00000253288.1	0	0
lincRNA	AC046195.2	ENSG00000254361.1	0	0
lincRNA	AC079015.1	ENSG00000253988.1	0	0
lincRNA	AC027541.1	ENSG00000253432.1	0	0
lincRNA	AC100807.1	ENSG00000253783.1	0	0
lincRNA	AC087354.1	ENSG00000254258.1	0	0
lincRNA	AC100860.1	ENSG00000253266.1	0	0
lincRNA	AC011676.3	ENSG00000254197.1	0	0
lincRNA	AC104417.2	ENSG00000261710.1	0	0
lincRNA	AF186192.1	ENSG00000254533.1	0	0
lincRNA	AF186192.3	ENSG00000263612.1	0	0

lincRNA	FAM138C	ENSG00000218839.5	0	0
lincRNA	PGM5P3-AS1	ENSG00000277631.4	0	0
lincRNA	AL449043.1	ENSG00000227917.2	0	0
lincRNA	LINC01388	ENSG00000231808.2	0	0
lincRNA	LINC01230	ENSG00000281769.1	0	0
lincRNA	AL353614.1	ENSG00000231052.1	0	0
lincRNA	LINC01231	ENSG00000236511.1	0	0
lincRNA	AL354977.2	ENSG00000237359.1	0	0
lincRNA	AL354977.1	ENSG00000226669.2	0	0
lincRNA	AL135786.2	ENSG00000272866.1	0	0
lincRNA	AL162253.1	ENSG00000231509.1	0	0
lincRNA	AL162384.1	ENSG00000228739.1	0	0
lincRNA	AL162411.1	ENSG00000236924.1	0	0
lincRNA	AL354707.2	ENSG00000229611.1	0	0
lincRNA	AL161443.1	ENSG00000285688.1	0	0
lincRNA	AL354694.1	ENSG00000273056.1	0	0
lincRNA	AL135923.1	ENSG00000226376.1	0	0
lincRNA	AL135923.2	ENSG00000231902.1	0	0
lincRNA	PTPRD-AS1	ENSG00000225706.1	0	0
lincRNA	AL513422.1	ENSG00000230920.1	0	0
lincRNA	PTPRD-AS2	ENSG00000226717.2	0	0
lincRNA	AL135790.1	ENSG00000232035.1	0	0
lincRNA	AL451129.1	ENSG00000230365.1	0	0
lincRNA	AL353595.1	ENSG00000285784.1	0	0
lincRNA	AL589678.1	ENSG00000224935.1	0	0
lincRNA	AL161449.2	ENSG00000285637.1	0	0
lincRNA	LINC01235	ENSG00000270547.5	0	0
lincRNA	AL583785.1	ENSG00000226197.2	0	0
lincRNA	LINC00583	ENSG00000205636.3	0	0
lincRNA	AL360089.1	ENSG00000235533.1	0	0
lincRNA	AL159169.3	ENSG00000273399.2	0	0
lincRNA	AL159169.2	ENSG00000272871.1	0	0
lincRNA	AL162725.2	ENSG00000237153.3	0	0
lincRNA	AL358117.1	ENSG00000285169.1	0	0
lincRNA	AL442638.1	ENSG00000227167.1	0	0
lincRNA	AL591222.1	ENSG00000261402.1	0	0
lincRNA	FOCAD-AS1	ENSG00000227071.1	0	0
lincRNA	AL353732.1	ENSG00000261696.1	0	0
lincRNA	AL157937.1	ENSG00000236921.1	0	0
lincRNA	AL391117.1	ENSG00000284418.1	0	0
lincRNA	AC017067.1	ENSG00000224549.1	0	0
lincRNA	AL445623.1	ENSG00000233906.1	0	0
lincRNA	AL365204.1	ENSG00000231460.2	0	0
lincRNA	AL365204.2	ENSG00000237414.2	0	0

lincRNA	AL353811.1	ENSG00000269957.6	0	0
lincRNA	LINC01241	ENSG00000236306.2	0	0
lincRNA	AL353753.1	ENSG00000276759.2	0	0
lincRNA	AL451137.1	ENSG00000254261.1	0	0
lincRNA	AL356133.2	ENSG00000253400.1	0	0
lincRNA	AL355432.1	ENSG00000254396.1	0	0
lincRNA	AL360014.1	ENSG00000260390.1	0	0
lincRNA	LINC01242	ENSG00000280683.1	0	0
lincRNA	LINC01243	ENSG00000260720.1	0	0
lincRNA	AL589642.2	ENSG00000230516.1	0	0
lincRNA	AL157884.2	ENSG00000231193.1	0	0
lincRNA	CYP4F26P	ENSG00000226562.3	0	0
lincRNA	AL356489.2	ENSG00000260947.1	0	0
lincRNA	AL356489.1	ENSG00000227301.1	0	0
lincRNA	DNAJB5-DT	ENSG00000281491.1	0	0
lincRNA	RMRP	ENSG00000269900.3	0	0
lincRNA	AL133410.1	ENSG00000227388.2	0	0
lincRNA	LINC01400	ENSG00000232920.1	0	0
lincRNA	AL512604.3	ENSG00000260100.1	0	0
lincRNA	LINC01627	ENSG00000224648.1	0	0
lincRNA	AL135785.1	ENSG00000223716.2	0	0
lincRNA	AL591543.1	ENSG00000284612.1	0	0
lincRNA	AL845311.1	ENSG00000228467.2	0	0
lincRNA	FAM74A1	ENSG00000215112.6	0	0
lincRNA	BX005214.1	ENSG00000224038.1	0	0
lincRNA	BX005214.2	ENSG00000231140.3	0	0
lincRNA	AL773545.1	ENSG00000228136.1	0	0
lincRNA	FAM95B1	ENSG00000223839.7	0	0
lincRNA	BMS1P14	ENSG00000276500.1	0	0
lincRNA	FP325318.1	ENSG00000276266.1	0	0
lincRNA	AL353626.1	ENSG00000275390.1	0	0
lincRNA	BX255923.1	ENSG00000225655.5	0	0
lincRNA	BX255923.2	ENSG00000276462.1	0	0
lincRNA	AL845472.1	ENSG00000275450.1	0	0
lincRNA	AL354718.2	ENSG00000276502.1	0	0
lincRNA	FAM242F	ENSG00000275239.4	0	0
lincRNA	FAM74A7	ENSG00000277260.1	0	0
lincRNA	AL445584.2	ENSG00000275649.1	0	0
lincRNA	BX088651.4	ENSG00000237357.2	0	0
lincRNA	BX088651.2	ENSG00000229311.1	0	0
lincRNA	BX664718.1	ENSG00000233424.2	0	0
lincRNA	AL591441.2	ENSG00000277697.1	0	0
lincRNA	AL591441.1	ENSG00000276128.1	0	0
lincRNA	AL590491.2	ENSG00000276422.1	0	0

lincRNA	BX005040.1	ENSG00000275676.1	0	0
lincRNA	FAM242D	ENSG00000231248.2	0	0
lincRNA	AL935212.1	ENSG00000204814.5	0	0
lincRNA	FAM27C	ENSG00000154537.4	0	0
lincRNA	AL391987.4	ENSG00000235659.1	0	0
lincRNA	AL391987.3	ENSG00000223379.1	0	0
lincRNA	FAM242E	ENSG00000229697.2	0	0
lincRNA	CR769775.2	ENSG00000231838.1	0	0
lincRNA	AL590399.3	ENSG00000231212.1	0	0
lincRNA	AL590399.1	ENSG00000204802.3	0	0
lincRNA	AL590399.5	ENSG00000233651.2	0	0
lincRNA	LINC01189	ENSG00000232827.2	0	0
lincRNA	BX005266.2	ENSG00000226007.2	0	0
lincRNA	LINC01410	ENSG00000238113.6	0	0
lincRNA	AL512625.2	ENSG00000229422.2	0	0
lincRNA	AL772155.1	ENSG00000224267.1	0	0
lincRNA	CR786580.1	ENSG00000225411.3	0	0
lincRNA	CR769776.3	ENSG00000285580.1	0	0
lincRNA	AL136317.2	ENSG00000274893.1	0	0
lincRNA	AL513478.2	ENSG00000278849.1	0	0
lincRNA	AL353770.4	ENSG00000277350.1	0	0
lincRNA	AL353770.3	ENSG00000276348.1	0	0
lincRNA	BX664730.1	ENSG00000276898.1	0	0
lincRNA	AL353608.3	ENSG00000234394.8	0	0
lincRNA	AL353608.1	ENSG00000225337.1	0	0
lincRNA	AL353616.2	ENSG00000226337.3	0	0
lincRNA	LINC01506	ENSG00000234506.5	0	0
lincRNA	BANCR	ENSG00000278910.4	0	0
lincRNA	C9orf135-DT	ENSG00000225626.2	0	0
lincRNA	LINC01474	ENSG00000236849.5	0	0
lincRNA	AL451127.1	ENSG00000232590.1	0	0
lincRNA	AL355674.1	ENSG00000227809.1	0	0
lincRNA	AL353780.1	ENSG00000236376.1	0	0
lincRNA	AL512634.1	ENSG00000260995.2	0	0
lincRNA	LNCARSR	ENSG00000233086.7	0	0
lincRNA	AL161912.1	ENSG00000232494.1	0	0
lincRNA	AL161912.2	ENSG00000260564.1	0	0
lincRNA	AL161782.1	ENSG00000280366.1	0	0
lincRNA	LINC01507	ENSG00000230945.1	0	0
lincRNA	AL138749.1	ENSG00000226798.1	0	0
lincRNA	AL591368.1	ENSG00000233926.1	0	0
lincRNA	AL158154.1	ENSG00000230846.1	0	0
lincRNA	AL158154.2	ENSG00000267559.5	0	0
lincRNA	AL162726.1	ENSG00000225085.1	0	0

lincRNA	AL162726.4	ENSG00000232749.2	0	0
lincRNA	AL354897.1	ENSG00000285556.1	0	0
lincRNA	AL583827.1	ENSG00000285634.1	0	0
lincRNA	AL157882.1	ENSG00000229454.1	0	0
lincRNA	AL353743.2	ENSG00000230303.5	0	0
lincRNA	AL158828.1	ENSG00000235819.1	0	0
lincRNA	AL353613.2	ENSG00000285686.1	0	0
lincRNA	C9orf170	ENSG00000204446.4	0	1
lincRNA	AL592486.1	ENSG00000228189.4	0	0
lincRNA	AL772202.1	ENSG00000228658.1	0	0
lincRNA	AL161910.1	ENSG00000285907.1	0	1
lincRNA	UNQ6494	ENSG00000237372.2	0	0
lincRNA	BX470209.1	ENSG00000226206.1	0	0
lincRNA	BX470209.2	ENSG00000236507.1	0	0
lincRNA	AL161629.1	ENSG00000237626.1	0	0
lincRNA	MIR4290HG	ENSG00000227555.1	0	0
lincRNA	LINC01501	ENSG00000229613.1	0	0
lincRNA	AL355607.1	ENSG00000228216.1	0	0
lincRNA	AL355607.2	ENSG00000260454.1	0	0
lincRNA	AL158071.2	ENSG00000230537.1	0	0
lincRNA	LINC00484	ENSG00000235641.4	0	0
lincRNA	AL390760.1	ENSG00000203364.2	0	0
lincRNA	AL442224.1	ENSG00000231990.1	0	0
lincRNA	BARX1-DT	ENSG00000235601.1	0	0
lincRNA	AL158152.2	ENSG00000269946.1	0	1
lincRNA	LINC02603	ENSG00000230262.6	0	0
lincRNA	AL691447.2	ENSG00000232063.1	0	0
lincRNA	AL161729.3	ENSG00000271384.1	0	1
lincRNA	AL392185.1	ENSG00000229345.2	0	0
lincRNA	AL354861.2	ENSG00000228142.2	0	0
lincRNA	AL354861.3	ENSG00000268926.3	0	0
lincRNA	LINC00092	ENSG00000225194.3	0	0
lincRNA	AL449403.1	ENSG00000237212.1	0	0
lincRNA	AL449403.2	ENSG00000275465.6	0	0
lincRNA	AL589843.1	ENSG00000224848.1	0	0
lincRNA	AL590705.3	ENSG00000242375.1	0	0
lincRNA	AL590705.2	ENSG00000235494.1	0	0
lincRNA	PTCSC2	ENSG00000236130.2	0	0
lincRNA	AL445531.1	ENSG00000260677.1	0	0
lincRNA	AL162427.1	ENSG00000227269.1	0	0
lincRNA	NAMA	ENSG00000271086.6	0	0
lincRNA	AL162394.1	ENSG00000234860.1	0	0
lincRNA	AL359710.1	ENSG00000237461.1	0	0
lincRNA	LINC00587	ENSG00000204250.3	0	0

lincRNA	LINC01492	ENSG00000225564.6	0	0
lincRNA	AL390962.1	ENSG00000226566.2	0	0
lincRNA	AL590381.1	ENSG00000285483.1	0	0
lincRNA	LINC01505	ENSG00000234323.6	0	0
lincRNA	AL732323.1	ENSG00000225002.2	0	0
lincRNA	AL451140.1	ENSG00000229297.1	0	0
lincRNA	AL512649.2	ENSG00000237062.1	0	0
lincRNA	LINC01505	ENSG00000234229.8	0	0
lincRNA	AL807761.3	ENSG00000226047.1	0	0
lincRNA	LINC01509	ENSG00000226825.1	0	0
lincRNA	AL353742.1	ENSG00000230030.1	0	0
lincRNA	AL358779.1	ENSG00000231678.2	0	0
lincRNA	AL592463.1	ENSG00000232896.1	0	0
lincRNA	AL162414.1	ENSG00000227531.1	0	0
lincRNA	AL162727.1	ENSG00000233817.1	0	0
lincRNA	AL138895.1	ENSG00000235119.1	0	0
lincRNA	AL162425.1	ENSG00000236461.1	0	0
lincRNA	AL355601.1	ENSG00000234692.1	0	0
lincRNA	LINC00474	ENSG00000204148.3	0	0
lincRNA	AL691426.1	ENSG00000228707.1	0	0
lincRNA	AL445644.1	ENSG00000233516.1	0	0
lincRNA	AL161630.1	ENSG00000233569.1	0	0
lincRNA	AL160272.1	ENSG00000284977.1	0	1
lincRNA	AL354754.1	ENSG00000231901.1	0	0
lincRNA	AL355592.1	ENSG00000225050.1	0	0
lincRNA	LINC02578	ENSG00000235201.1	0	0
lincRNA	AC006288.1	ENSG00000260970.1	0	0
lincRNA	LINC01613	ENSG00000261432.1	0	0
lincRNA	AL441989.1	ENSG00000225960.1	0	0
lincRNA	AL359636.3	ENSG00000233616.2	0	0
lincRNA	AL354979.1	ENSG00000231149.2	0	0
lincRNA	AL589923.1	ENSG00000232413.1	0	0
lincRNA	AL162391.1	ENSG00000228392.1	0	0
lincRNA	AL356309.2	ENSG00000233721.1	0	0
lincRNA	AL356309.1	ENSG00000226078.1	0	0
lincRNA	AL359091.3	ENSG00000272593.1	0	0
lincRNA	AL359091.1	ENSG00000207955.5	0	0
lincRNA	AL592211.1	ENSG00000267834.1	0	0
lincRNA	AL158151.4	ENSG00000268707.1	0	0
lincRNA	AL161785.2	ENSG00000235007.2	0	0
lincRNA	AL161785.3	ENSG00000241084.1	0	0
lincRNA	AL161785.1	ENSG00000224307.1	0	0
lincRNA	C9orf106	ENSG00000179082.3	0	0
lincRNA	AL353803.5	ENSG00000268615.1	0	0

lincRNA	AL353803.2	ENSG00000230676.1	0	0
lincRNA	AL353803.1	ENSG00000226355.1	0	0
lincRNA	AL353803.4	ENSG00000261334.1	0	0
lincRNA	AL360004.1	ENSG00000196979.1	0	0
lincRNA	AL160271.1	ENSG00000236687.1	0	0
lincRNA	LINC02247	ENSG00000223729.1	0	0
lincRNA	AL669970.1	ENSG00000223795.2	0	0
lincRNA	AL669970.3	ENSG00000228877.2	0	0
lincRNA	AL669970.2	ENSG00000227150.1	0	0
lincRNA	AL353611.1	ENSG00000236403.1	0	0
lincRNA	AL390778.1	ENSG00000227958.1	0	0
lincRNA	AL390778.2	ENSG00000233936.1	0	0
lincRNA	C9orf62	ENSG00000178243.4	0	0
lincRNA	AL353615.1	ENSG00000235572.1	0	0
lincRNA	LINC01502	ENSG00000237339.5	0	0
lincRNA	DKFZP434A0	ENSG00000262075.3	0	2
lincRNA	PRR31	ENSG00000198454.2	0	0
lincRNA	AL954642.1	ENSG00000237419.1	0	0
lincRNA	AC215217.1	ENSG00000260370.1	0	0
lincRNA	AL157709.1	ENSG00000231601.1	0	0
lincRNA	LINC00200	ENSG00000229205.3	0	0
lincRNA	LINC00700	ENSG00000234962.5	0	0
lincRNA	AL441943.1	ENSG00000226694.2	0	0
lincRNA	AL441943.2	ENSG00000227912.1	0	0
lincRNA	AL441943.3	ENSG00000231326.6	0	0
lincRNA	LINC00701	ENSG00000234556.1	0	0
lincRNA	AC024908.1	ENSG00000234170.5	0	0
lincRNA	AL713851.2	ENSG00000237500.1	0	0
lincRNA	AL713851.1	ENSG00000235281.1	0	0
lincRNA	AL731533.1	ENSG00000234182.1	0	0
lincRNA	AL731533.2	ENSG00000273001.1	0	0
lincRNA	AL451164.2	ENSG00000236968.1	0	0
lincRNA	AL451164.1	ENSG00000226762.2	0	0
lincRNA	AL139280.1	ENSG00000227338.1	0	0
lincRNA	AL357833.1	ENSG00000233321.1	0	0
lincRNA	AL357833.2	ENSG00000236892.1	0	0
lincRNA	AL513303.2	ENSG00000230573.1	0	0
lincRNA	AL513303.1	ENSG00000226005.4	0	0
lincRNA	AC025822.1	ENSG00000227101.1	0	0
lincRNA	AC025822.2	ENSG00000236990.1	0	0
lincRNA	AC022535.1	ENSG00000230096.1	0	0
lincRNA	LINC00705	ENSG00000225269.2	0	0
lincRNA	AL355303.1	ENSG00000231039.2	0	0
lincRNA	LINC02561	ENSG00000224034.1	0	0

lincRNA	AL365356.3	ENSG00000228951.1	0	0
lincRNA	AL596094.1	ENSG00000272764.1	0	1
lincRNA	AL137145.2	ENSG00000215244.2	0	0
lincRNA	AL137145.1	ENSG00000212743.2	0	0
lincRNA	AL158210.1	ENSG00000225948.2	0	0
lincRNA	AL158210.2	ENSG00000285743.1	0	0
lincRNA	AL392086.3	ENSG00000285988.1	0	0
lincRNA	LINC00706	ENSG00000281186.1	0	0
lincRNA	LINC00707	ENSG00000238266.2	0	0
lincRNA	AL392086.2	ENSG00000285845.1	0	0
lincRNA	AL392086.1	ENSG00000234248.1	0	0
lincRNA	AL590095.1	ENSG00000223581.1	0	0
lincRNA	AL139125.1	ENSG00000232591.1	0	0
lincRNA	AL390294.1	ENSG00000232638.1	0	0
lincRNA	LINC00708	ENSG00000232170.5	0	0
lincRNA	AL390835.1	ENSG00000226861.1	0	0
lincRNA	AC025946.1	ENSG00000226990.1	0	0
lincRNA	AC044784.2	ENSG00000234752.1	0	0
lincRNA	AC044784.1	ENSG00000223808.1	0	0
lincRNA	LINC00709	ENSG00000230014.1	0	0
lincRNA	AL138775.1	ENSG00000224788.1	0	0
lincRNA	AL583859.1	ENSG00000234306.1	0	0
lincRNA	AL583859.2	ENSG00000270111.2	0	0
lincRNA	AL136369.2	ENSG00000231496.1	0	0
lincRNA	AL162408.1	ENSG00000229206.3	0	0
lincRNA	AL512631.1	ENSG00000271046.1	0	0
lincRNA	AL139405.1	ENSG00000285671.1	0	0
lincRNA	AL355339.1	ENSG00000226140.5	0	0
lincRNA	AL512641.1	ENSG00000234244.1	0	0
lincRNA	AL158209.1	ENSG00000230109.1	0	0
lincRNA	AL157831.2	ENSG00000260205.1	0	0
lincRNA	AL158211.1	ENSG00000261671.1	0	0
lincRNA	AL158211.4	ENSG00000272516.1	0	1
lincRNA	AL158211.2	ENSG00000271981.1	0	0
lincRNA	AL158211.3	ENSG00000272366.1	0	0
lincRNA	AL513128.2	ENSG00000285254.1	0	0
lincRNA	AL157385.2	ENSG00000285750.1	0	0
lincRNA	AL512598.1	ENSG00000273107.1	0	0
lincRNA	LINC01516	ENSG00000231422.2	0	0
lincRNA	LINC00836	ENSG00000280809.3	0	0
lincRNA	AL390961.1	ENSG00000234296.1	0	0
lincRNA	AL160291.1	ENSG00000262412.1	0	0
lincRNA	AL355493.2	ENSG00000260151.1	0	0
lincRNA	AL138920.1	ENSG00000285737.1	0	1

lincRNA	AL390866.1	ENSG00000233472.1	0	0
lincRNA	AC022021.1	ENSG00000282996.1	0	0
lincRNA	AL358234.1	ENSG00000237128.1	0	0
lincRNA	AL355376.1	ENSG00000236716.1	0	0
lincRNA	LINC00837	ENSG00000235824.4	0	0
lincRNA	AL590068.1	ENSG00000228800.1	0	0
lincRNA	AL590068.2	ENSG00000230298.1	0	0
lincRNA	AL590068.3	ENSG00000285630.1	0	0
lincRNA	AL451107.1	ENSG00000230720.1	0	0
lincRNA	AL356258.1	ENSG00000272381.1	0	0
lincRNA	AL161935.1	ENSG00000223834.3	0	2
lincRNA	AL161935.3	ENSG00000237797.1	0	1
lincRNA	AL121748.1	ENSG00000233387.1	0	0
lincRNA	AL353600.1	ENSG00000273012.1	0	1
lincRNA	AC018980.1	ENSG00000226412.1	0	0
lincRNA	AL450313.1	ENSG00000203565.2	0	0
lincRNA	LINC00838	ENSG00000261683.1	0	0
lincRNA	AL117338.1	ENSG00000223470.3	0	0
lincRNA	AL117336.1	ENSG00000233200.1	0	0
lincRNA	PCAT5	ENSG00000280719.1	0	0
lincRNA	AL365500.1	ENSG00000227313.1	0	0
lincRNA	AL355300.1	ENSG00000227475.1	0	0
lincRNA	AL590730.1	ENSG00000237002.6	0	0
lincRNA	AL390061.1	ENSG00000238176.1	0	0
lincRNA	AL157387.1	ENSG00000234918.1	0	0
lincRNA	AL132657.1	ENSG00000226578.1	0	1
lincRNA	AL132657.2	ENSG00000260137.1	0	0
lincRNA	AL117337.1	ENSG00000226447.1	0	0
lincRNA	AL117339.4	ENSG00000272983.1	0	1
lincRNA	AL133216.1	ENSG00000203496.9	0	0
lincRNA	AL022344.1	ENSG00000234864.1	0	0
lincRNA	AL365199.1	ENSG00000234944.1	0	0
lincRNA	LINC01264	ENSG00000229630.1	0	0
lincRNA	AC068707.1	ENSG00000224265.1	0	0
lincRNA	AC068707.2	ENSG00000285712.1	0	0
lincRNA	AL645634.2	ENSG00000237389.1	0	0
lincRNA	AL645634.1	ENSG00000228426.1	0	0
lincRNA	AL355989.1	ENSG00000234973.5	0	0
lincRNA	LINC00840	ENSG00000226808.2	0	2
lincRNA	AL139237.2	ENSG00000236769.2	0	0
lincRNA	AL139237.1	ENSG00000227029.1	0	0
lincRNA	LINC00841	ENSG00000233395.1	0	0
lincRNA	AL137026.2	ENSG00000237590.1	0	0
lincRNA	AL356157.1	ENSG00000234580.1	0	0

lincRNA	AL353801.1	ENSG00000223462.2	0	0
lincRNA	AL358394.1	ENSG00000227683.1	0	0
lincRNA	AL356056.1	ENSG00000229227.8	0	0
lincRNA	AL356056.3	ENSG00000272430.1	0	0
lincRNA	AL731733.1	ENSG00000285662.1	0	0
lincRNA	BX547991.1	ENSG00000264404.2	0	0
lincRNA	AC068898.1	ENSG00000236800.1	0	0
lincRNA	LINC00843	ENSG00000178440.5	0	0
lincRNA	AL731537.2	ENSG00000261368.1	0	0
lincRNA	AC073174.2	ENSG00000285958.1	0	0
lincRNA	AC073174.1	ENSG00000227121.1	0	0
lincRNA	AC036101.1	ENSG00000226296.1	0	0
lincRNA	AL355314.1	ENSG00000223800.1	0	0
lincRNA	AL355314.2	ENSG00000228048.1	0	0
lincRNA	AC016396.1	ENSG00000228527.1	0	0
lincRNA	AC016396.2	ENSG00000261076.1	0	0
lincRNA	LINC00844	ENSG00000237949.1	0	0
lincRNA	AC026391.1	ENSG00000235140.2	0	0
lincRNA	LINC01553	ENSG00000235931.2	0	0
lincRNA	AC022390.1	ENSG00000254271.1	0	0
lincRNA	LINC00845	ENSG00000227244.2	0	0
lincRNA	AC022398.1	ENSG00000233643.2	0	0
lincRNA	AC024597.1	ENSG00000234756.1	0	0
lincRNA	AC022387.1	ENSG00000228566.1	0	0
lincRNA	AC012558.1	ENSG00000226426.1	0	0
lincRNA	AC024600.1	ENSG00000224714.2	0	0
lincRNA	AL513321.2	ENSG00000282906.1	0	0
lincRNA	AL592466.1	ENSG00000235356.2	0	0
lincRNA	AL133551.1	ENSG00000272892.1	0	0
lincRNA	AC016395.1	ENSG00000233590.1	0	0
lincRNA	AL359844.1	ENSG00000285871.1	0	0
lincRNA	AL450311.1	ENSG00000235645.2	0	0
lincRNA	AC024601.1	ENSG00000224222.1	0	0
lincRNA	AC073176.3	ENSG00000285300.1	0	0
lincRNA	AC073176.2	ENSG00000271409.1	0	0
lincRNA	AC073176.1	ENSG00000259267.2	0	0
lincRNA	AC022392.1	ENSG00000272988.1	0	0
lincRNA	AC022400.5	ENSG00000272140.2	0	0
lincRNA	AC063962.1	ENSG00000272748.1	0	0
lincRNA	ZNF503-AS1	ENSG00000226051.7	0	0
lincRNA	ZNF503	ENSG00000270087.5	0	0
lincRNA	AL731555.1	ENSG00000283096.1	0	0
lincRNA	AC012560.1	ENSG00000282863.1	0	0
lincRNA	AC010163.1	ENSG00000229543.1	0	0

lincRNA	AC010163.2	ENSG00000230229.1	0	0
lincRNA	AL583852.1	ENSG00000282952.1	0	0
lincRNA	AC016820.1	ENSG00000228683.1	0	0
lincRNA	AL356753.1	ENSG00000229569.1	0	0
lincRNA	AL133481.3	ENSG00000241317.1	0	0
lincRNA	BX248123.1	ENSG00000226676.1	0	0
lincRNA	AL132656.2	ENSG00000244733.5	0	0
lincRNA	AL359195.1	ENSG00000235858.1	0	0
lincRNA	AL731544.1	ENSG00000231082.1	0	0
lincRNA	AC069540.2	ENSG00000233945.1	0	0
lincRNA	AL390786.1	ENSG00000233258.1	0	0
lincRNA	LINC01519	ENSG00000237267.1	0	0
lincRNA	AC025428.1	ENSG00000223993.1	0	0
lincRNA	AC025428.2	ENSG00000224504.2	0	0
lincRNA	LINC01520	ENSG00000230962.1	0	0
lincRNA	LINC00864	ENSG00000228055.2	0	0
lincRNA	AL138767.2	ENSG00000223761.1	0	0
lincRNA	AL138767.3	ENSG00000225913.2	0	0
lincRNA	AL157400.1	ENSG00000225836.1	0	0
lincRNA	LINC01374	ENSG00000280560.1	0	0
lincRNA	LINC01375	ENSG00000226159.1	0	0
lincRNA	AL391704.2	ENSG00000236373.1	0	0
lincRNA	AL391704.3	ENSG00000238291.1	0	0
lincRNA	AL365434.1	ENSG00000225519.1	0	0
lincRNA	AL365434.2	ENSG00000273124.1	0	0
lincRNA	LINC00502	ENSG00000224851.2	0	0
lincRNA	AL358613.2	ENSG00000279607.1	0	0
lincRNA	AL157834.1	ENSG00000231829.2	0	1
lincRNA	LINC00866	ENSG00000227356.2	0	0
lincRNA	LINC01475	ENSG00000257582.5	0	0
lincRNA	AL513542.1	ENSG00000228778.2	0	0
lincRNA	AL353719.1	ENSG00000260475.1	0	0
lincRNA	AL133215.3	ENSG00000273476.1	0	0
lincRNA	TLX1NB	ENSG00000236311.6	0	0
lincRNA	LINC01514	ENSG00000237579.2	0	0
lincRNA	AL133387.2	ENSG00000229649.1	0	0
lincRNA	AL133387.1	ENSG00000225208.1	0	0
lincRNA	AL133387.3	ENSG00000230967.2	0	0
lincRNA	AC010789.1	ENSG00000224817.1	0	0
lincRNA	AL356608.1	ENSG00000272912.1	0	0
lincRNA	AL162742.1	ENSG00000228261.1	0	0
lincRNA	AL161646.1	ENSG00000225768.1	0	0
lincRNA	AL161646.2	ENSG00000237761.2	0	0
lincRNA	AL731574.1	ENSG00000229466.1	0	0

lincRNA	AL353148.1	ENSG00000229775.6	0	0
lincRNA	AL356476.1	ENSG00000223381.1	0	0
lincRNA	AL136368.1	ENSG00000232470.1	0	0
lincRNA	AC021035.1	ENSG00000227851.1	0	0
lincRNA	AC022023.1	ENSG00000285540.1	0	0
lincRNA	AL137025.1	ENSG00000228484.2	0	0
lincRNA	AL355340.1	ENSG00000236799.1	0	0
lincRNA	MIR3663HG	ENSG00000234474.2	0	0
lincRNA	AC005871.2	ENSG00000258114.1	0	0
lincRNA	AC005871.1	ENSG00000235198.1	0	0
lincRNA	AL139121.1	ENSG00000234952.1	0	0
lincRNA	AL513324.1	ENSG00000263041.1	0	0
lincRNA	LINC00867	ENSG00000232139.5	0	0
lincRNA	LINC01561	ENSG00000177234.7	0	0
lincRNA	AC010998.1	ENSG00000227307.1	0	0
lincRNA	AC010998.3	ENSG00000278484.1	0	0
lincRNA	LINC01153	ENSG00000227143.1	0	0
lincRNA	AC009988.1	ENSG00000223432.1	0	0
lincRNA	AL160290.2	ENSG00000230131.5	0	0
lincRNA	AL160290.1	ENSG00000225152.1	0	0
lincRNA	AL357127.1	ENSG00000234542.1	0	0
lincRNA	AL157888.1	ENSG00000282787.1	0	0
lincRNA	LINC00601	ENSG00000235180.1	0	0
lincRNA	AL158166.2	ENSG00000232259.1	0	0
lincRNA	LINC01163	ENSG00000280953.2	0	0
lincRNA	AL390763.1	ENSG00000234640.1	0	0
lincRNA	AL355537.1	ENSG00000224190.1	0	0
lincRNA	AL355537.2	ENSG00000232985.1	0	0
lincRNA	AL391869.1	ENSG00000225424.1	0	0
lincRNA	AL359508.1	ENSG00000285615.1	0	0
lincRNA	AL157832.1	ENSG00000227374.1	0	0
lincRNA	AL157832.3	ENSG00000283141.1	0	0
lincRNA	AC016816.1	ENSG00000285529.1	0	0
lincRNA	LINC01164	ENSG00000189275.3	0	0
lincRNA	FP565171.1	ENSG00000283095.1	0	0
lincRNA	AL451069.1	ENSG00000226900.1	0	0
lincRNA	LINC01166	ENSG00000232903.2	0	0
lincRNA	LINC01167	ENSG00000224758.1	0	0
lincRNA	LINC01168	ENSG00000240707.2	0	0
lincRNA	AL360181.4	ENSG00000274685.1	0	0
lincRNA	AC069287.1	ENSG00000254468.2	0	0
lincRNA	LINC01001	ENSG00000230724.9	0	0
lincRNA	AC136475.8	ENSG00000270105.1	0	0
lincRNA	AC136475.7	ENSG00000270030.1	0	0

lincRNA	AC136475.9	ENSG00000270972.1	0	0
lincRNA	AP006621.2	ENSG00000255142.1	0	0
lincRNA	AC139749.1	ENSG00000254872.3	0	0
lincRNA	AC136297.1	ENSG00000285985.1	0	0
lincRNA	AC091196.1	ENSG00000285581.1	0	0
lincRNA	AP006285.2	ENSG00000231487.1	0	0
lincRNA	FAM99A	ENSG00000205866.3	0	0
lincRNA	AP006285.1	ENSG00000227306.1	0	0
lincRNA	AC068580.2	ENSG00000230834.1	0	0
lincRNA	LINC01150	ENSG00000229671.2	0	0
lincRNA	LINC01219	ENSG00000232987.2	0	0
lincRNA	AC127526.1	ENSG00000254757.1	0	0
lincRNA	AC015689.1	ENSG00000254480.1	0	1
lincRNA	AC113331.1	ENSG00000236897.1	0	0
lincRNA	BGLT3	ENSG00000260629.2	0	0
lincRNA	AC068733.1	ENSG00000254443.1	0	0
lincRNA	AC027804.1	ENSG00000255179.1	0	0
lincRNA	AC044810.3	ENSG00000271758.2	0	0
lincRNA	AC091013.1	ENSG00000246820.2	0	0
lincRNA	AC111188.1	ENSG00000254401.2	0	0
lincRNA	KC877392.1	ENSG00000255260.1	0	0
lincRNA	LINC02547	ENSG00000254486.1	0	0
lincRNA	AC124276.1	ENSG00000254991.1	0	0
lincRNA	AC009806.1	ENSG00000254847.1	0	0
lincRNA	AC084859.1	ENSG00000254670.1	0	0
lincRNA	AC013762.1	ENSG00000255558.1	0	0
lincRNA	LINC02548	ENSG00000255411.2	0	0
lincRNA	AC027779.1	ENSG00000254927.1	0	0
lincRNA	LINC02545	ENSG00000254930.1	0	0
lincRNA	AC022240.1	ENSG00000254438.1	0	0
lincRNA	AC073172.2	ENSG00000254946.1	0	0
lincRNA	AC073172.1	ENSG00000254789.2	0	0
lincRNA	AC087379.2	ENSG00000254695.1	0	0
lincRNA	AC087379.1	ENSG00000254645.1	0	0
lincRNA	AC009869.1	ENSG00000254661.2	0	0
lincRNA	AC124301.1	ENSG00000254586.1	0	0
lincRNA	AC090099.4	ENSG00000255470.1	0	0
lincRNA	AC090099.5	ENSG00000285562.1	0	0
lincRNA	AC009652.1	ENSG00000255160.5	0	0
lincRNA	AC107882.1	ENSG00000254820.1	0	0
lincRNA	LINC01495	ENSG00000255323.5	0	0
lincRNA	AC055878.1	ENSG00000255357.1	0	0
lincRNA	AC090092.1	ENSG00000255418.5	0	0
lincRNA	AC100768.2	ENSG00000255193.1	0	0

lincRNA	AC100768.1	ENSG00000254861.1	0	0
lincRNA	AC015771.1	ENSG00000254594.5	0	0
lincRNA	AC090592.1	ENSG00000254456.5	0	0
lincRNA	AC013799.1	ENSG00000254754.1	0	0
lincRNA	AC099687.1	ENSG00000255243.1	0	0
lincRNA	AC021698.1	ENSG00000254819.1	0	0
lincRNA	LINC00678	ENSG00000254934.5	0	0
lincRNA	AC090159.1	ENSG00000255094.1	0	0
lincRNA	AC013714.1	ENSG00000254606.1	0	0
lincRNA	AC100773.1	ENSG00000248990.1	0	0
lincRNA	AC090124.1	ENSG00000254530.1	0	3
lincRNA	AC090124.2	ENSG00000255227.1	0	0
lincRNA	AC110058.1	ENSG00000255029.1	0	0
lincRNA	LINC02546	ENSG00000255117.5	0	0
lincRNA	AC107973.1	ENSG00000254734.1	0	0
lincRNA	LINC01616	ENSG00000261340.1	0	0
lincRNA	AC124657.1	ENSG00000254532.1	0	0
lincRNA	AC113192.4	ENSG00000255169.1	0	0
lincRNA	AC113192.1	ENSG00000254619.1	0	0
lincRNA	AC113192.2	ENSG00000255133.1	0	0
lincRNA	AL137224.1	ENSG00000255271.1	0	0
lincRNA	AC087783.1	ENSG00000255427.1	0	0
lincRNA	AL135934.1	ENSG00000254669.1	0	0
lincRNA	AL138812.1	ENSG00000254686.2	0	0
lincRNA	AC090692.2	ENSG00000285705.1	0	0
lincRNA	AC090692.3	ENSG00000285740.1	0	0
lincRNA	AC087277.1	ENSG00000254566.1	0	0
lincRNA	AC103798.1	ENSG00000254516.1	0	0
lincRNA	AC021713.1	ENSG00000255477.1	0	0
lincRNA	AC022796.1	ENSG00000255175.5	0	0
lincRNA	LINC01493	ENSG00000254562.6	0	0
lincRNA	AC021723.2	ENSG00000285751.1	0	0
lincRNA	AC090138.1	ENSG00000255132.1	0	0
lincRNA	AC021006.2	ENSG00000255388.5	0	0
lincRNA	AC023442.3	ENSG00000255279.1	0	0
lincRNA	AC023442.2	ENSG00000254822.1	0	0
lincRNA	AC079917.1	ENSG00000255300.5	0	0
lincRNA	AC090458.1	ENSG00000255109.5	0	0
lincRNA	AC068205.1	ENSG00000283217.1	0	0
lincRNA	MIR670HG	ENSG00000235661.3	0	0
lincRNA	AC087521.1	ENSG00000244953.1	0	0
lincRNA	AC010768.4	ENSG00000255451.1	0	0
lincRNA	AC104241.2	ENSG00000255079.1	0	0
lincRNA	AC104241.1	ENSG00000255032.1	0	0

lincRNA	AC068858.1	ENSG00000254654.1	0	0
lincRNA	AC018716.2	ENSG00000255267.3	0	0
lincRNA	AC103855.3	ENSG00000255041.1	0	0
lincRNA	AC018716.1	ENSG00000254651.1	0	0
lincRNA	AC103855.1	ENSG00000254514.1	0	0
lincRNA	AC044839.3	ENSG00000255426.1	0	0
lincRNA	AC044839.2	ENSG00000255226.1	0	0
lincRNA	DKFZp779M0652	ENSG00000205106.4	0	0
lincRNA	AC044839.4	ENSG00000255447.1	0	0
lincRNA	AC024475.1	ENSG00000254653.1	0	0
lincRNA	AC024475.4	ENSG00000255314.3	0	0
lincRNA	AC024475.3	ENSG00000255269.2	0	0
lincRNA	AC116021.1	ENSG00000254639.1	0	0
lincRNA	AC109635.2	ENSG00000254518.1	0	0
lincRNA	AP006437.1	ENSG00000254804.1	0	0
lincRNA	AP001803.2	ENSG00000255433.5	0	0
lincRNA	AP003557.2	ENSG00000285895.1	0	0
lincRNA	AP003557.1	ENSG00000255299.5	0	0
lincRNA	AP002358.1	ENSG00000214797.3	0	0
lincRNA	AP000442.1	ENSG00000255008.2	0	0
lincRNA	AP001257.1	ENSG00000254952.1	0	0
lincRNA	AP003721.2	ENSG00000256733.1	0	0
lincRNA	AP003108.1	ENSG00000255931.1	0	0
lincRNA	AP003559.1	ENSG00000256443.1	0	0
lincRNA	AP002754.1	ENSG00000250230.2	0	0
lincRNA	AP002754.2	ENSG00000255947.1	0	0
lincRNA	DKFZP434K0	ENSG00000124915.10	0	0
lincRNA	AP003733.3	ENSG00000255553.1	0	2
lincRNA	AP002793.1	ENSG00000285656.1	0	0
lincRNA	AP001453.3	ENSG00000257086.1	0	0
lincRNA	AP003774.3	ENSG00000231680.1	0	0
lincRNA	AP003774.2	ENSG00000231492.2	0	0
lincRNA	AP003774.1	ENSG00000181908.5	0	0
lincRNA	AP005273.1	ENSG00000232500.1	0	0
lincRNA	MIR194-2HG	ENSG00000229719.5	0	0
lincRNA	AP000769.3	ENSG00000279243.2	0	0
lincRNA	AP000769.2	ENSG00000270117.1	0	0
lincRNA	AP001266.2	ENSG00000255557.1	0	0
lincRNA	AP002748.1	ENSG00000250105.1	0	0
lincRNA	AP003419.2	ENSG00000274251.1	0	0
lincRNA	AP003419.3	ENSG00000275484.1	0	0
lincRNA	C11orf72	ENSG00000184224.3	0	1

lincRNA	AP003096.1	ENSG00000260808.1	0	0
lincRNA	AP003071.1	ENSG00000260895.1	0	0
lincRNA	AP003071.2	ENSG00000261070.1	0	0
lincRNA	AP005233.2	ENSG00000260877.2	0	0
lincRNA	AP005233.1	ENSG00000259854.1	0	0
lincRNA	AP000439.3	ENSG00000255980.1	0	0
lincRNA	AP000439.1	ENSG00000255606.1	0	0
lincRNA	AP000439.4	ENSG00000261347.1	0	0
lincRNA	AP000439.2	ENSG00000255774.2	0	0
lincRNA	LINC01488	ENSG00000285094.1	0	0
lincRNA	AP007216.1	ENSG00000260348.1	0	0
lincRNA	AP007216.2	ENSG00000260679.1	0	0
lincRNA	AP003555.3	ENSG00000255191.1	0	0
lincRNA	AP003555.2	ENSG00000254605.1	0	0
lincRNA	AP003555.1	ENSG00000248844.6	0	0
lincRNA	AP003498.1	ENSG00000254972.1	0	0
lincRNA	AP000593.4	ENSG00000285864.1	0	0
lincRNA	LINC01537	ENSG00000227467.3	0	0
lincRNA	AP002761.2	ENSG00000256568.1	0	0
lincRNA	P4HA3-AS1	ENSG00000246211.2	0	0
lincRNA	AP001972.3	ENSG00000255136.2	0	0
lincRNA	AP000785.2	ENSG00000255362.1	0	0
lincRNA	AP002360.2	ENSG00000271757.1	0	0
lincRNA	AP001189.5	ENSG00000255363.2	0	0
lincRNA	AP001189.6	ENSG00000255479.1	0	0
lincRNA	AP001189.3	ENSG00000254810.1	0	0
lincRNA	AP001189.4	ENSG00000254975.1	0	0
lincRNA	AP002957.1	ENSG00000255345.1	0	0
lincRNA	AP001547.1	ENSG00000254885.1	0	0
lincRNA	AP001978.1	ENSG00000255209.1	0	0
lincRNA	AP006295.1	ENSG00000254434.1	0	0
lincRNA	AP002759.1	ENSG00000255178.1	0	0
lincRNA	AP003398.2	ENSG00000285568.1	0	0
lincRNA	MIR4300HG	ENSG00000245832.6	0	0
lincRNA	AP002802.1	ENSG00000254511.1	0	0
lincRNA	AP000793.1	ENSG00000255246.1	0	0
lincRNA	AP001646.3	ENSG00000255382.1	0	0
lincRNA	AP001767.3	ENSG00000255503.1	0	0
lincRNA	AP003097.2	ENSG00000254699.1	0	0
lincRNA	AP003059.1	ENSG00000254731.1	0	0
lincRNA	AP003059.2	ENSG00000255250.1	0	0
lincRNA	AP000654.1	ENSG00000269895.1	0	0
lincRNA	AP000756.1	ENSG00000255183.1	0	0
lincRNA	AP000676.5	ENSG00000285835.1	0	0

lincRNA	AP005436.3	ENSG00000255516.1	0	0
lincRNA	AP005436.1	ENSG00000255102.1	0	0
lincRNA	AP003400.1	ENSG00000255429.1	0	0
lincRNA	AP003122.2	ENSG00000254971.1	0	0
lincRNA	AP003386.1	ENSG00000255187.1	0	0
lincRNA	AP003028.1	ENSG00000255332.7	0	0
lincRNA	AP003171.3	ENSG00000282916.1	0	0
lincRNA	AP003171.1	ENSG00000254874.1	0	0
lincRNA	AP003969.1	ENSG00000255233.1	0	0
lincRNA	AP002784.1	ENSG00000250519.6	0	0
lincRNA	AP000943.1	ENSG00000233536.2	0	0
lincRNA	AP000820.2	ENSG00000285842.1	0	0
lincRNA	AP000820.1	ENSG00000255605.1	0	0
lincRNA	AP000779.1	ENSG00000285921.1	0	0
lincRNA	JRKL-AS1	ENSG00000255679.1	0	0
lincRNA	AP003781.1	ENSG00000256684.1	0	0
lincRNA	AP003066.1	ENSG00000254587.1	0	0
lincRNA	LINC02553	ENSG00000255039.1	0	0
lincRNA	AP003730.2	ENSG00000255502.5	0	0
lincRNA	AP003715.1	ENSG00000254599.1	0	0
lincRNA	AP002428.1	ENSG00000254830.1	0	0
lincRNA	AP001527.1	ENSG00000260008.1	0	2
lincRNA	AP000851.2	ENSG00000281655.1	0	0
lincRNA	AP002989.1	ENSG00000254987.1	0	0
lincRNA	LINC02552	ENSG00000256422.5	0	0
lincRNA	AP001001.2	ENSG00000254580.1	0	0
lincRNA	AP003027.1	ENSG00000203334.4	0	0
lincRNA	AP003123.1	ENSG00000255528.1	0	0
lincRNA	AP002444.1	ENSG00000254659.2	0	0
lincRNA	AP000924.1	ENSG00000254416.5	0	0
lincRNA	AP003973.2	ENSG00000271390.1	0	0
lincRNA	AP003063.1	ENSG00000254968.6	0	0
lincRNA	AP003063.2	ENSG00000255484.2	0	0
lincRNA	AP003100.2	ENSG00000285769.1	0	0
lincRNA	AP003100.1	ENSG00000254626.1	0	0
lincRNA	AP000462.3	ENSG00000256972.1	0	0
lincRNA	AP000462.1	ENSG00000255580.1	0	0
lincRNA	AP003174.2	ENSG00000256281.1	0	0
lincRNA	AP003174.1	ENSG00000255689.1	0	0
lincRNA	AP000997.1	ENSG00000255599.1	0	0
lincRNA	AP000997.2	ENSG00000260254.1	0	0
lincRNA	AP002991.1	ENSG00000256717.1	0	0
lincRNA	AP000797.3	ENSG00000257067.5	0	0
lincRNA	LINC02151	ENSG00000236437.1	0	0

lincRNA	AP000770.1	ENSG00000237937.5	0	0
lincRNA	AP001554.1	ENSG00000270403.1	0	0
lincRNA	AP001267.2	ENSG00000255384.1	0	0
lincRNA	AP003392.5	ENSG00000271751.1	0	0
lincRNA	AP003390.2	ENSG00000255247.1	0	0
lincRNA	AP001360.1	ENSG00000254863.1	0	0
lincRNA	AP001360.2	ENSG00000255216.1	0	0
lincRNA	AP000755.1	ENSG00000255015.1	0	0
lincRNA	AP001782.1	ENSG00000254823.1	0	0
lincRNA	AP002765.1	ENSG00000254467.1	0	0
lincRNA	AP001804.1	ENSG00000285561.1	0	0
lincRNA	AP003501.3	ENSG00000285825.1	0	0
lincRNA	AP001007.2	ENSG00000255475.1	0	0
lincRNA	PKNOX2-	ENSG00000254880.1	0	0
lincRNA	AP000842.3	ENSG00000260209.1	0	0
lincRNA	AP000842.1	ENSG00000254790.1	0	0
lincRNA	AP000821.1	ENSG00000261257.1	0	0
lincRNA	AP001893.3	ENSG00000254833.1	0	0
lincRNA	AP002833.1	ENSG00000254938.1	0	0
lincRNA	AP001993.1	ENSG00000255087.3	0	0
lincRNA	AP003121.1	ENSG00000272642.2	0	0
lincRNA	AP003481.1	ENSG00000273409.2	0	0
lincRNA	AP000904.1	ENSG00000273415.2	0	0
lincRNA	LINC02098	ENSG00000272575.6	0	0
lincRNA	AP003500.1	ENSG00000281386.1	0	0
lincRNA	LINC01395	ENSG00000281097.1	0	0
lincRNA	LINC00167	ENSG00000233220.3	0	0
lincRNA	AP002986.1	ENSG00000175773.13	0	0
lincRNA	AP002856.3	ENSG00000236129.1	0	0
lincRNA	AP002856.1	ENSG00000227125.1	0	0
lincRNA	AP002856.4	ENSG00000237612.1	0	0
lincRNA	AP002856.2	ENSG00000231698.2	0	1
lincRNA	NTM-AS1	ENSG00000224795.1	0	0
lincRNA	AP001979.2	ENSG00000255258.2	0	0
lincRNA	AP000911.2	ENSG00000254648.1	0	0
lincRNA	AP004550.1	ENSG00000254573.1	0	0
lincRNA	AP001999.2	ENSG00000254989.1	0	0
lincRNA	AP001999.4	ENSG00000285640.1	0	0
lincRNA	AP001999.3	ENSG00000255344.1	0	0
lincRNA	AP001999.1	ENSG00000251226.1	0	0
lincRNA	AP003062.1	ENSG00000255327.1	0	0
lincRNA	AP003062.3	ENSG00000285735.1	0	0
lincRNA	AP005135.1	ENSG00000255512.1	0	0
lincRNA	FAM138D	ENSG00000249054.2	0	0

lincRNA	AC007406.3	ENSG00000256540.1	0	0
lincRNA	LINC02455	ENSG00000256672.1	0	0
lincRNA	LINC00940	ENSG00000235049.1	0	0
lincRNA	LINC02371	ENSG00000274659.1	0	0
lincRNA	AC005865.1	ENSG00000236908.2	0	0
lincRNA	LINC02417	ENSG00000257048.1	0	0
lincRNA	AC007207.2	ENSG00000256969.1	0	0
lincRNA	GAU1	ENSG00000255474.1	0	0
lincRNA	AC005833.2	ENSG00000256988.1	0	0
lincRNA	AC006063.2	ENSG00000284513.1	0	0
lincRNA	AC005906.3	ENSG00000285523.1	0	0
lincRNA	LINC02443	ENSG00000256115.5	0	0
lincRNA	AC007848.2	ENSG00000256218.1	0	0
lincRNA	AC005845.1	ENSG00000255775.1	0	0
lincRNA	AC005840.2	ENSG00000256433.2	0	1
lincRNA	AC233309.1	ENSG00000276144.1	0	0
lincRNA	AC006511.4	ENSG00000282080.1	0	0
lincRNA	AC092111.2	ENSG00000276417.1	0	0
lincRNA	LINC02449	ENSG00000215241.3	0	0
lincRNA	LINC00937	ENSG00000226091.7	0	0
lincRNA	AC092745.1	ENSG00000256101.5	0	0
lincRNA	AC092746.1	ENSG00000255801.1	0	0
lincRNA	AC092490.1	ENSG00000249790.2	0	0
lincRNA	AC010175.1	ENSG00000256427.2	0	0
lincRNA	LINC02390	ENSG00000256582.1	0	0
lincRNA	CLEC12A-	ENSG00000231560.1	0	0
lincRNA	AC022075.2	ENSG00000256288.1	0	0
lincRNA	LINC02598	ENSG00000256155.1	0	0
lincRNA	LINC02446	ENSG00000256039.1	0	0
lincRNA	LINC02366	ENSG00000256888.5	0	0
lincRNA	AC244131.2	ENSG00000275119.1	0	0
lincRNA	AC007450.1	ENSG00000256237.1	0	0
lincRNA	AC007619.1	ENSG00000255670.1	0	1
lincRNA	AC008115.2	ENSG00000257004.1	0	0
lincRNA	LINC01559	ENSG00000180861.9	0	0
lincRNA	AC007527.2	ENSG00000277935.1	0	0
lincRNA	AC092112.1	ENSG00000256084.1	0	0
lincRNA	LINC01489	ENSG00000255727.1	0	0
lincRNA	RERG-AS1	ENSG00000255660.1	0	0
lincRNA	AC073651.1	ENSG00000255565.1	0	0
lincRNA	AC007529.2	ENSG00000283240.1	0	0
lincRNA	AC092110.1	ENSG00000285724.1	0	0
lincRNA	LINC02378	ENSG00000256994.1	0	0
lincRNA	AC087321.1	ENSG00000256389.1	0	0

lincRNA	AC024901.1	ENSG00000255910.1	0	0
lincRNA	LINC02398	ENSG00000256287.1	0	0
lincRNA	LINC02468	ENSG00000256499.1	0	0
lincRNA	AC087318.1	ENSG00000256714.1	0	0
lincRNA	AC087241.1	ENSG00000250166.2	0	0
lincRNA	AC084816.1	ENSG00000256995.6	0	1
lincRNA	AC084819.1	ENSG00000256923.1	0	0
lincRNA	AC087258.1	ENSG00000256321.5	0	0
lincRNA	AC087235.1	ENSG00000115934.11	0	0
lincRNA	SOX5-AS1	ENSG00000256120.1	0	0
lincRNA	LINC00477	ENSG00000197503.4	0	0
lincRNA	AC023796.1	ENSG00000255745.1	0	0
lincRNA	C12orf77	ENSG00000226397.8	0	0
lincRNA	AC087239.1	ENSG00000278743.1	0	0
lincRNA	AC019209.3	ENSG00000256686.1	0	0
lincRNA	AC019209.2	ENSG00000256440.1	0	0
lincRNA	AC019209.1	ENSG00000256256.1	0	0
lincRNA	AC055720.1	ENSG00000255858.1	0	0
lincRNA	AC092747.1	ENSG00000256226.1	0	0
lincRNA	AC087257.1	ENSG00000248100.2	0	0
lincRNA	AC009509.2	ENSG00000256504.2	0	0
lincRNA	AC022081.1	ENSG00000256513.1	0	0
lincRNA	AC012150.1	ENSG00000257258.1	0	0
lincRNA	AC023511.1	ENSG00000257262.1	0	0
lincRNA	LINC02386	ENSG00000257756.5	0	0
lincRNA	AC078776.1	ENSG00000257932.1	0	0
lincRNA	AC012673.1	ENSG00000285870.1	0	1
lincRNA	LINC00941	ENSG00000235884.4	0	0
lincRNA	LINC02387	ENSG00000256232.1	0	0
lincRNA	LINC02422	ENSG00000255760.1	0	0
lincRNA	AC087311.2	ENSG00000257435.1	0	0
lincRNA	AC046130.2	ENSG00000256538.1	0	0
lincRNA	AC140847.1	ENSG00000255628.1	0	0
lincRNA	AC140847.2	ENSG00000255652.3	0	0
lincRNA	AC087897.2	ENSG00000258119.1	0	0
lincRNA	LINC02406	ENSG00000258144.1	0	0
lincRNA	LINC02555	ENSG00000260943.1	0	0
lincRNA	LINC02400	ENSG00000257784.1	0	0
lincRNA	AC090630.1	ENSG00000257239.1	0	0
lincRNA	LINC02402	ENSG00000257510.1	0	0
lincRNA	LINC02451	ENSG00000257164.5	0	0
lincRNA	AC012038.2	ENSG00000257849.1	0	0
lincRNA	AC012038.1	ENSG00000257373.1	0	0
lincRNA	LINC02461	ENSG00000258331.1	0	0

lincRNA	AC025253.1	ENSG00000275286.1	0	0
lincRNA	AC079950.1	ENSG00000257657.2	0	0
lincRNA	AC025031.1	ENSG00000257496.1	0	0
lincRNA	AC079906.1	ENSG00000274723.1	0	0
lincRNA	AC008083.2	ENSG00000258181.1	0	0
lincRNA	AC008083.3	ENSG00000276454.1	0	0
lincRNA	LINC02416	ENSG00000257924.1	0	0
lincRNA	LINC02156	ENSG00000257906.2	0	0
lincRNA	AC004241.2	ENSG00000274902.1	0	0
lincRNA	LINC02354	ENSG00000257488.5	0	0
lincRNA	AC004801.5	ENSG00000258203.5	0	0
lincRNA	AC004801.3	ENSG00000257955.1	0	0
lincRNA	AC024257.1	ENSG00000258234.1	0	0
lincRNA	AC024257.5	ENSG00000275228.1	0	0
lincRNA	AC024257.4	ENSG00000273765.1	0	0
lincRNA	AC131157.1	ENSG00000257253.2	0	0
lincRNA	LINC02396	ENSG00000258135.5	0	0
lincRNA	LINC02395	ENSG00000257771.5	0	0
lincRNA	AC008121.2	ENSG00000274156.1	0	0
lincRNA	AC068987.2	ENSG00000260122.1	0	0
lincRNA	AC068987.3	ENSG00000260473.2	0	0
lincRNA	AC068987.4	ENSG00000261586.2	0	0
lincRNA	AC068987.1	ENSG00000259887.1	0	0
lincRNA	AC025259.3	ENSG00000259884.1	0	0
lincRNA	AC078864.1	ENSG00000257989.1	0	0
lincRNA	C12orf80	ENSG00000257137.6	0	0
lincRNA	AC073573.1	ENSG00000257808.1	0	0
lincRNA	CISTR	ENSG00000260492.2	0	0
lincRNA	AC076968.1	ENSG00000260030.1	0	0
lincRNA	AC012531.1	ENSG00000260597.1	0	0
lincRNA	FLJ12825	ENSG00000248265.1	0	0
lincRNA	AC023794.2	ENSG00000249388.1	0	0
lincRNA	FAM242C	ENSG00000250432.5	0	0
lincRNA	LINC02381	ENSG00000250742.2	0	0
lincRNA	SMUG1-AS1	ENSG00000283073.1	0	0
lincRNA	AC023794.5	ENSG00000260470.1	0	0
lincRNA	AC023794.4	ENSG00000257534.1	0	0
lincRNA	LINC01154	ENSG00000257477.1	0	0
lincRNA	AC079310.1	ENSG00000257634.1	0	0
lincRNA	AC027287.2	ENSG00000257870.1	0	0
lincRNA	AC126614.1	ENSG00000258001.1	0	0
lincRNA	AC025165.4	ENSG00000269903.1	0	0
lincRNA	LINC02403	ENSG00000257541.1	0	0
lincRNA	LINC02388	ENSG00000257259.2	0	0

lincRNA	AC108721.1	ENSG00000258214.1	0	0
lincRNA	LINC02448	ENSG00000257230.1	0	0
lincRNA	AC080011.1	ENSG00000257294.2	0	0
lincRNA	LINC01465	ENSG00000221949.5	0	1
lincRNA	AC078814.1	ENSG00000257235.1	0	0
lincRNA	AC026116.1	ENSG00000258117.2	0	0
lincRNA	LINC02389	ENSG00000255693.2	0	0
lincRNA	LINC02231	ENSG00000248995.2	0	0
lincRNA	AC025419.1	ENSG00000250748.6	0	0
lincRNA	AC090023.1	ENSG00000255866.1	0	0
lincRNA	AC090023.2	ENSG00000256915.1	0	0
lincRNA	LINC02425	ENSG00000256259.1	0	0
lincRNA	AC073530.1	ENSG00000257083.5	0	0
lincRNA	LINC02420	ENSG00000256172.1	0	0
lincRNA	LINC02408	ENSG00000203585.4	0	0
lincRNA	LINC02442	ENSG00000256077.5	0	0
lincRNA	LINC02421	ENSG00000255970.1	0	0
lincRNA	LINC01479	ENSG00000255772.5	0	0
lincRNA	LINC02384	ENSG00000251301.6	0	0
lincRNA	AC022511.1	ENSG00000256273.1	0	0
lincRNA	AC008033.3	ENSG00000273824.1	0	0
lincRNA	AC127894.1	ENSG00000258140.2	0	0
lincRNA	AC020656.2	ENSG00000274979.1	0	0
lincRNA	LINC02373	ENSG00000257507.1	0	0
lincRNA	AC084032.1	ENSG00000257139.1	0	0
lincRNA	LINC01481	ENSG00000257613.1	0	1
lincRNA	AC123905.1	ENSG00000257265.1	0	0
lincRNA	AC025575.1	ENSG00000257454.1	0	0
lincRNA	AC011601.1	ENSG00000257515.1	0	0
lincRNA	AC089984.1	ENSG00000257410.1	0	0
lincRNA	AC133480.1	ENSG00000258235.1	0	0
lincRNA	LINC02444	ENSG00000258123.1	0	0
lincRNA	AC090503.1	ENSG00000257682.1	0	0
lincRNA	LINC02445	ENSG00000257750.1	0	0
lincRNA	AC136188.1	ENSG00000257587.1	0	0
lincRNA	LINC02394	ENSG00000258332.2	0	0
lincRNA	AC090502.1	ENSG00000251138.6	0	0
lincRNA	AC090502.3	ENSG00000257183.1	0	0
lincRNA	AC078923.1	ENSG00000258077.2	0	0
lincRNA	AC078820.1	ENSG00000258088.1	0	0
lincRNA	AC011611.2	ENSG00000257329.1	0	0
lincRNA	LINC02407	ENSG00000257219.5	0	0
lincRNA	AC107032.2	ENSG00000257526.2	0	0
lincRNA	LINC02464	ENSG00000257677.1	0	0

lincRNA	AC073591.1	ENSG00000257835.1	0	0
lincRNA	AC138331.1	ENSG00000258066.1	0	1
lincRNA	LINC02424	ENSG00000258220.1	0	0
lincRNA	AC128707.1	ENSG00000258084.5	0	0
lincRNA	AC130415.1	ENSG00000257879.1	0	0
lincRNA	AC079362.1	ENSG00000257165.5	0	0
lincRNA	AC068993.2	ENSG00000257948.1	0	0
lincRNA	AC027288.1	ENSG00000257474.5	0	0
lincRNA	AC073569.2	ENSG00000258048.1	0	0
lincRNA	LINC01490	ENSG00000257741.5	0	0
lincRNA	PPFIA2-AS1	ENSG00000257467.5	0	0
lincRNA	LINC02426	ENSG00000257747.1	0	0
lincRNA	AC089998.4	ENSG00000258170.1	0	0
lincRNA	AC089998.2	ENSG00000257837.1	0	0
lincRNA	AC089998.1	ENSG00000257431.1	0	0
lincRNA	AC093025.1	ENSG00000257124.2	0	0
lincRNA	AC090679.1	ENSG00000257729.2	0	0
lincRNA	AC131532.1	ENSG00000258815.1	0	0
lincRNA	AC016993.1	ENSG00000258178.1	0	0
lincRNA	AC010196.1	ENSG00000258185.2	0	0
lincRNA	LINC02258	ENSG00000257912.1	0	0
lincRNA	AC079598.1	ENSG00000257940.1	0	0
lincRNA	AC024941.1	ENSG00000278011.1	0	0
lincRNA	AC024909.1	ENSG00000257156.1	0	0
lincRNA	AC010201.1	ENSG00000271259.1	0	0
lincRNA	AC084200.1	ENSG00000258216.5	0	0
lincRNA	LINC02399	ENSG00000257725.1	0	0
lincRNA	AC126178.1	ENSG00000257194.2	0	0
lincRNA	LINC02392	ENSG00000258183.5	0	0
lincRNA	AC084365.1	ENSG00000257995.1	0	0
lincRNA	AC112481.1	ENSG00000257787.1	0	0
lincRNA	LINC00615	ENSG00000196243.5	0	0
lincRNA	AC025035.1	ENSG00000258100.1	0	0
lincRNA	AC025254.1	ENSG00000258125.1	0	0
lincRNA	LINC02404	ENSG00000257893.2	0	0
lincRNA	AC090049.1	ENSG00000257863.1	0	0
lincRNA	LINC02397	ENSG00000205056.8	0	0
lincRNA	LINC02413	ENSG00000257345.2	0	0
lincRNA	AC138123.2	ENSG00000257746.1	0	0
lincRNA	LINC02412	ENSG00000258171.1	0	0
lincRNA	CEP83-DT	ENSG00000278916.1	0	0
lincRNA	AC023161.1	ENSG00000257400.1	0	0
lincRNA	AC084879.2	ENSG00000258313.1	0	0
lincRNA	LINC02410	ENSG00000258292.1	0	0

lincRNA	LINC02452	ENSG00000283422.1	0	0
lincRNA	AC007513.1	ENSG00000258272.1	0	0
lincRNA	AC007656.1	ENSG00000258131.1	0	0
lincRNA	AC013417.1	ENSG00000257470.1	0	0
lincRNA	LINC02409	ENSG00000257920.1	0	0
lincRNA	AC016152.1	ENSG00000258312.1	0	0
lincRNA	AC063948.1	ENSG00000257543.1	0	0
lincRNA	AC010205.1	ENSG00000274560.1	0	0
lincRNA	AC079907.2	ENSG00000258308.5	0	0
lincRNA	LINC00485	ENSG00000258169.1	0	0
lincRNA	AC068643.1	ENSG00000257703.5	0	0
lincRNA	AC068643.2	ENSG00000257860.5	0	0
lincRNA	LINC02401	ENSG00000257762.5	0	0
lincRNA	AC012555.1	ENSG00000257327.1	0	0
lincRNA	LINC02385	ENSG00000257193.1	0	0
lincRNA	CASC18	ENSG00000257859.1	0	0
lincRNA	AC011595.2	ENSG00000257890.1	0	0
lincRNA	AC126177.2	ENSG00000257141.1	0	0
lincRNA	AC126177.8	ENSG00000274395.1	0	0
lincRNA	AC126177.4	ENSG00000257398.6	0	0
lincRNA	LINC01486	ENSG00000256560.1	0	0
lincRNA	AC007834.1	ENSG00000249094.2	0	0
lincRNA	AC002375.1	ENSG00000257268.1	0	0
lincRNA	LINC01405	ENSG00000185847.7	0	0
lincRNA	AC002351.1	ENSG00000258240.5	0	0
lincRNA	LINC01234	ENSG00000249550.6	0	0
lincRNA	AC010183.1	ENSG00000257603.1	0	0
lincRNA	AC010183.2	ENSG00000257997.5	0	0
lincRNA	LINC02459	ENSG00000257476.1	0	0
lincRNA	AC010177.1	ENSG00000258254.1	0	0
lincRNA	AC026765.2	ENSG00000257817.1	0	2
lincRNA	AC026765.1	ENSG00000257517.1	0	0
lincRNA	AC009804.2	ENSG00000283459.1	0	0
lincRNA	AC008125.1	ENSG00000257958.1	0	0
lincRNA	AC078880.3	ENSG00000271579.1	0	0
lincRNA	AC078880.4	ENSG00000276308.1	0	0
lincRNA	AC078880.5	ENSG00000276972.1	0	0
lincRNA	AC078880.2	ENSG00000257726.1	0	0
lincRNA	AC009803.2	ENSG00000258108.1	0	0
lincRNA	AC009803.3	ENSG00000258280.1	0	0
lincRNA	AC009803.1	ENSG00000257407.1	0	0
lincRNA	AC009387.1	ENSG00000257781.1	0	0
lincRNA	LINC02463	ENSG00000257683.1	0	0
lincRNA	AC079384.1	ENSG00000258346.1	0	0

lincRNA	LINC02457	ENSG00000258018.1	0	0
lincRNA	AC125603.4	ENSG00000275898.1	0	0
lincRNA	AC125603.3	ENSG00000258249.1	0	0
lincRNA	AC125603.2	ENSG00000257883.1	0	0
lincRNA	AC125603.1	ENSG00000257654.1	0	0
lincRNA	AC083806.3	ENSG00000275467.1	0	0
lincRNA	TESC-AS1	ENSG00000258285.1	0	0
lincRNA	AC073864.1	ENSG00000255686.1	0	0
lincRNA	LINC02460	ENSG00000255714.1	0	0
lincRNA	LINC02423	ENSG00000256750.1	0	0
lincRNA	LINC02440	ENSG00000255618.1	0	0
lincRNA	LINC02439	ENSG00000255814.1	0	0
lincRNA	AC087863.1	ENSG00000256149.1	0	0
lincRNA	LINC00934	ENSG00000281196.1	0	0
lincRNA	AC002070.1	ENSG00000248636.6	0	0
lincRNA	AC003982.1	ENSG00000256695.1	0	0
lincRNA	AC063943.1	ENSG00000278344.1	0	2
lincRNA	AC069209.1	ENSG00000274029.1	0	0
lincRNA	AC048337.1	ENSG00000258435.1	0	0
lincRNA	AC145422.1	ENSG00000256742.1	0	0
lincRNA	AC084018.1	ENSG00000272849.1	0	0
lincRNA	AC069503.3	ENSG00000278084.1	0	0
lincRNA	CLIP1-AS1	ENSG00000257097.1	0	0
lincRNA	AC127002.1	ENSG00000275265.1	0	0
lincRNA	AC026333.4	ENSG00000274191.1	0	0
lincRNA	AC026333.3	ENSG00000256249.1	0	0
lincRNA	AC027290.1	ENSG00000256152.2	0	0
lincRNA	PITPNM2-	ENSG00000251497.2	0	0
lincRNA	AC117503.1	ENSG00000255839.1	0	0
lincRNA	AC026358.1	ENSG00000256596.1	0	0
lincRNA	AC122688.2	ENSG00000256814.1	0	0
lincRNA	LINC00939	ENSG00000249267.6	0	0
lincRNA	AC005186.1	ENSG00000275212.2	0	0
lincRNA	LINC02359	ENSG00000284848.1	0	0
lincRNA	AC007368.2	ENSG00000256494.1	0	0
lincRNA	LINC02347	ENSG00000214043.7	0	0
lincRNA	AC006065.4	ENSG00000256732.1	0	0
lincRNA	AC006065.3	ENSG00000255944.1	0	0
lincRNA	AC069235.1	ENSG00000255998.1	0	0
lincRNA	LINC00943	ENSG00000189238.5	0	0
lincRNA	LINC02372	ENSG00000249873.6	0	0
lincRNA	AC078878.2	ENSG00000256286.1	0	0
lincRNA	LINC02405	ENSG00000249345.6	0	0
lincRNA	AC079949.1	ENSG00000256001.1	0	0

lincRNA	AC079949.2	ENSG00000278266.1	0	1
lincRNA	LINC02376	ENSG00000256292.1	0	0
lincRNA	LINC02375	ENSG00000256362.1	0	0
lincRNA	AC025252.1	ENSG00000255945.1	0	0
lincRNA	LINC02411	ENSG00000256022.5	0	0
lincRNA	AC087894.2	ENSG00000256922.1	0	0
lincRNA	LINC02393	ENSG00000256597.2	0	0
lincRNA	LINC02441	ENSG00000256643.1	0	0
lincRNA	LINC02369	ENSG00000256551.1	0	0
lincRNA	LINC02368	ENSG00000256659.1	0	0
lincRNA	AC061709.1	ENSG00000276487.1	0	0
lincRNA	AC108704.1	ENSG00000274695.1	0	0
lincRNA	TMEM132D-	ENSG00000256699.1	0	0
lincRNA	AC130404.1	ENSG00000256085.1	0	0
lincRNA	AC117373.1	ENSG00000256137.1	0	0
lincRNA	AC055717.1	ENSG00000251536.2	0	0
lincRNA	AC055717.2	ENSG00000261650.1	0	0
lincRNA	LINC02418	ENSG00000214039.8	0	0
lincRNA	AC135388.1	ENSG00000256298.1	0	0
lincRNA	LINC02419	ENSG00000256906.1	0	0
lincRNA	AC026336.2	ENSG00000259862.1	0	0
lincRNA	AC063926.1	ENSG00000256064.1	0	0
lincRNA	AC095350.1	ENSG00000256343.7	0	0
lincRNA	AC073912.1	ENSG00000256250.1	0	0
lincRNA	AC073912.2	ENSG00000256299.1	0	0
lincRNA	AC073862.1	ENSG00000256204.1	0	0
lincRNA	ADGRD1-	ENSG00000256151.1	0	0
lincRNA	AC078925.1	ENSG00000256810.1	0	0
lincRNA	LINC01257	ENSG00000204603.6	0	0
lincRNA	LINC02415	ENSG00000248703.2	0	0
lincRNA	LINC02370	ENSG00000230294.4	0	0
lincRNA	AC140118.1	ENSG00000256484.1	0	0
lincRNA	AC073578.2	ENSG00000275232.1	0	0
lincRNA	AC073578.1	ENSG00000256209.1	0	0
lincRNA	AC117500.3	ENSG00000256258.1	0	0
lincRNA	LINC02414	ENSG00000256424.1	0	0
lincRNA	AC117500.1	ENSG00000249926.2	0	0
lincRNA	AC117500.2	ENSG00000255933.1	0	0
lincRNA	AC131009.1	ENSG00000255992.1	0	0
lincRNA	AC137590.2	ENSG00000274670.1	0	0
lincRNA	AC138466.3	ENSG00000276693.1	0	0
lincRNA	AC138466.1	ENSG00000256312.1	0	0
lincRNA	AC148477.4	ENSG00000277011.1	0	0
lincRNA	AC148476.1	ENSG00000274373.1	0	0

lincRNA	AC079031.1	ENSG00000256783.1	0	0
lincRNA	AC079031.2	ENSG00000256875.2	0	0
lincRNA	AC131212.1	ENSG00000277186.1	0	0
lincRNA	AC073911.1	ENSG00000246394.7	0	0
lincRNA	FAM230C	ENSG00000279516.1	0	0
lincRNA	LINC00349	ENSG00000231238.1	0	0
lincRNA	LINC00388	ENSG00000229788.1	0	0
lincRNA	LINC00387	ENSG00000231914.1	0	0
lincRNA	LINC00408	ENSG00000226250.1	0	0
lincRNA	AL355516.1	ENSG00000231358.3	0	0
lincRNA	AL139327.2	ENSG00000232243.1	0	0
lincRNA	LINC00421	ENSG00000236834.1	0	0
lincRNA	AL356259.1	ENSG00000229791.1	0	0
lincRNA	LINC00350	ENSG00000225316.2	0	0
lincRNA	LINC01072	ENSG00000236076.1	0	0
lincRNA	LINC01046	ENSG00000233405.1	0	0
lincRNA	LINC00539	ENSG00000224429.7	0	0
lincRNA	LINC00424	ENSG00000226722.3	0	0
lincRNA	LINC00540	ENSG00000276476.2	0	0
lincRNA	LINC00621	ENSG00000262619.1	0	0
lincRNA	AL157931.1	ENSG00000262198.1	0	0
lincRNA	AL157931.2	ENSG00000283075.1	0	0
lincRNA	LINC00362	ENSG00000229483.2	0	0
lincRNA	SACS-AS1	ENSG00000229558.2	0	0
lincRNA	LINC00352	ENSG00000227893.1	0	0
lincRNA	SPATA13	ENSG00000228741.2	0	0
lincRNA	LINC00566	ENSG00000261498.1	0	0
lincRNA	AL391560.1	ENSG00000285806.1	0	0
lincRNA	LINC00463	ENSG00000234056.1	0	0
lincRNA	LINC01076	ENSG00000225105.2	0	0
lincRNA	AL590787.1	ENSG00000260509.2	0	0
lincRNA	LINC00415	ENSG00000231983.1	0	0
lincRNA	AL353789.1	ENSG00000285929.1	0	0
lincRNA	AL160035.1	ENSG00000283579.1	0	0
lincRNA	LINC02340	ENSG00000275294.4	0	0
lincRNA	LINC01079	ENSG00000229609.1	0	0
lincRNA	AL136439.1	ENSG00000273552.1	0	0
lincRNA	PLUT	ENSG00000247381.3	0	0
lincRNA	LINC00297	ENSG00000224329.2	0	0
lincRNA	LINC00544	ENSG00000122043.10	0	0
lincRNA	AL354674.1	ENSG00000266505.1	0	0
lincRNA	LINC00384	ENSG00000232117.1	0	0
lincRNA	LINC00385	ENSG00000232643.1	0	0
lincRNA	LINC00427	ENSG00000236463.1	0	0

lincRNA	LINC01058	ENSG00000225039.1	0	0
lincRNA	LINC00398	ENSG00000237879.1	0	0
lincRNA	LINC00545	ENSG00000236094.1	0	4
lincRNA	LINC01066	ENSG00000230403.1	0	0
lincRNA	AL161616.2	ENSG00000278305.1	0	0
lincRNA	AL138820.1	ENSG00000277151.1	0	0
lincRNA	LINC00423	ENSG00000226968.1	0	0
lincRNA	LINC02344	ENSG00000227911.3	0	0
lincRNA	AL161719.1	ENSG00000234535.1	0	0
lincRNA	AL139081.1	ENSG00000232986.1	0	0
lincRNA	LINC02343	ENSG00000271850.1	0	0
lincRNA	LINC00457	ENSG00000225179.1	0	0
lincRNA	AL161716.1	ENSG00000271901.1	0	0
lincRNA	AL138690.1	ENSG00000261728.1	0	0
lincRNA	LINC00445	ENSG00000236036.1	0	0
lincRNA	LINC01048	ENSG00000230390.1	0	0
lincRNA	LINC00547	ENSG00000275226.1	0	1
lincRNA	LINC02334	ENSG00000274317.1	0	0
lincRNA	LINC00571	ENSG00000223685.5	0	0
lincRNA	LINC00437	ENSG00000236354.1	0	0
lincRNA	LINC00366	ENSG00000229437.1	0	0
lincRNA	AL354809.1	ENSG00000273507.4	0	0
lincRNA	LINC00332	ENSG00000230710.1	0	0
lincRNA	LINC01050	ENSG00000271216.1	0	2
lincRNA	LINC00428	ENSG00000229546.1	0	0
lincRNA	LINC00400	ENSG00000229928.1	0	0
lincRNA	ENOX1-AS1	ENSG00000233821.1	0	0
lincRNA	AL162713.1	ENSG00000270522.1	0	0
lincRNA	LINC00284	ENSG00000233725.7	0	0
lincRNA	LINC00390	ENSG00000226519.1	0	0
lincRNA	AL589745.2	ENSG00000276854.1	0	0
lincRNA	AL589745.1	ENSG00000230731.2	0	0
lincRNA	AL138960.1	ENSG00000277831.1	0	1
lincRNA	TUSC8	ENSG00000237361.2	0	0
lincRNA	LINC00407	ENSG00000237585.1	0	0
lincRNA	LINC00330	ENSG00000235097.1	0	0
lincRNA	AL359706.1	ENSG00000223732.2	0	0
lincRNA	AL138963.1	ENSG00000228886.1	0	0
lincRNA	SLC25A30-	ENSG00000251015.1	0	0
lincRNA	LINC01055	ENSG00000235366.2	0	0
lincRNA	LINC00563	ENSG00000261097.1	0	0
lincRNA	LINC01198	ENSG00000231817.8	0	1
lincRNA	AL138686.1	ENSG00000277228.1	0	0
lincRNA	AL138962.1	ENSG00000228573.1	0	0

lincRNA	RB1-DT	ENSG00000231473.2	0	0
lincRNA	LINC01077	ENSG00000233456.1	0	0
lincRNA	LINC00462	ENSG00000233610.1	0	0
lincRNA	DLEU1-AS1	ENSG00000229323.1	0	0
lincRNA	AL157817.1	ENSG00000274652.1	0	0
lincRNA	LINC02333	ENSG00000271564.1	0	0
lincRNA	LINC00345	ENSG00000235660.2	0	0
lincRNA	LINC01065	ENSG00000237092.1	0	0
lincRNA	AL136359.1	ENSG00000279325.1	0	0
lincRNA	AL450423.1	ENSG00000273919.1	0	0
lincRNA	LINC00558	ENSG00000261517.1	0	0
lincRNA	LINC00458	ENSG00000234787.7	0	0
lincRNA	AL390964.1	ENSG00000277047.4	0	0
lincRNA	LINC02335	ENSG00000278630.1	0	0
lincRNA	AL354821.1	ENSG00000274090.1	0	0
lincRNA	LINC00374	ENSG00000232954.2	0	0
lincRNA	LINC00434	ENSG00000227336.1	0	0
lincRNA	LINC00378	ENSG00000225249.5	0	0
lincRNA	LINC01442	ENSG00000227510.3	0	0
lincRNA	LINC02339	ENSG00000279797.1	0	0
lincRNA	LINC00358	ENSG00000229578.2	0	0
lincRNA	LINC01075	ENSG00000230142.2	0	0
lincRNA	LINC01074	ENSG00000227611.1	0	0
lincRNA	LINC00459	ENSG00000229307.1	0	1
lincRNA	LINC00448	ENSG00000228669.1	0	0
lincRNA	AL354810.1	ENSG00000273550.1	0	0
lincRNA	LINC00376	ENSG00000227564.2	0	0
lincRNA	AL445989.2	ENSG00000285764.1	0	0
lincRNA	LINC00355	ENSG00000227674.2	0	0
lincRNA	LINC01052	ENSG00000234767.1	0	0
lincRNA	LINC00364	ENSG00000230040.1	0	0
lincRNA	LINC00550	ENSG00000281778.1	0	0
lincRNA	LINC02342	ENSG00000280580.1	0	0
lincRNA	LINC00383	ENSG00000235221.2	0	0
lincRNA	LINC00348	ENSG00000226846.1	0	0
lincRNA	LINC00393	ENSG00000224853.2	0	0
lincRNA	LINC00392	ENSG00000228295.1	0	0
lincRNA	LINC00402	ENSG00000235532.1	0	0
lincRNA	AL355390.1	ENSG00000177596.1	0	0
lincRNA	AL355390.2	ENSG00000281167.1	0	0
lincRNA	LINC00347	ENSG00000236678.7	0	0
lincRNA	LINC01078	ENSG00000223880.1	0	0
lincRNA	LMO7DN-	ENSG00000223458.2	0	0
lincRNA	LINC00561	ENSG00000261206.1	0	0

lincRNA	LINC01034	ENSG00000224933.2	0	0
lincRNA	AL365394.1	ENSG00000284809.1	0	0
lincRNA	AC001226.1	ENSG00000274898.1	0	0
lincRNA	AL159158.1	ENSG00000285714.1	0	0
lincRNA	EDNRB-AS1	ENSG00000225579.1	0	0
lincRNA	AL139002.1	ENSG00000233379.1	0	0
lincRNA	LINC01069	ENSG00000236133.1	0	0
lincRNA	LINC00446	ENSG00000229249.6	0	0
lincRNA	AL138957.1	ENSG00000235625.1	0	0
lincRNA	LINC00331	ENSG00000225427.1	0	0
lincRNA	LINC01068	ENSG00000227676.3	0	0
lincRNA	AL136442.1	ENSG00000285902.1	0	0
lincRNA	LINC01038	ENSG00000229011.1	0	0
lincRNA	LINC00382	ENSG00000229175.1	0	0
lincRNA	LINC01080	ENSG00000281721.1	0	0
lincRNA	AL137781.1	ENSG00000285684.1	0	0
lincRNA	LINC00377	ENSG00000229246.1	0	0
lincRNA	LINC00564	ENSG00000260094.1	0	0
lincRNA	AL353633.1	ENSG00000229309.2	0	0
lincRNA	AL445604.1	ENSG00000285742.1	0	0
lincRNA	LINC00333	ENSG00000233349.1	0	0
lincRNA	LINC00375	ENSG00000226370.1	0	0
lincRNA	LINC00351	ENSG00000226317.2	0	0
lincRNA	AL356413.1	ENSG00000285834.1	0	0
lincRNA	AL354994.1	ENSG00000232252.1	0	0
lincRNA	LINC00430	ENSG00000233528.4	0	0
lincRNA	AL360267.2	ENSG00000285699.1	0	0
lincRNA	AL360267.1	ENSG00000232636.2	0	0
lincRNA	MIR4500HG	ENSG00000228824.6	0	0
lincRNA	AL355578.1	ENSG00000272515.1	0	0
lincRNA	LINC00397	ENSG00000223404.3	0	0
lincRNA	AL445647.1	ENSG00000272046.1	0	0
lincRNA	AL354896.1	ENSG00000231019.1	0	0
lincRNA	AL355677.1	ENSG00000285811.1	0	0
lincRNA	LINC00433	ENSG00000229443.1	0	0
lincRNA	LINC00560	ENSG00000261666.1	0	0
lincRNA	LINC01047	ENSG00000232225.6	0	0
lincRNA	LINC00440	ENSG00000234660.1	0	0
lincRNA	LINC01040	ENSG00000226037.1	0	0
lincRNA	LINC00353	ENSG00000236176.2	0	0
lincRNA	LINC02336	ENSG00000271395.1	0	0
lincRNA	LINC00559	ENSG00000261446.3	0	0
lincRNA	LINC01049	ENSG00000234384.1	0	0
lincRNA	LINC00410	ENSG00000231674.1	0	0

lincRNA	LINC00380	ENSG00000234625.1	0	0
lincRNA	LINC00379	ENSG00000229557.1	0	0
lincRNA	LINC00363	ENSG00000232849.1	0	0
lincRNA	LINC00391	ENSG00000238230.1	0	0
lincRNA	LINC00557	ENSG00000260962.1	0	0
lincRNA	AL353581.1	ENSG00000272329.1	0	0
lincRNA	LINC00456	ENSG00000233124.1	0	0
lincRNA	AL359502.1	ENSG00000226134.1	0	0
lincRNA	AL356580.1	ENSG00000270725.1	0	0
lincRNA	AL136961.1	ENSG00000285448.1	0	2
lincRNA	AL139035.1	ENSG00000280710.3	0	0
lincRNA	LINC01039	ENSG00000234168.2	0	0
lincRNA	LINC00411	ENSG00000229599.1	0	0
lincRNA	AL158063.1	ENSG00000276957.1	0	0
lincRNA	AL137246.1	ENSG00000272542.1	0	0
lincRNA	AL137246.2	ENSG00000278445.1	0	0
lincRNA	AL162717.1	ENSG00000285789.1	0	0
lincRNA	LINC01309	ENSG00000234551.1	0	0
lincRNA	AL359745.1	ENSG00000280455.1	0	0
lincRNA	LINC00443	ENSG00000230156.2	0	0
lincRNA	AL162574.2	ENSG00000278698.1	0	0
lincRNA	AL162574.1	ENSG00000277863.1	0	0
lincRNA	AL354741.1	ENSG00000285643.1	0	0
lincRNA	AL136964.1	ENSG00000274718.1	0	0
lincRNA	AL138694.1	ENSG00000283384.1	0	0
lincRNA	LINC01067	ENSG00000236053.1	0	0
lincRNA	LINC00399	ENSG00000229792.1	0	0
lincRNA	AL163541.1	ENSG00000285534.1	0	0
lincRNA	AL355810.1	ENSG00000275248.1	0	0
lincRNA	AL355974.1	ENSG00000274331.1	0	0
lincRNA	AL355974.2	ENSG00000275830.1	0	0
lincRNA	AL355974.3	ENSG00000276269.1	0	0
lincRNA	LINC00396	ENSG00000231428.2	0	0
lincRNA	LINC00567	ENSG00000259831.1	0	0
lincRNA	LINC00368	ENSG00000225870.1	0	0
lincRNA	AL359649.1	ENSG00000204398.5	0	0
lincRNA	LINC02337	ENSG00000215881.3	0	0
lincRNA	LINC00354	ENSG00000226903.1	0	0
lincRNA	AL356961.1	ENSG00000260385.1	0	0
lincRNA	LINC00404	ENSG00000229520.1	0	0
lincRNA	SOX1-OT	ENSG00000224243.1	0	0
lincRNA	LINC01070	ENSG00000260102.1	0	0
lincRNA	LINC01043	ENSG00000260343.1	0	0
lincRNA	LINC01044	ENSG00000223626.1	0	0

lincRNA	AL139384.2	ENSG00000277159.1	0	0
lincRNA	ATP11AUN	ENSG00000197595.4	0	0
lincRNA	AL139384.1	ENSG00000274922.1	0	2
lincRNA	AL356740.1	ENSG00000267868.1	0	0
lincRNA	BX072579.1	ENSG00000237699.2	0	0
lincRNA	LINC00454	ENSG00000226921.6	0	0
lincRNA	BX072579.2	ENSG00000274766.1	0	0
lincRNA	LINC00452	ENSG00000229373.8	0	0
lincRNA	LINC00565	ENSG00000260910.1	0	4
lincRNA	AL160396.2	ENSG00000285672.1	0	0
lincRNA	LINC02297	ENSG00000257504.1	0	0
lincRNA	CR383656.12	ENSG00000258364.1	0	0
lincRNA	AL929601.1	ENSG00000257891.1	0	0
lincRNA	AL929601.2	ENSG00000258265.1	0	0
lincRNA	AL589182.1	ENSG00000258314.3	0	0
lincRNA	AL589743.4	ENSG00000277128.2	0	0
lincRNA	AL589743.1	ENSG00000257310.1	0	0
lincRNA	LINC01297	ENSG00000274827.4	0	0
lincRNA	AL512310.10	ENSG00000273558.1	0	0
lincRNA	AL512310.9	ENSG00000259069.1	0	0
lincRNA	AL512310.2	ENSG00000257395.1	0	0
lincRNA	AL355075.3	ENSG00000258908.1	0	0
lincRNA	AL133371.3	ENSG00000259130.1	0	0
lincRNA	AL355922.4	ENSG00000258918.1	0	0
lincRNA	AL355922.3	ENSG00000258772.1	0	0
lincRNA	AL355922.1	ENSG00000136315.4	0	0
lincRNA	AL161668.4	ENSG00000258604.1	0	0
lincRNA	AL161668.1	ENSG00000178107.2	0	0
lincRNA	AC243972.1	ENSG00000248358.2	0	0
lincRNA	AC244502.3	ENSG00000259042.2	0	0
lincRNA	LINC02332	ENSG00000259054.1	0	0
lincRNA	AC243965.1	ENSG00000259003.1	0	0
lincRNA	AL135999.3	ENSG00000274002.1	0	0
lincRNA	AL136419.2	ENSG00000285727.1	0	0
lincRNA	LINC00596	ENSG00000259334.1	0	0
lincRNA	AL136295.2	ENSG00000259321.1	0	0
lincRNA	LINC02286	ENSG00000258098.1	0	0
lincRNA	LINC02306	ENSG00000257986.2	0	0
lincRNA	AL132633.1	ENSG00000285633.1	0	0
lincRNA	LINC02294	ENSG00000257845.1	0	0
lincRNA	LINC02293	ENSG00000257185.1	0	0
lincRNA	AL110292.1	ENSG00000258081.3	0	0
lincRNA	MIR4307HG	ENSG00000257612.1	0	0
lincRNA	LINC00645	ENSG00000258548.5	0	0

lincRNA	AL139023.1	ENSG00000257869.1	0	0
lincRNA	LINC02300	ENSG00000258175.1	0	0
lincRNA	LINC02282	ENSG00000257056.2	0	0
lincRNA	LINC02281	ENSG00000257748.1	0	0
lincRNA	LINC02327	ENSG00000258038.5	0	0
lincRNA	LINC02326	ENSG00000257523.2	0	0
lincRNA	AL138974.1	ENSG00000258028.1	0	0
lincRNA	AL158058.1	ENSG00000258107.2	0	0
lincRNA	AL133372.2	ENSG00000248975.2	0	0
lincRNA	AL355112.1	ENSG00000257472.1	0	0
lincRNA	LINC02313	ENSG00000258474.1	0	0
lincRNA	AL352984.1	ENSG00000258386.1	0	0
lincRNA	AL445363.1	ENSG00000259002.2	0	0
lincRNA	AL133163.2	ENSG00000258860.1	0	0
lincRNA	AL133163.3	ENSG00000259017.1	0	0
lincRNA	AL162311.1	ENSG00000257272.1	0	0
lincRNA	AL133304.1	ENSG00000257614.1	0	0
lincRNA	AL133304.2	ENSG00000257826.1	0	0
lincRNA	LINC00609	ENSG00000257585.1	0	0
lincRNA	PTCSC3	ENSG00000259104.2	0	0
lincRNA	AL162511.1	ENSG00000258844.1	0	0
lincRNA	AL121790.2	ENSG00000259087.5	0	0
lincRNA	AL121790.1	ENSG00000258414.1	0	0
lincRNA	AL392023.1	ENSG00000258649.1	0	0
lincRNA	AL161751.1	ENSG00000259072.1	0	0
lincRNA	SEC23A-AS1	ENSG00000258651.1	0	0
lincRNA	AL049875.1	ENSG00000258526.5	0	0
lincRNA	AL352977.1	ENSG00000258418.1	0	0
lincRNA	AL352977.2	ENSG00000258480.1	0	0
lincRNA	AL121821.1	ENSG00000258636.1	0	0
lincRNA	AL445074.1	ENSG00000259149.1	0	0
lincRNA	AL450442.1	ENSG00000258850.1	0	0
lincRNA	LINC02307	ENSG00000258969.1	0	0
lincRNA	AL161752.1	ENSG00000259126.1	0	0
lincRNA	LINC02277	ENSG00000258487.1	0	0
lincRNA	LINC02302	ENSG00000258998.1	0	0
lincRNA	AL121809.2	ENSG00000278147.1	0	0
lincRNA	AL162632.1	ENSG00000257900.2	0	0
lincRNA	AL139354.1	ENSG00000258845.1	0	0
lincRNA	LINC02303	ENSG00000258616.5	0	0
lincRNA	AL358335.2	ENSG00000258751.1	0	0
lincRNA	AL110505.1	ENSG00000258868.1	0	0
lincRNA	AL627171.2	ENSG00000282885.1	0	0
lincRNA	AL359397.1	ENSG00000258857.1	0	0

lincRNA	AL118556.1	ENSG00000259113.1	0	0
lincRNA	AL133485.2	ENSG00000285747.1	0	0
lincRNA	LINC00519	ENSG00000258955.1	0	0
lincRNA	LINC02310	ENSG00000258808.1	0	0
lincRNA	FRMD6-AS2	ENSG00000258537.5	0	0
lincRNA	AL079307.2	ENSG00000259111.1	0	0
lincRNA	LINC02319	ENSG00000258694.1	0	0
lincRNA	AL139317.1	ENSG00000258698.1	0	0
lincRNA	AL163953.1	ENSG00000225680.1	0	0
lincRNA	LINC02331	ENSG00000235269.1	0	0
lincRNA	AL359792.1	ENSG00000258753.1	0	0
lincRNA	AL133444.1	ENSG00000285774.1	0	0
lincRNA	AL139316.1	ENSG00000258050.2	0	0
lincRNA	AL158801.2	ENSG00000258413.1	0	0
lincRNA	AL355773.1	ENSG00000258784.1	0	0
lincRNA	AL355073.1	ENSG00000259483.1	0	0
lincRNA	LINC02284	ENSG00000259719.5	0	0
lincRNA	AL355103.1	ENSG00000258803.1	0	0
lincRNA	AL161757.3	ENSG00000258703.1	0	0
lincRNA	OTX2-AS1	ENSG00000248550.3	0	0
lincRNA	AL161757.4	ENSG00000258776.1	0	0
lincRNA	AL355834.1	ENSG00000259008.1	0	0
lincRNA	LINC00216	ENSG00000279636.2	0	0
lincRNA	LINC01500	ENSG00000258583.6	0	0
lincRNA	LINC02322	ENSG00000258556.1	0	0
lincRNA	SALRNA1	ENSG00000258952.2	0	0
lincRNA	LINC01303	ENSG00000250548.6	0	2
lincRNA	HIF1A-AS1	ENSG00000258777.1	0	0
lincRNA	HIF1A-AS2	ENSG00000258667.1	0	0
lincRNA	AL390816.1	ENSG00000258842.1	0	0
lincRNA	AL390816.2	ENSG00000258903.1	0	0
lincRNA	LINC00644	ENSG00000259142.1	0	0
lincRNA	AL137191.1	ENSG00000259093.1	0	0
lincRNA	AL049871.1	ENSG00000258943.1	0	0
lincRNA	AL136038.2	ENSG00000258800.1	0	0
lincRNA	LINC02324	ENSG00000255002.1	0	0
lincRNA	AL355076.2	ENSG00000258760.1	0	0
lincRNA	AL391261.2	ENSG00000258847.1	0	0
lincRNA	LINC02290	ENSG00000258502.5	0	0
lincRNA	AL359317.2	ENSG00000258957.1	0	0
lincRNA	AL359317.1	ENSG00000258520.1	0	0
lincRNA	AL356804.1	ENSG00000259033.1	0	0
lincRNA	AL357153.2	ENSG00000257759.2	0	0
lincRNA	AL357153.3	ENSG00000259115.1	0	0

lincRNA	AL357153.1	ENSG00000245466.1	0	0
lincRNA	LINC01269	ENSG00000258689.1	0	0
lincRNA	AC004816.2	ENSG00000275630.1	0	1
lincRNA	AC004817.2	ENSG00000259907.1	0	0
lincRNA	AC004817.1	ENSG00000258517.1	0	0
lincRNA	AC004817.4	ENSG00000269945.1	0	0
lincRNA	AC005993.1	ENSG00000266869.1	0	0
lincRNA	AC004828.1	ENSG00000258871.1	0	0
lincRNA	AC005280.2	ENSG00000284930.1	0	0
lincRNA	LINC02274	ENSG00000258586.1	0	0
lincRNA	AC005479.1	ENSG00000259005.1	0	0
lincRNA	AC013451.1	ENSG00000258425.1	0	0
lincRNA	AC013451.2	ENSG00000258976.1	0	0
lincRNA	AL691403.2	ENSG00000273565.1	0	0
lincRNA	AL691403.1	ENSG00000258740.1	0	0
lincRNA	LINC01220	ENSG00000259687.1	0	0
lincRNA	TGFB3-AS1	ENSG00000258876.1	0	0
lincRNA	AC016526.1	ENSG00000258402.1	0	0
lincRNA	AC008050.1	ENSG00000259124.1	0	0
lincRNA	AC007376.2	ENSG00000258569.1	0	0
lincRNA	AF111169.1	ENSG00000258610.1	0	0
lincRNA	AF111169.2	ENSG00000259058.1	0	0
lincRNA	AC007686.4	ENSG00000285966.1	0	0
lincRNA	LINC01629	ENSG00000258602.1	0	0
lincRNA	LINC02288	ENSG00000246548.3	0	0
lincRNA	AC007686.2	ENSG00000258473.1	0	0
lincRNA	LINC02289	ENSG00000258819.1	0	0
lincRNA	AF123462.1	ENSG00000258416.1	0	0
lincRNA	LINC02308	ENSG00000258675.1	0	0
lincRNA	LINC01467	ENSG00000258977.1	0	0
lincRNA	LINC02311	ENSG00000258718.2	0	0
lincRNA	AL355838.1	ENSG00000259035.1	0	0
lincRNA	LINC02301	ENSG00000258743.5	0	0
lincRNA	AL355095.1	ENSG00000258683.1	0	0
lincRNA	AL359238.1	ENSG00000258600.1	0	0
lincRNA	AL162872.1	ENSG00000258426.1	0	0
lincRNA	LINC02305	ENSG00000258532.1	0	0
lincRNA	AL356807.1	ENSG00000285826.1	0	0
lincRNA	AL357172.1	ENSG00000259044.1	0	0
lincRNA	LINC02329	ENSG00000258814.1	0	0
lincRNA	LINC02316	ENSG00000258912.1	0	0
lincRNA	LINC02309	ENSG00000258994.1	0	0
lincRNA	LINC01148	ENSG00000258804.1	0	0
lincRNA	AL358292.1	ENSG00000259061.1	0	0

lincRNA	AL352955.1	ENSG00000258496.1	0	0
lincRNA	AL157688.1	ENSG00000258958.1	0	0
lincRNA	LINC02296	ENSG00000258859.1	0	0
lincRNA	AL135746.1	ENSG00000259028.1	0	0
lincRNA	LINC02330	ENSG00000258770.1	0	0
lincRNA	AL359237.1	ENSG00000258807.5	0	0
lincRNA	AL136501.1	ENSG00000259077.1	0	0
lincRNA	AL157955.2	ENSG00000258826.5	0	0
lincRNA	LINC01146	ENSG00000258867.5	0	0
lincRNA	AL133279.2	ENSG00000258975.1	0	0
lincRNA	AL133279.3	ENSG00000259998.1	0	0
lincRNA	AL133279.1	ENSG00000258538.5	0	0
lincRNA	AL138478.1	ENSG00000277801.1	0	0
lincRNA	AL137230.1	ENSG00000258792.4	0	0
lincRNA	AL512791.2	ENSG00000275198.1	0	1
lincRNA	LINC02317	ENSG00000258678.1	0	0
lincRNA	LINC00642	ENSG00000233208.5	0	0
lincRNA	AL096869.1	ENSG00000258935.1	0	0
lincRNA	LINC02321	ENSG00000258884.1	0	0
lincRNA	AL133153.2	ENSG00000258798.1	0	0
lincRNA	AL133240.1	ENSG00000258481.1	0	0
lincRNA	AL117192.1	ENSG00000258742.5	0	0
lincRNA	LINC02287	ENSG00000258499.1	0	0
lincRNA	AL121612.1	ENSG00000274379.1	0	0
lincRNA	LINC02279	ENSG00000258933.1	0	0
lincRNA	AL390254.1	ENSG00000258866.1	0	0
lincRNA	LINC02292	ENSG00000258630.1	0	0
lincRNA	AL133467.1	ENSG00000258572.1	0	0
lincRNA	LINC02318	ENSG00000258390.1	0	0
lincRNA	AL133467.2	ENSG00000258927.1	0	0
lincRNA	AL133467.4	ENSG00000270038.1	0	0
lincRNA	AL133167.1	ENSG00000258516.1	0	0
lincRNA	AL355102.3	ENSG00000258729.2	0	0
lincRNA	LINC02299	ENSG00000258979.2	0	0
lincRNA	AL049833.2	ENSG00000259026.1	0	0
lincRNA	AL049833.1	ENSG00000258393.1	0	0
lincRNA	LINC02304	ENSG00000259110.1	0	0
lincRNA	AL158800.1	ENSG00000285584.1	0	0
lincRNA	LINC02325	ENSG00000246084.2	0	0
lincRNA	LINC02291	ENSG00000197176.7	0	0
lincRNA	LINC02312	ENSG00000258869.1	0	0
lincRNA	AL355097.1	ENSG00000258379.1	0	0
lincRNA	AL163932.1	ENSG00000259097.1	0	0
lincRNA	LINC02295	ENSG00000258511.1	0	0

lincRNA	AL132719.1	ENSG00000258795.1	0	0
lincRNA	AL132796.1	ENSG00000237342.1	0	0
lincRNA	AL132796.2	ENSG00000259119.1	0	0
lincRNA	AL132819.1	ENSG00000258383.1	0	0
lincRNA	AL160313.1	ENSG00000247970.2	0	0
lincRNA	AL160313.2	ENSG00000258672.1	0	0
lincRNA	AL133368.2	ENSG00000258693.1	0	0
lincRNA	AL157871.1	ENSG00000258504.2	0	0
lincRNA	AL845552.2	ENSG00000259031.1	0	0
lincRNA	LINC00523	ENSG00000196273.7	0	0
lincRNA	AL132711.1	ENSG00000258717.1	0	0
lincRNA	MIR381HG	ENSG00000258861.1	0	0
lincRNA	AL132709.1	ENSG00000230805.6	0	0
lincRNA	LINC02285	ENSG00000259004.1	0	0
lincRNA	AL355096.1	ENSG00000258460.1	0	0
lincRNA	LINC00524	ENSG00000259023.2	0	0
lincRNA	LINC02314	ENSG00000259082.1	0	0
lincRNA	AL049836.2	ENSG00000259166.1	0	0
lincRNA	DIO3OS	ENSG00000258498.8	0	0
lincRNA	AL049836.1	ENSG00000258919.1	0	0
lincRNA	LINC02320	ENSG00000258404.1	0	0
lincRNA	AL118558.3	ENSG00000271780.1	0	1
lincRNA	AL132801.1	ENSG00000259508.1	0	0
lincRNA	LINC00677	ENSG00000259717.1	0	0
lincRNA	LINC00605	ENSG00000251533.2	0	0
lincRNA	LINC00637	ENSG00000258735.1	0	0
lincRNA	AL132712.1	ENSG00000258534.1	0	0
lincRNA	AL132712.2	ENSG00000258914.1	0	0
lincRNA	AL590079.1	ENSG00000258913.1	0	0
lincRNA	AL512357.1	ENSG00000258598.1	0	0
lincRNA	AL583722.3	ENSG00000258736.1	0	0
lincRNA	AL583810.2	ENSG00000258811.1	0	0
lincRNA	AL583810.3	ENSG00000259067.1	0	0
lincRNA	AL928742.1	ENSG00000253364.2	0	0
lincRNA	AC246787.2	ENSG00000244620.1	0	0
lincRNA	FAM30A	ENSG00000226777.7	0	0
lincRNA	AC247036.1	ENSG00000271201.1	0	0
lincRNA	LINC00226	ENSG00000276210.2	0	0
lincRNA	LINC00221	ENSG00000270816.5	0	0
lincRNA	AC087386.1	ENSG00000258410.1	0	0
lincRNA	AC026495.1	ENSG00000258654.1	0	0
lincRNA	AC012414.5	ENSG00000260409.1	0	0
lincRNA	AC012414.3	ENSG00000259383.1	0	0
lincRNA	AC012414.4	ENSG00000259443.1	0	0

lincRNA	LINC01193	ENSG00000258710.7	0	0
lincRNA	AC126335.1	ENSG00000260986.1	0	0
lincRNA	AC068446.2	ENSG00000247765.2	0	0
lincRNA	AC060814.5	ENSG00000280881.1	0	0
lincRNA	AC060814.4	ENSG00000280703.1	0	0
lincRNA	AC060814.3	ENSG00000268531.3	0	0
lincRNA	LINC02203	ENSG00000284988.1	0	0
lincRNA	AC135068.7	ENSG00000281113.1	0	0
lincRNA	AC134980.2	ENSG00000284500.2	0	0
lincRNA	AC010760.1	ENSG00000260739.1	0	0
lincRNA	AC021439.1	ENSG00000261622.1	0	0
lincRNA	PWRN4	ENSG00000260232.1	0	0
lincRNA	PWRN2	ENSG00000260551.1	0	0
lincRNA	AC087463.1	ENSG00000260780.1	0	0
lincRNA	AC087463.4	ENSG00000261621.1	0	0
lincRNA	AC087463.2	ENSG00000260959.1	0	0
lincRNA	AC087463.3	ENSG00000261598.1	0	0
lincRNA	PWRN3	ENSG00000260760.1	0	0
lincRNA	PWRN1	ENSG00000259905.5	0	0
lincRNA	AC124312.2	ENSG00000257647.1	0	0
lincRNA	AC124312.3	ENSG00000261069.3	0	0
lincRNA	LINC02250	ENSG00000235731.1	0	0
lincRNA	AC016266.1	ENSG00000259011.1	0	0
lincRNA	LINC02346	ENSG00000206187.3	0	0
lincRNA	AC100836.1	ENSG00000258931.1	0	0
lincRNA	LINC00929	ENSG00000259150.5	0	0
lincRNA	LINC02248	ENSG00000235160.1	0	0
lincRNA	AC011196.1	ENSG00000235518.2	0	0
lincRNA	AC021979.1	ENSG00000258594.1	0	0
lincRNA	AC021979.2	ENSG00000258853.1	0	0
lincRNA	GOLGA8M	ENSG00000261480.1	0	0
lincRNA	AC102941.1	ENSG00000259814.1	0	0
lincRNA	AC111152.2	ENSG00000259647.1	0	0
lincRNA	AC120045.1	ENSG00000259906.1	0	0
lincRNA	LINC02249	ENSG00000225930.4	0	0
lincRNA	AC026150.1	ENSG00000260693.1	0	0
lincRNA	AC091057.3	ENSG00000269930.1	0	0
lincRNA	LINC02352	ENSG00000259448.2	0	2
lincRNA	AC012236.1	ENSG00000259772.6	0	0
lincRNA	AC139426.2	ENSG00000260642.1	0	0
lincRNA	AC123768.2	ENSG00000244952.2	0	1
lincRNA	LINC02252	ENSG00000261304.1	0	0
lincRNA	AC114546.1	ENSG00000259181.1	0	0
lincRNA	AC114546.2	ENSG00000259587.2	0	0

lincRNA	AC021231.1	ENSG00000259336.1	0	0
lincRNA	AC021351.1	ENSG00000259639.6	0	0
lincRNA	AC040918.1	ENSG00000259245.1	0	0
lincRNA	AC087516.1	ENSG00000259395.1	0	0
lincRNA	AC087516.2	ENSG00000259737.6	0	0
lincRNA	AC068875.1	ENSG00000259434.1	0	0
lincRNA	LINC02345	ENSG00000259225.6	0	0
lincRNA	LINC01852	ENSG00000236914.3	0	0
lincRNA	AC087473.1	ENSG00000259380.5	0	0
lincRNA	AC022929.1	ENSG00000259731.2	0	0
lincRNA	AC087878.1	ENSG00000259278.1	0	0
lincRNA	AC113146.1	ENSG00000259423.2	0	0
lincRNA	C15orf54	ENSG00000175746.6	0	0
lincRNA	AC109630.1	ENSG00000259269.1	0	0
lincRNA	AC022196.1	ENSG00000259390.3	0	0
lincRNA	AC021755.2	ENSG00000259584.1	0	0
lincRNA	AC020658.2	ENSG00000259198.1	0	0
lincRNA	AC025166.1	ENSG00000274798.1	0	0
lincRNA	AC020661.1	ENSG00000251161.3	0	0
lincRNA	AC016134.1	ENSG00000260926.2	0	0
lincRNA	AC073657.1	ENSG00000260814.2	0	0
lincRNA	AC090519.2	ENSG00000259499.1	0	0
lincRNA	AC122108.2	ENSG00000276121.1	0	0
lincRNA	AC122108.1	ENSG00000259187.1	0	0
lincRNA	AC051619.10	ENSG00000285597.1	0	0
lincRNA	AC090527.1	ENSG00000259681.1	0	0
lincRNA	AC090527.3	ENSG00000275709.1	0	0
lincRNA	AC068722.2	ENSG00000273972.1	0	0
lincRNA	AC068722.1	ENSG00000259200.1	0	0
lincRNA	AC091074.1	ENSG00000285934.1	0	0
lincRNA	AC073941.1	ENSG00000259255.1	0	0
lincRNA	LINC01491	ENSG00000259572.5	0	0
lincRNA	AC092078.2	ENSG00000259754.1	0	0
lincRNA	AC092078.1	ENSG00000259730.1	0	0
lincRNA	AC066612.1	ENSG00000259235.1	0	0
lincRNA	AC084757.3	ENSG00000259705.1	0	1
lincRNA	AC084757.4	ENSG00000273925.1	0	0
lincRNA	AC084757.2	ENSG00000259469.1	0	0
lincRNA	AC013452.2	ENSG00000259656.1	0	0
lincRNA	AC022087.1	ENSG00000259715.1	0	0
lincRNA	AC012170.3	ENSG00000259618.1	0	0
lincRNA	AC021752.1	ENSG00000273674.4	0	1
lincRNA	MIR4713HG	ENSG00000259240.1	0	0
lincRNA	AC020892.1	ENSG00000259241.1	0	0

lincRNA	MAPK6-DT	ENSG00000259438.1	0	0
lincRNA	AC023906.2	ENSG00000259178.1	0	0
lincRNA	AC023906.3	ENSG00000259327.1	0	0
lincRNA	AC016044.1	ENSG00000259203.1	0	0
lincRNA	LINC02490	ENSG00000259237.1	0	0
lincRNA	AC084759.2	ENSG00000259669.5	0	0
lincRNA	AC025272.1	ENSG00000260937.5	0	0
lincRNA	AC084782.1	ENSG00000259941.1	0	0
lincRNA	AC084782.2	ENSG00000261823.1	0	0
lincRNA	AC090518.1	ENSG00000260392.5	0	1
lincRNA	AC010999.2	ENSG00000276524.1	0	0
lincRNA	LINC01413	ENSG00000260172.1	0	0
lincRNA	AC025271.3	ENSG00000261489.1	0	0
lincRNA	AC025271.2	ENSG00000261265.1	0	0
lincRNA	AC025271.4	ENSG00000276772.1	0	0
lincRNA	AC090651.1	ENSG00000261219.1	0	0
lincRNA	AC092757.3	ENSG00000277144.1	0	0
lincRNA	AC009654.1	ENSG00000259223.2	0	0
lincRNA	AC009560.1	ENSG00000273958.1	0	0
lincRNA	AC107905.1	ENSG00000276473.1	0	0
lincRNA	AC104574.2	ENSG00000259616.2	0	0
lincRNA	AC104574.1	ENSG00000259530.1	0	0
lincRNA	AC018618.1	ENSG00000259675.1	0	0
lincRNA	LINC02349	ENSG00000259284.1	0	0
lincRNA	AC126323.2	ENSG00000259697.1	0	0
lincRNA	AC126323.3	ENSG00000259878.1	0	0
lincRNA	AC126323.6	ENSG00000261296.1	0	0
lincRNA	AC126323.5	ENSG00000260957.1	0	0
lincRNA	AC103740.2	ENSG00000259727.1	0	0
lincRNA	AC087612.1	ENSG00000259672.2	0	0
lincRNA	LINC02568	ENSG00000259459.5	0	0
lincRNA	AC007950.1	ENSG00000259268.1	0	0
lincRNA	AC013553.4	ENSG00000278737.1	0	0
lincRNA	LINC01169	ENSG00000259471.1	0	0
lincRNA	AC093334.1	ENSG00000259437.1	0	0
lincRNA	LINC02206	ENSG00000259289.2	0	0
lincRNA	AC009292.1	ENSG00000245719.1	0	0
lincRNA	AC107871.2	ENSG00000260657.2	0	0
lincRNA	AC027088.2	ENSG00000259222.2	0	0
lincRNA	AC026992.1	ENSG00000259504.2	0	0
lincRNA	AC100826.1	ENSG00000259457.1	0	0
lincRNA	AC021818.1	ENSG00000259309.2	0	0
lincRNA	AC026583.1	ENSG00000259252.1	0	0
lincRNA	AC048383.1	ENSG00000259503.1	0	0

lincRNA	LINC02205	ENSG00000259473.1	0	0
lincRNA	LINC02204	ENSG00000280639.1	0	0
lincRNA	SALRNA3	ENSG00000281264.1	0	0
lincRNA	AC009269.2	ENSG00000259532.1	0	0
lincRNA	LINC02259	ENSG00000259783.5	0	0
lincRNA	AC100827.4	ENSG00000260672.1	0	0
lincRNA	AC103874.1	ENSG00000285729.1	0	0
lincRNA	AC022188.1	ENSG00000276807.1	0	0
lincRNA	INSYN1-AS1	ENSG00000260469.2	0	0
lincRNA	AC010931.2	ENSG00000248540.2	0	0
lincRNA	AC090826.2	ENSG00000274937.1	0	0
lincRNA	LINC02255	ENSG00000277718.1	0	0
lincRNA	AC012435.2	ENSG00000261775.1	0	0
lincRNA	AC019294.3	ENSG00000261043.5	0	0
lincRNA	AC027243.2	ENSG00000270036.1	0	0
lincRNA	AC090181.1	ENSG00000259652.1	0	0
lincRNA	AC046168.1	ENSG00000259362.2	0	0
lincRNA	AC046168.2	ENSG00000259420.5	0	0
lincRNA	AC104758.4	ENSG00000261244.1	0	0
lincRNA	AC104758.5	ENSG00000261403.1	0	0
lincRNA	AC090260.1	ENSG00000260988.1	0	1
lincRNA	ANKRD34C-AS1	ENSG00000259234.5	0	0
lincRNA	AC021483.1	ENSG00000259548.1	0	0
lincRNA	AC021483.2	ENSG00000261229.6	0	0
lincRNA	AC087761.1	ENSG00000259967.1	0	0
lincRNA	LINC00927	ENSG00000259361.6	0	0
lincRNA	AC023302.1	ENSG00000271983.1	0	0
lincRNA	AC068870.1	ENSG00000260619.1	0	1
lincRNA	AC109809.1	ENSG00000275995.1	0	0
lincRNA	AC060809.1	ENSG00000259543.1	0	0
lincRNA	AC023034.2	ENSG00000259610.1	0	0
lincRNA	AC025219.1	ENSG00000259445.1	0	0
lincRNA	LINC01583	ENSG00000259518.1	0	0
lincRNA	AC245033.2	ENSG00000278013.1	0	0
lincRNA	AC136698.1	ENSG00000259694.1	0	0
lincRNA	AC243562.3	ENSG00000259774.1	0	0
lincRNA	AC048382.4	ENSG00000259551.1	0	0
lincRNA	AC078905.1	ENSG00000259761.1	0	0
lincRNA	AC020687.1	ENSG00000259560.1	0	0
lincRNA	NTRK3-AS1	ENSG00000260305.1	0	0
lincRNA	LINC01586	ENSG00000249487.6	0	0
lincRNA	AC103982.1	ENSG00000259676.1	0	0

lincRNA	AC013565.1	ENSG00000260123.1	0	0
lincRNA	LINC00928	ENSG00000259218.5	0	0
lincRNA	IDH2-DT	ENSG00000259685.2	0	0
lincRNA	AC091167.5	ENSG00000275345.1	0	0
lincRNA	AC021422.1	ENSG00000278370.1	0	0
lincRNA	AC127520.1	ENSG00000285661.1	0	0
lincRNA	AC123784.1	ENSG00000285883.1	0	0
lincRNA	CRAT37	ENSG00000258551.5	0	0
lincRNA	AC107958.3	ENSG00000258954.1	0	0
lincRNA	AC107958.2	ENSG00000258765.1	0	0
lincRNA	AC090985.1	ENSG00000259170.1	0	0
lincRNA	C15orf32	ENSG00000183643.4	0	0
lincRNA	LINC00930	ENSG00000258647.5	0	0
lincRNA	AC106028.4	ENSG00000274215.1	0	0
lincRNA	AC106028.5	ENSG00000275175.1	0	0
lincRNA	AC108457.1	ENSG00000285663.1	0	0
lincRNA	AC112693.1	ENSG00000258888.1	0	0
lincRNA	AC091078.1	ENSG00000257060.6	0	0
lincRNA	AC091078.3	ENSG00000258909.1	0	0
lincRNA	AC110023.1	ENSG00000258631.2	0	0
lincRNA	AC103996.3	ENSG00000259123.1	0	0
lincRNA	LINC02207	ENSG00000258476.5	0	0
lincRNA	AC103996.2	ENSG00000258831.1	0	0
lincRNA	LINC01580	ENSG00000258785.5	0	0
lincRNA	LINC01581	ENSG00000259724.1	0	0
lincRNA	AC009432.2	ENSG00000285866.1	0	0
lincRNA	AC009432.1	ENSG00000259331.1	0	0
lincRNA	AC107976.1	ENSG00000270017.1	0	0
lincRNA	AC087633.1	ENSG00000258433.1	0	0
lincRNA	AC087636.1	ENSG00000258773.1	0	0
lincRNA	LINC01197	ENSG00000248441.6	0	0
lincRNA	AC104260.1	ENSG00000273923.1	0	0
lincRNA	AC104260.3	ENSG00000285700.1	0	0
lincRNA	LINC00924	ENSG00000259134.6	0	0
lincRNA	AC027013.1	ENSG00000258489.3	0	0
lincRNA	AC015574.1	ENSG00000275016.4	0	0
lincRNA	AC012409.2	ENSG00000275443.1	0	0
lincRNA	AC024337.1	ENSG00000259702.3	0	0
lincRNA	AC012409.3	ENSG00000277135.1	0	0
lincRNA	AC012409.1	ENSG00000259359.1	0	0
lincRNA	AC012409.4	ENSG00000278146.1	0	0
lincRNA	LINC02157	ENSG00000259763.2	0	0
lincRNA	AC103746.1	ENSG00000275322.1	0	0
lincRNA	AC087477.5	ENSG00000259542.5	0	0

lincRNA	AC087477.3	ENSG00000259312.1	0	0
lincRNA	AC087477.4	ENSG00000259534.1	0	0
lincRNA	AC020704.1	ENSG00000259403.1	0	0
lincRNA	LINC02254	ENSG00000259664.2	0	0
lincRNA	AC026523.1	ENSG00000259582.3	0	0
lincRNA	LINC00923	ENSG00000251209.8	0	0
lincRNA	AC024651.2	ENSG00000259870.1	0	0
lincRNA	AC024651.1	ENSG00000259478.2	0	0
lincRNA	LINC02251	ENSG00000258483.3	0	0
lincRNA	AC022523.1	ENSG00000259199.1	0	0
lincRNA	LINC01582	ENSG00000259611.1	0	0
lincRNA	AC022523.3	ENSG00000259320.1	0	0
lincRNA	AC015722.1	ENSG00000259041.1	0	0
lincRNA	LINC02351	ENSG00000277987.1	0	0
lincRNA	LUNAR1	ENSG00000278090.1	0	0
lincRNA	LINC02244	ENSG00000259590.1	0	0
lincRNA	AC015660.1	ENSG00000259341.1	0	0
lincRNA	AC090825.1	ENSG00000259363.5	0	0
lincRNA	AC087762.1	ENSG00000259579.1	0	0
lincRNA	AC015712.4	ENSG00000259604.5	0	0
lincRNA	AC019254.2	ENSG00000259365.1	0	0
lincRNA	AC019254.1	ENSG00000259182.5	0	0
lincRNA	AC090164.3	ENSG00000277210.3	0	0
lincRNA	AC090164.4	ENSG00000282793.1	0	0
lincRNA	LINC02348	ENSG00000273585.1	0	0
lincRNA	FAM138E	ENSG00000248893.3	0	0
lincRNA	AC140725.1	ENSG00000259553.5	0	0
lincRNA	WASIR2	ENSG00000231439.4	0	0
lincRNA	AL023881.1	ENSG00000276984.1	0	0
lincRNA	LINC00235	ENSG00000277142.1	0	0
lincRNA	Z97653.1	ENSG00000259840.1	0	0
lincRNA	AL031008.1	ENSG00000275438.1	0	0
lincRNA	AC009041.1	ENSG00000260496.3	0	0
lincRNA	AC009041.3	ENSG00000261720.1	0	0
lincRNA	AL031598.1	ENSG00000260532.1	0	0
lincRNA	AC120498.8	ENSG00000273551.1	0	0
lincRNA	AL031705.1	ENSG00000260646.1	0	0
lincRNA	LINC00254	ENSG00000281219.2	0	0
lincRNA	LINC02124	ENSG00000259947.1	0	0
lincRNA	AC005606.2	ENSG00000261790.1	0	0
lincRNA	AC005606.1	ENSG00000260107.1	0	0
lincRNA	AC009065.7	ENSG00000261532.1	0	0
lincRNA	AC009065.1	ENSG00000259780.3	0	0
lincRNA	AC106820.5	ENSG00000260874.5	0	0

lincRNA	AC141586.2	ENSG00000260176.1	0	0
lincRNA	AC092117.1	ENSG00000276791.1	0	0
lincRNA	AC003965.1	ENSG00000263280.1	0	0
lincRNA	AC004233.4	ENSG00000274367.1	0	0
lincRNA	AC004233.3	ENSG00000272079.2	0	0
lincRNA	AC108134.3	ENSG00000262370.5	0	0
lincRNA	AJ003147.1	ENSG00000262521.1	0	0
lincRNA	LINC00921	ENSG00000281005.1	0	2
lincRNA	AC006111.1	ENSG00000261938.1	0	0
lincRNA	GLIS2-AS1	ENSG00000262686.1	0	0
lincRNA	NAGPA-AS1	ENSG00000267072.2	0	0
lincRNA	AC026458.2	ENSG00000267070.1	0	0
lincRNA	AC074051.5	ENSG00000285567.1	0	0
lincRNA	LINC02164	ENSG00000261811.1	0	0
lincRNA	AC093515.1	ENSG00000260289.1	0	0
lincRNA	AC018767.2	ENSG00000260003.1	0	0
lincRNA	LINC02152	ENSG00000261319.1	0	0
lincRNA	AC018767.3	ENSG00000260058.1	0	0
lincRNA	AC018767.1	ENSG00000259819.1	0	0
lincRNA	AC074052.2	ENSG00000261066.1	0	0
lincRNA	AC022167.3	ENSG00000260979.1	0	0
lincRNA	AC087190.2	ENSG00000261392.1	0	0
lincRNA	LINC02177	ENSG00000261617.1	0	0
lincRNA	LINC01177	ENSG00000261397.1	0	0
lincRNA	LINC01195	ENSG00000261075.1	0	0
lincRNA	AC007221.1	ENSG00000260071.1	0	0
lincRNA	AC007221.2	ENSG00000283003.1	0	0
lincRNA	AC007218.1	ENSG00000260362.1	0	0
lincRNA	AC022168.1	ENSG00000285543.1	0	0
lincRNA	AC131649.1	ENSG00000260756.1	0	0
lincRNA	LINC01290	ENSG00000260468.1	0	0
lincRNA	AC007220.1	ENSG00000263033.2	0	0
lincRNA	BCAR4	ENSG00000262117.5	0	0
lincRNA	AC010333.3	ENSG00000274834.1	0	0
lincRNA	U91319.1	ENSG00000262801.5	0	0
lincRNA	U95743.1	ENSG00000262267.5	0	0
lincRNA	AC010401.2	ENSG00000263234.1	0	0
lincRNA	LINC02185	ENSG00000262097.1	0	1
lincRNA	LINC02186	ENSG00000261856.1	0	0
lincRNA	LINC02130	ENSG00000260009.1	0	0
lincRNA	AC040173.1	ENSG00000263257.2	0	0
lincRNA	AC126763.1	ENSG00000257391.1	0	0
lincRNA	AC138969.2	ENSG00000257366.1	0	0
lincRNA	AC092326.1	ENSG00000260126.1	0	0

lincRNA	AC009152.3	ENSG00000285850.1	0	0
lincRNA	AC109495.1	ENSG00000260945.1	0	0
lincRNA	AC025277.1	ENSG00000261555.1	0	0
lincRNA	AC010601.1	ENSG00000259929.5	0	0
lincRNA	AC091489.1	ENSG00000260242.1	0	0
lincRNA	AC126755.4	ENSG00000257563.1	0	0
lincRNA	AC126755.3	ENSG00000257527.1	0	0
lincRNA	AC136618.1	ENSG00000260550.2	0	0
lincRNA	AC099518.2	ENSG00000261357.1	0	0
lincRNA	AC099518.1	ENSG00000260430.1	0	0
lincRNA	AC130456.8	ENSG00000285869.1	0	0
lincRNA	AC130456.2	ENSG00000259925.1	0	0
lincRNA	AC092132.1	ENSG00000263253.1	0	0
lincRNA	AC141273.1	ENSG00000260201.2	0	0
lincRNA	AC005632.4	ENSG00000273956.1	0	0
lincRNA	AC092375.2	ENSG00000260306.1	0	0
lincRNA	AC009021.2	ENSG00000260973.1	0	0
lincRNA	AC009021.1	ENSG00000260905.1	0	0
lincRNA	AC130466.1	ENSG00000283213.1	0	0
lincRNA	AC127459.2	ENSG00000261090.1	0	0
lincRNA	AC099482.2	ENSG00000285613.1	0	0
lincRNA	LINC2194	ENSG00000260264.1	0	0
lincRNA	AC008938.1	ENSG00000260072.1	0	0
lincRNA	LINC01567	ENSG00000224310.1	0	0
lincRNA	LINC02175	ENSG00000262155.1	0	0
lincRNA	LINC02191	ENSG00000261636.1	0	0
lincRNA	LINC02195	ENSG00000236481.1	0	0
lincRNA	AC009035.1	ENSG00000260580.1	0	0
lincRNA	C16orf82	ENSG00000234186.2	0	0
lincRNA	LINC02129	ENSG00000261045.2	0	0
lincRNA	AC138904.1	ENSG00000246465.1	0	0
lincRNA	AC138894.2	ENSG00000270118.1	0	0
lincRNA	AC009093.2	ENSG00000260517.3	0	0
lincRNA	AC009093.5	ENSG00000273582.1	0	0
lincRNA	AC009093.4	ENSG00000260953.1	0	0
lincRNA	AC106886.3	ENSG00000274678.1	0	0
lincRNA	AC135050.3	ENSG00000260911.2	0	0
lincRNA	AC106730.1	ENSG00000261648.1	0	0
lincRNA	LINC02190	ENSG00000261475.2	0	0
lincRNA	AC074050.2	ENSG00000260568.1	0	0
lincRNA	AC133485.5	ENSG00000260847.1	0	0
lincRNA	AC133485.2	ENSG00000260402.1	0	0
lincRNA	AC138915.3	ENSG00000261541.1	0	0
lincRNA	AC138907.7	ENSG00000261569.5	0	0

lincRNA	AC138907.1	ENSG00000260311.1	0	0
lincRNA	AC138907.2	ENSG00000260845.1	0	0
lincRNA	AC137761.1	ENSG00000260158.1	0	0
lincRNA	AC142086.1	ENSG00000214614.2	0	0
lincRNA	AC142086.4	ENSG00000260921.1	0	0
lincRNA	AC145350.2	ENSG00000260141.1	0	0
lincRNA	AC145350.4	ENSG00000261682.1	0	0
lincRNA	AC138869.3	ENSG00000260827.1	0	0
lincRNA	AC141257.2	ENSG00000261507.1	0	0
lincRNA	AC141257.1	ENSG00000261009.1	0	0
lincRNA	AC136944.1	ENSG00000260626.1	0	0
lincRNA	AC136944.2	ENSG00000261200.1	0	0
lincRNA	AC136944.4	ENSG00000261466.2	0	0
lincRNA	AC140658.2	ENSG00000260312.1	0	0
lincRNA	LINC00273	ENSG00000256642.1	0	0
lincRNA	LINC02184	ENSG00000260854.1	0	0
lincRNA	AC135776.1	ENSG00000179755.3	0	0
lincRNA	AC135776.2	ENSG00000261566.1	0	0
lincRNA	AC023824.5	ENSG00000261800.1	0	0
lincRNA	AC023824.1	ENSG00000260073.2	0	0
lincRNA	AC023824.4	ENSG00000261398.1	0	0
lincRNA	AC023824.3	ENSG00000260958.2	0	0
lincRNA	LINC01566	ENSG00000259841.6	0	0
lincRNA	AC018558.2	ENSG00000261350.1	0	0
lincRNA	AC018558.3	ENSG00000261445.1	0	0
lincRNA	AC018558.1	ENSG00000260857.2	0	0
lincRNA	LINC02167	ENSG00000261122.6	0	0
lincRNA	AC106785.2	ENSG00000260611.1	0	0
lincRNA	AC092368.3	ENSG00000261512.2	0	0
lincRNA	AC018845.1	ENSG00000259821.1	0	0
lincRNA	LINC02133	ENSG00000261231.6	0	0
lincRNA	LINC02192	ENSG00000261325.1	0	0
lincRNA	LINC02134	ENSG00000260450.1	0	0
lincRNA	AC023813.1	ENSG00000259912.1	0	0
lincRNA	AC023813.4	ENSG00000272545.1	0	0
lincRNA	AC044798.1	ENSG00000261421.1	0	0
lincRNA	AC044798.2	ENSG00000261815.1	0	0
lincRNA	AC007861.1	ENSG00000262950.1	0	0
lincRNA	AC007861.2	ENSG00000263110.1	0	0
lincRNA	LINC02179	ENSG00000261623.1	0	0
lincRNA	AC007603.1	ENSG00000261751.1	0	0
lincRNA	LINC02178	ENSG00000261092.1	0	0
lincRNA	AC007493.1	ENSG00000260573.3	0	0
lincRNA	LINC02168	ENSG00000261703.1	0	0

lincRNA	AC025810.1	ENSG00000260042.1	0	0
lincRNA	LINC02127	ENSG00000261637.2	0	0
lincRNA	AC027688.1	ENSG00000260605.1	0	0
lincRNA	AC027688.2	ENSG00000260620.1	0	0
lincRNA	AC087564.1	ENSG00000285367.1	0	1
lincRNA	AC007344.1	ENSG00000260850.2	0	0
lincRNA	LINC01571	ENSG00000260057.5	0	0
lincRNA	LINC00919	ENSG00000260268.5	0	0
lincRNA	LINC02180	ENSG00000261749.2	0	0
lincRNA	AC007333.2	ENSG00000260975.1	0	0
lincRNA	AC007333.1	ENSG00000260478.1	0	0
lincRNA	CASC22	ENSG00000260887.2	0	0
lincRNA	CASC16	ENSG00000249231.7	0	0
lincRNA	AC026462.5	ENSG00000285800.1	0	0
lincRNA	AC026462.3	ENSG00000260963.1	0	0
lincRNA	AC026462.2	ENSG00000260393.1	0	0
lincRNA	AC026462.4	ENSG00000261261.2	0	0
lincRNA	AC007342.4	ENSG00000261804.1	0	0
lincRNA	AC007342.5	ENSG00000262714.1	0	0
lincRNA	AC007907.1	ENSG00000259759.1	0	0
lincRNA	LINC02169	ENSG00000259517.3	0	0
lincRNA	LINC02140	ENSG00000261803.1	0	0
lincRNA	LINC02183	ENSG00000283304.1	0	0
lincRNA	AC106738.1	ENSG00000259711.1	0	0
lincRNA	AC106738.2	ENSG00000259725.1	0	0
lincRNA	AC109136.1	ENSG00000285775.1	0	0
lincRNA	AC109462.3	ENSG00000283423.1	0	0
lincRNA	AC109462.2	ENSG00000263207.2	0	0
lincRNA	AC007336.2	ENSG00000274508.1	0	0
lincRNA	MMP2-AS1	ENSG00000260135.6	0	0
lincRNA	AC007495.1	ENSG00000246379.7	0	0
lincRNA	AC007495.2	ENSG00000260041.5	0	0
lincRNA	AC026461.3	ENSG00000260823.1	0	0
lincRNA	AC092118.1	ENSG00000187185.4	0	0
lincRNA	LINC02137	ENSG00000260186.5	0	0
lincRNA	AC092378.1	ENSG00000245768.6	0	0
lincRNA	AC092378.2	ENSG00000261638.1	0	0
lincRNA	LINC02141	ENSG00000261807.2	0	0
lincRNA	AC018554.1	ENSG00000261436.5	0	0
lincRNA	AC009081.1	ENSG00000261310.1	0	0
lincRNA	AC009110.1	ENSG00000231589.1	0	0
lincRNA	AC138305.1	ENSG00000260658.5	0	0
lincRNA	AC138305.2	ENSG00000261743.1	0	0
lincRNA	LINC02165	ENSG00000261014.1	0	0

lincRNA	AC092337.1	ENSG00000261653.1	0	0
lincRNA	AC093536.2	ENSG00000260715.1	0	0
lincRNA	AC012322.1	ENSG00000261028.1	0	0
lincRNA	AC092131.1	ENSG00000259846.1	0	0
lincRNA	LINC02126	ENSG00000259847.1	0	0
lincRNA	AC009055.2	ENSG00000260834.1	0	0
lincRNA	AC009055.1	ENSG00000260364.1	0	0
lincRNA	AC092138.2	ENSG00000261818.1	0	1
lincRNA	AC092138.1	ENSG00000260004.1	0	0
lincRNA	AC022164.1	ENSG00000260695.1	0	0
lincRNA	AC010542.2	ENSG00000260755.1	0	0
lincRNA	AC018557.2	ENSG00000260558.2	0	0
lincRNA	AC009061.1	ENSG00000261320.1	0	0
lincRNA	AC040162.3	ENSG00000263126.1	0	0
lincRNA	AC099521.1	ENSG00000274698.1	0	0
lincRNA	AC126773.4	ENSG00000275383.1	0	0
lincRNA	AC009137.1	ENSG00000260999.1	0	0
lincRNA	AC027281.1	ENSG00000262890.1	0	0
lincRNA	AC106736.1	ENSG00000261260.1	0	0
lincRNA	LINC02136	ENSG00000261348.1	0	0
lincRNA	AC009097.3	ENSG00000260612.1	0	0
lincRNA	AC009075.1	ENSG00000261673.1	0	0
lincRNA	AC004943.3	ENSG00000279206.1	0	0
lincRNA	AC116667.2	ENSG00000284675.1	0	0
lincRNA	AC140912.1	ENSG00000261227.2	0	0
lincRNA	LINC01568	ENSG00000258779.7	0	0
lincRNA	AC092114.1	ENSG00000260848.1	0	0
lincRNA	AC087565.2	ENSG00000283457.1	0	0
lincRNA	AC087565.1	ENSG00000259817.2	0	0
lincRNA	AC138627.1	ENSG00000261404.7	0	0
lincRNA	AC009120.4	ENSG00000261248.1	0	0
lincRNA	AC009120.3	ENSG00000260884.1	0	0
lincRNA	AC009053.2	ENSG00000261079.1	0	0
lincRNA	AC099508.2	ENSG00000261058.1	0	0
lincRNA	AC009163.5	ENSG00000273971.1	0	0
lincRNA	AC105430.1	ENSG00000261313.1	0	0
lincRNA	AC099511.1	ENSG00000260223.1	0	0
lincRNA	AC010528.1	ENSG00000260983.1	0	0
lincRNA	AC108125.1	ENSG00000273659.1	0	0
lincRNA	LINC02125	ENSG00000250514.1	0	0
lincRNA	AC106729.1	ENSG00000259995.1	0	0
lincRNA	LINC02131	ENSG00000261154.1	0	0
lincRNA	AC092134.3	ENSG00000285538.1	0	0
lincRNA	AC092376.1	ENSG00000261722.1	0	0

lincRNA	AC084064.1	ENSG00000261472.1	0	0
lincRNA	AC009159.2	ENSG00000277214.1	0	0
lincRNA	AC009159.1	ENSG00000275040.1	0	0
lincRNA	LINC01229	ENSG00000260876.5	0	6
lincRNA	LINC01228	ENSG00000261082.1	0	0
lincRNA	AC092130.1	ENSG00000260237.1	0	0
lincRNA	AC022166.1	ENSG00000260706.5	0	0
lincRNA	AC022166.2	ENSG00000261376.1	0	0
lincRNA	LINC01227	ENSG00000260737.5	0	0
lincRNA	ARLNC1	ENSG00000260896.5	0	0
lincRNA	AC092718.1	ENSG00000245059.2	0	0
lincRNA	AC092139.1	ENSG00000272923.1	0	0
lincRNA	AC099524.1	ENSG00000261218.5	0	0
lincRNA	AC092142.2	ENSG00000276519.1	0	0
lincRNA	AC138304.1	ENSG00000261029.1	0	0
lincRNA	AC009117.2	ENSG00000261285.6	0	0
lincRNA	AC010551.3	ENSG00000260858.1	0	0
lincRNA	AC010551.1	ENSG00000260504.1	0	0
lincRNA	LINC02176	ENSG00000260340.1	0	0
lincRNA	LINC02139	ENSG00000278214.1	0	0
lincRNA	AC026469.1	ENSG00000275088.1	0	0
lincRNA	LINC00311	ENSG00000179219.5	0	0
lincRNA	AC133540.1	ENSG00000278716.1	0	0
lincRNA	AC092127.2	ENSG00000270124.1	0	0
lincRNA	AC092127.1	ENSG00000260417.1	0	0
lincRNA	AC092723.5	ENSG00000285163.1	0	0
lincRNA	LINC02132	ENSG00000268804.1	0	0
lincRNA	AC092723.4	ENSG00000285040.1	0	0
lincRNA	AC092723.3	ENSG00000285012.1	0	0
lincRNA	AC135012.1	ENSG00000261177.1	0	0
lincRNA	AC135012.2	ENSG00000268078.1	0	0
lincRNA	AC135012.3	ENSG00000268505.1	0	0
lincRNA	LINC01082	ENSG00000269186.2	0	1
lincRNA	LINC02135	ENSG00000268532.1	0	0
lincRNA	LINC00917	ENSG00000168367.9	0	0
lincRNA	AC092327.2	ENSG00000269826.1	0	0
lincRNA	AC009108.2	ENSG00000260387.1	0	0
lincRNA	AC009154.1	ENSG00000261161.1	0	0
lincRNA	AC009154.2	ENSG00000274478.1	0	0
lincRNA	LINC02188	ENSG00000261175.6	0	0
lincRNA	LINC02189	ENSG00000260026.1	0	0
lincRNA	LINC02181	ENSG00000232190.2	0	0
lincRNA	AC106745.1	ENSG00000261363.2	0	0
lincRNA	AC136285.3	ENSG00000285527.1	0	0

lincRNA	AC136285.1	ENSG00000261095.1	0	0
lincRNA	AC092720.1	ENSG00000260750.5	0	0
lincRNA	AC126696.3	ENSG00000260498.5	0	0
lincRNA	AC126696.1	ENSG00000260177.1	0	0
lincRNA	AC134312.4	ENSG00000261193.1	0	0
lincRNA	AC134312.3	ENSG00000260166.1	0	0
lincRNA	AC134312.1	ENSG00000205037.2	0	0
lincRNA	AC134312.2	ENSG00000260162.2	0	0
lincRNA	AC134312.6	ENSG00000261816.1	0	0
lincRNA	LINC02182	ENSG00000260420.2	0	0
lincRNA	AC138512.1	ENSG00000261273.1	0	0
lincRNA	AC135782.2	ENSG00000259989.1	0	0
lincRNA	LINC00304	ENSG00000180422.3	0	0
lincRNA	LINC02138	ENSG00000205015.1	0	0
lincRNA	FAM157C	ENSG00000260528.4	0	0
lincRNA	AC133919.1	ENSG00000260507.1	0	0
lincRNA	AC133919.2	ENSG00000261172.1	0	0
lincRNA	LINC02193	ENSG00000260923.6	0	0
lincRNA	AC240565.2	ENSG00000280279.1	0	0
lincRNA	AC240565.1	ENSG00000280136.2	0	0
lincRNA	LINC02091	ENSG00000273172.1	0	0
lincRNA	AC144836.1	ENSG00000262213.1	0	0
lincRNA	AC090617.5	ENSG00000263050.1	0	0
lincRNA	AC005696.3	ENSG00000272911.1	0	0
lincRNA	AC005696.4	ENSG00000277200.1	0	0
lincRNA	hsa-mir-1253	ENSG00000272920.1	0	0
lincRNA	AC090282.1	ENSG00000261848.5	0	0
lincRNA	LINC01975	ENSG00000263312.1	0	0
lincRNA	AC087742.1	ENSG00000274363.1	0	1
lincRNA	AC118754.2	ENSG00000262921.1	0	0
lincRNA	LINC01996	ENSG00000261863.1	0	0
lincRNA	AC087500.2	ENSG00000263164.1	0	0
lincRNA	AC004148.1	ENSG00000262099.1	0	0
lincRNA	AC007846.1	ENSG00000284837.1	0	0
lincRNA	AC055872.2	ENSG00000285593.1	0	0
lincRNA	AC004706.1	ENSG00000261996.1	0	0
lincRNA	AC129492.2	ENSG00000263427.1	0	0
lincRNA	AC135178.4	ENSG00000266279.1	0	0
lincRNA	AC002091.2	ENSG00000266389.1	0	0
lincRNA	AC002091.1	ENSG00000265975.1	0	0
lincRNA	AC005695.3	ENSG00000273816.1	0	0
lincRNA	AC027045.2	ENSG00000282882.1	0	0
lincRNA	AC002347.1	ENSG00000261433.1	0	0
lincRNA	AC015908.3	ENSG00000264016.2	0	0

lincRNA	AC015908.4	ENSG00000266114.1	0	0
lincRNA	AC015908.2	ENSG00000263508.6	0	0
lincRNA	AC005725.1	ENSG00000264727.1	0	0
lincRNA	AC005244.2	ENSG00000265400.1	0	0
lincRNA	LINC00670	ENSG00000179136.6	0	0
lincRNA	AC005303.1	ENSG00000273098.2	0	0
lincRNA	LINC02093	ENSG00000234634.2	0	0
lincRNA	AC005304.2	ENSG00000265494.1	0	0
lincRNA	AC005304.1	ENSG00000233852.1	0	0
lincRNA	AC005304.3	ENSG00000266744.1	0	0
lincRNA	AC005224.1	ENSG00000231595.1	0	2
lincRNA	AC005224.2	ENSG00000262786.1	0	0
lincRNA	AC013248.1	ENSG00000226130.1	0	0
lincRNA	LINC02096	ENSG00000238212.2	0	0
lincRNA	AC005772.1	ENSG00000232058.1	0	0
lincRNA	CDRT7	ENSG00000259944.1	0	0
lincRNA	CDRT8	ENSG00000265163.1	0	0
lincRNA	AC005703.1	ENSG00000230971.1	0	0
lincRNA	AC005703.2	ENSG00000237377.2	0	0
lincRNA	AC005703.4	ENSG00000266667.1	0	0
lincRNA	AC005324.2	ENSG00000233193.1	0	0
lincRNA	AC015922.2	ENSG00000265519.1	0	0
lincRNA	LINC02087	ENSG00000237057.2	0	0
lincRNA	AC093484.4	ENSG00000266651.1	0	0
lincRNA	AC098850.2	ENSG00000264765.1	0	0
lincRNA	AC022596.1	ENSG00000264729.1	0	0
lincRNA	AC022596.2	ENSG00000283033.1	0	0
lincRNA	AC104024.1	ENSG00000230709.1	0	1
lincRNA	LINC02090	ENSG00000230969.2	0	0
lincRNA	SMCR2	ENSG00000223979.2	0	0
lincRNA	LINC02076	ENSG00000220161.4	0	0
lincRNA	AC007952.3	ENSG00000230197.6	0	0
lincRNA	AC007952.7	ENSG00000263394.1	0	0
lincRNA	AC007952.4	ENSG00000262202.4	0	0
lincRNA	AC007952.5	ENSG00000262292.2	0	0
lincRNA	AC007952.8	ENSG00000266311.6	0	0
lincRNA	AC106017.1	ENSG00000236022.6	0	0
lincRNA	AC106017.2	ENSG00000281856.1	0	0
lincRNA	AC004448.2	ENSG00000235979.8	0	0
lincRNA	LINC02094	ENSG00000227078.1	0	0
lincRNA	AC004448.1	ENSG00000231477.2	0	0
lincRNA	AC015726.2	ENSG00000276406.1	0	1
lincRNA	AC004702.1	ENSG00000263494.1	0	0
lincRNA	LINC01984	ENSG00000265556.1	0	0

lincRNA	AC015818.8	ENSG00000266925.1	0	0
lincRNA	AC015818.2	ENSG00000260907.1	0	0
lincRNA	AC015818.9	ENSG00000267075.1	0	0
lincRNA	AC015818.4	ENSG00000264422.1	0	0
lincRNA	LINC02088	ENSG00000227685.1	0	0
lincRNA	LINC01563	ENSG00000236819.1	0	0
lincRNA	AC068418.2	ENSG00000265043.1	0	0
lincRNA	AC068418.1	ENSG00000264023.1	0	0
lincRNA	AC233702.5	ENSG00000265265.1	0	0
lincRNA	LINC02002	ENSG00000263609.1	0	1
lincRNA	AC138761.3	ENSG00000264956.1	0	0
lincRNA	FAM27E5	ENSG00000178130.9	0	0
lincRNA	FLJ36000	ENSG00000276399.1	0	0
lincRNA	AC132825.3	ENSG00000261020.1	0	0
lincRNA	AC131274.1	ENSG00000264262.1	0	0
lincRNA	AC069061.2	ENSG00000264734.1	0	0
lincRNA	AC129926.2	ENSG00000285822.1	0	0
lincRNA	AC026254.2	ENSG00000266313.1	0	0
lincRNA	LINC01992	ENSG00000260019.1	0	0
lincRNA	AC061975.7	ENSG00000267644.1	0	0
lincRNA	AC061975.6	ENSG00000266830.1	0	0
lincRNA	AC061975.4	ENSG00000264486.1	0	0
lincRNA	AC024267.1	ENSG00000264066.6	0	0
lincRNA	AC068025.2	ENSG00000266111.2	0	3
lincRNA	AC011840.3	ENSG00000263860.1	0	0
lincRNA	AC011840.2	ENSG00000259928.1	0	0
lincRNA	AC127024.3	ENSG00000265443.1	0	0
lincRNA	AC127024.2	ENSG00000263603.1	0	0
lincRNA	AC138207.5	ENSG00000265743.1	0	0
lincRNA	AC003101.2	ENSG00000278546.1	0	0
lincRNA	AC003101.1	ENSG00000228768.3	0	0
lincRNA	AC007923.4	ENSG00000266877.1	0	0
lincRNA	AC007923.1	ENSG00000263567.1	0	0
lincRNA	AC004253.2	ENSG00000265046.1	0	0
lincRNA	AC004253.1	ENSG00000263990.1	0	0
lincRNA	AC005899.3	ENSG00000265139.1	0	0
lincRNA	AC005899.7	ENSG00000278668.1	0	1
lincRNA	AC005899.5	ENSG00000266385.1	0	0
lincRNA	AC005899.1	ENSG00000264083.1	0	0
lincRNA	AC084809.2	ENSG00000236377.1	0	0
lincRNA	AC024614.1	ENSG00000264643.1	0	0
lincRNA	AC024614.2	ENSG00000265689.1	0	0
lincRNA	AC024614.4	ENSG00000283417.1	0	0
lincRNA	AC024610.2	ENSG00000279668.2	0	0

lincRNA	AC004147.2	ENSG00000263571.2	0	0
lincRNA	LINC01989	ENSG00000261156.6	0	0
lincRNA	AC005549.1	ENSG00000264990.1	0	0
lincRNA	AC011193.1	ENSG00000225582.1	0	0
lincRNA	AC005552.1	ENSG00000264174.1	0	0
lincRNA	AC022903.1	ENSG00000264622.1	0	0
lincRNA	AC060766.6	ENSG00000267711.1	0	1
lincRNA	AC015911.2	ENSG00000267035.1	0	0
lincRNA	AC015911.9	ENSG00000267625.1	0	0
lincRNA	AC015849.2	ENSG00000270829.1	0	0
lincRNA	AC015849.5	ENSG00000270977.1	0	0
lincRNA	AC244100.4	ENSG00000278690.1	0	0
lincRNA	AC243829.5	ENSG00000278097.1	0	0
lincRNA	AC243830.3	ENSG00000276508.1	0	0
lincRNA	AC243830.1	ENSG00000275613.1	0	0
lincRNA	AC243585.1	ENSG00000274244.1	0	0
lincRNA	AC243571.1	ENSG00000275173.1	0	0
lincRNA	AC006441.4	ENSG00000266588.1	0	0
lincRNA	LINC02079	ENSG00000266013.1	0	0
lincRNA	AC090844.2	ENSG00000264968.1	0	0
lincRNA	AC090844.3	ENSG00000265799.1	0	0
lincRNA	AC090283.1	ENSG00000264488.1	0	0
lincRNA	LINC00974	ENSG00000226629.1	0	0
lincRNA	AC099811.3	ENSG00000267658.1	0	0
lincRNA	AC100793.4	ENSG00000273650.1	0	0
lincRNA	RAMP2-AS1	ENSG00000197291.8	0	0
lincRNA	LINC00671	ENSG00000213373.7	0	0
lincRNA	MIR2117HG	ENSG00000267151.5	0	0
lincRNA	LINC02594	ENSG00000267440.1	0	0
lincRNA	FAM215A	ENSG00000267496.4	0	0
lincRNA	AC007993.2	ENSG00000267420.1	0	0
lincRNA	LINC01976	ENSG00000261514.1	0	0
lincRNA	AC003043.2	ENSG00000285486.1	0	0
lincRNA	AC003043.1	ENSG00000267211.1	0	0
lincRNA	LINC01180	ENSG00000266979.1	0	0
lincRNA	AC005180.2	ENSG00000267505.1	0	0
lincRNA	AC005180.1	ENSG00000267405.1	0	0
lincRNA	AC015936.1	ENSG00000267334.1	0	0
lincRNA	AC015936.2	ENSG00000267788.1	0	0
lincRNA	AC003070.2	ENSG00000267446.1	0	0
lincRNA	AC091132.1	ENSG00000131484.4	0	0
lincRNA	AC091132.4	ENSG00000267198.1	0	0
lincRNA	AC126544.2	ENSG00000285668.1	0	0
lincRNA	AC217774.1	ENSG00000265547.1	0	0

lincRNA	AC217774.2	ENSG00000265964.1	0	0
lincRNA	FAM215B	ENSG00000232300.1	0	0
lincRNA	LINC01974	ENSG00000262031.1	0	0
lincRNA	AC005670.1	ENSG00000261886.1	0	0
lincRNA	AC018521.3	ENSG00000264243.1	0	0
lincRNA	AC036222.1	ENSG00000264451.1	0	0
lincRNA	AC091179.1	ENSG00000242407.1	0	0
lincRNA	AC091180.2	ENSG00000248714.6	0	0
lincRNA	LINC02075	ENSG00000249497.1	0	0
lincRNA	AC027801.4	ENSG00000262837.1	0	0
lincRNA	AC027801.1	ENSG00000248954.1	0	0
lincRNA	AC027801.3	ENSG00000254039.1	0	0
lincRNA	AC009720.1	ENSG00000248172.1	0	0
lincRNA	AC015909.1	ENSG00000249406.2	0	1
lincRNA	AC015909.4	ENSG00000263176.1	0	0
lincRNA	AC021491.4	ENSG00000275897.1	0	0
lincRNA	AC021491.1	ENSG00000249451.1	0	0
lincRNA	AC005921.4	ENSG00000285829.1	0	0
lincRNA	AC005920.4	ENSG00000262006.1	0	0
lincRNA	AC005920.1	ENSG00000247011.2	0	0
lincRNA	AC005920.3	ENSG00000251665.1	0	0
lincRNA	LINC02071	ENSG00000249383.2	0	0
lincRNA	AC005823.2	ENSG00000249982.1	0	0
lincRNA	AC005823.1	ENSG00000249658.1	0	0
lincRNA	LINC02073	ENSG00000267452.2	0	0
lincRNA	LINC01982	ENSG00000263317.1	0	0
lincRNA	LINC02089	ENSG00000226364.1	0	0
lincRNA	C17orf112	ENSG00000227011.4	0	0
lincRNA	AC007638.1	ENSG00000262079.1	0	0
lincRNA	AC090618.1	ENSG00000262052.1	0	0
lincRNA	AC015845.2	ENSG00000265542.5	0	0
lincRNA	AC015813.4	ENSG00000278642.1	0	0
lincRNA	AC015813.8	ENSG00000285930.1	0	0
lincRNA	AC015813.3	ENSG00000266290.1	0	0
lincRNA	AC025521.1	ENSG00000274198.1	0	0
lincRNA	LINC01476	ENSG00000265313.6	0	0
lincRNA	RNFT1-DT	ENSG00000267302.5	0	0
lincRNA	AC025048.1	ENSG00000267095.1	0	0
lincRNA	LINC01999	ENSG00000267772.1	0	0
lincRNA	C17orf82	ENSG00000187013.4	0	0
lincRNA	AC080038.2	ENSG00000277463.1	0	0
lincRNA	AC068512.1	ENSG00000265000.1	0	0
lincRNA	AC005972.3	ENSG00000283538.2	0	0
lincRNA	AC005828.4	ENSG00000265282.1	0	0

lincRNA	AC005828.5	ENSG00000265971.1	0	0
lincRNA	AC127029.2	ENSG00000263489.1	0	0
lincRNA	PRR29-AS1	ENSG00000264954.1	0	0
lincRNA	AC037487.1	ENSG00000265912.1	0	0
lincRNA	LINC02563	ENSG00000263520.1	0	0
lincRNA	AC005332.7	ENSG00000278740.1	0	0
lincRNA	AC005332.2	ENSG00000267731.1	0	0
lincRNA	AC079210.1	ENSG00000267461.1	0	0
lincRNA	LINC01482	ENSG00000267659.5	0	0
lincRNA	AC011591.1	ENSG00000267250.1	0	0
lincRNA	AC002546.1	ENSG00000267653.1	0	0
lincRNA	LINC01497	ENSG00000237560.6	0	1
lincRNA	LINC01028	ENSG00000267603.1	0	0
lincRNA	AC011990.1	ENSG00000267109.1	0	0
lincRNA	AC007423.1	ENSG00000271239.1	0	0
lincRNA	CASC17	ENSG00000260785.1	0	0
lincRNA	AC118653.1	ENSG00000225818.1	0	0
lincRNA	AC005144.1	ENSG00000283517.1	0	0
lincRNA	LINC02097	ENSG00000226101.1	0	0
lincRNA	LINC02003	ENSG00000264026.1	0	0
lincRNA	AC007639.1	ENSG00000263680.2	0	0
lincRNA	AC080037.1	ENSG00000263893.2	0	0
lincRNA	AC032019.2	ENSG00000265935.1	0	0
lincRNA	AC125421.1	ENSG00000263574.1	0	0
lincRNA	LINC00469	ENSG00000177338.13	0	0
lincRNA	LINC02092	ENSG00000234721.2	0	0
lincRNA	AC125421.2	ENSG00000265927.1	0	0
lincRNA	AC137735.1	ENSG00000266765.1	0	0
lincRNA	AC137735.2	ENSG00000285648.1	0	0
lincRNA	LINC02074	ENSG00000266357.5	0	0
lincRNA	AC064805.1	ENSG00000261222.2	0	0
lincRNA	LINC00868	ENSG00000267535.1	0	0
lincRNA	AC016168.1	ENSG00000267521.1	0	0
lincRNA	AC068594.2	ENSG00000265121.1	0	0
lincRNA	AC021683.3	ENSG00000267665.1	0	0
lincRNA	AC021683.2	ENSG00000267506.5	0	0
lincRNA	AC021683.1	ENSG00000267466.1	0	0
lincRNA	AC021683.5	ENSG00000285535.1	0	0
lincRNA	LINC01987	ENSG00000267790.1	0	0
lincRNA	LINC01973	ENSG00000204283.3	0	0
lincRNA	AC087645.4	ENSG00000283914.1	0	0
lincRNA	LINC01993	ENSG00000204277.1	0	0
lincRNA	AC087645.2	ENSG00000267737.1	0	1
lincRNA	LINC02078	ENSG00000267719.1	0	0

lincRNA	LINC01977	ENSG00000262772.1	0	0
lincRNA	AC100791.1	ENSG00000262343.1	0	0
lincRNA	LINC01979	ENSG00000262585.1	0	0
lincRNA	LINC01978	ENSG00000262188.1	0	0
lincRNA	AC120024.1	ENSG00000260369.2	0	0
lincRNA	AC127496.5	ENSG00000262873.1	0	2
lincRNA	AC127496.2	ENSG00000262098.1	0	0
lincRNA	LINC00482	ENSG00000185168.5	0	0
lincRNA	AC027601.3	ENSG00000275542.1	0	0
lincRNA	AC110285.3	ENSG00000263154.1	0	0
lincRNA	AC110285.1	ENSG00000262223.7	0	0
lincRNA	AC110285.5	ENSG00000274833.1	0	0
lincRNA	LINC01971	ENSG00000278200.1	0	0
lincRNA	AC145207.6	ENSG00000263859.1	0	0
lincRNA	AC145207.7	ENSG00000264735.1	0	0
lincRNA	AC132938.1	ENSG00000260011.2	0	0
lincRNA	AC024361.2	ENSG00000263063.1	0	0
lincRNA	AC024361.3	ENSG00000263321.1	0	0
lincRNA	AC087222.1	ENSG00000262663.1	0	0
lincRNA	AC130371.2	ENSG00000274370.1	0	0
lincRNA	AC139099.2	ENSG00000262898.1	0	0
lincRNA	AC139099.3	ENSG00000262952.1	0	0
lincRNA	AC139099.1	ENSG00000262094.1	0	0
lincRNA	LINC02564	ENSG00000262352.1	0	0
lincRNA	AP005530.1	ENSG00000263305.1	0	0
lincRNA	AP001005.3	ENSG00000282965.1	0	0
lincRNA	AP000845.1	ENSG00000263884.1	0	0
lincRNA	AP000915.1	ENSG00000264514.1	0	0
lincRNA	LINC01925	ENSG00000264265.1	0	0
lincRNA	LINC01904	ENSG00000259837.1	0	0
lincRNA	AP005328.2	ENSG00000272461.1	0	0
lincRNA	AP005328.1	ENSG00000263551.5	0	0
lincRNA	AC008109.1	ENSG00000266602.1	0	0
lincRNA	AC019183.1	ENSG00000266450.1	0	0
lincRNA	AP005057.1	ENSG00000264080.1	0	0
lincRNA	AP005230.1	ENSG00000263745.6	0	0
lincRNA	AP001025.1	ENSG00000266578.1	0	0
lincRNA	LINC01895	ENSG00000259256.1	0	0
lincRNA	AP005203.1	ENSG00000266268.5	0	0
lincRNA	LINC01892	ENSG00000265514.1	0	0
lincRNA	AP005380.1	ENSG00000266153.1	0	0
lincRNA	AP001496.2	ENSG00000265091.5	0	0
lincRNA	AP001496.1	ENSG00000264254.1	0	0
lincRNA	AP005671.1	ENSG00000266288.1	0	0

lincRNA	MIR3976HG	ENSG00000261738.6	0	0
lincRNA	L3MBTL4-	ENSG00000264707.1	0	0
lincRNA	AP005202.2	ENSG00000266065.1	0	0
lincRNA	LINC01387	ENSG00000265944.1	0	0
lincRNA	AP005205.1	ENSG00000261627.1	0	0
lincRNA	AP001033.1	ENSG00000264964.1	0	0
lincRNA	AP000902.2	ENSG00000285594.1	0	0
lincRNA	AC006238.1	ENSG00000272799.1	0	1
lincRNA	AP005271.1	ENSG00000265554.1	0	0
lincRNA	AP005209.1	ENSG00000264876.1	0	0
lincRNA	AP006219.1	ENSG00000266102.1	0	0
lincRNA	LINC01254	ENSG00000260913.1	0	0
lincRNA	AP001099.1	ENSG00000265728.1	0	0
lincRNA	LINC01887	ENSG00000266604.1	0	0
lincRNA	AP001180.2	ENSG00000264072.1	0	0
lincRNA	AP005229.1	ENSG00000267051.1	0	0
lincRNA	AP005229.2	ENSG00000273119.1	0	0
lincRNA	AP001109.1	ENSG00000267564.1	0	0
lincRNA	LINC01928	ENSG00000267425.1	0	0
lincRNA	LINC01255	ENSG00000267252.5	0	0
lincRNA	AP001120.2	ENSG00000267143.1	0	0
lincRNA	AP001120.1	ENSG00000260759.1	0	0
lincRNA	AP001269.2	ENSG00000267079.1	0	0
lincRNA	AP002414.5	ENSG00000267722.1	0	0
lincRNA	AP005264.4	ENSG00000267643.1	0	0
lincRNA	AP005264.3	ENSG00000267247.1	0	0
lincRNA	AP001029.1	ENSG00000267108.1	0	0
lincRNA	AP005482.2	ENSG00000266961.1	0	0
lincRNA	AP001198.2	ENSG00000267286.1	0	0
lincRNA	LINC01906	ENSG00000265786.1	0	0
lincRNA	AP005121.1	ENSG00000263618.5	0	0
lincRNA	LINC01443	ENSG00000266554.2	0	0
lincRNA	AP005121.2	ENSG00000263821.1	0	0
lincRNA	AP005242.3	ENSG00000278923.2	0	0
lincRNA	AP005242.1	ENSG00000263635.1	0	0
lincRNA	AP005901.2	ENSG00000264880.1	0	0
lincRNA	AC022809.1	ENSG00000265984.1	0	0
lincRNA	AC103987.2	ENSG00000264149.1	0	0
lincRNA	LINC01900	ENSG00000265758.1	0	0
lincRNA	AC091043.1	ENSG00000263588.1	0	0
lincRNA	AC091588.2	ENSG00000264825.1	0	0
lincRNA	AC010754.1	ENSG00000265752.2	0	0
lincRNA	AC023983.2	ENSG00000273321.1	0	0
lincRNA	AC007922.1	ENSG00000264695.1	0	0

lincRNA	AC007922.3	ENSG00000266489.2	0	0
lincRNA	LINC01915	ENSG00000265485.6	0	0
lincRNA	AC018697.1	ENSG00000266573.5	0	0
lincRNA	LINC01894	ENSG00000264345.1	0	0
lincRNA	AC104961.1	ENSG00000265552.1	0	0
lincRNA	LINC01543	ENSG00000263862.1	0	0
lincRNA	PCAT18	ENSG00000265369.3	0	0
lincRNA	AC012588.1	ENSG00000263677.1	0	0
lincRNA	AC018371.1	ENSG00000263382.1	0	0
lincRNA	LINC01908	ENSG00000265374.1	0	0
lincRNA	AC090403.1	ENSG00000264151.5	0	0
lincRNA	AC021382.1	ENSG00000264790.1	0	0
lincRNA	AC068408.1	ENSG00000263611.1	0	0
lincRNA	AC090365.1	ENSG00000265994.2	0	0
lincRNA	AC074237.1	ENSG00000265980.1	0	0
lincRNA	AC117569.1	ENSG00000264587.5	0	0
lincRNA	AC090506.1	ENSG00000266196.1	0	0
lincRNA	AC016382.1	ENSG00000265670.1	0	0
lincRNA	AC021549.1	ENSG00000266521.1	0	0
lincRNA	AC021224.1	ENSG00000262477.1	0	0
lincRNA	AC025887.1	ENSG00000263688.1	0	0
lincRNA	AC091198.1	ENSG00000267642.1	0	0
lincRNA	AC104985.2	ENSG00000278052.1	0	0
lincRNA	AC068506.1	ENSG00000278464.1	0	0
lincRNA	AC015967.1	ENSG00000274400.1	0	0
lincRNA	AC007998.4	ENSG00000275512.1	0	0
lincRNA	AC007998.3	ENSG00000267583.5	0	0
lincRNA	AC118757.1	ENSG00000267627.5	0	0
lincRNA	AC015961.1	ENSG00000267651.1	0	0
lincRNA	AC100781.1	ENSG00000268566.5	0	0
lincRNA	AC011139.1	ENSG00000283458.1	0	0
lincRNA	LINC01901	ENSG00000267413.1	0	0
lincRNA	LINC01902	ENSG00000283503.1	0	0
lincRNA	LINC01477	ENSG00000261715.1	0	0
lincRNA	KC6	ENSG00000267313.7	0	1
lincRNA	LINC00907	ENSG00000267586.6	0	0
lincRNA	AC110014.1	ENSG00000285550.1	0	0
lincRNA	LINC01478	ENSG00000267337.1	0	0
lincRNA	AC120049.1	ENSG00000267414.1	0	1
lincRNA	AC090376.1	ENSG00000267101.6	0	0
lincRNA	AC021517.2	ENSG00000285790.1	0	0
lincRNA	AC090241.3	ENSG00000274776.1	0	0
lincRNA	MIR4527HG	ENSG00000267761.3	0	0
lincRNA	AC102797.1	ENSG00000261307.1	0	0

lincRNA	AC114684.1	ENSG00000266905.1	0	0
lincRNA	AC091170.1	ENSG00000264464.1	0	0
lincRNA	AC090227.2	ENSG00000267674.1	0	0
lincRNA	AC105227.1	ENSG00000267695.1	0	0
lincRNA	LINC01630	ENSG00000227115.7	0	0
lincRNA	AC016383.2	ENSG00000285695.1	0	0
lincRNA	AC016383.1	ENSG00000266335.1	0	0
lincRNA	LINC01919	ENSG00000263438.5	0	0
lincRNA	AC007673.1	ENSG00000267311.1	0	0
lincRNA	LINC01929	ENSG00000267013.5	0	0
lincRNA	AC022031.1	ENSG00000267172.1	0	0
lincRNA	LINC01416	ENSG00000260930.1	0	0
lincRNA	AC006305.1	ENSG00000206129.4	0	0
lincRNA	AC006305.2	ENSG00000267399.1	0	0
lincRNA	LINC01539	ENSG00000267712.5	0	0
lincRNA	AC006305.3	ENSG00000267732.1	0	0
lincRNA	LINC-ROR	ENSG00000258609.3	0	0
lincRNA	AC100775.1	ENSG00000267789.1	0	0
lincRNA	LINC02565	ENSG00000267726.1	0	0
lincRNA	AC090340.1	ENSG00000277837.1	0	0
lincRNA	LINC01897	ENSG00000267209.5	0	0
lincRNA	AC105105.2	ENSG00000267675.1	0	0
lincRNA	MIR122HG	ENSG00000267391.4	0	0
lincRNA	AC105105.4	ENSG00000276403.1	0	0
lincRNA	AC104971.3	ENSG00000275418.1	0	0
lincRNA	AC104365.3	ENSG00000267705.1	0	0
lincRNA	AC104365.2	ENSG00000267501.1	0	0
lincRNA	LINC01926	ENSG00000267593.1	0	0
lincRNA	AC040963.1	ENSG00000267215.1	0	0
lincRNA	AC016229.2	ENSG00000267677.1	0	0
lincRNA	AC010776.3	ENSG00000273664.1	0	1
lincRNA	AC090377.1	ENSG00000267462.1	0	0
lincRNA	AC090771.2	ENSG00000267686.1	0	0
lincRNA	AC090621.1	ENSG00000267401.1	0	0
lincRNA	AC113137.1	ENSG00000267098.1	0	0
lincRNA	AC090409.2	ENSG00000267316.5	0	1
lincRNA	AC105094.2	ENSG00000267175.5	0	0
lincRNA	AC105094.1	ENSG00000267038.1	0	0
lincRNA	LINC01544	ENSG00000260440.2	0	0
lincRNA	LINC01924	ENSG00000267134.2	0	0
lincRNA	LINC00305	ENSG00000179676.6	0	0
lincRNA	LINC01538	ENSG00000266952.2	0	0
lincRNA	AC007948.1	ENSG00000263863.1	0	0
lincRNA	AC007631.1	ENSG00000264131.1	0	0

lincRNA	LINC01916	ENSG00000264232.1	0	0
lincRNA	AC090358.1	ENSG00000265217.1	0	0
lincRNA	AC023394.1	ENSG00000263622.1	0	0
lincRNA	AC022655.1	ENSG00000263594.1	0	0
lincRNA	AC100844.1	ENSG00000264634.1	0	0
lincRNA	AC068112.1	ENSG00000264869.1	0	0
lincRNA	LINC01912	ENSG00000264699.1	0	0
lincRNA	AC005909.1	ENSG00000265425.1	0	0
lincRNA	AC090337.1	ENSG00000264705.1	0	0
lincRNA	AC090337.2	ENSG00000285748.1	0	0
lincRNA	AC026585.1	ENSG00000278532.1	0	0
lincRNA	LIVAR	ENSG00000266304.1	0	0
lincRNA	GTSCR1	ENSG00000263417.4	0	0
lincRNA	AC090415.2	ENSG00000265781.1	0	0
lincRNA	AC091691.2	ENSG00000265484.1	0	0
lincRNA	LINC01541	ENSG00000260676.5	0	0
lincRNA	LINC01899	ENSG00000265352.1	0	0
lincRNA	AC027458.1	ENSG00000260457.2	0	0
lincRNA	AC069114.1	ENSG00000265995.1	0	0
lincRNA	AC091138.1	ENSG00000263958.1	0	0
lincRNA	AC079070.1	ENSG00000265380.1	0	0
lincRNA	AC090125.1	ENSG00000263655.1	0	0
lincRNA	AC090398.1	ENSG00000260569.2	0	0
lincRNA	LINC01922	ENSG00000259779.1	0	0
lincRNA	AC015819.2	ENSG00000278607.1	0	0
lincRNA	AC015819.4	ENSG00000285915.1	0	0
lincRNA	AC116003.3	ENSG00000276993.1	0	0
lincRNA	AC116003.2	ENSG00000266774.1	0	0
lincRNA	LINC01898	ENSG00000261194.1	0	0
lincRNA	AC021506.1	ENSG00000266460.1	0	0
lincRNA	AC090457.1	ENSG00000264108.1	0	0
lincRNA	AC103808.6	ENSG00000266743.1	0	0
lincRNA	LINC01893	ENSG00000264260.1	0	0
lincRNA	AC103808.2	ENSG00000264595.1	0	0
lincRNA	AC103808.4	ENSG00000266014.1	0	0
lincRNA	AC103808.3	ENSG00000265717.1	0	0
lincRNA	AC103808.1	ENSG00000264212.1	0	0
lincRNA	AC103808.5	ENSG00000266614.1	0	0
lincRNA	C18orf65	ENSG00000275763.3	0	1
lincRNA	LINC01927	ENSG00000266312.1	0	0
lincRNA	LINC01879	ENSG00000276397.1	0	0
lincRNA	AC100863.1	ENSG00000265844.1	0	0
lincRNA	AC124254.1	ENSG00000264015.1	0	0
lincRNA	AC123786.1	ENSG00000264693.1	0	0

lincRNA	LINC01029	ENSG00000265843.2	0	0
lincRNA	AC134978.1	ENSG00000264334.1	0	0
lincRNA	AC107892.1	ENSG00000266213.1	0	0
lincRNA	AC087399.1	ENSG00000265644.1	0	0
lincRNA	AC012572.1	ENSG00000265378.1	0	0
lincRNA	AC044873.1	ENSG00000266273.1	0	0
lincRNA	AC091027.2	ENSG00000266586.1	0	0
lincRNA	AC091027.1	ENSG00000265101.1	0	0
lincRNA	LINC01896	ENSG00000263146.2	0	0
lincRNA	AC068473.4	ENSG00000267409.1	0	0
lincRNA	AC068473.1	ENSG00000178412.4	0	0
lincRNA	AC021594.1	ENSG00000266924.1	0	0
lincRNA	AC021594.2	ENSG00000267780.2	0	0
lincRNA	AC114341.1	ENSG00000278075.1	0	0
lincRNA	RBFADN	ENSG00000261126.7	0	0
lincRNA	AC008993.3	ENSG00000282807.3	0	0
lincRNA	FAM138F	ENSG00000282591.1	0	0
lincRNA	AC092192.1	ENSG00000282535.1	0	0
lincRNA	AC016588.2	ENSG00000282393.1	0	0
lincRNA	AC010641.1	ENSG00000267443.2	0	0
lincRNA	AC004449.1	ENSG00000261204.1	0	0
lincRNA	AC006273.1	ENSG00000272473.1	0	0
lincRNA	AC004221.1	ENSG00000267778.1	0	2
lincRNA	AC005330.1	ENSG00000267372.2	0	0
lincRNA	AC005256.1	ENSG00000267073.1	0	0
lincRNA	AC012615.5	ENSG00000267232.1	0	0
lincRNA	LINC01775	ENSG00000267201.1	0	0
lincRNA	AC005786.2	ENSG00000267231.1	0	0
lincRNA	AC011498.1	ENSG00000267011.5	0	0
lincRNA	MIR7-3HG	ENSG00000176840.11	0	0
lincRNA	AC005523.1	ENSG00000268536.1	0	0
lincRNA	AC010336.3	ENSG00000268120.1	0	0
lincRNA	LINC01862	ENSG00000268480.1	0	0
lincRNA	AC008567.2	ENSG00000267273.1	0	0
lincRNA	ZNF426-DT	ENSG00000278611.1	0	6
lincRNA	AC008759.2	ENSG00000267612.1	0	0
lincRNA	AC008752.1	ENSG00000266950.1	0	0
lincRNA	AC008543.1	ENSG00000197332.8	0	0
lincRNA	AC008543.4	ENSG00000267089.1	0	0
lincRNA	AC010422.1	ENSG00000230310.1	0	0
lincRNA	AC018761.1	ENSG00000267004.1	0	0
lincRNA	AC018761.3	ENSG00000267212.1	0	0
lincRNA	AC011509.2	ENSG00000267723.1	0	0
lincRNA	AC011509.1	ENSG00000267406.1	0	0

lincRNA	LINC01841	ENSG00000266913.1	0	0
lincRNA	LINC01842	ENSG00000267147.2	0	0
lincRNA	AC004257.1	ENSG00000269635.1	0	0
lincRNA	AC005776.2	ENSG00000273218.1	0	0
lincRNA	AC093072.1	ENSG00000266951.2	0	0
lincRNA	AC004597.1	ENSG00000268673.3	0	0
lincRNA	LINC01855	ENSG00000267517.1	0	0
lincRNA	AC008894.1	ENSG00000267373.1	0	0
lincRNA	AC020917.3	ENSG00000277825.1	0	1
lincRNA	AC008764.2	ENSG00000268087.1	0	0
lincRNA	AC010319.5	ENSG00000269736.1	0	0
lincRNA	AC011477.4	ENSG00000267581.1	0	0
lincRNA	AC008554.1	ENSG00000269043.1	0	0
lincRNA	AC010636.2	ENSG00000269110.1	0	0
lincRNA	AC010636.1	ENSG00000268560.1	0	0
lincRNA	AC010329.1	ENSG00000268416.1	0	0
lincRNA	AC008739.1	ENSG00000268326.1	0	0
lincRNA	AC123912.4	ENSG00000268555.1	0	0
lincRNA	AC123912.2	ENSG00000268240.1	0	0
lincRNA	AC092364.1	ENSG00000268184.1	0	0
lincRNA	AC003973.2	ENSG00000269504.1	0	0
lincRNA	AC003973.1	ENSG00000269199.1	0	0
lincRNA	AC073539.1	ENSG00000267872.1	0	0
lincRNA	AC073539.3	ENSG00000268597.1	0	0
lincRNA	AC011516.1	ENSG00000269796.1	0	0
lincRNA	AC025811.1	ENSG00000270947.1	0	0
lincRNA	LINC01233	ENSG00000269364.1	0	0
lincRNA	LINC01785	ENSG00000268038.1	0	0
lincRNA	AC022145.1	ENSG00000268296.1	0	0
lincRNA	LINC01859	ENSG00000261558.1	0	0
lincRNA	LINC01858	ENSG00000261615.6	0	0
lincRNA	AC074135.2	ENSG00000269543.5	0	0
lincRNA	AC074135.1	ENSG00000267886.1	0	0
lincRNA	AC092329.1	ENSG00000269107.1	0	0
lincRNA	LINC01224	ENSG00000269416.5	0	0
lincRNA	AC139769.2	ENSG00000267924.1	0	0
lincRNA	ERVK-28	ENSG00000267696.6	0	0
lincRNA	AC006504.2	ENSG00000266977.1	0	0
lincRNA	AC010511.1	ENSG00000267509.5	0	0
lincRNA	AC020905.1	ENSG00000266928.1	0	0
lincRNA	AC005580.1	ENSG00000267320.5	0	0
lincRNA	AC093074.1	ENSG00000267238.1	0	0
lincRNA	AC005381.1	ENSG00000267243.6	0	0
lincRNA	AC005394.1	ENSG00000266893.1	0	0

lincRNA	AC005616.1	ENSG00000267423.1	0	0
lincRNA	AC079466.2	ENSG00000267537.1	0	0
lincRNA	AC007795.1	ENSG00000267617.1	0	0
lincRNA	AC008991.1	ENSG00000267528.1	0	0
lincRNA	AC008991.2	ENSG00000267683.1	0	0
lincRNA	AC011524.1	ENSG00000267027.1	0	0
lincRNA	AC011524.2	ENSG00000267240.1	0	0
lincRNA	LINC00906	ENSG00000267339.5	0	0
lincRNA	LINC01532	ENSG00000267014.5	0	0
lincRNA	AC007786.2	ENSG00000276251.1	0	0
lincRNA	AC007786.3	ENSG00000276631.1	0	0
lincRNA	AC011474.3	ENSG00000267574.1	0	0
lincRNA	AC008507.2	ENSG00000267006.1	0	0
lincRNA	AC011478.1	ENSG00000267760.1	0	0
lincRNA	AC011507.1	ENSG00000267081.1	0	0
lincRNA	LINC01834	ENSG00000261316.1	0	0
lincRNA	AC020897.1	ENSG00000267378.1	0	0
lincRNA	AC020912.1	ENSG00000284430.1	0	0
lincRNA	LINC01791	ENSG00000267777.1	0	0
lincRNA	AC008992.1	ENSG00000267636.1	0	0
lincRNA	AC008794.2	ENSG00000267599.1	0	0
lincRNA	AC008794.1	ENSG00000267407.1	0	0
lincRNA	AC011525.1	ENSG00000261400.1	0	0
lincRNA	AC011525.2	ENSG00000267465.6	0	0
lincRNA	LINC01837	ENSG00000267489.3	0	0
lincRNA	AC011456.1	ENSG00000267012.1	0	0
lincRNA	LINC01533	ENSG00000267779.5	0	0
lincRNA	LINC01782	ENSG00000267204.1	0	0
lincRNA	AC011518.1	ENSG00000267133.1	0	0
lincRNA	AC008736.2	ENSG00000267567.1	0	0
lincRNA	AC008738.6	ENSG00000270212.1	0	0
lincRNA	AC008738.2	ENSG00000267130.1	0	0
lincRNA	AC010485.1	ENSG00000266985.1	0	0
lincRNA	LINC01801	ENSG00000267767.2	0	0
lincRNA	AC008555.1	ENSG00000261754.2	0	0
lincRNA	AC008555.2	ENSG00000269086.2	0	0
lincRNA	AC008555.5	ENSG00000271109.1	0	0
lincRNA	LINC00904	ENSG00000271649.1	0	0
lincRNA	AC008555.3	ENSG00000270544.1	0	0
lincRNA	AC008555.6	ENSG00000271171.1	0	0
lincRNA	LINC01838	ENSG00000269037.1	0	0
lincRNA	ZNF30-AS1	ENSG00000270876.1	0	0
lincRNA	LINC01531	ENSG00000205786.8	0	0
lincRNA	AC002511.2	ENSG00000233214.1	0	0

lincRNA	AC002511.1	ENSG00000232680.2	0	0
lincRNA	LINC01766	ENSG00000224910.1	0	0
lincRNA	AC002115.1	ENSG00000267626.1	0	0
lincRNA	AF038458.3	ENSG00000267786.1	0	0
lincRNA	AF038458.2	ENSG00000267388.1	0	0
lincRNA	AC012617.1	ENSG00000267053.6	0	0
lincRNA	AC092296.3	ENSG00000267363.1	0	0
lincRNA	AC074138.1	ENSG00000276071.1	0	1
lincRNA	AC020928.1	ENSG00000267260.1	0	0
lincRNA	AC020928.2	ENSG00000267353.1	0	0
lincRNA	LINC01535	ENSG00000226686.7	0	0
lincRNA	AC016590.1	ENSG00000267605.5	0	0
lincRNA	AC022148.2	ENSG00000267115.1	0	0
lincRNA	AC005515.1	ENSG00000269460.1	0	0
lincRNA	AC005393.1	ENSG00000276445.1	0	1
lincRNA	AC010271.2	ENSG00000277453.1	0	1
lincRNA	AC008537.2	ENSG00000269843.1	0	0
lincRNA	PCAT19	ENSG00000267107.6	0	0
lincRNA	AC243960.3	ENSG00000268355.1	0	0
lincRNA	LINC01480	ENSG00000270164.1	0	0
lincRNA	AC243960.1	ENSG00000268027.5	0	0
lincRNA	AC243967.3	ENSG00000278239.1	0	0
lincRNA	PSG8-AS1	ENSG00000225877.1	0	0
lincRNA	AC005392.1	ENSG00000227606.2	0	0
lincRNA	AC005392.2	ENSG00000231412.2	0	0
lincRNA	AC115522.1	ENSG00000268601.1	0	0
lincRNA	AC067968.2	ENSG00000267144.1	0	0
lincRNA	AC245748.3	ENSG00000267614.1	0	0
lincRNA	AC092306.1	ENSG00000285128.1	0	0
lincRNA	AC007785.1	ENSG00000267922.1	0	0
lincRNA	AC006262.2	ENSG00000269729.1	0	0
lincRNA	LINC01595	ENSG00000259108.2	0	0
lincRNA	AC008403.3	ENSG00000269814.1	0	0
lincRNA	AC026803.2	ENSG00000267898.1	0	0
lincRNA	AC008687.3	ENSG00000268287.1	0	0
lincRNA	AC020909.2	ENSG00000268518.1	0	0
lincRNA	LINC01869	ENSG00000180279.6	0	1
lincRNA	AC011473.1	ENSG00000268390.1	0	0
lincRNA	LINC01872	ENSG00000268318.5	0	0
lincRNA	AC008750.7	ENSG00000268889.1	0	0
lincRNA	AC018755.1	ENSG00000267808.1	0	0
lincRNA	SPACA6P-AS	ENSG00000269959.1	0	0
lincRNA	AC006272.1	ENSG00000268316.1	0	0
lincRNA	AC011468.3	ENSG00000268458.1	0	0

lincRNA	AC010328.1	ENSG00000267943.1	0	0
lincRNA	AC092070.4	ENSG00000269758.1	0	0
lincRNA	AC011453.1	ENSG00000269842.5	0	0
lincRNA	AC008753.3	ENSG00000269877.3	0	0
lincRNA	AC008753.2	ENSG00000269564.1	0	0
lincRNA	AC008440.3	ENSG00000232324.1	0	0
lincRNA	AC245052.1	ENSG00000223660.1	0	0
lincRNA	AC245052.7	ENSG00000269524.1	0	0
lincRNA	AC245884.10	ENSG00000269873.1	0	0
lincRNA	AC245036.5	ENSG00000269271.1	0	0
lincRNA	AC245128.3	ENSG00000268734.1	0	0
lincRNA	AC010327.4	ENSG00000267649.1	0	0
lincRNA	AC020922.4	ENSG00000276831.1	0	0
lincRNA	LINC01864	ENSG00000267522.1	0	0
lincRNA	AC006116.9	ENSG00000267606.1	0	0
lincRNA	AC006115.1	ENSG00000268352.1	0	0
lincRNA	MIMT1	ENSG00000268654.1	0	0
lincRNA	AC003005.2	ENSG00000268266.1	0	0
lincRNA	LINC02560	ENSG00000268307.1	0	0
lincRNA	AL034548.1	ENSG00000272874.1	0	0
lincRNA	AL121760.1	ENSG00000230839.1	0	0
lincRNA	AL121899.1	ENSG00000226644.5	0	0
lincRNA	AL356414.1	ENSG00000205300.3	0	1
lincRNA	AL139350.1	ENSG00000236387.1	0	0
lincRNA	AL121781.1	ENSG00000238282.1	0	0
lincRNA	PRNT	ENSG00000180259.9	0	0
lincRNA	AL133396.2	ENSG00000278595.1	0	0
lincRNA	AL121890.3	ENSG00000277087.1	0	0
lincRNA	AL121890.2	ENSG00000276768.1	0	0
lincRNA	AL121890.5	ENSG00000278816.1	0	0
lincRNA	AL121890.4	ENSG00000277425.1	0	0
lincRNA	AL121757.2	ENSG00000276688.1	0	0
lincRNA	LINC00658	ENSG00000226995.7	0	0
lincRNA	AL121757.1	ENSG00000230563.2	0	0
lincRNA	AL109935.2	ENSG00000266908.1	0	0
lincRNA	LINC01729	ENSG00000225640.1	0	0
lincRNA	CASC20	ENSG00000229876.1	0	0
lincRNA	LINC01713	ENSG00000228482.1	0	0
lincRNA	AL096799.1	ENSG00000232271.1	0	0
lincRNA	LINC01428	ENSG00000228888.1	0	0
lincRNA	AL080248.1	ENSG00000238102.1	0	0
lincRNA	LINC01751	ENSG00000261249.1	0	0
lincRNA	LINC01706	ENSG00000224711.1	0	0
lincRNA	PARAL1	ENSG00000243961.2	0	0

lincRNA	AL354824.1	ENSG00000230506.1	0	0
lincRNA	AL354824.2	ENSG00000232448.1	0	0
lincRNA	AL135937.1	ENSG00000234900.1	0	0
lincRNA	AL050403.1	ENSG00000232900.1	0	0
lincRNA	AL158042.1	ENSG00000270777.1	0	0
lincRNA	C20orf187	ENSG00000125899.7	0	0
lincRNA	AL049649.1	ENSG00000230990.1	0	0
lincRNA	AL109837.2	ENSG00000282478.1	0	0
lincRNA	AL161938.1	ENSG00000261411.1	0	0
lincRNA	LINC00687	ENSG00000228422.3	0	0
lincRNA	AL109838.1	ENSG00000235292.1	0	0
lincRNA	AL136460.1	ENSG00000230437.1	0	0
lincRNA	AL078623.1	ENSG00000269931.1	0	0
lincRNA	LINC01723	ENSG00000234265.1	0	0
lincRNA	AL121782.1	ENSG00000278153.1	0	0
lincRNA	AL049794.2	ENSG00000275728.1	0	0
lincRNA	AL121892.1	ENSG00000236985.1	0	0
lincRNA	AL035045.1	ENSG00000233625.1	0	0
lincRNA	AL049646.1	ENSG00000230010.1	0	0
lincRNA	LINC00652	ENSG00000179935.9	0	0
lincRNA	AL135936.1	ENSG00000274863.1	0	0
lincRNA	AL121761.1	ENSG00000268628.2	0	0
lincRNA	AL133465.1	ENSG00000234104.1	0	0
lincRNA	LINC00237	ENSG00000225127.2	0	0
lincRNA	AL121759.2	ENSG00000283405.1	0	0
lincRNA	AL133325.3	ENSG00000278041.1	0	0
lincRNA	AL133325.2	ENSG00000233492.1	0	0
lincRNA	LINC01727	ENSG00000279082.3	0	0
lincRNA	LINC01726	ENSG00000280350.1	0	0
lincRNA	AL109807.1	ENSG00000238034.1	0	0
lincRNA	LINC01432	ENSG00000234435.2	0	0
lincRNA	AL035258.1	ENSG00000234862.1	0	0
lincRNA	LINC01427	ENSG00000225321.2	0	0
lincRNA	AL133464.1	ENSG00000204684.3	0	0
lincRNA	LINC00261	ENSG00000259974.2	0	0
lincRNA	LNCNEF	ENSG00000237396.1	0	0
lincRNA	LINC01747	ENSG00000230400.2	0	0
lincRNA	AL158175.1	ENSG00000283072.1	0	0
lincRNA	LINC00656	ENSG00000233746.1	0	0
lincRNA	AL118508.2	ENSG00000275179.1	0	0
lincRNA	AL118508.1	ENSG00000230387.2	0	0
lincRNA	AL390037.1	ENSG00000277901.1	0	0
lincRNA	AL109954.2	ENSG00000273838.1	0	0
lincRNA	AL109954.1	ENSG00000260202.2	0	0

lincRNA	AL109954.3	ENSG00000276638.1	0	0
lincRNA	AL121894.1	ENSG00000225056.1	0	0
lincRNA	AL110503.1	ENSG00000236151.1	0	0
lincRNA	LINC01721	ENSG00000230133.1	0	0
lincRNA	AL158090.1	ENSG00000232200.2	0	0
lincRNA	AL049594.1	ENSG00000231015.1	0	0
lincRNA	AL080312.2	ENSG00000275358.1	0	0
lincRNA	AL035252.2	ENSG00000230725.5	0	0
lincRNA	AL121772.1	ENSG00000274414.1	0	0
lincRNA	LINC01733	ENSG00000226203.1	0	0
lincRNA	AL078587.1	ENSG00000231081.1	0	0
lincRNA	AL391119.1	ENSG00000230440.1	0	0
lincRNA	AL121904.1	ENSG00000238129.5	0	0
lincRNA	LINC01597	ENSG00000205611.4	0	3
lincRNA	LINC00028	ENSG00000233354.1	0	0
lincRNA	AL110115.2	ENSG00000278276.1	0	0
lincRNA	NOL4L-DT	ENSG00000204393.7	0	0
lincRNA	AL121906.2	ENSG00000275223.1	0	0
lincRNA	AL031668.1	ENSG00000228386.2	0	0
lincRNA	AL035420.1	ENSG00000234139.2	0	0
lincRNA	AL035420.2	ENSG00000237063.1	0	0
lincRNA	LINC01746	ENSG00000274469.1	0	0
lincRNA	LINC00489	ENSG00000225759.1	0	0
lincRNA	AL162293.1	ENSG00000204117.1	0	0
lincRNA	AL031651.1	ENSG00000277550.1	0	0
lincRNA	AL359555.2	ENSG00000285100.1	0	0
lincRNA	AL391095.2	ENSG00000275285.1	0	0
lincRNA	AL391095.1	ENSG00000224635.1	0	0
lincRNA	AL391095.3	ENSG00000275401.1	0	0
lincRNA	AL023803.2	ENSG00000274825.1	0	0
lincRNA	LINC01734	ENSG00000230324.1	0	0
lincRNA	AL118523.1	ENSG00000229976.1	0	0
lincRNA	LINC01370	ENSG00000237767.1	0	0
lincRNA	AL133419.1	ENSG00000233415.1	0	0
lincRNA	AL009050.1	ENSG00000223410.1	0	0
lincRNA	AL035665.1	ENSG00000229771.2	0	0
lincRNA	AL049812.3	ENSG00000228959.1	0	0
lincRNA	AL021395.1	ENSG00000233508.2	0	0
lincRNA	Z98752.4	ENSG00000282876.1	0	0
lincRNA	LINC01728	ENSG00000233277.1	0	0
lincRNA	LINC01620	ENSG00000168746.8	0	0
lincRNA	LINC01260	ENSG00000283440.1	0	0
lincRNA	AL139352.1	ENSG00000132832.10	0	0
lincRNA	AL031687.1	ENSG00000229957.1	0	0

lincRNA	LINC01754	ENSG00000224565.1	0	0
lincRNA	AL357558.2	ENSG00000234967.1	0	0
lincRNA	AL357558.1	ENSG00000228503.1	0	0
lincRNA	LINC01522	ENSG00000237423.1	0	0
lincRNA	LINC01523	ENSG00000229522.1	0	0
lincRNA	LINC00494	ENSG00000235621.8	0	0
lincRNA	AL137078.1	ENSG00000236874.1	0	0
lincRNA	AL137078.2	ENSG00000274261.1	0	0
lincRNA	AL049541.1	ENSG00000281365.1	0	0
lincRNA	AL049541.2	ENSG00000281460.1	0	0
lincRNA	AL133342.1	ENSG00000278231.1	0	0
lincRNA	TRERNA1	ENSG00000231265.1	0	0
lincRNA	AL161937.1	ENSG00000234698.1	0	0
lincRNA	LINC01271	ENSG00000233077.1	0	1
lincRNA	LINC01429	ENSG00000227964.1	0	0
lincRNA	LINC01524	ENSG00000234948.1	0	0
lincRNA	AL109610.1	ENSG00000225785.1	0	0
lincRNA	AL049765.1	ENSG00000232465.1	0	0
lincRNA	AL031674.1	ENSG00000232294.5	0	0
lincRNA	AL121902.1	ENSG00000232286.1	0	0
lincRNA	AC005808.1	ENSG00000235415.1	0	0
lincRNA	AC006076.1	ENSG00000225563.1	0	0
lincRNA	LINC01440	ENSG00000235166.7	0	0
lincRNA	LINC01441	ENSG00000224008.1	0	0
lincRNA	AL160413.1	ENSG00000275923.1	0	0
lincRNA	AL139824.1	ENSG00000230843.1	0	0
lincRNA	LINC01716	ENSG00000225657.2	0	0
lincRNA	AL133232.1	ENSG00000231604.2	0	0
lincRNA	AL157414.2	ENSG00000231078.1	0	0
lincRNA	AL157414.1	ENSG00000203266.2	0	0
lincRNA	AL122058.1	ENSG00000226308.1	0	0
lincRNA	AL035541.1	ENSG00000227297.1	0	0
lincRNA	AL354984.1	ENSG00000203971.1	0	0
lincRNA	LINC01742	ENSG00000275852.1	0	0
lincRNA	AL354984.2	ENSG00000225708.6	0	0
lincRNA	AL050327.1	ENSG00000254620.1	0	0
lincRNA	AL132655.3	ENSG00000270279.1	0	0
lincRNA	AL132655.2	ENSG00000268649.5	0	0
lincRNA	AL132655.1	ENSG00000268333.1	0	0
lincRNA	AL035250.1	ENSG00000225129.1	0	0
lincRNA	AL035250.2	ENSG00000230352.1	0	0
lincRNA	AL389889.1	ENSG00000227282.1	0	0
lincRNA	AL132822.1	ENSG00000276093.1	0	0
lincRNA	AL117372.1	ENSG00000276627.1	0	0

lincRNA	AL139348.1	ENSG00000261035.1	0	0
lincRNA	AL121910.1	ENSG00000229755.1	0	0
lincRNA	LINC01718	ENSG00000229606.1	0	0
lincRNA	BX640515.1	ENSG00000276786.1	0	0
lincRNA	AL160412.1	ENSG00000277270.1	0	0
lincRNA	AL499627.2	ENSG00000272050.1	0	0
lincRNA	AL499627.1	ENSG00000260542.1	0	0
lincRNA	BX640514.1	ENSG00000264490.3	0	0
lincRNA	BX640514.2	ENSG00000273812.1	0	0
lincRNA	LINC00686	ENSG00000237687.2	0	0
lincRNA	LINC01749	ENSG00000272259.5	0	0
lincRNA	LINC00029	ENSG00000125514.9	0	0
lincRNA	LINC01056	ENSG00000237119.1	0	0
lincRNA	AL096828.2	ENSG00000260416.1	0	0
lincRNA	AL096828.1	ENSG00000231977.1	0	0
lincRNA	AL096828.3	ENSG00000273821.1	0	0
lincRNA	LINC00266-1	ENSG00000149656.8	0	0
lincRNA	AC079801.1	ENSG00000279669.1	0	0
lincRNA	LINC01670	ENSG00000279094.3	0	0
lincRNA	CU633967.1	ENSG00000274333.4	0	0
lincRNA	FP236315.3	ENSG00000279784.1	0	0
lincRNA	FP236315.1	ENSG00000279064.1	0	0
lincRNA	LINC01669	ENSG00000280191.3	0	0
lincRNA	CU639417.3	ENSG00000280095.1	0	0
lincRNA	CU639417.4	ENSG00000280179.1	0	0
lincRNA	CU633906.1	ENSG00000275496.4	0	0
lincRNA	CU633906.2	ENSG00000278903.3	0	0
lincRNA	FP236240.1	ENSG00000278927.1	0	0
lincRNA	CU638689.4	ENSG00000280145.3	0	0
lincRNA	CU638689.1	ENSG00000278878.1	0	0
lincRNA	CU638689.5	ENSG00000280164.1	0	0
lincRNA	FP475955.3	ENSG00000279998.1	0	0
lincRNA	FP475955.1	ENSG00000278955.3	0	0
lincRNA	FP475955.2	ENSG00000279751.1	0	0
lincRNA	CU634019.6	ENSG00000280018.3	0	0
lincRNA	CU634019.4	ENSG00000279313.1	0	0
lincRNA	CU634019.2	ENSG00000278884.1	0	0
lincRNA	CU634019.1	ENSG00000277067.4	0	0
lincRNA	CU633904.1	ENSG00000276077.4	0	0
lincRNA	FP236241.1	ENSG00000277991.4	0	0
lincRNA	FP671120.1	ENSG00000278996.1	0	0
lincRNA	FP671120.3	ENSG00000280800.1	0	0
lincRNA	FP671120.4	ENSG00000281383.1	0	0
lincRNA	FP236383.1	ENSG00000280441.2	0	0

lincRNA	FP236383.2	ENSG00000280614.1	0	0
lincRNA	FP236383.3	ENSG00000281181.1	0	0
lincRNA	LINC01666	ENSG00000279579.3	0	0
lincRNA	CR392039.3	ENSG00000279501.1	0	0
lincRNA	CR381653.1	ENSG00000278932.3	0	0
lincRNA	LINC01667	ENSG00000280081.3	0	0
lincRNA	AC018692.1	ENSG00000279321.1	0	0
lincRNA	CR382287.2	ENSG00000279851.2	0	0
lincRNA	AP003900.1	ENSG00000277693.1	0	0
lincRNA	AP001464.1	ENSG00000229306.1	0	0
lincRNA	AJ239318.1	ENSG00000280108.2	0	0
lincRNA	LINC01674	ENSG00000228159.1	0	0
lincRNA	AL050303.1	ENSG00000224922.1	0	0
lincRNA	AF127936.2	ENSG00000232884.7	0	0
lincRNA	AF127936.1	ENSG00000226751.2	0	0
lincRNA	AF127577.5	ENSG00000236471.1	0	0
lincRNA	AJ009632.2	ENSG00000229425.2	0	0
lincRNA	AJ009632.1	ENSG00000224247.1	0	0
lincRNA	AP001172.1	ENSG00000270071.1	0	0
lincRNA	AP001172.2	ENSG00000270139.1	0	0
lincRNA	AP000962.1	ENSG00000269950.1	0	0
lincRNA	AF130359.1	ENSG00000237735.2	0	0
lincRNA	AF212831.1	ENSG00000232886.1	0	0
lincRNA	LINC01549	ENSG00000232560.6	0	0
lincRNA	AP000432.1	ENSG00000226956.1	0	0
lincRNA	C21orf91-	ENSG00000240770.5	0	0
lincRNA	CHODL-AS1	ENSG00000231755.1	0	0
lincRNA	AF130417.1	ENSG00000227330.1	0	0
lincRNA	MIR548XHG	ENSG00000224141.5	0	0
lincRNA	AF240627.1	ENSG00000230233.1	0	0
lincRNA	AL157359.2	ENSG00000232193.1	0	0
lincRNA	AL157359.1	ENSG00000226204.1	0	0
lincRNA	AP000431.2	ENSG00000235965.2	0	0
lincRNA	AP000431.1	ENSG00000229289.1	0	0
lincRNA	AP000855.1	ENSG00000231620.1	0	0
lincRNA	LINC01683	ENSG00000233480.1	0	0
lincRNA	LINC02573	ENSG00000233236.1	0	0
lincRNA	LINC00320	ENSG00000224924.6	0	0
lincRNA	AP001117.1	ENSG00000234730.1	0	0
lincRNA	AF241725.1	ENSG00000237527.1	0	0
lincRNA	LINC00317	ENSG00000238265.1	0	0
lincRNA	LINC01425	ENSG00000233997.5	0	0
lincRNA	AP000472.1	ENSG00000227075.1	0	0
lincRNA	LINC01687	ENSG00000233215.5	0	0

lincRNA	LINC00308	ENSG00000184856.6	0	0
lincRNA	AP000561.1	ENSG00000226043.1	0	0
lincRNA	AP000959.1	ENSG00000234034.1	0	0
lincRNA	AP001116.1	ENSG00000230972.1	0	0
lincRNA	D21S2088E	ENSG00000228592.1	0	0
lincRNA	AP000459.1	ENSG00000227716.1	0	0
lincRNA	AP000474.1	ENSG00000231986.1	0	0
lincRNA	AP000477.2	ENSG00000235564.1	0	0
lincRNA	AP000477.1	ENSG00000226996.5	0	0
lincRNA	AP000470.1	ENSG00000224018.1	0	0
lincRNA	LINC01689	ENSG00000224832.2	0	0
lincRNA	LINC01692	ENSG00000226983.2	0	0
lincRNA	AP000402.1	ENSG00000227090.1	0	0
lincRNA	AP000146.1	ENSG00000230379.5	0	0
lincRNA	AP000235.1	ENSG00000244278.1	0	0
lincRNA	AP000233.2	ENSG00000232512.6	0	0
lincRNA	AP000233.1	ENSG00000223870.1	0	0
lincRNA	AP001341.1	ENSG00000222042.1	0	0
lincRNA	LINC00158	ENSG00000185433.8	0	0
lincRNA	AP000221.1	ENSG00000229962.1	0	0
lincRNA	AP000229.1	ENSG00000273492.5	0	0
lincRNA	AP001595.1	ENSG00000229025.1	0	0
lincRNA	AP001596.2	ENSG00000273115.1	0	0
lincRNA	AP001605.1	ENSG00000231236.2	0	0
lincRNA	AP001604.1	ENSG00000236332.1	0	0
lincRNA	LINC01673	ENSG00000234052.1	0	0
lincRNA	LINC00113	ENSG00000225298.5	0	0
lincRNA	AJ006995.1	ENSG00000234083.1	0	0
lincRNA	LINC00314	ENSG00000178457.3	0	0
lincRNA	LINC01695	ENSG00000236532.5	0	0
lincRNA	LINC00161	ENSG00000226935.6	0	0
lincRNA	BACH1-AS1	ENSG00000232118.2	0	0
lincRNA	BACH1-IT2	ENSG00000228817.4	0	2
lincRNA	AF096876.1	ENSG00000259981.1	0	0
lincRNA	LINC00307	ENSG00000227342.1	0	0
lincRNA	AP000251.1	ENSG00000237594.2	0	0
lincRNA	AP000253.1	ENSG00000234509.1	0	0
lincRNA	LINC00159	ENSG00000230323.5	0	0
lincRNA	AP000265.1	ENSG00000261610.1	0	0
lincRNA	LINC01690	ENSG00000228961.1	0	0
lincRNA	LINC00945	ENSG00000232539.1	0	0
lincRNA	AP000289.1	ENSG00000226527.1	0	0
lincRNA	LINC01548	ENSG00000229086.4	0	0
lincRNA	AP000569.1	ENSG00000273102.1	0	0

lincRNA	LINC00310	ENSG00000227456.7	0	0
lincRNA	AP000317.1	ENSG00000214955.5	0	0
lincRNA	AP000322.2	ENSG00000273104.1	0	0
lincRNA	AP000322.1	ENSG00000272958.1	0	0
lincRNA	LINC00160	ENSG00000230978.1	0	0
lincRNA	AF015262.1	ENSG00000234703.1	0	0
lincRNA	AF015720.1	ENSG00000230794.1	0	0
lincRNA	AP000688.3	ENSG00000236119.1	0	0
lincRNA	AP000688.2	ENSG00000233393.1	0	0
lincRNA	AP000696.1	ENSG00000231324.1	0	0
lincRNA	AP000704.1	ENSG00000224790.2	0	0
lincRNA	DSCR9	ENSG00000230366.9	0	0
lincRNA	AP001432.1	ENSG00000242553.1	0	1
lincRNA	DSCR4	ENSG00000184029.9	0	0
lincRNA	DSCR8	ENSG00000198054.12	0	0
lincRNA	AP001434.1	ENSG00000226012.1	0	0
lincRNA	LINC01423	ENSG00000231231.5	0	0
lincRNA	LINC00114	ENSG00000223806.7	0	0
lincRNA	AP001042.1	ENSG00000229986.1	0	0
lincRNA	AP001043.2	ENSG00000229925.1	0	0
lincRNA	LINC01700	ENSG00000232837.1	0	0
lincRNA	AF064858.1	ENSG00000235888.1	0	0
lincRNA	AF064858.3	ENSG00000237721.1	0	0
lincRNA	AF064858.2	ENSG00000237609.1	0	0
lincRNA	AF064860.2	ENSG00000231713.2	0	0
lincRNA	AP001610.2	ENSG00000232806.1	0	0
lincRNA	AP006748.1	ENSG00000223400.1	0	0
lincRNA	LINC00111	ENSG00000227702.1	0	0
lincRNA	ZNF295-AS1	ENSG00000237232.7	0	2
lincRNA	LINC01671	ENSG00000225431.1	0	0
lincRNA	LINC01668	ENSG00000283051.1	0	0
lincRNA	AP001628.2	ENSG00000233754.2	0	0
lincRNA	AP001628.1	ENSG00000225218.1	0	0
lincRNA	ERVH48-1	ENSG00000233056.2	0	0
lincRNA	AP001631.1	ENSG00000228120.2	0	0
lincRNA	LINC00322	ENSG00000237864.1	0	0
lincRNA	AP001046.1	ENSG00000225637.1	0	0
lincRNA	LINC00319	ENSG00000188660.3	0	0
lincRNA	LINC00313	ENSG00000185186.8	0	0
lincRNA	AP001048.1	ENSG00000223975.1	0	0
lincRNA	LINC01678	ENSG00000225331.1	0	0
lincRNA	AP001056.2	ENSG00000285413.1	0	0
lincRNA	AP001056.1	ENSG00000237604.1	0	0
lincRNA	AP001059.3	ENSG00000278158.1	0	0

lincRNA	AP001059.2	ENSG00000275799.1	0	0
lincRNA	AP001063.1	ENSG00000260256.1	0	0
lincRNA	LINC02575	ENSG00000228709.1	0	0
lincRNA	AP001065.2	ENSG00000277352.1	0	0
lincRNA	LINC00163	ENSG00000234880.1	0	0
lincRNA	LINC00165	ENSG00000261706.1	0	1
lincRNA	PICSAR	ENSG00000275874.1	0	0
lincRNA	AL133499.1	ENSG00000267857.2	0	0
lincRNA	LINC00334	ENSG00000182586.8	0	0
lincRNA	LINC00315	ENSG00000184274.3	0	0
lincRNA	BX322557.1	ENSG00000229382.1	0	0
lincRNA	LINC00316	ENSG00000237664.1	0	0
lincRNA	LINC01694	ENSG00000233922.2	0	0
lincRNA	AJ011932.1	ENSG00000274248.1	0	3
lincRNA	AP001476.1	ENSG00000224413.1	0	0
lincRNA	AP001476.3	ENSG00000228235.1	0	0
lincRNA	AP001476.2	ENSG00000226115.1	0	0
lincRNA	CU104787.1	ENSG00000279973.2	0	0
lincRNA	AP000542.3	ENSG00000280341.1	0	0
lincRNA	AP000542.2	ENSG00000279442.1	0	0
lincRNA	AP000532.2	ENSG00000230471.1	0	0
lincRNA	LINC01297	ENSG00000225255.6	0	0
lincRNA	AC145543.1	ENSG00000233866.1	0	0
lincRNA	AP000547.2	ENSG00000273362.1	0	0
lincRNA	AP000547.3	ENSG00000283633.1	0	0
lincRNA	AP000365.1	ENSG00000267338.1	0	0
lincRNA	LINC01665	ENSG00000235343.1	0	0
lincRNA	AC007064.2	ENSG00000237689.1	0	0
lincRNA	AC006946.3	ENSG00000273442.1	0	0
lincRNA	LINC01664	ENSG00000235478.4	0	0
lincRNA	CECR3	ENSG00000241832.1	0	0
lincRNA	LINC00528	ENSG00000269220.1	0	0
lincRNA	LINC01660	ENSG00000274044.4	0	0
lincRNA	AC011718.1	ENSG00000276874.1	0	0
lincRNA	FAM230A	ENSG00000277870.5	0	0
lincRNA	AC023490.1	ENSG00000277017.1	0	0
lincRNA	AC023490.3	ENSG00000278044.1	0	0
lincRNA	LINC01662	ENSG00000182824.7	0	0
lincRNA	LINC01663	ENSG00000276095.2	0	0
lincRNA	AC007326.2	ENSG00000280418.1	0	0
lincRNA	AC004471.2	ENSG00000272682.1	0	0
lincRNA	LINC00895	ENSG00000281548.1	0	0
lincRNA	AC000067.1	ENSG00000225007.1	0	0
lincRNA	AC007731.3	ENSG00000235578.1	0	0

lincRNA	AP000550.1	ENSG00000197210.7	0	0
lincRNA	AP000550.2	ENSG00000234503.1	0	0
lincRNA	AP000552.1	ENSG00000206142.9	0	0
lincRNA	AP000552.3	ENSG00000237407.1	0	0
lincRNA	LINC01651	ENSG00000279920.1	0	0
lincRNA	AP000553.3	ENSG00000273342.1	0	0
lincRNA	AC245452.5	ENSG00000278657.1	0	0
lincRNA	AC245060.2	ENSG00000234630.1	0	0
lincRNA	AC244250.1	ENSG00000223999.1	0	0
lincRNA	LINC02556	ENSG00000236611.1	0	0
lincRNA	LINC01659	ENSG00000234928.1	0	0
lincRNA	LINC01658	ENSG00000178248.11	0	0
lincRNA	LINC02557	ENSG00000225413.2	0	0
lincRNA	PCAT14	ENSG00000280623.1	0	0
lincRNA	AP000345.2	ENSG00000272733.1	0	2
lincRNA	AP000357.2	ENSG00000282012.1	0	0
lincRNA	Z99916.1	ENSG00000236641.1	0	0
lincRNA	Z99916.3	ENSG00000279298.1	0	0
[truncated: 1,530,750 more chars]
